# Analyses of an Expressed Sequence Tag Library from *Taenia solium*, Cysticerca

**DOI:** 10.1371/journal.pntd.0000919

**Published:** 2010-12-21

**Authors:** Jonas Lundström, Fernando Salazar-Anton, Ellen Sherwood, Björn Andersson, Johan Lindh

**Affiliations:** 1 Parasitology, Mycology and Water/Environment, Swedish Institute for Infectious Disease Control, Solna, Sweden; 2 Department of Microbiology, Tumor and Cell Biology, Karolinska Institutet, Stockholm, Sweden; 3 Department of Microbiology and Parasitology, Faculty of Medical Sciences, National Autonomous University of Nicaragua, Leon, Nicaragua; 4 Center for Genomics and Bioinformatics, Karolinska Institutet, Stockholm, Sweden; Universidad Peruana Cayetano Heredia, Peru

## Abstract

**Background:**

Neurocysticercosis is a disease caused by the oral ingestion of eggs from the human parasitic worm *Taenia solium*. Although drugs are available they are controversial because of the side effects and poor efficiency. An expressed sequence tag (EST) library is a method used to describe the gene expression profile and sequence of mRNA from a specific organism and stage. Such information can be used in order to find new targets for the development of drugs and to get a better understanding of the parasite biology.

**Methods and Findings:**

Here an EST library consisting of 5760 sequences from the pig cysticerca stage has been constructed. In the library 1650 unique sequences were found and of these, 845 sequences (52%) were novel to *T. solium* and not identified within other EST libraries. Furthermore, 918 sequences (55%) were of unknown function. Amongst the 25 most frequently expressed sequences 6 had no relevant similarity to other sequences found in the Genbank NR DNA database. A prediction of putative signal peptides was also performed and 4 among the 25 were found to be predicted with a signal peptide. Proposed vaccine and diagnostic targets T24, Tsol18/HP6 and Tso31d could also be identified among the 25 most frequently expressed.

**Conclusions:**

An EST library has been produced from pig cysticerca and analyzed. More than half of the different ESTs sequenced contained a sequence with no suggested function and 845 novel EST sequences have been identified. The library increases the knowledge about what genes are expressed and to what level. It can also be used to study different areas of research such as drug and diagnostic development together with parasite fitness via e.g. immune modulation.

## Author Summary

A method used to describe expressed genes at a specific stage in an organism is an EST library. In this method mRNA from a specific organism is isolated, transcribed into cDNA and sequenced. The sequence will derive from the 5′-end of the cDNA. The library will not have sequences from all genes, especially if they are expressed in low amounts or not at all in the studied stage. Also the library will mostly not contain full length sequences from genes, but expression patterns can be established. If EST libraries are made from different stages of the same organisms these libraries can be compared and differently expressed genes can be identified. Described here is an analysis of an EST library from the pig cysticerca which is thought to be similar to the stage giving the human neglected disease neurocysticercosis. Novel genes together with putative drug targets are examples of data presented.

## Introduction

*Taenia solium* is a parasitic tapeworm infecting approximately 50 million people worldwide [1]. Humans are the definitive host whereas pigs are the intermediate. Cysticercosis is caused when humans accidentally act as the intermediate host and ingest the eggs from tapeworms. The cysts are developed into oncospheres which penetrate the epithelial cells, then migrate to different parts of the body. After migration the oncospheres are established in the tissues as cysts. The cysts can vary in number and size. Cysts which end up in the central nervous system most commonly give symptoms and are the cause of neurocysticercosis (NCC) [2]. The clinical picture of NCC can range from asymptomatic, mild headache and seizures to death [3]. Annually NCC is responsible for at least 50 000 deaths [4]. In parts of Latin America more than 80% of the population with seizures has been diagnosed with NCC [5], [6], [7], [8]. Also a correlation between epilepsy and NCC can be seen and in endemic countries NCC accounts for 30–50% of all late-onset epilepsy [9]. It has also been demonstrated that oncospheres or the cysts have a rich source of antigens which can be capable of stimulating a protective immune response [10]. Several projects are underway to identify an affordable and effective vaccine, but currently the treatment of choice is drugs [11], [12], [13]. Different lines of treatment are available, such as albendazole and praziquantel. Those treatments are controversial as they only are partially effective and side effects have been reported [14].

Another drawback is the availability diagnostic methods in endemic areas. They include the use of X-ray computed tomography (CT), a method not affordable to the majority of the patients with NCC. Other methods include detecting specific antibodies or antigens, which seldom are used as routine within local diagnostic settings [15].

The above problems have been recognized and projects have been initiated to use molecular tools in order to give aid to drug discoveries, vaccine candidates and diagnostic antigens. Recently an EST library from the pig cysticerca stage consisting of 812 unique ESTs was published [16] and a consortium has been established in order to sequence the *T. solium* genome [1]. At the National Center for Biotechnology Information (NCBI), there are different EST libraries from the genus *Taenia* published. The published ESTs from *T. solium* are distributed between the adult stage (45%) and the larval stage (55%).

EST analysis is a very cost effective method to discover novel genes and to see expression of these and known genes together with identifying different protein groups (e.g. proteins with signal peptides). Once an EST library has been established it also can be used to identify genes behind different amino acid sequences which have derived from different peptides of interest [17], [18]. In order to extend our knowledge about these issues and to have a library for common use which contains sequence fragments from proposed expressed genes, an EST library from the cyst stage in the intermediate pig host has been made. The library consists of 4674 high quality sequences. Analyses of these sequences demonstrate that over 50% of the identified unique ESTs cannot be described, 25% previously identified within other worm species and 20% of the genes are conserved throughout the eukaryotic kingdom.

Among the 25 most highly expressed genes identified, 3 are suggested in literature to be associated to the oncosphere surface and therefore potential targets for vaccine development. Also identified within this group is a novel proposed surface protein with homology to *T. ovis* 45W antigen, designated Tsol15.

## Materials and Methods

### Transformation and excision

A cDNA library, Uni-ZAP XR (Stratagene), constructed from polyA+ selected mRNA from 1.75g cysticerci isolated from muscle of a naturally infected Peruvian pig was used (kindly given by Kathy Hancock, CDC [19]). The library was transformed into the ampicillin resistant vector phagemid pBluescript SK (+/−) and host bacteria (XLOLR) using mass excision protocol as described by the manufacturer (ZAP Express cDNA Synthesis Kit and ZAP Express cDNAGigapack III Gold Cloning Kit, Instruction manual, Strategene). As insertion of the cDNA disrupts the expression of the LacZ gene, X-gal [20 mg/ml] was streaked on 14 cm Ø Petri dishes containing agar and ampicillin. To induce the expression of LacZ, IPTG [100 mg/ml] was also added prior to streaking on host bacteria. Clones without insertion and thus a functional LacZ gene are able to digest X-gal, resulting in a blue staining.

### Preparation and sequencing

Positive, uncolored colonies were picked and dropped into 96 well microtiter plates. Wells contained 5µl TempliPhi Denature Buffer (GE HealthCare). The toothpicks were lifted into new 96 well microtiter plate wells containing LB media, DMSO and ampicillin, incubated ∼20h, at 37°C and then frozen for storage. Microtiter plates with denature buffer and bacteria were heated 95°C for 3min, and after an addition of 5µl TempliPhi Premix (GE Healthcare) incubated at 30°C overnight. A rolling circle PCR program with reverse M13 primer was run prior to ethanol precipitation. Samples were dried and 5µl loading buffer was added prior to sequencing. Preparation and PCR were conducted accordingly to DYEnamic ET Dye Terminator Cycle Sequencing Kit for MegaBace DNA Analysis System (GE Healthcare). The reagent amounts and concentrations were modified and optimized at the Center for Genomics and Bioinformatics, Karolinska Institutet, Stockholm, Sweden. The sequencing process was done by a MegaBace 1000 sequencing system (Amersham Biosciences).

### Assembly, sequence analysis and signal peptide prediction

Computational analyses were done using Phred for base calling and Phrap to assemble sequences. Cross match was used to do queries within the ESTs, cut out vector sequences and assemble contigs [20], [21], [22]. To compare the generated ESTs against database on both a protein and a gene level BLAST search was used (NCBI). To disregard irrelevant or low BLAST scores a cut off E-value of <10^−5^ was used.

The translation of the DNA sequences into 3 different frames was performed using Virtual Ribosome - version 1.1 [23]. Translation was done in 2 groups; first, all sequences were translated and second, only sequences with an initiation codon were translated. SignalP 3.0 Server was used in order to predict signal sequences within all open reading frames (ORF) where the start codon ATG could be detected [24]. The D-score in the implemented Neural Network together with Hidden Markov models was chosen. The D-score is an average of S-mean and Y-max, where S-mean is a calculation of the length of the predicted signal peptide and Y-max gives a cleavage site prediction. The result of the D-score is given as: yes or no to the question of whether signal peptide is present. Besides the straightforward prediction of the presence of signal peptide, it also shows a probability of signal peptide.

### Taxonomic classification

Of all the unique sequences the most probable putative protein of each contig and singlet were put into one of seven different hierarchic taxonomy categories. According to the BLASTX results, the organisms producing the putative proteins were set to the taxonomy group last shared with *T. solium* and BLASTX scores with E-value <10^−5^ were set as unknowns.

### Gene annotation

The software Blast2GO was used as described earlier [25]. Sequences in fasta format were loaded into the program and the default settings were used to assign GO terms. From a BLAST search, the annotation of the sequences were performed and pie charts were made using 2^nd^ level GO terms based on Biological processes, Molecular functions and Cellular components. InterProscan was also used within the Blast2GO software and result merged together with the GO terms, as described [25]. Analysis of metabolic pathways were also performed by Blast2GO and KOBAS [26], [27] using the KEGG data base [25].

## Results

### Overall EST analysis

Summarized in Table 1, a library consisting of 5760 ESTs from pig cysticercus was generated. Readable sequences were generated with 5551 ESTs (96.4%). After conducting base call and disregarding short sequences and vector sequences, 4674 high quality ESTs were saved (GenBank accession numbers, GT889435–GT894161). Using Phred and Phrap [20], [21], [22] 1650 unique sequences could be identified (Table 1; Figure S1). The contigs were comprised of 3462 sequences, which corresponded to 438 unique contigs, mean of 7.9 sequences per contig. Contigs mean length were 558 nucleotides (variation between 142–1886 nucleotides). The remaining 1212 sequences were identified as single unique sequences (Table 1). The percentage of ESTs identified with a poly-A tail was 18% and the majority of these were found within the contigs, 40%.

**Table 1:** Summary of *T. solium* ESTs.

Description	Number	Percentage
Total number of sequenced clones	5760	
Total number of successful sequences	5551	96.4%^a^
Number of high quality sequences	4674	84.2%^b^
Unique sequences	1650	35.3%^c^
Number of contigs	434	
Number of clones included in contigs	3462	74.1%^c^
Average clones per contig	7,9	
Number of singletons	1212	25.9%

Percentages are calculated as, part of total number of ESTs (a), part of successful sequences (b) and part of number of high quality sequences (c).

**Figure 1:**
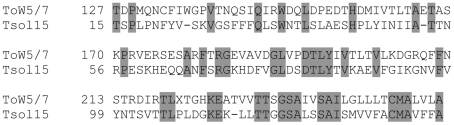
Alignment of Tsol15 and *T. ovis* 45 ToW 5/7 amino acid sequences. Alignment of Tsol15, *T. solium* (genbank accession no. GU338867) and 45 ToW 5/7, *T. ovis* (genbank accession no. gb|AAC47532.1|). Identical aminoacids are marked in grey. Numbers indicate the following amino acid number.

The expression pattern of the 25 most abundant ESTs with a BLASTX cut off E-value of 10^−5^, revealed these to make up 34.5% of all high quality sequences (Table 2; Table S1). The four most common are a set of conserved genes found within the eukaryotic kingdom, representing the antioxidant enzyme PHGPx, the structural proteins Tubulin and Actin and a lysomal enzyme, ATPase (Table 2). These enzymes and structural proteins accounted for 19% of all the ESTs sequenced. The other 21 putative proteins in the top 25 list are other conserved proteins specific to cestodes, unknown proteins and mitochondrial proteins. Among the highly expressed cestode proteins were the vaccine and diagnostic candidates Tsol18/HP6 [28] and T24 [19]. Also identified was a novel proposed surface protein with homology to *T. ovis* 45W antigen, ToW5/7 (Figure 1). The molecular weight of this protein is predicted to be 15 kDa and was therefore given the name Tsol15 (GenBank accession number, GU338867).

**Table 2:** Summary of the 25 most abundant ESTs and their putative identity.

Contig.	Putative identity (BLASTX)	Length (nt)	No. of ESTs	Percentage (%) of high quality sequences.	Identified within other EST libraries from *T. solium*	Poly-A tail identified
**Contig476**	gi|189235991|ref|XP_972419.2|PREDICTED: similar to DNA-J, putative	1012	17	0,36%	+	−
**Contig477**	gi|188485737|gb|ACD50951.1|Nc-DigChim-324430 [synthetic construct]	1120	20	0,43%	+	−
**Contig478** *	gi|59709858|gb|AAW88559.1|oncosphere protein Tso31d [*Taenia solium*]	1011	21	0,45%	+	+
**Contig479**	gi|149364041|gb|ABR24229.1|gyceraldehyde-3-phosphate dehydrogenase [*Taenia solium*]	1182	21	0,45%	+	+
**Contig480**	Unknown 6	851	21	0,45%	+	−
**Contig481**	gi|37778984|gb|AAP20152.1|alpha-actin protein [*Pagrus major*]	931	22	0,47%	+	+
**Contig482**	gi|221113094|ref|XP_002155286.1|PREDICTED: similar to Annexin-B12	1167	24	0,51%	+	+
**Contig483**	gi|116687782|gb|AAT74668.2|cysteine-rich secreted protein 2 precursor	878	24	0,51%	+	+
**Contig484**	Unknown 5	667	25	0,53%	+	+
**Contig485**	Unknown 4	1086	28	0,60%	+	−
**Contig486**	Unknown 3	767	29	0,62%	+	+
**Contig487**	Unknown 2	442	30	0,64%	+	+
**Contig488**	gi|13539680|gb|AAK29203.1|AF225905_1ribosomal protein S15a [*Taenia solium*]	982	34	0,75%	+	+
**Contig489**	dbj|AB086256.1| *Taenia solium* mitochondrial DNA	983	35	0,75%	+	+
**Contig490**	Unknown 1	991	37	0,79%	+	+
**Contig491** *	gi|158934366|emb|CAO82075.1|HP6 protein [*Taenia solium*]	1111	39	0,83%	+	−
**Contig492** *	gi|2114399|gb|AAC47532.1|45W antigen ToW5/7 [*Taenia ovis*]/Tsol15	1088	40	0,86%	+	+
**Contig493**	gi|56753429|gb|AAW24918.1|SJCHGC05540 protein [*Schistosoma japonicum*]	965	58	1,24%	+	−
**Contig494**	gi|37786712|gb|AAP47268.1| t24[*Taenia solium*]	817	69	1,48%	+	−
**Contig495**	gi|256050212|ref|XP_002569521.1|hypothetical protein [Schistosoma mansoni]	813	70	1,50%	+	+
**Contig496**	dbj|AB086256.1| *Taenia solium* mitochondrial DNA	1235	74	1,58%	+	−
**Contig497** *	gb|AAH30393.1| ATPase, H+ transporting, lysosomal V0 subunit B	803	99	2,12%	+	−
**Contig498**	dbj|BAD88768.1| tubulin	1865	143	3,12%	+	+
**Contig499**	gi|207298859|gb|ACI23578.1|beta-actin	1251	214	4,58%	+	+
**Contig500**	gi|117956206|gb|ABK58679.1|PHGPx isoform 1	1359	414	8,86%	+	+

BLASTX was used with an E-value cut off <10^−5^.

*Putative signal peptide.

### Taxonomic classification of the different EST

**Figure 2:**
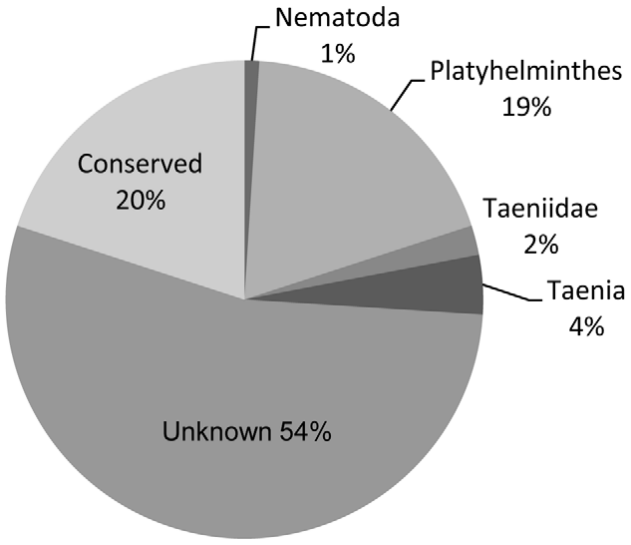
Taxonomical categories. EST submitted to Genbank NR database and categorized according to taxonomical closeness to *T. solium*. Scores with a value higher than E^−5^ were set as unknowns.

Of the 1650 unique EST sequences, 754 different EST sequences could be identified as putative genes found in other organisms in the eukaryotic kingdom. A classification according to taxonomical closeness to *T. solium* using NCBI Entrez Taxonomy database was conducted (Figure 2) [29]. Unknown or novel sequences accounted for 54% of 1650 unique sequences. The second largest group was Platyhelminthes, which accounted for 25% of the ESTs. Within this group, 5% of the ESTs were unique to the Taeniidae and 3% unique to *Taenia* species. Conserved within the eukaryotic kingdom was 20% of all the different EST. Also found was a small group, less than 1%, shared with Nematoda.

### Identification of putative secretory proteins

Secretory proteins have been suggested to play a major role in the pathogenesis of parasites [30], [31]. The secretory proteins may also act in immunomodulation processes [32]. We therefore searched our ESTs for putative signal peptides according to the criteria given by SignalP 3.0 [24]. The ESTs were translated into 3 different reading frames and the longest ORF from each EST was chosen as the relevant putative protein and used in the SignalP 3.0 prediction software. We found 154 ESTs sequences with predicted signal peptides, representing 9.3% of the unique sequences. Of these, 119 (78%) were derived from ESTs with no significant BLASTX hit (E-value <10^−5^) to the NR database in Genbank. Among the 35 predicted signal peptides with a significant hit in the BLASTX database were 4 ESTs identified within the 25 most abundant groups of ESTs (Table 2, Table S2). These 4 ESTs were found to have similarity to an ATPase, Tso31d [33], HP6/Tsol18 [11], and an antigen earlier described in *T. ovis* (Figure 1, Table 2, Table S2). Notable, also among the predicted secreted proteins are at least 2 different proteinase inhibitors, a serine proteinase inhibitor, detected as a single EST (TSCC.R39.esd, Table S2) and a proteinase inhibitor belonging to the Kunitz family. The ESTs with homology to the Kunitz family consisted of three ESTs (TS. Contig318_rframe2_ORF, Table S2). Putative roles of these proteins are immunomodulation of the host [32] or to act and interfere with host physiological processes at the initial stages of infection [34].

### Gene annotation

**Figure 3:**
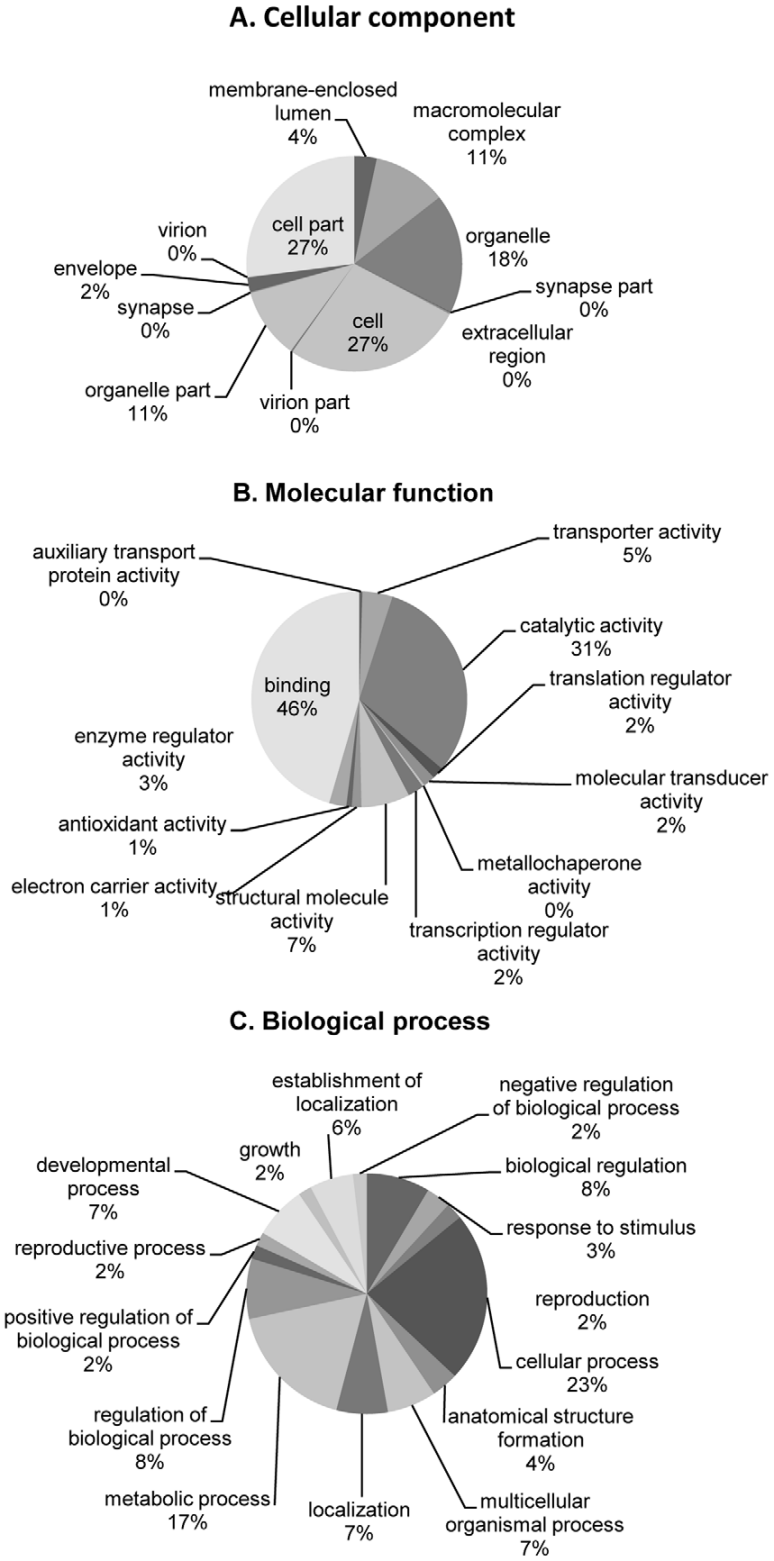
Pie charts of 2^nd^ level gene ontology (GO) terms. Together, 620 unique ESTs and contigs were given a GO category. The three GO categories is presented include, Cellular components (A), Molecular functions (B) and Biological processes (C).

All of the 1650 unique ESTs were analyzed for their similarities to proteins within the Genbank NR database. The automated software Blast2GO was used, which also annotated the different sequences when possible to a gene ontology (GO) group described by the Gene Ontology Consortium [35]. The annotations to GO groups were also performed with InterProScan. Results were merged together within the Blast2GO and further analyzed [25]. 754 ESTs scored a significant hit (E-value <10^−5^) and 634 of these were able to obtain a GO term (Figure 3, Table S3). Presented in Figure 3 are percentages of 2^nd^ level GO terms as graphs. The 3 different graphs are presented as Cellular components (Figure 3a), Molecular functions (Figure 3b) and Biological processes (Figure 3c). In the Cellular components, 3 different major categories could be found; cell (46%), organelle (30%) and macromolecular complex (18%, Figure 3a). In the Molecular function category the main categories were ESTs involved in binding activities, 50%, and proteins involved in catalytic activities, 33% (Figure 3b). In the third category, Biological process, 26% belong to cellular processes and 20% to metabolic processes. Other ESTs were divided into smaller categories and no major category could be identified (Figure 3c).

The ESTs with an assigned GO group were mapped into different metabolic pathways by 2 different web-based programs, KOBAS (KEGG Orthology-Based Annotation System) [26], [27] and Blast2GO [25]. The ESTs mapped in to 60 different metabolic pathways using Blast2GO and in most pathways several ESTs could be found (Figure S2). Similar results were also obtained from KOBAS web based program even if presented in a broader spectrum with no strict rule to metabolic pathways (Table S4).

## Discussion

NCC is responsible for over 50 000 deaths annually. In order to increase our understanding about the disease causing stage we have sequenced over 5000 ESTs from this stage. Another EST library containing 817 sequences from the same stage can be found in the literature [16]. However, in order to get an overview of the expression profile more ESTs are needed. This library will add data to the understanding of *T. solium* and it is to our knowledge the biggest EST library from *T. solium* cysticerca analyzed to date. A BLAST search against published ESTs at NCBI from *Taenia* species was performed (Table S5). The result demonstrates that 845 (52% of the whole library) unique ESTs are added to the NCBI archive. The library can be divided into 2 groups, ESTs represented as a single sequence and as contigs which contain several ESTs (Table 1). Most of the unique ESTs are found within the single group, 62%, meanwhile the more expressed contigs have less unique ESTs, 28%.

Previously described ESTs from different parasitic nematodes and trematodes suggest, in accordance with our study, that a large number of the ESTs belong to a group of unknown sequences with no similarity to other sequences found within the Genbank NR database [16], [36], [37]. The large size of this group is most likely due to the relatively few genome projects performed on trematodes to date (Figure 1). The only 2 whole genome sequences of trematodes that has been annotated and recently released to the public are the *Schistosoma mansoni* and *S. japonicum* genomes [38], [39]. This also is visualized in the GO hits demonstrated within this study; 57% of the ESTs are found to have similarity to genes in *S. mansoni* and *S. japonicum*. The GO predictions present data which differ from earlier described analyses by Almeida, C. R. *et al* [16]. In Figure 3c, a decrease in ESTs responsible for cellular processes is seen, from 61% to 26%. Meanwhile, the class for metabolic processes is found to be similar, 15% versus 20%. In the binding category (Figure 3b) an increase from 41% to 50% is seen and the catalytic activity increases from 29% to 33% [16]. The changes seen are most likely due to the size of the different data sets used, 96 annotated sequences [16] compared to 634 (this study).

Among the ESTs found to be most highly expressed were structural proteins (e.g. tubulin and actin) together with different enzymes (e.g. PHGPx isoform 1 and ATPase, Table 2). Genes proposed to be expressed at the membrane of the human oncospheres and involved in the establishment of the human larval stage also is highly expressed. Examples of such genes are Tso31d [33], T24 [19] and HP6/Tsol18 [11], a candidate vaccine protein [40], [41]. Several of the highly expressed ESTs have unknown functions and further characterization are needed. Other highly expressed sequences have homology to genes found in other species and can therefore be proposed to a function or to become a target molecule for example, vaccine development. An example can be found in Table 2 where a cestode specific EST, predicted to contain a signal peptide, is the ninth most common expressed protein in the cysticercal stage. The whole sequence of this mRNA could be obtained from contig 492 (GenBank accession number, GU338867, Figure S1) and analysis of the same revealed an open reading frame (ORF) corresponding to a protein with a predicted molecular weight of 15.3 kDa. Results from a BLAST search revealed a similarity to a family of 45W antigens from *Taenia ovis*, E-value <10^−6^ (Figure 2). The 45W family of antigens are known to be expressed at the surface of oncospheres [42]. The new protein was named Tsol15. Other Tsol-proteins have been suggested to be valuable target molecules for diagnostic and vaccine purposes and Tsol15 could also join in as a proposed diagnostic antigen.

This EST library could also give crucial information with different word searches. The searches for molecules that have effects on immunomodulation of host defense are presented here. Several molecules have been described to play a role in host cellular immune responses. Examples of such molecules are different protein inhibitors, cystatin, prostaglandin, nonintegrin, TGF-beta and macrophage migration inhibitory factor [32], [43], [44]. Of these a general serine protein inhibitor and a Kunitz type 8 of serine protein inhibitors were identified within the group of predicted secreted molecules (Table S2). Both inhibitors have been identified in the cestode *Echinococcus granulosus* and suggested to be involved in the interface between parasite and host [34],[45]. Also cystatin and TGF-beta signal transducer were identified (Table S1). However, more work is needed in order to be able to draw any conclusions.

Another example of the use is to identify drug targets. Patients suffering from schistosomiasis or cysticercosis will be treated (if at all) with Praziquantel, a drug that disrupts calcium homeostasis within the parasite. This drug is freely distributed among several (but not all) countries via the ‘Schistosomiasis Control Initiative’ (SCI; http://www.schisto.org). The ubiquitous use of Praziquantel will most certainly lead to the development of resistance among various species of flatworm. Because Praziquantel, in many cases, is the only available drug, the development of resistance is a very serious problem. In order to identify new drug targets metabolic pathways could be studied and enzymes unique to the cestodes be found. Presented in Figure S2 are single ESTs which have been annotated and mapped in to 60 different metabolic pathways. The code of single ESTs are given and from there the nucleotide sequence be obtained. In Figure S2 proposed drug targets can be identified, e.g. 6-phosphofructokinase (TSBR.R48.esd), glutathione synthetase (TSBV.R85.esd) and thioredoxin glutathione reductase (TS.seq. Contig445) [46], [47]. Another way of identifying possible drug targets would be to use the strategy by Crowther G. J., *et al* [48]. The authors describe an *in silico* method to identify prioritized drug targets in neglected disease pathogens. The work also highlights the fact that data are lacking for less studied pathogens, e.g. helminths and how to overcome this by mapping data from homologues genes in well studied organisms [48]. Using a similar approach several ESTs from this library could be identified as having homology to *S. mansoni* drug targets. Examples are ATPase subunit alpha (TSAO.R53.esd), tubulin alpha (TSAF.R50.esd), guanine nucleotide-binding protein (TS.seq. Contig375), beta-tubulin (TSBB.R23.esd), 6-phosphofructokinase (TSBR.R48.esd) and proteasome subunit beta 1 (TSAB.R74.esd).

Cysticercosis is a major health problem in many developing countries where this disease is endemic affecting both humans and animals. The persistence of this zoonosis is intimately associated to cultural patterns and mainly to the extreme poverty of the human populations. This disease is a neglected problem, probably partly due to it is being mainly restricted to low income countries. Although several predictions are made and EST libraries can be used to identify genes which might be important to novel drug discoveries or used in new diagnostic tools, the intriguing results are the vast majority of ESTs that have an unknown function today. A solution to increase the understanding of this group and possibly get data which will favor the patients with neurocysticercosis would be to increase the resources in the field of neglected parasitic worms.

## Supporting Information

Figure S1 Sequences from 1650 unique ESTs in fasta format.
(2.04 MB DOC)

Figure S2 Metabolic pathways and their mapped ESTs according to KEGG within the Blast2GO program.
(2.00 MB PDF)

Table S1 Result from BLAST search with a score <10−5 of 1650 unique ESTs.
(0.66 MB DOC)

Table S2 Result from BLAST search with a score <10−5 of 154 unique ESTs predicted to contain a signal peptide.
(0.05 MB DOC)

Table S3 Result from Blast2GO web based annotation program. Results are presented as hits with a score <10−5. Results are divided into C = Cellular component, F = Molecular function and P = Biological process.
(0.48 MB XLS)

Table S4 Result from KOBAS web based annotation program. Results are presented as hits with a score <10−5. Values in the column “Count and ratio” are divided into two rows per pathway. The top row corresponds to actual value. Bottom row corresponds to value in *C. elegans* for comparison.
(0.48 MB DOC)

Table S5 Result from BLAST search with a score <10−5 of 1650 unique ESTs towards Taenia solium total ESTs at NCBI.
(0.93 MB DOC)
