# Supplementary material for: Analyses of an Expressed Sequence Tag Library from Taenia solium, Cysticerca
Source: PLoS Negl Trop Dis. 2010 Dec 21;4(12):e919. doi: 10.1371/journal.pntd.0000919 (PMC3006133; doi:10.1371/journal.pntd.0000919)
Supplement: Figure S1 — Sequences from 1650 unique ESTs in fasta format. (2.04 MB DOC) [file pntd.0000919.s001.doc]

>TSBN.R16.esd 711 0 711 ESD GOOD: 95-246

ATCGGCACGAGGGCCTACGTGCCGAATTCGGCACGAGGCGTTTGCCTTTT

TTGCCTACCACGTTAGTTGACACTCAAGTTAACCAGCTGCTTCCTTCTCC

CTAAGTGCGACTGATCGATGTCAATGTCCTCCGAGTTGTTCGCTGCTCAC

AT

>TSBB.R20.esd 573 0 573 ESD GOOD: 89-572

TCGGCACGAGGGGCGACCCTGCATGACGTATCAGCGAACAGGTGCATGCC

AATACGGCGTAGCTTGCCGGTACTCACATTTGACAAGGGAGGAGGAGGCA

CGTCTTCAGGCAGCAGCGGAGCCGGTGCAGGATCCAATGCAAGCAGTTTG

GGAACTTGAGGAGTTAGTACGGAAAAGAAGAAACAGTTTAAAAAATTTGA

AATTGCCAAGGGGCTTCAGGTTTGAAGATTTCCCTCCTTCCGTCAAGCGA

TGTCTCGATAAAGGGAATGCTGACGATGCAAGCCAAGGGTGAACTTGGTG

TTCCGTGTAGGACACTTATGTAAAACAGGCAGGACTGGCTACCCAATGGG

TAAAAGGAGCTGTTCTCACACAANCTCTCCTGACCCCATAACGCCAAACG

TAGCCGTGAGCGGTCGCCATACTGCCCTTTAGGATGAGAAAGGAGGCGTG

ATGAAGACACCGGCTCGTCCTCATACCCATCCNN

>TSBM.R95.esd 627 0 627 ESD GOOD: 99-624

GCACGAGGGTCGTCTCTTGCAAATGACCCCTCAAGTGTCCCCAACCGTGA

GGACGCTACCTCCCTGTATCTCTGTTCCAGCTCTTAGGTTCGGTCTTGCA

GAACAGAAGTCCGAGAGCGCTACACGCACGGCCAGCACTACTACTTTTAT

TTCTGAAGGCAAGGGCGAAGAAAGCGAGAAGGGAAAGACGAAAGGGGAAG

AGGACATAACGAATTTGAGACCAGAGGAGTTGGATTCAAAAATCTGTCGT

ACATACCTTTTTGAGGAGGCCATGATCAATTCGACGGACTCGAAGCTCTG

GACTAACATGCAATTCTGGGAAGACCTATTCCTGGACACTGTAGTACAGG

AGCGAGGCTTGCTAGGCATGGACTTCGATCCAAAAGGTCTGCTGGAGCAC

TACTCTCGCCTGTCCTCAATCGCTCGAAAGCACCTGGAGTTGACCGAGGA

TGACCTTCTGGCCGGGGTGATGCACAATCTCACTGCCTTCATGGTGATGG

CTCGCATCCCACCCGATCTCATTTGN

>TSBW.R74.esd 577 0 577 ESD GOOD: 98-287

GCACGAGGGTTTTTTTTTTTTTTTTTTGATCATTCGTAAATACGCTTTTT

TTCAATAATGTAAGAGGCGGATTCAGGCCCGTGACTACTGTGAACCTGCA

TGAGCGACGACGAGGTAGCACGAATAAAACTTTCTGCGTAGAACCTGCGC

AGGACGCGAAAGCTGCGCAACACGCAACGCAAGCGCACGC

>TSAO.R59.esd 681 0 681 ESD GOOD: 97-677

GCACGAGGTTCTTTCAACAGCTTATGAGAGTGGTTCGGTGGCCTTTTCCA

AAGACTTCCTCAATGAATTTGGAAGGATTCTACAGAAGTCGAATGAGCTT

GCCGCTAATAGAGCTTTACCGGAAGGCATGCTCAGCCCCCTTTTAATGGC

GCAATCTGACCCGCCTCACGGAGCCTTCAAAAGAAGAAGGCGAACCTTGC

GTGTTCGGCGTCAACGTCGATCTCTTCAAACTAAGAACACTGTTACCGAA

TCAAGGTCATCGGGGTGTCATCCAAGTGACAGTGTGCCGCCGATACCGGA

GATGAGTCGCTTGTCTGACTCGGAGGTGTATAACCAATCCAGATCGCCTT

TGCAACCTCCGGTACACTCTGAAATGACTCTTGAGGATCTTTGTAGTTGT

AGTAGCTGCAGCAGTAGTTCATCGTCATCTCGAACTTCGACTTGTTCGTC

TCCATCGCCTTCGTCATCCTCATCGGACGCTGATCGTATCTAGACCAAAC

TTGGCTTTCATTGCGTCATCCGTTTTTTTATCACCACACCAAATACCGTA

TTTTTGGAACCCGCAAAAAAAAAAAAAAAAA

>TSAC.R39.esd 560 0 560 ESD GOOD: 93-501

GCACGAGGACTATTCCCTAACTTAATCCTCTGCTGACTTGTAGGTCTCGT

ATTCTTTCTCTTATGCGGCTAGCTAACACGCTGCTCTGTGTGTTTGACCC

TGATCGCCTCGTCTGTACTCCAACCTCCTCCTCCCTCCCCGTGGCCCTTG

CATTCAGTCTCGCTTTTGCACGACCGTTTATATGAATTAAAGCCATCTGC

TACTCTTGTCTGCATCGACGGATAAGTGGGCTGTCGTANGCATGATCTAC

GGGCCTTCATATCTGTTACACCTTCATTTCATACGTTTTGACACTATTAT

AATGAATCGAAATATTCTTTTAGTACATTTCCTTTCTGTCCACATTGTTG

TGTACCGGATGGAATAAACAAATACCTGGCAAGTTCGGAAAGTCTCAAAA

AAAAAAAAA

>TSAA.R30.esd 637 0 637 ESD GOOD: 102-299

GCACGAGGCTCCGTCTGAGTTATCCTACTCTCATTTCTCTCCACCGCCAA

CTCTCCTCTATCTTCTCTATTTCCTCCCATCCTCTCACACCCCCCCTCTG

GGCGTCCTCCTCCTAACAGCTTCGTTCGGGTGCTCTTTCCGCTCTCCTAA

TATGCATCCGCTTTGTCGGGCAAAGAATCAAAAAAAAAAAAAAAAAAA

>TSBD.R81.esd 709 0 709 ESD GOOD: 84-479

GCACGAGGCGGCACGAGGGTCAAATGGCAGCGAAATATACACGCGATTTG

ATGTATCCACTCGTAAAGTGGGTTGGAGAAAATTTGGATGTCACACATAT

GTGACGCTTGAGTTTAACGAACTAGATTTCCTAAATACTACGAAATTCCT

TGCAATTTTGAATCGTCCACTATATGCGAGTTGTTTATCCATCACGTTCC

TCGACAAAATCAGAACTTTTGGGGAAAAATTTTTCTGAAACCACATCGCT

TTCGGATTGGGTCGTGCTGGTCTAACGCCTCTCTTTCCCGTCTTTTCTCT

TCTGTTGCCTGATGTTGCAGTTTGATTCATTTGCGATTTTCGTGTCGAAT

TGTGCTGTAAATCATCTGGCATAATTATACGAATATTTTTGCTCAA

>TSBO.R4.esd 700 0 700 ESD GOOD: 112-350

GCACGAGGGAGGAGTTCAAAAGGCGAGTAGGACAGACCGTTCGAAAGAGT

CAAGAATCCTTCGAGTGAAATTGGTAATCTTTCAGTGAAAACGCTGTACT

GGTATTGATTTGGGCTGTATCCCTTATACTTGTGCGCCCTTTCAGAATAT

CGTTTTCGTTGCCTAAAAAAAAAAAAAAAAAAAAAAAAAAAAAAAAAAAA

AAAAAAAAAAAAAAAAAAAAAAAAAAAAAAAAAAAAAAA

>TSBG.R80.esd 714 0 714 ESD GOOD: 126-448

GCACGAGGCAAAGACTTGCTTCTTTTAACCAAACAACCTGTGACGTACCT

CACGGAAATACTGAAGGGAATTGCGGTCTTCAATTCCAGACCTCCACACC

AGAATATGTGGGAGCTAAAACCGGAGTACCGTCACTACACCTCAAAAGAA

GACTCAACAGCCTGAAAATGCCGCATGAACCACAGTTAATTCATGTTTGT

ACATATTTGTTAGTCGCTAAACGGATGGATACTGTCGAGGTGCTCATCCG

GCAAGGTTACAGAAGTCAACCACTCGCAAAAAAAAAAAAAAAAAAAAAAA

AAAAAAAAAAAAAAAAAAAAAAA

>TSBW.R53.esd 430 0 430 ESD GOOD: 91-411

GCACGAGGGTTTGCTGACCGGGTGTTTGCGCTTGGTTCTGAATGCTCCTT

CGGACAGCTTGCGCCAAGTAGCCGACTTAGCCGATGTTCGCGTCGGAAAT

CTTGTTGGAAGCCTAAACATTGGCGCTGAACTTTACGACGCTGCGGTTCG

ATCAGTCCATACTCGGATCTGTCCGATTCGTAGCCTCGAGACCGAAACCG

ATTTTATCGTCAACACACGCGCCAACCTTAACTCCATCCTCTTGAGCCCC

ATTTCAAGGGATGTGTGCTGGCGTCAGTTCAGTGGGGAGAAAGCTGTGCC

GTCCCTACTATGAGGTCACAT

>TSCA.R62.esd 700 0 700 ESD GOOD: 134-425

GCACGAGGGTCACAAGGCTTCATTCATTTACCCTTGTTTTGTGTGTTTAG

GAACAACTCCTACGCATTTCGTGGTTACGTGAGCTTTTCTTCTTGAAGTG

TCAGTTCTTGCTCCTGCTCTTTCCATAGCCACCTCTCTCCTCTCTTCTTT

CTTCACAAGTTATGATGATGATGCTACCGCATTTCCTCGTTATTCTCCTC

CCTCTTCTGAGTAATAATTAACTAATATATTTTCGAAATTAAAAAAAAAA

AAAAAAAAAAAAAAAAAAAAAAAAAAAAAAAAAAAAAAAAAA

>TSAV.R28.esd 603 0 603 ESD GOOD: 101-540

GCACGAGGCAGGAATTGGAGAATTTCTCAATTAACGAAAGGGATCCGATC

CACTTCCGAATTCGAAAACAAGACTCTTGGCAGATCTACAGGAGGAATTA

GCCATACCTTCTACTGATTTGTTCCCGACACGAAGCCCTGACCGTGAGGA

GGCGTCGATCCCCATCCAAGTAATGCTGTCCGCTCCCGATCGCGATCAGA

GTGAAAATAATGGCTCGTCTCTACCTCAAAAGGCGAATCATCCTCTGGAA

GCCTGTCAATACAAAGACTTCAATTTCCACGAGNAAGGCGAAGGTGCAGC

TCGCGGTCCCGTAAACCCTCGGAAACTGTGAGAGTGGTGAAATATTGTCA

GCCAATTTCCCATTCGGGCCGTTTTATCCTGCGGAAAACAGGGTACGACA

GCAGTGTAGTAAATGGCCAAGGGACCTCCTCGTCAAAACC

>TSBP.R71.esd 909 0 909 ESD GOOD: 97-539

GCACGAGGATCAAATGCTAAGCCGATGGTCTATCCTTATGATCTAGGATG

GAGACGGAATCTCCTACAGGTTTGCGTACTTGCCTCATAATTTGCTTTCC

ACACTTCAACCGCATTTGTTGCTATTTTTAATCCATTTGCGCTTTTTGTT

TCCAACGTGCTAAATAATCGGCAAATGTTTTGAGACTTCCCTTTTTTTCT

GGTTTCTAAGGTGTTGAGTTGGAGTGGAAACCCGATAGGTGACGGGTATT

TATGGCCGTTGAAGCCAGGATGCGGAACCTATGACCTTACTATAGAACAG

TTGAAACAAAAGAACCTGAAGAACAAGTCAGCCATTCCCTTCCAAATTGA

ACGCGCCTACTCGGGTAGCACGCTGCCTATATCGTTCGGCTGGCGCACCT

GCTGTTGTCCACCATGCAGTGGGGAGAACCGTATGGTTGTTGC

>TSBW.R22.esd 545 0 545 ESD GOOD: 90-545

GCACGAGGCCNTCGTAGCCGAATTCGGCACGAGGGAATAATTTTGGTGTT

CGTGAACCGAAGTCTCCTCTGAACGGCCTCTCCGTAAACCTACTTCCACC

CTCCGCTTCGATGCCGGAAATGTCGAAATCTCGAGTCGAAACCGACGATG

GGGTGTCCTACCAACGGTTGCAGCACTCGGAAGATCCTACAATTGATGCT

GCGATTCGGAGCATAATTGATCTGTAGAGATCCCATTTCTTGTTTGTGTA

AATACGACTGGTATACATACATACACACAAGTGCATACATACATATACAC

TTACACAATACAATATTCACTCCTCTTGTTTTCTGCCGTATCTTCAGATG

CTGTGCATACTTGTAATTTTTTGTATGATGCGATTTCCGCCTTCTCCTCC

ANCCTCCTCCATCTCCCTTGTATGATAGCCGCAAAAATGCAATAAAGGTT

TTAANG

>TSAH.R59.esd 648 0 648 ESD GOOD: 100-621

GCACGAGGCTCACTCTCAAGCGGCTCCCAATACCGTAGCCATCAGTTCAA

CAGTTCCATCAGCTAATCCCTCAACAGTCGGCGTCGGTGCCGCTGCTTCT

ATTGTAGCTACATCCGAGCATCGAACAACAACGATTCCACCCAGGCAACA

ACAGCAACAAAGGGGGCAAAAAATAGATCCTTCGCAAACACACGCAGCGC

AACTTCCTATGACAAGTATAACGCAGAGCTCAATGACAATAAGCAGTGGT

GTTGCGGTTCATCCCGGGGAGATGGCAACATTGCCTGATGGGATGTACGT

AGGCAGTGGGGCCGCAACTACTCAGAAGTATCCGCAGGGTTTCCCAATTT

CCGTAGTGGATGTGCCTCAGGTGCAGCAGTTGGACAATACAAATCCCTTC

GATTTTCCGCAAGGCGGCACCACGTACCCTGTTTCCGCCTCCAGCTCAAA

CGCCCAGTCCCGCGGACCCTATCGGGAGCAATTGCACATTCACTACCCAT

CACCGACAGGCTCAAGCACTTC

>TSAD.R47.esd 737 0 737 ESD GOOD: 94-372

GCACGAGGCTCCCGCTTCTTTCTAGTGATGTGTATCCCATCATACCCAGG

TCCCATACCCATCATTGTATCCCATCATACCCAGGTCCCATACCCATCAT

TGTATCCCATCATACCCAGGTCCCATACCCATCATTGATGGATTTATAAA

CACTGCGTCTTTCCCTTGGCCTTGACTCAAATGTGTGGTTGATATTTTAA

AGCTCCTACTGTTATTTTCAATTAGTTATTTATCTCAGCTTTTTCAGGGA

CCTGAAAGCTAAAAAAAAAAAAAAAAAAA

>TSBD.R22.esd 723 0 723 ESD GOOD: 99-473

GCACGAGGCTGAAATCGCCTGACATCCTTGTGCTTCGCTCTATTGTTCTA

TTTTTTTGATACCAAAACCACCACCAGCATGTTTTGAGTGCATTTTACTT

TGGCCCCCGTCCCCAGCCGTGAATTGGCTCGGCTTTTATGCAGCATTTAA

ATACTTTCAGCTACTCTGCAGTAATCTATGATTTTGTAAATCTTACGGCC

TTCCTTCCCTTCTATTCTCTGCTCCCTCTTCTTTCCTTTCTTCCTTGTAG

AGCACTTGTTGCCGGTTCGAGCTGTCGCCCAGTATTTAACTGCCTATTTA

CTTACCAACTTCCAGAAAGATAGTGTAAGTAATAAACGGACATTCCCTGC

CATTAGAAAAAAAAAAAAAAAAAAA

>TSBY.R75.esd 636 0 636 ESD GOOD: 103-486

GCACGAGGAGATCCTCGGCCAGCGATGAAAGTTCGGAGTCGTCTTTTTCC

CTTACTGACGGTGAAAGTGGTTCGAGAGAGGCGTGGGAGGAGAAAGCAAA

GGAACTTGATGAGGCCAACATCTGGTGGCTGTCAATTCTCAAGCTGGCCG

GTAGGTGGTATGCCGGCTCTCTCGACTTTGAGAGCGTCGCGCCGAATGAA

CGCTTCACTCCAAGCCTAATCGAAAGATGGNTGCTGATGCTCAACTAATT

TCACTGGCTTATAACCTATTGCATGTGACTCAATCCAAAGCCTTTAACGC

CCCTAAAACAAACCCAGAGGNNGGGTGGAGACGATGCNTTTTCCGAAGTC

TGGGGCGGCNTATTCGAGAAGGNTCTTCCGACTN

>TSAM.R54.esd 765 0 765 ESD GOOD: 104-440

GCACGAGGGTTAGGTATTGTTCTGTGTAGTATTTGAGTCCTGGCGCCTTG

ATCTTGCCATCCAAATTCTTCATTTGATTTTATGCTTAGATTCCTTATGA

AGCTAAATCATGAAACTGTGACAATCGAACTGAAAAACGGCACACAAGTC

CATGGTACCATCGCTTGTGTTGATGTTTCGATGAATACGCAATTGCGATC

CGTCAAGCTTACCATGAAGAAACGAGGAGAGCATAAATATGGATACTTTA

ACTGTCCGCGGCAATAACATCCGGTATTTTATTCTTCCTGATACTCTTCC

CTTTGGACACACTTCTTAATGACGAGGGTCCAACCAA

>TSAG.R77.esd 656 0 656 ESD GOOD: 90-468

ATTCGGCACGAGGAACATAATGTGCGAGAACACGGCCGCTGACTTGAAGC

AGTTATCTGCGTGTTTCGGTGAATTGGGACGAGTGCTTAATAGCCTTTGG

CTCTCCGGGCATTGGTCAGCCAGTGACACCATTGCTGTCTTGCATGAGAT

CACCAACACTCCTGATGGCTGGCTGGCTGAGAATGTCGCTTGGTTTCTTG

GCCTCCGCTTGTCCTCCCAACTGGCCTTCGTTGGCCGGTGCAGCACCAAG

GTTAACCAAGGGCTATTGTTCCCCCGAAATTCGCTTGTTCTTTCTCCCCC

CTCTTTTGTGGCCCTTGTTCCAGGTTTAACCCTGTTTTTTTACGAATTGA

AGCGTCTGGATTTCTTTCGGGGTTCGGGA

>TSBY.R10.esd 539 0 539 ESD GOOD: 96-539

GCACGAGGCTCTTGCTGAATAATTTGGTGCGTCTATTCGCCAAAGTTGCG

CATCCAGAAGTGGTGAGTGTCGCCCAGTCCCATATTCAGCCGATTCGGTT

CCCTAAAGTAAACTATAATGACATGACGAAAACGGAAGGAGCATGGCCAG

AAAAGTATGACGCCCTGAATGCGGAGTACTCGAGATATATGTACCTTGGT

ATATGCTCGTTTGTAGTGTCACTTGGCATCACCTTGTACACGCTCGACCC

ATTCAAATATATGGAGCCGGCGCACAGAAACACGCCGGAGGGCCTGGCGT

TTCTGAAACCGAAACCAGAGATTCTAGCCGATATTCACGAATGAACCAAA

AGGGCAGCGAGCCGTACAATTCATCACGCCTCCCCCTCCTTTCCTTCCCC

TGGTACTAAAACCTAAACGTTCTCTATTTTCAATAAATCACNCC

>TSAR.R16.esd 517 0 517 ESD GOOD: 122-310

GCACGAGGATCAACTCCACTCTTACTCGCAAGGCCTTCATCGTCTGACAA

TCTCTCCTCCTGTCTATGCTACCAGTGCCACTGTCCTCCTCCTCTTCATT

CGTCGGGTCATTTCATGCATCCATAACGGGTTATCATGTTGGTAGCCTAA

TGCATTTCATGTTTCCGTAAAAAAAAAAAAAAAAAAAAA

>TSAI.R31.esd 760 0 760 ESD GOOD: 95-580

GCACGAGGGTCACCTCCATGGCCGTCGCTTCCCTCTCCTGCAAATCAGAG

AATTGCGGTCGATCGTCTATCAGTATCCGATCTTCCGAAGCCCAGCGTCT

CCTAAAGGAAGGCTTTGATGTCATGAAAACGGTGGCCGATGCCAAGCTTG

TACTCTTTTTCTATCTCACTACGCTGCGTCTTGTTGTCTAGGTTGAAATA

CTTCAAACAATATTTGTTTCGGTTATTTAAATATCTAGCAGATTTAATTA

AAAGCTTACGTAAATGCTGAAATATGAATTTAATAAGTTACTTGTATGCG

TATGTGGTGGTGAATGACTTTCAGCTCCTTAACACCACTTCAATGACCCA

ACGGTGCTCTGGGGAAACACAATTTTGGGGCTAAGTCAAGCCTCCGTCTC

TGATCTTCTCTGCAAATGCCGACCGTGGGATCAGATCTCTGGCAACAAAC

CCAGAGAGTCTTATGTGCGACTCAAACTCTGGGTGG

>TSBH.R68.esd 563 0 563 ESD GOOD: 96-560

GCACGAGGGGTCGTTGGGGCCATGACGGGTTTAAGGAACTTGAGAAACCT

GGTCCCCCTGTCCCCCTATCGATTGATGTGCAGTTGCGACCAAGCAATAA

TGCTATCTCTACGAAGTTACCTCTCCTTGTTTGCAAATCACATTCTCCCC

TTGCTAAAACCTCTGCATCTTCCTCGTCCTCCAGTTCCTCGTCTCGTGTC

TCGGAAAGGACCCACAAAAGACGGAAGCAGGGGAAGAAAACGAAAAGTAG

GAAGGAGAAAAGGAAGAAGATGAAAAGGCGGACAAAGAAGAAGAAGAAGC

GTGTTTGCGATCCCTCCTGTTCTTCCTCTTGCCTTAGCAGTGACAGTGAT

CTTGATGTGGAGTGGGTTGAAAAAAAACCGTGATGTGCCTCCTGTTTGTG

CCTATTTTGTTATTTTTTATTAACGTAATATCTTTTACAGAAAAAAAAAA

AAAAAAAAAACTCGN

>TSBF.R89.esd 350 0 350 ESD GOOD: 21-262

GAACAAAGCTGGAGCTCCACGCGGTGGCGGCGCTCTAAACATGGATCCCC

CGGGNCTGCAGGAANTTCGGCACGAGGAGNNGGATTGGCGCGAGCACGCG

GCGCGCGTCCTTTGTGGCATGGACTCCAGACGGACGTCTCGGGAGTTCAG

GCCCACTTGTCTCTCCAGGTCTGCGAAGTCCGCAATGGTCATCCGGGCAA

TCTGATCACTGCGTTAGGTGTGGAAATCAAACAACGAGCTCA

>TSAO.R29.esd 678 0 678 ESD GOOD: 114-554

GCACGAGGATGATTTTCTGCAACACCGTATCCACTGCTTGTATGACGCGG

GTTACAATGCCCAAGATGCTGTCGTCCTACGAGAAGGACCCCAAGCATCC

ACCTTGTGTTATTAACATGTCTTCCGTCTCAGCCCTCTTCCCTCGCCCCT

ATAAATCCCTCTAAGCCGGAATGCAAGTTCATTTGTGCAGAATTTCTCGG

CGTCTGTTGCCGGAGAAACAAGGGAACTTCTGCGCACTCGAATTCGATTT

CTCACTCTCACTCCAGGCTTCGTCTGGACGCCTAGCAAGGGCGAAAAAGA

AAGTTTAATTTTTTCGTACCCACTCCCGAGGTATTTGCCAACTCCAGCGC

TGAACATGATTGGCATTGCCTCCCAATGTTGTTGGCTTCATGCCCACACG

AGTTAATGTTTCTTGGGATTTGAATTACTACCCGCCTCTTT

>TSCE.R60.esd 610 0 610 ESD GOOD: 100-610

GCACGAGGCGACCTTTCCGGCAACACAATTCGCGGTGGGTGACGCAAATG

GAAAAATACCTGGCTGAGAGTCGGCCAATCTTGATGTGAATGCAGTGGTA

TCAGTAGTGGTGGTGGGAATTTCGAATTCATCATCTTGTTCAGGCGATTT

CCGCGTTTTTTTTTTGTTCCAGTAACAAGTTTGTTATCAGCACCACCTGT

GCGACCTCCTAGTCTTCTTACCTGGTTCCGACGTGGCAAATACGTGCCGA

ACTGCTCCCTGTTAGATCATAATAACTAATGCACTATTGAATCGTGCGTA

GCAGAGGACGCAAACGTCTGGAGCCAGTGGCAGTGATCATACTCTCGGTG

ATAATGGCTAGTGTCTCAGTTCAAATAATGGTGGAAGCGGTGCAGACCAC

CTACAACATGGCCGCCAATGACCAGGGTCCTCCAACCATGAGTAATCTCA

CCATTGGCCTTGTCGCTTCCACAATCGTGGTAAAGGTCTCCCTCTTTCTG

GCCTGTTNNNN

>TSCB.R57.esd 699 0 699 ESD GOOD: 94-607

GCACGAGGCCTACCTCTTAGAGGGTGGTCGTCTAGACCCTCCTGAACATT

CGACAGCTGCCTCAAGCTCTTCATCAAACTCTGCGTTTTCCTGGTGGACC

TCTGGAGAAATACCACCCCAAGAGCAACTGGGGTGTCCTCTCCACATCCT

TTGTCGTCTGGTGCATTTTAGACAGTCGATTCTCGATCATGACGATGTTC

AACAACTTTGTCGACTGCTCAGTGAGTTTAGTACGTTTGCAAAAGCTGTG

ACTTCTACCAAACGATGGACTCGGTGTGCAAACTGTCCTTGCACTCTAGA

GGTACGCACCAAATCTCATACCTCTCATTCCTCTTTTTTAGGTTTATAAA

GCACTGATAGATCTGGGCTTACCTTTAGTGGAGTGGCTCTTTGCCTGGAA

GTTAAACCCAGACCTGAGAATTCTCTGCCTTTCGTCTTTTGGATGTTTGA

CTCAGGCTTTGCACACTCTTCTCCTTACGCATGGAGATGATACCTTTCAA

CTTATAACAGATCG

>TSAL.R77.esd 562 0 562 ESD GOOD: 105-456

GCACGAGGCTAAGAACGTGGTAGATAATGTCTGGAAGCGCGCTTAAAAGG

CTAATGGCTGAATATAAACAGCTCACTGACAACCCTCCGGAGGGAATTGT

GGCAGGTCCAGTGGACGAATCCAATTTCTTCGAGTGGGAGGCGTTCATTA

TGGGTCCAGATGGAACTCCTTTTGAAGGTGGAGTCTTCGCTTCGCGCTTG

TCCTTCCCTCCTGACTAACCTCTTTCGCCTCCAAAAATGCGTTTTACTAG

CAATATCTTTCATCCTAATAGTGCGTTTGCCTTTATCCGTTTTAAATTGA

TTTGGGCAGTTAAGTTATTTCATTAATACTGTGCTCAACAAATCTCTTCT

TT

>TSCD.R85.esd 518 0 518 ESD GOOD: 99-518

GCACGAGGCGCCAACTCTGTGACCTCCTACAGGACACTGTCTTCCCTGTT

AACCCGCTTCAGGTCTCCCTCCAGCAGGAACTGATTCTCTATGTTTCCGC

CCTGCTCATCAAGGACAAGTCGCTGTTTAAGGACATTGCTATAATTCGAC

TGGGTTGGTTCCTGGAGGCCATGAAGCTGCAACTCGAAGTGGAAGAGGCG

CAATCGGAGAATAAGCAAGGAGGTGGTGGTGACGATATCAAGGGTGCTGG

AATCAAAGTGGTGGGGAAGCTTGGAGATGCTGCCGAAGCGTCGGTGGAGT

CGCGTAGTCTTGGTGCATGTCGCCTGTATTCGCTTTCTCCCTCGAGTCTG

AAGAGTCTCCTACATCGCACTCTATGCTCCAACTCTGTGGAGCATATCCA

AAACTCCCAAAAACCTTGNN

>TSBA.R79.esd 636 0 636 ESD GOOD: 102-634

GCACGAGGGTTTCCTTCAACTATTAACTCTCCTCTCACCGAAACTGACAG

TGTCTACGTTTCAATTATCTTCTCGTGAGATATGCAACTGCATTCATCTT

CCCAAGGTTAAAACCGTCTTGGAGGAAAACCTACGAATTTTGCAAAATAC

AGACCTTCCCGAAGGATTTACGCGCGCGATGTTCGTAGAATGGGTACGAA

TCGCACATCGATTCTCCGCTACGGGACTAATCGAAAATAATTTGGATTAT

TTCGCCACAGGATTTGAGTTTTTAGCACGACTTAGTTGTGGCAGTTCCCG

CTCTGCAAACCTCGCTTTTCGCTTTGTAACTTTACCTCTCTACAATATGC

TTCGAATGGCACACTCAGAACTCCACGATCATACGCACCTATCCGTTAGC

CAGGTCTGCGAAGGAAATTTGCGAGAAATAGTAAATATATCTCAATCGGT

CGCCTCTGCCCTCGCAGAACCAGAATTAACATCTGCTATCGTGCGCTTGT

TTAAGGATTTCCTCTTACATTGTGCCTACTTGN

>TSAR.R46.esd 588 0 588 ESD GOOD: 104-497

GCACGAGGTACCGCGCTGATAAGCCTGGTTGACGGAAGTGGCAATCCGGT

CAGCAGTTCATCAATCACCGGATCGTAACGACGAACAAAACCGTTGGTGT

CCAGACAGGTATGAATGCCTTCTTTTTTGCAGGCGCGGAACCAGTCACGA

ACAAACTCAGCTTGCAGGATTGCTTCACCGCCGGATGCGGTAACGCCGCC

GCCGGAAGCGTTCATAAAGTGGCGATAGGTCACCACTTCCTTCATCAAAT

CTTCAACGGTAACTTCTTTACCGCCATGCGTGTCCCAGGTGTCGCGGTTA

TGACAATACAGGCAGCGCATCAGGCAGCCCTGGAAAAAGGTGATAAAGCG

AATACCTGGGCCGTCTACGGTTCCACAGGATTCAAAGGAGTGAA

>TSAR.R40.esd 584 0 584 ESD GOOD: 92-461

GCACGAGGGATGCGCGTAAGATTTGGTGCTTTGGTCCCGATCGTACTGGC

CCTAATATTGTTGTTGACGTCACGAAGGGTGTACAGTATCTTAATGATAT

CAAGGATAGTGTTGTGGCCGCCTTCCAGTTCGTCACAATGGATGGTGTTC

TGTGTGATGAAAACATGCGGGGTATTCGATTCAACATCGAGGATGTGGTC

CTCCACGCTGACGCGATCCACCGAGGTGGAGGCCAAATTATTCCCACTGC

CAGGCGTTGTTTCTATGGCGCTTGTCTTACCGCCTCGCCGGCTATCCTTG

AACCCGTCTATGTTTGCGAAATTCAGACGCCGGAAGAAGCTTTGGGAAGC

ATTTATAGTACACTTAAACG

>TSAC.R27.esd 648 0 648 ESD GOOD: 110-436

GCACGAGGGGATGTTGATCCTTCTTCATTCCTTCCTCTTCTTCTTCCCTT

TCGCACGAAGTTGATGCAAGAGATCGTGCTTCCAGTCTATAACTGTCGTT

CTCTCACAAATGCAGCAGGACTTCCTATGACTTCACTGCCTTCTCCTGCT

CCCTATCCTTGGTTTTACTTGCATTTCTTTTGGTTCCGATTCCCTACTCA

TGCGTAGTACCGCCGAACCCCTTTCTTTCGACTTTCCCATCCGTGTGTTT

TGCTTCATGGTCTTGCCTCCTCTTCTTTGATTTTNTTCGGTGCTGTGCTT

TGCGAAACAACGGCCTCTTTTCCTCCT

>TSAH.R76.esd 663 0 663 ESD GOOD: 122-387

GCACGAGGGCATCACCTGCTCGGTATTTTCTATTTAATAAGAGCAGCATA

TATTTAAGTAAAGAATGCGGAATAGTGCGTCAGACGTCTGCTTCCAGAGG

TTGAGCATTATAACTGGCAGATCTCAGTCGACTCCGATGCAGCGCTCTTT

AGGAATGATGAGTAAAGATCATGCTGTATCCAATGCTGAGATTCATCACT

GACTCCTTTCCCACTCACACACCATTGGAATTAAGACTTGCCAAATTTAA

CAGCATTCTGCAATGC

>TSAO.R19.esd 640 0 640 ESD GOOD: 108-460

GCACGAGGCTACATTCCTCTTGACTGTTAAAGCTTCACCTAATTAAGTGG

AGAGGTTGTCATCACCACACGAATGTTGCTGGCACGATGGATTGAGTTGA

AATGATAGCGTAACCACCAAAATTGTCAACGTTAGTGCAATCGGTTAAAA

AGAACCTTTCAATTCCTTTCTCTTTACCTCATCGGCACAGACAATTTCTT

TCTGCTTCGCTGACATCTGAATATGGTCCTGGAGTAAGCAAATTGGTTCC

CTCTAGTGCGAATGATTTGAGGTACCGTACCGGGTAGAGGAAGCGATGGA

GGTGAGGAGTGCGACGTTGCGGCGTTGACTGATCAGCATATCCACATGAA

AAA

>TSBG.R71.esd 766 0 766 ESD GOOD: 102-589

GCACGAGGAGCCAATTACATAACCTAAAATCAAGTTTGCACTACCTATAG

CACTGCTTAATGTGAAGTGTAGCATTTTTTGTGAAAAGTTAGCAAACTTA

CCCACCAGCCAATGTGATCGGTGTCCACCACAAAGGTGATGTATAGTGAC

CCCACCTTGAACGCGGTGTTTTATTGCTTAATGTTCCTTTATGGGCAAGA

CGAAAGGCGATAATCTCGACCTAACACGACGCAGCAATTGAAGTATCCAA

AATGTCCAAAATAGTCTCTTTAATTTCAAATGTTGTGAGTAAACACACAA

ACAAAACTAGGCTTATTAGCCTGGTATAGGATTGGGTATTCCTTGTGGTG

GTAGATAACAAGAGCCCAGAACTGTGTATCGTACGATCTCCTTGTACCAC

TTTAGACATGGCCTTCGACGAGGAGGCCTCTCGCGCTCTGGTGCATTGGG

CAGAGGAGAGAAACTTTTATGTGAGTCAGAGGGAGCGG

>TSAK.R54.esd 702 0 702 ESD GOOD: 113-583

GCACGAGGGTGACGTTGGGGGGCTGCACATCTGATCAAATTAATCACCTT

TGTGCGCCGTCTAGTCGTCCCACATGGCTCTTCTCAGTATGCTTCAGATT

TTGCTTACATCCGTGCGCTTCATTCGACATAAACCATGGGTTCCGAAATT

TCGTCTTATCCGTCAGATGCAACCGTGGGTGCAGCCCTTCGACCCTGATC

TCATTTCGATTGGTGGCAAAAGTGGGGACTGAACGTGCTACTTCATCGGA

AATCCGTGAGCACTTACGTCGAAACGGTCTAATGCCTCCCCTCCTGTTTC

AAGAGCATCGCATNTAATTTGACTCATTCCNGGTCGTAATTTTTGATGAA

TATGTTCCTCCGGAAGGCGACGGAAAATCGTCCCTCCTTTCTACTGGGAC

ATAACGGATGGGTAGGGATTCAGTGGTGAAAAAGCAAAACTACGCGAGCA

GCACTCCGTATAAGGAAGCAC

>TSBH.R45.esd 566 0 566 ESD GOOD: 101-539

GCACGAGGGTGCTGTGTGCCACAGGGCAGGCCTGCTGGCTGGTTGCTAAC

GAGGCTCTCCAGGCTTCAATCACCTTTCCCATCGCTGCTACTCTCCCCGG

CGCACTCTCCACCCTCCTTGGCACCCTCTTCTTCCGCGAGGTTCAGGGTA

CAAAAAATTACATCAAACTGGCGGTGGCACTGAGCTTCACTTTGGCTGGA

AGCGTTTTAACTGGTCTCTCTAAATAGAACTGATTCTGAACTGTTTCGTG

CCTGGACATATTTTGATTTAACTGATTTTATTATTTGCTTGGAATAAACT

TCTCCATCGGAATTCCTCAAAGCTTGTCACCCATAATCGTAATGTTTGTG

TGTGTATTCGATTCTTTTTCTGTACCCTTCCTCCCCTCCATTTCCAAATC

AAAGTTTTTTTGCGCAAAAAAAAAAAAAAAAACTCGAGG

>TSAZ.R36.esd 745 0 745 ESD GOOD: 113-431

GCACGAGGCTCACATCTCAGGACAACCGATTGTTTTTGTTGGAACTGGTC

AGACATATTCTGATCTACGTCAGTTTTCTGTACCAAGTGTGGTCAAAGCT

CTCATGAAGTAGATTCCTCTTCCTTCTGACTGCTTCATCACCACCTTTTT

TATTACAATTTTGTCCCGCTCTCTCATGCGCGGTGCTGTTATCAGTTGAC

TTCTCATCTGCGGCTCAATTGATCCCGTACTCTTTTCCCGAAAAAAAAAA

AAAAAAAAAAAAAAAAAAAAAAAAAAAAAAAAAAAAAAAAAAAAAAAAAA

AAAAAAAAAAAAAAAAAAA

>TSBA.R82.esd 589 0 589 ESD GOOD: 103-570

GCACGAGGCGCATTGCATAGCAGCTGATGAAAAGATCGGGTTCTGTTGAA

GTAGCCAAAAAGAGGCTTGGGATGTGAACTCCCCAAGATTGACCAAGTAG

TGCAGGCACTGCTAGAACAGGAATCGAAATGCGAAAGTGTGATGTCTTAT

AATTTGTTTTGTTATGAAAAAAGCGACCGAGATCGGATATAAAGCTTTGG

TTACGCCTCTCAGACAGTGATACAAGTCGCAAAGCATGTCGCCGAGCGAA

CAAAGCCTTGTATACAAACGATTCTGACTTCCCTCAAAACCTCACTGCTG

GGCGGTGCTTCTGTTCACATCGCGATCTTCGCTGTGCTTATGAGAGCCTT

ATTAGCTTTCAACCCAGAAAGAAGTGACTTGCACAAGAAGAAAGACAGAA

AATAACTTTAGGCAATGACAAGCTCCTTTAGAGGACCAAAGTGAGGACTT

CCAGTAGGCAGTCGAATT

>TSAW.R17.esd 733 0 733 ESD GOOD: 96-426

GCACGAGGATTGAGTTCAATTGTGTTCTTTATCACAACGGGGGTCAAGCA

GTGGTTCTTTCAGTGCCTTACTTGCNTCGCCGAGACACCATCGCTCTGTT

TGGAAGAGCTGATTTATTGGAAATTCATTCCATCGTCTTTGCCACTGCTG

CCTCCACTCGACCCAAGGTCCATAGGAGCGCCCAGTTTGGGGCGTTATCA

AAGGGCNCCCGGACAGTTGGCCANTGTCGATGTGCTACTGGCAAGTCAAA

GAAGCTCAGCCCCTTGGGACTGCCCTATCGAACGGTGGGGGTCTGAGAAG

GGTGGTCAAACGCAGCACCCCCAGTCCCTCT

>TSAP.R85.esd 723 0 723 ESD GOOD: 98-694

GCACGAGGGCACCAAAAACCACAACGTATTCACCTGCGTCACCTTTTGGA

CAATGTAGTAATAAATCGTCCTCCAAGCACGGGAAGAAGGGATCCCGGAA

AAGGTATATGTCACAGTCACCGCCAAAACTCGGAGTCTGCCACTCAGCGT

GTGGTAAGCGGGTTTTGGTCGATTCAATCTTCTCTGACGACGAAGAGGAA

GATGCAATTGTCAACACAGGAGACAGCCTAAGTATGCGTTCACGGGACAG

TCATCGAAGTCATGAAGGAGGCCGACACAGCAGCAAGAGGCGAAAGCTCG

AGGGGGATCGAGCCCAAACCCCAAGTCAGATCTCCCCTCCAACTACTCAG

ATTATTGCTGCCTCGGCAGGAACGTCCTACTACTTTAACAATGCTGGGGA

ACAGATATGGCTTTGCCCCATTTGCCGCACTGAGGATGACGGTGATCTAA

TGGTGGGGTGCGACAGCTGTGATGACTGGTACCCCAGGTATTCTTTAAGC

TTTTTTATCCGATCCTTACTTTTTTGAGTGATCTTGGTTCTTGGATCCGT

TCCGGTGGACCTACAGTAGTTGTTTGGGCCTATCGAAGGAACCGATT

>TSAL.R27.esd 655 0 655 ESD GOOD: 104-387

GCACGAGGCTAAACCATGAGCTTTGACGAGGAGCAGCTTAACGATCTTTA

TGCGGCCTTTCCTGACCTCCAAAACAATGGGGCCATCGCATCGGACGATA

TTAGACCTGCACTGGAAAAACTTGGCTGCCCCATTGCCGGTCACGAACTC

AGAGAAATCCAGGGTGGACGAGCTCGAGGAGGTGCTCTTTGTGATATTGA

TGAGTTGTACGAAATCTACACGAAGGCTANATTTTCTAAAAATCGACTCG

AAGAAAATCCTCAAAGGACGCCTTATTCTCAAAG

>TSBU.R26.esd 842 0 842 ESD GOOD: 103-618

GCACGAGGAGGACTTACGCGTGAATTTGTTAGTCGATGGCGGCGTAGTTA

GAATGCAGAGTCGTCCGTTGGGCACATGGAACATTGAACAGACTCGTGCA

TCCTGGAATATTCCTCTAACCACCGGTGATGCCTATCAACAGCAGCACCC

TGGTGTCGATGTCTCGGGTAACATTCGTGCCAAATTTTTCCTGGCTCACG

GTCCCGGAACACCACAACCCGTCGCTCTGCAGTTCTGTCGTGATGGTGGA

TGTCTTCCTAGCGGAGCCATTCTCTCTCTCGGAGCAGAATCCTTGCCAGG

AAGAGGTGGCGGTGGATACCGTCTGACCATGTGCAAATACCGTCTCCTCG

GCGATCGATACTTCTGCGACCCACCGGTGCCTATGAGGGCAATGACACAG

TGTCTATCTCTCCCTTCTCCGCCAAGCTCGAAAGCACAGTCGTTCGGCTG

AATTTTTACCTTAATTTAGCACCAAAATGCCTTTCGCATAATATCGTACG

CTGTACAAAATGCTCT

>TSBA.R2.esd 621 0 621 ESD GOOD: 96-571

GCACGAGGCGAGAAGTTCTTTTCCACGGCTTTTAGACTAAGGCGCCAAGA

TTATCGACAAGGTCAGCACATAGCATAAGCGGAAAGTAGATAATTAACAA

AGACATATTTCGGCTGTGCTAAAGATGGCAAAGCTGTGTTGGATTGATGC

GCTCACACGGCGCATATGCGGTGACGCAAAGTGCTGAGTCAATGTAGGCG

GGACAGACTGAAGTGGAACAGCAAATAGGAAATTGAGTTTAAGTTAGAGC

GGGACAATCAGCAATAAGATATGCGATGTGGTGATCGGTAAATTGCATTG

AAGTCTAATCCGTGCTCAGTCTGCTCTACAACATAAACTGGCTACTCATG

AAGCGCTGAGGTACAAATGAAGGCGGACGATGACCAGTGGTAGTCAACAC

TAAATATAAAACGAACTCACCAGAAAGGTTTGGAATAGGTCGAAGCGAGC

GGCGGAGGTGAGAAGATGGCAAGCCT

>TSBN.R56.esd 599 0 599 ESD GOOD: 94-599

GCACGAGGCGATGCGGACACCCTGTTCCACAAGGGTCAGACCGTGATGAG

TGAGTGCGAGCGCTACGAGGCTGTCCGACACTGCCGGTATGTGGACGAGG

TCATTGTGGCTGCGCCCTGGGAGTGTACTATCGACTTCCTAAGGCTACAC

AAGATTGACTTCATTGCTCACGACGACATCCCATACGCAAGTGATGAGTC

GAATGATATCTACCAGCCTTTTAAGGACGCCGGAATGTAGATACTCTCCT

TCTTCAAATAGCACGCCAATGCGGTCTTTGCATGCTTTGTGAACGTTTTT

TTTTCCCACCAGGTTTTTAACAACACAGAGAAGTGAAGGCATATCCACCA

CAGATGTGATAGCACGGATATTGAAGGATTATGATGTTTATCTCAGAAGA

AACATTAGTCGTGGTCTAACCCGNGCATGATTTAAATATTAGCTATGTCA

AGGTAAGTTTTTTTAATGAAAACGTGTTATCGTTCACATGAAAAACACCG

AANNNN

>TSBU.R94.esd 779 0 779 ESD GOOD: 97-497

GCACGAGGAGTTCCTCTACGACACCGCTAATGGCACAGTTTATAAGGTGC

TCTCGTCGCGTCGCAAGGGTTTTAACGAGGCCCGCTTCTATGCGGAGGTA

TTTGCACCTGAAAACGCTGACGATGCCGTGCTCAGTCGTCTGAGAACCTT

CTTGCCTGCCTATCGTGGTCTCTTTCACCAACCAGATTCTGGGAGTAAGC

TCCACTGTAGATGTCATTTTCGCTCTTCTGATTCACCTACATATTGTTCA

CGCGAAACTGGGCTGGTTAACTTCAAGTCACAGACATTCGCTTTACAGAG

CTTGTTGATAGAGCCGGGCACCCAAAGGAACTATAATAACCTCTCTCAGT

GGATATGAAATACAAATTTCGAATGCAGTAAAAAAAAAAAAAAAAAAAAA

A

>TSBJ.R39.esd 211 0 211 ESD GOOD: 91-211

GCACGAGGCTCACTTGTTGTTGGTGACAAGGGTTATGTTGCTGGTGCACA

GGTGGTAGTTGACACGAATGATTTCAAACTGAAGAAACATACTTGCTCCG

GTTGGATACAGTGGAAAGGGT

>TSAB.R23.esd 682 0 682 ESD GOOD: 101-590

GCACGAGGAACACGACTTACGACTTTGTGGGACAGCTCATAGGGGAGATC

GCTAAGACCTTCCCGGATAGCGCATTGCACCTGGGCGGAGATGAGGTGGA

TTTTGCATGCTGGAAGTCGAACCCGGAGATACGTGACTTCATGGTGAAGA

TGGGTTTTAACTGGAGTTACACAAAACTGGAGAACTACTACTTTGTCAAA

CTACTCAACAAAATTAAGGAGATCACGAATAAAACTATGAAGATTTACGT

GTGGCAAGAAATCTTTGACGATGGTGTCGAGGTAAACGGTTCAACAACTG

TCCACGTCTGGAAGAACGGAGCATGGAGAACGGAAATGAATATGGTAACG

AAAGCGGGCAAAGATGTTATTTTTTCGGCTTGTTGGTACCTAAGCAGCAT

CAGCTATGGCGAGGATTGGATCAACCGCTATCAGTGTGATCCGGCAAGCT

TTACGAATAACACTAAACAACTTGAGCTGATCAAAGGCGG

>TSBD.R7.esd 572 0 572 ESD GOOD: 98-363

GCACGAGGTAATACTGTGGCTTTCTAAAACACTCATTGTAGAGCCACAAA

CACTACATTTGAATCGTCTCGAATAGTACAAAAGATTGTTGGATGTGTGA

ACAAATGAAAGGCTTAACCAATAAGAAAAATCTGAACAGTCGCTCTCTGT

TTAATTTGAAAAGAACATAGGGGCACCAGATCAGCGAACGTAATCATACT

GTAAAATGCGGCTAGGCTATAAATTACACTGCTGACAGCGGTATTTGATT

AAATTGGATAAAAGAA

>TSAA.R4.esd 699 0 699 ESD GOOD: 101-557

GCACGAGGGACGGTCCTCATGCTAGCTGAGCAAATGCTCTGGCGTGTTGA

ATATGTTCACTCCAGAAACCTCATCCACCGTGATATTAAACCGGACAATT

TTCTGATGGGAATCGGCCAGAGATGCAACAGGCTTTTTTTAGTTGAACTT

GGGTCTTGCCAAGAAATACAGAGACAGTCGCACAAAGGTGCATATACCAT

TTCGGGATGACAAGAATCTCACCGGTACTGCCAGATACGCGAGCATCAAT

GCGCATGCTGGTCATGAACAGTCAAAGCGTGATGATATTGAATCTCTGGG

ATACGTATTTATGTACTTTCTTAGGGGCCAGCTTCCATGGCAGGGCTTAA

GGGCTGCGACAAAGAAGCAAAAGTACGAGAGAATCTACGAAAAAAAACTG

GGTACATCTCCAGAGCAACTGTGTCGAGGCTACGCAGTGGAATTCGAGAC

TTACCTT

>TSBN.R22.esd 389 0 389 ESD GOOD: 29-150

CGCGGTGGCGGCCGCTCTAAACTGTGGTCCCCCGGCTGCAGAATTGGCAC

GAGCCCACTGGACCGCTACAAATGCGCGTTGGGGATGGGTCAAGGACTGG

AAAGTTTAATTTTAAGGAGTCG

>TSBW.R91.esd 501 0 501 ESD GOOD: 87-501

GCACGAGGCTACAGGGGAGGTGGTTTCAAATAACCGAGAATCAAAGTCCT

CTAAAAATAAGAAATCGAAGGCAAAAGGTATTTCCACTGGAGCCAATAGG

ATCCAGTTAATGGAACAGAAGAAAGTGGAAGTGGTGGAATCACCTCCTGT

GGTAGAGGAAGAACTCGTCGTTAAATCAGTGAAGGCTTGGAGTGAGGGTA

AGGGAGAGAATGGGAGTGAGGAAGTGAAACGTCACCATCTAAGAGTAGAA

AGGAAGCGACTGCGGCAGATCGCTCGGTTAGCCGAAAAAGCCGCGGAAGG

CCATACTTTGAACCCTGACCAGCAAGCCAAACTGGCTCGTCGCGCCGAGG

TTGAGGCCCTCATCGCCCAACTGGAGGCTTCCTGTTCTTTCTCATCCAAC

TAACTTTGCGCCCNN

>TSBL.R12.esd 471 0 471 ESD GOOD: 94-471

CCTCTATCCTCTATATCCATTCCAATCTGTCCCCTCCGCCCTATCCCTCA

TTATTACTTCATTATATTCACGTGGTTTGTGTCGGGAAAATGTCTATTTC

CTGCTCAAATCCATTTGTGTTGCGTATCTCTACGCTTTACACAACGCACC

GTAGGCGACGGTGGTGGTGACTAATCCCCTCATCTGTAACCCGTTTCCGC

TCTCCCAAATCTGTCACCTCCTCTTGTCAAGTGTCACTCCACCTCTTTTG

TATGTGTGAGCACGGCGCATCTGTGTGTATGCGCGAGTGTGCTGTCATGT

CTCTGTGCTGCGAGATTTATATCCCATCCACTGATTTTTCTAGGAAACCA

AGAATTCCGCAATGAGACAATGTTTNNT

>TSBN.R76.esd 718 0 718 ESD GOOD: 102-201

GCACGAGGGCGACATGTGGAAGAAAGCTACCGGCGCCAGTATCGCCTACT

TTCATCACTCCACCCCCGCTCATTACTCCCCTATGATCATTTCGCTGAGG

>TSCC.R39.esd 422 0 422 ESD GOOD: 99-422

GCACGAGGCTTCTTTATGTCCCCACTGAGCATATACTCGGCGCTGTCGCT

TGCGCTGGCAGGATCAGAAGGAGACACGAGGCAGGAGTTGGTTTCAGTGC

TGGGACTTGCGTCCGGCAGGGACATTGACACCATAGTCAAGTGTCTCGGT

GAGGATTTACAGGCTGTGACTAGCAGTGATGCGAAGAAGACGTTGATTGA

GGCGAATGGTGTCTTCATCCAATCTGGTGGTCACATCAAGGAGGCTTACA

GGGGTGCTGTCAGTAAGCACTTGAGGGCTGTCTTCAAACAGTTGGATTTT

AGTAGTGATCCAGAGGGGTCGCNG

>TSAJ.R73.esd 558 0 558 ESD GOOD: 109-492

GCACGAGGAGAAACCGAAGCCCCGGGCGGAGAATAAAGCCAAGACAGAGC

TTCCTGCTCAAGTAGGAAGTGAATCTGAGGAGGAGGAAGAGGAGGGGGAC

GAAGAAGAAGAAGAGGAAGAAGATGATGATGACGATGAGGAAATCGATGA

GGGTGACAGTGGGGACGACAGTGTCGACGAAGAGGCTGATGAGGTTGAGG

GAGGTGACGTGTCTGATAGTGATGCTGAGGAGGACGGTCCGGTGTCCAAG

CGGTCAAAGCAGGACCAGACTCCTGCTGCTAAAGGTCGTGGTGGTAAACG

TGAGGCTCGCGGAGGACGTGGCGGTGGCTTCGTAAATCGTGGAAATCGTG

GCGGTGGGCGTTTCCAGAATCGTGGTGGCCGTGG

>TSBC.R73.esd 460 0 460 ESD GOOD: 105-427

CACGAGGTCTCATCAAAAGGGATTTATTCATGCGGAAAAAAGAGCCATTT

GGTATTGCTGGTGGATTCTTTTGGGGTTCTTGTCCTCATGTGGCTTTGGC

TCAGGGCTGCATACATTCGTGTTGTATCTTGGTCCCTTTATCGCTCAAGT

AACGCTAGCGCCTACGTGTGCCAATCTCTCAATTTTCCGGAACCTCCCTA

TCTGACGAGCTCATATGCCCAGAAAGGAACACAACGGTGGGGATGTTTCT

TTCTTCCAAATTGTTCCGGCAAAAACGAATTGGAGTCTGTACTCTGGGGC

TTCGGCACAGCAATTGGCGAACT

>TSAT.R52.esd 644 0 644 ESD GOOD: 101-338

GGCGGCCCTGCTAAAGGGACACGATTTCGGCCACTTTCGTTGGAAATGCC

GAAACCACTGTTCCCAATTGCTGGATACCCCATGATTTATCATCACATTG

AAGCATTTTCGAAGATACCCAACATGAAAGAAATCATTCTTATTGGGTTT

TACCAGCCGAACGAAATTCTTCTCTCGGCTCATAAATGATACCGAAAAAG

GAGTTTGGCATGAACATTCGCTACTTGCAGGAATTCAC

>TSBH.R8.esd 513 0 513 ESD GOOD: 88-508

GCACGAGGCCTCGTGCCGAATTTGGCACGAGGCCATGGCATCACAGTATC

GTGATGACAGAGGCAGGGAGCCGCCCTCCGCCTCCACATCCACGAGTGGG

AGTCGATCGACGGAGAGCAGCAAGACTCGGTGCTGGCCCTCGAATGCTGC

AGCACCAGCGGTGTTGCGAAGTGTCCTGCTACAGCGCGCATCGGGTGAGG

AAGGTTTCGGCTTCGTCCTCGCCAACGCACCTTCCAAACCGAATAAGTCA

AGGCGGGATGGTCAGGTCGACACCAGGTCTGGCGACCACTTTATCTCCAG

AGTGGTTGCAGGCAGTGCATCAGAACGATCACGTCTTCACGTGGGCGACC

GTCTGCTATCCGTCAACGGGGTCAACGTAACGAGTCTACCGCGCGAAGAG

GTCATTCGTCTGGTCAAGGCC

>TSBZ.R28.esd 500 0 500 ESD GOOD: 97-495

GCACGAGGGTCTCCGGCACCAATCTGCACAAATGGCAACTACGAAGTACA

CCCGATAAATAGCACGCATTCACCTACCAACAAGCAAAACAGTGACTTCG

ATAACCATGTGTTTACTACGCGGTGCATAAGAGTAATAAAGGTTCGCTCA

CCTCCTACTTTAAGAAGAAGAAGAAGACAACCTAAGCGGAAGTTAGATTA

CTTTGCGAAGTATATGCAGTTTGGTGGGGTCAATCCTGATACCGCCGCAT

CCATCGCCACCACCGTGGTTCATGCTGCTGTCGATGCCACCTCCTCCAGT

ACCACTAGGATCGATTGCAGCCACCAAACCAACAGTTTTCTCTGTAGGCT

GATTGTTGTACAGGTTGTCCTAAGGCCTTACACTGAGCCATGAAACAAA

>TSBP.R50.esd 685 0 685 ESD GOOD: 106-357

GCACGAGGCTTAGGTTTCTCTTCAACTTAGCCTGGAATATGAATGTGAAG

ATCTTCATACAAAACTACGAAGGACTGGTAATTCATTTCAGTTTTACTTT

CTCAAGAATGTTGCAATAAAATACACATAAGAAACCTATGTACATCCACA

CATGCCTTCGTCTCTATCTGTTACTCTGATAGACTCGGTTATGATTTAAG

AATGTTGTATTGATAGGTCCAAAAAAAAAAAAAAAAAAAAAAAAAAAAAA

AA

>TSCH.R92.esd 193 0 193 ESD GOOD: 51-167

TGCGGCGTCTAACTATGTCCCCGCTGCAGATCGGCCGAGGATGCCTCATG

TGATTTGCGCCAGACGGAAGGCGCCTCTCTTCTTCATTCATTATATCTTT

TATAGATGAAGTTTTAT

>TSBE.R5.esd 485 0 485 ESD GOOD: 99-306

GCACGAGGGTTTCTTCGGGGGTGGTTGATTTTCTGGTGACGTAAGGGACT

CGTTCATATCGCGCAAAATGTTTGAAGTGATCAACGCATCGATAGCCTCC

GCAAATTCCAACGCTCTCCTCTCCACAGATGGGTGTGCAGTCTCACCAGA

CTAAAGGAAATAAAATAATAGTCACAAACTGCTACGTAAGCCATGTGGCA

GTAAAATC

>TSAI.R12.esd 637 0 637 ESD GOOD: 98-603

GCACGAGGAAGAGATTGGGTGATGGAAGAACATTCTGATGGTGTGAGGCA

GTCATTCGAGTTCCTATTTTATGAAATTATTAAATATTTTACAAATCAGA

CAAAGCAGCTTTCTGATGGACATACCATGGCTATGCATCTAATCGAAAAC

GTGGGCTATCGTATTGGCCAGCGGTCCATTGAAAGGTTGACGAAAAATAG

TCCTAGATTTACATCGGAGATAGAGATCGTCAAGTTTATTTGTAGGGGGT

ATTGGTCTCACCTTTTCCAGAAGGATATTAATACACTGAAAACAAATAAT

GCCGATGTATATGTGCTTATTGACACTGATTTCCCATATCTCGCCCGAAT

CGAGCAAAGTCAGCTGTACAATCCAAATGTTGAGATGATTCTGGCCTTCG

CTTGTGGACTGCTAAGGGGTGGTCTGTCAAGCCTCGGCGTTAATTGCATA

GTANATGCGGAAATACCAAGGATTCCAGCGTGTCATTTCACTGTCCGAGT

GTCACC

>TSAO.R73.esd 648 0 648 ESD GOOD: 100-416

GCACGAGGGTCCCAGCTTTGATCTGTAGTCAACAGATTCTGCGAGTACTT

CTGGTCGCTTTAGTGGACCCATTAATGATTAATTTTTTTGCACAATCGCG

TAAAATGCAGGGAGATTTTAGCGCAAACAGTAGTGTATCTTTAGCTTCCA

GGTAGGCTGGCAAGGATTCTAGCAGTCTTTCGCTCTACCAGAGTAGAATT

GGAAGTAGACTTTGATATCTGAACTCAATGGTGCAAAAATGAGCGGGAAG

TTCTGTAGAGCCTTCTGCTAGTAAGTCCGCAGTGTCGCGCCCTTTTTAAT

GACAAACACTATGCAAA

>TSBA.R62.esd 652 0 652 ESD GOOD: 101-552

GCACGAGGTTTTTCACGTAATCCAAACATTTCTTTAGAAAAGACAGTAAC

AGAATCCTGGTGTACAATATGCTATTCAAATCATGGTTTTTAACAACTTG

TTGATGTAGTTTTCCCGATTACCGAAATCACCACCCTCAACAAAGTGTCT

ACCCTTCTTGCGGAAGCCACCCGTCGGATTGTTCAACTTGAAACGCCAAA

GGAAGGACGAGGCCGGTTTAAAGTTAGGACCAACCGTGTAAATCTCATGC

ACTAAGTCCTCAACGCACACAATTCCGAGGCGACCGAGCCGACGCTGGAT

GTGCTCGTTGCTGAGCGGCAACCTTTGGCCGCGAACTTTGCAGAAGCCGC

GCTTGTAAATGAGACGGCGAACGGTTCTTACGGAGGGATAACCCCAAGCG

ATGTANGGATCGATCAAGCGAAGCATGTTCAATGTAGCCTTGTTTAGCCG

AA

>TSAG.R28.esd 596 0 596 ESD GOOD: 98-559

GCACGAGGGTCGGTCTTACCTACGAGTGTTGTATGATCGTAAAATGGCCG

ATGACAGCACAAAGCTGAGCCCGCATTTGGGCATGCGAAAGAAACGGAGC

GTTCAATTTCTCGTCGACTGTCACCTTCAAGAGGCGGAGGGCATTGGAGG

AGGACAATTTGAGGGCTCAGGTTATTGCTCCTCTCACGAGGGTGACAATG

TCTCTGTTCGGAGCACGGGATCCTCTCACCGAGCAGTCGTCTGCCCATTT

CGTTCTGCTAGTCCGCGACCCTCCTCTGGAACTATGGATCCAGCATCAGC

AGATGAGGATGAGGGACGGTAGTCCAATTACCATCTCTCGACCTCCTGAT

GCTCCAGAGGACCTGGCGGTTTTGGAAATTGGTCTAGGAATGCTTAGACA

GCACACCCATCTACGAGGTGCAGTTCCTTCTTCCTGCTGCAGGCCGGTTG

ATTTGTGATGAT

>TSAV.R52.esd 641 0 641 ESD GOOD: 106-279

GCACGAGGGTTCTTACCCTACGCTATTGTATGGTGGAACAGTCTGCGACC

GCAGAAGGATGGGCCTACCTGCCAACTGGCCTGGAGAAAGTCGCGAATTG

CGTCACCCACCTTCCCTTGATACCTGTCAGCGTCTATCTAGCTGTCAGGC

TTATTAACAAGACAAGGACGTCTA

>TSAC.R80.esd 697 0 697 ESD GOOD: 106-567

GCACGAGGCCGCGATGAGCGCGTACGGATCTCCCTCTCCTAAGCGAGCTC

TGCTACCATCTTCAACGCCTGCTGTAAATAGGTTCAAAAATTTGATTTCT

CCTCAATTGGATCACAGTAATAGAGAAAGTCAGAGTGATGACAAGTACTC

TACACGTCTTCATGAATTCTCCCCAATTTTTGGAACTTTCCAGTCGCCAT

CCAGTACTATTCATCGGTCTAAGGGACCAAATAACTCAATTGCATCTTCG

TTTGCGAACAGTTTAGACCACAGAGACTTATCAAATTCGGAATTACTTGA

TAAGCTTTCAAAGGATCCTTCGGCGTACGCTGCTCATCTTAGGGACTCTC

TTGTTTACAGTGAACAAATACGGGCATCTATTTTCCAGTCCTCGTATCAG

TCGTCAAGTTCTTTACAAGCTTCATCCGCGGAGCCCGTTCCTTCGCATTC

CGCGCTTCTTGA

>TSBH.R65.esd 607 0 607 ESD GOOD: 95-270

GCACGAGGGTCACACTCCACTATGGACTCAAAAATTCGACCCATCCATCG

CAATTCCTGGCCACCCTTTCTATGCACCTATGTGAGTGCTTCCGTTCGTG

CAAACATTACGGGCAAGAAATTTGGCTCCGGAGATTCAGTGCCGAAAGAA

AGGAACCAAGACTAAAGTATGGGCAG

>TSBG.R5.esd 442 0 442 ESD GOOD: 89-442

GGCTGCAGGAATTCGGCACGACGGAGTCGGCCTCAAAGGCTGCGATTGCG

GAAGAATCGGAGGATGAGGAGGTCGATGAAACTGGTCTTGAGGCTAAAGA

CATTGAGCTAATCATGGAGCAGGCCAACGTTTCCCGATCGAAAGCTGTTA

ATGCGCTGCGCAAGAACAACAACGATATCGTTAACGCCATCATGTATCTT

TCAACCTAAATGCAGACCTATTCTGGAAGGAATTCGTGTGAACAAATACA

TGGGCTGGTTTGTACATTGCGAAGTGGTGTTTTTTATTTCATGGAGCGTA

TATTCTGGGAATGTCTTAATGCTGCTACCACTTTAGTGCCAATATATGCT

NNNN

>TSBS.R11.esd 676 0 676 ESD GOOD: 98-431

GCACGAGGGAGGCAATCATGCGAGCTTATTGTGGCTCTTGAGGCTTCTTT

TGACAATTTCTCTCTGAGGAATTTTTGTATTCAGAGTCGATCTTAGGCAC

CTCAAAAGCGATTACTAGTGTTGACCTCCTTGTTGACTGTTTTGTTGAGC

AATGCACGGTGGGGGGGGGGGAGCGGATTTGGCCTATATTCCTCACTCTT

CCTGACTGTAGTCGTCGCTTGATAGACGTCCACTCTACAAGCGAGATTCT

CATCTGTCATGGAGATAAAATTCTGTGCTTTTGGTGTTCGCACTCACTCC

CATCTACCTTATGCGTGATGGAATAGGAAAAATA

>TSAE.R60.esd 607 0 607 ESD GOOD: 94-605

GCACGAGNGGAGATGGTTACCCCTCCTATGGCGCCACCAAAGCCCGTGGA

GACAGTAGTGCCCATTGAACATCCTACAATTGACGCTTCGTCAATATCTC

GACTGGAGTACAAGGGGAGCTGTTCTGACGACTCAAAACAAGCTTTTCAA

AAATTTACAAATCCCCGCAACCTAGTAGTTGATGACGAAGATGCAGATAA

ATATAGGCGTCACTCGGTTGCGGCCTTCAAACCCAATGGTCGAGTTTTAG

GTTTGACGCGTTGCATCTCCTCGATCCCACCGCGGCTTCCTTGCCGGCGA

AGAGTGCTTTGCGTATTTGGCCTCGTTGTTCTCACCTGCTTCGCGACAAT

ACTAGTTACAAACCTGTTCGCCATTCCCTCACCCCCGGAAGACACCCTGT

TGCCTGCCCAANCCCACCTCCCCGCTGGTGCGTGACTCAGTCAACCGATA

CCAATCCGCTATTTGACTGTGATTCCCTGAATTGAACACCCTTCGAATTT

CCACTTTATTNN

>TSAJ.R39.esd 545 0 545 ESD GOOD: 97-540

GCACGAGGGCAGTACGTCCTTATGCTGTTGCAGTGAGTTATTTTTCATTG

CAATGGAGATTCCAGTGAGAAGAGGCGACCGCTCTGGAGACAGGCAACTG

GCTGTGAGGCATTCTAGCAGTGGTCGAGACACCTCCCATGAGCATGATTT

TCCTGTTTGTCCGTTCACCGATCTCAATCGCCATTTCGAAACAATGGAAA

AGCAGCTTGCAGATTTCAGGCCCTTTGACATGTTTCCCTTCTCTGGAAGC

AGTATGGGACCTTGGTTTACGCGCATCAATAATGAGATGCGAAGAGTCCA

CAAGGAGATGATGGATTTCATGCGCGATTCGGGCATGCAGCCAAATTTCA

TGATTTCTGGCACTTTCCTAACCATGCAGATTGCTTCACCAAGGATGAAA

ACGGGCAGGTAGTCATTACCTTTACATGACAAATCTACCAGGCT

>TSAR.R32.esd 659 0 659 ESD GOOD: 96-600

GCACGAGGATGTATAGATTTCAACTTTGTTATTCTCGATTTCACTAATGA

TGGCAACCGAGAAAGGCTTTTATATGTTAGCTTTAAATTTCAGTCCTATA

CGTGTTGGGTAGTAAATTTGTTCTTTTGAAGAGCTAATTCACTTTATTTT

TAATGGAAAACAGCAAGAGCAGTCGAGGCTAATTTGCGTTGACGTTTCGA

ATATGAATTGTTTTTGTTTTATCATGAGCATTTATATCGATCCTTCACTC

ACGGAGTTTTCTATCATTGAATCCCTTCCCAAAATTGACTCACGCCACAT

CCATCGCAATGTAAGCAGAAGCTTCAATTGCTCAAGGATGAGATATCTCA

ATGGCGTAAGGATCAATTCCACAACTGGTGCCGCCTCACCCAGTCGGCTA

TTGACGCCACNAGGGGAAGACTGGGGTGCTGAGGAGTGTCTGACCTTTGA

TCCCTCTGGTAGCCTTTTGCGCCTCTCCATGGCAGATGGGCGTCTGCGCG

TCGGT

>TSBC.R14.esd 407 0 407 ESD GOOD: 116-349

GCACGAGGATAACGCTGCAGCGTGATCTCATGAACAGCCGGCAATTAGAG

GCCGAAAACGCTATGCAGAGGCGCTTGCTATCGAAACTCTCCAGTGAACG

TGACGCCCTTGTCAGTGTGTTGAAAGAACGGGACTTAGGGTACGCTCATT

TACTTCAATCAATACGGCAAAACAACAAATCGTGGCTTCATGATGAGCAG

TTAACCCATCGTGAAAAAGAACTGGAAGCTCTAA

>TSBP.R33.esd 627 0 627 ESD GOOD: 90-557

GCACGAGGGTGCGGTGTAGTTTAGGTATGCGTTCTGGACTTCAGCCTTAC

AGAATTGGACCACGCGATTCGCGTCCTTCACAAGGTCATGGGTGTTTCTC

TCCAGCCGCACCAGCCAGTCCTGCTCAGCTGACGCATATTGAACCGAGCC

ATAGGCTGTTGTCCGTCTGTTGCCCTCAGCAGAATACTGATCAGTCTACC

CCAGGGCCATCGTGTGAATGTTATGGTCCATTTACAGCAATTGAATTGGA

TTCATCCCTCACCGTACCCGTCCCAGGAAACATGAAGTCTTTTCGGTTGA

CAATCCCATCTGTAGTGAGTTGGAAAGATCGGTGTTCCATGGATCCCTGG

NGAGAGTATTAAGCCTATATGGATACCTGATGGTGGTGAGCGTTTAGTTA

CATGCGTTGTCAACCAGCGACATACAGTTCTGTTCTCTCCACTTCAACAG

AAATTCTGAACTTCTCGA

>TSBQ.R32.esd 548 0 548 ESD GOOD: 89-506

GCACGAGGGGATATTCCAACTCTACGACACAGAATGTGACGGCGTGATCG

ACAAGACCGAGATGCTAAAGGTAGTCCAGTCCATTTACGACCTGCTTGGC

AAACACACAGACCCCCCCTACGAAGAGGCATCGGTGAAGGAGCATATGGA

GTTCGTGTTTAGTAAACTGGATATAAATCAGGATGGTGTCATAAGTCGAG

AAGAATTTATAGAAGCTTGTCAAAATGATCCCAATATATCAGACGCTCTT

GGGTGTTTACACACGACCATTTAACGTGTACCCCTTCAAAGTGGGAAGGA

CTGAGGTGTATGAACTCCATCACTGTGTATATGCAACAAATACAAATCCA

TAAGGGCTATACGTGCAATTCAGTAGTGGTTGATCCTGTGTCCACTCTCA

AATATTTCTTCTTCATTA

>TSBK.R48.esd 656 0 656 ESD GOOD: 103-651

GCACGAGGCTCTATTGAAATAGCAAATATGGACAAAGTTTTTAGCAGAAA

AGGTTCTTTTGTAGGCTTAACCGACACAAACAGCGAAAGTAACAGATACT

ATGGCTTGCCAACTAATGTTAGAGTTTTTATAAGAATTTAATGCATTTGT

AAACATTTCCTAGAAAAGTTTAAAATTTTACACTAAGTTCATTCAATTTT

GTCTTAATTAATTTGGGCATGTAAAAATTATCTGGATTATTTGATTTTAT

ATATTTCAAGGTTGAATATTTGTTTCGCTTTACCACAATAATACCGCGGC

ATACGAGCTTTGCTTGTCAAAATATTTGTAAAATCAACGAAAATTCGGTT

TTACTACATTGATGAAGTCTTAACTGTACTTCTTTGCCTAAGTTAGTTTC

ATCTTTTTCTTTTAAAGCAGTTGTCACTAATTTGGGTGGGCAATGAGACG

AAATTTTGCGATTCCTCGTGTGCCTCTTAAGTCCACTAAACACGTTGCAT

TTAAATTTTTTGGTGAACTCTTTTTAGAATCCCATGTTTTTCCCATATT

>TSBA.R46.esd 648 0 648 ESD GOOD: 113-647

GCACGAGGAGACTCTGATGTTTTAGGCATAAAATACTTGACTAATTTGGG

TTTCACTTTTTATCGAAGCTTTGTCACGAGTGAAGATACTTAGAGTTGCG

CCTTCTTTAGCATTGTCGCCACCCTTTTTTCTACTTCCATAAGTGGGGTC

CTTTTTTGACATGCTGCAAAATGACTGCGCCATGATTGTCTCGCCGACTT

TTTTGTGCAGAACCTGGAGTGTTTTGTTCTCTCTAATTACAGCAGACGAT

GGATTTACCTGACAGCAGTTCATCCGTCCACTAGGTTCCTTTTGCTAACT

TAGAGTGAAGGACTCAGAAATGGTGATCGGACGACTGTGGACAAGCAGTA

CTTCACGCCTGTTGTACTACGAACTGCTTTGTTTACATTTGGACCGTGAA

CCATATTGGAAGCTTCATGAGGAACTTTTCACCGACCTCGAAAGTCGTTC

AGCGCTCCATGTCTTAAATCTGCACAGGACTAATTATCCCAATGAGTTTC

AGCAGTTTGTTTTGGCAATTAATGCTGAGAAATNG

>TSAT.R35.esd 826 0 826 ESD GOOD: 108-456

GCACGAGGCCGTGACACCGGATATGTTCACCGCGCCTGTTTTACAGCTTT

GGAGACAGTAGCGTCAAGGGGGTCGACGATAGCGACGCCCCTTGGACCGC

GCGATGGCGATCCCGACCGCCGTTCCTGCCCTCGCTGCATCCGGCTGCGT

AGACGACGCGAGTTGGCTGACTCTCCCCTCACCTCCTAGCGGTTTCCTCC

TCCTTCGATTTCCTTGTGTACATAAGATTTTTCTCATCTTTTTTATGATT

AATATAATTTCGCCTGGATCTCAAGCTTAAAAAAAAAAAAAAAAAAAAAA

AAAAAAAAAAAAAAAAAAAAAAAAAAAAAAAAAAAAAAAAAAAAAAAAA

>TSBH.R67.esd 530 0 530 ESD GOOD: 98-452

GCACGAGGGATTCCTCTAAGGAGTCGGGAACGCTTTCGCCGCAGAGGTTG

GGGATGAAGGAAGAGGAGGAACGGGGAGACCCCTCTACACTGGTGGCAGC

GTTGGCGGACCTCCCCCACTTCAAAGAGGATATGAACTTTGAGGACTGGG

TGAAAACCGCTAGGTTTTACGTCCATTTATGTCCTCAGCGGTTGAGAGTT

CCGCTGATCCTCCGCGCTCTCCCGCAAGAGCTATTCTTGGCAGCGATAGA

CGCAGGGGTCACTGCTAACTCCTCCATCAACCATTGCTGCGAGATTCTCT

CTCAACTGGCCACCGATCAGCGAGAATAATCGCTGGCTAGAGAGTTCTTT

CACAG

>TSBC.R26.esd 534 0 534 ESD GOOD: 145-530

GCACGAGGGCCATTTTTGGCACAGTGTTGGCGGTGGACGCTTAAAAGACC

ACTACCACAGCTACTTTAACATCTGTGCACGCGTGCGAACAAACCAGCCA

GCGAACGTGCCCCCACCCCAGGCATCCGGCGGGTGGACGTGCGTTGTGTG

CGTGTGCCTTCTACAGGAGGGAAGGCGTGTCCCTGCTACTAAATTCCTCC

AACCCTCCCTCTCTTCTTGGCTCCTTCTGCTTCTTGGTACACTAATCAGC

CGTGCCCGCGATGGAAACAATGCATTACGGCACACTTACCTTGTAAGTTG

GCGAGTGCACCACTCCTTTCCCTAGCCCTCGCATCACGTGGGTTTGAATG

CGCTGCCGTCTCCCCAGGAAATGGCCACTCCATCGC

>TSAJ.R50.esd 701 0 701 ESD GOOD: 116-632

GCACGAGGGTGACAGCATTGGACAAGGCCCTCCGGGAGGCCTCCACTCTA

GAGGGGGTGCTAGAACTGCGAAATGAACACTTCTGGACAATCGGATTTGG

CGTCCTCGTAGGGAGCCTCTACGTACGTGTTCGACGTGACGCCTCCGACC

AACTGGTTTTGGCACACGTCACTAAGCGCCTTCATCCATTCGTTAAACAT

CTCACTATCCAGGTAATAAAGGACGATTGGGCACGGCCGGTAGCGGGATC

GAATTTTGATTGGTCCCAACCGACCGGCTATCTCCAGAGCGTGACGCTCT

TGCAGGCGCCCACTGGCCGCGTGCACTAACGTGTCTCCGTTCTGCCACTT

CTTCTGATGCTTTGTTTCACTTCCACTCCCCTATAAATGCAAAGTTCTTC

GTCTTTTCCCTCAGTCACTCCTCCACTAACCCTCCGCTTTTAGTTGGGGC

CATCTAGTACCATATGAACGGGTGATGAGCATTCTTGATTTCGACGCTAC

AAGATTGAAATTATAAT

>TSAY.R47.esd 563 0 563 ESD GOOD: 84-521

GCACGAGGCACAGGATCCCANCTAGTCCCCGTGAAGACTCGACTTTGGCG

CCGCGATTCTGCTGAACCCGCGTCACACAAACCAATCACCTCTCCGACCT

TCGCGGCTCCTTGGTCGCGAGCCAATCAGATACTGCCAATTGTCCGCACC

CATCGTATCGCCGTCTCCACATTGCCTCCGACCGCTGGTATCATGGAAAC

TGATATTGACAACGACTCTGTGTTGTCAACAGCAGCGATGGCGATTGCGG

TGGATGGTAGTCTCCGTCGCTCCGCTTCAGATACAATGCACCGAGCTCTG

GTTCGTCACCTCTGTGGCCAATCGCAACAGAGTAGCGCCTCCGCTGCTAA

CCAGTCATCATCCTCCTCGTTGATTGAGCGACGCATGGCGCAAATCCCGC

TGGTCCCAAGCCGAATCGTTCATACGTTGGCTCATGCT

>TSBK.R77.esd 630 0 630 ESD GOOD: 134-527

GCACGAGGGGTTAAACAGTAAGGAAACATTATTAAAGTGACTGGTGTTCA

ATGACTCTATGTACAGTGCCTTGCGAAAGTATTCGGCCCCCTTGAACTTT

GCGACCTTTTGCCACATTTCAGGCTTCAAACATAAAGATATAAAACTGTA

TTTTTTTGTGAAGAATCAACAACAAGTGGGACACAATCATGGAATGGAAC

TACATTTATTGGATATTTCAAACTTTTTTAACAAATCAAAAACTGAAAAA

TTGGGCGTGCAAAATTATTCAGCCCCCTTAAATTAATACTTTGTAGCGCC

ACCTTTTGCTGCGATTACAGCTGTAAGTCGNCTTGGGGTAATGTCTCCTA

TCAGTTTTTGANCATCGAAAGACTGACATTTTTTCCCATTCCTC

>TSBH.R84.esd 591 0 591 ESD GOOD: 82-589

GAATCGGCACGAGGCTGCCCTTCACAAAGCTGCAAGACATGGGCATATCG

GCTGCATGAAGATGCTCCTTGAAGCTGGTGCTTGTACAGATACCCGCAAT

TTCTATAAGCGAACGGCCTCTGAGGTAGCTGCAATTAACGGGCACTTCAC

GTTCGCATCACTAGTGAATGAGGCGGCTCGAATCAAAAAACGAGAAAATC

ATGTTCCTCCAAATGTTTCCATGTTGCGCAACAATAATGTGTTTGCATGT

AAGCGGTCAAGAGATAGTCTTGCCTCGGTGGGCGCTCTTGACGGAGACCT

TTCTGATAAACGACTGCGGTTTTCGGATCCCGTATTCCAAATTGCCGCGG

ACATGGTGGGACAAGGTGGACACAAGCTCTGGCAGAACGAGGTTCAATCA

AGCATCTTCGAGCATGCTGCCCAGCTGCGCATGACTGATGTCTACGAGGA

GTTGTACAACTCGCGCTACACAAACTTAATGAACTAAATCGTCTATACTC

GCACATNN

>TSBB.R65.esd 646 0 646 ESD GOOD: 97-521

GCACGAGGGTCGCACCCTCCCTGTGCAATATGACCCCTACCATAGAACCA

CAACTGTGATTTTATCTATCACTTCCCCACCTTGCTCACTCCTCCTCTAT

TACTGTATCTGCATCATTCAGATTTTCTCACCTCACTGTGCTTACCATCC

CTTTGATTCCCAAATCACTCACCCAACCAATCAATCAACTTTTTTTTCGC

AATTGAGTCTAATGCGAAATTGAGCCTCGTTCTCTCATTGTGCATTCCAA

TGCCTGTGTGTGTTGCGTGTGACTGCCTGTGTGCGTGCGTGCTTGTACAT

ATTTGAATGCGATCCCCCAACCTGGATTTTTGCGCCAAGAGGGATGAGCT

CATCCTGTTCAGAGACCTAATGATGCAAGCGATATATCTCTTTTTATCCC

CCTCCCCAATTCTACTCTCCTCCCC

>TSCC.R52.esd 476 0 476 ESD GOOD: 112-459

GCACGAGGAGAAGGCAAAGCTTAAAACGGTGTGTGAGGTGTTGATGACTG

CAACTGACGAACCGAACGAAGTTGCAGTCCAGTCGCCCACCACGATAGTC

AACGTGTCTGATGGTGCAAAAGTTGAGGCGTCCAACTCCAACAATCTTCC

TTCATTAGGTCATTCCCAGCCCTGCTTGTATGTTCCTTCCCCTCCCCTAT

TAAAGAATTTAACTTCCTCGTCAGTCAAACGTCCTGATCCTTCCAATCCT

CGGGAGCATACGGCACTTTGTTCCCCACCAAAAGTGCTCAAGACCACAGA

ATCTGTGGCAGTGGTGATGAGGGCACCTTTGAACCCATCTACGTCTCT

>TSBO.R74.esd 833 0 833 ESD GOOD: 95-480

GCACGAGGCAAAAAATTCAGTATATCAATGCTTCTCTCCAGCAGCTTCGA

ATGCTCCAGGGTCAATTCGCTTCCTCCAGAGATTGCTTGAAGAATTTCAA

TGTTGAGAACAAGGATAAAAGTACACTGATTCCCCTTACTTCTACCCTTT

GTGTCCCTGGAAAACTTGTCAATCCAACTCGTGTGCTTGTGGACATTGGA

ACAGGCTATTTTGTGGAAATGTAATGAAATGTGAGAACTGCATTTAACCT

GTGACGTACTTGTGATTTTTTGTTTTGTACCATTTCTGTACTTTTCTGAA

GCCGAGCCTTTCGTTAGTGTGATCAGGATCGGTTCCTGCTTTGAGTACGA

TAATTTTCTTACCTCTCCAAAAAAAAAAAAAAAAAA

>TSBE.R66.esd 656 0 656 ESD GOOD: 104-502

GCACGAGGGCCTCCCTGTTTTTGTGCACAAGATAACGCGCAATGGCATTG

CTCACCGAGCTGGACTCGAGCCTGGAGATGTGATTGTGAAAATCTGCAAA

ACCCCGCTTTCAGGCATGTCGCATAGCCAAGTGAAAGCTGAACTTCTTCG

CGCTGGAAGCGACCTTGATTTCACCGTCCTCAAACGTGCCTTCAATGTGA

CAAACTATAACATGAGCATGAAAATGATGGGCAGCCGAACAGGCTTCCCC

AACCGTCACTCCCAGGCGGACCCCGAAGAGCGATCTGAAATCGGTCAAAG

CATCTCTGGCGTCACGGTGGGCCCACTTTTCAAGAACGTCCAGCCCAAGT

CCCTACAAATTCTTGAACAACAGCTACCACAGTCGGAAAATGGGAGTTA

>TSBC.R60.esd 632 0 632 ESD GOOD: 94-610

GCACGAGNGGAGAATCTTANTGCCACAGCCCCTCATGGTGGAGCGACTTA

CCTGGGAGCCAGGGCCCCTGATCAAGGTTACCGATTTGAACTCGCTCGTT

GATTGGACAAGGAGCAGACGGAAGGGTCGGCTGACTCCCACATTTATGGC

ACCGAAGGACGTGTGGCGACTACTCTACCTCACCCAGCCCCTTAATCCAG

GCGCAGCGCCTGCTGCGGGTGAAGTGAAACTTGGCCACCTAGACATTGCG

TGGCGCACCACCATGGGTGAAACAGGTCATCTCCTAACCTTCCCCTGTGA

GTCCAGTACACCGCATTACTCCGATGTCTCGCTGAATATCATCAAACTTC

CAACACAGGTTGCCGTTGAAGAGCCTTTTCAAATGGTCTGTGAGATCACA

AACAGAAGTTCGCGTGTGCTAGAACTCCTGCTCTCTCGACCGGAGACAGC

CACAACAATGGCCGCGACGACGACTGCAAGCAGTAGTGGTTCTGGGACAG

CATCTGAGACCACTGCA

>TSCB.R33.esd 680 0 680 ESD GOOD: 97-589

GCACGAGGCTACGAGTTTTTTTTTTTTTTTTTTTTTTTTTTTTTTTTTTT

TTTTTTTGCTTGGGCAGCTGATACGATTTAGATCTATGGGATGCACAGGG

TAGACTCAAACGAGGGGAAGAAAGTAAATAATCGCTTTGCACACCAGTTT

TGCTCACAATTCGTCGTGTGAATAAAGTGCCTGCCCAAAGTCTGTCGCTT

GGCTTCCAACAAACACGTGATAATGCGACAGTATTTCATCCTTTGATAGC

TTCCCATCCTGATTATCGTCGGTCTCTGACATGAGGTGCTTCAGTTCGGA

TTCGATGTGATCATAGTCGGAAGGGAAAAGCCAGGCGTAAATTTCCTCCC

GATCGAGATAACCATCCTTGTTTTTATCGCGAGCCTCTTTAAACTGCTGC

TGCTCAGTCTTCACCCACTCCGGAGGCGATTGCGAATCATCAACCCACAT

GTCTCTAGTATACTCTTCCAAATCGATGCGACCGTCCTTATTT

>TSAF.R64.esd 465 0 465 ESD GOOD: 95-371

GCACGAGGGGGGCATTTCTGGCCACACACGATCACATTTGGCAGCGCAAT

TTAAGTTAGTAGAACAGAAGCTGTATCAGCTTTTGCAGATCGCTTTGCAC

CAGCGCACTTCTTCAATCGCAGCTCCTTCCAATCCTCCCAAATCTCCGGT

GATCACCAAATTGTTGGTGTTACACTGTCCTTTGGTGTTTAGCCCCTTCA

GCAAATAGATAACTAATCTATTTATACCATCCCTGGACCATTGATATGCG

TTGCACGCATTGCTTGAGCATGAGCTA

>TSAE.R40.esd 697 0 697 ESD GOOD: 116-475

GCACGAGGATCGACCGCTCTGGCTATCGAGTCGTGGCTCACTATTTGTAC

AGACATGTAATCCTTCAGGAGGCCATGTCGTGGCTTAGCACTTTGGGTGG

TGGATTCTCCTCTCTTGGAGACAAATTTAAGGATGCGGCGGAAATCGCTG

GAGAAATATCTCTGCGCCAGATGTATTTGGCTCTTTCAATGAAAATTCCT

GTCTTCCAAGCTCGTTGCAGGCTCTTTTTTGCTCAAAGTCTTATGCAACG

TGGTAGGCTCAAAGCGGCTGAACTGATAATTAGGGATGTCTATTCATTTT

CCAAATCTGCTTCTGCCTACGATAATCCACCTGAAGTGCATCTGGGTCTA

ATGTGTCACG

>TSBT.R71.esd 807 0 807 ESD GOOD: 102-669

GCACGAGGCGGAAGTTGAGGCAGGAGATGTCCCGGCATCAAAAAAGCCCA

AAGAATCAAGGTTTTACCAACAAAAACTGTGGGCATGGCAGCCTATTCTT

ACGGCCAGTAATATATGCCCCTACTTTTACATCGTTGCCATCCTATTTAT

TCCTCTCGGTGCCTTCTTTTTGGCAACATCTAACGGGGTTACTGAAATCT

CTATTCCTTATACCCACTGTGCTTCTTTGAGTTCCTCCGGAACATCCTGT

GCAGAACATGTAAAGGATCCGTCGTTGGGGCCTTGCCAATGCACCGTTAA

CTTCACCCTAGATGCAACTCTGCAGGGGCCGTTGTATGTCTTCTATGGAC

TAACAAACTTCTTTCAAAACCATCGCCGATACGTTATGTCTCGTGATGAC

GAACAACTCAACGGCAAGCCCATTACCGTTCCCAGCATTGATTGTGAACC

ATATCGTTACAGTGTGGTTGGCGGTGTAAAAGCAATAATCGCTCCATGTG

GAGCCATAGCTAACAGTCTATTTAATGATACGGTTATTTTAAGGCTGGTG

CGAAAATGAAGCGGGATT

>TSBS.R36.esd 732 0 732 ESD GOOD: 92-649

GCACGAGGTGGAGGTGATCGAGCCCCTTCCCCCGTCGGCCACAGCCTACC

CCACGGACTGAACGGAAGTGGGATTCGCTACGCTCTCTCTTCCCTATCTC

CTCCCCCTCTTCCCTCCTCTCGATCCCACAATATACCCTCACCCACGCAT

ACACCACGCGGTGGTGGGAATCGAATCAGTACTTTTCCCAACCAAAATTT

ATTTCGCCATCTGCGTTCTTCTCTGCTTTGCCGTCTCTGTCTTCTCCTCT

CACCTCATTTCCTGGAGGTCTCACAGGGACAAGTAGATTGATGCTAAGGT

CCTTCCTCCTCTCCCCAATTTATTTATTAAGATCGACCCGATCGCTCGTT

CCTCCCCTCCCACACGCGTAACTTTGGTCATATGCTATTATTTGCAGAAT

TGTTCAACTATGCGTCCATCTCTTTCAACGCGCGCCCCTGAACTGGCAGG

AGGAGCCCTCAGTCTTGCTTCTTTTCCTCTTACTTCTCTCGCTTTCTGTT

GTCTATTTACTGAAATACAATTAGGTAGCAACTGAAAAAAAAGAAAAAAA

AACTCGAG

>TSAO.R15.esd 546 0 546 ESD GOOD: 99-382

GCACGAGGAAGCGGTGTACGTTGAGCTCGTCTTCGTCCTCCTCATCTTCG

AGCATTAGCGAATCCGGAAGCTTCACCAATCCCCTCCAAAAACAGCGTCA

TCAGCAACTCTCTAGCCTTTCAAGTCTCAGCGAGCCGGATGATAAANCTA

CNACTGGTCCTTCCCCTGGTCCGCACCAGTCGCAGTCGATCTTAAAGGAT

ACTAAATAATTCACAGGCGGTCGGTCTCGATGCAGCGGGGCTTAGTTAGC

CACTACTTACACCTCCAGTGAATCCGAATCGGAA

>TSAU.R77.esd 695 0 695 ESD GOOD: 106-244

GCACGAGGCAGCCTTTTGTCTAACTGTAGGCATTAATAATGTGGACGAAC

ATCCCAGAGAGTGGCTGCCGGATTGCTGGTGCTGTGGCTGTGTTCGCACT

GCATATGCTTTTTGTGCTTTACCTCCACTTCGCCTATCA

>TSBV.R72.esd 418 0 418 ESD GOOD: 110-384

GCACGAGGGAATGATGGCANGAGGTTANGTATTTTAATGTTTAGAAATAT

GGAGCTTCTAGTGCTTCACATTCGTCAAGTGTTGTTAAAGACGTCGCTTA

ACTGCTATCAACCAGGNATTAAATATGCCAGATAGCATTGCAGTGCCGTT

GAGCGAATAAAANATTTTAAAANNAACTCCAAACAAGGAGGAGGACATTT

TAAACCATAGTTATGCCTTCTGATAAGCCTAACCAGAACATTCGGCACGT

TCATTCCTAGTGCAGATTTGCAAAT

>TSAR.R45.esd 681 0 681 ESD GOOD: 104-583

GCACGAGGACAGCGTCTACTTCAAACTTCGTCGACACTTGATTGAAAATT

TTCGGGCGGGCTTGGTAACTAAAATTTCATGCAACAATTGCTACGTTAAA

TTGACACCTTTGGATTTATCGAATTACATCTATACTGATTCGTCTGACAC

TGCAACTATTAGTACTGATGGTGGCAGTGAAAGCATTGCAAACGAGTCTA

CTTCCTCGCAATCATCAACAGCATCATTCGCTGATGACGATGTTGGCCCT

CAAGTTCCCTTCGAAAGGAACCTTCAAATAGCCCTCCCTCCACTCCCTAT

CCACGTTCTGGATCAACGGCCGGCTTCGGCTTCCTTTATGCGTGGCCGTA

CCTTTGATGTTTCAAAGCTGGATCAAGATCGCAGTCGTTAATTGATCTTT

CTGCGACTCACACTAATATAATGCTACTGTTCATTTTTTATGTGTACTGC

TTTTACCATCTTTGGACCATATTATTTAAA

>TSBG.R22.esd 749 0 749 ESD GOOD: 103-524

GCACGAGGCTTCTATCTTGGTACTGCAAGGCCTGGCACACTTCAGAATAA

TTTTTGGTGACCTATCATGCCCTCTGATGTGTGCCACATGAAGTATTAGA

CTCTAGTCACAAAAGGAAGTGGTTATTTCAGCAATAATAGTTTTCAGAGC

CCTCTACTGTTCTCTCTACCCATCCCTCAATCCCCTATCTCATCGTGCTT

TTCCCTCTTATGGCTTTTCCTACTTTTTGTTTTACACTTTTTCTGTTTTA

GTTTTTAAGAATGTAGGTACAGTAGCAATAATAAAATCATAATAACAATA

ATAAATAAAAAATGTGTACTCTGGGCAAACAAGAAAGTTCAAATATATAA

GTCAACTTCAGTGCATATCCATAACCACAGTAAACTTATAATAATATTTA

AAAAAATAAGAAAAAAATAAAT

>TSCE.R3.esd 589 0 589 ESD GOOD: 108-589

GCACGAGGCCTCTCTCTCTCTCTCTCTCTGACTATCACTAGCGATCTCAA

CATGCAAGACGCGCCACTAGATGAATTTCAGTATGCGTAGATCCTAATTT

TACACGTGACTCTGAGAGTGGGTGGATAAGTGTGTGTAAGTATATTTATG

ACTGTCATACTGCCATCCCTACTCCGCCTCCACCTCCACCTCCCCCTCCT

CGTCCACCACCACCTCTATTGGCCCTTCTTTCTGTCTGTCAGTCTACTTC

GAGTTTTATACCAGTAATGACCATTTTCATTTATTCCCACTTTATATTAT

TACCTATGTCTATTACCTCGATATGATACCTTTATGTATGCCTATGTATG

CTAGTGCACGTATGTGTGTGCTATTTGGGATCCGCAGACACATACACACA

CACACACACACGCACACGCAACACATGTCCATGAATCATTTTTTCATGAA

GATTTTCTTGGCATTATAAATCCATCTCCNNT

>TSAN.R75.esd 573 0 573 ESD GOOD: 100-443

GCACGAGGCGAAAACGGTATCTCAAAATGAGGAGAATTTGTGGAAGAGTT

GGTTGGAATGCTTTGGTCGGCATCATTTTTTACCAAAATTTTCAAGGCAA

CTGGTCTCACAGGTACATCTTGCCCGCCTACCCAATCCAGCTTATTTGAC

CGCTGTCGGGACTCTCGAAACAGACGACACGAGCTTGAGAGAGGCCCTCA

ACAGTTGCAAACTTCTTTCACCCCTTCTTACTGCCACTACTGATGCTGTC

AAGCTCCCCACTGATATAAACTTGGATCCAGTTGCCTGTCGACGGTTCCT

CAGCAGAATTGGGGACTGGAGAACGTCAGACTTTCCTATCTCCA

>TSCC.R2.esd 639 0 639 ESD GOOD: 106-548

GCACGAGGATTGTAATCGTTTTTGGGGTTGAGGTAAAAATATAAAGATTA

GAGCAGTAATCTTAAAACGCCTAACCCTTGGGCTCGTGCTAAAGTAGAAA

ACTAAAAAGTAGTTTAAAAAATGATAAACTGTAGGAATCGATGTTGGGGC

TAGCGGGAAGCAAGCCGAAGTTGGAACTGGCACCAGTGAGTAGTGTTGAC

TGAAGAGATTACGGAAGATACAATTCTTACCTGCCCAAACTCGCAAGGCT

GTCCAGATTCGCGAAGAGATGGAAATGCATCCGAAATGCGCCAAAGTACG

TGTTCCTTCCAGTTGGCATTTTTCATTGAAGTTATTTGTGTCCACAAGCA

CGACAAGTACTGTGGATGGAAAAAACTCAGAATGGAGAACAATTATTTAC

TTTTGATAGACGTACGGAGTAAACCGATACTTACATCCTCCTG

>TSCG.R75.esd 468 0 468 ESD GOOD: 111-467

GCACGAGGCCTCGTGCCGAATTCAGTCCCTGTTGCATGGGTAAAGCCAAA

CATCTTGTCGGAGGATGACAGCTGCACTCGCATCAAGCCATCGCCTTTGA

GCGCATTGGTCAGCGTGTAATCTCCTGCACTGTTCGTTGATGCGAGAATG

TCGAGCGTACCGGGGTTTGCAATGGTTACTTCACTCGACCCCATGCCCGT

TACCCCATCTGCCGTATGAGACGCAATGGTCAGGAGGCCGTCATTGACCA

GCGTTTTACCGCTATAAGTATTCCCTGCATTGAACACCGTATGGCCTGCA

TCATGGATCACTTTTCCGTCTTTATCGTCACCTGTAATCAGCATATCGAC

CCTGGTN

>TSBY.R58.esd 799 0 799 ESD GOOD: 96-663

GCACGAGGGTTATCTTCTCTCCAGATGACTTTGTAAAAGCATCGAAGTTT

GCCACAATGGTGTCGGAGCTGTCTGAGATGGGCTTTCCGCTGGATTCTGT

TGTTTCGGCGGTGCAGTCATCGAAAATGGACAAGGAAGAGGCGGTACTGC

GTCTTCTGGACTCAGAAAAGCATTCTGATGTCACGCCGTCCCCCAGCTAC

GCATCGAATAACGTCGGATTTCCCCAAGGCAGTGTATCGGACGCAATGCC

TAGACAAGGTGGGAGGAAGAGTAAAAAGAAAGAAAAGAAGCCCCATCTTT

CATCGTATCTGCATGCGAGGCACTAAATGAAGGCCTGCCTTCCTTTAGTT

AGAACAAATCCACGTCTTTCAGTCACCTGCGCATCTCGCCCACTCCTCTG

CCCCTGCGTCTGCTTAGCTTTGATTTTCCACCCTTCTGCATTGAAAAGCA

TTTCCACGATGCCAGGGTGATTTTTGTTGACACATCGCCCTTTTTCGTTG

CCAATCCAACATGTGTAACTAAATTGTAACATTAGCAAAAAACACAAAAA

AAAAACTCGAAGGGGGGC

>TSAG.R4.esd 656 0 656 ESD GOOD: 99-294

GCACGAGGCCCCATTGTTATTACTGGTTTTCTCCTAGTTACCGTGCCTAT

AGTTTGGATGGCTTATGACATCGCCTTTGAAACCGACAAAGACATTGAGC

CCAAATAGCTTCTTCTGCATCCATAGTTCCTTTGTTTAGTTAAATAAACC

TTTTTGCGTCAAAAAAAAAAAAAAAAAAAAAAAAAAAAAAAAAAAA

>TSBK.R32.esd 656 0 656 ESD GOOD: 122-622

GCACGAGGCCGAATACGGTGTATCGCGCGGTATTGACTTCAAATTAGTCA

GCAACGTTCTAAACTTCGACTTTCCACTGACGGCTAAACGGTACGTGCAC

CGAGTTGGGCGTACAGCACGGGCCGACCAGATGGGCACTGCAATCTCTTT

TGTTAATAGTTCGGAAGAGTCACAGCTTTCGAAAGTGGCCAATCTCCTCT

TGCAAAAGGAGGCTTCCAACCCTCACTCTGATCAAACTAACAAATCAGCT

GTTTCAGTTGATTTAATCTTCCGCCCCTATCAGTTCCGTCTGTCGGAAGT

GGATGGATTTAGATATCGTGCGATGGACGTTGCTGGAAAAATAACTCGCA

AGAGGATCCGCGAAGCGCGACTAAAGGAGATAAAGTTAGAGTTGATGAAC

TCCGAGCGCTTAAAGAGTTACTTCGAGGACCACTCGGCTGATATGGATGC

TCTGCGCCATGACAAGCCCCTTTCTTCGTCACATCAGGCTCATCTTAAGG

A

>TSBV.R95.esd 534 0 534 ESD GOOD: 110-516

GCACGAGGATCGTCCACCCCATCTTAATCCAGTATTTTATGATGGTGACC

CTGCAGCTCCAGCAAAGAATGTCTCCAGAGAAAAGCGCAGAGCTGCTAAA

TGGCTGCTAGAAAATGCCGATGTAGTGGAGGAGGAAGGATTTGCCTACCG

AAACTTACTATGGCTCCTCTAATTGTGACTCGAAATTCCTTGCCAAAATG

CGGGAAAGAGAAAAATACGAGGAGGAGAACTTTAACCGAATTTCCCTGAC

TAAACGCGATCGGTTGATGCAGAAACGGCTGGAGCGTGGTGAATTGCTCG

GTACTGGCGTACGCATGGTCACATTCGAAAGCTCCTTGAAAGGTCGGATA

ACGAGGAAGAAATGGCTCTAATTAATCCCAAGAGGAAAAAGAAGAAACAC

AGAAAAA

>TSBN.R10.esd 467 0 467 ESD GOOD: 94-466

GCACGAGGGCAGGGTCAAACCCTGGCCAGAAATTGGAATTGTGTTTTTAT

GGAGACCAGCGCCAAAGCCAAAATCAATGTTAATGAGGTGTTCTTTGATT

TGGTGCGTCAAATAAACAACCGAACGCCAGTTCCCAAAAAGACACGGACA

AAGAAGAAATGCATCATCTGCTAGGCACAGATGTCTCCTTCCCCTCTTCA

GCCACCAGTCACCACGTCTAGTTTCTTCGTCCGTCAAAGCCATGCAAAGG

GCAGTGGACTCTCCANTTTAAATCGTACGCCAACGCCGCCTACCTAACTA

CACTGCTCTCCTTCCTTTTTAAGAGATGACAATCTGCGCACTCGCTTGTC

CCGAAATTTGCCCGCCTCCATNN

>TSAI.R33.esd 594 0 594 ESD GOOD: 100-471

GCACGAGGCTAACGAGGATTCCCTCAGTAACGGCGAGTGAAGAGGGAGGA

GCCCAGCGCCGAAGCCTGCGGCAGGTTTGCTGTTAGGCGATGTGGTGTGT

GGGTCGACTGGCGGGACCACCGCTCCACTCGAAGTCCAGCATTGAGTATG

GCAATCAGTGGATATGGCCCATAGAGGGTGAGAGGCCCGTAGCAGAAGCA

GGAGTGGAGGCTCAGGCAAGTGGGGTGGTTCACCAGGTCGGCCTTAGAGT

CGGGCTGTTTGAGATTGCAGCCCAAAGTGGGGTGGTAAACTCCATCCAAG

GCTAAATACGGGCACGAGTCCGATAGCGAACAAGTACCGTGAGGGAAAGT

TGAAAAGTACTCTGAAGAGAGA

>TSBG.R29.esd 871 0 871 ESD GOOD: 102-228

GCACGAGGCTCCTCCTCAATGGATGAATGAGCTTGTTGTCACCTTGAGGC

CAAAACCAATCAGTTGCTGTATACAGGCGATACTGAGTCCGCCCACGTCC

GCGACAGTCTAAATTAACCAACCAGCT

>TSAG.R38.esd 587 0 587 ESD GOOD: 92-583

GGGCACGAGGGCGGGATGTCTTTCGCGCTTTTGGCTTCTAAGCACCTGAT

TCGCAGGGCTGCCGTCTCAAGTTTCATCTCAGCAAACGCCTGCAGAACTT

TGGTGTGCACTCCAAATAGTAACGCAAAACTGGGGACAGCTCCCCAGCCG

GTTACGTCCGGTTTCCTGCCTTCTCACCACTGGACGATAGAACGCGTAAT

TGCTGTCTCAATGCTTCCTATGTATCCCATTGCTCTAATCTACGAGCCCT

ATCTTATGGATTACGTGGTGTCCGCCGCCGTTTCTCTCCACGCCTACTGG

GGCTTTGGTGGTGTCATCCGTGACTACGCCATAGAGCGCAAATACGGTCC

TCTCATTCCCAAAGTCCTCCAGTTGCTCTGGAAGGCCATCTGCCTTTTTG

GATTCGCTGGCTTCACTTACTTCAACTACTACGACATTGGTGTTATTAAG

GGCATCAAAAAAATTTGGTCTCTTTGAACACGCACTGCTTAA

>TSBL.R59.esd 692 0 692 ESD GOOD: 99-599

GCACGAGGAGGCTGGCTTACTAGATTTGCTTGATGACCAGGTTGAATTAC

GGTCCTTTTCTAGACTCTCCATCCTTAGAGACGAAAATTCCAGTCTCCAG

TCAACTGTAGCCGCTCGAATCCGCGAATTAAATCTTCTAAACAGTCTACT

CAAACAAGTTAAACACGCGCCCCCAAAACGCCTCTCAAAGATCCTCAGTG

CCTATAACTTGGAGCATGGTCCCGATCTCTGTTCTGACAATCCCATCCTT

CAATCATCGACATCTTCAAGCTTTTCCCCTTCCTCTTCACCCCAGTCCAT

TCCATCTTCATCATCTGCAGCCACCAGGCTCATTTCCACCACTGCCTACA

GGCAACAACAGTAGGAAACTTACGATCATCATCACAATTCACACAATTAC

TCCGGTTGAATTGCTCATCGCCTCTCTTACCCTTCATCACTGTACATATG

TACTTCCTGAAAAACATGCGTTTTTGGTAAAGACACATTGTTTTTAAGAG

C

>TSAQ.R47.esd 706 0 706 ESD GOOD: 102-611

GCACGAGGCAACAGCGACAAGTGGAAGTGGGAACAGAATCGCCGGTGCGG

GTGACCACAAAACCTACTGAGTACCACCACCACTCTTCATCGTTCCTCCG

CAGTGTTTCCTCAAGGCTGAGCAAGAGCTCCAGGACCACACCGACACAAG

GGATCTCCAGGCAGGCGACAGAGGAGCGGGATGAGGCAGGCAAGCAGGGT

GCCCATTTAGTGACCACCGATTCGCCTATTCCTGCGCACCACCGCGCCTT

CTCCTCCGAGCGGAAACACGCCGTCATCACTACTGATGATCCCTGGAGTC

CCATGTTGCCTGAAAAGGGTCACTCTGAAGGAGAGGCGTTGGTAACCAAC

ATTAGTAACAGCTTGGAGCCACTGTTACCAGCCTGCTTTGATAAGAAGCC

GCGCAATATCTCCGTCTTCTCGGGGACCTGGCGGAAGGCCTCCTCCTCTA

CCACAGCCACCACCATCTTCGGCTCCCATCCTGACCGGTTAATGAGTCAG

CTTATCAGCG

>TSBI.R10.esd 718 0 718 ESD GOOD: 146-456

GCACGAGGCCAAGGAGCCCTACAACAAAAACCACTTGTCCCAACGCCCTT

TANTTCGTCTTTCGCTCCTCGATCTACAGCGCTTAATTGATTTGTCTCGT

ATTGATCCCACTCGTCCGATCGATTTGTCGACAATATGTAATACTGGTCT

CTTCCATTTGAGCGTAGACAAGGAGCGGCACTACGGATTTCACTTGACGG

AGGAGGTGAGTCTCAAAGACTGATTTTTTCTTGTATTTTAAGGCGCCGAT

GGGTTCGTGACACCAGTTAATATTGAAGTGCAGTACGCCAGCGAATTGGT

TATTGCAGCCG

>TSBL.R56.esd 365 0 365 ESD GOOD: 153-365

TCCGGATAAATCTGGTTACATTGTTTCAACAAAGGTGTCAAGTATGCCAT

GGGCTTCTCTCCACATTCCTGCTGTTCATACTTANGCCGATCTCCCTAGA

AACACTGTTTCACACACCGGCACCAACAGCATCCTCCTACCCGATAGCGA

CGCCAGACATAGTGAACGGCCGGGTCTCTACTCTTCCCAAGACAATCCCA

CTGCCGTCGGTAN

>TSAT.R19.esd 709 0 709 ESD GOOD: 120-633

GCACGAGGCGAGCTTAGCGACTGTGAATAGGGTTGACTCGGTAGAGTCGA

CAGGATACGATGCGCACCGAAATTCTGTGGGTTTCCAGAAGACCAAGAAG

GTCTTCACTAACCTCTTCTTTGGCAAGCATTCAAAGAAGTGTCGCAGTCC

CGCCACCACTCCCACTTCTGGTGGCTCCGGCGCGTCGCAGCACTCCGTAC

TGCCTTCTTCAACTCCATCCGTGCCGTCTCCCTCCATCTGTCCGCAACCA

CGCATCGATTCGCTGCCTCCACCTCACCCTACCGTCAGCACCGCCACTCT

GACCAACGCCTCGGATACCCTTTAGTTGTGCTCCGCCACCAAATGCGTGT

ACATGACGCCGCCGCTCCCTCCGACCTATCATCGTCACAACGACTCACCT

GTGTACATATCGCCTCCTCTTAGCTCGTCTGTCTGTCTGCATTTCGCCAG

ACTGGTTTATTTTTGTATGTATTTCTCTCTCTCATCCAACAAATAATGAA

GTGCAGGCAATAGT

>TSBZ.R23.esd 585 0 585 ESD GOOD: 106-406

GCACGAGGCTTAGAATTGCATCCTGATCCCACCGGGGAAATCACATACCC

TTTGAAGGTGTTGAAATTTGGGAATGTGCAGCATCTTGGTCTTCATATTA

GGAAGAACTTCGGTGCCGAATGCACTCGAATTCACTACATCGGTCTAAAG

GGCGACTTCATACAGGCAAGGCGTCAGGAGATTGTGATAACAAATTATGA

GGTCAAACCCAACGTTGCGGATCATAAGCTTGATATGCTAAAAACTTCAG

GCCATTTGATTGAATAAACACATCGTTTTCTAAAAAAAAAAAAAAAAAAA

A

>TSAB.R2.esd 597 0 597 ESD GOOD: 97-597

GCACGAGGACTGTTAGATTTCTTCATTACGTATGACGATGATGTCGAAGA

TTCTAAGTTTTATAAAGGCAGTGCTTTTCAACGTCGGCTCACTGATCGTG

AGGTTGAAGAGACAGCAGATGAAAGGGATAGGCAAAAGGAGTTGGAGGTT

TTCGAATCATCTAGTCGTAAGCTGATCGAGGAGGAAGATCCTAATGCAGA

GTCAATTATTTTGCAAATGGAACAAGCGATGCAGGAGCATTTACAGAAAC

GTCTCAACCTCGATGCTCACACCCCTATTGATCAAACGAGTTCTAGGTCT

GGTTCGAAGTCGAGAGATGTATCTCCTGCGCCACAATCTGTTTCGGGTTT

TGTTGAGGCACCCGTGATCTCTGCTGGCTTTAAACCAGTTTCTCCTGACG

ATGTTTCGAGAACTAGTGATAATGCAACGCCTAAAGAGGTTTCCGGTTCA

GAGCCATCTCGAAAGGATTCAAGCACCTTCCTGTTCTCCATGTCTTCNNN

N

>TSAL.R35.esd 819 0 819 ESD GOOD: 112-487

GCACGAGGACCAAGTCCAATACCGACACCCCGTCTACGTCTTTTACTCCT

CGTCGGTGTGCTTTTTTTGTAAAATACGACGCACTCACGAGTGAACTTCC

CCCTTCCTCCTTGCTGCACGCATCCAACCATTTCCTGATTCTGTCTCTCA

CGCTTCTACCAGGTCACCACGAAAACAAAGGAGAGACAAAGTCAAACAGG

TCCCTTAATCTTCCTTCCTCTCCTGTCCGGTCCTACCTACGCTCTCCCTA

ATCCGAATTCCCTTTTGTCCACCACCCCTTCCCCCTTCTCTGAACCCGCT

GTTTGCTCTTTTTGTGTAAAAAGGAAGAACCAGCAGAAAGATGATGATAT

ACTCTGTGAAAAAAAAAAAAAAAAAA

>TSAA.R63.esd 707 0 707 ESD GOOD: 101-358

GCACGAGGATTCAGCATTGCCCGCATGCCTTTTGGAACAATACCGGAGAT

TTTTCACATTGCCGAGTGGCGAGTACCTGGAAGCACACATTAAGCTCTAC

CAACTCTTGACTCCGTATTTGTCTGAAATTATGGCTCTTGATCCATATAA

CAAATATGTGTATGATTTATTGTGCTATGCATATCGTGCCGCGTATGCCA

GATATCTGTTTGATTAAAATTTTTATAATAAAACCTTTCAAAAAAAAAAA

AAAAAAAA

>TSBR.R88.esd 747 0 747 ESD GOOD: 96-503

GCACGAGGACCACTTCTCGCTCGACCACTGCCTCTTTGCGCTGAACTCGT

CTCTTCCTTTTCTGTACAGTTTCCCCTTGCTCCTCCAATCCGGCTACCTC

ACTCGACCGGATCTCATGCCCAAGTACTCTGGGAGTCTAAACCACCTTCT

TCCTTAACAGTCTTTCAAAAAGATAACTGATTTTAAGTCCCCTCGACGTC

GACACTCGAACTGTTTGCACAACTCTATGGGATACTTGACAACCTCTTTC

CTCTCTTCATCGTCCCCGAATGTGCTACTAATCCTAACAACTCTCACGTA

ACTTTCTATTTGTACGATCAATTGTTTCTGCCCTATGGCTGAAATTTTCT

GTTAATTTCACATAATTATTAGTAGTAAAAAAAAAAAAAAAAAAAAAAAA

AAAAAAAA

>TSCD.R57.esd 673 0 673 ESD GOOD: 87-604

GCACGAGGCCTCTTTTTCCGGCACATGCTGCACGGCGGTGATGGCATACG

TGCCGTCCAAATCTGTCTCCTTCCTCCCTACTCTTTTACCCCTTTTTTGT

ACAGAGCAGTGGAAGTGTCATTTCGACTATTCTATTAGCGCCTTCAAGAT

GTGCGTGCGTGAGCTAACGTGTGTTCCACTTTCTCCTCCAATTTGTCTTC

CACTCTTTTACGGCTTCCTACCCTGCCCCCCTCTCCTCCTCTGTGTGTAC

ATATTTACACAGCGCGTTTTTATCCATCATTCGACTATCAATTTGGTCTC

CTTATTCTTCCTTTCTTTTTTTTCGCAATGCCAAAATCTTCTCTAAAATA

CCATGTGAGAATAGCACTTCCCACCCTCTTTCTCCCCTCTCCCTCCCCCC

TCCTTGTGTAGAACAAAAATAATGCCCCGACTTTATACACATCTATCCAC

TTATATATATATATATACATATATTCTACATATACTAAAAAAAAAAAAAA

AAAACTCGAGGGGGGGCC

>TSBC.R17.esd 701 0 701 ESD GOOD: 102-430

GCACGAGGCCTTGTCATACCCGATGTGCGAGTCGTGCGTAGTCGATGTGT

CTGCATCCTATGATTGGCCCGGGATGTTATTACTGCTATTCCTTATGGTC

TCTTGTTAGTTTTCTTGGGAAATTCTAAGCCTTCAANCTACTTCGGTCGG

GGGCCTCGGTAATCTTNNTGAAAGGAACTGGTCTTTCTAAAACTTATCGG

TACGCCTACCCACCCCTTCCAGNAAGATTGGGAATTGGAATTAGTCGGAT

TCTACAAGAAATTTGGCTCCTAATACTTACGCCATTTATTTTCGGCTTGG

ACTTAGTGGAANATTTCGCACACCAATTT

>TSBD.R25.esd 517 0 517 ESD GOOD: 83-517

AAAAAAAAGCACGAGGCCTCGTGCCAAAAAAAAAAAAAAAAAAAAAAAAA

AAAAAAAAAAAACATTAGCGGCTATTTATCTGCACTCCGGTGCCCCCGTT

GCTATCGAAATGGATTCGAAGACCTTCTTCCCTGACTTCTCTAAAGTGGG

CACCCTGGTAGTATTTGTTGCCTTCATTTTGAGTTATATGGGCGTNAAGC

ATCCGCAACCACGTCAATGAAATGAGCAACCCAGGGCGCGACTATCCGTT

GGCTATGTTACTGCTGATGGTGGCGGCAATCTGCTTAAGCTCTGTTGGTG

GTTTGTCTATTGCGATGGTCATTCCGGGGTAATGAAATCAACCTCTCCGC

AGGGGTAATGCAAACCTTTACCGGTTCTGGATGTCCCATGGTGGGCACCA

GGAAATTGAGTGGACGGTTCGCGTGATCTCCGGNN

>TSAZ.R73.esd 671 0 671 ESD GOOD: 125-618

GCACGAGGATTTGACAGTATTTCTTTCCGTTCATTTGCCCAACTCCAGGG

TCAATTCGCGCGATTTTATGAGATGACCGGGGCATGCTATATTCGACGCC

ACACCACGAAGCTCCCTCATAATCATGTCGATTGTGGAACCTGTGTCTAC

CGACGTCTAACACTCGAATGCAAACACTCCGGCACCTTCAGAAGCCGTTC

TACACGCGGCGTATCCCGGAAATCAAAGATGATCAATTGCCCATCACGGA

TAAACTTTATCTACGAGAATGGCTTCCTAAAACTCCTTAACTATAACCTT

CGTCATAATCATCTTCTCTCCCCAGTTGCGATTCTCAGAGACGAGTCTCG

TAATACCGTTCCCAAACCACGTTTCCATTTGCGTCATCCAAGTAGTAAAC

TCGAGGACAAAGTTCCACCTGAACTGACTTCAAGCACCCTTTGGCACACA

GGACAGGCTTTGATCGCTGAGAGTCCAGGACAAATCGGTTCCTT

>TSBT.R24.esd 669 0 669 ESD GOOD: 97-543

GCACGAGGCTACTTTANCACTTTTGGGTGAAAGTCTGCTTCGATGATGCC

TAAATAAAGCTAGTGCGTGGTAACGCAAACACCACGTTGGCACCCACGCA

TGCACGCAGGTGGGAGAAGGGGGGCCGACCACCCACCCGGCGACTGAGCC

AACAAGAGGCATGGGTGGTGTTCAAGATGCCTCGCTTGCTCACCTCACGC

GTAGTGAGCTTACAGTGTTGAGCAACACCAATGCTTCAGCATCGCCTCCA

TCATTGACGGCGCTTCCACGCCAGACGGTCGGACACCATTTGTGTCCAGG

ATTAATTGAGGAATGCTTCCAACTTAACTTTCTGCTATAAGTTGGTAGGC

TTCAGTAAATACAAGGTTTTAAAACAATTAAAGTACCTCTCAGTATATTG

TGCAATGTAACGATTTATAGTGCTCGGCTGTTGTACGAATTACAATT

>TSCG.R69.esd 627 0 627 ESD GOOD: 94-468

GCACGAGGCTTCATTAAAGCGGACAATGGAGAGCTTTTGTTTGTCCACGT

TTTTGAGTGAGTTTTATAGTAAGTATTACTAGGAGCTTTTGTGTAGCGTT

GATGGTGAGGTAGTACCTCTGGCGGGTGATCGCGTTCTTTTTAAGAAAAT

CCTCATTCCTCCCAAAAATGAGAAATACCAGGCTGTTCATGTGAAAATAA

TCGACTTCGTTCCGGAACGTCACCAGCGTTGGCAAGAATTACCTTGTCAG

TAGACTCCTTATTGGACACCTCTTGTGTTTATTGTAGACTTCTGTTTGTG

CGGTGTACATATTACATAATCGTGAATTAAATACAATACCCTCCGGGCCT

TTCTGTTAAAAAAAAAAAAAAAAAA

>TSAO.R60.esd 681 0 681 ESD GOOD: 98-602

GCACGAGGGCCACACCTATGTCGAGTCTCGACCTTAATGAGCGTGTTTAC

TGTGCAGATGTAGTCTACCCTGCTGGTGTTGTTGGTTGCGCAGGCCGGCA

AATAATCGTCTATACTCTAGAGAAGGGACCTGAGGTTGCTGCTCAAATTG

AGTCACCGCTCTCTTACCAGAACAGATGCATTGCCATTTTTTTGGACAAG

CAAAAACAAACTCCCACGGGCTTTGCACTTGGTAGCACTGAGGGTCGTGT

GGCCATTCAGTACTTTCAACCTAATGCGCCGCGCGACAACTTCACTTTCA

AATGCCACCGATCCCCTACCACAGTTAATGGATACTACGAAATATTTCCC

GTGAACGATGTGGCTTTCCATCCGTTGTATGGAACGCTTGCAACTGTCGG

TTCGGACGGCCGATATGCATTTTGGGACAAGGATGCTAGGACTAAACTGC

GAGGCCTTGATAATGCAGAAATGCCCATAACCACTTGTACAATCGATCGA

AGTGG

>TSCC.R93.esd 647 0 647 ESD GOOD: 102-641

GCACGAGGCTCTTACATGCGGCGGAAAGTTTCTCAAATTCCTCGTGTTTT

TCTTCAACGCCATTGTCTTTATCGGTGGTGGTGTTGTAGGTGGCTACGGT

ATCTACCTCCTTGTTAAGGCCTCAAGCGCTGCCGGCACTGTTAATATAGT

CCTTCCCGCCTTTATCACTGCTCTTGGTCTGCTGCTCTTCCTCATAGGTT

TTCTGGGATGCTTCGGTGCCTGTTATAACAACGCCTGCATGCTGAAGACG

TTCGCAGCCATTGTAGCAGTTCTGCTTGTTGCTGAAATCATCTGTGGCAT

CGTCCTCCTTGTCTACCGCCATGACTTTGTCACACTTGTTGGCAATGAAA

TGCAAAAAGCGATCAATGAGCTTAAGAGCCAGGGACTAAACGAAAGCAAC

CCCACACTGAAGTCGATATACAAACTTCAGGAAGAGCTCAAGTGTTGTGG

AGGTACCGGCCCAAGTGATTGGGGCACTACTTACCCAAACTCATGCTGCG

AATCTGGGAATGCATGTACAAACCCATACAATCAGGGCTG

>TSBC.R71.esd 685 0 685 ESD GOOD: 104-653

GCACGAGGCGCCATATCTCTTGGTTGGTACGCAAATAGATCTTCGGGATG

ACACTACTACCTTGGAAAAGCTGACGCGTAATCGACAGAAACCGATAACC

TATGAACAAGGTGAAAAGTTAGCTAAGGAGATAAAGGCTTACAAATACGT

TGAGTGCTCTGCACTTACGCAGTGTGGCTTAAAAAATGTTTTCGATGAGG

CCATTTTGGCGACTCTCGAGCCGCCACAAACCAAGAAAGCCCGATGTCGA

ATTCTTTAAGCGAATGAGGAACACGAAATTCTCGCGCGACTGTTTCACTC

GCCAATTTCCATCGTTTTTTTATTTTGTGTATTCTGTAAAAGCACTAATT

CTGAACGAGTCAATTTGATTACTTAAGGCGGAATACGACAGGATGGCTAC

TATCCAATTGAAGAAGCCACTATCATCCAAAATGTCCATGCAGCCAAACG

TCTTTCCAATAAGTTTCTCCTGCACTGTCCCTCGAATCATGTTTGCACGC

GCATTACCGNCTTACGCTTTCTACTATTCCCTAACTTAATCCTCTGCTGA

>TSAY.R35.esd 738 0 738 ESD GOOD: 99-573

GCACGAGGAATTGGTAAGCGCTTTGCCGCCATCAACCTGGAGAATACCGA

GGAGAACCGTCGGGCTTACCGCGAGCTCCTCTTCACCACCGATCCTGAGT

TCGCCAAGCACATCTCTGGCGTCATCCTCTTTCACGAGACTGTCTATCAG

AAGACCAAGGACGGCAAACCATTCGTGGACATTCTACGCGAACGCGGTGT

CCTTCCTGGCATCAAGGTTGATCTTGGCGTTGTGCCTCTCGCCGGTACCG

CCGATGAGTGCACTACTCAGGGCCTAGACAATCTGGCTCAACGCTGCGCC

CAGTATTACAAGGATGGTTGCCGCTTTGCTAAGTGGCGCTGTGTCCTCAA

GATCTCCTCACATAACCCATCCTACCTCGCCATGCTTGAGAATGCCAACG

TCCTTGCTCGTTACGCTTCTATCTGCCAGCAAAACGGTCTTGTGCCAATT

GTGGAGCCCGAGGTTCTTCCTGACG

>TSBR.R39.esd 514 0 514 ESD GOOD: 108-432

GCACGAGGTATTGGCGTAAACCTCTATGCCTTGCATTTCAATCAGATGCA

TCCTCTGGAACTTTGGACTTGGTCGAAGACGGCGCGGACAGCTTGCCGAG

TTTGAGCCACCTGACGAGGACAGAGAAGTACCTTGTTGCATGTGTGCATG

CGATGCTGTGGTTCAATCGACTTGTGCGTCTCACTCCTCCCCAACGGCGT

CTCTTCTTTCCACCAAACATAGATGAACTACCCTCTCTCCTGACTTGGCG

ATCGCTTCCCTCCGTGTACAGTTCTTGTCATTCAGAAAAGTAAGGGATTG

TCGAAAGAAAAAAAAAAAAAAAAAA

>TSAB.R7.esd 675 0 675 ESD GOOD: 101-508

GCACGAGGACAAATAACTATATACAAAATACCAAAATGGTATTCTAACAC

CCCCCCAAAGAAATTGAGAAAAAATTGAAATAATAAAAGAATGGGAAAAA

AAAATTGATGAAAAAAATATGGGAAAAAATATATATACATTTACAAAATA

ACATGGGTAACTATTTACACACTTTCAATATTTGGAAGACCCTCAGTCCT

CTATCAAATCAATTTATATAGCCCCAGCCTAAAACCCCAAACAGCAAGCA

ATGCAGGTGTAGAAGCACGGTGGCTAGGAAAAACTCCCATGAAAGGCAAA

AACCTAGGAAGAAACCTAGAGAGGAACCAGGCTGTGAGGGGTGGCCAGTC

CTCTTCTGGCTGTGCAGGGTGGAGATCACAGTGCTTGTAGAGGGTGCAAC

AGGACAGC

>TSBT.R54.esd 785 0 785 ESD GOOD: 93-346

GCACGAGGATTAAGACCTTCGAATCCAGGGGTGAAGTCGATGGAAAGGTG

ACGTGGCAGGAGTTTTTGAGCTACTACAGCGGGGTAAGCTCATCCATTGA

CGACGACATGTACTTTGATCTGATGCTACGCAGCGCCTACAGACTATAGA

CTCTCACCCCATTCCCAACCAATCGCTTTTTTACCAGAATGCAATTAGGT

TGTCTTAAAAAAAAAAAAAAAAAAAAAAAAAAAAAAAAAAAAAAAAAAAA

AAAA

>TSAY.R77.esd 619 0 619 ESD GOOD: 96-201

CGTCTTTGTGATTTTTAAGTGTATCTAAATAGCAACGTTTGCAGCCCTTA

AGATGATGCCACTTCAGCGCCACATCAAAAATGTTGTCCATAACTACACA

GAAGCG

>TSCF.R66.esd 716 0 716 ESD GOOD: 104-465

GCACGAGGGGTACCTTGGCCCCGCTGTGTTGCTACACGCCTACCGTTGGA

TTGTTGATTCTCGCGATGACTACACCTTCAACCGCCTTGAGCAGATGCAG

AACAAGTGGTCTCTGTACGGTTGTCACACCATCATGAATTGTACCGAGAC

GTGCCCCAAGGGGCTTAATCCCGGTAAGGCTATCGCTGAGATCAAGAAGA

TGCTGCTCTACTACAACAGGTACAAAAACAAGAAGCCCATGAACGACCGT

TTGGAGAACACCTGCCCCACATGAACCGATTTGATTTGGTAATGCTGATA

TTTGCAAGCTTTGTGTTGCAGCATCAAACACTTCAAAGACTTTAAAAAAA

AAAAAAAAAAAA

>TSBY.R13.esd 730 0 730 ESD GOOD: 97-531

GCACGAGGGTTCAACGACTTTCGATCCGCAGTCTCTAGTGAGTACACGAT

GAGCGCGATCGACGAGCCATTTACTCTTTTCGCCTTACCCTCAACCTCTG

TATAGATGCCCGCACAAAAACCACACTCAAAATGCAAAACTCGTGTTCCA

TTCCCGCCCTCCACTTATCTACTCCCTTAATCTATCGTTCCTGTCGTCTG

TGCGTTTTTAAAACTTGTGATGACATTACTAGACGCGACTCTACTTTCGC

TCCATTCTTTCGAGACGTCTCATGATGATCTGACTTTATTTTGCTTTGTT

TCTCCATACTTTTCTTCTTTTCCCCCGTCCTCCCTACTTATAGTATTTTG

TTCCACCCCTAATTCCCGTGTGCTGGTGTTGTTTCTCCTACTTGCAATAT

TAAAAAGGATCAGACGAAAAAAAAAAAAAAAAAAA

>TSBK.R38.esd 497 0 497 ESD GOOD: 86-497

GCACGAGGGTGCCGTGGTCTCTGTGTGTCTGGTTAAACTTTTTGGTCAGT

TCATCAGTCTGCTGGATAATTCCGACACCAACGCCTCAGCCACAGAGAAA

AACAGGTTTTCGGCTGAGTGCATGCTCATAATTTCGAGCATGATCCACTT

GGCTCGCAGCGGTCTTGTAGCTCGTCACGTCAATTCCGACACTATTGACC

GCATGTGGATGTGCCTGGTTGCTCTCGCAGACGCCAGCACAAACGTGACG

GGCGCCTTTGGCAAAATTGGTCGCGATTTTCTGAAAGAATTGCTAGATGT

CCAGGATGCGGAAACGAAGAAACGCATTGCCAGCTGCGGCAACATGTCGT

CTACATGTACTGTCGAAAGCAATGTAGTTGACGCTCCAATCGATTTCGGG

TTGCTCGTGCNG

>TSBM.R20.esd 638 0 638 ESD GOOD: 96-569

GCACGAGGGATGATTCGGTTGTTAAATTCAAGCCTGATGTACTTGAATTT

ACATTCGATTCCATGAAGGCCTTTACTAACGATGCTATTGAGCGGAAAAT

CAAGCCTTACCTACAATCTGATGATATTCCCAATGACTGGGATTCTGAGC

CTGTTAAGGTACTGGTGGGCAAAAACTTTGACTCTGTTGCGCGCGACCCA

TCAAAAGCTGTCTTTGTCGAGTTCTATGCGCCATGGTGCGGCCATTGCAA

GCAGTTGAAGCCTCTGTGGGACCAACTTGGCGAGGCCTACAAGGATCACC

CTGAAGTCATCATCGCCAAGATGGATGCCACGACGAATGAGGTGGAAAAT

ATCAAGATTGGCAGCTTCCCTACAATTAAACTCTTCCCGAAAAATAGTGA

TGACGTTATCGACTACACTGGGGATCGCACAGTTGATGCTTTCAAGAAGT

TCATTCACAAGGAGGGTAAAATCA

>TSAR.R49.esd 667 0 667 ESD GOOD: 104-624

GCACGAGGCATCAATCTAACTCTCTCGGTGCAAACTCATCTCCACCTTAC

GGTTCCGCCTCTGGTGGGCGTTACCGAGATAGAGCAAAGGAGAGGCGGGA

AAAATTTGGTACCGTACCACCATCGAGATTCGATCAACAAGATGCTTCGA

TGACGGCCTCTTCCCCTCCTCCTGTGGATGCACCTCCTGCACTCGCCTCC

GCATCGCCCACAGCCAATAATGGTAATGTCGGTGGTAACGTAGGCAGCCG

TTTAATGGAGAAGATGGGCTGGCAAGCAGGACAGGGCCTTGGAAAGTCAA

ATCAGGGTCGAACGCAACTGGTTGAGCCCGAGTTCCGCGAAGTTGGCGTT

GGCCTGGGTGTCAAGGTCTCAAAGCGTCTCCCTCCTGCGGATAACTATAA

GGATAACGTGAAGCGTGCCATGTATGCTCGTTATCACGAACTAGGGTAAA

GTTCACCATGCTTCACCCAAGTCGTCGGCCTTGACGTCCGTGTACAGTAT

AAATAAAACGTATGAATCGTC

>TSAT.R95.esd 522 0 522 ESD GOOD: 102-522

GCACGAGGTCGAGGTTAACATCCGCCATGTAATGCTCCTTGCCGATCTCA

TGGCCAATAGGGGCGAAATCTACGGATATCAGCGTAGCGGCATGGCAAAA

GCGAAGAACAGTGTGCTGTGTCTTGCTTCATTCGAACGGACCGGCGACCA

CCTCTTTGAGGCTGCGTATCATGCCCAAGATGACGTATTAGCAGGCGTGA

CTGAGAGCATTATCGTTGGCAATCCTACGCGAATTGGAACGGGAACGTTT

GATCTTCTCGGGAAATGGTGGCAGCCGAAGGTTGATCAGGATACCAAGTC

AACACTGTTGGACGAAGTCTAAAATGATGTTGGAGAACTATCGAATGTAC

ATTATGCTGTAATTTATTAATATTTATCATGACAACCATCTTAGCAATCT

GCGACCTCTAGTATAAGAGTN

>TSBT.R79.esd 780 0 780 ESD GOOD: 97-489

GCACGAGGGCCCAGCTAACTCGAAACTAATTCATCCGTCATGGTTATCCG

TGAGGCGATGATAGTTTGGACAGTTCCAAACCGTTTATGAGAAAACTCGA

CAACCTTGTTGTGAATTGTTTAACACTGACCAAATCCTAATAGAAAATGC

TATCTTCCCACGTTGCTCCAAGCCATTTATCTTGCCGTTTGGTAGACGTT

CCTATGCCTTTGTGCAACCCCAATGCGAAAATGCAGCAAGTGATCATTCT

TAGCACAAAAATTGACAAATAACTCTCTTAGAGTACACCATTGCCTTTCC

AACGGGTCTGTATCACTAAAACGAAAAGCTATTTGGCTAACGTCCAAACT

TAATTAACGATGCCAGCCACTACCTGATCCAAGTGAATAACAA

>TSBZ.R77.esd 607 0 607 ESD GOOD: 97-537

GCACGAGGGTTCTTGGGCATTTTCACTTTCTTCCTTCTTCGTGTCAGCGC

CAAAGAGAATGTTCGCGTAACGTGTGGCTCTGTTGTAAAACTGGTAAATA

CCGATTATGATGTGAGGTTGCACTCACATGACGTCAGCTATGGATCTGGA

AGCAAACAGCAGTCTGTCACGGGCATAAAGGATATCACAGACGGTGGGAG

TTATTGGCAAATAAAAAACGAGGACAAGAACGATTACTCCTGCAGAGGTG

AGCCGGTGAAATGTGGACAGACAATTCGTCTCACTCATTCAGCATCTGGA

AAAAATCTGCACAGCCATCATTTCCAGGCTCCTCTCTCTCACAATTACGA

AGTTTCAGCTTTTGGCAATCATGGAGTTGGTGATGAGGGTAAGCTTAGCC

TAATTTTATGCACTTGTAACCCATTCCTNCCGTGTATTGTT

>TSAA.R85.esd 686 0 686 ESD GOOD: 129-594

GCACGAGGCTGCAACATGCCTTGCACCATCGCATTCAATGCAACGAAAGT

GGGCTTGAAGCAAGCGTCAACGCGTGCGAGTTCTGGTGACGTCGCGAGCT

CTCTGGTGTGCATTTTCGCTACCAATGAGGTGCTGCGCTTCCTGATGCAA

TTAAAATTCAGTGCGAAGCATAAAAGAACGCAAAACCTAAGCAGTTCTCA

TTCCACCCATTAGAGATGTTGGTAAGTTCATCAACAAGGAAGATCCACAA

CCTCATCCTTCTATTCGCTCTCACTTTTTTCGTCTTGACTGTTCAAGCGC

GTCCTCACCACGTGAGCAGAAGACCCTATCATGACGTATCAGCAGTTGAA

AAAAGGGCCAAAATTCCAGTCGAATTCAAAAGTGTATCCAGGAAATCTGT

GCTATGGTCAGGCGCGGCCAGTTATCCTGGCTACAGAGACAGCAGAAAGC

GGGGACATGTTAAGGG

>TSAM.R59.esd 706 0 706 ESD GOOD: 103-546

GCACGAGGGATAGTATCAGCACAACCGACTCAGAGATACGCCGTCTGCAG

GAACGCATTTCCATCTACGAGAGGACCTTCGGCCTGGAAATAAGGCGTAC

CAAAAACCACAATAATATTCAAGTTGTCATGCGTGGCTGCTGCGAGGACG

ATTACGATCTCCTGAGCTACGTGATTCTACGATTCAGTGCTGATTCGACT

CCGATGGAGCTAGTCAAATGCAACCCTCCAGTCGCCGGCGTACAAAAACT

TGTCGAACACTTTAATAATGTCGGTGACTTCCGCAGCTTTCTCCTCGTTT

TGAGGACCAGATTCATCAAGTACTTCCAACTACGAAAGAAGCGCAAGAAC

TGAAACCTCCCCGTGGATTTTACGATTTGCTAACACCTGTACATTGATCT

TATTTCATTACAAACAATACTTTCTCAAAAAAAAAAAAAAAAAA

>TSAF.R52.esd 654 0 654 ESD GOOD: 102-320

GCACGAGGCTTTGACCGACAATGGATTTACGTTGCCGGTCCAGTACTGGG

AACTGTGGTGGCGTGTGGCTTCTACGAGTTAGTGATTTGTCCCTTTGCCT

CACTCAAGCGAACCCGACACTACCTTTGCTCCGCAAAATTCAACCGCCAG

GAGACATATGCCCAGGAAGATGTGGGTGTCTAACGCGGATCCATCCCAAN

CCCCCTTCANCCTATTTGG

>TSAF.R5.esd 659 0 659 ESD GOOD: 99-505

GCACGAGGTTTTGGTTTTGCGTCCGCTTTGCGATGAGCAGAAATTTGTTT

GGCTTACCTGTGTGGTGCATGTGGTGAAAGAAGCACAGAGAGTACAGGGA

GCCCACACGCGCTATCGTCTGACTGAAGTGCCTTTAGGCAAGATAATTTA

CAGGCAGTGCATCGAAACTAGTTGTGACTAGTAGTATGTGGAGAACCATG

AAGTTTATAGGGGGAGTGAGTGAGGGGCACCACGATTCCAGTTGGTTGAC

AGAACCGTGCAGATTGTGCCGAATGTTACAATTCATCAGACGCCTGTCAA

ACCACGCACAAATGTGCCTCTGCAACAACAGAATTATCAAAATTTAATTA

CTCCATTTATGACTGGCTCCCATCTTCCCAATTTTCATTGGCACTTCATG

TCTTTTG

>TSCG.R24.esd 664 0 664 ESD GOOD: 106-629

GCACGAGGGAATACCGTCCCCATCAAGCTCGAGATCTCCTCCGTGAAATG

CTTACTTATCAGTCGATAAACCGTCCTCCATGATCGTTGCCCTTACTGCA

AGGGCTTCTCCTCATAAGGTTAGTAAGAGCAGTAACACATTCTTGTTCCC

TTTTTCAGGTGAAAATTGCCGGCGAAACGGTTCGCCTAGGTGAACAATAT

CTCGCCCGCGCCAATGAAACTCTCTCCAGTGCTGTACAAGCCATTTCCCC

TGACCTACCATCAGCCAGTAGTGCCCCTATATCTTTTGAAGACCTTGGCC

CCGAATTGGACGCTCTTCTGAGTGGAATTCAGGCTGATATGAGAAACAAC

CCACCGGCAGCAAATATTAAAATGTGTGAGGATAAGGTGGATCTAGCCAT

TGAATCTCCATCCCCCTCCTCCCTCTCTTCCGAAAATGCAGCGGACGGAC

AGATTGATGCTGCGCTTGATCCGTGTGTTTGGTCTTTCCTGCAGCAAAGT

TAGGAGTTTAAACTTGGGCTAATT

>TSBA.R52.esd 494 0 494 ESD GOOD: 96-336

GCACGAGGCAGTCATCCAATCCCAATCCCCCGGTCCCTTCTCATCCTGCA

AACGTGCCGTTGACGCCTTCGTTGAAGAATTGGTGCTCTGTGTTCTATTA

CGAGCTAAACAACCGTGTCGGTGACGTTTTTCATGCCTCAAAACCCAAAT

TTACCGTAGATGGATTTACGGCGCCGTCTTTGGGCACAGAAAGATTCAGT

CTAGGCGGACTCAGTCACGTGAATCGTCCACCTCAAGTTGA

>TSBB.R38.esd 407 0 407 ESD GOOD: 80-407

GCACGAGATAGGCAATATGGCCGTTGTTGCAGCAGCGTTACCGATTCATC

CTTGTGTTGGATGGAATGAAGGATATGGTAGTGACGAATTTTGGGGCTGC

GGGCTTCTCCGACTTTCGACTATCCAAAACACGTCTATTATTACTTCACG

GGACTTCCCACCCTCCAAGCTCTGGAACACCGAGCCAAGAGTGAGTACCA

ATCTAATGATCAATCTCCGTTTCTTGCTCCGTCCTCTGCTTCCCTTCTGT

ACACACACACATGCTTTGAGTATTAGCCCTCTTTAAATCGTGCTGATCCA

TCTTGCTGGAATATACAAGGAGCCTTNT

>TSAI.R42.esd 710 0 710 ESD GOOD: 102-355

GCACGAGGCGAACCCACCGAGTCCCTGAATTCACAGCTGTCTGCAATCCC

TTCTCAGTCTATCGATCCCCTTGAAGCCGCATCAGCTGCTGCCGCTGATA

TCAACGAAAAGATCCGTCGGGCTAGTGCCAGCGACGGTGTATCTGGTTAA

GCAATTGGGAATTGAAAATGGTAGCTTTGTACTGTAAATAGTCAAGTTCG

AACCTTATTGTTCACACTTTTAGCTCTTTAAAAAAAAAAAAAAAAAAAAA

AAAA

>TSAU.R49.esd 677 0 677 ESD GOOD: 98-639

GCACGAGGGCAAAGCCGACATTTGGAGGAAAAGTGCGCCTTCTTTGACAC

CGAGCAAAAGCGCTTTGAAGACCTCAAGATGGCGGAATTTGAGAAGGAGG

CGCGGAATCAGCGCAAGATGCTTGCCAAAGAGGTGAAGCAATATGAGGAG

GCGCTGCGACCACAGAAAAAAGGAGGGGGATGGCTTCTCAACCGCAAGAA

GGCCACTGCTGTATCGTCTTCCCAGCTGCAGGAGCCCTCCTCACCGGTGG

ACAAAAGCAAGGCGACAACGGCGTCTCCGAACAACTTCCCCCTAATTACA

GCCGGAAAGAGCAAATTGCCTTTAGAGGAGTTCCGCCGAAAGCGAACAGA

GGAACTGGAGGAGAGGCTCGCGCAGGATCGTGGTAAGCTGAATGAAAGGA

TGCGCCAACTGCGTGAACAGATGCACAGCTACCAGGACCGTCTGGCGCGT

GATTTTCGGGCCCAATGTGACCAAGAGAAAGCTGAGCTAAGCGGACGCAT

CAAAGTTCGTGCCGGAGAAACTCGAAGAGGCGATCGAGATGA

>TSAC.R70.esd 682 0 682 ESD GOOD: 99-560

GCACGAGGTGACTCTTCTTTGTCCTTCAAGCAGTCGATTGTAACCTTCTC

TTCGCCCTCACTTCAATACAGACTGATTGAAAGGTGGACTAACAGTCGAT

TAGGTGGTCTGTCGCTTCTTGACAATACTCATGTCGTTCTGGACACTTCT

GTATCTTCCATTCTAAAGGGCAATCCAACACAATCGCCAGAAAGTGGCGG

CAGGATTAGGTGTCTCCTCAAACAGCCTCGTGACTGTGAAGCAGCCTTTG

GCGTCGATAACTTACATCCCCTTGGTGGATCCATGAAGGCCATGGAAATT

GCTCAAGCTGCTACACAAGCTATGACCCCTTCAGTAGCTGTTAACTACAC

TGTTTGGAAACAGCTTTCAGAAACGATCAAGTAGTATTTCACTTTTCACC

TTGCATTGCTGGCAGATGCTTAATGTTGTATATTGACTAAGATTACGACA

TTTCCATTAAAA

>TSBA.R29.esd 545 0 545 ESD GOOD: 112-504

GCACGAGGAACGATCAGTAAATTGCATCGGCCCCTCAGCAAATCTGATCA

TCCACTTGCCGAGAATATGGATTAGGGCTGAGAATTAGTTACCGACATGT

AGTTCGTTTAAATCCCCATAAAACTTGGCCAATGCTTATAGGGTCGGTGG

TACTGTGTCACAGAAATATTTTCGATGAATTATTGTTAACAAGAGTGTGA

TGACGCGGTGTTAACTTCATGCATGTTTGTGTTTACAAAAGATAAGGAAA

TTCAAGTGCATTTTACTTAACTTGTGCATGATAAACTAGAAGCTGGCAAC

ATTTACTATCAAAATGCATCCCGTTTCTGCGATCTGCCAGTGGGACCAGC

TTCCACTACACCACTTGCCTTCACATGCATGCATACACGCTCA

>TSAD.R8.esd 707 0 707 ESD GOOD: 94-586

GCACGAGGGAAGTTTTTGGTGCCTCTGCACGTGTGCTTCAATTTGGCCTC

ATCACCGGCTCTGCGAACCCGTCGGCATGCCAACTTCCTCTTGGAAGGTC

TTGTTGAAGTTGAGAAAGAATGCAAAGATCTGGACATTGGATTTCACTTG

CTCCCTCTGCCCGCTACTAAATCCAACTCTGCCGGGAAGCGCAGTGTTGG

TGGTGATAGCGAGTGTCAAGGCTTTGATGCTGAGAAAATGGAGCCGATTG

TTGACTTACTGAAACGACTCGACGCTCGAGTGGTGGTAACTGATTTCTCG

CCCCTACGAGACGATCTGCAAGGAGTGCAATTTGTCTCAGGTCGCCTTCC

TCAGGACACACCACTGTATCAAGTGGACGCCCACAATGTGGTCCCAGTGT

GGGTTGCTTCGGAAAAACTAGAGTGCGGTGCGTGGACAATTCGTGAGAAG

CTAAACGATAAGGCGAAGGATCTCCTCACCGATTTTCCACCAA

>TSBK.R61.esd 323 0 323 ESD GOOD: 98-303

CACGAGGTCACTTCACGCCGGAGATCAAGCGTCGGATTGCCGAGCACTCG

GCGAAGAGAGCGCAGGCCGCAACTGGCTAGTGCCACTGGATGCCTGTGGT

CGAGCGCCGNCTTTCAGTTCATCATCTTGCACGATACTGTTGGCTCCACT

CAGCCCTGTAGATTGTCTCCTTTACTACTTTTTGTTTCCTTTGTGTAAAT

TAAGTA

>TSBW.R58.esd 624 0 624 ESD GOOD: 104-546

GCACGAGGGCGTGCTGGTATTCCTGCTGTTCTTTGTTGAGGCCGTTGATG

TCGTTACAACAGCAAGAGCAGGAGCGACCACCACTCCAACCAATCTACGT

CATTCCACTAGGTGGTGCTGCTGCTGCTGCTCCAATCGAACCAATCGTTG

TGGACCTATGCAAAACGCGACCACTTCCACAACCGCCAGTTCCCTCTATA

GCTCCGAAAATCGACGCTTCGATTACGTCCTCTCCGAAAAAAAGGCAACC

ACTTTTTCCGCTCGATGACCGGGAAATGAAGAGAAGGATTGTAAAACAGT

ATATGGAGCGCCGTCGGCGGGCCTGCATCTCCGATAAACTGTCTGCACTC

CACAATCTAGCTGTCTCTCTCATTGGGGAAAAGCCACAGCAGCAGTCACA

TCAGAGGTTAGAGATCACTGACATACTGAACCAGTGCGTGAGC

>TSAC.R71.esd 721 0 721 ESD GOOD: 98-416

GCACGAGGAAGAGAGCGAGAACGTGGCGCAACGGCGAAGGGAGGCGTCGG

AGATGCTTAGGGGACTTCAACGAGCCAATCAGATCATCAGTGAAGTCCGG

GAGGCCCCGATGTGGTAACTGTGGTTCCATTGGGGACGGCATTGTCTGTA

CCATATTCTCTTCCCCTCCTCTGATTCAAGTCATATTGTGGGTTTCGCCC

ACTCAGGAAGCATCCATTACGTCTAATATTCGCCAGAGGAACCTTCATTA

CCGTCTTATTGCTGTTCATTCTTAACAATATTAGCTTTTTCTAATCCCAT

AAAAAAAAAAAAAAAAAAA

>TSAT.R81.esd 681 0 681 ESD GOOD: 105-616

GCACGAGGATCCCTTTAAACGTTTAGGATGGCAGGCGCGACTGAGTTTAC

CAATCCTTTACGAAAATTTAAGCTTGTCTTCCTCGGCGAGCAGAGTGTTG

GCAAAACGTCACTGATTACACGGTTTATGTACGAAACATTCGATAGTGTT

TATCAAGCCACGATAGGAATAGATTTCCTATCAAAGACAATGTACCTTGA

GGATCGGACGATTCGGCTTCAACTGTGGGATACAGCTGGTCAGGAAAGAT

TTCGAAGTCTCATCCCCAGTTATATCCGCGATTCTTCAGTTGCTGTAGTT

GTGTACGATATTTGCATGCGAGAGTCCTTTTCACAGACTACAAAATGGAT

TGACGATGTTCGCAACGAGCGAGGAAACGACGTTATAATAATGCTGGTCG

GGAATAAAACTGACCTGGCAGATAAGAGGAAAGTAACTACCGAGGAAGGT

GAACGACTGGCCAAGGAACTCAATGTGATGTTTGTTGAGACCAGTGCTAA

AGCGGGTTACAA

>TSCG.R44.esd 761 0 761 ESD GOOD: 139-634

GCACGAGGATTTCATGAAGAGGAGGAAAATAGACGGATAGCCTATCACAG

CAGTCTGCCGTGGATCAAAATTGACAAGCTCTCACGTAAGCACGCGAGTT

TCATAATTCACCTGACCTCGGGCCGAAAGTTGACCTTCTTCTCAATCTCC

TCCCAAGAGGCACAGCATCTCTTCACGCTAAGCAGTCACCTGCACGAGCA

CCAGACTGTGGCTCACCTCGCCTCCCAACAGTCTGCTCGGCAATTGGAAC

AGCAAGATCGCTTGCAGTACAATGAAGCATACGTCTACAGTGCAGGCGAG

GTGGTGACGATGCGACGGGTTCACGCCGCACCAGACGGTCAGGCGAGGGA

GAGAATGCTGATGGAGACAGCGGAATCGTCTCTCTTCCCCCAAATCTCCA

GTTCTAACCCCAAAGATGATCGAGATGAGCAATCGCAATCAGTTTACATA

TAAATGAAGGCTGGAGATGGCAATCGTTTCCGCCCCCTATCTCCCT

>TSCA.R18.esd 583 0 583 ESD GOOD: 90-340

GCAACTCTTTTTGGAAGATGCTTGGAAGACCAAAGAGTTCAGGGTAGTGA

GGACTTGGTAAAAAAATTAGTGATGTTTAATGAATGCCCTTTTGGCGGCT

GTGAGTGGTTGGCAGTAGTGGCAAAGGGGAATGTGAGGCTATTGTGTTTA

ATGTAAGTGGGTATTAGTGGTAGTAATAATAGAAAAGATGTGGACTGGTC

CCTATAGCCTAGTCGGATAGAGCGCTTTACTTATANTGTGGAGGTCGAGT

G

>TSBI.R78.esd 786 0 786 ESD GOOD: 105-313

GCACGAGGGACTTGCAATCAGTAAATCAGGGCTCCCTTTTGTATATGGCG

CCGGAGGTAAAACAGCTCAATTGGAAGAAGCTGTTGTGGTACCTACCATA

CCAAAAATGAATTCTACACCTGCTACTCAAAGGCAGTGGATATTTACAGC

ATGACGATTTCACTTTGGGAAATGCTGACACGAAAGTTGGACCACGATGT

GAATCCCCA

>TSBW.R68.esd 331 0 331 ESD GOOD: 95-331

GCACGAGGCTCGTTGATTGCTCCCGTCCTCTGCGCGAGCTCTGTGTCTCT

TCATCACTGCGCTACTGCGGGCGCTCTATTCAGGAGCAATGTCGTCAATA

CACTGCGTCGGATGGCGTTGGAAGTGAGATTCGTCATGAGATGTGCAAAG

ATCGTCCCTTAAGCTTCTGTTCTGACCCAAGGATGGTAAAACTTTGTGGA

ATGGGTGAGTACTGCGAGGCAATGAGTATGACACCTC

>TSCB.R94.esd 563 0 563 ESD GOOD: 98-563

GCACGAGGAAGTGGCGACGCGCCTGCAGCTTCTATCTGCGTGGTCACTGT

AAAAAGGAGGACTGCGAGTTTGCCCACGACCTCACCAAGGTCACGTGTAA

GTTCTGGGAGGTGGGTGAGTGTTTCAAGGGTCTCACCTGCCCCTTCCTCC

ACGGCTATACACCTGAATTACTTCTGGAGGTTCGGGAACGGCAGCAGAAG

GAACAGCAACAGCTGCACCAGCAGGAACAGCAACAACTGCACCATCAGGA

AGCAGAACGTCTTGCAGACAAATCGTAGTGTGCTCTGCCCGCGCATTCTA

GTCGAAAATCATTCTCTGGTCGCGCATTTTCAGGATAATGTCAAGCTTTC

TCCTCCATTTATTCTTTTGCTATAATCTATACTAAGGTTTAATCAACAAT

CAAGTCTCCTTCCCTACTTCCTTTTCCCCTTTCTAACATAAAAACGGTAA

AGCTCTTTTTTATAAC

>TSBN.R21.esd 525 0 525 ESD GOOD: 88-525

CGGCACGAGGCTCATATCTTCACGGGCAAGCGGGAACACCTATGGCTCCC

CTCCCGCTAACACTGTCAGCCCTCTGAGTGTCTGTGTTCGCCGCTCGCAC

TCTATCGGGGCCGTGGCTGGTGCCACGGTGTCCCCTCCCAGTTCTTTGCT

AAAGAATGGTGGTTTCAAAAGCATCACTACTGCTGTCACCACCACCACTG

CTTCCAACACTTCATCCGGCTCGGCGCGTAACTCATTCTCTATCGGCCAG

GGTGGAGAGAGCTCCCTCCAGTCTTCGCCCACTGCTTCTACCGCCGCTCT

CTCTGACATCGATTTTGAGGCGACATCGGTGACGGGGACAGCAGCAGTGG

GTGGTCGATCCTCCACAGCTTCGTGTTTCAGCTCTCCCGCCTCCTCTCGT

CGAATTAATATATAGAAAAATAAATAATAAAAATANNG

>TSBW.R6.esd 674 0 674 ESD GOOD: 100-246

GCACGAGGGGAGATGGGTGGTGTCGTGGCTGGTCTGCTAGCCGTCCATCG

GCTGGTTGGTTGGTGTGCGGGCACCATCAGGCGCGGTGACGCAAACGAAC

TCGGAGACGTCGGCAGGAGTTCCCGGAAAGAGTTTTCTTTTCTTTTG

>TSAF.R44.esd 771 0 771 ESD GOOD: 144-649

GCACGAGGTTTGCAAAGTGCGTGTGCGCACCACGAAGACACCACAAGTTG

GTGACAAGTTTGCCTCGCGTCACGGTCAGAAAGGTACCTGCGGCATCCTT

TACAGACAGGAAGATATGCCCTTCACTTGTGAGGGGGTCCATCCCGACAT

TATTATCAATCCTCACGCCATTCCTAGTCGTATGACAATTGGCCACCTGA

TTGAATGTCTTCAAGGCAAAGTCAGCGCGAATAAGGGTGAGATTGGTGAT

GCTACTCCTTTCAACGATTCTGTGAACGTACGAAAGGTCTCCGTCGTCCT

GCAGGAGTACGGCTACCAGCACACGGGCAACGAGATCATGTACAATGGTT

TTACAGGTCGTAAGCTCAACTCCCAGATCTTCCTGGGTCCTACATACTAC

CAGCGTTTGAAGCATATGGTCGATGACAAGATCCACTCGCGCTCGCGTGG

ACCCGTTCAAATTCTCAACCGTCAGCCCATGGAGGGCCGCAGTCGCGATG

GTGGTT

>TSCH.R46.esd 603 0 603 ESD GOOD: 104-425

GCACGAGGCATTTGGTTGAATTTTCTTGGCTTTTTATGGCATAGGGCTTC

GTGAATTTAGCTGTTCACAGCAATTTTCTTCCCTCTACACTGAGCAGCCT

CCCTGCCTTTTTAAATTCCTCTCTCCTCTGCTTAGCTTCCGCACATTCTG

CAACGCCCCTGATACCGCACTCGCATTCCCCATATCCGATTGGTCAATGT

TCTCGGTACCAGAACGCATATTTGAATCATTGATAGTTGTAATGACCGCT

AGACGTTCTAAATCCTGGCAATGTGTTGCTGAAGAAAGGGTTCGGAACGT

CAATTATTTCCACATTTCTGTG

>TSBG.R12.esd 673 0 673 ESD GOOD: 100-517

GCACGCGGCTCAGATTCAATACGCCAGCAGTGGTGGAGGTTGTATGCCGT

TGCCCGACGAACCCTTTGCCAAGCAGAGTCCCTTCTAAACCGTCAAATGT

TTTTTTGACCATGACCGACAATTTGTGATAGGCTTTCTTCCTCTTCTCTC

CATATTTTTATCTTATTTCTTCCTACACCACTGTCTGCAGTCATTTTTTT

CCTCCCTTGCCTAAAAGAGAGAATACCCGCCTTCTTCCCTTTACCTCCTT

CCCACTTTTCAGGCCTCTGCTTATCGCATAAACTGGGCTGATTCCTTTTC

AGAGAGAATTCTATGTCTGTATATACACTTATACACACCATCAGAAGGCA

TTATGGTTAGCTGCTTATTTTACATTTTGTATAAACTCTTGTTTTGCTTA

AAAAAAAAAAAAAAAAAA

>TSBU.R46.esd 860 0 860 ESD GOOD: 98-648

GCACGAGGGTTGCTTAAGCGGCGCTACTGGGCTGTTGTCAATACGAATCA

TCTAGGGTCTTTATGGGGGAACAAGTCAATACATATCAGTAGCCTTTCCC

CCCATATATTTCGCATTGTCTGCAATACAGATTTTGGTATGGCCATGGAG

AACGCACCTATGAAAAATTGGGAACTCACTTTGTATGAAATGCAACGCAA

GCCGCAAGAACCGATCACTGATGGTACAAAAATAGCCGTTAGTCCTCGAA

GTCTTCAATCGGAGCTGATGTGCCCCATTTGCCTTGATATCCTCAAAGTT

ACCATGACTACCAAGGAATGCCTCCATCGATTTTGTTCTGATTGCATTGT

CACGGCTTTGAGAAACGGCAATAAAGAGTGTCCGACATGCCGAAAAAAGC

TGGTTTCGAAACGCTCCCTTCGACGAGACCCTAATTTTGACGCACTTATT

GCAAAAATCTACCCAGATCGAGAGGAGTACGAGGCGCATCAGAATAGAGT

TCTCGCCAAGTTGAATAAACAACAGTATAACGGCAGAGCATCTTTGCACG

A

>TSAV.R4.esd 653 0 653 ESD GOOD: 102-368

GCACGAGGGTCGGCTGTCACGGACCCTCTCATTCAGTTTACGAGAGCTTC

GATCCACTTCTTTCATCGATGCACCAAGCCAGATCGGAAGGAATTCAAGA

AGAATGCTATTGCCACAGCTGTTGGATTCCTGGCTATGGGTGTCCTCGGA

TTTATCATCAAGCTCGTGTTGGTGCCCTTCAAAATTATAATGGTCGGTGG

CTAGGTGTGTGATTTATTAACTTTAGTGTGCTCGTGNNNNNNNNNNNNNN

NNNNNNNNNNNNNNNNN

>TSAA.R46.esd 617 0 617 ESD GOOD: 98-363

GCACGAGGCTGGCATCTAAACGGGTGACGGTCATCACCGTTGTTGCTGAT

CCCTTCTCTGCATACGTATCAATCAACTATACGATGGGGCGTATGACAGC

ACCTCCCCCTTGCCTTGATGTGTACATATTTTAAGTGTGATCGACGCCTT

AAAACAGTTGCACTTCGCTTTATGTATAACACCTGCACCGCGCATATGGT

AATTGAAACAAGCGTCGTCGTTCCGTATTAAAAAAAAAAAAAAAAAAAAA

AAAAAAAAAAAAAAAA

>TSBJ.R42.esd 643 0 643 ESD GOOD: 91-538

GCACGAGGGCCTACGTGCCGAATTCGGCACGAGGTTCAAGTGCAGTTAGC

AGCTCTTGAAAGCACTGGACAGGCCAAATCGGAGGCTCAGAGCAAAGCGG

AGGCGATGCGAATCGCTAGTCAAGCCGAAGTTGAGAAGGCGCGTCTAGAG

GCGGAAGCAGACAGCATCAAAACGGATGCTGAAATAAAACGTTTGAGGTT

AGCTCGTGGAGTTGGGAGCTCGAAGTTTGTGGCAGAAAAAGAACGACCTT

GCACTGGAACGTAGGCGCCAGGAGCTTGAAATGGGAGACGGAGTTCTACC

TGAAAAAGGGTCCAGGCGATTGGTTCAGAGGAATCTGCGACACATTGCCT

GCGCAGGTGTGGAGCGTGAATGGCGAATGCTGAAAGCCTTAAATTTGAAG

AGCACTCTCATCACCGATGGAAAGGCGCCCATAAATCTACTCGATGCT

>TSAH.R38.esd 661 0 661 ESD GOOD: 127-561

GCACGAGGCATCTGCAAACACAGCGGCTTGGGGCCGTCCCGTGGTTGACG

AGAATAGCAACTGCGTCCAAGTATGCCTTCCAGCCCGCCCACTTCCCTCG

CACTCTTCAGCTTGTCATATCATCTCTCCATCCGCATCTGTCCCCTACCA

TGCTCACTGCGCTTTGCGTGTGTGTTTACCTCTCTGAGTATGCATCGTTT

GTCTGAAGTTCGTTGGGCTTGTGTTGCAGCCTGTCTATGTGGACACATTA

AAGGTTAAATTTTCATTTACATGCTCATTGTTTTTTTCTTTCGCATCGCT

CATCTATTCTTGGTGTGAGGCACACGATGCCTTCATCTGCGAATCCATGC

ACCATATCACATATTCACGTACGGTGACGCTGAAAAGTAAACAATGTGTT

GCCACGAAAAAAAAAAAAAAAAAAAAAAAAAAAAA

>TSAS.R70.esd 470 0 470 ESD GOOD: 113-444

GCACGAGGCGCTACTTTGCTGAAGACCGCACAAGGTACAGTATCGCTGAG

GTTGGGGCGGCTGAAGAACCAACCGATTCCGCTCAATTCTGTTGCCGCAG

CATTTAGCGCCGAACAATAAACAACCAGACGAAATCTACGGCAACGGCCA

AACGACTAGAAGATGCAGAATTAATTCTCTCCATCCAGAAGTTTTCCAAG

CCGCATCCCTCCCACACACGCAAACAAGTTTCCTACGGGCTTCCTTCACA

TGCCTACACACAAAAGCGTATCCCTTAGAGCTGAACATAAAATAAGACAT

TCCATAGTTTCCAAGACAGGAAACAAAAGATC

>TSAR.R54.esd 340 0 340 ESD GOOD: 100-340

GCACGAGGAAGCAGTGGCGCCGTCCACACAGACTCATATCACTGTGATTG

ATGGAGAACCAGAACACAAGAAGATGTGTCGTCGTGCTTTGGGCAAAGTT

TCTAGCACCGTTNAATTGGGATGCAGCTGCTGCGAAAGGCCATGACTAGT

GAATCATGCCATCCCCCAAAATCCTAACCGGATNATGCTCCTGGCACGGT

GATGATAGGTGTGGTCGGGTGTAGAATTCGTCTTTTCCGNT

>TSBY.R67.esd 688 0 688 ESD GOOD: 101-606

GCACGAGGGTTTGTCCGCCTCATGGGTCGAGGCGAGTGGTTTGGTGAGAA

GGCTCTCAAAAACGAGGATGTACGCACAGCCAACATAATTGCGGCCTACC

CAAACGGCGTGGACTGCTTAGTGCTGGATAGGGAATCCTACGAACTTTTG

ATCGGTGATCTGGGATTCTTGGAAAGACACTATTCGGACGAGAACTTACT

CGAGCATCAAGTGGAGAAGTCGGCGATTCTTGAATTCGGGAATAAGCGCC

TGCAGGACTTAAGCGTCGTAGCTACGTTGGGCGTGGGTGGATTTGGGCGG

GTTGAACTAGTCTACTTCGGGAAGGACAGCTCACGAACGTTTGCATTGAA

GAAGTTGAAGAAGCATAACGTGGTGTTGACGAAACAGGAGGAGCATGTGA

TGAACGAGCGTATCATTCTCATGTCCTGCAACTCGCAATTCATTGTTCGG

CTGTATAGGACATTCAAGGACCGAAAATATCTCTAACTCCTGATGGAAGC

ATGTCT

>TSBO.R5.esd 733 0 733 ESD GOOD: 101-531

GCACGAGGGTTGGGATGGGTGTTTGCGGTTACAAGATTGATCCACGCGCT

CCCTTAACTTCTCCAAATGTTATACTTCGCGGAGTTTCTGTCGTATTTTG

CATAATCATTTTAGCCTGTATTGACCGAGACGGTTATTTTGCTGGACGCT

GCATGTTTTACAGCGGCGGTTCAGCTTGTAGTTTCGCTTTAGCAGTCACA

AGCCTTGGATTGATTTTCTGTCTTATCTACATGACAATTGATGTTGGCTT

TCTTTCCTTANCACAAAAATATCGCAGACACATCACCATGTTTGAATTAG

GGTTTAGCGGCTTTTGGACATTTGTATTCTTTGTATTATTCTGTTACATG

GCGGACCACTGGCGAATTTCGGACGCTGAGAAGGAAGACTTAACTTTGAT

AAACCTGAGTAATATTCGTGCGTCTATCGCT

>TSBO.R19.esd 792 0 792 ESD GOOD: 99-685

AAAAAAAAAAAAAAAAAAAAAAAAAAAAAGCACGAGCAAGTGAAGTTTTG

AATTTTGGCCTTTGCTGCCTTTCAGAAGAATGTCTTTAAGGTTCAAAAGG

TACGAGTAGATCCAGCAAAATTTACTTTGAATTATCGAAAGCCCAAACTT

ATTTGTAAAGCACCTTAAAAACATTTCAATCGGCTCTGAACGGTTATAGC

TTCATTTGATTGACTTATGCATCATCATAAAATGATAGTTACCACAGTCT

CAAATCTGATTCCGATTCACTTTAATGGTCTTTACTTTCATCTTTTTTGA

GTCCAATGAAAAGAAAAAGGCAAGCCTCAACCCTGATGTTAACGCCACTG

GCACGGCAACACTAAACAAGGAAGGATCTGGCGCTGAGTCCCGAAACAGT

GAAGGTGGTCTCCCCAGTCCTGCCGCGCTTCCACGAGGCTCGTGCCACAC

GAGCGTTCCAGGTCGATCAAGGCAATCCGAGCCTATCTTTCCGAACACAG

AGGTATTTCCTGCCTTGGTGTGAAGGAATGAATCGGATGTGGAGAGTGAA

TCATACGGTAACACTATATCTCTTGGTTACTTATATC

>TSAW.R3.esd 724 0 724 ESD GOOD: 101-350

TGCAGGAATTCGGCACGAGGTTCAGTGGTTCATACTACTGTTACTTGATC

TGATCGCAGTAATTCCATTTGTAGTCGTTTCAGTTGTTTTGTACTATTTG

GAGCCATTCAAAAGAGGCTATTTTCCTAACGATGATTCACTAAAATATCC

TTACCATAGCTCAACAGTCAGGAGTATCTATTTATTTACAGTGGCGCCTC

TAACTACCATACTAATCATTATTGCGACGGAGCTTATACGAGCGCGTAGT

>TSAD.R61.esd 660 0 660 ESD GOOD: 97-569

GCACGAGGAATGCTTTCGGTCGACATTGCAAAGTTAATGCAAATGATTCC

TCAAATATTCCGTTCCCTGGACACCGCGGATGGCAAGATAAGTGGGGAGA

CGGCTCGTGTGGAGATGCTCAAGTCACAGTTGCCCGCTGCCACGTTGCGT

CGCATTTGGACCCTCTCCGACATTGATAAAGATGGGTACCTCACAGCGGA

TGAGTTTGCATTGGCGAAGTACTTGATCAAGCTTAAGTTGAATGGGCACG

AGCTGCCCTCCACTCTCCCTGATCACCTCTATCCACCATCAATGCAGTCC

TACCGGCCATCGCATGACGGGGTCAGTGGCGCAAACGCGGAGTCTTCCTC

GATCCACAGCATTCCACTCTCCTCTAAAAACGGCGACATTTGATCCTTTC

TCATGCTCCTTGCTCCAGCCAGGATATCTGAAACCTCCACTTCAACTTCA

TTTCTTGCTTTGAACTCATTTCT

>TSAW.R85.esd 549 0 549 ESD GOOD: 98-544

GCACGAGGGGATGGCAGAATACGAAGACTGGCGGTGGTGGCGAAGACGAC

CACATTCCGGACCTGCCACCACCGCTGCCTTCGCCACCATTACCATCAGC

CAACGGTGGCGACGAGATGAGTGGTGGTGGCATTGGAACGGAAGATGTGG

AAGGGACGGATGGAAAGGGAACGGGGTTAGCAACGGAAGATGAGGAAGAG

GAGGAGCCGCTCAACTCGGGTGACGATGTGACCGACGAGGAATTAGAGTC

CCTCTTTGAGTCGGATAACCTCGTCGTGTGTCAGTACGAGAAGGTGAGCC

ATTCGCGAACCAAGTGGCGTTTCTGGATGCGTGATGGAATAATGAAGATT

CGGGGGCGGGAACACCTCTTTCAGAAGCTGTTTGCGGAGGTCGACTGGTT

AATCGTTCCCCCCTAGCAGCGTGCTTGCTCTATGAGGTGTGCACAAG

>TSBB.R52.esd 711 0 711 ESD GOOD: 97-246

GCACGAGGGACCAATTAAGCCTTGACAGCCCCGACTCCTCTCAGGCAGTT

GCAAAGTCCCCGCACCCTGAACAAATGACCAGCAAAGCAAGATGGCTTCG

GGTTGTTGAGGCTGTGAAACAACCACCAAAACGTCGAAAACGTCGGTACC

>TSAG.R62.esd 656 0 656 ESD GOOD: 91-541

GCACGAGGCGGATTTGTCCTTTTTTATCAAGGCTTTATTTATTTTCTAAG

TAGGATAAAATGCAAGCGATTAAATGCGTTGTGGTTGGAGATGGAGCGGT

TGGGAAAACCTGCCTACTTATTAGTTACACTTCCAACGCTTTCCCCGGCG

GAATATATTCCCACAGTATTTGACAACTATTCAGCCAATGTTATGGTGGA

CAAGAAGCCTATTAATTTGGCGCTCTGGGATACTGCGGGTCAGGAAGATT

ATGACAGACTGCGACCTTTGTCGTATCCTCAGACCGACGTTTTCTTGATA

TGCTTTTCCGTGGTCAGTCCAGCGTCGTTTGAGAACGTACGGGCCAAGTG

GGTGCCTGAAGTGAGACATCATTGCCCCAAAACTCCGGTCATTCTTGTTG

GAACTAAATTGGATCTCCGTGACGATAGGGAGACGTTGGAAAAGCTAAAA

G

>TSAQ.R64.esd 694 0 694 ESD GOOD: 92-403

GCACGAGGGCGAAGTCGAAGATTGCTGCTCTTGAAGAGGCTGGTGTTGTA

GTGTACAGATCTCCAGCCCCAAATGGGCAGTGCTCTTTTGCAGGCTATGG

GCAGGCGCTAGTGAATCGGTCAAACTTTGATGAAGTGTGCAAAAAGTCGC

CGTCAAGTGCAAGCGTTGCCTTTCTGCATCGACCAAGGTCTCCGTCCAAA

TAGTGGATTGCGAGAATACTGGTACACTAACTTCTTCTAGATATTCGCTT

TTAGTTGTAACCAAATTTGTTTTCCTTTTTAATTAAACAGAGCATACTGC

CACTTTTTTAAA

>TSAC.R91.esd 707 0 707 ESD GOOD: 100-422

GCACGAGGCCCCTGCCAACCTATGAGGACACACCAATCAGTGTCTCAATC

CTACCGATCGTACTTCGAGGGATGTGACTCCTAAGCGACTCCTAAGCCAA

TCACTGTGACAATAACTACTAGCCCTTCGCCCTTTTTGACAATCCCAGCC

TCTCAGGCTGTCAGTGTTTATGTGTAATTTGTGTTTGAGAGTTTTTCATG

CATTTTCGGCCCACCCTCCTCCAGCCCGCTCTTCTCCTGTAACGGCAATC

ATTATTTTTGATACTTTTATAGAGTAAAACATATCAACTTTTTTGTTCAC

GTCAAAAAAAAAAAAAAAAAAAA

>TSAP.R47.esd 704 0 704 ESD GOOD: 103-664

GCACGAGGCTGAGACGAAAAGGCTCCAGCGGGCAACGTTTGCATAATCTC

CTCTCCCACTAGTCCTCCAATCTTTCACAATTGCAAACACTTAACCATGA

GTGCCTTACATCCTCTAGGGTCATATATTGTGTCCGGCCTCACCCTTCCA

TTAACATGCACAGAATAGAGTTCCCTACAAACTATTTTCATCCCAAAGAG

CACACAGCCCCTCCCTCCCCACCCATATATATGTACGTATATACATATTA

CATATTGCTTCATGCACCCACCAATAGCTCTCCCATTTGAAATGAGCGTT

GTGTCTATTATCTCCTTGCGCATACATACATTCATATCGGTCCGATCTCC

ATCGACACTCCTTATTTTGATTGCCTCATTTCCCCTCTCTGTCCCTCCCT

CTCTCTTCTAACTACCACCATCAACTTACAGGCAAATAGACATACCAAAT

TCATTATTTTTGTTTTCGACAGACAATTTGCGCCACTGAGTGGGCCAATA

TGCTCAGGTCACACAGACTCTTTCAAGTCCCTCTCTTTCTCATCCCCCTT

CCCCATCGTATC

>TSBM.R39.esd 589 0 589 ESD GOOD: 97-213

GCACGAGGCTCATGTGATCTCTCAAGATTTCCCGTGCCCACGCGTGCACA

AAACTTCGTATACAATCAAGTACTACACATACATACCCTGTCGTTTTCTG

ACAATCAATCACATCAT

>TSBN.R3.esd 411 0 411 ESD GOOD: 97-411

GCACGAGGCTCGAGTCTAAGTTAAATGAGGAATTAAAATTTATGCAATCG

CAAAGTTGTAACGTTTTACCTTATTGACTATCCAAAGTGTGTTCCGATGC

ACAAGCGTCAAGTGCCTGCTTCTTGTCTGTGTATGCTATGTTAGTGCTTT

TGTGGCGTCTGAAATTGTCAAGATGTGATGTAAAACACAATCCGGAAATT

TGGCATCCATCAGCGAGCAACTACTACTCAATGACCGGGTGCCTGCTTGT

ACTTCAAGAACTACTGGAATAGTGTTGCTTCTGCTGCTACACCTGTATTT

TCGATTGCTCGGGNA

>TSAG.R41.esd 802 0 802 ESD GOOD: 83-331

GCACGAGCTACCGCGTTCACGAATCGCTTTCCACCCCTCGGGCCGCTTCT

TGGGCACGACGTGTTTCGACTCCTCTTGGCGTCTGTGGGACCTGGAGGTA

TGCGTCGAGATCCTCCACCAGGAAGGTCACTCGAAACCCGTCTATGACAT

TGCTTTTCACCCTGATGGCTCTCTAGCCCTCACTGCCGGTCTGGACAGCT

ATGGGCGGGTGTTGGGATTGCGCACCGCGGCGATGGCATTATGTTTCCT

>TSAD.R12.esd 691 0 691 ESD GOOD: 107-575

GCACGAGGCGGTGATGACGAACATGACTTGTCAGAGGTCGATTTTGAGTC

TTCGAAAGTGCGTTCTCTACGGACATTGCACTTTGGCCGCGACCGTCCCA

TACACAGAATTTACGAGGGCAAGGAGGCATACTTTCCCCCCAGCTACCTG

ACCTCATCTCTCGAAACCGGTGCCCCATCCTTGATGCTTACAGGGGCGCG

GGGAAAGTCTATTTTTCCTGACAATAGTGTCAGTACCTTTCTAGAACAAC

CTGAACACCTGCGCTCACGTAGTGGCTATCGGTCGCATTTTTCTGGAAGC

TCTCGGTTCTCCGATGAGGATGCTTCACTGGAACGTCATCGGCGTAAGAT

CGGACGCCTTTCGACACAGACACATGGCACGTTAGTTGAAGATTTCGATG

GAGAAGCTGAGGAGTACAATATTCCACACTATCGAGGCTCTGGTTTCGAT

CGAGAACACAACGTTTTAC

>TSAD.R48.esd 661 0 661 ESD GOOD: 94-524

GCACGAGGGACGAACCTCAATACCCCAGATTATATTGAACGAATCAAGAA

CAAGTTGTTTGAGAACCTCAGAATGCTTCCTCATGCCCCTAGTGTTCAGA

TGGTTGACGTACCGCCTGACACGATAGACATAGAAGAGCAGGAAAAAGAA

GCAATAGAGAATGAGGATCCGGACAGACGAATCTCTATCATGGCTTCGGA

CAAGGCTGTGCATCGGGACAACGAGTTTTACGATTCGGGTGAGGAGGACG

GTATTGGGATGCCAGTGGCTCCCAAGGGCCCAAAAACTGCCAAGGACGTG

CACTCCTTCCGTAATCAAGCGAAGCGCGCTCGAATTGAGGAGTCTGGCCA

AGGGGATGTTACTACGATGGATACCGCAGAGTCCGCTGAGCCCGACAAAG

TGCGCAGCAGTGGAGACCACGAGACTGTGAA

>TSBP.R44.esd 647 0 647 ESD GOOD: 94-565

GCACGAGGAACTTGTTTGGTGTTGTGTATATTGTGTCGGCAGAAGCGTCT

TTGATATGTCTTTTTTGGAGGTCGACTATACAGAAAGCAGTCTAAGTGTC

TGCGGACCCCAAGACATCATATCCAGGTTGAATCTTCCTGAACACATTTC

CGGTATTGGACTCTAAATGTCAGAACTACCAACAGAAGCCGAGTACGAAG

AGCGCTTCAAATCACGTTTCTCAATTGATGACGAAGAATTCACTGCATAT

GTAAATGAGCCTTCACCGTCCCCACCCATTTTCGAGAGGTGGATTTCTAG

AAGCCCTCGACCCCAGCCCTCAGCAAGTCGCCGAAATTTCCACAAGTCCC

GATATTCCTGTCGGAATGACGAACAACCGCACCGAACTGACTGACCTTTT

GTAACCTCAGTGGTTTATTATCATTTTTAACGAACGAATTTGTCGTCAAA

AAAAAAAAAAAAAAAAAAAAAA

>TSBQ.R88.esd 865 0 865 ESD GOOD: 91-465

GCACGAGGGTTGTTGCCCTCTCGTTTCTCGAAGGTGTGGGTCCGCGCAGT

GACAGCACGGTAGGTTGGAGGTATGGTCGATCTCTCTTCGCGTTCCCCAC

TCCCCTCTCCCATTTCAACTTGACCTTCTGGCCCTGATCCGCCTTTGTGG

AGAACACAAACCGTTTCTGTTAGCGCACGCACAAACCCAAATGACCTAGC

GAGTGTGTCAGTGTTGAGTAAGCCGGAGTGTTATGACAGCATATGTACTC

TCTCTGACGCGTTTATGTGCATGCGTGTGTGTGTATGTGACTGTATATTC

AATTTGCAGTTCTTTTTAATTGGGTTAATATTACTCGGTTCCATTCACGC

ACAAGCAAAAAAAAAAAAAAAAAAA

>TSBF.R70.esd 617 0 617 ESD GOOD: 93-580

GCACGAGGCTATCTCTCACAATTGCCACTGCATCTTGGATTGTCGTTGTC

TCTCCCATGTCACATGTTACCATTATTGGGGCTAGAGGTGAAATCTCACC

CAATCGCAACCTGTTGTTCCCCCTCACTGGACCACTAAAATTTGGTAACA

CATCGGGTTCTCGTTTATGGATTACGAGTAACTTCGAAATAACTAACCTT

ATGTCCTTCGGTCGGCCTATTTCAATTCTAACATCAGTTTTCCGAACGGC

GTGTTCCGAATCATTGAACATTTCTGCTGTGCACGTTCACTGGCCAGAAT

CTGCCTGGCAAAGCACCTTGACTGTGGGAATGTCTGTGTGCTTGCATTGC

TTCCGTGTTTTAGAGGTAACAGGGCAAACGTTATAACTAAAAGATCAAAC

GCGGGTTTCGAAACAGCAGCGTTTCCTCTCTTTCAGTATGTTGAAGCAAA

ATCAGTAGTATTTTGATGCAAATGGGAAGTTGTGTATC

>TSAZ.R70.esd 471 0 471 ESD GOOD: 108-345

GCACGAGGGATGTGTTGCATTTACCGTGTCTGCTGTCACCGTACTCTCAT

CTCATCTCGCGTCTGCCGCCATCAGGCTTCCGCATCTCTTGTTTGTGGTC

ATCTCCCTACCTTGTGTTGTCTTTTCCCCATTACCAACCGATGCCTACTG

AAATGATTTTGATATACTTTGTGCTTGTCTATGATTTACCTGCGATTTTT

AGGTACTAATATTAAAAAAAAAAAAAAAAAAAAAAAAA

>TSAJ.R5.esd 666 0 666 ESD GOOD: 109-590

GCACGAGGGTACATAGATTTGCATTTACCATCAACTTTCAGTGATGCTTG

TCTCGTGTGAATTTTCCTATTTTCGTTTTTCTGTTGCTCTCTAGCACTTT

AGGGGACATATCCATCCGATCATCTGTAAAAAGAACAAAAAAAACAATGC

TTGTAAACAGCTTTCACCTGAGAAGACGTTGTTCCACCCGTTTCCTCTAC

TGCTCATCACATACCTAGCAAGCTGTAGAGAATTGAATCAGCCTATTTCG

CTTCTGATCTATGTTCGGACCCTCCTATGTATTCACATTTCTACTGTTTG

TCTTTGCTTTTTCGTCTCTGGCCCACATTCCGCTTTCTTCTGTTTAAGTG

TACTTCCACCTGGATACTTCTTGTGCTTGTTTGTATGTCCTGTTGATTCT

GTAGACGCGCCATTTTCATTATTATCATAGGCGCCATAAAAAAAAAAAAA

AAAAACTCGAGCTTCCTGATGGTCAAGTTATC

>TSAR.R59.esd 606 0 606 ESD GOOD: 101-428

GCACGAGGACCCGCTCAGCGCCAGGCTACCGATGGGAAGCCTTCTCGCCC

CGATTACGGAGACTCCGTCTCTAGTCTTGGCGGTTTCGACGGTAAAGCGA

GCGACAAAAATATCAAAGAGGAGGCCATCGTGCCTCGGAGCGCCCCTGGA

CCCGTCCAGCAGTCGGTCACCATTAGCTGAGCCGAGGAACGCCTTTGTCC

AGCATCCATGTGGCTTGTACTATAGACACACCTACCCTCAATAGGTCCGC

TTAAAACGTTTTCATTTTTGAATACGTATAAAGTGCCTTTATAGAGACAA

AAAAAAAAAAAAAAAAAAAAAAAAAAAA

>TSAK.R53.esd 382 0 382 ESD GOOD: 92-382

GCACGAGGGTAGCGTTGGGCCTTTTGATCCCCTTCTTCATTTCCCATGGG

CTGCAATTCTGCGACTATGGCAAACTGGAAGTGCTACACTGCTGGCAGTG

CAGAATAAGGCTACCTGATGCGAATGGCTGAATCTGTAACAGGATGATAA

GAAAGACGTCTACATTCGAAACCTTCCTCAACCCCGTGTTCTGTAAGACT

GCTATTTACCACTGAGGCAACGTTCTTGGAAACCCCCTATGGTGCTGTCT

ACAACTCCTGTATGAAGATGCCGACCCCGTGAAATAAAATA

>TSBH.R16.esd 566 0 566 ESD GOOD: 100-268

GCACGAGGCTCCAAAAAAAGACGAAGATGTCACAAAAGGAGCTGAAAGAC

TGGTACAAAAGATTTTGCAAGCAATTTCCTGATGGACAAATGAGCCGGTC

AGAGTTCGCTCAAGTTTATTCCGATTTTTTTCCTGGAGGTCCATTCAGAC

GCCTTTGCAGACATTGTCT

>TSAL.R59.esd 684 0 684 ESD GOOD: 102-417

GCACGAGGGCGATGCACAAGGTACTCGCCANGTGTGGCGAAGCGTGTACC

AATGAGTTGGGCGATGCTCTTCTGACAGCCGATGCTAGCTGACTTGAAGA

TAGGCAATGAAAGCATCTCCCTGTTTATATGCAGCGCTATGTCTCCACGC

ATCTCCTCGGGAAAGGCCTGCATTATCTGTAAAATGCATCGCCATTTAAG

ACTGGGACATAAAACGCTCTTTCTCTCCCCCCTACACCGTTCTCCCTCCC

ATCGCTAGGCCGTAGGGACTGGAGAGGGTTATGCTGTTCAAGCGAAATGC

ACACATAGGGTACATC

>TSAQ.R57.esd 715 0 715 ESD GOOD: 83-558

GCACGAGGCTGTTGTGGTCCTGGGAATAACGGTGGTGATGGATTGGTGTG

CGCTCGCCATCTCAAACTCTTTGGTTACAAACCAACCGTCTACTATCCGC

GTTCACCGTCGAAACAGCTCTACAAGAATTTGGTCACACAATGCGAAAAA

ATGGGGATTGCCTTCCTCTCCTACATTCCATCTGATGTGAAAATTCTTGA

GTCGGCGTATGATCTTATTGTGGATGCGCTTTTTGGTTTCGGCTTTCGTC

CGCCACTGAAACCGGATTTTGCTGAGACTGTTCAACGAATTGCTAGTTTG

AATGTGCCGCTTGTCTCCATCGATGTGCCTTCAGGTTGGGAGGTGAGCGA

AAAGACGGAGACAATGGATCTTCTGCAGCCGGATTGCTTAATCTCACTCA

CGGCACCAAAACTCTGCGCTCACCGGTTCACCGGTCGCTTCCACTTTCTA

AGTGGCCGATTCGTTCCTCCACTTTT

>TSBP.R73.esd 838 0 838 ESD GOOD: 55-313

CGGCACGAGCATATCCTAGTCTGTGTTACTCACTTGCCTGATTGTTCGCT

TTCTTCAATTCACACAATGCGCCCTTGTCTTTCACTCTAGTGTAGAACCC

TGTAACGATGACCGACTGCCTCGCTACTGGTGTTACATAAGTAGGGAGCC

ATGCACAGATGTGGATGTTTAGGATTCATTTCCTTGTCCCCATCCTTTTT

CCTTCTCTTCTGAAGAAATAAAAACGTTCTCAACCTAAAAAAAAAAAAAA

AAAAAAAAA

>TSBR.R84.esd 696 0 696 ESD GOOD: 103-614

GCACGAGGGTTTGTTTTTACTGGTTCTCTGATGGCGGAGTTGTATTTCAA

TGACCGCCGCGTCGATGGCCGTAAAACAACGGAACTGCGCACCATTCATT

GCGAATTCCTACCTGGACTGGCGGATGGTTCCGTTCTTCTACAACAGGGC

AATACAAAAGTAACTGCATCAGTATTTGGTCCTCATCCTTGCAACGTCAA

AGCTGACGAAGTACCTGATGAAGTTTGTGTCACTTGTCAGTACAATCGTC

CACCTTTTGTCAACACTTCTGGCAGCCGACAGAAACACACCCATTCCGAT

AAAGTTGCAGCAGAATACGCAATGTCTATTGAGGAAGTCTTTTCAGCACT

AATTCGTGGATCAATTTACCCAACGGCCCAAATCGATATATTCATCGAAG

TTCTCCAATCGGATGGTTCGGAATTTAGCACTGCGATCAACGCCGCTAGT

CTAGCGCTGGTGGCCGCTGGAATCGAAATGATTGGCTTCGCCATTGCGTC

AACTGTGGGCTT

>TSAG.R44.esd 701 0 701 ESD GOOD: 99-580

GCACGAGGACGACTTTCACCTTTACCAGTGATCCTCGTGAGACATTCCGA

GCATTCTTTGGCACGGATGATCCCTTCAGTACCTTTATGAGTTTTGGCAG

TGGCAATAAAGGTCCAGAGTTCATGGATGTTGACAGTGATTTCCTTAACC

GAGGTGCATTCAGTAGTATATTTATGAACGCGGGAGGCGGTGGTGCATCA

CGCAGTCGACGAACCCAGGATCCACCCATTCATCACGACTTGAGTGTGTC

CCTAGGAAGACGTGCTTTATGGCACCACTAAGAAGATGCGCATTACACGA

CGACGCATGGACGGTGGCACGGAGGAGAAGGTACTTACCATCGACGTGCG

CAAGGGCTGGAAGGCGGGTACGCGCATCACCTTCCCCCGAGAGGGTGATG

AGCGTCCCAACACCATCCCCGCCGACATCATTTTCACCGTTAAGGATCGC

TCACACAAGTATTTCAAACGCGATGGCGCCGA

>TSAS.R79.esd 604 0 604 ESD GOOD: 88-599

GCACGAGGGCCAATGTTGTCTTGAACTGCGTGGCGCATGGGCTCACTGAT

ATGGCCATGCCCTTCCTCATCCAAACTATCGTGAACTCACCGCCAGATTG

ACCACCTCGAGAAGTCGGAGCGAACTCGCTCGGAGGAGGAAGAGAAAAGG

AGCAGGCGGTGAATCCTCTTGTCCTTCACACGGATACGCCATTAATGCTG

ACGGGTGGGACGTTGCCTCAACTCGGTGCCTACGGAGCGGGAATGATGCC

ACCGCCCGCTGCTGGATCCGGTGGTTTCTATTCCACCAATGGGCCCTCCT

ACTAATACCCGCCTTTCACTGCCTTTCCCCTTCTCCTTCTGCCCTCCCAG

GTGTCTGCACATACATGGAAACGCATAATTTCCTCCCAACTCCTCCATTC

TCTGATCCTCCAAGCACGTGTGTGTTTAGCGCTAGCTGCTGCACTGTTGT

TCAAACCGGCCGCATTCACCGCTCTCACCTCTACCAAGTTGAATCACATT

GCGTCATTCCAT

>TSAY.R81.esd 702 0 702 ESD GOOD: 98-428

GCACGAGGATTTAAATAAAATTCGGCAAATTCCATGACTACAATGAGTGC

CAGGCTTAGGATGCTGTTGGTGATGAAAGTGTGCACTTCATCGGAGAGAA

AGTTAAATTATGTAAGCACCTATTAACTTACTGGTGAGCAATTCGTTGTG

TTTAAGAAGTCGGACATGACAGCCTATAGTTACATGTCCAATTGGTGGTG

TTCTTAACATTCAAAATCTTGCAAATGCATCCACATGGCTGCAGTGAAAA

CTCTCTCATGTCCAATGCCTATATTTAAAGAAGCGTTGAGTTTTACCAGC

CACCTCAGCGGTCACTGCTAAAGAATGACTA

>TSBQ.R28.esd 753 0 753 ESD GOOD: 91-293

GGCACGAGGCCTCGTGCCAATTCGGCACGAGGATCACGTCCAACAAGATA

TGGATAACACGCCAGAAGCTATAGCTAAGGTGGATGATATACTTGATAAG

GCATTTCATCAACTATTTACGAGGAAGCGACAAACTGAGGTTGCTATCCA

TTCCATACTGTGTGCCGAAAAATGGCAATTAATGGACTGTCCCCAAGCCG

ACC

>TSAP.R6.esd 620 0 620 ESD GOOD: 105-287

GCACGAGGGGACAAGGAGTGGCAACACCTGGCGGCGCGCCAACCGATGCA

GGCTCTGGTGGGGCTTCTCCGACCTCGGGGAGCTTGAAGAAAACTCCACC

TCTGGAGGGGCCTCAGCCTTCCGGGTCTACTGAGGGAACCTATATGTCTC

CTGTGAAGCGCATTAAGAAGCAAGATGACCAAC

>TSAC.R9.esd 652 0 652 ESD GOOD: 98-618

GCACGAGGCGACTGGAGATCGTGGACTGACGGCGACAAAAGAGAGCGAGA

TTGGAGGGACTCCGATATCGAAGTACTTTCATTGGGAACTCGTGAACGCC

CAGACTTATCGTTTAACTTGGGAGGTTCATAGCCTAACCAAGCTCGGTGC

TAACCTAATCGTCTTAAATGCACTAGCAGCTGATCTCTCTGGCGATCCGT

ATATGATAACCACCAAGTTCTTTCAAGGAGAAGCCACTCTCAGAGGGTTG

CAGCCCAATAAACCGTACAGTGTTCGTCTCGAAGCATTCAAAGATGAGGA

CAGCGTTTGGCTTTACAGTGGTACGATACACAGAGTACTCAGCGAGGTGG

TGATGACAAAAGCTAACAAAACTGAAGAGCTCATGCTTTCACGCCATTTC

GATTGGAGCCACGTGGGTCCTCAGACCTACCAGTTAAGTTGGGACACCAA

AGGCCTGGCTGAGCTTCATGCAGACAGAATCAGTCTGCATGCAATGCCAG

TTGATCCATCCCGCCCCTCTA

>TSBW.R84.esd 593 0 593 ESD GOOD: 85-548

GCACGAGGCACAACAACAACAGCAGAGGTGGGCCGCTTCTTCCCCAACTG

GCGTCTACCAGCAGCAGCAACAGCCCAACAATCCTTCGGATGCACAACAA

CAACAGCAGCAGCAACAGCAATTCCTTCATGCACAGCAGGTTCAGGCGCA

GGCACAGCATCAATATCTCCAAGCCCAGGTGCAGCAGCAGTTGCAATCCC

AACAGCAGCAAAATGTCTCCAATCACCACTCCTTTGGATGACTTATCGAG

TGCGCCGTTCACTTTTACAGTACCTGGACCGACTCAGCTCCAAGACAATC

TGAGCGTTTTACCTGCTTTTATAATGCACGCTCCTTTGCATTTTAAAATC

CACTATTATCCCATCCTTTACGTACATGAACCCGCCCCACTCAGAGATAC

GCCTTGGGGATCGCGATTTTTTTACTGCTCGTTGTCTTGTGTATGCGTGC

AACACCCTTCAACA

>TSAP.R61.esd 654 0 654 ESD GOOD: 95-563

GCACGAGGCGATAACATGCTCAATGGCAATTCAGCCAGGGATCCACGAAT

TGAAAGGAGAATTGTGGATTTTGGTGCATACCTTTTCCGAACTTTTGAAC

TAACCCATCTTTTGCGTAACCGATACACTCTCCGGTGAAATTATGCATGA

TTTGATGGTTGTATAGGTACACTAAAGCAACATTTTTTGGAAGGCATCGA

AACTGGTCGTCCAATTGAATAAAAGTTTACAAATGGCTTTCTGATAAAGC

TTAAATGAATTAAGCTTTTTTGGCTTTAGATTTATATCCTAAGCTGTAGA

TACAAAAGAATAGATATTCCCTATTTTATCTGAATACGTCTAAATGTTAG

AGCCTGGGAGGGAACTCCAAACACATATGGCTCTGGAACGTTGTAATCCT

GTTTCGTTTATTGCCTACACCTTTAAATTAGCCATAAAAAAAAGAATTTT

AAAAGAACAAACAAAAAAA

>TSAP.R92.esd 626 0 626 ESD GOOD: 93-561

GCACGAGGCATGCGCGAACTAGTTCACATGCAGGCAGGGCAATGCGGCAA

CCAGATCGGTTCCAAGTTCTGGGAGACAATCTCACAGGAACACGGGATTG

ATAATATGGGTTCCTATCATGGTGACTCGGATCTCCAGTTGGAGCGCATT

AACGTGTATTATAACGAGGGCCAAGGTGGCAAATATGTACCACGGGCCCT

CCTGATCGATCTAGAGCCCGGAACTATGGATTCGGTTCGATCGGGTCCGT

TGGGCAAGCTCTTTCGACCGGATAACTTTATCTTTGGTCAGAGTGGTGCG

GGCAACAATTGGGCAAAGGGTCACTACACCGAGGGTGCAGAATTGATTGA

AGAGGTGCTGGATGTCGTTCGAAAGGAGTGTGAGGCTTGCGACTGTTTGC

AGGGCTTCCAGCTATGCCACAGTCTCGGTGGTGGCCCAGGATCTGGCATG

GGCACTCTTCTTATCGCGA

>TSBO.R22.esd 680 0 680 ESD GOOD: 116-410

GCACGAGGCCGCCGTCACCGGCCTCGGGCTCATCATCTTCCTCGGCCTGC

GCGGCTACGATCGCGCCATCATGCTGGTGCCGAGCTGGGTGATGGTGTTG

TTATGGGCGGCGGGCTCGTGGATGGCGATTACCGGCCTGCTCGACAACGA

CATCGTGCAGCCTGCGCTGGGCGGCGGGCTGATCCTGGTGATCCTTTTGA

TCGGCTTCACCGTCATGCAGCACGCCTTCGCCGGCGGCGCCCTGCATCAG

GGCCTGTTCTCAAAAAAAAAAAAAAAAAAAAAAAAAAAAAAAAAA

>TSAN.R29.esd 649 0 649 ESD GOOD: 99-494

GCACGAGGGGTAAGTATTCGTGGGAACCCAAAACTGAGGATGAGTCCCGT

ACCTGCAAAGCTCGTGGTTCGTACTGGCGTGTGCACTTTAAGAACACCCA

TGAAGTTTGTCAAGCCATTAAGGGTATGACTCTATCACGGGCTCAGACTT

ACCTGAAGAATGTCATTGCCAAAAAGGAGATTGTGCCATTTCGTCGCTTC

AATGGCAGTGTTGGTCGACACGCTCAAGCAAAGGCCTGGGGCGTTGTTAC

AGGGTCGTTGGCCCANAAAGTCTGCTTGAAATGATTTTGCACCTCCTCCA

CAATGCCTTCAGCAATGGAGTGAACAAAGATATCAAGGGGCGGTGAGGCC

TATCGACTGTACATCAAGCACATCCAGGCTAACCCTGCTCCTGCCA

>TSAI.R20.esd 641 0 641 ESD GOOD: 94-601

CACGAGGGCGGTGGGTTGCTGTGTTGCTCTGTTGCCCTCTGAACACGTTG

GTTGTACTAGTTCCACAGGCTTTTGAGGGGGCTTTCTGCTAAATTTTAGA

AGCTTATGTGTTTCTATGCTTAATTCAATCTCGGATTCCCGAAATACTTA

ACTGGGGAACGACGCATTCTTACCAAATTATGCAAGAACCGTCTGTATCT

TCAACAACCTCTTCTCCTCACAAACTAACAACGACTGAAGCCGAAAAATC

TACCAAAGAAGCTGACCCAAAGACGAGTCCCAACTCCAAAGACACACAGG

AAAACACTGAAGAAATCTGCTCTAATCGAACTCCTCTCACCGAGTCTCGT

GTTAGTCGTCGCTCCAGACGTCGAACACACACATGTTCAAAAGCTGGGAG

GAGGCGATCCAAGTCATCAGACAGAAGGTCTTCTCGCAAATCTCATAACC

GTCGGAGACATCGTAGATCCCGTCGCACCCGTTCAAGATCGCGGCATTAC

TCTGGAAG

>TSBW.R83.esd 519 0 519 ESD GOOD: 102-519

GCACGAGGCTGACATTTCCAAAGGGAGTACCAGGCTTTCGCTCGTATAAT

GCGCTGGATTTATTGGCAGTGAGTGTTGATTCAGCGCCAGCGAAAGTCGT

TCAAGACAGTGTTCCAATTTAAGCCTTTGCCGTCGTCAAATCGCAATTGC

CTGAAGGCATTACTGAGCTTTGTCGACAGCCGCTCCCCTAACACCCACTC

ATTTATCCACGCACCCATTCAAGCAGCTATCGGTGATGAAACTTTGCATG

GTGCTGCCCTCATGGAAGCTGGAGAGAAGGTCTTGATTTGTTGGAAATCT

CCATCAAATCGCACAGTAATGCTACAGGGTGTGGGACATGGTTAAGGTGA

TTGGCTTCCAGCTGCGGAAACATCAACGATTTCTGTGAGTACCGATTCTG

CTGTCTCTCCTGAGTTNC

>TSBN.R17.esd 626 0 626 ESD GOOD: 102-377

GCACGAGGGCGCGCGCCAAACAGCGCGACATCGCTGCCGCTGACGCACAG

CAGGCCGCCGCGCATAAACGCTTCACCGACATCTCACACATGGCGCGTCG

TGAAATTGAAGACTTCAAGGTGCGAAGGATANAGTACTTCCAAGAAAATC

TCATAGCTCTCGCCGAACTGCAAATTAAGCACGCCAAGGGCTAGCATCGC

TTCTTCTTGCCAGAACGCTCTAGCTGACAATCAATCCCGATCAACTAACA

CAAACGCTGCTTGGCGGTGCAGTCAC

>TSAE.R43.esd 696 0 696 ESD GOOD: 101-625

GCACGAGGCTCTCATGCAAGCGAAAAGAAGTCGAAGAAGAAGCGTCGCTT

CAAGCTACCCTCACTTGCTCGTAAGACGAGAACCAAGGAGAAGAAGACGC

AGCAGGAGTCCCAGGCACAGAGCCCTAGTGCCGGCGCTGGCGAGGGCCCC

GCGGCGAATGAGATCGATGGTGAATCAAGCCAGAAGTAATTTCCAAGCCC

CGGAGCCACTGTATGTCCCTTACTGAGCATTCGCTTACCTCTAGGAATTA

TTAAAATACGCGCAAGCACCCCGCTATCAAGCCCTAACATGCTCTTGCAA

AGCTTTTATTGATTTTTCCATGGAGTGTCCTCTCTACATGGCCTCGACAA

TGTGAACGCTGGCTACCGCTCTATAGCATCCACAAACGCGAGCGTGGTTT

ACACTATTTCGCTTTTCGGCCGTCGCGGCCCATCTCTCCTAACATCCTCG

TATACACTAACGCCTCCAATGCCATGGATCCCAGTCCTTTTGGTTTGCTG

TCGACTCCTTTCCCCTTCCCATCAC

>TSAP.R96.esd 778 0 778 ESD GOOD: 64-609

CGGCACGAGGGTTTCCCACGGACATTACGACATTGACAATCGCCAAGAGT

TCATTGACCAAACGGTTGATTTCCTACTCAACACTTCAGTAAAGAAACAG

TTCACTGCCTTCCGGAGGGGCTTTCTGTCCGTTGTGGGAGATACGCCACT

ATTCCATCTATTTTCTCCCTTCGAGATCGAGCTCCTGTTGCTCGGAAGTC

AGCACTACGACTTTGGTGAGTTGGAACGCGTGACAGAGTATGAGGGCGAC

TACAGCGCGGAGACGATTGTGATCCGCCATTTCTGGTCGGTGGTGCACGC

CATGACGGAGGATGAGCAGCGCAAGCTCCTTCAGTTCACCACTGGTACCG

ACCGCATCCCCGTCGGCGGCATGTCGCGCATGAAATTTGTCATCGCCAAG

CAAGGTCCCGATTCTGACAGACTACCAACGGCTCACACCTGTTTCAACGT

CCTTTTGTTGCCGGAGTACTCGAGCAAGGAGAAGCTGGAGCGCTGTTTAC

GAGTGGCGATTACTATTCAAGGGCTTCGGCATGTTTGAGGGGCGAC

>TSBZ.R95.esd 714 0 714 ESD GOOD: 105-522

GCACGAGGCTACGACTACATGGGCTTCACGCAACAACTCTTTCAGAAGTA

GACTATTCCACTTCCTCCCCAATATTCCTCCTCCTTGCTTAGCGGCCAAG

TTAGTTTCCCTGTGTTTTCCTTTTGCCCAGCTTTCACTCTCTCCCTTCCC

AACTTTGTTCGAATTTTGTTGTTCTGATTCTTTTCTGGCTTCCTTCCCGG

CAACTTTTCGTCCTGTTTCTAATTGCTCTTGCGTTCCACCCGAATTCCGA

TGCTTCATGTGATGATGATATAATCGTTCTTGCTTTTAGGTTATGTTGTG

TGGTCGCCTTTTCTTCGTCGTCATCACTAGAGCAAGTATTGCCACCATGC

CTTTTAATTAATGAGTGAATAAATTAAAAAGAAAATATTCATCCCATAGA

AAAAAAAAAAAAAAAAAA

>TSBS.R35.esd 609 0 609 ESD GOOD: 99-545

GCACGAGGCGCAGTAATACCGAGGCCGAAATTGCTGCTAGTACGCAGAAG

ACCCTTGATATGCAGGCAGAGTCGGTACAGAAGAACAAGGACGTCGCTAT

CCAATCGCTGCTAGAACGAGTTCTTGCCGTAGATCCGCAAGTTCACATCA

ATTATCGCCCCCAACAGAAAGCTCAGTGAACAACTCTCACCGGTGCACCG

GAAAACAACGACAGAACCCCACCTCATCCCCAGTACCCTATGCTCCTCGT

TTTTCCAATGCGCATTGATCGAATGTATTTCTTCAACTGTCTTGAAGCCT

CAAAACCTGTGCACCAGGCACCGCGTACTGTGTTGTCATGCCACATTATG

TACCAACTGAGCCTTCTCAAGCGGTGAAACACTGACCACTATAAAATACA

AGTAGACTCTACCATCCCCACAAAAAAAAAAAAAAAAAAAAAAAAAA

>TSCE.R57.esd 685 0 685 ESD GOOD: 99-653

GCACGAGGGAGCGGTCCAGTCCTTGCAAAATGTGGACGTCTCCTACCGTT

CATTCCCACTCTTTCCCTCTTGGGCAAGATTCCGATTTCTTCTGCCTTCC

AAACCCAGCATCTTTGCCCGTTTTCCACTGGTAAATGCCTTGAATCTGGC

AAACTAATGACATCTCGTCTCACTGGGAAAAAGACTAAATTGTGGGAACT

CTACAATGAAGTCGTATATCCTCCGCGTGAAAATGCCACCATTACGACTG

ATGGCAGAGAACCCGTCGAACCTCGTCCTGCTGAAGTCACCTATACAAAG

GAGAACATCTTGTATAGTCAGAAAAAGCTCTGGTTGTTGGGCTTCATGAT

CCGCGGCTTGTCTGTGGACGAGGCTTTCCAGCAATTGGGTTTTCGACCAG

AAAAGGGAGCCCGAATCCTTGAAAAAGCTCTTGAAGCTGCCATTGAAAGG

GCAGTAACAGAACATGACGTCGAATTTTGCACAAATTTATGGGTTGAACA

AGCACTGGTATTGCGAGGCGACAGCGTTCCACGAATCCACAANGGTCTCC

GAACT

>TSAC.R69.esd 591 0 591 ESD GOOD: 113-588

GCACGAGGCCTCGTGCCGAATTCCCGAACTTCCGCAAAGGTACTGAAATC

GTGGCTAACGCTATCGCAGACAGCGAAGCGTTCTCCATCGCTCGGAATGC

CAAAGTGGAAGCAGTCGACGACTCAAACACAGTGCTCATGTCATGAGATT

CGCCAGCTCAGACCAAAAGCCTTACCACTGTCTGGACCAGCGGAAGGCGT

CTTCATGGTTGACGGCGAAACAGGAGACCCTCCATACCCAAGCCACTTCT

ACGCCTTCACTTTGCAGAATTCATAGTCGGCATTGCTGATGGAGGCACTG

AAGTGGGAGTTTGGAGGATTAGTGAACATACCTGTTGTACAGTGTGGTTG

TGGCAGCAGACGGGGTGCTTTGCTATAAGCTAGCTCGGTCTTCTCAGTTA

ATCAAAGGCTCTGCTATGACTTGACATTCAGCTGATAAATTAGAAGTGAG

AACTTTCGTTCATTCGTGAGCACCGN

>TSBG.R95.esd 532 0 532 ESD GOOD: 94-515

GCACGACGTGAAGGTATAGTACACATCAAAGTAATTTCTTTTTGATTGCA

GGATTCATTAGTGCCCTTCGTAAAGCCATGCAGTCACAGCAAGCAGCAAT

TTAGGATTAAGTAAATTAAAGAGTCATTCTTGGAAGAGATACAGGTGTAA

GAGTTGATCACTTAACTTGCTGTAGTGGTAAGTGAAATGCTATATATTGA

CGCTGCGGATTCTCACAGCATTGCACGACAAAAACGATGAAGTGTAGCAA

ATCCATTCATCCATTCTGCAAAATAAGTGAGCATAAAATCAAATAACAAC

GAGTGATCAGTGACGACACACAAATAGATTATGAAATCGAAAAGCATCGA

AGCTTTTTGGATTTACGATCATGGACACATTTGTATTTTACTGGAAAGCG

ACGGAACCCAGCTGGCATGAGG

>TSAR.R23.esd 598 0 598 ESD GOOD: 93-577

GCACGAGGGTCGCCGGCGGCAGTGTTCTGCGTGGTGTCCTTAAGGTCGGC

CAAGAAATCGAGATTCGACCTGGTCTAGTATCGCAGAAGGACGAGTCGGG

TCAAGTGAGCTGCCGTCCCATCCGTTCATGCATTCTCTCATTGCTGGCGG

AGCAGAACGATCTGGCCTACGCCGTGCCTGGCGGTCTCATTGGCGTAGGC

ACCAAAATTGATCCGCAGCTCTGCCGCGCCGATCGTTTAGTAGGTCATGT

CCTCGGTGCTGTTGGCACATTGCCCGAAATCTACGTAGAATTGGAGATCT

CCTTCTACCTCCTCCGGCGCCTTCTCGGTGTGCGAACCGAGGGCGATCGA

AAGGGTGCCAAGGTTCAAAAATTAGCGAAGCGAGAGATGCTCATGGTGAA

CATTGGCTCCCTCTCCAGTGGTGGTCACGTTGTTGCTGTAAAAGGAGACT

TGGCGAAGATCGCTCTAAACACTCCGGTTTGCACT

>TSBQ.R72.esd 848 0 848 ESD GOOD: 98-597

GCACGAGGGTAAGTGAATCTTCTCCACTTTCTTCCTATTGCAGTTTAAAG

TCTTGGTGCATTAGTTAATTTCAACTGATTTTGGTAGCAAATATATCCCA

CAGTCACAGTCGTGTTTATTCATCAGGAGCAAAACTGATACTACTTTGCA

AGTTGCTTTGCACACAATAGTCCCATTTTAAAGGCCAGAGAAAATGCACC

CCTACCGGATGCCTTCTGCGTCTCGTTAAGTAGAGCTGCATTGAAATATG

CCGATTCCGATGCTTGGAATCCCAAGCCACAGACAATGCCGAACGCGAAG

GGAATGCAGAAAGCCATGATGGTTGCCAAAAGAAGGCCGAGCACACCATA

GTTTGGCGGCAAGTTGATCGAGGTCCCCCATAGTGCCTGATCAAGCAACA

CCAGCACCACGCGTTCTTGTGAAAGGCATGCAAGTCAATTATTCTTGCAA

CCTGCCCCTTGATGGATGGTGAAAGCCACGCCTTACGAACAAGTAATCAA

>TSAI.R30.esd 668 0 668 ESD GOOD: 101-363

GCACGAGGGGCTACTCTTCTAAGATGGAGAGACTGATTAGGGGATGCTTT

TACGGAAGTGTTTTTGGCGATGTCTGTGGTGCACCGTTTGAGCTAAAACC

CGGGGTTTCTTTAAATCGTGTCCTTGACTACTTCGATGGATGGATTTCCA

AAGAGAAGAATGGAACTCTTCGTTACACAGATGATACGGAAATGGCGTTA

TGCATCTGCGATTCATTAAAACGGATGGACGGTTTCAGTGCAACTGATAA

TGGCTCGGACGTT

>TSAV.R54.esd 606 0 606 ESD GOOD: 107-474

GCACGAGGTGTGCGCCCTCCTTCACACCTTCGTACCGCAGCTCATTGCAC

CCAACTGGGACCGGGTCGTCAACGGCATGGATAAGCAGCGCCGCTTTGAG

GTGGCTTTCTCGGCAGCGGAAAGCCAAGGTATTCCAACGACACTTCGCCT

GAGTGATATGCTCACAAAGGACCGTCCTGATTGGAACCATGTAATGGCTT

ACATCGCCTCGATTTACAAGCACTTTGAGGCCATCCCTTAGTCCCCTCTG

CCTGCTCCCTTCCCATGTCCTAATTCGCGATTGTACATCCACGTTCGACC

TTCATCCCCCTTCATTTATATTACTTCTCAGTCCCAGTTGTACAAAACGC

AAGCACTGTTTTATCACA

>TSAQ.R66.esd 575 0 575 ESD GOOD: 91-546

GCACGAGGGGATGATATGCATAATTGGGTTGAAGCCATAAACTCTATTGC

GGCTTCGCTTCCAGCTCCTGTCACGGTAATGCCTCCCACAGAGGAAGAGG

AGGTAGAGGGTGGGTTGGTAGCCCCTGCCGGGCGCTCTGCAACTCTCCCC

TCTACAGCACTCCACCTCTCCGAACCCGGTACTAGTGATACGCAGGCTTC

TGGACCCTCGAAACCCAAGAAGAAGTTCCTCACACTCATGTGGAAAAAGT

AAACGATGAAGGAATGTCTCACTGCGAATGATGATTGCAATTCATCCGCT

TTACCGCCTAAAATCACTTTCGCGTCGTTTTACCCGTACACATCCACTTA

ACACTTGCTCTTTCTGAAGCAATACTAGCCCACTTTATTGTGCCTCTCTT

CATTCCCGCACACTTGAGCACTGATGAGCGTCGCTTGGTTTTCGTATCAC

CTACTC

>TSBF.R33.esd 548 0 548 ESD GOOD: 95-499

GCACGAGGGGTCTATCAGCCTGGGTTAGTAAGATCAATGCCGGACTGCCC

GCGAAAGGCGTGAAGGTGCTGGTTGGTCTGAAAGGGGACATGACGCGACA

GGTGCAGTCGGAGACTGCTCGCAGCTTCGCTACTCAAAACGGTTACATGT

ACTTCGAGGCCTCTGCCAAATCCGGACAAAAAGATGTCAACGAGGTCTTC

TGCGTGAGTGCTGCCAAGGCACTTGGACATACCGGTGGCACCTCCACCGG

GAAACCAAACCAAAGGGATCGTCCTGCTGCTGCTTTATGTAAAGCTAAAG

CAAACCTTAGACTTTTAATGTTCTGTTACGACTGGTATACCTCTTAAGCA

TCATTAAATAGTCTTTTGAGAAAAAAAAAAAAAAAAAAAAAAAAAAAAAA

AAAAA

>TSBN.R74.esd 550 0 550 ESD GOOD: 89-524

GCACGAGGTGCCTCGTGAGATGGAGCAGAAATGTCTGCCAGCGCAGCTTC

AAAGGCGCAGACTTCTTTAAATTGGTGTTTAGGCACCCCCAACTGAGCAT

CACTAAATGGAGGCTAAAATGCAAAAAATAATAAAACCAGGTCGGTAACC

AATCGTTGACAAACTCAAAAGAGGAGCTACGCAGGAACTTTTATCCTTCC

ATACAGGCAGAGGTGCTTTGTTGAACCGAGAGCACCACCACCACCACTTT

ATAACCGGTCACCACCACTGCGGTAATTGTGAGGCAGAAAACTAAATTCG

TATTATTTTTCAACAAAACGAAGCAACCTGTAAAACTTCGTAGCAGTAAT

CAAGACTGCAACCGAAGATCGAAGCCCTCCCAATAATACGCAAGCTTGGA

ATCAATTTTACTTAAGTAGTGGGAAACAAAATAGCA

>TSBQ.R66.esd 801 0 801 ESD GOOD: 94-446

GCACGAGGGTCAAAGCAGCGGAAAACTGCTGGTTTGATGCCTCTCGAAGA

GCTTATTCTCGAACTAAATGAAACAAAGAGTTCCTGCGAAACCAACATTT

GTCAAGATGATGTTGCAAGAGCCATAAAAAAAACTCCAATGCTTGGGCAA

TGGTTTTGAACTAATCAAAATTTCTGAATCGCGTCAACTGGTTCAGCTGG

TTCCAGGAGAACTGAGCATGGATCAGACTGAGGTTTTGAAATTGGCTGAA

TCTCACGCAGCTCATATTACTGTGAAGCAATGTGCGCAGAAGCTGGGGTG

GACGGATGAACGTGCACACGCGGCTTTGCAGTACCTCATGCGAGAGGGGA

TGG

>TSAH.R41.esd 450 0 450 ESD GOOD: 106-242

GCACGAGGTTGACAGATGCCTTCCATCCGATCAGGCAATTGATCTTATTA

CTGTTGCATTCCAACACCTCCAACGGGTAAATCGACCTCCAAAACGGCGA

GAAAGTTGGAATGACAACGCTTTTCAGCAGGAATACG

>TSAQ.R13.esd 813 0 813 ESD GOOD: 100-634

GCACGAGGCCTCCACGCCTCAAAGATGGGGCACATAACGATGCTCTCTAT

TATAGAGGGTGTTTCTCATCTATCAAATCCTATCTGGACAAATACGGGAA

ACCTATCGGCGTATCAGCATTCATAGCTTCTTTCGTTCTAGATAGATCCC

AGCTTTCGTTTGTTCCATCCTCCTCCTAATCAAGTTTAAAGAGACTACAA

AACAAAAGAAAAAAGAACGCATGCACACAACCTCATATTAATACAAGCCG

CCAATATGTACACAATAATACTTACAAAACTATGTGCGAAACTAACGGGT

CTTCTTGGAGGAACCAAAGCCACCGAATCCCATGGTAGCAGCCACACCTT

CGTCCAGAAGGTCAGGATCCTCGGCTTTTCGCTTCTTCTCTTTCCGTTTT

TCCCTTCTATAAGCCTTTATCTTTTCCTCTTCCTCTGCTATCTGCTTCAT

CCTCTCGTCAAGACTGTATTCCTGTGCCTTCTGCTCCTGAGCCTTCTTGT

GCATTGAAAAACGACCTTTCNACTCCTCTAAGGTG

>TSAA.R37.esd 615 0 615 ESD GOOD: 101-609

GCACGAGGGCTGGTCCAGCTACAACCAGGAATAACCGTGCAATAAGCCGC

AATTTGGCAGTTCGGCAAAGAATCATGGCAGTGAATCGACAGATCTTCCA

GCGTCAACGCCGAAGTCGCGGCGTTCCTGGGATAATGAATCGCCTGGGCG

TCCAATTTCAACGTCAAGTGCAAAGGCAGCAGGGACTGCGGAGATCAGAT

AATGTTCAACGTTTACCTCGTAACATCAACAGTAATAATTCATTTGGCAC

AAATTTTCAACGTCCCATTCGTCAGAATCCATCCCTCAATGCCGTTGTTA

GACAGCGTCGAGTAGCCCGAGTTGACAATCAGCGTTTCGGCATTCGTCGT

CGTGGCGGCGGACAAGTGATAAGAGCACAAACGAACATGAATGCGGTGTC

CTCGACGCGCTACCAGCATTACATTCAACAGGCTCGTGCCCTGATTCGTG

CCCAGGCAGAGCGTCTTGGAGGCACTGCTGGTGGGTTCAATAACTACAAT

CGCTACAGT

>TSAA.R58.esd 694 0 694 ESD GOOD: 96-556

GCACGAGGAAACGTTATGCTTCTAATCTAGGGATGGATTCGCGTCACACT

GATTCGTTAAGAAGACGAAAACATCGTTTGGCCTCTGCATTGGTGGCTGG

CGGCGAGGAGGAGTTTTGGGATTGCACGGTGTGTACTTACAAGAACTCTG

CCGAGGCTTTTCGTTGTGAAATGTGCCAGACACGCAAAGGCACTTCGACC

CGCAAACCAAAGCTGAATAGACAAATTGTGGAGGAGCAGAATCTCATTGC

TCGGGCCATTATTAAGGAACGCCAAGAAGAGAGTGGTGCCACAACTGGCG

GCACGGAATCCAGTGCGCCCCACCGTCGGAGGTATCATCGACATTTAAAC

AATAACGGTAGTGCATCGGCCTCTGTCTCAGCGCTAAAACATGTTGCATT

ACTACGCCCAGGATCTCTTCGTAATGTCGATCGATCATGTCCGCGACATT

TTGAGGTATTC

>TSAM.R64.esd 649 0 649 ESD GOOD: 106-297

CGAGGCGAAAGTGAATGCTTGAAAACTTGGGTTGATGATTACGCCTCATT

ACCAGAAGCAGAATCAGCATCACTACCACAATCATCACTCCTATCACAAC

CTGAATCACAACAACTAACATCAGAGGAGACGTTGGGTGAAATCGAAGTA

ATTCCACCAAGTTCTTGATCTCTCGCCTCTCCACCCCCTGTC

>TSCF.R46.esd 645 0 645 ESD GOOD: 99-630

GCACGAGGGTGAAGTGACAATTTTCTCTTGGTTTATTTGCCATACTCATA

ACCATAATCCTCCATATGCGTTAGCAAGCGTGTCGCCTCCGCCTATAGCG

GGGGTAGATCATTCTGTCATGGCCCCAGTTCTGTATAGAATTTAAATATT

GCGCGGACCGTAGTGTTGCTTGATGTCGCTGGGGGGTGTAATGGAATTCT

GTAGAACCTAATGCTTGCATACTATTACACTTAATCTCTGATTCATTGGC

GATGAAGGTATTGGGATGCATTTTTTGGTTCACTGCTATGAATTTCATCG

CTTAACTCTTTTCTCCCTCAGTTTATCGTTGTGATGCTGGGTAAAATAGC

AATGGGCAGAATGCCAAAGATAGAAGACGAAAGGGACTTCGACTCCGACA

GCTCCAGCAATGGCGTCTGCAAACCGGCTATAGCGAACGGTGTACAGAGA

AAGGAAAAGTTGCAATAGGCACAATGGTACCCCTCCTTCTTTGCCAGTCC

AACTCTCGAATACATCCAGACCGTCCGTGTGT

>TSBP.R80.esd 675 0 675 ESD GOOD: 84-552

GCACGAGGGTCGTCTCTAGATCTCCATTAGCGCTGTTTCAGTAGATTGAG

TTCAATTGACAGCCTCTTGATAGCAGAATGGTTTTCTTCATTGATTTCCG

TTTCATTCTACGCATTGCACGCAGGATAGAAAAAACAAGCGCTCACGGCG

ACTCCGTTTTTGCGATAATGATGAAACGCGAAATGAATGCGATGCCTTTT

CTGAAGTGGACACCGTTCCCTTTTACGAATCTTCCACTAATTATTTACGG

ATTCGCGACTCAACGTCGCCAACGGGTGGTTTTCGTGATGATCAAGTAAG

TAGTTCCCGTGTTCTTTTTTGTATCCTCATCTTGACTTCAAATGTGTTGG

AGCATCATTCAAGCCGTATTTCATTCGCATTAAATGGTTTAACTCAGCCT

ATTAGTGCTTCCGTGATATGGGTTAGTTTATTTATTCACGGGCTGCATGA

ATAACGGAATTAAACTTGA

>TSAW.R89.esd 576 0 576 ESD GOOD: 105-298

GTCTGCATGAACCCTACCCAACCCCGCGAGGGCAAAACTGTGCGTCCCAA

ATTTTCAGATGGGTTTGTCTTTACCTACATCTATCGCGATATTTTTTTCA

GTACTAAACCTATCTCGAAAACGTTTGGGAGTAACATTAAGCCATTTTTC

CTTTTTATTCAACGACTAATTGCTCATTTTCAGAAGCAATTTTT

>TSCC.R81.esd 691 0 691 ESD GOOD: 101-626

GCACGAGGGTTTTTTTTTTTTTTTTTTAATACTAACATACATACACTATA

GTATAAACAAGTAATCATATATAATCGACAAAATGTCAATACCATGTTAA

AACGGTACAACGATAAATTCCAAACTTATATCTACCTACTAATAAAATTG

TACTTAAACCTATAACCCCTAACAAAACATGACTAAAATGTAGTCCAACA

GTACAAAATCTACTAGCGTGAAACCTAGAATCAAATGCATTAACACCTAT

ATCTTCCATTTCAAAAATTTGCAACGCCACAAAACTCAAGCCTAAGACAA

TAGTCAAAAACAAAAAAAAATCACAACACTTTCAACCTAATAAATGATGA

AACGCAGTAACAGTTATACTAGATCCTAATAAAACAAAACACCCAACAAA

TGGTATTTCCAACGATCTAGACAACCTTTCATAAGATCAAGAATCAAAAA

ATAAACAACCCATTAAAAATCTACCAAAAATCATAACCTCACTAAAAACA

AACAGCCAAAAGGCAGACTCATAATG

>TSBT.R3.esd 292 0 292 ESD GOOD: 18-274

GGAACAAAGCTGGAGCTCCAGGGGCGGCGCTCTAACATGGATCCGGCTGC

AGAATTCGGCAGCGATTTCTTGGATCATTGAACTCATTGGAGTCTTGGCT

GGTACATTCTCACAGGAGCTTGATGCAACCTTACTAAACAGGATATTGAC

TTTCATCTGAAGTTTACAGCTAAATTCCCTAACTCGGTGGGCTTTGGTTG

GTACGTTCGGTAGACGTTCAGACGATCAAATGGTAGGTTTCTTACAATGG

TTCATCA

>TSBH.R52.esd 568 0 568 ESD GOOD: 89-329

GCACGAGGGTCGCATGTTCGCAGCGCTTTTGACGGGGCAAATAGTTCAAT

CGAATTTTGTTGAAGTATCTCCTGGAAGACTTCTTCTGGATTTACCGCCA

CTTGGCAAGAGCAACCACATAGTTGTAATTTTTGACTGGGCGGGTTCCGC

TCCCCGAAGGATATGGGGCTGGAGGTGAGTTAAGCCAAAATTATCCCTCA

TATTATTAGTCCACTTTGGTCTGTCGAAAATGGATTAGCAA

>TSBV.R74.esd 621 0 621 ESD GOOD: 113-557

GCACGAGGCTGGACCACTTCCCTCCATTCGCGGTTGAGCAATTTTCAAAC

TCCGTCCGGTGTTCCTACGTTAATGAAGAGAAAGATTTTAGCAAGTACAA

ACGTCCCTAGGAAGAAAGCGAAATTTGAAGTTTCGTGCATTCTGTACTAA

CAATTTAGAATTTCATGAAAACTGCATTTAAGTTTGCACGTCCGGAAGAT

ATTGACGCACTCTGGCAGCAAATAACGAGACGAGACAACGACGCTGGAAT

CGCCGTATCTAAGGCTTCAAGTACCGATGATACCAAANCGTCTAATGGAT

TTGAATAATGCGAAACAGAGTGAACGCCCTTCTGTAAAGAGTGAAGGTGG

CCTGAAAACTCAGCCGTCAGACATGGCTAACCAAAAGTCCGCCGATGATG

ATGCTTACGAAAAGCTTGATAAAATCTATAGGAGATTCGGAAAAA

>TSBW.R92.esd 637 0 637 ESD GOOD: 92-343

GCACGAGGGCACGTTGTGCGTATCTTATCTTCGTAATGGGTGATGAGGTA

GCCTATCGTGGAACACTTGACGGGCATAGTGATTGGGTTACGCAGATCGC

AACAAATGCTCAACATCCTGAACTTCTGCTCTCGTCATAAAAAAAAAAAA

AAAAAAAAAAAAAAAAAAAAAAAAAAAAAAAAAAAAAAAAAAAAAAAAAA

AAAAAAAAAAAAAAAAAAAAAAAAAAAAAAAAAAAAAAAAAAAAAAAAAA

AA

>TSAV.R78.esd 539 0 539 ESD GOOD: 99-471

GCACGAGGGATCTTTCTTGCATCTATGTGTAAAGATCGGGGCAATGGGTT

TCTCCAGGCTGACAATCTCGCTCGCGCTCTCTATGCCTACNAAGGTGGAA

TTCATTGTCTTGAAGGAAGCGTCAGTGTAAAAACAACAAAGCATGCGGCT

CAAAATCAAGCAAATTGAGGAGGCTCAACAACTCTACACTAGTCTTCTTA

CCAAATGCGGGCTCTTTGGTCTTCTTAAAAATCACCGTGCCACCAGAGCC

GGTCACCTCGAAGCGATTCATCACCCCATCAGCAATGATTTCGCTGAATT

GCGGGATTGCATTATCATGGTGCGAAANAGGCTCCGGAAATGGGATGCGA

CANATGCGAAAAGCTCTTTTATA

>TSBQ.R87.esd 650 0 650 ESD GOOD: 89-557

GCACGAGGAAACAACCCAAATGTGAAATATTTACTACCAACTTGCACCCG

TGAAATAGGAGAAGCTTTTCTATGCAGAATATGCTCAGAATAACAGACAA

TGTTTGGGTCACTTAAAGTCCACTTAGTTGGATGAACCACTAGAAGGGAG

GAGATACAATCCATCTTTAAAGAATTAATAAATTTGGGTAGATTTAATGC

CACCTTAACATGAGATTTCAAGGTGTGATAGGTAAGGATGCGAGACTGAC

TTCGAACGAGACAAAAGTGTCCATTAGCAAGAGAGTCTACGGGGCTACAG

ACTTTTGGGCAGAAGTGTGTATTCGCTTTACAAGAATCCAGTATAAAACT

TACTGTAAAATAATGGGTGAGTTAACAGATTAATGGTGAGATAGAAGAAA

AATTCCTTAGGTAAATCCAAGTAAGAGCTTAAGATTGGCTTAAGTTTTAA

GTATAATAAGGTACAGACT

>TSAS.R89.esd 755 0 755 ESD GOOD: 119-308

GCACGAGGTGGATCTTTACATCAATACTCTATTATAAAATGCAAGTCTGG

AAGTTATTGAGGAAGCTTTTGAATGAGTGTGATGGTTTCACCCCTTAAGT

AGGATACGACCAATCATTTTCTTGGCACCAGACTTCATATGCCTTTCACA

AAGCTTCCACCAGAACAAGATTCATATATTCATCAAAACC

>TSBV.R26.esd 431 0 431 ESD GOOD: 104-431

GCACGAGGGGGAGGAGTTTTACGAGAGGAAGGAGAGAGACCTAAACGGAC

TGGTAGAAAAGCTGACCGGTCTCGGTTTAGAAAGGAGCGATGCAGAAGCC

AAATTGCGCGATCTCTTCGGTATCGTGGTCAAAGAACGTCAAGAAGAAGC

ACTGGTACCAACTCAAGTGGATAAACTGCAATGGTACGCAAAAGAACGAG

TGATGAGGCGACATGTGATAGACTCGCTTCTTGAAAAAGAGGCGAAGCAA

CTGCGTTCAAGACGGAAGTAGGTGTACATAGACGCCGCCGATATAAAAAT

AGTATTTTGGTTGTTTTTTAAAAAANNA

>TSBJ.R82.esd 645 0 645 ESD GOOD: 96-605

GCACGAGGCTCAGTTTGGAGAGCAAATTTGTTCAAATTGTGGTTATTAAG

TCCTCTCAAATGACTTGTTGACGCAATATCAAAGGGTAGAAAGAATAGCG

AAGCAATTTAGTGAAGAATGGATTCAATCTACCACATGCATGTAAGCAAT

TTGAAGTAATTTATAAGGTATCGCCTGTTACAACACGTCAAGGTATTGCA

TCTGAAATCATAGATACTGCCAACAATGCAAGTCTCAATCAACTAGGATA

TAGCGCTGCTGATTAAAAATTCTTCTTCAACACGACCAATTTGGTTAACA

AATGAAATCGACCCCAGATATGCTGAAGTCCTGTGAATCCCAAAGTGCTG

TTAACTTCCAATGCAGCCACTTAGTACATGGGTTGAACTAAATGGGTTTG

ATTTTATAGCAGATTCGTATTAACAAACCGCTACTACTTATTCCTCCTTG

CGGAACGAATGCTTGCGCCCTATTAATGTGATTTGTCTGAGAGCAATTCC

TTTCGTAATA

>TSBN.R31.esd 586 0 586 ESD GOOD: 97-430

GCACGAGGCTTTCACCCCACCTCAAAAGTGGGATGTCTGATCTGTGCGGA

TGACGAGAAGATGGGTCGGGACTGCACACGGTTAGCCGCATGCGAACACG

TGACGTGTAATGAGTGTTTGAGGGCGGCACTGTCTGCGCATATTGCGGAA

GGCGTCACAGCTGGTGTGCTGCGGTGTCTGCATTGCAAGTCAGTGATGGA

TATAAATGAGGTGAAGGCGTTCGCCACGCCCTCACAATTCGCGGGCTATG

ACGGTCTGCTATTGCGACGCAGTCTGGCCCTTATGAGTGACGTAGTGCGA

TGTCCACGTCCTGACTGTTCGGGAACGTGTACTT

>TSBB.R45.esd 577 0 577 ESD GOOD: 93-575

GCACGAGGATTGGAGTTATACTGCTATTCATCACAAAATTTTTTTCGATA

ACTGACGAGCTTCCTCCAAAATCTGTCTTGCCTCGCTATAAATCCATATT

TCCCTTGCGGCAGTCGCTTACTCAACCATCATTGTTTGTAAACTTGCATA

TCATTTTCGGAGGGGTGCCAATAAGACTTTTGGTAACGTCGCAGTCACCC

CCTCACACATTGGAAGTTGGGCTACAGGTTCCTTATACTAAACATTGTGA

GGGGCAAATTCTGCAGTTCTTTCCCCTGGATTATTCTGATCAGATTTCCT

CAATTAGCTCAAGTTTGGTCGTTTATATTCGCTTGAGTACCTTCTAGCCG

TCCACTGGCTGCAACGGTGCTTCCACAGCTTGCTCCGAGATTTTCATCGC

TCATGATATGTGTGAAATTCGGTGCAGGTCCTGTGCTGTCTATAGCAAGA

ATTACGGACTGTGGGTTTTGTACATCGTCTTNN

>TSAJ.R49.esd 721 0 721 ESD GOOD: 103-719

GCACGAGGCAGAGATCGAAGCAACAACTTCTGTCGCCATGTTTACTGCAT

CCATTGCTGTAAAGCGTTCAGCTCCAAGTCCAGAAGGCACCGACGGAAAG

GATGGCATGGAATCCACGGTTTTACCTTCCACCTCAGAAGTCGAAACAAA

CGGTGCTCCTAGGGAGGATGAGTCAAGGCAGCCTAAGAAAGCAAAAGTTG

AATCGGAACCGGCGTCCGTACCCCAGCTTCTAACTCCTCTGCCCTCTCTC

AGTAACGATGTAGCACTGACAACTCCAAGTTTTGTAGCTCGGGGCATAAC

AAGGGTGGGCAACACAACCACCATGGTTCCGTCTGTTGTGCCTTCAGTTC

TGCCTCCTACCTCCACCACGGCCTCGACAGCTAGCACTTACGCGCTGGCA

CAGCACTTGGCCACCCTGCAGCAGCATCAGGCTCTATTGGCTTCCGCCGG

TCTCATCAACCCAGCCGACGCTGCTCACCAACAGCAGCAACAGCAACTAT

TCGAGCACTACAAACAGCAGCACCAACAACAAGCAGTCGCGGCGGCGGTG

GCTGCAGCGTTACTTTTGCAACAACAACAGCAACATCAATTGGCATTGCA

ACAGCATCTACAGANNN

>TSAI.R43.esd 648 0 648 ESD GOOD: 112-602

GCACGAGGGTTGAACAAGCGTGTTACCTTGCCTCCACCAGAGAGGTGCAA

TCACCACCCTTGTCCTCTGGATTCGCGTAAATGCGGAATTAGGGAGAGCC

ACAAGAAAGTCACCTTCGTCAGCTCTGTGGCAATGTTTGGTGATATTAGG

GACCAATGTACGCACGTAGCAACAGGAACCGCCGACCACTTCGAAATGGA

GGAATGGGAGTCCTCTTGCCTCCTCCTCTCCCACATGCTCCACTGCAATC

CATCTTCGGTGGGTCCTTTTCTTCTTCTTCTCCTTCTCCCACCGCATCCT

TCGCCGTCGTCCTTTTAATCGAAGAGGGCAGCTTGTGGCAGGGGAGAGGT

GCACCGATAAGAACATGAGCAATGGAGGCCATGGAAGTGGAGGAAGGCGT

CGCAGCGCGTGGAGGCCATTTGCCAGCCATGTGTCGAGGCTAACAGGCTG

TCTGTGCCCACACGGAGAAAAAGGACTTATTGAAAGAAGTG

>TSAU.R19.esd 656 0 656 ESD GOOD: 89-582

GCACGAGGCTTCTCCATTGCACATACTACGTGACTTTGCGCTGTTTCAGA

TGAAATATCCATGAATTCTCAAGCCATCTGAATGCTTTCCTTTTTGGAAC

AGGTGTTGAGGTGGCGTCTATGTGCAGCTATTTCATGCCCATATCCCTTG

ATCAAGTAACACGTCTTGCTTAACTTTAACTCATACCGTATTTTTAAAAA

TGGACTTCTTCAGCTCCATTTCGCGGCCCGGCTGCATAGCTTTATCAATA

ACGATCGGATACATGCTTGCAGACACAATCTCGATGTTCACATTCCAAAG

GGGGCTGGAGCTGCTTCTGTTTTGCACTCATCACCTTATAGTCGCCATCT

GTTTCACCTTCCTCCTGCCATAAACACAAATACTTTACTTGTTTATGGAT

GCGCTACTCACCATGGAGCAGGTTCCGCTGACTTCTCCAAGGCTGTTCAA

ATAGCATTAGACCTGGATCTACAGAATTGCTCCTATCTACGCCC

>TSAA.R20.esd 657 0 657 ESD GOOD: 102-628

GCACGAGGCGCGTTCGCTGTTTGCTCAAGGTTATCATTCCAGCATCATGA

ATCACCTGGCCATCTCAGCCAACGACCTCATTGCCATATTTCAGTCTGAT

GACGTATTGCTTTTCCGAATGCTTCTCACCCTTCTGTTGATTGAAAATAC

CGCTATAAAGAATGGCTGGGTAAATGGACTGAGAGTGCCGTCCGCGCACG

AACTCTTCACGAGCCTCCTCGAGTTAATTGGCTTTGATCGCTACTGCCTG

ATTGACTGGTTAGTGTCTCCGGAGACAGACTGCCTCGCCTACCTTCTGGC

CTACTCAAAGCGTCTAGCCGTGGCCTCCACCAACAACGACGACGACGAGG

TGCAGCAGCACCGCTGGCGTCCCCCTGCGTGTTGGCTGCAGCTTCACCGT

GAAGGCGTCAGGCAGGTAATGACGGATCTCGCTAAGAGTCTGAAGAAACT

GCAAATCAGTGGATCCTTGCCATTTGCGCCCGACCTACTCATTACTCGCA

TTCATGCGGCTGTAAAAGTTTTGTCCT

>TSAY.R49.esd 592 0 592 ESD GOOD: 93-591

GCACGAGGGGCAGCTGCTAGAACGGAATAAACATCTGGGAGCCATCAACC

AGATGGATCGCCTTGCGACCTTGGAATCAGAAACGCAGATGCAGGTCTTG

TGCATCCGGGAGTACATGAAAACTGCTCAAGAACTTAATGCCGTTCTTCA

AGAATCTTCAACAGACCTGGATGGGGTTCAGAGCAGTCTCTTCACCTTGA

AAAAGCAGTTGAACAAAGCTGAAGCGCTTGTCAATGTCCTTAAGGAAGAG

TACGAAAGGGTTTATCCCGGCCAGTCTGATTCCTTAATAACTGCGGCTTT

GACGAGGCTGAGCCAGCAAGCCATGGAAGTGGAAGCGGAAGGAGGCGAAC

CGCAGGACGATGGAGAGGGGAACCCCATTGTCGAACCTGCGGCACGCACC

ANCAACAACGACGTCCCCTCACACGAGCCTAAATAGACTCCAGTCCACCT

TTCCACCAAACCCTTTGTACATTTTACCTTGTGAATGAATTCGTAAANN

>TSAB.R74.esd 687 0 687 ESD GOOD: 100-437

GCACGAGGATTTGATCCGGTTGGATCTTATGCGAGGGAAGTATATCGCGC

GTGCGGTACAGGTGGTGCTATTTTGCAGCCACTCATGGACAGTCAGATTG

GCAATCCGGATCTCGCAGCTGATTGTGTTGAAATGGAAAAAGGTCCTGCC

GTCCGTCTTGCTAGGGATGTTTTTATTAGCGCGGCTGAAAGAGACATCTA

TTGTGGCGATGCCGTTGTCATCGACGTAATCACGGCTTCTGGGGTTAATC

GCATATGCTACCCACTACGGCGTGATTAGCTTTGCCTTTTGATGAATATA

TTGTTTCAATTTTTGGTCTAAAAAAAAAAAAAAAAAAA

>TSAQ.R73.esd 635 0 635 ESD GOOD: 90-357

GCACGAGGGTTTTTTTTTTTTTTTTTATGTGCTTGATGAGTGTTAAGTAA

AGTCAGGTAGGGCAGCTAATTTTGCTGCCAAAGCATGCCATGCACACCCG

CTCTGACTCCTGCGGCATTTCCGACCACGCAGATATTGGTCGGTCTGGGC

CGACGTGCCAACCGCGACTATAGCGCTCGACTGCTGCGTCGGGTGACAAA

CCAGCCTCCGACGTTAACGAAGGTAGAGCTGGGTGGAGAAAGGCTGGCGC

GTCGCGAGCAGGATTCGA

>TSCC.R17.esd 437 0 437 ESD GOOD: 102-416

GCACGAGGGCGGAACGATTAGTTCGCGAACGCGCTGAACGTGATCGGGCT

GCAGTGGTTTTATGCAGATCGATGGGTCTTTCGGCGGCAGCCAAGGCAGC

AGCAGCGAAGAAGGAGGAGGAGGTGACGGTAGTGGATGAGCGGCAGTTGC

CATTCAATTCAGCTTTCAATCCCGAACTCTCCACTCTGGCGGCGGAGCGA

CGGAACGCACATCGGTCAGAGGTGCATCGGAAGCAAAGCGAGGTCCGTCT

CCNTACAGATCTGTTATGTTTAAAAATCTCCCTGAATTTTAATCATGCTT

CTGTACCTATATTAA

>TSBH.R82.esd 621 0 621 ESD GOOD: 92-488

GCACGAGGCTCAGGTTTACTATGGTCGCGGACTGTTGAAGCGACCGACAG

TATTAGGCACGGTGTCAGCGAAGTTGAATGGACTAATGGCAAATGCCACA

TACAATGCCTGCTATGATCTGATGGATGGGAGAAAAGCTGTAGGCGGTCT

TCTTGAATTGCGTTTTAGGCATCGAGCACCACTTCACGGTGAAAAATTCA

GTGCTTATCAGAAACCTTGGCTGTGTCTTTCTACGTCCAAGACCAGGTCG

GTGCCCAAGAAAAAGACATGATCTTCGAAGGAGTTGGTAAAATGGAAGCT

ACAACTTTAGTAAAGATAGTTTTTTCATGTGATTTCACTAAAAAAACCTT

CAAGTAATCTAAAGTTTTGAAGCAGTAAAAAAAAAAACAAAAAAAAA

>TSAG.R25.esd 522 0 522 ESD GOOD: 103-522

GCACGAGGGTGGAATCCCTGGTCTATTTGTCCACCGTTCTGGAAAAGGAT

CTCCTCCCCCACCTTCGAGATTGTCTACCAGAGAGCAAACGTGGTCTCCT

GGACGCATTCCGTGACCAATCCCTCATTACAACCCTTGACGTCCGGGAAG

CCACTGCCCATCACCTCGCCTCTCGCATCTTTCCACCTCTCCTACTCCTC

GGTGGGCATCCAAACCGAGATTCCTCAGCAGACAAAGACACGTCACATCT

GCTACGGAAAATGGTAGCGGCGAACCCCGGTTGGACATCAAAAGCGGTTG

CGACGGAGCCCAGCGCCTACCTTCAGTCGGTTAATGCGGCGATATCACGC

TTATCAAGTGCTTTCAAGTCCCTCTCCCCACCTCCCGTTCCTCCGCCAGC

TATGCGCGCCCTCTGGATNN

>TSCH.R20.esd 739 0 739 ESD GOOD: 109-403

GCACGAGGCGGATGGGGAGCGCGCGTTTACCTAATGTAAGTTAATCTGTC

CCTCTCAATCATATCCTAGTGAGAAAGATAAAGTAACCATCACAGACCTC

AAAGGGCGCATGGACTGATTCTGTAGGTTGTACTGAAGGCGAAATAAACA

ACTCATGAACGTTTAAAAAAAAAAAAAAAAAAAAAAAAAAAAAAAAAAAA

AAAAAAAAAAAAAAAAAAAAAAAAAAAAAAAAAAAAAAAAAAAAAAAAAA

AAAAAAAAAAAAAAAAAAAAAAAAAAAAAAAAAAAAAAAAAAAAA

>TSCB.R73.esd 712 0 712 ESD GOOD: 99-629

GTCGACTGCTCTTATTACATACCACTCATTATAAGTTGGTGTTTGCGTAA

CTCGCTCGTTTTTTTCACCCCTTCCTATTCGCAGTGAACTGGCTTTCTGG

CCTTCATTATTTACTACGATCGGCGAGGCATTCTCGATTTCACAATGGCA

GAATCGGAAGAAAATATATCTTTGAAAGATCGTTATGAGTCTCTGAAGCT

GTTGTGCAGCGAGCAAGTTATGGTACTTCGTTTCCCGACTCTCCTACAGT

AACTTTTTAGGCCAACGAGCTCCTCAAGGAGGAATTAGATCGTCTCCGCA

AAAGCTTCCAAGAAATATCCAGACACCGGGCTTTCTTGTTAGAGAAGCTG

ATTGCACTGCAATCGAAACCGGCTCATAATGCTGAACAGGATAGCAAAAA

AACTGGTAAATCACCTGCCGTTCCGAATCATCAGAGCCGTAACAATCAGA

TTCAGCGAAGTAGGGCCCCTCCTGGAATCCGACGACCAACAATTCAACGT

CCTAAAGCCTATCCTCCTGATGGCATATCTG

>TSAQ.R15.esd 519 0 519 ESD GOOD: 88-374

GCACGAGGGATACTTCCACGATTAAAAGAATCGGGGCAATAAGATGATAT

GTAATGGTCAAATGTGTTCACCCGCGCAGGCTATTTTTCCGCCCTCCGGA

TATTCGTTTTACGAACTTGAACCTTTGTGCACACCAATGGCACTTCCCGT

TACATCTGTCGCTCTTCTATTTTTCTACGAGGACACTATTAATGACCAAA

GGCGACCAAGTTAGTTCGCGTAAATTTATCCTCGGCAATAAATAAACCAG

AAAATCCAAAACTTGGCTGAACACATTTAAGTGTACA

>TSCG.R92.esd 675 0 675 ESD GOOD: 103-494

GCACGAGGGTCTAACCCTCTACACTCTGCAAACAAAGCATGATTTCTTCT

CCTGGGCCGCTTGCCTTGGTGCCCTCCTGATGGCTCTCTTTATCGGTGGA

ATCTCGAACGTGTTCTTCGCTTCTCCTGCTATTCACCTAGCTCTATCGAT

CGGTGGCGCCTTCCTTTTTGCCTGCCTGTTGATCTTTGACACTCAGGTGA

TAATGCAGCGCTTCTCAGCGGAGGAATACATCGCCGCATCCATCACCCTC

TACTTGGATATCCTCAACCTTTTCCTTCACGTTTTACGTGTTCTCCAGGC

GGCAAACAATAAGTAGAAGCCGTAAATTCCTAACTTATTTTCTTCTGGAT

TTGTAATAAATCCTACTAATTTCGGAAAAAAAAAAAAAAAAA

>TSAD.R95.esd 757 0 757 ESD GOOD: 127-345

ATGACATCTAAATTCATGACTGACTCCCATCCGTATACCTCGTAAAGCGT

GATGAGCTTACCTTGAAGGAACTCACACAATTTTTTCGTGGCTGTTGATC

TAGACGAGTGGAAATTCGAAACGCTCTGTGACCTCTATCATACCCTTACG

GTTACCCATTCTGTCATATTTTGCAACACCCGTCGAAAGGCCGAGTGGCT

TGCTGATAAAATGCGTAAG

>TSAE.R73.esd 608 0 608 ESD GOOD: 91-527

GCACGAGGGTTTGTTTCTCAAAGATATCGTGGAAAAGTGAAATACGGCTG

TATGATCTCCCTCTAGAGTTCGGTGGTCTCGTATTGCCAGCTGGGCAGTT

GCTGTGGCAAGATGTGCTTGCTTCAAAAGTCATGCTATTGAACTTGGGAT

TCCTGATAGTGGGTCCCTATTCGCTCATCACTACAGTCGTATCGGCTGAC

CTTGGCACTCACCAATCACTGGCTGGTAACGCCAAAGCTCTGTCAACTGT

CGTTGCTATCATCGATGGCACTGGTTCAATAGGAGCTGCTTTTGGTCCTT

TCGTCGTGGGCTTGCTTCTTGACTACGGCTGGGCTGTCGTCTTTATAATG

CTCATTGCCTTTCTTGGAATTGCAATTTTGCTCCTTCTTCGACGAGTTGC

TAGGGAGCTCCGCGAATGCTGTCATCCCTCGGTTCAT

>TSBR.R47.esd 742 0 742 ESD GOOD: 112-462

GCACGAGGCTCCTTTGAAATTATCATTGTTGATGATGGGAGCGTTGATAA

TACTTACCAGGTTGCAATATCGTACTCCGAAAAGTATACATCCAATGTCG

TTCGTGTTTTGAAGCTTGCGCGGAATCGAGGAAAGGGTGCTGCAGTTCGC

ACCGTAAACCCGTCTAAAGCTTACGTCATCCGTTCTTTAATACTATTCTT

AGATGTGATCCATTATAGGGTATGCTTTCCGCTCGGGGTAAGTTCCTGCT

GTTTGCCGATGCGGATGGTGCTACACGCTTCCGGGACATCGAAAAACTGG

AGAAACAAATGGCATTTATGATTGCATCGAAATGGGACGGGAGGATGGCA

G

>TSAG.R49.esd 703 0 703 ESD GOOD: 89-526

GCACGAGGTCTAGTTTCACGCTTTGCGCTTGTAATGTCTCTATTTGCTTT

CAAGACGTGGGCTTTGTTCGTACAAACGAACTCAAAGCAGCAATTTTCTA

TTGCGGCCGTAAAGAAGCATGTACCTCTAATACGGTTTAGGGCGCGGCTA

AAGCGGACTTCTGAATCGAAGCGAGTCTCTGATGATAACACCTTCATGGA

ATCGACGTCCCACCCAAACCACTTCCGAAAACCTATTTCGGAGGACGAAG

TTTCGAATTTCCAGTTTGGTGGCTTGAATCCTTGATTGACATAAGCTCCC

ATATGAGGAGAATCATTGCAAACCTCTGCTCCTCAAATGTAATGGATTTA

CCAACTTGGAGTTAGATAAAGCCCTTCCAGTTGCAACGGCGTTCTTCTTT

TCCTATTCTTAGAATGCCCTCCCCCCTTTGTGAATATA

>TSBQ.R92.esd 786 0 786 ESD GOOD: 99-566

GCACGAGGGGCAGTTGAGAGAGGATGAGTTTAGGTCATTGGTAGACAAGC

TGTCGAAATATTTGCTAGAGACGATCTGGCTAAACACGCTGGGAACTTTG

CGCGAAAGAACCTTTTGAGCTTTGGGACTTGCTTCGGCAAATTTTCGAAG

AATAACAAATTTCGCCTTCATGTAACTGCTTTACCTTACTTGACGCCTTA

TGCCAAGTATCGTATATGGCTAAAACCATCCGCCGAGCAACATTTCCTCT

ACGGCCAGCATATTGTCAAATCCGGCTTGGCTCGTGTCTCGGAAGACACG

CCCCAGTACGCTGGCGTCATCGTAATGAATGTTGATGATATCCCCCTGGG

TTTTGGTGTCGCCGCGAAGTCTACCCTTCAAATGAAGAACACAGACCCTA

TGACAATTGTAGGTTTCCACCAAGCTGATATCGGAGAATATATCAGGAAC

GAGGAAACGATCGTCTAG

>TSAD.R46.esd 694 0 694 ESD GOOD: 99-622

GCACGAGNGGATAGCTCTNCACGGAAAAAGAGACGCTGCTGGCAAACGGG

GTATTGTCCCAGTGACCGCCAAAGGCACCGAGGGCATCGCGACGCGCGCT

TTCACCGATCACCACCACATAGGTCTGATACTTCGGCTTAACGGCAGTGA

CCGTCCAGGTATCTTTCATTCCGGAAAGTTTTGCCATCCGTTCTTGCTCT

TCAATCACCTCGTTATTATTGACGATGACATCCTTAGCAAAACGAAATAC

CGGATAGCCAGTATCTTTCAGTTTAAATACGCCACCCCAGGCCAGGTTTT

GCACGGGAGCAACAAAAAATGTCGCCACACTGAATACCAGACACAGGCTG

TCGAAGGTATTCCAGCGCGCTTTTTCATTCTCTTTTTTTCGCCTGATTGC

TATTACGCCGAGCGCAAAAATAAATAAGCCGACCAGGTAGCTGTACCACN

GGAAAAATCGTCAGGATTTCGGTCGACTCTTCCATATTGGTGGAGTGCAA

TGCCAGCAATGTATTGAAATTAGG

>TSAB.R70.esd 714 0 714 ESD GOOD: 101-533

GCACGAGGATCATCTGGAACCCGTAGCGGGCGAAGTGGGTGCTGGCGGTA

GCAGTGGAACGACGATAGAGGCGAAGCCGCAGCTAAAAAATCTGATTGGC

GACGCGACGCGTTTTGTGCCCACCTCCCTGAAGGTGCGCCGCACGGTAAA

AGATGCTCACGGTCGGCTTATCCAAATCGGCGGTGGAATCAGCTCCTCTG

GAATTCGGTCTGGGAGCAGTTTGCCACCTTCTCAGCGGCTGGGAAGTGTT

CCTGGAAGGGCTCTCGGTGTGCCTGCATCAGTGGTGTCTTCGAAGCCCAA

CGTCACGAATAAGGACGCAGCGTACGAGGAGTTTATGCGTGAAATGGAGG

GACTACTATAGTCTACGTGCAGTTAGCATTGTGTAAATAAGAATATACGT

TTTAAAGAACCAAAAAAAAAAAAAAAAAAAAAA

>TSBV.R85.esd 565 0 565 ESD GOOD: 93-559

CGGCACGAGCCTGTGCCGAATTCGGCACGAGGATTATCAACTTGGCTGTG

GCCCTTCATAGGTTCGAATAATGTTTGACTATGCATCTCCACCTCTTATA

GAACTATAGCAGATCTATCAAATCCACCTTCCAACTTAACCGGTAACCCT

ACTTGGCTTGAAAATATGGAGTCAGGAAACAAAATTGCCGTTCTCTCTGG

TGATATTTTATTGGCCAGAGCGTCGCTTAACCTGGCTTCCTTTCGTGTAC

CAAAGGCATGTTTTGTTTCACCCATTAGTCGAGGTCTATTCTTCCTTGTT

TTAAGTGTCGTCCTACATGTCTTCATAATAGGTTGTGGAGATGGTGTCTG

GCGCAATAGCGGATGCTATGAGGGCAGAATTTTCCGACCTTGCGGCAGTA

TCTTTAACCTCAAACACCGCGGTTGCTTTCCTGTCTAAGCTGTGGCTTGA

GCACATAACATTGAGGC

>TSAR.R22.esd 628 0 628 ESD GOOD: 88-509

CGGCACGAGGAGAAGGCAATCGACTTCGCTTGTTTGTCTTTTCAAGTCAG

AAACAGCTACTAATCAAATATAAAATGAAAAAAGGTGGAAAGTAAAAAAC

ACTCGACAGCTCCAATAAAAAAAACTGTTAAAGAATTCATATTTTGGCAT

CAATTCCACCCAAAAAAACTATCCAGTTAGAACTACCTTCCCAGCCCTGC

CTCAGCTTCACCATGAAATATGGGGCGAGATTCCAAGCAATCAGACTTAA

GAATCAAATATCACTAGAAAGCCTACAGCAGACGCAAATAACGTTTAAAA

ATCCACTTGACAGTAAAACCGATAAAGCAATTTCTCAGAAAGTTGACGAA

AAGACTACATAACTAATTAGAGGGAATTTAAAAAGGATAGCTCACACAAA

AACCCAGCTCTCTAATAAAGTT

>TSBU.R42.esd 760 0 760 ESD GOOD: 96-567

GCACGAGGCTTACTGGCCAGCGACACCGAAAGATTCCAGTCCTTTCTCCG

CTCTTTTGGTTGGTGTGTGGACTTCGCACAACACCCAGGCTGGTGTGGGC

CTCTGCAACGATCTCAGTCCTCTCGGCCGAATCACTCTGACGCTACTACG

GCGCACCTCTCTGGCTTTGATGCACGACTACAGTCTGCTGGCGTTGAACG

GTGGCGCTGCAGAGAGCCTCCAACAGAATGCGCCCGACGGGCGAAAGACC

TTCTTTTATTGGGCTGACGCCTTCACTGAGAATGGGGCGTTGTGCCCCTC

CAAACGGACGAAGATCGAAGCAAAGATCGTTTGGAAGAATCGTTCCCCAG

GCGGAAGGATTTCCGAGGCTGCAGTTTCTGTGGCTAAATCATGGGAACGA

CCGCTCTGGATTCCCGGCTGGGCAAATGAAAGGTGAGGCAGGTTCGGATA

CGCACAGCAAGCCAGGAACATT

>TSBK.R74.esd 633 0 633 ESD GOOD: 92-529

GCACGAGGGCCAGAAGATCGGTGACTACTTGGATCAGAACTGTGAAAACT

GATCCTCTTCGCCTCGCCACATGTCTGCTTCACCATAATCTACGATTGGC

GCTGTCAAAGATCGCCTCTCCCCTCCTCTCCTACAGGCTCCCAACGCATC

AATCGTCCTCGCGAAAACTGCTCCTTCCTCTCCTCTGCCCATCCGCTCGT

TGTGGCCGTCTTCTTGCGCATTTTCAGTTTTAATCAACTCTCTCCTATTC

TCTATCCTCTTCCACTATTTCTGCTCCCCTTCATTTCTCCTCCATGGAGT

TGACTATTTTTCTAAGTCAAATGCGTACGTGAGGGAGGGGAAGGTCACTC

CTCTTTCACCACGCATTTTCTCGCGTGAAGGAGGTCGTGAGGCTGTATAT

ATGCGTTATATCTATAAGAAAAAAAAAAAAAAAAAAAA

>TSBG.R81.esd 785 0 785 ESD GOOD: 102-392

GCACGAGCACTGGTTCAGCAAAGCCTAAGACTGACTAGAGCAATGAATTT

AACGATATACTTTGCACACACTGCTCTAGACCATTGTCCATATTGAGCCT

TTTTTTGGATTTCCCGATAGTTGTAGAGAGTTCTAGTAGCGCTATATTTG

TGTCTCTGTTTGTGCCCCTGTATATACAAGCCTGATTGTTCTACACGTTT

TTGGTTTAAATAACGCTCCTTAATTCATCGATGTATAACCCTCCATGACC

CAATAAATGCTGAAGGCGCTAAAAAAAAAAAAAAAAAAAAA

>TSAZ.R91.esd 768 0 768 ESD GOOD: 100-599

GCACGAGGGGCACATGCACCCTTCACCTCTCAACTCTCGAAACAGGATGT

GAAAATTGGTTGCTCTCGCTCTCTCACTCGCACGGTCGGTTGGTCACGGC

TATAAACTAAGCGACTGCAAACGGAAACAGTCTGTTTACGTCCAAAGTCC

GTGTGTGTCATATGTAGGCGTGTTGTGCATGCCAAATTCAATCGATAGGC

CCTCCTTGACTGGTGACAATCAGTTATTAAGATCTAAAAGTGGTTGACAG

AAAAAGGTGTATTTCAAGGCAGGCAAACCGGCTGCCGTGATGATGTGACA

ACCAGGATTGAAGGTGGAATTGAGTATCTTACTCCTACCATCGTACTTCC

CTCCAAAGGCTCGAGAAAATGTCTTCCTCAAAGCTTCCATGAGATCGCGT

TCACAGGACGGATTTGTACTTGACTCCTTTGCTTCCCTTTTTAGCTATCA

AGGACGGTCAAATATCCTCTTCTGTTTTTCCCCACTCCTTTGATTCTCTC

>TSAO.R70.esd 864 0 864 ESD GOOD: 101-612

GCACGAGGCTTTTACAGATCTTTGAGTCACACACGGGGGCCCTGTCATCG

CACCTCTTCGGTACCTTCGTGCTGCCTTATCTGCGGCAAATATATACGGG

TGTGCGCAGTCGTCTGCAGAACCAGCACGACTTCTCTGCGGACAAGTGCC

CACCTATTATAGTGTTTGCACTGGGTGGACACTATGTCCTTAGGGAAATC

GCTTCTGTGGGTTTCGAAGTAATTGGCTTGGACTGGACGATCGATCCAAA

GCGGGCGCGGCAAATTATCGGTTCACCAATTACGCTTCAGGGCAACCTAG

ATCCCTGTGCATTGTACTCTACAGAGGAGGACTTGGAGAATCGCGTGAAG

GAGATGCTGAACTCGTTCGCGTTCAAGGAAAGACGTTACATTGCCAACCT

CGGCCACGGCATCTACCCCGATGTGGAGCCCGAGAAGGTGGCTACTTTTG

TCGATTTGGTGCATCGGTACTCTGCTGATGACATCCCCAAATAATCGTCG

ACTTCCCCCGCA

>TSBZ.R14.esd 589 0 589 ESD GOOD: 94-536

GCACGAGGACGAAGGCTGCCCCGCCAGCTCAAACAGCTAGGGTCACGAGA

ATTCTCGGTCGCACTGGCTCTCAGGGTCAATGTACACAGGTTCGCGTCGA

GCTTGTTGAAAACAAGCGAAGTATCATCCGCAATGTGAAAGGTCCTGTTC

GCGAGGGCGATATTCTAGTCCTTCTCGAAACAGAAAGGGAAGCTAGACGA

TTGCGATAAATTGGCAGCAGACGTGGATTAATACATTCAATGGTATTGTT

TCTTCCCTTATCATACTGAGATACAGGTCGTTTTACTTAGTGCGCGTTGG

CCAGCTTATTAACCCAAGTGGTGGTGGGTTAACGGTTAACTCTGTGTCTA

TGCTCATCATGCATCGTTTATTCTCTATCAAAAAAAAAAAAAAAAAAAAA

AAAAAAAAAAAAAAAAAAAAAAAAAAAAAAAAAAAAAAACCCA

>TSBV.R11.esd 537 0 537 ESD GOOD: 101-537

GCACGAGGCACTGACGCCGGAAGGGGATGAACCGCTTCCCGGTGCCGTTC

GTGGATTGAGTACTACCGCAGGCAGGGTATGCATGAGTACGCTGATATGA

TTCAACGGCAGGCTCAGCAGCAGCAGCTTCAGCAATATCAACAGAGTTCT

GGTCCGTCGTCTCAGCCCGGCACCGCCCCAACTGGAGTCGCCCCGGTTTC

CCATCCTAGCCAAAGCGGTCAGCCATCAGGCGCGCACTCGACCGTTCAGC

AGGGTGGACCGCAATCATCCGGTGGCTACCCGGGTGATCCAAGTCAACAG

AGCGACTATGCTCAGTGGCAGCAATGGCAACAGTGGCAAGCGTGGCAGGC

ATGGCATCAACAACAGCAGCAGCAACAGCAACCACACAGTAGCAGTGGTG

GCGGCGCCGCCAGCGGCTCTGGTGCTCCCGGAGGNNG

>TSAZ.R12.esd 661 0 661 ESD GOOD: 89-632

GCACGAGGGTGAAATCACTGCCGACATTGCGGGATGCATTTAACAGGATG

GCCTCGTGGAACAACCGAAAAGTTTTTGCATCCTGCACCTGCTTCTAAGA

CGATACCTTTATATCCTCTGAAAAGTTACAGTTTTGGAAAAAAGGAGCCA

AATTATGAACGTGATCGTAGCGTGCCGGCACGATTTCAACGCCTACAAGA

GGATTTCGAGAAGTACGGGATGAGGCATTCCGTAGAGGGTGTTCTTCTGG

TCCATGAACACAATCTCCCTCACGTGCTTCTCCTTCAATTGGGAACATTT

TTCAAACTTCCCGGTGGTGAATTGCATCCTGGAGAGGAGGAAATGGAGGG

TTTGAAGAGATTACTCTCGGATATGTTGGGGAGGACAGATGGAGTGCCAG

TGGAATGGACTCCAGAGGAGTGCATTGGTAACTGGTGGCGGACGAATTTC

GAGCCTCCGCGATATCCCTACATCCCTGCTCACGTGACGAAGCCCAAGGA

GCACACTCGTCTCTATCTGATCCAGCTAACCGAAAAGACACTTT

>TSAT.R82.esd 735 0 735 ESD GOOD: 106-641

GCACGAGGGTTCGTTATTGCAGTCACACTGTTCATGATTGGTTTGCTACG

CAACCTATCTGGGAAGAAGCTGCTCCTGCACTTTCCCTCTATTTTTGAGA

CTGAGTTGACTCCACCCACGCGACGCACTTTATTAAGCCCATTTGACCGG

TGTTTTATAGAGGGAGTGTTCAAGGTCATAAGGAAGGAGCATATCACCCA

ACAAAGGAAGCCTAAGGACTGATGTTTATTCAGGCTTTATGATGTTTGTC

TAATTCAATCCCATTTGGCAAAAAATTCGGCTAAACTCAGCGCCCATTAA

GTGTTTTTCAAGGCGCAACTGATTGAAGCACTTTGCTCAGCATTACGAAG

AGTTTTCATTTTTTTCCAATTATCTTTCTGTTCTCGTTTCCAAATCCGAT

CCAAGTGGCTGACAGATGTTCAATCGTGGGTGATTCGACAACTGCTTGGT

TTACTCTTCCACTGTCAGCTCATACAACATTAGCCAGTCATCACAGACAC

AGACCCCCTGGGAGAGGCCCCTCACCTTTAATTTTA

>TSAV.R20.esd 585 0 585 ESD GOOD: 113-584

GCACGAGGCTCATTTTAAAATCACTTTGAAGTTCATTAATGTTTACACTG

CTTCAGAAAAAGGTGCCATTTCAGACTAATGCCTCGAGAACTCAGTGACT

TTGGAATTGTACTTTTCTACCTGTATTTCCTCATTGGCCCCAGTGGCTAT

GCTTACATTATTGGTTACCGCAAGGTACAGTTTTGCTAACCCAATCTTTT

CATCAAATGTAACCAGTTTTACTAAATCCTGGCCTGGCAGAATTCGATCA

CCTCTGGGCGCACAATGCGATCACGGCTTCACTTCTTCACAAAATACCCG

AGGTGTTATTTTTGAGGTAGTGCATATTGAAGGGCATTGGTGCTGTCGAT

TGGTGGATAAGGCTTTTAGCTCCGTATTTGATCATTTTTTGTCGTTTCAT

GGTCACATTCATTTCGCTGCGCATAGTGCTGCGGTGCTTGTTTTTTCTGA

CTTTTTATCGTGTGGTGTATNT

>TSAD.R23.esd 741 0 741 ESD GOOD: 99-554

GCACGAGGCAATGGCGAAGCACCTCAGTGATTCCTTTGTTAAAACTGAAG

TTTTAATCTACCAACCAACACATTACACCTTTCCCTTGAAATTCCTACCA

GAAGGCCTTCGGTTATGTTTGTCTAAGATTTCAATTACGCAATTTCTTTG

CATAAAACATCATTAGATGTGTAATTAAGCTTCTATTCCCAAAATGTTAT

AATTTGCGGCTGCTACCAATTCATATCTGCGCTTCTAATTACTAGTAAAG

TAACGGGTATTGCTTAGGGGTTCGCTACAGTGATCCACTAAAACGGTTGA

TACATCGAAAACCAATTGCCAAACCGACTGTATTGTGAGCAGTTCACTCA

AGTAACTATACCATTACCCTATTGTTGCATGTACCATGGTGCCAACTGAT

CTTATCAACTCTACCAATTGGATGGTGCATTANAAAGACCGTCCAATTTT

TAACGC

>TSAP.R68.esd 479 0 479 ESD GOOD: 91-479

GCACGAGGAGTAGGTGTGAATAGGATTAGGGTCGGTTATGATCCCGATTC

CCTACTTCCGTACGATCGCTCTCATTCTGGCGCTTATTATGCTGATTTCG

GGTCTGATCCTCACTAGTTTGAGGTGCCGCAGCAATATATTCAAGGAAAG

GTCTTGTTCAATAGGTCTCGCTCTGTTCTCCATTGGAATTGTGGCGACGG

TACCTCTTGTCGTCATGGCAGTAGCCAAGAACTCAGTTATCAAAATATGC

GCTTGTAAAGCTTCCGGTGTTGACGCCAACAACGAGTTGAGTAAAAAAAC

TGCAAAGGAGGATTTTGCGGAAACAATTTGAAGGTACTTCATATTCCATA

CACGCTCTCGCTGATCTTAAGGGATGGCCTGTCTAGTNN

>TSAB.R91.esd 756 0 756 ESD GOOD: 106-606

GCACGAGGCTCTACCACGCTCTACCCATATTTTTCTTTCTCTTATCAAGA

TGCTTTTCTCGCTCCTTCACCACTTGTATTAAAAGTGTTGCAATTTTGGC

GGCAACAGTATGTACAACCTTCTTTCTTGTTTTTCTACCATTCCTCACTT

CCTTTGATCGGCTGAAACATGTTGGGTTGCGCATGTTTCCCGTCGATCGG

GGTCTTTTTGAGGATAAGGTAGGGAATTTCTGGTGCATCTCTTCGGTCCT

CATAAAGTGGAGGCAGATTTTCAGCACTAACGTGCTTTTTTACATCTGTT

TCCTTGTTACTCTCCTCACTTCACTCCCTGCGTGTTTGAGCCTGATCCGA

AGACCAAGCATTTCTCGACTCCTTCTGACAGAGGTAATCGTGTCTCTCTC

CTTTTTCCTCTTCTCCTATCAGGTGCATGAGAAATCAATACTTCTTGTTG

CCATTCCTGTGCTTTGTCTTCTCCCACTATTCCCCTTATCGTCCTACTAT

T

>TSBF.R17.esd 593 0 593 ESD GOOD: 95-200

CCGGGCTGCAGGAATTNCGGCACGAGGGTTTGCTCTCATCTTCTTGGTGG

CCGTCGTTTTGCGAGACGACTCGAATTCGTGCCATACCTTCGCTGCTTCG

CCCTTA

>TSBL.R29.esd 604 0 604 ESD GOOD: 87-363

GCACGNAGGCAGTGTACGGTAGGAGCCATCTGCACCCTATCCCTCCTTGC

CCCATCGCGTCGAGTGACCTCTTTTCTCTCCCCTTTGACCTTTCTCCTGG

TGGGATAATAATCAAAGCTGTCGCTATTGCTAGCACCCTCGGCATTTACG

CATCGAAATCAATCCTCTTCCTCTTTTTGCTACCTTGATGTGAAATCGTC

ATTTACTTAATCTTTTTTATACTTGTTCAAAATAAATCTGTAAATTAATC

TTTAAAAAAAAAAAAAAAAAAAAAAAA

>TSAG.R32.esd 650 0 650 ESD GOOD: 94-551

ATTCGGCACGAGGGCTTATCCCGATCAGCTGACCTACGATCAAAGTCAGT

ACACTGCTACAGGCTACGAGGACTACTCGGCTCAGTATGGCCAAACCGAT

GCATGGGGCAAAATGCTTACAACCGATAGTCGAGGACGAGCGCGTGCACA

TCCTTATGCCAGACCAGGAGGGAAGGAGGAAACTGCTTGAGTGCCTCCTA

AACGCTTAAAATCTCCCAATTTTAAACTCCATTCATGCATTTGCGCGATC

GTTTTCGCGAGGACACTTCACCTGTAAAATTGCGTATAAACTGCCCCTCC

CTAAAATTCATAACTGTGTAGTGAAATACTTGCCCGTATTATATATATAT

ATACATATATGCAACCAATCTTCATCGCGTGTGCATAATGTTTTAAATGT

TTCCATCTGTTAATTCATTAACAAAAATCAATTTAAAATTAAAAAAAAAA

AAAAAAAA

>TSAP.R80.esd 694 0 694 ESD GOOD: 90-545

GCACGAGGGTCGGTTCTGCAGGAGGATGGCTTCCGAAGTTGAAAAGGCTC

AGACCACTTGTCCCTCAGAGGGAGACACTATTTTCGGCAAAATCGTTCGC

AAGGAAATCCCAACGAAGTTCCTTTATGAGGACGATCAGTGTGTAGCTTT

TGATGACATTTCACCTCAAGCCCCCACACACTTTCTGGTTCTACCCAAAA

AACCCATCCAACAGCTGGATTCCGCCACCGAGGATGACGAAGCGCTCCTG

GGTCATTTGCTCATAGTGGCAAAGAAAGTTGCTGCACAGAAGAATCTGGC

GAAAGGCTATCGGGTTGTGATTAATAACGGGCGTGAAGGTTGTCAATCGG

TCTACCATCTGCATCTCCACGTTGTTGGTGGAAGACAGCTAAGGTGGCCA

CCATGTTAGACCGGTTAATTAATAAACGCTTGAATTAAAAAAAAAAAAAA

AAAAAA

>TSCF.R83.esd 671 0 671 ESD GOOD: 98-621

GCACGAGGATCCACTGACTCGAGGGCGTCTTATTTGGATGAAAGCAAGCG

TATAGCCATGGCTTCATGGAGTTCGATTGGTTCCGCCGGTCGGGCCCGGA

CTCGAACTGAGAACAATCCAATACAACCATCTTACGCCTACCGTCATTTG

TCGCCTCCTGGTAACAGGGATCAGAATGAACAGCTGGAAGCTGTGAATGT

TTCTCAAGTGGAAAGGCGCGGCTCAGCCTCGGAAAATTCGAGCCTGTTTC

AGCGATGGCCTCCCTATGTGGACCCAACTAACTCTCAGGTTTCGCTTGCA

GAGGATAGTGGTTGCATGAATTCGATCCTCAAGCACCTTCAATATGGTGT

CTCAAATCAAGTCAAGGGTCTAGATCTCACCAAGGATCAAAAAAAATTAT

CCGATGATGTCACGTTTACAGCACATCAGGACACTGGTTTGAACTCCTTA

AACGGTGCTTCCGCTAGCGGAGACACTTCCTTTCGCCATTTGCAATGACG

GATTGATCGGCTGACCATTTGAAT

>TSBK.R2.esd 565 0 565 ESD GOOD: 90-560

GCACGAGGCTTTGACGACTTTGGCTCCCATGAACCTATATTCCCACCTCC

TTTTCCACTTATCTTTCCCGAAGAACAGATTTCAGCCTCCTCGCCTTCGG

TTGTACATACGTCTAGTGGACTAACCAACCTGACGACGGTTGTTCCAGGT

AGCGAGGCCCACAGAAATTTCGATCAGAACCATCCTGCTGTCTCAACACA

CCTCTCGAATCCCCTTGTCGCTCCGTCAAGTGGTTCAAGGAAGAGGTCTT

TAACCGGTTCAGATTCCCCAGAAAGTACCCACGTCTCTCCCCTGAAACGA

CTCACTCTCACCCCGTTTTAATTTCCTTTTCCACCCCTCCTTCTTGCTGT

ATCATAGATTTTATCTTGTTGAAGCAACAGGCACACATACATTCATACTG

ATTTTGTAAAGCATTGTGTTTGGTTTCTTCCCAAAAAAAAAAAAAAAAAA

AAAAAAAAAAAAAAACTCGAG

>TSBC.R83.esd 625 0 625 ESD GOOD: 91-400

GCACGAGGTTGATATATCAAGTGCCTCCTGNAAGACTATCTGATGAACAG

TTGGCTCTGCTGTATGAAACTCATGCCTTTGTTGGCAGCCTGGTGCACTG

GGCTCATTACGTCCCCAGTTGGGCCATCACCATGCTCTGGATACCGTTGT

GCGAGGCTACGGCGTCTCTCTTACTCTCCAAAGTTCAGAGGTGCATAGAA

GGAGGGGAAAAAAAAAAAAAAAAAAAAAAAAAAAAAAAAAAAAAAAAAAA

AAAAAAAAAAAAAAAAAAAAAAAAAAAAAAAAAAAAAAAAAAAAAAAAAA

AAAAAAAAAA

>TSAV.R40.esd 731 0 731 ESD GOOD: 102-340

GCACGAGGCTTGGCAGACTCTCTCCGAAAAGTGGGCACTACCAAAAAACT

CGTGTGCATGGTTACTAACTCTGTTTCGGACAAGATGAGGCCGGCTCTTG

AGGAGTTTTTCGACCATATTGAGTTGGTCGATGTTCTGGATAGCACCGAT

TCGGAAAATCTAAAGCTTCTTTCAAGGCCTGATCTTGGTGTGACCTTTAC

GAAGCTTCATTGCTGGCGGCTTACACAGTATTCCAAATG

>TSBJ.R86.esd 511 0 511 ESD GOOD: 99-511

CGGCACGAGGGGGTTATGGACTATCTCGTGNNAGACCTTTTGGGGTTAAC

CTCTCTTGGTTCAACCTCCTTCTCCTCATCTTCCTCACTATTCTTCTCCT

ACTTGCATGTGAGTCTCGAAAAACATGAGTGAATTGTGAGTGCCCGCAGC

TAATGTGAACTTCCGTCACAATTCGTGTTATAGCCCACGTGGTGACACGC

AGCCTCAGCGGCTCTGGACTCGGAGTTACCGATGAGCAGAAGAACCTACC

CCCACTGTCCAGCACCGAAGACGCGTCCAGCCGCGGTAGCCCACAATTGT

GGAGCCAGGGCTATGGGCTGCATGNGCAGAGGAAACTTTGCCACACCGTT

CCATGTTTCACCGAAGCTCCGCACCTTAGTCGACTCAGCCGCTTCTTCAC

CCTTTGGGGANNN

>TSBD.R35.esd 611 0 611 ESD GOOD: 83-573

GCACGAGGGTGGCTTATCACGAGGCGGGGCACGCGACAGTGGGGTGGTTC

CTGGAGCACTGCAATCCTCTTCTTAAGATCTCCATTATTCCGCGGGGCAA

GGCGTTGGGCTACACGCAATACCAGCCACGTGACGCGTACCTGCATACTC

AGGCCCAGATGCTCGATGAAATGTGTCTCGCCCTTGGCGGCCGCGCCAGC

GAGCAGGTCTTCTTCGGCAAGGTCGGCTCTGGTGCTATGGACGACCTCCA

ACGCGTTACTCGCTCTGCTTACGCTCAGGTTGTTCAACTGGGTTTCTCTC

CAAAAGTCGGCAATCTCTCCTTCGATCTACCTCAACAGGGTGAGCCGGTT

ATGACCAAGCCCTACTCAGAGCACACTGCTCAAATAATCGACGAGGAGGT

GCGGGATCTGGTTGCCAGGGCCTATGACCGAACCCTCAAACTTGTTGAAG

AGCATAAAAGTCATATCGAGGCGTTAGCACTTCGACTTTTG

>TSBS.R82.esd 541 0 541 ESD GOOD: 90-520

GCACGAGGACCATTCGGTAATCCGCAGTTGCACCAACGGGAAGCGCGAGG

GTGGCGAATTTGTCGGCCTGACGGTGAGTTCGGGCGGCGAGTGGATCTAC

GCGGTGTGTGAGGATCGTCTTCTCTACTGCTTTAACGTTAGCAGCGGTGG

CAAGTTGGAACGAACCCTTCCGGTACACGAAAAGGAGATTCTTGGGGTGG

CTCACCATCCACACCAAAACCTCATCGCCACATTCTCTGAGGATGGGCTG

TTGCGACTTTGGAAGCCTTGAACTGAGTCTCTATGCGTGTAGGCGAAGAT

TGTTTTTATACATCTGATGTTGCATAAATACAGTGTCTTAATACGCATTA

AAAAAAAAAAAAAAAAAAAAAAAAAAAAAAAAAAAAAAAAAAAAAAAAAA

AAAAAAAAAAAAAAAAAAAAAAAAAAAAAAA

>TSCD.R4.esd 551 0 551 ESD GOOD: 91-551

GCACGAGGGCCCATAGGGGTTCAACCGGTGCCTCCCGTCCCTCAACCCAG

ACAGGTGGAGGGGGGACCCCTGAGGACGCTCAATCTGCTGCCGATGTTGA

CGTCTCGGGATCAAACGAGGATACCCTCAACGAGACTAACAGCTCCAACA

CCAACTCCACCAATAAAAATAATTCCACTAAATCGCNCTTGAAGTTNGGN

ANNTATCNTGGNCNCNCNNTNTNTNCNNTNCNTNCCGGGTTANTACGCCC

TCCCCCCACCCACCCGTTGTCCCCCCCTTTTTAAAACGCCCGTTTTCCCG

TTTTTCCGCCCCTTTTCCCCAGTTTTCCCTTTTCCCAAAAGTTNTTTCCC

CGTTTCCCCGTTTCCCCGTTTTCCACGNTCATAANNGCCTGACTNTNNGA

ATACTTCGTGCTTTACCCCCGCTGCTGCCGCCTCTCCTGGGAGGGAAAAG

GAAGAAGANNN

>TSAF.R92.esd 340 0 340 ESD GOOD: 148-276

CCAGTGTCACGGTCGCGTCGGTCGAGGGACAGTCGATCGATGTCAATGTC

TCACTCGCGTTCGTACTCTCGCTCGATTCCGTCCCCTCGCCCTCCAGAAA

CCCATTTCACTGAAGCACGCATAAAGACG

>TSBN.R2.esd 467 0 467 ESD GOOD: 87-365

GCACGAGGATTTCAAAAATAAACTCGTTTTAAACAGGATTACTTTGTAGA

AACAGTTGTGTCCACAGAAATTAGGAGTGGAATCAAGGATTAGCTGCAAG

AAGATCCAATATTTCGGGGAGGCTGGAAAAATCAACTGCCATAATCACAT

ATGTGATAGTTTCCGAATCTCCATCAACAGCAACATCCACGAACTCAGTT

TCAACCACANGCAAAGGGCCACCCTTTCGTCACTATTTCCATTTCGGCTT

GAATGTATATAAGTTCATCCTCATCTTCA

>TSBS.R22.esd 537 0 537 ESD GOOD: 94-531

GCACGAGGGGCGAACTCACTCCCACAGAGGGTGAACGCAGGATTAATCAT

CGAGCCAAAATCGGCAAGTATGATCAACACTCGGCGGACCAACTGAATCT

CTCTGAGTCTCCAGCCGAATACCTTATGCGCCTCTTCAATCTCACCATCC

AGGAAGCAAGAGGCACATTGGGCAAATTTGGTTTGGAGAGCCACGCGCAC

ACAATCCCCAACGCTGACCTCTCTGGTGGCCAAAAGGCTCGTGTCGCTTT

TGCCGAGCTCTCGAAACGTGCGCCCGACATTCTTATCCTCGATGAACCCA

CCAACAATCTGGACATTGAGTCTATAGATGCACTGGCCGAGGCTATTAAC

AACTTCGAAGGCGGTGTGATCATAGTGAGTCATGACGAACGCCTAATTCG

TGACACAGAGTGCATTCTCTGGATCATTGAAGATCAGA

>TSBM.R93.esd 592 0 592 ESD GOOD: 105-575

GCACGAGGGTCAGCGGTGACAGCGAAGTCAACGTAATTGTCATTCTGTCT

CTTTCTCCCCCCTCCCTCCTCCACCTTGTTGGCTGTGCTTCTTCTGCTCC

CATTTTCATCACTGGTTATGTGCCCCTACCATTGGCTCCTTTTTTGCTCG

AGTGGTGTATCCTTGTCTCCTCTCTTTTGTGCTCGTGGTAACTCATGCTA

CATATCATTTTTTTCCCTTTCTCACTTTTCTGCACTTTTTACTTTAAACA

TCACTTTCTTATTTTACTTTATCAGTCGTGCATGTGTCTTTTTTGGGTGT

GACTTTTGAGCATTATAGGTGAGATGTAGAAATGAGTCCTTCAATGAGCA

TCTTGCTCGAGCTCACTTGATGGGATGGATTTCTCGGAGGGATTTTCGGT

ATTTTCGCAGACATTGTAACTTGTACCTGATTCGTTCTCTTTAACTCGGA

TGGAGATTATACGCGTTTATA

>TSCG.R49.esd 575 0 575 ESD GOOD: 98-449

GCACGAGGTAGGATTCCAAAGTATTACATGATTGTGTCATCTTGCTGCAG

TGATTTGTTTGATGCAACAATCGATAGCTTGGATCCCGCCTTGTATTGGC

AGTAGATCAGGTCTGCCCTTTCTGCACTCCTTTTATTCTCCTTTTCCTCT

CTCTTCAACAAAGACTTATCACCTGCGAAATTGGTCTGGTCAAGCCATGA

CCACGTAGAGCCGATCCTGCCATCCATTACGAAGACATGAGACTAGAGAA

GCAAGGAGGTAGAGGTAACTTTGGTGTGATCTACCTGGGTCGTATACGCT

GGATGAAGATGGCAGTGAAGAAAATTAACAAAAGCCCAATCCAGGAAGCC

TT

>TSAH.R83.esd 754 0 754 ESD GOOD: 105-589

GCACGAGGCGGACATCCATAGAGTGCAAGTAAAGGGTCAGCAAAATGTCC

CTTCCACGGTCACCAACGTCACCGCTGCCGCCGCCGCCACCTCCTCCTCC

CCCGCCTTCTCATAATGCCGCGTGTACACAGCCTCGATAGCCATGTCCAT

CGCCTGCTCCTCCCTTCTCTGTCTCAGTGCAATGGCGTCTCCACTCTATC

GAATTAACTTCACCCTCCCCTCATGTGTCATTTACCGCGTATTTTACCTA

GCCCTCCTTCCGTGCCTCTCGTTTCCTATCTTTTCAACAACTGCTAATGA

TGTCCCTACTTGTGTGTGTGTCTCCGTCTCTCGATTTATCAATCGGTGTA

TGCTACTCCCTCGTGCTTGTACATCTCTCCCCCTATCCTTCTTACTCGAG

GTATTTGATGATGGCGTTTTTTTTTGTTAAAAATCGCAACCGTCCTCTCT

TTTTACCAAAAAAAAAAAAAAAAAAAAAAAAAAAA

>TSAL.R79.esd 775 0 775 ESD GOOD: 109-472

GCACGAGGGCGTTCGTGGTAACTCGTCGCAAGACAATTAAAGTCAAGATG

TCATTTGAGAGTCATTTCATTCTCATTTCATTCTCAGTTGATTGAAGAAG

TACAAATAAACTCATTCGCCTAAGGCGTAGATTTGCTTTGGTACAAAATC

CTTGACTGTGACATCATTTTTGTCTAGCAAAAGGCGAAAAGACGAAAGAG

GAGGCATGAGTTGAGGTGAATTTTGGGGAGGAATGAGTCTGGTAGGCTTT

GAGGAATGCAAGCAGAGCGACAAAGAAGACGATCTGCAATCAACGTCCGA

TTTCCACATACCCTCTCTCGCAACAGTTTGGTCGTGTAAAACTTTCCTGA

CTACTTGAAAATAG

>TSBM.R24.esd 730 0 730 ESD GOOD: 99-623

GCACGAGGCTCCTCAATTGGGTTTGAGGTGGCAATGCAACATCATTTCCT

TTTAACTGCTTTAATTAACTTGGCCTCAAAAGTGCGAAACACATTTACGT

AAGCATTGGCCTCCTGGACGTGTTCCAATCTGTTCTTTTACCTATTGCTC

TTCATTTAAGATTTGGGGGCTTTGAAGAATTCAAGCGGCACAGCATTTCC

CCTGATGGCACTCTGACTCCGGGAAGAAAATTTCTCTGTGGATTGGGTGA

TTTCTCAAAAGCATATCTCACTTTCCTAAAAATAGCCTCCATACTGATTC

ATGAGGCCCACCTTTCTCCACACCATTCTTCCAATTCATTTCTTAAGAAA

CTAAAATGACGAAGGTGGAATATCGCAAATAAAGGCTTAGATGCTCACAA

ATACAAAAACATGTTCGACTGTGCCCTCAAGATTCTCCGAGATGAGGGTT

CCCTTGCGTTTTATAAGGGGACAGTTCCACGACTGGGGCGAGTTGCCTTG

ACGTCTGCATCTCATTCATGATCTA

>TSBO.R7.esd 664 0 664 ESD GOOD: 104-618

GCACGAGGGTCGTGGACTCCGCCACTGGCCGTATCGTTATTGAAAGTCAG

AAGCTAACAAAGTGGCTCATGACTGGGCATTTCATCGCAGGGTTGTCTCT

GCTTTTCACCTTGTTGGGATTTATTTATGGCAGTTACGACAGCCGTTGGG

TTCCTTATCCGAGATACAACAAGCTCTCTTGGGGGTTTGGCACTGGGGTA

CTCAGTTTGATTCTGATTGTTATCAGTTTCATTGCTATGCTCGTGTTTTG

CCTCAAAGTACACGTGAAAACTCTGAAATCACGGCTTGTTGGACAAGAAA

ATCGGGAAGACGATGATGACATGGCGCAGGATGATGACGACAGAAATGTA

GAGGATGAAATACGCAAACTGGCCTCGACATCCTATTCTCGCAACTACTC

ACAGCCTAGTGGATATTTGCATCCAAAAAGCGCAGGTTTCGTCATCCCAC

TGGAATCTGAAATTGTTGAGGATCTTGGTGACATTGACGATGGTGATAAT

GGCATGTACTCCCAT

>TSBT.R43.esd 731 0 731 ESD GOOD: 99-535

GCACGAGGTGAATTGAGGCCTCGTGATGCCACATTGCGCAAGCAAATCAT

CATTACCCAATGGAACTTGACACAGAAATTCATTGGGACCTATTGCGAAC

ACCTAAACAGTCAGTTCCTCTCCATGTTAACACTACAGCCGCATTCGCTA

ATCACTTTGTTAGCGAGGAACAGGTGCTGCTTTACGTGAATGCAGTCTAG

CCGCTTATAGGTCCTTGAGGCGAGTATCACTGTATTACTTGATGGCTATT

CATCAGAATGCGATAGAGATGGAAAAAGCAGAATTGTTGGATATGAATAC

TGGATACAAGAGCAGCAGGCGCCGATTTGAAGTAGGTGTCGAGAAGTGTT

TTAGTTAACAGCAAAAATGGAAAAGGAATGAGATTCCGTTGAGATGATCA

GGTACAAAGTTTTGCCAGTACAGACAAGGTCAAATTT

>TSBZ.R72.esd 574 0 574 ESD GOOD: 97-518

GCACGAGGGTCGTTTGGTCCCCCATCATCCATCTCGATCGCGAAAGGACA

ATGATGTGCATTGTGCAATCAGTCTGACAATCAATTGATCGATTGAAGGA

GAGATGAAAGGTCGTCCACATTTCCTTTCCTTCTCTTCTCCCCTTCTCGT

TTCGTCATCCTTCCTGCCTCTCTCTGTCTCCTCCTCCTGTCTCTAATCTG

TCAGCTTTATGATTTCCTTTGTTTTTTTCCTTACTCCATCTTTCTCTTCT

TCCACCTTGCTTTTTCCCCTTTTTTTGGACCACTAATTCACCCTATTAAC

TTGCCTCCTTTGCTCCAGTTTCTCATGGAATCCTTTGGCCTCCCATCTCC

ATTGCACACTGTCTATTAAAAATGGAATGCAAATAAATGCTAATTATTGC

TTAAAAAAAAAAAAAAAAAAAA

>TSBG.R59.esd 554 0 554 ESD GOOD: 105-333

GCACGAGGGTTGTTAAAAATGCAGTTACTGAGGCTGATACTGCCAAGGAT

GATGACGACGATGACATTGTATTTTCTTTAACAACTGTTATCAGTCTTTG

TGGATCGTCCGTCACGCGTTCATCTGTTGTCTCTGAGCTACGTGATTTTA

TAAGTTCCTGCATTTCCCGTGCTGAACAATCTAGTCCAGACGCTAAGACG

TTGCTTAGAGTTTTAATGGGCCAGGCTTC

>TSCC.R34.esd 695 0 695 ESD GOOD: 95-437

GCACGAGGGCCAGTTTTGCTGCGCTATTGCTGGCGTGTCATGTTTGAGGG

GGAGGAAGGAATCTCGTGCCTCCTGAGGCTGCGTCTTATTGTTCTCTCCC

TCTTCGTCTTCTTCTACGTCATCTCACCACTGGATATCTTTCCAGAGTCC

ATGGTGGGTGTCTTTGGTCTCCTAGATGACTGTCTCGTCTGTCTTGTCTT

CTTCATCTACGTGGCTGCCCTGTTCAGAGGCCATCTGATTGCTGATGTTT

GACCTATACTGATCTGTCTGGCTTATTCCATACCTGTTCAGGCGCTCTTT

ATTATCATTATAATTATTATTACCTAAAAAAAAAAAAAAAAAA

>TSCB.R22.esd 744 0 744 ESD GOOD: 114-637

GCACGAGGCCTCGTGCTTGTGAAAGTTGTTATAAATCAAATAAGTGGTTG

TGAAATTTGCACTCTGAAAAGGACGTCTTATCTTTAAATAAGTGGTAGCG

AATCGCTACGGAATAGAGATAACACGAGGAGTGGTTAGAAATGGCTAAAG

TTCTGGTGCTTTATTATTCCATGTACGGACATATTGAAACGATGGCACGC

GCAGTCGCTGAGGGTGCAAGCAAAGTGGATGGCGCTGAAGTTGTCGTTAA

GCGTGTACCGGAAACCATGCCGCCGCAATTATTTGAAAAAGCAGGCGGTA

AAACGCAAACTGCACCGGTTGCAACCCCGCAAGAACTGGCCGATTACGAC

GCCATTATTTTTGGTACACCTACCCGCTTTGGCAACATGTCCGGTCAAAT

GCGTACCTTCCTCGACCAGACGGGCGGCCTGTGGGCTTCCGGCGCACTAT

ACGGAAAACTGGCGAGCGTCTTTAGTTCCACCGGTACTGGCGGCGGTCAG

GAACAAACCATCACATCCACCTGG

>TSAA.R24.esd 713 0 713 ESD GOOD: 103-379

GCACGAGGCTCAAAGGTGCCAGCTGGTCAAAAAGCCCCGGGTGATGTTGA

AGTACAAGTTGATTACTGGGAGTGCATTGGAAAAGCTCCTGCAGGTGGTG

TTGAAGCTGTTGTTACGGACGAGTCTGAAATCGATTGGCAGTTGGATCAG

CGCCATCTCATGCTTCGTAGCAGTAAGGGATACACAATCGTCAAGCTGGT

CGCTGTAATTGCGGAGGCATACAGGGAACACTATAAAGATTGTGGATTTA

CCGAAGTCCATCCCCCAACGCTGGTGC

>TSBC.R84.esd 774 0 774 ESD GOOD: 99-682

GCACGAGGCTGGACCATGACAGAAAAGGGTCGTAAGATTGGAATTCCTGT

AGATGGCAGCGACAACTCCAAGAAGGCCGTTCACTGGTACTTGGAGAATA

TGGCAGACAAAGATGACTATGTGTTTTTCATCCACGTGCAGGAGTTTCTC

GATCTTCCACTTTTGCATTTCAAATCTGGACTCAGCATTCCCTCGGACCA

GTGGATGAAGGCTATTAATGAAAGGAATCGATTAGACGAGGAGATAAGCA

CGGAGGTGGTGGGGATGTGTCGAGCGAAAAAGATTGCCTGCGACTACATT

ACGGCGTCGGATAAACGTCCAGGCGACGGAATTGTCTCAATGGTGAATGA

CCTGGGCATTCAACTAATCGTCATGGGCTCTCGGGGCCTAAGCACCATTC

GACGCACCATCCTTGGAAGTGTCTCTGACTACGTTTTGCATCACTCTCAA

GTTCCTATCTGTATCGTTCCCGCCGAATATACACCCGCGTCTTCCACTTG

TCCCTAAACTAACCAAATAACGAGTCTGAAACAGACGTTCCGTTCTTAGG

CATGATTCCTGTGTCACATGCAAGTACTTACTTT

>TSAB.R3.esd 722 0 722 ESD GOOD: 99-529

GCACGAGGGCGAGTGTTAGATAAACGGAAGCGGCATCCTAGCTGGAAAAT

TGATGATGGCAGCACTTCAATTTTCATTCCACATCTAAGGTACCTCAAAG

GCAATGGTTACCTCCAAACAAAGTTGTGGACACTTTAAAAGCGTAAGGAA

ATAAATTCTAGTGAGATGTACACTTATTTATCATTCTCCCAATTTTTAAA

TCAAGGTAGGAATTGGAAAAAACTCTCCCAACTGCTTTTCACCAAGCAAA

ATTAACGAGACCTTGTAGATGTGTCACACTTAAACGAAAAAATGACCATT

ACTTCTCGATCTAATGAATAGATCAAATCAGCCATTTGATAATGACATCT

TAGTGCGCGAATTCGCCACAACTACTTCAACCCTGCTGTCCACTCAAGTA

CACACGGCTAATGGTACATATGTGAACTGTT

>TSBJ.R49.esd 595 0 595 ESD GOOD: 96-362

ACGAGGGAACGATACACCGCCACAANNAGCGAAAGGCAACCCNCATAGAA

CGCGGAACAACGGAAGATTTCGGAACGATCTTTTCCCACTTTACTTATTC

GCTTAACATTGTCGAAATCCCCAAAAGCATCTTTTTGGCTTTGTTTAGCT

TCAGACACCTTAAAATAATCATCCGAAGTTGCCATCACGTCCTTTTTTTC

ATTTCCTTCGACATGTACAAGTCATCAATATCAAAACCTACGCAAGTGTA

AAAAAAAAAAAAAAAAA

>TSBN.R15.esd 469 0 469 ESD GOOD: 77-306

CGGCACGAGGCTTAGATCTCCCACAAGACGTGACTTTAAGGAGGTCTTTC

CGTGGGCGTCGGACGTGATGTTGGACCTCCTCTCCAAGATGCTTGTCCTT

GATCCAGACCGCCGATTAAGAGCATCAGAAGCCCTGGCACATCCCTTCTT

TGCTGAATATCACGAAAAAGCTTGACGAGCAAGAGGGCACAGCATTGAAA

GATGAACTTATTTCATCCGGGCTCATTAAC

>TSBA.R28.esd 619 0 619 ESD GOOD: 105-359

GCACGAGGTGAAGATGGGTGCTGCGATCTTGCTACTTGCAAGTGCTGTGA

AGGCTGCAAGAGCAGGAAGGGCTGCTCATGTGAGCCCGGCAAATGTGCCT

GCGTCCAATGCCCTTGCGCGAGCTGCAAGAAGTGCTGTTCGGAGTCGAAG

TAAACACTTTTTGGCGTTTATATTGATATTCTATTCGGTTCAATAAAGAT

TCATTAATTTGAAAAAAAAAAAAAAAAAAAAAAAAAAAAAAAAAAAAAAA

AAAAA

>TSAS.R20.esd 551 0 551 ESD GOOD: 91-531

GCACGAGGAATTCTTCCAATGAAACCGAGTTAAAATCAACTGAGGACGAA

AGAAACGTTACTGGAGGAGTGTTCTACACGCCAGCCAGTGCTCCCGCCTC

ATCAGGAGGAGCGGGAGGTGGCACCTTTTATAACGCCTCCATGGAAAACC

AGTACCAAAATGCAGCGGCTTTTGTTCATCAGCACTTCCCAGAGGAAGTA

CAACAGCCCCTGAAGACAGAATGCCAGTCTCCGCACTATGAGGGTCCGAA

CCCGCTCTACGGGATCCTCTCATCAGTTGGTACTGCTGCTTCGGTCAACA

CAGGAGTCGCGATGTGGTCAGAGAATCCGGAAGCTCAGCGATCGAGCTAA

CCCGGCTCCTGCGAAGTGAACGTAGAAGTACCACAAATGTTTGACAGCAG

ACGATTTAGTATGCCATGGAAGCCCTGGGAGCCGAGTTCGG

>TSBY.R79.esd 760 0 760 ESD GOOD: 106-641

GCACGAGGGTCGACGTGACTCAATGCAAAAGGAGGATAAGCATGATCGCC

AAGCTTCCGCAAGTAATTCTCGGGATGTTAAAGCATCGAAAGAACACAGC

GCAAGTCGCGTTAGCCTTGAGAATGGTCAAAAAGCCAAGAAGTCTAAGGA

GGACGATGAAAAGATGAAAGACTCGGAGTCGTCACCAGTCTCGAAAGAAC

TAACCAACGGGGATTCTGCAGGGAAAGAGAAATCACCCATTTCAAAGAAA

CCACTAGGCGTTAAGCTAGCCGGACCTGAGGATGACATTGAAGGTCAAGG

ATGGTGTAAGGCGCATAGCCCTCTAGCCCCTTCCGATTGCGAAGGTGCTT

CCAGTTAAGGGAGTCGTTTAGCACCTCCACGTATTCGCCGGCCTTGAGGG

AGATCTCCTGATCACTTTCTGCGACGAAATCACGCATCACTCGGGCAACT

CTGAAGGACAATGCGAACCAGGACCAGTGAATCTCTTACCCAGTTTCTAA

GCAGAAGCTCCCGAAGTAGGAGTATCACAGGTGTGA

>TSAK.R79.esd 713 0 713 ESD GOOD: 93-584

GCACGAGGCAGCTTTGGTGTTCCTGTGGTTGCTCTTGTAAATACCTACCT

CTTTTCCAGCAAGAGTATTTAGTTCAGGCAGCTTTCATCCACCATGACTA

GCCTTATGGGTTCATCGAAATTTCCACTATTACCCGATCAAAATCCTCAG

GACAAAGTCCTCTTCATGGTTGAAAGATCTTCTCTACTTATGAAAATGCA

GAAAGACGAGGTGTTTTGTAATTGCCTAATTGATGTAGATGGTACGCTTT

TTAACGTCCATTTGGAAATCTTGTGTGCCTCCTCGGAATACTTCGACAAC

ATCATTAGGCGCGACTCTGTCATTGACGATGTGGTTTCCCTTCACAACAT

CACTCCTTCCACATTCTCAACTTTGCTTGACTACCTTTATACCGGACGCA

TGGAAGTAAACCTGTCCAACGTCATTGATGTCTATGTTGCCGCGGACTTC

CTCATTTTGAATTCTGCTCTTGATTATTGCCGAAAAGTCCTC

>TSBY.R62.esd 774 0 774 ESD GOOD: 111-574

GCACGAGGCTCTGACGCAAGTGGGGGCTTTTTTAAAAACGCTTCTGCCAC

TAATGCGAAGCCTAGAAAGTTCTGCAGAATCGAGGTGTAATGCCTTGTAC

TTGCGCAGAAATTGTCTCCGCTACTCAAGACGGCGACAAATTTGTGTCTT

CCACGGGTGTGCACTTCTCACAGGTCACCTTGGTCGGTTTGATTCGATCC

GTTAACGAATCCGCAACACGTATCGACTATGAGATTGATGACTACACTGG

ACCCTCTGTATTAGTAAAACAATTTGTCGATGATGATGCAGGGCCAGGCA

GCTATCGCACACTTCGAGAGCTTACCTACATTCGTGTCTACGGTCATGTA

CGTAGTTTCCAGGGCGACATTAATGTTGTTGCGTTCAAGGTTTTTCCCGT

AATTGACATGAATGAAATAACATGTCACATTATGGAGACCATCTACGCTC

GAATGTTACACGCC

>TSBU.R34.esd 869 0 869 ESD GOOD: 95-522

GCACGAGGGGGGTGTCCACTTATCTACATTTCTAAGGACAGTTTCCATGT

GTAAGGTCCATTTAAAAGGACTCCAGATTGCACATTTCTTGGCATGAACA

AGGAAATACTAAAGCACTTGTAGAGGGATGAAGGGGTGAAGCAGCACCAT

AGATAACGAATGTAGTTGGAGAGAGGACAGTTGGTTTCTAGCATAGCTGA

ATTCATCAGATGAATGTCCATCGTCGGTAGAGCGTTATTTTCACTCCTCT

CACGACGTTTGCATAGATTATGTGAACCGCTGTTTACCATCGCCGCGTAT

AGATTCACAACATTATTATTGCTGAGGTTCGAACTGCCGTCCATTCTGCT

AGCAATGAGCAGTTCGGCAGATGTAAACACTAAGTCACTCACGTCGGGTT

TTCTCCTATCCGAACTCTTTGGCCTCTT

>TSBG.R15.esd 637 0 637 ESD GOOD: 104-395

GCACGAGGGCAGCTCGATGCCTGTGAACAGTGCTTACCTTAAGTCAATTT

CCGGCCTTATCAAACTGTGCATATGCATAGTCTTGTTCATCACCTTAGTT

TGTGCGTGCGTTGGCTGCTCGTCCTACCGGATTTTCTTCCTTCTGGTTTC

CATTCTCGGCTTTATCATTGAGTTGGCGTTCTATTTGTGCTACCTCCTTA

GTCTCACAGGAAGACTTACCCTGAATTGGCCCTTTGCTGATTTCATCATG

TCGGCGGTGATGATAGCGCTTTCCTTTATCAACTTCTGTGTC

>TSBT.R66.esd 776 0 776 ESD GOOD: 97-502

GCACGAGGAGGGGCCGGGATTGTCAGCTTGCCACAACAGACACGATGGAC

AAGCGCGGGTTTGATACTCTGGAGGTCAGACACCCTTTCCCCCATGCGTC

TGGTCCCCGCTCTGAACGGGGACGACGAGGGAGCAGGTGTGAGCTACCCG

TCGGGATCGAGCATCATCAGTGAAGAGAGCTGTGTCCTCGGCTTTGGATG

GTGTAGGCCTGATCTTCCCCCCGCCGCCGTCTTCAATGAGATCACCTTCG

ATTTGGAGAATAAGGAAAATCTACGGGATATGCGTATGCTTCTTGTTGAT

AACGGTTCTAATCTACCCCGGTGTTCAACCAGCCAATCTTGTCTCCTGGT

CCCGTGATCGCCAGTTGGAGGGTTCACAATCTGCCGGCTGCTTTCTTGCA

CGTCGA

>TSBT.R26.esd 593 0 593 ESD GOOD: 18-570

AAGGAACAAAGCGAGCTCAGCGGGCGGCCGCTCTAACATGTCCCGGGCTG

CAGAATCGGCACGAGCATATCGCTTTCGTAAGAAGTAAGTCTCCTGTATC

ATCGCCTTGTCCGAACAGGACCTGCAGGGCCTGACTGTATCGGTTGGATC

TACTGAAATCAAGCCTGAAATTTTTTGCAAATTCCAACATACGCTTTAAT

CGACANAAATGTGGAATGGGCTATTCCNCCCCATCGGCGTCTGTGCCGAG

GGCAGATTTGTCACGTCATAATAAGAAACACATTCCCAATGCTCCTATCT

CAGCTTTTAGATCAAAGTACAAGATACACTACAAAAAGACAATCCCAATG

CAAAAAGGTCGGGGCAGCAGTTGTTCATTTAAAGTTAAACAAAGTCACAA

TACATTTCGACAGAGGTCGGAAAACCCGGCTGCCAGAACCATTTCTCCCT

ATAAGTGAGGATTTGTATAAATTATTTGTATAGGTTGAGGGTAAAGAAGG

CTATCGCGATGTTGCGCTACGAAAAAGCTATTTGTTCACATTACGTGAAA

ACG

>TSAU.R63.esd 551 0 551 ESD GOOD: 97-485

GCACGAGGGTCTGCTGCAGGCTAGGCGTGACCTTGAGAAGGCTCAGGACG

ATCTCCTTGCCTACGTGAAAGTAAGACCAGGTTGCGGCGCAGTGAAGACT

GATCTGGCCACCTTTGTGTCATTACGAACAGTACATAAGTTGAATTTGGG

GCAATCGCCAGATGCGCGTCGTCTTCTGGCACGCATCACAGTTCCCACGC

AGCGCCGGCTCTGCCGCAAGCTTTGGTGATATCGCGGAAATAAATCCGCG

CTATCCACGTCTCCTCCAATCAGCTGCGCGCCCTCATCTCTGCTTTACTA

ACTACACCAAACTAATGACTCCTTGTCACATCTTTTTTTTCTCCCTTTTG

CGGTGGAATTGGCTTACCCAAATCCGTAAAAAAAAAAAA

>TSCH.R42.esd 597 0 597 ESD GOOD: 100-549

GCACGAGGACGATGTTGACGAGGGAAAGAAAATGGTAAGGGCAGAATACG

ATAAGGGGAGCGAATGGTAGAAAACAACCGGTATCAGATTCTAACATCTG

CGCAGAGCTTGTGATTTGATTTGTGTCATTTCGCGATGCAATCCTACTTT

CGCCATTTCACTAATTCTTCCATCCAACCATCTATGCAAGCATTGCATCA

TCTGCATTTGCTCAACCGCTATTAAAACTAAGAATATTGTTTAATCCAAA

AAAGTATTAATTTGTAGCTGGAATGCTTGCTTATGGCACACAGGCCCAAT

TAAGAGCGTTGTCATTTGATTTAGTGGTTTGGCTGATGCGTTAAANATGA

AGGAGAAGAGAAAAGGGCAAATAATTTGTTAAAGGATTAAAGGAAGGAAA

GTCATGATTCATCTTCAATTTTTTACAGATGCGGGTTGAGCTTTGCTTCG

>TSBA.R93.esd 511 0 511 ESD GOOD: 96-511

GCACGAGGGGAGAGCAGTGCGTTTTGAGGGGAAGAAGAAAGCGCATACAC

ACACACAGCTAATTGACCGAACAACAGATGTGCGTACTTCCTGGTGGGCT

TTTCCTCGCGCTTCAATTTCTCATTCTCGCCGTATCGTCGCAAAAACAAT

CCCCATGGGCGTGCCTGATGGAAGGGGAGCCGTGTGATCAGGAGGTGGGA

CAGTGCTGTGCAGGTCTCTCCTGTCATCCCACCCTGGGGGTGTGTGCGCG

ATGCGCCGATCTCTACCAGTTCTGCTCACGCAGCAAACCCTGCTGTGAGG

GCACATGTCGCATTGTCTGCACCTGAGAGAGCCACCTCTTCGTCTCTCTT

GCCTCACTTCCACTTTTCCTCTCTTCCTTCTTCCCTCTCACCTTCCACTA

ATTGATCGTGGGNNNN

>TSBW.R94.esd 590 0 590 ESD GOOD: 95-590

GCACGAGGCTCGAGTTTTTTTTTTTTTTTTTTACAAATCCCATCAGCAGT

GCTCTATTTACACCAAAGAGAGGCGGTTCAGGCAAGGACGTCGCGGAAGT

CAGGTCGGGGCTCCATGTCGAGAGTCTGAAGTTCGCGCAGGCCCTGCAGA

CACACCTTGATGCGCGCACGGATCTCAAAAGCCTTCTGGCGAAAGAGCTC

TGCCCTTGCGGCGAGTAGTTCCACGCGCTGACGCACGAAGTCCAAGGCTT

CGGCGCGCGTTAACTCCACGTGGAAACCCAAACCTACGTCCACGTAGAGG

CGCTCCAGCGAGGAAATGTGGGCCTTGAGGTAGAAATTACAGCCCATGTC

AACCATGGTTTCGGTTACACCACCATTGTCGTCATCCCTAAAGCCTACCT

CGGAAAACTTTTCCAAGAGGTTCTGCAGCTCAAGATACTCAGATATCTCC

GCATAGATCGTGTCACCCTCATCAAGAGCAAGCTTGAGATCCTNNN

>TSAM.R75.esd 539 0 539 ESD GOOD: 86-311

CGGCACGAGGGTTGAGGCAGCATCAATGTCGACTCTTTGCAATCTTACTC

ACATAGTTAAGCACTAATGTGGGTGTAAGACAAAGGCATACTGACTTGCT

CATGCTCCAAGGACTCCGCTTAGAGCAACGCGGAGTCTGATTTGTGAGCC

ACATTTTCCCTACTCACGTCGGCTGGTGCGCCTATCCTGTTCATGCTTCA

TGTTTTAGGTAATCTGATCTAAAAAA

>TSBA.R29.esd 633 0 633 ESD GOOD: 102-375

GCACGAGGCATGGTCCAGATCCGGCTTCTGAAACTGTTGGTCTAGAGGGT

GATATCCAAGTCCCATCATCACAAAAGTCTAACAGGGGAACTATCGATGA

ATCTATTAAGGAAACTATCCTGAGAAGATGTTAGATCAGTCTGTCGAAAA

GTTGCACATGTTATGTTTCCTCGNACGGGGTCATGAACTTCTGAATGATT

GTAAGTCTTGATAAGTCCGTAACGGCTGCACTTTCCTAGGGNACTTGGGC

GGTTCCATTCTTCATCTTTATTGT

>TSAI.R7.esd 608 0 608 ESD GOOD: 101-561

GCACGAGGCAATTTCAAAAAAACCGTTGCCATGCACCAGGTCTTTCTTCA

ACGAATAGCTAATCATCCAGTTCTACGGAACGACTATGGTTTTCGGGTGT

TTTTGGAGTACGAGCAGGAACTCAGCGTGCGCACTAAAAATGCCAAGGAA

AAGGCGGCAGGCTTTCTCAAGTCGGTTACAAAAACGGCGGACGAAAACTT

GCTGTTATCTAACCAGCGAGAAGACGATCCCTTCTTCAGGGAGGAAAAGT

CCTATCTTGTTCGTTATTACAGTGCTGTGCATGACGCTCATCAGTCCACC

GACGCGGTGTGCCGGAACCGCCGGGTTGTGGGTGAGAGTGTCCTCCGTTT

GGAACTTCTGCTGCAGGACCTTGCTACCACTCCACCCATCAACCTCAAGT

CTGCTGAGTCTATGCTCACATCAAAATTATGCAATTTCTTCAGTGCACTC

TATCCGCTGCA

>TSAJ.R28.esd 612 0 612 ESD GOOD: 96-586

GCACGAGGCTACGTGGATCATTTAACAAAACGGACACAGTGGGAGAAGCC

ACAGCCTCTTCCAAGCGGATGGGAACGGCGAGTAGACTCGAACAATCGGG

TTTACTACATAGATCACAACACGCGCACAACGACCTGGCAGCCACCGTCG

GACCATTTACTCGACAACGTGGTGCGCTGGCGTCAGTGGTACGACATGAG

AGCAGGTAACATGCGGAACCATATGTCGCAGCTCTACGCCTCCTCTGGCT

GGGCTAATGGCGCCGGCAGCAGCCCAGCCCATTCCATCCCTGAGACATTG

GGTCCGTTACCCGAAAGGTTTTGAGCGCGTCGTAGAGACAGAAATGGTTC

GTGTGTAACTATGTGAACCATCGGACCAAAGACGACACAGTTGGGAGGAT

CCACGCCAAACCACAGTGCCGCTGCCATCCGGGCTGGGAGATGCGATACA

CTCCCTGAGGTTTGTCTTCTACGTCGACCACAATACCAGAA

>TSBS.R30.esd 554 0 554 ESD GOOD: 92-405

GCACGAGGCTACCTTATACTTTCGCCATACGATAGTGAGCAGAACGACTT

AATGCACCGTAGGATGAAACTTCGTGATCTTGAGGCTCTTCCTGAGTACC

TAGACATGCTTAAAGCATTCACCACACAGGAGCTTCTTAATTGGAAAAAT

TTCCGTGAGCGCCATGAAAAAACTCTACGATGTGAGAACTTGGCCTTCTC

TAGTTCTACTGACCGGAAAAGACCAGAAAAATCCTTCAAATCTTTGCAGG

AGCGTGGTCACTGAACACAACANTTCGGGTAATATCCGCATATTACACCC

AGATACGTCTCGCT

>TSBN.R92.esd 477 0 477 ESD GOOD: 90-439

GCACGAGGGTGGTTCTTGGCTAGTCCCCACTTGTCTTTCATGCAAAATGC

TCCTCCCTCTTTTTGGTCACGCGTATGCTAGATGGCTGAGATTTACACCA

TGGAAGCCAACAGGAACAGTAGTGAAGCTTCAACTTGCTCGAATTCAAGA

AGGGGTAGCTTGCCCGTCTAGTGCCTCAGATTCTCCTGGCATACTGCTGG

CTGATGTAATGCACTGGTTTGCTGTGCTCGATTTGAGTCCCTCTGACGTG

TGTGCTGAAAGTTGTACTGCGTCCACTCAACTGACTGGGCTGCTCTTACT

CCTCAATTTGAGCCTAAAGCTCCTTCTTTCAGCTGATTTGAAGGCGACCA

>TSAB.R56.esd 762 0 762 ESD GOOD: 101-624

GCACGAGGAGATCACCTACGACTACAAGTTTCCCTACGAGGATGAAAAGA

TACCTTGCCTATGCGGTGCCCCACAGTGCCGTGGAACTCTCAATTAAATG

GTCTCAGGATTATGTCTCACCGTCTCCATCCCTTCTCCTTGCTCTTTCCC

TCCTTAGCCTTTCATTCACAGCAATTTCTCTCCGAAGTTGGCTTTTTTTC

CCATTTGGCAGTTCTCAGCGCATGCGCTGATGCAGCTCAGTTTCACTCTC

TCCCTCTCTCCTCAGTCTTTCCCTGAGCCCTCCCTTCAGGCGGCTCCTTG

TCTGGTCACTTCGGGCTGCAGCAGTGGTAGCATTGTTGTGTATAATAGCT

CCCATTACCTACCTTCACCTCCTGTGCGCCTTTTGTCAGTTTTGTACAAA

TGTATGAACCCTACGTGGTTTCTCCCTCTCTCTATCCCCTCCTCACCCCG

CTGACCCTTTTACACCAATGCTAGTTTTGTGCATTTAATTTGCTTCGTTG

TACAGAGAATTTGCCTGTGCCTGT

>TSBB.R42.esd 618 0 618 ESD GOOD: 109-548

GCACGAGGGTCAACAGTACAGTACAGAATCAATTTGTCCTGGCATTGGTC

CAACCAAAATACGTCCTTGGATTGGCCAATGGCATCCTGCTGTCAGCATA

AAGTCCTCCCGAATTTACACCTTCCTGCTTGCTAAGCGGGAAACCCCCGT

CCTGCCCAAGGCTTTATGGTCTTTCAGATGGTATTTGGTGGCTCGAGGGC

GTCCCTGTCTTGTCAATGTATCGTCTTGTCCTAACCTCCCCCCTGTGCTT

GCGTGTAAAGTACATAAAAGCCTTATTCTCCGCCACAAACACGAATCCTT

TTTTCTGGTAATTTAAGTCCTTCCTTCCCCGTCCTCCTTGGTTTTGCATT

TAACCTTTTTTTTCCACAAAATTCATTTGATATGTTTTGGGATCGAATCG

TGGGGTGTGGTATGAGCTTTCCACTAACTCTATCATCCCT

>TSAQ.R17.esd 602 0 602 ESD GOOD: 87-408

GCACGAGGCCGAGATGCCAATAAGAAACGCATTCTCCGTCAAATGGCCAT

TGCCGATCGCAACATAACGGACGAGGAGCTAGAAGACATGTTAGAGTCTG

GAAATCCACAGATTTTCACGCAAAGTATCTTGGCAGACACGCAATTGGCC

AGACAGACACTGTCTTGAGATCGAAGCGAGGCATCAGGACTCTCATTAAT

TTGGGAACAGAAGTATCAAGGGAGTTGCATGANCATGTTCCAGCGATCTA

AGCAACTCTTGTGGATTCACAGCAAGAAACAATTGCAAGATCGAGTATAA

TGGACCCAAGCGAAAGACTACA

>TSAJ.R54.esd 663 0 663 ESD GOOD: 102-510

GCACGAGGCACGTACTTTACTTTAGTGATTGTAGATGCTCTATGAGGAAA

CTGTAATTTATCTACCAAGTGACAGTCAAGACATTCTGACTTTTTTTAAT

GCATAGAGGCTTAATTTTAAGCACAAAACCAACTAGTATTGGCAAGTTTT

CAGTAACCTCAGTGGGCAAATTTTCCTAATGCTTGATGTAGTAAATTCCC

CACTTTTTTTAACAAGCTGTGTAAGCGGGATACTTGTTTAAAGCTGATTT

CCCGGTTTGGTGGTCACTGAATTCACCAATAAGCTAGATTCGAAATAATC

ACTCATAGTACACGCTGAAAAGGCAGAGCCTTTTTAATTCGGCACAAATT

GGACCAACGTGGACAGAGACTATTACCCCGTAGATATGCTTTGTCAGAGT

CACAAAATA

>TSBN.R28.esd 596 0 596 ESD GOOD: 88-294

GCACGAGGCGGGACTCCAAATGCTGCCAGCCGTGGCGCTGCAGCAATGCG

TGGTCGCGGGCGGGGCCGCGGTGGCTTTGCGAATCAAGCTGCTTATGGAA

CTCCAGCAAACTTTGCCACCCCTCCAAGTGGAGCGATGCGTGGGGCTCGT

GGTGGAGCCACTAGGGGTCGCGGTTGGGGCGTGAGCAGCACGGCTCCTGC

TGCCGGC

>TSAZ.R78.esd 629 0 629 ESD GOOD: 97-210

GCACGAGGGGCAATCATTTAATTGCAATCGCGTTCGATGTCACCAGCCGA

AACGCAGTGACACGATACCTGGAATGCAAAGGAGCTGACGAAGTATTTGG

ATGTTTTTAAATGA

>TSBV.R89.esd 688 0 688 ESD GOOD: 99-292

GCACGAGGGTAATACATGTACAGTCCTACTAAGAACCTACCCACTGCTGT

ACCCTTTCTAGTAAACCTCGAAATCTCGTGATCATTCCAGCTGTAGAACA

CTTGCCGCGATCTGATTCAGTTGCTGCGCTCACTTTTAAATTAGCATCTA

AATATACTTTTCATCCATGAACTTGGTACTTTGCTTACTGTTTC

>TSBU.R84.esd 753 0 753 ESD GOOD: 105-442

GCACGAGGATCACTTTCGCCTCTGTTCAAGATGCTGAGTTTTTCCTAACT

CGAAGCACAGAGCTTCCGTTGAGACAGCCTCGACAGCAACCCGAAGTTGT

TTCTGCGGGTCGGCCCAGTCCCTCAGGTCTTACTGCGCCGCCAATATACC

TCACAGTGGAGGAGTTGAATAAACTGGACTCTAAGCTAACAAATCCCTTG

GGAAGCTGTACAAGCCCAGTGTTAGCTATTTTATTAATTTGTCATTATCT

GGGGCTAAGATCTGGAGGTGATGAAAAATGGACGAGCTGTTACTCTATTC

CGAACATGGCTTTATTCGGCTTTTTCTGGGGCTGGCAC

>TSCC.R3.esd 403 0 403 ESD GOOD: 93-403

GCACGAGGGGTCGTTTTGTGTGTGCTTGGCTGCGTTACGATCGTGAGGCG

TCAGCCCATTGTCTCTTTTACGAAACTTGCCCTTGAATGTTGGATGTTGT

TGCTAGCGTAATTGACTATGGTTGCGTCCTTATCGATAAACCCTGTACAG

GTGTTTCCGCCGCCCTCCAATGTCATGTGCCTCTGTTTCACCGAGCTTGC

AATGAATGCTCCTGTGAGTTGCGCTATCCTTGTCGTCAAAGGTCTCCATC

TCAAGCATAAGATTATCAAGCTTTGAAACAAATCAACGTGAGCTCACAAC

CTTGAAAAGGG

>TSBV.R3.esd 539 0 539 ESD GOOD: 103-484

GCACGAGGGCCTACAGCACCAATCGTGGCTCCCTTCGGGTCTGCGACACC

CGTAGTACCGCACTTTGTGATAAGCCTGCACTGGCAATCGACAACCTCCC

ATCTTCCGAGCAAAATGTCATTGTTATCTTACTCAATTCCATCGCCGACT

TGAAGTTTCTCAATAATTCAGATAGATTTATTGTTTCTCGGGACTTTATG

TCCGTCAAAGTTTGGGATCTGGCAAATACAACGGCTCCTGCTGAAGTCTA

TCCAGTACAATCGCATCTGCGACCATATTTGAACAGTATCTACGAGACGG

AATTGCTCTTCGATGATTTCAAACTCTCCCTTTCCTCAGACGATAGGTTT

ATAATTAACTGGATCCTATCAGTCATATATAC

>TSAO.R32.esd 632 0 632 ESD GOOD: 104-632

GCACGAGGGTGGACCAACTTCAGGAGAAGATCAAGACTTACAAACGACAG

GTGGAGGAAGCGGAGGAGATTGCTGCGGTGAACTTGGCGAAGTATCGCAA

GATCCAACACGAGGTGGAGGACGCTGAGGAGCGTGCCGATCAGGCGGAGC

AGGCACTACAGAAGTTGCGTGCTAAAAATCGCTCCTCGATTTCCATGTCC

CGTGGACCCAGCGCCACTCCGGCTGTGGCTGGTCGCACTGCTAATGCGCA

CACCACTCGTGTACGCGCCATGGCTGCTGTCTCCACGGATAATGAAGAGG

AGTAAACCGGCTCCGTGAAACGACCCACTACCCAAGCACACTTATGCACG

CACACACACATGCACATAAAAACGCCTACACAGCACTCTTCCCTGAACAG

TCACCATCCCTTCCCGTCTCCCAGGACAATTGCTTTCGTGTAGACGCCGT

GAAACCGCAGCAGCTTTCGCAAACATCCTCCACTGTCAGCTTTTTAAACC

TCTGCTCCCCTCCACTTCACTTCACNNNN

>TSCG.R72.esd 725 0 725 ESD GOOD: 103-628

GCACGAGGATGCTACCGCTACTGTAAAAACAAGCCCTATCCTAAGTCTCG

CTTTTGTCGCGGTGTTCCCGATCCCAAAATCAGGATCTTCGACCTCGGCA

AGAAAAAATTCGGTGTAGATGAATTCCCGCTTTGTGTGCACATGCTCTCC

GATGAGTATGAACAACTCTCAAGCGAAGCATTGGAGGCTGCTCGTATTTG

CGCTAACAAGTATCTTGTGAAGTACTGTGGCAAGGATGCTTTCCATCTTC

GTATGCGCGTGCATCCGTACCATGTTGTGCGCATCAATAAGATGTTATCT

TGCGCGGGCGCAGATAGGTTGCAAACCGGAATGCGGGGTGCTTACGGAAA

GCCCCTTGGCACTGTCGCCCGTGTCAACATTGGTCAAGTCATAATGTCTG

TTCGCGGCAAGGACATGCACAAATCTCAATTCATTGAGGCTTTCCGGCGC

GCTAAAATGAAGTTTCCTGGTCGTCAAAAGATTGCTGTTTCTCAGAACTG

GGGATTTACCAAATGGAAGCGGGAGA

>TSCH.R95.esd 501 0 501 ESD GOOD: 96-325

GCACGAGGCCTCCTTTCAACATTGTTTTTCAAGATGAATCACAAGAATCA

GAAGAAAATATCATGTGGAATGTGGTTTGCCTACTGTTTAAAAATACGTC

CATATACAAGCACTTGCACACAATCCTATCTTTTTATACTACCAATGCTA

CAACTCCTATATCTATACTATACGGATGTTTCTTTGAAAAGTAAATAAAC

AGATTCATTCTTAAAAAAAAAAAAAAAAAA

>TSAO.R58.esd 808 0 808 ESD GOOD: 107-581

GCACGAGGCCCAACCCCGCTCTATTAAGGAAAAATCAGCCCTGATCCCTT

TCCTTCAGTTGATGATCACGTGCTCCTTCGTCGTTGTTCAAAACTAGTTT

TATCGTTACTGCTTCCTCTTGCTGATGTTGTCAAATGAACTATTTGACAA

ATCACATCACAGTTAGATTTGTTAGCTGAATGCCTTAACGACGCTCCCGG

CGACGCCTGTGAGATGTGGTGGCAGTGGTGGCGGCCCTGCGGGCGAGGAA

GGCACGGGCGTAGGCGCTGATCACCCTGCAGGCCTCCGTCACCGCGGCGT

CATGAGCGTCCGCTGCTCGCTGTCGCTCCCTTTCTGCCTCTTCCTGCGTC

AGCATCTCTTCATGCTCTATCCACAGAGCCTGATGGAGGCAGAAGGGAAG

TTAATGGCATTCCATCTGTAAAATCTTGGACAAATTATTGGCTTCGTTTA

AAAGAACGCATGCCAGTCTGGAAAA

>TSAK.R96.esd 700 0 700 ESD GOOD: 68-514

GCACGAGGGAAAGCCTGGCAATCAAGCAATTGCAACAAGTGTGGCAGATA

AGCAAACTAGAGAACGTACTTGAAATGCTAATTTTATTGTCTTCCAAACT

TTAGAATCACTGTCCAACCATTTAATAATTGAGCAGCATTATTTTCCACT

TTGACTGAGATGGAAAAATTAGGCATTATTCATAAAGCTAGGCCATGCTG

GAATGTCAAAGATGGTTATTTACCATTTAGGCCATTTAGGTTGATTTAAT

TCCCTTGCTGAAAAACAGAAATGGTTAAACGCAGTTTTTTATCATTATCA

CGTGCCAAGGTAATGTCTGGCATCCCTTTCAGAAACGCCACACTTAGGTG

ATCACGGAATAAGGTCACAGTAAATATCAAGTCATTAAGCACTAAGCATG

ACAGTGGCATCCAATAAGTGACAAGACTTCAAGCACACTCGGTCCGA

>TSAI.R1.esd 636 0 636 ESD GOOD: 97-632

CTTTGGCATTGCGCTGTAGAGAAGAAGTTTTTAATTTGCCAGCATAGAAC

GAAGTGGTAAATTCTTTTTTCATCTTTATGATGGAGATTATATGAATAGC

ACTGATAATTGCATATTGCGGATGCAGTACTTGCAGTTGGGAGGAATTTG

GTTCCTCTCTATGGTTAGAGAATACTCATTTTCCCAACAAAATGGTGTGG

AAGACATTTCCATCCACGTAATAGGATTTAAACAGAATAAATGAGGAATT

ATTAGCTCAAAGCTAGGAAATCAATTTGGATAGAATCTAAGAGCTAAAGA

ACGCACGCATTTAAAAAGAAATATCTAGACACTGCATGTTGACGCAACTG

TAGCGGTCTTGAAGACGATAGAAGTCATAATACTAAGCAGAATATGTATT

GCCGACATAATGGGAAAGCTGGAGGATTCTAGTATCCTCTTTAGGCCATC

CTATTTTAGCGCTTACGTAACCAAAACGGAGACTTAAACACATAATCAAC

GTATTACCTAGCTCCTTCTCCTCCCTTGAGCATCGT

>TSBL.R14.esd 576 0 576 ESD GOOD: 86-575

GCACGAGGATTACTCCAAGGGAAAGATTCGAGCCAGAAGTGTTAATTTGC

TACGAACCACCGCCGTGAAAGCTATAACTCATCCTCTTCGTATTGCAACT

GTCGGCAAAGCCATGTACTTTCAGCCTCGTGAGCAGCTCCGTACTTTGGA

TTTACTTCTCAATCCTACCGTCTTGTGCATGGTCATACCTCTCCTCTTCT

TAATGCTCATTCCCAAGCTCATGGACACCAACGATCCGGAATTCAAGGAA

ATGCAGAAAAACAGCATTTTTGGTGGTCGTAGTGCCGAAACACCCGACAT

CAGTGATTATCTCTCATCAATATCACTTTTTGGTGGAAGCACCGCCAAAA

AGCCGCCACCCAAAAAGGCAATCGCTGCTTCCACTACGTCTACTCACTCC

GCGGTGCAAGAGTCGCGTAGAGGCGGCAATCCCCGAAGGCGCAACTAACT

AGCTTATCCTATGTCGAATTGTGATCTTGATCTTGTCGNN

>TSBC.R45.esd 624 0 624 ESD GOOD: 88-585

GCACGAGGCAGGTTTTTTTGGCGTCCATCTTTATTGGGATCGCAAGCTGT

TGTATCTCAGCAAACATTCTCGCCGCAGCCGGTGCAGTTCATGCAGCAGT

CGACATCGTTTGCTCCTTGGAATATTGCTTCGATACCCTACGCTTATTGC

GGACCTCAGATGCCTAGATGGCAATTTATGAATTCGAATTATTTCTCCCA

ACGAGCAATGGCGCCACAAGGTATACCACTGTCACGCCCACGGCAGCAGG

TGGTGCAAGAATTACACTCGGTTAACTATGAGGGCAACCTAGATAGTGTC

GTGGAGCCGACACCGGTACCGGCATTCACCACTGGTACTCCGCCGCGTCA

GGAGGATCCACCAGCGCCGTAGTGGAAAAGGCGTCGTTTGTGCCATTGAT

CGCAGATTTTATTCTTTCATAGACCGTATTGTGATGTCATTCTTGTTATT

TTTATATCTGGTTTAAAGGAATACATCTGGCCTCAGGGAAAAAAAAAA

>TSAU.R47.esd 559 0 559 ESD GOOD: 96-556

GCACGAGGGGCATCTGGTTTCACATCTCTTTCCAGACAAGGTTGGTGGTC

GTCCTGCATGGTTGGCCCTTAGTCACCTTCCTGGCGATCTGGAATGTCCC

CACTGTCATCACCGCATGGTCTTTCTGCTTCAGCTCTACTCTCCCCTCGA

TGACCGGGAGGACTGTTTCCATCGCATGCTTTTTGTTTTCATGTGTAAAA

ACGGCACTTGTTTTCTGCCACAGAATGGGGTTAAGACCCAGCCGTTTCGG

GTCTTTCGCTCCCAATTGCCAAAGGAAAATCCCTACTATAGCTCAAAACC

GCCGTCAGAGTCTGAGGAGATAACGGAAGAATCTATGGTGGAGCTCTTTC

GATCTGGCAAAGCTCCCATGGTCGGTCTCACGGTGAACCTCTGTCCGGTT

TGCGGATGCCCGGGTACCAAGTGTTGCGTTAACTGCGATAAGGCCTATTA

CTGCTCGAAGG

>TSAK.R32.esd 617 0 617 ESD GOOD: 100-563

GCACGAGGGGTTGGTTCGTTCGTTGGTTTGACAGGATGAATGTATTACAC

AACGTAGTCCGCGTCCATGTGCCTGATCTGCTCGCTGCAGTACCTATTCC

TGACACCTTTTCCGGACTCTTTTCACTGTCACTTAAGGACATTATCGGGC

TAACCATTTTCAGTGGTGTTTCTGCTGCTCTAGGTTATTCTGTGTACTTT

ACGATAAAAAATCGGTGCTTTCCATATCATATCAATAAAATAGTCAAAAA

GGATCAGGAAAAGGTGGTTGACTTTATTGACATCGAGTCGATCGGTAGGA

AAGGTGTTTACTGCCGTTGCTGGAAATCAAAGAAGTTCCCACTTTGCGAT

GGTACCCACAATGCTCACAACACGGAGACCGGTGACAACGTCGGTCCACT

TATCATTGAAGCAAAGAAACACGCCTGATTTTGTGATGGACATCATGATA

GCTTCATTCGAAGT

>TSAV.R59.esd 636 0 636 ESD GOOD: 100-599

GCACGAGGCCTGTTCGGAAATCATGATTGATCGTACGGTCTGCATCAAGT

GTAAAGGTGGATTTGGGCCTCCTGCACTCTTTGTTCGTAAGGACGATCCG

CCTCTATGCAAAACCTGCTTTCAGGCTGGATGCATGCACAAGTTTCGGTC

ATCTTTTGGGAAGGCAAACATCATTAGGAACCGGGAGGCGGTTGCGTTGG

CCTTTTCAGGTGGCTCTTCGTCATTAGCAATGCTTAATTTGGCGAAAATG

TTTACCATTGTGTCCATGCCTGCTTCTGATGCGACACCACCTTTCACTGC

TTAGTGCCAAGCTGAGACAGAAACTCGGAAACTACGCTTTGATCCAACGG

TTGTCTGTTTATACGATGCTGGACAACCATACCCCGAAAAGCAGGAGGAA

GCCATGAAAGATGCAGGCTTTGAGTACCGGATTGTGAGAACAGATGAGAA

AAACTGTAATTTCGACGGTTCGACTCGCATTACGAATTCTACTCTCACAG

>TSAM.R34.esd 659 0 659 ESD GOOD: 92-512

GCACGAGGTTCAGATGTCCCGCCGCCGCGCTTTGCTAGGGGTCCCATGCC

TCCTCCACCATTCCGCGGTCCACCCTCTATGTACGGCGGGGGTAGACCTC

CCATGAATGGGCCCGGTAGTGGAGGTGGTCGACGAGGAGGTCGGGTCGAA

CAGGTGTGCAAGTTCTTCCAACAGGGTAATTGCCGCAACGGAAGTAACTG

CCACTTTCTTCATCCTGGATATCAATCATCGAATTGGTGAAGGGGTCCCA

CACCTAGACACGGTCCTAATTATGTGTACATAATTATTCTTCTGTATATA

CTAAACAATAACTGTGCTGCCTTTTCCACTATAAATAGCGTGGCATGCTT

TCCTGACAGTGTGCCGTAGGTCGCTGGGACTTAATAAACTACTACTGTTT

CGTAAAAAAAAAAAAAAAAAA

>TSCF.R13.esd 591 0 591 ESD GOOD: 108-458

GCACGAGGGTGATTCCAGTTTCCACTTACCTTAAGGATAAACTCGGTATC

AAACATGAGGTGTTCACGAGCGTGCCCTGCGTAATTGACTCCTCCGGCGT

CTCTGCGGTGGTCAACCTCGACTTTTCTCCTAGTGAGAAACAAAGCCTGA

TGGCCAGTGTTGATACCCTTCAGGCGATTATCGCAGACATCAAGTGGTGA

ATGAAGTGAAAAATTTGCATCCGTTCCATATTACCGTATGGATTGTGAGG

TTTAGGAACTTTTTTTCACTTGAATACAGTAATTGTTTAGTGTTCTTGTG

TTTTTATCTGCGAAATATAATTTTTCTTCCATATAAAAAAAAAAAAAAAA

A

>TSCG.R95.esd 598 0 598 ESD GOOD: 103-486

GCACGAGGATTGAGATTCTGCAAAGACCACAGAGAATTGCTAGATCTCCA

AGAGACATCACGGAAGGTTTTAGATGCTGCTAAACTCTGTGAGGATCCAA

TGATCTTCTACTCTGTGTTTCGATTCTTTGAACGTGCTGCTCCACTCACC

CCTCCTCTCATTCAGGATCCCAAATTGCGTCCCTATTTAGCATATTTCGC

AAATTTATTCGGCCCAGGAAGTCTTGTTCTTACCAATTGAGTTGGAAAAC

TTGGACATTTTGTCTGCTTTCTTGTCGACTTGTCTGACTTTCCTTTTTTT

TACTAGTTTGTATTTGGCGTTGAAGTACAATGAAGGCATCTTCATGATCC

CACACAAATACATTTACGCTTTTTGCAGAACGAA

>TSBQ.R52.esd 632 0 632 ESD GOOD: 88-289

GCACGAGGCTTAAAAGTGCTCTTATTTTCGCCCTCATTGTCAGCTGTGTA

AAACGCACATTGGCACGATTTCTCCTTCTCATTGTTTGTGTTGGGTACGG

CATCACCAAATCACGACTGGGTACGGTCTATCGTCGCATCGTGTGGATCT

GTGTAGTCTATCTAGTTTGCTGCCTCCTTGACAGTGGTCTGCGTGCCTTC

CA

>TSAM.R80.esd 611 0 611 ESD GOOD: 93-437

GCACGAGGCGCGCATTAATGCGCACTTTACAACCCGGCTACCCGTGCATA

AATTGAAATACCTTAACTGTTGTATATTTGATGGGCGTATTGTATTATCA

AGTTAAAGTGTTTAAGGGCTAAACCTTTTCCCTGTGGAAACAACCACTGA

GAGCCTCCTCCATTGTACCCTCTTGGTGATCCGGCCTACGGGCTATGTCA

GTAGCCCCCCAAGCCCTTTTATGCTTTGATGGTACACATTCTGTCTTCTC

ACGTCTTTGAAAAAGTCTCGGGACACCAAAAACCTTCCAGCTTAACTGCT

GTTGGGTTACCCCTATTGAAATGAGTTTGAGAGTCTTTTTAATAA

>TSAF.R50.esd 478 0 478 ESD GOOD: 92-408

GCACGAGGCAAAGAGCTGATCGATCAAGTGTTGGACCGCATCCGCAAGCT

TACCGATCAGTGCACGGGACTCCAGGGCTTCTTGCTATTCCACTCTTTCG

GTGGTGGTACAGGTTCGGGTTTCACCTCTCTTCTTATGGAGAGACTGAGC

GTTGACTACGGTAAAAAGGCCAAACTTGAGTTTTCGATTTACCCGGCTCC

CCAAGTATCAACTGCTGTGGTTGAGCCCTACAATTCCATTCTCACAACTC

ACACAACCCTGGAGCACTCTGATTGCGCTTTCATGGTTGATAATGAGGCT

ATTTACGACATATGCAG

>TSAO.R43.esd 774 0 774 ESD GOOD: 108-463

GCACGAGGGACACCTCCACGACAGTCCATAAAATTGGCGGAAGATAATCC

CGTAAGAGCCTCAGAGGAGCTCCCTTCTGTCATTATTCGTCCAACCACAG

CTAACGAATTCTACTCCCTGTTGAACCAAGCAATGGACTGCGAAGAGGAA

ATTCCAAACGCTTCAGTGCCCATGGCGTCATCCTCACAACCCGCTGTCAA

ACAGGAAGAACCCCAACGACCTGCTAGACCCACCTCCGCCCCGTCCTAAT

TGTAGAATCGAAACATAAAGCATCGTGACTGTACTCTGCGCTGTACAAGA

TGTCGGTGATTGAATATATTAGTTCTATTACCTTGTAAAAAAAAAAAAAA

AAAAAA

>TSBH.R95.esd 449 0 449 ESD GOOD: 79-443

CGGCACGAGGCTCACTCCATGAGTGATTTAGGATCTTGTGCGCAAGTTTC

AGTGCCTTTCTCAAGAATGGAACAAAAAAAACCATGATTTAACTATTTGT

CAGGAAAAACTGGAAAAGCTGACGCTTGACTTGACTCGGTTTTGCTATAT

GCCTAATCGTGAAACATCCGCTTCCAAAAGAGAGCTTCTCATTGCACGTG

ACACCCTGGAGATTGGCGCTATTTTGTCTCTCGAAAAGCGTGATATGGCT

GCCTTTGAACGTTCTATGACACAATTGAAATGCTACTACTGTGATTACAA

GGTCAATTTACCTGATTCTCCCTATAAATATGAATTGTAAGGGTTGAATT

TGTTGCGCCTACTTG

>TSAO.R54.esd 734 0 734 ESD GOOD: 109-527

GCACGAGGCTTAATACGCTTTAAGTAACACTAACGAAAGCTTTTATCTAA

CTTGCCTTTTTTTAAAACTCGGGAAGAATTCAATTGATTCGCTGTACAGC

TTCTTGCATCTTCCCTTTATTCCCTACAATTTTCCTAACTTTATTTAATT

TGACCTCCCTGTGGCTGTGTTAGGTAACCTGGGACATTTTCTCCCGTATC

GCTACAACTATTTTGTTTTCAGTGACCAGCGTTGTTTTTTTTTGGTGGCT

AAGAGTTGAAAAGTGCTCTCTTAGGTGAGGAAACGCTGCTCTTTTGTTTA

CTAACTGAGAGGAGAGTGTTTTATTGTCTAGGTGACAAAACAACAACGGC

GGGTGATAATCACGAGGCTTCACTCATCTTGTCGCACGACACCCCTGGGT

TACTGGAATGGGCCTCATT

>TSAR.R73.esd 652 0 652 ESD GOOD: 118-422

GCACGAGGGTCGTCTGACTGACCGACAATCTGTGTTGCATGTCTCTTTCA

CCCTCTATTTCTTCCCTCACTCCCCTCTTCCGACTCTTACTTGAAATAAG

GAGAGGAAATATTTATGCAATCGTTGACCATCGCGATGATCTCATATTTT

CTCTCCTAATTTCTTCTCTCCCCTCCCGCTTTTATTCGACCTCCCTCCCC

ACCCCTTTCCATTCTCCATATCTTCCATCGTCAATTGCGCGTGTTTTCAT

TACGAGTACTTATTAAAGTATAATATCATCGCTAAAAAAAAAAAAAAAAA

AAAAA

>TSAC.R62.esd 286 0 286 ESD GOOD: 94-284

GCACGAGGCTCAGATTCTTCCGCCTCTTCCTCGTCCACTAACACGACCAC

GAATACCACCACGACATCAATGTCGGGAGAGTCTCTAGACTCCACGAGAT

CCTTCTCCCCCGCTGCAGGCACCCCCAAGGTCAAATATTCTCCAAAGAGG

GCAGTGAATCCTGGTAGTATCGAGCTTGTGAAAACAGACNN

>TSAF.R75.esd 575 0 575 ESD GOOD: 93-394

GCACGAGGGTCGATCAACTAGGTTTAGGTTTTTATCTTCGGACATATGGA

TGCTCTGAAGCAGAGGATTTTAGAAACGCATGAGAGTAATGTAAAACTTG

AAGCTGAGCTTGCTGAGAAGGATAAGGAACTTCAATCAATCATACAAGAG

CAGAAAGCCCTGGAAGCAGAAGTCAGGGCTGCTCATAAAAATCGTGACAC

TCTTGAAATGAAGGTAGAGGCTGCACAGAAGGACCTAAAGGAGTGTCTCG

TAAAGCTGGAGGAGAGTCAAGAGCGGCTGACGAGAGTAAAAAAGCTGCTG

CA

>TSBT.R91.esd 782 0 782 ESD GOOD: 90-621

GCACGAGGCACAAGTGCTTTGCCCGGAACAGTGGCCACCGGTGCGGCCGG

CGACCACACTATTGCGATGAACCCAGCACCTCCCACAGATAGTACAAATA

TGTGTTATCGCTCCCTATGGCCAACTACATCGGCCTTTCCTCAAGCTGCC

GCGTCATTTCTTCCGTACATTGCGTCACAGCATCAATCGTTTTATGTTTC

GTTGTTGCAGCCACCACCAGCAGGTTTGGCCTCTGCTGCTTCGGTGCATA

CTAATATGTCGCGGAATCCACCTGTGCCGTTTTTCCCTGCCCCTGTTGCG

GTTTCAAGTACGCAATTGTCGTCTCCACAACAGAGGGCGTATGCCGTTTC

CGTTAGAGCACCACATCGACCTCCCAGCAATCGAACCATTAGTGGCGACT

GCAAAGTCATCGACGAAACTTTGGAACAAAATCCGTAAACTACACTATCT

GGCTTCTCTATTTAACCCTCCCTTGTACATAACCTCGATTTTTATTCCAT

TTTCATTTTGGGACTGGATACAAACTGTACAG

>TSCG.R34.esd 664 0 664 ESD GOOD: 95-638

GCACGAGGAGGAGAAGGGACCGGTTGCTCCTGTAAAGCGTGTCAGACAGA

AACAGCCTGGTAGATTATACGTGAAGGCTGTTTTCACTGGCTACCGTCGC

GGACTTAGAAATCAACACGAAAAGACAGCTCTTCTTAAGATTGACGGTGT

TAACACTGCTAAAGAAATTGAGTTTTACATGGGGAAACGATGTGCCTATA

TTTATCGCGCGCAGAAAAAAAAGCACTTGCCCCGTCGACACAATCCCACT

AGGATACGCGTTATCTGGGGGAAAGTAATCAAACCGCACGGTCGAAGCGG

AGCCGTCCGAGCACAGTTCAAACGTAACCTACCCGCTAAGGCTATGGGTA

AACGGATCCGAGTTATGCTGTACCCGTCTCGTGTATAACTTCCAATAAAG

GTTACTTGGCCAATGCTTGTCGAGGAACACACCACTTCCGCATTTTTATC

CCTGAGGTGTTCACATAAGTAGCTGTTATCCATAAGCGATTGTTTGGGAG

TCGTCCCTGTAAATTGTGTTTACATACAGTCTGCCTGTGTTGTC

>TSAO.R39.esd 534 0 534 ESD GOOD: 105-534

GCACGAGGGTTAGTTTTCTGCTGCGCAACACTTACAGTTTTCAACAAATA

TTCTTCCGGCAGTTTGGTGACAAATCTGAATAGGGTGACCGTGAAGACGT

GCTCTCCTCTTCGGCTTGCAACGCCTAGTTGCGCTTATTTGTAGCTGCAA

CATACCTCTGGAGACTAAACGTTGTGGAGCTTGAATTCGACGAATGTCAT

TTATCATCGTCATCACCACTTTGTGAGGCACTTGTTTGGCAAGCATGGTG

GCCTTACTTAGCAGTGATGAATGCGAGGCTTGCCCCGTCATGATTTCAGA

CCGATTGGATAGCTGGTTTATTATGCCCATTATCACTCCCTCCTTCCGAA

ATGGGATTGAAGACGCGTGGATTGTAGTGTGATTGTGGTGTCGCCTGCTG

CGACGTTAGTTGTTATCGGAAAATGGATNN

>TSCD.R86.esd 610 0 610 ESD GOOD: 96-589

GCACGAGGATTCAATCCAGATTTGTTCCAGGCTAGGAAGACACTCTCATT

AAGCCTTAAATTCCGCGATCAATATTTCTGTTAGATAAATTTCAGAATGC

ATTAAAAGCTCACCAGGGATACCATCATTATCATCATCATCATCCCACTC

GTTGAGTATTCCATCGTTATCACTATCCTGAAGACACATACAAACAGTAA

TAATTAACGCCTCAATTTAAGCGTCAGCTCCTATTCGCCACAAATTTTCA

TATTTACCTGCTTGTTGTCAAAGAATCCATCGTTATCATCGTCAGGATCG

ATGATATCTGGAATACCGTCACAGTCGTGATCCTGTAGATGGTCGGGAAT

GCCATCATCGTTCATATCATCATCAATGGCATCGGGGAACCCGTCACCAT

CGGAGTCAATTCCAAGACCCCAAACTCCGTCAGTGTCGAGGTGATCGGGG

ATTCCATCATTGTCATCGTCTCGGTCGAGAATATCAGGAATTCC

>TSCB.R77.esd 605 0 605 ESD GOOD: 106-523

GCACGAGGCCTGGTTCTATCAATCTGGAAAGGCTACATACGGTGCGAATA

TTAAATGAGAGGAAATGAAGAGCACTTCTGAAGGCCAATGCATTCCCTAA

ATCTAGGTGCAAAAATCGAGGAGTGTTGTCAATGCCCTGGCGCCGTTTCT

AATATTTTCCCCGTCATGTGTTTCTTTAGTTGAATAATGCCAAGTCAAGC

GCCAAACTAACTTGTTCAATCGGTCCCATTAGCATTGCGCCAATGTCGCT

CGAAAGCGTGGTCACTGCTACTAACCAAATATAGTAGTATGCGAATCTAC

GTGGCAAAAGAAATCGTGATTGTTGGTGGAGTAAATCAATTGTAAGCAAT

AAATGTTAGTCGTTTGGCACTGTTGTTCTTTAAAATGTCGCCTATTACTC

AAACACGTATTCACTTCT

>TSBP.R29.esd 658 0 658 ESD GOOD: 93-350

GCACGAGGGCGGCTGCAAGAGTTGTTGAAATAACTGCAGTACTTGACTTC

CCTCCTCCAGCCACTAACCGCTTCGGTGATGGATGGCTTAATACCACCGA

TAGCAGCGATACTTTGTTCAAGCCCCTCGTAATGCCAATGGTGCCTCTAA

TTTATACGAAAAAAAAAAAAAAAAAAAAAAAAAAAAAAAAAAAAAAAAAA

AAAAAAAAAAAAAAAAAAAAAAAAAAAAAAAAAAAAAAAAAAAAAAAAAA

AAAAAAAA

>TSAI.R45.esd 844 0 844 ESD GOOD: 109-600

GCACGAGGGCCATTTCCTGTTTCGTCTGGCCGAAGCTTCTATCTGGCACG

GATACTTTTTCCTTGTTGGGTGGTACCCATCGTTTACAGACATTCAGGAG

ACCGGTTTGCCACCACCATGCGCAACCAGTGCAAAACAGATCGAGCAGGC

TTATAACGCCCTGCTGAGGGACTCACAAACTGACACTGGAGGTTGCAGTA

GGGAAGGAGAAGGGAGAGGTGTGTTGCGTTGCTTGCTCTGCGGTGAGTTT

CGCACCAACAGTGCTGCTGCCCTGAGGGCACATCTCAACGGCAGTGGGAG

TGATGCCGATGCCTCGCCTGCAAAATGCGCTCTCTGTGCCCTCTCGGTGG

TGCACAATCGGAGGGACATGGTACTGTGCTCCATTAAGGCACACCTCCTC

CTACATTTGAACATTTACCTCATGTGTCCGCAGTGTGGCTTCACCCCGCC

TCCGGATTTGACCCCATCTCTGGCCGAAATTTGTTTGCGCCT

>TSAF.R73.esd 670 0 670 ESD GOOD: 115-612

GCACGAGGGCTTTTCGTCTAGTGTGTCGCGACAGTTTGTTCATTGTTTTT

GATGCTATTACCTTTATTTTTTAACCTGACTTAATAAGTAACTGATAATA

TGCAGTCTCACTTCCACGAACCGGGGTCTGTGGCCGTGTCTTAGAGTTTT

TTCTGTGTGATTGAAACACCAGGTGCTTTAGAACTTAGGTAGGCAGATCA

ATCAAAGCATTCAATCAGTTTTTTTGGAAATATATTTATGTCATGCCACT

TTAACGTTCAGTATTGATTGTCACCCCTAGTGATGCCATTTATGTATCTA

GATCTTGATGAAAGTATTATACTGCGGGTTCTAAGTTGCGCAGGTACTGA

TTAGAATTCAAAATAGGAGTTCTAAAAGAAGAGAACCTGGTGGAAGACGT

CCGTATTGGCTATGCTAACCATTTTACCATTTTTTATCATTGCTTCCGGG

TTGTACAAATGAAAATCGTGCCTGATTTACATATCGACACCAGTGTTC

>TSBW.R40.esd 626 0 626 ESD GOOD: 95-320

GCACGAGGGCTAGTCGACTACTCCCGCTGCGCCAGGACCAGGTGTTTGCA

GCACGTCCGTCAACAATGCTTCAACTGGATGGCGCCCGCGAGGGCACACA

CAGCGATTTTGCAGACTTGGATATTTCTGGGTCTTCCAGGCGACCACCAT

ACTCGTTCCATTTCGCGCAACTCGTCTGAAGAGCAGGCCTATTTGCACAA

ACGCCTTGTCGTCGGCTTCCCACTAA

>TSAM.R91.esd 520 0 520 ESD GOOD: 101-274

GCACGAGGGTTCTCTCTAGAGTACCGCACCACACGGGAGCGAATTCTCGA

GCAGAATGCCAAGAAGGCAAATGACCAGGAGCGCAAACAGACCCGCGGCA

AGCTCATTGACGACGTACCAAAAATCCGCACAACTCCTCCAGTATCAAAG

CAGCAAAGCGGCAGATGATATGGA

>TSBP.R94.esd 743 0 743 ESD GOOD: 91-412

GCACGAGGATACACTCACACCTCTAAGGAGGTCATGCTTGTTTCCTGAGG

TGAGTGAACTGCGGGTGGAAGCATTCACGTTTTGGAGGAAGTGCAGTAAT

TGAGGTTACTATCTTTGCCTGCTTGGTATGGCGCAACTGAAAGGTGTGCA

ATAACAGTGTTTTCTTCAGAATCAGCCAGAACTAGTGGTTGCGAAACCGT

CTTCTTCCAAGAAAACGCAACGCAAGGTCTCGTATCACCGTAAAATATAG

CACTTGAGTTGACGATTGAAAAAAAAAAAAAAAAAAAAAAAAAAAAAAAA

AAAAAAAAAAAAAAAAAAAAAT

>TSBW.R48.esd 523 0 523 ESD GOOD: 100-523

GCACGAGGCTCCGAATGGCTGGAAAAAATCAGCCGCTGGGAGGATGTCTC

CGCTCTACTGTCCACGGCAAACACCTACCTCAAAACCCTCCTTCCCGAAG

AGCATCATAAGAAGCTGGGTATCCTCGGCTTTTCATGGGGTGGAAAGCAG

GTTCTGCGTGCCTGCTGTTCCCGAGACTACGGCTATCTAGCGGGAGTTTC

AGTTGATGGCTTTGCGCTCGAGCCGGAAGATGCTGAAAATCTCTCCATTC

CCGTGTTCTTTATGCCCTCCGGTGAAAATACTTCTATCGAGCCTATTAAA

AGGATTCTAGACAAGAGGCCGTTTGGTGATCGGTGTANGTACTACACTTT

TGCGGAAGAGAGCCACGGATATGCTTCGTCTTTGGGGGATCTGAATAATC

AGAGCACTCTTGAAGCCGTAANNC

>TSBN.R9.esd 544 0 544 ESD GOOD: 91-544

GCACGAGGTGAAGATCTTGAGGTGGAACTGGCAAAAGCTTCCTCTGATAT

TGATTTCAGGGTTCTTAAGTCCAGTCTAGAGGCACAGAAGGCCGAATTGG

CAGATCTGTGTCGTAAATTGGAAAGCACGCAGGATGAGTTGGAGGCGATG

AAAGGTCTCTACGCAAATCACAGTGCAGACTGTGAACATTTCGGAGCCAT

TGATGGCCCAAACCGTTAGTCAAGTAAACAGTCGTCACTCGTCAATCACA

CCGATCTCCACTCCACAACTGTTTAAACACTCCCTTCCCCACTGTCCCAC

CTGAATTGATTCAAAAACATGCATCTCTCTTTGACCACCTTTCGACTCCC

CTTAGCTTTGCATGCTGCAGCGCATTCAGCTGTTGCGTATACACGGCCAC

AAGTCTCTCTCCTCGCCTCCGGTTTTGTATCCTCTTCACCACCTCTCTTT

CNNN

>TSBB.R50.esd 597 0 597 ESD GOOD: 97-578

GCACGAGGCGGCATGCAATCTTCGTAATGTAACCCACAGGATATGTGGAT

GGAAATTAAAGTTTTACGAGTCTGCGCATGAGACGCTAGTTTGCCCTACC

GTCCGCGACCCTTATTCCTGCACATTCACAAACTATGCCGTCACATGCCT

CCGGGATACCCTCGTGATTCCCCAGGCACGTATCTTCAACGATTCCTCAG

TCGCCCTCTCCCGCGATGGTCAGTTAATTGCTGCTGTCGTTGCCCCTTCT

GGTGATCATGGTTGCCCAGATGTTCTTCAGGTGGAGGAGAGTGTAGTGGC

TGTATATTGGACAGAGCCTGTGAGTCGTCGCGGACACTGCATTTTAGCGG

TGAGTATGGTGCCACCTGCTGAACCCGTGTGCCTCCATTTCTCGCCTTCT

AGCAGTTTCCTCGTCGTTGGAACAGCTAACTCACCCCTCGCTATTAACCA

TCATGTCCCACCTATGGTGCAGCAGTACAACG

>TSBF.R20.esd 372 0 372 ESD GOOD: 107-370

GCACGAGGATTTTTGCTCTCAGTCGATTGTACATTTAAAATACATTTTTT

AATGTGTGACACAGTACCCTTTCATGGAGAAAATCGGACAAGTCCCAATT

ATCTGCTCCGGGTTTGGACATTTCCCTAACCAAGCCATTTCCTCTCCTCT

AGCAGGAGGCAGGCAAGGACAATATCTCTNGACCTAGCCTCAGATCTAGG

GGCAGACTTGCCTCTTCCATTTATTGCCTATCTTATGTCAAATAATCGTA

ATCAACTCCCCTTC

>TSBU.R78.esd 840 0 840 ESD GOOD: 106-545

GCACGAGGGTTCGTTGTGGTCATGCAGAATCTTAGGCATTTACCAGTCTT

TTTTCCCCCGTTACAAGTGCGTAATATGGCGACGTTGAAGGACGTCGCGA

TGCAGCTGAAATCCATTACTAGCATCCAGAAGATTACGTCTTCCATGAAA

ATGGTTGCTTCAGCTAAGTTTTCCAAGGCTGAGCGTGAGTTGAGGTTGGC

TATTCCTTACGGTACCAGTGCTGCTACCTTTTACGAGAAAACGGGCATCG

TGGGCGGGCAAGAAAAGAGGATCAAACTCCCGTCAAAAACCATCTTGTGA

TCGCGGTCAACTCTGATCGTGGTCTTTGTGGGAGCTGTCAACNAGCACCA

TCGGAAAGCTTTTACGTGAAATGCTCTCAAACCCTCCTGGCCGTAANGAC

ATCAAGCTTTGTCTGGACTCGGGACAAGGCTCGCGCCTTC

>TSAJ.R70.esd 636 0 636 ESD GOOD: 101-591

GCACGAGGCTTCTGTTTTGGAACACTTTCTAGATATTTAGTTTTGAAGTG

TGGTTGCATTATACACTATAAAAGTTGTGACTCTAAATCTAGATTTTTTG

CATAAGACCCTTAGTCGGAATGAGAATCGTTCCCCTTAAAATAATACCCT

AATAAAGGTCAATTTCTNCGGAAAAAATTCTTTGCACTTATTCCTAAGCA

TTTGTTATTCTTTGCAGATGCTACATCACTAGGAGAACCAAGTGATTGGG

AATGCATTGAGGTCGTGCTATCACGTCCGGCCGGTAGAAACGCGAGTTTT

GGCTTTAGCATAGCTGGCGGTTATGATGCTCCTCAAGAGAATGGAGATCC

AAGCATCTACGTTACAAGAATTGCGTCTGGAAGCATAGCCGAAGCTGACG

ATCGGTTGCGGTAGGTCTTGTTAATCGTCGACTTCATACTCGATCGAGAC

AAGGTTGTTGGTGTAACTGGGTTGGTTGTACAGATCACTCC

>TSCC.R62.esd 671 0 671 ESD GOOD: 93-598

GCACGAGGATTTATTGGAAGCTGTTCGTGATCAGGGTAGATGGCTTCGAG

AAGTGCTACTTATGAGTACTCTCCACGTGCACAGCCTTTGGAAGGTGTGG

AGTATCCTCTTACTGTACAGTACTGCGGAGAATGTACGCTGCCTATTGAG

TACTGTGAATTTTCCAGTGATCCGGCGAAATGTAAGGCATGGCTTGAGAA

GAATCTTCCTGAAGCGTTTGAACAACTGAACACCGCGGAACAGCTGGCTG

ATGAAAGTAACTCAAGGAAAAAGTCCCGTCAAAGTCGAGGTGGCAAAGGA

CCAGGTCCTAAAAAACCTTCCGAACAGAGGGTGTCCGTCTCTCGTGCTAC

TAGGGGCAAGAATAAGTTTACGACAAGCGTTATCGGCCTGGACACATACG

GTTTGAGCACAATTTTTTATGTGATTTACTTCGACATGCTGGCCGTACTT

CACCTTATCTGTTTGCCAATTTCTAGCCTTGTTTAGCTGCTAAAAAAAAA

AAAAAC

>TSBA.R28.esd 294 0 294 ESD GOOD: 178-294

CCTTAAGGCCAATTTTGTCACTGTTACACTCGAAACCGGTTTCGCAGAAG

CTTCTCAAGCAGTAAAAACAAGGGAATTTATCGATCACAAACAAAAGAAC

ACCGGCATCCATCAANC

>TSBM.R87.esd 527 0 527 ESD GOOD: 94-527

GCACGAGGGAAGGCCCTGCTGGTCTCACGCCTTGCCGCCCACCCCGATCT

CCTCTCACGGCTTGCTGCTGCTGTCTACGAGAAGAAACCGAAACCTTCTA

AGGAAGCAGCTGCTGAGCGTTTGCTCTCCTTCGTTAGGAAACTCACTCCT

TTTAATGAGATCGAATTCGCCGCTAGTAAAAGGGGTATGTTCAATTGTTC

TCCAATTTTCCTCAAACATCAGTCGTCTTTGGGTCTGTTACCAGTTAGAT

GCCTTGTGTGAATGCCTCATTAAGTTTCTCTCCACTGCCGGTTTACCACA

AATCTGTTTTATCCCTGTCGTTACTAATGAATGCGATAATAAATCAACCG

TTTCGTAAGTTGGAAGCCAAGACTTCGTCAAATCCTTCAAGCTTTACAGT

GGCAAATTATTCGAGCTGTTCTCCTGCAGCATNT

>TSBN.R35.esd 594 0 594 ESD GOOD: 100-563

GCACGAGGCGACGTTTTACTGGATGCGTTATAAGACTTCGAGAACCGCGA

GTAACTTGCCTGCTTTTTAGTACTGGCAAAATGGTTACTACCGGTGGCAG

ATCAGAAGAATCAAACAATTTGGGCGCTAGAAAATGTGCTCGTGTGATAC

AAAAGCTGGGTTTTCCCGTTCAGTTCAATAATTTCCGCATACAAAACGTA

GTTGGAATAGTGGATCTGAAGTTCCCCATTCGCCTTGAAGGTCTGCTTAT

GGCTAATGAACAAATGTCCCAGTATGAGCCGGAAATTTTCCCTGGCCTTA

TTTACCGAATAATTAATCCGAAATTGGTATTTTTGATTTTCGTAAATGGG

AAAGTCATCATAACTGGTGCGAAGTCAGTTGAATTAGTTTACGAGGCTTT

GAATAAAGTATACCCGGGTTTTGANGGAACTTCAGGGAAGATGGCTTGAA

TCTCGTACCATTAC

>TSAW.R94.esd 572 0 572 ESD GOOD: 91-555

GCACGAGGGCGGCAGTGAGTACGGGTCTCGCCTTCGCCATCACTTCACGC

ATTTTTCAGGCGCGCGTGACTTGGGACCCGCCACCACGGCCCGTGCTACA

GTAGTATCATTGCCTATCGTGTTGCTTGGACAGTGGACAATAAGGGGCGA

AAGCATTATATTCTCCCTTACTCAAAAGTTTTGACTTTATCGGTGTAATC

CCAAAGCTAGCCATTTCTTCCGCTGTTGTTTATGCATGCACTGAAAATGG

CGATTCAGATGACAGGAAATATTCTAGCGCCTACAGTAGTGAAGTGTAGA

TAATTGCACCAGCTTTGAAAGAAGAAGGGGAAGAAGGGTCTGTCACGAGT

GGAAGAAGAAGAACCTCAACGACTCTGGGAATGGCAACGGACAGCAGGTC

TGCCTCCAGACTCCTGTTGCAGCAGCACCAACGATTGGAGGTTACTCTAC

CTGCGCTGTTTTGAC

>TSBA.R41.esd 582 0 582 ESD GOOD: 101-272

GCACGAGGGAGAGAACTAGCTGACAATGTCCTCTCCAGTAAGACCGAAAT

GCAGAACACTATTGACAAGCTGGAAGAACCAGTGGAGTCGTTGAATCAGG

AACTGATGAGTGTGCGCCACGAGTTGATGGAGAAACGGGAGGAGTTGCGC

ACTGCGTTTGAAAGGGGATTCC

>TSAJ.R41.esd 666 0 666 ESD GOOD: 101-466

GCACGAGGACTAACTCGGCTCTGGGGCGAACATAAAAACAATATCAAAAT

GAGCTTTGATTCATTGTCTCGTAGCTTGAGGCTCTACTACAAGCCGGGGA

AGCTGGAGCGAATTCCAGGCACTCGTCATCAATACCGACTGATTCAAAGA

CCCCTGAATCTCCAGATCTTCTGCGAGAATACCTCCTCCTATGGATGCCA

GTTTGGAANGCATCGAAATCTGGCACACACGGTGACTCAATCCACAGCTC

TAAAACATCATCCGTCCGTCTCAGAAACCCTAACTCTAAAATGCCGTCTC

CATTGTATAGACCGTTCCATCGGCACTCCGACTCTTGTTTCACTGTCTCT

CGTTCTCTCTTCCTCC

>TSCH.R27.esd 459 0 459 ESD GOOD: 108-459

GCACGAGGATTCCCTCTATGTGACCTTCTAATGCTGCGTATATCTGTACC

CATCTGATTTGTTTTTTTTTTTCTCCTTATCTCCCCTAACGCTGGCTCAA

ATCCCGGTTATTTGCCCTGCCCCCCCCGCCACCCCCCTCTTCCTCTGTTT

TGTTTGAGATGTGTGGGCCACTAAGTCGACAAAGCGATTGTGCTCCTCCA

CCCCTACTTTCCTCCTTTCTTTTAACCTGAAAGCGCCTCCACTCTGTTCC

TCCCTATAGTCCTCCTCGCCCCTTCTCTCTGCGTATCTCTCCCCCTCTCC

ACCTATCTATTCTCTCGAGTAATTTGTACAAAAAGCATTTTTCTGCAATT

NC

>TSCD.R7.esd 744 0 744 ESD GOOD: 99-609

GCACGAGGAAATTTTCCTTTTTATTTTTTAGCGTAGGGAAGGGCTGAGAT

GCGTTGAGAGGGAATGGGAAGCTATGGCGTTAAGATGGCGAAAGGTTGGT

TCGAACAGATGGCCCACTAAAATTGGACTCTATGACACTGTGTTATAACA

ACAACTGTAGTATTTTGATTGCTTAACCCTCAATTTAGCAAACGGTGGAT

CTAAGGCTTCTCTATTTTACTTTCTATGGGTGGTATGGCCTCCGAGCCGT

GTCATTATTCCTAATACTTTGTCTTCTTGCGTATTCAGGGAAGTCTCAGG

GAACTGCGTTGGTGCCAGAGATCAGCGAACCCGGTTCTAAAACACAACTA

CCCTGATATCTACTACAGAAGGCTTGAATGCTCTTCACAACTGAACTGCC

ACTAGCCGTGGTCATGAACCATCTGGAAAGTTCGTTTTGCAGCTGACAAC

GGCACCTTCATGAAGATATTATTCAAGAGAACACAAGATAATTCCTCCGN

CTATCTACTAG

>TSBV.R83.esd 552 0 552 ESD GOOD: 100-532

GCACGAGGCGACGAAAAAATCAACTGGGCATTCGATCTATACGACCAAGA

CAAAAACGGTGTTATCACTTTGGATGAACTCACGACTATGCTTAAGGCTC

TCTACAAGTTGGTGGGTGTCCTTGACACAAGTCAACTTCCACCAGGCCAT

TTGACCCCCGAGCAGCACGCAGCGGCCATTTTCGAGAAGTTGGATGTCAA

TAGGGATGGGACTCTCTCGCGTGAGGAATTTATTCGCGGTACCAGTGGCG

ATCAAAATGTGATGGCTATGCTGAAATGCACCGAACCTTGATAACTGCGA

GACGTTGTAGTTTACAGAGGTTTTTCTATGCTCTCGACTCATTGTGATAA

CTTTTACAAAACACCCAAGCCCATCAGTGACACAAAATGGCCACCATTGG

TCCCAATCACTCTATCCCTCCGATAAGCAAATG

>TSBS.R52.esd 490 0 490 ESD GOOD: 96-306

GCACGAGGCGTTCTTTGTGGCCTCGTCCTGTCTGACGCGTTTGCAAATTC

TCTGCTTTTTTTTGTCACTCTCTCTCACACTCACAATGGGAGGCAAATCC

TCCAAGGTTGTTATTACTCCGGAAGCTCCAAAAACGACACCTGAATCCGA

GGGAACGATTACCCAATTGAAGGAGGCTGGAGAGAATGTGAACGGTGAAA

GTATTCCTCCT

>TSAC.R17.esd 588 0 588 ESD GOOD: 111-472

AGGAATTCGGCACGAGGGCCCCGCGTCACCGCCAGGTAGAGCACCTTGCC

GTCCGGAGAAAGGGCCACGCCGTTCGGGCTGGGCACGTTCGACAGGAGCA

AGTCGAGCTGGCCGTTCGGGCGCAGGCGATAGAGCCGGCCCGATGGATCG

TGAAGCCCGCTTTGTCCCTNGTCCGTGAAATAGATTTTGCCCTGGGCGTC

GAGCACGAGGTCATTCACGCCCTTNAAGCTCTCGGTGTTGCGACGTTNCA

GGTGCGGCGTGACCTGCCCGGTCTGCACGTCCACGCGCATCAGGCCGTTC

TTGTAGTCGGTGACGAGCAGGGCTGTGGGGCGTCGACGAAACTTCATGGG

CGCTTTGGGTTC

>TSAZ.R56.esd 667 0 667 ESD GOOD: 101-618

GCACGAGGGCGATATTAAGGATCCCGCGTACCGTACGCTCTTCGTCGAGA

CAGTAATGGTGATCGCCGTCATTCTGGAGCGCAATCAGGAGCTCTCCTTC

ACCGAAGTGGTCGATTTCCGTCTCGTCATTCAAAACGCCATCAAGGATTT

TGCCATCACCAATCACATCACCTCTAATGAGGATTCTCGAGAGAATAGAC

TGCCAACGCCAACTCGACGTTCAAAACGGCGTCCCTCGTGGCCGGAAAAT

GTGGCGGTGACAGACAACTGGGAGGCCTACGCCCGTTTCGCTTCCACACC

TGCCAACATTCGTCTGGGAACCACTTCCTTCCTCGCTAAGGCTTCCATAG

CCCTTCTCCTTCATGGGACCATCAACCTGAGTGATGTCCACAAGGAAGCT

TGCTGTCTTTCTTAATTGCAACACTTTTTCTTCCTTGTCATCGATGTGAT

TCGCTATCTTAGCCCGAAGTCTACCTATAGGCGTGAATTCCTGCGCAATA

TTTATTCCTATTTCTCTT

>TSAQ.R20.esd 657 0 657 ESD GOOD: 86-476

GCACGAGGCACAACTCTTCATTGACTTCGCCAAGTTGGTACGGCTGCAGG

AGAAGCGCTGGGCTGCGTACAATCGTCGCAACTCCACAGTAAGCTCCGCC

TCCGATGAGTCCGGTATTCGTCGAAGTTGGAAGGCCCGATTCTCTGCACG

CGTACCTCATATATTTAGACGCAGTGGTGAGACTCCGTGTGTGAAGTAGC

CACAGTGTTCATACCTCACATTTTGTTTTCTTTTTATCCGAGAGCATTTA

ACCAACACTCCTCCTCTCAGCATTTTCACACTTCGGTCTCACTTTCCCCT

CTCCCCCCTCCCCACTCCAAGTGTACATATGACATTTACCAAGCTATTTA

CAAAATAACACTTTTTTAACTTGAAAAAAAAAAAAAAAAAA

>TSAQ.R62.esd 679 0 679 ESD GOOD: 93-595

GCACGAGGCAAGTGCTCACTTTTCCAGCGGGACAAGAGAACTTGGCAGGG

GAAGCTGGAACCAGGAGCCGTTCCACCAACCCGTGCCCCGACCTCTGAAC

TGTGTGGTTGTTACCATGGTTCCTGGTTATGCCAGGGATGCTCACCTCGT

TGTTGAGAGACTGCCACAGGCAGCCCAAAAATTTGGGGTGCAAATGCAAA

TACAGAGAGACGTTCTTCGGAAAGCGATTGTTGATCTGCCGCGGTTGTTT

AATGAATTCCGCGCAAAGGGAGTGGATCTAGCGATTTTTGTTTTAACTGG

CACCAAAGAGTATCCCTATATCAAGCGCCAGGGGGATCTGCACAATTTCA

TGTTCACCCAATGCATAAAGGATAGCACAATTAAAAAGCCGAATGTCTTC

AACAACCTGATGCTGAAGATAAACGCCAAGATGGGTGGCATCAATTGGCT

GGTGAATGACTTGTCCCGACGGTGGAATGAGGAACTGGTGATGGTGGTGG

GTG

>TSAU.R55.esd 498 0 498 ESD GOOD: 103-482

GCACGAGGAGACAATCAAAAAGATGTGGTGTGCGTGTCTAGATTGGTTGA

AATATCACAAGGTACGCGCTAATGCTTTACGGCGCAGCAATATCTCAGGC

ATTCAGTATAATTTGCCTGCTTCTGATGACTTCTGTTTCTAAAAGAAGTG

AAGGCAAACGGAAGACCTATTAACATTACTTTTAGTTAAGGAAACTTCTA

ATCGTCACCAAGAGACCACCATGATGTCCCCATGGGGATTCGAGGGCATA

ATCNAGTCAATACGCAATCATAGGAAAACGCCAACATATCGTTTGGTGGT

GGAAAGGGGTCGGATTACGAACTGGACCAGGGGACATTTAAGGTCTGGGT

CCACGTTGCTTCATTACAAGTACTAAACTT

>TSBL.R4.esd 531 0 531 ESD GOOD: 96-441

GCACGAGGCAGAAAACCAGTCGTATTGAGTTGGACCAGCTCTACGCACAC

GAGCCCTCTGTTCACACATCCACCAAAAACAGCTACTTTTAGCGCATTCA

CAGTGTTCCTCTCTCGCCTTTCTCCTTTCCACACTTTACCAACTTCTGAG

CGTCTTCCCTTCCCCTTACCGATTTAGGGGATATTTTTACTTAAGCAGAT

GTTCTTTCTTACTAGTGTGCAATCGCCGCGTTTCCAGGTGCTTGTTGACA

CCCCTCCAATCCGTGTGAACGAATACGTCTGTGTTAGCTCAACTTTCACG

CGATTTCTTTACTGCGTTAACTTTTATCCTTATCCCACTCCCTTAT

>TSCA.R93.esd 754 0 754 ESD GOOD: 99-616

GCACGAGGGAGAAGTGGTGGAATAAGCTCTTGGCCAATCGATATGTTTGT

CTCATTAGGAGCTAAGCGTAGGCTCATTCAACTGGCCGCAGAAACGCTAG

ATGGCGATTTCCTAATAAAGGTGATCCTAATCGTAAGGTCACGACTCGAT

CGAGATCTTTTCTCCTCAATTCTTCTTGAAAACGAACTCGGCTACAACCA

CTACCTGCATTTCCTTACCGAGGCTTCGCAAACGCTGGAGGCGGATGAAC

TGATGGTTGCTCATGGAGCCTTGGAGGATTCAAAAGTCTCCACTTTTAAA

AGCCGTATGGAATCCATTCAAACGGAAAGCATTGAAAAGGCGGAAAAGGC

TATGCATCAGTTGCATCTCTTTGCCGCGTATGATCTGGCAGTTAACTTAC

CCCTGTTGGCAGGACTGAACTGAAGTCTGCGCCTTCTCTCGTCCTCATGC

ACGAGTCGATTACGGACGCTGCAGAGTTGCTGAAATTTCAACTTAAAATT

AGGTCGGAGTGGGCTGCG

>TSBA.R96.esd 766 0 766 ESD GOOD: 105-630

GCACGAGGCATGAGCCTCATGATCTTACGTTAGTGCCGATACCGCTGCCA

ACACCGAATCCCACTGAACTAGCTTTCCTTCTAGAAATCCCGGTTGAGAA

ATCCAACGGAAAGGTGGAATTATTCGTAGTCCATCTCACTGCCTTCTGTG

ACAATTGTGCCGACGCGGCCGAGATTTTTGCTTCTTCAATCAATCTCCTC

TTCTCCACTGATACGGAAGCGTCTGGAAAGCCCCGCATTCTGTGGAAATG

CTTCTTTACCTTGCCTGATCTCTCCAACGTAGCCTGTCGAACCCTCGGAC

ACGACTACGGACTCGGTGAGGAGGGGATTTTTGTGGTTCCCGGCCTCGAT

GCCTCCATGTTCATGGACAAAGCTGTTTCTGAGGCGGCAACCATATTCTA

CGACGTCTTCTCGTTGGTGCGGCACGGTACACCATCGGCTGAAGGCGTTT

CAGAGGAGGGCGAAACCGCAGCTCCCGTTTTTGCGTCATTAATGTCGCTG

GAACAACACTGGGACGGTGCCTTCCT

>TSBT.R41.esd 660 0 660 ESD GOOD: 108-332

GCACGAGGCCAGAACAGGATACGAAATGTGCTAGGATTGGTTGCGACAGC

AAATGTAATATTCAGACACATGAGATGCGGCAGCTGTTTCAGTGCTTGGT

TGTAGTCATCTTTGTTTATGCTAGAGGTCGTCTCCGGTTGTTTTGAGTTG

GCTTGGCCCCGGGGCGGTCTCCCACTTTCCAAGTTACTCACCGAGACAAT

GACAGCTTTGTAGGCCCCATACTTA

>TSAA.R62.esd 594 0 594 ESD GOOD: 99-439

GCACGAGGGCCCGCTGACGATTTGACCGATCCCGCTCCGGCCACAACATT

CGCTCATTTGGACGCTACCACTGTACTGAGCCGTGGCATTGCCGGCCTCG

GTATCTACCCTGCCGTCGACCCTCTTGATTCTGTTAGCCGTATCCTGGAC

CCCAACATTGTCGGAGAGGAGCACTACACGGTCGCTCGTGGCGTGCAAAA

GATTCTTCAGGACTATCGTTCGCTGCAAGACATCATTGCCATTCTGGGCA

TGGACGAGTTGTCTGAAGAGGACAAACTGACTGTGGCGCGCGCTCGTAAG

ATCCAGAAGTTCCTCTCTCAGCCCTTCCAGGTGGCTGAGGT

>TSAP.R67.esd 832 0 832 ESD GOOD: 98-438

GCACGAGGCTTCAACCCTCTTCCTAATCCCACTGTAAACACACTCAGACA

GAGACAGTCTCCAACCCTCTTCTTGATCCCAATTAACCACACTCAGACAG

AGACAGTCTCCAACCCTCTTCTTGATCCCAATGTAAACACACTCAGACAG

AGACAGTTTCCAACCCTCTTCCTAATCCCACTTTAAACACACTCAGACAG

AGACAGTCTCCCACCCTCTTCCTAATCCCACTGTAAACACACTCAGACAG

AGACAGTCTCCCACCCTCTTCCTAATCCCACTGTAAACACACTCAGACAG

AGACAGTCTCCAACCCTCTTCTTGATCCCACTGTAAACAAA

>TSCB.R28.esd 501 0 501 ESD GOOD: 101-498

GCACGAGGTAAGACGTGCCTTGTCCAATGCCTACTGTGCTATCTCTGATC

TTTACACGACGGATCTGTGTGATGAGCCCGAAGCTGAATCGAGCTGTCGA

GAAGCGATTGAAAAAGCAACGGAAACAGATGAGTCTAATCCACAGGCCTG

GTTCTGCGCCGCAAATTTGCTGACCATAAAAGCCCAGGATGATGAGGCTA

AGCAGGCTTTGGAAAAGTGCCTTTCGCTTTATTGGCCTCAAGTTGAACAG

GTGGTGTCAGTTCTTCGGTCTCGTGAAAATGGTGGCGGTGGTGCTACCGG

TGATAATGAGGAAGTAGAGGAACTAGATTTGGAAGAGATTTCAGGGATTC

CATATGAAGCACACATTCAAATGGTCAAGATGATGATGGAGTTGGACA

>TSAG.R40.esd 696 0 696 ESD GOOD: 100-348

GCACGAGGCATCGTTTTGCTTATTGCCGTCGCCTACAGCACGGGCATTTG

TAGGATTGCATCAGAAAAAATTGAAATGCTATACTGCTTCCGTGAAACAG

GAGAGAACCCAGTGTTGTGGGGATCTCGAATCTCTGGAACACCCAACGGA

CCATCACCCTCCATGTATTTTCGCCTACCTGGAGGACCCGTTATAAAGGC

TTTATTGAAAGAATGGCAATACGGCTCAAATCAACATTGCACATGGCGA

>TSAN.R66.esd 700 0 700 ESD GOOD: 105-553

GCACGAGGGTTAATCGGGGCCACCAGATTGAACTCACTCGCCGGCACATT

GGCCATGGTGTTGCTCTCTACTACATCGGTGGTGAGGTTTTCGCCGAGTG

TCTGAGTGACAGCGCAATCTTCGTCCAGTCGCCCAATTGCAATCACATGT

ACAACTGGCATCCGGCTACGGTCTGCAAAATTCCACCAGGTTGCAATCTG

AAAATCTTCAATAATCAACACTTTGCGGCATTGCTGAAAGAGAGTGTCAA

CAATGGTTTCGAGGCCGTCTACGCGCTTACCAACATGTGCACTATCCGAA

TCAGTTTTGTCAAGGGCTGGGGCGCCGAATACAGGCGTCAAACAGTGACA

AGCACGCCTTGCTGGATAGAGCTTCATTTGAACGGTCCTCTCCAGATGTT

GGACCGTGTTCTCAGTCAAATGGGGTCTCCAAATCATCCATGCACGTCA

>TSAM.R43.esd 676 0 676 ESD GOOD: 75-580

GCACGAGGCTTCGTTTGGCCCGCTATGAGGAGAAGACTGGTCCAAGCGAA

GAATTTGCTGATTTCATTACTCGCGTAAATGAGGAGCTCGTAGCCCACCA

GTGTCTTTACCTCTCCAAAATGATCATTTTCGCTGACGATGGAGCAAAAG

AGGACGACTCTCGAGACAATCTCAAGGTCGCCTGCTTGGAAAAGTGGCAG

ATACCTGAATTTGCCCGGGAACCCTGCTCGTGGCCGTTGTGGTCCGATAA

CATTCCTCCGGCCCTGAAGAGCATCATACATGATTCCACCAAGGGCACAG

TTCACAACAGCTTGCCTGCAAATCTATGTCGTCCAACGAATATTAGTCTA

TTCACTAAAGACGACCTTTCCCGTTCCTCCTTCTCTCCCCACTCCCTTTG

CGCTGTGGTTTTGAGCGAACTCTTTCAGACTTCCAATAATCTGCCTAAGC

AACCCGTGATGGTCATATCTTTGGATGGAGGCTCTGCACAAGGCAGAAGA

AACCTG

>TSBA.R44.esd 440 0 440 ESD GOOD: 160-440

TTACTCTAGGGGAGCTTGCTCGGTATATGTCAGAACGGGTGTCCTCCTCA

ATTCTGCCAGCGTCTTTTCTCACCAATGGAGCAGAGAGGTTTCAACAGTC

GTGCCCCTGGCCACCGACCCCTGCTGCCGGTCAGTCCGGTTTTCTTTGAG

ACTGCTCAGCAGAAATGTGGCCATCGCTTTGTGGCAGCCAGTTCTAAGGG

CATTCAGGGTATGATCGGCCTCTCCCAGGTGAGGTTATATGCACTTTATG

TGAAAGCCCGGTCGGCAAACGAATGGTCCNN

>TSBU.R24.esd 1009 0 1009 ESD GOOD: 101-297

ACGGCACGAGGGGACTAAGTGCTTCCCTCTTCCCCTTTGGAGCCCCCGTG

CCGCAATCCCGACTCGTGGAAGTTTGTCACAACCTGTAGGTCCTCCATTT

CACATCCATTTCTCATATATTTCAGATTTTCCACCTCTCAAGTCACCTGT

GTCTCTCTGGAAGCCCACGTCTATCACATAAGCACTCTGTTGGGTCT

>TSCG.R30.esd 532 0 532 ESD GOOD: 99-484

GCACGAGGGCCACACGCACAATTAAACCCATTGAGACTCAACCAAGTCGA

CCCACCACGCTAAATACGGACAAACGTGCAAAGTCAACCTCCCACCTTGT

CGCAGCACCTAGTGCTAATACTCGAACTCGACAGGACGTTGATACATCAA

AAATGAATGTTTGGGAGCGTCTGTCTGCTAATGTGCCAAGAAATCCAACT

GCACCAAAGATCGTTTCTCCTCCCGCGACCCCGTTAAAGCGACACCAACC

ACAACAAAATCACACCCAAATGCAGAGCAATCGAACCTCAAATATGAAAC

GACGCAACTCAACCATCACAACAAAGAAGATTACTTGTCCGGAGGACAAA

CCTGGAGTCGTGAAAGCTTCCACACCTACTGTTCAA

>TSAR.R33.esd 565 0 565 ESD GOOD: 102-565

GCACGAGGTTTGTTTACCGGTGACTTCTACGTGTCACACAGAGAACTTGT

TGAAAGTCGTCTGGAGATGATTAGCTCTGCGAAAAGGTTTTTACAAACAT

CGGAAACGGAATCGGTGGTTGCGGATCCAATCTCGCAGTTGTTGTCCGAC

ACATGGACGAAGCACAAGGACGAGCGTTGTATCGGTGTCAATTGGAATCT

TTTTCCTGATGGTGAAAGTGATGTTGTGGCAAGTGATGAGTCAGTTGTGT

TAGGAGGTATTCTGGTGTTTGGGACCACAATTGGTAGCGTCCATCTGCCG

TCTACTGATTAGTGACTACACTAATTGGTGTTCTGGACTGCCTGATCTGA

CGGTCTGGAGCCCTTCCTCTGGGAAAAGCCAAGCTTGTCGAGGTAAAAGG

TCCTAGCGATCAGCTCTCGTCCCAACAAGTGGTTTGGATGGACAGCCTCC

TTTCGTTTGGGNNG

>TSAJ.R95.esd 659 0 659 ESD GOOD: 117-618

GCACGAGGGTGCTCGCTTCTCCACCACTAGTTCCGTATTTCTCCCCCAAC

TTGAGGCCTCGTTTAACGAGCGACAGAGTAAACAATCAAGCACCCTCACC

CTACTCTGCGTCTCCTTCTACCTTATCTTTGCACACCTCTCCGTCGCTGT

GATGGTTGTCCTCTACATAATCCTTCCCATGGGGGACATATGCATGACGG

ATGAGGAGATATCCAAGGATCCTGTGTGGCGGCGGCACTTCCGATTTTTC

ACTGTCCGCTCACTCATCGAGTCTTTCGGGATATCGCACTATGCAGCGAA

GTTCTACATCTACCTGGCCACCAGCGCCGCCTTTAGGGAACAGTGCGCGA

ATGTATTGTGCTGCTGCAAACAACACAATGTCACCGTGGAACCATCCTCC

TATCAAGCACAGATACCGGCTAGTCTCACTAATGGGGATTCGACATTTAT

GGGTGGAATGATGCATTCCACAGCCTCATCCAACGCTTATCGGGGACACT

CG

>TSBL.R31.esd 747 0 747 ESD GOOD: 105-467

NNNNNNNNNNNNNNNNNNNNNNNNNNNNNNNCTGAACAAGCCAGAGCGGT

GTAATTGTAAGCCTACGATAATATCTAACTCAAATTGTACCAAATATACT

GTCCCATAGGGATGTGCTTACCAAGATATAGNCCTCCTCTACCCCTCCCT

CTATGCAAGTCCGTCCAACGCCGAGGAAGACGAAAGGATGGAAACATAAT

GGCATCTGAGCAGAACACATATATAGTCCATATGCACCAACGCCCGCCAT

ATCCACAAATCACCGACGCGTTGAATAAATACACTTCCCATACACCCGCC

CGCAGCGCCGTCCTCCATGCTGCCAGTCTATCCTTCCATAGGGGCAGGGC

GACGAGGCTAGAT

>TSAH.R70.esd 561 0 561 ESD GOOD: 103-561

GCACAAGGTGATCCTCGGATCTCATTAATGCATAAAAGATAGGTTTCCGC

TAGTGCATATAATATATGCTTCATTTGTTAGTTTTTTTCTCATTATTTGC

CTAAAGGTTGAGGATTGTGAGAAATCCCCATGTTTCAGCCAGCAGGTTGG

GTTTCACAACAAGAGGTTCTCATACGGGCAATGCCAAAAATTTGGCAGAA

TTTATTCTCNGATAATTATACTAAGGAAGTCTCAGAGAAACCGATTCTCT

TACTCACTGGAGCAAGACATAGCCCTGTTTTACCAAATAGATTACGTGAA

GCCGGTTTGATAGTAGAAGAAGTAATTTCATATGATTCTACTCCAAATCC

AAATTTCGAGACTCAGCTCCTTTCACTTTTGAAGAAGGAAGAAAATCGTT

CGCAATGCCTGGTATTTTTCAGTCCATCAGGTGTTGAAATGGCATGGGCC

ATTTTGNNN

>TSBT.R51.esd 678 0 678 ESD GOOD: 99-513

GCACGAGGGTGGCTTTTGCCCTTGCGGCTGCGACTCTATTCTTCGTCGCC

ATCGGCTATGATGGCTGGAACTGTAAGGGTGGCATTCTGGCTGAGGAGTG

TCAAAAGGTTGGTGCCTACAGGCTGACAGGTATTCTCCTCTTAGCAGCCG

GTTCGGTTGTTTCCCTTGCAGGCATCTTCCTCATCTTCCTAACTGTATGT

AAATGCGCCTGGAGTGCGACAGTAGCCTGTATACTGGCTGTCGTCTCCGC

TGGTCTCTCCATCACTAAATATGGTCTTTCTATACNAACACACTCAACTT

ATTGGTCGCCCTTTCATAGCTTACCGCTTGGCAACGGGCCTTCCATTGAC

TTGCTCTGTCTGCGCACTCTCATTCCTTCGATCTGGCCAGCCAATAACTG

AATTCCTCAAGCATT

>TSCG.R78.esd 463 0 463 ESD GOOD: 99-431

GCACGAGGGAGAAGCTGACGCAATCACGACCAGTGAATTGAAATTGGCTC

CAGTCGAAATCCAAACCCACATTGCGAACACGATACTGCACACTAAGGTG

CGTCAATCGATCCAGTTTTGCGATGACCTGACCCAAATCCAAGTGGTCTA

CCGCGGTCGTGATCTCGCCCTCCTCATCGTCTTCACTGCCACCATCGCCG

CCAGTGCCCTCGTTTCCACCCTCCTCTACCCCACCAGCAAACGCTCCCTC

TCCACCACCAATACCACCACCCTCTTCATCGTCTTGTTCGTCAATTTCGT

CGCTATAGTCGTCGGAAAAAAAAAAAAAAAAAA

>TSCG.R16.esd 457 0 457 ESD GOOD: 98-457

GCACGAGGGGCAAAAGACGCTCCTCAGAAGACGAGGACATCCAGACGGAA

GGGGCTGTGGTGCCCCTAAAAGAAAGGCCCAACTCTCTTGAAGGCCCCTC

CTTGGAGAAATCGCCTTGTCTGGAGAAGATTAGCCGAAATTACTCGGTTT

CTCCAGCTTCAGCGGAAATCAGTCCAGCTCAAGGGCTTAATCAGCTGAGT

CTACAGGCTACCAGCAATACGACCATCTCCTATGGCGAGGATTCCTGTCC

AGGTTGTCAACGCTGTTCCATGTGGATGCAGCAGCAAGAGCAGTTGCGGC

CGAACACCTCGAAATTCCAACTGTGTGAGCCCGTCCAGCAATGTTAAAGG

TCATTCCTNN

>TSAJ.R19.esd 678 0 678 ESD GOOD: 100-575

GCCGAGGGAGACGAATGATGGGGATGATGATGATGACGAAGACGCAACGC

AACGGATATCTCTCAAATCTGCAGTAGGTGGTCCGCGAGATGTTGAAGAA

AGGGAGGAGGAAGATGAGATAAAACTCTCGGTAGAATGCCTTCAAGAAGA

GGCAGCAAAACTAAGGGCGGAGTTGGGAACTGGAGAGGATGATAAGTCAG

AGGCCGGTAGTGACTGGGATAAATGGTCGTAGTTCAAAGCTTCGAAAACA

TGCGTGTTCGTTGTTCACGAATTTCCCTCACTATTGCCCTTCATTAATGT

GCAAGGAAAAATTGCACATCCTTTGATATTCAGATTTACTTTCCCTGCCT

CTTCACTGTTAACAAAAAGACACTCACACCTCCCATCGTCCTTACCTTTT

CACCCCCAGTTTTAAATTTCCATCGTGCATTGATGTCTTTTTCTACATTA

TTATGGGACCTGTCAAAAAAAAAAAA

>TSAA.R54.esd 593 0 593 ESD GOOD: 111-515

GCACGAGGGTAGGCCGGATAAGATTTTTATCTATGCTTGGGGTTTGTTTT

TGCTGGACAATTTCTGATTTGTCTGAATTGGCCTCTCATTTCAGATATGA

CAATGTCTGTTGTAGTTCCCACTCGCCGGGCGACTGCTAATGCATTTCAG

ATGCTCATGACGCACGCCTTTGGTGATGCCATCAGTCCCTTCATTATCGG

CATCATCGCTGATGCTCAAGCAAACGTCTGATTCGGACGATGTCGCGTTA

CTTGGGCATTGCAACGGTGCCCTTTTTACTACCGTTTTCATTTGCATCCT

AATCTGGATTCCTTATCCTTTGTGCTTTCTGGTACTTGGAGCCAGCGAAG

GGGCGCGCGTCAATTCATCATTGATGCCTTCACAGTGGGACACTTCGATA

TTCAG

>TSCE.R62.esd 644 0 644 ESD GOOD: 97-585

GCACGAGGGCCGGTCCAATCTAAAGGCAACCTGGGACTTAGTTTCGCAGC

TTATGCCGATCCTGCGGACCACAGACAATCATGTCATGGCGACCAGCAAT

CCAACCCCGGCCAAGCGCCCTAAAAAGGAGTCAAGCATCGAGTCTAACGC

GCTACCTGGACAAGCACATTCGTGTTAAATTCACCGGTGGTCGGGAGGCT

ACAGGCGTCTTGAAGGGTTGCGAGAACCTTCAAAACATGGTACTCGACAA

CACAATTGAATATCTCCGAGACCCTGCTGATCCTACCCGTCTCACCGAGG

ATACTCGCGAACTTGGTCTGGTCGTCTGTCGTGGCCCTTCTGTGGAACTG

GTCTGCCCGGCCGAAGGAATGGAGGTCATCTCGAACCCGTTTGTGGAAGC

GGAATAATACGCGACTTTTATGAAGTTTTACATTCCAAGCCGACACTTCA

TGCCTGTGTATAAAATGTATGCCTTCTGAAATCTAAAAA

>TSBZ.R67.esd 572 0 572 ESD GOOD: 100-456

GCACGAGGGAAAATCTCATGCATCCCCCGTATAGCACAAAACTAAGTGAT

TTTTTTCTGGTTTGAACACTGCATCTGCCGTAAATAAACCGGAATTTTCT

TTCAGTTATGTGAAAATGAGTGCTAGCACACACAGTTAGCCAATTTTGAG

AGTTATGTATGTGTAGAAATTATTTTTCTCTAACCTTTTCAAACGTGAAT

CCTTTCTTACCCTAGCGCATAGGGCTGCGCATTTTGGGAATTCCTAAATG

GAGAATTTGGGAATATCTTGAAGTATAGACAGGTTGAAAACGTTCTTAAC

TCTAGTGCTTTACAAACGTTTGTGCACTAGGTATTTGCACATAAACAATG

TTAGGAA

>TSCG.R50.esd 602 0 602 ESD GOOD: 99-465

GCACGAGGCTCCCGCTTTCTGTGTGCATTTGAAGTGACCATGTTCTGACC

CCTCCTCCTCCGCCTCTTCGCACCCTTTCCTCCCTCTCTCCATCCATGCT

CCGAGTGTGTTTTGCGGCAATGTCTCCCTGCCTTCCACCACTTTTGTCCC

GTTAAGTCTGCGCTGCTCCACCCTCCCTCTCCTCCTCTTCCACCTCACAC

ACACCCATGCCTATCACTGCCCTGTTTTACTTCATCAATTCTTGTCTGTC

TCTTTGCCACCCTCACTCACACATTTTCTTCTTTCTCTCTTCATTTATGC

GAGTATTTCACTTCTTCAATAGAAGTAAATCATACTGTCGTGTGAAAAAA

AAAAAAAAAAAAAAAAA

>TSCH.R88.esd 611 0 611 ESD GOOD: 97-533

GCACGAGGTTTTCATTTACTCTTTTAAATCAACCCAGCTTTTACGCCCAT

AAACTCAGCGACAGTACCGGAAGCGTGGATCGGTGACTTGAGACAATTCC

CTACTTTGATCAATTATTCTGTTCCTCAATTTCACGCCCAAGAAGGCTTA

ATAAAAAATGAGTAGCATCTCTTCCAGGAAGAATCATTCGGTTTTTTATT

ACCACTGCCTTAAGGCTGAGGTATTTTCCCAAAAAGTCGCTAATATTTTC

TAAAAAGGGGAAAGTGGCAAAAAACATGCCATCTTATGGGTTGTCATTTC

ACTTTTTTGTCAGGAAAAAAAAGACTAGTTGGGTCCCATCCCATCGCATA

AACAAATGATGGATCTCATCCACCCATTGAACATATAATTAACCATCGAC

AAAATATTTTTATTTATTTCTATTAACACTATAACAA

>TSAK.R14.esd 717 0 717 ESD GOOD: 88-487

GCACGAGGGTCAGCTCCCATCTGGCGCAAGAAGGTTCTTTCCTCGGGTTG

TCGTGCCATGGTTGGTCTTGTTGCCGGTGGTGGACGTATTGACAAGCCCA

TTCTAAAGGCAGGTCGTGCCTATCACAAGTACAAAGCTAAGCGGAACTGT

TGGCCGCACGTACGTGGTGTCTGTATGAACCCCGTCGAACATCCTCATGG

TGGTGGTAATCATCAACATATTGGCAAGCCGTCAACCATCCGTAGAGATG

CCTCCCACGGTCGGAAGGTCGGTCTTATTGCTGCTAGACGTACTGGTCGC

ATTCGTGGTACTCGTGTCATTGTTGCGAAGTCCGATAAGGAGTAAATGAA

TAAAGTGTACGCGTGGATACATTGTTTTATGCGGTAAACTGTGTTATCAC

>TSBA.R34.esd 537 0 537 ESD GOOD: 98-537

GCACGAGGGTTGGCGTTGNCTAGTTCGTTGTAGCGTTCACCGTTGACGTC

ACCGCGTCAAGCGTACACCCTTTCCATGGGACTGGCTCTGTTGGCACTTC

CTTTTCTGCCTGCATCCAACCTCTTCTTTTATGTCGGGTTCGTGGTGGCG

GAGCGTGTGCTGTATGTGCCCAGCGCGGGATTCTGTCTCATTTTGGGGTT

GGGATTTCAGTACCTTCTTCAAAAATGCCGTCAATGTCGGCAGGCTGGTC

TATTTCGCTTGAAAGCTCTTGCCTATCTTCTACTGGCCATCGTTTGTTCG

AGGATTTTCCGCTGAAAAACGTTCTATCGCAACTACGATTGGGAAAACTT

AATGGTAGAGAAAAATCCTGGAAGGACGAGTACTCCTTGTTCACCTCAGC

TCTAAAGGTGAATCACCGTAATGCAAAGCTCTGGAACNNN

>TSBA.R38.esd 627 0 627 ESD GOOD: 94-624

GGCACGAGGGGGGATTGGGAGCGAGCTGAATGGTTCTATTTTTACACTTT

GTTGTAAATGTAACAGAAGGGTTCCCTTGGTTTGGGCGGTCACTTGGTAA

TTCATGTCAACGCCATTTTGACTTTCAGCCCACTCTGTAACGCAGTATTA

TATTGTTCTGAGCATTGTCGACAGCAAGATATGGAAGGTATGTTTAATCC

ACAACATTCACATAAATTGTGGTGTAAATTTATGAAGATATTCATGGAAT

ACAACGTTTCTCTTGCGAATTTCCCCTTCCAGTATGCCCGTCGTATGTAT

CATTTTATCTACTCTTATTCTTGCAGAAACGACATCTTTGTTCTTTTCTC

GGTCTGAATTATTCACTCTTTTACAAAAGCACGGCGTGTTTGTGACGGGG

TTGTGGAAGTATCTGTTTAACACATTTGGCCAAGGTCCAGGGGATGGTTT

AAAGGAATGGGCTGGATTGATATACGATTCATGCTCACTTGAACAACTTG

AACCTGACAAGTTTTTTCAGAATCCTCAGGN

>TSBA.R33.esd 583 0 583 ESD GOOD: 124-535

GCACGAGGGAACCTCCAGACAGGAAGTGGCAATATCTCCTGTTTGCTGCT

GAACCTTATGAAACCATCGCCTTCAAAGTTCGTAGCCGGGAGGTTGACAA

GGATCCAAAGAAGTTCTGGACTTACTGGAATCCCGCTTCGAAGCAGTTCT

TCCTTCAGTTCGCCTACAAACTTGAAAATCAATCGACTTCATCTGCGATG

ATTCGTGAGCTGGAGATGCAGAAGGCGCGCGCTCAGCAGGCACAGCTACA

GCTTCAGTTGCCCCCTCCACCGCCGCCTCCACCTCCTCCACCGGCGATTC

CAGCCTTCGGAACAGCACCCTTTTGGGTATGTCTCAGGCAGCGCAGCCGC

CACCGGCTCCANCACCATCGCTCACTGCCATCGGAGTGGGAGTACCACAG

GCCCCGCCTCCA

>TSAP.R2.esd 806 0 806 ESD GOOD: 90-584

GCACGAGGGGCCTTGACCGTGCCCTGTGTTACATGAGCGAAGGCGAGGAG

GCTCGAGTTAGAATTTGCGGACCAATGGCCTTCTCATCTTGCGAAAGTAC

GAAACGCGGTATACCCAAGGATTCTACTTTGTATTATCTTGTAAAGGTTA

AATCCTGTGAACAGAGGAAGAGTGTGACCGTCTTTTCAACCTTTCCTGAA

AAGATGTCACACATGCAGTCCCTAAAGGAAAAAGCTAATGAACTTCTCAA

GGCTGGGAAATATCGAATAGCAGCTGACATGTACTCGGATCTGCTAAGTG

ACTGCCTAGGAGTCGCAACCAGCGGCTATGATCAGTACGCGGAATTGAAT

AACTTGAAGTGCGTTCTACATCAGAACAGAGCGTATGCATTTCTAAAGTT

GAAATCACCTCAGGAATGCCTTGAGGCATGTAAGCAGGGTCTCGAACTTA

ATTCGCGCAATGAGAAGTGCTCTTCAGGAAAGGCGAGGCGTATCT

>TSAO.R88.esd 655 0 655 ESD GOOD: 116-552

GCACGAGGGCTGCACGCACGCATGCAAAACTAGAGGAGGCTCTTCAGCTA

GGTGGATTTGGTTATCCGACTATGGTTGCGATCAACATGAGGAAACAGAA

ATTCGCCGTGATGCACGGCTCCTTCTCAGCCGATGGATTGCGCGACTTTC

TATACGACTTGAGCCAGGGCCGAGGCTCCGTGTCGCTCAACTCCATGTCT

GCCTTACCCGAAATTCAGTCCTCCGTACCTTGGGACGGAAAAGACGCTCC

CGTAAGGATATTCACACATTGTAGTCTTGCAGTCCAATCTAGCAATGAAA

ATTACGTTGCACAACACATCATGCTAACTGATAGAGCATCAAACGCTGTA

CCTCTTTCGCGGAGAATTGTGCGTGGCCACAGGTCAATGCTCTTCTCGGC

AATAATGTAGGCTTATGCGGGTGGGAGACTATTGCGT

>TSAS.R83.esd 559 0 559 ESD GOOD: 98-559

GCACGAGGGTTACCTCGTCAAGTGCGACTACATGGAAGGCAGATTTTCAA

TGTCCGGGTCACTCAAGCTTCAAAAGCAATGAGATAAAGCAGCAGTCGAT

GGACCAAATGTCCCAACAGCACTGTTGTTTTCCTCCGATCATGAGCGGGG

GAAGTGTTGAAATCGTAAAGGTTGGTGAAGGGCCCCCTCTCACACTAGCT

GATCTTCAAAGACACGCAGCATTCCCGTTTCCCGATGGAGACGAATGGCC

AACGCACATGCATCCAAAACGAACATCGTCAATTCAGCAAACAGGCCAAG

TGCCGATGCAAAAGTGTCCTTCCTGGAGGACATTCACCATGCACGAGTCC

AAACGTACACCTATCTCCGAGTACAGCAATTGGTCACAACGAAAGAAGTC

GGCACAGCAAGTTCATGAATTATCCCATGACTCCATAATCCATCAACCAT

CACCACCGNNCN

>TSCH.R41.esd 519 0 519 ESD GOOD: 102-468

GCACGAGGCGGCAGTTTACTTGTGTGCCTGCGACATGCGGGTTCTTAGTA

AAAGCATACACAAGTATATGTCTGGTAGTATTTCGATGATTGCTGAGAAT

GAGGAAGATATGTGGCTGGTCTACAATCTTGTTCAAGTTGGTGATAGGGT

TTGCTGTTCTACGGTGCGCAAAGTGCAGACCGAAAACGCCACTGGGAGTG

TTACCTCCAAAACCGTTCGAACCAATCTTACGATCGAAATAGAAACTATC

GNTNTTGACACACATGGTTCAGTGCTCCACCTTAAAGGCAAAAACGGGTG

TGAAAAACCAGCACGTAAAAATGGGCGCGTATCACACGCTACAAATTGGT

GTCCAGACAAATTTACC

>TSCB.R51.esd 526 0 526 ESD GOOD: 108-474

GCACGAGGCAGACTTGTGCGTCACTTGCAGACTTACTTCAGAATGGAACC

GGCTGGATCCCATGGTGTTTGGAGTCTTGATGACTTTCAATTCGTCCCGT

ACATCTGGGGGAGTAGTCAGCTTATTGGCACAGGCCTCTACGAGCCGTCA

GCTGTACCCGATCGGGAAATCGCCGAAAAGGAAGCTAATACATGCCTTCT

CTTTTCTTGCATCAACTACATCTATCAAGTAAAGACGGGCCCTTTTGCGG

AGCATTCCAGCTGCCTCCAGAGTCTCAGTGGCGTGCCGAATTGGGAGAAG

GTTAATTCAGGAATGATCAAGATGTACAAGGGAGAGGTTCTGAATAAATT

TCCAGTAGTGCAGCACT

>TSAB.R57.esd 610 0 610 ESD GOOD: 98-440

GCACGAGGCAGACACACAGCAGCATGTTCGCCTATGGACAGGACTCGTCT

GCATTCGCTGTGCTGACGGATGACGTGTCGTTACAAGGCTTCATGGATCA

CTTGAAGAAATTGGCTGTCTCATCCTCCGTCTAGCCGTCTCTCTCTCTTC

ATTTTCTCTGACATGTTCTTGTATTTCTCTAGTCCCGTTTGGCGTGAATG

TTTTCGTCTGCCTGTTTACCAGTTTCTGAACCCCTTCTTTCTCCATATCT

TCTGAATGGAAATCTAATCATATGGCACCAAAAAAAAAAAAAAAAAAAAA

CAAAAAAAAAAAAAAAAAAAAAAAAAAAAAAAAAAAAAAAAAA

>TSCB.R67.esd 723 0 723 ESD GOOD: 99-329

GCACGAGGGTTGTGTTGTGGATGTTTTGCTGACTCGGCCGGTCGCCTGGT

TGATCGCATTGCTGGCACGCATGGTGTCGTGGCACCGTTGTCACCGCCGC

AGTCGCGCCAGACGATGGGTCGGACGAGTGCTGAGCGGNAAAAAAAAAAA

AAAAAAGAAAAAAAAAAAAAAGAAAAAAAAAAAAAAAAAAAAAAAAAAAA

AAAAAAAAAAAAAAAAAAAAAAAAACCCCAA

>TSAZ.R57.esd 576 0 576 ESD GOOD: 92-546

GCACGAGGAGGACAATTGCATTGTCTGTGCCATTTTTCTGTTTCCATCTT

ATTGCGAGACATATTGTCCAGCATTGCGTTTGTTGTCCGGTAAGGCACAT

TGTGCGGGAGGTTAATTCGTGCTGCATTCGCTAGGAAGCTGAAACTGCTC

TCACGGTAGTGCGTCGATCTTGTGTCAGGAAAAGGCGTCGGAAACGTGGT

GCAGAAGCTCTGCCAGACAGACCAGTTGAGGTGAAAACAGACGATGGTGT

AATGTGAGCCACACACCTTAGTGTACCTACTTAGTACCGTCGTCGGAGGA

CGTAAACTACCGACTCGTGTGTACGACAGCTTCCTTTCGCCTTTCTGCTA

AACCTATAGCCAATGATAGAATTTGTCCAGCAATGCGGTGCAACAGACAT

GGCAGAAATTGACAAGACCTTCTCTAATAGAAATATTGGCAGAATATTAT

TAAGG

>TSBO.R49.esd 424 0 424 ESD GOOD: 93-409

GCACGAGGGGAGAATGAGGCGTTACACANGTTACNAGCAGATTTCCCCTC

TCGTCGTGATGCAGATGTACGGTTCATTCAACCCTCCTCATCCTCCGTCC

CTCGAAATCGCACTAAGAGTCGTACAAGGTTCTCCAGTGAAAAAACGCAG

GCGACGACGTCCCAGAGATCAAGCACGACTCATCAGCCACCTGTCCACAT

TCGGCTCTCTATGTCACCGGGTGTCTATCATCCCACGGCGAATGGGAGTG

CCCTTTTGGAGAATCCGACTAACCTGATTGCCAGCACGCCAGTGAGTACA

ACTCGCAAGGCTAACTC

>TSAR.R75.esd 568 0 568 ESD GOOD: 103-440

GCACGAGGCGACTCCTTCATTGGAGACCGCTCGCGCACCTGCATGATAGC

CATGATCTCGCCCGGCATCGCCTCCTGCGAACACACTCTCAATACCCTTC

GCTATGCTGATCGTGTAAAGGAACTGGGTCCGGCAAATACGAATGGTTTA

AATCGAAACGCTGCATCACTTGCCTCCATTCCACGTGGCGTTGGTGGTGG

CATGGCGATGGGTGATGGGAAGTTATGCGGCCATACCCTCATCTCGCGGT

CCCGTGGTCAATCGCGTAAGTGGCCGTGGCGGGNTTGANTCCGGTGGTCT

ACTCTACTCTTCCTATAANCGGGGGCTCTTCTCTCCGT

>TSBN.R12.esd 559 0 559 ESD GOOD: 86-455

GCACGAGGGTTTCTTTGGATTTGCTTTCTTCGTTTTAATGACCCCGCTAA

GTTGACTTCATACACTATTTCGTTCGAATTTTGCATCGATTTGCTTTTTA

CCCCAACTTAAGTGAAAGTGAGATGCCAGAATAAGACTTATGCGATAAAA

TTGCCTGGTCCTCTCAAAAAAACAATGACCACTAAACTCCGGCAACACAA

ACTTCTCCTTACCGACCTTTATTGGGATATTCAAGCTAATAGGAGCTGCG

CGTATCTTCAAAATGCGACAGAAAGTTAATCTTGATTTGATTCTTTTTTT

GTCGAATTGAACCCCTAGACTTCAAGATTGGATGCGAGGAAAATTTAAAT

AAAGGTAAACAATAAGAATG

>TSAM.R57.esd 372 0 372 ESD GOOD: 99-370

GCACGAGGCCAGATTCTCGTTCCAGACTCACCGCCCCTCCCTTTGGAAAA

CACAGAGTCCCTTGAAGACGGCGCAGATAAACTTCTAGGCGATGTCCTGG

AAGCAGCTATCTTGGGCGACTCCTTTAAATTGTGACAAAGCGAAGTTGGC

CTTGGTGTTTGCTTGTATCATAGTGTACATAGTATTGTGTATAGAGTGAT

GGAAGAGCGGCTGACGTATGATCTTCACAAATCGCCTTCTGACGTCTTTT

TTTCTCGCAACTTAGGCACGNN

>TSBN.R63.esd 513 0 513 ESD GOOD: 103-204

GCACGAGGGGGTCATGCCTGTTACTCAAAAGAAGGTTATTAAAGCCAAAA

AGATAAAGGAGATTAAGACACAGGATGTGCCTGTACTTCCTGAAAAGGGC

AC

>TSAC.R53.esd 477 0 477 ESD GOOD: 109-477

GCACGAGGTAGCCTTCACTATGAGTTTGAGAAGAATGTGCGGTCTTTTGC

GTTTTCCATCTCATTTAAAAAAAATGAACAAAGATTTGCGTTTTGGAATA

ATGACTGTTGTGGGCGTTACACTTATCCACCACACATTCGGGTTTTTGTA

CGCAAGTAGCGATTTGGTTCCATATCTCCAGGACTATTTTAACGCTTCAT

CCGGCATAATAACCTTTTATACCAGCTCTCTTTTTGCCTGCGAGGCGATA

TCCACACCTCTGGGGGCCATGCTGTCCAAAATAATTGGTTTTCGTTGGTC

CACTTTTATCTCGGTATTACTAACTAGTGGTGGTCTTTCTTCTATCGCGA

TTAACATTGAACTATGGNN

>TSBV.R92.esd 575 0 575 ESD GOOD: 102-513

GCACGAGGAGATGATCCAAAATCAGCAGGTGTGCGTCGATGGGTTGACCA

AGTATGCACATCTTCTACAAGTCATCGATGAAATGGGACGGGATATAAAG

CCTACTTACGTCAACAACAAAAATGCAGCTGAAAGGTTAAAGAAGAATAT

TCATTCTGCTCGTATTCTAGTTCGTGATTGTGTAGCAGAGCTGGAAAGGG

TTATGAAAAGTTGAATGTGATATTTTAATAAATGTTTTGTACCTCTCCAC

TCCACCGCTAAGTTGCCGTGTTTTATTTTGGTGCTGGCGATGGTGACGTT

TCACACATTCATCCTCTCTTTCCCCTCCCCTTCCTCTCACTCTTCTCACA

GGCACCTCAGTGCGATTTGACATTGTCACCCTGTCCCTAACTCTGAGCCG

TTGTGCAAGATA

>TSBV.R21.esd 556 0 556 ESD GOOD: 107-440

GCACGAGGCTAAAGCCTCTCCTCGATCCTATTGCCACCTACACCTCCCGG

GCAGGCTAAGTCTCCCTTACCAGCCCTTTCTCTTGACCCCNGAATGTACA

CATAACCCTCTCACATGAAATATCTAACACTTGATTGGTTGTGCGTGTGA

TTTGGGTGCGTTTACCCCACTTGACGCACGCGGGCGCGTGTACACATACT

TGAATGCATGCGTGTGAAATCTATTTACCCCTCTCCATTTTTGGATTAGG

TAAGCCGAAATTTGAAATAAAACCAACCTGTTTGCTAAAAAAAAAAAAAA

AAAAAAAAAAAAAAAAAAAAAAAAAAAAAAAAAT

>TSBR.R43.esd 686 0 686 ESD GOOD: 100-605

GCACGAGGGCAGAATCATCACCATGTTTTCACCACTTGGCACCCTGGTAA

TTCGCGTTCCTTTGCGCACAATCAGAGATTGCAAATAAGATGCTCTCCTA

TCCATCCAGGCTGTACAATTCATAGCATAAACAAACAAACACTAGAGCTG

TATTTGATTTAGGAATAGATGCGGCAGAGAACAGTCAGGCGATACCAGAG

CTTGCATCACTTTGTAAACCCAAGCCATCGCAGAAGCAATATTTTACTCC

TCTTGTGATTTCTGTTGAATTGTACTCGAAGTGAGACCACGAATTTAGCC

CATTACAGCGGCCAGCAATGAGAATGGACAGGAGTGGAATGGAGAGACTG

AATTCGATGCATGTGAATATGCTAGCGGCCTCTGATACGATTCGCAGATT

GCTTTGAGACATTGACAAACGGTTGTTAACACTTGAGTCAAGCGAAAGTA

GAGACTTGCTTCTAGAAGCACAATACCGTTTAAAAAAGTGGATAAAGAAT

ATACAG

>TSAG.R17.esd 622 0 622 ESD GOOD: 91-426

ATTCGGCACGAGGGTTGGTTAGGTGCCTAGGGGGCTGTCGAGTCGTGTTT

GATTCAACGGAATGAGTATTCAACTGGGTTGCCACTNGATGAGGCCTTCA

AAGAAGCATTCAAAAGTGTATCCAACGATTCAAACCGCCTTATTGTTTGG

ATTTCTTTGCCACCTGGTGCGGTCCTTGCACTGCAATCGAACCTGAGGTT

GCCCGAGCTGAGTAGCGATTTCCCTCCGTTAGTTTCATCCCAAGTTTGGA

CGTTGACACCATGCTCGGAGTGTGTTGCTAATTTGGGGCGTCGTTCAAAT

TCCCAACTTTTGTCTTTTCTCCGTGGGTGGCAAACT

>TSCA.R22.esd 628 0 628 ESD GOOD: 95-627

GCACGAGGCTACCTTGGGAGTACACGGAAGCTGCAAGCATTGAGGGGATG

ATTGATGTCTCATCTGAATTCACTTAACATGCACATTGTACACTACGTGA

CACGTCAATTACGGACATCCGAAAGTTATGTTGGGGAGTCATCTCAGAAA

GTCCTAATGATCTACTTTTCAAGATGTCTCCTCATCCTCTCTCGATAAAA

TGCCAATGTTTGTAGCAATTAATCCCCTTCTCTGAAGTGACACTCGAGTC

GACGAGGAAAGTGTAATCCGCTGCTTTGACTCACATGTTAATAACTTTGC

CGTCGAGTCAGAATCATCATTATTATCATGCATACCTGCCAAAATGCACA

TAAGCGTTTTTGAAACGAGATGCAGTGATTAACATGATCATGACCGGTCC

CATTTGCACGAGCATTCCTATTCGCTTTAACTCATGATTGAAAAATTGCA

TTACTGAATAAGGAAATTATTTTGGATTGGGATTAAAGGCTGGATAATTT

AGTATAGAGAATGAATTATGACCGCAAAATTNN

>TSBZ.R96.esd 792 0 792 ESD GOOD: 102-632

GCACGAGGGAAGGTGGATGAGACGATATTGGACGTGTTTGTACCTTGCAA

TGCACCGGCGGCTCCCAGCAGCATCTCCTGGACAGCAGAGCATCTAGTGC

ATCGCTTATTGCCTCCTTCTTCCTTCAATCGTCCTCTTACTGACGCTATT

TCCAGCCATCTCCGCAGACAACTCGACGAGGGATCAATTTCATCTTGTCT

TTCTGGATGCTGATTGTCGACCTTTGTTTGTGACCGCACAGCGGGGTTTT

ATGGACGCATTTGAGTTTGATACTGTGATGTCGGAACGTCGTCAAACACG

CCATCCTCCTCGCCGGTTGGCATCGTCCATTGTTCGCTCTGGTCATCCCC

TCCCCATCATGGAGACATTTGATCTCCCAGATCCTGGTATCAGTACCGAC

ACAGATGACCAGTGCATATAGGCATGTTCGTGTGCAGCGAGTCCTAATTT

TTACACAATAAATTCTGTGGCAATGGGGTAAAAAAAAAAAACAACAAACA

GAAACAAAAAAAAAAAAAAAACACACAAAAA

>TSCE.R5.esd 605 0 605 ESD GOOD: 93-561

GCACGAGGGTTTGATCTACCTCATCACTGGCGTACCCTTAGAAAAACTGG

CTAAAGTCAACGCCATCGTACGGGGCAAAGTCAACCTTCCTCAGTCTTTA

TTCACCGAACAACCCCTCACAACAGAGGCTCTTCTCTCCTACCCAGCGCA

GCAGCAGCCACGACCGCCTTACCTCGGAACTGCCCACTTTGCGGGCCCTG

GTCGAACCGGCCTTCCCACGCGTCGCCACCATCCTCGCGTGATGCACCAC

TCACCGGGGTCCCTGCGGGATTCTGCACAGATCTGAGACACGTATTATTG

TAACAATCAAATTAATAAGTGTGTTCCTCTGTTTATTCACCTATGATTGT

CCCATTCGTTTGGCTATGGGTATATATAAATGCGCCGGGAGCGTGTATAC

CCATCACACATTACCTTGTGTGACATCAATGTAATCCTGCCATCATTTTT

AAAAAAAAAAAAAAAAAAA

>TSBR.R44.esd 706 0 706 ESD GOOD: 99-631

GCACGAGGCCTGCAGAAAGATTCGAGCAGCCTGTGCTTTTTGAGAATTTG

ACGCACAGTCATTTGCCTGAAATCATGTCGTCTTTGCCCTTGCAATTTGT

TTAAAGCGGGCTTGGCGCGTGCGAGCTGAAGAGACTTAGAGAAGGTGGAC

AAATAGTATGCACATACAGTACACATTCCAAGAGCCTATTAACGCCCAAT

GCTGACAGCTTCTGCTCTGCCACCGTTGATCAAATATCGCCTTCGATGCC

ATTATTGTTTGGATGGGCAGCTCCGAGTAAATGACACATAAATTCCCATA

TAAAATATGGTCGTCCCAGTGGTAACGAAGTAGTCTCAACATTCGTCAAA

CCTCACGTTAGAATGGTGGTGGCTACGCTGTTTACGGCGACTACGGTTAG

ATGCGGTGGTCATTCCTGTCAGTGAAAAGCTGCGCTAGGGATTAGTGCAA

TCCCTTCAGCTATTCATGCATTCATGATGGTCTTCAATCAGCCAGCTGTG

CTTTAGCTCACATACTCCACTATGATAGTTGCT

>TSBF.R88.esd 463 0 463 ESD GOOD: 89-463

GCACGAGGCCAACGCGAAGGGATCTGTGTCTTTATGCTATTAAGGCAATT

TTGCTTCTCGACAACGAAGGGAAGCGGATAATTGTCAAGTATTATGATAA

TACATTCCCGTCCCTGAAGGCACAATTGGATTTTGAAAAGAAGTTGTTTT

CTAAGACTAACAAGAGTGCATATGCTGAAATCACTCTCTTTGAGGGGACT

ACATGTGTCCATCGTACAACCTCGGGATATTTATTTCTACATAATCGGTG

ACGTTAATGAAAATGAGCTCATGCTCATGAATGCGCTCACCTGCTTGCAT

GACTCAATCACGCAAATTCTCCGAAATAACCTGGAAAAGAAAACCCTTCT

TGACAACCTCGATCTTATCTACCNN

>TSBF.R5.esd 588 0 588 ESD GOOD: 105-309

GCACGAGGATGGTATTAGAGTGCCTTTGCACGCGTGTAGTGTCGTTTTTA

ATCTGCTTAATATATGGGCATAGTCCAACTTTCTTTCCCCTAAAAAAAAA

AAAAAAAAAAAAAAAAAAAAAAAAAAAAAAAAAAAAAAAAAAAAAAAAAA

AAAAAAAAAAAAAAAAAAAAAAAAAAAAAAAAAAAAAAAAAAAAAAAAAA

AAAAA

>TSAV.R81.esd 775 0 775 ESD GOOD: 104-429

CGTCAGTCTGCTGTTTTGTGTACTGCTGGCCGCACTTCACATAATATTAC

ACCAAACCTCAGACGTCCACTATTCACTCGCCTCAACATCACTACGATAC

TTAGCATCTACTTACTCGTCGATTCACCCCACTGCATCCTCACTAAGGCG

GTGGTTGGTGCTTCATTATCCTACTCGCCTTGTTTTATTGAAGGCTGTAA

ATATAAAAAAACACGCTTTGCAAGTCAAAAAAAAAAAAAAAAAAAAAAAA

AAAAAAAAAAAAAAAAAAAAAAAAAAAAAAAAAAAAAAAAAAAAAAAAAA

AAAAAAAAAAAAAAAAAAAAAAAAAA

>TSAR.R80.esd 686 0 686 ESD GOOD: 99-498

GCACGAGGCTCAAACTTACAATTTCGAAGTCGAAATGACCTGCGAGGGGT

GTGCCAATGCAGTGAAGCGAGTGTTGTCGAAGCTCGGAAGCGATGTTTCT

TCTGTACACACAGATGTTGACAAAAACACCGTCACAGTGACTTCCACTCT

TCCTGAGAAAACTATACTGGAAACGCTTCTAAAGACTTCAAAACCTGTGA

ACGTCATCCACTAAATGCTGATATTTTGTTTTGGTTTTTGTGTCTTTTTC

ATAAATTAGACTTATTTGTTTCTTGTTGTTAGGTCTTGTGCTCTCTTATG

TTGCCTTTGTACTTTTATCTCGATTAGCTTATTTTTATTGCAACGAAGTG

TTTTGAAGCGGTTTAAAAAAAAAAAAAAAAAAAAAAAAAAAAAAAAAAAA

>TSBA.R30.esd 372 0 372 ESD GOOD: 98-263

GCACGAGGCTCCAGCCAAGGGACCTCGGCAGAATTACGATGCTCGTAGCT

TTAACGAGGCGATGGCAATGCAATACGGTGGTTGGTGTGGTATGGGTATT

CCCTCTTCCGGGAGCAGCTACGGAACACGTCGCAGCAACTTTGCGAATAA

TAGCTACCCACAGGAG

>TSAU.R30.esd 663 0 663 ESD GOOD: 88-439

CGAGTGTGTTCCTTTCCTGTTCTAGATCGTTTGCTACCGGATGGTCCTCC

TAGCGACTGGTGGTTCGGAGGTGGATGAAATGTAAAGGGCATTTGATCGG

TGGGAGCTGACCAGGAATGAAATCTCGATCACGTGTTGGTGGATCTCGCA

TTTGCAACGGTGGCGAATTCTGTCTTGACAATTGGAGAATGGCAGAGATG

AGTCGGTAGATGCGTTGGGCAAGTCCGAATTCTTGTGTTGACCTGGTTGG

GGAATCTGGGCATGCACGAACTTGTGAAAATTACCCTTGGATTAAATTTC

CTTGTCAGTGCTAGCTTTTTAACCGGTGTATTTGGCTTCCCAAAAAAAAA

AA

>TSAJ.R32.esd 660 0 660 ESD GOOD: 123-624

GCACGAGGAGATCCGCCGTCTTCCTGACTAGACTCACCTCGATAGGCTGT

TTTAATTTATCCTTATTTATGATTGTCATGACAACCGTATTCTGGCTAAA

TTAAAAAATCTATCAATTTTTCTTGTTTACCAGATGGTGGGATGTACTAA

GACCTGCTGATAGGAAAATTGGACGATTTATTCTGCGAAATGAAAAGTTC

TTTTAAAATTTTGGCTATAATGGTAGGCTGCTGGAAAGTATGCTATGCCG

CTGCTTCTTCACAATGGAAGCTCTACCCACCACCACCGCAGCCAGCTGCT

CGCTCTTCCCTACGGGGATAGAAAGTGCTTCACCTTTTTATCACCCTTTC

AACGCAACTCTCTACCGCATCTAAACGTCATAAATTGGGACGATGGTTGG

GATCCGAGATTGGACTTTTTAAAAAACCTTGTACAAAAAGTGAAAACTTA

GGAGTCGGTTCTACATCCAAAAGTGGGCTTTCCGGTGGATAGCAACATTT

TG

>TSAV.R6.esd 277 0 277 ESD GOOD: 103-277

GCACGAGGCTTCGACGACAAGGTTCCTGCCCGGTATGTAAATATCGCATT

CATCGCAATCATGATGATCCGGAAACCGATAACGAGGAGAACCACGCTGA

TGAGGAAGGTGGGGCTTACGAAGACGGGGTGAACGAAGGCAGTGCCGAAC

TACCGAATTCACAGAATGTGGNNNN

>TSAI.R4.esd 531 0 531 ESD GOOD: 97-467

GCACGAGGATTGACTACAAATCCTCCTCTGATCCTGCTTGTGTTGAAAAA

GGTCTGCTAGCTGAGGAGTTTCTCGAGACCGCAGCCTCCCAACTGACCAT

GTGTGGTCTTTGTGAACTCTCTGAACACCTCCAGGAGAACCAACTGGCAG

TCCTCTTTCGCAACAATCACTTCAACACCATCTACAAGAAGGCGGTGGGT

CTTGGTTTAACTAATGCACTACAGCAGCGGACCATGCTGCTTATCGATCT

GGGTTTCTAGAATGACTTAGAAACCACAGCGTTGTTTCATGTTGGCCCTA

CCGACATAAAGTGACTTTTAGGACTAACTTTATTTTTTGCTTCCACCGAA

AAAATCACAATATTTTCTTTC

>TSBL.R26.esd 587 0 587 ESD GOOD: 92-568

GCACGAGGGGAAATTGTAAATTTGCGCATCTAAAGTCAAGTTCGGGGGAA

ATGGAGTACGATCCACCTCGCGGAACGTTTGAAGTCCTAGTCACTTGTCG

GGCGTAAACTAGTGAATCGCAAAACAAGCCTGGAGTGTCCGGTCGGCCTG

ATTTTTTTCATCCTCTGTAGCATCTGCGAATGACTCTTAGCCCCGATGGC

CAATGCCAAGTTCAGCACCTTCCTTTCGCCTCAATCTGCGAGATGCTGGA

GCACTTTCATCAGGAACCCATCCCCCTCGAACATCCTCCGTCCTCCGCAT

CTCAGCAACAGCAGCAGCAACAACACCCACCTCCACTACCGCATTCCTCC

TCAGCGGGCGACGCGAAACCCACTGTCGATGCGGCCCCTCCGCCCGTCAC

ACTTTCAGCCTATGTGGTGAACACGCGTCGTGCGGCTACTGCTGGTCGTC

TTTGTGGCCCTCAGTGGCCGCGTCTCG

>TSCC.R47.esd 710 0 710 ESD GOOD: 111-563

GCACGAGGCTTGGAGGTTGTGATGTTTGGTTCGGTGCTCGCGTTTGGTGT

TTTAGCCTTTCTTGGCGTTGTTCTCTCAGACGACACTTCAAAAGTTATTG

TTTTAACTGAAAGCAACTTTGATGAAATAATTTCGACCCATAAATACGTT

CTTGTTGAGTTCTATGCCCCGTGGTGTGGACATTGTCAACGACTGGAACC

TGAATATAACAGGGCTTCAATATTGCTTGATGAAGAAAATTCGGAAATCA

GACTTGCCAAGGTTGATGCTACTAAAGAAACTGGTCTAGCATCGAAATAC

GAGGTTGGGGGTTATCCAACCCTTAAGCTTTTCAGGGAGGGTAGTCCTAT

AGAGTTTGACGGTGAACGGTCTGCTGAGGGTATTGTTAGCTGGCTTAAGC

GGAAAACAGGACCAGCTGTGCTTACTGTTGAGTCTGCTGATGAGTATGAA

GCA

>TSAW.R72.esd 621 0 621 ESD GOOD: 94-584

TTTCTGTGAGGGGTCATTGGGCTGCTTTGGTTTCTGTCCTTTGCGCAAAC

CGATATGGCTAAAAAAACCTACGATCTCCTATTTAAATTGATGCTAATTG

GCGACTCTGGTGTCGGCAAAACATGCATTCTTTTCCGCTTTGTGGATGAC

TCCTTTTCCTCGTCTTTTATTTCTACAATAGGGATTGATTTTAAAATAAA

AACTGTTGAAATCGACGGTAAGCGTATCAAGCTGCAGATATGGGATACCG

CTGGTCAGGAGCGTTTCCAGACCATAACCGCGTCCTACTATCGTGGTGCG

ATGGGTATAATGCTGGTCTATTCTGTGACGTGTCGCAAATCTTTTGAAAA

TATTTCAAAGTGGATGTCAAATATAACAAATTTAGCCTCAGAAGAAGTTG

AGAAAATCATCGTCGCTNATAAGACTGACAAGGTTGATCAACGTCAGGTG

TCAGAAGAGGAGGGCCTTGCTGTTGCTGAAAGATACGAAGT

>TSBN.R78.esd 629 0 629 ESD GOOD: 100-199

GCACGAGGCTCAAGCTTGCCTTGCCCAATCGCCTCAGCTTTACAAACAGA

TAGCGATTGCTGCTGATTTTGGGCGTGTGTACACCATTGGTGGCGTGTTT

>TSBY.R37.esd 671 0 671 ESD GOOD: 100-425

GCACGAGGACCGACATTTATTTGGGCAGGGTGTCAGGTCTGTGTACAGTG

TGCGTGATGAGTGGAGTGACCTCGGAGGCAGTGCTGACAGCAGCCCGTGC

AGATCCGGCCAAGGCCGCTCTCCTGTCGCCAGATTTGGTGTACCCCAGCG

TCTTGGAGATGTATCAGCAGCTGTTAGAGGAAGACAAAAAAGCAGAGTAG

CCTCCGCGGGAACCTGATTTTTAAAAACTGATTTTACCAACCCACTCACT

AATTAGGGCCTTGCTTTCAACGACAGGGAATACCAATAGCCGTCGGTATT

TCAAAAAAAAAAAAAAAAAAAAAAAA

>TSBY.R83.esd 606 0 606 ESD GOOD: 95-606

GCACGAGGGCAGACTTTGCAATCGTTTTTACCACCATTGTAACCTTCTTC

CTACCCGTGATAATAGTCCTCATGCTCAACCTCGTCATCGTGCAACAACT

CTGGAGGGTCCACCGGTGGCGTACAACGATGACAGTTGGTGGAAGAGGTG

CTCAGGAGATGGGACGCATTACCGGCCACTTGGCAATGAGCACATGCTTT

CTTCTTCTCTACCTTCCCCTTGGTATCGTCGTGCTCATGCGCCTTCATGT

GACCGTAAGCTTAGGTGAAGAAAACACACCGCGGGCCATTCGTATTATCA

ATTTGAGCAAGTTCCTCTCCTCCTTGAAGGACGTCTCTTACGCGGTCAAT

TGTTTAATCTACGCTGTCTTTCTCCCAAAATTTAGAGCTCGTCTGGCCAG

TCTTTACGGCTATCGCTGTATCCACAACTAATGTTAACAAAAAAATTAAC

AAATTCAAAAGGCTTCTAACCTCAGCTAATGGGGATGCTTCTTCCTCCAA

TCTGGACATTNC

>TSCE.R89.esd 543 0 543 ESD GOOD: 103-512

GCACGAGGTATTCATTTGCCCACTGTTTTAATACAGAATCAATACTAAAA

CTTTTTAATGTCTTTATGCTTACTGCTTAATACTTGGGAATGCTTTTTGA

AAAATTCATTTCTTTGATGCCTGCTGAACGAGCTTATTGGAAGTTTCATA

ACTTACTTTTTTAGTGTACATCACTCTGGAAAAGCGGTTTGTCGATAATG

TTACAACAGTGAGCTTGTAGAAATCAACACTAGTTTAAACTATCTTGCGT

CAATCATTATATGCGTTAATCGTACTTGCTTTTAGTGTATATACCCTTGG

TTGTGATCTGAGTAAATTTAATTTTGTGCCTTGGTTTGTTGCTCATGTCC

TGACTTAGTCAGTTTTTCTCCGGTCGTCCCATATTTCCAGGGCTGAGGGG

ATCGGGTCTT

>TSAT.R56.esd 671 0 671 ESD GOOD: 106-645

GCACGAGGGAAGAACGCGGCGCTGAAGAACAAGTCAAACAAGCCGACGCA

AACGGTGGCAGTGGGTCCTGACTTGCCGGGCGCGTTTAACGTCGGTGGAG

CGGCAGGTGCTGCCTCGGACGCCTGTGCAAAGGCCTGCGGTGGACACCTC

ACCGATGTTGTTGGTCTGCGACCTCTCAATTCTAGCATTCTTGCTAATCA

TCCCCACTTCTCCGCCTCAGCCTCTACCTCCTCCAATTCCAATTCCGTAA

CAATCACCAATGCCGCCAACGGCGAGGAGACGAAGCCTGCCTTTGTTTGA

TGGAGATAGACTTTTTCTACGCGATCACTTTTGGTCCACGTAGTCTAGTG

TGTGTGCTTGTCTATGTGTAAATAGGATACCTGCCAAACTCTGCTACCGT

CCATTCGCCTACCTGCCCACCCTCTTATCAATCCTGGCAATCAATGGACT

CCTTCTCCCCTCCCGTAATGCTTCTTCCTGCACCGATAACGATTATTAAA

CGATTCCCTTGCTCCTCACATTGTCACTCTCTGTGTGTTT

>TSBC.R48.esd 578 0 578 ESD GOOD: 98-523

GCACGAGGGATTTGTACGGGTCCAGGTTTTGCAGCTCTTTCAGAAAATCT

TCTATCTGGATTTGGGATAAGGAGAAGCTAGGGAGGCTTGTGCAGGTAGC

TGCAGGGCAAGTAGCTGCAGGGGATGCAGAGCAGTTGGCCGGGGTTCGGG

TAGCCAGGAGGAAAGCAGCCGTAGAGAAATGCTTATTGAAATTCTCTACT

ATTGTGGATTTAACGGTGGTGACAGTGTTTCCTAGCCTCAGTGCATTGAG

CAGCTGTGAGGAGGTGCTCTTATTCTCCATGGACTTTACATTGTCCCAAA

ACTTTTTGGAGTTTGAGCTACAGGATGCCAATTTCTGTTTGAAAAGCTAG

CCTATGCTTTCCTAACTGACCGTGTGTATTGGTTCCTGACTTCCCTGGAA

AAGTTGCATATCGCGGGGACTATTCG

>TSBD.R89.esd 572 0 572 ESD GOOD: 87-207

ATTCGGCACGAGGTGAACTCTCACGTACTTCAGCACCTACGCGAGCGCCA

GCTTCTCCAATGTGGAGGACCTCAAGTCGCGAGTCACCTCCTCATGGGAT

GCAGCGAACGAGAAGCTCATT

>TSBU.R90.esd 935 0 935 ESD GOOD: 101-523

GCACGAGGTTTGTGTTTACCTTCTTCTTTTTCGACAAGCTGCTTCCGTGT

ACGTATGTTCAATTACGTCTCCCCTCTTCCCACCTCTCCTTCTCCATTAT

TTAATGTAGGCATGTGTGTTTCTCTAGGCGGACCTTTCTCAAGTGAATGT

AACACTTTTTCAGGCCTTCTGGTGATATCCGCACACACAAACGCATTCAA

CTTTCGACTTTTAGGCGCGACCATTCATCATTGTACAAATACTCTACCAA

TCGAATCATTCACCCACGCGCGCATTATACTAGGCCCAATAATTATGTTA

TTCANCTTATTCGTGCATTTACTTAATCCATTCCCCCTCTATACCCTCTC

CTTGGGGGGATTGTTTTCGTCCATGCTATCCCCTCGCCCCAANAGACCAC

TGTGGGCCCCCACCACTACAANC

>TSAF.R38.esd 655 0 655 ESD GOOD: 101-616

GCACGAGGGAGGAATATGTAACATGGATGCGTAGTCGGGCTCCTGCCGAC

ATCTCGTGGGAATGTTTGAACGCAGTGCGCGAAGAGTTCCCTGCTGGTGC

GGGTTCGTCCGCAGTAACCGGCACCGCTGCCGGTAGCGGAGGCGGCGTGA

GTGGTGCTGGTGCCGCTGCCCCTGTTGTCAGCACAACTGCTGCTGCCAAC

CCCGGCGGTACTCCCAAAGCCTCCTCTCCTGCCCCTCCACCTCCAACTCA

ATCCTCGACACCGTCAGCTGCTGCCTCGTCCACATCCACTACCTCTAACT

CCGGCGTCACCGTGACCATGATCAAGCCCGCTAATGAATAACCATTGCAC

ACTATTTTTCTGCCTTGGCTACACTTTGTCTCTCTGTGTCTGTGTCAGTA

TGTCTGTCTAGTTGTTTTCTCTGTGGATTTAATCTCTTGAGTGTGTGTAC

GTGTGATCACGTGACTGGAGGTGCCCCTTTCGCCCCTCTACCCCGTGTAC

AGACACGCTGTGATGC

>TSBA.R69.esd 490 0 490 ESD GOOD: 98-490

TCAGATGACACTTGTCTGACAGAGGAATCCTCCGTCTGTGATCGTACCTT

TGAGCTTCCAGATGCGACCTTCCCTTCTCTTACTCCTGTGATTGATCGGA

AAGAACCCAACTGCGATAGAGACTTAGTTTCACCCCCTTCTCNGATGCCA

ACTGGTCGGGCNGTGGAGGCTTAGTGGATTGGNCGCTGGTATTCTGGATT

GGGTTCACNCCGACTTGGAGACATTTTTCCAAGGGTGCACTGGATTTCCC

CTGTGTTATTTGATGAGCTACGCTGCTATTGCTGGAGACTTCAGTTTGCG

AAACCAAGGTGGTTATATTCATGGCCAAATTGGGCTACTTCCTGAGGAGA

TATTGCGGCTAGTGGCTAGTTTCTGGTGTCTCCACAAAGANNA

>TSBB.R63.esd 590 0 590 ESD GOOD: 95-305

GCACGAGGGTCTGTTAGACCTCTGCATGAATGAGGATCCGGGCAAGCGAC

CGCGTTTCGACATGCTCCTCCCGCTACTCGATAGGATGCGCGAGAGAGCC

GGACAATCCACCCCGGACTAATTTGACTCTTCATCTCCCATGGACATTGG

TTACACCGGCCGGCAACAATCACGGGGCGACAGCTTTTGGCTCCCCTTCC

ATCCCTTCCTT

>TSBG.R66.esd 678 0 678 ESD GOOD: 106-415

GCACGAGGAAAGAGGTTATCACTCACGATACAACACTATACACTTTTGGT

CTGCCGTCTACCGAATATGTGCTTGGTGTTCCTGCTGGTAACTGCGTTAT

ATTGCACGCAAGAATCGGTGGCGAAGATGTTAAACGTCCATATACTCCAG

TTACTTTGGATGACTACAAAGGATATGTGAAGTTCTTAATTAAGGCCTAC

CGTAAAAATGTGAATCCCAATTTTCCTGCTGGCGGTAAAATGACCCAATA

CCTCGAGTCGCTTGATGTTGGAGACGCTATGGAAGTCAGCGGTCCTTCTG

GACTTATCCA

>TSAK.R49.esd 710 0 710 ESD GOOD: 95-588

GCACGAGGCACCGAACGACGGACTGGAAAGTTCTTGCGGCGGTCAGCTTC

GGGTGTCATGACTACCATCAAGAGTCCAGACTGTCTCTTTACACACCTAC

CACTACTACTACTACTACTACTACTACTACTACAATTAACACCGATCCTG

TTTATACGTATGTCGATGCATTTGTTTTACACTAATAATACCACTTTTGT

CTCGCAACAATAAAAAAAAAAAAAACCGGATTGACCGCGTTGACCTATTA

ATCGGTTGGCGAGACGAAAATTATTTTTGGACAATTTTTTTTTGCAGGCT

AAATTGCCGTGACTGAATGGAATGCATGTGGTTTGAGATAGCTGAGCAAT

TCCAATGCAACAATATAGTGCTACCATGTTATTAAGCTGAGGAATCTAGA

GCTGGGCCCCAAATACGACGCTTTCCCTCCTTTTCCGTCTAAATTCTTTA

TCCGACAAATCCTCTGTTACGTTTTGGGAACATATCTGAGGAGT

>TSAA.R76.esd 757 0 757 ESD GOOD: 99-207

GCACGAGGGAAGAATCTCGGCTCAAAAGTTGAAATCTAATTGGGATTAAA

ATGTACAATTGCAGTCTCTATTTTCCTCAGACCGCACTATTCACCCAGCC

CCCTTCCGT

>TSAP.R22.esd 699 0 699 ESD GOOD: 97-622

GCACGAGGAAAAATGTGGTTCCAACTCTGCCTCATTTTATTGGCAACGTT

CACCGTGTATGGACGAACTCAAATTAAAAAAGAACTTGGTGCTCCCCAGC

GTGTGAGCCTTCAAGCAGTCAACTCTACCGCGGTGCATTTGGAGTGGACT

CCACCACTTGATCCAGAGAACCTTCTTGTTGGCTACAGTATCATTTGGCA

GCTCAATGGTGTATGGGAGCATAACATCAATCTTCATTTGGTTAACAATC

ATACGATTGGAGGTTTACAGGCAGGACAAAATGTTACTGTCTACGTTAAA

GGAATGACCCAAAACGAAAAATTGTACCCAACCAAATATTATAATGAAAT

TAGCAGACAATTGGTAGTAAAGTTACCAAGTCCGGGTGAAAGAGGCGAAA

AAGTGACATATACTTCGAGGGAACCCGAAACAACTCCTGGAACCACAACG

AAAGGCACAACCTTTACGAGGTCTGTGACGACCGATGGAACTACTTCCAC

TTCTTCGGCCACTGCAGCCATATTCA

>TSCF.R43.esd 766 0 766 ESD GOOD: 111-657

GCACGAGGCTTCATGTACTTTTAACGATTGCCACTTTTTCTTTATCCAGT

TATGGCTGCAGACATAATTAACCTGGAAAAACATGAGTCCCCTCCGTATT

CTAGTCACACTTATTATATAAACTGCCTATGCTCGGGTTCTGCAACCGTA

AAATAGCCTTGCTTCAAGATATTTGGTCTTAATAATGTCGATGTCAAAAT

CCTAAAATAGTTCTTCGTTTGATTAAATACGCAACGCGGATGAAAGCACA

GTTAGCCACATGTATTTTGGCTGCAACTTACACTCTATCATCTTTGCTTA

ATTATTTCTTGTCCTTCCCTTCTCTCACTACTTACTCACTTCGCGGATCC

TGCTTCCCTTAGACAACTGATTTTCACGTCTACTGACGTCGTACCTAATG

TCTTGCCACCTCTAAAACACCTGCAGTCACTTCAACTTCCCTAGTAACCA

TAACCGCGCAAAAGTGTGGCAAGTATCTCAACGATCCGTGAACGGCAAGA

CTGCATGCGCATGATCCAGTGCCTGTTATTTTCTTATGTACTCATTA

>TSAS.R71.esd 318 0 318 ESD GOOD: 102-216

GCACGAGGCTGATGATGGCAATAGAAGCAAAAAACACTCTTCCAACGAAC

GGACGTCTGTGGCATTACCCTGATACCATGATGACAATGAATCGTGATGG

CCCATATAATGCCTC

>TSAI.R53.esd 437 0 437 ESD GOOD: 104-437

GCACGAGGATTTTTACTGCAGGATGCATCCATCCATCAAACGCCCTTACT

TCCAACAAACAGTGCAACGAATGCAAATCTTGACGCCTTTAGTCCATTGT

CCTCGATACTGGAAGAGGTTCACCCTCGTTGGGCATCAAATCGGAAGGCA

TTGAATTAAAAATGGATGAATTAATTAGCATAATGATGCTTGGTGGTGGA

GAGGAATTGAAGGCAAGCCTGTAGTACAGAGTGTTTCATCGAAGCCAGGG

AGAAAGACGTTACTGTTTTAAGTCTTCCAGAAGTTGTCTTTCTTCGTAGT

AGCTACATTGGCCGGCTCATTAGTTATCGCCGNN

>TSBN.R59.esd 487 0 487 ESD GOOD: 73-268

CGGCACGAGCTTGCCTGAAGTCCACCTCGGTCGCTTGCATCAGTGGTTCT

TCACACCTTTCGTGGATCATTGTGGCAGATCTTCTCCTTGAACTCCGTTT

TCTTGGGCCTCTCTTCGTCATCGATTTGGAGGGTGAAACGTATGATTCGG

AACTCTGCTTTTAATGAAATAAATTCCACTCTGCGTCTCTTAAAAA

>TSBC.R12.esd 863 0 863 ESD GOOD: 117-581

GCACGAGGCGATGTCTTTCAAAGTCAGACCCAGTTGTGGTCGTTTTCGAG

TTCTGTCCAGCTCTTGAGGATTGGAGGGAGATTTGGAGGTCTGAAACAAT

ACAAAACAATTTGAATCCTAACTTTTCAAGGAAGTTGGTGCTGCAGTACC

ATTTTGAGGAGCAGAAAAAATTAAAATTCGCTGTGTAATTTAGATTAAAC

CCAAATGGCATTTCTAGATACGATGTCGACGATCCTAATGAACCTTTGCG

GCATCAGGATTTTTTGGGTTCCTGTGATGCAACACTAGGAGAAATAGTTT

CAGTTGGGGAATTAGAGAGGAGTTTACGCGGTGGACCTTCAGGAAATAAA

GGAACGCTTATACTCATTGCTGAGGAAACAGTCACATGCAATGATGAAAT

CACTCTCAATATTAGTGCCAAAAATCTGGACAGGCAAGATATTATTGGGC

TTTCGGGATCCGTTT

>TSBH.R30.esd 368 0 368 ESD GOOD: 110-279

GGCTGCAGGAATTCGGCACGAGGGTCAGCTGAATCCGAAACGGCCAATAT

AAATGCCCTTGAAAAGCAGATATTGGAGACTACCAAGTTGCTGGATGCTG

CAAATTTGAAGGCGAAGAATGCTGAGGAACAAGCCCTTTTTAATCAAATG

GAGCTCGCCATGCTGGAGGA

>TSAI.R62.esd 561 0 561 ESD GOOD: 96-396

GCACGAGGGAGAGACGAAAGCGCCTCACCTGGCGAAAGTTCGTCGACCGA

GCCAACCCTGCCTCCTGAAAAACAGCACCATTGTGGTGGAAGTTCTGACC

AACTCCAAACATTTTGCACTGTTTTTATTTTTTAACACCCTACTGGTTAC

TTCCCTTCTTCCCCTTTCTTAACATCTCCGACCTTTGTATGCGCGCAGAG

CGGTGTGGCCCCATTAATTATTTCCTTCCTTTTCTTTGTACTAACCGCTT

ACTTGGATCGTCTTTTAGGTTCTTTTGAAAAAAAAAAAAAAAAAAAAAAA

A

>TSBZ.R32.esd 552 0 552 ESD GOOD: 94-467

GCACGAGGGTGGGAATGGGGCGTCGTTCATGTCTGAAGAAGCCTCGAATC

CTCTCATATCAGAAAGCAACAATCGTCTCTTGTCCACCGACGTTATGCGT

CGCATCCCAAGCGCTGGGAGCCTCAATTCGATGGATCACAATGTGCCAGA

TCATGATTTCGGTACTTTCCAGGCAGATTCTCAGGACTACTGCGATGACG

ATGAAGACGATCTCCAAATTAGTTTCGATCCCACTAAGCGATCACGTACA

CTTGACACTCCTCAACGGCTTACGGGAATTTCAATGGATCATCACAGGAT

CCATCACGGCGGATCATCATGCTACTCCCCTTTCGTGAATACATCGGACA

CTGAAGCAGTATCTCTCGGTGCCG

>TSAQ.R70.esd 628 0 628 ESD GOOD: 87-593

GCACGAGGAAAATATGCTTTGGAACTGAATTCTCTGACGATGACGACGAG

GAGGAAAGCGATGATCAGTCGCTGGAGGAAAAATACACCACCGCCACTGC

AGAAACCAACGAAAACGATCTTACTCCTTCTATGACCAGTCTACCTACTG

TGGTGTCAAAGAAATCGCTGGTGAGGGTCTGCTTTCTTTCGGCCGCGACT

TCAGAGTTGATCGGTGTAATCGAAACTGAAGATGGCATGCTATCACTACA

GTCTCTTGCAAACCTTTCGCTTCCTCAGATCCAACTTATTGTCAACGACT

TGCTCAATCAAATAAGTGAAAAAAATATTGAACTTTTGAAAGAATTACCC

ATTCGTGATGACTTAACTCTCGAAAAGGAGGAGAAAAAGGCGTATCTGGA

CAGATGCGCTGCGCAATACCGTGGGAAAAGCCATCATTCAGTGGATCGCT

ACTACACTTCGCTCCTCCGTCCGAATAACTTGACATCTGCAGATGCATTT

AACCCCA

>TSAL.R13.esd 777 0 777 ESD GOOD: 104-259

GCACGAGGCTTTTTGGTACCCTTTTCGACCTTTTTCGGTGCTACCTGCAT

CGGCAAGGCGCTTATTAAGGCGCACATGCAACAGTTTGCTGTGATCGCCG

TGAGTCCGGAGGATCATGTCAACCTTCTCGTCGAGTACATTGGGAAACTC

CCCATA

>TSAR.R77.esd 538 0 538 ESD GOOD: 113-538

GCACGAGGCACAACCCCCCTCCCGATCTGTGTCCATGGCGAGTTCTTCGG

AGCACGCGAGAGTGTCCCACCATACATCCTCAGTGGTCTCGGAAAGTCAC

AACGGTCCCTCTACCTCCTCTGACCACGACCAAGGTTCCCTGAAGGTGCA

TGGGAGATTATGGCCGAGATGGGTCGCATGGTGGGCAAACTATCTGACCA

GGTTGAACGGTTGGAGCACCGGGTGCGAAACCCTGGAGGAAAGGTACCAA

AAGGGTGATGACGCGCTCCNATTCCAGCGACTGCAGTGGGCTCCCGATCC

AGTAACCTGGAGCGGTATCTACGCATCGAGGTTGTGGCATGCACCCTCTT

TGCTAATACCGCCTCCTTCATCCTACAGCAAACCTCTTGTGTCCATTATC

TATCCACTTATCTCATCCTCACTNNN

>TSBQ.R61.esd 784 0 784 ESD GOOD: 98-334

GCACGAGGATCATTTCCCCCTTCACCGTTAAGTCGCCGCGTTGGCTCTAC

CTCAAGAAGAAGGATGAGAAAGCTGCGCGCGAAGCCTTCGCTCGCATCAA

TGGCTCCGAGAATGTGGATATGTTCATCGCCGAGATGCGTGAAGAGCTAG

AGGTAGCCCAGAACCAACCAGAGTTCAAATTTACTGAGCTCTTCCGTCGC

AGAGATCTTCGCATGGCTGTCATCATAGCGGGTCTCA

>TSCE.R1.esd 628 0 628 ESD GOOD: 98-628

GCACGAGGGTTCCGTGTTCTCGACAAGAATCGCAGAGGCGAAATCGATGT

GGACGATCTTCGATGGATCCTCAAGGAGCTGGGTGACGACTTAACTGAGG

AGGAGATCGACGAGATGATTCGCGACACAGATCGCGACGGCTCGGGATTC

GTAGACTTTGATGAATTCAAGAAGCTGATGACATCAGAATAGATAGACCC

GGCTTCCACAAACGCCTTTTTCCCTTCCTTTCTCGTCTACTTCATAACTT

CTTTCCTATTTCGCAAGAAAGCGAATATCAATTCGCCCAACACCTTACGA

TGGCTTCTCTTCATCCGCTTATTTTATATGACTCTGCAAGGCAAAGAATT

TATTTCTGGTCTTTTCGATCAACAAAAAAGAACAAAACAAAACAGACTTC

CTTTCCAACCATCTCTTGTCTGCAGATGCCCTCCTTTCTCAGCCAACTTG

ACTCGTTCGCTGATACATGATGGAAAGCATAAGTTATCTTTCCTCAAAAA

AGCAGCCCTTCTAATACTATATTCTTANNNA

>TSCC.R72.esd 746 0 746 ESD GOOD: 117-612

GCACGAGGGTCAAAATCATTGAGGTTTCATAATTGTTGAAGTCTGTGGTT

TTTTGCAATGCTTTCAGGTGCCTAACGCAGGCTTGCATCTACCCTTGCTC

TATCAACGCCTTAAATTAAGGAAAAGCGTAGGTGTGTCTTCTTAACTGAG

TGATGTTTTTACGGTCCAGCAACAGCATAATGGGCGAATTGCACTGTTAC

TGGGGGTGATCCAGTTCCATACGTTTGGAGTGGGGGCTTTTAGTAACACT

TCTAGCACCATTCCGATTGGAATCTCAACAAATGTGCTGTATAATTAACC

AGCCATCATGATCTGCATTGATCTGATGGTATTTCAGCTGAATGAGCGTA

TTTATTCAATGCACGCAGCAACAAAAATGTTTGGAAAATATCCCATTTAA

TAAAACTGTCAACCCTGTTTGGTAACAAATACAGTTCTATTGTCATTGGT

CCCATAAATTCTCTGCAATCTACTTTGTTGACCTCAAAAAAAACTC

>TSBR.R35.esd 704 0 704 ESD GOOD: 104-670

GCACGAGGGCGGCATGAGGTCGCATACTTTGTGGAACGAACAGCCGATTG

GATGAACCTCAGGCGCAACTACTTGGTTCTCAGGAGACGATCGTTTCCTA

GTGGAAAAACTGCGCGGCCCAGAAGTCTGGAATATTGAGTAGCTCACTTG

GGAACACTCCACGATATGCTCCCTCAATTGACTGCTGGTGAAACAAAAAA

AATCCCCTTCCCACCAACCCATTGATAGCTTCTGAGGCTTACTGCCACCC

GCTCATTAATACATGACGTGCAGAACTCATTTGTTTAACTTCCAAAACAA

TTCTTCCAGTTGGCGTAGGTGGACACGAATTGCTCCATCATCCACATTGA

ATTCTAATGCGAGGAATTCCTTGACACGTTAACCTTAGCTAAATCTGTTC

ATAGCAAAAGTTAACTCATCAGTTTCCAGGATCGGAAATCCTCCCGGTAG

TGCTCCNACCTCGAAAGTGTCATCACCAAAACTGTTCAAANCACCCCTGG

AGGTTATGTTCCGCTACCACATTATCCACCTTTTCCAAGTGCTAGACAAG

AAGCCATCTGAGATGCT

>TSBJ.R74.esd 579 0 579 ESD GOOD: 96-523

GCACGAGGGAAAAGCCTTGGCTCAGTGCCGCTGACCTCAATTGTAAGGTT

AATGGAGCCTCCATGGACGCTTCCAGTATCTTAGATACCGAATCCATACC

GACAGATTCAGATACAGATGTTCATTGACTTGCTGCCACTGATTCTATTC

TCAATCCTTTTCTTAATGCTCATTTGCCTGATTTCACCTGTATTGTTTTG

TCATTCCGTCGAAAGAATCCCCTCACCATTTGCTCAATGGCACCAACCCG

CTATACCTTAGGCCCAACCGATGCAGACAGATAGCTACTGCCTGTACCGA

CAATCGAGTCACGCACAGATTGGCTTTGTATGCATTTATTTATGTACCTG

AACCCTCTGTCTTTCCTCCCTTCATTTCATCACAGCTAAGTCAAGAATTT

ACACATTAAAAAAAAAAAAAAAAAAAAA

>TSBP.R20.esd 609 0 609 ESD GOOD: 91-568

GCACGAGGGATTCGCGGAGGCCTACTCGTGTTTGGGTCGATGGGTGGTAG

CTTTTGTGTCAGAATGTCTTCCTTTTAGCTTTGATATGGTGCACTTCGGA

CATGCCAATCTCGTTCGACAGGCCAAGTGTTTGGGTGATTATCTCATCGC

AGGCATCCACAGTGATGAGGAAATCACGAGGCACAAGGGGCCGCCTGTGT

TCACCCAGGAGGAGCGCTATATTCTATTGAAGGCCATTAAATGGGTTGAT

GAGGTCGTCAAAGATGCTCCTTACATGACCCAACTAGAGGTTCTACAGGA

CCATAACTGCGACTTCTGTGTTCACGGAAATGATATTTCTTTTACCGAGA

ATGGTTCCGATGCCTACGAGATCGTCAAAAAGGCCGGAAAATATGCAAAG

ATGTTTTGTCAACTAAGACGGTCAGGGTCCTTGGGACGGTACCAAACACA

CATCGAAGATTAACTGCTCTCAATGAAG

>TSAC.R56.esd 721 0 721 ESD GOOD: 101-212

GCACGAGGCATCGGAATCTCGGCTCTTCAGGNCAGCAAAGATCTTGAAGA

TGACGAGGAGGACTATGTTCCCGGCTGATGGACAAGATGATGCGGAAGAT

GACGAAGAGGAG

>TSBV.R51.esd 605 0 605 ESD GOOD: 115-487

GCACGAGGACCAAATGGCATATACCAACGGTGCTTCAGCTTAGCCATGGT

CAGAAAGAAAACGCTGGCGCTCCGCAATTCGCACAAGTATTCACCGTCTT

TTGGTTTGGTGTCATAGCTGTATCTCTCAACACAAAGCTACTTGGAGGAA

ACCTATCTTTCTGCCAAACCGTTTGCGTTCTGGGGTACTGTATCCTCCCA

TTGGTAACCGGCCTAGTAATCAACGTTGTCATCAAGCTCTCTACATCGGC

CGGCAACTGGGTCCTCCTTTATTCGGCTTCCGCGGTTGCCCTTTGTCCGG

TTTGGCCTATTCCCTCCTTTCTCGGCCGCCAACCTTTTCCTGGGCACCCC

AGTTTCGAAAATCGACGGCGAGT

>TSBQ.R74.esd 867 0 867 ESD GOOD: 97-427

GCACGAGGGCCGAGTTAACACCTGGTCGATTCCAGTCTCGATGCCCGAAT

AAACACGCGGGAAAACAGCATTTACAAAATGAAAGCAGCCCTGAAATGTA

CCCTCTCGGCGACCCCCAACTCTCACAAATCCAGTATTTCGGAATGTAAA

TAAACGCATGCCCAAAAATAAACGTCCTCCTTTGCTCTCTCTCTCCTCCT

CCTCCCCTCCCCTCTTCTTCATGTTCCTCGGCTTCAACATGTACATCGCG

CGCGCACTCCCTACTAGCCTTCTATCATAAGTGTTGGTTCCCTAAATAAA

TAATAAAATCACAAAAAAAAAAAAAAAAAAA

>TSBT.R18.esd 741 0 741 ESD GOOD: 93-281

GCACGAGGGAAACATTTACGCTTCTTATGGCGCTTAAGGCTAGGTATCCT

GATAAGATTACTCTTCTTCGCGGCAACCATGAAAGTCGTCAAATAACACA

GGTTTATGGATTTTACGATGAGTGCATTACCAAATATGGTAACGCTACAC

CCTGGAAAAATTGTTGTAAAGGCTTCGAATCTTCTCACA

>TSBN.R58.esd 621 0 621 ESD GOOD: 88-323

GCACGAGGGCTCTATGGTCACCCATCAAGTCCGGTGGACGTGCTGGAGTA

CGTGGTTTTCGAGAATTACATCACCGACGAGTACGGTCGGTGGCGAATTC

ATGGAAAGGTGGCACCTGCGTGGGCACGTGGCTTTACCGCCGTCGCTGCT

CCCCAGAAGAACCGCCGTCTCACCTCCTCCCCTGACCCCACGACACAGTA

ATAAATATTCTCTTCCCAAAAAAAAAAAAAAAAAAA

>TSBI.R52.esd 588 0 588 ESD GOOD: 104-305

GCACGAGGGACACAAGTAGTTGCGACACCAACTATGCTCACATCCCTTCC

TTCGGAGGTGTACGCGCCGCCGTCAGCAGTCAATTCTGCACCAGCAACAG

CTTCTGTCGTTCACGATCCACCCGTTATCCTAGCTTCTGCGCCTGAGATG

GCAAGCTGATCGACAGTGGGCACTCTTGCGGTTTTTTTTTTGAAAGAGCG

AA

>TSAD.R9.esd 675 0 675 ESD GOOD: 94-623

GCACGAGGTGATTTTATCCCCAATGGCAACCTTAGTTGAAGCCCTCCATC

GCAAGTTTAAAGCTCTAGTTTCCGACGAAATCACACCAGAATGCAAGCTG

AAATTGTCTTCACGCGCAGGTGGAGCTCCCGCCATTTGCACACGTCAAAT

TGCATTGGATCATCAGGGAATCACCAGTATAGGTGATCCTAAAGAAGTTA

TTTATATTTTTGAAAACGTTCTCGAGCTGGATATATCAACGAATCGGATT

CATTTCTGGTCAGATGTCTTTGCTCTTTTGGAGTGCTCTCCCAACCTGAA

ATCTCTTAATCTAAGCTACAACCCCTTACGACACTCCACTCTTTTTTGCG

AAACCGAGGAGTTTGATGATTTAACAGTCCAAAAATCCGAGGTTTCCACA

GCTTCCTCGCCATCTCCTTTAGAATTTAATGTGCAATCCAAAACCGTTGA

TTCTAATGAGGAGGAAAGAAATGAATCGATACCCAGCAAACAAATCTCCG

TAGCGCCAGACCTATCCGCATCTTGCCTTT

>TSBJ.R78.esd 659 0 659 ESD GOOD: 95-213

CGGCACGAGGCCAAAGCCCCCCCACCTCAGCCACCACCACCGCAGATGAT

GATTCAGATGATGAGCTCGTCTACATCCGTTCCTGCTCCGTCCATGCGGA

CTTCAGCAGTAGTAGGAGA

>TSBK.R26.esd 648 0 648 ESD GOOD: 94-566

GCACGAGGCTTAGTTAGCTTTGAGCGCAAGTGTGCATCGATGTCGAATGT

CGAGGTGGGAAAGAAGTGTGCGGCTTATAAGGCAGTTGATGAATGGCTTA

GGGATGGACAGGTTGTTGGTATCGGCAGTGGCACGACGGTTGTGTACGCA

GTGGAAAGAATAGTGAAGTTGGTTACCAAAAATCGGTACAAAATTCAGTG

CATACCAACATCCTACCAAGCTCTTCAACTAATAAAGGAGGCTAAATTGC

CCCTTACCTCTCTGACAGACTATCCTTTGCTGGATATCGCTTTTGACGGA

GTTGACGAGATTACTCCTGATTTTTTATCCATCAAAGGGGGTGGTGGTTG

CCTCATCCAAGAGAAGATAGTTGATGCAAACGCCAAGCACTTTATTGCGG

TAGCTGACGAATGCAAATTGTCAAAATACCTTGGTGAGCACTGGATTCGC

GGACTTCCAATTGAAGTTATTCC

>TSAU.R66.esd 609 0 609 ESD GOOD: 100-497

GCACGAGGGTCTTACCTACGAGTGTTGTATGTACTTCGTAAAAATAACCG

CCTTTGTTGAAAGCCGGCTTCTAAACCTGGACTGAAGTTATTGGAACATG

TATCGAATTTTCGTCCTTCTGAATAATGCTGCAGAATACGACTTGCAACG

TCAGGCTTTCGTCGGCTTTGATTCCATTGAATTGGCCACAGAAGCGCTGA

GTGCTACCCACGGCTTCATGCTCAACGGGAAGCCTATGCACGTTCAATTC

GCACGCGGTGTCATTGCCAAACCCGATCCCAACAGCCTCATTACGTGAGA

CGAGTAATGTATCACTCCCACTTCACTTGCTCCTCCCATGGACACAAATC

GATTCTGTTAATAATAGTCTTCTGTACATAAAAAAAAAAAAAAAAAAA

>TSCD.R37.esd 426 0 426 ESD GOOD: 103-426

GCACGAGGCACCAGGTCGCGATGATTAGCCTCGTCCTTCTTGCTCTCATG

GTACTTTGTGTTGCCGGCCACGAGAAGGGCAACAGAGATATCTGCAGCCA

ACCAATTGATAAAGGATATGGCAGAGCTCGCATCAGCGCGTGGGGCTATG

ACGTCGCAAGAGGTCACTGTGTCCATTTCTTCTACAGGGGTGCCGGAGGC

AATGAAAACCGGTTCCAAACACTGAAGAAATGCAGACGTGCCTGTGGCAA

TGTTTTCCTCAATTAGGAGAAACTCACTTCTCTTCGATTAACAATAAGCT

TGAAAAACATCTATGCGCTCCNNN

>TSAH.R78.esd 588 0 588 ESD GOOD: 105-537

GCACGAGGCGAAAGAGCAATGGCTGAAGAAATAACTCCTCTTGTTGTGGA

CAATGGTTCGGGTATGTGCAAGGCTGGCTTCGCTGGCGATGATTCCCCTC

GTGCTGTCTTCCCCTCCCTTGTCGGTCGTCCACGTCAACAGAGCATCATG

GTTGGTATGGGCAACAAAGATAGCTACGGGGGTGATGAGGCGGCAGTCGA

AGGAGAGGGTATCNNCTCACCCTCAAATACCCCATCGAGCACGGCATTGT

GACCAACTTGGGACGACATGTGGAAATCTTTGGCATCACACATTCTTACA

TGAACTGCGTGTGGCGCCTGAGAGCACCCTGTCCTCCTCACCGAGNGCTC

CACTCAACCCGAAAGCCAATCGTTGANAATTTGACCAATCTCTGTTGAAC

CTTCAACGTGCCAGCCATGTTTGTGGCTATTCA

>TSAW.R54.esd 601 0 601 ESD GOOD: 100-405

GCACGAGGCTTAAAATAAGCACTCAATGTTGTTCTTCTCTACCGGAGAAG

TCGAATCATTTCTGTCATCTAATTAGTTTGGGACACAAATTTACGCAGGC

AGAAACTAACACGCTTCATTATGAATTTGCACTCAAGGGTGGGCAACCAT

CTCTTCTGCTTCCATATCTCAATTGAATTGGCTTTGGGCCAATCCGTCAC

AACGCCCTTTTTTGGGCCACTATTTGAAAACTTGACATTAAGCCATCAAT

TAGAACGTACCCACACCAAATCAATTGGTCACTTATCATTCACGGCNTTT

AAGAAA

>TSBU.R19.esd 673 0 673 ESD GOOD: 101-564

GCACGAGGCACAGATTATCAATCAGCAACGTCAGATCGATCAGCTTCTTG

GTCTACGTGAGGCTTCTCGTGCTAACGCTTTGAATGGTGGAGGAGGGGAT

GGAGCTGGTGATGATTACAACAGTGCTGGTGAACCAATACCACTGCCTCT

GATTCCAGCAAGTGTATGGACTCGAGGGGATATTGCGGACTTTAAAAAAG

ACATTCTGACGAATCATCGCGAGAGTTGCATCAAAATTTCCTCGCTTGCA

TCGGCCACCCTACGGGTTCCAACTCACGAGGAGGGATCCAGCATTTTTTG

GGAGTTTGCAACTGACTCCTACGATTTGGGATTTGGTCTCTTTTTTGAAT

GGAACCTTACACCTCAGGAGAACATCACGATAACCATTTCTGAGTCGAGC

GATGAGGAAGAAGAAGAAGAGGGAGAAGAAGATGGGGATGGAAATCCAGT

TGAGTCTGCCTCAA

>TSAL.R42.esd 246 0 246 ESD GOOD: 111-210

GCACGAGGATTGCTTCTGCTTTGTAGGCGAGTTATGAAGGATTGTTTGCA

TGTTTAAAAAAAAAAAAAAAAAAAAAAAAAAAAACAAAAAAAAAAAAAAA

>TSBY.R9.esd 512 0 512 ESD GOOD: 98-512

GCACGAGGGCCGATGATAATACTCTACCCCCAAATGTCCCCTTTCTGGAG

CGTCCAGAGGGTCAGCTGCTTGTCAGTCGACTGCTACAGAATGAAAAGAA

GAGCGGCTCACACGAATTTGCTCGTCTCGTCTGTGAACTGGTGCCGGAGG

AGACGCTGCAGGCTTGGCTCACGTGTAATCGGTCGTGCTTCACTCTGGTC

CACCTTTACGAATTGAACGACGAGGACATATGCGATCGGTTGAAGAAGGT

ACTGTGCTCTCGAAAGGACCTGATCATGGCTTCTCCGTTGCCTGGAGCCA

AAATTCTGGCAAAGCACCTGTTTTTGAAGCAATGAGAGCACTTGTAGAAT

TTCACCCATCCGTCGCGTCTGCATATTCAATCAACAGAGCACTGACATTG

ACAATTAACGCANNC

>TSBS.R25.esd 481 0 481 ESD GOOD: 94-463

GCACGAGGGTCTGGGAGACGTGCCTCGACCTACCACTAGCGTCTTTCGTC

CTCCCGCTTTCGTCCCTCCTCCTTAATCCTCTCCTTCCCTGTACATGGAT

CTTGTTTGTGGATCTGCTGCTGTCGTTATTTCCTGACACCCGTCGCCTCA

TGCTTCTCTGCTCAACTCACTTCTACCACCGCCACTAATACCACAAGCAT

CCCAATCGCTCCGATAATAGTAATGACGTTTCTGATGATGATAGTGTTTC

TGTATTGATTAAAACGATTTATAACTTTCTTTTTCACCTCCTCTCCTTAC

TTGCGTGACTTTTAGCGTTCGCTATGTTACTCCGATCCCCCTTTTCCGTC

CCCCTCTGTCTGGCCGTCCG

>TSAQ.R8.esd 773 0 773 ESD GOOD: 91-582

GCACGAGGGTCTCATCCCATTATCCAAGTTCCAACTATCGCTCCGCTTTG

AGAGTAATAGAAGATAAGCAAATGCAACACTGAATGGACAAATGTGAAGT

AAACCCGAGGTAAAAACGGAGGTGCCGAAGACAGGTCTGATTATTTGCTT

TTAGTTCGTGGCTTGTAAGAAGTGAATTGTAGCGTGCAATGTTTGTTACT

GTACCGGACAGGTCGGGCTAAAGATTCTATAATTGCTTACTGGTTAATAT

ACATGTCCAAGCAGTTTCCTGATCGCTTAACACTTGTAGTACAATGCGTA

TGTACCTAAGTTGGAAGAGGCAAGAAGCTAGCAGTCCATCGGTTTAAGTC

CACCATCCACCTATTACCACTCCCAGGAGCTCAGAAGTCGATTTACAGAG

CCCTATTATTAGTTAATTTGGGTCTTTAATCATTCGCTCTGCCGATTTTT

TTCAATTTTTACGTCTAACAAGCAAAATTTCTAGCCAGGAAG

>TSAS.R34.esd 644 0 644 ESD GOOD: 100-617

GCACGAGGAACAGGCGTTGTATGCCCTACAGAAATGCTTTGCCAATCATG

AGTTTCAAGAGGCCCAAATCCTGGGTCACTTCCTTCTCTACCACCTCTCC

CCATCCAAATACACCACAGAGATTCGTCTGCAGCACACTGCTTCCTCGCC

TCCACCTTCTTCCCACGCTCCAGCAGCATTCCATTCCAGCGAGTCTTCTA

ATGGGCGACAAGAGATGGCCTCACAGACGAATAATAACAATTCCGCGGCA

AACAATGACCGCATTCCCAATGGACGTGTAAGCCCACCGAAACGACACCT

TTATACTCCACCCTCTTCCAACGGTTCTCGAAGGCATTGGCGTCGAAGGC

ACACTCCTTCCGGCAACTCCGGTCTACGCCCCGCTATGCGCTATGCACCT

GTGGAGACCCTGCCTTCGACAGACGAGGATGAGGTGGAGGACATTTCCAT

GGAGGAGGGCGTGCAATTGCATCACCATGAGGAGCTTGAAGATGATCAAG

AGGAGATTGAGTCTTTGG

>TSBR.R82.esd 685 0 685 ESD GOOD: 82-619

GCACGAGGCGCCATTTTGTGTTTAACCAAATTAACATTTGATTGTCAAGT

ATGCCGTGCATAACTATTTTGTAGATTCACGAGTTTAAACCTAACAGAAA

TTTTACTTAGGATGGCTACAAACCTGTTTTGTTACGTGGTAAGCGAGAAA

TACGTCAAATATGTGATTGCGGGAGCACATGTTCTCTCACCACTACAAAT

TGTTCTAAGATTTTTACATATGGTATTCGCTTCTTCGTGTTTGACCTCAC

CTGATCGGCATTTGCGCGATTGCATTTTCCTTCCCGTTTCTCTTCCCTTC

CTCCTTTCCTTCTTCCTTTTTATGTTTTCAGATTACGGTAGCATTACTTA

TGCTACATTTTTCAATACCAAGCAGTGAATCAACTATCAACCAACTTGTA

GTATTAATAAAAGCGAAGCAACGAGAAGTACGGTTAGACCTACGCACATG

TGCACATCGTTGCACTCATTACCAATTTGCAACGACCGGTTCCCGACGTT

CCATTTGGTTACTTGGCTTAAGTTAATCCAGTTCAATA

>TSBE.R85.esd 608 0 608 ESD GOOD: 107-602

GCACGAGGGTTTCATTTTGCGGTGGCTTACCCACAAAGACCTAGTTAATG

CAGTTTGTTTTATGTTACTTTTGGTGGTGGAGGGAACGAGGTGCATGCAG

CGTTAGGAGTTTTAAATGAACTTTAAGAGCGCATAGATCTTGACCTATAA

TTTTGAAAAGAAAACCGCATATTTGTATCGGACTTCTTAAATTAGGTAGG

CAATGCTCTTTGTTTCCCTCTGTCAGAATAATATTTAATGTTTCATGAGT

CTTTTTAATGCTGAAGTATATTTAGACGCTTGATAATATAGTCCAGGAGT

GATTAATCTTAGTGCCGCATCTAATTATGTATATTCATTGGAAATTCGTT

CCACTCAATGCAAAAGCGTGTTGGCCTAAGAAAACGCACACTGTAATCGT

CAAGTTAGGATACTGTGCAAAAATAACCAACCAGACTTTTTTACCCTTGC

CACAGATGAGAATGGCTTCTCTCTTAGATGACCTTCTCGAGGGGGG

>TSAT.R13.esd 682 0 682 ESD GOOD: 105-476

GCACGAGGGATCGAGCGAGCCGATGTGACTTCACTGGACCGGCACTCCGG

CACTCTACCGCCGCCTGGCCCCGGAGGCCGAAGTAGCCACCACTCGTTCG

GCAGACACCCCAGCATTGCTTCAAGTTTTGAATCCTTGGGAACCCTAACC

TCGTCAGGCAAAACACCCATGAATTCATTCCGGTGTTGAAATTACCCTCT

CCCCCCCCTTCTCGGCACGCCCCACACAACCGACCACTCTGATTCCCTCA

GCACAAACATTCACTTAACTTATATATTTCTCCAGTCGTACTCCCCAGTC

GCCTGATAACGGGATGTATTCGTTGAAATACAATTTGATAAATTAAAAAA

AAAAAAAAAAAAAAAAAAAAAA

>TSBT.R28.esd 688 0 688 ESD GOOD: 94-267

GCACGAGGGCTCCCCCCGCCTTAGCCGCTCCCAGGTCTCCACACAATCTA

GCTGCGCGCTTGCGCAACTGGACTTTCCATTTCTCCCGCTCCTCCAGCAT

GTTCGAACGCCAGCGTAATCCTCCATCGTCAAAATCACCTCCCCAAGGTT

CAAACCACACCAATATGAAGATGA

>TSBC.R81.esd 660 0 660 ESD GOOD: 84-550

GCACGAGGCACCAGTCAGAAGTGCGCATCTCATCGGCTGCTGGGAATCCC

CTCCTTCCGTCTTCAGGCTGTGTAGTAGATTAACCGCCAAGGCGAAACTG

GACGATGGGACTGTGGAGGCTGTGGCACTGCTCATTTTTATCCTCTTCGA

CCGCGCAGTGAAGTACTCGGGTTTCGGGAACTTTGTGGGCTTCCTCTCAC

GCCACGGTCTCATGGCATCGTCCGGAAGTCGTAGCGGCGGTGACACATAT

TCCTCGGCTTCCTCAGACTCCGAGACAGAGGACTATCGGGAGTTAAAAGA

CCAGATTAACCCTGTCACAGGTCGTGTTGAACCACCGCGTCGTAATCCCA

TGGAGGGTCTCAGTGATGAGCAGAAGGAGTTTGAGGCTATGCAACTGGTC

AACAAGATTGATCAACTTCAAAGAAGCGGTGTAATCCAACCGGGAGTTGT

GGGTGAGGATGGACGAG

>TSBJ.R36.esd 513 0 513 ESD GOOD: 120-513

CACGAGGCCACNGATTGCCTTAATNNCTGTTACCCAAGCTGCTGTGTTGG

ATGCTCTACATGTGAATTCGTAGTTTTATGCTACTACTACCATGTACATA

ACATGACAGGTACTTTGAATGGTATTTACCATTCGATATCTTGCTTGCAC

GTCGTCTGCTTTTGGGATGTGAATAGCTGTTATCTAGGAATAACTGACTT

AGCTAACAAATCACCCTCTGTTCGCCCGGTTGATCGCTTCTCGTGCCCTC

TTATCAGCAAGAATATACTTTTGCCTATTCACTGATTAAATTTAAGTGAA

ATAGTAGGTGAAATGTCACCGTTGGAGTTGGCATTGGGTCAGTTGTTCCT

TCACCACTCGGCCAGTACACTCCTGAGGTCAGCCGGTCAATGNG

>TSBJ.R43.esd 571 0 571 ESD GOOD: 93-571

GCACGAGGCTTTATTGTTTTANATTTTTGCCAAATTCTTTCCAGGCAATT

TATTACCACAGCAACAAAAGACATTTTTGACCAACAGTTATTTCACCGGT

GTTGTTCTACCTCGACCGCAAAGCATTGTAAGTCTTGTTCCTCTGTAAAT

ACGGTGTTCTTTTTTTTTCATTACCACCCAGAAGCAATATCCTGAGGTTG

TTTTAAACCCAACAATAGGCTTCCAACAGAATTTGGTGATGTTATGTGGA

CATTTGCTTTTTATCTCCGTTTTTAGGAACCTCTGGAAGTTAAATTCGAA

GGAATGAGGCCAAAAATTACACCAGCTGAGTTGGAGCTCTTAAAGGTGAG

TTCATTTGGCACCCAAACGTCAGAAATGTGGATTGCAGTAACTGTTGAAC

TCTTAGTCCCCCGCTCAGGTGTGCATATTGGGACTAAGAATTACAAGATG

CTTCTCTTTGCGTCCACTTACAGCATNNN

>TSBI.R5.esd 681 0 681 ESD GOOD: 125-511

GCACGAGGAAAGCATTGGAATTCCGTGAGTACGAAAGCAATTATCTTAAT

CTACTTGGTGACACCTTGCCCCATGTTTGTTCTCAAACATTTTAGGAATG

CGTTTTATGTTGATTTGGAGTAATTATCGCTTGCGTAGTACTTCTTTCTT

GACTCGCACCCATCTGTTTATTTAATCTTTTCATTAAGGTGCTCTATCAT

CTTATGGTAAGTTGTCCTCTACGTTAATTTTTAGTTCCTTTCTGGAAATG

ATCGAGCATTACCTGGCAAAACAGACGGGGTCGGAACGGAAGCCGCGATC

TGAAGGACTGAAAGAAACCCGCATGGCTTAGTCTGTATGCTTGATTAGAC

ATGGACTTGGTGAAAAACCCAACCAAAGAAGCGTCCC

>TSBM.R84.esd 643 0 643 ESD GOOD: 101-643

GCACGAGGGTCGCTTCTCCAGTGGGTTTCCATTACTAGCCTCGAATGCAA

GACCACCTTCCACAAATACGGGTTTAAGTGAATTGAAGTGTTTCGTCTTG

GGCACAATCCGTCCAAATCGACCACAGTCGGTGAGGATGCGCAAGTTGAC

CTGCGTGTAGACGGGCACAATTGCCAAGTAGATGAGGCCAGCGTTGAACA

TTCCAAGTAGATTTGTCAGCATTTGACGGTGGGTTAGAGTTACTCTTTTC

ACTCACCAAATCCACAAAGTGCGAATAGGCAAGAGGTGCGTTGAGGTCCC

CCTCCATCATCTGGCCAATTCATCACACTCCTTCCCGCTCATCAGCCATC

CAGCTGTCCAGCTGAGTAGCCATGTAAGATAGCCATGTAGGCATGCAGGT

CACGGCCTGCAGATGTGAAACACATGAGGAGTTAAGGAGAGCTTCATGAC

CAACACTAGAAGTCGTGCAGCCCAGGTGAGAGTGGGAATTTCTAGGTGTC

AGGCTCTCAGAATGACGGTAGTTAGCGCCGCATATCATTNNNT

>TSAQ.R65.esd 632 0 632 ESD GOOD: 95-558

GCACGAGGCAATAAACGTGTCCGTCTCACAACTGCTCTACACATTGCTGC

AGAACTTCAGGACTTGAAAATGGTTCAACTATTGCTACGTTACGGTGCTT

TTGCTTCTCCGACCGATTTCAGAGGACGCAAACCAATCGATTTTGTTGAA

GAGAATTCTTCCATCTACAAACTTCTCTCCGCTTATACCGGTTGGGTGCC

CTCACTGCAATTTCTCTCGCGTCTAGCTATCAAACGGCAAATTCCCTCCC

TCCTGCCCACCTACCTATCTCGTCTCGATCTGCCCAGCATTCTTCGCGAT

TACCTGACTTTTAAGTTTTAACCTCTCAATGGTTTTTTATTACTCCTTTG

GTCTCATTTGCTTGCCATTCTTTTTTCACCATTTTTTTGTCACTTTTTCT

TACCTCCTCATTGTGACATGCTTATATTTATCTTTTCATAAATAGTGTTA

GTTCAATCCTCATT

>TSAJ.R91.esd 703 0 703 ESD GOOD: 162-657

GCACGAGGCTCAGGGTGTTTCGTTTGGGAATTTACTTCTGTCGTTAAATG

TTAGAGATCTTATTCCGTTGCGTTTTAGGTCTTTTTTCGCAATCTTTTCC

TACGAGGAAGATATATCACTTATTGTGAATAGATTGTCATTTCTTTTTAG

CCGTTACCCTTTTTGGGAACCCAGCGTTTCAGGGACCTTTTGGTTATTTC

TCACCGCGTTGTCCAAATAAAGATAGACATGGAGCACCTCACACTTTGTT

TACGGAGGGATATTCTTAGAACTTTTGTACGAAGCTTTGCCGTCNAGAAG

AAACCCAATTACGCTGACCAACCTGAGAAAATCAGAAAACCCGTTCTGGT

GAAGACACTGAGAGGTTTTCAGTTTAATGATGGAGATCATGTCTACCAGG

GTGATATTTTAGTACGACAGATGGGTCTCGAGATCTACCCGGGTGAGAAC

GTGAAGCTGAACAGAGACACCTGGGACCTTGTTGCTTTGCGCGGCG

>TSBJ.R64.esd 754 0 754 ESD GOOD: 99-217

GCACGAGGGTTATCTTTGGACCAGAAGAAACTGCTTTCGAAGATGGGACC

TTCAAACTGAAGCTGGAATTCACGGAAGAATATCCCCAATAAGGGCGCCT

TCGCGGTTGTTCTTTCCCT

>TSBT.R40.esd 895 0 895 ESD GOOD: 89-193

GCACGAGGGGATATTCTCGTCCCGTGCAGCACCGATAAGTGAGAGTGAAC

GTGCGCGGATTGCAGCAACCACCATTTGGCTCCCAGTTCCGAATCAGCAG

TGCGC

>TSAP.R63.esd 475 0 475 ESD GOOD: 99-416

GCACGAGGCTCTCCGCTTCCTACGATCGTTTCATCAAGCTGTGGGATACG

GAGTCGGGTAAGTGCACGGGCCAGTTCAATCTGAAAAAGGTGGCTTACTG

TGTNCAATTCAATCCTGATGAAGAACAGCAACATCTCTTCTTGGCTGGAT

GCGCAGAACAGAAGATCCTCTGTTATGACGTGCGCAGCGGTGAGGTGGTA

ACAACCAGTAACGATCGCCACTTGGGTTGCGGTCAACACAGTGACGTTTG

TCGACCAAAAACCGGCGCTTTGTCTCCACCTTCGATGACCAGTCGTTGCG

TGGTGGGGAATGGGACAT

>TSBI.R2.esd 720 0 720 ESD GOOD: 149-585

GCACGAGGGGTTAGCCTACCCGGCTGGGCAATGTGACCACCTAAGCTCAA

ACCAAAACCGACAGTCGGAACAAACAAGGTATTGCACCTATGTCGTGGGA

TGTTTAAGAGTCATTGCAATGTTCAGTGCAATAATTAAGATAAAGCACAC

AAATATTACTCTACTGCCTATTTTTTATTATTTACTGTTTTAGTTTACCT

TTTAAATGCATGGTCTTGCATAAATCTTCTGATTAATGCATTTACAAAGT

AAGATACATGTTTAGGTGTACATCTGTTTGCCATACTGATTGGCAAATAC

AAATCAGCATAGCGAGTTTGTTTATTTGTAGTTTAAAAAGACAAAAATCT

AAACAGGCTTATGTTTTTATTGATAACACATTGAGCGGCTACCGTGAATT

TTGCTTTCGTGTGGATTCTGGGACTGCTCCTGATGTG

>TSBH.R55.esd 415 0 415 ESD GOOD: 114-414

GCACGAGGTCTGGACGTACTCTTGCAGCAATGGAAATGCGAAGACTCACT

GCAGCCCTTTTGGGCATTGCTATCGGCCTCATCATCATCGCTCTAGCCAT

CACCGACTGGCGTTATGGCCTGGCCTTCGGAGGCGCGACCCACGACAAGG

AGGCGATGACTGCGGTCACCTTCCTGATCATCATAGGCCTAATCTGCCTC

ATTATTGTCTTCATCCTCGACATTGTCATGCTCTGTCAGACTGCTGTGCC

CTCGGGCATGATCACAGCACGCTTCGTGATCCTCTACATCAGTGTAGCGN

G

>TSBR.R16.esd 553 0 553 ESD GOOD: 102-509

GCACGAGGCGGCACGAGGATCCTGATGTAGGTTCGCGGAACTTATCGCTG

ACGAGAACAAGCTTAAATCTCATTGCCTCCTGGTGCCATCCTACTGTCAC

CTTGCAAACTCTTGATGCCTTTTTATGAATCAGGTCTCACTGGAAAGCGA

GAATCCGGATAGAGTGACTGCGATACTGGCAAACGATTCATTTCTCTTCA

TTGGCACTGGCTCGGGAGTCGTTTGCGTCTACAAGACGCTTAAGTGGAAA

AATTCAACTCTTACACANGATGGAAGTCGTCTAGCATTGCTTCGCCACAG

TGTAAACCTTCCCGACTACGCGCCCAGTTCAAAGAATTTGAACTGGAGTT

TGCGTCAGCCCCGANAAGGGAAGNGGATNGGTCGGATTNTTCAAGGCCCC

TCGTTGTC

>TSCD.R49.esd 571 0 571 ESD GOOD: 96-550

GCACGAGGGGTTCAAAGTTGGCGGCATGCTGCAAGGATGGAATAACGCGC

ATAGTGGATCCGAGAACTGGAAGGTTCACCGAAACGGGCAATTGTCACGA

AGGTAAGAAGCCACAGATGTGCGTCTTCGTGGAAAGTGATCGCCTCTTCA

CCACAGGCTTTTCGAGGATGAGTGAGCGTCAATTGGCTCTTTGGGACTCC

AGCGACCTGAGTAAATATCTCTGGCGCGAGGAGCTGGACATCACCAACGG

CATCCTCTTTCCCTTCTACGACAATGACACAAACCTTATATTTGTCTGCG

GTAAGGGTGACAGTTCGGTGCGGTACTTTGAATACTCTCCGCAAGACAAA

CAGGTATACTACCTGAGTCGCTACGACAGCACCGACCCACAGCGAGGTTT

CGCCTTCATGCCCAAACTTGGACTCAACGTGGCGAACTGCGAAATCGCTC

GCATG

>TSBG.R40.esd 697 0 697 ESD GOOD: 98-225

GCACGAGGGGTCTCTGATTGGAATTAATCTGTGTACTTCCAGAGGATGGC

GCTTTTTCGTTGTCTAAACTTTTCGCTACGACAGGAGATTATGTTGCCTG

TTAGACTCATCGTTTCGAAGACACCTCC

>TSBP.R3.esd 424 0 424 ESD GOOD: 90-424

GCACGAGGGGCACATCAGCTGTAACCACCTCTGAGGCTGCTTCCCACAGC

CATCACCAGGTGTCAGGGGAAGGAGAGGGGGCTAAGGCAAAGGATAGCGC

TGTGGTAGAATGCGATCCCGAATTCCTCCAGTCTGTATTGCAGTATCTGC

CTGGTGTGGACCCTCAAAACGAGGAGATGCGCAAGGCAATTGAATCCCTT

ACTACGGGCTTCAAATCGGATCAAGGCGCGTCCAAAGATACCTCGGAGGA

AAAAAAAGACAATGACGGGGGTAGTGACATTGATAAAGATGAGTAATTTG

GATTTGTAAGTTGGCCACAATAAAGTTCAATANNN

>TSBA.R72.esd 712 0 712 ESD GOOD: 116-594

GCACGAGGCTTTAAAACATCTACAGATAGAGGAACGGAGAGGCAGGTGGT

TAAGCAAAGAGACAGATGAAGAGGCAGATGTTGCCTTAAACGGACAACTC

AACTCAAGCTAACTGAGTAAACAGTTGAATTCCAATGTTTCCAAAAGTAT

CATCGCGAACCGGCTATGGTGGTGGGTTCTAATGTGGAAAAAGAAAGGGT

AGGTGAAAAAGGAAAGTGGAACATGGCATTGCCGCAAAGACTGCCGTCAC

CAGGCAGTGATCAGAGCGATAAGCATGCTGCAGACTGGGCGGTGCACCAC

AAAGTGGGGCTCGCCAATTGATATCAAGAGACTGGCGTGTGGGCGGTGAC

TACTCTACAATTTTGTCCCTCTTTGCATACGCCTCGTCAACCAACTACCA

GATGCCACATTACGGACAGGCCGTAAATAACGCCCAGCAGCATCTTGTAT

CTGAGTCATGGGGTAGGTAGGGGCGGGTG

>TSCH.R90.esd 668 0 668 ESD GOOD: 102-576

GCACGAGGGGAACGCCCCAGATGTTCGCTCAATTTTCTCAAGTTGTGTAA

AGTTTGCTACTACAACGGATGCTTGTTTCACAGTGTTCAGCGAAACTTGG

TAGCCCAGACTGGAGACCCCTCAGGCACCGGCCGCGGTGGTAGCTCAATT

TTTGAGCAGCTCTACGGAGAACAAGCTCGGTTCTTTGACTGCGAAGTTTT

GCCAAAAATATATCACAAAAAGCGTGGTTTGGTTTCTATGGTTAATAACG

GCAGTGGCCAACACGGATCACAGTTCTTCATTACCCTCGCTGATAATCTC

GACTATCTCAATAACAAACACAGCGTCTTTGGCATTGGTAGCAGAAGGTG

AGGACTTTTTGTCGAAAATTAACGACGCTTATTGTGGACAGGAGTCGCGT

CCCTATCGCAATATTCGCATCCATTCGGCGATCATTTCTAAGATCCGGTT

GCGGATCCGGATGGGCTTGAGGTTC

>TSBV.R13.esd 554 0 554 ESD GOOD: 107-402

GCACGAGGCTTATCATAATCATCTCCCTGGCAGCCTCGCTGGCACCAAGA

TGAGTACAAGCGCTCCCAGACAAGAAGTCGTCCGGTTTGTCTTCGCCGGC

GCGTTGTTTGAAAGTGCTTCCTCGTCTTTCATTTCCCCTCTCTCGACGTA

TCTACTTCCAAATGTACAGGAGACTGAATCAATTTTTATACTCCCCCGCG

CCTGACCTGATCAGACCTGCACAACATTTATTTGATTTGAATTTGGTGTC

GGTGGTTTACAAGCAACTTTTTCCTGCGAAAAAAAAAAAAAAAAAA

>TSBK.R28.esd 590 0 590 ESD GOOD: 103-347

GCACGAGGCTCACCTTCTTGATGACTTCCAATTGCCGGACATCTGCAACA

CTAACCTAGCTTGTGATTTCCGACCATGAACATTCCATCCAATAAATGCT

TCCTATTTGTAGTGCTCCAATCAGGTGCCCGTGAATATTAACCAATCATG

ATGCTGCGAACTGCCGATTCGTTAAGCCGTTATTCTTTTTTTAAAAAAAA

TTATTGGACAAAGTAAAAAAAAAAAAAAAAAAAAAAAAAAAAAAA

>TSAB.R53.esd 741 0 741 ESD GOOD: 112-586

GCACGAGGGTCTCCTTGATTAGGTAGATGTGTCCGCTTACTCACTAACTC

CCTGGCTTGCCGCCTCCGTGTCTAGGAGCATACTTTTCGCCACCACAATT

TGTTCTCGCCATCCTTCTCGCCGCCGCCGCTGCGTGTATTACTGATGTGG

TTGATAGTAGTATTTTTCGTGATGATGACGACGATGATAAGTGGATAAGG

AAGAGGAGGAGGCAGTAAGGGAGGTGAAAGAGCACCAAAGGAGATCCCCC

TTCCCTCCCCCTCCCTCACCCCTTGACCTATTTAAATCGTCCATCTCCAT

CCCAATTATGCCAGTGTAGGTGCGTGTGTGTGTGTGTACATGTGCATCTG

TGTGCGATGTCCCCGATTTCTTTCTCCCTCTCTGTCCCACCTTCCTTCTT

TCTTGCTCCACCCCTCTCTCTCTCTTACACCGCCTTTTTTGTCTGCCTTA

CAGTCAGTAATAATTATTATTACAC

>TSCE.R96.esd 792 0 792 ESD GOOD: 111-475

GCACGAGGCAACGCCGTCAGTGGACATCTTCGTCAAGCGCCAGGATGTTA

ATGAAGAGTTTCGACGACTCTCTTCTATTGACTTTGACAAACTCGAGGTC

GTCTCCACTTAAGTTCTTTCCTCCTTTCCCGCCCACCTTGCCTAACTCAT

GCAGTTTGTAAATAATTGAATAACTGTCTTCGCTAAAAAAAAAAAAAAAA

AAAAAAAAAAAAAAAAAAAAAAAAAAAAAAAAAAAAAAAAAAAAAAAAAA

AAAAAAAAAAAAAAAAAAAAAAAAAAAAAAAAAAAAAAAAAAAAAAAAAA

AAAAAAAAAAAAAAAAAAAAAAAAAAAAAAAAAAAAAAAAAAAAAAAAAA

AAAAAAAAAAAAAAA

>TSAE.R92.esd 676 0 676 ESD GOOD: 93-448

GCACGAGGCCTACGTTTGCCTCATCACCAACAGCACTACCCATGTTCGAG

CCAAAATTGAGAACACTATTCCCCGTAAACGAGCTAACATGCCTACTTCT

CCCCACGATAAGGCGATTGCCAGGTTCTTCGATCAGATCATCCAAGCTAT

TGAACGTCACGTCCGCTTTGATGTCGTAAAGTGTGTAATAATAGCCAGTC

CAGGCTTCCTGAAGGATCATTTTTTCGAGTACCTTTGCCAGCAGGCGTTG

CGTGGGGCAGAGGATAAACGGGTATTTTTGGACAATAAGAGGAAGTTCGT

GCTAGTGCACTCCTTCTTCGGGTCATAAGCACGCGTTAAAAGAAGTTCTC

GCCGAT

>TSBJ.R51.esd 600 0 600 ESD GOOD: 86-262

GCACGAGGATTAAAGTTGCCTGGTCGGACGTTCCTGCGCCTCGTTGTGTA

TGGATCTCCCCAGCGTGAGCAAGAAATACATTTACCTCCCAGAGGCCTCT

ATGGGTGTGCATGTCGTAAAGCTTGAATTCTCCCATCTCATATTTGTGAT

CGTTTCGACTAACGGGAAGCTGGGAGA

>TSBL.R11.esd 546 0 546 ESD GOOD: 95-545

GCACGAGGCGCTTTCGTCCGATCGGGTGGGCGATAAAATCCATAAGGAGA

CACGTCGCATCTCTTTCGCTCAACGACTCTCCCCAATACCTAAACGCCCA

AGCGGTTCACGCGGTTCCAGTAATCTTTCCACGTCCGGATCCAAGGTACA

TCCACACGTCATTCTTGTAACGAGGACGCCTCGAACTACGAGGAGTGGTG

GAGCATTCAACTCGCATTGAAGCCCTACAGGAGTTGATCTTACAATTGGT

TAAATACACTTGGCCCATTCACAATGGTCTCGCATTTCTTCCATATATTA

TAATTATTTTTTTACATAGAAACCACTTTACCAGGGTATTTGACCTCCAT

CCCTATATCCTTTTTAATTTACCGGATATTTATTCAGCTACCCACGAATA

GTACTATTACTGTACCTGCTGATAAAGGTCAGCAAAGTTGTATTCCATTN

N

>TSCC.R89.esd 554 0 554 ESD GOOD: 103-329

GCACGAGGTTTACTTCTTTCACCTAATAAAAACGCGCTCTTGGGAAGGAT

TTGTGATGAATGGTGGCTTTTGTATTTCACCTGTCACTTTGCGTATTCTT

TCAATGGTAGTTCTTTGCGTGAGTGCCACCTTTTCTGGCTATTAACGCCT

AAAACATCCTAATGTTAATATAACACATTGGAGAAGACGCAAAGATGTAA

AGCACGAAGTGTTGTCTTGGGGGTCTT

>TSBR.R2.esd 821 0 821 ESD GOOD: 98-637

GCACGAGGGCGGTGTGGGTCGGTCCCGTTCACCTGTTTCATTCGAATGAC

ATGTCTTGTGTTGCAATAAAGAGATCTGCTCCATTTGATTCGATCGGTTC

TACTTCGGTAAAAAGGCATAGGTGCTCTTTAAATCTTCCCTGCAATTGTC

AGCTGACATCCCAGGAATCCGTTTTTACACCTTCTTCTCATCTTACAAGA

GATCAAATACACCGACGCATTCGACATGAGGTATGGCGCCTTCAGCGTCG

TAGAATTATCCCGAAATTCCCACCAAATTGTCTTCCAAGTCGTCGCGATG

CACATGATGTATACAATGAACGGCCAAGTTCACCAGAGGAGTGTACACCG

CAGTCGTGCTCACAAGTGATAAGCAGCATGCGTCAGATGGCGATTCGTTC

ACCTAAGTTGGATTCACCAGCTTCCTCAGACTCTGATGAGACCTCAGATG

GCTCCTGCACTCCACCACACACAGAACCGTCTTCTTCCTTGCGGCCAATG

CTCCCACAATCGCCCTCTTCCGCTGCACCGAAAAGTGCTC

>TSBI.R23.esd 710 0 710 ESD GOOD: 145-371

CACGAGGCTAAATCTTGCATCATCTAGAGAGTGTTAAACAGTAATTTATC

TTTCCACTTTCTTCCTTTTCCTGGGTACCTGCAGGTATCCCGTAGTGTAG

AATCCATGCGTAAGTAACGGACTAGTCTTTTATACATAAGTCTTTCTGCT

TCCGCATGAGTTTCTACTGTACATGGGATTTTCCGAATAAAAACAAATTG

ATATTTGAAAAACAAAAAAAAAAAAAT

>TSBK.R87.esd 604 0 604 ESD GOOD: 86-551

GCACGAGCACCATTACTGTAGTGTGCGTACCGCCACCGTCACGTCAGGTA

TGGAAGCTATCACCAATTCAGGTGTCCGCAAGCCACTGTTTCACACCTCG

GCGGCCAGCAAATATTTTCAGGAACTACCCTCAAACTCTTTTTCATCATT

CAACCTTAGGGCCACACACCTATGATTTAAACGGGTTTCCTCCTTTATTT

GACCCCGCTATTGTAAATTATCCTCCAAATGTGTATTTCTCTGATTATCT

CGCCGAATCCTATGCTTATCCTGCGAATTACCTAAGTCCACTCCCCCACA

TAAACTCATCAGCCACGCAACTCAGCGACAATTTGCACATACTCATATCC

TCTCTCTTACTTAGTGCTCTAATAGGCTTAAGCACCAACCCCCCACAGAA

GCAAGGTGAACCTGAGGGTAGATGATGGCGTCCGTGTTATCATCCGGCCA

CTTGTGAAGAGTGTGA

>TSBH.R75.esd 459 0 459 ESD GOOD: 94-455

GCACGAGGGAGGATTGTCTCCAGACCTTCAAACCATGGAACAGATTCGAC

GAGTTTTACGCCCTACCGATGTTCCCGATACCGGTCTACTTTGCGATATT

CTATGGGCCGACCCGGACAAGGAGACGAGAGGCTGGGGTGAGAATGACAG

AGGCGTTAGCTTCGTATTCGGGCGTGGATTTGGTGGAAAAGTTCCTTACT

CGTCACGACCTTGACCTTATTTGCCGTGCCCATCAAGTGGTGAAGATGGG

ACGAGTTCTTTGCGCAGCGCCGTCTCGTCAACCCTCTTCTCGGCGCCCAA

TTACTTGGGCGGGGTTTGGTAACGCCCGGTTGATGATGTTCGTCGATGAG

ACACTCATGTGC

>TSBU.R43.esd 786 0 786 ESD GOOD: 96-433

GCACGAGGGTGGAGGGCCTGCGACAGTTGGTGGAACAGCACATCAAGCCT

AGGATGGGGGGCGGATGTAGCCTACCCCTCAAAGGATGACATCTTCCACT

CTCTCCGCTTCTAACTTCTCCCTCTCCCTCACGCCTATTGTTTGAGCGTG

TGCATTTAGAGAAGTTCGTCATACCGTCTTGCTGCGCCCTACCCGCCATA

CTGCACTTTCATGAGACACCTATCCATTTTCATGTGGCGTGCCTGTAGGC

CTATCAGCGTGTGTTTGCGTTGTCAGCGGCTAGGATTCTGCGTGATTGTA

GTTACGTTATTATTGATCCTTATGCATCGTCTAAAAAA

>TSCF.R20.esd 735 0 735 ESD GOOD: 110-641

CGGCACGAGGATCAGTTTTAAATCAATGGAACAAACTTTGAGACTCGCAG

AGTATCATTCAAGTGATTACTGTCTCTACACTTTAGATCTCTTGGTTGCG

TGCATCATGTTACGCCTCCCGCTATTTTGTCTCCAAAGGCAAATCAAATA

CGTTGGTACTTTGTCTTAGATTTTGAATCTACGTGTGAAATAAAACCGAA

GTCTGACACTAAGGCCGAAATAATTGAGTTTCCTGTAGTTGTAGTTCAGG

CGGAAACTGGACAAATTGTTGATGAATTTCATCGATACGTTCGGCCAACT

GAAAGTCCCACAATTAGTGACTTCTGTAAAAAATTAACAGGGATCTCTCA

ACAAACCGTAGACACATCTCCTGACTTGAAGCATGTCTTGAAGGAATTCG

AAAATTGGCTACAACTGAAGAAAAAAGAGTTTAATTGCGTATTCAAGTTG

GATAGTTCTAACGCTGCGGTCTTCGTCACTTGGACTGATTGGGACATTAG

CACGTGCCTTTGGAATGAGTGCAACGAAAACA

>TSBW.R80.esd 757 0 757 ESD GOOD: 97-572

GCACGAGGGTTGTAGACCTGTCCTGGTGATGGCCACGAAAAGTCCTCGTG

AGGCTCTTCTTACCAAAGCCAAATTGGCCGAACAAATTGAGCGATATGAT

GACATGGCTCAGTTCATGCACAAAATTGTGGATCTTAACGATGAATCCGC

TCCCGAGCTAACCGTTGAAGAGCGTAATCTCCTTTCCGTTGCCTACAAGA

ATGTGATTGGCGCTCGCCGTTCCTCGTGGAGAATTATTAGTGCCATTGAA

TCCAAGGATGAGGGCAATGAGCGCGTCAAGAACATCAGGGCTTGTCGTGA

ACAAGTTGAGAAGGAACTAAAGCAAATGGCTAATGAGATCTTGTCACTTC

TTGAGAAGACACTCATTCCTCGTGCACAGAATGCTGATTCTAAGGTGTTC

TACTATAAGATGCAAGGTGACTACAACAGGTACCTTGGCTGAGATCGCTA

CGGCATCTGAACGTGATGAAGTAGCA

>TSBY.R16.esd 601 0 601 ESD GOOD: 108-583

GCACGAGGGGAAGGACTTTGGCCTTGGCTAGCATATCGCCCTGCTAGCAG

CGCAATGATGACTTGTGACAGCGGAGAATGCGACACTGAACCCACTCTCC

ATCCAGCCACCTCTCGATAGAACTATGAGAGACTCGAGAATTAGGCACTG

AGACCCCCTTCGCGAAGGAAAGAAGTGTTTGGTTAGCTATCGTGTTCGGT

TGGAGGAGTCCTGGTGCCAGCATCACCTCGCCAGTGTCGCCGCAGTCGAC

AATGTGCCCTGCCATCGCCAGTGCCCGCGTTCGGCGCAGAAATTGTCNTC

CTACTTCTTCGCTTCTTTCTCCGTTCCACCGCCTTCCCTCTCGCCCTCCC

TCCCTTCCTCCCTTTCTCCCTGCCTCCCTGCCTCCTTGGCTCCTTGCCTC

ACTTCCATTCGCTTCCCTTCCCTTCTTCGAGTAGTTTTTGCCTTCCACCT

CCTCTTCCTTTCGTATCCACATCCCT

>TSBN.R18.esd 622 0 622 ESD GOOD: 98-372

GCACGAGGCTCGATTTCACATCGCCTGTGTCCTACTGTGGTGTCGAGGTG

ATTGAGGCTTCTGATGCGAATAATACACCCNGTAAAGTTCTCCACAGAGG

TAAATTTCCCATCCGGGATCGGTATGAAATTGTGGTAAAGAGAGGGTAAG

CAGGAAGCGCGACTCCCGTCTTTGCATACGTTCATTACCTGGGATTGGGA

GACTTTTTAACGTGTTTTCTCCACTTCTTTTTCATACCCACTACAAAGCC

CACATGGTCTATCTCTCCTGCTTTG

>TSBL.R77.esd 655 0 655 ESD GOOD: 110-223

GCACGAGGCTCGATTCAGTTGCNCACGCGTTAAATGAACTGTGGTGGCCA

AACCCACTCCCAGTGACGGCTTCCAACATTGCGATCAAATGGTCCACACT

TCCAAGTGCTTCAA

>TSBY.R40.esd 782 0 782 ESD GOOD: 103-438

GCACGAGGACCTGTTGATGCGACAGCGCTTTTGTGCGATGAAAATGGGGG

AGAGGATAGTGATAATTCCTTTTCTTCTTCCCCTTCATCCACCTCTTCCA

GCTCTCAGGAGTATTCTTTCCTCGACTTCACGATCCTCTTCATCACCGTC

ATCGGCCACGCCAGCACCGCGAGTTGAAAGCATCTGGTCCGCACCACCAA

GTGGCAAGGCTCTCGAACCGATTAAGGTTCTGAGCAAATTCCCCCATCGG

GGTAATGAAGAATCCTCTCCATCTCTCCCCATTCGTCGACGTCNACTACT

TGGCTANCCTTGTCCCGCGCGGGAAAATCGACCAAC

>TSBM.R10.esd 565 0 565 ESD GOOD: 98-565

GCACGAGGCAACTGTCAACGCTGCAGTGGTGAAAGCAATGTGTGCTGTTT

GGAGCAGGTGCAAGCGAACAAGAATCTCGCCACGTGCGCATTGGTGCCTC

ATCCGAATGTGGAGGATGGACATTCATCCATGGTGGCACACAACAGATTG

CCCGACCGACCGACCGACCGACCGACTGACCCATGACGGTTTCTGAAACC

ACTCCAAACCCCATGTTCCTTTCAATGTTCACCAATAATTTGAGTGGTCA

ATTTTAGACTTTTAAGGATGACTGCCATTGAGTCCAATCTTGTCTCAAGC

AGATCCGCTGAGAAGATGCCTCCTGATGCGATCATTTTAATTAAGAAACA

GTCGATGTTCTGTGTTGAACTTATGGCTGCGAGCTTTGCCAATTGCAATC

CGCAGTCAATTCATCCAGTCTTTCTACAGATTAGCTCCTTTTGAGCACTC

CACATTTTGTGAGGANNT

>TSBQ.R23.esd 751 0 751 ESD GOOD: 90-597

GCACGAGGCGAAACACTGCGCTTCTGGAATGTCTTCTGCCGCGCCAAATC

ACCCAAGCCCACTTCATCAACTTTCAATCTTTTTGGAGCAATTCGATGAT

TATTTAAGCGTATGGGGCGTCACACCGTTACAACCATCAGGATCTGGCGA

AGCAGTTCAAACCAATCCTTTTCATGTAAATTTGCATCATCTTTGTCACC

CATCTGACAGATTCCTCCAATGATTTTTATGCCGCAATTCGCATGTCTCA

CCTTGTCTATAATCACTATTTCCTTCTCTCTTTCTGTCGACGCATTGTAA

ATCGGAGGTTTTTCTTGCAAAATTCGTGACCAGCATTTCATTTTTGGAAT

GATCTTGCTTATCTGCTCTCTATATCTACGTCTCAGCCTTTTCCATTCGC

CAAATTTGGCTCTCTATTGAAATAGATGCTTTTAGTTCTTTTCCAACGAG

TCCGGGAAGTATTCCTCTGTGTAAGGCTTCCTTTCCACGCATTTTTCTTC

TGTATGTA

>TSAI.R2.esd 813 0 813 ESD GOOD: 114-650

GCACGAGGCAACGTCTTAAGTACATGCTCCAGTGTTTCCCCCGTACGCTT

ATCCGGTCACGTACCGCTGGACTCTGGGATCAGTTCTCTAAGTTGGAATG

TCTCTAGTAGGAGGTGGACTCTAATGGAGCTGACAGTCTGCTGTTTTGTT

CTGTGCATAAGGCCACAGCACATATATTTGGAATTGTGCCAGCCAAGAAC

AGCTTTGTCTTTGGGGTTGGTGGGAATCATGGTCCAGACAATGAGAAAGG

CAAATACCCTTAATTGATTAACTTTATTTGAATATGCACAGACTTCTAGT

TACATAAGGCGTTGGACCCAAAAGAGCATTTGAATGATTAACTCTGAACA

TCTGTGATGTTATCAGTAGCACACAAATGACACGTCACGGGCGTAGACCA

CATGAAATCGATGAAATACACCCTCCAAGAAACTAATTGAGCAACCACAT

CCGAGAAGTAGGTTTGTGTTGGTTACTTTTTAGTGAGGCGCTGAGGCTCG

CTTGTTGCCTTAAGACGAAATGCTCCTCTTCCTGCTG

>TSAP.R16.esd 612 0 612 ESD GOOD: 110-471

GACGAGGCAAGACGGTGCGATGTCGACTGACTCACGCCGACGGTTCAAGT

GAGGAAATTGAGCTGACCCACACCTTTAATGCCAACCAGTTGGGGTGGTT

CGTAGCCGGCAGCGCCCTCAATCACATGAAGGAGACCACGTCTTGCCATC

ATCAGTGAAAAAAGCCTTCCCATTCCTCACCGCTTCCTCTTTCTAGTAGT

AGTTATTTCCCCTTCCTTACCTCCCCTTCTGCCCTAAACTATCATATTGA

AGTTGGTGGCTGATTTGTACTTCGTTATTTATTTGCCCTTTGTGAAAAAT

TGGTTAAATGTGATATTATTGAGTCACATTCATTGTCATCCTAAAAAAAA

AAAAAAAAAAAA

>TSBP.R66.esd 649 0 649 ESD GOOD: 97-579

GCACGAGGGTTAGTGGCCATAGCCGAGCGGATCCCCATATCCGTAACGTC

AGTCCCGCCGTTTCCACAGGGACGGTACTCATTGCCGCACTTACAAACCT

GTGCTCCCACCGCTTACACCCTGCTCCACCTATGAGACTCGGCTAGCGAC

AGTACGTCATCCCCTCTCCACTCAAGGATCCCCTCCCCCTCCTCCTTCCA

GGTCTACCTTTACTGGAGTTTTTAAAAAAATTCTAAGCACGCTGGTACTG

CATTTACCATTGACCAAGAATTTGCACTTGTTATTTGATTTTGCCAAGTC

AATGTTTTATCGCTACTCTTAAGCCCGAATTCCCAAAATGAACCGCCTAA

ATCAATTCAACACCGCATTCTTCAAGGCAATGCTAACATAATCAACTTTA

TCTATAGCAGATCGAACTCCCTTAATACTTTCGTAATGTTCCACAAGTCC

TCTAACTCATACAGCGTTGCAAAAAAAAAAAAA

>TSAC.R2.esd 575 0 575 ESD GOOD: 99-575

GCACGAGGGTGTATCCTTGGCCTGGGAATGAATGCTCCTGCCGCGTTTGA

GTCGTTCCTTTTGACAGAAGGCGAGAAGAAGGTCTTTGTTGAGAAGGACA

CGCATGTCCCACATGCTGCCATCTTCACCTTAAACCGTGAAGACCATACG

CTGGGTAACATGATCACTAGGTTAGCAAAAATGGGTTCATGACATCTTCT

TTGCATCAGCGTCTGCTTTTTGACACTTGTACTAGTAGTGAAGTTGATAT

TGGGACGTGTTCTTGGCATTTTTTTTGCCACGACCACACTGCTTATGCCT

ACACTTGATACTCTGTTGAACCCGTAGACAGTTGCTAAAGGACCCTCGAG

TGCTTTTTGCCGGTTACAAGGTTCCGCACCCTCTCGAGCATAGGATAGTC

ATTCGGGTCCAAACGACTCCCGCCGTTACCCCGCTTGAAGTCTTCTCCTC

TGCCATTAAGGACCTCATTAGTGAANC

>TSAN.R50.esd 720 0 720 ESD GOOD: 101-402

GCACGAGGATCATGCAGGACCTCAGCGGTTTTGCTGAACGCATTGAGCGT

GAGTTACGCAAAAACTTGGATGTGGATCCGGATGAACAAGTTGAACCTGA

TGAAAGTATTCCTCTGGAAACGGATAGTGCTAATGTGACTGAGATGGAGT

CAGCGGATATAAAGGAGGAGTTGTAGAACGATACAGGATCTACCAGCATT

TCTGTTTTATGTTGTAACTGTAAATTTACACCAGTGATTTGTTTTTGCAC

TCTAATATAAAACTAGTTTCACAAAATAAAAAAAAAAAAAAAAAAAAAAA

AA

>TSBF.R69.esd 626 0 626 ESD GOOD: 106-569

GCACGAGGCACGCTTCCTTATCAAGTGCAAAAATGTAACGTTCGGCCGAG

CTGTGCACAAATTCGCTCCAGACATCGACTTATCTCGGGAAGGCGAAGCG

TCGCGTATCTCTCGACGCCACGGAAGAATCAGTCTTGGCTGACAACGGTG

TGTTTTGGCTCACCAATCTCTCCAAACATCCTGTTTTGGTAGACGGGACT

CCTGGTGCTGAAAGATAAAAAACACCAACTCGCCGACAAGTCAATCGTGT

GCATCTCACATCTTTCTCTCCGCTTTGATGTGGAACATGAGCACTTATCG

ATGCTGTCAGGACTTGAAAACGCCGCTGTTGTTGGCGCCCTGCCTTCTCT

CCACCCTACAAACGACAATCTAAACGATGATGATAAAGCTAGGGATAAGG

AAGCATGACCACACTGTATTGTCCAATCCGTTTTCACCCTGTTGTGCATC

GCCTACAATTTTCA

>TSBA.R22.esd 652 0 652 ESD GOOD: 102-615

GCACGAGGGACGATATCGCCTTCTACCTCTCCCACATCCTACCACCTCAT

GACGGCATCGGTTAGACTCTTCGGGGTCCACTATCTACTGATGCCAGTGA

CGATACAGATTGAAACGTCCCTTTCTTCTTCTCTAACACTACTCTCTTCC

AATTCTGATGTCGTATTGCTACTGTGGTTCACCTGTGACTACTTCTTTAT

CCTTTTTTGCAGTGTGTAAACTACCTCCTATGCGTTCGAGGCGTCTAAGA

TGGTGACGGAGCTGTTACCTGCGACGACTATTAATTAGGAGGATGTACTT

GTGCGTCGATTCTGTGGTGCAAGCACCTGACGTTGTGACTGTATTCGTCA

TTCAACGAAGCAATGCTGTGAAATCGCTTTACCCTTTCGAGGGCGTCTAA

GCTTTGGGATACGCTGTTTGAATAACAGGCGTCAGTCTTCTTCAGAAGAA

ATTGTGAGGTTTCATTCTTTTTGTCCAGCCAGATGCGTTCATGCGATTTT

TGATAATAAAAACC

>TSBG.R13.esd 673 0 673 ESD GOOD: 112-267

GCACGAGGGTACTAATGTCTCGCCAATTGTGATACTTTTCTATGGTGAAT

CGAGCCCATACCAATTTTTAATACAAGTGTCGCTATTGTTGGTAAAAAAA

AAAAAAAAAAAAAAAAAAAAAAAAAAAAAAAAAAAAAAAAAAAAAAAAAA

AAAAAA

>TSAF.R24.esd 688 0 688 ESD GOOD: 111-640

GCACGAGGCGTCATTTTGACAGGCTCTTTCAATGCGTAAATCGAATCCCA

TCCTTATTTGCCGCTCGACTGCCAAAGACGCATTCTGTTTCGCATCAGTC

CAACCTTAGGGGGTCCGGAAACTCTGTAAGCACCATCTTCCAAAGTGAGA

ATTACTATGTAAGTGGACAAGGTGGAAATGGAGGATCACCCTCTGCAAGG

ACGCGCAGAGCTAGATGTAGAACACAAGTTCTTCAATCATCCTCATCGCA

CCTAATGACACGTCAAGTAGTTACCGTACTCCGACAGGTCGGGCTGAAAC

CTTACTTGGTAGCTGAGGATCGACTGACAGTGACTTGGCCGACAGAGACG

AATCTAGATCGCTTGGAGACGCAGGAGCCACTTTCAGTTGAACTGCAGAT

TTACAGAGATCAAGGGATGAAGTTTCGGCGGCTCTCCGGGCCAGAGAGTC

TTTTCCGATGGAAACTACAGGAAGTTCTTTCTCTTGTCTACGCTCAGTGT

GGATGGTGAAGGAGTATTCTCCTTGCTGCA

>TSAU.R67.esd 658 0 658 ESD GOOD: 62-460

TGGCGGCGCTCATAACTATGGATCCCCGGCTGCAGGAATTCGCACGAGGG

CGAATTTTTCTCTGAGCACATTAATATGATGAATAGTTTTATGGAGCCGT

TCACCATGAATATGGCCAATGTTCGTAGGGCCGAGTCGAGTCGTCCAGCG

GCAAATCCAACCACACGTTACTCGCACCCCGTGCGCCGAGCGAACACACA

CCTATCCGATCGAAGCGGGCCCCATGTCTTCTCCTTCATGTACACCTCTG

GGGAACATGATATCGCCTAAAGATGGGCTTTCACAGAACTTCGCTTTCTT

CTCCTCTTCTCCCAACGTCACCGCAANCGTCCACCACGGTGGATCAAAAT

TCACTTTCGGCGGTGGCAGTGGTAATGTACCCATCAAGGGCACTTTTCG

>TSBQ.R77.esd 913 0 913 ESD GOOD: 99-461

GCACGAGGGTGTAATTCTAGCCTTCTCTCCCCTCCCTTTTCTACGTGGTG

AGCAGGCATCACTCCTCTCAGGTCTGTCACTTTTATCGCGCCAGTGGAGG

CGCTTCTTGTCTGGCATTTTATTTTTTCACATTCGATTGCACGCTCCACC

CATCTANTTGTGTGTGTGTGTGTGCGAGTGAGTAAGTAGAGTGTCCATAT

TATTTGCGAAGAAAGAAGAGACTCAGCCGCCATCTAACTTTGCTGCCACT

CTTTGTCATTAACACATTCCCCCCATCTCGCCACACCACATTGCATCACA

ACCTACCAACATTAAGGTATTTACAATCTCAGGCCTCTCTCCTTCCCTTC

TGGTCCCCCTTTG

>TSBP.R95.esd 683 0 683 ESD GOOD: 100-369

GCGACCTGCCACACTCCTTATCGAACTCTCTTCCCCTCATCTATACATAT

TTTCATTTTCTCTTCCATTTTGGAAACTACTCTCTGCTGTCTTTCCCCTT

TTTTTTCAATGCAAACACTGCGATTTATTTCATGCCTACACTCAATGCTA

AAACACTCTCATGGATTATATGTAACAAATAATTTGGTTAACTGAATTGA

TCGGTTTTTGAAAAGAAAAAAAAAAAAAAAAAAAAAAAAAAAAAAAAAAA

AAAAAAAAAAAAAAAAAAAA

>TSBY.R45.esd 749 0 749 ESD GOOD: 103-613

GCACGAGGGGCCTTCCTCTGCTGGGTCTCCATCCTCGCCTCTCGCCAGGC

TGAGGGCAAGTCGGCCAACATTCGTGATGTCATGCGCGAGCACTGGAGCA

AATATGGACGGGACTTCTTCACTCGCTACGACTACGAGAACTGCACAGAA

GAGCAGGGTAAGCAGGTGATGGAACGCGTGAAGTCCTTGCTCGCCGATCC

CGAATTCGTGGGTCGCAACTACACTACCAATTCCGGGGCCTGCTTCAAGG

TGCTCAGTATCGACGATTTCTGCTACACCGATCCAGTAGATCATTCCGTC

TCCAAAAATCAGGGCATTCGCATAATGCTGAGTAATGACACGAGAATCGT

CTACCGAATGAGTGGAACCGGTAGTTCCGGGGCGACAATTCGTATCTATG

TGAACACCTTCTCTGCCGACCCGAAAACCCATGACCTTCCGGCTACAACC

TACATGGCACCGCATATCGAGCTGGCTCTGGGACTGTGCGGGATTCAATA

CTTCACAGGAC

>TSBH.R40.esd 643 0 643 ESD GOOD: 96-305

GCACGAGGGTGGGCCCCCTTCCTACATGGTTTTGGGCAGCCTGTCAGCAG

ACTCGTCATAATAGTCCAGTGTGCTTGAAGTCCTCTCTGCATTTGCACTG

CACCCTGGTGGGAGTGGATTGACGATGCAGCGGCCAATGGCGGTCAACAA

TGTCCCTCCTCCAACTCTGCCACGGCAGGCGGTCACCTACTTGACTCGAG

CGTCACCTGT

>TSAA.R36.esd 481 0 481 ESD GOOD: 103-481

GCACGAGGGGAGAATCGGGAAGAGAGGATATGTCTGCGAAGAGCCCCATT

CAGGGTTGTGGAAGGGGCGATGATGATGAATGCAATAGTGCTGATGGATC

GTCGGTGAAAGGTGGCGATAGAAGCATAACATACTACCCCTTTCAAGGAC

CAGTCTATCCGGACTGAGGGAATGAACGGAGAGGGTGTGGATCGCCTCTT

TCGATTACCCAAATATTCCACTCTTCTTCACTCTCCAACGATGCACTTTC

ATTAATATTCCAATAAACGTCCTTCTTCCCATTCAAAATTAAAAAAAAAA

AAAAAAAAAAAAAAAAAAAAAAAAAAAAAAAAAAAAAAAAAAAAAAAAAA

AAAAAAAAAAAAAAAAAAAAAAAAANNNN

>TSAS.R13.esd 745 0 745 ESD GOOD: 103-621

GCACGAGGGAGTTTTTTTTTTTTTTTTTTTACAAGCAGTCACATCTAATT

GCAGAGACTGGAAAATGTAAGTTGTTAAGTATGGCACACGTTTACAAGGC

GACTTATATGGCGTTAACGTCAAGTGAACTTACTGGGGGTCCATTTGGGA

GTCAGTCAAACCTCGCTGAGGGATTCATTGGAAGGTGTTCCCAGTCGGTG

TGCAGTGGTCGAGTTTGTGCGTATCGTAATAACTTTACAATAGGAAAAAG

TTGACAGACCCTGCGACTTGCACAGGCCTTATGAGCTTTGCCTGTGTGGT

TACAGTTCCACATAAATGCGAGCCCACGCTGGGTGTGATGATGCTAAACT

AAACATAAAAGGATCTTTTAAGGCACCACAAGTATTTTCCCATGCATGCA

TTGGTATGCAAGTTCCTCATGTGGTATCAGCCACAAAGTCCCGNGAGATT

GGAGATTATTACTGAGTCTTCCAACAAGGAGGATATTCTTGCTACAAACG

ATCACAGCATGAAGCGAGC

>TSAO.R6.esd 567 0 567 ESD GOOD: 99-448

GCACGAGGGTTTCAACAATAAATTTCAATGTGAATAATGGGTATCTAGAG

GGTCTCATACGTGGGTTAAAGGGTGGGATTCTCAAACAGTCGGACTATCT

TGTACTAGTTCAATGTGAAACCTTGGAGGACCTAAAGCTTCACTTACAAG

ACACTGATTACGGAAACTTTTTGGCTAACGAACCGGGACCACTGACTGTT

GGGATCATTGAAGAAAAAATGAGGGAAAAGCTTGTGAGTGAATTTAGATA

CACTCGGAACCAGGCCTTTCGTCCTTTGTCGGTGTTCCTCGGACTACATA

ACATATAGCTATATGATTTGATCAACATTGTTCTCCTGATTACTGGAACT

>TSAI.R14.esd 411 0 411 ESD GOOD: 115-411

GCACGAGGCTAACTTCGGTCCCTTACTGGTCATGTCTTGGGTTCGAGCCA

ACGCAGTCTATGCGTTTAAAAATTACCCTCACTTTGTGTACGGGATTCCT

CTTGCTTCTGCGTGTTTCTGCTATTCTGTATACCGCCACTTGTGCCGTAG

AGCCGATGGCCTGGAACGTACATTCAAATACAAAAATGTTTATTCGGTCA

AACGTCGTGAAGACGTGGTGGTGACCGATGAAAACCGGGATCTGTTCAAT

TGATTTGATCCTTTTAAAAGTTGTAGTTGTATTATTAAGGGTGANNN

>TSAB.R11.esd 704 0 704 ESD GOOD: 94-646

TTTCTCTGATGCAAAACAGGGTGACGAAAAAGCAGAGTATGAGCACACTG

GCGGTAATAAAGAATACCGGACGTAAGCCGTAGCTATCGGCGAGCAGGCC

GCCAGCCATTGGGCCGAGCAACGCACCACTAACGCCGCCTGTGGAGAGCG

TACCCAGCGCCCAGCCGCTTTTATTACGCGGTACTTGTGTGGCGATAAGA

GCATTAGCGTTGGGGACAAATCCGCCAAGTAACCCAAGAAGCGCCCGCAG

GATCAAAAACTGCCAGATATTTTGTGCCAGCCCCATCAACACCATCACGA

TGCCCATGCCGAGGGCAGAGCGTAATAGCATGAGTTTTCGGCCTTTACGG

TCGGCGAGTCCACCCCAAAACGGTGAGGCGATGGCCGAAAATAAAAATGT

AATGCTGAAGACAATACCGGACCACATATTCAGGGCGGAGTGACCGGTAA

CGCCAAGCTGCTCAACGTAGAGGGGTAAGAAGGGCATTACCAGACTGAAG

GCGGCACCGGTAAGAAAACAGCCTAGCCAGGCGACGATCAGGTTTCGTTT

CCA

>TSBW.R15.esd 466 0 466 ESD GOOD: 97-202

GGCACGAGGGTCAGATTGACCTCTCACAATTCGGCGAACACCTCCTCGAT

CCCAACATCGACCATCTCAAGACTACTTTTGAATAGTCGTTCCTCATTAC

CATCCC

>TSBE.R18.esd 479 0 479 ESD GOOD: 98-339

GCACGAGGTAGCTGCTTTGTACGTGCAGACTGCTTTCCGTAGTTGAATTG

GAACATCCTGATCAAATGTTCCACACATGAGCCTTCCTCCTTCCGTGTAT

TCACCAAAACAATGAAAGGAGTATAAGTGCAGAAGTGAAACTGGCAAATG

GCTTCAAAAAAAGCAGGGGCTAATTGTCCCAACCAATTAAGCCTGGATTT

CGGGTTCCTGCCCTCACCAAAAATTCCCAGTATTATATTGGT

>TSBG.R83.esd 594 0 594 ESD GOOD: 111-498

GCACGAGGCAACGTTTCAAATGGTGACATTTCAGCGCCTAGAAGACATCT

GAAGATTAAAGGATCCAGTGTGGTTAAGCATGCAGAACAGACTGCGGCTA

CTGGAATGGTCCGAGTGGAAACTTCATGACAGCGGTTAAATTGCGGACCT

TCTAAGTAGCCTGTTATTGTAACTGACTAAAACTAACAGTTAACTCCACT

ATCGCCATCACTAAACTTCGTCCAAAAAGCATACTAATTTTTCTTTATTT

TGCTTAGCACTGAGAAACAGTAACAAATAGAGCGCTCAGTCCTTCGTATT

TTGGCGTAAAATATGGGAATATGACTACTTTTGATGTCTGCATTACCGAT

TCCATAATTCTAAAAAGTTCGATTAGGTTCCAAGAAAA

>TSBR.R54.esd 492 0 492 ESD GOOD: 99-410

GCACGAGGATTTGATGTCCATCCTACAGGGAAAATTAATCAAGGGCTATC

TCAGGTCTCTCTTATTTCATTGAGGTTAGGCTGGGATACATAGTCTGCCT

TTCGTGGATGATGCGAAAATTGATAGAGCTATGAACAAACCTTGAACCAA

GTGAGTTGTGGAGTTGGATTTCCGTCAGCGGTGCAGGTGAGTTTCACAGA

ATGTCCATCTTGGATGAAACGGTTTCTCAATGACAGTGAGAATCTCGGCG

GGAATTCTTCCGGCAGAATAACCTCGTACTCCTTCAGAACGAACTTCTTC

CTTGGCATCACG

>TSAY.R83.esd 514 0 514 ESD GOOD: 101-452

GCACGAGGGAGGCGTCGTTGGCGCAGGCTAGGTCAGCGACTGAGGCGGCT

CGACGATTGATGACAAATACTGACGGGGATCGGGAGGATTCCTCGGACCA

CGAAAGTGATGCCCTTCGTGATGAAATTGATGCCCTCAAGGCTAAACTGG

ACACCGAAGTGACCGCTCGTAAATCGGCTGAGACCCAGATGGAGGTCATT

AAAAAGCAAGCCGAACAGGTATCAAAGGAGTACGATCGTGTTTCAGCCGA

ATGTCAACAGCTTCAGAAAGAACTGGCTGCTGTGACTGGCGATGACAGGG

ACCAGAAGAAGGATTAGGTTCGGCGTTTACACGCTTTAGTTTCACTGTTC

AA

>TSBH.R47.esd 563 0 563 ESD GOOD: 99-532

GCACGAGGCCTTGTTCCTTTGCCGCGAGAATGGCGGCCAACAGGTCATGT

TTTTCTGGCATCTTCATGTCCATGGGCCGTAAAAAAGACGCTGGTATAGG

CTTATGAAAATTAGGCATCACGTCTCACATATGTAAAATAGATCAAGGAG

GAAGAGGTCAGGTTGTTTGCGAAACAAAGAAACCAATCAAAGTGTACTGT

GTTTTTTTCCTGTACGCAGGTTCACGTTGGTCGGTGATGATTGTGAAAGA

GACCTCTGTACAAAGTGGAACCAAAAACTTATCTTAAACTGAGTATATTG

CCGTCTCAGGCAATGTAACTGGAGGGTAAGGCGTCACTACCTCGGTCGTT

TTATCCACCGATTGCAGATGAGGCAAGAGATAGTCGGGAGGCGTAATCAG

CACCAAGCAAGAGGGATGCGAAGGCGGTTGTGAC

>TSAK.R2.esd 530 0 530 ESD GOOD: 99-432

GCACGAGGCTTCGCTGCAAAGGCCTCTATGGGTCGGAAGAATTCATCGAG

TCGCCTTCCAATTGACAATTTTCGGCGTGCCATCGGTGATAATCAGCGTA

GAAGACAGATGGATCAAGTCGCGCGCCTGAAAACTAGTATTGACAAGAAA

AATGAAAATAAACCTTCATCTGGGCGCTCACAATATCATTGTTTCTTGGA

ATGGTCGTTTCCGTTGCCGTCATCGCAGGAATGAAATATGTTTACGAGTT

CCCTGTGAGTGTTCCGATGCGTTAGGTACACTGAACTCAGAGTGGTAGTC

CTTACTCATCTTGTCATTTCAATTGAATTTCTAA

>TSBL.R46.esd 619 0 619 ESD GOOD: 94-333

GCACGAGGGTTGACGCCCGCATGACTGATGGAGTTGTAATGGGTGCGCCT

TTTTCGTAACAGCCGAGGGTTTTATACTTTAAGTTTGGGTTCCCACTATC

AAAGGCAGGCTTTTCCCAGGTCATTTCCCCGGCGGATTCTTCGCCACTAG

CCTCGACACCAGAGCCCTCCTGCAACTGCTTGGTCCCGGACATTATTCAG

GGGATGAATCCAGCAAGAAAAAAAAAAAAAAAAAAAAAAA

>TSAA.R14.esd 646 0 646 ESD GOOD: 100-466

GCACGAGGTGCAAACGACTCATCCTATACGTCTTGGTCTTGCTCTCAACT

TTTCCGTGTTTTACTATGAAATTATGAACAATCCGAAGCGAGCTTGTGAG

CTTGCTAGAAAGGCTTTCGATGACGCAGTTGCTGAGTTGGATACTTTGCC

AGAGGAGAGCTACAAAGACGCGACTCTCATCATGCAGCTTTTACGCGACA

ACCTAACCCTGTGGAATAGTGACACTGGTGATAACGATGCCGCTGAACAG

CCGAAAGCTGAATGAATGAAGAAACGTCGGGGTTAATAACTCTGAACTCG

CTTTCGACAGATCAGTTAATTAAATAAACTTTCTTACGAGAAAAAAAAAA

AAAAAAAAAAAAAAAAA

>TSBE.R90.esd 639 0 639 ESD GOOD: 103-543

GCACGAGGCGTGCGAGTAGTTTCGCTTTCAATTTAGGATGCCTAGGCACG

TTGCTTCGGTTCATATAAAAAATCTCGCTGATGGAGTTCGGTAATACATT

CTTTTTGCTTGAGAAGTCTGTGTCGGGGTTTTGTTTACCCCATTTGTTTG

CGTAGGTTTAGGGCCGGGTCTCTTTATTGTTNACTGTGGCGTCAAGTTCT

GGTTCGCGGAACGTGAATTCATCTCATGTTGTCATCTCGGCTTTAATTTT

CGCGGGTGCGGTGGTTTACTAGTTTGCTTCATTAACTTCATGAATATCAG

AAGGGGATGGTGATCCTATCTGNTTTCTTGGTGTGTGCTGGCCTGGTTTA

CCTTTTCCCTTTTAGCCCACGAGATCTGCGCCACGCCTTTTTGGGAGTCG

GCCGTGTTAATCGTGGTCACGGTGCCTGTGAACTATCACAC

>TSAQ.R76.esd 761 0 761 ESD GOOD: 91-203

GCACGAGGGACTAGTGATCGATTTCCGTCTCAATATCAGCCCCCTTGAAG

CCATTTTCTACTTCTAGTCTGCTGAATAATTGTCGTTTAGACCCTTAGGG

GCGGCTCGTTTGG

>TSAV.R33.esd 766 0 766 ESD GOOD: 101-636

CACGAGGATCGTGTTGGCAGGCCAGGAAGGTCCCATAGTGACCACGGTGC

GCGGTATTCGCATGCCAGCGCCCATGTCGGAGTTGCGTGTGAAAGGAACT

TACAAGCATCTAAAAGAGGTGATGGGTGCACAGGGGGTCAGACTTATTGC

TCGCGACCTCGAAAAGGCATTGGCGGGGCTTCCATTATATGTCGCTAGGG

ATTTGGCCGAAGAGCTTTACTTTAAGGATGAAGTGAGTAATGGCTTGAAG

GCGGCGCTGAAAGCAATCGCCGTCAGTCCTGTGGGCGTCTACGTTGTGGC

GAGCACTCTCGGTTCTCTTGAGTCCTTACTCACCTACCTTAAATCTGTTA

ACATTCCGTACTCGGGCATCAGTATTGGTACAGTACATAAAAAAGATGTT

ATGAAGGCATCTATTATGGTGGAACGAGACAAGAAGTGGGCCGTCATTCT

CGCCTTTGACGTGAAGGTAGACAGGGACGCGCAAAAAATGGCCAATGAAG

TTGGTGTGCAGATATTCACCGAGGACATCATTTATC

>TSAK.R13.esd 792 0 792 ESD GOOD: 101-463

GCACGAGGATCACTTCCACTAGCAGCAGTAGCACCAAGCCGTAGGCTGCA

TGTTGGTCTCTTGCGATCGTAAATGCGAGAAAGGCAACAAGGCTCTCCGT

CTGTAAATCACTCTATCTCCCTTCTCCCCCAAACTCGAAACTCCCAAATC

TCGTACCTCTGTTTAGATTTCTGCTGTGTGTATGTTTAACCAAACAGCGG

TTCCCCTCTTTTCTGTGACATATTTTCCTGGACCTTTTTATGTTTAACCT

TGGTAGAACATAACTCTTCATGTATGCGTTGACGCTGGACACACTTATCA

TGAATATGTGTGAGAGAGTTCCATATTATCAGAGCTAGTGTGTATTGAAA

AACAAAAAAAAAA

>TSAS.R81.esd 525 0 525 ESD GOOD: 94-456

GCACGAGGGTTTCAGGGTCTTGCACAGTTAGGCCAGTTGCATCAACTGGC

GGCTAAGGGTGTGGGCGATCAACAACCGCCGACCCAGCCCTACAACAGCA

TGGACTTTTGAATGTTTGTGTTTGTGTGCGTATGTGTCCTTGCTTATATA

AGTTATTTCGTCTACCCACCTACTCGTTCATTCATCAACTCCGTATTCGC

CTCCTACTCCTTCACCTTCATTTCTTTATCTTTCTCTATTTTTCCCCATC

TCCTTGCTCCTCATACTGCTTCTTTTCGTGATCCTGCAAATGCACATGCT

ACTTGTGTAAATAGATTCACTTGATATTACTGTGTATGTCGCATTAAAAA

AAAAAAACTCACC

>TSAH.R47.esd 623 0 623 ESD GOOD: 97-573

GCACGAGGCTGGAGGCTATGGAGTTGCATTTTAAGTTGGTTGGTCTTGCA

CCTCCCCGCGGCAGTTTGGCGTCATTCAAGCTGGAACCGCACTGCCAGAT

TCTCGGTGCAACGACACTTGAAAAGGAGAATGCGACGACGGTTAACGCCT

ATCGATACGCATCGGACATTAACCTTCCCTGGCGCATTTACGCCACGCTA

CGCTGGGCTCTGGTCTCGTGTGTTCGGCGATTCGGCGATCATCTCAGAAC

AATACATATGGATGCCAAACGCAAAAGAAGACTTCAGCATTTATGGGAAG

CGATAGCGGTGCCGGATGACGGATGTTGAAAGAACAGATTTCACGAAGAG

AGGGTAATTATAGCCACCGCGTTGCGTCTTATGTATGCATAAATTTGCAT

AAGCTATTTTTAAGAAGATGCGATTGATTTCACATAAAAAAAAAGATTTC

TTAAGAAAAAAAAAAAAAAAAAAAAAA

>TSCG.R36.esd 694 0 694 ESD GOOD: 106-587

GCACGAGGGGCTGCTCCTTTGCTCTCTAAGGCTGGGCGTTGCCTCTCGAC

ATCCAGTAACTATGCTCAATCGCCGGAGTTTTCGAGCATTTTGGAGGAGC

GCATCCTTGGACAGGCCCAACAAGCTGAGTTGGAGGAAACTGGACGTGTT

CTGAGCATTGGTGATGGTATTGCTCGTGTGTATGGTCTGAAGAACATCCA

AGCTGAGGAAATGGTGGAGTTTTCTTCCGGTCTTAAGGGCATGGCTCTCA

ACTTGGAGCCTGACAATGTGGGTGTTGTCGTCTTCGGCAACGACAAACTC

ATTAAAGAGGGCGATATTGTCAAGCGTGCCGGTGCTATTGTCGATGTTCC

TGTCGGCGAGGAACTTCTTGGTCGTGTTGTGGACGCCTTGGGCAACCCAA

TTGATGGTCTGGGAGCTATTTCCACTAAACAACGCCGTCGGGTTGGTGTT

AAAGCCCCTGGTATCATCCCACGTACCTCCGT

>TSBY.R76.esd 850 0 850 ESD GOOD: 119-283

GCACGAGGCTTGAAGCCATTGCGTGATTTTCATATGCTAATGAGGGTGAC

TTGAAAGCATGTCAATGTGCAGTGTTGCATGTGCTTCTTGAATTTCACAG

TGCCTGGATGGACTCTGTTGGTGATTTAGAATTAGGGTTTTTTAACGCTA

AAGTAAACAAACTCG

>TSBC.R23.esd 730 0 730 ESD GOOD: 82-597

CCCCCGGGCTGCAGNGAATATCGGCACGAGGTTCCNCCAAATTTTTTGGT

TTCTGTGTGCAGTTATGCTGCTACGTTCGGTGCAGCGTGTTTCAGGCCTC

TTTACTGCTCAATTCATACGGGCTAGCTATGCTCAGAAGTACACGTCGCT

GAGGTATTCTACTGATTCTTCAAATGTAGTGCAGTTGACATTTGAGTGGC

TTTGCAATGGAGTTCGGAAAACGGTTCCTGCAAATAAAATGAACTTGACA

TCGATGGATTTGGTGCCTGTGGGGGTGAACTTGCTTGTTCAACCTGCCAT

CTAATTTTGAGTGATGATGTTTATAACAACCTTCCGAACCCACCCTCTGA

AGAAGAATTAGATTTGCTGGACATAGCTCCAATTGTAACAGATACCTCTC

GACTTGGTTGCCAAGTTGTGGTTTCTGATGAAATGGATGGTGCTGTGATC

AAAGTGCCCGAATTCGTCGTCGATACTCGTGCCTAAACAACCCCCTCGTA

GAACGATTCCTCTCCG

>TSAG.R53.esd 427 0 427 ESD GOOD: 97-318

GCACGAGGGAAATGTGCCAAGGACGAGTGATGCGGTGGGGCTTTGCGTGG

ACCTTCATCCCAAACTTCGTTTTTCCGGTTTCTGATTTTAGACAAAAACG

TAGATATCATCGACAGCCGCTCACATACATTCTCCCGGCGAGTGTCTCAT

GGTCTGGCTAGGTACACGAAGAGGTGTCTTCTGGGAATGGCTATCATCGG

GAATTTAAGGAAGCTCAAGATG

>TSBI.R27.esd 407 0 407 ESD GOOD: 104-310

GCACGAGGCTTCAACACTGGATGCCTGAACACTTTGCCCGTCTGGCGCGG

TATGACTCGGGTCTCACCTTCACCCAACCCGTGGCTGCCAGTGTGGCTTC

GCGACTGAAGCAGTGGTAACTGCCTCACCTCTCCCCTTCCCTAAATCGTG

CACATTTCCTTGCATTTCGACCCTGTCATTTTCATCGTCGTGTCTCCACT

GACTGCG

>TSCF.R96.esd 372 0 372 ESD GOOD: 94-372

GCACGAGTCACCTAAATCCTGAGCTGTAGTGAATAGGGTGATCGACAGGA

GTTGCGTTTGAGTTGGAGTTTATCAGAACCTTCTTAGACTTTACAACGGA

GCCATCCATCGCACGACCACAGCCGAACCGACGACGTATCACCAAACTCG

CGCAGATCATTCGAGCCTGAGACTTGTGGCAACCTCTACATCAAACGTGT

CTACCTTTTATTACTTCCTTTTCGCCAACCTTTAGCACCTGACTTTGATT

GACTACCTATTTTCTTCCGTGTCAATTNG

>TSBB.R23.esd 518 0 518 ESD GOOD: 103-518

NNGCACGAGGATATGGTTCCCTTCCCACGTCTTGGCACTTCTTCGTNNGC

CCNCGGTTTCGCGCCGCTCGCCAGTAGAAACTCACAGTCCTACCAGAGCT

GTACGATCCTTGAACTGACCCGACAGATGTTCGACGCGAAAAACATGATG

GCAGCGTGTGACCCTAGCCATGGTCGTTACCTCACCGTTGCTGCCATGTA

CCGTGGACGAGTGTCCATGAAGGAGGTAGAGGATCGGATCCTGGAGATTC

AGACTCGTAATTCCACCTACTTTGTGGAGTGGATCCCGAACAACGTCAAG

ACCGCAGTGTGCGATATTCCACCAATCGGCTTCAAGGTAGCTGGCACGTT

CATTGGGAATACCACGGCGATACAGGAATTGTTTACCCGTGTCAGTGACC

AATTTTCAGCCATNGG

>TSAW.R78.esd 724 0 724 ESD GOOD: 101-224

GCACGAGGCGCGCTTTTCGGTACGTGAACGCGGTGACTTCAGCGCCGGGA

CCAGCAGGCGTGATGCAACATCTCTCACTCAGGTAGAATTTCCCTCCAAT

CAACCCTTATTCGCCAGTTTCGCG

>TSAD.R15.esd 705 0 705 ESD GOOD: 123-536

AACGGTGGCGATGTCTCACTCAGCGTCGCTACATCTGTTGATAGACCCTC

ATCTTGTGGCTCTGGGGCTCGCGACAGGCGAATCTGCGTCTGGGACGTTA

NGGCTGCTACATGCCTTTTCATACTGACTGGGCGCGACAACTGGGTGCGT

CAGTTGGTCTTTCACCCGCATATCAAATACTTGCTCTCAACATCTGATGA

CCAAACGGTTCGTGTGTGGGACTTAAGGAATCGTCGTTGCCACAAGACTC

TCGAAGCACATTCACACTTTGTTACTACGCTGGATATTCATCGAACGGCT

CCCTAACTCATTACGGGTAGTGTTGACCAGACGATTCGAATTATGGGAGT

GCCGTTGATGTTTGCATTCCTTGTGTGGGTAAGTTGAGTCATTTCAGTCC

CTCTTTTGTCCCCT

>TSAY.R79.esd 628 0 628 ESD GOOD: 97-568

GCACGAGGCTTGATTCAGGCAGTGACTTTGCAACATTACCCCTTGAAAAT

GAGGGTCCGCATAATGGAGCAACTTCTGCTTCTCTCGGTCATCGTTGGAC

GTTGGCTGCTTCCCCGCCTCGGGATAAGTCGCGATCAATTGTCTCAGTTG

CTTCTCATCAACATTGGCAATGCAGCAGACATACTGGAGCTCTTTGAGGC

CTTCAACGAAGAAGCCGTGCGAACAAATACCTCCCTCAATGTCTGTATTC

TCTGCTTATGGCAGGCCTCCCTTCTTCAATTCTGCTTCAATAAGACGGCC

ACATTGGAACGTCGCAAAAATACGATCCAGCAAGTGCCCCCGCTTCTTCG

ATCTGTCTCAAGAAGCACCACTTTTAATGACCTCGGACATATGGAGGCTA

TTCAAATCCAGCCGGAAATTCTTTTGTCCCCTCCTCTAAGGCGCCACTTC

TGTGATTGCTGTTCTAGCCGTT

>TSCA.R88.esd 607 0 607 ESD GOOD: 105-605

GCACGAGGCACCTCTGGAGTCACTGACTCGACTACCGAAGAAGTGGGCAG

TTGTGTGAATGAAACTGACAGCCTACTCTCTGCACTTCCCTTCATCTCCA

TTGATGTCTCCTCCCAGAAACCCACCTACGAAATTAAGATGGAGATCAAG

CTTACTGAAGTGGGTTTGATGCCAAGCGTCCAGGAGGACCGACGCCGGTT

CGCAGTGTGGACGGCGTGTCGGGCGCAGCTGTACATATTCCAGCCGAGCA

ATTTGCAAGTGCGCACCCAATGGGTTCGTGCCATCAATGATCTCCTTTCG

GCCCAATTGAAGCGCATCCGTGACGATGTGAATCGGCAACATAACATTGT

CACGTGTGCGTCATCATTGCTCCAACCATCGTCAAAGTCGTCGTGTTGTC

AACGCTCTGACACCCTCCGTGACGACACTGATGAACAATTCGATGAGGAC

GCCGACGTGACCGTCACGGACTTTTAAGAGTTATTATCGACTGCAAGAGN

N

>TSBA.R5.esd 620 0 620 ESD GOOD: 101-605

GCACGAGGGCATCGAGGTGTTGTGGAGGTAACTGCGCAAATTCCAATTTC

TCATTTGAGGACCCAAAGCATAGAGGAGTTAATGTAAGGCATAGGTTTCT

TCAGAAATGAGTGGTCAAAGAGATCCAAGCATATTCAAACTGTTCAAACC

CTACGACCAGCTCCTGTTTGAATGTACTGGTCTTTTGCATTTTAGTCAGT

TCTCACTGACTTGTGTTCAAGTATCTGGTTACACAATGTTCCCGCCTACA

GCGCAGGTCCAGAAAAAACGGTACTTCGATTTCGTGCACAACAAAGCACT

CAGGACGTACCATTTGCCGTGCTTTCCGCGTTCAAGCACCACATAGGGAA

CATTTCTAGAAAGGGTAAACTGATAATTGATGCTGAGAAATCCCTGGCCT

GGATTGAAGAGCATGAGAGTGAACTAAATCAGATTCAAACATATTTCACA

TGGGAGAGAGGCCTAGTGGCCCTTGCAAACGAAATACGCATGCAGCACGA

GTCCA

>TSBB.R76.esd 784 0 784 ESD GOOD: 94-203

GCACGAGGAGCACCTAAATATCCCCATATTTTTCTCGTAGGCTTTTATGG

GTCACGATTGTTCGCTGACTTGTGCCGATATGAGGAGAACTCTCGCGTTA

CTCGAGTCAC

>TSAV.R24.esd 674 0 674 ESD GOOD: 102-581

GCACGAGGGCTACTTGGATTTAGGCCCCTGAGTACCTAACGTTACACGTC

AACGGGTTGCCTATTTGGAGAGCCAGTTGTCAAATATGCGCCGACTTAAG

AGTTCCATAAGGTAGACAGCAAACCGCAGTAGGAAGGCGGTCTACTCTAA

ACGAGCGCGCTCTTTACATTGTGTGAGGATGAAGACGAGCGTGGATGGTA

AACAGACCTTGTGTACAGGTACGCGTGTGATTTGCCATCGGACGGCTGCC

GATCAGACTGCTGTCTTGCCTGCGCGATCGTCCTTCATCTCCATTCGAAG

AAACGCGGATGTGTGTGTCGGCGAAGAGGTTTCAGCCGCTGCTACTCGTC

CGAAATGCTGCCGATACCGTATAACTACCAGTCGCCCGCCTGTCTAACCA

TCATTCATCTGTGTGCAGGACTGATTTAATGCTCCCCCCTATGCAATCGC

AATAAATTCTCTTCTGTAAAAAAAAAAAAA

>TSBT.R88.esd 779 0 779 ESD GOOD: 106-464

GCACGAGGGTTTTTTTTTTTTTTTTTTTTGAACAGACAACAATACTTATC

TTCATTCTAACACATTCAACAAGCGAAGGGTAGAAAACTACTAGAGAAGG

GGAAAGGAAGGAGTGATAGGTGGGGAAGGGAACGCGATGAACGAGATGTC

AGATGTCCTGCCAGCAGAGGCGTCACTTCCGAACGAATACCTCAAGGGGC

AGATTTTATCCATGGTGCCAAGACAGTCAATCACTATGGTGTTCAAAATG

CGTGTGCAGGGGGACAGGATGTGAAAGGGTAAGCAAAAATTACTCCGTAG

GGCATTCACTCTCCTTTGGAGNTGGGACCAATACGAAGGAGGGTAAGGGA

TGCATTCCT

>TSAM.R37.esd 508 0 508 ESD GOOD: 94-377

GCACGAGGTTTTATAATAATAAAATAGCCAAGTTTTATCCGTCAATAAAA

ATAGTTTTTGTCCATGGACGGTCATTAATTTCGCCGTATGAAAGTGATAT

GATCTGGTTATTGGTGGAGAATAAAATACTGTCTTGATTGTTTTAGCTCT

CAAAATGCATATATCTTGGGTTGGGATTGACAGCTTTCATCCGCCATTGC

TTCAACCGACCTCTTTCGCGTGGCGGGCGAATTTCCGAGAATTGAAACCG

TGTCATTGCTTTCAGCACCAGCAGTTCCGCTGGC

>TSAL.R57.esd 647 0 647 ESD GOOD: 135-448

GCACGAGGCAGAAGGGCATGACTTTTGGTGGGGTTCGTCATGCTGCCGAT

ATCAAGGCTGATGACATTAGTCTCGAAAGTCAGGGCATCTGTAGCCTCCA

AATGGGAACCAACAAGTTCGCTTCACAGAAGGGTATGTCGTTTGGTTCGG

TGCGTCACGTTGCGGATATCCGTTGTGATGATGTCACCAAAAAGGGCCAG

GGCGTCATAACTCTCCAAATGGGCACCAACCAATGCGCGTCTCAAAAGGG

CATGTCAATGGGTGGAGTGCGTCACGGTGCAGACATCCGATGAGACAACA

TGTCCCGTGAGGGC

>TSAK.R30.esd 602 0 602 ESD GOOD: 100-302

GTAAACTGCCGCCAATTGTTGCTCTGGGATGAAGTCGAGACACATTTCCC

AAACACTCTGTCTTTTAGAAAGGACGCTCCCGAACGTTTCCCAAAGCCCG

AATTACAGCCGGGAATTACAGCCGATTAAAAAAGAAGGGAGAGACGTGAG

CTGAAAGCAAACAGAGTACACAGCAGATAACTAATACATCGAGGAGATTT

GAG

>TSBC.R5.esd 613 0 613 ESD GOOD: 103-469

GCACGAGGCCCTCCTTCTTCGTCTCTCCCTCTTCCTGGCAGTAAGCCTAA

CCGGAGCTTCTACAATCGGGCCGCGAACCTCTTCAGAGTTGGAGAACACT

AACACTGAGACACCAAATGTGCGCAAAGACTTTTTCAGTCTTCCCCATGG

TCCTCTCATGCCAGCAGCCTTCATAAAGTCTCGTGGTGCTAGAAACACGC

CTCGTTGTAACGAGGAATCTCCCTACGTANACTTGGACAATGAAGTAATC

GGTAAAGAANTTCCCAAGGTCCACGGATCGAGAGCAAAGTTAGGGAGATT

ATAAACACTTTTGTAGAGACGCTAAAGCACGACAAATCATGGTTTGTCTC

CCTTCTCTTCCAAATCC

>TSAZ.R63.esd 708 0 708 ESD GOOD: 102-411

GCACGAGGGTGACGATGAGTATTCCGATAGTAGCCGTTTCAGCTGGTTTG

GTGAAATTTGTGATGATTTCCGTCTGCCTTTCAAACGCCTTTGTACAAAA

GAGTTTTCACTCTCCAATTTGGTAAATGAGGAATCCGAACTTTACAAAAA

ATCGATAGAGATTCATCGCAAATGAAACGTTCGAGGGCAGCAGTTAAAGT

GGTTCAAGAATCGGTGCTACTTCACTTCGTGGGGATAACTTTAAGCATGG

GTGCGGATCGTCTGGCCGTAATGCACCCGTATGAATAAAGGCATTCGGAT

GAAATCGAGT

>TSBO.R35.esd 896 0 896 ESD GOOD: 111-613

GCACGAGGGCTTACCGACGAGGAGGTTGACGAAATGATCCGCGAGGCTGA

TATCGACGGTGACGGTCAAGTGAATTATGAAGAGTTCGTTACTATGATGA

CGACGAAGTAGTCCACGTCTTCAACTCATGTACCATCAAGTCGATGTGAC

ATTCACTTTTTCTTTCCACCTAACTGCGTTCGCTTATCGTTCCTCGCACC

TTCCATTGCATGGGTTCCTTGAGAACACTTCTATCACTTACTTGCTTACT

AATATCGACCACTGGACCCGCGAAGGGACCAGATCCCTGGTTATGATGTG

CGGTCTGTTCCCATTTCTCCCTAATTTTCCGCCTCCACTGTACATTTCCG

TCGCAAGCGAATCTACCTAAGTAGATCCCACAGTAAGGTTTTGTGTTTAG

TGGTCCTTACCAGCATCGATGGCTTCTCCAATTATCCCTTCATCAAGTCA

TCAACCTCTCATCTTTAGAAGTCCACTCAAAAGTTGGCTTTCGCTTATGC

AGA

>TSAF.R45.esd 781 0 781 ESD GOOD: 141-657

GCACGAGGGGGCGAACTGCCTCAAGGTTTCGCGCGTCTCTCCGCGGTTTA

TGGAGGCACCTACATGCTGAACAAACCCTTTGAAGGGTTTGTCATGGAGA

ACGGCAAGGTTGTTGGTGTGAAATCGGAGGGTGAGGTGGCCAAATGCACT

AAGGTTATTTGCGACCCCAGCTATGCGCCTGATCTTGTTCGACAGGTTGG

AAAGGTTGTGCGTGCCATCTGCATTCTGGATCACCCTATCGACGGCACGA

ATAACATCACCTCGTGCCAAATTATTATTCCCCAGAACCAAGTTGGCCGA

AAGCACGACATCTACATCTCCTGTGTGTCGCACGACCACAACGTGGCGGC

AAAGCCCTTCTTCATCGCCCTCGTTGCCACCACTGTGGAGACCCAACAGC

CAGAACGTGAGTTGGTTCCCGGCATTCGTCTCCTAAGTCACATGATGGAC

TGTTTCGTCTCCGTCTCCAACCTCTACGAACCTATCAACGACGGCAAGGC

GAATAATATTTTCGTAT

>TSBQ.R4.esd 706 0 706 ESD GOOD: 85-364

CGCCGAGGCCTCGTGCCGAATTCGGCACGAGGCACATTCCTTGTACTCCT

CCAAACCCATCTCCCTTGCCACAGTTCCACGAAAGTCATTCCTTGTCTAT

GACAGTTCCTTACCCTCCACCTCCGTGCCTGTTTCATCATGCTATCGACG

TCAGTTAACCACCTCGCGGAAGATGCTTCGCTGTGAGGAGTGTGGCAAGT

ACTATGGGAATGAGTACAGCCTGCATCATCACATCTGCCTTCGACGGAAA

GGATGTCTGGAAAAAGGGCGATGTGCCCAC

>TSBA.R51.esd 255 0 255 ESD GOOD: 97-221

GCACGAGGGAAAGAAGTTAATGGTTTCCCTACTTGAAGCAGTCAATAACT

GTCAGTGTGTAATACCCAACTCCACTGCGATTCTCTTAAAGGATTGGTTT

GAAAAACACGGGTGGCGTGGGACGA

>TSAH.R11.esd 692 0 692 ESD GOOD: 103-570

GCACGAGGCCGCTTTCAGCTTCGCCACCGCTCCAAGCCCTCGAACTCCCA

CGACGCAACCTCCCGTCTTCCCTTCGCCCGTCACTGCTCCACATCTGCTC

AAAGTCAATAATAACAATGCAGTTGAATCAATGCTTGTACGTCCACAGGT

TCGGCCAAGGCGTCAACAGAACGAGGTAGAGTAGCTGCTCTGTAGACGCA

AGGTCGTTGTTGCGTATCTCGTCTCGTCAACGTCTCTTCTGTCCTTGTGA

GGAAGGCGGTTGATTTTCGCTCCATAGGTTTCTTCCACTTTCGCCCACAT

CACCGAGTGTTACATCTCAACCCCAATTTTGATCCCTCTCTCGTTCCCCT

GCCTCTTCCTCTTCGTCATTTTCCTTGGCATCTATTCAGTTGTACATTGA

CTTCAGTGATACTTGCATATACACACATACACAGTTCCACTCACTCCACG

AAAAAAAAAAAAAAAAAA

>TSBZ.R79.esd 796 0 796 ESD GOOD: 98-589

TGCGGCACGAGGGTGGATGCGAACAGCCATTTCGACAAACTGATATTGCC

AAGAGGAAACCCATACGGACTCACTTTCAAACAGAAAAGAGCTAATTCTA

GTAATTATCGCATATGAGTTAGGTGAAATCGTCAAACATTTTCTTGTAAA

GACTAAAGATTGATTCCTTATTTACCATCGCTTTTTTCGCTTTAGGCAAG

AAGTACTAAACTTATCCTCTTACAAATATAAAAGACATTATATAAAAATA

TTCTAAAGGGTATTATGAATTTATTATTTTGCTAATAGATGTTCCTTGTA

CACCTATAGGTATCAAATTTATAAGTCATTTCATTAAGGTAGGTCTCCTC

TTCATTTAGAGCGAACATTATATGCTTGCCCCAGCTTTACTTTGCCACCT

AGGCAAAACTGATCTCGAAATAATATATTGAAAATAATAACACCACTTAG

TCACGCTATATGGACCAATCAGTTTTCAGCGGATTTTGTTGA

>TSBO.R16.esd 612 0 612 ESD GOOD: 98-374

GACGAAATTGAACGTCTACGGCCCAAGTCTCCCCCTCCACAAACCGCCGC

CTGGCACTCCGGGGTTGCGAGCACTGTAGGGCAGCAGGCAGCGCGAACCG

TCCTCTCACAGCCTAGCGTCACTCCTTCCCTCCTCGGCAAACCGACTGGC

TGTCGCCTTCAGCAGACTCTACCACGGGCACTATCTCATAAAAAAAAAAA

AAAAAAAAAAAAAAAAAAAAAAAAAAAAAAAAAAAAAAAAAAAAAAAAAA

AAAAAAAAAAAAAAAAAAAAAAAAAAA

>TSCB.R15.esd 580 0 580 ESD GOOD: 103-324

GCACGAGGCTTATCTCATTCTTTTCCTTCACAGCCTGTCTCGTGCAGAAG

TACGCCTACGCGCCGCGCTTTGCTACTCACCGCCAGTGTCTGGCCTCCTT

CGGCGCAGAGGTAACGACAGAGTCTTCAGACTCTCAGCAGAGGCCCTCTG

CGATGGAAATGGCGAGTGCAGGCTTCTTTCACACCGGCCGTGCGGACGAA

GCAGTGTTGTGTGCCTGCTTCC

>TSBK.R88.esd 690 0 690 ESD GOOD: 107-500

GCACGAGGGAACAATTTGAAAAAATTCCGTACCTCAAAAAGCAAAAACAA

AACTAGACAAGTGTGTTTCCGTGTGCCATGCGCGTGTTTTTGTACGTTCG

TGCTTGCGTGTGTGAGGGAGGGATCCTATCGCGATTGCGTGTATCTACTA

CATTTCCCATTTTCCTCTCTGTAACATCTCCTTTGCTATTTAAGTCCTGA

TCCTGATTGAGTTTTCATGTACGTGCGTGTGTAACAACAATACGCCGACC

CCTTTGTGTCATTCTCGTCTATTTGTGTTAGAAAGTACCGCTTTTTCATA

GGAGTGTGTGGTGGTGGTACCCTGAGTGGTGTGTACATCAAACGCTGGCA

AGCAAAGTAAAGCATTTCACCAGTCAAAAAAAAAAAAAAAAAAA

>TSBG.R1.esd 676 0 676 ESD GOOD: 168-616

GCACGCGGATTCACTTTAGCAGCACTGGGCTATCATGATCATCACTGTGA

AGAACGACTTTCTGAAGGAACTCTGCCTGTTAAGAGCTCCAGTGAAGATT

TCGGGATCACTCATTGTTAATCTCTTTGAAAAAAATCTCGCAAGACCAAT

GGATACAATGAAAGGGTTCCAATGCCGAAAATCGAATTTGCGGTCAGTGT

AAACTCATTGCTACTAATGTTTGTTAGTAGAACTAATTTTGTAGATCCAT

ACAGCGCAAAAGGCGCACCCAACGATCAACGAAACAACTGTTGAGATACA

TTACAATCAGGTCTAGCTGCAATGATTCAAGCTTCACAAGAATTATTTCT

AGCCATTCGACCGGATTCACGATGAGGGAGAATGCCAAGGGTTGAGAAGT

GAACAGTGAAACTGAGGTGGCCANCCATTCCATCTGCTTTCCCACCCGA

>TSBR.R52.esd 590 0 590 ESD GOOD: 104-341

GCACGAGGCTGTCTCTGTGCATTCTGGCAAAGCAACTTCGTAACAGGTCC

ATACGTGATCACCCGATATCCCGTCGGTCAGAGCGAATCCGTTCACTGAT

TTCAGTCAAAAAAGTAGGAAGTTTCATCCTACGTGGTCACTGGCCAAGGC

CATGGCAATGTGTGCGGCTGCTTTCCTATCCATTTCGCATCCGGGGGTAT

ACACGATTTCTGATGACAGTCACATTGAACTCCTGTTG

>TSBD.R52.esd 575 0 575 ESD GOOD: 101-315

GCACGAGGCTTGTGGTTCCACTGATTATTATGTTCGTGTTTTCTCTGCTT

TTATCAAAGAAACAGATTCCGAGGACAGGGATTCTGCTTGGGGCGCTAAA

AGCTCATTCCAGACTCAGCTTTTCGCAGCTTACAATGGTCACGGCTCATG

GATTCACAGTGTCTGCTTTTCGTCTGATGGTAATAAACTGGCTTGGACGG

GTCACAACTGCTCCT

>TSBU.R21.esd 736 0 736 ESD GOOD: 106-621

GCACGAGGGATGGGTGACAAATGGCTCCAGGGCGATTTTGGTTACTGTCC

TCGCGTTTACTGCGATAATCAACCCTGTCTGCCAATCGGTCTTTCGGACA

TTCCCGGTGAAGCAATGGTGAAAATCTACTGTCCTCGTTGTCAAGATGTC

TATACACCAAAGTCGACGAGACATCATCACACTGACGGTGCCTACTTTGG

AACCGGCTTCCCTCACATGCTTTTCGCTGTCCATCCTGAGTATCGACCTA

AGCGGGCTCCTAAACAGTTCGTCGCTAGGCTTTACGGCTTCAAAATTCAT

CCGCTGGCCTACCAACTTCAATACCAAGCGGCTGCGAACTTCAACGCTCC

GATGCGAAGTGGAGGGTTCGGGAAGCGTTAAAGAGGCTGAACCGCGTCTG

ACAAATGTACAAATGTGGCGCTTGTCCGTCCGTGGGTAATCCCCCACTTG

AATTACGCCACTCTTGTCTTCTCATGTGTCCTCGCGGTGTTTGCTTCCAT

CCTCATACTCGCCCTT

>TSAV.R86.esd 614 0 614 ESD GOOD: 106-613

GCACGAGGTAGGTGCGATAACAATGAATGACTACGGTCAAAGCCAGCCAA

TGTCAGAACTCGAGCTTCTTCGGATGCAGGCAAATAAGAAAACGGATGAA

TCTCTAGAGAGTACACGGAGAATGGTTACGATGGCTGAAGAGAGCCACCA

AATGGGTGCAGCTGCGATGGAACAACTCTACCACCAAGGAGAACAACTGG

ATCGCGTCAATGACCAGATGAACGTCATTCACGAGGACATCAAAAGCACC

GAGAAGAATTTGGACAATTTGGAGAAGTGCTGTGGCATCTTCATATTGCC

ATGGAAAAGAGTCAAGCGTCCTGGTAATGACAAGCACTTCAAGGCACGTG

AATACACGGCACCGACAACCGAGCAGCCCTCTAGGTTGAATTTTGGCAAA

AAAGCGCCTAACGGTGATCTTTCTCAANCCGATGGTCCCTTCATCCAGCG

CATCCTGGATGATGATCGGGAGACAGAGATGGAGCAGAATTTGCAACACG

TCTCCCNG

>TSBB.R44.esd 652 0 652 ESD GOOD: 101-598

GCACGAGGCGGAAATATCTAGGCTAAGCTTTTCTGGGAATGCCGACGAAT

CTTTTTTTCTCCTGATTTTGTTTTAGAATCAATCGATTGTCGGTGATCCT

CGGACAAAACTAATGGAACTCTGTGGTCAAACAGAAATTAGGTCGTTTTC

ATCTTTCTTTGACGCAGAGAGCTTGGTAAATATTTCCAAGATTGGTGAAG

GGGTCTACGGTGAAGTCTTTCAAGCCAACAAAACCTGTGTTATTAAAGTA

TTTCCCATTGATGGCAATATCCCTGTTAATGGAGAAAAGCAAATGGAGTC

ACATCGTGTATATCCGGAAGTTTTCATTTCAAAACAGCTCACAGAACTTG

GCTTCAAGTACCGCCAAAATCGAACCGTCAATTTCATTCAACTGCGGAGA

GCTGCCATCGTTTGTGGCAAGTGGCCAGCAGAGTTGACTGCAGCTTGGAA

AAAGTACGAGGCAGAGCACGGCTCTGACAATGAATGCCTCGATTTCCT

>TSAZ.R75.esd 533 0 533 ESD GOOD: 99-512

GCACGAGGCCGTCTTGAAGCTTCGTCGCAGCCACCAACTCACGCTCCTCC

CCCTCTTTCTGCCTACTTTCTCTCTCTACCGCCGTCTTGAACTGGACGCC

AGTGTGCGAGTGCGTCGGCGTTCGCGTGGCGAGTCGTGTTTCGTGTCCAC

TTTTTTCTCCCTCATTTCCCCCCCCCTCCTCCTGCCCCGTCTTTTTTTTA

GCTCAAAACCAATGTCTCTACCTTTTTCTCTCATCCCGTGTTGATCGCGT

AGCGTCCTACATCTACCTATCTATCTATCTGTATCCATCTACCGCTCTCC

TTGTTCACACCACTAGCAAAATTTGAGTACAATCCTTCCCCTCTCACACA

CACACGCACACTCGTTCGCTCATACACTTGCACACACACCACACCCACAC

CGTGAAAATCTCTC

>TSCA.R55.esd 649 0 649 ESD GOOD: 89-474

GCACGAGGCTCTGGATCTGACACCCGAACAATTAGCTTATCGGCAGATCA

TCGGGGAGCAACTCTTCGCTAAGATTCAAGTAACTCATCCTACGAATACC

GGAAAATTGACTGGAATGTTACTTAACTTGGGTCCATCATACTGGGAAAA

GATGATCAACTCGGAGGAATACCTACACCAGAGGATTAATGAGTGTATCA

ACCTCTTGGAAAAGAAGGAGAAGGATGAGCAAGCCGCTCGCTCTGCTCAG

TCGGAGGTTAAGAAAACTGATCGTTCTTAGGCCATTTGGACTACCAACTT

CGTTGGCAATTTCCCCCTCTTGGTTCTATAAATGATTGGTTTTGTTGTGT

TGATTGTTCTATAAATGGTTATAAAAACGCAAAAAA

>TSAF.R26.esd 412 0 412 ESD GOOD: 100-412

GCACGAGGCCGCTTGATATCGGTGGCAAAGGATGTCGACTCCGAGTTACA

GTTTGCTCTTTCGGATGCATCAACATTTGCGGATGAGCTCTATGAGATGG

GTTTCACCGCAAAGGATGAGGGTACTATGGTGATCATCATGGGAGAAAAT

GGCAAGAACTACTTGATGAGTGACAAGTTTGGTGTGTCGGAACTGAAGAA

GTTTGTGAGCGACTTTGCCGCTGGGTCCCTTGAGCCATACGTAAAATCGG

AACCAATACCAGAGAAGCAAGAAGGCAGTGTCATGAAGGTCGTTGGAAGG

ACATTCGAGNNNN

>TSCG.R33.esd 564 0 564 ESD GOOD: 97-564

GCACGAGGACCTGTACCTGTTTTTCTCTCTCCCGCAGAAAAATGTACACC

AAATTTGCGCACATCTTTAGCACCCTTCTTACGGCTCTTCTTCTTTTCCT

AGCCGTAGGCTATCATGGCTGGATTTGCATTGGCAGCGTATTCAGTGAAG

ACTGTAAACACTTGGGGTATATGCCAACTGTCGGTGGACTTCTGATTACC

GCCGGTATCCTCATTTGTCTAGCCGCTCTCATTATTGTCTTAGCCATCTT

TCGCTGTGCGGATTGGCTTAATCTTGCCGCCGTTTCAGTGGTCATCCTAT

CCACCATCTTTTCTGCAGCTGGAGTCTTCTTCTACTACAACAATACCCAC

ACCTGGTCTCCGTTCATGGCGACAATCGCGATGACCATCTCGTTTGTTCT

CACCGCCCTCCTTCTCATTGATGTCATTTCTAAACGCGCCAATTCCAGCT

GAAGTTCGTCTTGATNNN

>TSAJ.R93.esd 685 0 685 ESD GOOD: 95-396

GCACGAGGCGCTTACAAGGAAGAGATCAAAGATTGGCGACGACGGGGAGA

CTTCATCAAAGAACAAAGGCGCTGGCAACACATCTGACGATGAATTATAT

TTTAGATCGTATTCCTTCACTGATGTTCATGCCCTCATGCTGTCGGACAA

GGTTCGCACACTGACGTATCAGTAGGTGATACCTTTGCTGCAAATCAACC

CCGAAATTCCACTATTGCTATAAACGGATCCCCGAATTCCACTTTTTAAA

TGACTTTTTAAGTACAAAAGGCAGAACCCTAAAAAAAAAAAAAAAAAAAA

AA

>TSBI.R83.esd 711 0 711 ESD GOOD: 104-461

GCACGAGGGTCAGCATACGTGCTCTACAAGGTGGCGATGCCTTTTCGGTA

CGCTCTTACCCTCTGGCTCACTCCCGTCATCGTGCACCGCCTGCGCTCAG

GCGGTTGGCTACCACCGCTGGCCGAGCAGGATCGACTGCGCAACTTGGCC

CTTGAAGGTGCCAAGAAAACACGGGAACGGCTGAGACGGAGACGGAAGAA

GCACTCACAAACGGTGCGGAAGTAGTAGGTCGGTCTTCCCGCATATATTT

GTACGTATTTTGCCACTTTTATACTGTTCGTCGCAAGGATCAGAAGTCAC

GCCCTAATTCTACCCCCTTATTTTCCCACTTGCTTCGTTAAGCAAACCCT

TTTTGATT

>TSBV.R2.esd 570 0 570 ESD GOOD: 105-570

GCACGAGGGATCAGCAAGGCTCTGACCCCAGGTGGAGGCTCAGCATTGCA

CTCCGCTGTGTTGAAGCCTGCGACGGACTCTAATCGGGTTCGCTCGGGTG

CACAGTTTTTGCCAGTGGGGAGTCCTCGGGCGTCCCTTTACCTACCAGCT

ACCTTTCACCTTCTAGTCTAGTCGCGTCACTCACTGCTTTGCTTCCCTGC

ATCGTGCTTTTTCTTTCTTCCTCCGGTGACAGTGTCCGTGACGTTCCGCT

GCAAAAGCGCGCCAAATGCCAATGACAGCACCTCGGCCCTCTACAACCGC

ATCAATGCTTGCTGCGCATTGTCAGGCAAACATTGTAAATGTATGCAAGT

TCTTAATCACTAACAATCAGTCTGCAGATAGCTCACTGACGGTAACTCCG

TGATGCTGTAGTGCTTTTTGCACAAGGGTCATATTCCAGTATTGGCTGTG

GCTGGTTTCCGTANNN

>TSAL.R85.esd 701 0 701 ESD GOOD: 107-558

GCACGAGGAACAAATCTAGGAGAAGTCGCAGTGAAGATGTTTCACCCAGA

CGAAAGTATGGGTGCTGAAGCTTTGCTTCAAGCAATCGCTGTGCTTGAAT

CGCAGCCTTCAACTCAGGAGCAACCTGATGCAAAGCATACAGTGCCGCCT

GACGCATAGCTAAGGTTTCTTGCGCTAACTGTTTCACTGCCCTATTATAT

GAGGCTGCGCAGCACCCGTCATAGAACCATGCATAACTCTTAACTGCGAA

TCCAGTTTTCATTACTGTGAAGTGAGGGGGGTTATAAATCGCCTAACTTA

AAGCCTTGCCTCCCAGTCGTCAAAGAATTACAGTCTTTAACCACTGCATG

GATATTTCTTCACGCGACATCCATGCTAGAACCCAGCGCCCCTTTGAAGG

CAGACATGTTGGGAAGCCTGGACTGCAAGTGATGCTCATCCGACCCTATT

TT

>TSBU.R68.esd 768 0 768 ESD GOOD: 111-406

GCACGAGGGTTCGAGTGACCGCCTCCAACATCTTAAAGGCGTGGCCGTCA

GAAGAGGTGCTGAAGACGAATTCTCGATGATAGCGCGGTGACAATATCAT

TCTCTCCCTCTCTCTTTCTCTTTTTCCCCATCTCCGTCTGTGATAGTGAC

ACCCTTGCACACGAGCTTCTCTTTCACTTGAACACACTCAGGATTTGCAA

ATTTTACATGCTTGTCTGTAAACGCGTGTATGTGTGCGTGTGGTTTCTTG

ATTATCTTGACAATAAAGCTGACATTCAAAAAAAAAAAAAAAAAAA

>TSAA.R89.esd 750 0 750 ESD GOOD: 106-212

GCACGAGGCATTAGTCGGTTTCAACCACTCTCGTTCACCTCAAAGTGGCC

GGTGTTAGTCGTTCTATGCTTCCAAAAAGGCTAATCAGGGAGATCAGATG

CATACAG

>TSCC.R68.esd 765 0 765 ESD GOOD: 107-409

GCACGAGGGTTTGTACTATCATGACCTTCTCGCGTTTCGCAGTTTTAATT

CTTGTCCGTGAGGCATTCAGATGTATTTATGCGCCTACCTCCACAGTTCC

TTGATATCGTCCTTGTCATTTCATTCTTTAATGTGTTTTTCAGATTCTTT

GCTTATTAACTACGTCGTTAGAATGTTCTCCAAGTAAAAAAAAAAAAAAA

AAAAAAAAAAAAAAAAAAAAAAAAAAAAAAAAAAAAAAAAAAAAAAAAAA

AAAAAAAAAAAAAAAAAAAAAAAAAAAAAAAAAAAAAAAAAAAAAAAAAA

AAA

>TSBB.R18.esd 523 0 523 ESD GOOD: 76-267

CGGGCTGCAGGAATCGGCACGAGCTCTCTCACCTCCCCCCATCTTCACCT

TCCACTTTGGAAAAGACCAAACCGTCCCAAAAATCGGGGCGGCGCAGCAA

GACGTTTGCATACTTTTCATCGCGCAATCGGTCCAAGGAGCGTCACACAA

AACCGGAGTTTAGTGGTCGAGGAACCCCGAACACCACCCACA

>TSAR.R20.esd 648 0 648 ESD GOOD: 103-472

GCACGAGGGAAATGTCTACTTACGTGGGAACGGAAGAAGGCGTTGTCACA

ACCAGCAGATCTGCCTTCAACTGGGCAATTTCTGCCATCATATTCACCTG

TGTGGCGATAGTCCTTGCATGAGCGTTCGCGTAAGTGCGTCCGAACCAGC

AACATAATTGGAATTATTTGTAAATTGTGGCTTGCAAGCTCTGCCCGATG

CCCCAGTACCATTCACTCGTGTTTGGCTACCACTAATGCATCCTGCACTG

ATTGGGAGTTTACTGACAAATGCAGCACTTTGTTATCAAAAAAAAAAAAA

AAAAAAAAAAAAAAAAAAAAAAAAAAAAAAAAAAAAAAAAAAAAAAAAAA

AAAAAAAAAAAAAAAAAAAA

>TSAV.R61.esd 509 0 509 ESD GOOD: 114-509

GCACGAGGGCGCCTTGGAGGACCAATTAGCCGAAAGTCGTCTGGCTCTTC

AGACTGCTGAGAATAGAACATCAGACCTCACCAGTCAATTGGAAGGCGCT

CGCCAGGCGACTGAACGGGAAATACGTGACCTCTTGCTCAAGGCTGCGTG

TTTGAAGGCCAAACAGGATGCCAGCGCTATTGAATCACTGATGGAAGACC

CGGAATTTATCCAGTGTCGGAGCTGTTCAGATGTCGTCAACAATTTCGCA

AAATCGGCCACCACTAAAGTGATTCATCTGAAAAGCATTGTTTCTGATAC

AGATGTCAGAAATTTGACAACGCTACCTTTAGACGTGGCTCATCTCTCCT

CTTACCTCAATGCTAACCTCCTCCATGCCAAATCCATCGCTAATNG

>TSBV.R42.esd 583 0 583 ESD GOOD: 102-370

GCACGAGGATTTGGAGCAACTCAAGGATTTGAAGAAACATCTCGTTGATG

AAGTCAAATTTCATGAGGAGCAAATTGAGCGTCACAAGGCCGCAATTGAA

CGTCATCTCAAATCGCTGCAAAACATTGACTAACTCCCCTTCCTTCCCCT

CATAGTCCTCCTTACTTCCTACTTCATAGTCACGGTCCTCCTCTTTGATG

TTTATATTTCACTAGTTGTCAATTTTGGAGAAGTAATAACAGTTTACGAT

TAAAAAAAAAAAAAAAAAA

>TSAH.R45.esd 768 0 768 ESD GOOD: 105-617

GCACGAGGTCGAGACGCGCGTTCAGCAAGCCATAGCAAACTTGCACCGCA

TGAAAATGCGATTCTTCCTCGTTCCGTGAGTGTCGGCTCCTCCACCCATT

GCTCTCCAGTTTATGTGAGTGTTTGGGCCAACAAGACGTACAATCAACAG

CACCTTCGGAAATCAGTGATACCTTTCAGTATATCAAAACGTTCCCTAGT

GTATACTAAAGCAGTCACATTTATGGTCTTGTACGTGAGGCTGAGTTAGA

GCCAGAAGCACTACAGACGCTTTCGTATTCCTCAAACAAGCCAAACGGAC

TTGTTTTGAAATTGTCCCCATGCTGGAGACATCACTCCTTTTCTCATGGA

GAGAAATTCAAGCTAACTGCTAGCTTTTCACACTCAATTACTCCTTTTGG

AGGTCGGCGTCCTTATTCTTCATCGTATCAAGGACCTCCGGGATTGAATT

TTGGTCAACCATTAGTCAATACAATGTGTTCTTCAGAATGTGGCGGCGTT

CCCCCACTTACGA

>TSAT.R84.esd 791 0 791 ESD GOOD: 105-640

GCACGAGGGCCATGCAGACAAAATTTGCGATCAAGTCAGTGATGCCATTC

TGGACGAGTACTTGAAGCTTGACCCCAACTCAAAAGTTGCATGTGAAACT

TTCACAAAAACTGGTATGATTCTCATCGGTGGTGAGATCTCCTCTAGAGC

CACTATCGATTTTCAGTCTGTCATACGACGTGTGATTGAGCGAATCGGTT

ATGATGATTCCCGCAAAGGTTTCGATTTCAAGACCTGCAACGTGCTTACG

GCCATTGAGCAACAGAGTCCTGACATCGCTCAATGTGTTCATGAGGGTAA

ATCGATGGAAGAACTGGGAGCTGGCGACCAGGGCCTTATGTTCGGCTATG

CAACTGATGAGACTGAAGAATGTATGCCTTTGACCATCGTGCTTGCTCAT

GCTCTCAACGAACGTCTCGCGATATGTCGGCGTGATGGAACTCTTCCCTG

GGCTCGCCCCGATTCGAAGACCCAGGTCACCGTGGAATACAAAAAGGATA

ATGGCGCTTGTGTCCCACTTCGCGTTCACACCGTTG

>TSBT.R50.esd 553 0 553 ESD GOOD: 97-553

GCACGAGGAGAACTTGGAGTGAAAGTGATGACTGTGGAGGAGGTGAATGA

AGTCAACTCCAACTCCTCTCAAATGCAAAATTGCCTGTGTGCTCTGAATG

GTGGCACTCCCGTGCATTTGCCTCCTTCAATTACACAAAATGAACGCCTT

TCATATGTCGCGTGGTGCCTTTGCCGAGTTGCCGGAGTGGAAAAAGCTGA

ATTTGAAGAGGAGCGTTAACCTATTTTAATGGTCTGCTCACCGTTCACCT

CTTTACCTCTGTGATACCACTTTTTCTTTAGTATTATAGTACCCCCAATC

GCAGTTAAAACGGACACTATTCTGCTGGTATTATTTGAGTTGTGCATCCT

CCACTGTGAGGACGATGCTTATTTATTTTTTTTATAGACGACACCACCTT

AATATTCGCATGCTTTCCATTTACCTTACTAGTTCCCTCGGACACCATAT

CAGCNNG

>TSBR.R27.esd 562 0 562 ESD GOOD: 100-489

GCACGAGGCAAAGCTGGAGTTGAACAGGGTCCCCAGGTAAATCAAACACA

GTTTGACACAGTCATGAAATACATTGCCTCGGGTCGTGAGGAGGGCGCTC

GTCTAGTGGCTGGTGGTAAGCGTGTGGGCGACAAGGGGTTCTACATTGCA

CCCACTGTATTCGGTGACGTCAGCGATGATATGCGGATCGCACGTGAGGA

GATCTTTGGACCGGTACAGGTAGTGCTACGATTCCACGACATTGAGGAGG

TGCTTCAACGTGCTAATGCCTCACATTATGGCCTCGGCGGCGGCGTCTTC

ACCAGTGACATGGACAAGGCCTTTCGCGTTGCTCANGGCCTTGAAGCTGG

CACTNGCTGGATCAACAGCTACAATACTTCGCCTGTAATG

>TSBG.R21.esd 847 0 847 ESD GOOD: 105-633

GCACGAGGGTTAAAGTGGGGCATCAATACGTATCACCTCTCATTAGGTGG

ACATACAGAGCTGCTACACAGTCCCACTAGCGGCGGAGGGACCACTAAGC

GCACCAAATCGGTCGACCTCTCCGCCATCGCTTTTCATGAAACCGCTCTG

TCCGAGGTCGCTCCGCTCAAGGCAGTCGCCGCTGGCGCCGCCACCACTGC

CACCACGCCGCAGGAGCATATCTGGCGCCACTTCACCCAAAAACGGCTAC

ATCCGTGGTTCCGGGGAATCGGCAGTACCTTACCCTTCGTCTCGGACCCT

GGATTAGTTCCCACCCCTTCCCTTTCTCCTGTGTACAACATTTAACTCGT

CTTTCTTATCAAACCCTTCACTCATGTTTCAAAAAGGTTCCCCACTTTCC

TCTGGGTATNATTTGCACTCTTCTACGTGTCTTGAAAAAAATAAGCAAAG

TTTAACCCCCAAGTGGAACCCATATGTACAGCTACCTCTAGCTTGATATT

TGTCTGTGTATCCCCTTTATCTCTAATAC

>TSBK.R16.esd 543 0 543 ESD GOOD: 97-461

GCACGAGGATTCAATGTGTTGCATTTGCATTTTCAAATTTCTCAATCACT

TTTCCTATAGCGCTGTACAAATTATTTCTTATTCCTCGCTAATTCTCTGG

TTCTTTAAAACTAGAATTTGTGGTACTTGATGCTACTACCTGTTAGCGTC

ATGTCCACCTTCCGATGTGACTGAACAGCTTTGTGGGTCTTCAGGTCCAT

AGTCTGCGACATCACCTTAACTATTTAGTTTCTTCAATTCTACCGATTCT

CTCTTCNAGTGTCTTCGGAAAACCATTTCAATTAATCGTACCACCCTGGG

GAGCTCAAATCGGACAGAAATTTGTTACNTTGTGGATCCAGTTTCCCAAT

CTTGACCAGCAAAAA

>TSAK.R16.esd 581 0 581 ESD GOOD: 99-446

GCACGAGGGCAACAACCCCCCCAAATGCTCTCACAACTACTTGTTCAATC

ACATTCACAACTGCAGCAGCACCAGCAGTTACTACAGAGGCCACCGCAGG

ACGGCACATCCCAGTCACAGCCTCCCCCACCCCCACAACAACACCAAATA

TCTCGTTGATGTGTTGCCCCTCCTTCCTTTCAAGCCTCCATACTGTCTTT

CGGCATCCTGTATATTTGAAATTTAACCTGCACCGACCCACCTAGATGCT

GAGTGCGCTCGACCAATCTTTTACTGTAGCGTGCACCTGTTCAAGCATTT

GGACCGGCAATAATATAGCCCTGTTTCTCTAAAAAAAAAAAAAAAAAA

>TSBM.R58.esd 606 0 606 ESD GOOD: 111-375

GCACGAGGAAGGAGTGGAGAGGGAGGCGGAGAGACGTATTTCTCAGTTCA

CAGCTCTCTAGAGCGTCTGTGCCCAAGTCAGCCTGACCACTTGGATGTTA

GCGTGGAGGGTGATCGACCTGGTGCAGGCAGCGGTCGAATTTGGGAAGAC

GGCGTCGGTAAATTTTAACTGCATTTTCTCACCATCTCCCTACTTTTTTA

AGTCTTTCGTCTCCTGCCTACTTCGCACACTCTGATGCGTAATAAATGTG

AAATACACTTAAAAA

>TSBT.R35.esd 688 0 688 ESD GOOD: 103-589

GCACGAGGCCTCTCTTGGCTGGAGCGACAATTCGTCTCGCGCCTCTTCGC

ATCTCCACCCACGGCAACCCTCGAGGAAGCCACGGCTGAGTTCCTGGAGT

TCTTCACTACACCATTCGACACCGGGCTCAGAACGTAAGGTCCGACGATG

CAAAGGTGTACAAATCGGCTTCATTTTTAATAGGCGGAACGTCTGGAGCC

TAGTCGCTGGGTAATAAACGCCATCTTCCTGGCCAAATGCGAGTATGCAG

GATCGCACTACACTGAGGCGATGAAATGGCTGGAGACAGCCGAAAGCCTT

CTTCGTTGCGAAGATAACAGCAACGAGAGTGACCAGCACGCCGGAGGTGA

GGATGGCGAAGAGGGGAGAACGCTGAGGACGTCGAGCATCAGAGATGCGG

TAGTGGAAAGACCGGCGTTACTGGCGGAGATTGAGGCGTTGAAACCGCGT

TATCGCGCCTACCTGTCCAATTAGTGATTATCCACCA

>TSBV.R53.esd 456 0 456 ESD GOOD: 108-303

GCACGAGGGAACAGTCTAGTTGACAAGCCAAAGAAAAGAACAGCACTTCT

GGAAGCTAAGATGAAGCTTCAAGAAAGGTATCGCACCAACGAAAACCCCT

GGCTCTTCCGTAAACTGCGATTCTAGTTTACCTACGAACGTGTAAAAATC

TAATAAATTCTTTGGCTATCAAAAAAAAAAAAAAAAAAAAAAAAAA

>TSBY.R90.esd 860 0 860 ESD GOOD: 101-532

GCACGAGGGAGAGAACTAGTCTCGAGTTTTTTTTTTTTTTTTTTTTTTTT

TTTTTTTTTTTTTTAAAACCTCAGTTGTACATTTGAAAGGTGTGCTATTT

GACAGCTGACAACTTAATCAATATGGAAGAATAAAGAGGACTTAAGGAAA

TTAAAAGCCAAATTAACGATCTTCCAGTTCTTCTGCATTCAACTCTTCCC

CACTTCCAACATCGTCAAAAATCTCCTCTTCTTTCGACGATCGCACAGCT

TGGCCCCGTTGGAGAATCAGTGCTTTTAAGTCAGCTGAAATGTGGAACGC

CTTAATGGAGGTTTCTGTTAGCGCCTCGGTTGCAATTACTCCACCAGCCT

CTTCAGGATTACCGACATCGAGAAAATTGAGTTGCCATCCGACTGTGCTT

GTGTGGGCAGCACTTTCTGCGTCCTGACAAGA

>TSAU.R92.esd 385 0 385 ESD GOOD: 90-246

GCACGAGGATGGCTAGATAGCTAAAAGCCATGACCCATATCGCCACCAAC

GCACAGCATATATGACCGCCCCTACCAGATTTTTCGCTATTTATTATGGC

CAGTGCCAGCTTCACCGATTTTGCTATTACTGTTTCTTTCCTGTAAGACT

GGTTATT

>TSCD.R46.esd 540 0 540 ESD GOOD: 89-540

GCACGAGGCCGGGGTTCTGCCAAAGCGATGCTTTGGCGATCAACAACGCG

ACAAATAACTCCCTATTCCACCTGCTCTCAAATCCTGATCTTCGAGTGGC

AACATCACTGGTTAAAATGATCATAGGATCTGCCTGCAGTCAGATCACAG

GGAGTGATCCAGAGCACTGTCTTAACCGGCTGAACTATCTCCTGGCCTTC

TTTGAGGGCGAGCCGGTGCGTCCACCAGGCGCGGCGGCTGACGTTTTCCG

CCTGCCTGAGAGTATGGGCACGGTATTTGCGTGGCATTGCCTCCTGAATG

CTGCTTTGGCCCAGGCCGAACGCCAGTTCGCATTCTCACTGCAAAGTGTT

CTTCCTTTTGCCGCCGTCTTGGCCACACTCCTTGCGCGGTATTCTGAGTA

TACGCCCAACTTCCTCGCACGAATGGCGTCAGCGTGTCCCGTACTCACAN

NN

>TSAK.R66.esd 559 0 559 ESD GOOD: 102-535

GCACGAGGGTGGTTTGTCAGCTTCTGTTCAGCCATCTGATGAAGATAAAC

AGCGCCTCACGCCAGTTGTAAAGGATTACATAGCTCAACAGACCGGTCAG

GAACCGTCGGAAGTCAAAATCACTGAAGTCAGTCGTCAGCTTGTTAACGG

AATCAACCACTTCCTCAAGGTTGAACATGGTGATAATGTATGGCACATTC

GAGTTCACGAGGCTTTACCTTGCTACGGCAGCAAAATTGAGGTACATAGC

CACAAGGTTGCTGCCGCTGGAGACCCTCTGACATACTTTTAATCTCTATC

ACCCTTCTTCCGCTATATTTTCTTTATTGTATCGACCAATGATCAAATTC

CTCGATTTCTATCATCCTAATGCTCAAATTCCGAAATCGAAATCTGCTTT

CTCACTGGCCTTTACAGGATACCATTTATCCCAT

>TSAQ.R40.esd 635 0 635 ESD GOOD: 93-466

GCACGAGGGTCACATCAGAAAATACAAAGTGTAAACTTCCATCTTCCCTT

ACTAAGCAAAGTTCAGAACCAACGCCTCTGCCCTGCTGCACTCGACAGCA

GCAAACCTTCCTCTATATCCCTTCTGGAAATCGGGAAGCACCTGTGCCAA

TACGTCCTTCGGTCATGCGAACACCACTTGGCGCCCCAAGGAAGCAACAT

TCAGAGTGCGGTTAATCAANACGTAGACGGAGTATTTTGTTAAGTTCCAT

TAAATAGTTTACTCTTTTTCTCTAGATTCTCAAAATACTCCCGNGTCCGC

TGAAACATCAAATCTTCAAGTTCCTGTGGTTCCAGTACCAGCAACTGCCC

TTGCCTCAACACTGCTTCATAAAA

>TSBS.R32.esd 558 0 558 ESD GOOD: 99-553

CGCCTGAGTCAACTTCAGCCCCCCTTTAACATGCCTTCCGAACTGGTAAA

ATCTAACGGGAGGTAGGGGTTACTCATGTGCTGGTGGAATCGCTCTGCTC

TGTCTCTGCATACTGTCCGTTGTTCCCTTATTTAGCAGTGATCAGGAGGC

TACAAACGATGCTGTTTTTGAAGTGGAGCAGCGCTATCGCGGGTATTTGG

CCAAGGCTGTGGAGGTCATTCGACAGCTGGATCGTAGAGCAGTTGCCGCG

GAAACGGCGGCGGTCAATCAAAACTGCGAGCAGAAATCCGATTCTGAAGT

CTCTCGTCTGCAGGCGCTGTTGGCAGAGAAGGAGAATATCATAGAACAGT

TGGAGCGTCACCACGAGCAGGCACGGAGGCAGCGAGACCTTGAGGATCGT

ATGGTGCTAACGGCGTTGTACCACTTCGGGATGCGGGTGAATCGTGCTCA

CACGG

>TSBC.R52.esd 854 0 854 ESD GOOD: 80-181

GCACGAGGGAGGCTTGCGGTCTACACTGTGAATATGCGGCTCACAGGAGG

ATTAGTCGCCTATGCGATTACGCGGTGATGAGCTGCCTTAGGTGAATGTT

TC

>TSBC.R28.esd 637 0 637 ESD GOOD: 99-372

GCACGAGGCCACCGCTGCCACTGCCAGCAGCAAGGGTCGGGTAATGCTGC

TACTCCCTTGTCTTTGGGTGGTGAGCGTGTTAGTGGTGATAGTGTGCGCA

AGCAAAATGTGATGGCCGCTATTTCTATCGCCAACATTGTAAAGACCAGT

TTAGGTCCCGTGGGTTTAGACAAAATGCTCGTGGACGACGGTGGGTGATG

TAACTATTACAAATGACGGAGCTACGATATTGAAGTTACTTGTGACGTAA

NNCAACCNGGCAGCCAAGGGTCTT

>TSAA.R59.esd 696 0 696 ESD GOOD: 98-479

GCACGAGGTAGGAGAGCTTCAATATATGAAATCACCTACTAAATAGTGTC

CCTCTCCTGTCCCCTCCCTCTTACATCCCACTTGTACAGACGTCTCCGTG

GTCACTTCGATTTCATTTCCTATCTGTCATCATTACCCCCCTTGTATACA

CACACACACACACACACAGGCACGCATAGTTTGCTTTAATGAGTCCCCAT

CATTACTGAACATTTTAAACCGTATACACAAAAATATACATATGTATTCT

TATTAGTAAACTGTGTGCGTTATTAGCAGTGAAGTTCACAAACACACACT

CTCTCTCACACACATGTTGCATCAAATATTTCCAGTCGTTTTTTGAATAA

ATTTTAAGAATCTAAAAAAAAAAAAAAAAAAA

>TSBW.R2.esd 239 0 239 ESD GOOD: 80-239

GCACGAGGATACGACTCACGGTCAAGCCCCGCAATAGCTTCTCAGGTCTC

CATCTAAGTATGGCCAGTGACTTGGAGCAGCAACTGCGATCTCTCATCCA

ACGTCTAGACACCATGTATGAGCTGCAATACACGTATCAGCGAACTCTTA

GCAAGCCNNC

>TSAT.R89.esd 598 0 598 ESD GOOD: 100-372

GCACGAGGGATACCGGCTCGTTGTGGCAATCACCCTTTCCACCTTCCTTG

CTCTCCTTTTTTTCACTCTTGCGGCGGTCTACTGCGTGGTCTGGAGACAG

CGCTGCTGGCAGCGTAACCAGCGAGGAGGTACCTTGGGACTGCTAGCCAG

TTGCCCTATTCACCGCTTGTCTCGACACCTCCCACTGCCTAATGCTCATC

TCGTCTCGTGTGTGTCTTCATTTTTAACCCCGATAAAGGTGGTGTTTGCG

AGTTCTCTTTTCTCATGCCACTT

>TSAU.R46.esd 668 0 668 ESD GOOD: 87-368

GCACGAGGCCCGATGGGTATTGTGGCCTACGTGGCACTGGAGTCACGTGC

CCCCTTGATGAGCGACCTTAGAGCCCTCCCTCAGACGTGACTTAGCACCT

CATAATCAAACAAAGTGCCTCTTTTTCATGTTTACCAAATGGATTTTCAG

AATGCTGACTTTATTACTTTCGTAAACAACTTCTCCATGATCTGAATGTG

GGCAGAATACAACTTGAAACCCAAAAAAAAAAAAAAAAAAAAAAAAAAAA

AAAAAAAAAAAAAAAAAAAAAAAAAAAAAAAA

>TSAJ.R1.esd 627 0 627 ESD GOOD: 104-627

GCACGAGGATCCTTGTTGGAATCAGACATTTATGTTCGAAATTGAGACAT

CGCAGACGATTTGCTTTACCGTGTTTCGACAATCAATTGTTTTTGGACAA

GTAGAGTTGCCGCTTGAAGATGATCAACTTGATCAAGAAAAAGTATCCCA

CCGTTTAAGGACTGACGAGACCGTCTCAAAACCAGTGTATTTGCAAATAT

CAATTTTGTACAATGAGCGAAAGAAACTCAATGTGAAGTCGAGATCTAGG

AAACGATCGGGTGTGTTTGGACACCCTCTTGAGGAAGTTTTGAAACGGGA

CCAGGCCGACCAGGAGAGATTAAAGAAACAAATGAGTAGCACTAAAAACT

GCGAGGAGTTGCGAGTTCCTGTGCTGGTTACCGCCTGCGTGGAGGAGATA

GAACGACGAGGATCTCAGGAAGAGGGTATTTATCGAATTAGTGGTGCAGT

CACTACCGTCTCCAGCCTTCAGGGTCTTTTTGATCGTGATACCAACTTGG

CAGTTAAACAAATTGGTGACNNNT

>TSAA.R52.esd 634 0 634 ESD GOOD: 100-515

GCACGAGGCGGAGACAAACATGCCGAGAAGTGTCACCAAAATGGGTACAT

CTCCACAAGGTACAAGTCACCGGCTCCCCACTTTCCATGACGGTGCTGGG

TGTTGATAAGGCGGTATCATCTGGCCACAAAGGTTGGCAATCGTACTGCC

ATTGAGCTGTCAGCACAGAGCGAATGCATTGCCAGCTGCTGGGCGACGCA

ATCTTGCATCTATCCTTGGTTTCCAAGGAAGAAACATTGATTAATTAGGG

AGGGACCGGCTTTCTAAGTAGAGACAATACGTTTGCAAAAGCATAAAATA

TTTAATGGCTGGAAATGANAGTGGATGTTTGTTTAAGGGTTAGCCGAAAC

AATCTTTCTCGGAGCGCAGTGATTATCTGTATGCNCTACCGATTGTNGCT

TGACCTGCATTTGGGC

>TSCG.R25.esd 569 0 569 ESD GOOD: 101-569

GCACGAGGATCAGTAGTTCGCAAAGACCAAAGCTAGTCGCGTCCGTTTTG

AGTTTCGTCCTGCTCATGAGTCTTGGGTAATTTTCGACGAGCTTGGATGC

TAGCTTGAGAGGCTTTTCTTGCACCAAATCTGGCTAATCTTAGTGTACGC

TTCGCAGCCATTGGTGTGAATAATGTTTTAACCTTCATTTATTGGGGAGG

TGCATGTTTCTAGTGGAGTGTCCTATGCTAAGAATGGAGGCATCGCCTAG

CAGCATTGGATGTGTATCTAGCCCTGCATATTGCTTTACGAATTTCGCGG

GTTAGAATGAATTTTGGCTTGCCTAACTTTGTCACTTATTAGTTGTTGGT

AGGTCCTTTCATACCTAATGCCTGGTGAAATGGCGGTTATTTTCATTACT

ACGATTTTCATGTTGGTAGAACAGATATCGGGGCATGGGGCACTTTGCAA

TTTAGTTGCTTGTTAANNN

>TSBV.R6.esd 436 0 436 ESD GOOD: 108-416

GCACGAGGCCTAACCCTGTCTTTCCATTTTCGTTATAGTACCTTCTAATT

TGCCTTCAGAGGCTTTACGACAAGGCATCCATCTAAGAATTTCCTTATCT

TTTGGACGGTGACGTAGATGGTATTATCCAAGTCGATTGTGTGAAATATC

TGTGGTGCGTGAACTTGAATTACAACACATACGTTGGTTGTGTTTGTTGA

TGGAAAACACCCCACCCGTCTTTTTTCCAATTGGCGACCGAGCTTACGAC

TAGGTTCTCAATAGGTGGTCATCCTTACTCCCTTCACTGTCCCAGCCTTC

CACCCCAAT

>TSBR.R3.esd 657 0 657 ESD GOOD: 102-544

GCACGAGGTGATTGTCGAAGACATTGTTGACTCCGGAAAAACCATGAACA

GACTCCACAAGTACTTGGATACCCTTCAAGCCAAATCAGTCACTGATGTC

TGTCTCCTAGTTAAAAGAACTCCTCGTAGTTCTGGATACCGGCCTTGCTT

TGCTGGTTTTGAAATTCCAGACGACTTTGTTGTTGGCTACGCCCTAGACT

ACAACGAATATTTTCGAGACCTCCATCACATCTGCGTTCTCAACAAAGCT

GGGTTGGAATATTTTGCAGTCCCCGAAGGGCCTGCAAGTCACGCAGTAGA

GGAATCCCAAGCCTTCTAAATGACTGCCACAGCTCTCTTATCCCGATTTT

ACTGGAAGTGGCATTCCTTTGACGTCTCATTAACTTGTGTCCTAGGTATG

GCGAGACTTGGTTTTTAATAAATCGATGTGCTAATCAAAAAAA

>TSAL.R68.esd 499 0 499 ESD GOOD: 105-499

GCACGAGGGTGATCGAGACGACGGCGCGGATTTGGCCATATCAAATCCTC

CGGCAACTAAGGTCGGTAACATGCGTGTTGCCCAAAAACATAGGGATACT

GGAGAGAAACAGCTGTCAAGGTCTGAGTATATGAAACAGGCACAAGAGTA

TAACTCAGAAATAATAGTTCAACCGAAGCACGACTTTGAAACTCGAAAGG

AATTGGATTTTCATGATCAGAGCGTCGTTCACAGCCAACAAAAACCACTT

CCACGTGTTTCCAAGGCGCCGAAACCTGTCCAGCCGATCATTTATCAACC

TCGCAGTGATTAAATGCCTTAGCAACTGTTTGCCTCGTAAATTTTACTAA

TTCTCTGAACATATAGTGTCAGAAACGCTTTTATGTACGTNNNAN

>TSBD.R55.esd 579 0 579 ESD GOOD: 96-559

GCACGAGGATGAAGTTGACCATATCAGAGGTGAAAACTTTGCGTCCAGTA

AAATATTTTAAGGAAAACTGCGATAAGATTAATTCTTTGAGCTATACAGA

CAACGGAGAAGCTATCATATCTAGTAGCGACGATGATCAAATGATAATGT

ACGACTGCAACTCTGGAACGTGAGTGCTCTCATCGCCTCTAACTCCTTAC

TATAATGTTTTACCTTCTTGGTAATTTTTTTTGAATTTAAAGCGTTGTGA

GTTCGTGGGCAATAAATTTAATAGCCTTTGCGCGTGGGCGCTTATAAATG

AATCTTGGACCGTCCACTTGGTGCACCACGTCCTGTATCACATGGGCTCT

TTCACGTTGATTTTGGCATGCACATTTTATCAGTTTGTTACTGTTCACTG

TTTTTTTAGCCCTCAACGGACAATCAATAGCAAGAAATATGGTGTTAATC

TTATCCCCTTTACT

>TSCB.R23.esd 612 0 612 ESD GOOD: 102-612

GCACGAGGGTCCTGTGTTGGGAGCTCTGATCGCAGCCCTCATCTCCCCTT

GTACAAAAATTAACAATTTGAGATGTGCGTTGCATAGTGTATCTCCACAC

ACGCTCGTATCGCAGGAATGATGTCTGCTCTGCGACTAATACTACCCTAC

CCATCTCTCCCCAGCCCTATCTCATTGCGCCTTAACTTAGCTAACATTCA

ATCCCCGAACCTCTTGTCCTGCCCACCCTGCTCCATGTCATCGCGCCTAC

GAATAACCTCACTTACCCCTCCTCTTTCCCAATGAAACACATGTCTTTCT

ATCACCCCTTCTCCGTCCTCTCCCTCTCTTCCACTCACTCTTTTCCCTTC

CCCCTTCCACTCCTCCTCCACCTCCTCTCATACACACACACAAACACACA

CACACACCACCGCGTACTCACTTACATCCGCCTTATGGCCTCGGTTCTCT

CTCCCTTCTTCAACAATCCCTTCCATAATCCATTAAACCCTCTCGTCCTT

TTGTCTGGNNN

>TSBC.R29.esd 561 0 561 ESD GOOD: 92-537

GCACGAGGGGCCAGTGCAGAGCATACGGAGGATCCATATCCGCGTTATCC

CAGCGTCATATGCCCATCCTGTCTGCAAGGGAATCTACTGGGTTTCCGAA

AACGCCGCAGTCTCTTTTCTGAAAGCTTTGGATGTTGGACACACTTAAGG

ATGTCACGAGCACAGACAAATACTGCAAACATGGGAAGCGGAAATAAGCG

TTAATTCATCAAGCCTCTTCTGATGATACTCGCAAGCGTTCACGGAAACT

TCAGTTCATTTTCGGCTGAGGGGGATGTAAAGCTNTAATGGCGGATGAAC

CTCGGGTCTAGCCAGTTAAACGTTGGGGCTTCCGAAGTTCAATCCTCTTC

GAGAGCTCCTCAATCACCNAATTGGCAANAAGAAAAAGTCCGGAGGATAT

TTGACCAGAATTTAATTCAANAGTAGTAACCAACCAATCTTAACTG

>TSBB.R70.esd 639 0 639 ESD GOOD: 93-623

GCACGAGGAAGAAATGCATGGAGAAGTCTGCTTACGAAGGAGGAGAGTGA

ATCAGTTTCATTGCTAGGAAATAGCTATTTCAATAGACATTTCGTTTAGC

ATCTCAATTGCAGACTTGTCACTTTTAATTGACGCACTCAGCATTTGCGC

GTTGCTTCACATTTGAATGGAAGGTTTCCGAAGGGAAAGAGCAATAAAAG

GCTCCACCGTTACACCTTCCTTTAAAGGCATTGCTGCCAAATATGGCCAT

TGGTCTCTCTAGCTCCTTCCTGCGTCGTCTTTACACACTTTCTTAGCATC

ACGACCCGTTCTCTCTTTGGGAATACCTCCGCGACCTACGCAAATGCATT

CATCTCAATTCGCCAGACAGTAAATTTCAAAACATTCATAACTGTAAACA

ACTCAAAGCACGAGTTACAAATCGTGTAGGTGGCACAAATCTCTTTCTCA

CTGTGATTAGTCCTCGGGTTAGTGTCACCTCAAAATCTGTTGTAAGTAAA

CCCTGCCAAAGAGCAGCAATGCCATCGCTTA

>TSBI.R33.esd 594 0 594 ESD GOOD: 91-371

CGGCACGAGGGTACCATGTATCCTGGGTTGCCTAGTCGGCTTGAACGGGA

GCTTAAGCAACTTTGTGTGCAGAACGTTCTGAAGGGCGATGCGACTGGTC

TTGCCAAAATGAAAATCCGAATTGAGGATCCACCACAACGCAAGCACATG

GTCTTTCTTGGTGGTTCAATTTTGGCTCGTTTGATGCAGGGAGGATCGGC

CAATTACTGGATTACTAAGGCGGAATGGAAGGAACAAAGTGCGCGTTTGC

TTGATCGGATGGAAAGTGTCTGATTAGGGCA

>TSBG.R2.esd 351 0 351 ESD GOOD: 92-316

TTTGGGACAGTTCCGATTATTCTTCATGGTGTATTGCCTTCAAGTGTGGT

TTCCTGCTCTTTCGATTGTGTTAAGAGTTTTCATCTGTTCTACGACGCGT

TAAACTTTAGCCACTGCTCTGACTACCATTTCAGTTAGCGTATCAATGGA

TCACGATAGCGTTGTCAGTGCTCGCAGCGACAAGAGTGGCATTAATCAAC

AGCAGCAGCCTGTTCTTCAGAACTT

>TSCC.R54.esd 694 0 694 ESD GOOD: 102-499

GCACGAGGTGGTTGTATACCCTCTGGTGCCTGCCGACTGTTCCTACTCCA

GACATGCCCGGAAGTCAGTGTGAAAAGCGTGATGAGTTATCAAGAGATGA

TTTGCGATACCTTGATACACGCCCATATTTGGATCGCACGGTGGTCCCCG

TCCTGATGCAAGCATTAACTGTTATCGCAAAAGAAAGACCACCAAATCCA

GTTGAGGCACTTGCGAATTACCTCTTACAACACGCCCACAGTACTGACTC

GTAGACTGGCATGGATGTGTGAACCACCCTTTGCCTCCATCCCTCGCATG

CTTTTCTTGTTACAAGCTACGTTAGAAAATCTGTTANCTGTAGTTATGGN

ATGTGTAACTTTNNNNNNNNNNNNNNNNNNNNNNNNNNNNNNNNNNNN

>TSAR.R21.esd 641 0 641 ESD GOOD: 104-412

GCACGAGGGGAAAATAACGCTCCGGTGGAATAACCGGTGTGCTGGACTAG

TTCCAGGCCTATCTTTGGAGGGCTCAGGCCTTAAAAAAAAAAAAAAAAAA

AAAAAAAAAAAAAAAAAAAAAAAAAAAAAAAAAAAAAAAAAAAAAAAAAA

AAAAAAAAAAAAAAAAAAAAAAAAAAAAAAAAAAAAAAAAAAAAAAAAAA

AAAAAAAAAAAAAAAAAAAAAAAAAAAAAAAAAAAAAAAAAAAAAAAAAA

AAAAAAAAAAAAAAAAAAAAAAAAAAAAAAAAAAAAAAAAAAAAAAAAAA

AAAAAAAAA

>TSBR.R42.esd 646 0 646 ESD GOOD: 104-567

GCACGAGGAGACAACTCATGGGACTTTATCTGCCATCAATGGAGTTGTTC

CTAAGGTTACATGCAACCGGAACTAAGATGTGCTAATTCAATTAAATGCC

AACTATAAGATTGCATCCATTGTAACAACAGTTTCATGCCGTCGGAAGAA

TGCATTGTCAAAGCTAAGTGAGCCTTCAATTTTCTTTATTATTTGCATAT

ACAATTTGACAGTCAACTGTCAGAAATAGGCAACCAATTTTCGAACGGTA

ACACAGTCGGATGCAGAGCAACTCCCGATTCGAACATAATAGTGCTCCTG

ACAGAAATGCTAACTAGCTCTGTCCACTTCCCTACATAGCATTCGCCTTC

TTCGGTCAATTTATCAGATTTCAGTTACGCGTCTGTTGTGCAATATGGGA

CGTGACAGACACCATAGTAACGTTAATGAAACCTAGGTTTCCCTTATTTT

TTCTCAAGAAGTAC

>TSBA.R73.esd 642 0 642 ESD GOOD: 125-560

GCACGAGGCAGGCAACGGGTGGCAGAACTTCACGCGCGGCGTGACAATCT

GATTTCCGAAGCTCTTGCTGAATTGCCGCATCCGGCAGCGATAGAGGTTG

CTCGGGGTATCAATCGATTTAGAGAAGCGCTTACGGACTTCTTAACCAGA

CCACGTGAACAAAATGTTGTCACGGAGGAAGAGGTCAAGGAGTTTTGCGA

GGATCTGAAGAAGGACTTGTGATTCACAGCGAAGCATTTATACTTGTTTT

AAATGTGCGCATGTATTGACTGTACAATCCTCAAATACCTTTTGCCTTTA

CTCCAGTCGCTTCAGCTTCGCTACAAAGAAGCCGTCCACATTATGTTTGT

GGGGGTATAAGCGTCGTGTCCGATTCATCTTCGGTGAGAAGTGCAGGCCC

TTAAAACTGCGATAAAAGTTTTATCACAGAACAAAA

>TSAJ.R22.esd 605 0 605 ESD GOOD: 101-364

GCACGAGGGCAAGTTTATGGCCGAGGCAAAGCGCAGTAAATTCGTCAAAA

TTTCTGGTAAGAGTCAGGCGCTGTCGCAGAAATCCATCTTGGACTCTTAG

ATTGTACAGTACAGAATATGGTAGAAATGTTTCTGCTTCAATAACGATGC

GCGTGCAGTTTCGCGGTTGTTAAAAAAAAAAAAAAAAAAAAAAAAAAAAA

AAAAAAAAAAAAAAAAAAAAAAAAAAAAAAAAAAAAAAAAAAAAAAAAAA

AAAAAAAAAAAAAA

>TSAD.R90.esd 515 0 515 ESD GOOD: 92-500

GCACGAGGCTGATGCCACCCTGTCGGGCAACCCTGGGAGTCGCATCCGAC

TCAATGGGCCAAATGCAACGTCGTATCTGCTCCACGACCCCCTATGCTGC

AGCCCACGAGACAGCTTTCCCATTGGCCTCTAAAGTGGGCATCGGTGGAG

GCTGGTTGCGTGGCTCGATGTACCTTGAAGTTCCCCCCGAAAAGCATCTA

CCAGGGTGGATTCTCCCCGACCTTGGTCCTTTCTTCTTTTAAGTGGCCGT

GTGTCCCGTTGTCTTCCGTCTTCACTTCTTTCCCTTGCTCTCCTTCCTCC

CCTTCACTACTCCTCTCCTCTTGTCTCAACTCTTACTTTTCTTCCTCTTT

GATCGTTTCTTATGACTTCTCCATCAGGCCCATCCATTCGTACCTCTGTC

CTTTTCTAC

>TSAY.R88.esd 572 0 572 ESD GOOD: 87-298

AAATGTGGAGCTTCACATAGATTCCCGNGAGTTGTACGGCACTGGTCTCT

TCTTGGGCACAGCTCACCAAGCTTATTTGGGCAAGGCGGACGGAAGTGTC

CTCGTGATGAAAAATCCCAGTGCCTAACTTTCTAAACTTCTTTGCATCTT

TTTAGTCAATTTAAGTACTTCAAATACATTACTAAAATTCCTTTAAAAAA

AAAAAAAAAAAA

>TSBD.R29.esd 551 0 551 ESD GOOD: 90-245

ACGGCACGAGGGTCCAACCCGCCATCGCGTGGAGTTTCCCAAGATCTCCG

TTATCTCTACTACGGCACCGTGTGTATATATCTACAACGTGCTCCTGGCA

CTATCCGGCTACGCCCGGTGTGCCCTGCCGTGTCACTCAATGGGCTGTCA

GTCCAT

>TSCB.R83.esd 585 0 585 ESD GOOD: 103-585

GCACGAGGGCGGATCTTACCAGAGCCTGTGGCAAGTACTGTTCTGATGCG

ATGATTTGCGTCATAACGAATCCGGTGAATAGTACAGTGCCAATAGCGGC

GGAGGTACTGAAGAAAGAGGGTGTGTACAACCCGAGGAGGCTGTTCGGTG

TGACAACTTTGGATATCACGCGGTCGAATGCCTTCATCGCTGAGGCGAAG

GGGCTGGATGTTGCAAAGGTCTCCTGTCCCGTCATCGGTGGTCACTCCGG

CAACACCATAGTCCCAGTCTTTTCTCAATGCACTCCGTCTGTAAGCTTCC

CCCAAAGAACGCGTGAACAATTGGTGGCTCGAATACAAAACGCAGGCACT

GAGGTGGTCAATGCGAAGGCTGGCGAGGGCTCGGCAACCCTGTCGATGGC

TTACGCGGGGGCGCGTTTCGCCAACTCCCTGTTGCATGCAATGAAAGGTC

ACGCGGACATTGTGGAGTGCGCGTTCGTGNNNG

>TSBY.R5.esd 503 0 503 ESD GOOD: 97-284

GCACGAGGGCGGGGCGCTTTCTGCTCCAGAGCTTCAGAAAGCTCTTTCAA

ATGGAACTTGGACACCGTTCAATATCATAACTGTACAGGCAATGATAGAT

CTTTTTAGTCTTAACCACTCATTGGACATAAATTTTGATGGATTCCTCCG

TCTTTGGGATTTCGTTGAAGGCTGGCAGCGCTACTTCC

>TSAU.R84.esd 400 0 400 ESD GOOD: 91-400

GCACGAGGATAGGTTCCGGTTTTGGCTATCCTCTTGGAAAAAAATTATGT

CTGGGTGTCATCGCAGCAATCTCTTCCAAAGTCTCCAATTTTTGCCAATC

GAATGAGGCAAAAGGGAGTTATTGAGATCACGAGAAAGGCTCCTAGATTC

TCCTGTTCAACTTTGATTTCCCCGAAACTACTTTTCTGAAAGGAGAATGA

GTAACCTGTTGGCAGATTTCAACAAAGAACAAGTTTTTTGGATCTTCACA

TTTTTGTTTTTAAGCACTCCTTTTCAATTACTTTTGGTGGCTCTATGAAT

GTGCTCCNNA

>TSCH.R19.esd 752 0 752 ESD GOOD: 110-620

GCACGAGGGTAATACCTTGCTTTCCGTATGCTCGGCAAGATAAAAAGGAC

AAATCTCGGGCACCAATTTCCGCAAAACTTGTTGCAAACATGTTGTCAGT

TGCCGGTGCTGATCATATCGTGACAATGGATCTCCATGCCAGCCAAATTC

AGGGCTTTTTCGATATACCTGTGGATAATCTCTACGCGGAGCCAGCTGTC

ATCAAATGGATTCAAAAAAACATTGACGATTGGCAAAACTGCTGTGTCGT

CAGTCCTGATGCTGGTGGTGCTAAGAGAGTGACCTCACTTGCCGATCAAC

TGAATGTTGAGTTTGCTTTGATTCATAAGGAGCGAAAACGCGCTAATGAA

GTTGATCGAATGGTCCTTGTCGGTGACGTCAAAGGAAAGGTTGCCATTCT

TGTCGATGACATGGCTGACACTTGCGGTACCATCTGTACTGCCGCTGAAC

GTTTGACTGAGGCTGGAGCTACCCGCATTTATGCAATCTGTACTCCATGG

TATCTTCTCCG

>TSAY.R75.esd 547 0 547 ESD GOOD: 89-356

GCACGAGGTTCACGTCTTAGCCGTTCGAGGTTGGGGTTTTAAAAAAAAAT

ACTTAAATTGGGTTGAATGATAAAACACTGTCATCTATACGGATGGATAG

AGCCTTCGAGCTTGCTGAAGAGGCGTTGAATGCTGGTGAGGTACCTGTCG

GATGTGTATTCGTATATTACGGAAAAGTAATTGCGTGGTGGTAGAAATGA

AACGAATATTTGTGGTGATGCAACTCAACATGCTGAAATTGTGGCAATAA

AACGGCTGGAACAGTGGT

>TSAB.R72.esd 625 0 625 ESD GOOD: 94-546

GCACGAGGCTCAGGCAGGCCTAGAATGCGGGGATTCCGTCCACTCTACCT

GTGGATTTTTATTGCCCGCAGACGTGCTGATTAGCTGCATCGCTGACAGG

GTTGAGTACTGTTGCGGGACGTGGCGACACTTCAATTCTAAAACTACTCC

CAGGGTGTTGGTTCCCACGTACGATGAGTGCGGCGCGTTCATGATGCTGT

TAAAGTGCTTCGACTGGAGCAACTCAACGCGGGAGTTTTCGAGTTGGAGG

GGTCGCCAGGTGCGTCAGTGTTAGAATTCTTGGATCTGTGGTTGTTCATG

TATCGCTTTTAGTTACAGAACGCTGAAATCGGCATTGCCTTCCACGGCAG

AAGCGACGCTGATCACCACCAATGAATGTGTCGCCTCTTAACGTGCCATC

GTTGATATTACGCTTTGATCAATAAATACATTTGCAAAAAAAAAAAAAAA

AAA

>TSBU.R3.esd 677 0 677 ESD GOOD: 92-317

TTCCAATCCCTCCTCCTCGTCCAACGATGCTCTATTTTCTCCAATCCTCC

CTTCGTCCCCCCTGCCCTATCCCGTCCTCAACTTACATCATGTCTGTGTA

TTTTGGAAAAGATGCCTTTTTTCATTATGCATGATTTACCAGGCTTTCCC

GTGCTTTGGTAAAGTAGATTTTTTGCTTTTTTGTGTTGAAGAAGATCTGC

TTTTGGTTAAAAAAAAAAAAAAAAAA

>TSAT.R57.esd 679 0 679 ESD GOOD: 107-631

GCACGAGGGTCATGGTAGTTACTGGGGGGAAAAAGGGGATGTGTGCGAAC

TGCACTTCCTGAATTTCTGTTGCTTTCCCTCAAAACCGTGAAATAGCATT

CACTTATTGCTGGTAAAACGTGCTTTTTCTTCTCATTATTTTTGGGTAGA

TCATTATCGCCAACTTACGTATGAGTGACTTTTATGTTAGGCAGAGATGT

GGTGCTGTAGTGGTGACCACGTTGTGAGGCGAAGCACACACCCACGAACC

ACCTCGACGTATCTGCTTCCTCTACTGCAACGTACTGTAGTAATAGTGGC

AGTAGTTGCAGTTGTACCGGTAGCCCCAGCGTGGTCTTGCCCACCCCTTA

CCGCATTACTGTGTTATTTCCCTTGCCATGTGGCTGAGGCCTCCTGGTGG

GTCTCACTGCCACAAGCAAAGGCACCTGCTTGAAACTTCCTGCATCCGGA

GCACCTCAGTATAATTGTAAACACCTCAATTCATTTACAAATTCTTTATT

CACCTCCTAGCACTAAGAGTGGTTT

>TSBV.R58.esd 593 0 593 ESD GOOD: 123-578

GCACGAGGTGCCTTCAGAGGAAACAATTCATCACGTCCTAGCCTACCTCT

CTAGCATAACACGCCCTTCAAAAGCCGTCAATGTGGAGAGCGAGCCTTCT

AGAACCAGCATCACATCCAGACAGCCAACAGTCAAGTTTTCACCCAATCC

TCTTATGATTCCTGCTGATCAGACCCAAGATGGATTCTATGTGGAACCAC

TGATAGTTGGCATCGATCGACTTACCACAAAGGTAGGCTCCCAATCCTCC

GAAATTAGTAGAACCTCCTCTACAAAAAAAACCTCCAGTTCCAGCGGGGG

AGTTCAACTGCGTAGACCAAAGCGATGTTTCACTGGCTATGCCTCTCAGG

GTAGCAGTAGTCTGGGTGTTTCCAGTGGTGGAAGTGGTGGAAGTGTTGGA

TCAAGCAATCGTAACCGTATCTGTTCGCATCGCGACAACGATGAACTCTT

TGCAGA

>TSAI.R84.esd 769 0 769 ESD GOOD: 100-579

GCACGAGGATTGGATGGATCAGCAGCAACAACCCCGCCACCTTCCCTCCT

CCTCTCTCACCGCTATTCTTTCGAGTGGTGGTAGCACCGGTAGTAGGCCT

CCACGAGGCAGTCTCCCGGAGAGCACTAGTCCTCGAGCAACGTGGCAGCA

GCATCAGCAACAACTGCGACAGGGTCTACTGGGAGGACACTCCAGCACTC

CGCCACAGGCAAGAAAGCGACATGTCGAGGACTCCAGACTGTGTTCAGAC

ACTTCCTCAGACGATGATGGCCGGTTTGGCGATTTCACTCACCGCGGATT

CTGACGCGCTCACAGGGCGACCGCTTGTAAATGTTTATCGAGTAAAGTTA

GTCATCCTCCGTGCTGTTCAGCGTCTTTAATGACTGGACCAAGCACTTAC

GTTCTACCTGCGAAGCTGCGGCGCTGAAGTTTACCCGCAGTCTTGGTGCG

TACACTGTGTACACTGATCTCTTCCCCTAC

>TSAE.R12.esd 712 0 712 ESD GOOD: 95-587

TTTCACTCGATTTTAGTGAACACTTTAATCGACTAGGTAATCTGCGTCAA

GGTGGCCAAACGTTTGCCTAAGCAATATAAATTCTCTTTTGTAACCAATA

GCAGCATATCAAGCTGCCACTCGGAAATGTGGGTATGGCCAACTCGAACA

AATTCCATACCACTGCTTGTTTTAAAATTTGTTTTAGGCTAGATTCTGAC

AAGCTTGTTGCAGTCGGTAAAAGGTTTGCATATGGTTTAGATATGTGGCA

CCTTTAAATTATGGATAAGGACGGTAACCCTGTTCCCCCAAATTTCCTAT

TATCATTAGTTAAAACCAAACCCAGGCGCCGGTTCCCCCTTTTACACTCA

GACACTTCCAATTAGCTGGCGTGGTCTGGTAAGAAAAATATTTTCCCACC

TCTCAGTCCAGAGCAAATTAGAAGTGCGATCGTGAAGGATTGCTTCAAAA

GCGAAATGATCACTCAGGGCCTAATCACAGACACAATATTCTT

>TSBC.R7.esd 623 0 623 ESD GOOD: 100-623

CACGAGGAGTGCGGGAGGTTTCCCTTAGGAAACTTCGGTGCTTGCTGTTA

GTTGGTTATGGCTGGCTCAAGTCTTGCTATCAACCGCATAAAAAAGGAGC

TTTTGGAGTTGCAGAAGTCTACCGACAATGCTGATAATCCCATTGAAATT

TATGCAGAGACCGATAATATAATGAAGCTGCGTGGTGTTATAGTCGGTCC

GCCAGATACGCCTTATGCAGGGGCGAAGTTTATATTGGAAATAATCATAC

CTGAAACCTATCCTTTTCTGCCCCCAAAAGTCAAGTTTCTAACGAAGATC

TGGCATCCGAACATTAGTAGCGCTACTGGTGTCATTTGTCTGGACATACT

GAAGGACCAATGGGCGGCTGCAATGTCGCTGCGTACGATGTTGCTTTCGA

TCCAGGCGTTGCTTGCCAGCCCTGAGCCGGATGACCCACAAGACGCGGTT

GTGGCAAAACAGTTCAAATCGAATCGAAACGCCTTCAATATAACTGCTAA

ACATTGGGCCTCCATCTATGCNNG

>TSBN.R27.esd 452 0 452 ESD GOOD: 78-420

GCACGAGGGTGGGATGGCCAAGAGTGGGCGTAGTAAGATCAAGAGGAAGT

TTAGAGCGATGAAGCGACATAAGTGTAGTGCGAAGTCTGCTGCNTTTGGC

TTAAAAAGGTTATAAATATGCCCGTGGGTGGTACAGGTCTGCCTTATGTC

GATGCCTCGAAAGCAGAGGTAATTGAAATTGTAAAAAAGGAGGAAGTGAT

GGGGACTGAGGGCAAGAAAGAAGCCACCTCCTTTAATGAATGAACATGGG

AACTATCCTGTAGGANGAATAAAAGGAGATGCGTCGGGCATATTAACCAG

CGGAAACGTATCCTCCGCAAGGAAAAGGAAAACTGGGCAAAAA

>TSAI.R89.esd 819 0 819 ESD GOOD: 104-238

GCACGAGGCCATGATCAAGACGCGGTTTTACCACAAGGCAATCGAACTGG

AGAAAGATACTAGCTTTACGCGAATAGAAATAGAGGGACTGCTGCGTATA

TATGACCAGATTGTTGTTAAGTACCCGTTCGCCCC

>TSBT.R67.esd 730 0 730 ESD GOOD: 108-424

GCACGAGGCACAGGTGTTAATCGTGAAATGTGTGCTTTGTACATTAATCA

ACTGTATACTGTTGTTACTCTTCACCTGTAAATTTGGATTTATGTTTTTT

TCGCCACATCACTATCTCTTTTTACCGGCCCATACCTTTTGCCGTTTCTT

TGGCATCCAAATATATGTTGTAATCAATTTCTAAAAAAAAAAAAAAAAAA

AAAAAAAAAAAAAAAAAAAAAAAAAAAAAAAAAAAAAAAAAAAAAAAAAA

AAAAAAAAAAAAAAAAAAAAAAAAAAAAAAAAAAAAAAAAAAAAAAAAAA

AAAAAAAAAAAAAAAAA

>TSBK.R9.esd 550 0 550 ESD GOOD: 108-550

GCACGAGGAAATGTCTCAAACATCATCGAGAGCAACTGCCAGGAAAGTAG

TTGTCCCATCGCGATATCGTTCTGCAACACAGGAGCGTCCATGCAATTTT

TCGACTGCGTCTACGACAGGTCCATCCACTTCATCTCCGAAGAATGATCA

AAAGGCTTCTCTCGATATCCATGACTCCGTTGTGTCGATTATTCCTTCCA

AAACAGGGTCTTCTCTGGTCTCAAAATCGGTCCTTGGGTTACAGCGATGT

CGGTCAATGTCGGATGTTGGGAGAACTCGATTGGATTTGACAGATTCCGA

GATAAAACTCCCGATCCCAATAGAAGCCACTTTGGGGNNCGTTTCTTTTG

GGAACACTCCTAGAAAGGCAGTCGCAGAAGCAAAATAAGAAGGAAGAGGC

TCTTAAAAAGGCTATTGCGGAGGCACACGATCGCGATGCTTNN

>TSAO.R27.esd 605 0 605 ESD GOOD: 90-370

GCACGAGGGAGAAGCTGATGATTTTCTCGGAGGTAGAAAGAATGAAATAT

GTGAACCTGTATATGCAAATATTTTTCCTACCGAGTGGTGGTCCGCCTTG

CTTCGCAGGGAATCCGACGAGTGGTTTGATGCGACAAGAGAGGTATTGGG

CAAAATAAATGCTGATGAGCCTGTTCTTCGTTCAGTTGCAGTGGTTTCTG

AAGTCTCCGNGTTTTGAGAAAGAGCTTTTGGGCAACAAAAGAGCCATTAT

GAGACTAAAAAACATCATCAAATTGCCAGTC

>TSBQ.R34.esd 778 0 778 ESD GOOD: 90-575

GCACGAGGCTGGCTTGAGGCAAGTTTTAGCCCAGCTAAGATGCATGGTGG

GCATTTTCTGGTTTAAGGAAGCTACAAACGTGTTGAGACTGGCAATTTCT

TATAGGAACATTGGCGCATTGGTTCCTCAAGGAAATTTATTTTTACTATC

TTCCATAAATGAATGCTCCACTTATTGGTTAACTTCCAGCTATTTCCCAA

CCTTCCAACCCTTCTGCGCTCCTTCTATGAACGTACTCTTTCCTTCCCCT

TGATTCGCAACTGGAAGCTCTGCCGAAAGGTTCAAGCCGACGTTGCTTCT

CTGCTTCAGCACGAAAATGCAAAAGCGTGGATCCTCTACATTCTCTTGGA

GATTCGGAGAATACTCATCGACTATCCCGGATACTATATCTTCGTTGACC

TCTTCGTCGATGACTACATCATTTGGCTCCAAAAGTCCGCCAGCACTTAC

GTCCTGAAAAACTTGGGTGAAGCGATTGGATCAGTG

>TSCA.R83.esd 709 0 709 ESD GOOD: 107-613

GCACGAGGGCACCACAGCAACTGGAGCACATTATCGGTCTACAAGTGCTC

AACGTCACAAAACTCCACAGGCATCAGCGACTCTACAACGATGCAAAGAG

GTTGACAACTGTGACACTGATAATAGTGGTGCCACTTCTACGACTAGCAC

CTTACCCACACTTCCACAGAAGTGGTCTAGTGGACCAGAACTGCATACGG

GCTCTCCTGTTATCCTTAAATTTCCACCCCCTCCTCCAGCTTTTCCTCAA

CAACAGAAAGAAAAAGGTCTTGGGGGTAGTTCCACTAACATTCGGATGGG

GACTCGGGCTCTTCGAATGTCAAGAGAAAGAGACCTGACCGGAAATATTG

AATCAGGACCTCTAACACGAAACGGAGAGGCTGGGAGGGATGACATTCAA

CTTTCCACTGTCCCTGTCATCTTACAATGTGAAACCAGCGTTGTTACTAG

AAGTCGTACAGCGACATGTCATGGGGTTGTGGACGTAGAAAAAGGAGCAG

CAGATGG

>TSBR.R80.esd 558 0 558 ESD GOOD: 101-507

GCACGAGGCTCAACAGCATGTCCAGTCCAGTGTTGAGGACGGCCAGGAAA

CACGCGAAATCGATCAGTCAAAGTTAAGGTCATGTGCTCACCTTCACGCA

CAACGTATGCTCCAGAAGCCGCGACCTTCACTCCAACAGAGGAGCCGTGG

ACTCGTCCATGTCACTTGTTGTTATCCTCCCTTCCCAACACTTTATCGAT

CAATTCGTGGGAAAGGGGGCTTGGCACTTGATCACGCTCGCAAATATTTT

TTCGTGCCTTTGGGGCGTCTGCGCAAGTCACTTGTGTGGTTTCGACGGCT

ACGGCAAGTGGAAGTTTTGCTTCAGTACTACACCCGAATGCCAAAATCGG

CTCGTCTACTCCTTCTATGCCAAAAGCGGAGTGCTAAAGTGCTGGATGAG

TCTCGGT

>TSAU.R16.esd 506 0 506 ESD GOOD: 93-464

GCACGAGGCGTTGCAAGTCCAGGTTCAGAACGCGAAGAACGAGCCCTTTG

GAACACCGGAACAGGTTTGTCCTGCTCTCATCTTAGTTTTTTACCATTTC

ATGCGAATTTCTAAATAATTGTACTGGCTTTGCGCAATTTAGCAGGTTCA

ACATGCCAAGGAGGTAAGGTGGCTTCANAAGGCAAACTAAATTCGCGAGC

TCACAATTGGATAATCCTGTGTTCTTCAGCTTCCGCTGGATTCTCACCCC

TCTTTGAATTATTTCACGCTTTGAAGAAAATAAATTTGGAGGATGATTAA

ACATTGCTAGAAAGCATTCCAATTGGAAAATCCGTGGCCATTGAATTTAG

TTCATTGGGAATTTCGATTTTT

>TSBQ.R46.esd 801 0 801 ESD GOOD: 99-606

GCACGAGGATACCGCGAAAGAGCAGATTTATAACCGCTTCACACTGACGC

CGGAAGGCCGATCTACCAAACAGTCTCTAGGACGTCGACACCAACGGTAA

CATTGAGCGCTACAACATCTTCATTGGGTACCAAGTCTGCTCCCACTCCT

CCACCTCCGCTTCCTGTTGTATCTACTCCATCCTCTTCCACGATTTTGAC

GGTTCCTCAATCCCTGACGTCATCAACAACATTACGGACTCATCCTACTT

CGATTGCCAAGCCATCGTTATCGACAACGCTGGCAACGACGGCAACAAAT

ATCGCTAGTAGTTGCAGTAGTGGCGGCAGTAGCGCACGTCTACAACAGCT

ACCTCCTCCACTGCGTAAACCGATGAAGTCGTGGTCCCCCACCCTGGTGG

CAGAGTTTGTGCGTGGCACGCCGAGCTGCTCTACCTATGCGGATGCCTTT

GTGGAGAACGAGATCGATGGCGAAGCTCTGCTTCTCCTTACACCAGCTCA

CTTTATTG

>TSBO.R53.esd 699 0 699 ESD GOOD: 98-479

GCACGAGGATCCTTACCTGAAGGCCCAGGCTTATATGCACTCCCTCTTTC

TACCAGCTTATTATTCGTGTAATTGTGCCGTACCCAATTTTTCACTCCGA

TGAAATCATGCTTCATAGTCGACCCTCTGAAAAGAAAAATTGTGAGACGC

CTTGCGAAGATGGTGAGTTATTCACGGAATATTTGGGGTGGATGAACGAT

CACACTTCTGAAATTCAGGGGAAGCTCTACTGCACCCGATGCCTTGCAAA

ACTTGGATCCTACAATTGGTNTGGTGAGCGGTGTACCTGCGGCCAGTGGA

TCACTCCGGCCTTCCACTTCTCCCGGAAACATCTTAAAGAAATACGAACG

CCTACATTGGCAATGACAACAGCGACACAACG

>TSBT.R58.esd 803 0 803 ESD GOOD: 107-545

GCACGAGGGCAAGGTGTACCTCGGAGGCGAATCAGAGGTGGGAAGGCCGG

TCACTCTCCACATCGTAGTCCTTTCCGTAGACAAGCCCAGTAGTGACCCT

AATGATGGATCTTAAGCCCTGCATTTGTTGATACATGTGAAGACAGAAGG

ACTTCTACTGTTCCTTCTGCAACTCTATGTCTAGTTTCCATACGAGGCTC

CTGCCTTGTGGAACAATTGTTGGCCTCTGCTTCCTCGAGCACGGCGCGTC

ACCAAGTCCAAGTGGTGGTTGACCACCCTACGCTGACCGTTTCCTTGGCA

AAACTGTGTCATCAGCAAGGACTGCTCTCAATTTCCGCTCCCTTGCGATT

GTCTGCCACGTTCATATCTGCATCCTGCTTGGTTTCCTTGTTAGATCTGC

CTTCGACGCGAAACGACTTGAGCAGCATATCCCCTTAGT

>TSBW.R31.esd 633 0 633 ESD GOOD: 95-563

GCACGAGGCACTTCACAGCACAAATGAGACCTTCTGCATGCTGAGTGCTT

TCCGGCTGGGAAGGCGAACTGGCGCTACATTCTGGCGCATTAACTGGCAC

ACAAAACAACAACAACAACAACTACTACTACTACTGATGATGATGATGAT

GATGATTTTTGATAAAAACTGTAAGAACTAGCGGCATGAAATGACCTGCC

ATCGTTAAATGAACTAACAATTACTAAAAGCCTGCTATTAACAGAACAGA

AATTGTTCCAACACATTTCCCCAACCAAACGGGTGGAAGTCCTACCTCAT

CAACCTACGCTGGCAATCGCGTCTGCCTGCGTCGACATTCCTTCCATGAC

TACTTTCCCTTCCCGTCTCTTTGTGGGCCAACAGTCGTCCTCTCCCTTTG

AGGCGCAGCGACTTGTTGTGCCTTCAACCACCACCTCGGTTTGTACAATC

AAGCCATCCACTCACTTCG

>TSAA.R28.esd 654 0 654 ESD GOOD: 102-598

GCACGAGGGAACCATCGTCTTTGCAGCAACGAACCGAGCTGATCTGTTGG

ACAAGGCTTTGCTACGGGCTGGTCGATTTGATCGTCACATCTTCATCGAT

CTACCCAATATAGCTGAACGCAAGGAACTTCTGGACATGTATCTCGGGAA

GTACAAACTGGCCACTAGCATCGACGTGCTGGCATTGCGCGACCGCCTAG

CCACGTGGACGTCTGGCATGTCCGGTGCGGACATTGCGCGACTCTGCAAT

GAGGCCGCCCTCATCACGGCCAGAGGTGTCCATGCTGAAGAGGGCATCCA

AGTTGCAGATTTCGATAGCGCCTTAGAGCGTGTTCTCGCGGGTGCAGCAA

AGCGTTCAAGTCCGCTTTCGCAGCCTGAACGTCGAGTGGCGGCAGTACAA

GAGGCGGGACGGGCACTAGTTGCTTCGTTGCTGCCCTCCACGGGGCTAAC

TCCCTTCCGCGTTTCCATTGTGCCTCGTGCCTCCACTGGTGACTCGG

>TSBH.R87.esd 510 0 510 ESD GOOD: 89-508

GCACGAGGGGTTGGCGATTTGAATTCTAGCTGGTGTTTACTTTCCGCATT

GCCATTGTCCATCCTTTGCCATGTTAGACGATCTACATACCTAGTCCGTT

ATGGGCACTTATTAAGTGCATACCCATCCTCTAGTCGTTATTGCCACTAG

CCTGTAGGGCACAAAATATACACTCGTCTTGGAAGTAGAAGAAGGACAAT

TCGTCATTATGTGTTGATGAGAAGTACAAGACATCCATGTGGTATGATGC

TACCTGTTAAGGTGCCGGCTGGGGAAGCTTAGTGTCAAATTCTTTATTCG

TGGACAGGAATGCAGCACCTGCAATAACAATAAAAAAGGACATTACCGTG

TTTAGAGTCCATGAGAATAAGTGAAAAGTAACAGCTGGAATGGAGACACC

TTTAAGTACTTAGTGCGCTN

>TSAO.R63.esd 780 0 780 ESD GOOD: 100-762

GCACGAGGCCTCGTGCCGATTCGGCACGAGGCATCAATTTTGCTAGCACA

AACAAAGCAAGCCCTTTCTCCTCATTTGAAGTGCGTTGCCAACGAGATGA

ATTCGCGTCTGAGGCAGAATGGAAACCATCGCTGCGTAGCAAGTCATTTC

TACTTTCCACGAATTTCACGCGACCTGAAACTCACACGGGCTGTTCGCAT

CACCATAGTCAGGCCCAAGTGCCTTCCATGGAAAGTTGGGTGTTTTTGGT

CCGGCTGTAGCTTTACCCAAGCTGGCCTTAAACACCCAAATCCCAATTTG

CCGGGCTCTGCCCAACTTTCCGGGGGGCCCTGTGCAAAGCCATTTGAATT

TAGTGTGAAGATGTTGGCTGGGCACTTTTGAACCACGAATGCTCGCGAAT

GCAGGCATCTGGAATTAACCTTAAGTTGTGGTCCAGAGAAAAAAATGGAA

AGCAGGGGGCAAAGAAGGTCCGAATTTAGGCCTCTCGAGGGGGGGTGGGG

GCCCCGGTTAACCCAATTTTCTGGCCGCTTTATAAGTTTTGAGTTCGTTA

TTAACAATTTCCCCTGGGCGCCGGCTTCGGTTTTACACACACGTTCGTGG

TCACTTTGGAAAACCCCTGGCGTTACCCCAACTTAAATTCGCCCTTTTGA

GGACATTTCCCCC

>TSBH.R6.esd 407 0 407 ESD GOOD: 98-341

GCACGAGGTTTTCTTTCTTTTTTTCACGGACTATTTGCGTGATTGTAAAT

AGTAAGGAGGGCGTTGTTAGGTCTTTGAAGTAGGATGTGTAGTCTCCCAC

TCCTTCTTCCCTCTCCCCTCCTCCCTCCCCAGCCCCTTTGGTGGGGGTTG

TTAACCTGGGATTTTTGCCGCCGAGCGCTAGAAGAGTAGTCTTTCTTCGG

GAGCTCCTGCAAACTTTTCCCAGTCTCCTCCTGGTTGCTCCCCT

>TSBF.R1.esd 564 0 564 ESD GOOD: 94-564

GCACGAGGCCTCTCCCTGTTCTTTAATATGGAAGAAGTTGATTATTATAC

GGTACTAAAGGTTGATCGAAGTGCTGACGGTGAATCCATCAAGAAGGCGT

ATAGGAAATTAGCACTTAAATGGCATCCCGACAAAAACCCGGATAACAAG

GAGGAGGCCGAGAGACAGTTTAAACTGGTAAGCGAGGCCTACGAAGTGCT

TTCAGATCCACGGAAACGGGAAATATATGACAATTATGGCAAAGAGGGGC

TTACCAATGGAGGTGTTGGTCCCAGCGGCTTTCCTGGCTTTGGCGGTTTC

TCGTTTCATTTCACAGATCCTATGGAGATATTTGCTCAAGTTTTCGGGCC

TTCGCTGTTTGATGTTTTTGGGCCCAACTTTGTCGCATCTGGGGTTGCGC

GAGCCCACCGTTCTCACACAGGTTCTCGTGCTCATCGGCGTCAAAACCCT

TATGAACAAGGTCGTCGTNNN

>TSCD.R13.esd 787 0 787 ESD GOOD: 101-627

GCACGAGGGGTGTCAATGGCAGCAGCAATGTTTCAACCCAATCCCCTTTT

GTCTACTCCTCCTATACACCCCCACAGCCCTGTTGTTCCATGTATGTCCC

GGGCGTTGCGTGGATGGCCACTCCCCATCACTTTTCCTCCACCTTTGTAC

CTCCACCCACACATCCATCCCCTACTTTCTTTGATCCCACCACCGGTGCC

CCGGTCTTTGCTTGTTGTCTGCCACCTCAGCCAACGCCACCACTACCACC

GCCACCGTCACAGGTCCCGCAGGTGCCTTTACAAACGTTCCTCCCACAGC

CAGATCAGGCTTTACTGTATTCACCACCACCACCACCACCGCCTCCGCCA

TTAACATCCCAGCAGCAGGCGTTTACGCCCCAAATGGGCTACGCGCCAAC

ACAACCCATGCCGATTCTAGAGCATCAGAATCAGCATCAACAGCAGCAGT

TTCAACACCTACAACCGCAGTTTCCTCTCGTAATGGATGCGACGGAGTGG

GTACTTGGAGCGTCGCCTTCAGCATAC

>TSAT.R39.esd 526 0 526 ESD GOOD: 102-521

GCACGAGGGTTTTGTGCTTAAATACTGAAGTATGTTTACTTCCTAATTGA

AGTGGTTATCTATTTGCTGCATTTTGTCTTCATTTCCGTACCCACTCACT

TTATGTGCGATATGGAAATTCAGTAATGTGCTTGAAATTTCCAAAGGTAT

GGATTCCCTACACTGTGGGAGCTATTGGTGCGCTTAACTTGGTCGTTAAA

TGCATCCTTCTACTCTTGATTCTTGCATGGCACGAGTATTTGGTAATTTT

CCATGAATTCGGATTTGTTCTTGAGGATAGCATGGTTGCGAAATTGGTAC

GACATTTGTCCTCATTAGTCCATCTTCCTCGGAGCTTTCCGGTCCGTTCA

AGGAAGTTCTGGCGTCCTTTCAAGATAATATCCCCTTTCTCTTGTAAATA

AATAATCGCTGCGAGCTTGT

>TSAR.R13.esd 652 0 652 ESD GOOD: 101-611

GCACGAGGGGAAAAGGGAGATGACTCCAAAATAAAAGGAAACCAATCTGT

GCAAACGGGTGTGGCTAGTCCAACCTGTCTAATTCTTGCACCTACGCGAG

AGCTCTCCTGCCAAATATTTGACGAGGCTCGTCGGTTTGCCTACCGTTCG

GATTTGAAGCCCTGTGTGGTCTACGGTGGTGCGCCCGTAAACAATCAATT

GCGCGATCTTTCGCGAGGCTGCGATCTTCTTGTTGCCACCCCTGGCCGTC

TCGTCGATGTAATTGGGCGGGAGAAGGTCACCCTGGAGAATGTCAAAATG

TCAGATGTAGTCAATGCGGTTGAGCTACGTTTGGCGAGCCCACTTGATAT

CAGAGCAATTCAACTGCTCTGCCTTGAATGTTTCCCTGTGCGCTATCCGG

ATCTGTGGTACAGCGAAATAGTTTCCACCGGCCGCTATCTCACCATTCTT

GCTTGTCTTCCTTATGCCAAGTACCGGCAGCTTGAGCTTGCTGGAGAGGA

GGATGGGGGGC

>TSBI.R82.esd 770 0 770 ESD GOOD: 104-575

GCACGAGGCCGAAAAATTAACTTCTCTAGATCTTGTACAGGCGACCCTCG

TTGGGTTGGTTTTCTCGATGATTCTCTGCTGCGCTGCAAGGGATAGACCC

GACATGGAATCTTACGAACCTGTGCATACATAAGTATCCATTTACGCGGG

AGCATCTTCAAAAGACTTCCTGTAATTGTCCTTCCTATCAATTAACAGTC

CAAAAAACCGGCTTTCGGCACAATACACACTCCAATCACTAATAGTGATA

TCAGCATAAACCGTTGCCATTTTACATAATTCCTTTAATTTTTGTGTCAA

TCCCCATCGCTATTGCCCCTCTTGCTGTTAATAATTTCTTATGGTACAAA

CTACTAGCCCATTGTTATTCCAGGCATTCTTGAGTGTTTAAATATTGATT

ACTTCATTTGATAGCATTTCTTCGACTATCCTTTTCGAACATGAAAGGCT

TACTCCAAAAAAAAAAAAAAAA

>TSCD.R3.esd 543 0 543 ESD GOOD: 93-304

GCACGAGGCTGGGTGAAGAATTTCTAGTGCTGGATGTTGATTGCTTTGCC

ACAAGATCATGGATGTGTCCGAAGTTTTTGATTCAGAGATAGGGAACAAA

TCCTTCAAGGACTTTTCTACTCTCCAAGAACTCCTTTCCGAATTTAGTCG

TAAGCATGGAGTGAAGTATAAAGTTTCGAATTCTCTCCGCTACAACGATG

GGGATCCGCGCA

>TSAO.R3.esd 705 0 705 ESD GOOD: 105-591

GCACGAGGCTAAGAGGAAGATTTCTAATTGGTATCAGGTCAAGAGGGAAA

CACTCGTGAAATCATACCGGGCTAAAGTTAACCACTTCAAGAAGATTTTC

GATGGCACGCCCGTGGACACGGATCGCCTTCTTGTCGATTATCCCTTGGT

TGGCCTCTTGGCAAAGAAACAAGAATTGGGTCTCCTCCTCCTAAGGGCGA

ATGGTTTAATACTGGAAAATGGGTATTGGTTTTATATCCAAAATTCTATT

TTATAACCTAAGGCTCCACTATAAAAGTGGAGAGACTGTCCAAACGTGTT

GTCAAAGGGCCAAAAGGGCCGCGTCTCATTCCAAACGCCATTCATATAAC

ACTCATGTCGAACAGTGAGCGATACTTCTTTATATCTTGTGCATCCCGAG

AGAGACCCTTTGCCATCCTCAAAAAAGTCTGCGAACACAGCAGAAATGGT

GGAGTATGTTTTCCTATCCATCCATTTCATCCTTAGG

>TSBW.R79.esd 588 0 588 ESD GOOD: 91-588

GCACGAGGCGAAAGGAGAAGGATAATGTCACTTTTGACAAGACTGTCGGA

GGACAGTTGACAAGTACGGTTTCTGCAACCGAGGGGGCACAAGGTAGCCA

GTCCAAGGCCTCACAGGCACTACCTGACGCTTTTGGAATGTTCCAAAAAA

CTTGGCTTCCGGCCTTCCTACCTTGGCGCAGCAGATTCTCCACCGAGGAC

CCTACACGAGACCCAGACTTTGCAGAGGCCACACTCCACTTCTCATCTTG

CCAGCCGGATATTCCGGGGCCCATCATACAGGAGGTAACAGAGACGACAA

CTGCAAGCGACGACAGTAGCAAAAGTGCTGAAGATGCTAGCAGCGAGACG

CAACAACGAACCGAAGCCAGAGCGGGCCAGATGCTTCCCTGCCATGACGT

GGGCGACGAAAAGTGAACAAAATCACCGTTTTGTCTTTCGAGTACATTCA

GGAAAGTTAGACCTACTCCCAACACACACCCACACGCATACACAGNNC

>TSCH.R24.esd 605 0 605 ESD GOOD: 103-488

GCACGAGGACGTCGTATTTTCTTGTGGCGTGCATCCACTCAACCAGAATG

ATCCCTACGATGTAGAAGATTTACGCCGTCTGGCGGCAGAAGAGGGCTTC

TGTCCATCTCATATACACTTTGTTTTTATGGGCTGCGGTGAAAATTCTTG

GTACCTTGAGCCCATTGCTGCTACCACTACCACCTCTCATGCATCAGTAG

AATTAAGACTTACCCACCGCCTTTCCTGCTAAACACCTCGATTTCTACCC

TAACCTACCAAGTGCGTGCTCCTGTCGGCAATGTTGCTCTCCTGTACTGC

GCGCGTGCTTGTGTGCAAACTCTGTAAATTGTTTTTCTTCTCCCCAAGAC

AACTATTTCTAGCGAAAAAAAAAAAAAAAAAAAAAA

>TSBD.R1.esd 539 0 539 ESD GOOD: 94-539

GCACGAGGCTTGTGTTCACATCGATGGTGTTCGTCGGTTTTCTACCTCGT

TTGATGTGGGTTCGTCAAGTTTCACCACTGCTTTGCGCAAAAACTCTACT

TAACATTCAGGCGAGACCCTTGAGCATATTATCCTTCATTTAGGCTTAAA

GTACACAAAGACGCATGAGTGGGTCTGCTCAGAAAACGGTACATACACTG

TCGGCCTGTCCAAATTTGCCACTGATTCCATCGGCGATGTAGTCTATGTC

GACCTCCCCGATCCCGGAACTAAACTACAAAAAGGTGAAAGCTTCGGTAA

TGCCGAATCTAGCAAAGCTACCTCGGAACTTTTTGCACCGATCAGCGGGA

CTGTCGAAGAAATAAATGAGGCTATCAAAGACAAACCATCCCTCCTAAAC

AAGTCACCTGAGAAGGATGGTTGGCTTCTAAAGATGAAGTCCGANN

>TSCD.R55.esd 714 0 714 ESD GOOD: 99-402

GCACGAGGCTTGATTCGGTGACGGCTCCTTCAAATTGTCTGCCGCTACTC

AACTGTTCGGATTTCGTAGGTGACCGTGCCTTATCACTGTGTGATAACAG

GGTAATCGGGGAATCACGGNTTCGATCCCTGGGTACGGAGAGCCTTGAAA

CAGCGAGGTATCAGCTTTCCGAGCGGTCAGGCAGTGATCGCGTCAGCAAA

GTTACCCTACTCTCCGGCATGGGCGACGTGGTCGACTGAAAAATACCCGA

TGCTGGGAGTCCACTCTAGGCGGCTCCTGCTATTCGGCACTGCGTGGACT

CTAG

>TSAU.R27.esd 672 0 672 ESD GOOD: 95-314

GCACGAGGCGGATGCCAGTGCCGGTAACCCACCGGTCGTGCGAACTATTC

GTACCCAATATCTTCCCCTTCTGAGTTGCGTCTGGGTGGGACAAAACAGC

CTCCTCGGCGGTGGTCACGATTGCTGTCCCATTCTATTCAACTAACATGG

TCCAGCCCGGAAAAATTTCGGGAAGGCCAAAATGATTGGATGTAANAGAG

TGAANCCGCAAGATGCACCC

>TSAY.R7.esd 657 0 657 ESD GOOD: 98-499

GCACGAGGGCAAGCTTAAGCAGAATCCACGTCTTCACAGACTTAGAGCCG

GGACAAACAATTTCGGCCACTGTCGCTGCAGTCTTTCGGAAGAATGATTC

GACGACGCCTGAATACATTGGTACCTACAGCGGAAGAGTGATCGCTCTTA

CGCCATATTCGACAGGAGAGGGTTCGTCCTCATCGTCCGCTGCTACCACC

ACTGCCGGGTCTGCCTACAGTGCATTGGATAGCATAACACTTTTCACCTC

CATGATAATAATGCTGACGTCAACCTCAATGACCCACTGATGCAGAGGAG

AGTTGGGCTGATTACCGTCCACCTGTATTTAGCAATCAGTTGTCTAATCA

CAGCAAGCTATTTTGCACTGAGAAATATAGCCATTGCCACTTCGCAAAAA

AA

>TSAA.R46.esd 639 0 639 ESD GOOD: 105-494

GCACGAGGCCGTGCTTATATCCGTCGAAGCGTGCAACGTGAAACACTTTC

ATTGTTTGTTTAACTCTTGGTTTTGGTTATGCAGGCACCAAAGGCTCCGT

CGATTGCACTATCGCGAGGCGAAATGGTTTTCTGGCACAGTACGATTTCG

CCTTTGACTATGGGAAAGGGTCGGTGCGCTACAGACAGAACGTTATCAAC

ACGGAGCGGTGCAAATCCACAAAAGCTTATTTTAATTAAACAGGAAGCAC

CAAGCAGACAAAGTAAGGAATTTGGAAGATGCGGAGCTAGTCGAATGGGC

GTTTAGAAGTAAAAATAAAAAGGGAAACCAGAGGCTGCCGACCAATTGAT

AGCATAAAAGTCACCAAAGCAGAGGTTGGGGCGTCAACCA

>TSBN.R57.esd 594 0 594 ESD GOOD: 99-494

GCACGAGAGCTGGGTTTAGAACGTCGTGAGACAGTTCGGTCCCTATCTGC

CGTGGGCGCTGGAGAACTGAGGGGGGCTGCTCCTAATACGAGAGGACCGG

AGTGGACGCATCACTGGTGTTCGGGTTGTCATGCCAATGGCACTGCCCGG

TAGCTAAATGCGGAAGAGATAAGTGCTGAAAGCATCTAAGCACGAAACTT

GCCCCGAGATGAGTTCTCCCTGGACTCCTTGGAGAGTCCTAGAAGGAACG

TTGAAGACGACGACGTTGATAGGCCGGGTGTGTAAGCGCAGCGATGCGTT

GAGCTAACCGGTACTAATGAACCGTGAGGCTTAAGCTTACAACGCCGAAG

CTGTTTTGGCGGATTGAGAGAAGATTTTCAGCCTGATACAGATTAA

>TSBT.R36.esd 552 0 552 ESD GOOD: 93-510

GCACGAGGGATTTGTTGTCTCAACTCGAGTTGCGTAGAAGCCNAACCCCC

CNTGNGAAANCCNTTTGGGAAAACCGGGGCCAAGTAGTGGGGGCCNCAAA

AAAANAAAAAANTTTTTGGGGGGGCCCCCCCCGGGGGGGGGGGGGGGAAA

CAGGGGGCGCACACTTTGGGTTAACTAACGAAAGATGGCGTGACTCCTTT

CTGTTTGGAGCGAAATCAACGCTGCAGGCATCATTGGGGAGGTAGTGAAT

TCGAACTCAAGAAGCGGATCTCCAACTTCACCTTGTTGTTGCCGCAGCAA

TTCCATCGAGAACAGTTCTACCGCTCATCCCATACGTCTTGACCCGATTT

ACCCCAACCTTGATATGTACTACAGGCTGGCTCCAAAATAACTAAGTTTC

CTTGCCAACTTCTGTGTG

>TSBI.R42.esd 637 0 637 ESD GOOD: 109-447

GCACGAGGGATTGTCTTAGCCAGTCGAGCTGTTGGTTCTTCGTCTAATGC

TCATTTGCTTTTTTGAAAATTCCAATACATCTGGTCGAATCACTCAACTT

TGTGGAAGAATTTAAACTTGTTGCAGAATATGCACCCATCATGAACGCAT

TAACACCTGAACCATCAGAGACAGATTCCGACTGCGAGTACAAGTATGTC

TTTCCACTTCGGCACGAAGTTTCGCCTCGTGATTACCTGAAATTTATCCT

CACCGTCTGGTATTGCCCTGAAACCAGTGCTGCTAATGCTGACCTCATGT

CCAATTCTTATGGCGAAGCACAGCTCAAAGAGCTTCTGG

>TSBE.R51.esd 260 0 260 ESD GOOD: 32-139

CACGCGGTGGCGGCCGCTCTAAACTATGGTCCCGGGCTGCAGGAATTCGG

CGAGACTTGTCGATAATATATAGTGTATATTATCAGTTAAAANTTAAGTA

ATTATATT

>TSAD.R84.esd 669 0 669 ESD GOOD: 95-565

ATTCGGCACGAGGCTTTCAATAGCTGACGCTGAAGCATCTTTCGATAAGA

TTACTTTTGAGAAGGAGTGGAGGCTCTCAATGCAATGCAATGGATGCTTG

TTTAATTCATGGTTGCAACTTAAGCGTCACTCAGATGCTCCGCCACTCCA

CCTCCTTCGAGTGGACTAGCCTCTGGGCCTTGCCAATAGCTTCCAAGTCT

TCCCAACTATTGTCCCTTTTTCGTCTCGGGGCGCCACACTCAATGGCAAT

GCTCCTTGAAGTCTATCCAAGTTGCATCATAAAGTGTGGCGGCAAAATAA

CGCGATTACTTCATCCGAGGACCCAGACAACGGAATAAGGAGATGCATTA

TATTGCTTCTTCAACACCGGCGAAGCCTCATATTGCTGTCGGATCTTAAA

AAGTCGGTCCTGATGTCTACTACTAGATGATAGATCTTAGTTGAACGGAA

GAGGTGCATATGCTGCGGATA

>TSAP.R82.esd 605 0 605 ESD GOOD: 96-303

GCACGAGGCTTTCTCATTCAAATGCAGGCCATGTACTTCCGGCGGCGGAA

GAAGCTGACGTCGAAGTTGAGCGTCAGGNTAGGTAGCGGACGAGCTACAT

TGCAGACCCCTAACAAACCCTCTCTCCGCTAAGTCCTATTTACACTCCCT

CTCTCAAAAAAAAAAAAAAAAAAAAAAAAAAAAAAAAAAAAAAAAAAAAA

AAAAAAAA

>TSBT.R9.esd 496 0 496 ESD GOOD: 27-490

CACGCGGTGGCGGCGCTCTAAATATGACCGGGTGCAGAACGACAGCCTCG

TGCCGAATCTGTAAGCAAATGGGAAAGCTCACGACAAGCCACTCTTTTTC

ACGCCACAAGCATCGCGCGTCCTGCACAATAACCATTGATAAACACCCCA

CCGCATTCCAGACGTGCGCGCCACTCTGTCTGGCCTGTGTTTCGTCAGTG

GTAAAAATGGTCGCTGAAAGGCCGAACTCACTATCATTAGCCAGTTCCAG

TGTCATGTGTTCCTAGGGGCGAAAANTTTACCCCCCTTNTTTTTCGGCAA

ATGGGTGTAATATGGGGCGCCGGGGCCCGAAAAAAAAACCCCGGGGGGCC

CGGGGGGGGGGCCCACAAAAANCGAATATTCTTCCCGAAACGCGGTCATT

CTGGGGTAACATTCGCCAGAAACACGGTTTGGTGGCCGGTGGATAGTAGT

TACCTGCCCCAGCC

>TSAA.R85.esd 718 0 718 ESD GOOD: 108-602

GCACGAGGACACAGCAACCGGAGCGCACAGCACTACCTTCTCTTCTATGT

AGACAGCCATGCATGTCGCTATTGTTACAGGAGCTAACAAGGGCATTGGA

AATGGGATCGTGGAGCTACTGGCTCGAGGCTTAAAGCCATCTTCCGATTG

GCACATCTACCTGACGGCGCGAAATGAGAAGCTGGGTTTGGAAGCCGTGA

AGGCGCTCGAGGAGAAGGGTCTAAGCGTCAAGTTCCACCAACTGGATATT

ACTGACGCCGATAGCCGGCACAAACTGGCCAAATTCATCAAAGCTAACTA

TCCAGAAGGGATCGACATTTTGGTGAACAATGCTGGGATCGCGTACAAGA

ATGACTCTACAGCCCCCTTTGGCGAGCAAGCTCGAGTCACTATTGCTACG

AATTACACGGCCAATGTGCAAATGTGTATCGAGTTTTTGCCACTGATGGG

GAAGGATTCAAGACTTGTTAATATCGCTAGTACAATGGCCATGGT

>TSCH.R25.esd 611 0 611 ESD GOOD: 97-480

GCACGAGGCCAAAAGTGCAACTCTGGGAGGGTCTTCGGCTAACCATCAAG

GCATTTCGTGAGTCCCTTCGCAACAACTCTATTCTTCACCGTCCAGAACT

GCCTCGTTTGCGTGAACATCGGTTCCTCTGAATACTTTATTTCGCATTGA

GTTGAGCCACCTTGTGTGCGTTCAATCGACGGCTCTACTTTTACGAGGTA

TTTTTTCCCAAGTGGTGGCAATGAGGCCAGCCAGTCTGCATTACTTGGCG

TACTTGTAACTTGTAATTATGTAGAACGAAATCTTACTTTAAGTTTTTTT

TGTTCGTCTGTTTTTTTTCCCTCATCACGCATTTTCGTGAATAAATAAGT

CTTGAGTTTAAAAAAAAAAAAAAAAAAAAAAAAA

>TSAA.R29.esd 707 0 707 ESD GOOD: 101-468

GCACGAGGACTTAAAATACACGATCCCTTCTCTGTGACCATAAGACGTGT

CCAGGCTGACCTTGCACCATCTCTGTGCTCGATGCGTTGGCCAGTAGGGA

CGTCTCTTCCGATGTCGTTTTCAGTTGTCACACTCGTTTTGTGGCCTCCT

TGGGATTTATAAAACTGCTCCACACACTAATGAGCTGTGAAGTGATTTTT

TGTCTCCCTAAAATTTTGCCCCTTTTTCATTGAGATTTGTTTAGCAAAGG

ACTTTATTAATGGACTTTTTTATTGGAAAAAATCTGAATTCTCAAACACT

TTTTTTGGCCTTCCACAATGTCCATCTCCAAATTGCTCGGAACATGTCCC

CAATTAACAATTGGGAAA

>TSCB.R62.esd 683 0 683 ESD GOOD: 105-641

GCACGAGGTACACCTCCACATCCTCTACCACCGTCAGCCCCGCCACTGTT

GCCTCCACAGTCTCTCCTGCTCCTTCCTCCTCCTCTCGTGCTGCGGAGTC

AAGCCTGGACTACAAGCGCCTCTACGAGGCCGAGCGTCAGGATACGCAAG

AACTGCGGACACAAATCGATCGCGCCCAGCAGGAGTTACGGGATCTGCGC

GCCCAAATTGAAGGGGCGCGTCGGTTAACATTACAACAGCAACAGAGGCA

GCAACAGCAGCAACAACAAAATCCACAGCAACAACACTGCAACGATGCCG

ACCAGCGGACCCTGGAAGAGCTTCGTGCCTCCGCAGAAAAGTTAAAAGCA

GAACACCGAGCCCTAACTCGAGTGATTTCCCGTCTCATGTGACCGCGACG

GCAACCATGACAATAAGACCACCTCGCGCTCTGACATCGCGGCGGCCAAT

GCGATTCTTGCTAATGTGCTTATTTTGTCTACGGTTGAATTCACTCCTCC

TCCCGTCTCCTCTTCCTTTCCTCTTTCTCTCCTCACT

>TSBH.R22.esd 591 0 591 ESD GOOD: 89-549

GCACGAGGGTCATGGAAATCGCGGCCCCCAAACGCTTATCACTCCAAAAG

CCACATTCCAACAGCCATGCATTACACAGCACTCTCGTTGTTTTGGGCAC

CGTTCCCGGTGCTCATCTTACAAGTGTCTTCTGCATGTATGCTATCGCGG

CTGGCTTTCTATCTTCCATTAATTTGCAAGCCTTTTCTTCACATCTAACA

GGTGGTGCGCGGAGTTGTATACTGCTGGCGGGGTGGCACTTGAAATCCCT

ATTTGTTCCACAGATGGTAGAACTTGGTCTGTGGCTCACGTGGCTGGACT

GGCCTGCATAGCTGTACCAGATTCTCTACTCTGTAAACATTGTTCTGCCC

GGTATATGACAATTGTATTTCCGTACAACATTGAAAGTTTGTCTCATCCC

TTCCTGTAGTCACCGCTGCTTTTGCATTTGGTAACGGACTGGCGATTCGT

CCCTTCGAATC

>TSBM.R59.esd 632 0 632 ESD GOOD: 101-596

GCACGAGGTACGGCCAGTGGGATGGTGTAGGAGGGGTGGAAAAGCAGGAA

CTCTTCGATAAAAGCGGCTTCTTTCTCCTCCAATTGGCCTGTCTGGTTGG

ACGCTCACCAGACTTGAGACGTCGACGTCGAAGGCCTATACGCATTAGGG

TCTTTGTTCCTGCATTGCCGGCGCTGGCTGACGTGGTTCTCACTTCTCGG

GTAAAGGAGGTACTTCAAGGCCTCAGAATTGAGGCCGAAGTCCATGTGAT

TGAGTTCAACCGCCAGTCAGGCGAAAACGAAGTGGAAACAATCAACCATC

TGATCTCCTCCCACTGCAATGCAGAGAAAGGAACTTCGGTCGTCTTTCTA

CGGCTTCCACTACCACCATCGTCGTCACAACAGACCTCAAATGAGGCGTC

AAAACTCACCTACCTGGATCAGTTGAGAGGGCTCACAGAGGAGCTTCCGG

CAACCCTTCTTGGGTTGGGAATGCAGGAGGTGACCTCGGACTCCCT

>TSAV.R51.esd 625 0 625 ESD GOOD: 106-378

GCACGAGGGGAATACTTGGCTGATCCAAGCAAGTTCGCCAGTGCTATTGC

TACTGCCCCAGTTGCATCCGAAGAAGCCAAGCCTACCGACAAAGGTGCAG

CCCCCGCCGAAACAAAGGCTCCTGAACCGGAGAAAGAAGAATCGGAGTCC

GAAGGTGATATGGGCTTTAGTCTCTTTGATTAAATAAATGTTTTAATTTA

ACGTGGGGNNNNNNNNNNNNNNNNNNNNNNNNNNNNNNNNNNNNNNNNNN

NNNNNNNNNNNNNNNNNNNNNNN

>TSAV.R18.esd 338 0 338 ESD GOOD: 101-271

GCACGAGGGTTTCTGGTTCCTCATAGTTAGTGCAAAATCGTTTCAACTCC

CAGCACTGGGAATGGCGACTAAAACCGATTCTCGAGATTCGAGAAGACCG

TCAGTTGAAATATACGTTCCTCCCAGCCTCCGAAGCTCCCGCTTGTTACA

ACAAAGGGAGCGCACTTCACC

>TSAF.R49.esd 690 0 690 ESD GOOD: 82-595

AGACTCGCGGTGCAAATGTGTTTTACAGCGTGATGGAGCAGATGAAGATG

CTCGACACGCTGCAGAACACGCAGCTGCAGAGCGCCATTGTGAAGGCGAT

GTATGCCGCCACCATTGAGAGTGAGCTGGATACGCAGTCAGCGATGGATT

TTATTCTGGGCGCGAACAGTCAGGAGCAGCGGGAAAGGCTGACCGGCTGG

ATTGGTGAAATTGCCGCGTATTACGCCGCAGCGCCGGTCCGGCTGGGAGG

CGCAAAAGTACCGCACCTGATGCCGGGTGACTCACTGAACCTGCAGACGG

CTCAGGATACGGATAACGGCTACTCCGTGTTTGAGCAGTCACTGCTGCGG

TATATCGCTGCCGGGCTGGGTGTCTCGTATGAGCAGCTTTCCCGGAATTA

CGCCCAGATGAGCTACTCCACGGCACGGGCCAGTGCGAACGAGTCGTGGG

CGTACTTTATGGGGCGGCGAAAATTCGTCGCATCCCGTCAGGCGAGCCAG

ATGTTTCTGTGCTG

>TSBJ.R48.esd 610 0 610 ESD GOOD: 96-404

GCACGAGGGCTCAGAGAGCCGAGAAGCGATAGCCCGTGCGTCCACCTAAC

CTACGGCCGTGAGTAGCCTTCACATGCTCCCCGCGCACTACGCGATCGCC

ACCTGGCAGCCAAAGTGCAACCCACTTTTGTGCCACCTCTCTGCTCTATG

CCTGATTCTCTCTACCTATTGCTCACTGTTTGTCTCTTTTCTTTCTTTTT

CCCCCATTCATTCCCATTTAATTTACTCTCGCCTAATCACACGCATATCT

CCGCACGCTACTCACTTTCTACTTCAGTAGACAGTCATCAGCCTATCTCT

GGCCGACCT

>TSBS.R71.esd 511 0 511 ESD GOOD: 94-511

GGCACGAGGCTTCGATAGTGGTAATTGTGTGTAAACTGATTTGATTTGCC

TTGGCTGTTTGCCAAACAAATGCGCGCACAGAAATGCTTTTCCTGCCTCG

ACTTGTGTAAAAGACTCACACCTTACGTGCGTGAACAAGCTTTCCGCTGC

TCCTCTATACTCCCCTTCAGGAAACTGATCTAATGAATGGAAAAAAAATT

GCTCCGGGACTTTTCCTTGTGTGTTAGTTAATTGGAATCCTCGTGATGTA

GAGTGGCGCTTGAGCCGGTTATTCGCCGGTGGCCGTACACATTAGTTACC

TGGACTAAATGTCCTCTAACTAGCTTTTAGCTGCCCTTTCAACAAGAGAA

TCGGCTCATTTCCGCAGCGGGAGTGTTTCAAGTGTGCAGCCGCATCCAAG

AGTGTAGGTCAGCCTNNG

>TSBL.R43.esd 628 0 628 ESD GOOD: 93-509

GCACGAGGGCATGCTGGCAGAGCTCTGTGCCATGAATGCAATGCTCGAGA

CAAGGCGAATAGCTCTGACCGAATTCTCTGCTTCAAATGTCACTCCTACG

TTGATGAAAGCCAGCAACTTAAGTACCAGTCAGAAGTATATCATGCCTAT

CACTTCACTTGCTCATCGTGTGGCTCTGAACTCAGTGCTGATGCTCGGGA

AAAAGGATGGTGACCTTCTACTGCCTCCGATGCTTCGAACAAAATGGGGA

TACCGATTTGTGGTGCTTGTCGTCGGCCTATTGAGGAGCGCGTGGTTCAG

GCTCTGGGGAAGACCTGGCATGTGCAGCACTCCGTTTGTGCACCGCTGTG

AAAAGCCATTTTTGGGTTCCACGACACTATGAGAAAAAGGGATTGGCCTA

TTGCGAACTCCATTACC

>TSBK.R60.esd 732 0 732 ESD GOOD: 126-411

GCACGAGGGTGATATTGTGAACATTAATGGGAGATAAGCCTTCTGGCAGT

TATCCAGATAGAAAACTGATTTATACAGCACTAATATGAAGCAGCAATTG

GAAATTCTTGCCTTCTTCGCTCAGGATCGTAGCCTGGAGAACAGTTGATC

ATCCATTGGAGTAGTTTCTGAGCAATTTCTGCCCTATCATCAATCACAAG

CGCAGCCGAATATCTGACATCTTTTCCGATTAAAATTCCTATAAAATCGT

ATTTGATATTCGAAAAAAAAAAAAAAAAAAAAAAAA

>TSAL.R45.esd 714 0 714 ESD GOOD: 108-618

GCACGAGGCGACGAGGAATCTGTTAATCGGTTCAACCAAGCCGAAGCTTT

CATTGTGGATCGACTTGGAGTACCAGTCAAGTGGCTCCACGAAGCTAAGG

CCTCCCTGGCTCGGTCCCTGTTGGTCACACAATCGGCGTCTAGCAATGCG

GAAACCCATAGGCTACTAGCCAGCTTGGAGGCTGCACATTGGTTGGCGGC

GGGCCACTTGGAGGCCGCCCATGATGTATACGTGAAGTACCTTCTTCCAG

ATATAGTGCTTCACTCCGATGCTCTATCGGTTTCTTCAACTTCGGCTGTG

AAAATTGCTTCGAAGAAGTTCAGCAATTGCTAGCTTTCAAGCTGTTGACA

GCTTTACAAACCTTCATTGCCATCCCCCAAGAAAGTCTTCCTACCGCATT

TGAGACAGGTGCTGGTGTGTATCTGGCGTATGCTCGTATTCTGCACCTAG

TGGGTCAGCTTGCTCCTTCAAACAGAGTTGAAGGTGAGGAAATTGAGAGA

GACGTTAATGT

>TSBA.R20.esd 553 0 553 ESD GOOD: 39-552

CACCGCGGTGGCGGCGCTCTAAACTATGATCCCCGGGCTGCAGAATTCGG

CACGAGGACAGGTGGAAGTGATTGCTATGGCTCCTAAGTATGTCAGGCAA

AGCCGCTAAGAAGGCATCGAAAGTGAAGGTGCCGAAGACGGACAAGCGAG

AAGAGGAGGAGGAAGAGAGCTACGCCATCTACATCTACAAGTATTGCGTC

AGTGCACCCAGACACGGGCATCTCATCAAAGGCGATGTCGATCATGAACT

CCTTTGTGAATGACATCTTCGAACGGATTGCCGCCGAATCGAGCCGTTTG

GCGCATTATAACAAGAAATCGACAATCACGAGCAGAGAGATCCAGACTGC

TGTGCGTCTGCTGCTGCCCGGTGAGTTGGCAAAGCACGCGGTGTCGGAGG

GCACCAAGGCTGTGACCAAGTACACAGGCTCCAAGTAGATGGGATAGGTG

TGAAGTGCAGGAGTGTCACTGCCAGAGGTGGTGCTAATCCACCTCAATGC

GCTCTGCTGCACNN

>TSAM.R14.esd 616 0 616 ESD GOOD: 99-561

GCACGAGGGAAGGGCACCACTGCATTTGTGCGGAAGCAACAAAGGAAGGC

AAATAATTATCAACTTCTGGTGGAAGGAGGGATTTTCAGGAGAAGGAAGA

AACGAAATCAGACCATTTGTTATTGGGGACATATTTGAAGGTCCTTGAGA

ACTGTGTCCACAAGTCGAGCAAATTGCCAAATGTGGGCTCAACTGTGCAT

TGACACCAGCGTCAAGATTGAATCCGTTGTGAGCGCTGTCAGCATAACAA

CCTGACCGTGTTTTGAGAACTCACAGTGCCTTACCTTACTCTGATTTACT

AGAACAGGCAATTGTGGGGTAGAAAATTTGCTTTTTTGTCTGACCACATC

GGCAGTGTGTTTCCAGTGCTCTGGCTACCCTAATTATCGCGTAGATCCAA

GTCACAGAATGCGTCGTAGTAGTACCAAAGTTGGTAAACTTTCTGGCAAC

AAGTGGGCATTTT

>TSCC.R77.esd 630 0 630 ESD GOOD: 110-348

GCACGAGGATCCCACCCGTGATTGCATTGATTATGAGCTCACACAAGTAC

AATATTATAAACGCTCTTTTGGTTCTCAGTGCTCATCATATTCATTTTTC

ACGTTACTACTTTGTGAAATTAATGGCACTCTATCACCTTGGGTTAGTGT

GACAATCAATTGATGGTCTCTCTTTCATCGTATCTTTTGAAGTAGACCCC

ACGCAAAAAGTATTTGACCCTCCTCAAGCACATTAAGCA

>TSBG.R77.esd 699 0 699 ESD GOOD: 139-419

GCACGAGGCCCGCCTCCACCGCTAAGAAGGCTGAGTCGAAATCTGTCGCT

CAGCCAGCTTCAAAAAAGACTAAAGCGCTGGACTCCATCAACAGTCGTCT

TGCTCTAGTTTTCAAAAGTGGTAAGGCTGCCATAGGATACCGACATACAC

TTCGCACTCTTCGGACGGAAGGTGCAAAGCTCATTAATTATGGCAAGCAA

AATTCCTCCCCTAAAAAAACCGGGAGATAAAATATAATGCTATGTTGTCT

CAAAACTGTGCCGTAAATAATAACAAGGGCA

>TSBP.R31.esd 583 0 583 ESD GOOD: 130-267

TAGCATTTTTTGTGTATTTTGTGTGCGTTGGTTTCCCCATTTTTTGCATC

CTTGTTTGCCTTCCTGTTGTCAGTGTGGCTTTTATGTGCGTAGAATTATG

ATATTGCGGGCTGTGGACGTAAAACATTTGTCTCTTCT

>TSBM.R85.esd 497 0 497 ESD GOOD: 105-497

GCACGAGGAGTCGATGCATTTATGTTGAAACAATATCAAAGCCCATTATG

TTACTATTGCCTGGAGCGTTTTATAGGCCATCGCTCAATACTCTGGGGAT

GTCAAGCTTTTTCTAAAAGTTCTGCGTTGATTATAGACGTCGATCAAGGG

GTATCGACTTTCAATACCCACGAGGTCGAATGCAGCGGTTCGCAGAGAAC

CCTTTCCTTATAATAGGTCAGGCTTTTCCGTGATGAGCCTCACGGTATGA

ATATTGTAGCGTCGTATTTTACAGGTTATGTGAGAGACAATCGCGATAAT

TTATGTAAGGACGTTGGTGGAGGTGACCTGCTCCCATGAGCAGTTAGGGA

GTTGCATGTACTATCTTTCCACTTCTTTTTTAAGCTGCTGNNA

>TSAH.R46.esd 602 0 602 ESD GOOD: 98-584

GCACGAGGGTTGGTGCTGGGTTGGCTTGGTACCTGCTGTTAATTTCTGAA

ACCCTCTTTTAGGATAGGCTCGTTGGGTCACAACGATGGGTGATTCAGGA

AGTTGGTGCTTGATTGAGAGCGATCCGGCAGTTTTCAACGAGCTTATGCG

TGGATTTGGTACGTAGCACTATATATCTGCATGTATTTTCTGAAGGCGTT

GATGGCCTTGACTGCATCGAAGTTTATGATACCCGCAGTACCGATTCATT

TAACGACGCTCTTGGTCTAATTTTTTTATTCAAATGGGATGGAAACGGGC

GGGACGCCTTGCAATCACTAAAATATGTTGAGTCGGGTTCGATTTTCTTC

GCAAAACAAGTCATTACAAACGCCTGTGCTACCCAGGCTCTCATCAACGT

TCTTTTCAATTTGCCGCCTGCCTCACTGAAGCTCGGTGATACACTCACCG

ATTTCAAATCATTCGTTTCCGACTTTGACAGTCAGAT

>TSCF.R41.esd 527 0 527 ESD GOOD: 100-504

GCACGAGGGAATGTAGAGCTCAATTTCTTCTCTCTCACCATATACTCGCT

CTCTGCTCACGTGAGCTAAATCGATGAATGTAGAGCTCAATTTCTTCTCT

CTCACCATATACTCGCTCTCTGCTCACGTGAGCTAAATCCATGAATGTAG

AGCTCAATTTCTTCTCTCTCACCATATACTCGGCTCTCTGCTCACGTGAG

CTATATCCATGAATGTAGAGCTCAATTTCTTCTCTCTCACCATATACTCG

CTCTCTGCTCACGTGAGCTATATCCATGAATGTAGAGCTCACTTTCTTCT

CTCTCACCATATACTCGCTCTCTGCTCACGTGAGCTAAATCGATGAATGT

AGAGCTCAATTTCTTCNCTCTCACCATATACTCGCTCTCTGCTCACGTGA

GCTAA

>TSAS.R44.esd 640 0 640 ESD GOOD: 104-622

GCACGAGGCCCACTCGCGCATCTCGCCTTCAAAATCAAGTCCCTCCCATA

CCCAACATGGATGTTGGAGATGAAGAAATGCCCTCCTGCCACCGCCAACC

TCCCGTCCGACATCACCACGTTCCCGTTTCCTCCAGAGTGGTATGGCATA

ACGGCCTGCCGCTTCCAGTTGTGGACAGTCCAGAACAGGAGAACTGCCCA

CCTCCTGACGTTCCGATGCCTGAGGCTCCACGAACCTCTCGCCTGGAGGC

TGGTCAAGCCTGCAAGGTAGCAACGAGTGGTGAAGTTTTACGCACTCTGC

CTTGCCATACCTCTTCCCACGTCTCCAAACGCACTATCGCTGAAGTGGGG

CTCAGCGACGATGACCACGCCAGTTTCAACGTTGGCGGTGGCAGTCATGG

CAACGCTGGGGGCTGTGGTGCTACCTGTTCCGTGATGAATGAGGGCAGTG

GTGATGACAACGGTAACGAGGAAGATGACGACGAATTGTGCACAATCCGC

GGGCACCACAATCGCCGCG

>TSAD.R4.esd 712 0 712 ESD GOOD: 94-506

GCACGAGGGTTGCATTACAGAGCCCCTCAAAGGAGTCATTAATCACATGA

TTCATCGTGGGATGCCAGAATACACTCCAAAGCATTGTGATCGCTATAAG

ATTGGCTTGATTTTCTGCATCCCTCTTGTTCTTGAGAAGTGAGAATAGAC

ACTGCCACAATCAACTAGGAGCTTATCACAGTCTTTATCTCTTCTGCCTG

CCGTTTCACCTAGGGCCGTCGGGTAAGCAAAGATTTAATTAACGGCTATG

CGAAATTGGACAATGCCTTTGTCCTGCCACTGATGCTCTTTTTTCCATTT

TACCTCAGAATAACATTGCTTGACGCCACTTATTGGCGGCAAACATGAGA

ACTGGTGTAAGTTTTGCCAGTTATGTATCCTTACAGGTTCCGCTTTGAAG

CTAGTGCAGTCCT

>TSAG.R88.esd 640 0 640 ESD GOOD: 97-481

GCACGAGGGATCTACCAATGTCAGAAACCAAAAAGCTAGTCGATCTATCG

AAGCTCCCTGAGAGAATACTGTCAATTCTGGTCTCGCATTCCGATCCAGT

AACTCAGAAGACCTTTGAGGAGGAGCTTCCCGATGTAACTCTTACTCAAC

TTCTCCCAGCTCTGAACCGGATGCAAAAGGAGGGCCAGGTGGAGGTCTTA

GTCAANCCCAATCGTACCCTCTCCTGGCGCATGCGTGAGATCGGCAACAT

CGATTAATTAAAATCCCTTACAGATTTGGAAGAGAGTCTGGTGTACAACT

GTATCCGCAAAAACGGAAACGAGGGTGCGACGGTCAAGGCTATTTCAATA

GATACCACATTGGGCCAGAATCGGCTCCCTCGCAT

>TSAA.R90.esd 704 0 704 ESD GOOD: 103-649

GCACGAGGCGATCAAAGTGAGTCCTTTCCTTACTATGATATCTCCTATAT

GTTTTATGATTTATTAACTCGTCAATTCAGTTTAACCGGTGGGAACCTTT

CGTTATACATATTTAATTGTCTACAATACTTGTTTTTTAACTATAAATAG

ACTTGTGACTTGAATAGCTGTGTTATGAACCCTTGAATGAACCAGATTAA

ATTGTTCCGATTATACACTCTTGTAAACGCAAATTTTCTGTTATAAAAGT

TGGTACCATTTATTCTCTTGCTGATAAACTTATTTCCGAAATTTGTTGCA

ACTTATTGTCTTTAACTTGCTCAAAAACTCCTTTGAGGAAAAGTTTCGCC

AATTACCGTGGAACGATAAGAGCATATAATGAACAAAGAAAAAATGCCCC

TTTTCACGAAATCGGTTTTTCAAATGAATCATTTGGAATTTTTCACAATT

TTTTCGAACAATTATGTGTTGCACTTTGGTTGTGAAATTAATGAGGAAAT

GCAGTCCTCCTATGAACGAGAATGGAGCAGCGTTTCATGCAAAACCT

>TSAC.R13.esd 740 0 740 ESD GOOD: 97-620

GCACGAGGCTTTCGCTGGGTGTTGGCAGATTTGTATGTCAGTTAAAAAGA

ATTACTTTTAAATTCTGCAAATCTCGAGGCGACAGTCGAGGTGTTCGTGA

TTTTATTGAAAGTGAAATCGTCAATTTCGCTCGAGCAAATCCTTCTGTGG

TGTTTTACGTCAAGCCTCGGAGACACAGAGCACCTCTTCTCGTCGCCGAA

TATCTGAATGGAAACTGGCAGTATTTACGAATGGCGAAAATGTCCTCACA

GGAGATTGGTGCATGGCTCCAGTTCATGATCACCCGTTCGGGTGAAAACA

TTTTCCCCATTTACAAACCACGAAGCAGTTACTTGCCATCGATACAGGGC

ATGTGGTCCCCTTATGACACCCCCGATCTACCTGATCCTCTGATGACACC

ATCAGAGATTATTGATAACATACAGGACCTTTCCGCTTGTAAATTCCCAT

GGGAGCAGTCTGCGCAGGATTATCTTCAACAAATCCACGAGCAGAGGCAA

AGACAACGAAAGGACTCACCTCAC

>TSCD.R94.esd 603 0 603 ESD GOOD: 89-532

GCACGAGGATCCGTGAAGCATTTGAAGACGGAGGAGGACGGTAAAAAAAA

AAAAGAAAAGGCTAATGTGAAAATGCGGTCAGACGGGATTGACGCGCATG

GATCTCGGAAGGCCGACTCCTCGGAAAACGTGACTGAGTTTATATCTAAG

AAGCAAGACCTAGATCAGAAGTGCGCCTCCTCTGGTGGTTTCCTTGGATC

GAAGAATATCTCGAATGCGAGTGCAGATGAAGTCGGCTCCCCAAGAGAGC

GAAAAAAGTGACTATGAAGTGACCTCTCTATTCTCTTTTCCTTTGTTCTT

CTTACCCCTGCCTGCCTTGCTTCCAGTTCATTCCTTTCGATTTTTCACTC

CATCAACTACCAAGTCGATACCTCTTTTTTTACTTATTTTCACATTTTTG

TGAAATCCGCTTTTACGTAGAACAAAAAAAAAAAAAAAAAAAAA

>TSAP.R24.esd 579 0 579 ESD GOOD: 95-370

GGCACGAGGGTCAAAGGCCCGAATTAGCGTCTCAACCTTTCGGTCTGCAT

ACCGCATTCCCTCGAAGTGAGCTCACGGACAACTCGGCCACGATGGTGAG

CGCCAACCTACTCAACTCAACGCTCCTTGTTACTAAGACCTGAAGATTAC

CGATCTTGCTTCACTTCCCTCCCATACGTTCATTTTCATTTACGGACTTC

CCCTTTGACATTGTCACCAGACCCATGTCTGCAATTTAAATACGTTTAGG

CAAAAAAAAAAAAAAAAAAAAAAAAA

>TSAF.R29.esd 659 0 659 ESD GOOD: 98-470

GCACGAGGCTCAGGCGGCTCTGATGGAACAGAAGAGCGCGTTGCCCTTTA

CGAGCGCGCAGCCAACTGCTTTAAAATGGCCCACAAGTGGCAGGAGGCCG

GAGATGCCTTTGTTCAAGCCGCTGAGCTGAGCGCCAGCAACAAATCCCAG

TTGGACGCCGCTACGCACTACGTTAACGCTTCCGTGGCCTACCGCAAAAC

TGATCCCAATCGCGCCATCACCTGTCTCACTCGTGTGCCGCAGACATTAT

TTATCGAAATGGGTCGTTTCACCATTGCCGCCAAGCACCACATGACCATT

GCCGAAAATCTACGGAACAGGATCTAGCCAACGAGGCCGAGGCCTGCAAG

CATTACGACGAGCTGCTGATTTC

>TSBA.R43.esd 644 0 644 ESD GOOD: 108-610

GCACGAGGATTGCTATAGCTCTTGTAATTTAACAAGACACATACGAACAT

CTGAAGTGCTTTGCTCTTACTTCCGCTTTATCGAGGTAAAATAATCTGCA

TTCAATCAGATAGGTAGTAAAGCGGAATAACTCTCTAATGGCACGACGAG

AAGCTTAGGCAGACAGCAAAGTCTCTGCTTTTATTGCGGCACATTTTGCG

CCCACCTCCTTCCACACCCTGTCTCTCCACACAAGCTAACTGGCATTGGA

AGTTACTTTCCACTTTGCATCCACAAGCGCAACTGCATCATCAAAAAAGC

AATAAATACACCACAAGTTGTTATGCACTTGTACATTTGGGGATGTCGAT

TTACCGTAAAGTTGTTTAAAGATTCTCAGCGCTACATTGTCCCTCGCACC

GGCAATACTGACTTCGACAAGACGACTTTGCAGAAGGCGACTCCCCCGTG

TGTAAGTGGATTTCACACTGACTCAACAGCCTTGGAGCTTGCAGCAATAG

CAG

>TSAL.R53.esd 450 0 450 ESD GOOD: 104-251

CGGCACGAGGGTTGAGTTTCGCGTTCCTAGGTAGATTATTTTCCAGTGGA

CTAGAGTTATGATACGTCGCTTCCCAAGGTGTCGTAGTTAAGTGCCTCTA

TGTTATCAGATGTGATGACATAGTTCGAGCAAATAAAACAAAGACTCG

>TSAZ.R60.esd 710 0 710 ESD GOOD: 115-615

GCACGAGGAGAGATCTCTATGACCTCTTTTCCCGCTACGGCGAGGTGGAG

GATGTGACCATCGTTTATGACAACTTCACGTGCCACTCAAGAGGATTCGG

TTTCGTGTACATGAGACATCTTTCTGACGCTAAGGAGGCAAAACACGACG

CCCACGGTACCGAGCTCGACGGGCGTCCTATTCGGGTAGATTATAGCGTT

ACTGAAAGACCCCATTCTCCGACACCTGGTGTTTATATGGGGAGACCAAC

AAGGCGCTGCGGTGCTCGTCGGCGCACACCTTCACCAAGGCGTCGCTACT

CACGCTCACGCTCTCGAAGCTATGATTAAGAAAACTTTTGCATTTATTGT

TTTACACCTACCACACAACTGAATTTGTGCCGTATAGGCGAGTTCTGCAC

ATCGCTATACAGGTAACCACCAACACAGTGCTCCCACTCTGCCGTTATGA

AAATCCGAAAAATAAATCCTTTGTTTTTGTCAAAAAAAAAAAGAAAAACA

A

>TSCD.R43.esd 654 0 654 ESD GOOD: 100-389

GCACGAGGCTGACTTCATCCTTCCCTCCTCCTCCCATTTCTGTCTCGCTT

CTGCATTTATCGTAGCCTCTGACGCCTCACTGTTTATCCTATCATGACAC

TTTTTATACACAACAGAATTTGTTGAGATTATCATAAAAAAAAAAAAAAA

AAAAAAAAAAAAAAAAAAAAAAAAAAAAAAAAAAAAAAAAAAAAAAAAAA

AAAAAAAAAAAAAAAAAAAAAAAAAAAAAAAAAAAAAAAAAAAAAAAAAA

AAAAAAAAAAAAAAAAAAAAAAAAAAAAAAAAAAAAAAAA

>TSBK.R96.esd 815 0 815 ESD GOOD: 93-587

CGGCACGCGGGTGAGCACAAAGATTTGTTAGCATGACCATAGGCACCCTA

GAACGGCCTAAATGACCTGATTGTAGGTGCGAAAATGTCAACCTCTAAGG

GACTCTTTTGCAGAATCAAAGTATGTGAGTGATCACTCACAGCGAGGTCC

TGAGACATAGAGCCGAATTGCTGCAGTGAGCAGTCCGAAGTCGCATGTAT

TTCTCTCCAAAAGTGAATGGCCTTAGTGACCAAGCTTGACTGTTCACTCG

ATGGATGAACGCGACATTTATGGTCACTGCTCAGTGCTGTGCCATGGAGC

AAAGGATCCAGAGTGCGCTCACTATCAGTTTACTTGTAGATAAGCCTTGT

TCGGGCATTTTTGTCGGTGTTTCTACTGCTTTTATGACTACTAGTTGCGG

GTTCTCGATACCATCTACATTAGCACAAGGACATGATTCGGAAGGCATGG

ATTTGAAGCAAAGAGCGCCGAGCTAGTCAGAATGCTTCACACATT

>TSAB.R17.esd 628 0 628 ESD GOOD: 103-498

GCACGAGGACGTCTTTAATGAACACATGGCTGCCGTCAAGGCCATTGCCT

GGTGCCCTTGGAAACCGACTCTCCTTGCAACTGGTGGTGGCACTAGGGAT

CACCACCTTCCCGGTTTTGGGAATGTCTATTCTGGGGCTTGTGTGCGTAC

TGTAGATGTGGAAACACAGGTGTCTGGGATTATTTGGAATGGGGAGTTTC

GTGAACTGATAACTGGCCACGGGAGTGGTAGCCTTTGTATTTGGAAATAT

CCACACATACAAAAGGTTAAAGAACTCACTGAACATCAGGACAGAATTCT

CTCAATTGTTGCCTCACCGGATAAGGAAATGGTGGCAAGCTGCTCAGGCG

ACGAGAACAATTCGCATTTGGCATTGCTTTAAAGTGGACAAAGCAA

>TSBB.R28.esd 452 0 452 ESD GOOD: 96-447

GCACGAGTTGCCTGCTATGTCGATGCCGAAAACGAAGGCTCATTTTCCCC

AAATCCGAGCCCCTACCACCACCTTCTTACGGAGAACCGTTCGACTGGAG

CGAATTTTTCAGTTCTATATGGCAATGCTGTCGTGGATGCAAGCCATACT

TCGATAACGATCCTCCGATAATCGTTGCATTCGGACCACGGATTCCAAGG

ATTGATCAAGTTTGGTCACATTTTCACGACAATGGAAGCTCCTCATTGCG

AAATGGTCACTCTGTGAAGGCGGTCACAGCGGAATTAAATGGCAAAGCTT

CGCATACGGTCGTGTACCCGGTGGCAGCCAACGAGCCACACGGTTTTTCA

CC

>TSCB.R88.esd 622 0 622 ESD GOOD: 103-547

GCACGAGGGTCACATTGAACTCCTGTTGTCAAGTTCGTCCTTGACCTCGC

CTACGATCTCCCCACAACTTCCGGATCCGTCCATCCTGCTTTCTGACCAA

CTAATGATTCCTATGGCTGGCTTCAATTACACCATGCCCAGGTTCCAACC

CTGCCAGACCATACGTAACGAAAGCAGTACCTCTCTCGTCTTCGAAAATC

ACCTGTATTATAACTCCGCGAACGCTGTCCGCACGCACTCGATCGTGAAA

AACCCGAATTTCGTTCCAACCTTACCAGTCGTCGGAGTGAGCTAGTAGGC

TTACATTGCCACTACTACGCTTCATTTCGTTATCAAACGCTGCTGAACTG

TGAGAAGGGATAAGAATCTCGCATTCTTATTCTGTCTTAGTTTTCCATTG

AAGTGTTTGTTTTAAAAAAAAAAAAAAAAAAAAAAAAAAAAAAAA

>TSCC.R9.esd 785 0 785 ESD GOOD: 102-632

GCACGAGGGTTGCTACCATTCAGTAGAAATTTCTCCAGAGCCACCGAAGT

CATTAGACAGCACTGCTGGACAAAGCAAGAACAGATGGATAGATGGGAGC

AAAGGAGAGGATTTTGTGTCAATTTTTGGCGCACACTTTGATGTTGCCTG

TGCCAGAAGGGGAGGAGGTGAGAAAAGGCTGGGTGAGCTTTTGCTTGAGG

CGAGAAGAAAACATTTCTACTCACAAGTGGGAAAAACCGAGAGGAGCAAG

CACACCCAGACACATGAGGCAAACACGCCTGTGGGTATGGTTGAATGTGC

CACTGGGAATAACGCGCCTCCACTAAGGCAACCACCCCGTTTTCGGGATG

AGTCAAACCCACGCAAACGGAGATTAGACTCGACGGTTGGAAAATGAATG

GCGACTTCTATGCACCCTTCCCCACACAATCTCATCACCTGCATTACTAG

AACTAGAAATTTGGCCTTTGAGGACCTTAACTCCACCCATGACTAAAAAT

TGCTTGTGATTCCGAAGTCATGGTACTAAAT

>TSAT.R7.esd 689 0 689 ESD GOOD: 105-592

GCACGAGGGCCTTATCGAAGGCCAACTTCTATCTACGGGTGCATTTGAGA

TTACTTTCAACGACATGCCAATCTGGTCGAAGTTGCAGTCCAATAGGGTG

CCCCAGCCACAGGAGCTTCTATCAATTATCAACGCACACCTGCAGTTTCA

GACCCCGGGCTCTGCTTCCCATAGAATGTCTTTCAATTCGCACAACAATC

CCCAAATCCCAAGGTCCTAGTCTGTCTTTCCCGCTATCTCGCCTCCTTTC

CTTTCTCTGATTGACGAAGCTGGGTACATCCGTGTGTGATCTCGGGAATC

CAATGACACTGACGGAGTAAAACCGTGACCTCAGATGAGCGGAGCTCTGT

TTCAGTCGATGCCACCGTCCTCACCTCACATGCCGCTGTCTCAGTTCTCC

TCAAGTCTGTTGTTAATGTTGTTAATCTTTTCATGCATTCACTCCTTTCT

ATGTTTTCATAAACTCTTCTTTGAAAAAAAAAAAAAAA

>TSBC.R58.esd 587 0 587 ESD GOOD: 70-520

GGCTGCAGGAATTCGGCACGAGGCAGAGTTTCAATATGGGTCAGGCCAGT

TTTGCCCTCCAAAGTGTAAAGGACACTCAGACGACTGTTAAGGCTATGAA

ACAAGGAGTTAAGGAATTTAAGAAGGAAACCAAAAACTTGAAGTTGGATG

ATGTTGAGGATCTGCAAGACGATCTAGCTGATATGCTTGAACTTAATGAC

GAATTTGGGGAAGCGCTTGGTCGTTCGGTCATACCAACTACTATCGACGA

ATCAGAGCTGGATGCTGAGCTGGAAGCCCTCGGTTCTGAAGCCGATCTGG

AAGGTTTTCTTGATGCAGATACTGCTCCCTCCGTCCCTAGTGCAGACATT

GGTTCCTCTGTCTCCGTTCCAAACGCCCCGAACGCTGCGGCTAGCGGCAA

CGTTCCGTTGGATGAATTTGGACTTCCCCAAATCTAGAACTCGGTTTTGA

G

>TSBQ.R56.esd 808 0 808 ESD GOOD: 95-620

CGGCACGAGGCCGTGTATCTGCATTACCGTATGCCAGACATTGGAATGGG

TTCGGCACTGCAGGCCTTTGTCCAATCTGCCGCTGTGACAGCCGCCTTCT

ACGTTGGTATGACGCGAATTTCTGACTACAAACACCACTGGACTGACGTT

CTTGGTGGCATGATAATTGGCGCCTTGGTTGGTATTTTTACCGTTCGTTA

CGCTTCAGACGTGCGTGAATTTGATGTCGCAGAAGGGGAAGAGGTGAGAT

AGCGAACATCGCCTCATCCTCCTAATAGCAAGCTCCGCCTAAGCACCTCG

TTATATTCGGTGTGGAATTCATCCCTCGCCCTCGCTTTTGCGCCTTTGAC

CCCATCTCCTCCAATCCCCTCCCCCACCTACTCCTCCGTACCCTACACAC

CACTACACTCGCCTTTGTTTAATTTCCTTACCTCACACTTCTCATTCAAC

TGGTTTTAAAATCTATGAATTTGCGCCACGAAAATAAGATGTGCATGCTC

ACTTGTGTGGGAAAAAAAAAAAAAAA

>TSCD.R92.esd 682 0 682 ESD GOOD: 94-562

GCACGAGGTAAACCAATTAATAAAAGCACAACTCACACGCAGGCACCAAG

GATTAGTGAGTTTAACTATAAGCTTCCTACTCTTTTCCACTTTGAAAAGT

GATTCTAATTGCATCAGACAAAGTGGTTTCCACCATTTTGAGGATTCACT

TTACCATATTGATGGCGCAAAGCCACTTGCCAACTTAGAGGGCTCGATCC

ACGGTCAAAAGATCAGTTTCCACATCAGAACTATAAAGTGTTTTTTCATT

GCTTTAGACTATTCGGCTGCAGAAGCAGAAGATGTCGGAGCTGAAAAGAG

CTCTCGGTCAGGGATTGAGACCAGTGGCGGTGTCAAATCTCTCCCTCATT

TCCACTGATGAGGAGGTCCACAATCACCACCGCCTCTCCGTCAGGTTCGT

CCGTTCCACTCTTTTCTGTTCGTCCTCAACANCTTCCCTAAATTGTGACG

CCCATTTTTACTGGAGAGG

>TSAW.R24.esd 609 0 609 ESD GOOD: 96-590

GCACGAGGGTCAATCAGCACANGTCAGTCCAAGTTATCAGCATCATTTGC

ATGTGGAATCTGCGGTAAGGAGTTCGGAATGCGATGTCGCTTGGTTGCCC

ATATTCGAAGACACACTGGCGAACGACCCTTCTCTTGCACTGAGTGTGGA

CGCGCGTTTTCTGATGCTGGCAATCTCCAAAGGCATCGCTACGTTCACAG

CTCAGAGCCTCGGTTCCATTGTACTGTCTGTGGAAAGTCCTTCAGACAGG

CTTCTTGTTTGTCCACGCATCGACGATTCCACTGTCCTGGAGCTGAGGGC

CGAATATGCGTTTTCTGTCGGCGTCATTTCAAATCATCCGCATCCCTGCA

AATGCATTTGCGATTTAAACACCGTGCCGACGCTGAAGCTGTCGTTGCTG

TTGCAGTGGGATCTATAAACTCGGACACCGTAAACTCCTCTGGAGAAAGA

GCTAAAGACGCTCAGCAACAAGAAGCGTTGTCGCTATCAGCGTCC

>TSBJ.R94.esd 582 0 582 ESD GOOD: 90-551

GCACGAGGGTTTGGGAGACCAGACACAAGAAATGTATGAAATCAAACGCA

ATCTATACCTTTATAGTGTTTTGCTGCATTTAAATGCACAACAACTTTCT

CCACAAATTTTATTAAGTACTTGGTATGTTAAAACGCACTCCACATTGAT

GCCTACACAATAATACCCCCAAATATTGCCACAAATAGATGAAACTATGT

CCCACTCAGGCTTTATATTCTTACAATGCGAAATCAGTGGGAGGTTATGA

GTCCCATAAAAAATCTATTCTTGAGTGAATGGCTTATAGAATGCCCGATG

TCTCCTATGAGTGCAGATGGGTAACATTCAAAGAAACATATTTTGTCACA

AGTATAAAGTTGCGCTGACGACACGTAGAAGTTGAGGAAATTATTCTCAT

GGCGAAGTATAACGGGGTATACTCACGTGCTTAAATTCTACTGCACTTGA

CGTCCAGAGATT

>TSBJ.R54.esd 580 0 580 ESD GOOD: 76-278

TAGTCCCGGTGATGAATTCGGCAGAGCTTCTAGCACTCGAAAACAGGTCG

CAGTATGATGCTGCGGCGTTGAGGAAATTTTATAAGCCCATTGGACTCTC

AATAAGAAGGATCATTTTTAATGCCATTTAATGTTTATTGTGTGCGCTTT

TTTAGCTTAAATTAAACTAAAGAATTTACACCCTTAAAAAAAAAAAAAAA

AAA

>TSAA.R56.esd 756 0 756 ESD GOOD: 110-598

GCACGAGGAGGTGTCAAGAGCTGTCTGAAATGTAATTATGAAGTTGTTAA

AATGACTAAAGTTATTTTCTACTCTCCACCCTATAAATCTTTAAATAAAT

GCCAGCAAAATTTATGGTTTTTTATTACAGGCAGCTGCAATATGCTGCGG

CCTTGATGGCTACGAGGGTTTCAGTATTTTACGGAAAGGCAAAATGCCGT

CTAAAGAATGTTGCAATAATACGTCCGATACTTGTAAAGCTTTGTAGGCT

AAAGTGGCTAATGTGACTAGGTGCAGGAAAAAACCGCAAGATTACTATTG

ACAGTACGGAACATATAATGTCTGTCCCCATTGGCTCGATCCTGTTGCAG

CTGAGTCATGATTCCATCTTTTGTACTGCCAGCTAATGGTGTTAATGAAA

GTCATTTCCTTTTATGTTATATCAGTCGTAGTTGCGATGGTTATAATTTG

CTTGTACACAATTGCCGGAGGCAAGAGAAGATGGAGACA

>TSAT.R9.esd 648 0 648 ESD GOOD: 105-648

GCACGAGGGTGCATTCGAGTTCTGATGGGCGATTGTGGTGATGAGTGTGT

TATTGACAAGCTTAGAGAGCCCCTTACTGTGTGGCCCAGAGAAGTTGCAA

ACTTCATTGTCCCAAGCGCACCGAAGACATTTTTCTATATTCCTGACTTC

CTTGATTCTGACGCTGAAAGTGAGCTCTTGAATAACATCTATTCTGCTGG

GAAATGGAAGTGTCTTTCTCACCGACGCCTTCAGATTTGGGGTGGAACTC

CTCACCCAAATGGTATGATTGCGGAGGAAATTCCAGGATGGCTTCACAGG

CTAATGGATCGAGTTTCAGACTTTGGTATTTTTGGTCACAATCGGGCCAA

TCATGTACTAATTAACGAATACGAACCGGGACAAGGAATAATGCCCCATC

ATGATGGTTCTCTTTATTATCCAGTCGTCGCAACTGTTAATTTGGGCGGA

CATAGTGTTTTAGACTTTTACAAGCCCATTACGGCAGAAACGTCGAATGC

ATCATTGTTCGCGCGTTATGTTGGGTCGGCACTGCTGATGCNCC

>TSCC.R7.esd 699 0 699 ESD GOOD: 103-557

GCACGAGGCACAGATGGTGCAGTATTACCCGAGAATCTCCCCGTGAGCTG

CCCTGGTCCGCAAAGCGAATCTGCTGGCCGTGACGCCTTTTGCGCTGGAT

GTCCGAATCAAGCTCTGTGTGCAAGTGGCGCTGCTAGATTATCATTAGAA

GAGCGCGAACCCGAGGTTGTAGGTAAGCTTCGCAACCGTCTCGGTCGGGT

CAGACACTGCCTCTTTATTCTCTCTGGTAAGGGCGGTGTGGGCAAGAGCT

CTGTGTCAGCCTGTCTGGCTTGGGCTTTAGCACGTCAAAACCACTGTCGT

GATCAAATTGGGCTACTGGATTTGGATATTTGTGGGCCTTCAATACCCTG

CTTAATGGGATGTTTGGATTCTGAGGTTCACCAAAGTGCCTCAGGTTGGT

CGCCAGTTTTCGTGACTGACAACCTAGCGCTGATGTCTGTGGGATTTTTG

ACATC

>TSAO.R47.esd 712 0 712 ESD GOOD: 103-629

GCACGAGGGGGACACCCAGTGAACCGTCGGAATTTCTGGATCTCTTGTTC

AATATATCATCATATAAGGAAAGTATGGAAACCACAGGTCCAATGGTCGT

TCATTGTAGCTCCGGAATCGGTCGGACTGGAACCCTAATTGTCACGGACA

TGCTCCTGGGCTGCATTCGCGAAGGCGGTCTTCATACTGACATCGATATA

GCTCGCACTGTCCAAGCAGTGAGGGAGCAACGCTCTGGTATGGTTCAAAC

CGAGGCTCAATATCGATTCATTTACAAGGCCGTCCAGGAGTTCGTCTCCA

GTCTTCTACTTCGTGTGAAACTTCGAAATGCCCTGAAACCCTTTGGAATT

GACTATACCAATTTGAAACAGGCGCCACGAAGCGAGGGATGCCTGTGTTG

CACGGGGACAGCCACCTTTCCCCACTCCCCATCATCCACTCTCGCTTACT

CATTGACCAACTCTTCCCGTGGATATTGGACTAGCAGAAGCGTAAGCTGT

CGAAAACCCATGCCAACTCCGTTAACC

>TSAJ.R89.esd 676 0 676 ESD GOOD: 96-241

GCACGAGGGCTATGCTGAAAAACTTCTACATGTGTCTCAGGACCTTGAAC

GTGAATACAACATTCAGACAAAGTCTTTCGTTGCTGATTTCACTAAAGTA

CGGTGTGTGTGTATATCTTGTGTTTTCTTTGTTAAATTTTCATCTT

>TSBQ.R51.esd 798 0 798 ESD GOOD: 99-278

GCACGAGGGGTTCAATAGGAAGAAATTGACCCTTAAGGAGCGCCGCGAAC

GCGTGCGCCAAAAGAAGGCGCACTACCTTTCTCAACTTCAGAAGGATTTG

GCTGTTGTTGGGGCTTAAAGTATATTAGTGTTTAGCCAAAAAAAAAAAAA

AAAAAAAAAAAAAAAAAAAAAAAAAAAAAA

>TSAB.R80.esd 722 0 722 ESD GOOD: 96-583

CCGATGTCGTGCTCACCTTCCGGGACATAACCCAAGATAAGCAAACCCTC

TACTGCCACGAATTCCTCAAGGCCATGTTCACCCTCCCCGGATCTCTTCC

ACGATCTCTAATCCGCAAACTTCGTGGCAAATGCCTAGAAAAAGTCAAGG

TCACATTAAGTGAATTAGAGGCACAAGAACGTGACTATCGTGAGCGAATG

GCGCTGCAACTTCAACAACAGCAAAAACAAGCTCAACAGAAGGACGAGGA

GCAACCGACTAGCGACTCTCACAGCGAAGAATTTGACGACAGTGATGATG

ATGAAACTGATGAGATGTTGAAGCCTACACGTTGCGTAGACGACGCGGAG

TCCTCGCCATCCTTGAATGCTCCAGCAGACCATGGCAACGGCAAAGGCAA

CTTATGAGATAATTTTCCTTTAACCATTTGCTTTTCTTCGTTGTTTTCCA

TTGCAGTGTAAAAATCCCTTCCTACTTGAAAAAAAAAA

>TSBF.R44.esd 650 0 650 ESD GOOD: 94-598

GCACGAGGCTGCTCCGTCATCCGCGTGATTGCTCACACCCAGATGAGGTT

GATGAAGCACCGCCAGAAGAAGGCCCACATCATGGAAATTCAGGTTAATG

GCGGCACCGTCGCTCAGAAGGTTGACTGGGCTCGTGAACACCTTGGAAAA

ACAGGTTCCTGTTGCAAATGTTTTTGCTCAGGATGAGATGATCGACGTCA

TCGGTGTGACGAAGGGTAAAGGCTTCAAAGGCGTCACATCTCGATGGCAC

ACCAAGAAACTGCCTCGCAAGACTCACAAGGGTCTTCGTAAAGTTGCCTG

CATTGGGGCTTGGCATCCAGCTCGTGTGGGTCGTACTGTTGCCCGTGCTG

GTCAGAAAGGTTACTTCCACCGCACCGAAATGAACAAGAAGATCTATCGT

ATCGGTCTTGGTCTTCAGGCTCAAATCGAGGCCGCTAAGGTAGAGGCTGC

TAAAGAGGAAAATAAGGAACTTGCTGCCCTTATTAAGCCTAAGGGCAATG

CTTCC

>TSBW.R29.esd 507 0 507 ESD GOOD: 99-364

GCACGAGGACCCAAAAACATGATTTGGTGTCATTTCTTGTCTTTAATCAA

GAGGGTGCATTGTTAACGTTGTTCACCCTTGAGTGGTCGGGTCAAAAGAT

TGGTCGAACTTTCCTCCATTTTGTTAGCACTCATCATTCTGATGGTGGCG

CACTGTTTTGGAATCCTAAACTGTTTAAAATACGGCGGGATTAGGTCTCG

TTTTTCTGCACTGTAACGCTCCCGACACCCAGTTGACCGGTAGTTAACAG

CCTTAGTGAGATTTGA

>TSAA.R66.esd 609 0 609 ESD GOOD: 110-521

GCACGAGGGTTGTATGGCATCGAGGTGTCTCTTACGTTGCCTGGCTACGT

CTTGCCACCTAGTGCAACCATTCCACCTCCCGCTGACCTACGCCGCCCAG

CGATGCGTCCAGACCTCCAGCATGTGGCTCGGCTTCTGGTCCAGACTGAG

GTCGTCCTTCAATGCGCTGGAGGTGGAGGTGGAAGGTCGATTGGTTGGTA

CCGATGCGATGGGAAACCGGTACTTTGAAGTGGAGCCAGATAGGAATAGT

GAGACTCCACATCGCGCATCCAGACCGAAGCGCTTCTTCCTTCTTCCCGG

GCAGAGGTCTGTCGAGGACTCATGGATTGCATTTGAATACCGAACTTCCG

CGTCTTCCGAGTGAGTGGATGCGTGTTGGGTCATCGACGTGCAGATCCAC

CTAACAGAGGAG

>TSAW.R4.esd 619 0 619 ESD GOOD: 88-237

GCACGAGGCTTCAATTTCCTTTTTTCATATCCCAATTAAACATTAGGTGG

TTAGGCAATTTGGAGCAGGCATTGGGTATTAAAACCTTCTTCCTTCCGAA

TTCCGACGTGGCACAAGGGCGTATTCCGCAATTAACCCATGTACCATTGA

>TSAG.R78.esd 845 0 845 ESD GOOD: 103-307

GCACGAGGCCCCTCTTCATGTAATTCCATCTAACCTCTTCCAAATGAACC

GCTTCGGTGATGACCACAGATGTGGGGATGGGGATTATGACGACGATGAT

GTTAACTAATTGCCTTTGCCCTCATACCTTTTTCTGATTCCGCATTCAAC

CACGCACGTACAACCTTAGACTCTGTTCCACACTTTAATGCAAATTTGCT

AAGCT

>TSBC.R74.esd 480 0 480 ESD GOOD: 91-477

GCACGAGGCACACTTTGAGACGACCTGCGATGACGTTCTCGACATCTACG

AGCGAATCATGGGTGAGAAATTTGACTTCAGCAAGGTGAAGGCAAACCTG

GATGATGAGCAGTAATTACCACTACTACTGTGTGATGTGCTTAGTCTCTA

GGCCACTCCTGTCTCCTCTCCTCCTCCTGCTTCACAGCTTCTTTCTCTCT

TGCTGTTGTTCCCCTGACCTGATCTCTTCTCATTCCACCTATGTTCTTTT

CCCTGGCCCCCTTTGTTGTGACCCATTTATCACCAGTGTCATCGTGTCGT

GCGGTTTAAGCCGTCTTTAACCACTTTAGTCTTGGTTTAAGGACGTTTTC

TGTGGTGACTGGTCCGTTTTGCTGTCAGCCACTTTTT

>TSAW.R55.esd 503 0 503 ESD GOOD: 70-503

GCACGAGGTTTGGTGCAGTACCGGTTGCGGATGAAACTGGTCACCAGATC

GACATTACCTGCCTTAATGGGCATTGACGCGGTACTGATTGTGGACTGGA

TCCATCAGCTGGACTATATGCAAATTTGGTACTACAGCTTAGAAAGGAGC

CATTTACACGATGTCCCGTCCTGTACTTCCCAAATCCTAACAAAGGCGAA

CCTCTTTAAGAATCCCGAAATGCAAGATTACATCGTCCGGCGAATCAAAG

TAACACTCTCCATGGGCCTTGAGGTGAGACTGCTGCGTGCGACAAATAGG

AACATCCCTCAAATCAAGCAACGGTTGTCGTTGTAACTCCGATGTCCAGA

GTAATTAGGATGCGCGCGGTAACCCAAGAGCTTTGGCGAAGGCAGTACAC

ACCTGCTCGAAAAACCCGGCTTTGACTCATCGNN

>TSBR.R48.esd 696 0 696 ESD GOOD: 106-608

GCACGAGGCTTTTTCGCTGATGCCAACAAGGAGGCTGCGAACATTCGAAT

GAAAGCCGCACGGGAAGCTTTGGATGATCTCACGGAGTGGTTAGAAGATG

ATGGAGAAATAGCCGTTTTTGATGCTACAAATACCACTCGCAAGAGACGC

GATTTGATACTCGCTCACTGTCGACCGCATAATTTTAAGGTTATCTTTGT

GGAGTCCATTTGTGACAATCAGGACGTCGTTCAAGCAAGCATTTTGGAAG

TTAAGGTTAATAGTCCCGACTATATTGGTATGGACAAAGAATCTGCAATG

CAAGATTTCCTTAAACGAATTGAACACTACGCTGCCCGATATGAACCCCT

CGATGTCGAGCGAGATAGAGACGTCTCCTTTATTAAAATCATCAATCAGG

GTCAACGGTATCTGGTAAACCGAATTGCAGGCAATGTGCCCAGTCGAATT

GTCTATTACCTGATGAATATCAACGCCGCGAAACGCACAATTTATCTAGT

TCG

>TSAY.R46.esd 557 0 557 ESD GOOD: 100-489

GCACGAGGGCCGACTGGACGAAGCTGATGCCCTGTTGAGGAGGGCCATTT

CACTGCGCGAGGACAACGTGGATGCCTATCAGAATCGTGGCAGCATTCTT

GTTCGCCAGCAGAGATTTGTGGAAGCCGAAGACATGTATAGGAAGGCGTT

GAGGTACAAGTACACCAGTGCGGCCTTGCACTACAATCTTGGCGTCGTTC

TCTTGGAGACAAACCGAACCGAAGAGGCCTATGCTAGCTTTAACCAAGCC

CTTCGACTTGATCCTCATCATGAGCAAACGCTGTTCGCTCTTGCCTCTTC

CTAATCCGAGACAACAGATCCCGTGTTACACGAAAAGGCGATGACTTTTT

TTGAAGACCTGGCGGGGCGTAATTACGAACCTGTTCGTGT

>TSAI.R47.esd 706 0 706 ESD GOOD: 97-613

GCACGAGGCGAAGGACAAGTACGGCAACACGGTAACAAAGATAGGACGCC

CTCTGCCGGTGGAGTATCTGTTGGTGGATATGCCCGCCGCCTTCCCTGTA

GAGCAGACTTTCACCTTCGCCGAGCAATCCACCTACCACATTTCACCCCA

CGACAAGTTCCCTGTGGAGAATCGGGAGGACCTAGGACTGAAGCAGAGTG

TGGAGGCCTTTGCACGCCAGTTCGCAGCCTACGGGCGGGATCGATTGTAC

GCGTGTCTGAACAACTTCCACGTATTGGCCTGGTTGGCGGAGCAGGTGGA

TCGACTGCCGTTAGATCAGTCAAGTTTCAACAGCCTGTTGGCCACCCTGC

GTGCCGCCAAGTCTGTCACCGAGTGGGCTGACAGCTGTCCTGCCTGGGGA

AGCCTTGAGCTCATCCTCAAAACCCTCGGCGGCGGTGGAACCACACCTTC

TTCAGCAATGGATACGAGCAGTAGCAGTGGTGGTGGGTCGCATCGAGTCG

CACAGACGTCATCGGCG

>TSBA.R48.esd 572 0 572 ESD GOOD: 103-393

GCACGAGGCTAGAGCAGACCATTCGTGCTAGGATTGGAGATCATGTCCGT

GGCAGAAGAGACGGACCGCAAGAGGTCTCTCGCCTTATCGAGTTGGCAAT

CATTCACCCGGACTATCGAAGAGGATTCTCCGAGCAAGGTTTTGATGTGG

CCCTGCTGAAAATGGATGAACCAGTGGAATTTGGTGGGTAAAAATTTATA

ATTTGTCTACTTCGGGATGTGCCCAGTAGCTGTTAATCCTCCCAACGACG

TTTCAATAGAGGAATTTTAACTAAAAAAAAAAAAAAAAAAA

>TSAS.R87.esd 656 0 656 ESD GOOD: 97-255

GCACGAGGCTTACCCTACGCTATTGTATGATCAATGGCGGAGTCGAGAAG

TAGAAGGTTGAATCCAGTGGACGAATTTAAGAATATGGAATTTGAAACGT

CTGAGGACGTGGACGTCTTACCAACTTTTGATTCAATGAACCTTAGAGAG

GACCTTTTG

>TSAH.R65.esd 483 0 483 ESD GOOD: 98-462

GCACGAGGCTCACCTTTGTCCCTGCCATCGGAATCGGGAACTAAGCAATA

TTTGTCGGAGAGCCTAATCTAAGTGTCAACTGCTTGGAGATACGTAATGT

CTACCCTCCTCCCTGCTTCGTGTACGGACTGCTCGGATTCACGCAATTCT

GATCGCCTCCAATTACTGACCGCAGCATCTAGCCTGATAATGGGACGAAT

GCAGTTCTGGTAGCCCTCCTCCTTTCCTTCCTTCCCTCANCTCCTCCCTC

AACTCCTCCCTCAACTCCTCCAAATCGGCGCTAACGCTTGAGTCGCCTCA

CAAAATTTGCACGTATGCGGTTTTAGAGCAATCTCTGTGAGCCAAAATGT

GTTAAAATGGTGTCT

>TSAT.R80.esd 776 0 776 ESD GOOD: 110-604

GCAAGGGATGCAGGCTGCGGGCGATTCGGATGGTCCTAGTTGTGGATCTC

AAAACCCTTTGATCATGCATGCCAATCCTGCTACAGTGGCTGGATTGTCG

TCAGGTGTTGTATCGGCTACGTTTGCTTCCAAGATCATGCAGGCCGTGGA

CGAGCCCACCTCGGATTTACCCGACAACAGGATTTTGAGACAAATTGAGT

TTTACTTCTCTGACGCAAACATCTTGAAAGATCAGTTCCTTTTGAAATCC

GTTAAAGCTAATAAAGATGGCTGGGTGAGGCTGTCTACAATTGCCGGATT

TAGACGCGTTCAAGCTCTAACAAAAGACGAAGATGTGGTTCGGAGGGCTT

TAAAAACTTCAACACAAATAGAGTTGTCTGAAGATGGCACGCATATAAGG

CGTAAGCATCCACTTCCGGAATGGGACAAGTCTGTGTATTCAAGGACTAT

TCTCGTAACGCGCTTTAAGGAGAAGGACATTGTGACGGTTTCTTC

>TSBE.R9.esd 572 0 572 ESD GOOD: 104-572

GCACGAGGGATTTTTTCCCTTCAACAGATAGAGTCATCGGAATGGGAGGA

TTACGGCCGGACACTGTTTGAGACCGCGCTGACTCTAAAGTTGAATCCAC

GACGCTTAAAAAAACTTCGGAATGAATATATCTTGGCAAGAAGTCAGGAG

CAGGCGGAGCAAATAGAAGTTAGGCGACTTCGAACAGAAAATGGACTCCT

ATTGCAACGATTGGCGCGTTTGGAGGAGGACAACACCTATCTGGCAAAGC

AGTTGGTGGATTGTAACGTGGAGCGCGCCGAGTTGTCGGAGGAGGTGTCG

CGCTTGCGTGGTTGCATTGCGCGGTGGCGGACGACGCAAGAAGAGGCGTC

ACTCCGCACCCCCACCACCTGTTCCCCACCACAGGCTACGACTGAGGCGG

TAACACTGGAAAGTGGTGATAGCAAAGCCTCCGATTCCTCTTCTTCCATC

TCTCTCTCCGCCTTTNNNT

>TSCE.R49.esd 583 0 583 ESD GOOD: 98-581

GCACGAGGCGCAAGCATCTGAAGCCCGTCCTTGGCGGATACGTCACTCCT

CATTTTTTCAATGGCGTTTCGTTGGGTGGCTTTTGCACAATCTGGCTTCG

CTTTTCCGCCATTTCGCCTCTTTTCTGCTGGGTGCAGCCGCAATGTGTAT

CCTCCTGAAGAAGCGGGGAAAACTTGCACCCTTCGGTCACGTCCCACTGG

AATAACGGGTAGAGTTTCTTCGCTGTCACGTTATTTGGTTCTCTTCACCC

AACAGCTCAGTATAACACCCGCGTCGCAAAATTTTCGTGGCTACTGCTTA

CTTCAATATGGTGTTCAAAGCATGCTCACAGCCATGGCGATCTATTATCT

CATCTTTTTAATGGTATATGTGCCCTTGTGTTCGTTACCGTGTAATCTAA

CCTCATGCTGTTCTCCTTTCCATTCCTTGTATCACCATGTCAGATGTATT

TACTATCTACGGGTACATGCATATTGATATAATT

>TSBN.R71.esd 484 0 484 ESD GOOD: 104-449

GCACGAGGATGCTAAAATATCATCCCTTGCGGTAACAACGGATCTGGACG

TAAAGAAATTCAAGGGGAATTTTGAAACGGTTACCGACGATGCTAACGTA

GACTCCATCAGCGATGGTCCAAGTCTTTCATCCTCGATCGACTCCTTGGT

CAAGCGCATCGTACGACAAAACGAGGCCGCCATGAATAATCTCCACGTCT

ACTTGCGACAAGTTAACCCCTCGGCTGCCTCTGAACTCAATAGGATCTTA

AATCTCAGCAGTTCTGGTGCCGATGACGGTTCAGAAGCTCCCCTGGCTAG

CGTTGCGGGTAAGGCGTTCAAACGACCCGATGTTCTGACACTGAAC

>TSCE.R75.esd 463 0 463 ESD GOOD: 109-461

GCACGAGGCAGCGTCCTGTTCCAGTGGTTTCGACCAATGCAGTCCGGCCT

CCTGTCATAGCGCCTCGATGTTACTTGCCCAACCAGAATGGCATAAGCGG

GGGTACTGTAACGGGCTACGCGCCGAGAGTGTTTCTCAACTGTGATTCGA

AGCAGTCTTCCTCCACGATATCGAGTCAACACCATGCTCCCTCCTCTAGC

CTCTTTGGTCAGTACCTCAGCCATCAGCAATTTCATAATGGCACCGGTGG

TGGTGGTGGTGNTGTATCGNGATGGCCCCTCTCCCCTACAGCACGCGAGC

CATCGGGGATTTATATGACGATCGTCGCAAGAGCATGCTTTCATAGGAGG

AGA

>TSBL.R19.esd 506 0 506 ESD GOOD: 105-502

GCACGAGGGGGACATGTCTTTCGGGCTATGGTGAGCGGCTCACTGATGAA

GAGATTGATGAGATTGTCAGACTCACCAAGCTCCACGTCGATTTGGATGG

CAACGTCAAATATGAAGAATTTATCAAAGGAGTCCTTGAGGGTCAGCCCA

AGTAGAAGAGAGGATATCCTCTGCTAGACTACGCAGCTATGCACCATATC

CAAATGTATAATTTATAACAGAAAGTGCAAATCGACACAGGCGTTCATTT

TTCTTTGTTTTTCTTTTCGCGTGGACTTGCTTCAACCTCACCCGCACCCT

CTTCACATCTCTTCCATGCGTCCTATGTGCTCCGGTAGGGCTTCTCAATT

CTTTTCAGCCGTTTTGACATTCATTTCGAGCAAACTGAGTCATGGAAA

>TSBE.R2.esd 622 0 622 ESD GOOD: 98-617

GCACGAGGAGCGAGGAGAGCCTAATTGAAGAGGATCCCTTTGAAGAGAAG

GAGGAAAAGGGCGATTTACTCAGTGGTGATGCTGACACTGATTCACTCAT

GTCTGTCGAAGACGAAAGCAGTTTTTCTACTCTTGATATCAGCGGGGAGT

CAGACCCAGCCCCAAGTCATGTCATCATCTTCCATTGCGAGTTTTCATCC

AAAAGGGCGCCCAAAATGGCCAGGTTACTGAGGAAACTTGATCGTTCATC

AAATCTCAGTCGGTATCCCTTCCTATTCTTCCCCGAGCTGTATGTGATGA

AGGGTGGATACGCAGAGTTCTACAAGCGTTTCCCAGATCTATGTGAGCCG

TCCGAGTATATGAAAATGTTTCACAAAAGCCACAAGTCGGAACTCCGCTA

TTACTGCCGTCTCATGGATCAGGTCTCCGATTCCTGCGATGCCACTTTTC

GCAATTCGACCCTTTTCAGCCTCACCCCTCGGAGTGGCTCAGAAAACAAG

GAGAACGAAGCGCATCCTCT

>TSCE.R79.esd 776 0 776 ESD GOOD: 98-633

GCACGAGGTTCTATTTAAATGGAAATCCCATTTTAATTTTTCGTTGCGGT

GAAATGCAACGATTTAGTCTGCCCCTAGATAACTTGAGGTGGATTTCTTT

GTTCTTTGCTCATAAACTAGAAGTTAAAGGTGCTTATAGGAGAGGAAGCT

TGAACCCATTTAATTGTTTTAATTTCAAACAATTCACCTTGAGAAATAAT

CTTTGGCTTGTCACAGTTGACTCAAGCTGTAAAGGGTCACGTGTCACACA

TTGGTGACGACGGACTTGGGTTAGTGAATCAAGTCCACTCATCCATGGTA

GGCCAGTGATAGTTTTGCTTTTTATTTTTTATGACGACGAGAATGACATA

ACTGCAATTACAAATCTTTATACTATTTTAGGAAGAGGTTTGCTGTCGCC

AACTCATGGGACGACTTTGAGGATTTTAAGTAAAGGCCTTGCTTTCACAT

CTTTGCTTAAAACCGAGAGGGCTCTAAACCGTTATTATTTGTGCCAGAAT

ACCTACCTATAGCTTAAGTTATATTTGGGCCGATTG

>TSAQ.R91.esd 675 0 675 ESD GOOD: 30-481

CGCGGTGGCGGCGTCTGACTATGATCCCCGGGCTGCAGAATTCGGCACGA

GGGGAATTAAGTCAGAGTCCAGGGGCCTCCACCCTCATGAACCCATAATC

AAGCACGCAATCATCTCTGAATTTTTCGACTTTTTCGATGGGAAGAAGTT

TCACCCCGACCGTGGCTTCGACGCATCTACCCTCAAGGGTGAAGATTCAT

CGCTCTGCTGTAATGACTTGAGTTTTTACGAAGTGCCTTCCAAGTACAGT

CAATGCATTGCATACATCCCCTTCTCCAACTTTTTTAAAACCGATTTTCG

TGCTGTGTTAGTTGTTGCTTTTGAATTCTTATAGACGAAAAATGGATATG

ACTCGAAGCTAATTTAACCTCAAAAACATATGAACTTTTTGTGCTTAACC

AGATGCTTTGGTTGATTGCAAAAAAAAAAAAAAAAAAAAAAAAAAAAAAA

AA

>TSBD.R57.esd 489 0 489 ESD GOOD: 99-457

GCACGAGCCTCTTGCCGAATTCTGCCCAGGGTCTGACGAATGCCTAATTC

GTGACCAGAGTGCATTCTCGTGGGGATCCCATTGGAAGGATACAGAATAT

TGTCGAGATTTGTGGAGAAGCTTGAAGACTATCGACGAGAAATCTGGAAG

CCCTGGGCGAGGAATTGGCCGATCCGAGCCAAAGAGGGAGCTGCTGCTGG

ATGTCTACAATGATTGCTTCTCCTTCCTTCTTTACCTTTGGCTCTGTGTC

TTTCATGCCTCCTTCTACTCCTCTCCGTCTGTTCGCGTTCATCCGTCCCC

GTGAATGATCCTTGTGCCAGTTTATACAGGCCCTCTTTGAATGACAGCGA

TTTCGGTCT

>TSAW.R12.esd 580 0 580 ESD GOOD: 70-537

CGGCACGAGGCTCAATCCCTCCGGAGTGTGAGTTTGTAAAGTGTACAACT

GTTAGGCATCGTTGGCTTCATTACATGACAGACGTGACTCCAATACAAAG

TCCCCCGGAGCGCCGTCCATGGATGTTGGACCACCAAGAAAACCGCACAC

TTGAAGAATCGGCCAAATATGTACCCTATTCCACAACAAAGCCAAAAGTG

GAACCCTGGATTTCAAAATGATTTATCGTAGCCCTCTGTTCGATTTGTCA

GTTAGTACAGCTATCTATTCTAGAATAAAGATTTACGAAGCCTACGGTTC

TCACCTCTGTCTCCTGTGTTTTTTTTGAATAATGCTTATCTTTACTCCCG

NGTAGATGAAAGAGGTTAGGCTCCCTCCTTCTGCTTATCAGACGATCGTT

ACTCATGCTCTTTCCCACGAAAATGAAGAAATCATAGGTCTCCTCTGCGC

TAAAGAGTGTGATTCGTA

>TSBD.R33.esd 559 0 559 ESD GOOD: 96-558

GCACGAGGCTCGAATTAACCACACCTAGCCACCTTCCGTCTCATATGGAG

CACTAGTGATGACTTGGATGACGATGCACTTTGAACCTTGTGAGGCAGAC

TTCGAAGAATTAGCGTGGAGGTGCGGCCTCGTTTGCCGTGTGGCCCGCCA

TGATGCAGCGCGCATGCAGCCAATGGTGCGCGATGATGCGCCCGCATATG

AAGCAGACCACGAAAATGCCCAGGATCCAGCCATCAGACCACGTTGAGCT

TTCTACCTAGTGCTTCACCTCGACTGCCATGCAAAAGGGTGCTTCAGTAA

AGTCGATCATTTCAAGATCCGACTGTCGAGACGTCATATGAGTGGCCGAA

ATTCGTAATGAAAATATGACCCAGATCAGCAATGGGGCACTGGTAAGCTT

GACGTCTAAACGGACGATGCGCATGGCGTGAAGATGCTCTATACCACTGA

TGATTTCGCANNG

>TSCB.R30.esd 520 0 520 ESD GOOD: 117-467

GCACGAGGCTGAAATTGACAGGCCGTAGTTTCTACGCGGAGAATGGCTAC

CTCTACTGCGAGCAGCACCATCGTCGCAACCTGGGCATGGTGTGTGCAGC

GTGCCAGGAGCCCATCTCGAGCAATAGGTGCATCAATGCGTTGGGCAACC

GGTACCATCCGCAACACTTTGTGTGCACCTACTGTCTGCGTCAGCTCAGC

ACCGGCACCTTCAAGGAGCGCCTCGGCAAGCCCTACTGCGACTCCTGTTT

TCGCCAGCTCTTCGGCTGAGGCCTCGCGCTGATCGTCTCACCGCAACCAC

GAGGGCTTTCATACTCCCTGCCGTTACAATCTCTCCTCATCATGACATTT

C

>TSBS.R88.esd 607 0 607 ESD GOOD: 96-343

GCACGAGGACCGATCTTTATCATCAAAAAAATATGTTACATAAATGTAGA

CCTTCGGAACACATGGGTATAAACGGTCAAAAACGCAAATGCTACGCGCT

CTCCCAACACAGTTCTCCTAGCTGCTGAGTGGCGTGGAGAGAATGTACAT

AAGGCTGCGACAGGCAGGACAAGAGCGTGCACAAAATTCAACACTACTCC

ACCGCGAGGAGTTGGCGTAGCTGCTCGGCCAGGACCGACGAGTTGTCG

>TSAG.R50.esd 674 0 674 ESD GOOD: 92-447

GCACGAGGGGTGAGCATCGATGCAGCTCGGGCTTTGTCCAGGAGCTGTTG

TAGTATTTGGCTGGCCCGGCGGGGCCTAGATCTGATAAAAGGGGTCTGAT

GGGTCTTGGTCCGTCGCCCGCCTCGTCCATTCACCTGTGGAGCGCTAGTT

GCGTGGCTTCCACTGTTATGGGCTAAGCCGTAGACTCTAGGCTTCAGGCT

GCTTGATATTGTTGCCTAGTACTGCAACGTGTCGCACCGCATTGTTGTGG

TACGGGATGGCAAGGTGTCAGGTTGCAGCGTCAGTGAACCCCCACGTTTT

CAANGGCTCTGTCGCCAGAGAACGTCTTCGATTCTATAAAAAAAAAAAAA

AAAAAA

>TSBA.R10.esd 563 0 563 ESD GOOD: 98-522

GCACGAGGCACATCTTCAGCAAACCAATGTTGTCCTTACCCACTCCCTCT

GCCGAATCCTCACCTCGTTCCTTCCAGGTGTAAGCAGGCCATGAAAATCT

GTTAGTTATATCGCACCCACAGTCTCCTTTTCTATCCAAAAAGAAGTTTG

CTCCGATCTCCCAAACTCAGTCAACCTGGCTTTTGGCACAGACAACCTGG

TGATACCAGTTCTCATTAAGCGGTAGGGTACACTTGTCATATCGGTGACG

TTTGAGCGCGGGTAAGAGTCCAGGCGTTACATAGGCATGATGGTGGTATG

GAATGCCTTAAAAGTAGGTTGCCTTCACTTTTCACACCCAATAACAGTCC

GGTGTTGTTGTCTAGGAGACATCCTCCAATCAAGTATCATTGCCCAAAGA

CGATGCGGAAACGGTGAGAGAACTT

>TSBJ.R95.esd 424 0 424 ESD GOOD: 92-358

GCACGAGGGAGACGCGGTTCCTCCGAGGGGTGAGGGACACTCTCGTCATC

GATATACGCTTACCACACTTTTCCTACTCAGTCATGTTAAAGATGGCCCG

CAGCACTCTGCCTCCTAATAGCATGGCCGGAAGGAGGGAAGGAAAGGGGA

GATTACAGTTACCATGGCTCACGAGTGAAGGCGGGTTCGTCGAGGATGAA

GAAAAAATTAAAAGTGTGAGAGGCGGGAGGACATTTTTAGCCTGGTCGCG

GACGCATGTGCTTGCTC

>TSAS.R96.esd 753 0 753 ESD GOOD: 103-595

GCACGAGGGAAATCTATTCCACTCCGGGCAGTGAGTACAGTTTCCTATTC

ATGGCCAAGGGTGGTGGGTCGGCCAACAAGTCCTTCCTATTCCAACAAAC

AAAGGCCGTATTGAACGAAAAGAGTCTTTATGAGTTTGTTGCAGATAAAA

TGAAGACTCTCGGAACCGCTGCGTGCCCACCTTACCATTTGGCTATTGTG

ATTGGAGGCACGTCTGCTGAATTCAATCTAAAAACGGTTAAACTGGCATC

TGCACGCTATCTTGATCACCTTCCTACATCCGGAAATGAACATGGCCGAG

GATTTCGCGACTTAGAGGCTGAACAGCGCATTACCGAAAGCTCTCAAAAG

TTGGGAATTGGGGCTCAGTTTGGTGGGAAGTACTTCTGCCACGATGTTCG

AGTCATCCGTTTGCCGCGACACGGTGCCTCTTGTCCCGTTGGAATTGGCG

TCTCGTGCTCCGCTGACCGACAGTGTTATGGTAAGATCACAGA

>TSAV.R74.esd 697 0 697 ESD GOOD: 104-595

GCACGAGGGAATATCATCACCGTCGGAATGGCCGCATGGCTAGGGTGCTG

CAAGTTCTCCCCGGCAAAAAGGCATGGCAATAACCTCATCTACTATTACA

TCTTCATCAAGATTCTTTACGTTGCCATTGGGATCGCGCAACTCTATCTA

ATGTACGCGTTCCTTCGTTTCGATAAGAAGGAGGGGTACCTTTTCTTTGG

CTGGCGGATTCTGGATGACATTCGCAGAGGAAGGCCTTGGACGGAAACAC

AAGTATTTCCACGTATCGGCGCTTGTCGACACACACTTCAGCACGTGGCT

GCTGGCAACAATTTGTTCGCTCAATGCGTTCTACCCATCAACATGCTGAA

CGAAAAGATATACATTTTTCTCTACTTCTTTTTGGCTTCTGTCATGCTTT

TCACCATCGTTAGCATTCCACTTTGGATGTTTAGAATATCAAAGCATCGT

CAGAGACATTTTGTTAAACGTTTTTTGAAAATGGCGGATGTT

>TSBW.R86.esd 742 0 742 ESD GOOD: 72-535

CGGCACGAGGACAGCGTTCGAGGCTCTTATGCCGAGGTGGACACCTTAAT

TGAGGAAGCGCTTCTTGAAGAATCTGGACATGATCCAGCCAGCGCTTACA

CTTTGCTCATCATTCGAAGCATCGAGTCTAGCCAATATCCGACTGGTGTC

GACGCAAACGATGCGTCCACTCCCAGTATTCAGACCTTCCGTGATAAACG

AAAGCAGAACTTTGTTACCATAATTCGTAATTTAATTGAGAACCCCGAAA

AGGAGAATTTTCGCCGACTCAGAGTGGGTAACAAGTCAATAGCGGATCTT

TTGGAGTTGAAGTTTGCACTCAATTTTTTTGAGACCTGCGGTTTTACACA

AAGAGAGCAGCCAATTAATTCTGAGGAGGATGCCCAACGGAAAGGTGTTT

AGTCTTCCCGGCAGCGCCAACTGAAAGGGAGTTACACCACTTGGAAAAGT

ATGCTTAATCTCCT

>TSAG.R19.esd 751 0 751 ESD GOOD: 96-540

GCACGAGGCTTCCTACCCGCTCACAAGGACATTCCCACCATTGCTGTTTG

GAGTGCCGAACTGGACATTTCTAATTCTTTGAATCTGCATCAAATTGATT

CCGCCTACGCACTGTGGCTCCAACACCCCCATCGTCTGATTGGATTCACC

GCTCGCGCGCATATCTGGGATAGCAAGATGGGTGCCTGGAGCTACTCTGT

CAATGCCTCTTTCCATGGCAACTTCTCGATGGTCCTTCTTAATGCCGCAG

TTTATCATAGATACTACCACACATTGTATTGGAGGCTGACGACGCCCAAG

ATGCGAGAGACTATCGACACCCTGGCTACAGGAGAGGACATTCTGTTTAA

CTGCGTGGTGGGCTATGCAACTCAGTCGCCACCGCTCCTCTTGACTGCCA

AGGGCGCGCGCTTCCCAATGTCCGCCGAGGCTTCTAGTGAGTTAG

>TSBF.R58.esd 559 0 559 ESD GOOD: 90-553

GCACGAGGATTTTGTGCAAAGAATCGTGAGATGAGAAATCTCGTTCTCCT

TGATCCCATGGCACTATCGCCACTGTCGCCCCTGCTTAACCTGATGCGTC

ACTGCGTGGAGCCGAGACCTTGCCTTAGTGGTATCCTCTGGATGACGCCA

ATGGACGCCCTCTCGCCCTCCTACCACGTTCCCATTCCTATAACGACACG

GCAGAGAGAGACAGAAGGTGATGGCAGAGGAAGAGACAACGATGCCTACC

CCTCAGATGCTCATTTGCGCTTCCTTACCATCACCGCTTTCCTCACGAAT

TGGGTGGCATGGCTATCACGAGTTGAGGCATACATGGTGCTCGGTACGTC

GCGCTTCTGCCCAGCCTTGATCACTGCTCCCATAGCCGCGTATGTTCTAC

AGCCCCTCAGTCTGGGCTGTGGACAGGCACCGCTTCTCGATCTCTGGCCC

TTGTGGCTGCTCCG

>TSBC.R72.esd 735 0 735 ESD GOOD: 97-246

GCACGAGGCTCTTTTCCAGTGAATGTATATTATTTGAAAAGCAATATCCT

CAGGCTTCTATTCTGAAGCTCTTTTTCATCAAAAAAAAAAAAAAAAAAAA

AAAAAAAAAAAAAAAAAAAAAAAAAAAAAAAAAAAAAAAAAAAAAAAAAA

>TSBO.R32.esd 757 0 757 ESD GOOD: 111-600

GCACGAGGCTCTCTCTAAGGTGCGTCACACTTCCAGTTACTTTTCTTCCA

GAGCGCTGATGTTGGAAAAGTGCTCAAAGACCAGTACGAAAACAAGCGCT

ACATTGCGGCAATTTGCGCTGCACCCTGCGCTCTCAAGGCGCACGAAATT

GGCAAAGGCGCGAAGGTGACCAGCTATCCGTCGATGAAGGACGATCTAGA

CGCCTACTACAAGTACGTGGATGAGACGATTGTTGAAGACGGGCATCTCT

TGACCAGCCGTGGACCGGGTACTGCCACCGCTTTCGGTTTGAGATTGGCT

GAGCTGCTAACGGATCACGCCACTGCTAAATCAGTTGCCTCGGGCATGCT

CCTCCCCTTCAAAGCCTAATGTTCTCCAATGTTTGAGTCGCTAATCGTGC

GCTTCTTGAAACTTAAAGGCTCTCGTCCCACTTTGAGCCCTTAGAAAATT

TAAATAAAATCCCCTCATTCAGAAAAAAAAAAAAAAAAAA

>TSBI.R4.esd 757 0 757 ESD GOOD: 181-327

GCACGAGGGGAAGAGGTGAGCGCTAGTCGTGGTGACAATGGCCGTCTGGA

ACCCATGACTGCGCTCTACTCTGTCGATATGGATGACGATCAGTTAGTGC

ACATTCTTCCCTGGGCCAACCTGGGCGACTATAAACAGACCTTCTTT

>TSBB.R53.esd 366 0 366 ESD GOOD: 95-363

GCACGAGGGAACGCTGTTCTCCTCGCTGAGGTTTGCGTGGGACCAGCCCC

TGTTCGCTCTCGACGTGGTTGGCTCCGCAGTGTGCTCTGGCTTTGGGCAG

ATACTGATTTTCGGCACCATAGCCGAGTTCGGTGCCGTTACCTTCACCAT

CATCATGACAATCCGGCGTGCTGTCCATCCTCGTGTCCTGCCTCCTCTTC

GCTCATCCTATGAATGTAGTCGGCGCCTTGGGCCTGCTAATCACATTCGG

TGCAATCCTCTCTCGTGTT

>TSBL.R81.esd 599 0 599 ESD GOOD: 86-527

GCACGAGGGTCTCTCCGACGTTGATCGTGAGGAGAAACAACGACTTATTA

AGCAGGTTTTGGAATTGCAACACACCTTAGTGGATTTATCGAAGCGTGTA

GATTCAGTGAAGGAGGAAAACGTAAATCTACGTACTGAAAATCAGATATT

GGGGCAATATATTGAAAAATTGATGGCCACATCGTCCATCTTTCAGTCCT

CATCGCCACCACCTCACGGTGTTCACTCCACCACTCCCCCATCCGCCGTC

GCACCGTTCATTGCTGAGGGCGTCAACAACTCGGACGCTGCCTTTCACTA

CGAGGAGGGCTCCGAGTTAGCCAGTGAAGACGATATCGCCTAACTACTTC

TGCCCATTTCCCTCTCCTTCCGTTGCATTTCACTTCATTAAGTGATTTTC

TGACTTTTAATTGAGTCTGATAAAAAAAAAAAAAAAAAAAAA

>TSAU.R24.esd 579 0 579 ESD GOOD: 114-515

GCACGAGGTGGTGATGCTGATGCTGATGATCAACGCTGAAAAGTACTCTG

GTTTATTATTTTTTTCCCCTTTTTTCTCCGTTTCTCTCTCTCTCTCTCTC

ACCCCTTCTCTCTATATATCTCCTTTTTCTTTTAATTTGGAAGTTCTCAC

CTCCCCTCCACCTTCCACAGACGCCCTCCTTGTGCGTGCGTGTGTGCGTG

TGTGTGTCTGCAATGACTTTCCCGAAGAATGACTCCTACTGAACCCTAAC

CCTTCTTGTTCTGATGAGCTGAGCCGATCCGACTTGATCCGATTCGGTCT

GATCTGTTCTATCTTGCTGCTGTTGATGCTGTTGAAGATTACAGTCTAAG

TGGCAATATCATGTAATAAATTTGCTACTGTGAAAAAAAAAAAAAAAAAA

AA

>TSAK.R19.esd 835 0 835 ESD GOOD: 103-499

GCACGAGGATCCGCGCCAGATTGATCATGGCGCAGCGTCTGGCTGACCTG

GATCCCTCCTTGGCTTCGGTTCGAGCACAGGCTGTGCGTGAGAGTCAAGA

GATTCTGCTGCGTCTCAACAACCATATCCGAGCCTCACCATGGAGGTCAC

TGCCTGAGTTAACCCAGGTCAATGGGGAGTTCTGTCCGGGGTAGTTGTGC

CTCGCAGGCATGGAGCGTCGGCACAGCGGATTGAAGCCATCTACGATCTC

CTCGAGACCAATGCCCGTATGCATGGTTTACCGTTCCCCCCGGCCTGACC

ACCCTTTCCCATAGTTTTTTTCTACTTGTTCCCTTTTCTCCTGCTTTATC

TTATTTTGGCGTTCGTTTTCTATTCATCTCTCGTTCTGAATCCTGTT

>TSAF.R71.esd 672 0 672 ESD GOOD: 114-612

GCACGAGGGCAGATTGTGCTCTCTGCTGCCGACTCTGTTAATGGCGATGT

TGGTGATTTTGCGGCTGGCTCTTCGCGACAGCGCTACGCTTTAATGCATG

TCACGGGGTATGTCAAGAGCGTTCCCTCAACCGTTGAGTCGCCGAACACG

GTCAATGCAGATGGTTTTCCGATTTCACGTTCGTCTTCGGACTTTGTTGT

TTCACTCTTGGATCGTGCCACATCGGAAGGGATGAATGGAGCAGGCAGCG

GCGTTCAAGAGCCGGGAACGTCGCGCGAACATTCGCAACCCCTGTATTTT

GTGGCGCTGGCTCGCCTCCAGCTCACTAACCTCCCCAGTGCCGCCGATCT

GACGCCTCACCGGACTTACCAATTCACTGTTCGTTTGGATGAAGAAAAAC

AAATCACTTTTTGTGATCACCGCATCTCCAACGTTTTCACATCTCCAGCC

TACAGTTCTGTCTCCCCTGAGCACATCCTCTGCACAAAGTTCACCGATC

>TSAA.R33.esd 634 0 634 ESD GOOD: 97-566

GCACGAGGAAGGCGCAGAATCGTCATATATATCTCCATTCGGTCATCAGC

TGTCTTAAAAACTTAAATTCCCAACCGGCTTACGAAGCCGAACAGGCACC

CCTGCCTGCAAAGGACAGTGCAAAGCCAACGACAGTTAACAAGACCGACT

TATCAGATAGGAATTTTGATGAAATGGTCAAGGGTCCAATATTTTACAAA

GAGTTAAAGCCTTTTCTCCTGACAGAGGAGGAGCTCATTCATAACAAGTT

TCCACGGCCTGATAACTCGGCTGGGGCTCCTCGAGGAAGGGCGACTATTG

CTGTTCCTGATGAGAAGAAAAATGCTGTGGAATTGTATAAGAGTACGCCG

GCTAATCACTTCACCTGTTGTGATTTTTGTATTGCCTTGCTATTTTTATC

TTGGTAGCCACTTTCTGGCAATTACTTTGATTTATAATTACCTTTTCTAG

CCAAAAAAAACAAAAAAAAA

>TSBG.R78.esd 724 0 724 ESD GOOD: 94-273

GCACGAGGCACATTTGTAAGTGATTTGGAGAGAGTTGTGATGGAGGCTGA

AATGCAGTGCCACAAGTGGTAGCGCGGCATCCGTTAGCTTGTCACCTGCG

ACACTTCTGCATTCTCTTGTAAATTATAGACTCGCTGTTCTCTACATGTA

TGCTTCTTTTCATTCGCCTTGTCAACTTCT

>TSBD.R19.esd 612 0 612 ESD GOOD: 105-595

GCACGAGGCGTAAACTTGCCAAGACTGATGGGGTCGATGGGCTCACGCTG

GAAGTATGGATGCAGCACTTTGGGACATCGCAGAATATTTTGATTGACTT

TTTAATTGAACTCGCTAAGGGTCTTCATAAAGATGGGAAGATCCTTATTC

TTCCCATACCGCCTTCTGTCTATAAGGGAAACTTCGAGGGAAGATTTGGC

AAAGCCCACTTTGACATGCTGGTGAATCATGTCGACTTCTTCAGTCTAAT

GACCTATGACTACTCCAACCCTTATGCACCTGGGTGAGAATGCCCCCCTA

AAGTGGGCAACTCAGTGCGTCAGAAACCTCGTTCCAGACGGAAAGGATGA

TGAAAATCAAGCCACAAAACGAGCCAAAATTCTCCTTGGGGTTAATTTCT

ACGGTTATGACTATGTGCCTGATAAGAGGCAGGGNCAGAGCTGTCCTGGG

CCATGATGTGGTGGAAGTGGCGAAAAAGTACTCTCCTTTTT

>TSCG.R80.esd 669 0 669 ESD GOOD: 127-572

GCACGAGGGGGGTCTATGGAAATTATGGGGAGTTCCGACACAACACTGAG

AATTATGCCCAAGTCCTCATCGAATCAGGGAATTAGCTTCCAAATGACCA

GGAAGAATGTGTGCTCTAGCCAAAGACGCAGACATATGATTGAATACCGA

TATTCGTCTCAATACATTGATGAGTGCGCAAATCAGGAGGCACCCTCTCT

ACAAGCCGTTCTTGACTCACTGACTTCGGCATCTTCATTTGTGCATGTTT

GTGAGGAATCTGCTAGGGATATCCATGATACCGGCAGCACGCTATCATCT

GGGTACTTTAGCACCAGTAGCCAATCGTCTCAGCAGACTTGCGAAATGAG

TGATCACTTAGGCCGATTTGTACCATCCCGTTACGAAGGTGGACTATCCC

GATATCAACGTCGCGCGCGGTACACTATACATGAGGAGTCTACAAG

>TSBP.R13.esd 846 0 846 ESD GOOD: 95-489

GGCACGAGGGGATCGCGTAGGTGAGTCCGCCTCTACCACCTCCTCTACCG

TCACTGCTTCTGTCGACACCTTGGCACCGGACAACAGTCCCAAGACAACA

AAAACACCCAAGCATCCCCAAGGCTCTTGAGGTGCTTCACGAGAGTGCGG

TGGAGTTTGATTTGTGAATGCTGTCCTGCCGCACATAGATGTCCTCACCG

GTGCCTCCGTTCTTCTTTCGTATTGAGACAGATTCTGCCCTCCTTCATTT

CCCTTTATTCAATTACTGACCTGTCTGCCCGCCTAACGAGTCGGCGGCCA

GCCACCCGACAAGGAGCAAAACGCCAGCGCGCTTAACCTTATTTCGCTTT

TGTTACAATAAATGGCTGTTTTATTAAAAAAAAAAAAAAAAAAAA

>TSCB.R55.esd 665 0 665 ESD GOOD: 98-538

GCACGAGGCCTCGTGCCGATTGCCACGCGGGAGTGATATTCCCGGCCACG

GATATCTCATAGCCTTCGACGCGTTTCTTACCGTATTGCGAGTAAGTTCC

GTCATCATTTTGCTCAACTTCATTTTCGATATCAGTGCGGAACAGCGCGG

CGGTGAGCAACAGACGTGTATCCAGAACCTGCCATTTGGTGCCAATCTCG

CTGGTGTTGGCTTTTTGCGGTTTAAAATCGGTGCGGTTGGCACTGTTACC

GCTGCCAGACTGCGCAAGGGCGAAGTTGTTGCCGCCCGGAGGCTGCTGGG

AAACGGCATAGTTAATATAGACATTGCCGTTTTCCGTCAGGTGATACAGC

GCCCCGGCTTTCCAGTTCATCAGATTGCCCGACTTGGCGGTGTCGAGCGA

CATCAATCCGGTGGTAAATTCCATGAAAACACTCCTGCGGA

>TSAV.R72.esd 702 0 702 ESD GOOD: 105-619

GCACGAGGCGATCAACTAGCCGCTCTGTTGGGGGTAACCAATGGAGCAGT

TTCACCGAGCAGCGCTGCCAGTTCTGAGACCTCCAGCCAATCGATGGCTG

CCATAGCTGCCGGATTCCCTGGCACCTTTACATCCCTCCTTCCATGCACC

TCCGCGAATCCGTCCAATCCCACGGCTGGAACCATTATTGGTAGCTACTC

CAATGCTGGTGATCTTGAAAATAGCCCAGGTTCACTGAAATTGCGAAGCA

CCACAGTTCCTACCGTGCGAATGCATCCCTATCTACGACAGTAGTTTGGT

CGGTCCAATTTTTATCACTCAATAACGGGGTGTGTTTTGCTCTGTTTGCT

ACACTGTGTACACACAAACAATGGTGATGAATCGACTCAACACCATCAGC

GCCCTCTACATGAAACAGTGGTTGTGGTAATCTATGTAATCCTTGCCCCT

CTTTCCTTGTCTTGTACTCTTTACATTTCACTTTTGACTTATTTTTATCC

CTCTGTCTTTTTTGT

>TSAG.R60.esd 658 0 658 ESD GOOD: 95-533

CGGCACGAGGGTCGAGTTGAGCATCCTAAACGACTCAAGAAGGATTCTGC

GCTTCATAAAGTTCATCAATCGAGGCACAGAGATGGTCTCCCCTCCTGTT

CATCCGGCGTATCATCAACAAGTTCACAGGATGGTTACGATTTCAGGCAT

CACTCCACTCTCCCGCGGTCTATGAACCGACAATCGTACTATAGCAGACC

AGTTAGTGATGATCGCAAGAACTATTTTGATTTCTCTTCAACTACTTTTA

AAGAGATGAAATATCGTCCCAAAAGGTTCGAAAATGCGAAGCAAATACCC

GTTCCTTTGTGGCGAAAAGTTTCTTATCCTGCATCATTAGACGTAGGAAA

GCGAGCTGAAGGGTCAGGTGTCTCACGCAGTACTCGTTTCATCAATTCTC

CCAGAAATAGTCAAATTATGTATAATTGTCGGATATCTC

>TSCC.R43.esd 776 0 776 ESD GOOD: 109-612

GCACGAGGCTCGAATGCATGAGGAAACTCTGAGAAAGCAGGAAGAATCCG

TTCAAAAGCAGGAAGCAATGAGACGACAAACTATCGAATACGAGGCGAAT

TTGCGCCACCAGAATGAGCTTAAGCAAATAGAAGCTCGGCTCCGTGGCGA

AGCCCAAATTGAACGGGAAAATCGTGACATAAGGTTGGAGAGATCCCGTA

TTGAAGCGAGAGAGCATCGGGAGACTATTCTGCAATCAATACAAACCGCG

GGGTCTGTGATTGGTGCTGGTTTCAATGCTTTCTTTGCTGATCGATTCAA

GGTTGCAACGGCTGTCGGTGCTGCAACTGTTTTGGCTGGTGGCGTTTATG

CTGCTAAATTTGGAATGGGCACCTTAGCTCGGTACGTAGAATCCCGAATT

GGTAAGCCCTCGCTGGTGCGAGAAACATCTAGGCTCAACATTGTGGATCT

AATTCGACATCCAGTTCAGGCTTTTAAAAAAGCGTTCAACCGTCCTGGTG

ATCC

>TSBO.R90.esd 386 0 386 ESD GOOD: 94-386

GCACGAGGCTTGAACAAAACAACAGTGTATATTCAACGCTCTTTTTTTCG

CGCTTTGTACTTGTGTAATATTATCGCTCGTTCCACTGACCTCTTCCTTA

TCCTTTTTCCATCATCATCTTTGCCCCATATCACCCACGCACCCGTTTTT

TTTTCCTTCAGCCTTGGATTTTGAGCGATGCCACTAAAACTTTGACGACT

ACCCTTGTGTGTTAGCATGGTCTCTACTTTCATTTATTTCAATCTGAGCT

TGCTTTGTCTTATATTGAAAGAAAAGAAATCTGATTCGAATNN

>TSAZ.R13.esd 793 0 793 ESD GOOD: 100-439

GCACGAGGGGAAGGTTGAGGTCTTCTACGGTCAATTGTCGGTCCTTCTCT

TCTTCTGGCTGTGAGGAGGCTGGCAAAGGACTTAACCGTGCCTTGGAACC

TCATCCAGATGCCGAAAAAGAACCACTCGAGAGATTTCCTAATGATATAA

ACCCAAAGACTGGCGAGAGGGACGGACCCAGAGGTCCGGAACCAACTCGC

TACGGAGATTGGGAACGCAAAGGAAGATGTATAGACTTTTGATGACATTG

GTGCTCTTGTTTTATCATGGGGTATTCTAAATAAATAAACATTCACCTCT

CCTATTTTCCTCTTCGTAAAAAAAAAAAAAAAAAAAAAAA

>TSBG.R38.esd 631 0 631 ESD GOOD: 94-613

GCACGAGGCTCTTCTTCACCGACATCAAGCCAAAGATCAGTCCTTCTGTT

AACATGGGTGGTTTGGTTGGGCATCTGGCGACTCTCGAGGTGCGCGAGGG

TCCCATTCTACGTGAGCAACCACTGGCCTCGTCTCCCGTGGATCGTCAGG

GCAGCTGGGAGCGTGGTGTGATCGACTACACTGGTGCTGATCGATTCTCG

AATATTCAGGCTGCTCTTGACAAACATCTGGGCAAGTGAAGGCCTTCCGA

CATCCACTTGGCCCCAGTAGATTATCACCGATTTGTATTCTACTGGTACT

GCACTCGTTGATCACGCTAGTCCCTTACCCACTTTTTAGCGCACGTCCTT

CGATAAACTGGTTTTGCTAATCTTTCCCTTCACACCCTTCAATCCATCTT

GCCTTCTTTCTGTTTCCTCTACTTTTTGTCCTTTCTTTAAGCCACTTTGT

ATCATGACAAGATTACACGAAACTTGACGATTTTTGTGCATTCATGAGGC

AATGCTAATGGTGCTGACTA

>TSBT.R55.esd 743 0 743 ESD GOOD: 106-546

GCACGAGGGGGAGAAGTCGTCTGATCCGTCGAAATTCGATATCCTCGTCA

AGGTATATCCCAATGGCACAGTTTCCGGGTTCTTGGATAATGCAGTAAAA

GGTTCCGTGGTTAAAGTCAGTCTACCTCAATGCCAGCTCAGTGCCAGCAT

CCTGCTGAATCGTTTTGGAGATGTATTCAGGCCATGGTCATCCGTGTGCA

TGCTGTGCGGGGGGAGCGGAATAACACCCTTTCTACCTCTGATTCAGTAT

CTTTTGGCTCTGCGTGAATCTCGTCTCCACCTCCTCTGGTTCAATCGACG

CGAGAAAGATTTGATTCTGCGCTCGGAACTCGATGATCTCGCTGCATCGT

CAGATGCCCGACTTCAGGTACACTATTGGCTAGTGGAGCCCGAGGAAAGG

ATCATGACATGCAGACAACCAAATGTCACCATTGGTGGGGA

>TSAB.R46.esd 594 0 594 ESD GOOD: 99-306

GCACGAGGAACAGATGGTCGTAGTGCTTATCGCATAGGTACTGTTGGCCA

AGCTGATGCCACCAAGGAAGCTACTGTTGTGCCTGAGGGTGAAGTACACT

TCCGTGGTGGACCTGGTGGGAGGCCTATGTAAAGTCTTCTGAAATAAATC

TTGTTGGATATTTCAAAAAAAAAAAAAAAAAAAAAAAAAAAAAAAAAAAA

AAAAAAAA

>TSCA.R77.esd 635 0 635 ESD GOOD: 112-569

GCACGAGGGCCACGTTAGAGCCTACATTAAAGACCTGGAAAAACTGCGCC

CACGAAGCATCCTTGATGTCATCTCTTCTGGAGAACAAATGGTCATCAGA

GAGGGTGTAAAGGTTGCCGCCAAGGGAAACTGCGTTGCTTGCGGGTACAT

TTCCAGCCAGAAGTTCTGCCAGGCCTGCATCCTTCTCAAGACGCTCAACG

ACGGCCTTCCGAAAATTTCAATTGGTGAAGATGGTGTCATACGGCGGCTT

CGTTCAAAGAAAATCACCGAAGAGGTAGCCAAGTCCCTGAACGAAGTTGC

CCTTGACAAATTTACCTCCTGTGACGCTGGATGCATCATGTCCTGCCTGC

CCCACGACGATTGATGAAAGGTGCTCAAACCTAACTCTGTTTTACATATA

TGTCTTTCAAAACTTCACCTTGAAGCCGAGACGGCAAAATTTGTTTTTAA

TAGTTCAA

>TSCA.R20.esd 652 0 652 ESD GOOD: 107-628

GCACGAGGGTCACATTGAGAAAGACCTGTCGTGTCCTCACTTGTTTTCCC

GTTGAATTATATTTAGACAAATTTGTTTACTGACTACATAATCGGCATGA

TTTAGAGATACTTCCTATTTCGATTTAATGAAACTACACCTAAATCCGAC

TGTCTTGATTATGCACTTTGTACTTATTATCTCAAAAGGGCAAAGGTTAC

CTGCGATGCTTCGCCTTTATTATACTGTGTCAGATTGAAATCGTCCAGAA

ACTTACTTTGCTTAAACAGGGAGCCCAATGTTGGTACATGATCGTCAAGA

GATATATTAGATGCTTTTCAAATAATTTAATGCATTCATTCCGACCAAAA

TGTTTTCTGTTTTAAATAGACAGATGAGTACCAGGTTTTGATTCCCATTA

TTTTTCGCGAACTCCTTCACTATTTATGGCTACAAAGTTATTTGAATTAG

CCTTCTACGGGAATTCCGTCCTCGGGTCTGGCCCAACTAAGCTATGCAGC

ATTGCAACTACAGCAGCAACAG

>TSCA.R11.esd 731 0 731 ESD GOOD: 104-586

GCACGAGGGAGACATCGACTCTACAATGTCTCAGTCACGATAATCATGCT

GTACGGTGGCCGCGTTTTTCTCCTGATGGCTCGAAATTGGTTTGGTTCGA

AATGCCGGCTGGTGGACCGCATGGACAGTGTTTTGCGATGATAGCTCAGC

AATGGCCCCCAAACCAGTTTAAATCTGAAGTCGTTGTTCCTCTTATTGCT

ATGGTGAAGGAGCGGTCAGATTTTCCAGGTCTTTATCTTTGTGACGGGGT

TCCTGAAAGATGTTGGACAGCTGACAGCAAAGGAGTCGTTTTGTCAACCA

TCTGGGGCGCCGAAAAGGCTCTAATCCATGTTAGTTTGGATTCGAGCCCG

TTGGAGCGAATTTTCCGTTTTCCATCGCCTTTGGCTGATGTGGAAAATGG

TGGCGGTTATGGAACTGTCACATTAATGGACATAATTAACAATGTGCTAC

CTGTCGATGTTAGTTCACCGACTGTCCCCAACT

>TSAT.R53.esd 511 0 511 ESD GOOD: 104-505

GCACGAGGCCTCGTGCCGAATTCGCACGAGGGAAAGAAGTACGGATCGTG

ACGATAAGTATTTTGGAGAAAAAAAATCTGTGGAACTTTATTCAGTAGCT

TAGATTTTCATGCAAAAAACAGGCTGGAATTGTTCGTTCTTCCTTCACTT

GCTTTCACTTGCCCTTTTGTGCTCACACCAGAAATTTCCCCCTGCGAGGT

CAACAGGAGCGCTTTTGCTTTTGTTATCATCACGATTTACCAAAACGGTA

GTGCACATATTCCTAAGAATTTCGCCCCACTGCATTCGACAAAACTCGAT

CTCAAAAGCTATTTTTCAACCGACTATTTTTCTCACATTAGCCCTCCCTA

AGAACCTAAAACGTTCTTTAACCACTCAGTATTACGTTTTATGGATTAAA

AA

>TSBH.R29.esd 678 0 678 ESD GOOD: 93-497

GCACGAGGATTTCATCACTTTTGAAGACCGTATTCCCTCAGACTTCCTTT

TTAAAATTGAGGCTAGACTGGGCCAAACTTTCGTTATTTGTTTTCGATAA

CCAATTTATAAGCTGATGGATCATAGCTTAGCCATCACGTACTTCTGTTG

AATTTTCAAATGGCTTATTTTTAAATTGCCCAGAAAAATATTGTAATTAA

AAAATGCACTGATTATGCTAATACAGTTTTTTTTGGGATCCGAGCCAAAA

CAATGCATCGTCCCATCTTAATTTTATTGTTCCGGGCATCTGATTTCTNC

CTATAAATTTGGGCTGTAAATTACTAATTTATGGTTTAGAAATGGGCTCC

TTGTGAAGGCCCGCTTTGGGTCACTTATGTAAGCTCCTTAACTTCGAATT

AAAAC

>TSCD.R88.esd 598 0 598 ESD GOOD: 88-507

GCACGAGGACCTGAAGATAGTGTACTTCTGAGTAATTGGCTCTTCGCCAT

ACGTGAACACATTGAAGAACAAGTGAAATGGGGTGTTGAGGCATTCGGGC

ACGAGGTATACATTCCAGAGGCGACCTCTCTTACCAAGCATGGGACCGTT

ATAAGAGCCTCAACAATTCCAGACCTTCCCGCTGCATCTTTGGAAGCATT

GAGTTCTACCATCGCCGAAGAGGCCTCAGCATCTCCTATCACTGTACTCT

GATCGTTCGCTCCTTCACCCTCCTTGTTTCCATCGTCTCCTACTCTCCCC

TTACGCGCCTAATTCTCAGCTCTTTCCTTGGATCATTTTAATCATGCCTA

TTTTGTAACTTTTATAGCAATATTCTCAANCCCGTTTCCCAATTTTGATA

CCGCGAAGTTGAGATTCGGT

>TSAT.R77.esd 651 0 651 ESD GOOD: 102-321

GCACGAGGAAGTGGAGTCTGTGTAGCGAGACCAACCCATCTGCTCCTAAG

GAGCTTCGTGAGCACAGAGCACCGCCAAGTTGGCACCACTTACACAAGTG

AGAGTTGGCAAAGGTAGCACTGCCCTTTTGCTAAAGGGAAACAAACCCGG

TCTACACTTACGAAATTACTCTTGGTGGAACGAATGTTGCAACTAAGTGT

CTGAGAGGCGAAGGGTATTG

>TSAA.R48.esd 645 0 645 ESD GOOD: 95-513

GCACGAGGAATGGCTGCCCAAGCTTTCAAGCGCTTTTTGCCTCTCTTCGA

TCGCATTCTCGTGCAGCGCTTTGAGGCGACTTCCACCACGAAGGGTGGGA

TTATGCTTCCGGACAAGTCCAAGGGAAAGGTATTGGAAGCAACCGTTGTT

GCTGCTGGTCCAGGTCGTGTTACTGAGCACGGTACTACCGTACCTCTCTG

CGTCAAAGTCGGTGACAATGTTTTTCTTCCCGAGTACGGAGGTACCAAAG

TTGTTTTGGAGGAGAAGGAGTATTATCTCTTCCGTGAGTCGGACATCTTG

GCGAAAATACAAGATTAAGTTGTGTACAAACGATTGTCAATGCGAGTCTT

AGCATTCACTGTTGTAAATCTGCATTTTAGTAACATACTTCCGTACAATC

AAAAAAAAAAAAAAAAAAA

>TSAM.R62.esd 447 0 447 ESD GOOD: 94-396

GCACGAGGCTCCTATCACTTGGAAGCCGGGGAAGAACGTAACCGTTAAGT

TCCTGAGGAAGGTAAAGAAACACAAGAATCGGAAAGAGGTGCGCACAATT

ACAAAAACTGTGAAACAAGATTCATTCTTTAACTACTTTGATCCTCCAAA

TTGGAATCGCTAACCGATGAAGACTTAGATGAGGATACCGCAGAGTTACT

GCAGGAGGATTTTCGAATTGCCAACTTCATGCGTGATGTGTTGGTTCCCC

GGGCTGTTTTGTTTTTCACTGGTGAAGCTATTGAAAGTGATGGTGAAGGT

GAT

>TSBC.R56.esd 611 0 611 ESD GOOD: 94-610

GCACGAGGGATCGCTGATCCCATTCCACGCCCAACAACGCGTGTCCTTCG

CTCGACAATTTTAAATACCACCCCAACGACCATCATCAATGACACCGTAA

GTAACGGTGGAGGTGGGGACAGGCGTATCTCGCCGGCGACATCTCAGTTC

TCCCCATCCCCCTCCTCCGCCTCCAACTCCTCTATCGGCAAGCTGGGCGA

CCACCGGTACAACTGGCGCGAGAACCTAGACTACCGCCTTCTGTATGAAG

AGGAGCGAAGCGAGAAGGAGCGACTAAAGCGCACCCTTGAGCAGGTCAGT

CGTGAAGTGGCTGCACTGCGTAGCGAACTCGCCCGTCTTCACGTCTCCAA

CTCCATCTCCTCCGCCCCCTTTACTGCAAACAGGCACGGTAGCTCGACGT

CCAACACCGACACCTCCCCCGCTGCGGAAATAGATCGGCTGCGTCAGGAA

AATGCGCGCATGGCAGACGAGAACAAGTCGTTGATTCGAGTGATCAGTAA

GCTGTCGGCGCGCACNN

>TSCB.R50.esd 692 0 692 ESD GOOD: 97-629

GCACGAGGCTACATCGATAGTGTGCTCCAGCTTCATCGACGGCTTCTCTC

GTGCCTTGCAGTCTGTCCCGAAATGCCGCAGTACCAACCCTCGATTGCTT

CTACTCCTCAATCCAAAAAGTTGCGGTCTCCTCTTGCTCCTCCTCCGGCT

CCTCCACCTATTGCCAAACACTCCACAAAGCAAAAAAGTAAACATCAAAA

GTCGCCTATACCTCCACCACCGCCCGCATTTGAAGATCTCCCCATCCGTC

CACACGACCCGGTTGTGAGTAAACGCTCTAAGCATCGAAAACACCGACAG

AGCAGCCAATCCGATGAGACCGTAAGCTCAGAATTTAGTGAGGTGACGAA

GAAGAGAAAACACAGAGACTTGACTTCGCCATCAGGAAAGGTGCCTGAGC

CTCCTCCTCGCATGCATGGAAAACACAAGGTGGTGGAAGACGAGCGACCA

AGGAAGCACAATTCACGTAGCTCAAGTGCGGCTCCCTTGCGTAAAAATTT

TGATCGAGATGAGGGTGATGATACTGACGGCTC

>TSAW.R77.esd 698 0 698 ESD GOOD: 123-302

GCACGAGGACTCACTACTGTATAAATGTACATGCACTCCTTCCTTCCTTC

CTTTCTCCCTCCTTTCCGCATTTCACCCATCTCTCGCAAGTCTTTTTGTT

GTCTGCATTTCAGCATTAACAACAATTAATTTTACGCTGTTTGAAAAAAA

AAAAAAAAAAAAAACCTCGAGGGGGGGGCC

>TSBB.R43.esd 598 0 598 ESD GOOD: 96-571

GGCACGAGGGTCTCCTTCCCTGATCCGACTCAGCCTTTCGCTTGTTTGAT

CGACCTCCCCTGGCCGTCTTTTCCAGACGAACACTCTGGTCTCCCATGTG

GTGGAAACACTGACTCAGACGCTTCGCCTCCTACACCACACTGCGACCGC

ATCTCTCACCGCTTCACCGTCTTCTCGTGGAAGCAGTGACAGCAGAAAGG

TGGCCACGCCCTCCCCTCGCCCCGTGGTTACAACAGCCACCTCAACTTGC

AGCTGTTCATGTCGGCGTCGGTGCGGGCAGATGGCTGCCTCGGTACAGGA

GCTGACGGCTCTGTTGACTAACACTGGCAATGCGCTCTCTGGACGTGGCA

GTCACGAGACGTCGGACGAGGTGGTGGAGGTGGAGGGAGGGTCTTCCACC

TCGGCTTGACGACTTTAATCCATTTTGTCTGAGACGCTTTTTGTACTCCT

TGTGATTTTCGTCTTCCTTCTATGGC

>TSBT.R73.esd 764 0 764 ESD GOOD: 104-379

GCACGAGGGTTAATAGGCGTTTCCCAAAGGTTAATTCCTTGTTCATGTAG

TTGGCTTGCGCTCGATCTCTGTTTGTAAGCCTCTTCAACAGTTATAACTC

ACATTCTTTTCTGCAGTAATAAAGACTGTTTAGTGTCGTAAAAAAAAAAA

AAAAAAAAAAAAAAAAAAAAAAAAAAAAAAAAAAAAAAAAAAAAAAAAAA

AAAAAAAAAAAAAAAAAAAAAAAAAAAAAAAAAAAAAAAAAAAAAAAAAA

AAAAAAAAAAAAAAAAAAAAAAAAAA

>TSBM.R76.esd 799 0 799 ESD GOOD: 101-214

GCACGAGGGCTCGCTGTTGAAGGCGGGTCTTCACGCTCCGCTACCATTGA

ATCACCCTTCTGTGGAGCTACGCTAATTGCACCTTCGTGGCTTATCACAG

CAGCTCACTGTCTC

>TSAM.R40.esd 676 0 676 ESD GOOD: 110-379

TCGGCACGAGGCCTCGNTGCCGAATTNCGGCACGAGGACTACCTCATCTC

TATCTTGCCGACGGACCTTGCGCGGGTGCGTATGCTGCTGCGCAGTCTGA

GGCGCCTTGTTGCGAGTGAGAGAGAGGTGGTAGGGGTGGAAAGCAGGACT

GGTGGAGATGCGTGGCGTCTCGCGTTCAATCGCCTCCGCCTCACCATTGA

TTCCGCCTGCCGTCGTCACTATGACGCTGATGTGCTCGAAATTGACCTCG

ATTAGTTCCTCCGCCTTTTT

>TSAQ.R58.esd 674 0 674 ESD GOOD: 87-557

GCACGAGGGTGAAGTTGATCGATCTCTTGAACGAAGGGGTGGATCGCGTG

AAAGAGAAATTAGCTGAAAAGGGCAGGGATAAGGAGTTGACCCCGGAGGA

ACAGCAGATAGCAGCTGAGGCTATTGCCTATGGTTGCATTAAGTATGCTG

ATCTCTCGCACAACCGGATCAACGACTACGTCTTCTCGTTTGATAAGATG

TTGGATGACAAGGGCAACACAGCCGCCTACCTTCTCTATGCCTACACCCG

AATTCGGTCCATCATTCGAAAGACAGGCTGGAATGATGAAAAGCTTGCCG

AAGTGTGTAAAACTGCTCACGTCAACTTGGAGCATCCGGCTGAATTAAAG

CTGGGTATGATTCTGTGTCGTCTGCCAGAGATTATAATCAAACTGGAGGA

GGATCTGCTTTTCAACAAACTCTGCGACTACCTCTATGAAGTTAGCGGTG

TGTTCACGGGAGTTTTACGAT

>TSAN.R57.esd 700 0 700 ESD GOOD: 102-549

GCACGAGGGGTGCGTCGGTTTATCTCGCCATCTGGTGCACCTTCACCACC

AGTGGAGCCCAAATCAATCCTATGGTCACCCTAGCAGTCGTTCTCACCCG

TCGAATACCGCCACTCCTTGCTCCTGTCTACCTAGTGGCCGATTTCGTGG

GCACCTTGACTTTCATGGGGATAGCGTGGGCCATACTACGCCTTTCAAGG

ACCAGGCGCCGGGTATACTACGGAATGACGCTTCCCGCAAATGGAGTTAG

TCCCCTCGTAGCAACTTTTACAGAGGCTGTGTCCACCTTCATTCTTCTCA

TAGTCATCCTGGCCTCTTTGGACGAGTTACGTGCTAGAGAGTGGAGACCG

GAGAAAGGAGCTCCCTTTCCCCTCGCTGTCATGTTAGCTCTCACTGTCAA

TGTGGTGCTCACGTCGCCTGTTTCCGGAGCCAGTATGAATCCCACTAG

>TSAL.R49.esd 772 0 772 ESD GOOD: 113-293

GCACGAGGGCTTGCATGAACTTCCTTGCCTCATAACTCCAGAGAATGCTC

AGTTCGACAACCACCCACCCTCACTCTCAATTAATGCAGCAACCCTACTA

GATGATTGTTGAATGACAAATAAAACTTCCTTACCCCATAAAAAAAAAAA

AAAAAAAAAAAAAAAAAAAAAAAAAAAAAAA

>TSBR.R86.esd 735 0 735 ESD GOOD: 104-672

GCACGAGGTGCAAGTCGAACTCTGCAATCACTTGAGTCTTCCAACGCGTG

CCATCCTACTTGATTCCTCTCCCTGGTATGCTGAGATGTATTTCAGTAGT

CTGTCTGTCACCTACCAACCTGGAATAAATGACTCTCCGATCAACATGGC

GCCAGATAAGGTGATATTCCAGCCCAGCGTTCAGCTAGAGCAGATGGCCT

TTTTGGAGCTTCTGCTCACACTTCCCTCGAGAGCTTGTGTTACTGTTTCT

TATGCCTTCAAGAAGATTCTCATGCATTGGAACGAGTACCTCCCCGATGC

GAACCACGGAATCTTCTTGCCTGCTGCCACTGTCATTTACCAGCTTTCTC

CCGACCAACTCGACCGACAGCGGTCTGGCAGTAGCCCTATCTTCTGGGAG

TTGGCGTTACCTCAATGGGCCAGTACTTACGCTGAATACCTTTCTACCAT

GAATCGCACGGAGCTCGGAATGGGCGAGGGTTTCGTGCGGCTATACTCTG

AGACGCCACTCGTTCGCCTCCCCGTGCCCGACTTTAGCATGCCGTTCAAC

GCCCTCTGCTTGGTCTGTT

>TSAU.R18.esd 393 0 393 ESD GOOD: 104-210

GCACGAGGATCACACTTCTTCGTCTTCTTCATCTATTTGCCCCTCCTGGT

GGTCAGTCTCGCCATTTGGACCAACGCCANCTCCTCGANNACCCAACATT

CACACTC

>TSBA.R90.esd 658 0 658 ESD GOOD: 104-583

GCACGAGGGCCATGCAGAGGGCTTGTTCCAACCTTAGACCCAACTACGAG

CCGGGAATAACGTTTATTGTAGTGCAGAAACGCCACCACATCCGATTTAA

TCCGTTGGTGAGGGGGGCGAAAAACGTTCTGCCGGGCACAGTTGTTGACA

CAGAAATCACTCACCACCGAGAATTCGATTTCTATCTTTGTTCGCAGGAT

GGGATCCAGGGGACCTCAAAACCTGCTCATTATCATGTTCTTTATGATGA

CAACGATTGGGNGTGGCGTTGATCTGCAACAGTTCACGTATTGTCTGTGC

CATGCGTAACATGCGATGCTGCCGCAGTGGTGTCTTATCCGGCACCGACG

TACTTACTCGCATCTGGCCGCCTTCCGTGGCACGCGATTGGCTAAAAATC

TCGGAACTGAGACTATTTAATACGAAAACGCTTCACCATTCATCCGGGCC

ACAAGACCAATGTTCTTTTTATGATACCTT

>TSBT.R89.esd 799 0 799 ESD GOOD: 95-233

GCACGAGGCTCTTTAGGAGTTCTCCATCTGAGGACTTCGATAGAAAATAC

TGCTGCTAGGTGTCTTGTAATGGAGTCCAGAAACGTGTTTAATGTTGATA

GCAAAATCACTTCCAAGAAGAGAAAGTTTGCTCCGAACA

>TSBT.R65.esd 763 0 763 ESD GOOD: 107-494

GCACGAGGGACACTCCTAACCGCCTCCTCTGAATTGGCCAACAAGATGCA

GTCCTCTCCTCCTCCCATCCCCATCCTTCTTCCCCTTTTCTTTCCTCCTT

CTGTCTCAATTATCACCAATTCCTGCAAGCATACTACGTACACTCAGTCT

GCGTTTGTTGGTTTGTTTGTTTGGTTGTTTTATGTCCATGCTGTCCTTAT

GAAGTGAACCAAACCATCCATGTAAGTTCCGTCACGACGACACGCGTTAC

TTTATGCTTTGTTTTGCGACTCCTTTTGCGTTATTTGTACATTGCCATGC

CGCGTTCTCCTCCCTCTATCTCTACTTGTATCAACAGATGTCTCTCTGTC

TGTCTCTCTCTCTCTCCTATGCATTCAACCAGCCATCC

>TSAM.R32.esd 612 0 612 ESD GOOD: 85-528

NCGGCACGAGGGTAGAGTTCCANCGAGATTCTGCTCTCCAACTTCTTGGC

TCAGACGGAAGCTCTGATGCTAGGTAAGACACCGGAGACGGCGAGGAAGG

AGCTGGAAGCCCAGGGCGTGTCTGGTGACAAGTTGGAGATGTTAGTAAAA

CACAAGACCTTCCGTGGTAACAAACCCACCAACTCCATTATCTTCACTGA

GCTCAATCCCTTCATGCTGGGTGTCATCGTAGCGATGTACGAGCACAAAA

TCTTCACTCAAGGCGCCATTTGGAACATCAACTCCTACGATCAGTGGGGC

GTTGAGTTGGGCAAGGAGCTGGCTAAGAAGCTGCAGCCTGAACTTCACTC

AGGATGATGTGGTGACCTCCCATGACGGATCAACCAACGGTTTGATTGCC

TTCATCAAGCACAACAGAAGAGTTCATTAGTGTTAATATACGCT

>TSAK.R28.esd 606 0 606 ESD GOOD: 95-322

GCACGAGGGAATTTATGCCACCTTTTTCAACGTTCCAGACTCAAACTCAC

CCAATTTTCAAGATGAGTCCAGTGGACTGACAAACTCAAGGGGTGGATTA

ACCAATCAATCTCCCTGAGAGAGGCTCCATGTAAAATAAGGTCAATTTAT

GTACAAACTTATACATTTTTACTAATAGAACGTTGTTTCTGTAAAAAAAA

AAAAAAAAAAAAAAAAAAAAAAAAAAAA

>TSBT.R96.esd 729 0 729 ESD GOOD: 119-558

GCACGAGGCCATCGGCTTTACCTCTGAACACATTGAAACCCTCTACGAGT

TGGACCTTGAGTACTGCTCCGACTTGGCTAGGGAGCTTGGGATGGTCAGC

GTCCGACGTGCCTCTGCCCCAAATGATCATCCTCTCTTTATTCAGGGGAT

GGCGAATTTGGTTACAAGACACCTGCGTAGTGGGGAGGTGGCTTCGCGAC

AGTTCTTTCTGCGTTGTCCAGGCTGTACCAACGACCGCTGCTCAAAGGTG

CGGCATTTCATCGCTGGCGAGGCAGAGCGTCTACAGACATGGACTAGATC

TCAGGCCTTCCGTGACATCGCTGCCACAATCACCACCTATAACAAGGTCC

TCTAGCTACCTCCTCTATAGCACCCTTGCTCGTAGATCCCTTAGGCAGTT

TCGATAAACTATTGTATTTTTAAAAAAAAAAAAAAAAAAA

>TSBU.R50.esd 844 0 844 ESD GOOD: 97-627

GCACGAGGCTCATCTATCTCAGCGGTTGAATAAATTGGAGGTGGATAGGT

TGATTCGCAGACATGGAGATGGACCCTAGGCTAGCATGAACGTGTGGATG

TGCTTCCTCAGGTGCCACTTGCCTCATCATGATGCATCTTGTTTTTTGAG

TGTGGAGATGCAGCGAATGCCCTCTACCTGCATCAAAGTGCTATGCCTTT

TATGTGTCATCGAAAGTAGCAGGGACGACCCCTTCTAAATTATCGATTAG

ACTGGCACAATCGTTGATTGGCTGTTTTAAGTAAGGAATGGGGATGCTGT

TGGGCATTACGGGATGTAGAGGAAGAGTCTCAGGCCGATATCTCATCGGA

TAAAACTATCTGGTCTTCTCCTTTCCTCTCCGTCTTCCCCACTGCCATTG

CCTCAATGCGGCAGCATTATGCGTACCTGCGTTACTGTATTATCATCATT

TAGTGTATATGTGCACTGATACCGCCCTTGTTGAACAAATTCTCACCACC

GAGGTGCGCACACACCCACGAAAAAAGGGGG

>TSAP.R91.esd 757 0 757 ESD GOOD: 93-586

GCACGAGGCTCGAAAGCGTTTTCGCGCAGTTGGTCTGCTTGCAAAAATGT

CGGTCCTTACAATGACTCAACGTATAGAAGCGGAAAGGGAAGGCCGAAAA

CTTGGCGAAATCAAGGAACTAGATTTGGAAGGTTGTTACGCCACTGAGGT

TGAAGGCTTGACAGACGAATACACGTGCTTGGAGAAACTCAACATGTCTA

ACGTCGGTTTAACAACCCTTGCGGGACTTCCAGCTTTGCCGGCATTGACG

CATCTGGATGTGTCAGGTAATCCTATTTCCGAGGGTCTTGAGGCTTTGAA

GAGCTGTCCGAGTTTAATATCCCTCAATCTCTCTGGCAATAAGCTGGCAG

CTATCGCCGACCTGGCTCCTCTTGCTTCGTTAGAGAATCTGTCTTGTCTT

GAGATGGGCGATTGTGACTTGGTGAGTGTAGGTGGCTACCGGAAGGAGGT

CTTCTCCATCTTGCCACAGTTGAAGGTTCTTGACGGTTTGGACC

>TSAQ.R55.esd 706 0 706 ESD GOOD: 86-570

ATTCGGCACGAGGCAGCGCCTTATTGCCTTATCGAGTGCCTTTGCTTTAT

GTGTAAACTACTACCTAACGCCTTTGACATGCAACGATACCCAATCAGGT

GCCGTCATTGCCTTGCCCACAACCACCGCTTTTGCGGGCATACTTTTTAA

TGCAGTCTCTCAAAGCCGTTACAGCCAAGTGAGACATTGCATCATGAGAC

ATCACTTCGCAATTCGTCGTCCCAAGGTGATGGAGTGAAGAATCAAGAAT

ACGCCTGGTTGTCCGTTAGTAGTGAAACTCCTTGCAGTCGCGCGTTTAAA

GACGATGAGTGGTTAAAGAGATTATCCCACAAGGTTCCTAATCGGCCAGT

GTCTATGGCTGCAGAGTATCCGCTGTCATCGACAGCCATTCCCATTCGGC

CTTCATTTGCCACTGAAAAGGTGACCCCACCGGCTTCAAGACTGGTGGAA

GTCGAAGATGGTGGTGGGAAGGAAATTCGACTGTC

>TSAC.R16.esd 608 0 608 ESD GOOD: 96-521

GCACGAGGGTTCATCAGAGACCAATTGACCCCTAACCAATGGAAGTTCTA

CCTCTTCTCAGAGGAGAAACTGAATCCATATAAACAAGCTAGAATAACCA

AAAAGAACGAGCGCAAGGATAGGCCTTCCAACGGTGTTCCGTCGCCACAG

GTGAATCTCACGCCTTTTTTTCATCCTAACGACGTCAATGGTATCTCAAC

CCCTTCTAGTGTGGTATCCAATCTTCGGCCTCGTGGTGGTTTCCCTGTAA

ACGGTGCCGGGTGTCAATCGCGCTGCTGCTCCGTTTGAATCGTGCACGCA

CGTCCAACCACCACCATCAGCAGCAAGCAAGAACAATAAATAACGAGATC

TCAACATGTGGGGGCATCAGCACCCGTCTCTCGAGGAGGTAGTGTTTGGC

CACCGTGGGGTGCTGGAGCTATCCGC

>TSBD.R56.esd 528 0 528 ESD GOOD: 95-456

GCACGAGGGCCGAGGTGGGGAAAGTCTATGAAAATGCGCCTACTCCACCG

CAGGCGTCATTGCGCGTGGACACCGGAGGAGCCGGGAAGAAGAAACTCAA

AATGATCTTGGTGGATGGACAACAATTCAGCAATCGGATGAGGAGTTGGT

GGAGGAAAGGAATGTAGGACCTCAGACGGATCCTGTGCCATTACCGGAGA

CCATACAAGTTGGCAGAGTGCGTGAATTCAATGTGGATTTGGATCAAGAA

GATTAGATGGAGCCGCCACGCTTCAGTGATTATGTTATTCGTCATTTTCT

CCCGTTTGTAACAAAAGAAATATAGTGTCTTAGTCCGAAAAAAAAAAAAA

AAAAAAAAAAAA

>TSBU.R74.esd 494 0 494 ESD GOOD: 107-348

GCACGAGGCGCAATTGAAGGTCATTCAGTTTCTCTTCGCTCAAAGGCATA

ATTATTTCTGCTCGGCGTGTCACTTCGTGTGTGGCTGGAACTGCGTGAAT

CCAGTGGGATCAGAAGATCAATGTCTGTGACCTTGAGACCACGTGGTGAG

GCGCCCATGGGACGTTGAGACCTCGGTGAGCGACTTGGACCACCAAAGAG

TCCACGAACACACATAATTTGTCCGTCTTTTGCTGGTTGACT

>TSBA.R77.esd 594 0 594 ESD GOOD: 103-546

GCACGAGGGTTATTCTACGCGTTTCCAAATGACCAACAAGATGGAGGTGG

AGGGTGGAGCAGATTCGTATCGACGCTCATGTCCTTCTATGCTACTCAAT

TCTGCATTGATCTCTTCATTGGAAATTTTCGTTGAGGCCGGTGGAATGAT

TCCCATCCGCAAAAAGTCAATTAATGCTGACGAATTATCTACAATCTCTT

CTCATAAATAGCCCTATTGCTAAATCTTCAGGGTTTAGAGGTTATTACCC

CCGATTGATCCACTTATCTAAAGGCTCTCAACTTTCCATTAAAGTCAAAG

TCGNAATGTTGAGATTTTGACGATTCCGATTGATTCAAAAGAGGTGTCCA

TTTGTGGACACCCGAAAAAGCCTTGCTAACATTCGGTCTTCAGCGCCCTT

TTTGGGTCTCTTAACACCTTCCTTTTTGCTGATTCTTCCTTTTA

>TSBU.R59.esd 680 0 680 ESD GOOD: 98-606

GCACGAGGCCTCGTGCCGAATCGGCACGAGGTCGACTACCATCGACGCAC

TAGCATCTCCCGCGACAGTTTTCTTCTCCTCGTCGATAGACAGCCCTCCA

GGATGATGAGCATGGCTCGCGGGGACCAACAGTCTCAGATGGTGCAGCGG

GTAATCGCACGCTTTGATTTTAACGCCTCGGAGCCGGAGGAGTTGTCATT

CCATCGGGGTGACGTGATCGAAGTATTGGGTCAGGAGGACGAAAATTGGT

GGCGCGGACGTATCTCCAGTACCGGCTCCACCGGCCTCTTTCCTGCCAAC

TACGTGGACACCCTACCCCCCATTATGCCCAACGTTGCTCACCGCCCCGC

CAACTAGGCTACTGCCACCAATTTCGAAATTGCCAGTCTGACTTCCCACT

CTCTTTCCTCACCCCCACTTTCTTGTGATGCTGACCACGTCGGCACGGGA

CTATTGACTCACTTCTGATCCCTCATCTCATCTCCTTTGTTTGATCGATA

ATGCTCTTG

>TSAL.R95.esd 758 0 758 ESD GOOD: 111-510

GCACGAGGGTTGTCTCCAGTTTTTACCTAGTCCGTTCCCATTGCGTGCGT

ACTTCGTGATATTGTCCTCAAGTCATTGCGATCGTCAGTTACGGAGCGAC

AGAACGAGATGAACTCGATGTCAAGGAAGGCGACTTGATGAACGTTATCG

TAGAATTGTCAGACGGCGCACCTTTTACCCACCGGGAGTAGAAACCTCCA

AGCTTTACACCGATTCAATTGTAGTTGAAAAGTGTCCTGGAGCGTCTCCC

GCAGAGACACCTCGCCTTTACTATTTCGATGGAAACTTCTTTTCCCTTGT

AGCAAACAATTGTTTATGTCATTCAACTCGCCAAATTTTTCTGCCATTAA

AATTCTGATTTGACCCTGGAGAAAAAAAAAAAAAAAAAAAAAAAAAAAAA

>TSBQ.R6.esd 929 0 929 ESD GOOD: 98-563

GCACGAGGCCATCACCACCACGTGGTGGTAGGCAGCAATGTGGAGGTGGT

CAGTTCTTCCACCACCCCTGCTGTTCCCATTTCTGTCGCCCCAGCGTCCA

ATATAGCATCGCCGACTCAAGTTTTGGGTCATCCGCGTCGTGTTGTCATC

GTGTCGGCGCAGCCCCATCAGGAGTGCCCACCGCCTCCATCCCAGGCTGC

CCCCATCGTTTCATCGAAACAGCCACCTCACTAGCGCTTCGCCTCTTCCG

CCTCATTCCCCTTCTCTCTTCATGCTCCCAACTCCACTCCCATCCTCTCC

TGTCCAGCGACCCTTATAAGGTCTTTGTAGTTGTTTCGATCTCAATTAAG

CATAGGGATTTNCTGTTTCTGGTGGGTCCACACTCGTTCTAACGTTATCT

TCCACTTTTCCCCCTTGTTACATTATTTGTAATTCCTAANATATTGGATT

TGTTACTTTCTTTTTC

>TSCC.R4.esd 557 0 557 ESD GOOD: 97-450

GCACGAGGCCCAGGTCGACTGCTATTCGGTGGATACAACGATCATTCTCT

CAATATTTGGGACACTTTAAAAGGGAAGAGAGTGTCCATTCTCTACGGTC

ACGAAAATCGTGTCTCAGCACTTCGTACCAGCCCTGATGGCAATGCTATC

TGCACTGGAAGTTGGGACAGCACGCTGCGCGTCTGGGCATAGAGTACTGT

GGAGGAGCCATCCCAAGGACGTACCTGCGGCGACCTTCCACTTCCTTCAC

TAACTGCCATTTAACTACCCTACAAGTTACTTGTCCCATGCAAAATGGAT

GCTGATACATTAACCTAAAATTGCACCACTAAAAAAAAAAAAAAAAAAAA

AAAA

>TSBG.R11.esd 782 0 782 ESD GOOD: 103-459

CCCCGGGCTGCAGGAATTCGGCACGCGGAGCTAATCTCAGTCTCTTCTCG

ATGTGTTCCGTAACACGCAGATTTGACTGCAAGAGCAAGAGAAGTGGTGA

CTTGAGTGACTGATGTTTACATCTTTACCGAGTGCAAATTGGATGGAAGA

GCTGCTCAATCCAAAGCTCTTTTTTGCTGCAACGATGCAGCAGACGTCGC

GAATGAACGAGTAACGAAGAGGCCGAGTGGGGTAGCATGTGTAGTTGACG

AGGAAGATATGGGATAGCCTGTTCGTACAAGGCGCACGGTGGAACAGGCA

GACTGGTCAGCTAGCGGAGGCGATGCCTGAGGAGTTGGTCGGGTGACAAA

CAGGACA

>TSAQ.R3.esd 658 0 658 ESD GOOD: 95-558

GCACGAGGGCGGAAGTGGTTCGACTGGCCCGTACTTTCACCAATGACATT

ACGCTGGCAATTGGCGACGGGGCGAACGACGTCGGCATGATTCAGGCAGC

ACACATTGGAGTGGGTATCTACGGCGTGGAAGGTCGTCAGGCGGCTTGCT

CCTCAGACTACGCCATTGCGCAATTTCGCTTTCTCAACAAACTTCTCCTA

ATTCCACGGAGCTTGGAGTTTCAACCGCATCACCAAGCATGGTCTCCGGC

TTCTCGGGACAGATCATCTTTGATAGGTGGAGCATGGCCCTCTACAATGT

GGCTTTCAGCGCTGCACCACCACTGGCTCTCGGTTGTGTGGAGCGAAATA

TGATTGCTAAGAATGGCCTANAAGTGCCCGTCTTATACAAGGAAACGCAA

AGAACCACCACTTTCGACAAAGTGACCTTCCTCACGTGGTCTGGTGAACG

GAGTCTTCCATTCA

>TSBE.R78.esd 603 0 603 ESD GOOD: 96-291

GCACGAGGCTTGTCAAACAAGCTCGTAAATCCGCGCAGGCTTATCAGCTG

GCCTATGATGAGTCCATTTCTCCTGAGCAATTAGTTACGCGCATCGCCGC

TGTCATGCAGGAGTACACTCAGTCTGGAGGGGTACGACCATTTGGCGTGT

CTCTGCTTATTGCTGGGTTGGGGACTACCATTCCAATTCGAGCCTA

>TSBB.R30.esd 763 0 763 ESD GOOD: 67-267

CCCGGGCTGCAGGAATTCGGCACGAGGGCCAGTCGTTGACGATTTCAATC

AGGTCCGGGGAATGATCACACGCAAAGATCTCTGCCGATTTCGTTTTGCA

CCGGCCGGTAAAGTTGCCGAACGAGTATTCTCTCGCATTTTTTAAAATAT

TTCCCCCCTCCCACACAAACGCACATTCGCAATGTTCAATCTCTCCTAAT

T

>TSCG.R94.esd 661 0 661 ESD GOOD: 96-586

GCACGAGGCTGACCTCCAAACTTGACTCAGATGACGACCAGCCACTGGTG

GACCCTCAGGACACGTTAGGAGACGAGGACGAGGATGATGAGGACACTTC

TGAGTCATCCTCTTCTGATGAAGCTCTTCCCCCAATTTCTGACCACCACG

CCAAACAGATAGCAGTGCTAAAAACTCGTCTTTCCCAATTGGAGAAGGAG

ATGCAGGAGTTGGAAATACAAGGTGGGGCAAAGGAGCGTGCTCTAAGACA

GTTGGAGGAGGGAGAGGGGGAGGAGGTCATGGGACAGCTAGAAGAGCATT

CCATGACCCAGGTCTCCTCCACCAGTGAGCCAGACCTCTTGCAGACCGCC

AACCAGTCGTCCAAACGGGGACCAAATTCACGGTGGAGGTGGGTGCGGTC

TAAGCGGTCCGTCCATAGACAGAACAGTGGCATAGCGGTCTCACGTCCAC

TCACCTCGGAGCAAGAGATGGCTGAGCGTTCGAAGAGGGAG

>TSAM.R13.esd 724 0 724 ESD GOOD: 95-415

GCACGAGGGTCGTCTCTTGATGCGTTGTCCATCTGTGTGCAACCTCCATT

GTTGCTTTCCATTTCTGTGTCTTCCGAAGGAGTTTGCCTTCAATGGGAAC

TTGACTCTGCGGAATTTTCATTCGAACCGGCAGCATTCTACGAATTATAC

AGCTACGCGAGCTCTGATGTTGACCCTCGTTTTCCACTCTCCAGCGACGT

ATGGCAAAAAGTCGGCAGCGTTGAGGCCTATGCCACTGCCGATGGCCTGC

ACTCTGACAAGTGTGCAACCGCAGAATATCTACTACTATCGCTGTACGCT

ACCATTGATCGGTTCCAACGC

>TSBO.R18.esd 754 0 754 ESD GOOD: 97-314

GCACGAGGATGGTCACTTGTATAGAACTAATGTATTATAAATGTAAATTG

TTTAACTCCCAAATCCCATCGCCCGACTCTCGACCCCATCTTTGTCACCT

GTGACAGTCTGATGCACAAAACAAAGTGTGCATAAGCTCGCTTTACACTC

CAGTGAGCTTCAGTGATTACCTGTGTGTATACATAACTTTACGTTTTTAA

AAAAAAAAAAAAAAAAAA

>TSBU.R35.esd 789 0 789 ESD GOOD: 89-673

GCACGAGGATTTTACTGCCTGCTACGCGAAAATTGCCTTTCCATAGCAAC

TATAAAAGCTGCCAAACAGGGCATAATGCAAGATGCTTTGGTGCTATTAG

CTTATGAAGCAAGAAGTCGCTGCACTTTTTCCAGGCGCATCTCGCCTAAG

GCCTTCACTTGTAGATTTCAATGCTCCTGAGGGCTCATGGTCATTGCCGG

TTTTATTTGTTGACGGGAATTTGCGTGTCACAGCTCTTTAGAGACATCAT
[truncated: 426,019 more chars]
