# Supplementary material for: Analyses of an Expressed Sequence Tag Library from Taenia solium, Cysticerca
Source: PLoS Negl Trop Dis. 2010 Dec 21;4(12):e919. doi: 10.1371/journal.pntd.0000919 (PMC3006133; doi:10.1371/journal.pntd.0000919)
Supplement: Figure S2 — Metabolic pathways and their mapped ESTs according to KEGG within the Blast2GO program. (2.00 MB PDF) [file pntd.0000919.s002.pdf]

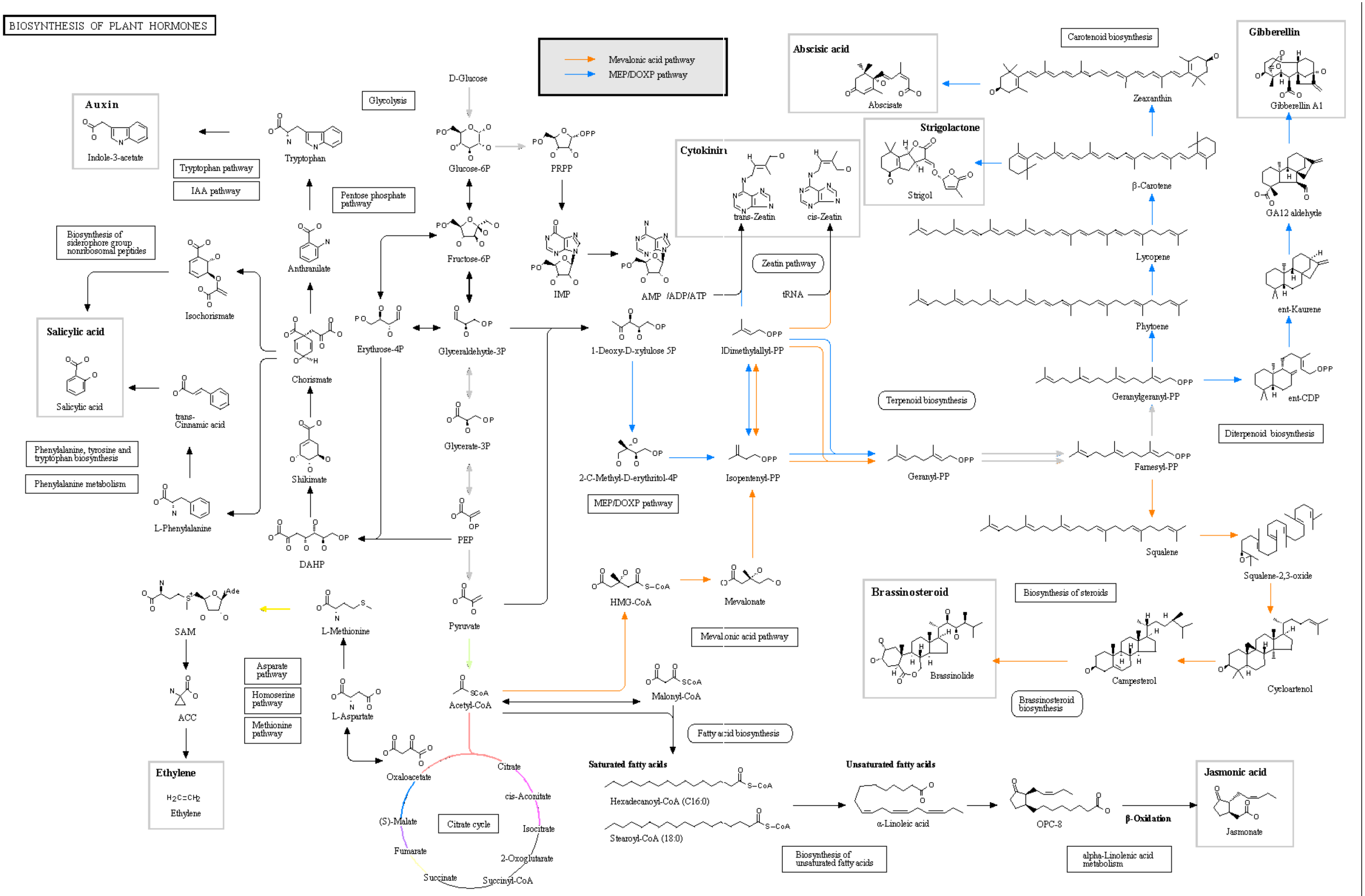

BIOSYNTHESIS OF PLANT HORMONES
Mevalonic acid pathway
MEP/DOXP pathway
Auxin
Indole-3-acetate
Tryptophan pathway
IAA pathway
Tryptophan
Glycolysis
D-Glucose
Glucose-6P
PRPP
Pentose phosphate pathway
Anthranilate
Biosynthesis of siderophore group nonribosomal peptides
Isochorismate
Fructose-6P
IMP
AMP /ADP/ATP
tRNA
Zeatin pathway
Cytokinin
trans-Zeatin
cis-Zeatin
Abscisic acid
Abscisate
Carotenoid biosynthesis
Zeaxanthin
Gibberellin
Gibberellin A1
Strigolactone
Strigol
β-Carotene
GA12 aldehyde
Lycopene
ent-Kaurene
Phytoene
Salicylic acid
Salicylic acid
Chorismate
Erythrose-4P
Glyceraldehyde-3P
1-Deoxy-D-xylulose 5P
Dimethylallyl-PP
Geranylgeranyl-PP
ent-CDP
Terpenoid biosynthesis
Diterpenoid biosynthesis
trans-Cinnamic acid
Shikimate
Glycerate-3P
2-C-Methyl-D-erythritol-4P
Isopentenyl-PP
Geranyl-PP
Farnesyl-PP
Phenylalanine, tyrosine and tryptophan biosynthesis
Phenylalanine metabolism
MEP/DOXP pathway
L-Phenylalanine
DAHP
PEP
Squalene
Squalene-2,3-oxide
HMG-CoA
Mevalonate
Brassinosteroid
Biosynthesis of steroids
SAM
L-Methionine
Pyruvate
Mevalonic acid pathway
Brassinolide
Campesterol
Cycloartenol
Asparate pathway
Homoserine pathway
Methionine pathway
ACC
L-Aspartate
Acetyl-CoA
Malonyl-CoA
Brassinosteroid biosynthesis
Fatty acid biosynthesis
Ethylene
$H_2C=CH_2$
Ethylene
Oxaloacetate
Citrate
cis-Aconitate
Citrate cycle
(S)-Malate
Isocitrate
Fumarate
2-Oxoglutarate
Succinate
Succinyl-CoA
Saturated fatty acids
Hexadecanoyl-CoA (C16:0)
Stearoyl-CoA (18:0)
Unsaturated fatty acids
α-Linoleic acid
Biosynthesis of unsaturated fatty acids
OPC-8
β-Oxidation
alpha-Linolenic acid metabolism
Jasmonic acid
Jasmonate

Biosynthesis of plant hormones

Colour	Enzyme	EST clone
red	ec:4.1.2.13 - fructose-bisphosphate aldolase	TSAY.R35.esd
yellow	ec:2.5.1.6 - methionine adenosyltransferase	TSAT.R84.esd
orange	ec:1.1.1.44 - phosphogluconate dehydrogenase (decarboxylating)	TSBR.R51.esd
green	ec:4.2.1.11 - phosphopyruvate hydratase	TS.seq.screen.trim.Contig231, TS.seq.screen.trim.Contig433
blue	ec:1.1.1.37 - malate dehydrogenase	TS.seq.screen.trim.Contig383, TSCB.R83.esd
pink	ec:4.2.1.3 - aconitate hydratase	TSAP.R16.esd
violet	ec:4.2.1.2 - fumarate hydratase	TSAS.R96.esd
light-red	ec:2.3.3.1 - citrate (Si)-synthase	TS.seq.screen.trim.Contig330
light-green	ec:2.3.1.12 - dihydrolipoyllysine-residue acetyltransferase	TS.seq.screen.trim.Contig176
light-yellow	ec:1.3.5.1 - succinate dehydrogenase (ubiquinone)	TS.seq.screen.trim.Contig279, TS.seq.screen.trim.Contig370, TSCF.R66.esd
gray	ec:2.7.6.1 - ribose-phosphate diphosphokinase	TSCH.R19.esd
gray	ec:2.5.1.29 - farnesyltranstransferase	TS.seq.screen.trim.Contig110
gray	ec:2.7.1.40 - pyruvate kinase	TS.seq.screen.trim.Contig291, TS.seq.screen.trim.Contig452
gray	ec:5.4.2.1 - phosphoglycerate mutase	TSAP.R46.esd
gray	ec:2.7.1.1 - hexokinase	TS.seq.screen.trim.Contig224
gray	ec:1.2.1.12 - glyceraldehyde-3-phosphate dehydrogenase (phosphorylating)	TS.seq.screen.trim.Contig479

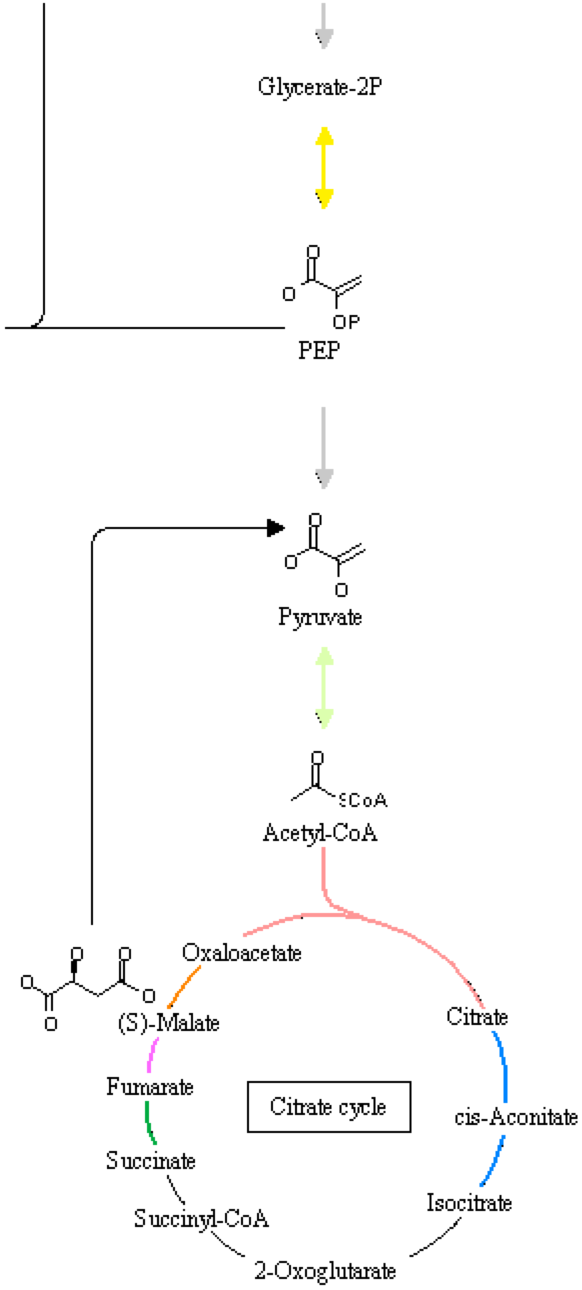

BIOSYNTHESIS OF PHENYLPROPANOIDS

**Colour**	**Enzyme**	**EST clone**
red	ec:4.1.2.13 - fructose-bisphosphate aldolase	TSAY.R35.esd
yellow	ec:4.2.1.11 - phosphopyruvate hydratase	TS.seq.screen.trim.Contig231, TS.seq.screen.trim.Contig433
orange	ec:1.1.1.37 - malate dehydrogenase	TS.seq.screen.trim.Contig383, TSCB.R83.esd
green	ec:1.3.99.1 - succinate dehydrogenase	TS.seq.screen.trim.Contig279
blue	ec:4.2.1.3 - aconitate hydratase	TSAP.R16.esd
pink	ec:4.2.1.2 - fumarate hydratase	TSAS.R96.esd
violet	ec:1.11.1.7 - peroxidase	TS.seq.screen.trim.Contig503, TSAG.R41.esd, TSAM.R92.esd
light-red	ec:2.3.3.1 - citrate (Si)-synthase	TS.seq.screen.trim.Contig330
light-green	ec:2.3.1.12 - dihydrolipoyllysine-residue acetyltransferase	TS.seq.screen.trim.Contig176
light-yellow	ec:1.3.5.1 - succinate dehydrogenase (ubiquinone)	TS.seq.screen.trim.Contig279, TS.seq.screen.trim.Contig370, TSCF.R66.esd
gray	ec:2.7.1.40 - pyruvate kinase	TS.seq.screen.trim.Contig291, TS.seq.screen.trim.Contig452
gray	ec:5.4.2.1 - phosphoglycerate mutase	TSAP.R46.esd
gray	ec:2.7.1.1 - hexokinase	TS.seq.screen.trim.Contig224
gray	ec:1.2.1.12 - glyceraldehyde-3-phosphate dehydrogenase (phosphorylating)	TS.seq.screen.trim.Contig479

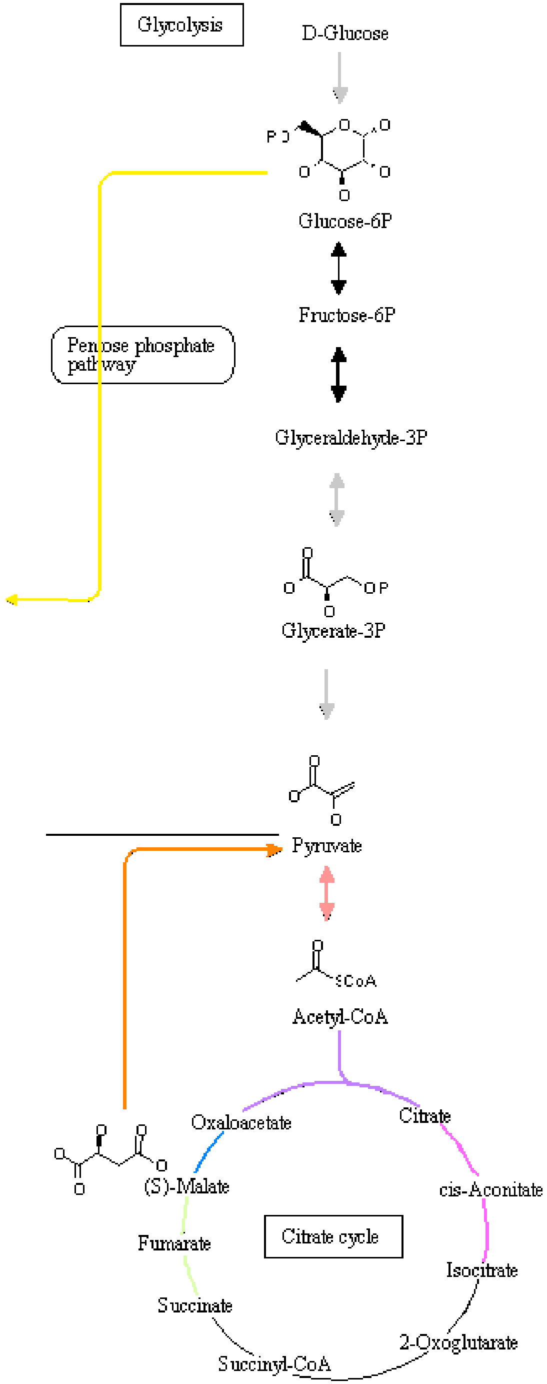

BIOSYNTHESIS OF ALKALOIDS DERIVED FROM HISTIDINE AND PURINE

Colour	Enzyme	EST clone
red	ec:4.1.2.13 - fructose-bisphosphate aldolase	TSAY.R35.esd
yellow	ec:1.1.1.44 - phosphogluconate dehydrogenase (decarboxylating)	TSBR.R51.esd
orange	ec:1.1.1.40 - malate dehydrogenase (oxaloacetate-decarboxylating) (NADP+)	TS.seq.screen.trim.Contig443
green	ec:4.2.1.11 - phosphopyruvate hydratase	TS.seq.screen.trim.Contig231, TS.seq.screen.trim.Contig433
blue	ec:1.1.1.37 - malate dehydrogenase	TS.seq.screen.trim.Contig383, TSCB.R83.esd
pink	ec:4.2.1.3 - aconitate hydratase	TSAP.R16.esd
violet	ec:2.3.3.1 - citrate (Si)-synthase	TS.seq.screen.trim.Contig330
light-red	ec:2.3.1.12 - dihydrolipoyllysine-residue acetyltransferase	TS.seq.screen.trim.Contig176
light-green	ec:1.3.5.1 - succinate dehydrogenase (ubiquinone)	TS.seq.screen.trim.Contig279, TS.seq.screen.trim.Contig370, TSCF.R66.esd
light-yellow	ec:2.7.6.1 - ribose-phosphate diphosphokinase	TSCH.R19.esd
gray	ec:6.3.5.2 - GMP synthase (glutamine-hydrolysing)	TS.seq.screen.trim.Contig177
gray	ec:2.7.1.40 - pyruvate kinase	TS.seq.screen.trim.Contig291, TS.seq.screen.trim.Contig452
gray	ec:5.4.2.1 - phosphoglycerate mutase	TSAP.R46.esd
gray	ec:2.7.1.1 - hexokinase	TS.seq.screen.trim.Contig224
gray	ec:1.2.1.12 - glyceraldehyde-3-phosphate dehydrogenase (phosphorylating)	TS.seq.screen.trim.Contig479

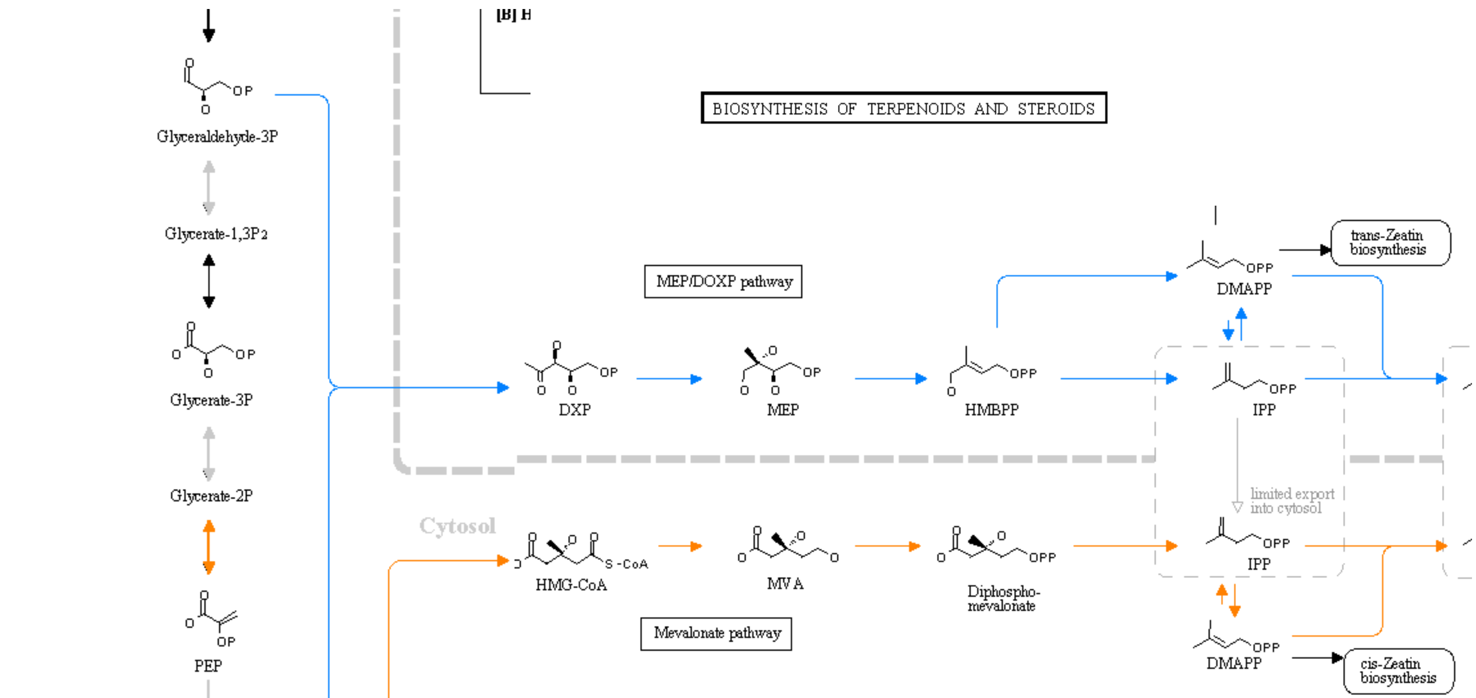

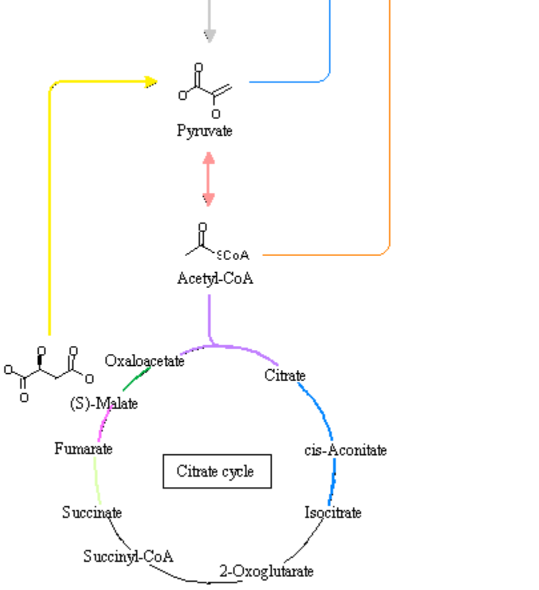

Colour	Enzyme	EST clone
red	ec:4.1.2.13 - fructose-bisphosphate aldolase	TSAY.R35.esd
yellow	ec:1.1.1.40 - malate dehydrogenase (oxaloacetate-decarboxylating) (NADP+)	TS.seq.screen.trim.Contig443
orange	ec:4.2.1.11 - phosphopyruvate hydratase	TS.seq.screen.trim.Contig231, TS.seq.screen.trim.Contig433
green	ec:1.1.1.37 - malate dehydrogenase	TS.seq.screen.trim.Contig383, TSCB.R83.esd
blue	ec:4.2.1.3 - aconitate hydratase	TSAP.R16.esd
pink	ec:4.2.1.2 - fumarate hydratase	TSAS.R96.esd
violet	ec:2.3.3.1 - citrate (Si)-synthase	TS.seq.screen.trim.Contig330
light-red	ec:2.3.1.12 - dihydrolipoyllysine-residue acetyltransferase	TS.seq.screen.trim.Contig176
light-green	ec:1.3.5.1 - succinate dehydrogenase (ubiquinone)	TS.seq.screen.trim.Contig279, TS.seq.screen.trim.Contig370, TSCF.R66.esd
light-yellow	ec:2.5.1.29 - farnesyltranstransferase	TS.seq.screen.trim.Contig110
gray	ec:2.7.1.40 - pyruvate kinase	TS.seq.screen.trim.Contig291, TS.seq.screen.trim.Contig452
gray	ec:5.4.2.1 - phosphoglycerate mutase	TSAP.R46.esd
gray	ec:2.7.1.1 - hexokinase	TS.seq.screen.trim.Contig224
gray	ec:1.2.1.12 - glyceraldehyde-3-phosphate dehydrogenase (phosphorylating)	TS.seq.screen.trim.Contig479
gray	ec:2.5.1.10 - geranyltranstransferase	TS.seq.screen.trim.Contig110

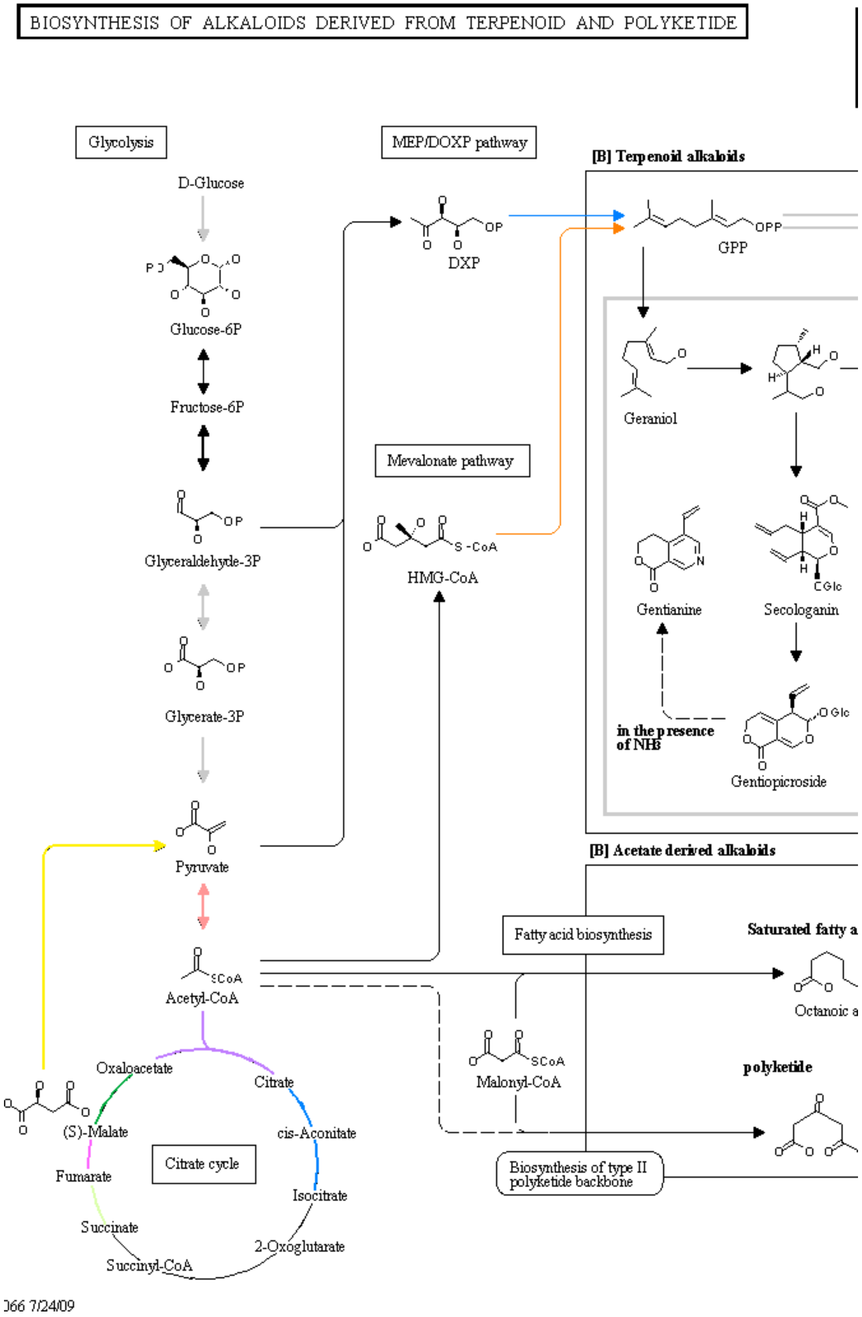

Colour	Enzyme	EST clone
red	ec:4.1.2.13 - fructose-bisphosphate aldolase	TSAY.R35.esd
yellow	ec:1.1.1.40 - malate dehydrogenase (oxaloacetate-decarboxylating) (NADP+)	TS.seq.screen.trim.Contig443
orange	ec:4.2.1.11 - phosphopyruvate hydratase	TS.seq.screen.trim.Contig231, TS.seq.screen.trim.Contig433
green	ec:1.1.1.37 - malate dehydrogenase	TS.seq.screen.trim.Contig383, TSCB.R83.esd
blue	ec:4.2.1.3 - aconitate hydratase	TSAP.R16.esd
pink	ec:4.2.1.2 - fumarate hydratase	TSAS.R96.esd
violet	ec:2.3.3.1 - citrate (Si)-synthase	TS.seq.screen.trim.Contig330
light-red	ec:2.3.1.12 - dihydrolipoyllysine-residue acetyltransferase	TS.seq.screen.trim.Contig176
light-green	ec:1.3.5.1 - succinate dehydrogenase (ubiquinone)	TS.seq.screen.trim.Contig279, TS.seq.screen.trim.Contig370, TSCF.R66.esd
light-yellow	ec:2.5.1.29 - farnesyltranstransferase	TS.seq.screen.trim.Contig110
gray	ec:2.7.1.40 - pyruvate kinase	TS.seq.screen.trim.Contig291, TS.seq.screen.trim.Contig452
gray	ec:5.4.2.1 - phosphoglycerate mutase	TSAP.R46.esd
gray	ec:2.7.1.1 - hexokinase	TS.seq.screen.trim.Contig224
gray	ec:1.2.1.12 - glyceraldehyde-3-phosphate dehydrogenase (phosphorylating)	TS.seq.screen.trim.Contig479
gray	ec:2.5.1.10 - geranyltranstransferase	TS.seq.screen.trim.Contig110

BIOSYNTHESIS OF ALKALOIDS DERIVED FROM SHIKIMATE PATHWAY

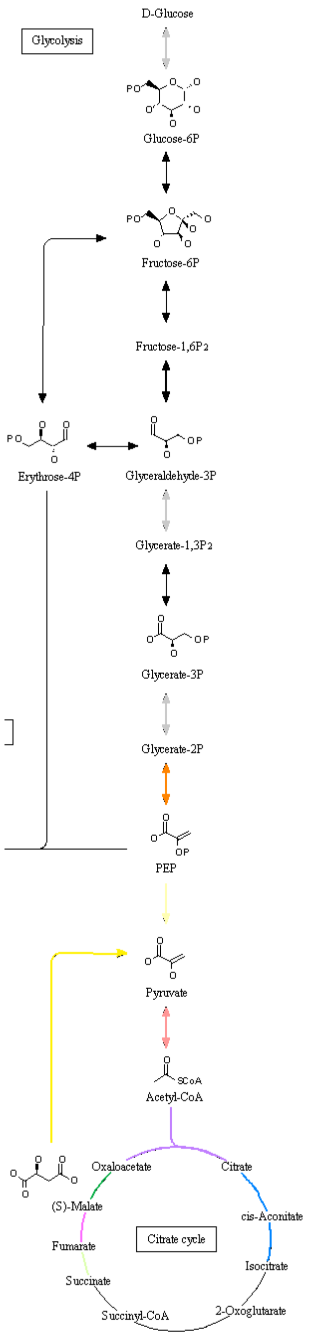

Colour	Enzyme	EST clone
red	ec:4.1.2.13 - fructose-bisphosphate aldolase	TSAY.R35.esd
yellow	ec:1.1.1.40 - malate dehydrogenase (oxaloacetate-decarboxylating) (NADP+)	TS.seq.screen.trim.Contig443
orange	ec:4.2.1.11 - phosphopyruvate hydratase	TS.seq.screen.trim.Contig231, TS.seq.screen.trim.Contig433
green	ec:1.1.1.37 - malate dehydrogenase	TS.seq.screen.trim.Contig383, TSCB.R83.esd
blue	ec:4.2.1.3 - aconitate hydratase	TSAP.R16.esd
pink	ec:4.2.1.2 - fumarate hydratase	TSAS.R96.esd
violet	ec:2.3.3.1 - citrate (Si)-synthase	TS.seq.screen.trim.Contig330
light-red	ec:2.3.1.12 - dihydrolipoyllysine-residue acetyltransferase	TS.seq.screen.trim.Contig176
light-green	ec:1.3.5.1 - succinate dehydrogenase (ubiquinone)	TS.seq.screen.trim.Contig279, TS.seq.screen.trim.Contig370, TSCF.R66.esd
light-yellow	ec:2.7.1.40 - pyruvate kinase	TS.seq.screen.trim.Contig291, TS.seq.screen.trim.Contig452
gray	ec:5.4.2.1 - phosphoglycerate mutase	TSAP.R46.esd
gray	ec:2.7.1.1 - hexokinase	TS.seq.screen.trim.Contig224
gray	ec:1.2.1.12 - glyceraldehyde-3-phosphate dehydrogenase (phosphorylating)	TS.seq.screen.trim.Contig479

BIOSYNTHESIS OF ALKALOIDS DERIVED FROM ORNITHINE, LYSINE AND NICOTINATE

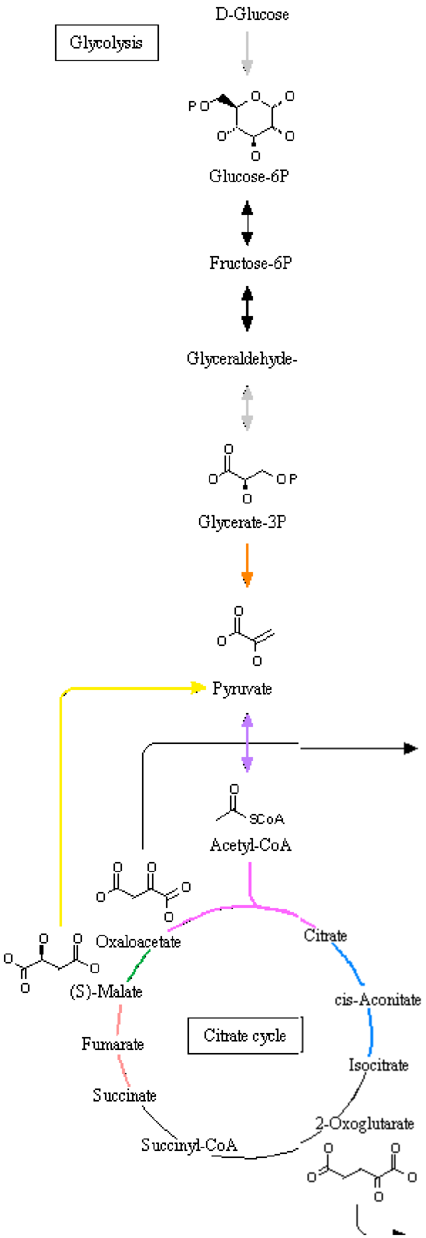

Colour	Enzyme	EST clone
red	ec:4.1.2.13 - fructose-bisphosphate aldolase	TSAY.R35.esd
yellow	ec:1.1.1.40 - malate dehydrogenase (oxaloacetate-decarboxylating) (NADP+)	TS.seq.screen.trim.Contig443
orange	ec:4.2.1.11 - phosphopyruvate hydratase	TS.seq.screen.trim.Contig231, TS.seq.screen.trim.Contig433
green	ec:1.1.1.37 - malate dehydrogenase	TS.seq.screen.trim.Contig383, TSCB.R83.esd
blue	ec:4.2.1.3 - aconitate hydratase	TSAP.R16.esd
pink	ec:2.3.3.1 - citrate (Si)-synthase	TS.seq.screen.trim.Contig330
violet	ec:2.3.1.12 - dihydrolipoyllysine-residue acetyltransferase	TS.seq.screen.trim.Contig176
light-red	ec:1.3.5.1 - succinate dehydrogenase (ubiquinone)	TS.seq.screen.trim.Contig279, TS.seq.screen.trim.Contig370, TSCF.R66.esd
light-green	ec:2.7.1.40 - pyruvate kinase	TS.seq.screen.trim.Contig291, TS.seq.screen.trim.Contig452
light-yellow	ec:5.4.2.1 - phosphoglycerate mutase	TSAP.R46.esd
gray	ec:2.7.1.1 - hexokinase	TS.seq.screen.trim.Contig224
gray	ec:1.2.1.12 - glyceraldehyde-3-phosphate dehydrogenase (phosphorylating)	TS.seq.screen.trim.Contig479

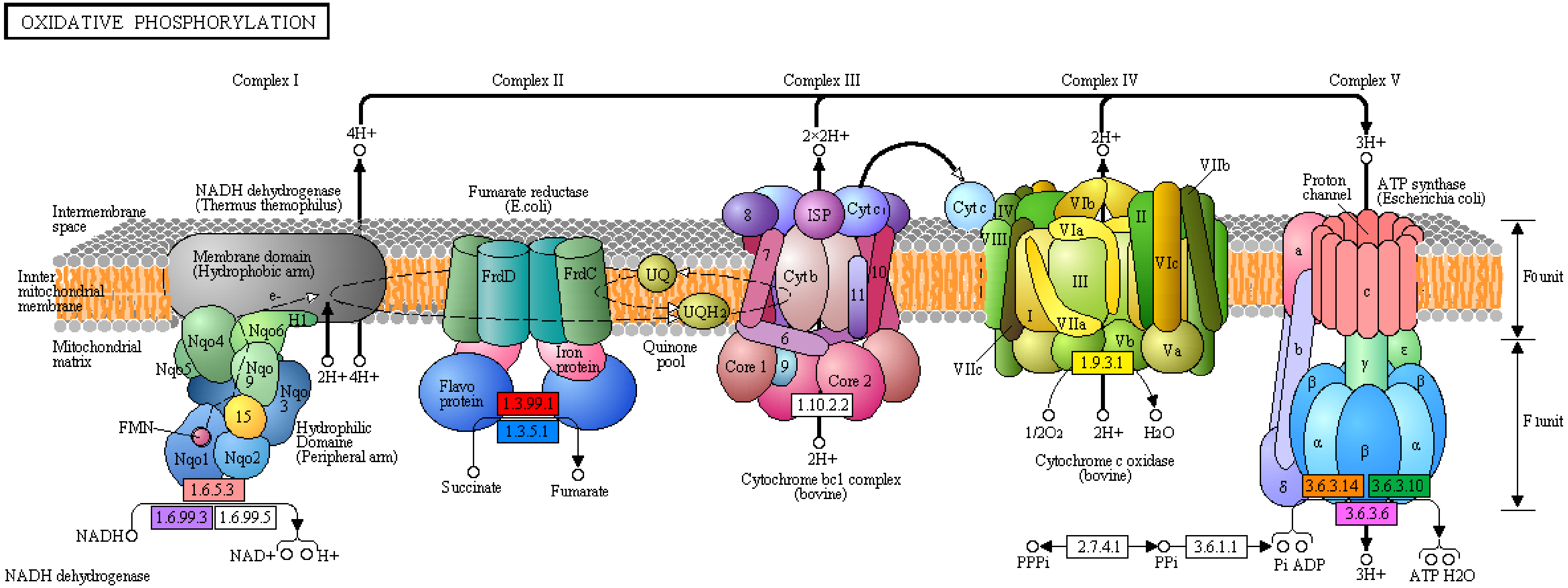

Colour	Enzyme	EST clone
red	ec:1.3.99.1 - succinate dehydrogenase	TS.seq.screen.trim.Contig279
yellow	ec:1.9.3.1 - cytochrome-c oxidase	TSCC.R81.esd, TSAN.R68.esd
orange	ec:3.6.3.14 - H+-transporting two-sector ATPase	TS.seq.screen.trim.Contig251, TSBU.R78.esd, TSAX.R62.esd, TSCG.R36.esd, TSAF.R45.esd, TSAO.R53.esd
green	ec:3.6.3.10 - H+/K+-exchanging ATPase	TSBX.R81.esd
blue	ec:1.3.5.1 - succinate dehydrogenase (ubiquinone)	TS.seq.screen.trim.Contig279, TS.seq.screen.trim.Contig370, TSCF.R66.esd
pink	ec:3.6.3.6 - H+-exporting ATPase	TS.seq.screen.trim.Contig499, TSAX.R62.esd, TSAO.R6.esd, TSCG.R36.esd, TSAO.R53.esd
violet	ec:1.6.99.3 - NADH dehydrogenase	TSAW.R12.esd
light-red	ec:1.6.5.3 - NADH dehydrogenase (ubiquinone)	TSAN.R80.esd, TSBA.R45.esd

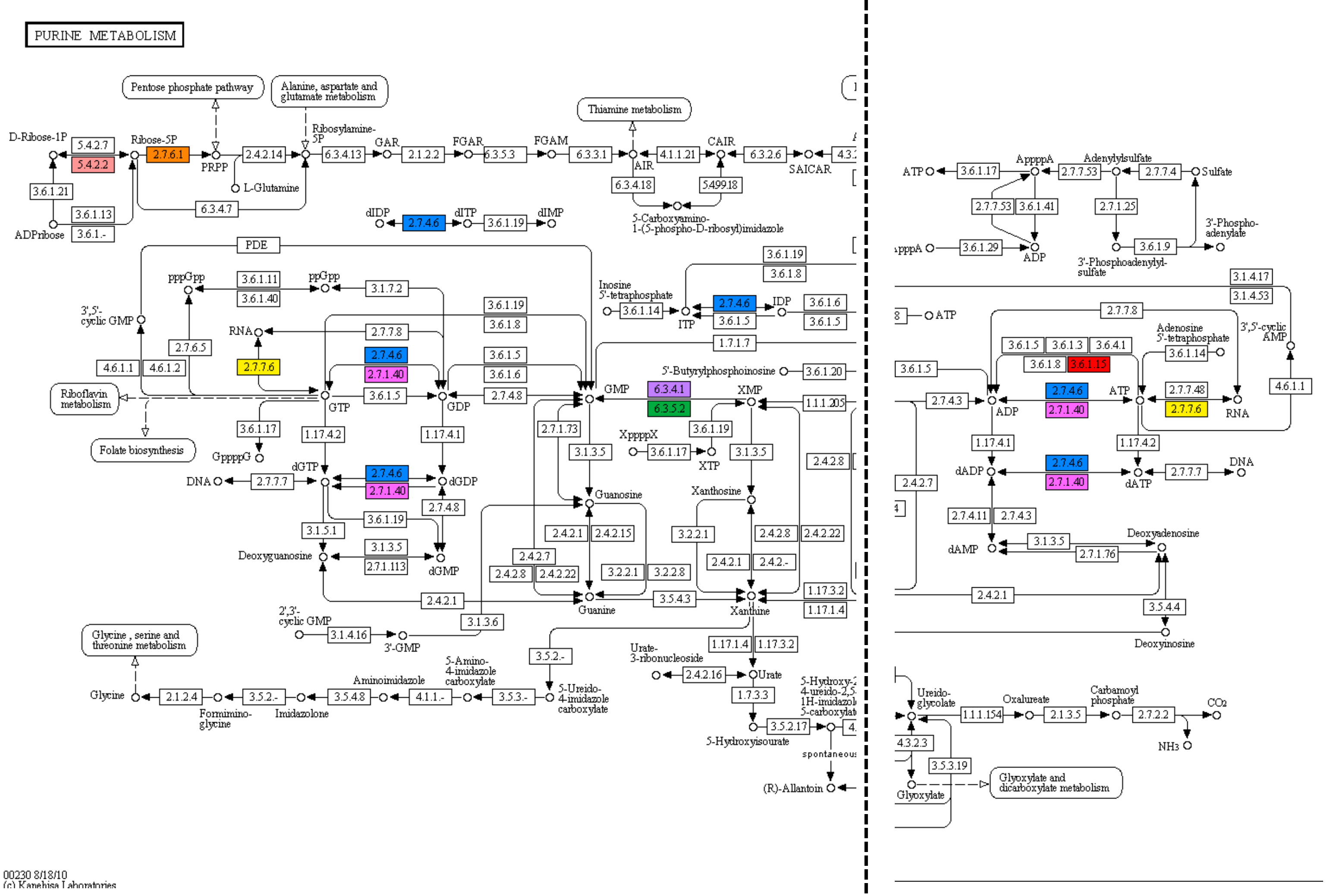

**Colour**	**Enzyme**	**EST clone**
red	ec:3.6.1.15 - nucleoside-triphosphatase	TSAA.R28.esd, TSCC.R43.esd, TSBS.R78.esd
yellow	ec:2.7.7.6 - DNA-directed RNA polymerase	TS.seq.screen.trim.Contig89, TS.seq.screen.trim.Contig326, TSAT.R95.esd, TSAF.R44.esd, TSAC.R2.esd
orange	ec:2.7.6.1 - ribose-phosphate diphosphokinase	TSCH.R19.esd
green	ec:6.3.5.2 - GMP synthase (glutamine-hydrolysing)	TS.seq.screen.trim.Contig177
blue	ec:2.7.4.6 - nucleoside-diphosphate kinase	TS.seq.screen.trim.Contig75
pink	ec:2.7.1.40 - pyruvate kinase	TS.seq.screen.trim.Contig291, TS.seq.screen.trim.Contig452
violet	ec:6.3.4.1 - GMP synthase	TS.seq.screen.trim.Contig177
light-red	ec:5.4.2.2 - phosphoglucomutase	TSBY.R45.esd, TSAX.R47.esd

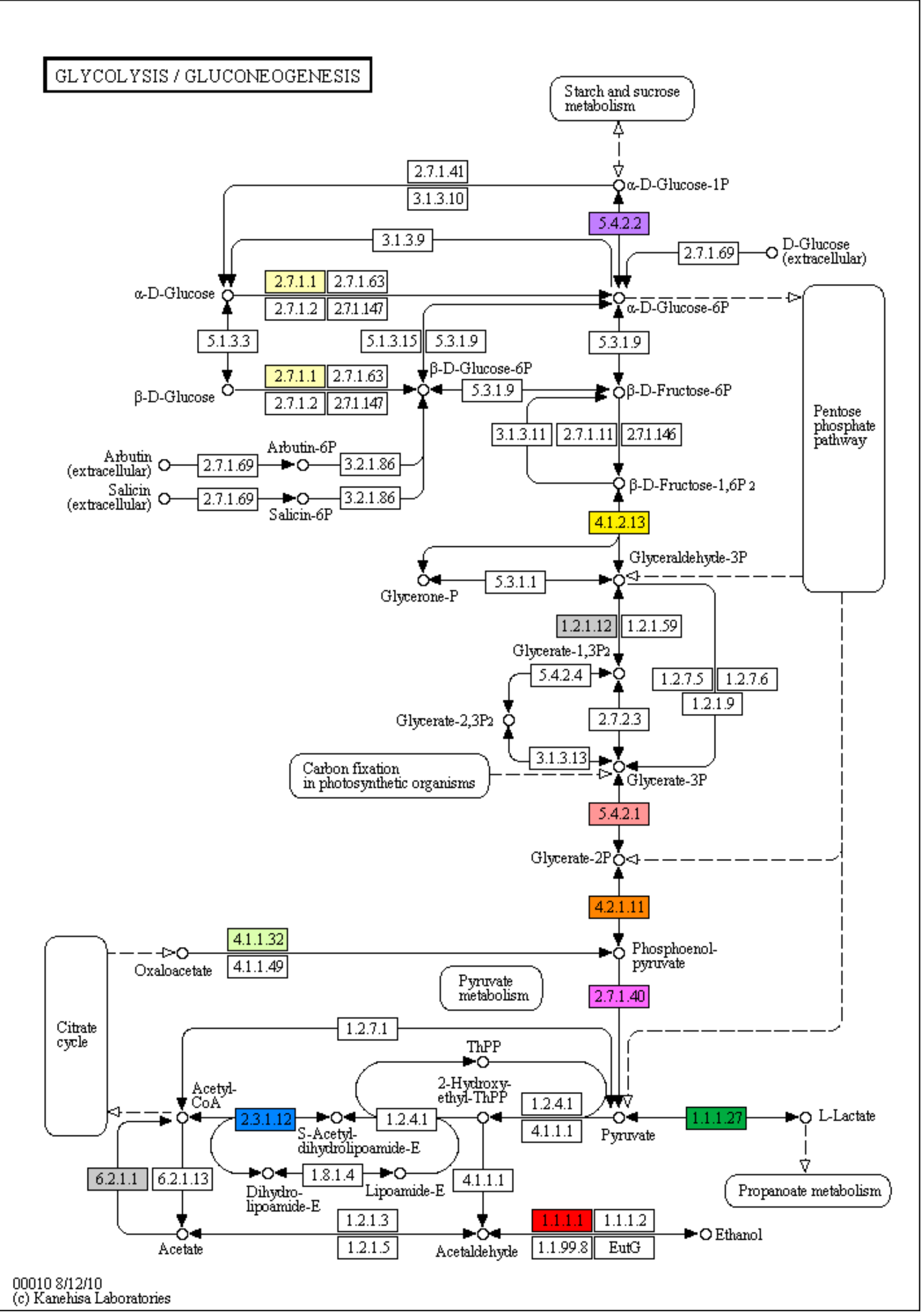

Colour	Enzyme	EST clone
red	ec:1.1.1.1 - alcohol dehydrogenase	TSCE.R92.esd
yellow	ec:4.1.2.13 - fructose-bisphosphate aldolase	TSAY.R35.esd
orange	ec:4.2.1.11 - phosphopyruvate hydratase	TS.seq.screen.trim.Contig231, TS.seq.screen.trim.Contig433
green	ec:1.1.1.27 - L-lactate dehydrogenase	TS.seq.screen.trim.Contig434
blue	ec:2.3.1.12 - dihydrolipoyllysine-residue acetyltransferase	TS.seq.screen.trim.Contig176
pink	ec:2.7.1.40 - pyruvate kinase	TS.seq.screen.trim.Contig291, TS.seq.screen.trim.Contig452
violet	ec:5.4.2.2 - phosphoglucomutase	TSBY.R45.esd, TSAX.R47.esd
light-red	ec:5.4.2.1 - phosphoglycerate mutase	TSAP.R46.esd
light-green	ec:4.1.1.32 - phosphoenolpyruvate carboxykinase (GTP)	TS.seq.screen.trim.Contig237
light-yellow	ec:2.7.1.1 - hexokinase	TS.seq.screen.trim.Contig224
gray	ec:1.2.1.12 - glyceraldehyde-3-phosphate dehydrogenase (phosphorylating)	TS.seq.screen.trim.Contig479
gray	ec:6.2.1.1 - acetate---CoA ligase	TS.seq.screen.trim.Contig7

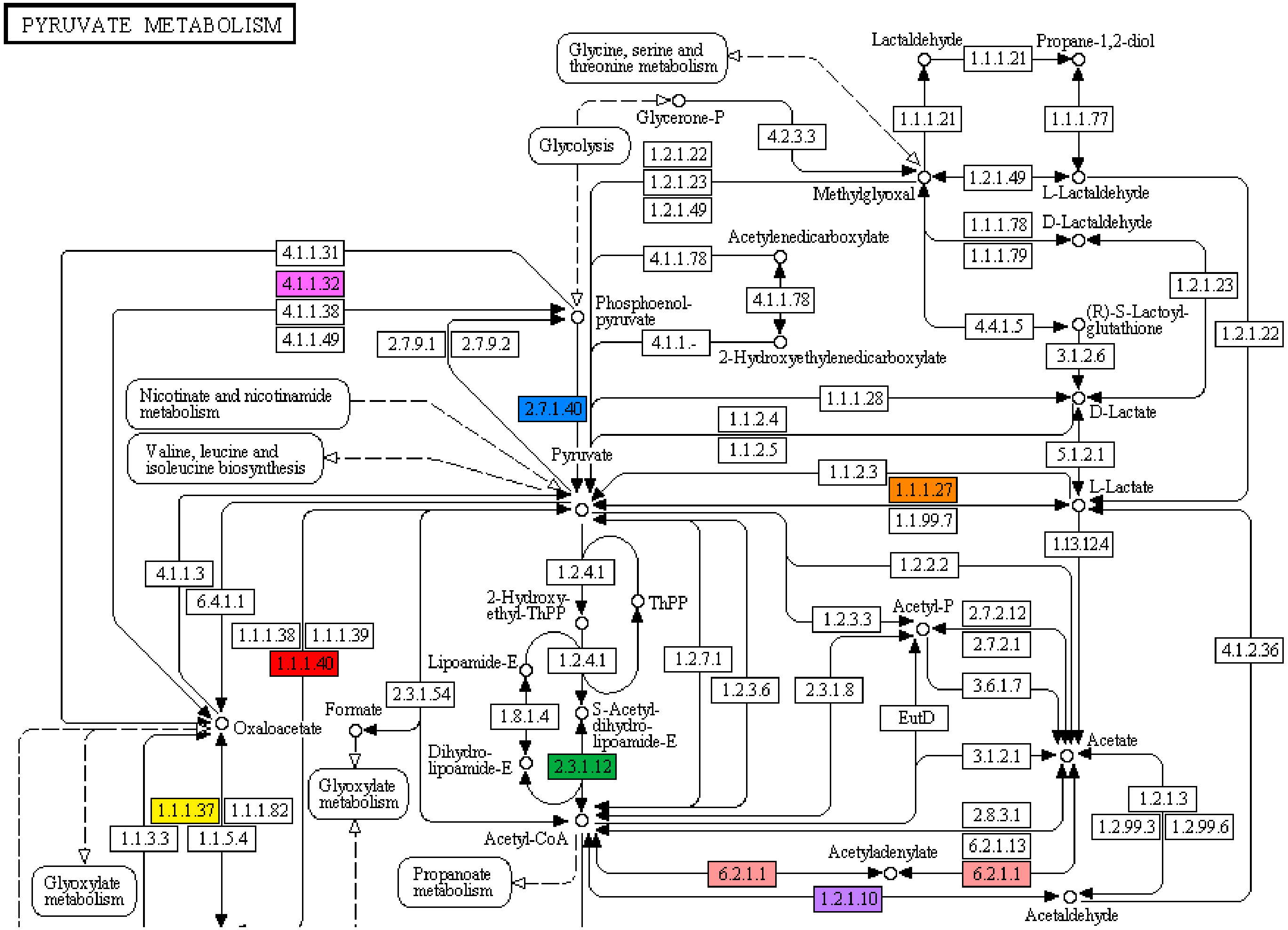

Colour	Enzyme	EST clone
red	ec:1.1.1.40 - malate dehydrogenase (oxaloacetate-decarboxylating) (NADP+)	TS.seq.screen.trim.Contig443
yellow	ec:1.1.1.37 - malate dehydrogenase	TS.seq.screen.trim.Contig383, TSCB.R83.esd
orange	ec:1.1.1.27 - L-lactate dehydrogenase	TS.seq.screen.trim.Contig434
green	ec:2.3.1.12 - dihydrolipoyllysine-residue acetyltransferase	TS.seq.screen.trim.Contig176
blue	ec:2.7.1.40 - pyruvate kinase	TS.seq.screen.trim.Contig291, TS.seq.screen.trim.Contig452
pink	ec:4.1.1.32 - phosphoenolpyruvate carboxykinase (GTP)	TS.seq.screen.trim.Contig237
violet	ec:1.2.1.10 - acetaldehyde dehydrogenase (acetylating)	TSCE.R92.esd
light-red	ec:6.2.1.1 - acetate---CoA ligase	TS.seq.screen.trim.Contig7

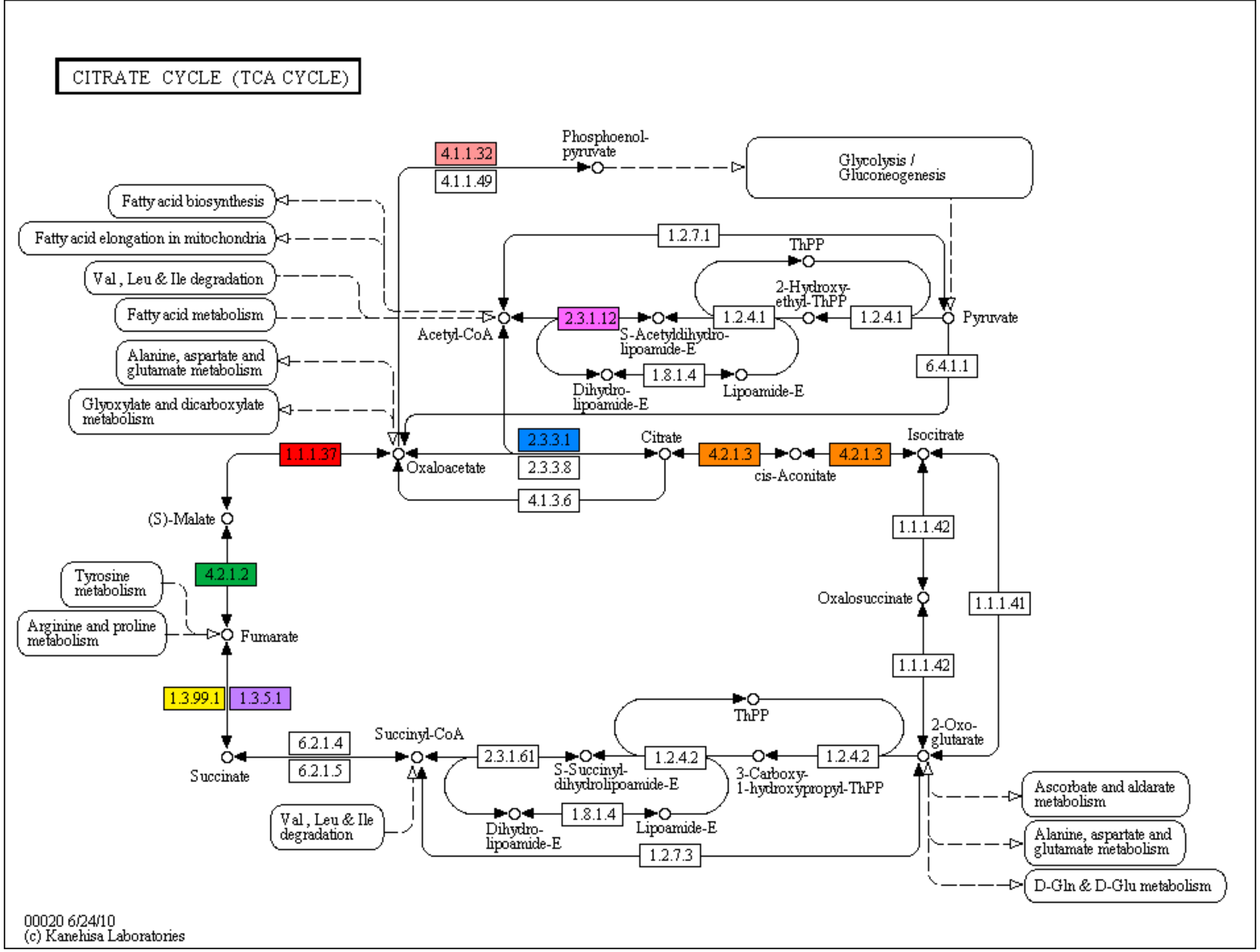

Colour	Enzyme	EST clone
red	ec:1.1.1.37 - malate dehydrogenase	TS.seq.screen.trim.Contig383, TSCB.R83.esd
yellow	ec:1.3.99.1 - succinate dehydrogenase	TS.seq.screen.trim.Contig279
orange	ec:4.2.1.3 - aconitate hydratase	TSAP.R16.esd
green	ec:4.2.1.2 - fumarate hydratase	TSAS.R96.esd
blue	ec:2.3.3.1 - citrate (Si)-synthase	TS.seq.screen.trim.Contig330
pink	ec:2.3.1.12 - dihydrolipoyllysine-residue acetyltransferase	TS.seq.screen.trim.Contig176
violet	ec:1.3.5.1 - succinate dehydrogenase (ubiquinone)	TS.seq.screen.trim.Contig279, TS.seq.screen.trim.Contig370, TSCF.R66.esd
light-red	ec:4.1.1.32 - phosphoenolpyruvate carboxykinase (GTP)	TS.seq.screen.trim.Contig237

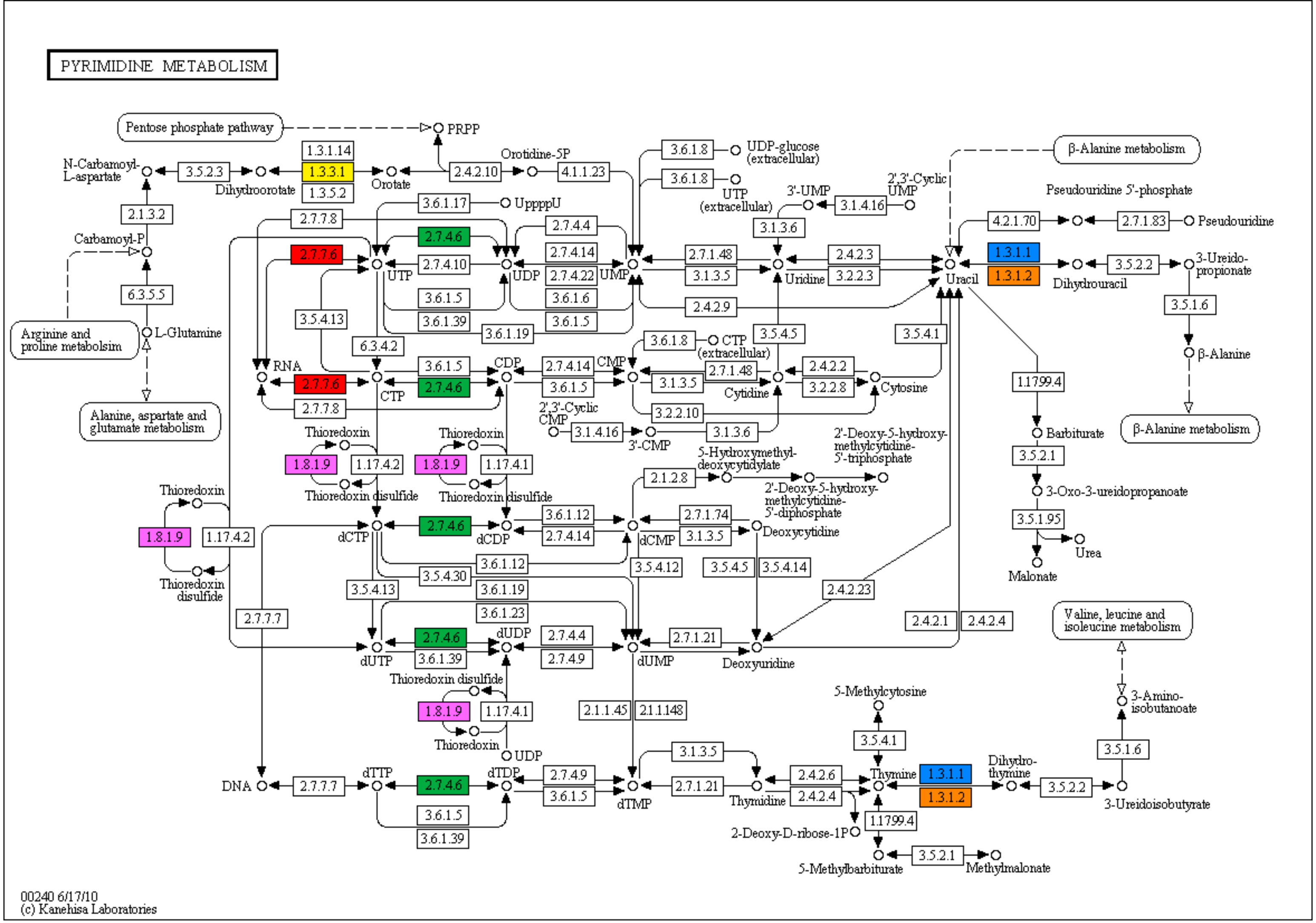

Colour	Enzyme	EST clone
red	ec:2.7.7.6 - DNA-directed RNA polymerase	TS.seq.screen.trim.Contig89, TS.seq.screen.trim.Contig326, TSAT.R95.esd, TSAF.R44.esd, TSAC.R2.esd
yellow	ec:1.3.3.1 - dihydroorotate oxidase	TSCF.R86.esd
orange	ec:1.3.1.2 - dihydropyrimidine dehydrogenase (NADP+)	TSCF.R86.esd
green	ec:2.7.4.6 - nucleoside-diphosphate kinase	TS.seq.screen.trim.Contig75
blue	ec:1.3.1.1 - dihydrouracil dehydrogenase (NAD+)	TSCF.R86.esd
pink	ec:1.8.1.9 - thioredoxin-disulfide reductase	TS.seq.screen.trim.Contig1, TS.seq.screen.trim.Contig445

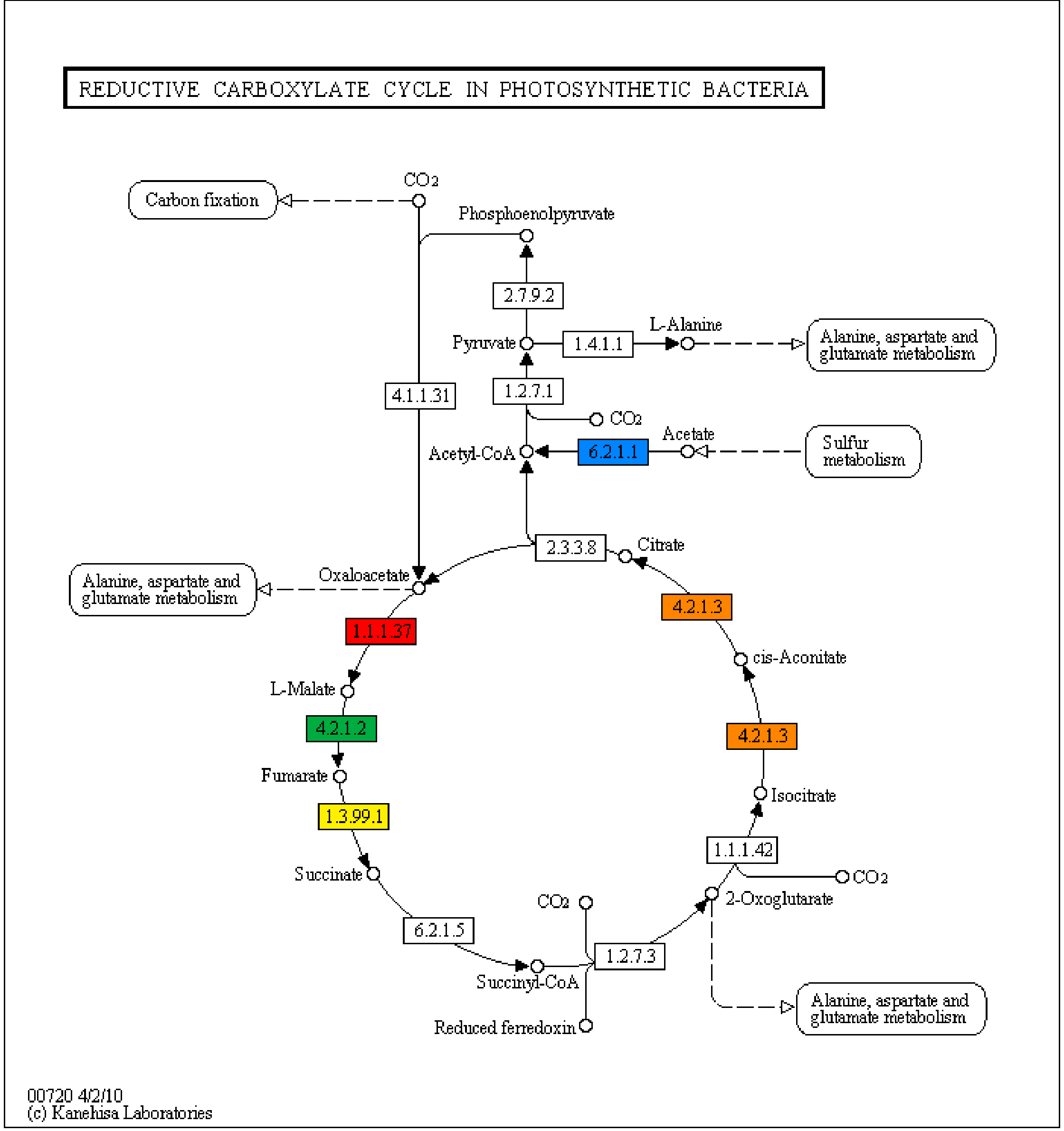

**Colour**	**Enzyme**	**EST clone**
red	ec:1.1.1.37 - malate dehydrogenase	TS.seq.screen.trim.Contig383, TSCB.R83.esd
yellow	ec:1.3.99.1 - succinate dehydrogenase	TS.seq.screen.trim.Contig279
orange	ec:4.2.1.3 - aconitate hydratase	TSAP.R16.esd
green	ec:4.2.1.2 - fumarate hydratase	TSAS.R96.esd
blue	ec:6.2.1.1 - acetate---CoA ligase	TS.seq.screen.trim.Contig7

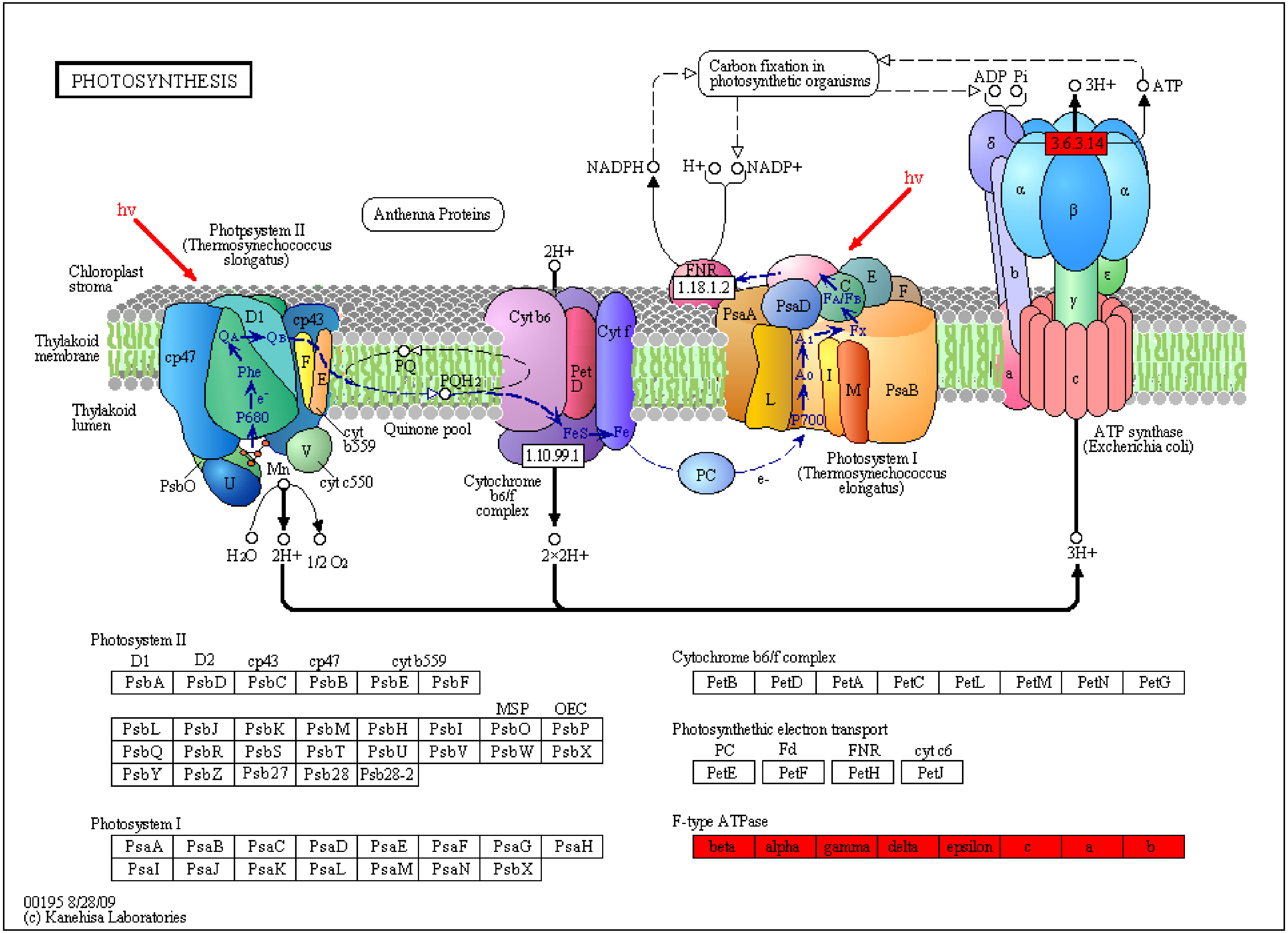

Colour	Enzyme	EST clone
red	ec:3.6.3.14 - H+-transporting two-sector ATPase	TS.seq.screen.trim.Contig251, TSBU.R78.esd, TSAX.R62.esd, TSCG.R36.esd, TSAF.R45.esd, TSAO.R53.esd

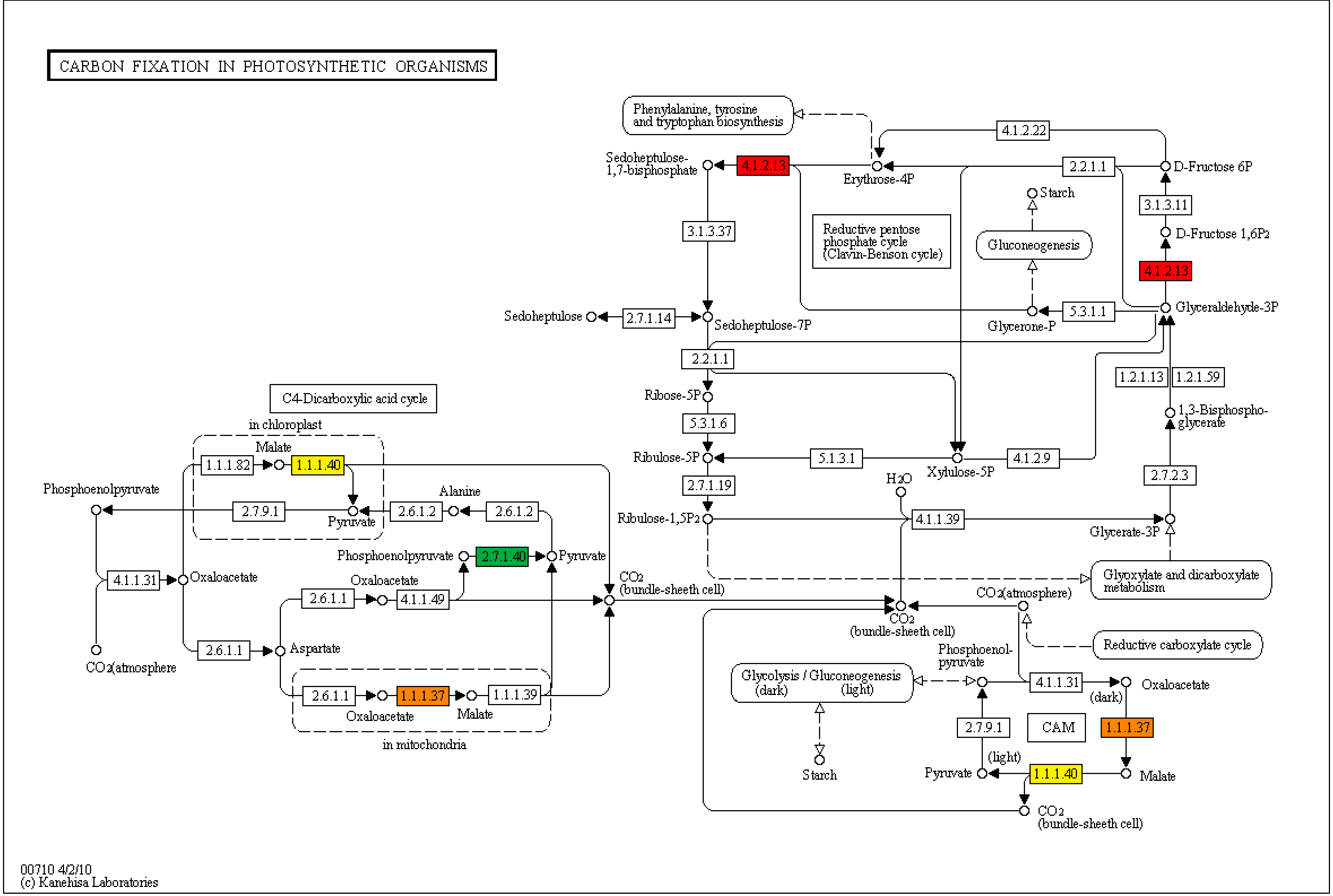

Colour	Enzyme	EST clone
red	ec:4.1.2.13 - fructose-bisphosphate aldolase	TSAY.R35.esd
yellow	ec:1.1.1.40 - malate dehydrogenase (oxaloacetate-decarboxylating) (NADP+)	TS.seq.screen.trim.Contig443
orange	ec:1.1.1.37 - malate dehydrogenase	TS.seq.screen.trim.Contig383, TSCB.R83.esd
green	ec:2.7.1.40 - pyruvate kinase	TS.seq.screen.trim.Contig291, TS.seq.screen.trim.Contig452

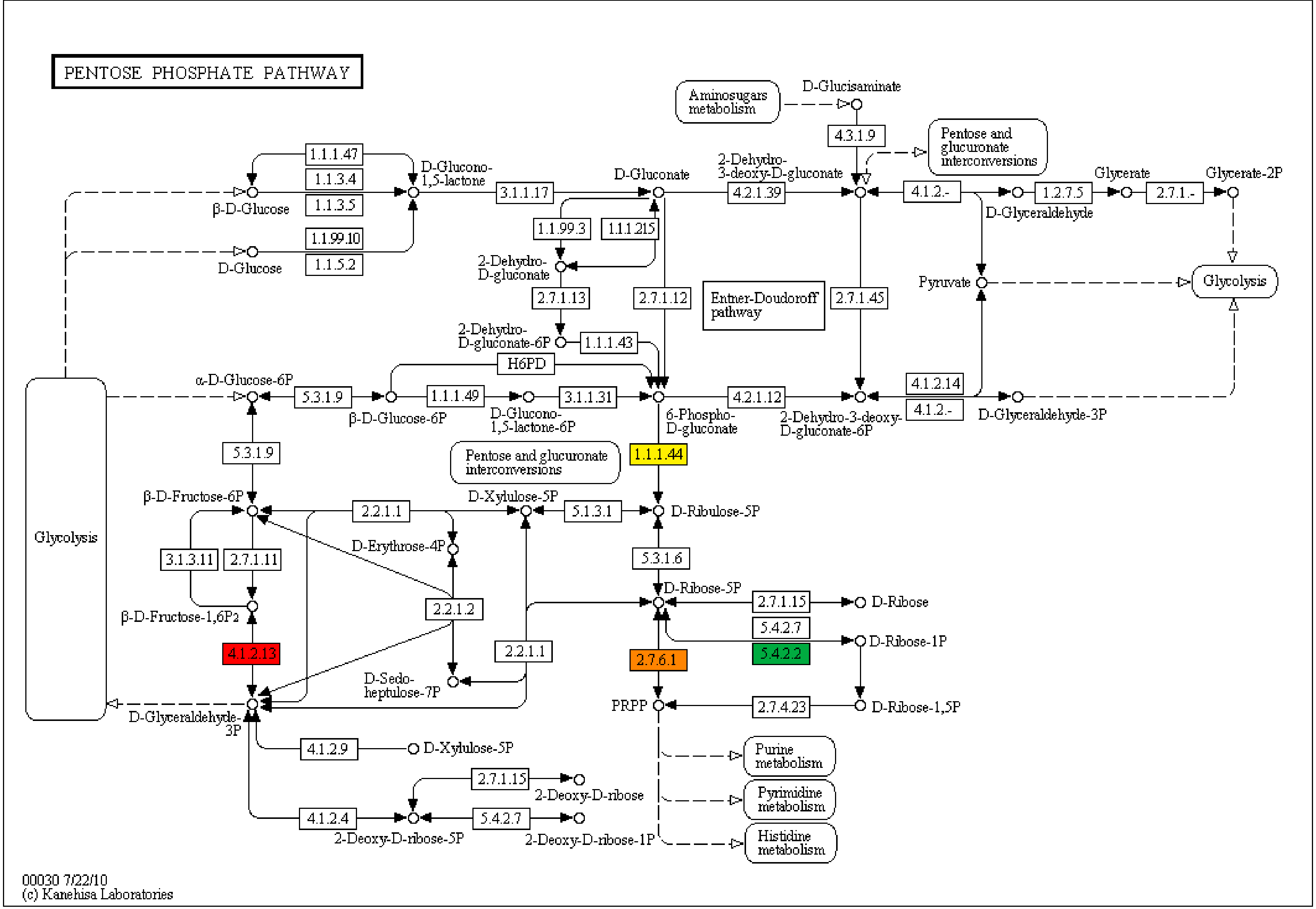

Colour	Enzyme	EST clone
red	ec:4.1.2.13 - fructose-bisphosphate aldolase	TSAY.R35.esd
yellow	ec:1.1.1.44 - phosphogluconate dehydrogenase (decarboxylating)	TSBR.R51.esd
orange	ec:2.7.6.1 - ribose-phosphate diphosphokinase	TSCH.R19.esd
green	ec:5.4.2.2 - phosphoglucomutase	TSBY.R45.esd, TSAX.R47.esd

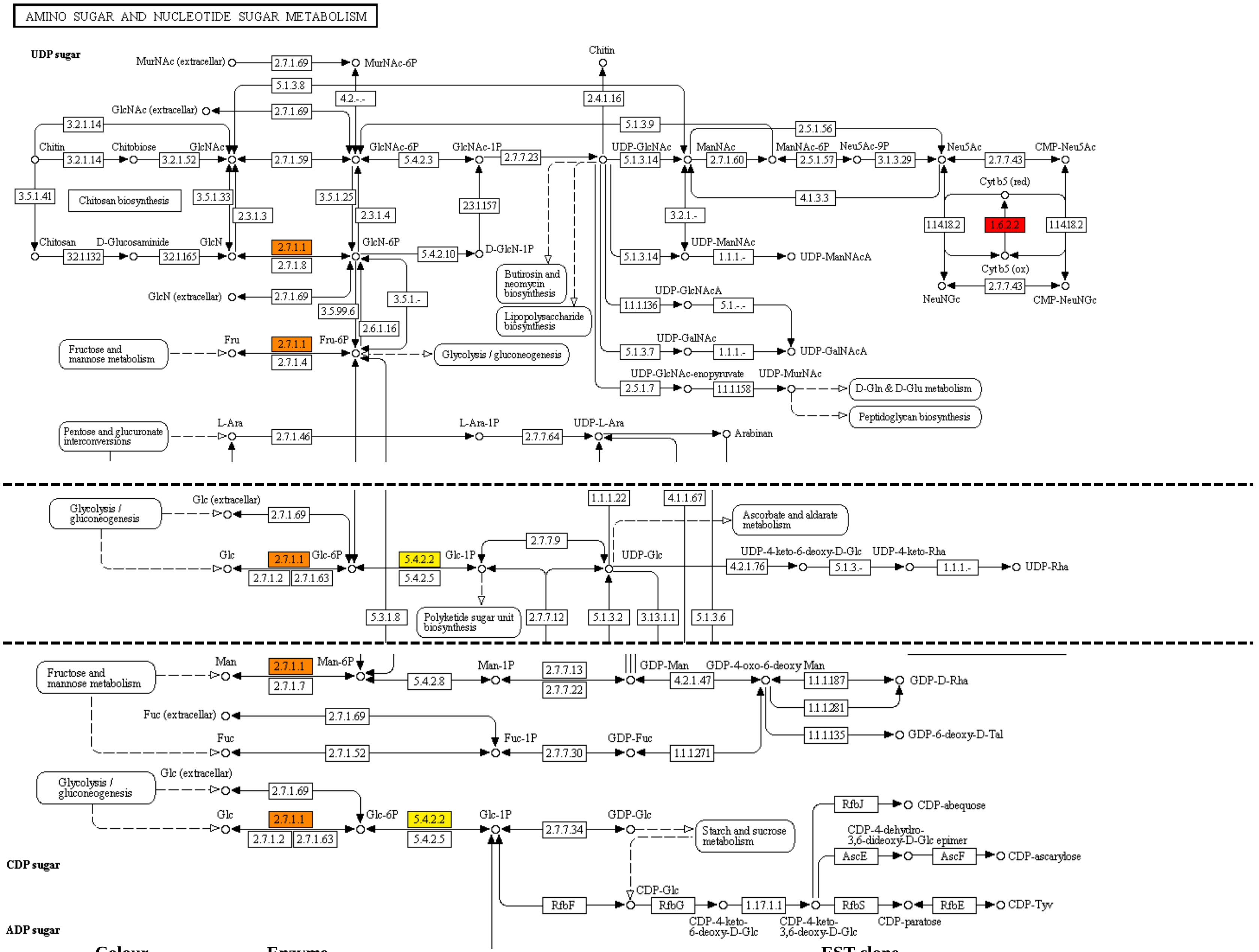

Colour	Enzyme	EST clone
red	ec:1.6.2.2 - cytochrome-b5 reductase	TSBG.R66.esd
yellow	ec:5.4.2.2 - phosphoglucomutase	TSBY.R45.esd, TSAX.R47.esd
orange	ec:2.7.1.1 - hexokinase	TS.seq.screen.trim.Contig224

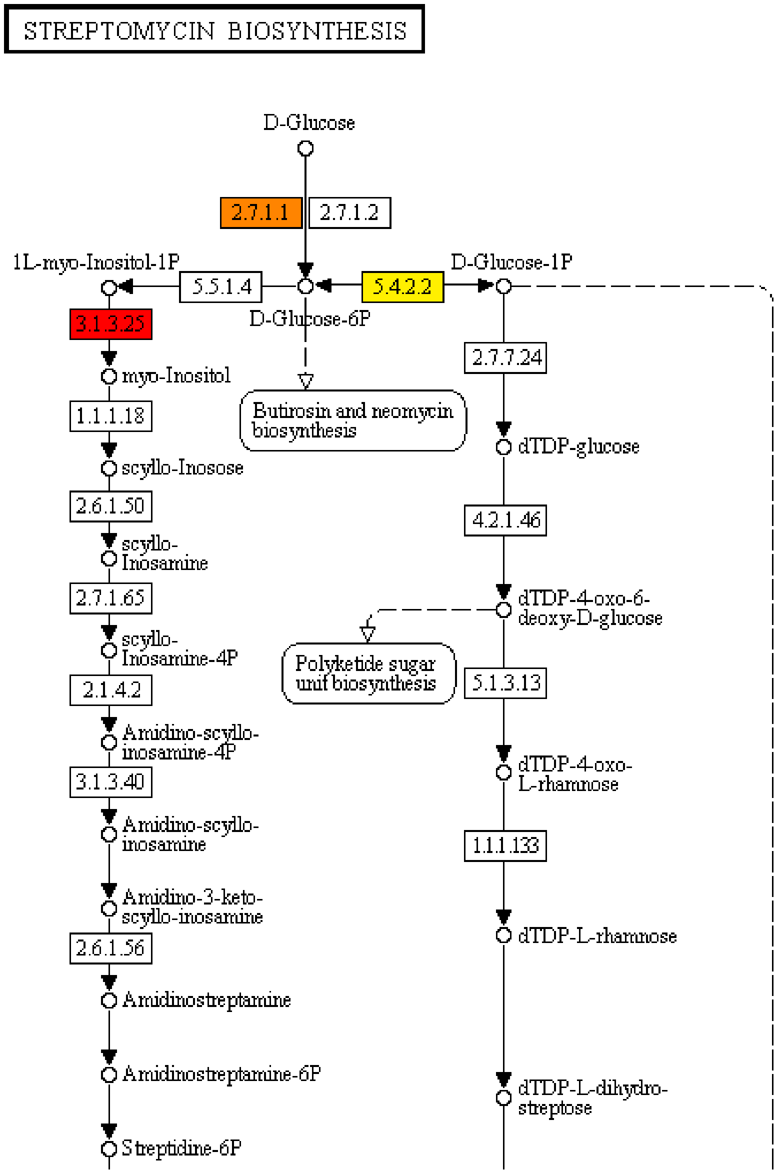

Colour	Enzyme	EST clone
red	ec:3.1.3.25 - inositol-phosphate phosphatase	TS.seq.screen.trim.Contig284
yellow	ec:5.4.2.2 - phosphoglucomutase	TSBY.R45.esd, TSAX.R47.esd
orange	ec:2.7.1.1 - hexokinase	TS.seq.screen.trim.Contig224

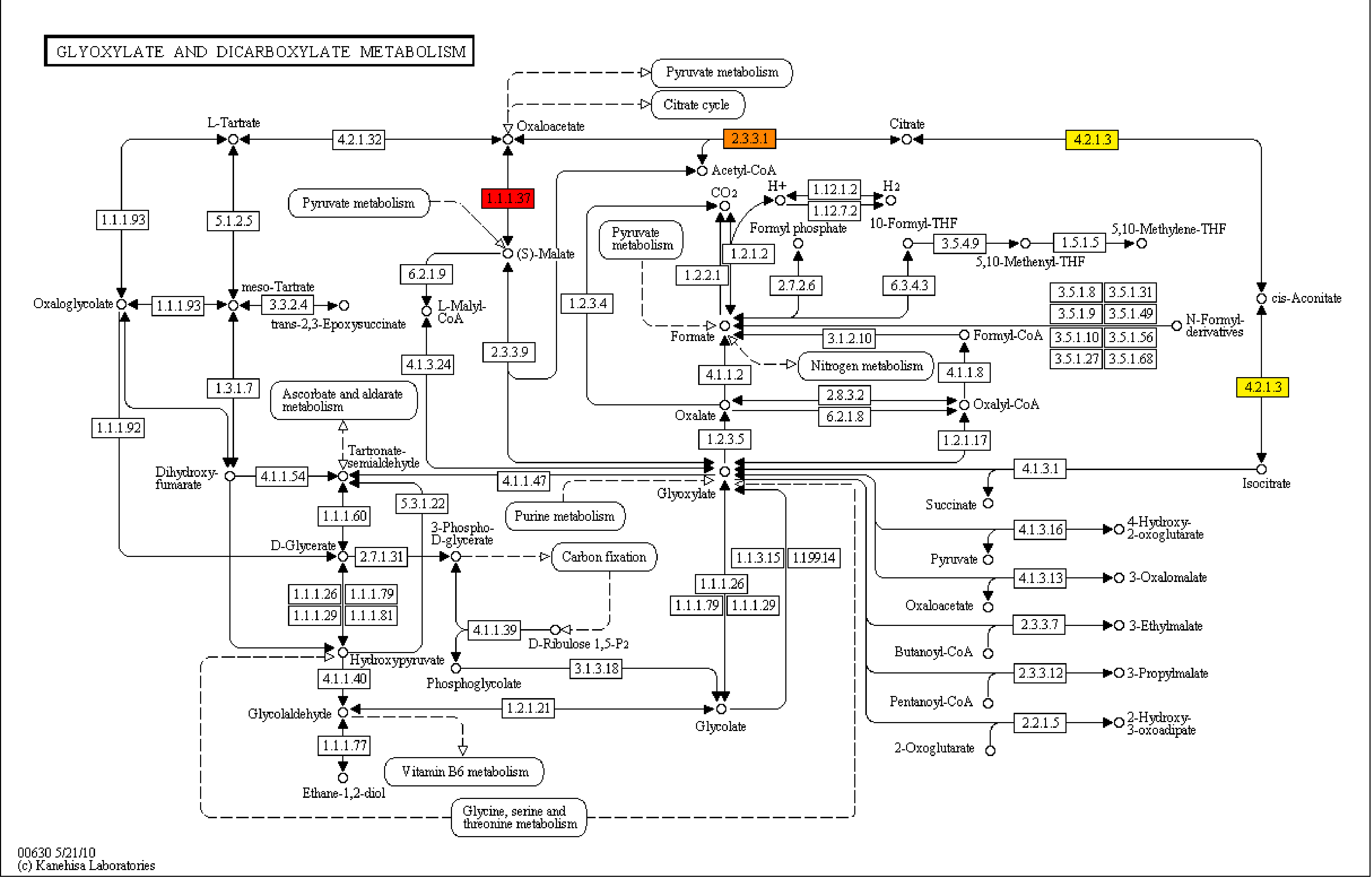

Colour	Enzyme	EST clone
red	ec:1.1.1.37 - malate dehydrogenase	TS.seq.screen.trim.Contig383, TSCB.R83.esd
yellow	ec:4.2.1.3 - aconitate hydratase	TSAP.R16.esd
orange	ec:2.3.3.1 - citrate (Si)-synthase	TS.seq.screen.trim.Contig330

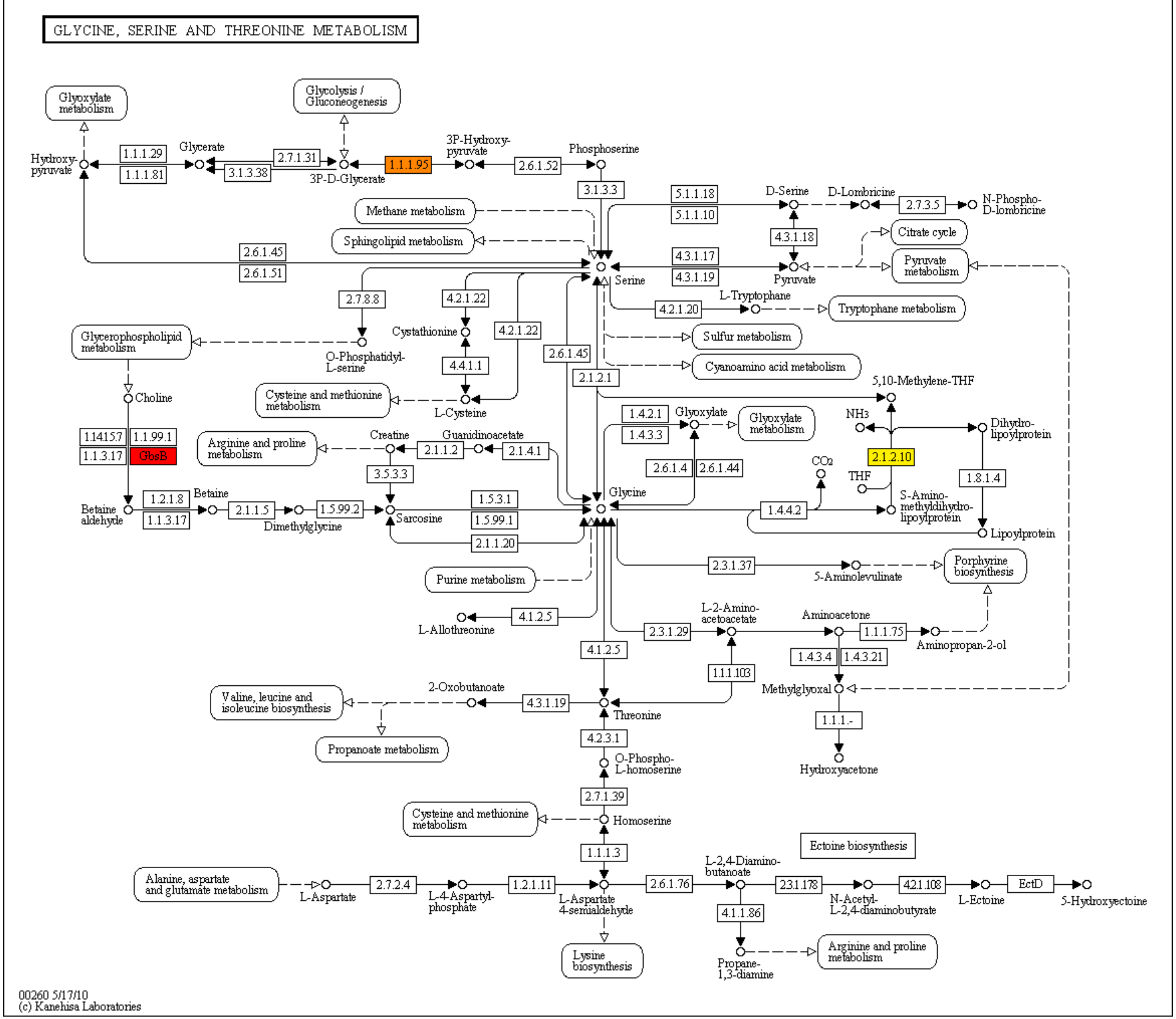

**Colour**	**Enzyme**	**EST clone**
red	ec:1.1.1.1 - alcohol dehydrogenase	TSCE.R92.esd
yellow	ec:2.1.2.10 - aminomethyltransferase	TSBD.R1.esd
orange	ec:1.1.1.95 - phosphoglycerate dehydrogenase	TSBR.R51.esd

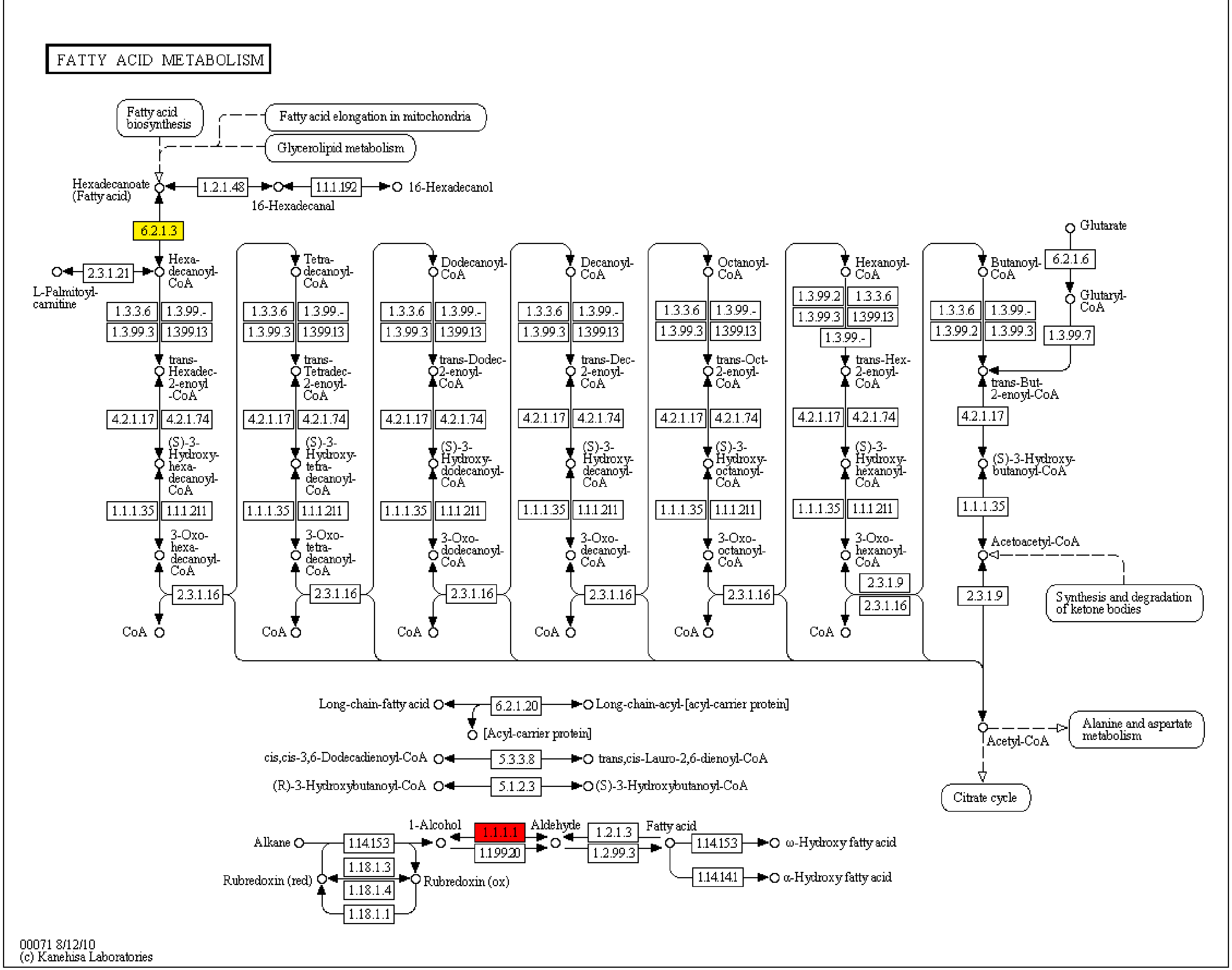

Colour	Enzyme	EST clone
red	ec:1.1.1.1 - alcohol dehydrogenase	TSCE.R92.esd
yellow	ec:6.2.1.3 - long-chain-fatty-acid---CoA ligase	TS.seq.screen.trim.Contig7, TS.seq.screen.trim.Contig264

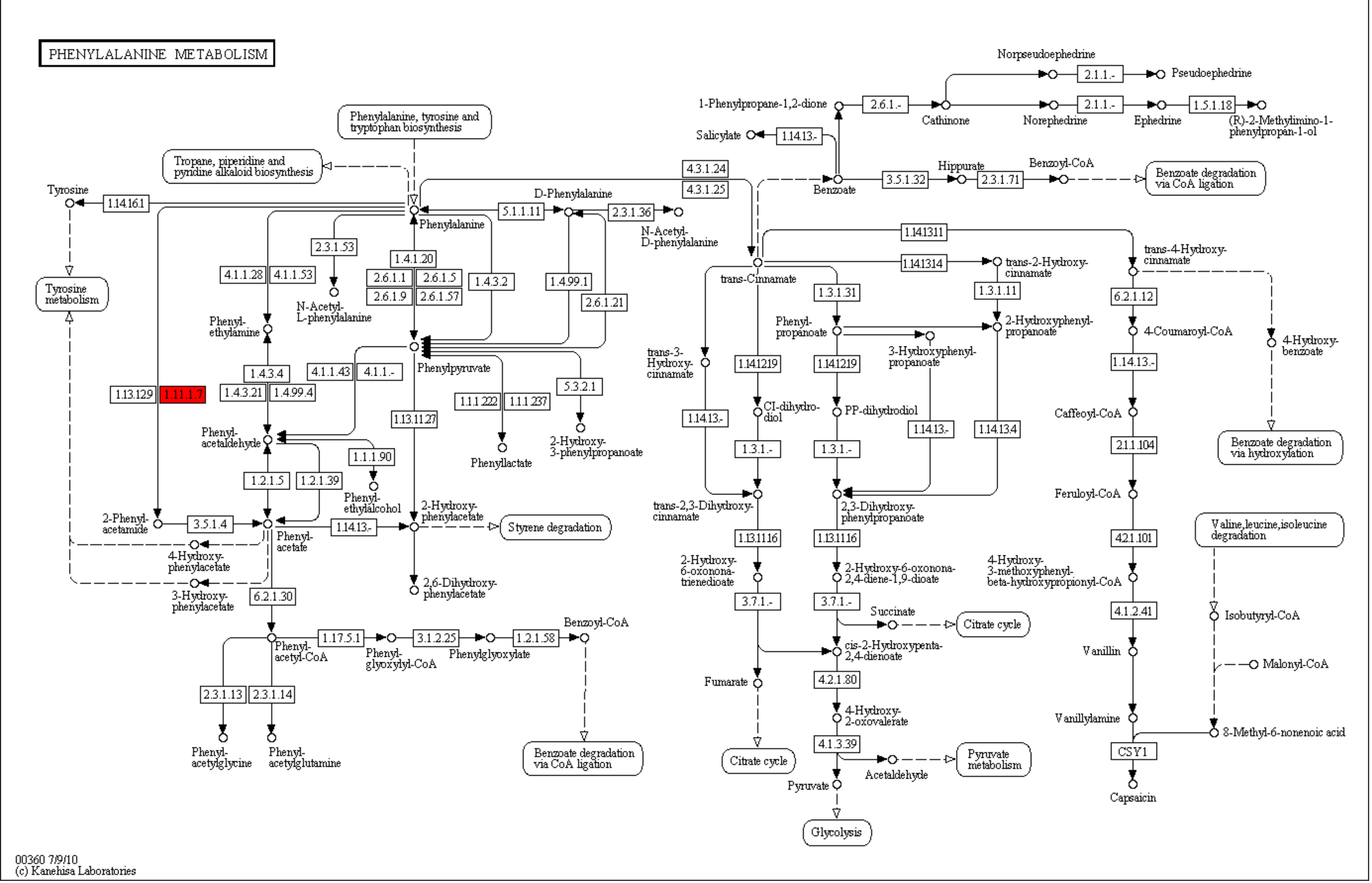

Colour	Enzyme	EST clone
red	ec:1.11.1.7 - peroxidase	TS.seq.screen.trim.Contig503,<br>TSAG.R41.esd,<br>TSAM.R92.esd

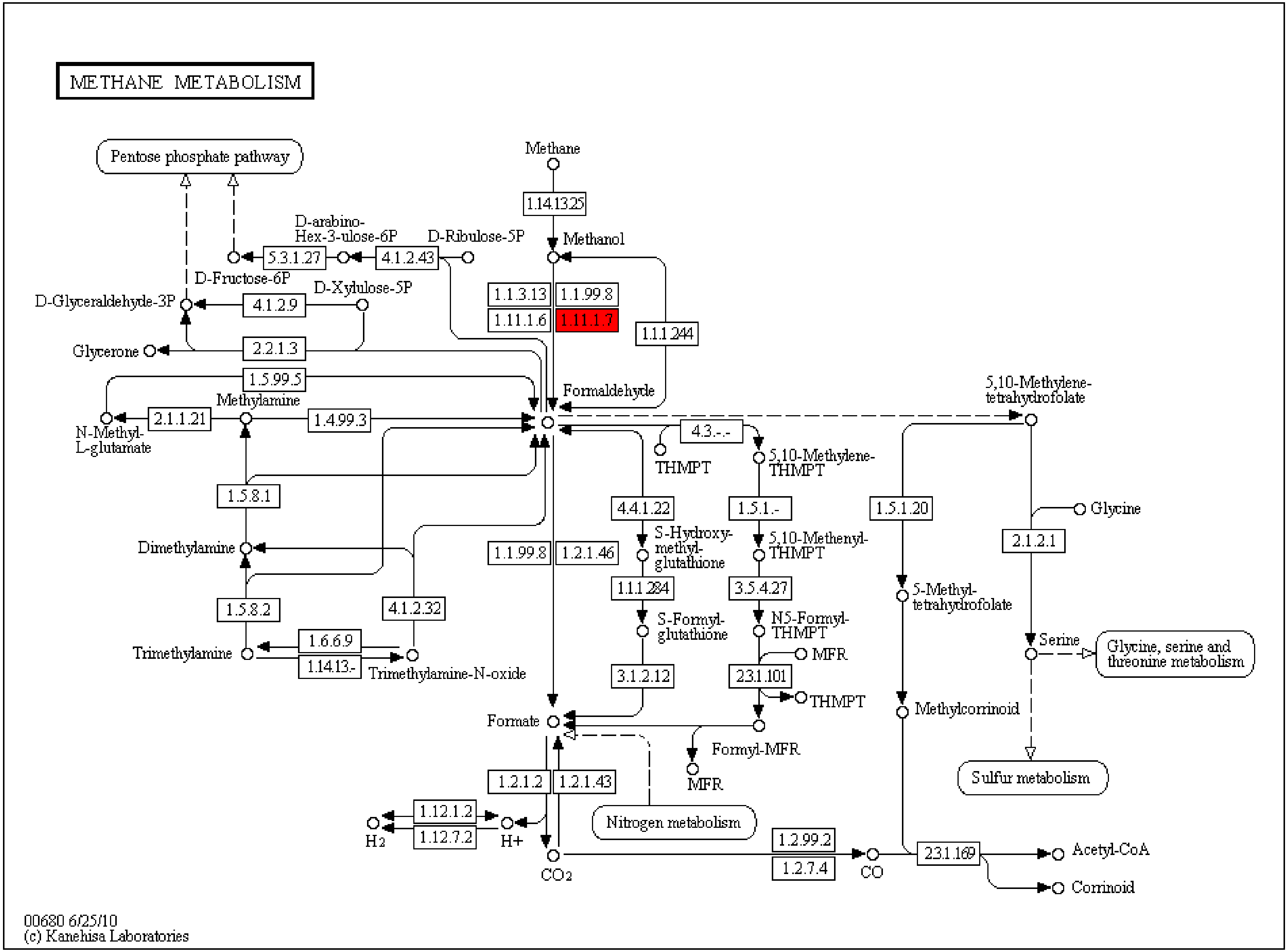

Colour	Enzyme	EST clone
red	ec:1.11.1.7 - peroxidase	TS.seq.screen.trim.Contig503,<br>TSAG.R41.esd,<br>TSAM.R92.esd

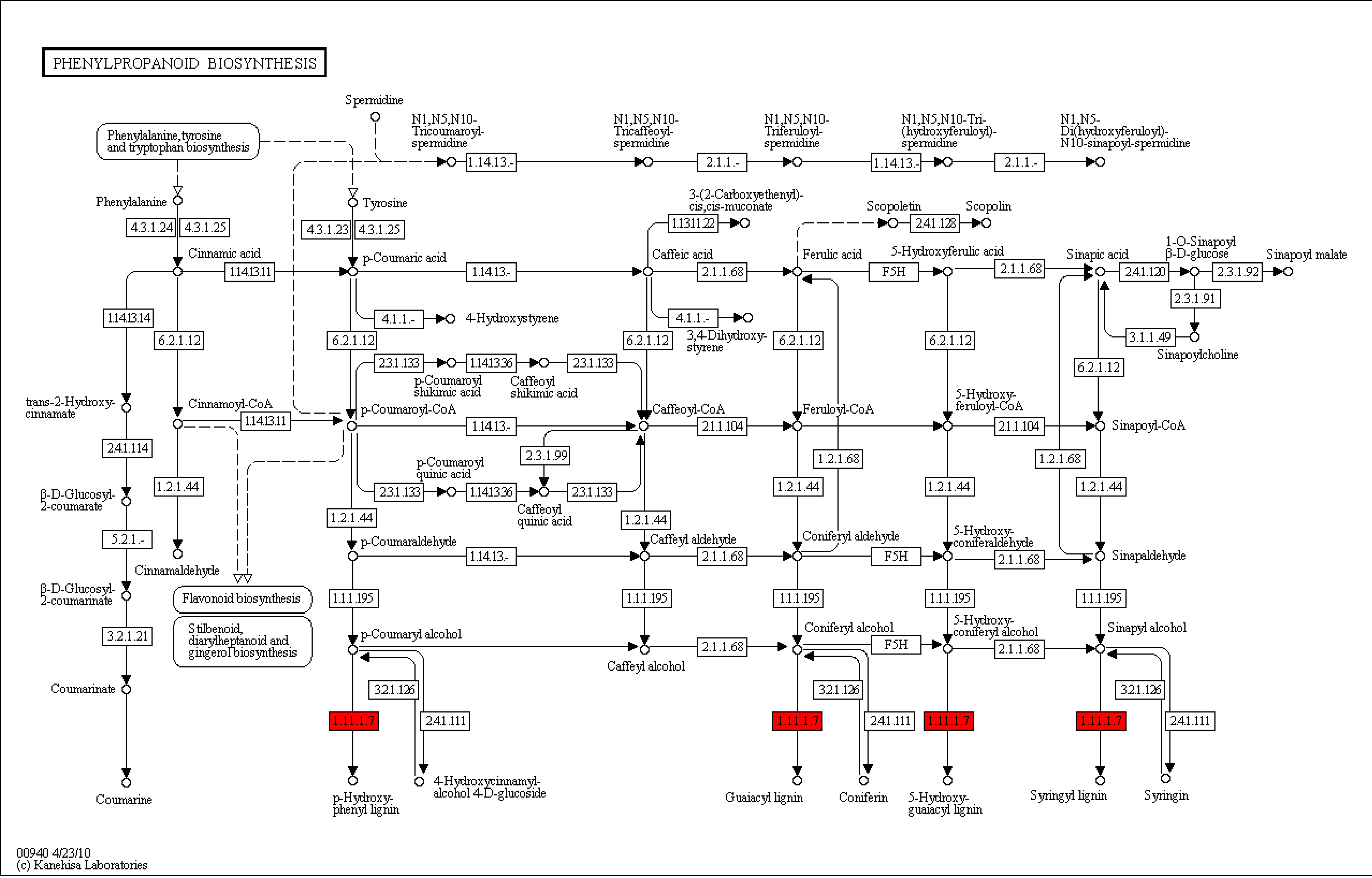

Colour	Enzyme	EST clone
red	ec:1.11.1.7 - peroxidase	TS.seq.screen.trim.Contig503,<br>TSAG.R41.esd,<br>TSAM.R92.esd

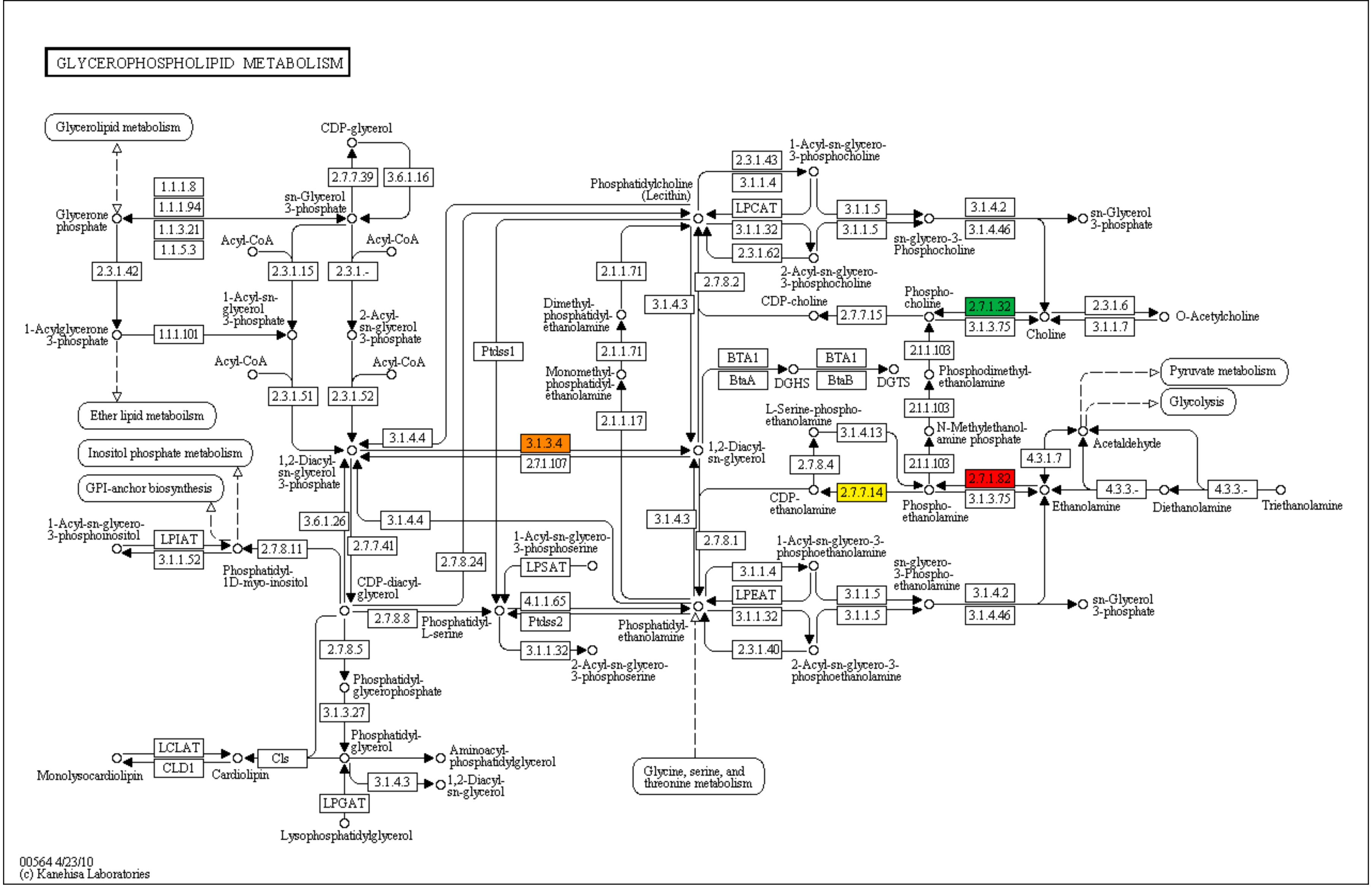

Colour	Enzyme	EST clone
red	ec:2.7.1.82 - ethanolamine kinase	TSBX.R81.esd
yellow	ec:2.7.7.14 - ethanolamine-phosphate cytidylyltransferase	TSBP.R20.esd
orange	ec:3.1.3.4 - phosphatidate phosphatase	TSBQ.R56.esd
green	ec:2.7.1.32 - choline kinase	TSBX.R81.esd

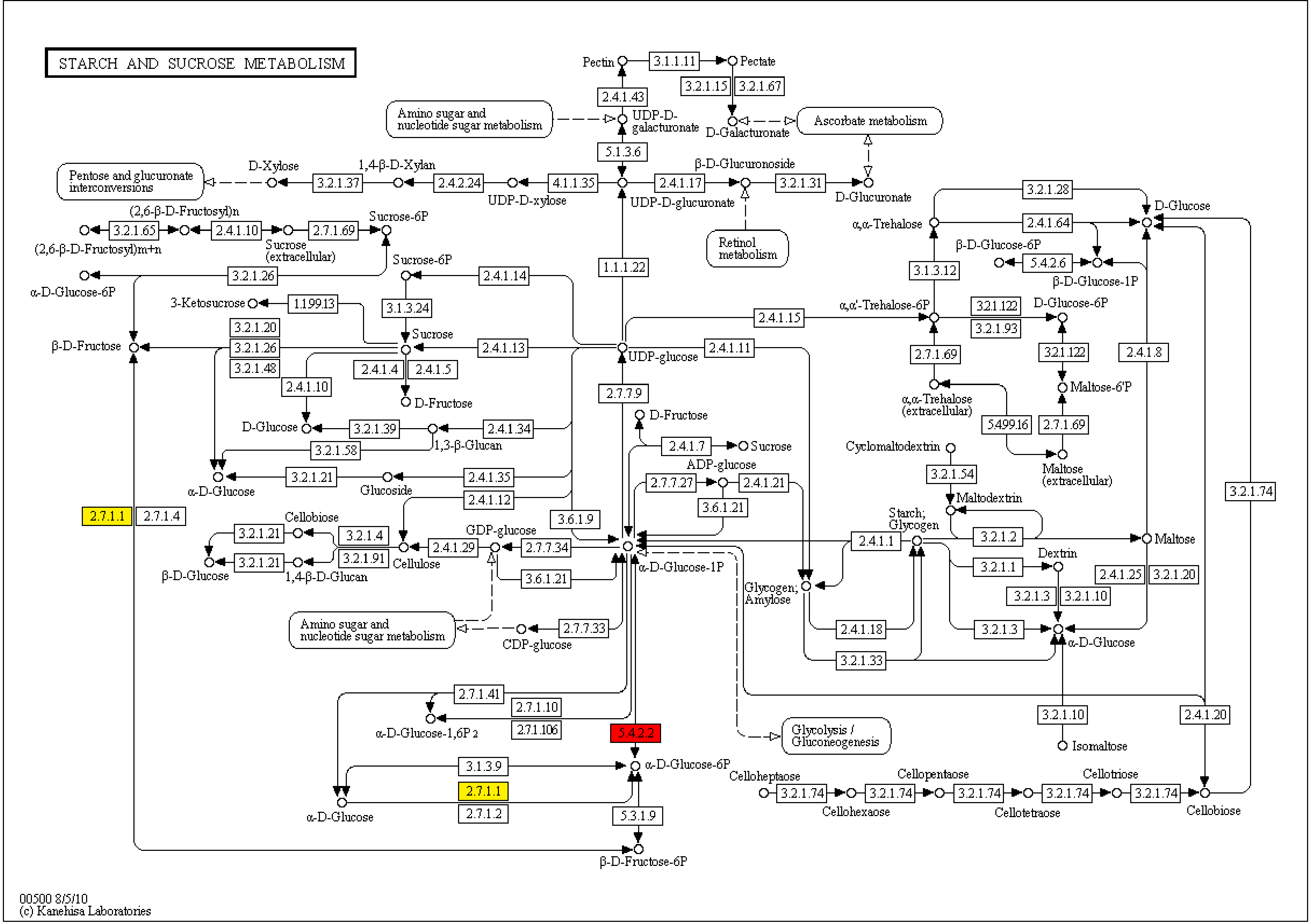

Colour	Enzyme	EST clone
red	ec:5.4.2.2 - phosphoglucomutase	TSBY.R45.esd, TSAX.R47.esd
yellow	ec:2.7.1.1 - hexokinase	TS.seq.screen.trim.Contig224

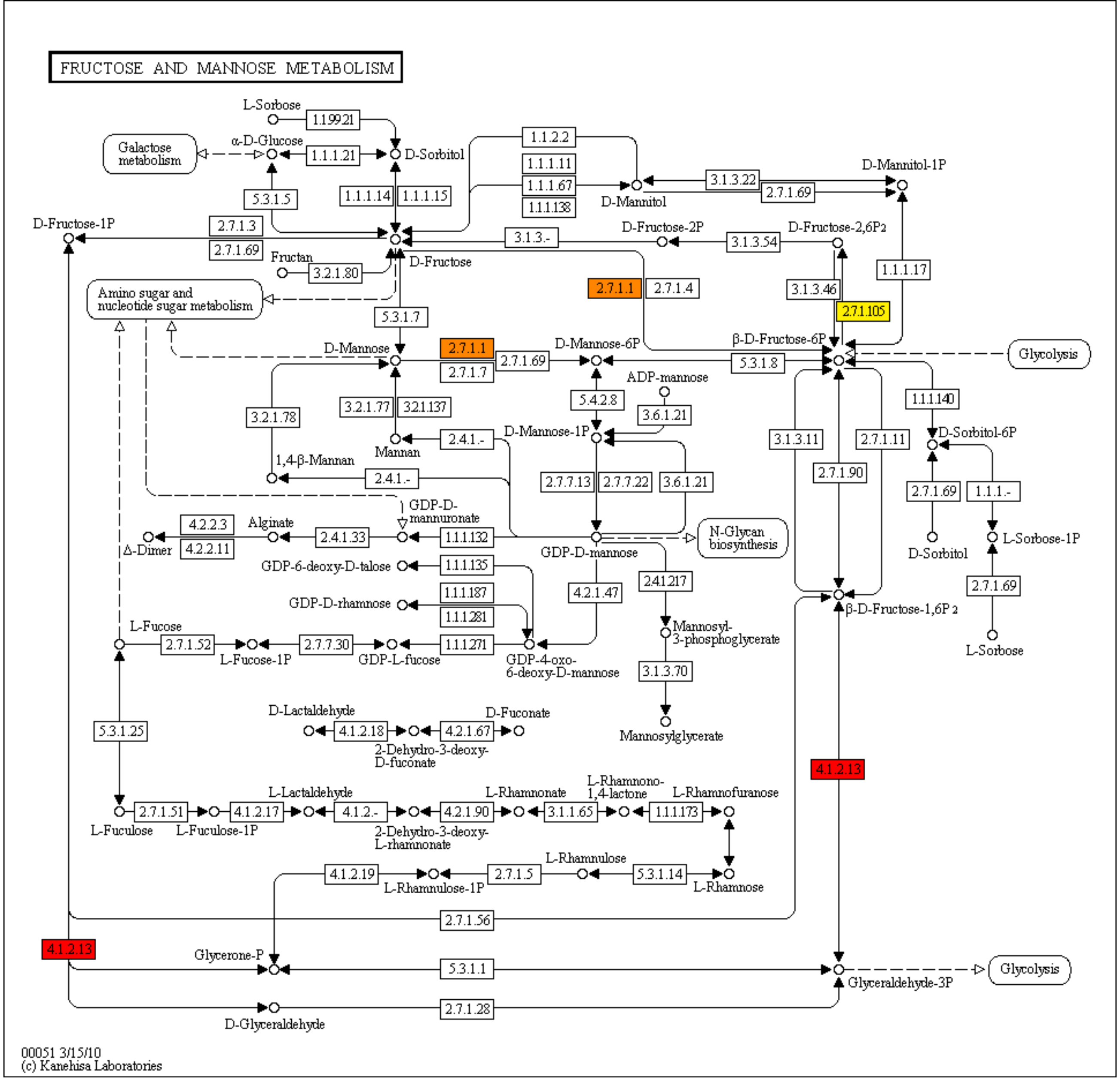

**Colour**	**Enzyme**	**EST clone**
red	ec:4.1.2.13 - fructose-bisphosphate aldolase	TSAY.R35.esd
yellow	ec:2.7.1.105 - 6-phosphofructo-2-kinase	TSBR.R48.esd
orange	ec:2.7.1.1 - hexokinase	TS.seq.screen.trim.Contig224

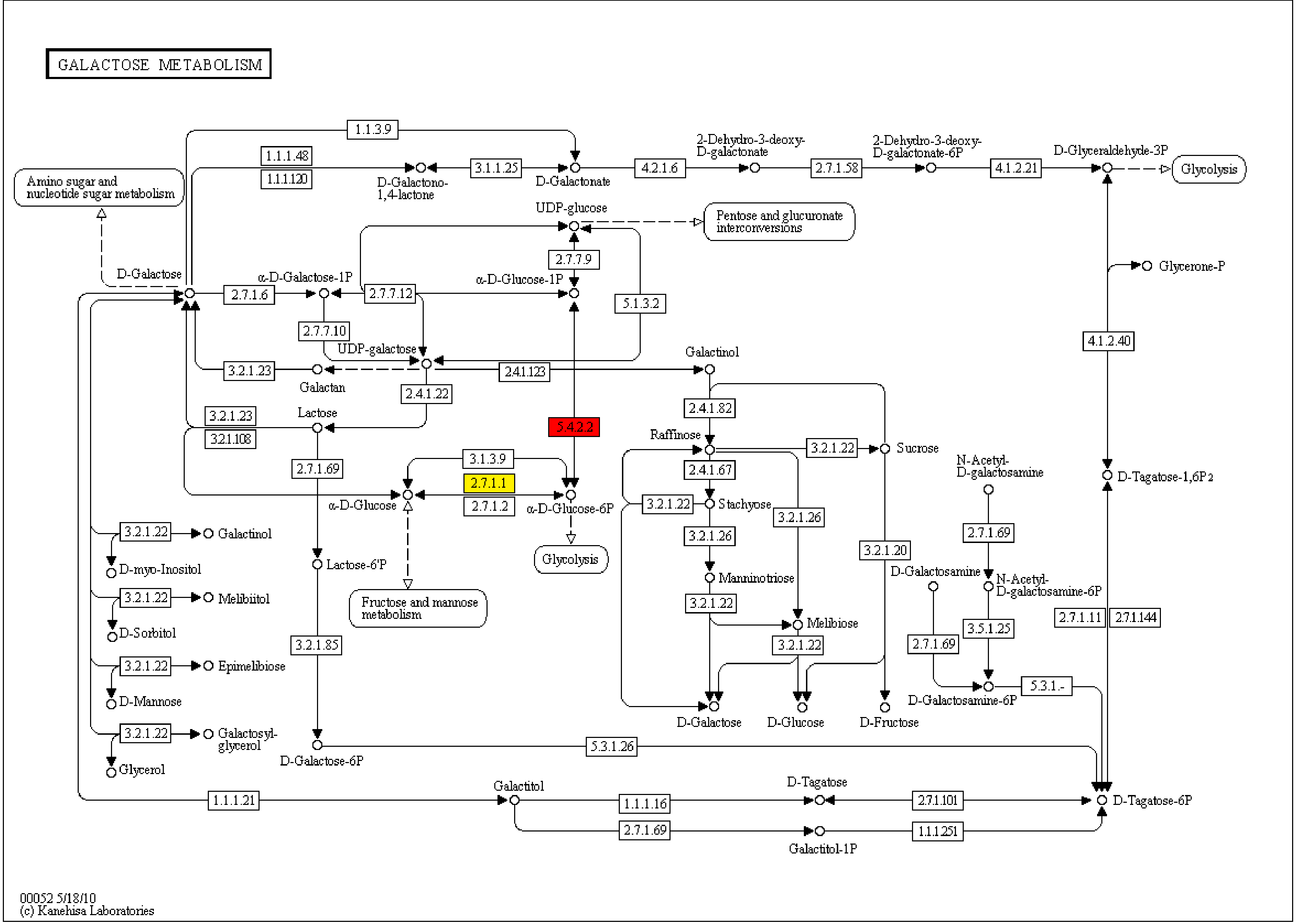

Colour	Enzyme	EST clone
red	ec:5.4.2.2 - phosphoglucomutase	TSBY.R45.esd, TSAX.R47.esd
yellow	ec:2.7.1.1 - hexokinase	TS.seq.screen.trim.Contig224

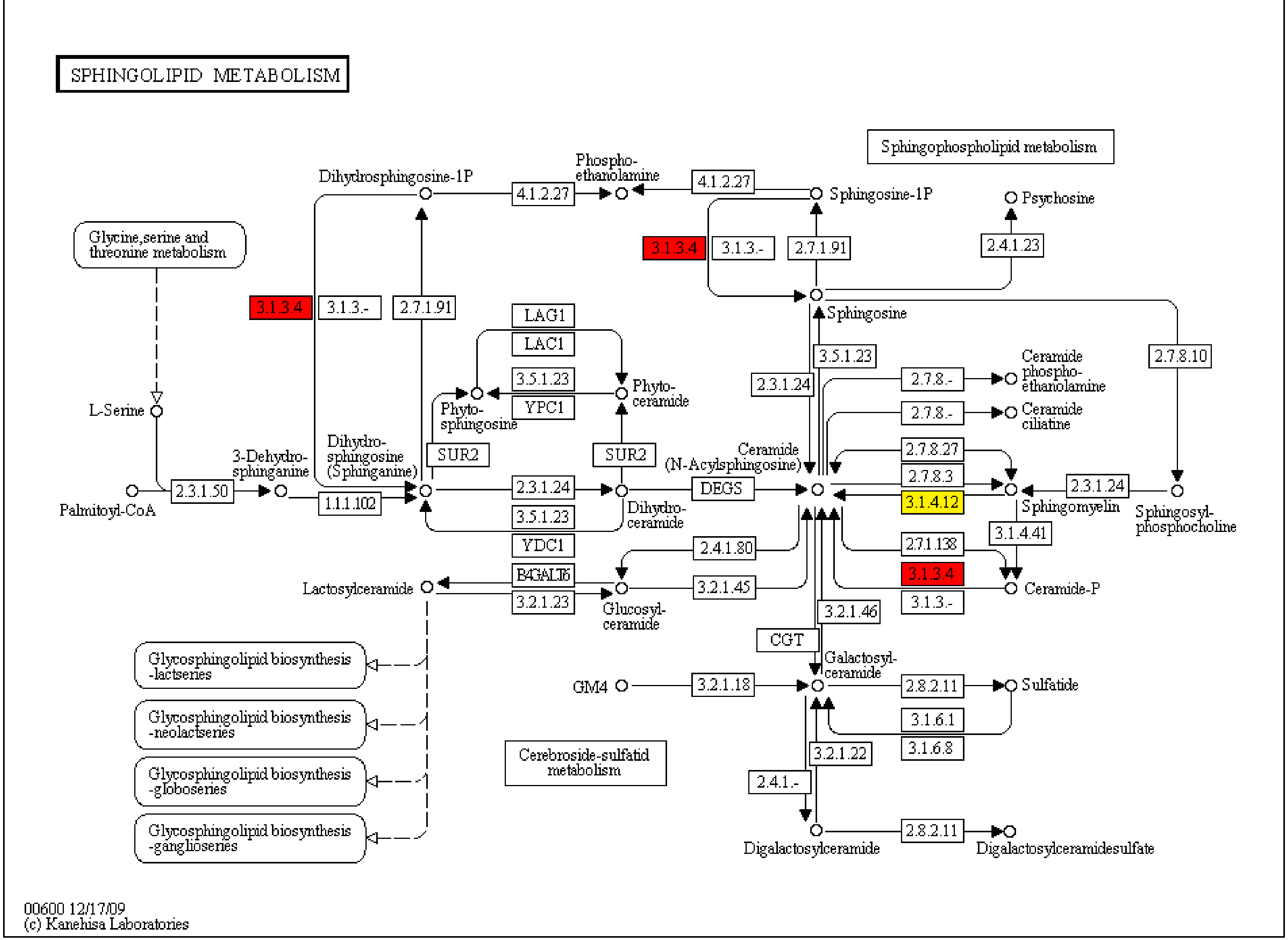

Colour	Enzyme	EST clone
red	ec:3.1.3.4 - phosphatidate phosphatase	TSBQ.R56.esd
yellow	ec:3.1.4.12 - sphingomyelin phosphodiesterase	TS.seq.screen.trim.Contig146, TSAT.R81.esd

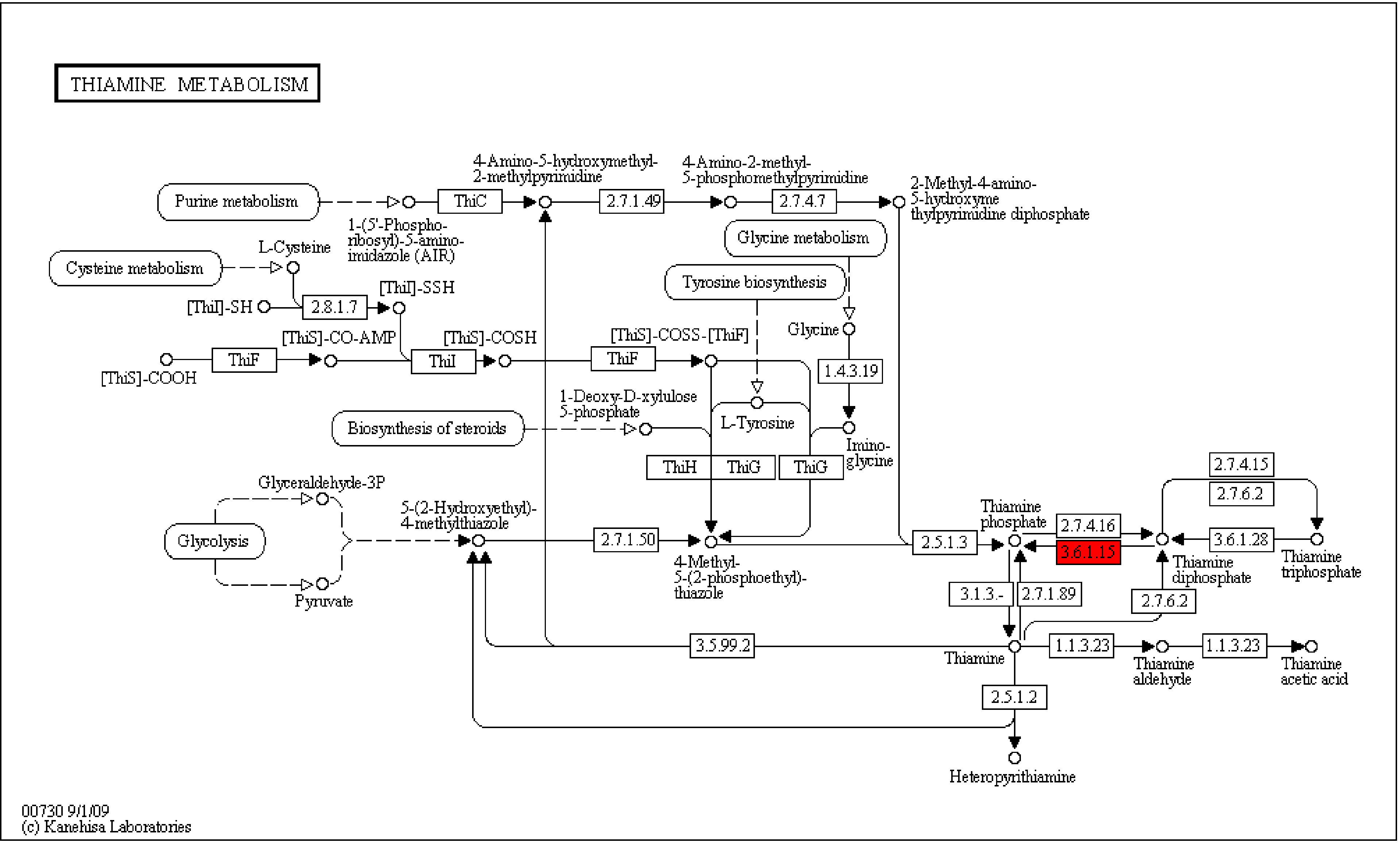

Colour	Enzyme	EST clone
red	ec:3.6.1.15 - nucleoside-triphosphatase	TSAA.R28.esd, TSCC.R43.esd, TSBS.R78.esd

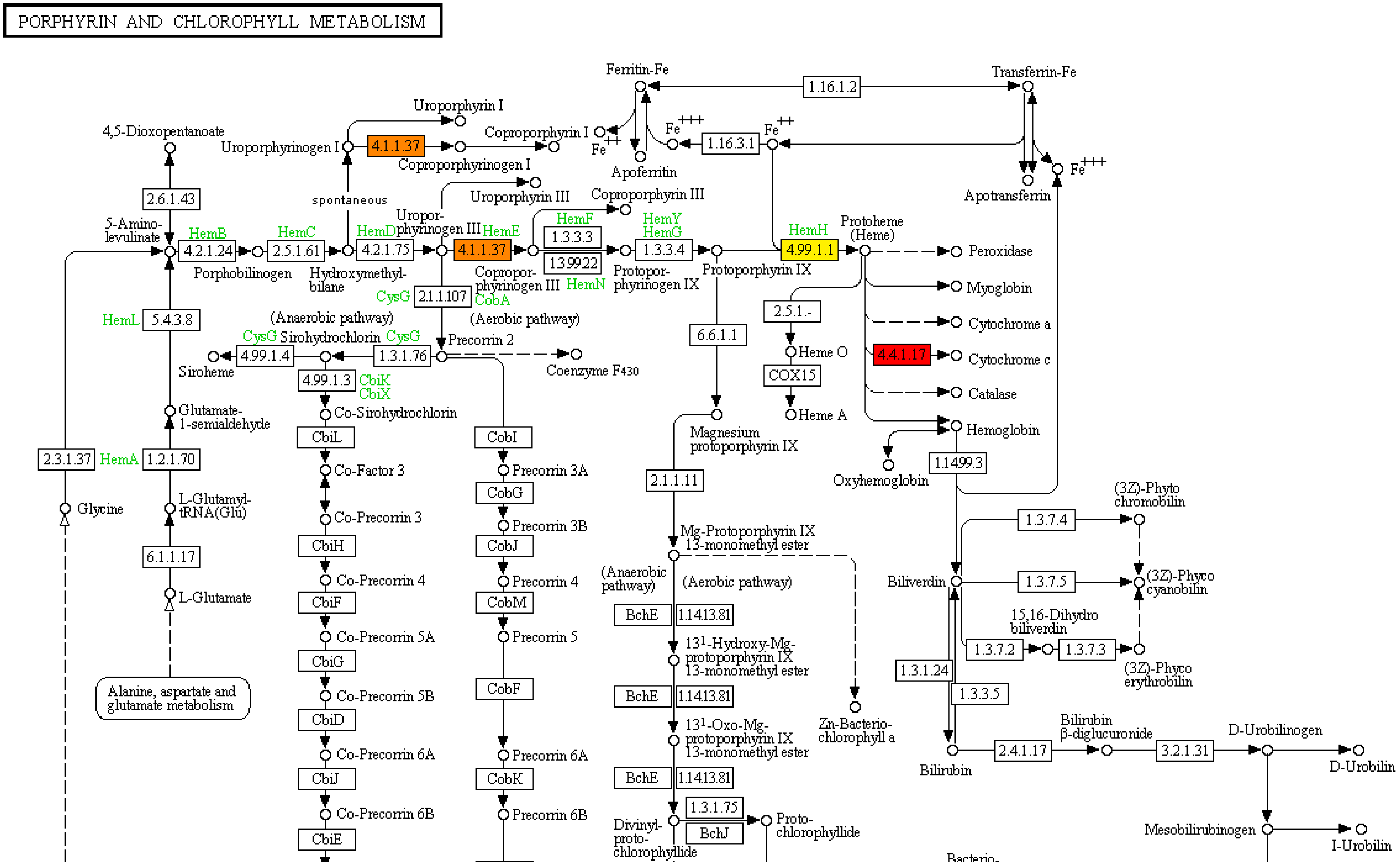

**Colour**	**Enzyme**	**EST clone**
red	ec:4.4.1.17 - holocytochrome-c synthase	TSAX.R60.esd
yellow	ec:4.99.1.1 - ferrochelatase	TSBT.R96.esd
orange	ec:4.1.1.37 - uroporphyrinogen decarboxylase	TSAO.R70.esd

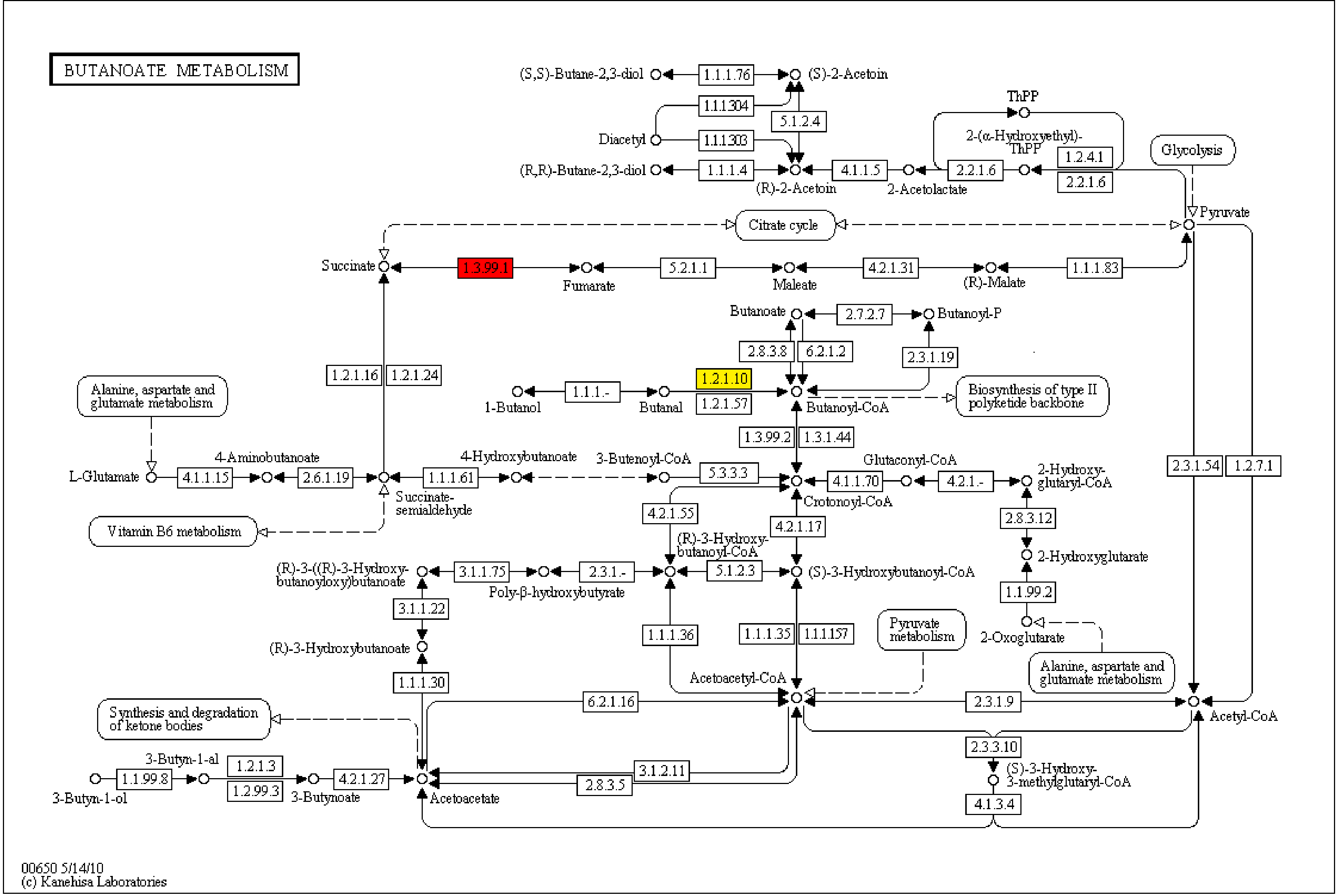

Colour	Enzyme	EST clone
red	ec:1.3.99.1 - succinate dehydrogenase	TS.seq.screen.trim.Contig279
yellow	ec:1.2.1.10 - acetaldehyde dehydrogenase (acetylating)	TSCE.R92.esd

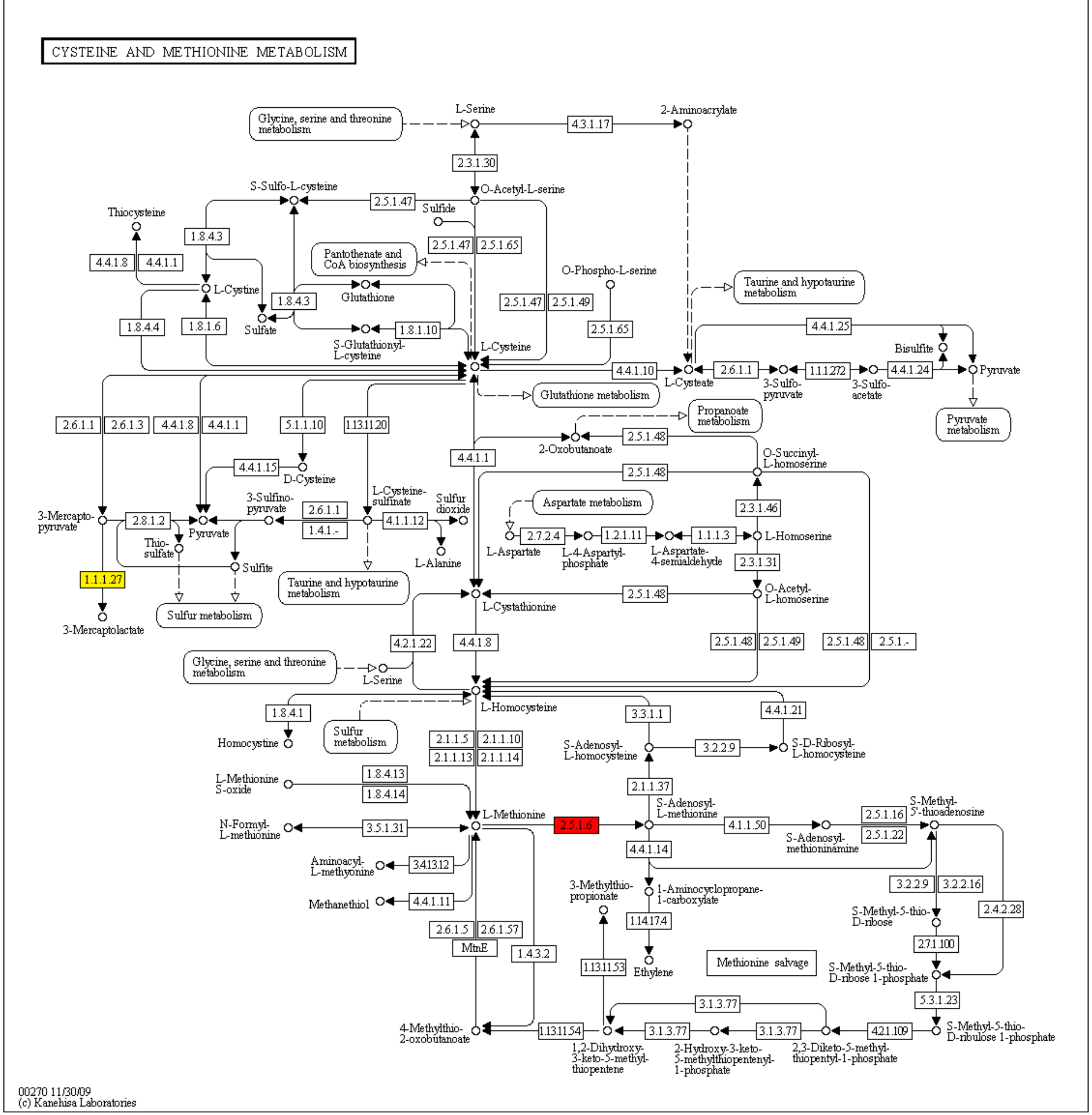

Colour	Enzyme	EST clone
red	ec:2.5.1.6 - methionine adenosyltransferase	TSAT.R84.esd
yellow	ec:1.1.1.27 - L-lactate dehydrogenase	TS.seq.screen.trim.Contig434

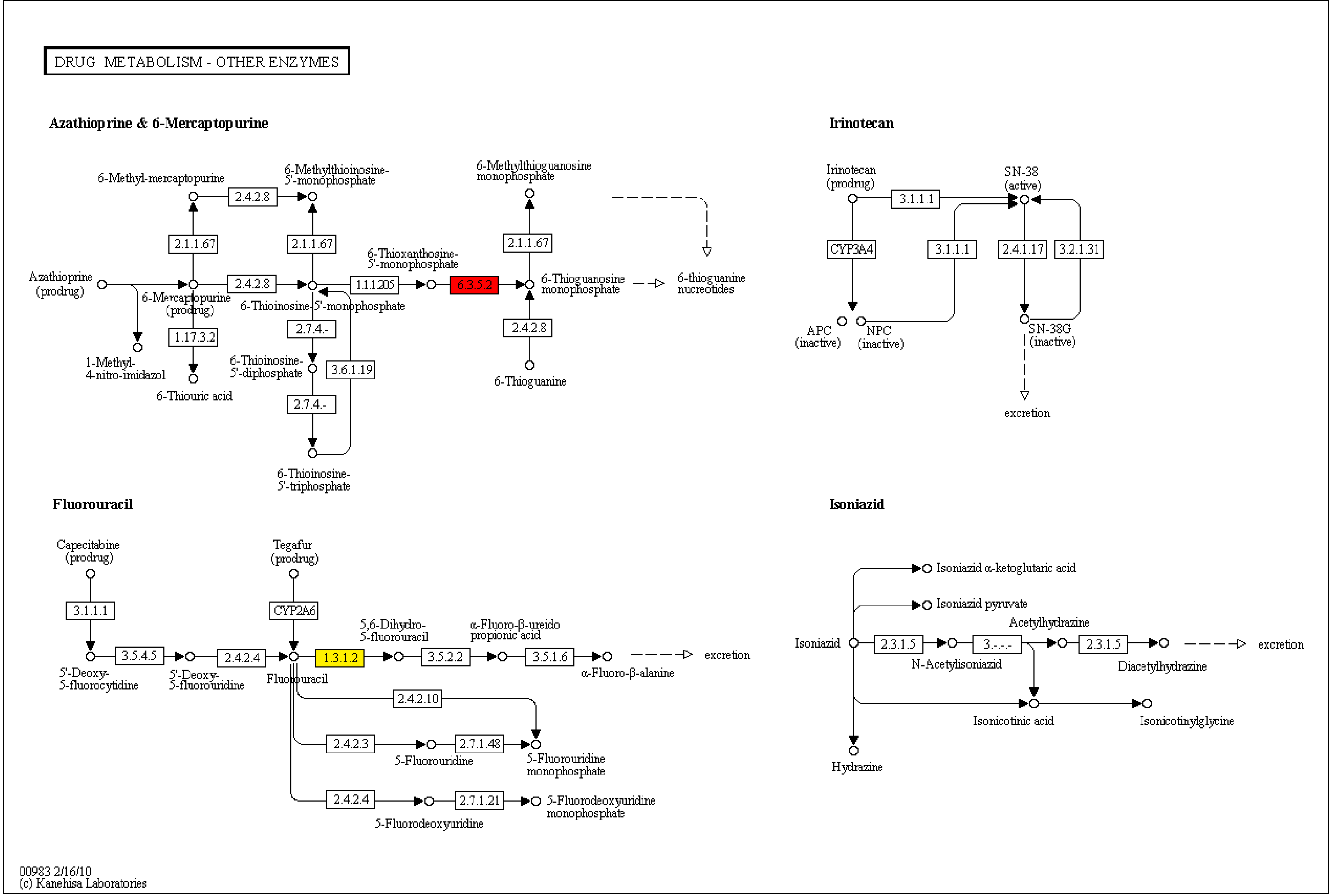

**Colour**	**Enzyme**	**EST clone**
red	ec:6.3.5.2 - GMP synthase (glutamine-hydrolysing)	TS.seq.screen.trim.Contig177
yellow	ec:1.3.1.2 - dihydropyrimidine dehydrogenase (NADP+)	TSCF.R86.esd

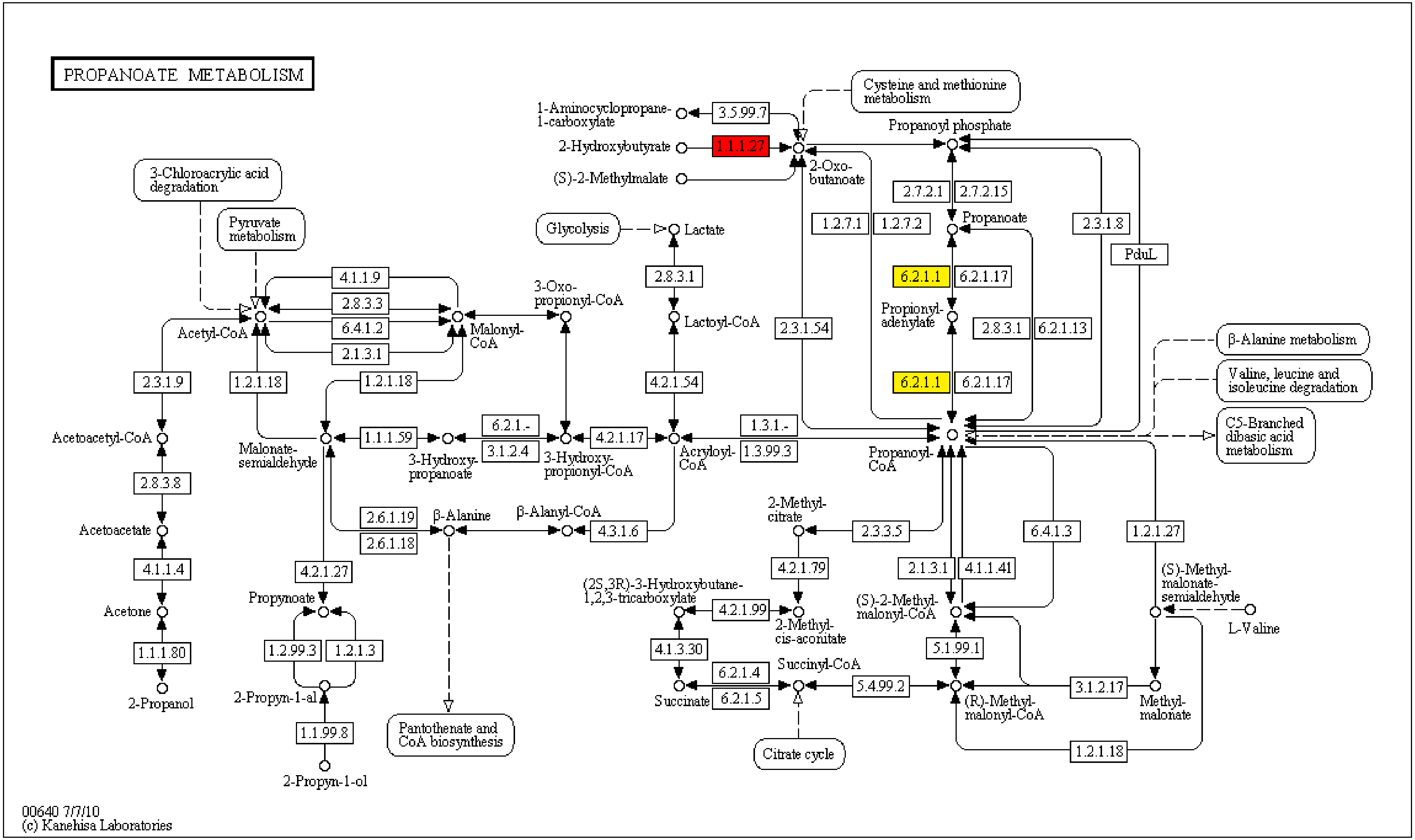

Colour	Enzyme	EST clone
red	ec:1.1.1.27 - L-lactate dehydrogenase	TS.seq.screen.trim.Contig434
yellow	ec:6.2.1.1 - acetate---CoA ligase	TS.seq.screen.trim.Contig7

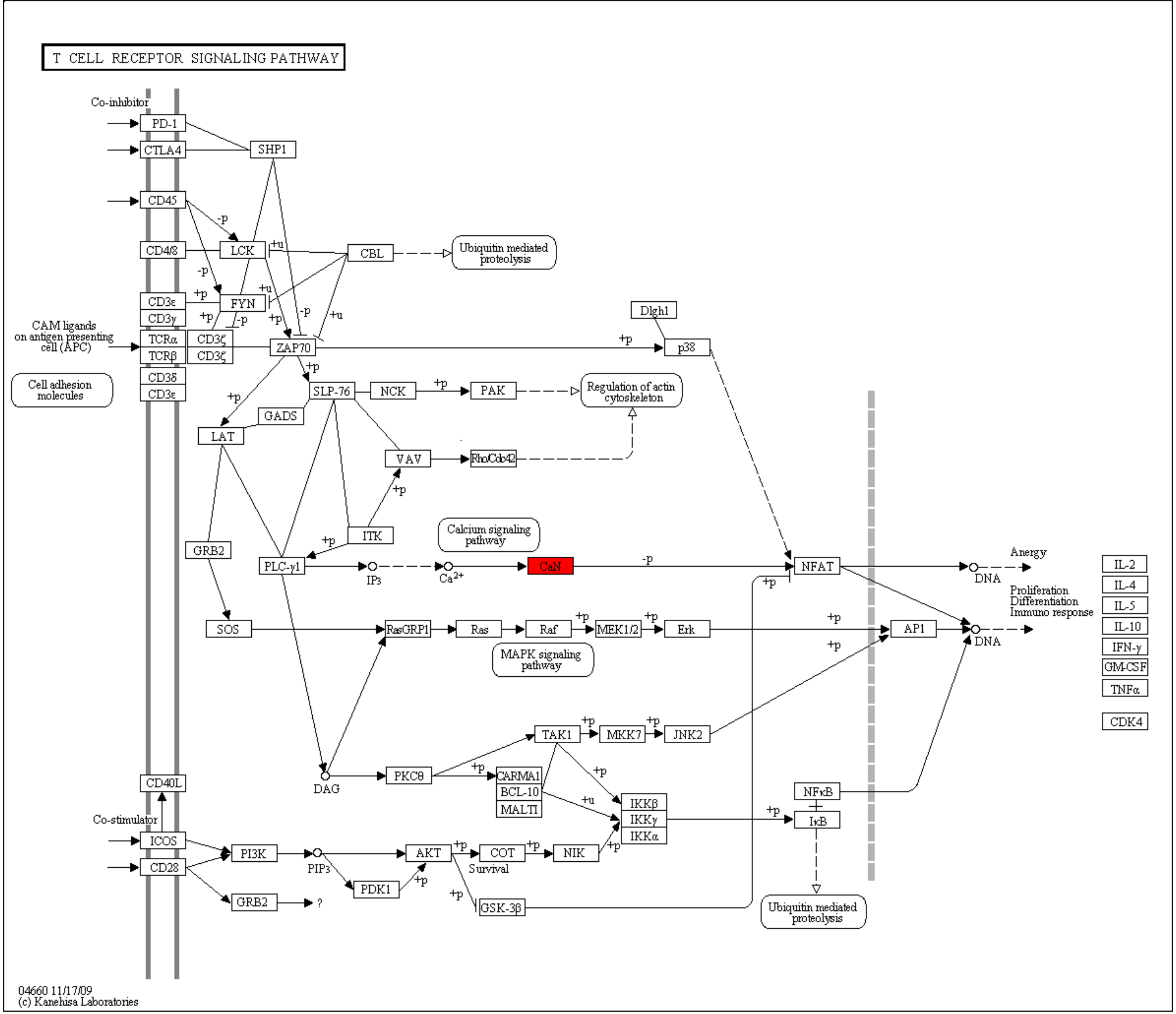

Colour	Enzyme	EST clone
red	ec:3.1.3.16 - phosphoprotein phosphatase	TS.seq.screen.trim.Contig350, TSBT.R18.esd

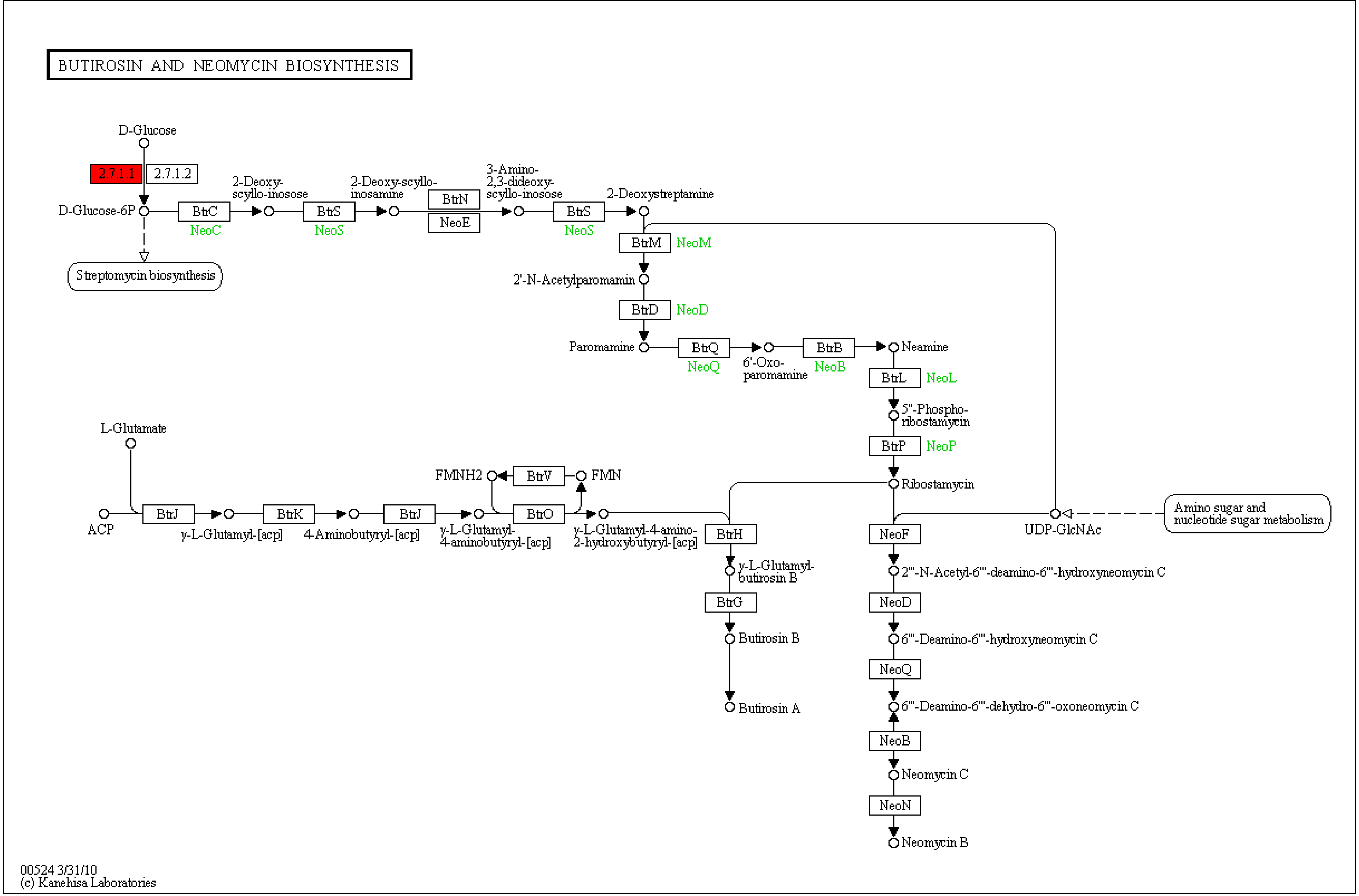

**Colour**	**Enzyme**	**EST clone**
red	ec:2.7.1.1 - hexokinase	TS.seq.screen.trim.Contig224

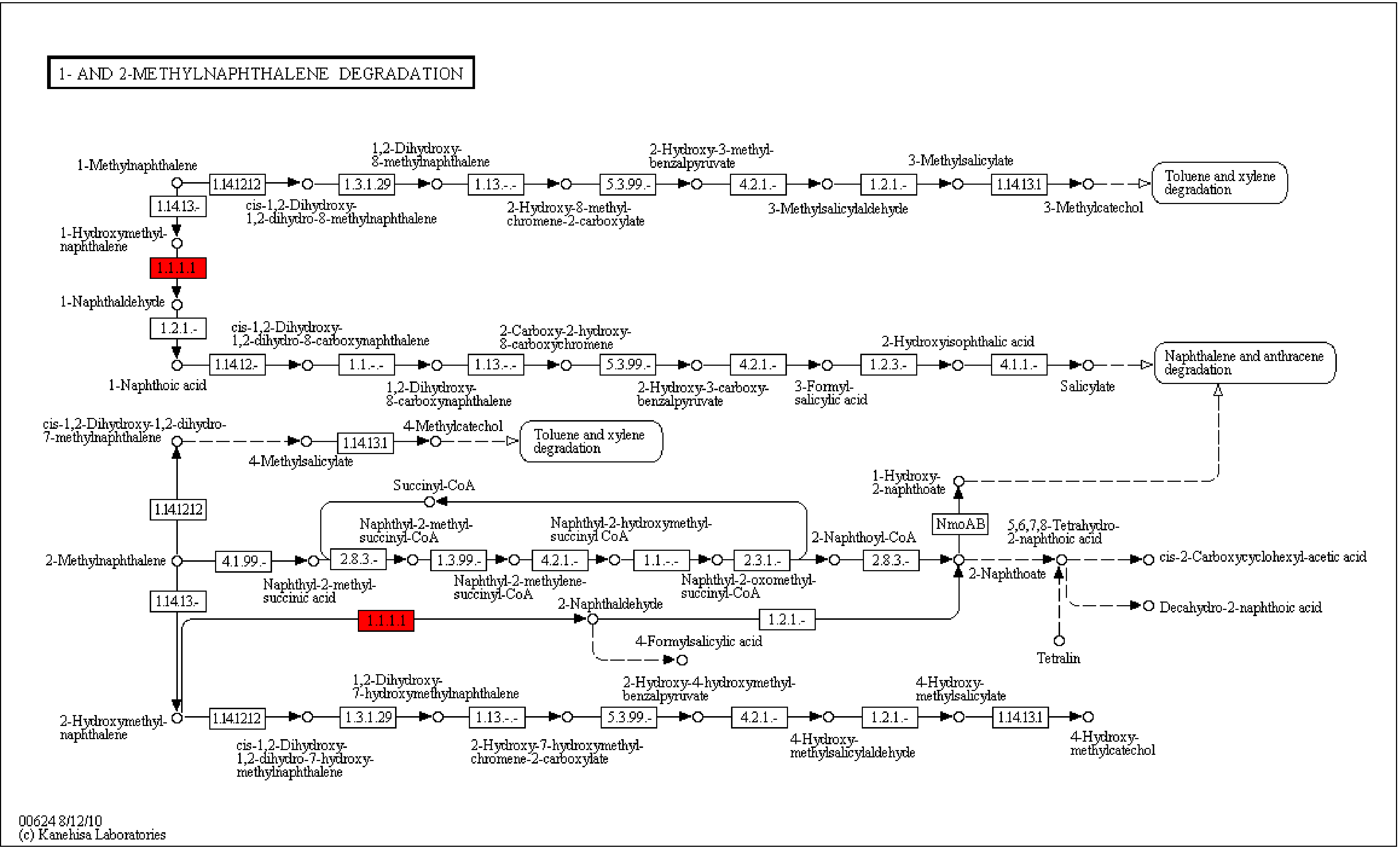

Colour	Enzyme	EST clone
red	ec:1.1.1.1 - alcohol dehydrogenase	TSCE.R92.esd

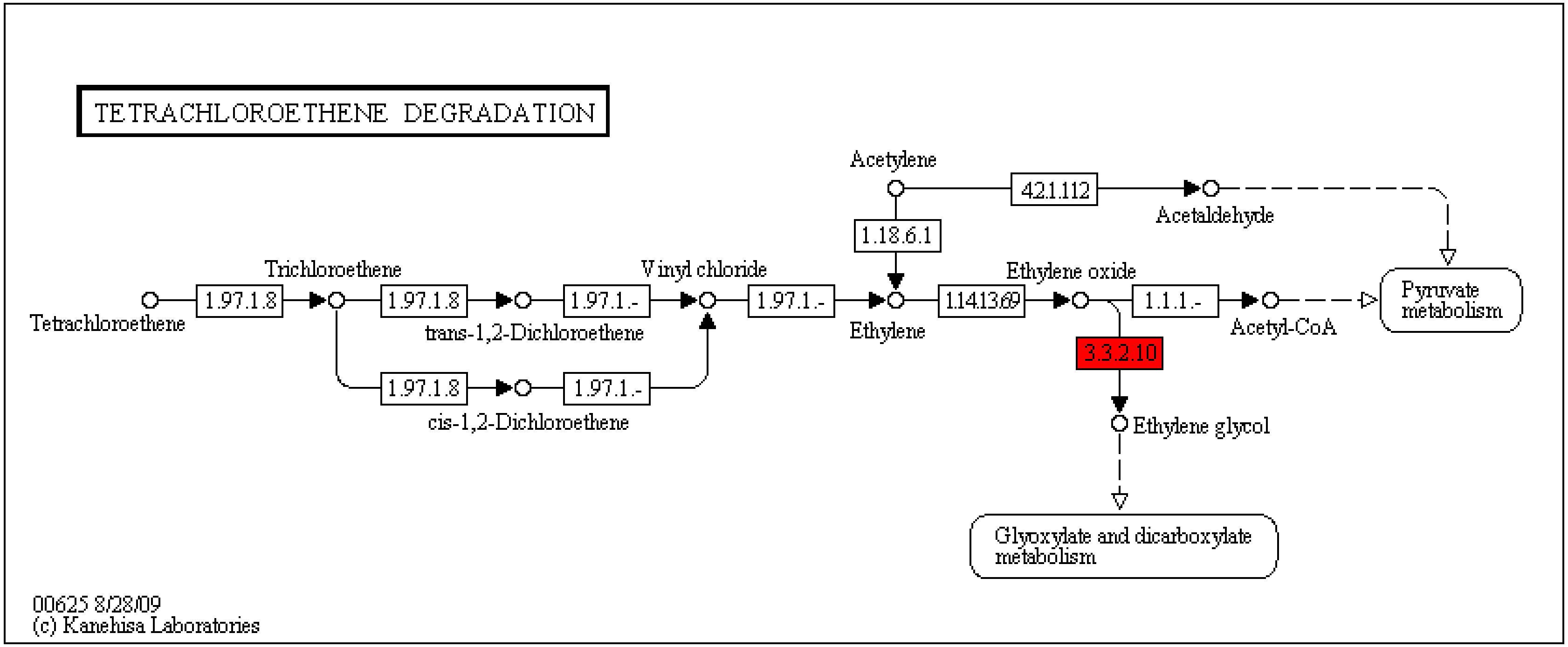

Colour	Enzyme	EST clone
red	ec:3.3.2.10 - soluble epoxide hydrolase	TS.seq.screen.trim.Contig283

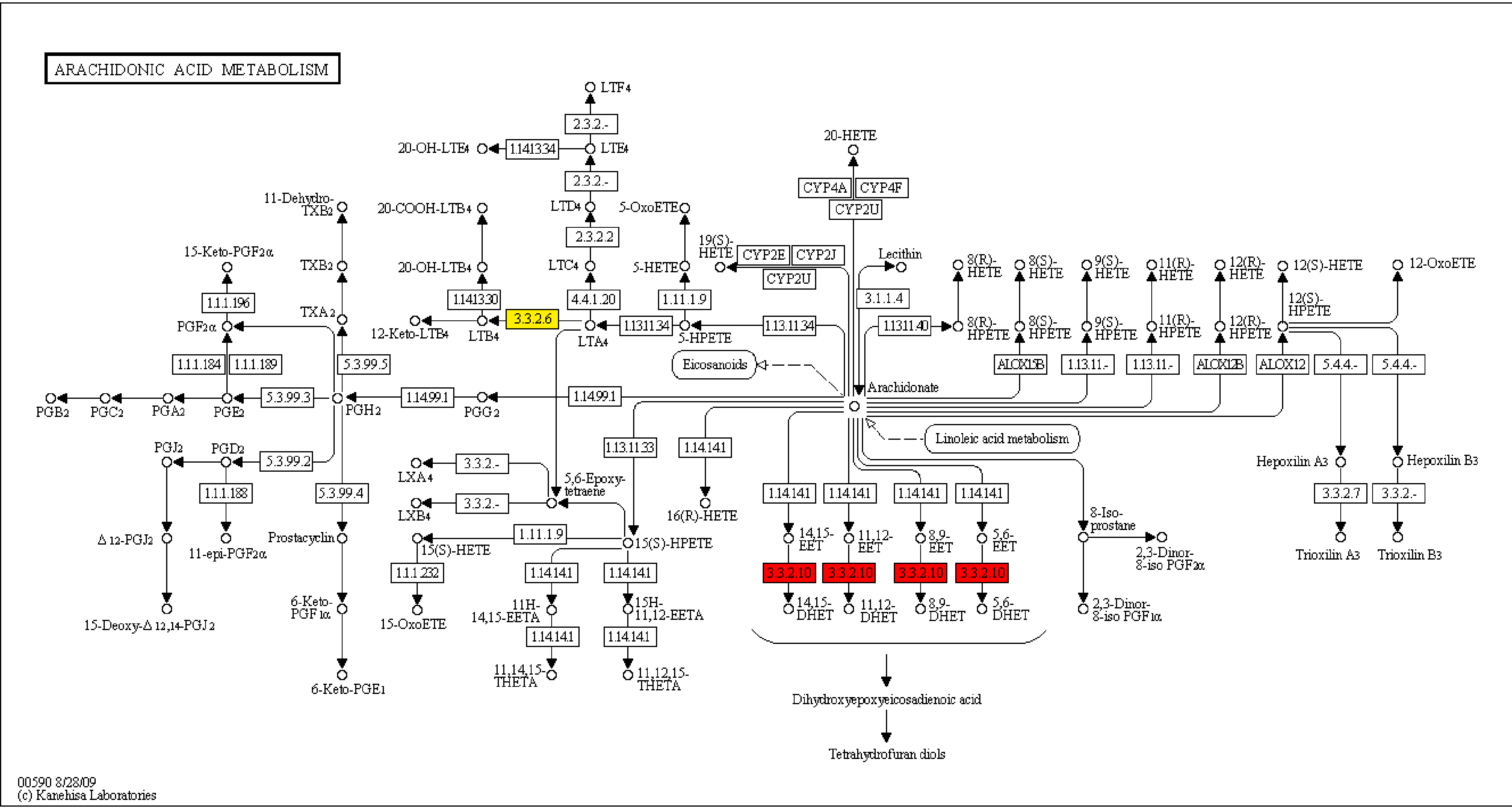

Colour	Enzyme	EST clone
red	ec:3.3.2.10 - soluble epoxide hydrolase	TS.seq.screen.trim.Contig283
yellow	ec:3.3.2.6 - leukotriene-A4 hydrolase	TS.seq.screen.trim.Contig283

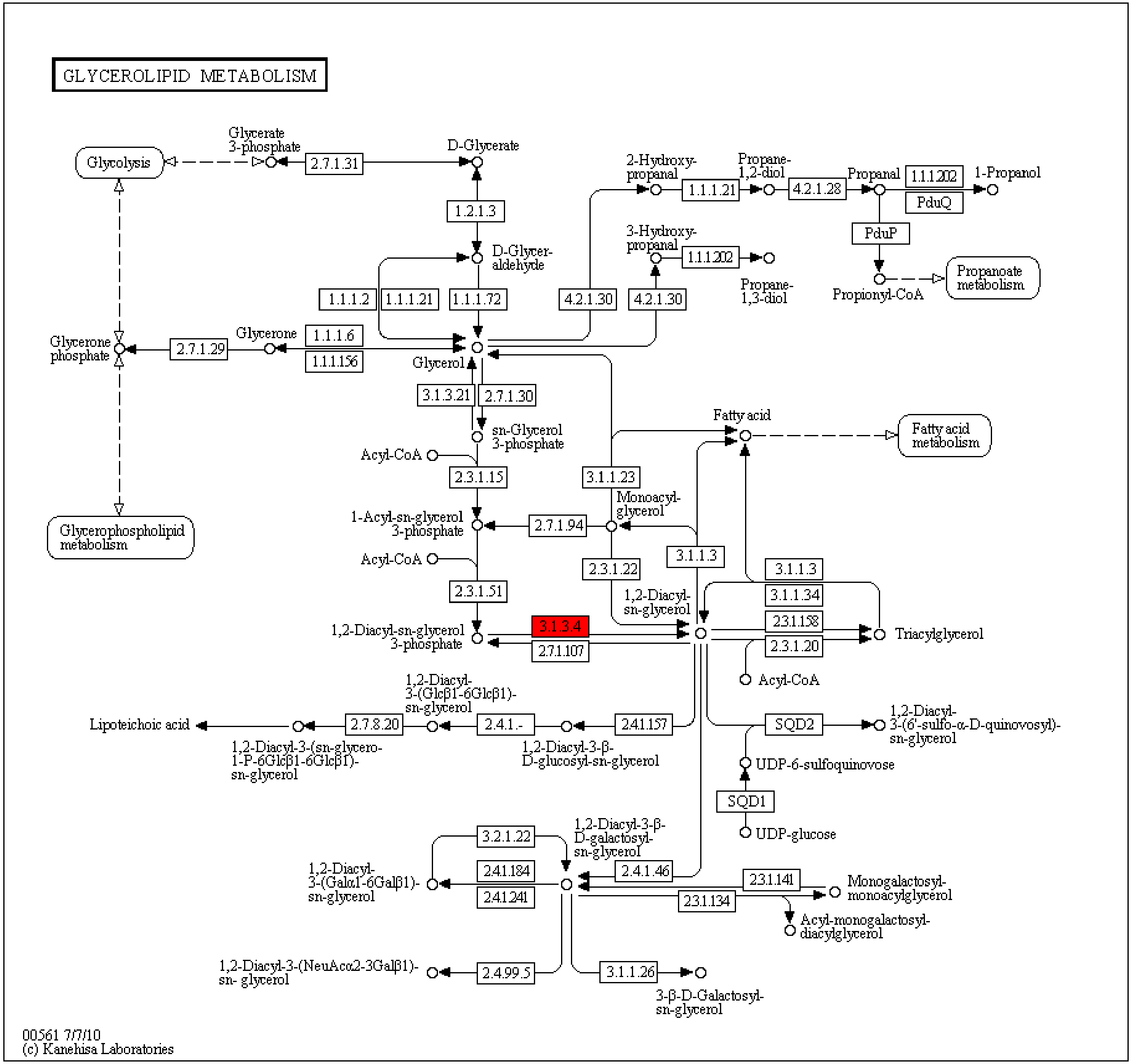

Colour	Enzyme	EST clone
red	ec:3.1.3.4 - phosphatidate phosphatase	TSBQ.R56.esd

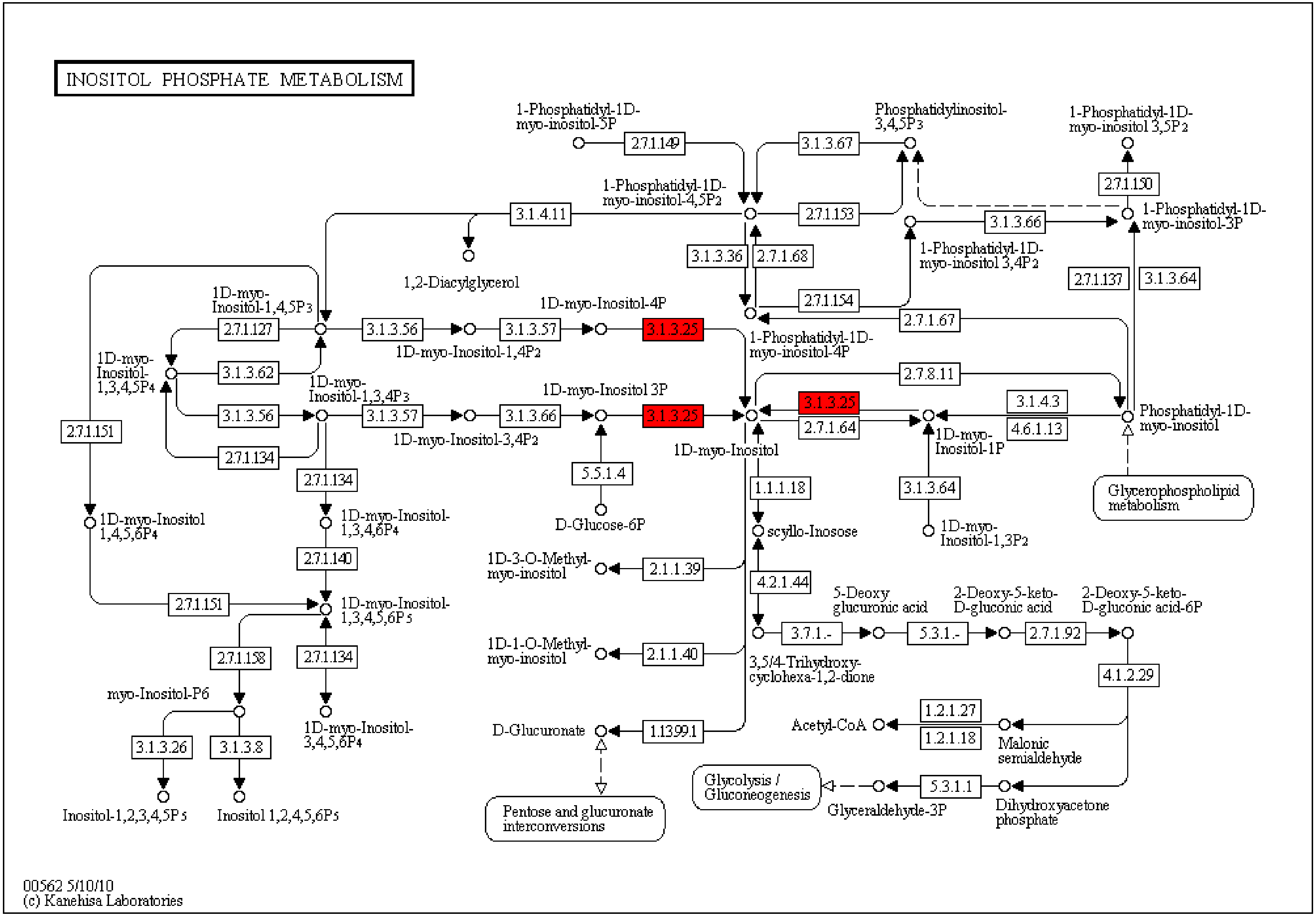

**Colour**	**Enzyme**	**EST clone**
red	ec:3.1.3.25 - inositol-phosphate phosphatase	TS.seq.screen.trim.Contig284

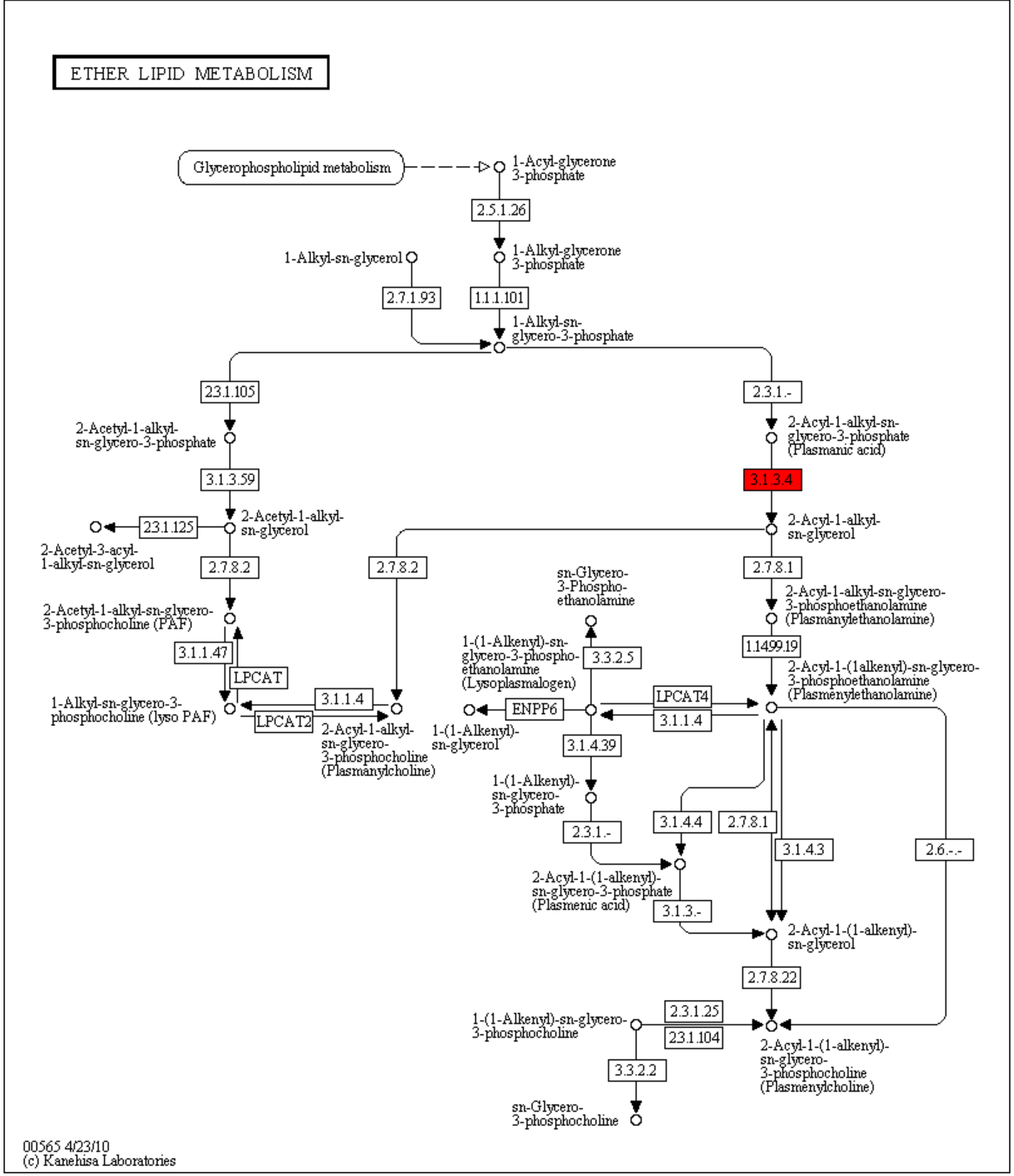

**Colour**	**Enzyme**	**EST clone**
red	ec:3.1.3.4 - phosphatidate phosphatase	TSBQ.R56.esd

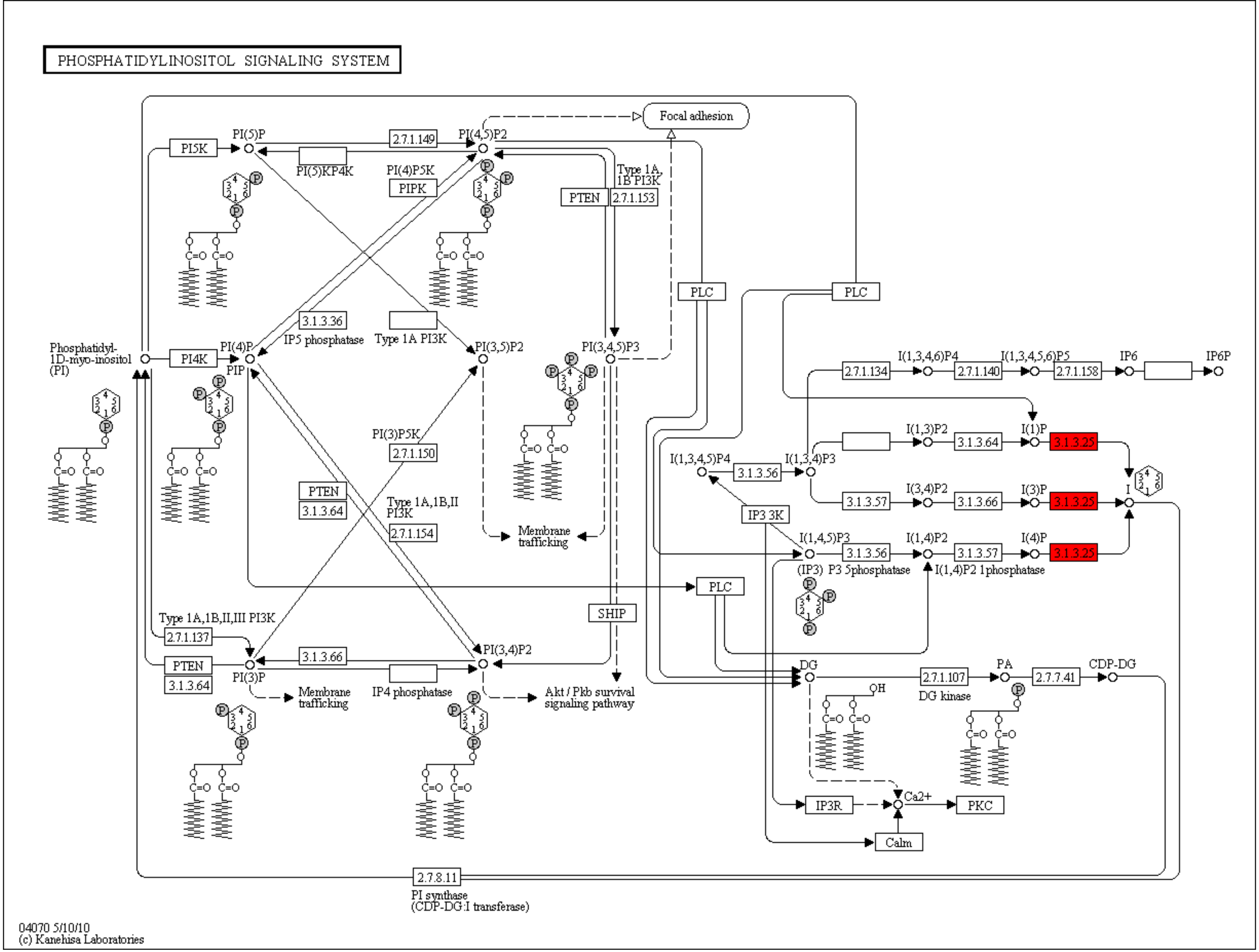

Colour	Enzyme	EST clone
red	ec:3.1.3.25 - inositol-phosphate phosphatase	TS.seq.screen.trim.Contig284

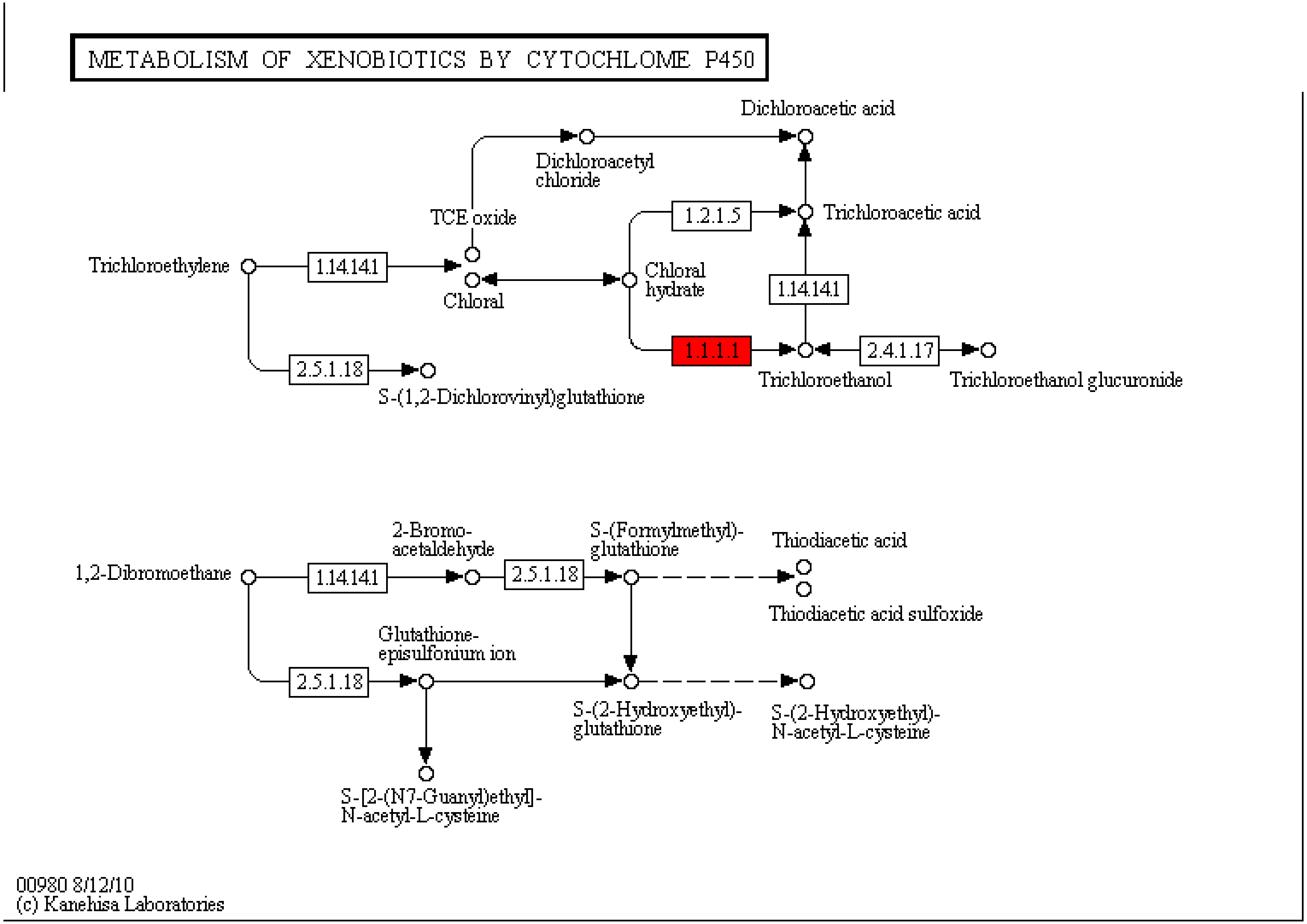

Colour	Enzyme	EST clone
red	ec:1.1.1.1 - alcohol dehydrogenase	TSCE.R92.esd

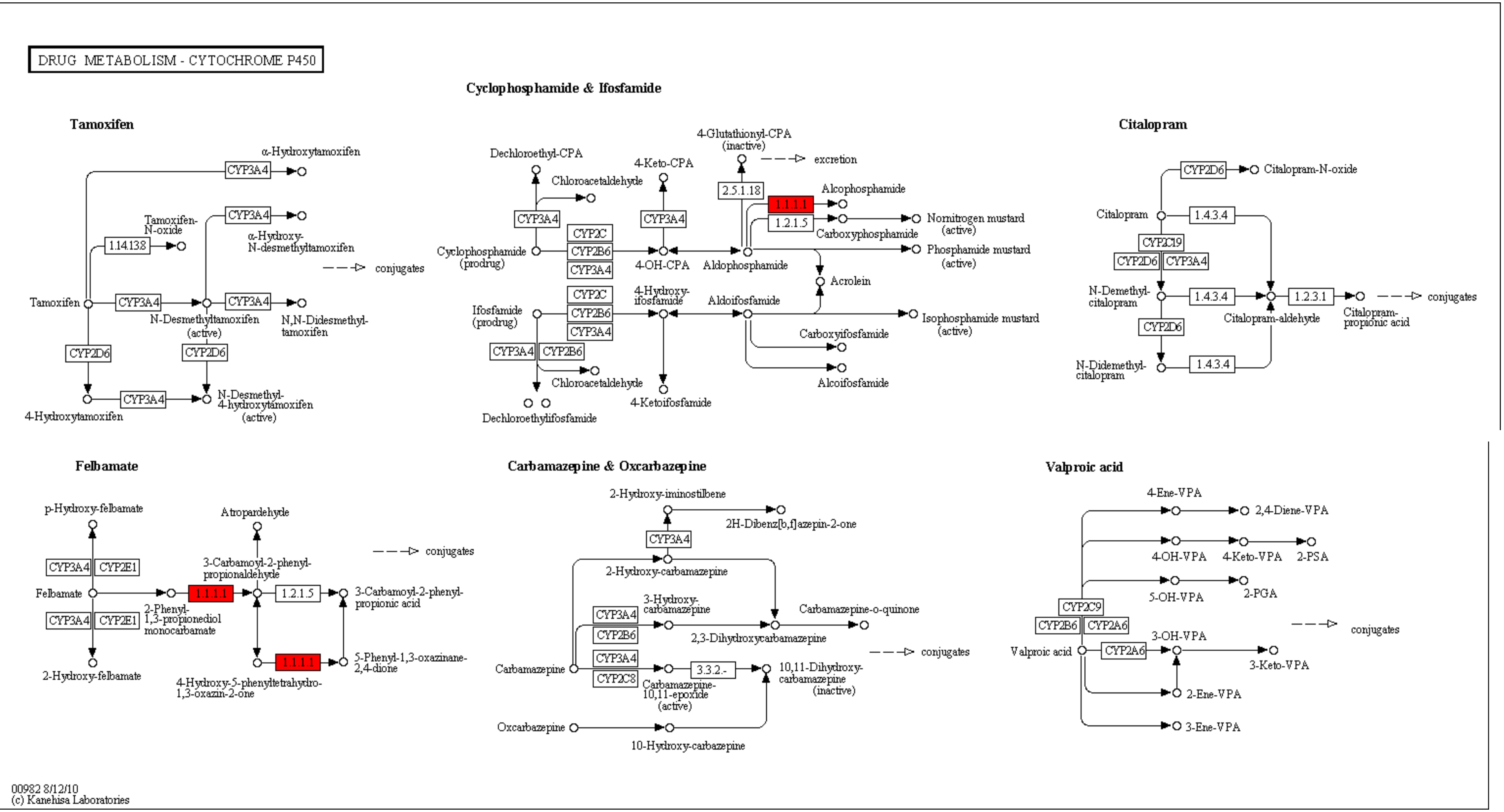

Colour	Enzyme	EST clone
red	ec:1.1.1.1 - alcohol dehydrogenase	TSCE.R92.esd

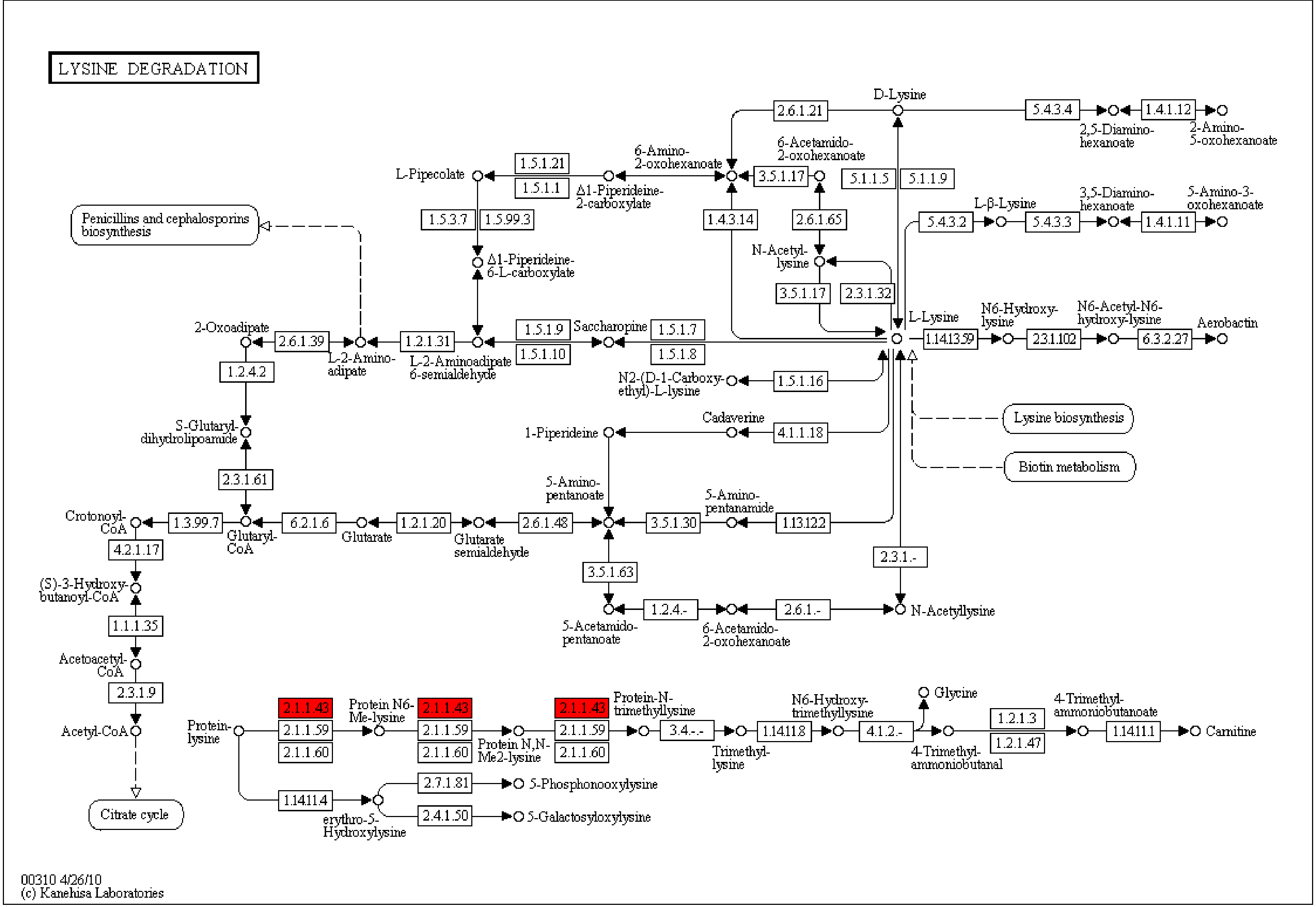

Colour	Enzyme	EST clone
red	ec:2.1.1.43 - histone-lysine N-methyltransferase	TSAR.R23.esd

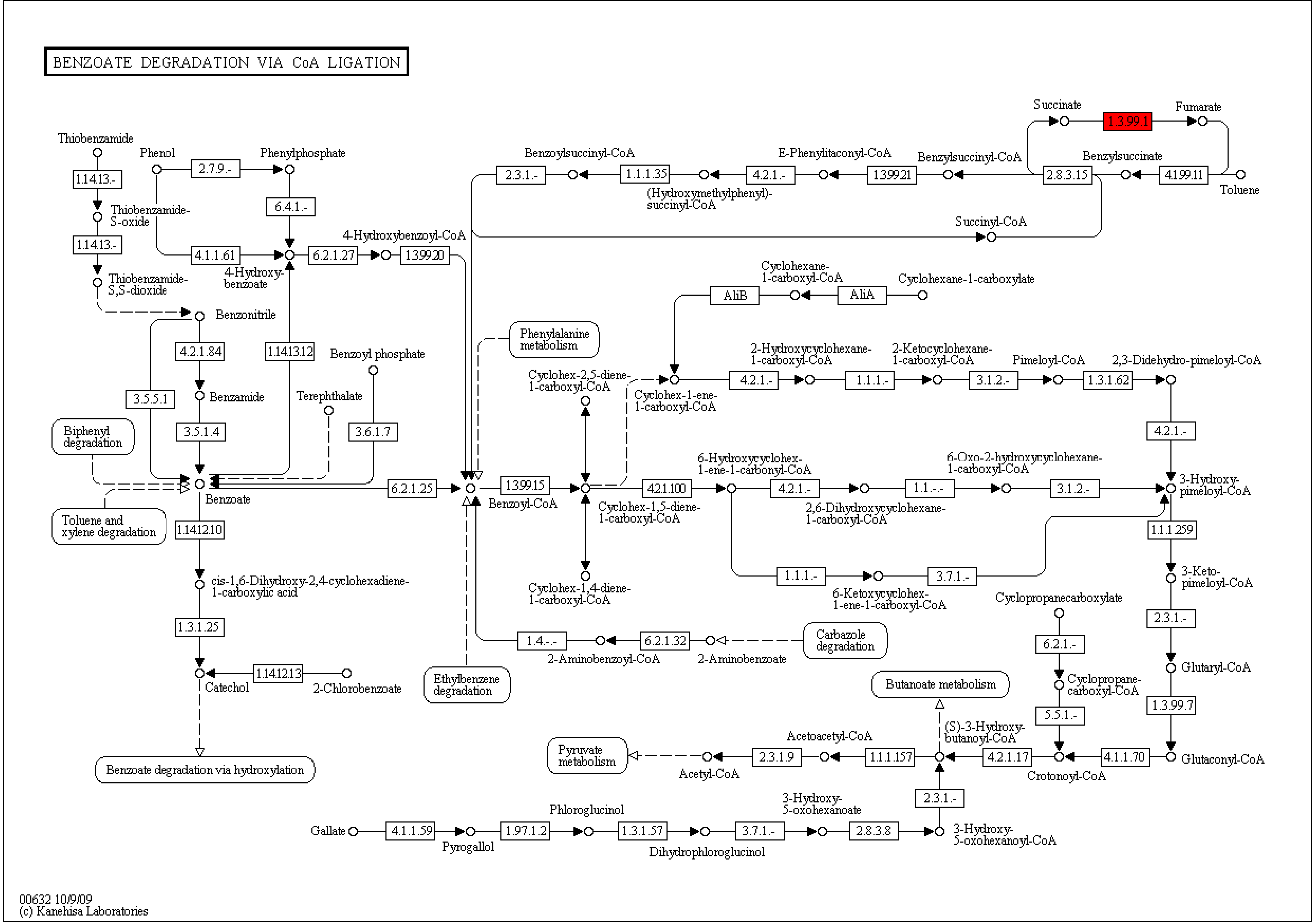

Colour	Enzyme	EST clone
red	ec:1.3.99.1 - succinate dehydrogenase	TS.seq.screen.trim.Contig279

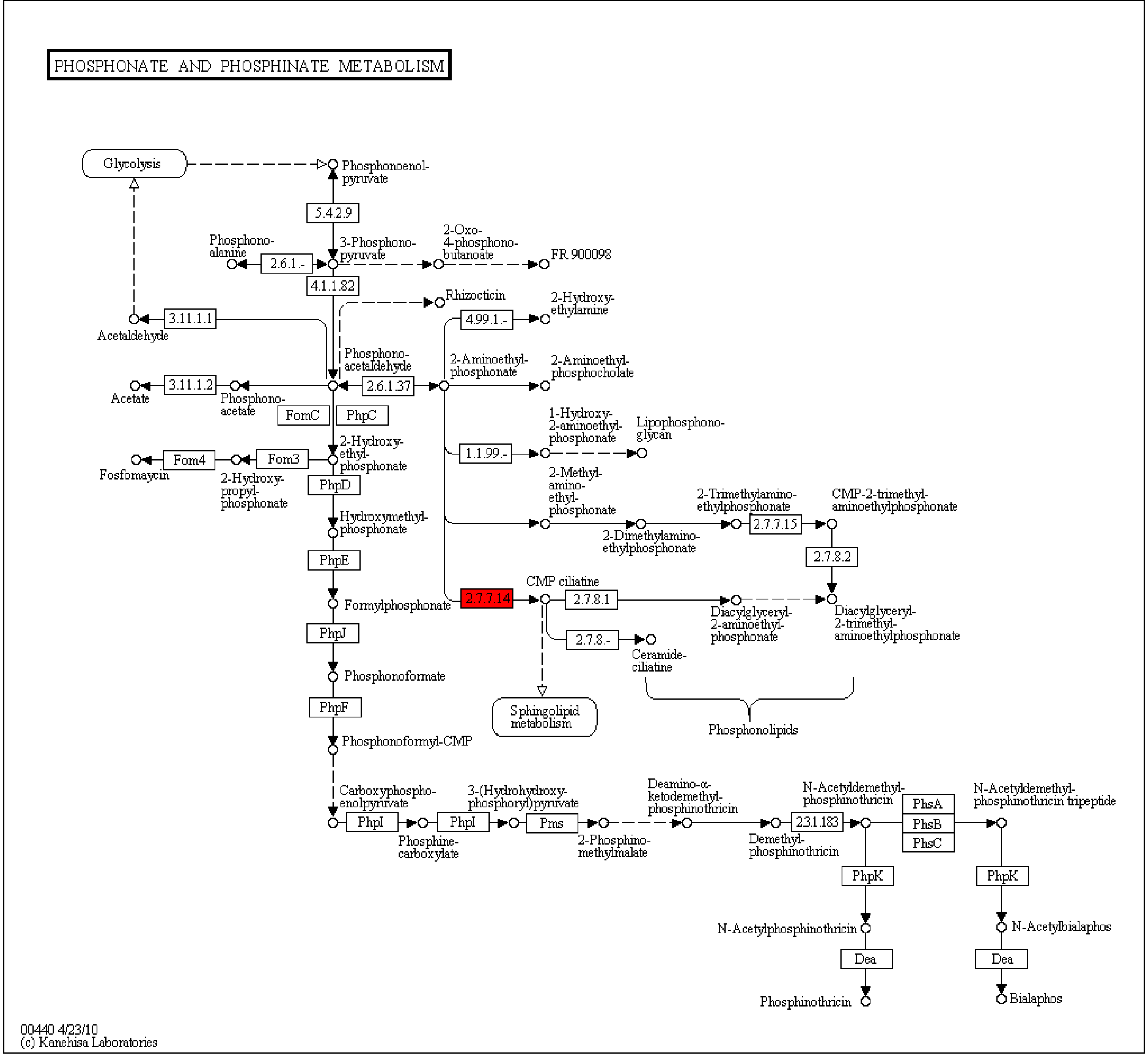

Colour	Enzyme	EST clone
red	ec:2.7.7.14 - ethanolamine-phosphate cytidylyltransferase	TSBP.R20.esd

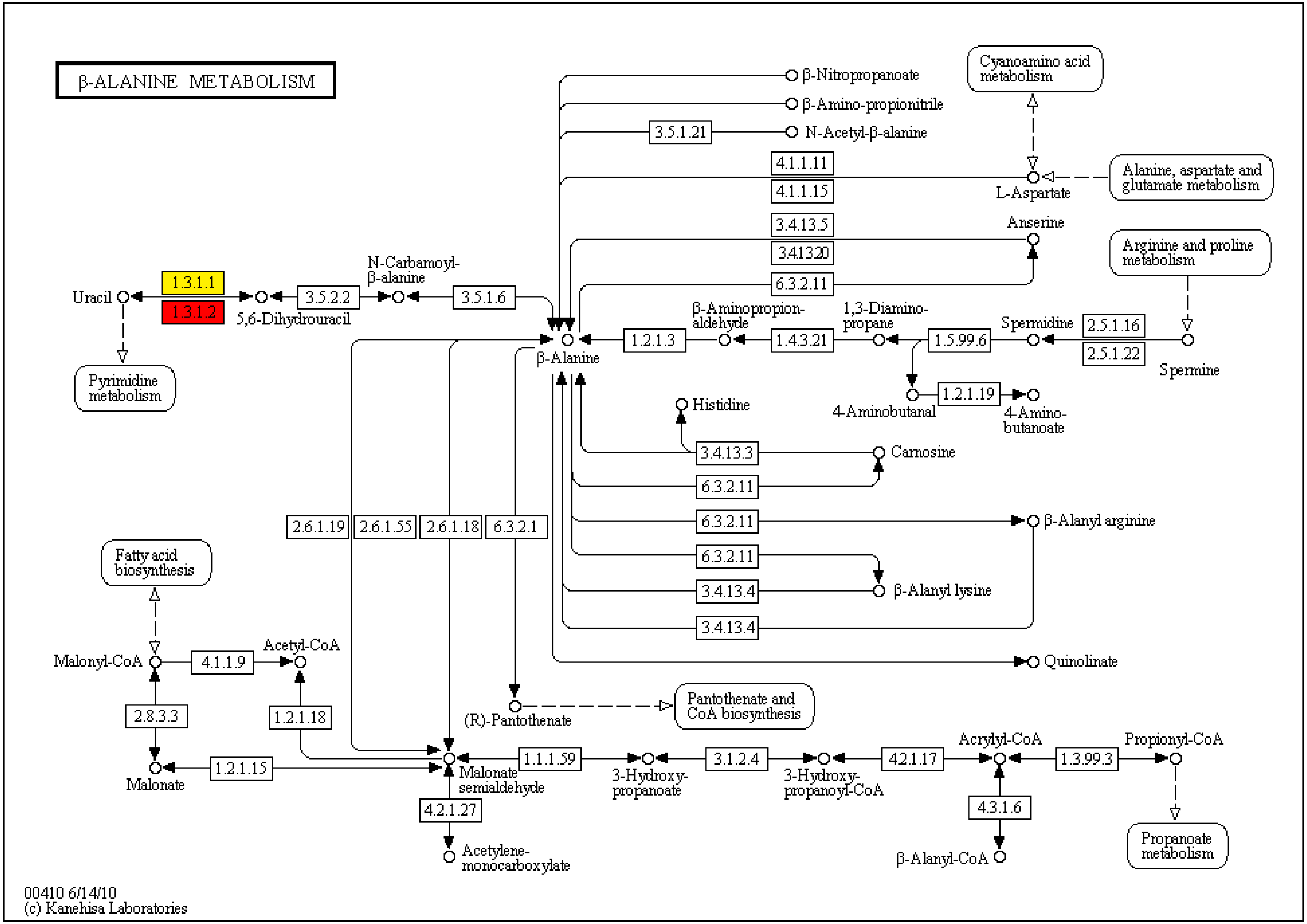

Colour	Enzyme	EST clone
red	ec:1.3.1.2 - dihydropyrimidine dehydrogenase (NADP+)	TSCF.R86.esd
yellow	ec:1.3.1.1 - dihydrouracil dehydrogenase (NAD+)	TSCF.R86.esd

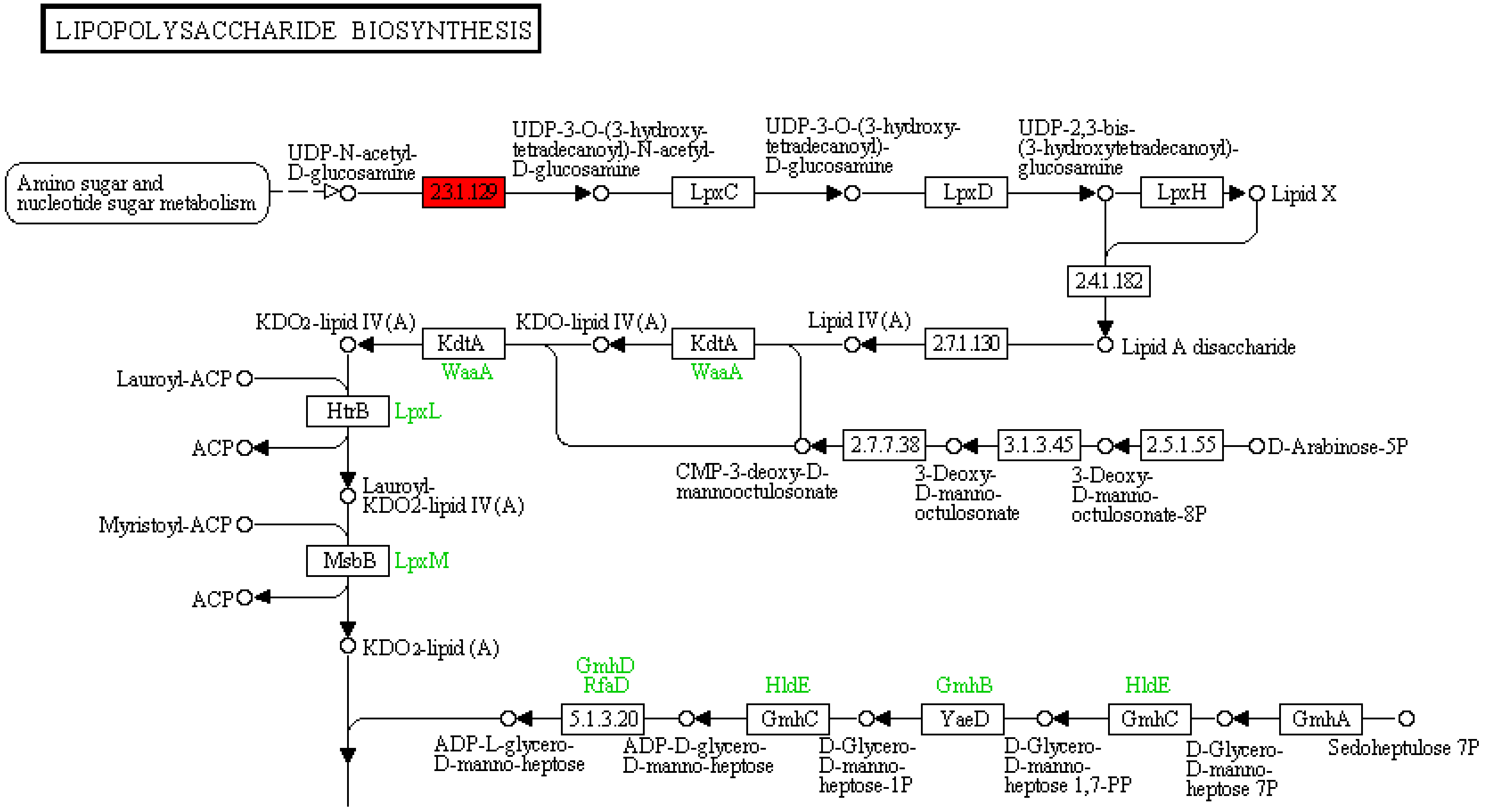

Colour	Enzyme	EST clone
red	ec:2.3.1.129 - acyl-[acyl-carrier-protein]---UDP-N-acetylglucosamine O-acyltransferase	TS.seq.screen.trim.Contig169

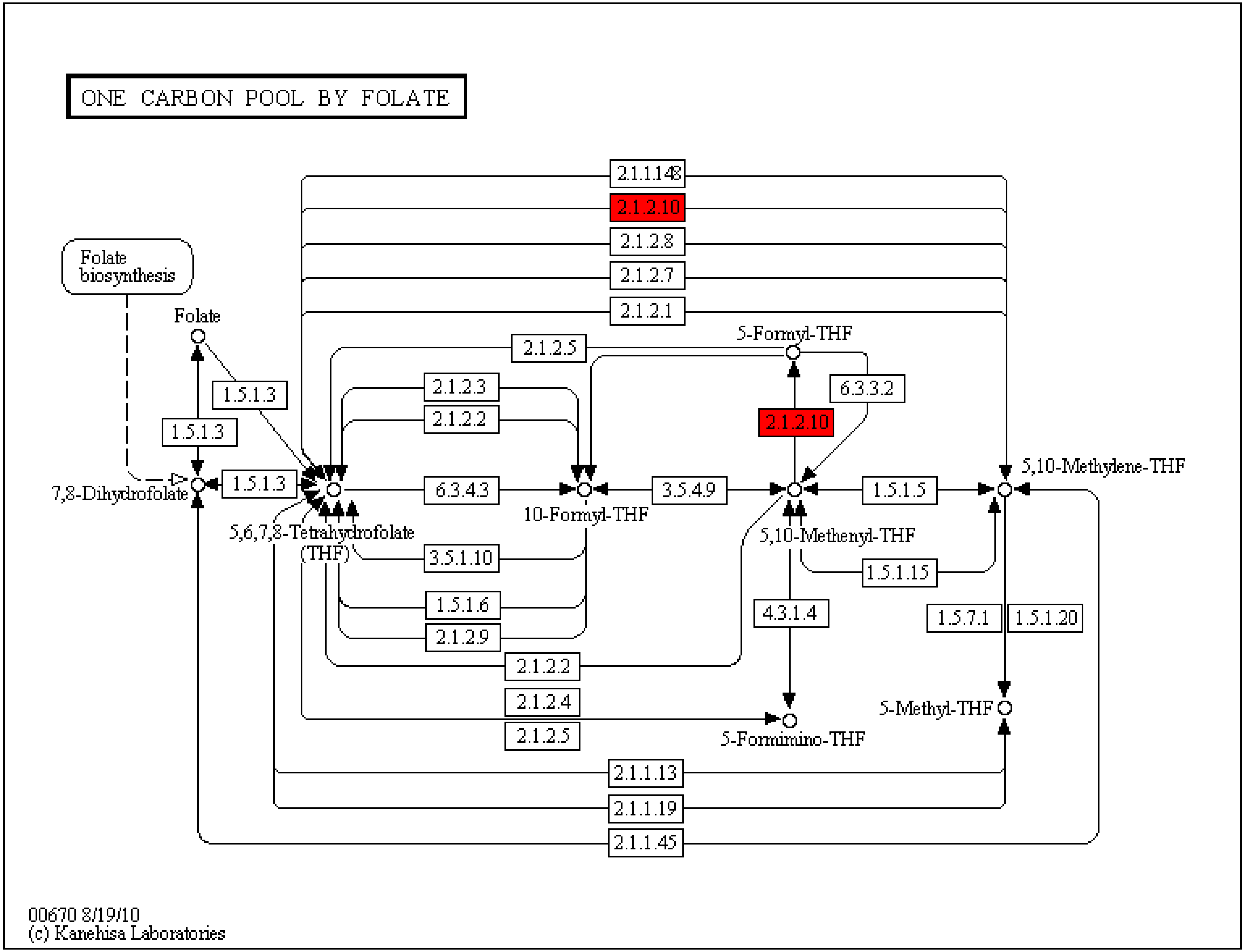

Colour	Enzyme	EST clone
red	ec:2.1.2.10 - aminomethyltransferase	TSBD.R1.esd

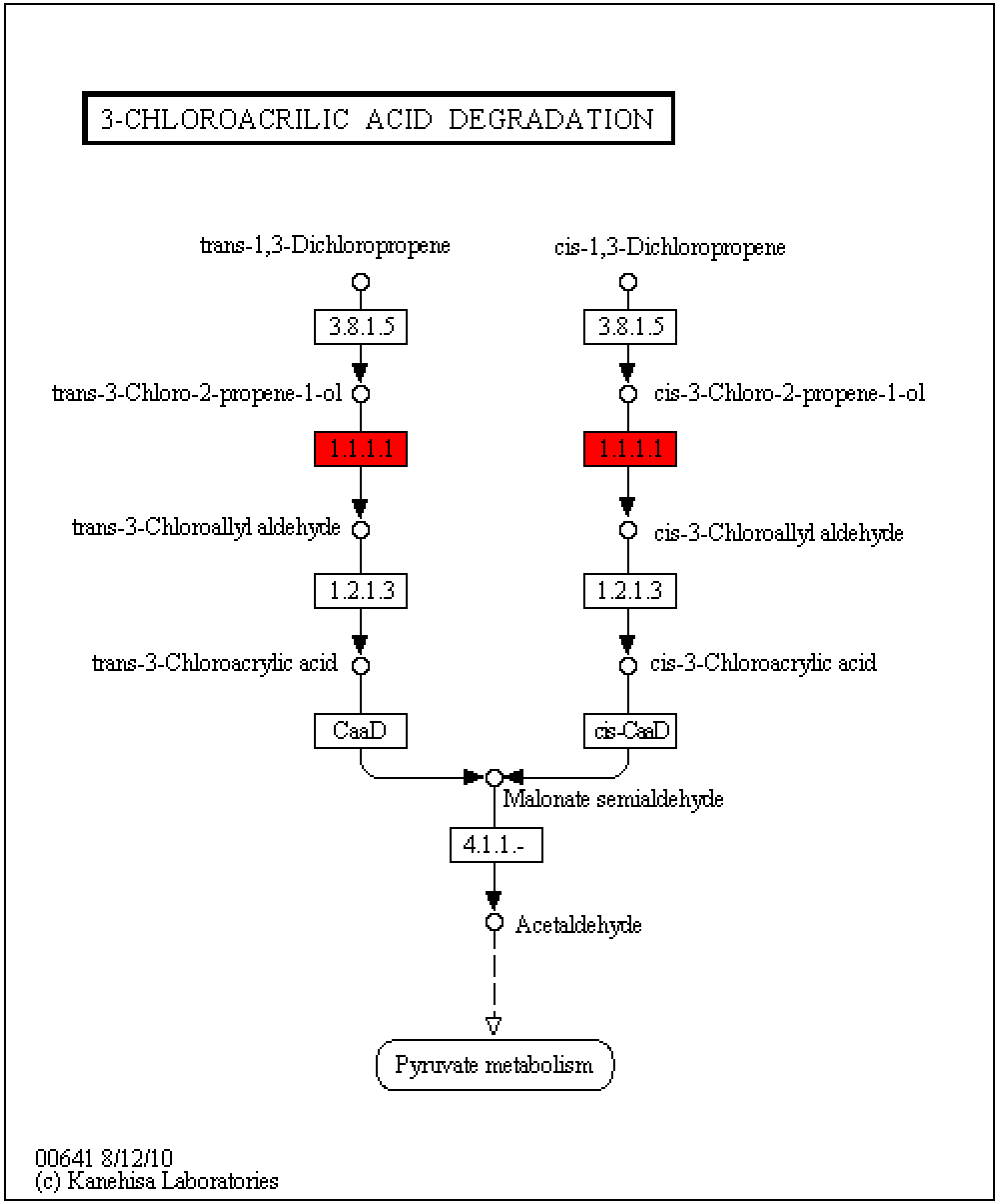

Colour	Enzyme	EST clone
red	ec:1.1.1.1 - alcohol dehydrogenase	TSCE.R92.esd

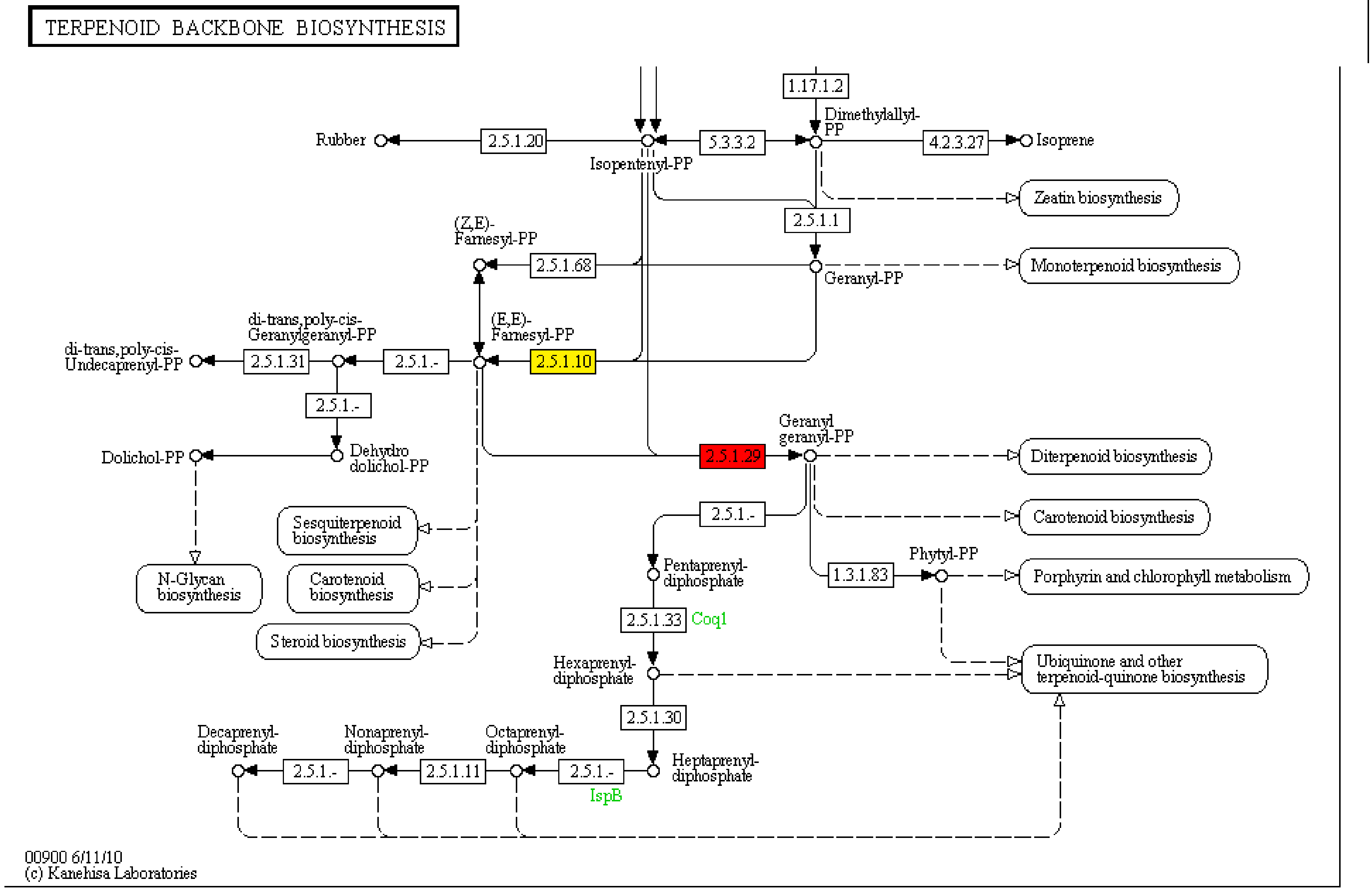

Colour	Enzyme	EST clone
red	ec:2.5.1.29 - farnesyltranstransferase	TS.seq.screen.trim.Contig110
yellow	ec:2.5.1.10 - geranyltranstransferase	TS.seq.screen.trim.Contig110

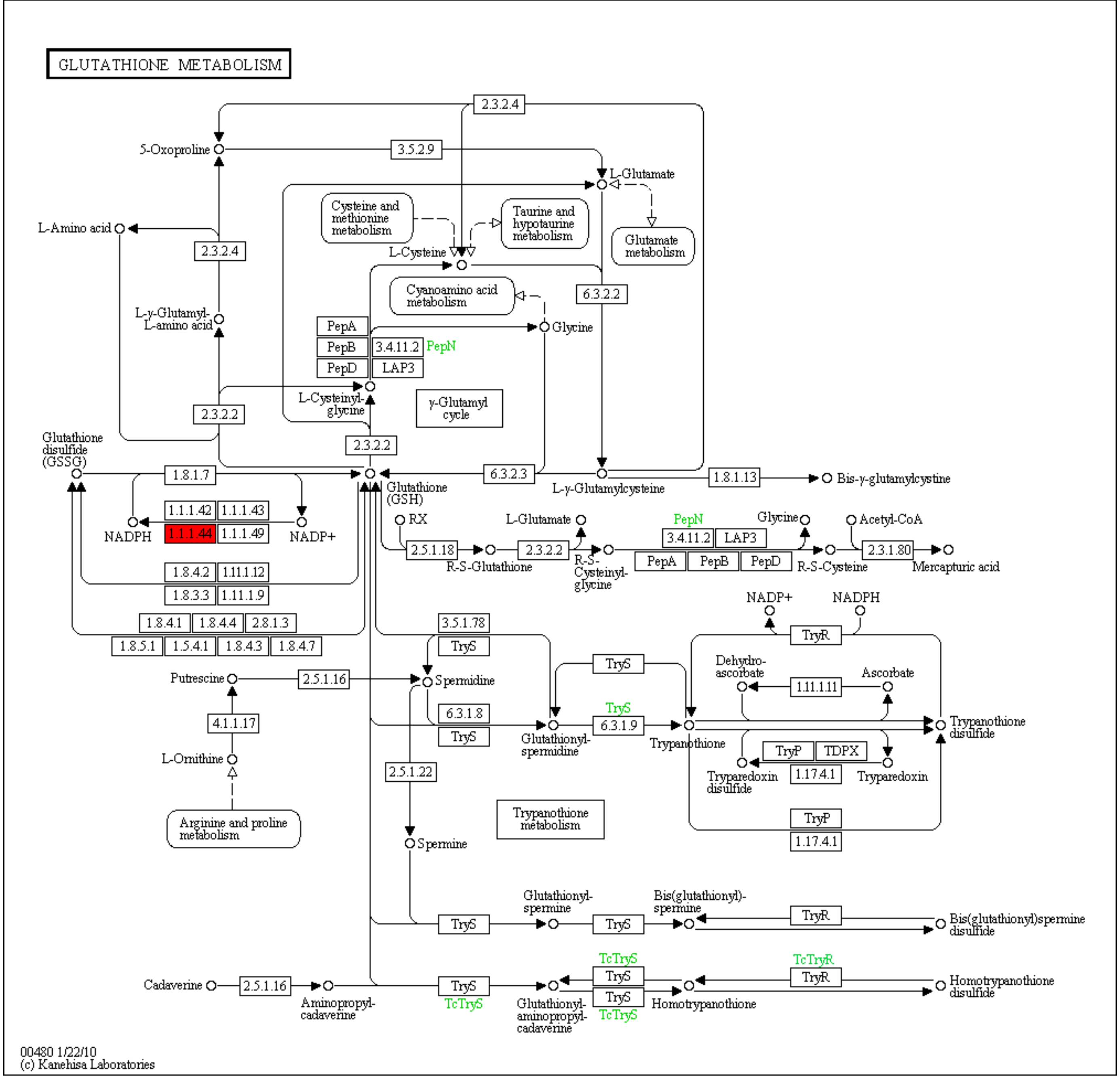

Colour	Enzyme	EST clone
red	ec:1.1.1.44 - phosphogluconate dehydrogenase (decarboxylating)	TSBR.R51.esd

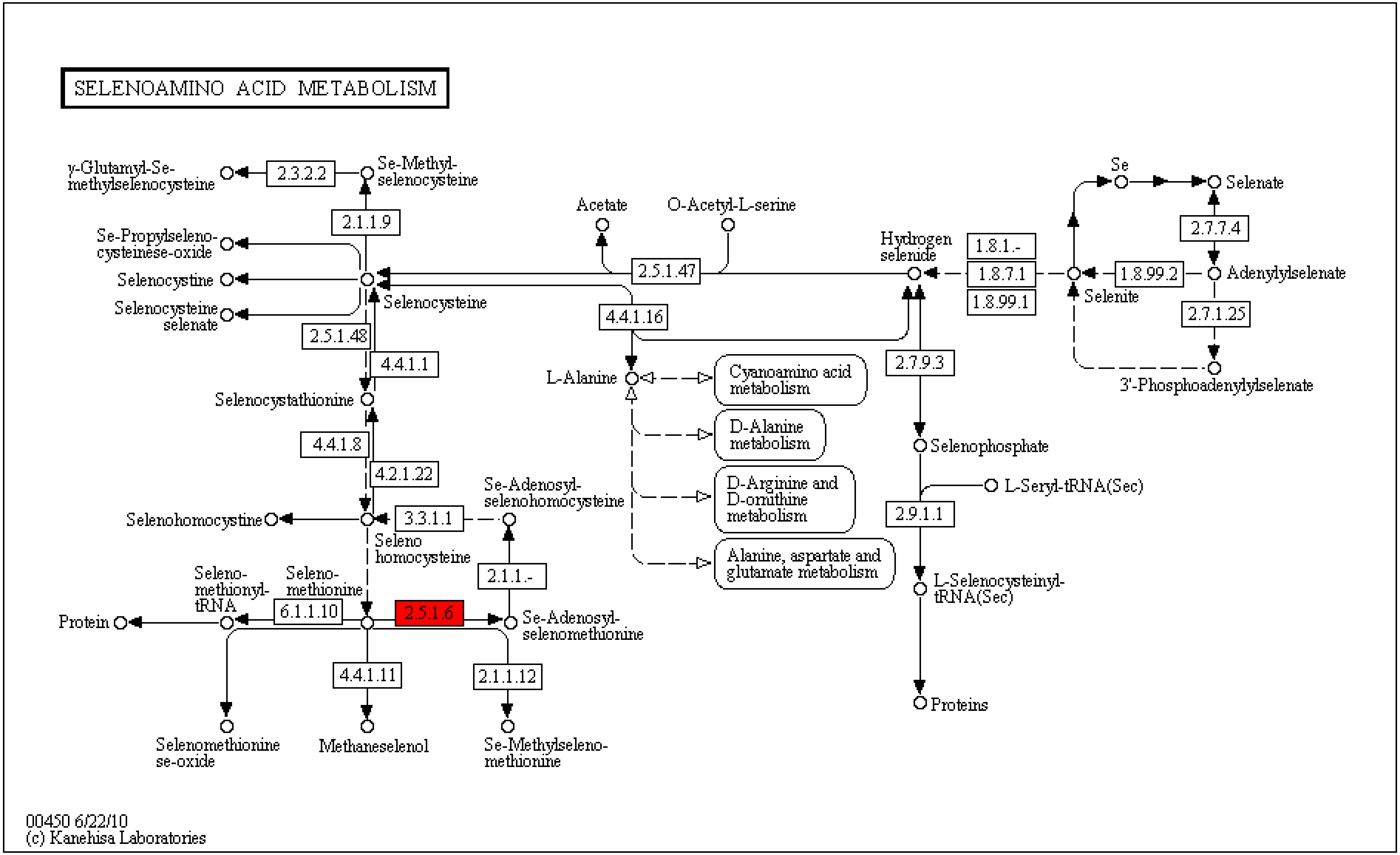

Colour	Enzyme	EST clone
red	ec:2.5.1.6 - methionine adenosyltransferase	TSAT.R84.esd

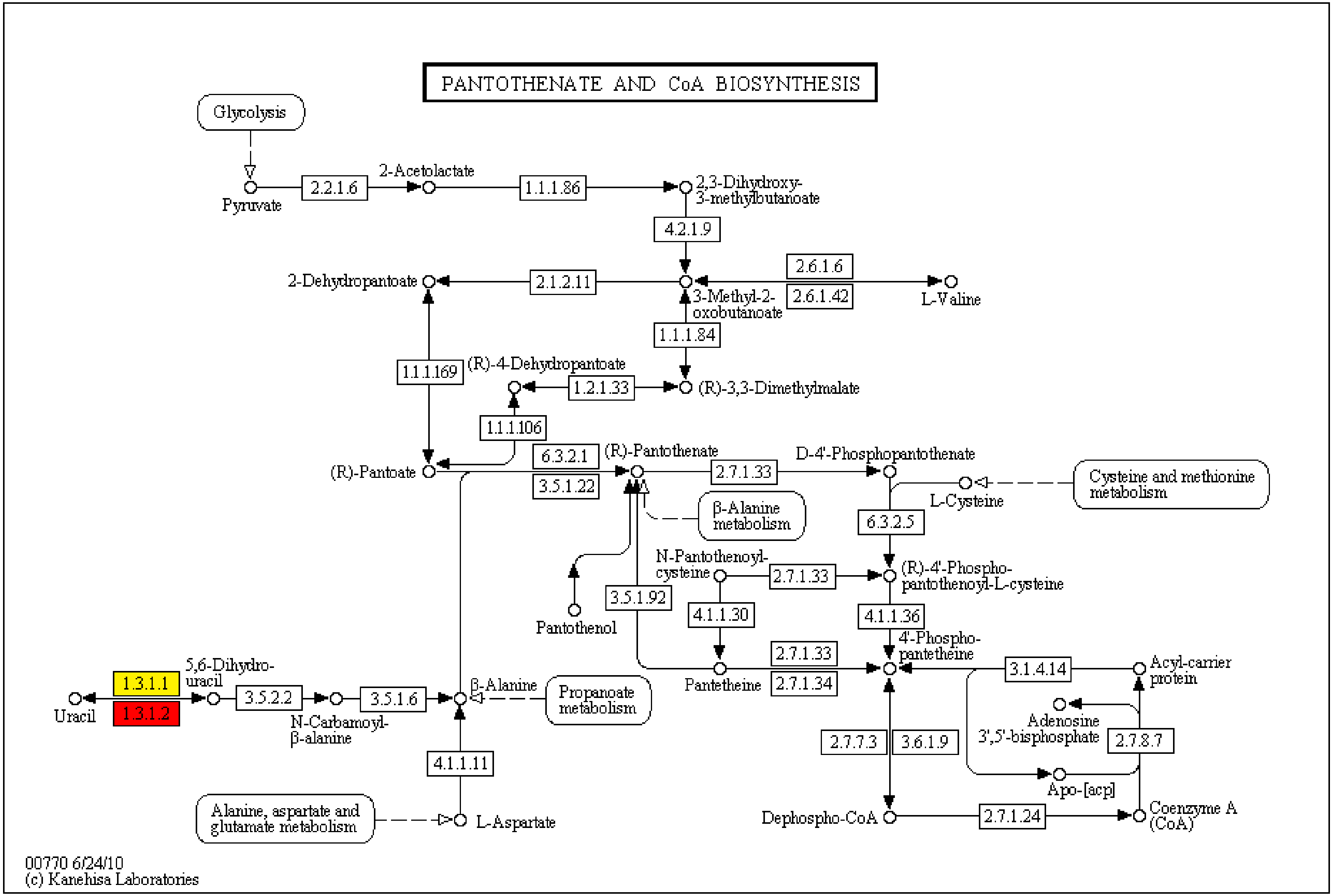

Colour	Enzyme	EST clone
red	ec:1.3.1.2 - dihydropyrimidine dehydrogenase (NADP+)	TSCF.R86.esd
yellow	ec:1.3.1.1 - dihydrouracil dehydrogenase (NAD+)	TSCF.R86.esd

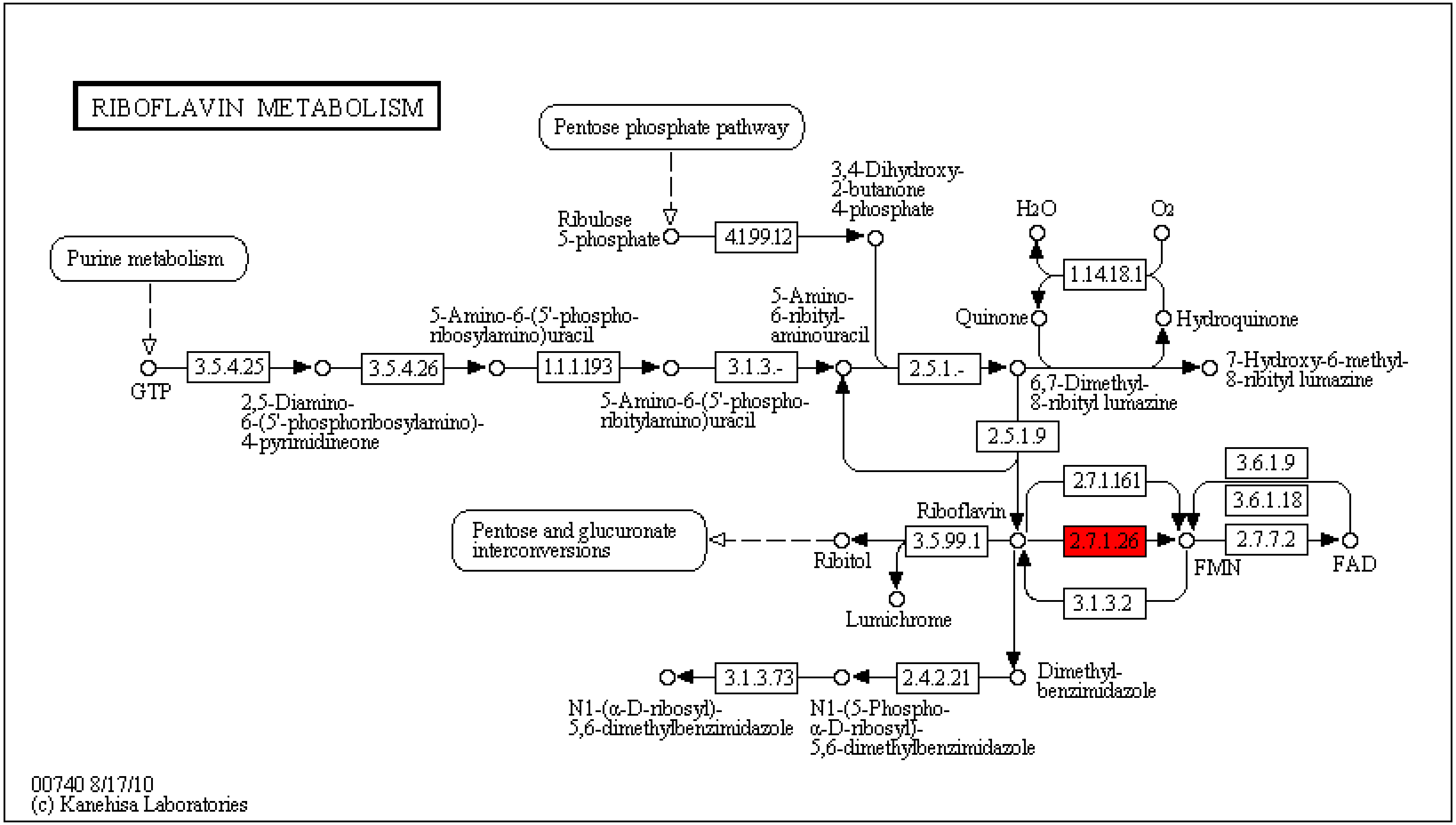

**Colour**	**Enzyme**	**EST clone**
red	ec:2.7.1.26 - riboflavin kinase	TSAO.R20.esd
