# Supplementary material for: Analyses of an Expressed Sequence Tag Library from Taenia solium, Cysticerca
Source: PLoS Negl Trop Dis. 2010 Dec 21;4(12):e919. doi: 10.1371/journal.pntd.0000919 (PMC3006133; doi:10.1371/journal.pntd.0000919)
Supplement: Table S1 — Result from BLAST search with a score <10−5 of 1650 unique ESTs. (0.66 MB DOC) [file pntd.0000919.s003.doc]

| **Sequence name** | **E-Value** | **Hit desc.** |
| --- | --- | --- |
| TS.seq. Contig1 | 8.97636E-33 | gi|29825896|gb|AAN63052.1|thioredoxin glutathione reductase [Echinococcus granulosus] |
| TS.seq. Contig3 | 1.72696E-15 | gi|194668093|ref|XP_607200.3|PREDICTED: similar to ARD1 homolog B protein [Bos taurus] |
| TS.seq. Contig5 | 6.63635E-8 | gi|256074434|ref|XP_002573530.1|cysteine and histidine-rich domain (chord)-containing zinc binding protein [Schistosoma mansoni] >gi|238658711|emb|CAZ29762.1| cysteine and histidine-rich domain (chord)-containing, zinc binding protein, putative [Schistosoma mansoni] |
| TS.seq. Contig6 | 5.00275E-7 | gi|91076110|ref|XP_969015.1|PREDICTED: similar to synapse-associated protein [Tribolium castaneum] |
| TS.seq. Contig7 | 6.44055E-32 | gi|73979494|ref|XP_532845.2|PREDICTED: similar to acyl-CoA synthetase long-chain family member 1 isoform 1 [Canis familiaris] |
| TS.seq. Contig10 | 2.22968E-25 | gi|256076375|ref|XP_002574488.1|camp-dependent protein kinase type I-beta regulatory subunit [Schistosoma mansoni] >gi|238659694|emb|CAZ30721.1| camp-dependent protein kinase type I-beta regulatory subunit, putative [Schistosoma mansoni] |
| TS.seq. Contig11 | 9.21958E-35 | gi|256071531|ref|XP_002572093.1|hypothetical protein [Schistosoma mansoni] >gi|238657245|emb|CAZ28323.1| expressed protein [Schistosoma mansoni] |
| TS.seq. Contig13 | 1.31409E-23 | gi|13603807|gb|AAK31941.1|AF267116_1activated oncosphere TSO45-2A [Taenia solium] |
| TS.seq. Contig14 | 1.56527E-27 | gi|256090758|ref|XP_002581348.1|hypothetical protein [Schistosoma mansoni] >gi|238667217|emb|CAZ37587.1| conserved hypothetical protein [Schistosoma mansoni] |
| TS.seq. Contig18 | 5.15098E-4 | gi|59709852|gb|AAW88556.1|oncosphere protein Tso31a [Taenia solium] >gi|111146850|gb|ABH07373.1| oncosphere protein Tso31a [Taenia solium] |
| TS.seq. Contig19 | 2.24333E-10 | gi|229367026|gb|ACQ58493.1|CWC15 homolog [Anoplopoma fimbria] |
| TS.seq. Contig20 | 1.11255E-6 | gi|226473722|emb|CAX71546.1|small subunit ribosomal protein S27e [Schistosoma japonicum] >gi|226473932|emb|CAX77412.1| small subunit ribosomal protein S27e [Schistosoma japonicum] >gi|226473934|emb|CAX77413.1| small subunit ribosomal protein S27e [Schistosoma japonicum] >gi|226473936|emb|CAX77414.1| small subunit ribosomal protein S27e [Schistosoma japonicum] >gi|226473938|emb|CAX77415.1| small subunit ribosomal protein S27e [Schistosoma japonicum] >gi|226473940|emb|CAX77416.1| small subunit ribosomal protein S27e [Schistosoma japonicum] >gi|226473942|emb|CAX77417.1| small subunit ribosomal protein S27e [Schistosoma japonicum] >gi|226473944|emb|CAX77418.1| small subunit ribosomal protein S27e [Schistosoma japonicum] >gi|226473946|emb|CAX77419.1| small subunit ribosomal protein S27e [Schistosoma japonicum] >gi|226473948|emb|CAX77420.1| small subunit ribosomal protein S27e [Schistosoma japonicum] >gi|226473950|emb|CAX77421.1| small subunit ribosomal protein S27e [Schistosoma japonicum] >gi|226473952|emb|CAX77422.1| small subunit ribosomal protein S27e [Schistosoma japonicum] >gi|226473954|emb|CAX77423.1| small subunit ribosomal protein S27e [Schistosoma japonicum] >gi|226473956|emb|CAX77424.1| small subunit ribosomal protein S27e [Schistosoma japonicum] >gi|226473958|emb|CAX77425.1| small subunit ribosomal protein S27e [Schistosoma japonicum] >gi|226473960|emb|CAX77426.1| small subunit ribosomal protein S27e [Schistosoma japonicum] >gi|226473962|emb|CAX77427.1| small subunit ribosomal protein S27e [Schistosoma japonicum] >gi|226473964|emb|CAX77428.1| small subunit ribosomal protein S27e [Schistosoma japonicum] |
| TS.seq. Contig24 | 1.78712E-12 | gi|156396512|ref|XP_001637437.1|predicted protein [Nematostella vectensis] >gi|156224549|gb|EDO45374.1| predicted protein [Nematostella vectensis] |
| TS.seq. Contig25 | 1.45066E-4 | gi|196006087|ref|XP_002112910.1|hypothetical protein TRIADDRAFT_56522 [Trichoplax adhaerens] >gi|190584951|gb|EDV25020.1| hypothetical protein TRIADDRAFT_56522 [Trichoplax adhaerens] |
| TS.seq. Contig26 | 2.91225E-10 | gi|256090758|ref|XP_002581348.1|hypothetical protein [Schistosoma mansoni] >gi|238667217|emb|CAZ37587.1| conserved hypothetical protein [Schistosoma mansoni] |
| TS.seq. Contig27 | 3.5733E-13 | gi|59709856|gb|AAW88558.1|oncosphere protein Tso31c [Taenia solium] >gi|111146852|gb|ABH07375.1| oncosphere protein Tso31c [Taenia solium] |
| TS.seq. Contig32 | 3.11004E-25 | gi|261286856|gb|ACX68651.1|beta actin [Anguilla japonica] |
| TS.seq. Contig33 | 5.2604E-12 | gi|242009491|ref|XP_002425519.1|conserved hypothetical protein [Pediculus humanus corporis] >gi|212509374|gb|EEB12781.1| conserved hypothetical protein [Pediculus humanus corporis] |
| TS.seq. Contig35 | 2.8396E-18 | gi|260401142|gb|ACX37099.1|beta actin [Polyrhachis vicina] |
| TS.seq. Contig38 | 4.51093E-73 | gi|8886013|gb|AAF80342.1|AF157514_1beta-actin [Oncorhynchus mykiss] |
| TS.seq. Contig41 | 6.84656E-12 | gi|256599961|pdb|6LDH|AChain A, Refined Crystal Structure Of Dogfish M4 Apo-Lactate Dehydrogenase >gi|256599962|pdb|8LDH|A Chain A, Refined Crystal Structure Of Dogfish M4 Apo-Lactate Dehydrogenase |
| TS.seq. Contig43 | 4.09148E-30 | gi|17433119|sp|O93542.3|LDHA_ELEMCRecName: Full=L-lactate dehydrogenase A chain; Short=LDH-A >gi|3719279|gb|AAC63283.1| lactate dehydrogenase-A [Eleginops maclovinus] |
| TS.seq. Contig45 | 2.78302E-29 | gi|13603817|gb|AAK31946.1|AF267121_1activated oncosphere TSO45-1B [Taenia solium] |
| TS.seq. Contig47 | 3.3114E-19 | gi|124783036|gb|ABN14898.1|ribosomal protein S8 [Taenia asiatica] |
| TS.seq. Contig48 | 9.47493E-14 | gi|242229758|ref|XP_002477792.1|predicted protein [Postia placenta Mad-698-R] >gi|220721945|gb|EED77009.1| predicted protein [Postia placenta Mad-698-R] |
| TS.seq. Contig51 | 3.80044E-39 | gi|189235535|ref|XP_972604.2|PREDICTED: similar to AGAP006448-PB [Tribolium castaneum] |
| TS.seq. Contig52 | 1.95195E-31 | gi|60687866|gb|AAX30266.1|SJCHGC02603 protein [Schistosoma japonicum] |
| TS.seq. Contig59 | 9.51299E-14 | gi|159507454|gb|ABW97741.1|beta-actin [Crassostrea ariakensis] |
| TS.seq. Contig60 | 9.23443E-38 | gi|256080191|ref|XP_002576366.1|n-terminal acetyltransferase complex ard1 subunit [Schistosoma mansoni] >gi|238661632|emb|CAZ32603.1| n-terminal acetyltransferase complex ard1 subunit, putative [Schistosoma mansoni] |
| TS.seq. Contig61 | 2.36939E-17 | gi|198425003|ref|XP_002123716.1|PREDICTED: similar to Cytochrome c oxidase copper chaperone [Ciona intestinalis] |
| TS.seq. Contig63 | 1.80688E-10 | gi|260827521|ref|XP_002608713.1|hypothetical protein BRAFLDRAFT_120586 [Branchiostoma floridae] >gi|229294065|gb|EEN64723.1| hypothetical protein BRAFLDRAFT_120586 [Branchiostoma floridae] |
| TS.seq. Contig64 | 5.71897E-7 | gi|256083017|ref|XP_002577747.1|UDP-galactose transporter [Schistosoma mansoni] >gi|238663077|emb|CAZ33985.1| UDP-galactose transporter, putative [Schistosoma mansoni] |
| TS.seq. Contig66 | 1.77519E-20 | gi|226490144|emb|CAX69314.1|Inhibitor of growth protein 3 [Schistosoma japonicum] |
| TS.seq. Contig68 | 7.42379E-27 | gi|226490224|emb|CAX69354.1|hypothetical protein [Schistosoma japonicum] |
| TS.seq. Contig69 | 1.45472E-33 | gi|124784148|gb|ABN14965.1|small GTPase RhoA [Taenia asiatica] |
| TS.seq. Contig70 | 1.47155E-7 | gi|256078279|ref|XP_002575424.1|cytoplasmic dynein light chain [Schistosoma mansoni] >gi|238660662|emb|CAZ31657.1| cytoplasmic dynein light chain, putative [Schistosoma mansoni] |
| TS.seq. Contig72 | 5.53105E-6 | gi|256074495|ref|XP_002573560.1|peptidyl-prolyl cis-trans isomerase-like 2 ppil2 [Schistosoma mansoni] >gi|238658742|emb|CAZ29792.1| peptidyl-prolyl cis-trans isomerase-like 2, ppil2 [Schistosoma mansoni] |
| TS.seq. Contig73 | 4.75558E-13 | gi|226470502|emb|CAX70531.1|Salivary secreted ribonuclease [Schistosoma japonicum] >gi|226470504|emb|CAX70532.1| Salivary secreted ribonuclease [Schistosoma japonicum] >gi|226470506|emb|CAX70533.1| Salivary secreted ribonuclease [Schistosoma japonicum] >gi|226487038|emb|CAX75384.1| Salivary secreted ribonuclease [Schistosoma japonicum] >gi|226487040|emb|CAX75385.1| Salivary secreted ribonuclease [Schistosoma japonicum] |
| TS.seq. Contig74 | 2.43457E-46 | gi|21912582|emb|CAD21546.1|hypothetical protein [Taenia solium] |
| TS.seq. Contig75 | 5.2504E-46 | gi|45384260|ref|NP_990378.1|nucleoside diphosphate kinase B [Gallus gallus] >gi|82190139|sp|O57535.1|NDK_CHICK RecName: Full=Nucleoside diphosphate kinase; Short=NDP kinase; Short=NDK >gi|2827444|gb|AAB99856.1| nucleoside diphosphate kinase [Gallus gallus] |
| TS.seq. Contig79 | 1.05648E-4 | gi|76156821|gb|AAX27944.2|SJCHGC09141 protein [Schistosoma japonicum] |
| TS.seq. Contig80 | 5.27743E-9 | gi|256078753|ref|XP_002575659.1|SmIrV1 protein [Schistosoma mansoni] >gi|348677|gb|AAA02575.1| SmIrV1 protein >gi|238660901|emb|CAZ31892.1| SmIrV1 protein, putative [Schistosoma mansoni] |
| TS.seq. Contig81 | 3.10317E-18 | gi|256075341|ref|XP_002573978.1|lipopolysaccharide-induced transcription factor regulating tumor necrosis factor alpha [Schistosoma mansoni] >gi|238659170|emb|CAZ30211.1| lipopolysaccharide-induced transcription factor regulating tumor necrosis factor alpha, putative [Schistosoma mansoni] |
| TS.seq. Contig82 | 3.94944E-26 | gi|21912552|emb|CAD21531.1|hypothetical protein [Taenia solium] |
| TS.seq. Contig83 | 7.18822E-16 | gi|226479126|emb|CAX73058.1|BTB/POZ domain-containing protein 12 [Schistosoma japonicum] |
| TS.seq. Contig84 | 1.03407E-20 | gi|256072726|ref|XP_002572685.1|guanine nucleotide-exchange [Schistosoma mansoni] >gi|238657848|emb|CAZ28917.1| guanine nucleotide-exchange, putative [Schistosoma mansoni] |
| TS.seq. Contig85 | 9.97933E-32 | gi|56756350|gb|AAW26348.1|SJCHGC02419 protein [Schistosoma japonicum] >gi|226469116|emb|CAX70037.1| heparin binding protein [Schistosoma japonicum] >gi|226475098|emb|CAX71837.1| heparin binding protein [Schistosoma japonicum] >gi|226475100|emb|CAX71838.1| heparin binding protein [Schistosoma japonicum] >gi|226475102|emb|CAX71839.1| heparin binding protein [Schistosoma japonicum] >gi|226477080|emb|CAX78193.1| heparin binding protein [Schistosoma japonicum] >gi|226477082|emb|CAX78194.1| heparin binding protein [Schistosoma japonicum] >gi|226477084|emb|CAX78195.1| heparin binding protein [Schistosoma japonicum] >gi|226477086|emb|CAX78196.1| heparin binding protein [Schistosoma japonicum] >gi|226477088|emb|CAX78197.1| heparin binding protein [Schistosoma japonicum] >gi|226477090|emb|CAX78198.1| heparin binding protein [Schistosoma japonicum] >gi|226477092|emb|CAX78199.1| heparin binding protein [Schistosoma japonicum] >gi|226477094|emb|CAX78200.1| heparin binding protein [Schistosoma japonicum] >gi|226477096|emb|CAX78201.1| heparin binding protein [Schistosoma japonicum] >gi|226477098|emb|CAX78202.1| heparin binding protein [Schistosoma japonicum] >gi|226477100|emb|CAX78203.1| heparin binding protein [Schistosoma japonicum] >gi|226477102|emb|CAX78204.1| heparin binding protein [Schistosoma japonicum] >gi|226477104|emb|CAX78205.1| heparin binding protein [Schistosoma japonicum] >gi|226477106|emb|CAX78206.1| heparin binding protein [Schistosoma japonicum] >gi|226477108|emb|CAX78207.1| heparin binding protein [Schistosoma japonicum] >gi|226477110|emb|CAX78208.1| heparin binding protein [Schistosoma japonicum] >gi|226477112|emb|CAX78209.1| heparin binding protein [Schistosoma japonicum] >gi|226477114|emb|CAX78210.1| heparin binding protein [Schistosoma japonicum] >gi|226477116|emb|CAX78211.1| heparin binding protein [Schistosoma japonicum] >gi|226477120|emb|CAX78213.1| heparin binding protein [Schistosoma japonicum] >gi|226477122|emb|CAX78214.1| heparin binding protein [Schistosoma japonicum] |
| TS.seq. Contig86 | 3.16748E-10 | gi|256088528|ref|XP_002580383.1|oxysterol binding protein 9 [Schistosoma mansoni] >gi|238665952|emb|CAZ36622.1| oxysterol binding protein 9,, putative [Schistosoma mansoni] |
| TS.seq. Contig89 | 9.70064E-54 | gi|256071684|ref|XP_002572169.1|DNA-directed rna polymerase subunit rpb8 [Schistosoma mansoni] >gi|238657322|emb|CAZ28400.1| DNA-directed rna polymerase subunit rpb8, putative [Schistosoma mansoni] |
| TS.seq. Contig92 | 2.64654E-16 | gi|256087057|ref|XP_002579695.1|hypothetical protein [Schistosoma mansoni] >gi|238665169|emb|CAZ35934.1| expressed protein [Schistosoma mansoni] |
| TS.seq. Contig93 | 7.72303E-16 | gi|193605860|ref|XP_001944617.1|PREDICTED: similar to cation-transporting atpase [Acyrthosiphon pisum] |
| TS.seq. Contig94 | 2.42581E-58 | gi|256081089|ref|XP_002576806.1|lin-9 [Schistosoma mansoni] >gi|238662090|emb|CAZ33043.1| lin-9, putative [Schistosoma mansoni] |
| TS.seq. Contig96 | 3.06892E-41 | gi|256084674|ref|XP_002578552.1|gaba(A) receptor-associated protein [Schistosoma mansoni] >gi|238663929|emb|CAZ34790.1| gaba(A) receptor-associated protein, putative [Schistosoma mansoni] |
| TS.seq. Contig97 | 6.19943E-5 | gi|213513620|ref|NP_001133220.1|Vps20-associated 1 like 1 [Salmo salar] >gi|197632647|gb|ACH71047.1| Vps20-associated 1 like 1 [Salmo salar] |
| TS.seq. Contig100 | 1.05408E-8 | gi|171473939|gb|AAW25160.2|SJCHGC07996 protein [Schistosoma japonicum] |
| TS.seq. Contig101 | 1.56991E-24 | gi|76159349|gb|AAW26450.2|SJCHGC09642 protein [Schistosoma japonicum] >gi|226469696|emb|CAX76678.1| ribosomal protein L36 [Schistosoma japonicum] >gi|226469698|emb|CAX76679.1| ribosomal protein L36 [Schistosoma japonicum] >gi|226469700|emb|CAX76680.1| ribosomal protein L36 [Schistosoma japonicum] >gi|226469702|emb|CAX76681.1| ribosomal protein L36 [Schistosoma japonicum] >gi|226469704|emb|CAX76682.1| ribosomal protein L36 [Schistosoma japonicum] >gi|226469706|emb|CAX76683.1| ribosomal protein L36 [Schistosoma japonicum] >gi|226469708|emb|CAX76684.1| ribosomal protein L36 [Schistosoma japonicum] >gi|226473032|emb|CAX71202.1| ribosomal protein L36 [Schistosoma japonicum] >gi|226479996|emb|CAX73294.1| ribosomal protein L36 [Schistosoma japonicum] |
| TS.seq. Contig102 | 6.08701E-37 | gi|256076344|ref|XP_002574473.1|prefoldin subunit 2 [Schistosoma mansoni] >gi|238659678|emb|CAZ30706.1| prefoldin subunit 2, putative [Schistosoma mansoni] |
| TS.seq. Contig104 | 2.33855E-37 | gi|226472060|emb|CAX77068.1|Splicing factor, arginine/serine-rich 7 [Schistosoma japonicum] >gi|226472062|emb|CAX77069.1| Splicing factor, arginine/serine-rich 7 [Schistosoma japonicum] >gi|226472064|emb|CAX77070.1| Splicing factor, arginine/serine-rich 7 [Schistosoma japonicum] >gi|226472066|emb|CAX77071.1| Splicing factor, arginine/serine-rich 7 [Schistosoma japonicum] >gi|226472072|emb|CAX77074.1| Splicing factor, arginine/serine-rich 7 [Schistosoma japonicum] |
| TS.seq. Contig107 | 9.97645E-37 | gi|256089348|ref|XP_002580773.1|fragile X related 1 frx1 [Schistosoma mansoni] >gi|238666382|emb|CAZ37012.1| fragile X related 1, frx1 [Schistosoma mansoni] |
| TS.seq. Contig109 | 4.43862E-43 | gi|256073099|ref|XP_002572870.1|adenylyl cyclase-associated protein [Schistosoma mansoni] >gi|238658036|emb|CAZ29102.1| adenylyl cyclase-associated protein, putative [Schistosoma mansoni] |
| TS.seq. Contig110 | 8.04818E-44 | gi|29841005|gb|AAP06018.1|similar to NM_010282 geranylgeranyl diphosphate synthase 1; GGPP synthase in Mus musculus [Schistosoma japonicum] |
| TS.seq. Contig112 | 6.70011E-31 | gi|256081813|ref|XP_002577162.1|hypothetical protein [Schistosoma mansoni] >gi|238662458|emb|CAZ33399.1| hypothetical protein [Schistosoma mansoni] |
| TS.seq. Contig113 | 2.12741E-22 | gi|91090858|ref|XP_967143.1|PREDICTED: similar to annexin B13a isoform 1 [Tribolium castaneum] |
| TS.seq. Contig114 | 2.16158E-11 | gi|226469090|emb|CAX70024.1|hypothetical protein [Schistosoma japonicum] |
| TS.seq. Contig115 | 9.26859E-17 | gi|242010178|ref|XP_002425853.1|predicted protein [Pediculus humanus corporis] >gi|212509786|gb|EEB13115.1| predicted protein [Pediculus humanus corporis] |
| TS.seq. Contig117 | 4.03466E-40 | gi|56754608|gb|AAW25490.1|unknown [Schistosoma japonicum] >gi|60687656|gb|AAX30161.1| SJCHGC01237 protein [Schistosoma japonicum] >gi|226468508|emb|CAX69931.1| NADH dehydrogenase (ubiquinone) 1 alpha subcomplex 8 [Schistosoma japonicum] >gi|226468510|emb|CAX69932.1| NADH dehydrogenase (ubiquinone) 1 alpha subcomplex 8 [Schistosoma japonicum] |
| TS.seq. Contig118 | 9.61279E-57 | gi|256074599|ref|XP_002573611.1|bicoid-interacting protein related [Schistosoma mansoni] >gi|238658795|emb|CAZ29843.1| bicoid-interacting protein related [Schistosoma mansoni] |
| TS.seq. Contig119 | 1.04507E-39 | gi|256074726|ref|XP_002573674.1|hypothetical protein [Schistosoma mansoni] >gi|238658859|emb|CAZ29906.1| expressed protein [Schistosoma mansoni] |
| TS.seq. Contig120 | 4.35282E-25 | gi|15010834|dbj|BAB62184.1|cytochrome c oxidase subunit 1 [Taenia solium] |
| TS.seq. Contig121 | 5.11613E-4 | gi|148223119|ref|NP_001088471.1|hypothetical protein LOC495336 [Xenopus laevis] >gi|54311231|gb|AAH84798.1| LOC495336 protein [Xenopus laevis] |
| TS.seq. Contig122 | 4.25051E-84 | gi|157160998|ref|YP_001458316.1|LysR family transcriptional regulator [Escherichia coli HS] >gi|170020145|ref|YP_001725099.1| LysR family transcriptional regulator [Escherichia coli ATCC 8739] >gi|157066678|gb|ABV05933.1| transcriptional regulator, LysR family [Escherichia coli HS] >gi|169755073|gb|ACA77772.1| transcriptional regulator, LysR family [Escherichia coli ATCC 8739] |
| TS.seq. Contig125 | 1.59994E-61 | gi|21912540|emb|CAD21525.1|hypothetical protein [Taenia solium] |
| TS.seq. Contig126 | 2.79434E-22 | gi|129166|sp|P22080|ONCA_TAETAOncosphere antigen A >gi|161654|gb|AAA63536.1| oncosphere antigen |
| TS.seq. Contig127 | 8.07221E-13 | gi|242014975|ref|XP_002428154.1|H/ACA ribonucleoprotein complex subunit, putative [Pediculus humanus corporis] >gi|212512697|gb|EEB15416.1| H/ACA ribonucleoprotein complex subunit, putative [Pediculus humanus corporis] |
| TS.seq. Contig129 | 6.56747E-18 | gi|256086089|ref|XP_002579238.1|hypothetical protein [Schistosoma mansoni] >gi|238664658|emb|CAZ35477.1| hypothetical protein [Schistosoma mansoni] |
| TS.seq. Contig130 | 3.09236E-78 | gi|62868248|emb|CAI59265.1|ski interacting protein [Echinococcus multilocularis] |
| TS.seq. Contig131 | 4.62019E-8 | gi|41054085|ref|NP_956163.1|insulin induced gene 1 [Danio rerio] >gi|189521532|ref|XP_001922160.1| PREDICTED: similar to insulin induced gene 1 [Danio rerio] >gi|82177146|sp|Q8AV61.1|INSI1_DANRE RecName: Full=Insulin-induced gene 1 protein; Short=INSIG-1 >gi|23344037|gb|AAN28327.1| INSIG-1 membrane protein [Danio rerio] >gi|28278849|gb|AAH45341.1| Insulin induced gene 1 [Danio rerio] >gi|182890692|gb|AAI65115.1| Insig1 protein [Danio rerio] |
| TS.seq. Contig132 | 6.8623E-25 | gi|256074151|ref|XP_002573390.1|translation factor sui1 [Schistosoma mansoni] >gi|238658568|emb|CAZ29622.1| translation factor sui1, putative [Schistosoma mansoni] |
| TS.seq. Contig133 | 3.42882E-16 | gi|256083339|ref|XP_002577903.1|hypothetical protein [Schistosoma mansoni] >gi|238663243|emb|CAZ34141.1| conserved hypothetical protein [Schistosoma mansoni] |
| TS.seq. Contig134 | 4.0936E-14 | gi|256074621|ref|XP_002573622.1|ccr4-associated factor [Schistosoma mansoni] >gi|238658806|emb|CAZ29854.1| ccr4-associated factor, putative [Schistosoma mansoni] |
| TS.seq. Contig136 | 7.98651E-37 | gi|56757827|gb|AAW27054.1|SJCHGC04800 protein [Schistosoma japonicum] |
| TS.seq. Contig137 | 1.90375E-37 | gi|256083836|ref|XP_002578142.1|protein kinase [Schistosoma mansoni] >gi|238663501|emb|CAZ34380.1| protein kinase [Schistosoma mansoni] |
| TS.seq. Contig138 | 1.09898E-30 | gi|256075800|ref|XP_002574204.1|40S ribosomal protein S3 [Schistosoma mansoni] >gi|238659403|emb|CAZ30437.1| 40S ribosomal protein S3, putative [Schistosoma mansoni] |
| TS.seq. Contig139 | 3.59072E-45 | gi|256079299|ref|XP_002575926.1|protein phosphatases pp1 regulatory subunit [Schistosoma mansoni] >gi|238661180|emb|CAZ32161.1| protein phosphatases pp1 regulatory subunit, putative [Schistosoma mansoni] |
| TS.seq. Contig142 | 5.6478E-6 | gi|193632114|ref|XP_001945054.1|PREDICTED: similar to CG17184 CG17184-PA [Acyrthosiphon pisum] |
| TS.seq. Contig143 | 1.04232E-52 | gi|148229787|ref|NP_001089376.1|hypothetical protein LOC734426 [Xenopus laevis] >gi|62471500|gb|AAH93564.1| MGC115350 protein [Xenopus laevis] |
| TS.seq. Contig145 | 6.64028E-19 | gi|256082579|ref|XP_002577532.1|n-terminal acetlytransferase-related [Schistosoma mansoni] >gi|238662854|emb|CAZ33770.1| n-terminal acetlytransferase-related [Schistosoma mansoni] |
| TS.seq. Contig146 | 1.38897E-82 | gi|124784242|gb|ABN14969.1|RAB4B protein [Taenia asiatica] |
| TS.seq. Contig147 | 2.97073E-27 | gi|56758906|gb|AAW27593.1|SJCHGC09090 protein [Schistosoma japonicum] >gi|226467191|emb|CAX76076.1| putative ribosomal protein S13 [Schistosoma japonicum] >gi|226467193|emb|CAX76077.1| putative ribosomal protein S13 [Schistosoma japonicum] >gi|226467195|emb|CAX76078.1| putative ribosomal protein S13 [Schistosoma japonicum] >gi|226467197|emb|CAX76079.1| putative ribosomal protein S13 [Schistosoma japonicum] >gi|226467199|emb|CAX76080.1| putative ribosomal protein S13 [Schistosoma japonicum] >gi|226471666|emb|CAX70914.1| putative ribosomal protein S13 [Schistosoma japonicum] >gi|226471668|emb|CAX70915.1| putative ribosomal protein S13 [Schistosoma japonicum] >gi|226471670|emb|CAX70916.1| putative ribosomal protein S13 [Schistosoma japonicum] |
| TS.seq. Contig149 | 9.34415E-56 | gi|124784584|gb|ABN14984.1|sodium/potassium-transporting ATPase beta nervous system antigen 1 [Taenia asiatica] |
| TS.seq. Contig153 | 5.39365E-13 | gi|260830579|ref|XP_002610238.1|hypothetical protein BRAFLDRAFT_116266 [Branchiostoma floridae] >gi|229295602|gb|EEN66248.1| hypothetical protein BRAFLDRAFT_116266 [Branchiostoma floridae] |
| TS.seq. Contig154 | 1.15181E-9 | gi|256088712|ref|XP_002580470.1|Spindle assembly checkpoint component MAD1 (Mitotic arrest deficient protein 1) [Schistosoma mansoni] >gi|238666049|emb|CAZ36709.1| Spindle assembly checkpoint component MAD1 (Mitotic arrest deficient protein 1), putative [Schistosoma mansoni] |
| TS.seq. Contig155 | 9.2534E-9 | gi|256087610|ref|XP_002579959.1|hypothetical protein [Schistosoma mansoni] >gi|238665458|emb|CAZ36198.1| expressed protein [Schistosoma mansoni] |
| TS.seq. Contig156 | 6.98962E-25 | gi|256066153|ref|XP_002570485.1|peripheral-type benzodiazepine receptor [Schistosoma mansoni] >gi|227299147|emb|CAY18002.1| peripheral-type benzodiazepine receptor, putative [Schistosoma mansoni] |
| TS.seq. Contig158 | 3.38309E-48 | gi|67083925|gb|AAY66897.1|ribosomal protein S16 [Ixodes scapularis] |
| TS.seq. Contig160 | 5.01236E-15 | gi|241629293|ref|XP_002408271.1|conserved hypothetical protein [Ixodes scapularis] >gi|215501168|gb|EEC10662.1| conserved hypothetical protein [Ixodes scapularis] |
| TS.seq. Contig163 | 5.03435E-40 | gi|256070715|ref|XP_002571688.1|transcription factor btf3 [Schistosoma mansoni] >gi|238656834|emb|CAZ27918.1| transcription factor btf3, putative [Schistosoma mansoni] |
| TS.seq. Contig164 | 1.01415E-22 | gi|59709854|gb|AAW88557.1|oncosphere protein Tso31b [Taenia solium] >gi|111146851|gb|ABH07374.1| oncosphere protein Tso31b [Taenia solium] |
| TS.seq. Contig168 | 9.89036E-49 | gi|226476062|emb|CAX72121.1|ribosomal protein S14 [Schistosoma japonicum] >gi|226476064|emb|CAX72122.1| ribosomal protein S14 [Schistosoma japonicum] >gi|226476066|emb|CAX72123.1| ribosomal protein S14 [Schistosoma japonicum] >gi|226476068|emb|CAX72124.1| ribosomal protein S14 [Schistosoma japonicum] >gi|226480972|emb|CAX78951.1| ribosomal protein S14 [Schistosoma japonicum] >gi|226480974|emb|CAX78952.1| ribosomal protein S14 [Schistosoma japonicum] >gi|226480976|emb|CAX78953.1| ribosomal protein S14 [Schistosoma japonicum] >gi|226480978|emb|CAX78954.1| ribosomal protein S14 [Schistosoma japonicum] >gi|226480980|emb|CAX78955.1| ribosomal protein S14 [Schistosoma japonicum] >gi|226480982|emb|CAX78956.1| ribosomal protein S14 [Schistosoma japonicum] >gi|226480984|emb|CAX78957.1| ribosomal protein S14 [Schistosoma japonicum] >gi|226480986|emb|CAX78958.1| ribosomal protein S14 [Schistosoma japonicum] >gi|226480988|emb|CAX78959.1| ribosomal protein S14 [Schistosoma japonicum] >gi|226480990|emb|CAX78960.1| ribosomal protein S14 [Schistosoma japonicum] >gi|226480992|emb|CAX78961.1| ribosomal protein S14 [Schistosoma japonicum] >gi|226480994|emb|CAX78962.1| ribosomal protein S14 [Schistosoma japonicum] >gi|226480996|emb|CAX78963.1| ribosomal protein S14 [Schistosoma japonicum] >gi|226480998|emb|CAX78964.1| ribosomal protein S14 [Schistosoma japonicum] >gi|226481000|emb|CAX78965.1| ribosomal protein S14 [Schistosoma japonicum] >gi|226481002|emb|CAX78966.1| ribosomal protein S14 [Schistosoma japonicum] >gi|226481004|emb|CAX78967.1| ribosomal protein S14 [Schistosoma japonicum] >gi|226481006|emb|CAX78968.1| ribosomal protein S14 [Schistosoma japonicum] >gi|226481008|emb|CAX78969.1| ribosomal protein S14 [Schistosoma japonicum] >gi|226481010|emb|CAX78970.1| ribosomal protein S14 [Schistosoma japonicum] >gi|226481012|emb|CAX78971.1| ribosomal protein S14 [Schistosoma japonicum] >gi|226481014|emb|CAX78972.1| ribosomal protein S14 [Schistosoma japonicum] >gi|226481016|emb|CAX78973.1| ribosomal protein S14 [Schistosoma japonicum] >gi|226481018|emb|CAX78974.1| ribosomal protein S14 [Schistosoma japonicum] >gi|226481020|emb|CAX78975.1| ribosomal protein S14 [Schistosoma japonicum] >gi|226481022|emb|CAX78976.1| ribosomal protein S14 [Schistosoma japonicum] |
| TS.seq. Contig169 | 1.7226E-42 | gi|56756647|gb|AAW26496.1|SJCHGC05724 protein [Schistosoma japonicum] |
| TS.seq. Contig170 | 1.73661E-31 | gi|226480424|emb|CAX78876.1|eif5 protein [Schistosoma japonicum] |
| TS.seq. Contig172 | 9.21152E-64 | gi|12641925|gb|AAK00053.1|actin-filament fragmenting protein [Echinococcus granulosus] |
| TS.seq. Contig174 | 1.95135E-32 | gi|156395200|ref|XP_001636999.1|predicted protein [Nematostella vectensis] >gi|156224108|gb|EDO44936.1| predicted protein [Nematostella vectensis] |
| TS.seq. Contig176 | 2.19385E-11 | gi|17560088|ref|NP_506579.1|hypothetical protein F23B12.5 [Caenorhabditis elegans] >gi|74964045|sp|Q19749.1|ODP2_CAEEL RecName: Full=Dihydrolipoyllysine-residue acetyltransferase component of pyruvate dehydrogenase complex, mitochondrial; AltName: Full=Dihydrolipoamide acetyltransferase component of pyruvate dehydrogenase complex; AltName: Full=E2; Flags: Precursor >gi|3876313|emb|CAB01163.1| C. elegans protein F23B12.5, confirmed by transcript evidence [Caenorhabditis elegans] |
| TS.seq. Contig177 | 7.5571E-19 | gi|66522082|ref|XP_393336.2|PREDICTED: similar to GMP synthase [glutamine-hydrolyzing] (Glutamine amidotransferase) (GMP synthetase) [Apis mellifera] |
| TS.seq. Contig178 | 3.12417E-9 | gi|226479728|emb|CAX73160.1|SH3-binding, glutamic acid-rich protein [Schistosoma japonicum] |
| TS.seq. Contig183 | 1.47733E-71 | gi|13195551|gb|AAK15753.1|actin-binding and severin family group-like protein [Echinococcus granulosus] |
| TS.seq. Contig186 | 1.03303E-13 | gi|110558960|gb|ABG75848.1|EF-1 protein [Echinococcus granulosus] |
| TS.seq. Contig187 | 1.08856E-8 | gi|256072429|ref|XP_002572538.1|phosphatidylserine synthase 1 [Schistosoma mansoni] >gi|238657698|emb|CAZ28770.1| phosphatidylserine synthase 1 [Schistosoma mansoni] |
| TS.seq. Contig191 | 3.6252E-42 | gi|256071265|ref|XP_002571961.1|50S ribosomal protein L20 [Schistosoma mansoni] >gi|238657111|emb|CAZ28191.1| 50S ribosomal protein L20, putative [Schistosoma mansoni] |
| TS.seq. Contig192 | 1.66448E-50 | gi|226479208|emb|CAX73099.1|Four and a half LIM domains protein 2 [Schistosoma japonicum] |
| TS.seq. Contig193 | 9.46328E-83 | gi|124783075|gb|ABN14902.1|60S ribosomal protein L10a [Taenia asiatica] |
| TS.seq. Contig194 | 2.59394E-11 | gi|29336892|sp|Q967W0.1|CAB2_ECHGRRecName: Full=Calcium-binding protein 2; AltName: Full=Calcium-binding protein II; Short=CaBP-II; AltName: Full=EgCaBPII >gi|13774988|gb|AAK39122.1|AF361472_1 EF-hand calcium-binding protein [Echinococcus granulosus] |
| TS.seq. Contig195 | 2.2262E-19 | gi|60692150|gb|AAX30612.1|SJCHGC05705 protein [Schistosoma japonicum] |
| TS.seq. Contig197 | 3.58722E-53 | gi|124783111|gb|ABN14905.1|ribosomal protein S12 [Taenia asiatica] |
| TS.seq. Contig198 | 1.59013E-29 | gi|256073634|ref|XP_002573134.1|NDRG4 protein (S33 family) [Schistosoma mansoni] >gi|238658307|emb|CAZ29366.1| NDRG4 protein (S33 family) [Schistosoma mansoni] |
| TS.seq. Contig199 | 3.66561E-35 | gi|226486910|emb|CAX74532.1|Eukaryotic translation initiation factor 3 subunit 6-interacting protein [Schistosoma japonicum] |
| TS.seq. Contig201 | 1.68695E-37 | gi|158285298|ref|XP_308233.4|AGAP007636-PA [Anopheles gambiae str. PEST] >gi|157019924|gb|EAA04097.4| AGAP007636-PA [Anopheles gambiae str. PEST] |
| TS.seq. Contig203 | 8.23495E-6 | gi|226479728|emb|CAX73160.1|SH3-binding, glutamic acid-rich protein [Schistosoma japonicum] |
| TS.seq. Contig204 | 5.51826E-30 | gi|256084723|ref|XP_002578576.1|zinc finger protein [Schistosoma mansoni] >gi|238663954|emb|CAZ34814.1| zinc finger protein, putative [Schistosoma mansoni] |
| TS.seq. Contig205 | 1.00013E-39 | gi|226470036|emb|CAX70299.1|ubiquitin-conjugating enzyme E2G 2 [Schistosoma japonicum] >gi|226470038|emb|CAX70300.1| ubiquitin-conjugating enzyme E2G 2 [Schistosoma japonicum] >gi|226489126|emb|CAX74912.1| ubiquitin-conjugating enzyme E2G 2 [Schistosoma japonicum] >gi|226489128|emb|CAX74913.1| ubiquitin-conjugating enzyme E2G 2 [Schistosoma japonicum] |
| TS.seq. Contig206 | 4.20077E-54 | gi|194324935|gb|ACF49124.1|FK506-binding protein-like protein [Echinococcus granulosus] |
| TS.seq. Contig207 | 4.16234E-7 | gi|256087668|ref|XP_002579987.1|macroglobulin/complement [Schistosoma mansoni] >gi|238665488|emb|CAZ36226.1| macroglobulin/complement, putative [Schistosoma mansoni] |
| TS.seq. Contig208 | 2.20699E-23 | gi|256083113|ref|XP_002577794.1|tumor protein d52 [Schistosoma mansoni] >gi|238663126|emb|CAZ34032.1| tumor protein d52, putative [Schistosoma mansoni] |
| TS.seq. Contig209 | 4.25243E-38 | gi|170783721|gb|ACB37345.1|Ts4 protein [Taenia solium] |
| TS.seq. Contig210 | 2.25802E-23 | gi|226484978|emb|CAX79766.1|Phosphatase 2A inhibitor I2PP2A [Schistosoma japonicum] |
| TS.seq. Contig211 | 8.78744E-23 | gi|155964455|gb|ABU40277.1|Ts1 [Taenia solium] |
| TS.seq. Contig212 | 3.60586E-21 | gi|256075222|ref|XP_002573919.1|hypothetical protein [Schistosoma mansoni] >gi|238659110|emb|CAZ30152.1| expressed protein [Schistosoma mansoni] |
| TS.seq. Contig213 | 1.34878E-14 | gi|256089445|ref|XP_002580820.1|tetraspanin 42 invertebrate [Schistosoma mansoni] >gi|238666432|emb|CAZ37059.1| tetraspanin 42, invertebrate, putative [Schistosoma mansoni] |
| TS.seq. Contig214 | 1.40766E-25 | gi|226472398|emb|CAX77235.1|Ribosomal protein S11 [Schistosoma japonicum] |
| TS.seq. Contig218 | 1.58648E-40 | gi|56752659|gb|AAW24543.1|SJCHGC05202 protein [Schistosoma japonicum] |
| TS.seq. Contig219 | 2.30085E-12 | gi|257216051|emb|CAX83169.1|Ribosomal protein S5a [Schistosoma japonicum] |
| TS.seq. Contig220 | 4.55846E-25 | gi|124784850|gb|ABN15004.1|proliferating cell nuclear antigen [Taenia asiatica] |
| TS.seq. Contig221 | 2.85205E-43 | gi|94449819|gb|ABF19508.1|unknown [Schistosoma japonicum] >gi|226474526|emb|CAX71749.1| small subunit ribosomal protein S30e [Schistosoma japonicum] >gi|226474528|emb|CAX71750.1| small subunit ribosomal protein S30e [Schistosoma japonicum] >gi|226474530|emb|CAX71751.1| small subunit ribosomal protein S30e [Schistosoma japonicum] >gi|226476160|emb|CAX77931.1| small subunit ribosomal protein S30e [Schistosoma japonicum] >gi|226476162|emb|CAX77932.1| small subunit ribosomal protein S30e [Schistosoma japonicum] >gi|226476166|emb|CAX77934.1| small subunit ribosomal protein S30e [Schistosoma japonicum] >gi|226476168|emb|CAX77935.1| small subunit ribosomal protein S30e [Schistosoma japonicum] >gi|226476172|emb|CAX77937.1| small subunit ribosomal protein S30e [Schistosoma japonicum] >gi|226476174|emb|CAX77938.1| small subunit ribosomal protein S30e [Schistosoma japonicum] >gi|226476178|emb|CAX77940.1| small subunit ribosomal protein S30e [Schistosoma japonicum] >gi|226476182|emb|CAX77942.1| small subunit ribosomal protein S30e [Schistosoma japonicum] >gi|226476184|emb|CAX77943.1| small subunit ribosomal protein S30e [Schistosoma japonicum] >gi|226476186|emb|CAX77944.1| small subunit ribosomal protein S30e [Schistosoma japonicum] >gi|226476188|emb|CAX77945.1| small subunit ribosomal protein S30e [Schistosoma japonicum] >gi|226476190|emb|CAX77946.1| small subunit ribosomal protein S30e [Schistosoma japonicum] >gi|226476192|emb|CAX77947.1| small subunit ribosomal protein S30e [Schistosoma japonicum] >gi|226476194|emb|CAX77948.1| small subunit ribosomal protein S30e [Schistosoma japonicum] >gi|226476196|emb|CAX77949.1| small subunit ribosomal protein S30e [Schistosoma japonicum] >gi|226476198|emb|CAX77950.1| small subunit ribosomal protein S30e [Schistosoma japonicum] |
| TS.seq. Contig222 | 1.58098E-16 | gi|256085472|ref|XP_002578945.1|annexin [Schistosoma mansoni] >gi|238664334|emb|CAZ35183.1| annexin, putative [Schistosoma mansoni] |
| TS.seq. Contig224 | 6.38752E-71 | gi|256078729|ref|XP_002575647.1|hexokinase [Schistosoma mansoni] >gi|18277270|sp|Q26609.2|HXK_SCHMA RecName: Full=Hexokinase >gi|157830239|pdb|1BDG|A Chain A, Hexokinase From Schistosoma Mansoni Complexed With Glucose >gi|11387389|gb|AAA29894.2| hexokinase [Schistosoma mansoni] >gi|238660889|emb|CAZ31880.1| hexokinase [Schistosoma mansoni] |
| TS.seq. Contig225 | 1.49847E-11 | gi|256079165|ref|XP_002575860.1|hypothetical protein [Schistosoma mansoni] >gi|238661112|emb|CAZ32095.1| expressed protein [Schistosoma mansoni] |
| TS.seq. Contig226 | 3.96366E-36 | gi|257205870|emb|CAX82586.1|Smoothelin [Schistosoma japonicum] |
| TS.seq. Contig227 | 2.29082E-15 | gi|76155755|gb|AAX27034.2|SJCHGC08105 protein [Schistosoma japonicum] |
| TS.seq. Contig231 | 2.16492E-61 | gi|261266611|gb|ACX56268.1|enolase [Taenia asiatica] |
| TS.seq. Contig233 | 1.19335E-48 | gi|41055694|ref|NP_957253.1|ubiquitin-conjugating enzyme E2D 2 [Danio rerio] >gi|29124433|gb|AAH48896.1| Zgc:55886 protein [Danio rerio] >gi|41351441|gb|AAH65678.1| Zgc:55886 [Danio rerio] >gi|182890642|gb|AAI64949.1| Zgc:55886 protein [Danio rerio] |
| TS.seq. Contig235 | 4.88478E-10 | gi|45238335|emb|CAD12372.2|serine protease inhibitor [Echinococcus multilocularis] |
| TS.seq. Contig236 | 1.10049E-70 | gi|29841212|gb|AAP06225.1|similar to GenBank Accession Number AJ312339 putative ribosomal protein L27A protein in Oncorhynchus mykiss [Schistosoma japonicum] >gi|226470842|emb|CAX76854.1| large subunit ribosomal protein L27Ae [Schistosoma japonicum] >gi|226470844|emb|CAX76855.1| large subunit ribosomal protein L27Ae [Schistosoma japonicum] >gi|226470846|emb|CAX76856.1| large subunit ribosomal protein L27Ae [Schistosoma japonicum] >gi|226470848|emb|CAX76857.1| large subunit ribosomal protein L27Ae [Schistosoma japonicum] >gi|226470850|emb|CAX76858.1| large subunit ribosomal protein L27Ae [Schistosoma japonicum] >gi|226470852|emb|CAX76859.1| large subunit ribosomal protein L27Ae [Schistosoma japonicum] >gi|226470854|emb|CAX76860.1| large subunit ribosomal protein L27Ae [Schistosoma japonicum] >gi|226470858|emb|CAX76862.1| large subunit ribosomal protein L27Ae [Schistosoma japonicum] >gi|226470862|emb|CAX76864.1| large subunit ribosomal protein L27Ae [Schistosoma japonicum] >gi|226470864|emb|CAX76865.1| large subunit ribosomal protein L27Ae [Schistosoma japonicum] >gi|226470866|emb|CAX76866.1| large subunit ribosomal protein L27Ae [Schistosoma japonicum] |
| TS.seq. Contig237 | 1.28053E-52 | gi|56753812|gb|AAW25103.1|SJCHGC06900 protein [Schistosoma japonicum] |
| TS.seq. Contig240 | 2.43641E-53 | gi|256086414|ref|XP_002579394.1|inhibitor of apoptosis (iap) domain family member [Schistosoma mansoni] >gi|238664827|emb|CAZ35633.1| inhibitor of apoptosis (iap) domain family member, putative [Schistosoma mansoni] |
| TS.seq. Contig241 | 6.0299E-47 | gi|256083541|ref|XP_002578001.1|uracil-DNA glycosylase [Schistosoma mansoni] >gi|238663347|emb|CAZ34239.1| uracil-DNA glycosylase, putative [Schistosoma mansoni] |
| TS.seq. Contig242 | 6.8962E-9 | gi|226480834|emb|CAX73514.1|hypothetical protein [Schistosoma japonicum] |
| TS.seq. Contig244 | 1.56235E-8 | gi|56756264|gb|AAW26307.1|SJCHGC09085 protein [Schistosoma japonicum] |
| TS.seq. Contig245 | 8.11966E-37 | gi|66536937|ref|XP_623945.1|PREDICTED: similar to Transmembrane 9 superfamily protein member 3 precursor (SM-11044-binding protein) (EP70-P-iso) [Apis mellifera] |
| TS.seq. Contig246 | 5.70368E-51 | gi|256073492|ref|XP_002573064.1|40S ribosomal protein S15 [Schistosoma mansoni] >gi|238658235|emb|CAZ29296.1| 40S ribosomal protein S15, putative [Schistosoma mansoni] |
| TS.seq. Contig247 | 2.47772E-46 | gi|226475180|emb|CAX71884.1|ribosomal protein L21 [Schistosoma japonicum] |
| TS.seq. Contig250 | 5.96389E-24 | gi|256082883|ref|XP_002577681.1|26S proteasome subunit [Schistosoma mansoni] >gi|238663009|emb|CAZ33919.1| 26S proteasome subunit P28-related [Schistosoma mansoni] |
| TS.seq. Contig251 | 9.86506E-53 | gi|116325975|ref|NP_001070823.1|ATP synthase, H+ transporting, mitochondrial F1 complex, alpha subunit 1, cardiac muscle [Danio rerio] >gi|115528192|gb|AAI24812.1| ATP synthase, H+ transporting, mitochondrial F1 complex, alpha subunit 1, cardiac muscle [Danio rerio] |
| TS.seq. Contig253 | 4.95129E-29 | gi|226479094|emb|CAX73042.1|IWS1 homolog [Schistosoma japonicum] |
| TS.seq. Contig254 | 7.20285E-6 | gi|193589554|ref|XP_001944016.1|PREDICTED: similar to vesicle amine transport protein [Acyrthosiphon pisum] |
| TS.seq. Contig256 | 3.21965E-24 | gi|256076310|ref|XP_002574456.1|regulator of G protein signaling [Schistosoma mansoni] >gi|238659661|emb|CAZ30689.1| regulator of G protein signaling, putative [Schistosoma mansoni] |
| TS.seq. Contig257 | 1.11061E-38 | gi|256089948|ref|XP_002580997.1|lupus la ribonucleoprotein [Schistosoma mansoni] >gi|238666758|emb|CAZ37236.1| lupus la ribonucleoprotein, putative [Schistosoma mansoni] |
| TS.seq. Contig259 | 1.04217E-34 | gi|6016537|sp|Q04820.2|MDHC_ECHGRRecName: Full=Malate dehydrogenase, cytoplasmic >gi|3386331|gb|AAC28239.1| malate dehydrogenase [Echinococcus granulosus] |
| TS.seq. Contig262 | 5.37095E-6 | gi|47209404|emb|CAF90870.1|unnamed protein product [Tetraodon nigroviridis] |
| TS.seq. Contig264 | 4.55461E-8 | gi|223648408|gb|ACN10962.1|Long-chain-fatty-acid--CoA ligase 4 [Salmo salar] |
| TS.seq. Contig265 | 9.84564E-10 | gi|186926582|gb|ACC95495.1|EG95 [Echinococcus multilocularis] >gi|186926584|gb|ACC95496.1| EG95 [Echinococcus multilocularis] |
| TS.seq. Contig266 | 1.15062E-11 | gi|256080272|ref|XP_002576406.1|hypothetical protein [Schistosoma mansoni] >gi|238661673|emb|CAZ32643.1| hypothetical protein [Schistosoma mansoni] |
| TS.seq. Contig267 | 1.08334E-17 | gi|198425003|ref|XP_002123716.1|PREDICTED: similar to Cytochrome c oxidase copper chaperone [Ciona intestinalis] |
| TS.seq. Contig269 | 1.97812E-19 | gi|56758692|gb|AAW27486.1|SJCHGC05764 protein [Schistosoma japonicum] |
| TS.seq. Contig271 | 2.5864E-11 | gi|256075607|ref|XP_002574109.1|hypothetical protein [Schistosoma mansoni] >gi|238659305|emb|CAZ30342.1| expressed protein [Schistosoma mansoni] |
| TS.seq. Contig274 | 3.55313E-5 | gi|256079426|ref|XP_002575988.1|hypothetical protein [Schistosoma mansoni] >gi|238661245|emb|CAZ32223.1| expressed protein [Schistosoma mansoni] |
| TS.seq. Contig275 | 1.56861E-33 | gi|256077388|ref|XP_002574987.1|msf1/px19 related [Schistosoma mansoni] >gi|238660208|emb|CAZ31220.1| msf1/px19 related [Schistosoma mansoni] |
| TS.seq. Contig278 | 2.26768E-22 | gi|256078439|ref|XP_002575503.1|hypothetical protein [Schistosoma mansoni] >gi|238660743|emb|CAZ31736.1| expressed protein [Schistosoma mansoni] |
| TS.seq. Contig279 | 1.20719E-32 | gi|58269760|ref|XP_572036.1|succinate dehydrogenase iron-sulfur subunit [Cryptococcus neoformans var. neoformans JEC21] >gi|57228272|gb|AAW44729.1| succinate dehydrogenase iron-sulfur subunit, putative [Cryptococcus neoformans var. neoformans JEC21] |
| TS.seq. Contig280 | 8.66834E-52 | gi|256071126|ref|XP_002571892.1|vacuolar ATP synthase proteolipid subunit 1 2 3 [Schistosoma mansoni] >gi|238657041|emb|CAZ28122.1| vacuolar ATP synthase proteolipid subunit 1, 2, 3,, putative [Schistosoma mansoni] |
| TS.seq. Contig283 | 3.90144E-15 | gi|224094412|ref|XP_002189060.1|PREDICTED: leukotriene A4 hydrolase [Taeniopygia guttata] |
| TS.seq. Contig284 | 1.79472E-29 | gi|226489809|emb|CAX75055.1|inositol(myo)-1(or 4)-monophosphatase 2 [Schistosoma japonicum] |
| TS.seq. Contig285 | 1.68741E-31 | gi|256083401|ref|XP_002577933.1|40s ribosomal protein [Schistosoma mansoni] >gi|108861818|gb|ABG21811.1| 40S ribosomal protein S20-like protein [Schistosoma mansoni] >gi|238663275|emb|CAZ34171.1| 40s rRNA protein, putative [Schistosoma mansoni] |
| TS.seq. Contig286 | 6.03571E-12 | gi|256075188|ref|XP_002573902.1|multidrug resistance protein [Schistosoma mansoni] >gi|238659093|emb|CAZ30135.1| multidrug resistance pump, putative [Schistosoma mansoni] |
| TS.seq. Contig287 | 4.10927E-41 | gi|29497690|gb|AAN74963.1|oncosphere protein TSOL16B [Taenia solium] |
| TS.seq. Contig290 | 4.25971E-26 | gi|260867960|ref|YP_003234362.1|altronate oxidoreductase, NAD-dependent [Escherichia coli O111:H- str. 11128] >gi|257764316|dbj|BAI35811.1| altronate oxidoreductase, NAD-dependent [Escherichia coli O111:H- str. 11128] |
| TS.seq. Contig291 | 1.66851E-10 | gi|157756067|ref|XP_001670377.1|Hypothetical protein CBG05956 [Caenorhabditis briggsae AF16] >gi|187034581|emb|CAP26398.1| Hypothetical protein CBG05956 [Caenorhabditis briggsae] |
| TS.seq. Contig292 | 7.87481E-17 | gi|256062499|ref|XP_002570342.1|arp2/3 complex subunit 41-related [Schistosoma mansoni] >gi|227294401|emb|CAY18732.1| arp2/3 complex subunit 41-related [Schistosoma mansoni] |
| TS.seq. Contig293 | 5.91639E-5 | gi|256076905|ref|XP_002574749.1|tetraspanin-CD63 receptor [Schistosoma mansoni] >gi|238659963|emb|CAZ30982.1| tetraspanin-CD63 receptor , putative [Schistosoma mansoni] |
| TS.seq. Contig295 | 2.07529E-5 | gi|68480044|gb|AAY97892.1|unknown [Schistosoma japonicum] |
| TS.seq. Contig298 | 2.44559E-20 | gi|256088800|ref|XP_002580512.1|zinc finger protein [Schistosoma mansoni] >gi|238666095|emb|CAZ36751.1| zinc finger protein, putative [Schistosoma mansoni] |
| TS.seq. Contig299 | 3.24426E-34 | gi|256084664|ref|XP_002578547.1|arp2/3 complex 16 kD subunit (P16-arc) [Schistosoma mansoni] >gi|238663924|emb|CAZ34785.1| arp2/3 complex 16 kD subunit (P16-arc), putative [Schistosoma mansoni] |
| TS.seq. Contig300 | 1.97283E-22 | gi|256073473|ref|XP_002573055.1|hypothetical protein [Schistosoma mansoni] >gi|238658225|emb|CAZ29287.1| expressed protein [Schistosoma mansoni] |
| TS.seq. Contig301 | 4.89788E-20 | gi|256092874|ref|XP_002582102.1|hepatoma up-regulated protein [Schistosoma mansoni] >gi|238804745|emb|CAZ39202.1| hepatoma up-regulated protein, putative [Schistosoma mansoni] |
| TS.seq. Contig302 | 3.07048E-65 | gi|256072591|ref|XP_002572618.1|40S ribosomal protein S4 [Schistosoma mansoni] >gi|238657780|emb|CAZ28850.1| 40S ribosomal protein S4, putative [Schistosoma mansoni] |
| TS.seq. Contig303 | 8.87554E-58 | gi|256079007|ref|XP_002575783.1|zyxin/trip6 [Schistosoma mansoni] >gi|238661031|emb|CAZ32018.1| zyxin/trip6, putative [Schistosoma mansoni] |
| TS.seq. Contig304 | 4.33502E-44 | gi|257206712|emb|CAX82984.1|hypothetical protein [Schistosoma japonicum] |
| TS.seq. Contig305 | 4.30702E-64 | gi|256076747|ref|XP_002574671.1|arp2/3 complex 21 kD subunit [Schistosoma mansoni] >gi|238659883|emb|CAZ30904.1| arp2/3 complex 21 kD subunit, putative [Schistosoma mansoni] |
| TS.seq. Contig306 | 2.76955E-30 | gi|241998218|ref|XP_002433752.1|AP-2 complex subunit alpha-1, putative [Ixodes scapularis] >gi|215495511|gb|EEC05152.1| AP-2 complex subunit alpha-1, putative [Ixodes scapularis] |
| TS.seq. Contig307 | 1.51464E-38 | gi|226472996|emb|CAX71184.1|deoxyribonuclease [Schistosoma japonicum] |
| TS.seq. Contig308 | 9.36089E-38 | gi|56755932|gb|AAW26144.1|SJCHGC02223 protein [Schistosoma japonicum] |
| TS.seq. Contig309 | 1.55022E-20 | gi|56759044|gb|AAW27662.1|SJCHGC09280 protein [Schistosoma japonicum] |
| TS.seq. Contig310 | 1.53296E-33 | gi|148717323|dbj|BAF63675.1|elongation factor 1 alpha [Echinococcus granulosus] >gi|148717325|dbj|BAF63676.1| elongation factor 1 alpha [Echinococcus canadensis] >gi|148717327|dbj|BAF63677.1| elongation factor 1 alpha [Echinococcus canadensis] >gi|148717329|dbj|BAF63678.1| elongation factor 1 alpha [Echinococcus ortleppi] >gi|148717333|dbj|BAF63680.1| elongation factor 1 alpha [Echinococcus oligarthrus] |
| TS.seq. Contig312 | 1.10758E-15 | gi|226484644|emb|CAX74231.1|Pre-mRNA-splicing factor cwc15 [Schistosoma japonicum] |
| TS.seq. Contig316 | 3.85861E-107 | gi|13603811|gb|AAK31943.1|AF267118_1activated oncosphere TSO45-5A [Taenia solium] |
| TS.seq. Contig317 | 2.66496E-60 | gi|256083900|ref|XP_002578173.1|tubulin tyrosine ligase [Schistosoma mansoni] >gi|238663534|emb|CAZ34411.1| tubulin tyrosine ligase-related [Schistosoma mansoni] |
| TS.seq. Contig318 | 7.5917E-11 | gi|223037336|gb|ACM79010.1|Kunitz protein 8 [Echinococcus granulosus] |
| TS.seq. Contig321 | 1.74255E-7 | gi|260792904|ref|XP_002591454.1|hypothetical protein BRAFLDRAFT_187345 [Branchiostoma floridae] >gi|229276659|gb|EEN47465.1| hypothetical protein BRAFLDRAFT_187345 [Branchiostoma floridae] |
| TS.seq. Contig326 | 5.63473E-18 | gi|171473960|gb|AAW26809.2|SJCHGC04889 protein [Schistosoma japonicum] |
| TS.seq. Contig327 | 2.76333E-13 | gi|1710592|sp|P50879.1|RLA2_TAESORecName: Full=60S acidic ribosomal protein P2 >gi|662268|gb|AAB03732.1| acidic ribosomal phosphoprotein |
| TS.seq. Contig328 | 8.57856E-35 | gi|256087629|ref|XP_002579968.1|family C2 unassigned peptidase (C02 family) [Schistosoma mansoni] >gi|238665468|emb|CAZ36207.1| family C2 unassigned peptidase (C02 family) [Schistosoma mansoni] |
| TS.seq. Contig329 | 1.01815E-21 | gi|256088230|ref|XP_002580252.1|copine [Schistosoma mansoni] >gi|238665785|emb|CAZ36491.1| copine, putative [Schistosoma mansoni] |
| TS.seq. Contig330 | 2.62898E-65 | gi|66521738|ref|XP_393545.2|PREDICTED: similar to lethal (1) G0030 CG3861-PA, isoform A [Apis mellifera] |
| TS.seq. Contig332 | 6.12938E-48 | gi|13603807|gb|AAK31941.1|AF267116_1activated oncosphere TSO45-2A [Taenia solium] |
| TS.seq. Contig333 | 7.70777E-73 | gi|229366216|gb|ACQ58088.1|Ras-related protein Rap-1b precursor [Anoplopoma fimbria] |
| TS.seq. Contig334 | 2.39008E-97 | gi|4960053|gb|AAD34598.1|AF147955_1antigen cC1 [Taenia solium] |
| TS.seq. Contig335 | 2.27129E-47 | gi|256093010|ref|XP_002582170.1|adenosine deaminase [Schistosoma mansoni] >gi|238804813|emb|CAZ39270.1| adenosine deaminase, putative [Schistosoma mansoni] |
| TS.seq. Contig337 | 6.09063E-16 | gi|226487920|emb|CAX75625.1|hypothetical protein [Schistosoma japonicum] |
| TS.seq. Contig338 | 1.49646E-20 | gi|157929876|gb|ABW04126.1|ATP synthase H+ transporting F0 complex subunit c [Epinephelus coioides] |
| TS.seq. Contig341 | 4.09664E-7 | gi|56756116|gb|AAW26236.1|SJCHGC06142 protein [Schistosoma japonicum] |
| TS.seq. Contig343 | 3.67921E-46 | gi|256085166|ref|XP_002578794.1|hypothetical protein [Schistosoma mansoni] >gi|238664179|emb|CAZ35032.1| WD-repeat protein, putative [Schistosoma mansoni] |
| TS.seq. Contig344 | 1.68007E-47 | gi|256080234|ref|XP_002576387.1|hypothetical protein [Schistosoma mansoni] >gi|238661654|emb|CAZ32624.1| expressed protein [Schistosoma mansoni] |
| TS.seq. Contig345 | 1.64609E-74 | gi|22000696|gb|AAM88223.1|AF523865_1TSO45-A2 [Taenia solium] |
| TS.seq. Contig346 | 2.32997E-21 | gi|195016646|ref|XP_001984454.1|GH16468 [Drosophila grimshawi] >gi|193897936|gb|EDV96802.1| GH16468 [Drosophila grimshawi] |
| TS.seq. Contig347 | 2.50877E-14 | gi|256080568|ref|XP_002576552.1|hypothetical protein [Schistosoma mansoni] >gi|238661823|emb|CAZ32789.1| expressed protein [Schistosoma mansoni] |
| TS.seq. Contig348 | 3.5226E-82 | gi|1053159|gb|AAB05911.1|TGTP1 [Taenia solium] |
| TS.seq. Contig349 | 7.23017E-38 | gi|256084813|ref|XP_002578620.1|30S ribosomal protein S12 [Schistosoma mansoni] >gi|238664000|emb|CAZ34858.1| 30S ribosomal protein S12 family member, putative [Schistosoma mansoni] |
| TS.seq. Contig350 | 5.72735E-133 | gi|256076076|ref|XP_002574340.1|protein phosphatase-1 [Schistosoma mansoni] >gi|238659543|emb|CAZ30573.1| protein phosphatase-1, putative [Schistosoma mansoni] |
| TS.seq. Contig355 | 6.44575E-10 | gi|226485797|emb|CAX75318.1|hypothetical protein [Schistosoma japonicum] |
| TS.seq. Contig356 | 2.98289E-23 | gi|60687708|gb|AAX30187.1|SJCHGC01650 protein [Schistosoma japonicum] >gi|226469656|emb|CAX76658.1| ribosomal protein L38 [Schistosoma japonicum] >gi|226469658|emb|CAX76659.1| ribosomal protein L38 [Schistosoma japonicum] >gi|226469660|emb|CAX76660.1| ribosomal protein L38 [Schistosoma japonicum] >gi|226469662|emb|CAX76661.1| ribosomal protein L38 [Schistosoma japonicum] >gi|226469664|emb|CAX76662.1| ribosomal protein L38 [Schistosoma japonicum] >gi|226473004|emb|CAX71188.1| ribosomal protein L38 [Schistosoma japonicum] >gi|226473006|emb|CAX71189.1| ribosomal protein L38 [Schistosoma japonicum] >gi|226473008|emb|CAX71190.1| ribosomal protein L38 [Schistosoma japonicum] >gi|226473010|emb|CAX71191.1| ribosomal protein L38 [Schistosoma japonicum] |
| TS.seq. Contig357 | 3.19976E-34 | gi|256082510|ref|XP_002577498.1|myosin light chain [Schistosoma mansoni] >gi|160971|gb|AAA29873.1| essential myosin light chain >gi|41176441|gb|AAR99584.1| myosin light chain [Schistosoma mansoni] >gi|238662819|emb|CAZ33736.1| myosin light chain, putative [Schistosoma mansoni] |
| TS.seq. Contig359 | 1.15408E-68 | gi|41055694|ref|NP_957253.1|ubiquitin-conjugating enzyme E2D 2 [Danio rerio] >gi|29124433|gb|AAH48896.1| Zgc:55886 protein [Danio rerio] >gi|41351441|gb|AAH65678.1| Zgc:55886 [Danio rerio] >gi|182890642|gb|AAI64949.1| Zgc:55886 protein [Danio rerio] |
| TS.seq. Contig361 | 6.85427E-4 | gi|226472250|emb|CAX77161.1|hypothetical protein [Schistosoma japonicum] >gi|226472258|emb|CAX77165.1| hypothetical protein [Schistosoma japonicum] >gi|226472262|emb|CAX77167.1| hypothetical protein [Schistosoma japonicum] >gi|226472266|emb|CAX77169.1| hypothetical protein [Schistosoma japonicum] >gi|226472268|emb|CAX77170.1| hypothetical protein [Schistosoma japonicum] >gi|226472270|emb|CAX77171.1| hypothetical protein [Schistosoma japonicum] >gi|226472272|emb|CAX77172.1| hypothetical protein [Schistosoma japonicum] >gi|226473446|emb|CAX71408.1| hypothetical protein [Schistosoma japonicum] >gi|226473448|emb|CAX71409.1| hypothetical protein [Schistosoma japonicum] >gi|226473450|emb|CAX71410.1| hypothetical protein [Schistosoma japonicum] >gi|226473452|emb|CAX71411.1| hypothetical protein [Schistosoma japonicum] |
| TS.seq. Contig362 | 8.3311E-35 | gi|146741270|dbj|BAF62290.1|calpain [Schistosoma haematobium] |
| TS.seq. Contig363 | 3.12358E-21 | gi|260804392|ref|XP_002597072.1|hypothetical protein BRAFLDRAFT_57028 [Branchiostoma floridae] >gi|229282334|gb|EEN53084.1| hypothetical protein BRAFLDRAFT_57028 [Branchiostoma floridae] |
| TS.seq. Contig364 | 2.30736E-22 | gi|224922691|dbj|BAH28838.1|G1Y162 protein [Echinococcus granulosus] >gi|224922693|dbj|BAH28839.1| G1Y162 protein [Echinococcus granulosus] |
| TS.seq. Contig367 | 7.12653E-14 | gi|223037336|gb|ACM79010.1|Kunitz protein 8 [Echinococcus granulosus] |
| TS.seq. Contig368 | 1.28668E-18 | gi|158934366|emb|CAO82075.1|HP6 protein [Taenia solium] |
| TS.seq. Contig369 | 1.2271E-42 | gi|37786712|gb|AAP47268.1|T24 [Taenia solium] >gi|112785172|gb|ABI20734.1| membrane protein T24 [Taenia solium] |
| TS.seq. Contig370 | 3.58323E-26 | gi|56435063|gb|AAV91323.1|succinate dehydrogenase iron-sulfur protein [Schistosoma japonicum] >gi|56757549|gb|AAW26938.1| SJCHGC06829 protein [Schistosoma japonicum] >gi|226470530|emb|CAX70545.1| succinate dehydrogenase complex, subunit B, iron sulfur [Schistosoma japonicum] >gi|226470532|emb|CAX70546.1| succinate dehydrogenase complex, subunit B, iron sulfur [Schistosoma japonicum] >gi|226470534|emb|CAX70547.1| succinate dehydrogenase complex, subunit B, iron sulfur [Schistosoma japonicum] >gi|226470536|emb|CAX70548.1| succinate dehydrogenase complex, subunit B, iron sulfur [Schistosoma japonicum] >gi|226487080|emb|CAX75405.1| succinate dehydrogenase complex, subunit B, iron sulfur [Schistosoma japonicum] |
| TS.seq. Contig372 | 1.23031E-89 | gi|21912540|emb|CAD21525.1|hypothetical protein [Taenia solium] |
| TS.seq. Contig373 | 5.27393E-12 | gi|242009491|ref|XP_002425519.1|conserved hypothetical protein [Pediculus humanus corporis] >gi|212509374|gb|EEB12781.1| conserved hypothetical protein [Pediculus humanus corporis] |
| TS.seq. Contig374 | 8.80537E-33 | gi|226470668|emb|CAX76767.1|Ribosomal protein L37a [Schistosoma japonicum] >gi|226470672|emb|CAX76769.1| Ribosomal protein L37a [Schistosoma japonicum] >gi|226470676|emb|CAX76771.1| Ribosomal protein L37a [Schistosoma japonicum] >gi|226470678|emb|CAX76772.1| Ribosomal protein L37a [Schistosoma japonicum] >gi|226470680|emb|CAX76773.1| Ribosomal protein L37a [Schistosoma japonicum] >gi|226473148|emb|CAX71260.1| Ribosomal protein L37a [Schistosoma japonicum] >gi|226473150|emb|CAX71261.1| Ribosomal protein L37a [Schistosoma japonicum] |
| TS.seq. Contig375 | 6.52101E-117 | gi|257449357|gb|ACV53651.1|guanine nucleotide-binding protein beta polypeptide 2-like 1 [Paramisgurnus dabryanus] |
| TS.seq. Contig376 | 4.58268E-8 | gi|256076887|ref|XP_002574740.1|calcium-binding protein [Schistosoma mansoni] >gi|115391|sp|P13566.1|CABP_SCHMA RecName: Full=Calcium-binding protein; Short=CaBP >gi|160939|gb|AAA29860.1| calcium-binding protein >gi|160941|gb|AAA29861.1| calcium binding protein >gi|238659954|emb|CAZ30973.1| calcium-binding protein, putative [Schistosoma mansoni] |
| TS.seq. Contig377 | 3.15717E-33 | gi|62178030|gb|AAX73175.1|putative 14-3-3 protein [Echinococcus granulosus] |
| TS.seq. Contig379 | 2.12103E-45 | gi|29841130|gb|AAP06143.1|SJCHGC06082 protein [Schistosoma japonicum] >gi|226468854|emb|CAX76455.1| Cytochrome c proximal [Schistosoma japonicum] >gi|226468856|emb|CAX76456.1| Cytochrome c proximal [Schistosoma japonicum] >gi|226468858|emb|CAX76457.1| Cytochrome c proximal [Schistosoma japonicum] >gi|226468860|emb|CAX76458.1| Cytochrome c proximal [Schistosoma japonicum] >gi|226468862|emb|CAX76459.1| Cytochrome c proximal [Schistosoma japonicum] >gi|226468864|emb|CAX76460.1| Cytochrome c proximal [Schistosoma japonicum] >gi|226468866|emb|CAX76461.1| Cytochrome c proximal [Schistosoma japonicum] >gi|226468868|emb|CAX76462.1| Cytochrome c proximal [Schistosoma japonicum] >gi|226472868|emb|CAX71120.1| Cytochrome c proximal [Schistosoma japonicum] >gi|226472870|emb|CAX71121.1| Cytochrome c proximal [Schistosoma japonicum] |
| TS.seq. Contig380 | 1.13827E-22 | gi|59709854|gb|AAW88557.1|oncosphere protein Tso31b [Taenia solium] >gi|111146851|gb|ABH07374.1| oncosphere protein Tso31b [Taenia solium] |
| TS.seq. Contig381 | 3.59067E-23 | gi|22004048|dbj|BAC06474.1|ubiquitin [Ciona savignyi] |
| TS.seq. Contig382 | 3.7141E-6 | gi|256070457|ref|XP_002571559.1|hypothetical protein [Schistosoma mansoni] >gi|238656703|emb|CAZ27789.1| hypothetical protein [Schistosoma mansoni] |
| TS.seq. Contig383 | 1.85207E-129 | gi|6016537|sp|Q04820.2|MDHC_ECHGRRecName: Full=Malate dehydrogenase, cytoplasmic >gi|3386331|gb|AAC28239.1| malate dehydrogenase [Echinococcus granulosus] |
| TS.seq. Contig385 | 1.32197E-68 | gi|124783098|gb|ABN14904.1|ribosomal protein S18 [Taenia asiatica] |
| TS.seq. Contig387 | 5.2412E-10 | gi|256072528|ref|XP_002572587.1|Sj-Ts4 protein [Schistosoma mansoni] >gi|238657748|emb|CAZ28819.1| Sj-Ts4 protein , putative [Schistosoma mansoni] |
| TS.seq. Contig388 | 6.26438E-42 | gi|155964455|gb|ABU40277.1|Ts1 [Taenia solium] |
| TS.seq. Contig389 | 1.96567E-44 | gi|116687782|gb|AAT74668.2|cysteine-rich secreted protein 2 precursor [Mesocestoides vogae] |
| TS.seq. Contig391 | 1.16797E-92 | gi|76156528|gb|AAX27719.2|SJCHGC00991 protein [Schistosoma japonicum] |
| TS.seq. Contig392 | 1.45777E-39 | gi|189502988|gb|ACE06875.1|unknown [Schistosoma japonicum] |
| TS.seq. Contig393 | 1.42429E-4 | gi|257216364|emb|CAX82387.1|Lymphocyte cytosolic protein 1 [Schistosoma japonicum] |
| TS.seq. Contig396 | 3.14077E-134 | gi|148717323|dbj|BAF63675.1|elongation factor 1 alpha [Echinococcus granulosus] >gi|148717325|dbj|BAF63676.1| elongation factor 1 alpha [Echinococcus canadensis] >gi|148717327|dbj|BAF63677.1| elongation factor 1 alpha [Echinococcus canadensis] >gi|148717329|dbj|BAF63678.1| elongation factor 1 alpha [Echinococcus ortleppi] >gi|148717333|dbj|BAF63680.1| elongation factor 1 alpha [Echinococcus oligarthrus] |
| TS.seq. Contig397 | 1.10191E-81 | gi|260799919|ref|XP_002594905.1|hypothetical protein BRAFLDRAFT_289556 [Branchiostoma floridae] >gi|229280143|gb|EEN50916.1| hypothetical protein BRAFLDRAFT_289556 [Branchiostoma floridae] |
| TS.seq. Contig398 | 3.60703E-33 | gi|56758982|gb|AAW27631.1|SJCHGC05857 protein [Schistosoma japonicum] |
| TS.seq. Contig400 | 1.29207E-53 | gi|56753784|gb|AAW25089.1|SJCHGC01515 protein [Schistosoma japonicum] |
| TS.seq. Contig401 | 7.7594E-13 | gi|256079415|ref|XP_002575983.1|hypothetical protein [Schistosoma mansoni] >gi|238661239|emb|CAZ32218.1| expressed protein [Schistosoma mansoni] |
| TS.seq. Contig403 | 6.98717E-44 | gi|256083504|ref|XP_002577983.1|programmed cell death protein [Schistosoma mansoni] >gi|238663328|emb|CAZ34221.1| programmed cell death protein, putative [Schistosoma mansoni] |
| TS.seq. Contig404 | 2.94059E-24 | gi|226485827|emb|CAX75333.1|Rho GDP-dissociation inhibitor 2 [Schistosoma japonicum] |
| TS.seq. Contig405 | 2.41537E-70 | gi|209967605|gb|ACJ02407.1|TSP6 [Echinococcus multilocularis] |
| TS.seq. Contig407 | 2.21846E-59 | gi|256084646|ref|XP_002578538.1|platelet-activating factor acetylhydrolase ib [Schistosoma mansoni] >gi|238663915|emb|CAZ34776.1| platelet-activating factor acetylhydrolase ib, putative [Schistosoma mansoni] |
| TS.seq. Contig409 | 4.79765E-7 | gi|256074267|ref|XP_002573447.1|protein phosphatase 1 inhibitor potentiated by protein kinase C [Schistosoma mansoni] >gi|238658627|emb|CAZ29679.1| protein phosphatase 1 inhibitor potentiated by protein kinase C, putative [Schistosoma mansoni] |
| TS.seq. Contig411 | 1.98996E-11 | gi|1658497|gb|AAC47529.1|W1/2 [Taenia ovis] |
| TS.seq. Contig412 | 1.47643E-67 | gi|56753167|gb|AAW24793.1|SJCHGC02369 protein [Schistosoma japonicum] |
| TS.seq. Contig413 | 3.5697E-46 | gi|256073903|ref|XP_002573267.1|cell division control protein 48 aaa family protein [Schistosoma mansoni] >gi|238658443|emb|CAZ29499.1| cell division control protein 48 aaa family protein (transitional endoplasmic reticulum atpase), putative [Schistosoma mansoni] |
| TS.seq. Contig414 | 6.6627E-15 | gi|256050212|ref|XP_002569521.1|hypothetical protein [Schistosoma mansoni] >gi|227282394|emb|CAY18490.1| hypothetical protein [Schistosoma mansoni] |
| TS.seq. Contig415 | 1.47145E-23 | gi|156550793|ref|XP_001607240.1|PREDICTED: similar to DnaJ homolog subfamily A member 1 isoform 1 [Nasonia vitripennis] |
| TS.seq. Contig416 | 2.12378E-5 | gi|226480632|emb|CAX73413.1|hypothetical protein [Schistosoma japonicum] |
| TS.seq. Contig417 | 3.74697E-84 | gi|20161472|dbj|BAB90396.1|ADP-ribosylation factor [Oryza sativa Japonica Group] |
| TS.seq. Contig418 | 1.54869E-73 | gi|256072403|ref|XP_002572525.1|arp2/3 [Schistosoma mansoni] >gi|238657685|emb|CAZ28757.1| arp2/3, putative [Schistosoma mansoni] |
| TS.seq. Contig420 | 1.38469E-97 | gi|124782938|gb|ABN14891.1|ribosomal protein L7a [Taenia asiatica] |
| TS.seq. Contig421 | 3.61955E-55 | gi|56754181|gb|AAW25278.1|SJCHGC01355 protein [Schistosoma japonicum] |
| TS.seq. Contig426 | 1.51339E-20 | gi|256088617|ref|XP_002580425.1|hypothetical protein [Schistosoma mansoni] >gi|238665999|emb|CAZ36664.1| hypothetical protein [Schistosoma mansoni] |
| TS.seq. Contig427 | 2.15144E-10 | gi|256075417|ref|XP_002574016.1|hypothetical protein [Schistosoma mansoni] >gi|238659208|emb|CAZ30249.1| expressed protein [Schistosoma mansoni] |
| TS.seq. Contig428 | 2.44479E-21 | gi|242054715|ref|XP_002456503.1|hypothetical protein SORBIDRAFT_03g037490 [Sorghum bicolor] >gi|241928478|gb|EES01623.1| hypothetical protein SORBIDRAFT_03g037490 [Sorghum bicolor] |
| TS.seq. Contig432 | 3.18992E-56 | gi|261289849|ref|XP_002611787.1|hypothetical protein BRAFLDRAFT_115833 [Branchiostoma floridae] >gi|229297158|gb|EEN67796.1| hypothetical protein BRAFLDRAFT_115833 [Branchiostoma floridae] |
| TS.seq. Contig433 | 1.90745E-123 | gi|261266611|gb|ACX56268.1|enolase [Taenia asiatica] |
| TS.seq. Contig434 | 1.721E-42 | gi|109087215|ref|XP_001085541.1|PREDICTED: lactate dehydrogenase B [Macaca mulatta] |
| TS.seq. Contig435 | 4.25367E-14 | gi|226484872|emb|CAX79713.1|ubiquitin C [Schistosoma japonicum] |
| TS.seq. Contig438 | 7.98378E-121 | gi|226486796|emb|CAX74475.1|pyruvate dehydrogenase E1 component, beta subunit [Schistosoma japonicum] |
| TS.seq. Contig439 | 5.50699E-34 | gi|256078558|ref|XP_002575562.1|dynein light chain [Schistosoma mansoni] >gi|238660803|emb|CAZ31795.1| dynein light chain, putative [Schistosoma mansoni] |
| TS.seq. Contig440 | 3.14892E-30 | gi|24158629|pdb|1GZS|AChain A, Crystal Structure Of The Complex Between The Gef Domain Of The Salmonella Typhimurium Sope Toxin And Human Cdc42 >gi|24158631|pdb|1GZS|C Chain C, Crystal Structure Of The Complex Between The Gef Domain Of The Salmonella Typhimurium Sope Toxin And Human Cdc42 |
| TS.seq. Contig441 | 3.08471E-24 | gi|148906792|gb|ABR16542.1|unknown [Picea sitchensis] |
| TS.seq. Contig443 | 2.97944E-52 | gi|226478978|emb|CAX72984.1|NADP-dependent malic enzyme [Schistosoma japonicum] |
| TS.seq. Contig444 | 6.69529E-74 | gi|124783010|gb|ABN14896.1|ribosomal protein L23a [Taenia asiatica] |
| TS.seq. Contig445 | 1.59087E-124 | gi|29825896|gb|AAN63052.1|thioredoxin glutathione reductase [Echinococcus granulosus] |
| TS.seq. Contig446 | 2.84006E-82 | gi|39919140|emb|CAD89011.1|PDZ-domain factor 1 [Echinococcus multilocularis] |
| TS.seq. Contig447 | 2.56888E-10 | gi|226479728|emb|CAX73160.1|SH3-binding, glutamic acid-rich protein [Schistosoma japonicum] |
| TS.seq. Contig448 | 3.08546E-6 | gi|209967595|gb|ACJ02402.1|TSP1 [Echinococcus multilocularis] |
| TS.seq. Contig449 | 4.02177E-31 | gi|226479818|emb|CAX73205.1|Four and a half LIM domains protein 3 [Schistosoma japonicum] |
| TS.seq. Contig450 | 2.59397E-4 | gi|39940656|ref|XP_359865.1|hypothetical protein MGG_04912 [Magnaporthe grisea 70-15] >gi|145010852|gb|EDJ95508.1| hypothetical protein MGG_04912 [Magnaporthe grisea 70-15] |
| TS.seq. Contig452 | 5.76233E-64 | gi|56757978|gb|AAW27129.1|SJCHGC06305 protein [Schistosoma japonicum] |
| TS.seq. Contig453 | 1.3098E-16 | gi|156765944|dbj|BAF79609.1|EMY162 protein [Echinococcus multilocularis] |
| TS.seq. Contig454 | 1.02945E-126 | gi|29336772|sp|Q8MM75.1|14332_ECHMURecName: Full=14-3-3 protein homolog 2 >gi|22256010|gb|AAM94864.1| putative growth regulator 14-3-3 [Echinococcus multilocularis] >gi|22256012|gb|AAM94865.1| putative growth regulator 14-3-3 [Echinococcus multilocularis] |
| TS.seq. Contig455 | 4.03025E-50 | gi|13359451|dbj|BAB33421.1|putative senescence-associated protein [Pisum sativum] |
| TS.seq. Contig456 | 5.18419E-12 | gi|242009491|ref|XP_002425519.1|conserved hypothetical protein [Pediculus humanus corporis] >gi|212509374|gb|EEB12781.1| conserved hypothetical protein [Pediculus humanus corporis] |
| TS.seq. Contig457 | 1.62115E-27 | gi|110456474|gb|ABG74714.1|Ac1147-like protein [Diaphorina citri] |
| TS.seq. Contig458 | 1.81308E-4 | gi|149019918|gb|EDL78066.1|rCG36507, isoform CRA_b [Rattus norvegicus] |
| TS.seq. Contig459 | 1.33689E-29 | gi|256084169|ref|XP_002578304.1|formin binding protein and related proteins [Schistosoma mansoni] >gi|238663672|emb|CAZ34542.1| formin binding protein and related proteins [Schistosoma mansoni] |
| TS.seq. Contig461 | 1.6705E-19 | gi|156358531|ref|XP_001624571.1|predicted protein [Nematostella vectensis] >gi|156211360|gb|EDO32471.1| predicted protein [Nematostella vectensis] |
| TS.seq. Contig463 | 8.20205E-105 | gi|124783036|gb|ABN14898.1|ribosomal protein S8 [Taenia asiatica] |
| TS.seq. Contig464 | 4.37169E-18 | gi|186926582|gb|ACC95495.1|EG95 [Echinococcus multilocularis] >gi|186926584|gb|ACC95496.1| EG95 [Echinococcus multilocularis] |
| TS.seq. Contig467 | 3.58783E-169 | gi|21665905|emb|CAD36617.1|small heat-shock protein [Taenia solium] |
| TS.seq. Contig468 | 2.90432E-5 | gi|129166|sp|P22080|ONCA_TAETAOncosphere antigen A >gi|161654|gb|AAA63536.1| oncosphere antigen |
| TS.seq. Contig469 | 4.08139E-68 | gi|256082102|ref|XP_002577301.1|hypothetical protein [Schistosoma mansoni] >gi|238662608|emb|CAZ33538.1| expressed protein [Schistosoma mansoni] |
| TS.seq. Contig470 | 3.02853E-107 | gi|124783372|gb|ABN14924.1|alpha-actinin isoform B [Taenia asiatica] |
| TS.seq. Contig471 | 8.13027E-22 | gi|59709854|gb|AAW88557.1|oncosphere protein Tso31b [Taenia solium] >gi|111146851|gb|ABH07374.1| oncosphere protein Tso31b [Taenia solium] |
| TS.seq. Contig472 | 3.19909E-40 | gi|226484500|emb|CAX74159.1|Cell cycle control protein 50A (Transmembrane protein 30A) [Schistosoma japonicum] |
| TS.seq. Contig475 | 1.31991E-11 | gi|242009491|ref|XP_002425519.1|conserved hypothetical protein [Pediculus humanus corporis] >gi|212509374|gb|EEB12781.1| conserved hypothetical protein [Pediculus humanus corporis] |
| TS.seq. Contig476 | 3.60398E-18 | gi|189235991|ref|XP_972419.2|PREDICTED: similar to DNA-J, putative [Tribolium castaneum] |
| TS.seq. Contig477 | 1.35557E-16 | gi|188485737|gb|ACD50951.1|Nc-DigChim-324430 [synthetic construct] |
| TS.seq. Contig478 | 1.12293E-11 | gi|59709858|gb|AAW88559.1|oncosphere protein Tso31d [Taenia solium] |
| TS.seq. Contig479 | 0.0 | gi|149364041|gb|ABR24229.1|gyceraldehyde-3-phosphate dehydrogenase [Taenia solium] |
| TS.seq. Contig481 | 9.7042E-52 | gi|37778984|gb|AAP20152.1|alpha-actin protein [Pagrus major] |
| TS.seq. Contig482 | 2.10979E-68 | gi|221113094|ref|XP_002155286.1|PREDICTED: similar to Annexin-B12 (Annexin-12) (Annexin XII) [Hydra magnipapillata] >gi|113972|sp|P26256.1|ANX12_HYDAT RecName: Full=Annexin-B12; AltName: Full=Annexin-12; AltName: Full=Annexin XII >gi|159256|gb|AAA29206.1| annexin XII |
| TS.seq. Contig483 | 3.03112E-44 | gi|116687782|gb|AAT74668.2|cysteine-rich secreted protein 2 precursor [Mesocestoides vogae] |
| TS.seq. Contig488 | 1.23986E-68 | gi|13539680|gb|AAK29203.1|AF225905_1ribosomal protein S15a [Taenia solium] >gi|36288852|gb|AAP35026.1| ribosomal S15a protein [Taenia solium] >gi|36288857|gb|AAP35027.1| ribosomal S15a protein [Taenia saginata] |
| TS.seq. Contig489 | 6,00E-164 | dbj|AB086256.1| Taenia solium mitochondrial DNA |
| TS.seq. Contig491 | 3.14128E-66 | gi|158934366|emb|CAO82075.1|HP6 protein [Taenia solium] |
| TS.seq. Contig492 | 7.00788E-5 | gi|2114399|gb|AAC47532.1|45W antigen ToW5/7 [Taenia ovis] |
| TS.seq. Contig493 | 3.52973E-68 | gi|56753429|gb|AAW24918.1|SJCHGC05540 protein [Schistosoma japonicum] >gi|257205794|emb|CAX82548.1| hypotheticial protein [Schistosoma japonicum] >gi|257206024|emb|CAX82663.1| hypotheticial protein [Schistosoma japonicum] >gi|257206664|emb|CAX82960.1| hypotheticial protein [Schistosoma japonicum] |
| TS.seq. Contig494 | 1.69254E-14 | gi|37786712|gb|AAP47268.1| Taenia solium  membrane protein t24 |
| TS.seq. Contig495 | 2.41089E-14 | gi|256050212|ref|XP_002569521.1|hypothetical protein [Schistosoma mansoni] >gi|227282394|emb|CAY18490.1| hypothetical protein [Schistosoma mansoni] |
| TS.seq. Contig496 | 6,00E-164 | dbj|AB086256.1| Taenia solium mitochondrial DNA |
| TS.seq. Contig497 | 3,00E-15 | gb|AAH30393.1| ATPase, H+ transporting, lysosomal V0 subunit B [Mus musculus] |
| TS.seq. Contig498 | 0.0 | dbj|BAD88768.1| tubulin [Crassostrea gigas] |
| TS.seq. Contig499 | 2.35266E-132 | gi|207298859|gb|ACI23578.1|beta-actin [Acipenser transmontanus] |
| TS.seq. Contig500 | 1.43417E-48 | gi|117956206|gb|ABK58679.1|PHGPx isoform 1 [Clonorchis sinensis] |
| TSBM.R95.esd | 7.6811E-18 | gi|256080574|ref|XP_002576555.1|map-kinase activating death domain protein [Schistosoma mansoni] >gi|238661826|emb|CAZ32792.1| map-kinase activating death domain protein, putative [Schistosoma mansoni] |
| TSBG.R80.esd | 8.60205E-15 | gi|256070160|ref|XP_002571412.1|transcription initiation factor iif (tfiif) beta subunit-related [Schistosoma mansoni] >gi|238652642|emb|CAZ39097.1| transcription initiation factor iif (tfiif), beta subunit-related [Schistosoma mansoni] |
| TSBP.R71.esd | 2.13249E-5 | gi|126273099|ref|XP_001368417.1|PREDICTED: hypothetical protein [Monodelphis domestica] |
| TSAG.R77.esd | 5.54565E-14 | gi|256082292|ref|XP_002577392.1|hypothetical protein [Schistosoma mansoni] >gi|238662707|emb|CAZ33630.1| hypothetical protein [Schistosoma mansoni] |
| TSAI.R31.esd | 5.58238E-6 | gi|256078010|ref|XP_002575291.1|cut-like 1 (ctl1) [Schistosoma mansoni] >gi|238660526|emb|CAZ31524.1| cut-like 1 (ctl1) [Schistosoma mansoni] |
| TSCE.R60.esd | 1.80306E-13 | gi|226479866|emb|CAX73229.1|Putative metal tolerance protein C3 [Schistosoma japonicum] |
| TSAL.R77.esd | 1.84082E-25 | gi|226478076|emb|CAX72731.1|ubiquitin-conjugating enzyme E2G 2 [Schistosoma japonicum] >gi|226478556|emb|CAX72773.1| ubiquitin-conjugating enzyme E2G 2 [Schistosoma japonicum] >gi|257205698|emb|CAX82500.1| ubiquitin-conjugating enzyme E2G 2 [Schistosoma japonicum] >gi|257206552|emb|CAX82904.1| ubiquitin-conjugating enzyme E2G 2 [Schistosoma japonicum] |
| TSCD.R85.esd | 2.59617E-11 | gi|256084500|ref|XP_002578466.1|phosphorylase B kinase beta kpbb [Schistosoma mansoni] >gi|238663841|emb|CAZ34704.1| phosphorylase B kinase beta, kpbb, putative [Schistosoma mansoni] |
| TSAR.R46.esd | 1.28597E-34 | gi|210060915|pdb|3C8F|AChain A, 4fe-4s-Pyruvate Formate-Lyase Activating Enzyme With Partially Disordered Adomet >gi|210060916|pdb|3CB8|A Chain A, 4fe-4s-Pyruvate Formate-Lyase Activating Enzyme In Complex With Adomet And A Peptide Substrate |
| TSAR.R40.esd | 3.2751E-30 | gi|32967432|gb|AAP49564.1|elongation factor 2 [Proterospongia sp. ATCC 50818] |
| TSAK.R54.esd | 1.76853E-19 | gi|256083996|ref|XP_002578220.1|mitochondrial 39S ribosomal protein L45 [Schistosoma mansoni] >gi|238663583|emb|CAZ34458.1| putative mitochondrial 39S ribosomal protein L45 [Schistosoma mansoni] |
| TSBH.R45.esd | 1.69306E-10 | gi|118089776|ref|XP_420383.2|PREDICTED: hypothetical protein [Gallus gallus] |
| TSAZ.R36.esd | 8.65443E-7 | gi|256088727|ref|XP_002580477.1|signal recognition particle receptor alpha subunit (sr-alpha) [Schistosoma mansoni] >gi|238666057|emb|CAZ36716.1| signal recognition particle receptor alpha subunit (sr-alpha), putative [Schistosoma mansoni] |
| TSAP.R85.esd | 3.3254E-17 | gi|256072183|ref|XP_002572416.1|hypothetical protein [Schistosoma mansoni] >gi|238657574|emb|CAZ28647.1| expressed protein [Schistosoma mansoni] |
| TSAL.R27.esd | 6.25583E-5 | gi|256077573|ref|XP_002575077.1|fimbrin [Schistosoma mansoni] >gi|238660303|emb|CAZ31310.1| fimbrin, putative [Schistosoma mansoni] |
| TSBU.R26.esd | 1.83973E-5 | gi|256075911|ref|XP_002574259.1|proline-serine-threonine phosphatase interacting protein [Schistosoma mansoni] >gi|238659459|emb|CAZ30492.1| proline-serine-threonine phosphatase interacting protein, putative [Schistosoma mansoni] |
| TSBN.R56.esd | 1.23153E-36 | gi|256071232|ref|XP_002571945.1|choline-phosphate cytidylyltransferase [Schistosoma mansoni] >gi|238657094|emb|CAZ28175.1| choline-phosphate cytidylyltransferase [Schistosoma mansoni] |
| TSBU.R94.esd | 8.06353E-5 | gi|256072952|ref|XP_002572797.1|inositol polyphosphate multikinase [Schistosoma mansoni] >gi|238657962|emb|CAZ29029.1| inositol polyphosphate multikinase, putative [Schistosoma mansoni] |
| TSAB.R23.esd | 2.03576E-16 | gi|256080836|ref|XP_002576682.1|beta-hexosaminidase B [Schistosoma mansoni] >gi|238661961|emb|CAZ32919.1| beta-hexosaminidase B, putative [Schistosoma mansoni] |
| TSAA.R4.esd | 2.69565E-61 | gi|226466806|emb|CAX69538.1|Casein kinase I isoform alpha [Schistosoma japonicum] |
| TSCC.R39.esd | 1.27465E-26 | gi|45238335|emb|CAD12372.2|serine protease inhibitor [Echinococcus multilocularis] |
| TSBC.R73.esd | 1.27567E-11 | gi|12052942|emb|CAB66646.1|hypothetical protein [Homo sapiens] >gi|49065468|emb|CAG38552.1| VMP1 [Homo sapiens] >gi|117645922|emb|CAL38428.1| hypothetical protein [synthetic construct] >gi|117646072|emb|CAL38503.1| hypothetical protein [synthetic construct] >gi|261860936|dbj|BAI46990.1| transmembrane protein 49 [synthetic construct] |
| TSAT.R52.esd | 8.63871E-23 | gi|76154237|gb|AAX25729.2|SJCHGC07080 protein [Schistosoma japonicum] |
| TSBH.R8.esd | 5.06674E-7 | gi|149639255|ref|XP_001507322.1|PREDICTED: similar to membrane associated guanylate kinase, WW and PDZ domain containing 2 [Ornithorhynchus anatinus] |
| TSAI.R12.esd | 2.83036E-24 | gi|115743000|ref|XP_001201457.1|PREDICTED: similar to Trafficking protein particle complex 6b [Strongylocentrotus purpuratus] >gi|115912769|ref|XP_798724.2| PREDICTED: similar to Trafficking protein particle complex 6b [Strongylocentrotus purpuratus] |
| TSBA.R62.esd | 4.96353E-39 | gi|226474792|emb|CAX77606.1|large subunit ribosomal protein L7e [Schistosoma japonicum] |
| TSBG.R5.esd | 3.84119E-7 | gi|229366834|gb|ACQ58397.1|Nascent polypeptide-associated complex subunit alpha [Anoplopoma fimbria] |
| TSAJ.R50.esd | 1.31538E-11 | gi|148229178|ref|NP_001086831.1|solute carrier family 30 (zinc transporter), member 6 [Xenopus laevis] >gi|82182033|sp|Q6AZN8.1|ZNT6A_XENLA RecName: Full=Zinc transporter 6-A; Short=ZnT-6-A; AltName: Full=Solute carrier family 30 member 6-A >gi|50603718|gb|AAH77520.1| Slc30a6-prov protein [Xenopus laevis] |
| TSBO.R74.esd | 3.6814E-18 | gi|256084632|ref|XP_002578531.1|prefoldin subunit [Schistosoma mansoni] >gi|238663908|emb|CAZ34769.1| prefoldin subunit, putative [Schistosoma mansoni] |
| TSBE.R66.esd | 2.75218E-29 | gi|189503090|gb|ACE06926.1|unknown [Schistosoma japonicum] |
| TSBC.R60.esd | 1.77368E-16 | gi|190360172|sp|Q6GPR5.2|CE044_XENLARecName: Full=UPF0533 protein C5orf44 homolog |
| TSCB.R33.esd | 1.34044E-60 | gi|124784033|gb|ABN14961.1|calumenin [Taenia asiatica] |
| TSAE.R40.esd | 5.68188E-11 | gi|195165013|ref|XP_002023340.1|GL20249 [Drosophila persimilis] >gi|194105445|gb|EDW27488.1| GL20249 [Drosophila persimilis] |
| TSBT.R71.esd | 1.66733E-38 | gi|195164915|ref|XP_002023291.1|GL20269 [Drosophila persimilis] >gi|194105396|gb|EDW27439.1| GL20269 [Drosophila persimilis] |
| TSCC.R2.esd | 5.99573E-8 | gi|226479212|emb|CAX73101.1|Nuclear cap-binding protein subunit 1 [Schistosoma japonicum] |
| TSCG.R75.esd | 5.47399E-38 | gi|256022811|ref|ZP_05436676.1|putative lipoprotein [Escherichia sp. 4_1_40B] |
| TSAG.R4.esd | 1.28536E-10 | gi|21912592|emb|CAD21551.1|hypothetical protein [Taenia solium] |
| TSBK.R32.esd | 7.76837E-27 | gi|257215840|emb|CAX83072.1|DEAD (Asp-Glu-Ala-Asp) box polypeptide 56 [Schistosoma japonicum] |
| TSBV.R95.esd | 5.4898E-8 | gi|256077452|ref|XP_002575018.1|hypothetical protein [Schistosoma mansoni] >gi|238660241|emb|CAZ31251.1| expressed protein [Schistosoma mansoni] |
| TSBN.R10.esd | 5.41453E-9 | gi|256077651|ref|XP_002575115.1|rap1 and [Schistosoma mansoni] >gi|238660343|emb|CAZ31348.1| rap1 and, putative [Schistosoma mansoni] |
| TSAI.R33.esd | 5.06785E-7 | gi|238014838|gb|ACR38454.1|unknown [Zea mays] |
| TSAG.R38.esd | 2.38815E-25 | gi|226478056|emb|CAX72721.1|CybS domain-containing protein [Schistosoma japonicum] >gi|257206462|emb|CAX82859.1| CybS domain-containing protein [Schistosoma japonicum] |
| TSBI.R10.esd | 3.18751E-30 | gi|124783031|gb|ABN14897.1|mitochondrial ribosomal protein L15 [Taenia asiatica] |
| TSBZ.R23.esd | 7.19688E-22 | gi|171473946|gb|ACB47092.1|SJCHGC09585 protein [Schistosoma japonicum] |
| TSAB.R2.esd | 6.35493E-29 | gi|256085601|ref|XP_002579005.1|RNA binding motif protein 25 [Schistosoma mansoni] >gi|238664403|emb|CAZ35243.1| RNA binding motif protein 25, putative [Schistosoma mansoni] |
| TSBD.R25.esd | 1.27502E-31 | gi|194431852|ref|ZP_03064142.1|glutamate:gamma aminobutyrate antiporter [Shigella dysenteriae 1012] >gi|194419760|gb|EDX35839.1| glutamate:gamma aminobutyrate antiporter [Shigella dysenteriae 1012] |
| TSCG.R69.esd | 1.62378E-5 | gi|256088411|ref|XP_002580331.1|hypothetical protein [Schistosoma mansoni] >gi|238665887|emb|CAZ36570.1| expressed protein [Schistosoma mansoni] |
| TSAO.R60.esd | 2.30466E-42 | gi|226468272|emb|CAX69813.1|mRNA export factor [Schistosoma japonicum] |
| TSCC.R93.esd | 1.71898E-31 | gi|37786712|gb|AAP47268.1|T24 [Taenia solium] >gi|112785172|gb|ABI20734.1| membrane protein T24 [Taenia solium] |
| TSBC.R71.esd | 3.56483E-11 | gi|225710426|gb|ACO11059.1|Cdc42 homolog precursor [Caligus rogercresseyi] |
| TSAY.R35.esd | 2.69254E-61 | gi|29336561|sp|Q9GP32.1|ALF_ECHMURecName: Full=Fructose-bisphosphate aldolase >gi|11602727|emb|CAC18550.1| putative fructose-bisphosphate-aldolase [Echinococcus multilocularis] |
| TSBT.R54.esd | 2.8965E-10 | gi|6560653|gb|AAF16704.1|AF117582_1calcyphosine-like protein [Manduca sexta] |
| TSCF.R66.esd | 6.89746E-33 | gi|56435063|gb|AAV91323.1|succinate dehydrogenase iron-sulfur protein [Schistosoma japonicum] >gi|56757549|gb|AAW26938.1| SJCHGC06829 protein [Schistosoma japonicum] >gi|226470530|emb|CAX70545.1| succinate dehydrogenase complex, subunit B, iron sulfur [Schistosoma japonicum] >gi|226470532|emb|CAX70546.1| succinate dehydrogenase complex, subunit B, iron sulfur [Schistosoma japonicum] >gi|226470534|emb|CAX70547.1| succinate dehydrogenase complex, subunit B, iron sulfur [Schistosoma japonicum] >gi|226470536|emb|CAX70548.1| succinate dehydrogenase complex, subunit B, iron sulfur [Schistosoma japonicum] >gi|226487080|emb|CAX75405.1| succinate dehydrogenase complex, subunit B, iron sulfur [Schistosoma japonicum] |
| TSBK.R38.esd | 6.4728E-15 | gi|256071116|ref|XP_002571887.1|coatomer beta subunit [Schistosoma mansoni] >gi|238657036|emb|CAZ28117.1| coatomer beta subunit, putative [Schistosoma mansoni] |
| TSBM.R20.esd | 2.89681E-39 | gi|193209454|ref|NP_001123100.1|Protein Disulfide Isomerase family member (pdi-2) [Caenorhabditis elegans] >gi|126541576|gb|ABO16445.1| Protein disulfide isomerase protein 2, isoform c [Caenorhabditis elegans] |
| TSAR.R49.esd | 3.76303E-30 | gi|256072054|ref|XP_002572352.1|RNA binding protein [Schistosoma mansoni] >gi|238657509|emb|CAZ28583.1| RNA binding protein, putative [Schistosoma mansoni] |
| TSAT.R95.esd | 4.2694E-22 | gi|156370189|ref|XP_001628354.1|predicted protein [Nematostella vectensis] >gi|156215328|gb|EDO36291.1| predicted protein [Nematostella vectensis] |
| TSBZ.R77.esd | 1.2053E-16 | gi|147901115|ref|NP_001088792.1|hypothetical protein LOC496057 [Xenopus laevis] >gi|56269212|gb|AAH87463.1| LOC496057 protein [Xenopus laevis] |
| TSCG.R24.esd | 5.40248E-8 | gi|256073976|ref|XP_002573303.1|hypothetical protein [Schistosoma mansoni] >gi|238658480|emb|CAZ29535.1| expressed protein [Schistosoma mansoni] |
| TSBX.R52.esd | 6.06245E-29 | gi|37992036|emb|CAD68073.1|SmadA protein [Echinococcus multilocularis] |
| TSBB.R38.esd | 1.45964E-6 | gi|256052582|ref|XP_002569842.1|hypothetical protein [Schistosoma mansoni] >gi|227284572|emb|CAY17267.1| expressed protein [Schistosoma mansoni] |
| TSAD.R8.esd | 1.44884E-22 | gi|226479206|emb|CAX73098.1|photorepair [Schistosoma japonicum] |
| TSBW.R58.esd | 1.16743E-11 | gi|76157604|gb|AAX28479.2|SJCHGC09047 protein [Schistosoma japonicum] |
| TSAT.R81.esd | 5.19767E-45 | gi|226478096|emb|CAX72741.1|RAB family [Schistosoma japonicum] >gi|226478548|emb|CAX72769.1| RAB family [Schistosoma japonicum] >gi|257206356|emb|CAX82829.1| RAB family [Schistosoma japonicum] >gi|257206638|emb|CAX82947.1| RAB family [Schistosoma japonicum] |
| TSCB.R94.esd | 6.35019E-26 | gi|256074388|ref|XP_002573507.1|ccr4-not transcription complex [Schistosoma mansoni] >gi|238658688|emb|CAZ29739.1| ccr4-not transcription complex, putative [Schistosoma mansoni] |
| TSAF.R44.esd | 4.7722E-64 | gi|256073421|ref|XP_002573029.1|DNA-directed rna polymerase II subunit [Schistosoma mansoni] >gi|238658199|emb|CAZ29261.1| DNA-directed rna polymerase II subunit, putative [Schistosoma mansoni] |
| TSBU.R46.esd | 2.8826E-53 | gi|226469290|emb|CAX70124.1|ring finger protein 2 [Schistosoma japonicum] >gi|226486844|emb|CAX74499.1| ring finger protein 2 [Schistosoma japonicum] |
| TSAV.R4.esd | 7.75054E-8 | gi|256077524|ref|XP_002575053.1|sec61 gamma subunit [Schistosoma mansoni] >gi|238660278|emb|CAZ31286.1| sec61 gamma subunit, putative [Schistosoma mansoni] |
| TSBJ.R42.esd | 3.9869E-4 | gi|66825911|ref|XP_646310.1|major vault protein [Dictyostelium discoideum AX4] >gi|462665|sp|P34118.1|MVPA_DICDI RecName: Full=Major vault protein alpha; Short=MVP-alpha >gi|167960|gb|AAA03153.1| major vault protein-alpha >gi|60473991|gb|EAL71928.1| major vault protein [Dictyostelium discoideum AX4] |
| TSBY.R67.esd | 7.21428E-20 | gi|256086993|ref|XP_002579665.1|serine/threonine protein kinase [Schistosoma mansoni] >gi|238665135|emb|CAZ35904.1| serine/threonine kinase [Schistosoma mansoni] |
| TSBO.R5.esd | 1.77928E-4 | gi|147904463|ref|NP_001083789.1|P7D11 protein [Xenopus laevis] >gi|17976979|dbj|BAB79596.1| P7D11 [Xenopus laevis] >gi|213623146|gb|AAI69351.1| P7D11 protein [Xenopus laevis] >gi|213624890|gb|AAI69355.1| P7D11 protein [Xenopus laevis] |
| TSAW.R3.esd | 3.54195E-5 | gi|189239065|ref|XP_971190.2|PREDICTED: similar to phosphatidate phosphatase [Tribolium castaneum] |
| TSAD.R61.esd | 1.83113E-25 | gi|256083058|ref|XP_002577767.1|eh domain containing/past-1-related [Schistosoma mansoni] >gi|238663098|emb|CAZ34005.1| eh domain containing/past-1-related [Schistosoma mansoni] |
| TSAW.R85.esd | 1.27661E-18 | gi|256085877|ref|XP_002579137.1|tfiia large subunit [Schistosoma mansoni] >gi|238664547|emb|CAZ35376.1| tfiia large subunit, putative [Schistosoma mansoni] |
| TSAG.R62.esd | 1.97959E-29 | gi|119613210|gb|EAW92804.1|hCG39634, isoform CRA_a [Homo sapiens] >gi|119613211|gb|EAW92805.1| hCG39634, isoform CRA_a [Homo sapiens] >gi|119613212|gb|EAW92806.1| hCG39634, isoform CRA_a [Homo sapiens] |
| TSAG.R41.esd | 9.601E-19 | gi|256087108|ref|XP_002579719.1|hypothetical protein [Schistosoma mansoni] >gi|238665196|emb|CAZ35958.1| expressed protein [Schistosoma mansoni] |
| TSAD.R48.esd | 3.11894E-25 | gi|256071317|ref|XP_002571987.1|histone deacetylase [Schistosoma mansoni] >gi|238657137|emb|CAZ28217.1| term=histone deacetylase 1;db_xref=PMID:18977200;with=UniProt:A5H658;evidence=tracea ble author statement [Schistosoma mansoni] |
| TSAL.R59.esd | 1.74628E-15 | gi|256081147|ref|XP_002576834.1|voltage-gated potassium channel [Schistosoma mansoni] >gi|238662120|emb|CAZ33071.1| voltage-gated potassium channel, putative [Schistosoma mansoni] |
| TSAQ.R57.esd | 6.60126E-76 | gi|223640019|emb|CAX16804.1|apolipoprotein AI binding protein [Echinococcus multilocularis] |
| TSBR.R84.esd | 1.91305E-39 | gi|256072702|ref|XP_002572673.1|ribonuclease pH related [Schistosoma mansoni] >gi|238657836|emb|CAZ28905.1| ribonuclease pH related [Schistosoma mansoni] |
| TSAG.R44.esd | 6.71466E-30 | gi|256074453|ref|XP_002573539.1|DNAj homolog subfamily B member 4 [Schistosoma mansoni] >gi|238658721|emb|CAZ29771.1| DNAj homolog subfamily B member 4,, putative [Schistosoma mansoni] |
| TSAS.R79.esd | 1.80725E-5 | gi|256082104|ref|XP_002577302.1|clathrin heavy chain [Schistosoma mansoni] >gi|238662609|emb|CAZ33539.1| clathrin heavy chain, putative [Schistosoma mansoni] |
| TSAC.R9.esd | 2.07926E-4 | gi|225703014|dbj|BAH30229.1|G1Y162 protein [Echinococcus granulosus] |
| TSAP.R92.esd | 1.29696E-79 | gi|29337143|sp|Q9NFZ5.1|TBB3_ECHMURecName: Full=Tubulin beta-3 chain; AltName: Full=Beta-3-tubulin >gi|7838203|emb|CAB91642.1| beta-tubulin, Tub-3 [Echinococcus multilocularis] |
| TSBO.R22.esd | 3.25428E-22 | gi|260460285|ref|ZP_05808537.1|Diverse 7TM receptor transmembrane region [Mesorhizobium opportunistum WSM2075] >gi|259033930|gb|EEW35189.1| Diverse 7TM receptor transmembrane region [Mesorhizobium opportunistum WSM2075] |
| TSAN.R29.esd | 2.7635E-29 | gi|257216011|emb|CAX83155.1|Ribosomal protein L17 [Schistosoma japonicum] >gi|257216013|emb|CAX83156.1| Ribosomal protein L17 [Schistosoma japonicum] >gi|257216015|emb|CAX83157.1| Ribosomal protein L17 [Schistosoma japonicum] >gi|257216017|emb|CAX83158.1| Ribosomal protein L17 [Schistosoma japonicum] |
| TSBN.R17.esd | 3.80564E-7 | gi|226477898|emb|CAX72656.1|Sorting nexin-6 [Schistosoma japonicum] |
| TSAP.R96.esd | 1.12071E-25 | gi|256086813|ref|XP_002579581.1|ubiquitin ligase E3a [Schistosoma mansoni] >gi|238665039|emb|CAZ35820.1| ubiquitin ligase E3a, putative [Schistosoma mansoni] |
| TSCE.R57.esd | 2.23098E-24 | gi|256084709|ref|XP_002578569.1|hypothetical protein [Schistosoma mansoni] >gi|238663947|emb|CAZ34807.1| expressed protein [Schistosoma mansoni] |
| TSAC.R69.esd | 1.60435E-5 | gi|227334735|ref|ZP_03838391.1|hypothetical protein CIT292_04493 [Citrobacter youngae ATCC 29220] >gi|228553927|gb|EEK18592.1| hypothetical protein CIT292_04493 [Citrobacter youngae ATCC 29220] |
| TSAR.R23.esd | 1.73121E-31 | gi|256088600|ref|XP_002580417.1|eukaryotic translation initiation factor 2 gamma subunit (eif-2-gamma) [Schistosoma mansoni] >gi|238665990|emb|CAZ36656.1| eukaryotic translation initiation factor 2 gamma subunit (eif-2-gamma), putative [Schistosoma mansoni] |
| TSAI.R30.esd | 3.06942E-4 | gi|221107829|ref|XP_002170398.1|PREDICTED: hypothetical protein [Hydra magnipapillata] |
| TSAV.R54.esd | 4.92481E-18 | gi|256073851|ref|XP_002573241.1|hypothetical protein [Schistosoma mansoni] >gi|238658417|emb|CAZ29473.1| expressed protein [Schistosoma mansoni] |
| TSBF.R33.esd | 6.54211E-7 | gi|123492785|ref|XP_001326143.1|Ras family protein [Trichomonas vaginalis G3] >gi|121909053|gb|EAY13920.1| Ras family protein [Trichomonas vaginalis G3] |
| TSBQ.R66.esd | 3.2569E-14 | gi|256073336|ref|XP_002572987.1|hypothetical protein [Schistosoma mansoni] >gi|238658156|emb|CAZ29219.1| conserved hypothetical protein [Schistosoma mansoni] |
| TSAQ.R13.esd | 3.10844E-9 | gi|226469272|emb|CAX70115.1|hypothetical protein [Schistosoma japonicum] |
| TSAX.R58.esd | 1.81123E-17 | gi|257215746|emb|CAX83025.1|hypotheticial protein [Schistosoma japonicum] >gi|257215748|emb|CAX83026.1| hypotheticial protein [Schistosoma japonicum] |
| TSCC.R81.esd | 1.01905E-78 | gi|21449864|ref|NP_659225.1|cytochrome c oxidase subunit III [Taenia solium] >gi|21388753|dbj|BAC00834.1| cytochrome c oxidase subunit 3 [Taenia solium] |
| TSBW.R92.esd | 1.06608E-4 | gi|257214420|emb|CAX82992.1|Guanine nucleotide-binding protein subunit beta 2-like 1 [Schistosoma japonicum] |
| TSAU.R19.esd | 8.83068E-4 | gi|56755555|gb|AAW25956.1|SJCHGC06538 protein [Schistosoma japonicum] |
| TSAA.R20.esd | 1.0743E-11 | gi|256076724|ref|XP_002574660.1|hypothetical protein [Schistosoma mansoni] >gi|238659871|emb|CAZ30893.1| expressed protein [Schistosoma mansoni] |
| TSAY.R49.esd | 4.46383E-11 | gi|256080616|ref|XP_002576575.1|hypothetical protein [Schistosoma mansoni] >gi|238661848|emb|CAZ32812.1| expressed protein [Schistosoma mansoni] |
| TSAB.R74.esd | 3.91163E-20 | gi|256075409|ref|XP_002574012.1|proteasome subunit beta 1 (T01 family) [Schistosoma mansoni] >gi|238659204|emb|CAZ30245.1| proteasome subunit beta 1 (T01 family) [Schistosoma mansoni] |
| TSCC.R17.esd | 1.63827E-5 | gi|256081827|ref|XP_002577169.1|hypothetical protein [Schistosoma mansoni] >gi|238662465|emb|CAZ33406.1| expressed protein [Schistosoma mansoni] |
| TSBH.R82.esd | 1.68435E-10 | gi|76152813|gb|AAX24491.2|SJCHGC04528 protein [Schistosoma japonicum] |
| TSAG.R25.esd | 1.68279E-10 | gi|226478904|emb|CAX72947.1|Non-receptor tyrosine kinase spore lysis A [Schistosoma japonicum] |
| TSCG.R92.esd | 4.63001E-5 | gi|91083101|ref|XP_969476.1|PREDICTED: similar to MGC88883 protein [Tribolium castaneum] |
| TSAD.R95.esd | 1.43801E-9 | gi|11602713|emb|CAC18543.1|translation initiation factor 4A-like protein [Echinococcus multilocularis] |
| TSAE.R73.esd | 1.02941E-20 | gi|149028715|gb|EDL84056.1|similar to solute carrier family 37 (glycerol-3-phosphate transporter), member 2 (predicted) [Rattus norvegicus] |
| TSBR.R47.esd | 1.48882E-13 | gi|256076838|ref|XP_002574716.1|dolichyl-phosphate beta-glucosyltransferase ((EC 2.4.1.117) dolp-glucosyltransferase) [Schistosoma mansoni] >gi|238659929|emb|CAZ30949.1| putative dolichyl-phosphate beta-glucosyltransferase ((EC 2.4.1.117) dolp-glucosyltransferase) [Schistosoma mansoni] |
| TSBQ.R92.esd | 4.52005E-32 | gi|226470478|emb|CAX70519.1|60S ribosome subunit biogenesis protein NIP7 [Schistosoma japonicum] >gi|226487008|emb|CAX75369.1| 60S ribosome subunit biogenesis protein NIP7 [Schistosoma japonicum] >gi|226487010|emb|CAX75370.1| 60S ribosome subunit biogenesis protein NIP7 [Schistosoma japonicum] >gi|226487012|emb|CAX75371.1| 60S ribosome subunit biogenesis protein NIP7 [Schistosoma japonicum] >gi|226487014|emb|CAX75372.1| 60S ribosome subunit biogenesis protein NIP7 [Schistosoma japonicum] |
| TSAD.R46.esd | 6.15369E-78 | gi|170680896|ref|YP_001742919.1|sulfatase family protein [Escherichia coli SMS-3-5] >gi|170518614|gb|ACB16792.1| sulfatase family protein [Escherichia coli SMS-3-5] |
| TSAB.R70.esd | 5.14285E-12 | gi|226480888|emb|CAX73541.1|WW domain-binding protein 11 [Schistosoma japonicum] |
| TSBV.R85.esd | 2.67408E-4 | gi|256077798|ref|XP_002575187.1|glutathione synthetase [Schistosoma mansoni] >gi|238660418|emb|CAZ31420.1| glutathione synthetase [Schistosoma mansoni] |
| TSAO.R70.esd | 5.03435E-40 | gi|256078081|ref|XP_002575326.1|uroporphyrinogen decarboxylase [Schistosoma mansoni] >gi|238660562|emb|CAZ31559.1| uroporphyrinogen decarboxylase [Schistosoma mansoni] |
| TSBZ.R14.esd | 8.08469E-13 | gi|149251798|ref|XP_001477845.1|PREDICTED: similar to ribosomal protein S28 [Mus musculus] >gi|148680335|gb|EDL12282.1| mCG1045593 [Mus musculus] |
| TSAZ.R12.esd | 6.79446E-76 | gi|256070717|ref|XP_002571689.1|pre-mRNA cleavage factor im 25kD subunit [Schistosoma mansoni] >gi|238656835|emb|CAZ27919.1| pre-mRNA cleavage factor im, 25kD subunit, putative [Schistosoma mansoni] |
| TSAB.R91.esd | 3.60855E-24 | gi|226486774|emb|CAX74464.1|hypotherical protein [Schistosoma japonicum] |
| TSAG.R32.esd | 6.92928E-9 | gi|256071559|ref|XP_002572107.1|gap associated protein-related [Schistosoma mansoni] >gi|238657259|emb|CAZ28337.1| gap associated protein-related [Schistosoma mansoni] |
| TSAP.R80.esd | 6.84939E-49 | gi|195117796|ref|XP_002003433.1|GI22541 [Drosophila mojavensis] >gi|193914008|gb|EDW12875.1| GI22541 [Drosophila mojavensis] |
| TSAV.R40.esd | 6.97642E-17 | gi|76154347|gb|AAX25834.2|SJCHGC05968 protein [Schistosoma japonicum] |
| TSBD.R35.esd | 4.08294E-33 | gi|28190030|gb|AAO32953.1|putative AFG3-like protein 2 [Schistosoma japonicum] |
| TSBS.R82.esd | 2.23001E-31 | gi|76154626|gb|AAX26076.2|SJCHGC06036 protein [Schistosoma japonicum] |
| TSBS.R22.esd | 3.48145E-56 | gi|256086079|ref|XP_002579233.1|ATP-dependent transporter [Schistosoma mansoni] >gi|238664653|emb|CAZ35472.1| ATP-dependent transporter, putative [Schistosoma mansoni] |
| TSBM.R24.esd | 3.4559E-10 | gi|189522228|ref|XP_001333006.2|PREDICTED: similar to citrate transporter, partial [Danio rerio] |
| TSBO.R7.esd | 1.12673E-7 | gi|226481345|emb|CAX73570.1|3' histone mRNA exonuclease 1 [Schistosoma japonicum] |
| TSCC.R34.esd | 3.90119E-7 | gi|21554064|gb|AAM63145.1|unknown [Arabidopsis thaliana] |
| TSCB.R22.esd | 6.29357E-57 | gi|148264|gb|AAA24759.1|trp repressor binding protein |
| TSAX.R24.esd | 7.1605E-14 | gi|260822036|ref|XP_002606409.1|hypothetical protein BRAFLDRAFT_118529 [Branchiostoma floridae] >gi|229291750|gb|EEN62419.1| hypothetical protein BRAFLDRAFT_118529 [Branchiostoma floridae] |
| TSBC.R84.esd | 2.29556E-17 | gi|29841454|gb|AAP06486.1|hypothetical protein, putative Universal stress protein Usp [Schistosoma japonicum] |
| TSBN.R15.esd | 7.3108E-6 | gi|251829646|gb|ACT21201.1|p38-like protein [Echinococcus granulosus] |
| TSAK.R79.esd | 9.41292E-14 | gi|94158217|ref|NP_001035340.1|zinc finger and BTB domain containing 8A [Danio rerio] >gi|92098319|gb|AAI15303.1| Zgc:136874 [Danio rerio] |
| TSBY.R62.esd | 1.24485E-21 | gi|256085911|ref|XP_002579153.1|replication protein A [Schistosoma mansoni] >gi|238664565|emb|CAZ35392.1| replication protein A, putative [Schistosoma mansoni] |
| TSBG.R15.esd | 2.35157E-4 | gi|195566099|ref|XP_002106628.1|GD16987 [Drosophila simulans] >gi|194204010|gb|EDX17586.1| GD16987 [Drosophila simulans] |
| TSAI.R7.esd | 1.86998E-22 | gi|226477898|emb|CAX72656.1|Sorting nexin-6 [Schistosoma japonicum] |
| TSAJ.R28.esd | 4.998E-23 | gi|256070687|ref|XP_002571674.1|nedd-4-like E3 ubiquitin-protein ligase [Schistosoma mansoni] >gi|238656820|emb|CAZ27904.1| nedd-4-like E3 ubiquitin-protein ligase [Schistosoma mansoni] |
| TSBS.R30.esd | 1.03917E-10 | gi|256081799|ref|XP_002577155.1|proteasome regulatory subunit-related [Schistosoma mansoni] >gi|238662451|emb|CAZ33392.1| proteasome regulatory subunit-related [Schistosoma mansoni] |
| TSAQ.R17.esd | 2.05218E-8 | gi|226483575|emb|CAX74088.1|Syntaxin 1A [Schistosoma japonicum] |
| TSBU.R84.esd | 1.59549E-5 | gi|256069458|ref|XP_002571151.1|hypothetical protein [Schistosoma mansoni] >gi|238652200|emb|CAZ38836.1| hypothetical protein [Schistosoma mansoni] |
| TSBV.R3.esd | 2.92197E-23 | gi|76156546|gb|AAX27732.2|SJCHGC05922 protein [Schistosoma japonicum] |
| TSAO.R32.esd | 1.57993E-18 | gi|256086967|ref|XP_002579653.1|myosin heavy chain [Schistosoma mansoni] >gi|238665121|emb|CAZ35892.1| myosin heavy chain, putative [Schistosoma mansoni] |
| TSCG.R72.esd | 1.48178E-53 | gi|256072944|ref|XP_002572793.1|60S ribosomal protein L10 [Schistosoma mansoni] >gi|238657958|emb|CAZ29025.1| 60S ribosomal protein L10, putative [Schistosoma mansoni] |
| TSBL.R14.esd | 6.98061E-25 | gi|226483639|emb|CAX74120.1|hypothetical protein [Schistosoma japonicum] |
| TSAU.R47.esd | 1.38039E-33 | gi|256085919|ref|XP_002579157.1|pcdc2/rp-8 (programmed cell death protein 2) [Schistosoma mansoni] >gi|238664569|emb|CAZ35396.1| pcdc2/rp-8 (programmed cell death protein 2), putative [Schistosoma mansoni] |
| TSAK.R32.esd | 1.28523E-26 | gi|256056843|ref|XP_002570239.1|hypothetical protein [Schistosoma mansoni] >gi|227291871|emb|CAY19101.1| expressed protein [Schistosoma mansoni] |
| TSAV.R59.esd | 3.9105E-7 | gi|156387908|ref|XP_001634444.1|predicted protein [Nematostella vectensis] >gi|156221527|gb|EDO42381.1| predicted protein [Nematostella vectensis] |
| TSCF.R13.esd | 2.41536E-9 | gi|238800095|gb|ACR55893.1|lactate dehydrogenase B [Plectropomus leopardus] |
| TSBQ.R52.esd | 1.46097E-6 | gi|260804695|ref|XP_002597223.1|hypothetical protein BRAFLDRAFT_203346 [Branchiostoma floridae] >gi|229282486|gb|EEN53235.1| hypothetical protein BRAFLDRAFT_203346 [Branchiostoma floridae] |
| TSAF.R50.esd | 1.51921E-35 | gi|224054831|ref|XP_002195767.1|PREDICTED: tubulin, alpha 8 isoform 1 [Taeniopygia guttata] >gi|224054835|ref|XP_002195802.1| PREDICTED: tubulin, alpha 8 isoform 2 [Taeniopygia guttata] |
| TSBH.R95.esd | 1.58387E-29 | gi|256079628|ref|XP_002576088.1|26S proteasome non-ATPase regulatory subunit [Schistosoma mansoni] >gi|238661347|emb|CAZ32323.1| 26S proteasome non-ATPase regulatory subunit, putative [Schistosoma mansoni] |
| TSCG.R34.esd | 7.36098E-30 | gi|256082290|ref|XP_002577391.1|60S ribosomal protein L35a [Schistosoma mansoni] >gi|238662706|emb|CAZ33629.1| 60S ribosomal protein L35a, putative [Schistosoma mansoni] |
| TSAI.R45.esd | 4.99116E-15 | gi|256078457|ref|XP_002575512.1|hypothetical protein [Schistosoma mansoni] >gi|238660752|emb|CAZ31745.1| expressed protein [Schistosoma mansoni] |
| TSBU.R78.esd | 1.29633E-10 | gi|164608836|gb|ABY62749.1|ATP synthase gamma subunit [Artemia franciscana] |
| TSCC.R62.esd | 1.03933E-26 | gi|256085343|ref|XP_002578881.1|hypothetical protein [Schistosoma mansoni] >gi|238664269|emb|CAZ35119.1| expressed protein [Schistosoma mansoni] |
| TSBN.R35.esd | 1.61455E-45 | gi|226466670|emb|CAX69470.1|TATA box binding protein like 2 [Schistosoma japonicum] |
| TSAJ.R41.esd | 1.21584E-8 | gi|256077175|ref|XP_002574883.1|hypothetical protein [Schistosoma mansoni] >gi|238660099|emb|CAZ31116.1| conserved hypothetical protein [Schistosoma mansoni] |
| TSBV.R83.esd | 5.87054E-16 | gi|198435829|ref|XP_002126517.1|PREDICTED: similar to conserved hypothetical protein [Ciona intestinalis] |
| TSAZ.R56.esd | 7.69365E-20 | gi|256084500|ref|XP_002578466.1|phosphorylase B kinase beta kpbb [Schistosoma mansoni] >gi|238663841|emb|CAZ34704.1| phosphorylase B kinase beta, kpbb, putative [Schistosoma mansoni] |
| TSAQ.R62.esd | 4.8771E-21 | gi|256090160|ref|XP_002581080.1|eukaryotic translation initiation factor 2c [Schistosoma mansoni] >gi|238666887|emb|CAZ37319.1| eukaryotic translation initiation factor 2c, putative [Schistosoma mansoni] |
| TSCA.R93.esd | 5.34039E-13 | gi|226484634|emb|CAX74226.1|hypothetical protein [Schistosoma japonicum] |
| TSAX.R62.esd | 4.15357E-30 | gi|46909253|gb|AAT06144.1|ATP synthase beta subunit [Metridium senile] |
| TSCB.R28.esd | 3.1692E-17 | gi|225710418|gb|ACO11055.1|TPR repeat-containing protein YDR161W [Caligus rogercresseyi] |
| TSAN.R66.esd | 7.84916E-85 | gi|88999679|emb|CAJ77649.1|TGF-beta signal transducer SmadC [Echinococcus multilocularis] |
| TSAM.R43.esd | 4.38692E-9 | gi|256070532|ref|XP_002571596.1|hypothetical protein [Schistosoma mansoni] >gi|238656741|emb|CAZ27826.1| expressed protein [Schistosoma mansoni] |
| TSAR.R33.esd | 3.14528E-21 | gi|256072887|ref|XP_002572765.1|hypothetical protein [Schistosoma mansoni] >gi|238657929|emb|CAZ28997.1| expressed protein [Schistosoma mansoni] |
| TSCG.R78.esd | 1.31019E-7 | gi|149508354|ref|XP_001507977.1|PREDICTED: hypothetical protein [Ornithorhynchus anatinus] |
| TSAX.R54.esd | 2.02896E-16 | gi|256082775|ref|XP_002577628.1|transporter spinster-related [Schistosoma mansoni] >gi|238662954|emb|CAZ33866.1| transporter spinster-related [Schistosoma mansoni] |
| TSCE.R62.esd | 1.36235E-20 | gi|256075162|ref|XP_002573889.1|family S9 non-peptidase homologue (S09 family) [Schistosoma mansoni] >gi|238659080|emb|CAZ30122.1| family S9 non-peptidase homologue (S09 family) [Schistosoma mansoni] |
| TSAK.R14.esd | 4.37359E-43 | gi|257215788|emb|CAX83046.1|60S ribosomal protein L8 [Schistosoma japonicum] |
| TSBA.R34.esd | 1.63739E-11 | gi|260825694|ref|XP_002607801.1|hypothetical protein BRAFLDRAFT_199459 [Branchiostoma floridae] >gi|229293150|gb|EEN63811.1| hypothetical protein BRAFLDRAFT_199459 [Branchiostoma floridae] |
| TSBX.R33.esd | 1.09898E-30 | gi|226466746|emb|CAX69508.1|Splicing factor 3A subunit 2 [Schistosoma japonicum] |
| TSAP.R2.esd | 9.39111E-6 | gi|226471566|emb|CAX70864.1|hypothetical protein [Schistosoma japonicum] |
| TSCH.R41.esd | 7.84438E-24 | gi|256070868|ref|XP_002571764.1|pelota [Schistosoma mansoni] >gi|238656911|emb|CAZ27994.1| pelota, putative [Schistosoma mansoni] |
| TSCB.R51.esd | 5.97843E-40 | gi|226470488|emb|CAX70524.1|Serine/threonine-protein phosphatase 2A regulatory subunit B' [Schistosoma japonicum] |
| TSAB.R57.esd | 3.65141E-5 | gi|170037865|ref|XP_001846775.1|transport protein sec23 [Culex quinquefasciatus] >gi|167881217|gb|EDS44600.1| transport protein sec23 [Culex quinquefasciatus] |
| TSAR.R75.esd | 2.00895E-16 | gi|256070733|ref|XP_002571697.1|hypothetical protein [Schistosoma mansoni] >gi|238656843|emb|CAZ27927.1| kif-2, putative [Schistosoma mansoni] |
| TSAC.R53.esd | 8.98122E-4 | gi|56756088|gb|AAW26222.1|SJCHGC08054 protein [Schistosoma japonicum] |
| TSBV.R92.esd | 7.13994E-22 | gi|256076128|ref|XP_002574366.1|deleted in oral cancer 1/cdk2-associated protein-like [Schistosoma mansoni] >gi|238659569|emb|CAZ30599.1| deleted in oral cancer 1/cdk2-associated protein-like [Schistosoma mansoni] |
| TSAG.R17.esd | 9.35362E-6 | gi|90819972|gb|ABD98743.1|thioredoxin-like protein [Graphocephala atropunctata] |
| TSCE.R5.esd | 2.18289E-10 | gi|256073914|ref|XP_002573272.1|hypothetical protein [Schistosoma mansoni] >gi|238658449|emb|CAZ29504.1| expressed protein [Schistosoma mansoni] |
| TSBF.R88.esd | 1.47712E-18 | gi|256074329|ref|XP_002573478.1|zeta-coat protein [Schistosoma mansoni] >gi|238658658|emb|CAZ29710.1| zeta-coat protein, putative [Schistosoma mansoni] |
| TSAR.R80.esd | 6.81037E-12 | gi|170582694|ref|XP_001896244.1|heavy metal-associated domain containing protein [Brugia malayi] >gi|158596589|gb|EDP34911.1| heavy metal-associated domain containing protein [Brugia malayi] |
| TSBL.R26.esd | 1.10346E-14 | gi|256052186|ref|XP_002569657.1|signal transduction protein lnk-related [Schistosoma mansoni] >gi|227284361|emb|CAY16905.1| signal transduction protein lnk-related [Schistosoma mansoni] |
| TSCC.R47.esd | 1.70636E-23 | gi|112984454|ref|NP_001037171.1|protein disulfide isomerase [Bombyx mori] >gi|12025459|gb|AAG45936.1|AF325211_1 protein disulfide isomerase [Bombyx mori] |
| TSAW.R72.esd | 1.65381E-42 | gi|195448332|ref|XP_002071611.1|GK25049 [Drosophila willistoni] >gi|194167696|gb|EDW82597.1| GK25049 [Drosophila willistoni] |
| TSBY.R83.esd | 9.89374E-12 | gi|256078512|ref|XP_002575539.1|peptide (allatostatin/somatostatin)-like receptor [Schistosoma mansoni] >gi|238660780|emb|CAZ31772.1| peptide (allatostatin/somatostatin)-like receptor, putative [Schistosoma mansoni] |
| TSBG.R66.esd | 3.29856E-27 | gi|260798316|ref|XP_002594146.1|hypothetical protein BRAFLDRAFT_120433 [Branchiostoma floridae] >gi|229279379|gb|EEN50157.1| hypothetical protein BRAFLDRAFT_120433 [Branchiostoma floridae] |
| TSBC.R12.esd | 3.36716E-27 | gi|256082199|ref|XP_002577347.1|copine [Schistosoma mansoni] >gi|238662659|emb|CAZ33585.1| copine, putative [Schistosoma mansoni] |
| TSBQ.R61.esd | 1.90427E-6 | gi|1053159|gb|AAB05911.1|TGTP1 [Taenia solium] |
| TSBP.R20.esd | 2.7931E-34 | gi|156553785|ref|XP_001602017.1|PREDICTED: similar to ethanolamine-phosphate cytidylyltransferase [Nasonia vitripennis] |
| TSBV.R51.esd | 2.50932E-14 | gi|74007526|ref|XP_856214.1|PREDICTED: similar to Yip1 domain family, member 6 isoform 3 [Canis familiaris] |
| TSBT.R18.esd | 2.35789E-12 | gi|158290265|ref|XP_311859.4|AGAP003017-PA [Anopheles gambiae str. PEST] >gi|157017811|gb|EAA07900.5| AGAP003017-PA [Anopheles gambiae str. PEST] |
| TSBN.R58.esd | 2.04153E-8 | gi|126308283|ref|XP_001372116.1|PREDICTED: hypothetical protein [Monodelphis domestica] |
| TSBK.R26.esd | 1.35485E-28 | gi|126305312|ref|XP_001379233.1|PREDICTED: similar to ribose 5-phosphate isomerase A (ribose 5-phosphate epimerase) [Monodelphis domestica] |
| TSAU.R66.esd | 2.7828E-5 | gi|256074910|ref|XP_002573765.1|hypothetical protein [Schistosoma mansoni] >gi|238658952|emb|CAZ29997.1| expressed protein [Schistosoma mansoni] |
| TSCD.R37.esd | 4.86618E-10 | gi|148665902|gb|EDK98318.1|amyloid beta (A4) precursor protein, isoform CRA_a [Mus musculus] |
| TSAH.R78.esd | 6.73236E-4 | gi|195144378|ref|XP_002013173.1|GL23535 [Drosophila persimilis] >gi|194102116|gb|EDW24159.1| GL23535 [Drosophila persimilis] |
| TSBU.R19.esd | 2.11847E-29 | gi|256086438|ref|XP_002579406.1|hypothetical protein [Schistosoma mansoni] >gi|238664839|emb|CAZ35645.1| expressed protein [Schistosoma mansoni] |
| TSBY.R9.esd | 1.87542E-14 | gi|56758682|gb|AAW27481.1|SJCHGC04646 protein [Schistosoma japonicum] |
| TSAS.R34.esd | 2.65656E-4 | gi|256073843|ref|XP_002573237.1|ran binding protein 9-related [Schistosoma mansoni] >gi|238658413|emb|CAZ29469.1| ran binding protein 9-related [Schistosoma mansoni] |
| TSBC.R81.esd | 7.53613E-11 | gi|76154807|gb|AAX26223.2|SJCHGC09551 protein [Schistosoma japonicum] |
| TSAQ.R65.esd | 2.96982E-15 | gi|256079340|ref|XP_002575946.1|ankyrin repeat and socs box protein 13 (asb-13) [Schistosoma mansoni] >gi|238661201|emb|CAZ32181.1| ankyrin repeat and socs box protein 13 (asb-13), putative [Schistosoma mansoni] |
| TSAJ.R91.esd | 1.59817E-13 | gi|76153199|gb|AAX24853.2|SJCHGC08931 protein [Schistosoma japonicum] |
| TSAP.R63.esd | 1.27826E-20 | gi|256084792|ref|XP_002578610.1|hypothetical protein [Schistosoma mansoni] >gi|238663989|emb|CAZ34848.1| expressed protein [Schistosoma mansoni] |
| TSCD.R49.esd | 2.08105E-29 | gi|256072833|ref|XP_002572738.1|coronin [Schistosoma mansoni] >gi|238657902|emb|CAZ28970.1| coronin, putative [Schistosoma mansoni] |
| TSCH.R90.esd | 6.52742E-23 | gi|226466706|emb|CAX69488.1|peptidylprolyl isomerase-like 4 [Schistosoma japonicum] |
| TSAE.R92.esd | 4.51343E-32 | gi|226490222|emb|CAX69353.1|pelota [Schistosoma japonicum] |
| TSBR.R2.esd | 6.14229E-13 | gi|256085136|ref|XP_002578779.1|hypothetical protein [Schistosoma mansoni] >gi|238664164|emb|CAZ35017.1| expressed protein [Schistosoma mansoni] |
| TSBH.R75.esd | 3.32331E-19 | gi|170031305|ref|XP_001843526.1|phosphatase-1 [Culex quinquefasciatus] >gi|167869786|gb|EDS33169.1| phosphatase-1 [Culex quinquefasciatus] |
| TSCF.R20.esd | 2.25483E-12 | gi|256080351|ref|XP_002576445.1|3'-5' exonuclease [Schistosoma mansoni] >gi|238661713|emb|CAZ32682.1| 3'-5' exonuclease eri1-related [Schistosoma mansoni] |
| TSBW.R80.esd | 5.13713E-20 | gi|170576532|ref|XP_001893666.1|14-3-3-like protein 2 [Brugia malayi] >gi|158600194|gb|EDP37497.1| 14-3-3-like protein 2, putative [Brugia malayi] |
| TSAP.R16.esd | 2.38718E-9 | gi|226467446|emb|CAX69599.1|Aconitase [Schistosoma japonicum] |
| TSAC.R2.esd | 4.74313E-28 | gi|25012186|gb|AAN71209.1|GM15177p [Drosophila melanogaster] |
| TSBF.R69.esd | 2.4405E-13 | gi|76154588|gb|AAX26042.2|SJCHGC02622 protein [Schistosoma japonicum] |
| TSBY.R45.esd | 6.83576E-21 | gi|56753919|gb|AAW25152.1|SJCHGC09069 protein [Schistosoma japonicum] |
| TSAO.R6.esd | 2.25377E-31 | gi|226478616|emb|CAX72803.1|Vacuolar H ATPase [Schistosoma japonicum] |
| TSAB.R11.esd | 1.70477E-85 | gi|218704462|ref|YP_002411981.1|drug efflux system protein MdtG [Escherichia coli UMN026] >gi|226702283|sp|B7NAS9.1|MDTG_ECOLU RecName: Full=Multidrug resistance protein mdtG >gi|218431559|emb|CAR12438.1| putative metabolite efflux transporter [Escherichia coli UMN026] |
| TSBR.R54.esd | 1.21667E-8 | gi|256071821|ref|XP_002572237.1|protein kinase; titin [Schistosoma mansoni] >gi|238657391|emb|CAZ28468.1| protein kinase; titin, putative [Schistosoma mansoni] |
| TSAY.R83.esd | 3.04837E-12 | gi|256089046|ref|XP_002580629.1|B-cell receptor-associated protein-like protein [Schistosoma mansoni] >gi|238666224|emb|CAZ36868.1| B-cell receptor-associated protein-like protein [Schistosoma mansoni] |
| TSAA.R14.esd | 7.80808E-40 | gi|62178030|gb|AAX73175.1|putative 14-3-3 protein [Echinococcus granulosus] |
| TSAV.R33.esd | 2.87587E-47 | gi|226479772|emb|CAX73182.1|translation initiation factor IF-2 unclassified subunit [Schistosoma japonicum] |
| TSCG.R36.esd | 2.6993E-21 | gi|124784512|gb|ABN14981.1|mitochondrial ATP synthase alpha subunit [Taenia asiatica] |
| TSBC.R23.esd | 1.60795E-9 | gi|48141081|ref|XP_397185.1|PREDICTED: similar to CG1319-PA [Apis mellifera] |
| TSAG.R53.esd | 2.36231E-12 | gi|256072897|ref|XP_002572770.1|hypothetical protein [Schistosoma mansoni] >gi|238657934|emb|CAZ29002.1| expressed protein [Schistosoma mansoni] |
| TSBB.R23.esd | 3.39412E-40 | gi|29337143|sp|Q9NFZ5.1|TBB3_ECHMURecName: Full=Tubulin beta-3 chain; AltName: Full=Beta-3-tubulin >gi|7838203|emb|CAB91642.1| beta-tubulin, Tub-3 [Echinococcus multilocularis] |
| TSAD.R15.esd | 2.13088E-34 | gi|256072958|ref|XP_002572800.1|hypothetical protein [Schistosoma mansoni] >gi|238657965|emb|CAZ29032.1| expressed protein [Schistosoma mansoni] |
| TSAY.R79.esd | 2.93024E-15 | gi|256075601|ref|XP_002574106.1|hypothetical protein [Schistosoma mansoni] >gi|238659302|emb|CAZ30339.1| expressed protein [Schistosoma mansoni] |
| TSCA.R88.esd | 1.98467E-14 | gi|256086054|ref|XP_002579221.1|dbl related [Schistosoma mansoni] >gi|238664640|emb|CAZ35460.1| dbl related [Schistosoma mansoni] |
| TSAL.R57.esd | 2.77898E-37 | gi|124783983|gb|ABN14959.1|calponin-like protein [Taenia asiatica] |
| TSAF.R45.esd | 2.21784E-51 | gi|256088487|ref|XP_002580365.1|rab GDP-dissociation inhibitor [Schistosoma mansoni] >gi|238665929|emb|CAZ36604.1| rab GDP-dissociation inhibitor, putative [Schistosoma mansoni] |
| TSBQ.R4.esd | 5.29131E-9 | gi|256084723|ref|XP_002578576.1|zinc finger protein [Schistosoma mansoni] >gi|238663954|emb|CAZ34814.1| zinc finger protein, putative [Schistosoma mansoni] |
| TSBD.R52.esd | 5.29376E-9 | gi|156353410|ref|XP_001623059.1|predicted protein [Nematostella vectensis] >gi|156209713|gb|EDO30959.1| predicted protein [Nematostella vectensis] |
| TSBU.R21.esd | 1.1007E-50 | gi|256075276|ref|XP_002573946.1|casein kinase II beta subunit [Schistosoma mansoni] >gi|238659137|emb|CAZ30179.1| casein kinase II beta subunit, putative [Schistosoma mansoni] |
| TSAV.R86.esd | 1.30376E-16 | gi|226466638|emb|CAX69454.1|Synaptosomal-associated protein 25 [Schistosoma japonicum] |
| TSBB.R44.esd | 3.64318E-21 | gi|256083552|ref|XP_002578006.1|hypothetical protein [Schistosoma mansoni] >gi|238663353|emb|CAZ34244.1| hypothetical protein [Schistosoma mansoni] |
| TSAF.R26.esd | 7.38128E-6 | gi|195582552|ref|XP_002081091.1|ERp60 [Drosophila simulans] >gi|194193100|gb|EDX06676.1| ERp60 [Drosophila simulans] |
| TSBI.R83.esd | 2.48006E-6 | gi|256073682|ref|XP_002573158.1|hypothetical protein [Schistosoma mansoni] >gi|238658331|emb|CAZ29390.1| expressed protein [Schistosoma mansoni] |
| TSAT.R84.esd | 1.16219E-64 | gi|260802572|ref|XP_002596166.1|hypothetical protein BRAFLDRAFT_57135 [Branchiostoma floridae] >gi|229281420|gb|EEN52178.1| hypothetical protein BRAFLDRAFT_57135 [Branchiostoma floridae] |
| TSBR.R27.esd | 7.12653E-14 | gi|18652196|gb|AAL77005.1|aldehyde dehydrogenase [Allium cepa] |
| TSBV.R53.esd | 1.47065E-6 | gi|226473910|emb|CAX77401.1|ribosomal protein L27 [Schistosoma japonicum] |
| TSCD.R46.esd | 3.59026E-5 | gi|256052760|ref|XP_002569920.1|hypothetical protein [Schistosoma mansoni] >gi|227284672|emb|CAY17428.1| expressed protein [Schistosoma mansoni] |
| TSAK.R66.esd | 8.77237E-12 | gi|150404780|gb|ABR68548.1|cystatin-1 [Clonorchis sinensis] |
| TSBW.R2.esd | 1.24394E-5 | gi|256083438|ref|XP_002577951.1|Mername-AA168 protein (M67 family) [Schistosoma mansoni] >gi|238663294|emb|CAZ34189.1| Mername-AA168 protein (M67 family) [Schistosoma mansoni] |
| TSBR.R3.esd | 7.32928E-6 | gi|56682906|gb|AAW21808.1|hypoxanthine-guanine phosphoribosyltranferase [Schistosoma japonicum] >gi|56759064|gb|AAW27672.1| SJCHGC02257 protein [Schistosoma japonicum] >gi|226469132|emb|CAX70045.1| hypoxanthine phosphoribosyltransferase 1 [Schistosoma japonicum] >gi|226469134|emb|CAX70046.1| hypoxanthine phosphoribosyltransferase 1 [Schistosoma japonicum] |
| TSAL.R68.esd | 2.60923E-11 | gi|56758488|gb|AAW27384.1|SJCHGC02564 protein [Schistosoma japonicum] >gi|226471024|emb|CAX70593.1| hypothetical protein [Schistosoma japonicum] >gi|226471026|emb|CAX70594.1| hypothetical protein [Schistosoma japonicum] >gi|226487290|emb|CAX75510.1| hypothetical protein [Schistosoma japonicum] >gi|226487292|emb|CAX75511.1| hypothetical protein [Schistosoma japonicum] >gi|226487294|emb|CAX75512.1| hypothetical protein [Schistosoma japonicum] |
| TSBI.R33.esd | 2.14327E-18 | gi|260781191|ref|XP_002585705.1|hypothetical protein BRAFLDRAFT_114166 [Branchiostoma floridae] >gi|229270737|gb|EEN41716.1| hypothetical protein BRAFLDRAFT_114166 [Branchiostoma floridae] |
| TSCC.R54.esd | 9.87461E-11 | gi|226487680|emb|CAX74710.1|Dpy-30-like protein [Schistosoma japonicum] >gi|226487682|emb|CAX74711.1| Dpy-30-like protein [Schistosoma japonicum] >gi|226487684|emb|CAX74712.1| Dpy-30-like protein [Schistosoma japonicum] |
| TSBA.R73.esd | 5.66169E-11 | gi|256071293|ref|XP_002571975.1|hypothetical protein [Schistosoma mansoni] >gi|238657125|emb|CAZ28205.1| expressed protein [Schistosoma mansoni] |
| TSCB.R83.esd | 1.27795E-42 | gi|46406288|emb|CAF18421.1|malate dehydrogenase [Echinococcus granulosus] |
| TSBY.R5.esd | 9.93879E-11 | gi|260834677|ref|XP_002612336.1|hypothetical protein BRAFLDRAFT_280861 [Branchiostoma floridae] >gi|229297713|gb|EEN68345.1| hypothetical protein BRAFLDRAFT_280861 [Branchiostoma floridae] |
| TSCH.R19.esd | 9.42854E-63 | gi|226470110|emb|CAX70336.1|hypotherical protein [Schistosoma japonicum] |
| TSBC.R7.esd | 5.71876E-42 | gi|256086201|ref|XP_002579292.1|ubiquitin-conjugating enzyme E2-25kD [Schistosoma mansoni] >gi|238664716|emb|CAZ35531.1| ubiquitin-conjugating enzyme E2-25kD, putative [Schistosoma mansoni] |
| TSBQ.R34.esd | 6.35751E-18 | gi|256070193|ref|XP_002571428.1|hypothetical protein [Schistosoma mansoni] >gi|238652659|emb|CAZ39113.1| expressed protein [Schistosoma mansoni] |
| TSBQ.R46.esd | 4.96579E-8 | gi|256053210|ref|XP_002570094.1|polyhomeotic [Schistosoma mansoni] >gi|227287465|emb|CAY17765.1| polyhomeotic, putative [Schistosoma mansoni] |
| TSBO.R53.esd | 1.40459E-17 | gi|56754185|gb|AAW25280.1|SJCHGC02500 protein [Schistosoma japonicum] |
| TSAA.R28.esd | 6.22658E-42 | gi|256073978|ref|XP_002573304.1|paraplegin (M41 family) [Schistosoma mansoni] >gi|238658481|emb|CAZ29536.1| paraplegin (M41 family) [Schistosoma mansoni] |
| TSBF.R1.esd | 5.44982E-62 | gi|124783152|gb|ABN14908.1|Hsp40 [Taenia asiatica] |
| TSAR.R13.esd | 1.0191E-24 | gi|156053564|ref|XP_001592708.1|ATP-dependent RNA helicase DED1 [Sclerotinia sclerotiorum 1980] >gi|160380639|sp|A7EJY3.1|DED1_SCLS1 RecName: Full=ATP-dependent RNA helicase ded1 >gi|154703410|gb|EDO03149.1| ATP-dependent RNA helicase DED1 [Sclerotinia sclerotiorum 1980] |
| TSBI.R82.esd | 7.72906E-8 | gi|209967593|gb|ACJ02401.1|TSP7 [Echinococcus multilocularis] |
| TSAO.R3.esd | 4.72577E-5 | gi|256079832|ref|XP_002576188.1|hypothetical protein [Schistosoma mansoni] >gi|238661451|emb|CAZ32423.1| conserved hypothetical protein [Schistosoma mansoni] |
| TSCH.R24.esd | 8.22034E-10 | gi|56479821|ref|NP_707016.2|putative metallodependent hydrolase [Shigella flexneri 2a str. 301] >gi|82544434|ref|YP_408381.1| putative metallodependent hydrolase [Shigella boydii Sb227] >gi|56383372|gb|AAN42723.2| orf, conserved hypothetical protein [Shigella flexneri 2a str. 301] >gi|81245845|gb|ABB66553.1| conserved hypothetical protein [Shigella boydii Sb227] |
| TSBD.R1.esd | 1.51459E-19 | gi|194667683|ref|XP_001788022.1|PREDICTED: similar to Glycine cleavage system H protein, mitochondrial [Bos taurus] |
| TSAU.R27.esd | 7.89061E-8 | gi|256062499|ref|XP_002570342.1|arp2/3 complex subunit 41-related [Schistosoma mansoni] >gi|227294401|emb|CAY18732.1| arp2/3 complex subunit 41-related [Schistosoma mansoni] |
| TSBN.R57.esd | 3.0625E-20 | gi|247533615|gb|ACS96861.1|cell wall-associated hydrolase [Aggregatibacter aphrophilus NJ8700] >gi|247533751|gb|ACS96997.1| cell wall-associated hydrolase [Aggregatibacter aphrophilus NJ8700] >gi|247534519|gb|ACS97765.1| cell wall-associated hydrolase [Aggregatibacter aphrophilus NJ8700] >gi|247534830|gb|ACS98076.1| cell wall-associated hydrolase [Aggregatibacter aphrophilus NJ8700] >gi|247535008|gb|ACS98254.1| cell wall-associated hydrolase [Aggregatibacter aphrophilus NJ8700] >gi|247535198|gb|ACS98444.1| cell wall-associated hydrolase [Aggregatibacter aphrophilus NJ8700] |
| TSBT.R9.esd | 1.55969E-6 | gi|209770298|gb|ACI83461.1|putative aldehyde dehydrogenase [Escherichia coli] >gi|209770304|gb|ACI83464.1| putative aldehyde dehydrogenase [Escherichia coli] |
| TSAA.R85.esd | 9.30439E-38 | gi|226472450|emb|CAX77261.1|carbonyl reductase 1 [Schistosoma japonicum] >gi|226472452|emb|CAX77262.1| carbonyl reductase 1 [Schistosoma japonicum] |
| TSCB.R62.esd | 8.42556E-7 | gi|226469080|emb|CAX70019.1|Myosin binding subunit [Schistosoma japonicum] |
| TSBM.R59.esd | 3.44848E-8 | gi|256081715|ref|XP_002577113.1|cation chloride cotransporter [Schistosoma mansoni] >gi|238662409|emb|CAZ33350.1| cation chloride cotransporter, putative [Schistosoma mansoni] |
| TSAF.R49.esd | 6.30952E-51 | gi|260855723|ref|YP_003229614.1|putative portal protein [Escherichia coli O26:H11 str. 11368] >gi|257754372|dbj|BAI25874.1| putative portal protein [Escherichia coli O26:H11 str. 11368] |
| TSBL.R43.esd | 6.39185E-26 | gi|226480012|emb|CAX73302.1|LIM and senescent cell antigen-like-containing domain protein 2 [Schistosoma japonicum] |
| TSBX.R20.esd | 5.48347E-35 | gi|194776926|ref|XP_001967857.1|GF20679 [Drosophila ananassae] >gi|195388846|ref|XP_002053089.1| GJ23530 [Drosophila virilis] >gi|190631494|gb|EDV44911.1| GF20679 [Drosophila ananassae] >gi|194151175|gb|EDW66609.1| GJ23530 [Drosophila virilis] |
| TSBG.R77.esd | 2.71753E-5 | gi|158288505|ref|XP_310377.6|AGAP003816-PA [Anopheles gambiae str. PEST] >gi|157019114|gb|EAA05968.5| AGAP003816-PA [Anopheles gambiae str. PEST] |
| TSAH.R46.esd | 1.03472E-15 | gi|226488116|emb|CAX75723.1|Ubiquitin C-terminal hydrolase [Schistosoma japonicum] >gi|226488118|emb|CAX75724.1| Ubiquitin C-terminal hydrolase [Schistosoma japonicum] |
| TSAG.R88.esd | 4.51065E-8 | gi|256086074|ref|XP_002579231.1|rna polymerase III (DNA directed) 39kD subunit-related [Schistosoma mansoni] >gi|238664650|emb|CAZ35470.1| rna polymerase III (DNA directed), 39kD subunit-related [Schistosoma mansoni] |
| TSAC.R13.esd | 6.77105E-35 | gi|226487218|emb|CAX75474.1|mitochondrial ribosomal protein L43 [Schistosoma japonicum] |
| TSAZ.R60.esd | 1.849E-28 | gi|56755992|gb|AAW26174.1|unknown [Schistosoma japonicum] >gi|226474196|emb|CAX71584.1| Splicing factor, arginine/serine-rich 10 [Schistosoma japonicum] >gi|226474198|emb|CAX71585.1| Splicing factor, arginine/serine-rich 10 [Schistosoma japonicum] >gi|226474564|emb|CAX77529.1| putative transformer-2 protein [Schistosoma japonicum] |
| TSAB.R17.esd | 4.34924E-29 | gi|256071077|ref|XP_002571868.1|cell division cycle 20 (cdc20) (fizzy) [Schistosoma mansoni] >gi|238657016|emb|CAZ28098.1| cell division cycle 20 (cdc20) (fizzy), putative [Schistosoma mansoni] |
| TSAT.R7.esd | 3.99134E-12 | gi|29841281|gb|AAP06313.1|similar to GenBank Accession Number AE003608 putative selT-like protein precursor, CG3887 gene product in Drosophila melanogaster [Schistosoma japonicum] >gi|226480076|emb|CAX73334.1| SelT-like protein precursor [Schistosoma japonicum] |
| TSBC.R58.esd | 2.74259E-13 | gi|198429317|ref|XP_002131784.1|PREDICTED: similar to chromatin modifyinG-protein 5 [Ciona intestinalis] |
| TSBQ.R56.esd | 1.31324E-9 | gi|147905458|ref|NP_001085338.1|MGC81169 protein [Xenopus laevis] >gi|49256534|gb|AAH71101.1| MGC81169 protein [Xenopus laevis] |
| TSAW.R24.esd | 7.62914E-32 | gi|256085436|ref|XP_002578927.1|zinc finger protein [Schistosoma mansoni] >gi|238664316|emb|CAZ35165.1| zinc finger protein, putative [Schistosoma mansoni] |
| TSAT.R9.esd | 9.31166E-25 | gi|56758904|gb|AAW27592.1|unknown [Schistosoma japonicum] |
| TSCC.R7.esd | 4.18346E-38 | gi|256086014|ref|XP_002579202.1|nucleotide-binding protein 1 (nbp 1)/nbp35 [Schistosoma mansoni] >gi|238664619|emb|CAZ35441.1| nucleotide-binding protein 1 (nbp 1)/nbp35, putative [Schistosoma mansoni] |
| TSAO.R47.esd | 3.44789E-18 | gi|256092940|ref|XP_002582135.1|protein tyrosine phosphatase n11 (shp2) [Schistosoma mansoni] >gi|238804778|emb|CAZ39235.1| protein tyrosine phosphatase n11 (shp2), putative [Schistosoma mansoni] |
| TSAB.R80.esd | 2.50582E-6 | gi|256070568|ref|XP_002571615.1|gh regulated tbc protein-1 [Schistosoma mansoni] >gi|238656760|emb|CAZ27845.1| gh regulated tbc protein-1, putative [Schistosoma mansoni] |
| TSBF.R44.esd | 5.33258E-57 | gi|148232513|ref|NP_001087574.1|MGC84749 protein [Xenopus laevis] >gi|51261715|gb|AAH80121.1| MGC84749 protein [Xenopus laevis] |
| TSAX.R66.esd | 3.08275E-41 | gi|21912554|emb|CAD21532.1|hypothetical protein [Taenia solium] |
| TSBC.R74.esd | 3.80304E-7 | gi|221131842|ref|XP_002155612.1|PREDICTED: similar to GDP dissociation inhibitor CG4422-PA isoform 2 [Hydra magnipapillata] |
| TSBR.R48.esd | 4.85453E-37 | gi|256073439|ref|XP_002573038.1|6-phosphofructokinase [Schistosoma mansoni] >gi|238658208|emb|CAZ29270.1| 6-phosphofructo-2-kinase/fructose-2,6-bisphosph at ase,putative [Schistosoma mansoni] |
| TSAY.R46.esd | 1.09947E-14 | gi|76154880|gb|AAX26281.2|SJCHGC02516 protein [Schistosoma japonicum] |
| TSAI.R47.esd | 1.18696E-20 | gi|256077991|ref|XP_002575282.1|nuclear protein localization [Schistosoma mansoni] >gi|256077993|ref|XP_002575283.1| nuclear protein localization [Schistosoma mansoni] >gi|238660516|emb|CAZ31515.1| nuclear protein localization, putative [Schistosoma mansoni] >gi|238660517|emb|CAZ31516.1| nuclear protein localization, putative [Schistosoma mansoni] |
| TSBA.R48.esd | 2.77782E-5 | gi|226488803|emb|CAX74751.1|Mastin precursor [Schistosoma japonicum] |
| TSAS.R87.esd | 1.91468E-14 | gi|11602713|emb|CAC18543.1|translation initiation factor 4A-like protein [Echinococcus multilocularis] |
| TSAT.R80.esd | 4.05471E-25 | gi|256084295|ref|XP_002578366.1|lupus la ribonucleoprotein [Schistosoma mansoni] >gi|238663736|emb|CAZ34604.1| lupus la ribonucleoprotein, putative [Schistosoma mansoni] |
| TSBE.R9.esd | 2.18289E-10 | gi|256093036|ref|XP_002582182.1|ecotropic viral integration site [Schistosoma mansoni] >gi|242322389|emb|CAY17078.1| ecotropic viral integration site, putative [Schistosoma mansoni] |
| TSCE.R49.esd | 2.27681E-7 | gi|257205820|emb|CAX82561.1|hypothetical protein [Schistosoma japonicum] |
| TSBE.R2.esd | 1.29334E-30 | gi|256079458|ref|XP_002576004.1|m-phase inducer phosphatase(cdc25) [Schistosoma mansoni] >gi|238661261|emb|CAZ32239.1| m-phase inducer phosphatase(cdc25), putative [Schistosoma mansoni] |
| TSAW.R12.esd | 2.49176E-14 | gi|256086393|ref|XP_002579384.1|NADH-ubiquinone oxidoreductase [Schistosoma mansoni] >gi|238664816|emb|CAZ35623.1| NADH-ubiquinone oxidoreductase subunit B17.2, putative [Schistosoma mansoni] |
| TSBD.R33.esd | 8.10638E-5 | gi|256083078|ref|XP_002577777.1|serine/threonine protein kinase [Schistosoma mansoni] >gi|238663108|emb|CAZ34015.1| serine/threonine kinase [Schistosoma mansoni] |
| TSCB.R30.esd | 4.26352E-6 | gi|226479796|emb|CAX73194.1|Paxillin [Schistosoma japonicum] |
| TSAS.R96.esd | 3.90008E-60 | gi|256071735|ref|XP_002572194.1|pol-related [Schistosoma mansoni] >gi|238657348|emb|CAZ28425.1| pol-related [Schistosoma mansoni] |
| TSAV.R74.esd | 3.81274E-23 | gi|86355167|dbj|BAE78818.1|innexin10 [Dugesia japonica] |
| TSBW.R86.esd | 4.28841E-14 | gi|256074369|ref|XP_002573498.1|hypothetical protein [Schistosoma mansoni] >gi|238658678|emb|CAZ29730.1| expressed protein [Schistosoma mansoni] |
| TSAG.R19.esd | 1.67458E-18 | gi|256088012|ref|XP_002580154.1|exostosin-1 [Schistosoma mansoni] >gi|238665665|emb|CAZ36393.1| exostosin-1, putative [Schistosoma mansoni] |
| TSBO.R32.esd | 1.04311E-20 | gi|66531474|ref|XP_624271.1|PREDICTED: similar to dj-1 CG1349-PA [Apis mellifera] |
| TSBL.R81.esd | 8.91797E-20 | gi|226467508|emb|CAX69630.1|short coiled-coil protein [Schistosoma japonicum] |
| TSAK.R19.esd | 1.20468E-5 | gi|256071832|ref|XP_002572242.1|hypothetical protein [Schistosoma mansoni] >gi|238657397|emb|CAZ28473.1| expressed protein [Schistosoma mansoni] |
| TSBD.R19.esd | 7.32681E-29 | gi|226466718|emb|CAX69494.1|Chitinase domain-containing protein 1 precursor [Schistosoma japonicum] |
| TSCB.R55.esd | 7.46247E-59 | gi|188492390|ref|ZP_02999660.1|catecholate siderophore receptor fiu [Escherichia coli 53638] >gi|188487589|gb|EDU62692.1| catecholate siderophore receptor fiu [Escherichia coli 53638] |
| TSCC.R43.esd | 6.4798E-45 | gi|156387846|ref|XP_001634413.1|predicted protein [Nematostella vectensis] >gi|156221496|gb|EDO42350.1| predicted protein [Nematostella vectensis] |
| TSAZ.R13.esd | 1.38592E-17 | gi|72016290|ref|XP_782052.1|PREDICTED: similar to MGC89089 protein [Strongylocentrotus purpuratus] >gi|115976620|ref|XP_001178432.1| PREDICTED: similar to MGC89089 protein [Strongylocentrotus purpuratus] |
| TSBG.R38.esd | 2.2215E-6 | gi|242002502|ref|XP_002435894.1|glycogenin-1, putative [Ixodes scapularis] >gi|215499230|gb|EEC08724.1| glycogenin-1, putative [Ixodes scapularis] |
| TSBT.R55.esd | 1.33571E-7 | gi|238501792|ref|XP_002382130.1|cytochrome B5, putative [Aspergillus flavus NRRL3357] >gi|220692367|gb|EED48714.1| cytochrome B5, putative [Aspergillus flavus NRRL3357] |
| TSCA.R77.esd | 6.26727E-10 | gi|242022948|ref|XP_002431899.1|cancer-associated gene protein, putative [Pediculus humanus corporis] >gi|212517240|gb|EEB19161.1| cancer-associated gene protein, putative [Pediculus humanus corporis] |
| TSCA.R11.esd | 8.36061E-10 | gi|260832702|ref|XP_002611296.1|hypothetical protein BRAFLDRAFT_210907 [Branchiostoma floridae] >gi|229296667|gb|EEN67306.1| hypothetical protein BRAFLDRAFT_210907 [Branchiostoma floridae] |
| TSAX.R48.esd | 7.83165E-16 | gi|256089044|ref|XP_002580628.1|groes chaperonin [Schistosoma mansoni] >gi|238666223|emb|CAZ36867.1| groes chaperonin, putative [Schistosoma mansoni] |
| TSAM.R62.esd | 7.39092E-17 | gi|109097844|ref|XP_001117631.1|PREDICTED: nucleosome assembly protein 1-like 1 isoform 5 [Macaca mulatta] |
| TSBC.R56.esd | 1.55742E-4 | gi|256077971|ref|XP_002575272.1|protein phosphatase 1 regulatory subunit 12b (myosin phosphatase targeting subunit 2) [Schistosoma mansoni] >gi|238660506|emb|CAZ31505.1| protein phosphatase 1 regulatory subunit 12b (myosin phosphatase targeting subunit 2), putative [Schistosoma mansoni] |
| TSAQ.R58.esd | 4.19218E-46 | gi|76155576|gb|AAX26868.2|SJCHGC06108 protein [Schistosoma japonicum] |
| TSBR.R86.esd | 2.94311E-35 | gi|256074323|ref|XP_002573475.1|hypothetical protein [Schistosoma mansoni] >gi|238658655|emb|CAZ29707.1| expressed protein [Schistosoma mansoni] |
| TSBX.R90.esd | 5.07886E-28 | gi|256090156|ref|XP_002581078.1|eukaryotic translation initiation factor 2c [Schistosoma mansoni] >gi|238666885|emb|CAZ37317.1| eukaryotic translation initiation factor 2c, putative [Schistosoma mansoni] |
| TSAM.R32.esd | 1.95855E-29 | gi|154369446|gb|ABS81352.1|phosphoglucose isomerase [Echinococcus multilocularis] |
| TSBT.R96.esd | 5.42117E-25 | gi|226467616|emb|CAX69684.1|ferrochelatase [Schistosoma japonicum] |
| TSAP.R91.esd | 1.76698E-28 | gi|226487954|emb|CAX75642.1|Acidic leucine-rich nuclear phosphoprotein 32-related protein (ANP32/acidic nuclear phosphoprotein-like protein) [Schistosoma japonicum] |
| TSBX.R77.esd | 1.62573E-5 | gi|74774988|sp|Q5DGJ1.1|KYNU_SCHJARecName: Full=Kynureninase; AltName: Full=L-kynurenine hydrolase >gi|56753736|gb|AAW25065.1| SJCHGC00887 protein [Schistosoma japonicum] |
| TSBU.R59.esd | 1.03064E-21 | gi|226470562|emb|CAX70561.1|growth factor receptor bound protein 2 [Schistosoma japonicum] |
| TSAL.R95.esd | 2.34613E-4 | gi|256072797|ref|XP_002572720.1|guanine-nucleotide-exchange-factor [Schistosoma mansoni] >gi|238657884|emb|CAZ28952.1| guanine-nucleotide-exchange-factor, putative [Schistosoma mansoni] |
| TSCC.R4.esd | 2.2415E-23 | gi|226467584|emb|CAX69668.1|guanine nucleotide binding protein (G protein), beta, other [Schistosoma japonicum] |
| TSBG.R11.esd | 7.21588E-6 | gi|256052362|ref|XP_002569741.1|dynein heavy chain [Schistosoma mansoni] >gi|227284453|emb|CAY17032.1| dynein heavy chain, putative [Schistosoma mansoni] |
| TSAQ.R3.esd | 1.7289E-27 | gi|256090526|ref|XP_002581238.1|phospholipid-transporting atpase [Schistosoma mansoni] >gi|238667095|emb|CAZ37477.1| phospholipid-transporting atpase [Schistosoma mansoni] |
| TSBE.R78.esd | 1.62222E-13 | gi|256083548|ref|XP_002578004.1|proteasome subunit alpha 2 (T01 family) [Schistosoma mansoni] >gi|238663351|emb|CAZ34242.1| proteasome subunit alpha 2 (T01 family) [Schistosoma mansoni] |
| TSAM.R13.esd | 1.40442E-8 | gi|256073411|ref|XP_002573024.1|hypothetical protein [Schistosoma mansoni] >gi|238658194|emb|CAZ29256.1| hypothetical protein flj40251 (C02 family) [Schistosoma mansoni] |
| TSBE.R41.esd | 1.61382E-5 | gi|256082026|ref|XP_002577264.1|hypothetical protein [Schistosoma mansoni] >gi|238662569|emb|CAZ33501.1| expressed protein [Schistosoma mansoni] |
| TSCG.R43.esd | 1.34205E-12 | gi|156546851|ref|XP_001606455.1|PREDICTED: similar to conserved hypothetical protein [Nasonia vitripennis] |
| TSBX.R38.esd | 5.20761E-12 | gi|256081083|ref|XP_002576803.1|histone-lysine n-methyltransferase seto7 [Schistosoma mansoni] >gi|238662087|emb|CAZ33040.1| histone-lysine n-methyltransferase, seto7, putative [Schistosoma mansoni] |
| TSBB.R85.esd | 6.00773E-16 | gi|226486980|emb|CAX75355.1|hypothetical protein [Schistosoma japonicum] >gi|226486982|emb|CAX75356.1| hypothetical protein [Schistosoma japonicum] |
| TSCE.R69.esd | 5.05895E-33 | gi|73993760|ref|XP_543203.2|PREDICTED: similar to cathepsin B preproprotein [Canis familiaris] |
| TSAW.R93.esd | 6.24987E-50 | gi|226466746|emb|CAX69508.1|Splicing factor 3A subunit 2 [Schistosoma japonicum] |
| TSCF.R86.esd | 2.35459E-33 | gi|194211086|ref|XP_001490668.2|PREDICTED: dihydropyrimidine dehydrogenase [Equus caballus] |
| TSCE.R88.esd | 2.21825E-23 | gi|226480514|emb|CAX73354.1|protein kinase like protein [Schistosoma japonicum] |
| TSBG.R70.esd | 8.26913E-10 | gi|221123264|ref|XP_002157956.1|PREDICTED: similar to KIAA0174 [Hydra magnipapillata] |
| TSCD.R72.esd | 4.06387E-65 | gi|256089808|ref|XP_002580948.1|hypothetical protein [Schistosoma mansoni] >gi|238666667|emb|CAZ37187.1| actin-related protein 2, arp2 [Schistosoma mansoni] |
| TSAJ.R47.esd | 2.07693E-4 | gi|189530654|ref|XP_694757.2|PREDICTED: similar to CG6650 CG6650-PA [Danio rerio] |
| TSBV.R45.esd | 5.64478E-51 | gi|256070997|ref|XP_002571828.1|DEAD box ATP-dependent RNA helicase [Schistosoma mansoni] >gi|238656976|emb|CAZ28058.1| DEAD box ATP-dependent RNA helicase, putative [Schistosoma mansoni] |
| TSAJ.R6.esd | 5.51503E-6 | gi|226479774|emb|CAX73183.1|ribosomal protein L29 [Schistosoma japonicum] |
| TSBM.R27.esd | 2.46801E-18 | gi|6679441|ref|NP_032934.1|peptidylprolyl isomerase C [Mus musculus] >gi|231961|sp|P30412.1|PPIC_MOUSE RecName: Full=Peptidyl-prolyl cis-trans isomerase C; Short=PPIase; Short=Rotamase; AltName: Full=Cyclophilin C >gi|192899|gb|AAA37511.1| cyclophilin C >gi|19483859|gb|AAH25861.1| Peptidylprolyl isomerase C [Mus musculus] >gi|74151911|dbj|BAE29741.1| unnamed protein product [Mus musculus] >gi|74191680|dbj|BAE30409.1| unnamed protein product [Mus musculus] >gi|74203447|dbj|BAE20880.1| unnamed protein product [Mus musculus] >gi|74223175|dbj|BAE40724.1| unnamed protein product [Mus musculus] >gi|148677963|gb|EDL09910.1| peptidylprolyl isomerase C, isoform CRA_b [Mus musculus] |
| TSBR.R14.esd | 2.46449E-7 | gi|256080950|ref|XP_002576738.1|hypothetical protein [Schistosoma mansoni] >gi|238662019|emb|CAZ32975.1| expressed protein [Schistosoma mansoni] |
| TSBS.R78.esd | 1.42194E-33 | gi|256088478|ref|XP_002580361.1|26S protease regulatory subunit [Schistosoma mansoni] >gi|238665924|emb|CAZ36600.1| 26S protease regulatory subunit, putative [Schistosoma mansoni] |
| TSBZ.R35.esd | 7.44953E-35 | gi|256088395|ref|XP_002580324.1|eukaryotic translation initiation factor 2 alpha subunit [Schistosoma mansoni] >gi|238665878|emb|CAZ36563.1| eukaryotic translation initiation factor 2 alpha subunit, putative [Schistosoma mansoni] |
| TSAD.R14.esd | 4.24699E-14 | gi|112419228|gb|AAI22510.1|Unknown (protein for MGC:154251) [Xenopus laevis] |
| TSAA.R1.esd | 1.09074E-54 | gi|226466728|emb|CAX69499.1|Tubulin gamma-1 chain (Gamma-1 tubulin) [Schistosoma japonicum] |
| TSAO.R53.esd | 6.19202E-38 | gi|74794482|sp|Q6RWA9.1|AT1A_TAESORecName: Full=Sodium/potassium-transporting ATPase subunit alpha; Short=Sodium pump subunit alpha; AltName: Full=Na(+)/K(+) ATPase alpha subunit; AltName: Full=TNaK1-alpha >gi|45360118|gb|AAS59168.1| Na+/K+-ATPase alpha subunit [Taenia solium] |
| TSAT.R72.esd | 5.0977E-19 | gi|256074517|ref|XP_002573571.1|importin 7 [Schistosoma mansoni] >gi|238658753|emb|CAZ29803.1| importin 7,, putative [Schistosoma mansoni] |
| TSBN.R66.esd | 1.09225E-9 | gi|70982776|ref|XP_746916.1|metallo-beta-lactamase domain protein [Aspergillus fumigatus Af293] >gi|66844540|gb|EAL84878.1| metallo-beta-lactamase domain protein [Aspergillus fumigatus Af293] >gi|159122841|gb|EDP47961.1| metallo-beta-lactamase domain protein [Aspergillus fumigatus A1163] |
| TSCG.R85.esd | 5.76189E-11 | gi|60691297|gb|AAX30558.1|SJCHGC05055 protein [Schistosoma japonicum] >gi|226489779|emb|CAX75040.1| DUF543 domain-containing protein [Schistosoma japonicum] >gi|226489781|emb|CAX75041.1| DUF543 domain-containing protein [Schistosoma japonicum] >gi|226489783|emb|CAX75042.1| DUF543 domain-containing protein [Schistosoma japonicum] |
| TSAA.R75.esd | 3.15308E-18 | gi|56755980|gb|AAW26168.1|SJCHGC02873 protein [Schistosoma japonicum] |
| TSCA.R72.esd | 4.89391E-39 | gi|256074426|ref|XP_002573526.1|ankyrin repeat-containing [Schistosoma mansoni] >gi|238658707|emb|CAZ29758.1| ankyrin repeat-containing, putative [Schistosoma mansoni] |
| TSAN.R68.esd | 7.11857E-46 | gi|21449873|ref|NP_659234.1|cytochrome c oxidase subunit II [Taenia solium] >gi|21388762|dbj|BAC00843.1| cytochrome c oxidase subunit 2 [Taenia solium] |
| TSBO.R89.esd | 5.93467E-8 | gi|226489518|emb|CAX75903.1|hypotheticial protein [Schistosoma japonicum] >gi|226489524|emb|CAX75906.1| hypotheticial protein [Schistosoma japonicum] |
| TSAM.R92.esd | 1.1129E-14 | gi|118511754|emb|CAL81524.1|putative peroxidase [Taenia solium] |
| TSBO.R37.esd | 1.67303E-18 | gi|256076696|ref|XP_002574646.1|tuftelin interacting protein [Schistosoma mansoni] >gi|238659857|emb|CAZ30879.1| tuftelin interacting protein, putative [Schistosoma mansoni] |
| TSBP.R43.esd | 1.56527E-27 | gi|114786425|gb|ABI78941.1|mitochondrial ATP synthase B subunit [Clonorchis sinensis] |
| TSCG.R88.esd | 2.74663E-53 | gi|226469236|emb|CAX70097.1|RNA-binding protein 39 [Schistosoma japonicum] |
| TSAO.R22.esd | 4.90112E-34 | gi|242247027|ref|NP_001156150.1|hypothetical protein LOC100162761 [Acyrthosiphon pisum] >gi|239788729|dbj|BAH71032.1| ACYPI003891 [Acyrthosiphon pisum] |
| TSAG.R96.esd | 3.63737E-5 | gi|226474034|emb|CAX77463.1|hypothetical protein [Schistosoma japonicum] >gi|226474046|emb|CAX77469.1| hypothetical protein [Schistosoma japonicum] |
| TSAD.R19.esd | 2.54561E-35 | gi|76162964|gb|ABA40864.1|SJCHGC07036 protein [Schistosoma japonicum] |
| TSAN.R80.esd | 6.79453E-64 | gi|225706152|gb|ACO08922.1|NADH dehydrogenase iron-sulfur protein 8, mitochondrial precursor [Osmerus mordax] |
| TSAX.R60.esd | 1.41256E-17 | gi|256074710|ref|XP_002573666.1|cytochrome C1 heme lyase [Schistosoma mansoni] >gi|238658851|emb|CAZ29898.1| cytochrome C1 heme lyase, putative [Schistosoma mansoni] |
| TSBN.R13.esd | 4.22854E-30 | gi|226481419|emb|CAX73607.1|uracil phosphoribosyltransferase 1 [Schistosoma japonicum] |
| TSCG.R58.esd | 5.41919E-9 | gi|256075962|ref|XP_002574284.1|hypothetical protein [Schistosoma mansoni] >gi|238659485|emb|CAZ30517.1| conserved hypothetical protein [Schistosoma mansoni] |
| TSBO.R6.esd | 9.43919E-14 | gi|256085947|ref|XP_002579170.1|hypothetical protein [Schistosoma mansoni] >gi|238664584|emb|CAZ35409.1| conserved hypothetical protein [Schistosoma mansoni] |
| TSAX.R71.esd | 2.69455E-32 | gi|56756156|gb|AAW26256.1|SJCHGC06423 protein [Schistosoma japonicum] >gi|226466776|emb|CAX69523.1| ORM1-like protein 2 [Schistosoma japonicum] |
| TSBR.R17.esd | 1.37648E-4 | gi|156543782|ref|XP_001606306.1|PREDICTED: similar to ENSANGP00000021528 [Nasonia vitripennis] |
| TSBX.R60.esd | 1.24548E-4 | gi|194912542|ref|XP_001982526.1|GG12689 [Drosophila erecta] >gi|190648202|gb|EDV45495.1| GG12689 [Drosophila erecta] |
| TSCF.R14.esd | 2.24328E-31 | gi|56753872|gb|AAW25133.1|SJCHGC06289 protein [Schistosoma japonicum] >gi|226474552|emb|CAX71762.1| F-type H+-transporting ATPase subunit f [Schistosoma japonicum] >gi|226474554|emb|CAX71763.1| F-type H+-transporting ATPase subunit f [Schistosoma japonicum] >gi|226476200|emb|CAX77951.1| F-type H+-transporting ATPase subunit f [Schistosoma japonicum] >gi|226476202|emb|CAX77952.1| F-type H+-transporting ATPase subunit f [Schistosoma japonicum] >gi|226476204|emb|CAX77953.1| F-type H+-transporting ATPase subunit f [Schistosoma japonicum] >gi|226476206|emb|CAX77954.1| F-type H+-transporting ATPase subunit f [Schistosoma japonicum] >gi|226476208|emb|CAX77955.1| F-type H+-transporting ATPase subunit f [Schistosoma japonicum] >gi|226476210|emb|CAX77956.1| F-type H+-transporting ATPase subunit f [Schistosoma japonicum] >gi|226476212|emb|CAX77957.1| F-type H+-transporting ATPase subunit f [Schistosoma japonicum] >gi|226476214|emb|CAX77958.1| F-type H+-transporting ATPase subunit f [Schistosoma japonicum] >gi|226476216|emb|CAX77959.1| F-type H+-transporting ATPase subunit f [Schistosoma japonicum] >gi|226476218|emb|CAX77960.1| F-type H+-transporting ATPase subunit f [Schistosoma japonicum] >gi|226476220|emb|CAX77961.1| F-type H+-transporting ATPase subunit f [Schistosoma japonicum] >gi|226476222|emb|CAX77962.1| F-type H+-transporting ATPase subunit f [Schistosoma japonicum] >gi|226476224|emb|CAX77963.1| F-type H+-transporting ATPase subunit f [Schistosoma japonicum] >gi|226476226|emb|CAX77964.1| F-type H+-transporting ATPase subunit f [Schistosoma japonicum] >gi|226476228|emb|CAX77965.1| F-type H+-transporting ATPase subunit f [Schistosoma japonicum] >gi|226476230|emb|CAX77966.1| F-type H+-transporting ATPase subunit f [Schistosoma japonicum] >gi|226476232|emb|CAX77967.1| F-type H+-transporting ATPase subunit f [Schistosoma japonicum] >gi|226476234|emb|CAX77968.1| F-type H+-transporting ATPase subunit f [Schistosoma japonicum] >gi|226476236|emb|CAX77969.1| F-type H+-transporting ATPase subunit f [Schistosoma japonicum] >gi|226476238|emb|CAX77970.1| F-type H+-transporting ATPase subunit f [Schistosoma japonicum] >gi|226476240|emb|CAX77971.1| F-type H+-transporting ATPase subunit f [Schistosoma japonicum] |
| TSBA.R25.esd | 5.1701E-18 | gi|76155836|gb|AAX27110.2|SJCHGC04022 protein [Schistosoma japonicum] |
| TSAB.R82.esd | 4.31546E-18 | gi|256074570|ref|XP_002573597.1|hypothetical protein [Schistosoma mansoni] >gi|238658780|emb|CAZ29829.1| expressed protein [Schistosoma mansoni] |
| TSBP.R25.esd | 8.75274E-29 | gi|226479834|emb|CAX73213.1|putative RNA-binding protein Raly [Schistosoma japonicum] |
| TSBL.R3.esd | 1.0085E-15 | gi|189533707|ref|XP_688089.3|PREDICTED: neural precursor cell expressed, developmentally down-regulated 4-like [Danio rerio] |
| TSAL.R22.esd | 1.62377E-13 | gi|256090229|ref|XP_002581107.1|myosin regulatory light chain [Schistosoma mansoni] >gi|238666929|emb|CAZ37346.1| myosin regulatory light chain 2 smooth muscle, putative [Schistosoma mansoni] |
| TSAA.R42.esd | 1.67079E-42 | gi|256072936|ref|XP_002572789.1|poly [ADP-ribose] polymerase [Schistosoma mansoni] >gi|238657954|emb|CAZ29021.1| poly [ADP-ribose] polymerase, putative [Schistosoma mansoni] |
| TSBU.R27.esd | 8.73473E-12 | gi|256083550|ref|XP_002578005.1|cdc37-related [Schistosoma mansoni] >gi|238663352|emb|CAZ34243.1| cdc37-related [Schistosoma mansoni] |
| TSBL.R60.esd | 4.52979E-40 | gi|149593415|ref|XP_001517120.1|PREDICTED: similar to drug resistance-related protein LRP [Ornithorhynchus anatinus] |
| TSCE.R78.esd | 5.62225E-35 | gi|256073903|ref|XP_002573267.1|cell division control protein 48 aaa family protein [Schistosoma mansoni] >gi|238658443|emb|CAZ29499.1| cell division control protein 48 aaa family protein (transitional endoplasmic reticulum atpase), putative [Schistosoma mansoni] |
| TSAP.R36.esd | 1.3322E-43 | gi|47223818|emb|CAF98588.1|unnamed protein product [Tetraodon nigroviridis] |
| TSCH.R61.esd | 4.70152E-35 | gi|256081213|ref|XP_002576867.1|ribonucleoside-diphosphate reductase small chain [Schistosoma mansoni] >gi|238662153|emb|CAZ33104.1| ribonucleoside-diphosphate reductase small chain, putative [Schistosoma mansoni] |
| TSAC.R61.esd | 5.36503E-32 | gi|256072056|ref|XP_002572353.1|RNA binding protein [Schistosoma mansoni] >gi|238657510|emb|CAZ28584.1| RNA binding protein, putative [Schistosoma mansoni] |
| TSAP.R46.esd | 4.10882E-50 | gi|167541050|gb|ABZ82035.1|phosphoglycerate mutase [Clonorchis sinensis] |
| TSBP.R86.esd | 4.07356E-9 | gi|256087964|ref|XP_002580131.1|hypothetical protein [Schistosoma mansoni] >gi|238665640|emb|CAZ36370.1| expressed protein [Schistosoma mansoni] |
| TSBK.R42.esd | 6.98854E-49 | gi|126323328|ref|XP_001377339.1|PREDICTED: similar to protein kinase A alpha [Monodelphis domestica] |
| TSAQ.R31.esd | 4.99751E-15 | gi|74835178|dbj|BAE44472.1|Vasa [Botryllus primigenus] |
| TSBX.R81.esd | 1.32415E-7 | gi|224050967|ref|XP_002199515.1|PREDICTED: similar to choline kinase alpha isoform 2 [Taeniopygia guttata] |
| TSAT.R78.esd | 2.48314E-13 | gi|56756869|gb|AAW26606.1|SJCHGC06728 protein [Schistosoma japonicum] >gi|226487018|emb|CAX75374.1| Augmenter of liver regeneration [Schistosoma japonicum] >gi|226487022|emb|CAX75376.1| Augmenter of liver regeneration [Schistosoma japonicum] |
| TSBT.R7.esd | 2.10005E-9 | gi|256075656|ref|XP_002574133.1|lipase maturation factor [Schistosoma mansoni] >gi|238659330|emb|CAZ30366.1| lipase maturation factor [Schistosoma mansoni] |
| TSBU.R81.esd | 6.39505E-39 | gi|256070187|ref|XP_002571425.1|50S ribosomal protein L13 [Schistosoma mansoni] >gi|238652656|emb|CAZ39110.1| 50S ribosomal protein L13, putative [Schistosoma mansoni] |
| TSAY.R44.esd | 6.11467E-53 | gi|256073227|ref|XP_002572933.1|proteasome subunit alpha 6 (T01 family) [Schistosoma mansoni] >gi|238658101|emb|CAZ29165.1| proteasome subunit alpha 6 (T01 family) [Schistosoma mansoni] |
| TSBO.R48.esd | 7.08982E-17 | gi|225711972|gb|ACO11832.1|Chromobox protein homolog 1 [Lepeophtheirus salmonis] |
| TSAQ.R46.esd | 4.75327E-42 | gi|256088516|ref|XP_002580378.1|DEAD box ATP-dependent RNA helicase [Schistosoma mansoni] >gi|238665945|emb|CAZ36617.1| DEAD box ATP-dependent RNA helicase, putative [Schistosoma mansoni] |
| TSCA.R15.esd | 7.62369E-6 | gi|149773480|ref|NP_001092729.1|RAB8B, member RAS oncogene family [Danio rerio] >gi|148744724|gb|AAI42847.1| Rab8b protein [Danio rerio] |
| TSCE.R92.esd | 8.06043E-21 | gi|260449630|gb|ACX40052.1|iron-containing alcohol dehydrogenase [Escherichia coli DH1] |
| TSAT.R71.esd | 5.48169E-6 | gi|251765182|sp|A8Q2R5.2|WDR48_BRUMARecName: Full=WD repeat-containing protein 48 homolog |
| TSBY.R46.esd | 5.30968E-17 | gi|226480692|emb|CAX73443.1|NADH:ubiquinone reductase 42kD subunit precurs [Schistosoma japonicum] |
| TSCB.R6.esd | 3.33016E-19 | gi|116791141|gb|ABK25871.1|unknown [Picea sitchensis] |
| TSBE.R29.esd | 6.33344E-10 | gi|256091723|ref|XP_002581693.1|hypothetical protein [Schistosoma mansoni] >gi|238667840|emb|CAZ37934.1| hypothetical protein [Schistosoma mansoni] |
| TSAD.R73.esd | 3.70673E-10 | gi|153792659|ref|NP_001093278.1|actin-depolymerizing factor 1 [Bombyx mori] >gi|95102548|gb|ABF51212.1| actin-depolymerizing factor 4 [Bombyx mori] >gi|95103010|gb|ABF51446.1| actin-depolymerizing factor 1 [Bombyx mori] |
| TSBX.R71.esd | 1.14215E-12 | gi|256079165|ref|XP_002575860.1|hypothetical protein [Schistosoma mansoni] >gi|238661112|emb|CAZ32095.1| expressed protein [Schistosoma mansoni] |
| TSAR.R69.esd | 8.74324E-12 | gi|29378341|gb|AAO83852.1|AF484095_1munc18-1-interacting protein 1 [Lymnaea stagnalis] |
| TSBT.R8.esd | 3.92425E-28 | gi|163759690|ref|ZP_02166775.1|Fumarate lyase:Fe-S type hydro-lyase tartrate/fumarate alpha region:Fe-S type hydro-lyase tartrate/fumarate beta [Hoeflea phototrophica DFL-43] >gi|162283287|gb|EDQ33573.1| Fumarate lyase:Fe-S type hydro-lyase tartrate/fumarate alpha region:Fe-S type hydro-lyase tartrate/fumarate beta [Hoeflea phototrophica DFL-43] |
| TSAQ.R67.esd | 2.37242E-4 | gi|256076655|ref|XP_002574626.1|phosphoinositol 4-phosphate adaptor protein [Schistosoma mansoni] >gi|238659836|emb|CAZ30859.1| phosphoinositol 4-phosphate adaptor protein, putative [Schistosoma mansoni] |
| TSAZ.R48.esd | 7.80717E-16 | gi|224076335|ref|XP_002197327.1|PREDICTED: PRP8 pre-mRNA processing factor 8 homolog (S. cerevisiae) [Taeniopygia guttata] |
| TSBR.R51.esd | 3.01136E-8 | gi|224079946|ref|XP_002186915.1|PREDICTED: putative cortistatin [Taeniopygia guttata] |
| TSAD.R18.esd | 1.08665E-9 | gi|189525823|ref|XP_001923096.1|PREDICTED: hypothetical protein LOC393849 [Danio rerio] |
| TSBR.R72.esd | 5.25829E-5 | gi|256082354|ref|XP_002577422.1|Ubiquitin-protein ligase BRE1 [Schistosoma mansoni] >gi|238662739|emb|CAZ33660.1| Ubiquitin-protein ligase BRE1, putative [Schistosoma mansoni] |
| TSAT.R90.esd | 1.59666E-56 | gi|218689415|ref|YP_002397627.1|altronate oxidoreductase [Escherichia coli ED1a] >gi|218426979|emb|CAR07850.2| altronate oxidoreductase, NAD-dependent [Escherichia coli ED1a] |
| TSAX.R47.esd | 1.10079E-38 | gi|256090542|ref|XP_002581246.1|phosphoglucomutase [Schistosoma mansoni] >gi|238667103|emb|CAZ37485.1| phosphoglucomutase, putative [Schistosoma mansoni] |
| TSAJ.R55.esd | 1.09439E-25 | gi|256081277|ref|XP_002576898.1|hypothetical protein [Schistosoma mansoni] >gi|238662186|emb|CAZ33135.1| conserved hypothetical protein [Schistosoma mansoni] |
| TSCH.R48.esd | 1.27401E-20 | gi|226484664|emb|CAX74241.1|Dentin sialophosphoprotein precursor [Schistosoma japonicum] |
| TSAY.R37.esd | 6.85098E-12 | gi|226468234|emb|CAX69794.1|Cleavage stimulation factor 50 kDa subunit [Schistosoma japonicum] |
| TSAJ.R30.esd | 3.02658E-4 | gi|256077977|ref|XP_002575275.1|guanyl-nucleotide exchange factor [Schistosoma mansoni] >gi|238660509|emb|CAZ31508.1| guanyl-nucleotide exchange factor, putative [Schistosoma mansoni] |
| TSBX.R44.esd | 4.11624E-9 | gi|56755121|gb|AAW25740.1|unknown [Schistosoma japonicum] >gi|60687690|gb|AAX30178.1| SJCHGC01506 protein [Schistosoma japonicum] |
| TSBZ.R36.esd | 1.15178E-17 | gi|241568971|ref|XP_002402616.1|conserved hypothetical protein [Ixodes scapularis] >gi|215500057|gb|EEC09551.1| conserved hypothetical protein [Ixodes scapularis] |
| TSAA.R21.esd | 7.6034E-46 | gi|256089271|ref|XP_002580736.1|TATA-box binding protein [Schistosoma mansoni] >gi|238666342|emb|CAZ36975.1| TATA-box binding protein, putative [Schistosoma mansoni] |
| TSAO.R20.esd | 2.14576E-10 | gi|256072934|ref|XP_002572788.1|riboflavin kinase/fmn adenylyltransferase [Schistosoma mansoni] >gi|238657953|emb|CAZ29020.1| riboflavin kinase/fmn adenylyltransferase, putative [Schistosoma mansoni] |
| TSAH.R28.esd | 2.21826E-39 | gi|56759068|gb|AAW27674.1|SJCHGC04249 protein [Schistosoma japonicum] |
| TSBA.R45.esd | 4.18346E-38 | gi|21449863|ref|NP_659224.1|NADH dehydrogenase subunit 5 [Taenia solium] >gi|21388752|dbj|BAC00833.1| NADH dehydrogenase subunit 5 [Taenia solium] |
| TSBV.R32.esd | 4.01383E-12 | gi|225704428|gb|ACO08060.1|Golgi SNAP receptor complex member 1 [Oncorhynchus mykiss] |
