# Supplementary material for: Analyses of an Expressed Sequence Tag Library from Taenia solium, Cysticerca
Source: PLoS Negl Trop Dis. 2010 Dec 21;4(12):e919. doi: 10.1371/journal.pntd.0000919 (PMC3006133; doi:10.1371/journal.pntd.0000919)
Supplement: Table S2 — Result from BLAST search with a score <10−5 of 154 unique ESTs predicted to contain a signal peptide. (0.05 MB DOC) [file pntd.0000919.s004.doc]

| **Sequence name** | **E-Value** | **Hit desc.** |
| --- | --- | --- |
|  |  |  |
| TS. Contig74_rframe3_ORF | 1.0685E-44 | gi|21912582|emb|CAD21546.1|hypothetical protein [Taenia solium] |
| TS. Contig84_rframe1_ORF | 1.56257E-21 | gi|226490138|emb|CAX69311.1|MON2 like protein [Schistosoma japonicum] |
| TS. Contig220_rframe1_ORF | 2.18236E-15 | gi|124784850|gb|ABN15004.1|proliferating cell nuclear antigen [Taenia asiatica] |
| TS. Contig280_rframe2_ORF | 2.24793E-51 | gi|256071126|ref|XP_002571892.1|vacuolar ATP synthase proteolipid subunit 1 2 3 [Schistosoma mansoni] >gi|238657041|emb|CAZ28122.1| vacuolar ATP synthase proteolipid subunit 1, 2, 3,, putative [Schistosoma mansoni] |
| TS. Contig286_rframe1_ORF | 3.39746E-5 | gi|256075188|ref|XP_002573902.1|multidrug resistance protein [Schistosoma mansoni] >gi|238659093|emb|CAZ30135.1| multidrug resistance pump, putative [Schistosoma mansoni] |
| TS. Contig317_rframe2_ORF | 5.4016E-31 | gi|256083900|ref|XP_002578173.1|tubulin tyrosine ligase [Schistosoma mansoni] >gi|238663534|emb|CAZ34411.1| tubulin tyrosine ligase-related [Schistosoma mansoni] |
| TS. Contig318_rframe2_ORF | 5.28511E-10 | gi|223037336|gb|ACM79010.1|Kunitz protein 8 [Echinococcus granulosus] |
| TS. Contig374_rframe1_ORF | 1.62257E-29 | gi|256077305|ref|XP_002574947.1|60S ribosomal protein L37 [Schistosoma mansoni] >gi|6094071|sp|O44125.3|RL37_SCHMA RecName: Full=60S ribosomal protein L37 >gi|2665824|gb|AAB88508.1| ribosomal protein L37 [Schistosoma mansoni] >gi|238660165|emb|CAZ31180.1| 60S ribosomal protein L37, putative [Schistosoma mansoni] |
| TS. Contig381_rframe3_ORF | 1.45062E-22 | gi|22004048|dbj|BAC06474.1|ubiquitin [Ciona savignyi] |
| TS. Contig389_rframe3_ORF | 5.30367E-44 | gi|116687782|gb|AAT74668.2|cysteine-rich secreted protein 2 precursor [Mesocestoides vogae] |
| TS. Contig391_rframe1_ORF | 8.51772E-63 | gi|76156528|gb|AAX27719.2|SJCHGC00991 protein [Schistosoma japonicum] |
| TS. Contig401_rframe3_ORF | 4.38368E-10 | gi|256079415|ref|XP_002575983.1|hypothetical protein [Schistosoma mansoni] >gi|238661239|emb|CAZ32218.1| expressed protein [Schistosoma mansoni] |
| TS. Contig469_rframe2_ORF  TS.Contig478_ rframe2_ORF  TS. Contig491_rframe1_ORF | 5.30931E-64  1.12293E-11  3.14128E-66 | gi|256082102|ref|XP_002577301.1|hypothetical protein [Schistosoma mansoni] >gi|238662608|emb|CAZ33538.1| expressed protein [Schistosoma mansoni]  gi|59709858|gb|AAW88559.1|oncosphere protein Tso31d [Taenia solium]  gi|158934366|emb|CAO82075.1|HP6 protein [Taenia solium] |
| TS.Contig492_rframe1_ORF | 7.00788E-5 | gi|2114399|gb|AAC47532.1|45W antigen ToW5/7 [Taenia ovis] |
| TS. Contig497_rframe2_ORF | 4.14398E-10 | gi|73977215|ref|XP_862657.1|PREDICTED: similar to ATPase, H+ transporting, lysosomal 21kDa, V0 subunit c isoform 3 [Canis familiaris] |
| TSCC.R39.esd | 3.88078E-27 | gi|45238335|emb|CAD12372.2|serine protease inhibitor [Echinococcus multilocularis] |
| TSBC.R73.esd | 1.76543E-5 | gi|242013395|ref|XP_002427393.1|vacuole membrane protein, putative [Pediculus humanus corporis] >gi|212511767|gb|EEB14655.1| vacuole membrane protein, putative [Pediculus humanus corporis] |
| TSAG.R38.esd | 8.75638E-35 | gi|256077722|ref|XP_002575150.1|succinate dehydrogenase [Schistosoma mansoni] >gi|238660379|emb|CAZ31383.1| succinate dehydrogenase, putative [Schistosoma mansoni] |
| TSBD.R25.esd | 6.20726E-12 | gi|218548930|ref|YP_002382721.1|glutamate:gamma-aminobutyric acid antiporter [Escherichia fergusonii ATCC 35469] >gi|218356471|emb|CAQ89094.1| glutamate:gamma-aminobutyric acid antiporter [Escherichia fergusonii ATCC 35469] |
| TSCC.R93.esd | 3.60783E-29 | gi|209967599|gb|ACJ02404.1|TSP3 [Echinococcus multilocularis] |
| TSAG.R62.esd | 3.25024E-52 | gi|23095931|dbj|BAC16311.1|Raichu-1011X [synthetic construct] |
| TSBO.R22.esd | 1.79175E-18 | gi|260460285|ref|ZP_05808537.1|Diverse 7TM receptor transmembrane region [Mesorhizobium opportunistum WSM2075] >gi|259033930|gb|EEW35189.1| Diverse 7TM receptor transmembrane region [Mesorhizobium opportunistum WSM2075] |
| TSAE.R73.esd | 1.4461E-20 | gi|224083628|ref|XP_002193242.1|PREDICTED: solute carrier family 37 (glycerol-3-phosphate transporter), member 2 [Taeniopygia guttata] |
| TSBA.R34.esd | 1.78848E-6 | gi|195342049|ref|XP_002037614.1|GM18357 [Drosophila sechellia] >gi|194132464|gb|EDW54032.1| GM18357 [Drosophila sechellia] |
| TSCC.R47.esd | 1.09441E-39 | gi|112984454|ref|NP_001037171.1|protein disulfide isomerase [Bombyx mori] >gi|12025459|gb|AAG45936.1|AF325211_1 protein disulfide isomerase [Bombyx mori] |
| TSCD.R37.esd | 6.97453E-8 | gi|553819|gb|AAA36828.1|amyloid b-protein precursor |
| TSAH.R78.esd | 7.13273E-13 | gi|47229974|emb|CAG10388.1|unnamed protein product [Tetraodon nigroviridis] |
| TSAY.R79.esd | 3.63854E-26 | gi|256075601|ref|XP_002574106.1|hypothetical protein [Schistosoma mansoni] >gi|238659302|emb|CAZ30339.1| expressed protein [Schistosoma mansoni] |
| TSBI.R83.esd | 3.55325E-4 | gi|256073682|ref|XP_002573158.1|hypothetical protein [Schistosoma mansoni] >gi|238658331|emb|CAZ29390.1| expressed protein [Schistosoma mansoni] |
| TSBQ.R56.esd | 2.20894E-10 | gi|49116627|gb|AAH73658.1|LOC443680 protein [Xenopus laevis] |
| TSAA.R66.esd | 1.82355E-56 | gi|21912554|emb|CAD21532.1|hypothetical protein [Taenia solium] |
| TSAN.R57.esd | 1.05644E-5 | gi|115720963|ref|XP_001175524.1|PREDICTED: similar to aquaporin-4 M1 isoform [Strongylocentrotus purpuratus] >gi|115803224|ref|XP_799266.2| PREDICTED: similar to aquaporin-4 M1 isoform [Strongylocentrotus purpuratus] |
| TSBM.R27.esd | 6.83472E-15 | gi|6679441|ref|NP_032934.1|peptidylprolyl isomerase C [Mus musculus] >gi|231961|sp|P30412.1|PPIC_MOUSE RecName: Full=Peptidyl-prolyl cis-trans isomerase C; Short=PPIase; Short=Rotamase; AltName: Full=Cyclophilin C >gi|192899|gb|AAA37511.1| cyclophilin C >gi|19483859|gb|AAH25861.1| Peptidylprolyl isomerase C [Mus musculus] >gi|74151911|dbj|BAE29741.1| unnamed protein product [Mus musculus] >gi|74191680|dbj|BAE30409.1| unnamed protein product [Mus musculus] >gi|74203447|dbj|BAE20880.1| unnamed protein product [Mus musculus] >gi|74223175|dbj|BAE40724.1| unnamed protein product [Mus musculus] >gi|148677963|gb|EDL09910.1| peptidylprolyl isomerase C, isoform CRA_b [Mus musculus] |
| TSAC.R61.esd | 5.1722E-26 | gi|256072054|ref|XP_002572352.1|RNA binding protein [Schistosoma mansoni] >gi|238657509|emb|CAZ28583.1| RNA binding protein, putative [Schistosoma mansoni] |
| TSAH.R56.esd | 2.88996E-50 | gi|256075044|ref|XP_002573831.1|ribosome biogenesis protein bop1 (block of proliferation 1 protein) [Schistosoma mansoni] >gi|238659020|emb|CAZ30064.1| ribosome biogenesis protein bop1 (block of proliferation 1 protein), putative [Schistosoma mansoni] |
