# Supplementary material for: Analyses of an Expressed Sequence Tag Library from Taenia solium, Cysticerca
Source: PLoS Negl Trop Dis. 2010 Dec 21;4(12):e919. doi: 10.1371/journal.pntd.0000919 (PMC3006133; doi:10.1371/journal.pntd.0000919)
Supplement: Table S4 — Result from KOBAS web based annotation program. Results are presented as hits with a score <10−5. Values in the column “Count and ratio” are divided into two rows per pathway. The top row corresponds to actual value. Bottom row corresponds to value in C. elegans for comparison. (0.48 MB DOC) [file pntd.0000919.s006.doc]

| Pathway | Count and ratio | P-value | Q-value |
| --- | --- | --- | --- |
| [Ribosome](http://kobas.cbi.pku.edu.cn:80/program.run.do#Ribosome) | 41 / 10.30%  84 / 2.40% | 0.0 | 0.0 |
| [Primary immunodeficiency](http://kobas.cbi.pku.edu.cn:80/program.run.do#Primary%20immunodeficiency) | 2 / 0.50%  1 / 0.03% | 0 | 0.0 |
| [Pores ion channels](http://kobas.cbi.pku.edu.cn:80/program.run.do#Pores%20ion%20channels) | 1 / 0.25% | 0 | 0.0 |
| [Pathogenic Escherichia coli infection](http://kobas.cbi.pku.edu.cn:80/program.run.do#Pathogenic%20Escherichia%20coli%20infection) | 23 / 5.78%  31 / 0.88% | 3.33066907388e-16 | 1.46856764327e-14 |
| [Leukocyte transendothelial migration](http://kobas.cbi.pku.edu.cn:80/program.run.do#Leukocyte%20transendothelial%20migration) | 19 / 4.77%  26 / 0.74% | 2.34035013591e-13 | 8.25530824538e-12 |
| [Regulation of actin cytoskeleton](http://kobas.cbi.pku.edu.cn:80/program.run.do#Regulation%20of%20actin%20cytoskeleton) | 25 / 6.28%  45 / 1.28% | 4.37649916307e-13 | 1.28646525551e-11 |
| [Cytoskeleton proteins](http://kobas.cbi.pku.edu.cn:80/program.run.do#Cytoskeleton%20proteins) | 32 / 8.04%  72 / 2.05% | 7.10209668853e-13 | 1.78941470743e-11 |
| [Adherens junction](http://kobas.cbi.pku.edu.cn:80/program.run.do#Adherens%20junction) | 17 / 4.27%  23 / 0.66% | 3.36641825527e-12 | 7.42165134655e-11 |
| [Glycolysis / Gluconeogenesis](http://kobas.cbi.pku.edu.cn:80/program.run.do#Glycolysis%20/%20Gluconeogenesis) | 21 / 5.28%  36 / 1.03% | 9.76596581381e-12 | 1.9137938396e-10 |
| [Pyruvate metabolism](http://kobas.cbi.pku.edu.cn:80/program.run.do#Pyruvate%20metabolism) | 16 / 4.02%  23 / 0.66% | 6.70195010599e-11 | 1.18201680854e-09 |
| [Viral myocarditis](http://kobas.cbi.pku.edu.cn:80/program.run.do#Viral%20myocarditis) | 12 / 3.02%  15 / 0.43% | 1.30961907985e-09 | 2.09978471758e-08 |
| [Focal adhesion](http://kobas.cbi.pku.edu.cn:80/program.run.do#Focal%20adhesion) | 23 / 5.78%  52 / 1.48% | 1.57196566875e-09 | 2.31038455663e-08 |
| [Hypertrophic cardiomyopathy (HCM)](http://kobas.cbi.pku.edu.cn:80/program.run.do" \l "Hypertrophic cardiomyopathy (HCM)) | 9 / 2.26%  9 / 0.26% | 2.9024900261e-09 | 3.75646875563e-08 |
| [Arrhythmogenic right ventricular cardiomyopathy (ARVC)](http://kobas.cbi.pku.edu.cn:80/program.run.do" \l "Arrhythmogenic right ventricular cardiomyopathy (ARVC)) | 10 / 2.51%  11 / 0.31% | 3.19483606237e-09 | 3.75646875563e-08 |
| [Dilated cardiomyopathy (DCM)](http://kobas.cbi.pku.edu.cn:80/program.run.do" \l "Dilated cardiomyopathy (DCM)) | 10 / 2.51%  11 / 0.31% | 3.19483606237e-09 | 3.75646875563e-08 |
| [Citrate cycle (TCA cycle)](http://kobas.cbi.pku.edu.cn:80/program.run.do" \l "Citrate cycle (TCA cycle)) | 16 / 4.02%  32 / 0.91% | 6.184020096e-08 | 6.81668729082e-07 |
| [Tight junction](http://kobas.cbi.pku.edu.cn:80/program.run.do#Tight%20junction) | 17 / 4.27%  39 / 1.11% | 2.98737732662e-07 | 3.09930003486e-06 |
| [Neurotrophin signaling pathway](http://kobas.cbi.pku.edu.cn:80/program.run.do#Neurotrophin%20signaling%20pathway) | 15 / 3.77%  35 / 1.00% | 1.98248644812e-06 | 1.94249622814e-05 |
| [Vibrio cholerae infection](http://kobas.cbi.pku.edu.cn:80/program.run.do#Vibrio%20cholerae%20infection) | 15 / 3.77%  37 / 1.06% | 4.60515664868e-06 | 4.27477507811e-05 |
| [Fc gamma R-mediated phagocytosis](http://kobas.cbi.pku.edu.cn:80/program.run.do#Fc%20gamma%20R-mediated%20phagocytosis) | 12 / 3.02%  28 / 0.80% | 2.17455540907e-05 | 0.000191762173992 |
| [Carbon fixation in photosynthetic organisms](http://kobas.cbi.pku.edu.cn:80/program.run.do#Carbon%20fixation%20in%20photosynthetic%20organisms) | 9 / 2.26%  19 / 0.54% | 9.37436345179e-05 | 0.000787308526346 |
| [Glyoxylate and dicarboxylate metabolism](http://kobas.cbi.pku.edu.cn:80/program.run.do#Glyoxylate%20and%20dicarboxylate%20metabolism) | 5 / 1.26%  7 / 0.20% | 0.000319053804515 | 0.00255778307263 |
| [Reductive carboxylate cycle (CO2 fixation)](http://kobas.cbi.pku.edu.cn:80/program.run.do" \l "Reductive carboxylate cycle (CO2 fixation)) | 6 / 1.51%  11 / 0.31% | 0.000580245201537 | 0.00444944849239 |
| [Chemokine signaling pathway](http://kobas.cbi.pku.edu.cn:80/program.run.do#Chemokine%20signaling%20pathway) | 11 / 2.76%  35 / 1.00% | 0.00116796771184 | 0.00858305819816 |
| [Insulin signaling pathway](http://kobas.cbi.pku.edu.cn:80/program.run.do#Insulin%20signaling%20pathway) | 13 / 3.27%  50 / 1.43% | 0.00295914507367 | 0.0208760683721 |
| [Chaperones and folding catalysts](http://kobas.cbi.pku.edu.cn:80/program.run.do#Chaperones%20and%20folding%20catalysts) | 19 / 4.77%  87 / 2.48% | 0.00330289663193 | 0.0224049560435 |
| [Renal cell carcinoma](http://kobas.cbi.pku.edu.cn:80/program.run.do#Renal%20cell%20carcinoma) | 8 / 2.01%  24 / 0.68% | 0.00365898758873 | 0.0239011960516 |
| [Spliceosome](http://kobas.cbi.pku.edu.cn:80/program.run.do#Spliceosome) | 21 / 5.28%  102 / 2.91% | 0.00434562990543 | 0.0273726703777 |
| [Pentose phosphate pathway](http://kobas.cbi.pku.edu.cn:80/program.run.do#Pentose%20phosphate%20pathway) | 7 / 1.76%  20 / 0.57% | 0.00479525474154 | 0.0291632652261 |
| [Gap junction](http://kobas.cbi.pku.edu.cn:80/program.run.do#Gap%20junction) | 8 / 2.01%  26 / 0.74% | 0.00633810882772 | 0.0372615485056 |
| [Translation factors](http://kobas.cbi.pku.edu.cn:80/program.run.do#Translation%20factors) | 12 / 3.02%  49 / 1.40% | 0.00712986216407 | 0.0405641053781 |
| [Oxidative phosphorylation](http://kobas.cbi.pku.edu.cn:80/program.run.do#Oxidative%20phosphorylation) | 21 / 5.28%  108 / 3.08% | 0.00861896693114 | 0.047503728503 |
| [Parkinson's disease](http://kobas.cbi.pku.edu.cn:80/program.run.do#Parkinson's%20disease) | 19 / 4.77%  98 / 2.80% | 0.0125556726452 | 0.066175362286 |
| [MAPK signaling pathway - yeast](http://kobas.cbi.pku.edu.cn:80/program.run.do#MAPK%20signaling%20pathway%20-%20yeast) | 4 / 1.01%  9 / 0.26% | 0.0129599918219 | 0.066175362286 |
| [Huntington's disease](http://kobas.cbi.pku.edu.cn:80/program.run.do#Huntington's%20disease) | 22 / 5.53%  119 / 3.40% | 0.0131323337011 | 0.066175362286 |
| [Long-term potentiation](http://kobas.cbi.pku.edu.cn:80/program.run.do#Long-term%20potentiation) | 7 / 1.76%  24 / 0.68% | 0.0143364643129 | 0.0702363637319 |
| [Adipocytokine signaling pathway](http://kobas.cbi.pku.edu.cn:80/program.run.do#Adipocytokine%20signaling%20pathway) | 6 / 1.51%  19 / 0.54% | 0.015429953619 | 0.0735504541301 |
| [Axon guidance](http://kobas.cbi.pku.edu.cn:80/program.run.do#Axon%20guidance) | 6 / 1.51%  20 / 0.57% | 0.0199876817093 | 0.0927686614792 |
| [Pancreatic cancer](http://kobas.cbi.pku.edu.cn:80/program.run.do#Pancreatic%20cancer) | 5 / 1.26%  16 / 0.46% | 0.0278517114175 | 0.12447259462 |
| [Olfactory transduction](http://kobas.cbi.pku.edu.cn:80/program.run.do#Olfactory%20transduction) | 4 / 1.01%  11 / 0.31% | 0.0282300255862 | 0.12447259462 |
| [Epithelial cell signaling in Helicobacter pylori infection](http://kobas.cbi.pku.edu.cn:80/program.run.do#Epithelial%20cell%20signaling%20in%20Helicobacter%20pylori%20infection) | 8 / 2.01%  34 / 0.97% | 0.0328172069814 | 0.141169287089 |
| [Bacterial secretion system](http://kobas.cbi.pku.edu.cn:80/program.run.do#Bacterial%20secretion%20system) | 2 / 0.50%  3 / 0.09% | 0.0357068304551 | 0.149942405213 |
| [Alzheimer's disease](http://kobas.cbi.pku.edu.cn:80/program.run.do#Alzheimer's%20disease) | 19 / 4.77%  113 / 3.22% | 0.049326732567 | 0.199137341557 |
| [Streptomycin biosynthesis](http://kobas.cbi.pku.edu.cn:80/program.run.do#Streptomycin%20biosynthesis) | 3 / 0.75%  8 / 0.23% | 0.0526842284287 | 0.199137341557 |
| [MAPK signaling pathway - fly](http://kobas.cbi.pku.edu.cn:80/program.run.do#MAPK%20signaling%20pathway%20-%20fly) | 3 / 0.75%  8 / 0.23% | 0.0526842284287 | 0.199137341557 |
| [Type II diabetes mellitus](http://kobas.cbi.pku.edu.cn:80/program.run.do#Type%20II%20diabetes%20mellitus) | 3 / 0.75%  8 / 0.23% | 0.0526842284287 | 0.199137341557 |
| [Ubiquitin system](http://kobas.cbi.pku.edu.cn:80/program.run.do#Ubiquitin%20system) | 37 / 9.30%  251 / 7.16% | 0.0530674355329 | 0.199137341557 |
| [Pyrimidine metabolism](http://kobas.cbi.pku.edu.cn:80/program.run.do#Pyrimidine%20metabolism) | 12 / 3.02%  65 / 1.86% | 0.0589072863596 | 0.216446337512 |
| [Phosphonate and phosphinate metabolism](http://kobas.cbi.pku.edu.cn:80/program.run.do#Phosphonate%20and%20phosphinate%20metabolism) | 2 / 0.50%  4 / 0.11% | 0.0660838668933 | 0.237860227416 |
| [Protein export](http://kobas.cbi.pku.edu.cn:80/program.run.do#Protein%20export) | 3 / 0.75%  9 / 0.26% | 0.0725463467321 | 0.251662561238 |
| [GnRH signaling pathway](http://kobas.cbi.pku.edu.cn:80/program.run.do#GnRH%20signaling%20pathway) | 7 / 1.76%  33 / 0.94% | 0.0727723377162 | 0.251662561238 |
| [T cell receptor signaling pathway](http://kobas.cbi.pku.edu.cn:80/program.run.do#T%20cell%20receptor%20signaling%20pathway) | 6 / 1.51%  27 / 0.77% | 0.0775683391799 | 0.253345500248 |
| [Amino sugar and nucleotide sugar metabolism](http://kobas.cbi.pku.edu.cn:80/program.run.do#Amino%20sugar%20and%20nucleotide%20sugar%20metabolism) | 6 / 1.51%  27 / 0.77% | 0.0775683391799 | 0.253345500248 |
| [Glycerophospholipid metabolism](http://kobas.cbi.pku.edu.cn:80/program.run.do#Glycerophospholipid%20metabolism) | 6 / 1.51%  27 / 0.77% | 0.0775683391799 | 0.253345500248 |
| [Protein folding and associated processing](http://kobas.cbi.pku.edu.cn:80/program.run.do#Protein%20folding%20and%20associated%20processing) | 4 / 1.01%  15 / 0.43% | 0.0812030877453 | 0.255744898079 |
| [Base excision repair](http://kobas.cbi.pku.edu.cn:80/program.run.do#Base%20excision%20repair) | 4 / 1.01%  15 / 0.43% | 0.0812030877453 | 0.255744898079 |
| [Endocytosis](http://kobas.cbi.pku.edu.cn:80/program.run.do#Endocytosis) | 12 / 3.02%  69 / 1.97% | 0.0854896625285 | 0.259960923536 |
| [Oocyte meiosis](http://kobas.cbi.pku.edu.cn:80/program.run.do#Oocyte%20meiosis) | 12 / 3.02%  69 / 1.97% | 0.0854896625285 | 0.259960923536 |
| [Colorectal cancer](http://kobas.cbi.pku.edu.cn:80/program.run.do#Colorectal%20cancer) | 6 / 1.51%  28 / 0.80% | 0.0897337247713 | 0.268241609152 |
| [Replication, recombination and repair proteins](http://kobas.cbi.pku.edu.cn:80/program.run.do#Replication,%20recombination%20and%20repair%20proteins) | 2 / 0.50%  5 / 0.14% | 0.102003989219 | 0.29743802777 |
| [Vascular smooth muscle contraction](http://kobas.cbi.pku.edu.cn:80/program.run.do#Vascular%20smooth%20muscle%20contraction) | 6 / 1.51%  29 / 0.83% | 0.102873582886 | 0.29743802777 |
| [VEGF signaling pathway](http://kobas.cbi.pku.edu.cn:80/program.run.do#VEGF%20signaling%20pathway) | 5 / 1.26%  23 / 0.66% | 0.111333706036 | 0.311679744027 |
| [RNA polymerase](http://kobas.cbi.pku.edu.cn:80/program.run.do#RNA%20polymerase) | 5 / 1.26%  23 / 0.66% | 0.111333706036 | 0.311679744027 |
| [Glycosphingolipid biosynthesis - globo series](http://kobas.cbi.pku.edu.cn:80/program.run.do#Glycosphingolipid%20biosynthesis%20-%20globo%20series) | 1 / 0.25%  1 / 0.03% | 0.113584474886 | 0.313012342444 |
| [Apoptosis](http://kobas.cbi.pku.edu.cn:80/program.run.do#Apoptosis) | 4 / 1.01%  17 / 0.49% | 0.118402212524 | 0.321269075322 |
| [GTP-binding proteins](http://kobas.cbi.pku.edu.cn:80/program.run.do#GTP-binding%20proteins) | 13 / 3.27%  81 / 2.31% | 0.123385667604 | 0.329718444076 |
| [Starch and sucrose metabolism](http://kobas.cbi.pku.edu.cn:80/program.run.do#Starch%20and%20sucrose%20metabolism) | 5 / 1.26%  24 / 0.68% | 0.128248517559 | 0.337598115022 |
| [PPAR signaling pathway](http://kobas.cbi.pku.edu.cn:80/program.run.do#PPAR%20signaling%20pathway) | 6 / 1.51%  31 / 0.88% | 0.131956022531 | 0.342249455389 |
| [MAPK signaling pathway](http://kobas.cbi.pku.edu.cn:80/program.run.do#MAPK%20signaling%20pathway) | 10 / 2.51%  60 / 1.71% | 0.136437148574 | 0.348743406765 |
| [Natural killer cell mediated cytotoxicity](http://kobas.cbi.pku.edu.cn:80/program.run.do#Natural%20killer%20cell%20mediated%20cytotoxicity) | 4 / 1.01%  18 / 0.51% | 0.139314122211 | 0.351010061072 |
| [Proteasome](http://kobas.cbi.pku.edu.cn:80/program.run.do#Proteasome) | 7 / 1.76%  39 / 1.11% | 0.146642084771 | 0.361390735293 |
| [Ether lipid metabolism](http://kobas.cbi.pku.edu.cn:80/program.run.do#Ether%20lipid%20metabolism) | 3 / 0.75%  12 / 0.34% | 0.147532278275 | 0.361390735293 |
| [RNA degradation](http://kobas.cbi.pku.edu.cn:80/program.run.do#RNA%20degradation) | 7 / 1.76%  40 / 1.14% | 0.161526985356 | 0.390251597606 |
| [Ubiquitin mediated proteolysis](http://kobas.cbi.pku.edu.cn:80/program.run.do#Ubiquitin%20mediated%20proteolysis) | 13 / 3.27%  86 / 2.45% | 0.171862604878 | 0.409603299608 |
| [Pathways in cancer](http://kobas.cbi.pku.edu.cn:80/program.run.do#Pathways%20in%20cancer) | 11 / 2.76%  71 / 2.03% | 0.176282019113 | 0.409603299608 |
| [Arachidonic acid metabolism](http://kobas.cbi.pku.edu.cn:80/program.run.do#Arachidonic%20acid%20metabolism) | 3 / 0.75%  13 / 0.37% | 0.176504010063 | 0.409603299608 |
| [3-Chloroacrylic acid degradation](http://kobas.cbi.pku.edu.cn:80/program.run.do#3-Chloroacrylic%20acid%20degradation) | 2 / 0.50%  7 / 0.20% | 0.184195526892 | 0.415435445566 |
| [Cysteine and methionine metabolism](http://kobas.cbi.pku.edu.cn:80/program.run.do#Cysteine%20and%20methionine%20metabolism) | 5 / 1.26%  27 / 0.77% | 0.185199498725 | 0.415435445566 |
| [Purine metabolism](http://kobas.cbi.pku.edu.cn:80/program.run.do#Purine%20metabolism) | 15 / 3.77%  103 / 2.94% | 0.186083633548 | 0.415435445566 |
| [Porphyrin and chlorophyll metabolism](http://kobas.cbi.pku.edu.cn:80/program.run.do#Porphyrin%20and%20chlorophyll%20metabolism) | 3 / 0.75%  14 / 0.40% | 0.206869215683 | 0.452039915934 |
| [Chronic myeloid leukemia](http://kobas.cbi.pku.edu.cn:80/program.run.do#Chronic%20myeloid%20leukemia) | 4 / 1.01%  21 / 0.60% | 0.209476598153 | 0.452039915934 |
| [Circadian rhythm - plant](http://kobas.cbi.pku.edu.cn:80/program.run.do#Circadian%20rhythm%20-%20plant) | 1 / 0.25%  2 / 0.06% | 0.214296258784 | 0.452039915934 |
| [Glycosphingolipid biosynthesis - ganglio series](http://kobas.cbi.pku.edu.cn:80/program.run.do#Glycosphingolipid%20biosynthesis%20-%20ganglio%20series) | 1 / 0.25%  2 / 0.06% | 0.214296258784 | 0.452039915934 |
| [Cell cycle](http://kobas.cbi.pku.edu.cn:80/program.run.do#Cell%20cycle) | 12 / 3.02%  82 / 2.34% | 0.215294834229 | 0.452039915934 |
| [Propanoate metabolism](http://kobas.cbi.pku.edu.cn:80/program.run.do#Propanoate%20metabolism) | 5 / 1.26%  29 / 0.83% | 0.227427791378 | 0.467650066117 |
| [Cytosolic DNA-sensing pathway](http://kobas.cbi.pku.edu.cn:80/program.run.do#Cytosolic%20DNA-sensing%20pathway) | 2 / 0.50%  8 / 0.23% | 0.22803262638 | 0.467650066117 |
| [Lipid biosynthesis proteins](http://kobas.cbi.pku.edu.cn:80/program.run.do#Lipid%20biosynthesis%20proteins) | 4 / 1.01%  22 / 0.63% | 0.234786444868 | 0.475966327284 |
| [Secretion system](http://kobas.cbi.pku.edu.cn:80/program.run.do#Secretion%20system) | 3 / 0.75%  15 / 0.43% | 0.238285747667 | 0.477570901215 |
| [Cellular antigens](http://kobas.cbi.pku.edu.cn:80/program.run.do#Cellular%20antigens) | 4 / 1.01%  23 / 0.66% | 0.260776495148 | 0.516774290598 |
| [RIG-I-like receptor signaling pathway](http://kobas.cbi.pku.edu.cn:80/program.run.do#RIG-I-like%20receptor%20signaling%20pathway) | 2 / 0.50%  9 / 0.26% | 0.272457277708 | 0.533922675516 |
| [Meiosis - yeast](http://kobas.cbi.pku.edu.cn:80/program.run.do#Meiosis%20-%20yeast) | 6 / 1.51%  40 / 1.14% | 0.29880579538 | 0.569617682357 |
| [Transcription related proteins](http://kobas.cbi.pku.edu.cn:80/program.run.do#Transcription%20related%20proteins) | 1 / 0.25%  3 / 0.09% | 0.303590972949 | 0.569617682357 |
| [1,2-Dichloroethane degradation](http://kobas.cbi.pku.edu.cn:80/program.run.do#1,2-Dichloroethane%20degradation) | 1 / 0.25%  3 / 0.09% | 0.303590972949 | 0.569617682357 |
| [Taste transduction](http://kobas.cbi.pku.edu.cn:80/program.run.do#Taste%20transduction) | 1 / 0.25%  3 / 0.09% | 0.303590972949 | 0.569617682357 |
| [General function prediction only](http://kobas.cbi.pku.edu.cn:80/program.run.do#General%20function%20prediction%20only) | 4 / 1.01%  26 / 0.74% | 0.341213080242 | 0.626869154002 |
| [Basal transcription factors](http://kobas.cbi.pku.edu.cn:80/program.run.do#Basal%20transcription%20factors) | 4 / 1.01%  26 / 0.74% | 0.341213080242 | 0.626869154002 |
| [Dorso-ventral axis formation](http://kobas.cbi.pku.edu.cn:80/program.run.do#Dorso-ventral%20axis%20formation) | 2 / 0.50%  11 / 0.31% | 0.360430610354 | 0.642109262504 |
| [Regulation of autophagy](http://kobas.cbi.pku.edu.cn:80/program.run.do#Regulation%20of%20autophagy) | 2 / 0.50%  11 / 0.31% | 0.360430610354 | 0.642109262504 |
| [Pentose and glucuronate interconversions](http://kobas.cbi.pku.edu.cn:80/program.run.do#Pentose%20and%20glucuronate%20interconversions) | 2 / 0.50%  11 / 0.31% | 0.360430610354 | 0.642109262504 |
| [Glioma](http://kobas.cbi.pku.edu.cn:80/program.run.do#Glioma) | 3 / 0.75%  19 / 0.54% | 0.368406473381 | 0.649755126528 |
| [Proteoglycans](http://kobas.cbi.pku.edu.cn:80/program.run.do#Proteoglycans) | 1 / 0.25%  4 / 0.11% | 0.3827600083 | 0.655408082275 |
| [Glycosaminoglycan degradation](http://kobas.cbi.pku.edu.cn:80/program.run.do#Glycosaminoglycan%20degradation) | 1 / 0.25%  4 / 0.11% | 0.3827600083 | 0.655408082275 |
| [Function unknown](http://kobas.cbi.pku.edu.cn:80/program.run.do#Function%20unknown) | 1 / 0.25%  4 / 0.11% | 0.3827600083 | 0.655408082275 |
| [Galactose metabolism](http://kobas.cbi.pku.edu.cn:80/program.run.do#Galactose%20metabolism) | 2 / 0.50%  12 / 0.34% | 0.40301027677 | 0.664285549507 |
| [Ascorbate and aldarate metabolism](http://kobas.cbi.pku.edu.cn:80/program.run.do#Ascorbate%20and%20aldarate%20metabolism) | 2 / 0.50%  12 / 0.34% | 0.40301027677 | 0.664285549507 |
| [Valine, leucine and isoleucine biosynthesis](http://kobas.cbi.pku.edu.cn:80/program.run.do#Valine,%20leucine%20and%20isoleucine%20biosynthesis) | 2 / 0.50%  12 / 0.34% | 0.40301027677 | 0.664285549507 |
| [Hedgehog signaling pathway](http://kobas.cbi.pku.edu.cn:80/program.run.do#Hedgehog%20signaling%20pathway) | 2 / 0.50%  12 / 0.34% | 0.40301027677 | 0.664285549507 |
| [Fc epsilon RI signaling pathway](http://kobas.cbi.pku.edu.cn:80/program.run.do#Fc%20epsilon%20RI%20signaling%20pathway) | 3 / 0.75%  21 / 0.60% | 0.432651778176 | 0.705783372501 |
| [Calcium signaling pathway](http://kobas.cbi.pku.edu.cn:80/program.run.do#Calcium%20signaling%20pathway) | 5 / 1.26%  38 / 1.08% | 0.436491685845 | 0.705783372501 |
| [Glycerolipid metabolism](http://kobas.cbi.pku.edu.cn:80/program.run.do#Glycerolipid%20metabolism) | 2 / 0.50%  13 / 0.37% | 0.444193234353 | 0.705783372501 |
| [Jak-STAT signaling pathway](http://kobas.cbi.pku.edu.cn:80/program.run.do#Jak-STAT%20signaling%20pathway) | 2 / 0.50%  13 / 0.37% | 0.444193234353 | 0.705783372501 |
| [Glycosylphosphatidylinositol(GPI)-anchor biosynthesis](http://kobas.cbi.pku.edu.cn:80/program.run.do" \l "Glycosylphosphatidylinositol(GPI)-anchor biosynthesis) | 1 / 0.25%  5 / 0.14% | 0.452949013071 | 0.706957548932 |
| [ECM-receptor interaction](http://kobas.cbi.pku.edu.cn:80/program.run.do#ECM-receptor%20interaction) | 1 / 0.25%  5 / 0.14% | 0.452949013071 | 0.706957548932 |
| [CAM ligands](http://kobas.cbi.pku.edu.cn:80/program.run.do#CAM%20ligands) | 1 / 0.25%  6 / 0.17% | 0.515174303953 | 0.790094092625 |
| [Pantothenate and CoA biosynthesis](http://kobas.cbi.pku.edu.cn:80/program.run.do#Pantothenate%20and%20CoA%20biosynthesis) | 1 / 0.25%  6 / 0.17% | 0.515174303953 | 0.790094092625 |
| [p53 signaling pathway](http://kobas.cbi.pku.edu.cn:80/program.run.do#p53%20signaling%20pathway) | 2 / 0.50%  15 / 0.43% | 0.521510543587 | 0.792745178341 |
| [Butanoate metabolism](http://kobas.cbi.pku.edu.cn:80/program.run.do#Butanoate%20metabolism) | 4 / 1.01%  33 / 0.94% | 0.525892538461 | 0.792745178341 |
| [Wnt signaling pathway](http://kobas.cbi.pku.edu.cn:80/program.run.do#Wnt%20signaling%20pathway) | 7 / 1.76%  60 / 1.71% | 0.530908398108 | 0.793523969855 |
| [Fructose and mannose metabolism](http://kobas.cbi.pku.edu.cn:80/program.run.do#Fructose%20and%20mannose%20metabolism) | 3 / 0.75%  25 / 0.71% | 0.552552997094 | 0.810554343774 |
| [Phosphatidylinositol signaling system](http://kobas.cbi.pku.edu.cn:80/program.run.do#Phosphatidylinositol%20signaling%20system) | 3 / 0.75%  25 / 0.71% | 0.552552997094 | 0.810554343774 |
| [Riboflavin metabolism](http://kobas.cbi.pku.edu.cn:80/program.run.do#Riboflavin%20metabolism) | 1 / 0.25%  7 / 0.20% | 0.570337433463 | 0.810554343774 |
| [NOD-like receptor signaling pathway](http://kobas.cbi.pku.edu.cn:80/program.run.do#NOD-like%20receptor%20signaling%20pathway) | 1 / 0.25%  7 / 0.20% | 0.570337433463 | 0.810554343774 |
| [DNA repair and recombination proteins](http://kobas.cbi.pku.edu.cn:80/program.run.do#DNA%20repair%20and%20recombination%20proteins) | 12 / 3.02%  108 / 3.08% | 0.579348933 | 0.810554343774 |
| [Melanogenesis](http://kobas.cbi.pku.edu.cn:80/program.run.do#Melanogenesis) | 3 / 0.75%  26 / 0.74% | 0.580119073205 | 0.810554343774 |
| [SNARE interactions in vesicular transport](http://kobas.cbi.pku.edu.cn:80/program.run.do#SNARE%20interactions%20in%20vesicular%20transport) | 3 / 0.75%  26 / 0.74% | 0.580119073205 | 0.810554343774 |
| [Fatty acid metabolism](http://kobas.cbi.pku.edu.cn:80/program.run.do#Fatty%20acid%20metabolism) | 6 / 1.51%  54 / 1.54% | 0.587652601533 | 0.810554343774 |
| [Cardiac muscle contraction](http://kobas.cbi.pku.edu.cn:80/program.run.do#Cardiac%20muscle%20contraction) | 3 / 0.75%  27 / 0.77% | 0.606590722727 | 0.810554343774 |
| [SNAREs](http://kobas.cbi.pku.edu.cn:80/program.run.do#SNAREs) | 3 / 0.75%  27 / 0.77% | 0.606590722727 | 0.810554343774 |
| [Terpenoid backbone biosynthesis](http://kobas.cbi.pku.edu.cn:80/program.run.do#Terpenoid%20backbone%20biosynthesis) | 1 / 0.25%  8 / 0.23% | 0.61923812019 | 0.810554343774 |
| [Heparan sulfate biosynthesis](http://kobas.cbi.pku.edu.cn:80/program.run.do#Heparan%20sulfate%20biosynthesis) | 1 / 0.25%  8 / 0.23% | 0.61923812019 | 0.810554343774 |
| [Glycan bindng proteins](http://kobas.cbi.pku.edu.cn:80/program.run.do#Glycan%20bindng%20proteins) | 1 / 0.25%  8 / 0.23% | 0.61923812019 | 0.810554343774 |
| [1- and 2-Methylnaphthalene degradation](http://kobas.cbi.pku.edu.cn:80/program.run.do#1-%20and%202-Methylnaphthalene%20degradation) | 1 / 0.25%  8 / 0.23% | 0.61923812019 | 0.810554343774 |
| [Signal transduction mechanisms](http://kobas.cbi.pku.edu.cn:80/program.run.do#Signal%20transduction%20mechanisms) | 4 / 1.01%  37 / 1.06% | 0.619697186006 | 0.810554343774 |
| [Mismatch repair](http://kobas.cbi.pku.edu.cn:80/program.run.do#Mismatch%20repair) | 2 / 0.50%  18 / 0.51% | 0.623238212528 | 0.810554343774 |
| [B cell receptor signaling pathway](http://kobas.cbi.pku.edu.cn:80/program.run.do#B%20cell%20receptor%20signaling%20pathway) | 2 / 0.50%  18 / 0.51% | 0.623238212528 | 0.810554343774 |
| [Peptidases](http://kobas.cbi.pku.edu.cn:80/program.run.do#Peptidases) | 16 / 4.02%  148 / 4.22% | 0.625026719931 | 0.810554343774 |
| [Lysosome](http://kobas.cbi.pku.edu.cn:80/program.run.do#Lysosome) | 7 / 1.76%  66 / 1.88% | 0.635726311326 | 0.818412174442 |
| [Notch signaling pathway](http://kobas.cbi.pku.edu.cn:80/program.run.do#Notch%20signaling%20pathway) | 2 / 0.50%  19 / 0.54% | 0.653218417134 | 0.828791760301 |
| [Histidine metabolism](http://kobas.cbi.pku.edu.cn:80/program.run.do#Histidine%20metabolism) | 1 / 0.25%  9 / 0.26% | 0.6625857255 | 0.828791760301 |
| [Other glycan degradation](http://kobas.cbi.pku.edu.cn:80/program.run.do#Other%20glycan%20degradation) | 1 / 0.25%  9 / 0.26% | 0.6625857255 | 0.828791760301 |
| [Two-component system](http://kobas.cbi.pku.edu.cn:80/program.run.do#Two-component%20system) | 1 / 0.25%  9 / 0.26% | 0.6625857255 | 0.828791760301 |
| [N-Glycan biosynthesis](http://kobas.cbi.pku.edu.cn:80/program.run.do#N-Glycan%20biosynthesis) | 3 / 0.75%  30 / 0.86% | 0.679150052026 | 0.834407389339 |
| [Drug metabolism - other enzymes](http://kobas.cbi.pku.edu.cn:80/program.run.do#Drug%20metabolism%20-%20other%20enzymes) | 3 / 0.75%  30 / 0.86% | 0.679150052026 | 0.834407389339 |
| [beta-Alanine metabolism](http://kobas.cbi.pku.edu.cn:80/program.run.do#beta-Alanine%20metabolism) | 2 / 0.50%  20 / 0.57% | 0.681268284638 | 0.834407389339 |
| [Antigen processing and presentation](http://kobas.cbi.pku.edu.cn:80/program.run.do#Antigen%20processing%20and%20presentation) | 2 / 0.50%  21 / 0.60% | 0.707438443212 | 0.849711741682 |
| [Amyotrophic lateral sclerosis (ALS)](http://kobas.cbi.pku.edu.cn:80/program.run.do" \l "Amyotrophic lateral sclerosis (ALS)) | 2 / 0.50%  21 / 0.60% | 0.707438443212 | 0.849711741682 |
| [Chromosome](http://kobas.cbi.pku.edu.cn:80/program.run.do#Chromosome) | 29 / 7.29%  276 / 7.88% | 0.708217236377 | 0.849711741682 |
| [Amino acid metabolism](http://kobas.cbi.pku.edu.cn:80/program.run.do#Amino%20acid%20metabolism) | 1 / 0.25%  11 / 0.31% | 0.735067322673 | 0.870088262966 |
| [Prion diseases](http://kobas.cbi.pku.edu.cn:80/program.run.do#Prion%20diseases) | 1 / 0.25%  11 / 0.31% | 0.735067322673 | 0.870088262966 |
| [DNA replication](http://kobas.cbi.pku.edu.cn:80/program.run.do#DNA%20replication) | 3 / 0.75%  33 / 0.94% | 0.741319111803 | 0.871638490073 |
| [Caprolactam degradation](http://kobas.cbi.pku.edu.cn:80/program.run.do#Caprolactam%20degradation) | 1 / 0.25%  12 / 0.34% | 0.765254326846 | 0.888086305952 |
| [Glycosyltransferases](http://kobas.cbi.pku.edu.cn:80/program.run.do#Glycosyltransferases) | 5 / 1.26%  55 / 1.57% | 0.765378572061 | 0.888086305952 |
| [Glutathione metabolism](http://kobas.cbi.pku.edu.cn:80/program.run.do#Glutathione%20metabolism) | 3 / 0.75%  35 / 1.00% | 0.777189800188 | 0.895897084989 |
| [Retinol metabolism](http://kobas.cbi.pku.edu.cn:80/program.run.do#Retinol%20metabolism) | 1 / 0.25%  13 / 0.37% | 0.792009417887 | 0.904296323958 |
| [Transporters](http://kobas.cbi.pku.edu.cn:80/program.run.do#Transporters) | 2 / 0.50%  25 / 0.71% | 0.794730721312 | 0.904296323958 |
| [Non-small cell lung cancer](http://kobas.cbi.pku.edu.cn:80/program.run.do#Non-small%20cell%20lung%20cancer) | 1 / 0.25%  14 / 0.40% | 0.815721893304 | 0.908418199659 |
| [Acute myeloid leukemia](http://kobas.cbi.pku.edu.cn:80/program.run.do#Acute%20myeloid%20leukemia) | 1 / 0.25%  14 / 0.40% | 0.815721893304 | 0.908418199659 |
| [Small cell lung cancer](http://kobas.cbi.pku.edu.cn:80/program.run.do#Small%20cell%20lung%20cancer) | 1 / 0.25%  14 / 0.40% | 0.815721893304 | 0.908418199659 |
| [Lysine degradation](http://kobas.cbi.pku.edu.cn:80/program.run.do#Lysine%20degradation) | 3 / 0.75%  38 / 1.08% | 0.82324568771 | 0.908418199659 |
| [Translation proteins](http://kobas.cbi.pku.edu.cn:80/program.run.do#Translation%20proteins) | 2 / 0.50%  27 / 0.77% | 0.829096969529 | 0.908418199659 |
| [TGF-beta signaling pathway](http://kobas.cbi.pku.edu.cn:80/program.run.do#TGF-beta%20signaling%20pathway) | 3 / 0.75%  39 / 1.11% | 0.836683521409 | 0.908418199659 |
| [Toll-like receptor signaling pathway](http://kobas.cbi.pku.edu.cn:80/program.run.do#Toll-like%20receptor%20signaling%20pathway) | 1 / 0.25%  15 / 0.43% | 0.836736989712 | 0.908418199659 |
| [DNA replication proteins](http://kobas.cbi.pku.edu.cn:80/program.run.do#DNA%20replication%20proteins) | 7 / 1.76%  82 / 2.34% | 0.839558511436 | 0.908418199659 |
| [Homologous recombination](http://kobas.cbi.pku.edu.cn:80/program.run.do#Homologous%20recombination) | 1 / 0.25%  16 / 0.46% | 0.85536085847 | 0.919873244104 |
| [ErbB signaling pathway](http://kobas.cbi.pku.edu.cn:80/program.run.do#ErbB%20signaling%20pathway) | 2 / 0.50%  30 / 0.86% | 0.870989177078 | 0.931003419925 |
| [Progesterone-mediated oocyte maturation](http://kobas.cbi.pku.edu.cn:80/program.run.do#Progesterone-mediated%20oocyte%20maturation) | 3 / 0.75%  43 / 1.23% | 0.881976791606 | 0.932073089941 |
| [Inositol phosphate metabolism](http://kobas.cbi.pku.edu.cn:80/program.run.do#Inositol%20phosphate%20metabolism) | 1 / 0.25%  18 / 0.51% | 0.886490084707 | 0.932073089941 |
| [Long-term depression](http://kobas.cbi.pku.edu.cn:80/program.run.do#Long-term%20depression) | 1 / 0.25%  19 / 0.54% | 0.899449621794 | 0.932073089941 |
| [Nitrogen metabolism](http://kobas.cbi.pku.edu.cn:80/program.run.do#Nitrogen%20metabolism) | 1 / 0.25%  20 / 0.57% | 0.910932850066 | 0.932073089941 |
| [Aminoacyl-tRNA biosynthesis](http://kobas.cbi.pku.edu.cn:80/program.run.do#Aminoacyl-tRNA%20biosynthesis) | 2 / 0.50%  35 / 1.00% | 0.920407903378 | 0.932073089941 |
| [Endometrial cancer](http://kobas.cbi.pku.edu.cn:80/program.run.do#Endometrial%20cancer) | 1 / 0.25%  21 / 0.60% | 0.921107570408 | 0.932073089941 |
| [Sphingolipid metabolism](http://kobas.cbi.pku.edu.cn:80/program.run.do#Sphingolipid%20metabolism) | 1 / 0.25%  21 / 0.60% | 0.921107570408 | 0.932073089941 |
| [Tyrosine metabolism](http://kobas.cbi.pku.edu.cn:80/program.run.do#Tyrosine%20metabolism) | 1 / 0.25%  21 / 0.60% | 0.921107570408 | 0.932073089941 |
| [Selenoamino acid metabolism](http://kobas.cbi.pku.edu.cn:80/program.run.do#Selenoamino%20acid%20metabolism) | 1 / 0.25%  21 / 0.60% | 0.921107570408 | 0.932073089941 |
| [Nucleotide excision repair](http://kobas.cbi.pku.edu.cn:80/program.run.do#Nucleotide%20excision%20repair) | 2 / 0.50%  36 / 1.03% | 0.927870797094 | 0.932073089941 |
| [Prostate cancer](http://kobas.cbi.pku.edu.cn:80/program.run.do#Prostate%20cancer) | 1 / 0.25%  22 / 0.63% | 0.93012255375 | 0.932073089941 |
| [Limonene and pinene degradation](http://kobas.cbi.pku.edu.cn:80/program.run.do#Limonene%20and%20pinene%20degradation) | 1 / 0.25%  24 / 0.68% | 0.945185920056 | 0.938133431349 |
| [Metabolism of xenobiotics by cytochrome P450](http://kobas.cbi.pku.edu.cn:80/program.run.do#Metabolism%20of%20xenobiotics%20by%20cytochrome%20P450) | 1 / 0.25%  25 / 0.71% | 0.95145488667 | 0.938133431349 |
| [Cell cycle - yeast](http://kobas.cbi.pku.edu.cn:80/program.run.do#Cell%20cycle%20-%20yeast) | 4 / 1.01%  70 / 2.00% | 0.965685567968 | 0.938133431349 |
| [Drug metabolism - cytochrome P450](http://kobas.cbi.pku.edu.cn:80/program.run.do#Drug%20metabolism%20-%20cytochrome%20P450) | 1 / 0.25%  28 / 0.80% | 0.966286105309 | 0.938133431349 |
| [Others](http://kobas.cbi.pku.edu.cn:80/program.run.do#Others) | 5 / 1.26%  84 / 2.40% | 0.969897045769 | 0.938133431349 |
| [Systemic lupus erythematosus](http://kobas.cbi.pku.edu.cn:80/program.run.do#Systemic%20lupus%20erythematosus) | 4 / 1.01%  79 / 2.25% | 0.984211107108 | 0.938133431349 |
| [Arginine and proline metabolism](http://kobas.cbi.pku.edu.cn:80/program.run.do#Arginine%20and%20proline%20metabolism) | 1 / 0.25%  36 / 1.03% | 0.987268878549 | 0.938133431349 |
| [Tryptophan metabolism](http://kobas.cbi.pku.edu.cn:80/program.run.do#Tryptophan%20metabolism) | 1 / 0.25%  37 / 1.06% | 0.988729947274 | 0.938133431349 |
| [Valine, leucine and isoleucine degradation](http://kobas.cbi.pku.edu.cn:80/program.run.do#Valine,%20leucine%20and%20isoleucine%20degradation) | 1 / 0.25%  43 / 1.23% | 0.994580736346 | 0.938133431349 |
| [Protein kinases](http://kobas.cbi.pku.edu.cn:80/program.run.do#Protein%20kinases) | 7 / 1.76%  133 / 3.80% | 0.995615795163 | 0.938133431349 |
| [Transcription factors](http://kobas.cbi.pku.edu.cn:80/program.run.do#Transcription%20factors) | 5 / 1.26%  283 / 8.08% | 0.999999999964 | 0.938133431349 |
| [Receptors and channels](http://kobas.cbi.pku.edu.cn:80/program.run.do#Receptors%20and%20channels) | 1 / 0.25%  853 / 24.34% | 1.0 | 0.938133431349 |
