# Supplementary material for: Analyses of an Expressed Sequence Tag Library from Taenia solium, Cysticerca
Source: PLoS Negl Trop Dis. 2010 Dec 21;4(12):e919. doi: 10.1371/journal.pntd.0000919 (PMC3006133; doi:10.1371/journal.pntd.0000919)
Supplement: Table S5 — Result from BLAST search with a score <10−5 of 1650 unique ESTs towards Taenia solium total ESTs at NCBI. (0.93 MB DOC) [file pntd.0000919.s007.doc]

BLASTN 2.2.23+

Reference: Zheng Zhang, Scott Schwartz, Lukas Wagner, and

Webb Miller (2000), "A greedy algorithm for aligning DNA

sequences", J Comput Biol 2000; 7(1-2):203-14.

RID: 3708ENFS01R

Database: GenBank non-mouse and non-human EST entries

53,100,827 sequences; 30,110,148,220 total letters

Query= TS.seq.screen.trim.Contig3

Length=353

Score E

Sequences producing significant alignments: (Bits) Value

gb|EL741854.1| LV0232059 Taenia solium UNAM-cd2_larva Taenia ... 56.5 3e-08

Query= TS.seq.screen.trim.Contig4

Length=486

Score E

Sequences producing significant alignments: (Bits) Value

gb|EL744461.1| LV0273019 Taenia solium UNAM-cd2_larva Taenia ... 728 0.0

gb|EL743670.1| LV0260058 Taenia solium UNAM-cd2_larva Taenia ... 728 0.0

gb|EL742432.1| LV0241058 Taenia solium UNAM-cd2_larva Taenia ... 728 0.0

gb|EL741914.1| LV0233047 Taenia solium UNAM-cd2_larva Taenia ... 728 0.0

gb|EL747602.1| LV0326018 Taenia solium UNAM-cd2_larva Taenia ... 702 0.0

gb|EL745580.1| LV0293058 Taenia solium UNAM-cd2_larva Taenia ... 298 5e-81

Query= TS.seq.screen.trim.Contig5

Length=512

Score E

Sequences producing significant alignments: (Bits) Value

gb|EL749350.1| LV0359028 Taenia solium UNAM-cd2_larva Taenia ... 76.8 3e-14

Query= TS.seq.screen.trim.Contig6

Length=494

Score E

Sequences producing significant alignments: (Bits) Value

gb|EL740648.1| LV0213030 Taenia solium UNAM-cd2_larva Taenia ... 340 9e-94

Query= TS.seq.screen.trim.Contig8

Length=274

Score E

Sequences producing significant alignments: (Bits) Value

gb|EL759117.1| AD0117020 Taenia solium UNAM-cd1_adult Taenia ... 126 3e-29

gb|EL758888.1| AD0113029 Taenia solium UNAM-cd1_adult Taenia ... 126 3e-29

gb|EL741803.1| LV0232008 Taenia solium UNAM-cd2_larva Taenia ... 69.4 4e-12

Query= TS.seq.screen.trim.Contig9

Length=417

Score E

Sequences producing significant alignments: (Bits) Value

gb|EL749331.1| LV0359009 Taenia solium UNAM-cd2_larva Taenia ... 200 2e-51

gb|EL743364.1| LV0256025 Taenia solium UNAM-cd2_larva Taenia ... 183 2e-46

gb|EL741854.1| LV0232059 Taenia solium UNAM-cd2_larva Taenia ... 56.5 3e-08

Query= TS.seq.screen.trim.Contig17

Length=360

Score E

Sequences producing significant alignments: (Bits) Value

gb|EL744392.1| LV0272007 Taenia solium UNAM-cd2_larva Taenia ... 361 7e-100

gb|EL742596.1| LV0244027 Taenia solium UNAM-cd2_larva Taenia ... 359 2e-99

gb|EL753067.1| AD0016105 Taenia solium UNAM-cd1_adult Taenia ... 357 8e-99

gb|EL747569.1| LV0325048 Taenia solium UNAM-cd2_larva Taenia ... 357 8e-99

gb|EL746523.1| LV0310020 Taenia solium UNAM-cd2_larva Taenia ... 357 8e-99

gb|EL744844.1| LV0281004 Taenia solium UNAM-cd2_larva Taenia ... 357 8e-99

gb|EL758248.1| AD0102059 Taenia solium UNAM-cd1_adult Taenia ... 355 3e-98

gb|EL758206.1| AD0102017 Taenia solium UNAM-cd1_adult Taenia ... 355 3e-98

gb|EL749253.1| LV0356031 Taenia solium UNAM-cd2_larva Taenia ... 355 3e-98

gb|EL749222.1| LV0355036 Taenia solium UNAM-cd2_larva Taenia ... 355 3e-98

Query= TS.seq.screen.trim.Contig19

Length=459

Score E

Sequences producing significant alignments: (Bits) Value

gb|EL759795.1| AD0129035 Taenia solium UNAM-cd1_adult Taenia ... 684 0.0

gb|EL757781.1| AD0095056 Taenia solium UNAM-cd1_adult Taenia ... 669 0.0

gb|EL751163.1| AD0008174 Taenia solium UNAM-cd1_adult Taenia ... 654 0.0

Query= TS.seq.screen.trim.Contig20

Length=240

Score E

Sequences producing significant alignments: (Bits) Value

gb|EL745806.1| LV0297035 Taenia solium UNAM-cd2_larva Taenia ... 326 2e-89

gb|EL743628.1| LV0260016 Taenia solium UNAM-cd2_larva Taenia ... 324 9e-89

gb|EL747793.1| LV0329031 Taenia solium UNAM-cd2_larva Taenia ... 322 3e-88

gb|EL744601.1| LV0276019 Taenia solium UNAM-cd2_larva Taenia ... 322 3e-88

gb|EL743276.1| LV0254057 Taenia solium UNAM-cd2_larva Taenia ... 322 3e-88

gb|EL742730.1| LV0246013 Taenia solium UNAM-cd2_larva Taenia ... 322 3e-88

gb|EL740289.1| LV0206021 Taenia solium UNAM-cd2_larva Taenia ... 322 3e-88

gb|EL745863.1| LV0298020 Taenia solium UNAM-cd2_larva Taenia ... 318 4e-87

gb|EL750719.1| AD0006158 Taenia solium UNAM-cd1_adult Taenia ... 316 1e-86

gb|EL759467.1| AD0123046 Taenia solium UNAM-cd1_adult Taenia ... 311 7e-85

Query= TS.seq.screen.trim.Contig21

Length=298

Score E

Sequences producing significant alignments: (Bits) Value

gb|EL746964.1| LV0316041 Taenia solium UNAM-cd2_larva Taenia ... 377 7e-105

Query= TS.seq.screen.trim.Contig22

Length=230

Score E

Sequences producing significant alignments: (Bits) Value

gb|GT227395.1| tscaa0_001093.z1.scf Taenia solium adult full-... 364 5e-101

Query= TS.seq.screen.trim.Contig24

Length=351

Score E

Sequences producing significant alignments: (Bits) Value

gb|EL741516.1| LV0227072 Taenia solium UNAM-cd2_larva Taenia ... 324 9e-89

Query= TS.seq.screen.trim.Contig25

Length=549

Score E

Sequences producing significant alignments: (Bits) Value

gb|EL743219.1| LV0253066 Taenia solium UNAM-cd2_larva Taenia ... 270 1e-72

Query= TS.seq.screen.trim.Contig28

Length=295

Score E

Sequences producing significant alignments: (Bits) Value

gb|EL746823.1| LV0314036 Taenia solium UNAM-cd2_larva Taenia ... 414 5e-116

gb|EL745626.1| LV0294041 Taenia solium UNAM-cd2_larva Taenia ... 374 8e-104

gb|EL746648.1| LV0312009 Taenia solium UNAM-cd2_larva Taenia ... 274 9e-74

Query= TS.seq.screen.trim.Contig30

Length=271

Score E

Sequences producing significant alignments: (Bits) Value

gb|EL740322.1| LV0207006 Taenia solium UNAM-cd2_larva Taenia ... 241 9e-64

gb|EL740570.1| LV0212006 Taenia solium UNAM-cd2_larva Taenia ... 213 2e-55

gb|EL748656.1| LV0343041 Taenia solium UNAM-cd2_larva Taenia ... 174 9e-44

gb|EL744879.1| LV0282005 Taenia solium UNAM-cd2_larva Taenia ... 167 2e-41

gb|EL761850.1| AD0172017 Taenia solium UNAM-cd1_adult Taenia ... 165 6e-41

gb|EL744330.1| LV0271012 Taenia solium UNAM-cd2_larva Taenia ... 165 6e-41

gb|EL763301.1| AD0202001 Taenia solium UNAM-cd1_adult Taenia ... 163 2e-40

gb|EL748734.1| LV0344057 Taenia solium UNAM-cd2_larva Taenia ... 163 2e-40

gb|EL744495.1| LV0273053 Taenia solium UNAM-cd2_larva Taenia ... 163 2e-40

gb|EL743779.1| LV0262020 Taenia solium UNAM-cd2_larva Taenia ... 163 2e-40

Query= TS.seq.screen.trim.Contig31

Length=392

Score E

Sequences producing significant alignments: (Bits) Value

gb|EL748097.1| LV0333048 Taenia solium UNAM-cd2_larva Taenia ... 660 0.0

gb|EL749603.1| AD0001226 Taenia solium UNAM-cd1_adult Taenia ... 636 0.0

gb|EL757298.1| AD0034114 Taenia solium UNAM-cd1_adult Taenia ... 625 2e-179

gb|EL763347.1| AD0202047 Taenia solium UNAM-cd1_adult Taenia ... 610 6e-175

gb|EL763042.1| AD0197051 Taenia solium UNAM-cd1_adult Taenia ... 610 6e-175

gb|EL755634.1| AD0026157 Taenia solium UNAM-cd1_adult Taenia ... 604 3e-173

gb|EL757969.1| AD0098054 Taenia solium UNAM-cd1_adult Taenia ... 601 4e-172

gb|EL761113.1| AD0157009 Taenia solium UNAM-cd1_adult Taenia ... 560 6e-160

Query= TS.seq.screen.trim.Contig32

Length=408

Score E

Sequences producing significant alignments: (Bits) Value

gb|EL746287.1| LV0307001 Taenia solium UNAM-cd2_larva Taenia ... 675 0.0

gb|EL740984.1| LV0219034 Taenia solium UNAM-cd2_larva Taenia ... 675 0.0

gb|EL745611.1| LV0294026 Taenia solium UNAM-cd2_larva Taenia ... 671 0.0

gb|EL748642.1| LV0343027 Taenia solium UNAM-cd2_larva Taenia ... 669 0.0

gb|EL748529.1| LV0341045 Taenia solium UNAM-cd2_larva Taenia ... 660 0.0

gb|EL751385.1| AD0009123 Taenia solium UNAM-cd1_adult Taenia ... 656 0.0

gb|EL742380.1| LV0241006 Taenia solium UNAM-cd2_larva Taenia ... 654 0.0

gb|EL761249.1| AD0159046 Taenia solium UNAM-cd1_adult Taenia ... 647 0.0

gb|EL752363.1| AD0013130 Taenia solium UNAM-cd1_adult Taenia ... 645 0.0

gb|EL750856.1| AD0007092 Taenia solium UNAM-cd1_adult Taenia ... 645 0.0

Query= TS.seq.screen.trim.Contig33

Length=344

Score E

Sequences producing significant alignments: (Bits) Value

gb|EL740723.1| LV0215013 Taenia solium UNAM-cd2_larva Taenia ... 420 1e-117

gb|EL762052.1| AD0177023 Taenia solium UNAM-cd1_adult Taenia ... 355 3e-98

gb|EX151213.1| TPEG001001H09 Cysti-host Taenia solium cDNA, m... 315 5e-86

gb|EX151282.1| TPEG001002G05 Cysti-host Taenia solium cDNA, m... 135 4e-32

Query= TS.seq.screen.trim.Contig34

Length=281

Score E

Sequences producing significant alignments: (Bits) Value

gb|EL750539.1| AD0005202 Taenia solium UNAM-cd1_adult Taenia ... 457 8e-129

gb|EL747846.1| LV0330011 Taenia solium UNAM-cd2_larva Taenia ... 457 8e-129

gb|EL747528.1| LV0325007 Taenia solium UNAM-cd2_larva Taenia ... 457 8e-129

gb|EL747161.1| LV0319020 Taenia solium UNAM-cd2_larva Taenia ... 457 8e-129

gb|EL746120.1| LV0302015 Taenia solium UNAM-cd2_larva Taenia ... 457 8e-129

gb|EL744157.1| LV0268024 Taenia solium UNAM-cd2_larva Taenia ... 457 8e-129

gb|EL743630.1| LV0260018 Taenia solium UNAM-cd2_larva Taenia ... 457 8e-129

gb|EL743103.1| LV0252004 Taenia solium UNAM-cd2_larva Taenia ... 457 8e-129

gb|EL741491.1| LV0227047 Taenia solium UNAM-cd2_larva Taenia ... 457 8e-129

gb|EL741002.1| LV0219052 Taenia solium UNAM-cd2_larva Taenia ... 457 8e-129

Query= TS.seq.screen.trim.Contig35

Length=426

Score E

Sequences producing significant alignments: (Bits) Value

gb|EL749007.1| LV0350016 Taenia solium UNAM-cd2_larva Taenia ... 736 0.0

gb|EL743844.1| LV0263013 Taenia solium UNAM-cd2_larva Taenia ... 736 0.0

gb|EL753928.1| AD0019215 Taenia solium UNAM-cd1_adult Taenia ... 732 0.0

gb|EL755637.1| AD0026160 Taenia solium UNAM-cd1_adult Taenia ... 730 0.0

gb|EL754384.1| AD0021174 Taenia solium UNAM-cd1_adult Taenia ... 730 0.0

gb|EL746720.1| LV0313003 Taenia solium UNAM-cd2_larva Taenia ... 723 0.0

gb|EL751513.1| AD0010006 Taenia solium UNAM-cd1_adult Taenia ... 719 0.0

gb|EL762162.1| AD0180004 Taenia solium UNAM-cd1_adult Taenia ... 713 0.0

gb|EL749883.1| AD0003025 Taenia solium UNAM-cd1_adult Taenia ... 695 0.0

gb|EL749204.1| LV0355018 Taenia solium UNAM-cd2_larva Taenia ... 695 0.0

Query= TS.seq.screen.trim.Contig36

Length=350

Score E

Sequences producing significant alignments: (Bits) Value

gb|EL747944.1| LV0331037 Taenia solium UNAM-cd2_larva Taenia ... 93.5 3e-19

Query= TS.seq.screen.trim.Contig38

Length=559

Score E

Sequences producing significant alignments: (Bits) Value

gb|EL760926.1| AD0152051 Taenia solium UNAM-cd1_adult Taenia ... 941 0.0

gb|EL760697.1| AD0147029 Taenia solium UNAM-cd1_adult Taenia ... 941 0.0

gb|EL758168.1| AD0101048 Taenia solium UNAM-cd1_adult Taenia ... 941 0.0

gb|EL757617.1| AD0035210 Taenia solium UNAM-cd1_adult Taenia ... 941 0.0

gb|EL756846.1| AD0031119 Taenia solium UNAM-cd1_adult Taenia ... 941 0.0

gb|EL756496.1| AD0030028 Taenia solium UNAM-cd1_adult Taenia ... 941 0.0

gb|EL756333.1| AD0029128 Taenia solium UNAM-cd1_adult Taenia ... 941 0.0

gb|EL756302.1| AD0029097 Taenia solium UNAM-cd1_adult Taenia ... 941 0.0

gb|EL755733.1| AD0027029 Taenia solium UNAM-cd1_adult Taenia ... 941 0.0

gb|EL754015.1| AD0020060 Taenia solium UNAM-cd1_adult Taenia ... 941 0.0

Query= TS.seq.screen.trim.Contig40

Length=426

Score E

Sequences producing significant alignments: (Bits) Value

gb|EL749034.1| LV0351022 Taenia solium UNAM-cd2_larva Taenia ... 159 3e-39

Query= TS.seq.screen.trim.Contig41

Length=464

Score E

Sequences producing significant alignments: (Bits) Value

gb|EL758825.1| AD0112024 Taenia solium UNAM-cd1_adult Taenia ... 815 0.0

gb|EL763026.1| AD0197035 Taenia solium UNAM-cd1_adult Taenia ... 809 0.0

gb|GT227052.1| tscaa0_001458.z1.scf Taenia solium adult full-... 787 0.0

gb|EL747796.1| LV0329034 Taenia solium UNAM-cd2_larva Taenia ... 252 4e-67

Query= TS.seq.screen.trim.Contig43

Length=498

Score E

Sequences producing significant alignments: (Bits) Value

gb|EL758825.1| AD0112024 Taenia solium UNAM-cd1_adult Taenia ... 684 0.0

gb|EL747796.1| LV0329034 Taenia solium UNAM-cd2_larva Taenia ... 619 1e-177

gb|EL763026.1| AD0197035 Taenia solium UNAM-cd1_adult Taenia ... 604 3e-173

gb|GT227052.1| tscaa0_001458.z1.scf Taenia solium adult full-... 340 9e-94

gb|EL747994.1| LV0332016 Taenia solium UNAM-cd2_larva Taenia ... 259 2e-69

gb|EL742881.1| LV0248043 Taenia solium UNAM-cd2_larva Taenia ... 243 2e-64

gb|EL742752.1| LV0246035 Taenia solium UNAM-cd2_larva Taenia ... 119 4e-27

gb|EL741105.1| LV0221006 Taenia solium UNAM-cd2_larva Taenia ... 113 2e-25

Query= TS.seq.screen.trim.Contig47

Length=337

Score E

Sequences producing significant alignments: (Bits) Value

gb|EL743911.1| LV0264040 Taenia solium UNAM-cd2_larva Taenia ... 555 3e-158

gb|GT227629.1| tscaa0_001811.z1.scf Taenia solium adult full-... 536 1e-152

gb|EL740514.1| LV0210038 Taenia solium UNAM-cd2_larva Taenia ... 534 4e-152

gb|EL760824.1| AD0149040 Taenia solium UNAM-cd1_adult Taenia ... 490 8e-139

gb|EL759027.1| AD0115052 Taenia solium UNAM-cd1_adult Taenia ... 444 6e-125

gb|EL741854.1| LV0232059 Taenia solium UNAM-cd2_larva Taenia ... 58.4 1e-08

Query= TS.seq.screen.trim.Contig48

Length=326

Score E

Sequences producing significant alignments: (Bits) Value

gb|EL744398.1| LV0272013 Taenia solium UNAM-cd2_larva Taenia ... 560 6e-160

gb|EL740371.1| LV0207055 Taenia solium UNAM-cd2_larva Taenia ... 542 2e-154

gb|EL744320.1| LV0271002 Taenia solium UNAM-cd2_larva Taenia ... 353 1e-97

gb|EL758859.1| AD0112058 Taenia solium UNAM-cd1_adult Taenia ... 344 7e-95

gb|EL758465.1| AD0106008 Taenia solium UNAM-cd1_adult Taenia ... 339 3e-93

gb|EL758117.1| AD0100062 Taenia solium UNAM-cd1_adult Taenia ... 333 1e-91

gb|GT227236.1| tscaa0_000541.z1.scf Taenia solium adult full-... 329 2e-90

gb|EX150988.1| TSEDTS1028B09 Cysti Taenia solium cDNA, mRNA s... 296 2e-80

gb|EL743278.1| LV0254059 Taenia solium UNAM-cd2_larva Taenia ... 89.8 3e-18

gb|EL749400.1| AD0001023 Taenia solium UNAM-cd1_adult Taenia ... 82.4 6e-16

Query= TS.seq.screen.trim.Contig49

Length=124

Score E

Sequences producing significant alignments: (Bits) Value

gb|EL748446.1| LV0340020 Taenia solium UNAM-cd2_larva Taenia ... 207 9e-54

gb|EL762205.1| AD0181002 Taenia solium UNAM-cd1_adult Taenia ... 182 5e-46

gb|EL761222.1| AD0159019 Taenia solium UNAM-cd1_adult Taenia ... 182 5e-46

gb|EL749283.1| LV0357027 Taenia solium UNAM-cd2_larva Taenia ... 182 5e-46

gb|EL744499.1| LV0274002 Taenia solium UNAM-cd2_larva Taenia ... 182 5e-46

gb|EL742278.1| LV0239030 Taenia solium UNAM-cd2_larva Taenia ... 176 3e-44

gb|EL742156.1| LV0237044 Taenia solium UNAM-cd2_larva Taenia ... 167 2e-41

gb|EL748988.1| LV0349037 Taenia solium UNAM-cd2_larva Taenia ... 165 6e-41

gb|EL748989.1| LV0349038 Taenia solium UNAM-cd2_larva Taenia ... 161 7e-40

gb|EL740876.1| LV0217059 Taenia solium UNAM-cd2_larva Taenia ... 161 7e-40

Query= TS.seq.screen.trim.Contig50

Length=476

Score E

Sequences producing significant alignments: (Bits) Value

gb|EL749331.1| LV0359009 Taenia solium UNAM-cd2_larva Taenia ... 521 3e-148

gb|EL743364.1| LV0256025 Taenia solium UNAM-cd2_larva Taenia ... 207 9e-54

Query= TS.seq.screen.trim.Contig52

Length=516

Score E

Sequences producing significant alignments: (Bits) Value

gb|EL746886.1| LV0315028 Taenia solium UNAM-cd2_larva Taenia ... 857 0.0

gb|EL750719.1| AD0006158 Taenia solium UNAM-cd1_adult Taenia ... 411 6e-115

gb|EL747793.1| LV0329031 Taenia solium UNAM-cd2_larva Taenia ... 411 6e-115

gb|EL744601.1| LV0276019 Taenia solium UNAM-cd2_larva Taenia ... 411 6e-115

gb|EL743628.1| LV0260016 Taenia solium UNAM-cd2_larva Taenia ... 411 6e-115

gb|EL740289.1| LV0206021 Taenia solium UNAM-cd2_larva Taenia ... 411 6e-115

gb|EL759467.1| AD0123046 Taenia solium UNAM-cd1_adult Taenia ... 405 3e-113

gb|EL758034.1| AD0099046 Taenia solium UNAM-cd1_adult Taenia ... 405 3e-113

gb|EL745806.1| LV0297035 Taenia solium UNAM-cd2_larva Taenia ... 403 1e-112

gb|EL745863.1| LV0298020 Taenia solium UNAM-cd2_larva Taenia ... 399 1e-111

Query= TS.seq.screen.trim.Contig53

Length=335

Score E

Sequences producing significant alignments: (Bits) Value

gb|EL749173.1| LV0354032 Taenia solium UNAM-cd2_larva Taenia ... 407 8e-114

gb|EL748227.1| LV0336003 Taenia solium UNAM-cd2_larva Taenia ... 407 8e-114

gb|EL742012.1| LV0235001 Taenia solium UNAM-cd2_larva Taenia ... 407 8e-114

gb|EL757934.1| AD0098019 Taenia solium UNAM-cd1_adult Taenia ... 313 2e-85

gb|EL749069.1| LV0352024 Taenia solium UNAM-cd2_larva Taenia ... 278 7e-75

gb|EL748268.1| LV0336044 Taenia solium UNAM-cd2_larva Taenia ... 270 1e-72

gb|EX151197.1| TPEG001001F11 Cysti-host Taenia solium cDNA, m... 226 3e-59

gb|EX150534.1| TSEDTS1003D09 Cysti Taenia solium cDNA, mRNA s... 148 6e-36

gb|EX151449.1| TPEG001004H05 Cysti-host Taenia solium cDNA, m... 121 1e-27

gb|EL741854.1| LV0232059 Taenia solium UNAM-cd2_larva Taenia ... 56.5 3e-08

Query= TS.seq.screen.trim.Contig54

Length=483

Score E

Sequences producing significant alignments: (Bits) Value

gb|EL742391.1| LV0241017 Taenia solium UNAM-cd2_larva Taenia ... 357 8e-99

gb|EL755947.1| AD0028005 Taenia solium UNAM-cd1_adult Taenia ... 298 5e-81

gb|EL755189.1| AD0024206 Taenia solium UNAM-cd1_adult Taenia ... 298 5e-81

gb|EL752700.1| AD0014241 Taenia solium UNAM-cd1_adult Taenia ... 298 5e-81

gb|EL746011.1| LV0300042 Taenia solium UNAM-cd2_larva Taenia ... 294 7e-80

gb|EL755875.1| AD0027171 Taenia solium UNAM-cd1_adult Taenia ... 289 3e-78

gb|EL755188.1| AD0024205 Taenia solium UNAM-cd1_adult Taenia ... 287 1e-77

gb|EL749126.1| LV0353038 Taenia solium UNAM-cd2_larva Taenia ... 268 4e-72

gb|EL748829.1| LV0346019 Taenia solium UNAM-cd2_larva Taenia ... 248 5e-66

gb|EL748941.1| LV0348052 Taenia solium UNAM-cd2_larva Taenia ... 230 2e-60

Query= TS.seq.screen.trim.Contig55

Length=528

Score E

Sequences producing significant alignments: (Bits) Value

gb|EL741960.1| LV0234021 Taenia solium UNAM-cd2_larva Taenia ... 278 7e-75

gb|EL741645.1| LV0229056 Taenia solium UNAM-cd2_larva Taenia ... 278 7e-75

gb|EL749126.1| LV0353038 Taenia solium UNAM-cd2_larva Taenia ... 276 2e-74

gb|EL746378.1| LV0308028 Taenia solium UNAM-cd2_larva Taenia ... 274 9e-74

gb|EL758765.1| AD0111030 Taenia solium UNAM-cd1_adult Taenia ... 259 2e-69

gb|EL740492.1| LV0210016 Taenia solium UNAM-cd2_larva Taenia ... 259 2e-69

gb|EL741803.1| LV0232008 Taenia solium UNAM-cd2_larva Taenia ... 254 1e-67

gb|EL746011.1| LV0300042 Taenia solium UNAM-cd2_larva Taenia ... 246 2e-65

gb|EL748941.1| LV0348052 Taenia solium UNAM-cd2_larva Taenia ... 244 7e-65

gb|EL746307.1| LV0307021 Taenia solium UNAM-cd2_larva Taenia ... 244 7e-65

Query= TS.seq.screen.trim.Contig57

Length=142

Score E

Sequences producing significant alignments: (Bits) Value

gb|EL745731.1| LV0296021 Taenia solium UNAM-cd2_larva Taenia ... 207 9e-54

Query= TS.seq.screen.trim.Contig58

Length=158

Score E

Sequences producing significant alignments: (Bits) Value

gb|EL741803.1| LV0232008 Taenia solium UNAM-cd2_larva Taenia ... 102 4e-22

Query= TS.seq.screen.trim.Contig59

Length=348

Score E

Sequences producing significant alignments: (Bits) Value

gb|EL746630.1| LV0311059 Taenia solium UNAM-cd2_larva Taenia ... 577 6e-165

gb|EL746287.1| LV0307001 Taenia solium UNAM-cd2_larva Taenia ... 577 6e-165

gb|EL742380.1| LV0241006 Taenia solium UNAM-cd2_larva Taenia ... 577 6e-165

gb|EL740984.1| LV0219034 Taenia solium UNAM-cd2_larva Taenia ... 577 6e-165

gb|EL745611.1| LV0294026 Taenia solium UNAM-cd2_larva Taenia ... 573 8e-164

gb|EL748642.1| LV0343027 Taenia solium UNAM-cd2_larva Taenia ... 571 3e-163

gb|EL748529.1| LV0341045 Taenia solium UNAM-cd2_larva Taenia ... 562 2e-160

gb|EL751385.1| AD0009123 Taenia solium UNAM-cd1_adult Taenia ... 558 2e-159

gb|EL761249.1| AD0159046 Taenia solium UNAM-cd1_adult Taenia ... 549 1e-156

gb|EL752363.1| AD0013130 Taenia solium UNAM-cd1_adult Taenia ... 547 5e-156

Query= TS.seq.screen.trim.Contig65

Length=471

Score E

Sequences producing significant alignments: (Bits) Value

gb|EL747407.1| LV0323016 Taenia solium UNAM-cd2_larva Taenia ... 616 1e-176

gb|EL749107.1| LV0353019 Taenia solium UNAM-cd2_larva Taenia ... 606 8e-174

gb|EL748061.1| LV0333012 Taenia solium UNAM-cd2_larva Taenia ... 604 3e-173

gb|EL762205.1| AD0181002 Taenia solium UNAM-cd1_adult Taenia ... 601 4e-172

gb|EL761222.1| AD0159019 Taenia solium UNAM-cd1_adult Taenia ... 601 4e-172

gb|EL749283.1| LV0357027 Taenia solium UNAM-cd2_larva Taenia ... 601 4e-172

gb|EL751655.1| AD0010148 Taenia solium UNAM-cd1_adult Taenia ... 558 2e-159

gb|EL748446.1| LV0340020 Taenia solium UNAM-cd2_larva Taenia ... 544 6e-155

gb|EL748391.1| LV0339029 Taenia solium UNAM-cd2_larva Taenia ... 542 2e-154

gb|EL740819.1| LV0217002 Taenia solium UNAM-cd2_larva Taenia ... 521 3e-148

Query= TS.seq.screen.trim.Contig69

Length=365

Score E

Sequences producing significant alignments: (Bits) Value

gb|EL742396.1| LV0241022 Taenia solium UNAM-cd2_larva Taenia ... 326 2e-89

gb|EL752414.1| AD0013181 Taenia solium UNAM-cd1_adult Taenia ... 255 3e-68

gb|EL743795.1| LV0262036 Taenia solium UNAM-cd2_larva Taenia ... 239 3e-63

gb|EL741854.1| LV0232059 Taenia solium UNAM-cd2_larva Taenia ... 58.4 1e-08

gb|EL745526.1| LV0293004 Taenia solium UNAM-cd2_larva Taenia ... 54.7 1e-07

Query= TS.seq.screen.trim.Contig70

Length=515

Score E

Sequences producing significant alignments: (Bits) Value

gb|EL744918.1| LV0282044 Taenia solium UNAM-cd2_larva Taenia ... 931 0.0

Query= TS.seq.screen.trim.Contig71

Length=253

Score E

Sequences producing significant alignments: (Bits) Value

gb|EL756177.1| AD0028235 Taenia solium UNAM-cd1_adult Taenia ... 359 2e-99

gb|EL742059.1| LV0235048 Taenia solium UNAM-cd2_larva Taenia ... 359 2e-99

Query= TS.seq.screen.trim.Contig72

Length=429

Score E

Sequences producing significant alignments: (Bits) Value

gb|EL747055.1| LV0317052 Taenia solium UNAM-cd2_larva Taenia ... 427 6e-120

Query= TS.seq.screen.trim.Contig73

Length=315

Score E

Sequences producing significant alignments: (Bits) Value

gb|EL747623.1| LV0326039 Taenia solium UNAM-cd2_larva Taenia ... 453 1e-127

Query= TS.seq.screen.trim.Contig74

Length=536

Score E

Sequences producing significant alignments: (Bits) Value

gb|EL759566.1| AD0125024 Taenia solium UNAM-cd1_adult Taenia ... 970 0.0

gb|EL750123.1| AD0004038 Taenia solium UNAM-cd1_adult Taenia ... 968 0.0

gb|EL756085.1| AD0028143 Taenia solium UNAM-cd1_adult Taenia ... 965 0.0

gb|EL760180.1| AD0137013 Taenia solium UNAM-cd1_adult Taenia ... 963 0.0

gb|EL757469.1| AD0035062 Taenia solium UNAM-cd1_adult Taenia ... 963 0.0

gb|EL755537.1| AD0026060 Taenia solium UNAM-cd1_adult Taenia ... 963 0.0

gb|EL754609.1| AD0022134 Taenia solium UNAM-cd1_adult Taenia ... 963 0.0

gb|EL754342.1| AD0021132 Taenia solium UNAM-cd1_adult Taenia ... 963 0.0

gb|EL751106.1| AD0008117 Taenia solium UNAM-cd1_adult Taenia ... 963 0.0

gb|EL751102.1| AD0008113 Taenia solium UNAM-cd1_adult Taenia ... 963 0.0

Query= TS.seq.screen.trim.Contig75

Length=723

Score E

Sequences producing significant alignments: (Bits) Value

gb|EL752970.1| AD0016008 Taenia solium UNAM-cd1_adult Taenia ... 1075 0.0

gb|EL762526.1| AD0188016 Taenia solium UNAM-cd1_adult Taenia ... 1059 0.0

gb|EL748758.1| LV0345010 Taenia solium UNAM-cd2_larva Taenia ... 1000 0.0

gb|EL758405.1| AD0105024 Taenia solium UNAM-cd1_adult Taenia ... 983 0.0

gb|EL757551.1| AD0035144 Taenia solium UNAM-cd1_adult Taenia ... 944 0.0

gb|EL763405.1| AD0203050 Taenia solium UNAM-cd1_adult Taenia ... 800 0.0

gb|EL762741.1| AD0192039 Taenia solium UNAM-cd1_adult Taenia ... 385 4e-107

Query= TS.seq.screen.trim.Contig77

Length=477

Score E

Sequences producing significant alignments: (Bits) Value

gb|EL747521.1| LV0324057 Taenia solium UNAM-cd2_larva Taenia ... 265 5e-71

gb|EL741678.1| LV0230025 Taenia solium UNAM-cd2_larva Taenia ... 248 5e-66

gb|EL746557.1| LV0310054 Taenia solium UNAM-cd2_larva Taenia ... 137 1e-32

Query= TS.seq.screen.trim.Contig80

Length=539

Score E

Sequences producing significant alignments: (Bits) Value

gb|EL761664.1| AD0168024 Taenia solium UNAM-cd1_adult Taenia ... 291 9e-79

gb|EL762021.1| AD0176027 Taenia solium UNAM-cd1_adult Taenia ... 274 9e-74

gb|EL749329.1| LV0359007 Taenia solium UNAM-cd2_larva Taenia ... 220 1e-57

gb|EL748570.1| LV0342021 Taenia solium UNAM-cd2_larva Taenia ... 209 3e-54

gb|EL749074.1| LV0352029 Taenia solium UNAM-cd2_larva Taenia ... 111 7e-25

gb|EL741854.1| LV0232059 Taenia solium UNAM-cd2_larva Taenia ... 56.5 3e-08

Query= TS.seq.screen.trim.Contig82

Length=504

Score E

Sequences producing significant alignments: (Bits) Value

gb|EL745136.1| LV0286053 Taenia solium UNAM-cd2_larva Taenia ... 303 1e-82

gb|EL745465.1| LV0291057 Taenia solium UNAM-cd2_larva Taenia ... 292 2e-79

gb|EL741854.1| LV0232059 Taenia solium UNAM-cd2_larva Taenia ... 60.2 3e-09

gb|EL740885.1| LV0217068 Taenia solium UNAM-cd2_larva Taenia ... 52.8 4e-07

Query= TS.seq.screen.trim.Contig83

Length=731

Score E

Sequences producing significant alignments: (Bits) Value

gb|EL745297.1| LV0289009 Taenia solium UNAM-cd2_larva Taenia ... 867 0.0

gb|EL745468.1| LV0292002 Taenia solium UNAM-cd2_larva Taenia ... 503 1e-142

gb|GT227268.1| tscaa0_000631.z1.scf Taenia solium adult full-... 102 4e-22

Query= TS.seq.screen.trim.Contig84

Length=661

Score E

Sequences producing significant alignments: (Bits) Value

gb|EL757977.1| AD0098062 Taenia solium UNAM-cd1_adult Taenia ... 979 0.0

gb|EL747924.1| LV0331017 Taenia solium UNAM-cd2_larva Taenia ... 590 8e-169

Query= TS.seq.screen.trim.Contig85

Length=476

Score E

Sequences producing significant alignments: (Bits) Value

gb|EL756582.1| AD0030114 Taenia solium UNAM-cd1_adult Taenia ... 782 0.0

gb|EL759909.1| AD0131048 Taenia solium UNAM-cd1_adult Taenia ... 780 0.0

gb|EL755883.1| AD0027179 Taenia solium UNAM-cd1_adult Taenia ... 780 0.0

gb|EL754048.1| AD0020093 Taenia solium UNAM-cd1_adult Taenia ... 780 0.0

gb|EL753073.1| AD0016111 Taenia solium UNAM-cd1_adult Taenia ... 780 0.0

gb|EL752977.1| AD0016015 Taenia solium UNAM-cd1_adult Taenia ... 780 0.0

gb|EL752141.1| AD0012169 Taenia solium UNAM-cd1_adult Taenia ... 780 0.0

gb|EL751590.1| AD0010083 Taenia solium UNAM-cd1_adult Taenia ... 780 0.0

gb|EL754353.1| AD0021143 Taenia solium UNAM-cd1_adult Taenia ... 776 0.0

gb|EL752380.1| AD0013147 Taenia solium UNAM-cd1_adult Taenia ... 776 0.0

Query= TS.seq.screen.trim.Contig87

Length=453

Score E

Sequences producing significant alignments: (Bits) Value

gb|EL762941.1| AD0195061 Taenia solium UNAM-cd1_adult Taenia ... 399 1e-111

gb|EL754685.1| AD0022210 Taenia solium UNAM-cd1_adult Taenia ... 399 1e-111

gb|EL752489.1| AD0014030 Taenia solium UNAM-cd1_adult Taenia ... 399 1e-111

gb|EL751924.1| AD0011167 Taenia solium UNAM-cd1_adult Taenia ... 399 1e-111

gb|EL751815.1| AD0011058 Taenia solium UNAM-cd1_adult Taenia ... 399 1e-111

gb|EL751698.1| AD0010191 Taenia solium UNAM-cd1_adult Taenia ... 399 1e-111

gb|EL751575.1| AD0010068 Taenia solium UNAM-cd1_adult Taenia ... 399 1e-111

gb|EL751417.1| AD0009155 Taenia solium UNAM-cd1_adult Taenia ... 399 1e-111

gb|EL751167.1| AD0008178 Taenia solium UNAM-cd1_adult Taenia ... 399 1e-111

gb|EL750913.1| AD0007149 Taenia solium UNAM-cd1_adult Taenia ... 399 1e-111

Query= TS.seq.screen.trim.Contig88

Length=441

Score E

Sequences producing significant alignments: (Bits) Value

gb|EL747878.1| LV0330043 Taenia solium UNAM-cd2_larva Taenia ... 560 6e-160

gb|EL741267.1| LV0223045 Taenia solium UNAM-cd2_larva Taenia ... 555 3e-158

gb|EL747478.1| LV0324014 Taenia solium UNAM-cd2_larva Taenia ... 553 1e-157

gb|EL741341.1| LV0224061 Taenia solium UNAM-cd2_larva Taenia ... 316 1e-86

Query= TS.seq.screen.trim.Contig89

Length=603

Score E

Sequences producing significant alignments: (Bits) Value

gb|EL760757.1| AD0148035 Taenia solium UNAM-cd1_adult Taenia ... 220 1e-57

gb|EL760703.1| AD0147035 Taenia solium UNAM-cd1_adult Taenia ... 220 1e-57

gb|EL760151.1| AD0136042 Taenia solium UNAM-cd1_adult Taenia ... 220 1e-57

gb|EL759905.1| AD0131044 Taenia solium UNAM-cd1_adult Taenia ... 220 1e-57

gb|EL759705.1| AD0127046 Taenia solium UNAM-cd1_adult Taenia ... 220 1e-57

gb|EL759663.1| AD0127004 Taenia solium UNAM-cd1_adult Taenia ... 220 1e-57

gb|EL759493.1| AD0124007 Taenia solium UNAM-cd1_adult Taenia ... 220 1e-57

gb|EL757417.1| AD0035010 Taenia solium UNAM-cd1_adult Taenia ... 220 1e-57

gb|EL757223.1| AD0034039 Taenia solium UNAM-cd1_adult Taenia ... 220 1e-57

gb|EL757074.1| AD0032086 Taenia solium UNAM-cd1_adult Taenia ... 220 1e-57

Query= TS.seq.screen.trim.Contig90

Length=521

Score E

Sequences producing significant alignments: (Bits) Value

gb|GT226920.1| tscaa0_000515.z1.scf Taenia solium adult full-... 592 2e-169

gb|GT226913.1| tscaa0_001902.z1.scf Taenia solium adult full-... 180 2e-45

gb|EL750271.1| AD0004186 Taenia solium UNAM-cd1_adult Taenia ... 163 2e-40

gb|EL750503.1| AD0005166 Taenia solium UNAM-cd1_adult Taenia ... 152 4e-37

Query= TS.seq.screen.trim.Contig93

Length=449

Score E

Sequences producing significant alignments: (Bits) Value

gb|EL743066.1| LV0251031 Taenia solium UNAM-cd2_larva Taenia ... 226 3e-59

gb|EL741854.1| LV0232059 Taenia solium UNAM-cd2_larva Taenia ... 56.5 3e-08

Query= TS.seq.screen.trim.Contig95

Length=314

Score E

Sequences producing significant alignments: (Bits) Value

gb|EL740723.1| LV0215013 Taenia solium UNAM-cd2_larva Taenia ... 364 5e-101

gb|EL762052.1| AD0177023 Taenia solium UNAM-cd1_adult Taenia ... 292 2e-79

gb|EX151213.1| TPEG001001H09 Cysti-host Taenia solium cDNA, m... 285 4e-77

gb|EX150563.1| TSEDTS1008G12 Cysti Taenia solium cDNA, mRNA s... 193 3e-49

Query= TS.seq.screen.trim.Contig96

Length=588

Score E

Sequences producing significant alignments: (Bits) Value

gb|EL744767.1| LV0279036 Taenia solium UNAM-cd2_larva Taenia ... 1027 0.0

gb|EL746531.1| LV0310028 Taenia solium UNAM-cd2_larva Taenia ... 1018 0.0

gb|EL748573.1| LV0342024 Taenia solium UNAM-cd2_larva Taenia ... 990 0.0

gb|EL749091.1| LV0353003 Taenia solium UNAM-cd2_larva Taenia ... 885 0.0

gb|EL740221.1| LV0205001 Taenia solium UNAM-cd2_larva Taenia ... 686 0.0

gb|EL745570.1| LV0293048 Taenia solium UNAM-cd2_larva Taenia ... 501 4e-142

gb|EL757443.1| AD0035036 Taenia solium UNAM-cd1_adult Taenia ... 379 2e-105

gb|EL743123.1| LV0252024 Taenia solium UNAM-cd2_larva Taenia ... 315 5e-86

gb|EL763105.1| AD0198043 Taenia solium UNAM-cd1_adult Taenia ... 276 2e-74

gb|EL747936.1| LV0331029 Taenia solium UNAM-cd2_larva Taenia ... 276 2e-74

Query= TS.seq.screen.trim.Contig97

Length=397

Score E

Sequences producing significant alignments: (Bits) Value

gb|EL745633.1| LV0294048 Taenia solium UNAM-cd2_larva Taenia ... 686 0.0

gb|EL744631.1| LV0277008 Taenia solium UNAM-cd2_larva Taenia ... 686 0.0

gb|EL742324.1| LV0240019 Taenia solium UNAM-cd2_larva Taenia ... 669 0.0

Query= TS.seq.screen.trim.Contig99

Length=499

Score E

Sequences producing significant alignments: (Bits) Value

gb|EL742924.1| LV0249039 Taenia solium UNAM-cd2_larva Taenia ... 907 0.0

gb|EL748402.1| LV0339040 Taenia solium UNAM-cd2_larva Taenia ... 739 0.0

gb|EL763206.1| AD0200021 Taenia solium UNAM-cd1_adult Taenia ... 712 0.0

gb|EL743292.1| LV0255006 Taenia solium UNAM-cd2_larva Taenia ... 689 0.0

gb|EL745508.1| LV0292042 Taenia solium UNAM-cd2_larva Taenia ... 654 0.0

gb|EL742884.1| LV0248046 Taenia solium UNAM-cd2_larva Taenia ... 472 3e-133

gb|EL761979.1| AD0175025 Taenia solium UNAM-cd1_adult Taenia ... 414 5e-116

gb|EL742449.1| LV0242013 Taenia solium UNAM-cd2_larva Taenia ... 407 8e-114

gb|EL740563.1| LV0211040 Taenia solium UNAM-cd2_larva Taenia ... 372 3e-103

Query= TS.seq.screen.trim.Contig100

Length=569

Score E

Sequences producing significant alignments: (Bits) Value

gb|EL741854.1| LV0232059 Taenia solium UNAM-cd2_larva Taenia ... 56.5 3e-08

Query= TS.seq.screen.trim.Contig101

Length=320

Score E

Sequences producing significant alignments: (Bits) Value

gb|EL741945.1| LV0234006 Taenia solium UNAM-cd2_larva Taenia ... 571 3e-163

gb|EL742948.1| LV0249063 Taenia solium UNAM-cd2_larva Taenia ... 547 5e-156

gb|EL744067.1| LV0267006 Taenia solium UNAM-cd2_larva Taenia ... 545 2e-155

gb|EL762842.1| AD0194013 Taenia solium UNAM-cd1_adult Taenia ... 490 8e-139

Query= TS.seq.screen.trim.Contig105

Length=513

Score E

Sequences producing significant alignments: (Bits) Value

gb|EL742149.1| LV0237037 Taenia solium UNAM-cd2_larva Taenia ... 889 0.0

Query= TS.seq.screen.trim.Contig109

Length=674

Score E

Sequences producing significant alignments: (Bits) Value

gb|EL744016.1| LV0266016 Taenia solium UNAM-cd2_larva Taenia ... 957 0.0

gb|EL743547.1| LV0259010 Taenia solium UNAM-cd2_larva Taenia ... 628 2e-180

Query= TS.seq.screen.trim.Contig112

Length=549

Score E

Sequences producing significant alignments: (Bits) Value

gb|EL755508.1| AD0026031 Taenia solium UNAM-cd1_adult Taenia ... 392 2e-109

gb|EL757108.1| AD0032120 Taenia solium UNAM-cd1_adult Taenia ... 320 1e-87

Query= TS.seq.screen.trim.Contig113

Length=566

Score E

Sequences producing significant alignments: (Bits) Value

gb|EL745372.1| LV0290027 Taenia solium UNAM-cd2_larva Taenia ... 466 1e-131

gb|EL742183.1| LV0238002 Taenia solium UNAM-cd2_larva Taenia ... 435 4e-122

Query= TS.seq.screen.trim.Contig114

Length=617

Score E

Sequences producing significant alignments: (Bits) Value

gb|EL743381.1| LV0256042 Taenia solium UNAM-cd2_larva Taenia ... 359 2e-99

gb|EL741854.1| LV0232059 Taenia solium UNAM-cd2_larva Taenia ... 60.2 3e-09

Query= TS.seq.screen.trim.Contig116

Length=627

Score E

Sequences producing significant alignments: (Bits) Value

gb|EL759121.1| AD0117024 Taenia solium UNAM-cd1_adult Taenia ... 750 0.0

gb|EL747945.1| LV0331038 Taenia solium UNAM-cd2_larva Taenia ... 641 0.0

gb|EL743708.1| LV0261017 Taenia solium UNAM-cd2_larva Taenia ... 612 2e-175

gb|EL759120.1| AD0117023 Taenia solium UNAM-cd1_adult Taenia ... 355 3e-98

gb|EL743432.1| LV0257049 Taenia solium UNAM-cd2_larva Taenia ... 350 1e-96

gb|EL743433.1| LV0257050 Taenia solium UNAM-cd2_larva Taenia ... 283 1e-76

gb|EL757861.1| AD0097004 Taenia solium UNAM-cd1_adult Taenia ... 219 4e-57

Query= TS.seq.screen.trim.Contig117

Length=571

Score E

Sequences producing significant alignments: (Bits) Value

gb|EL748690.1| LV0344013 Taenia solium UNAM-cd2_larva Taenia ... 865 0.0

Query= TS.seq.screen.trim.Contig119

Length=503

Score E

Sequences producing significant alignments: (Bits) Value

gb|EL756785.1| AD0031058 Taenia solium UNAM-cd1_adult Taenia ... 900 0.0

gb|EL755589.1| AD0026112 Taenia solium UNAM-cd1_adult Taenia ... 900 0.0

gb|EL755288.1| AD0025058 Taenia solium UNAM-cd1_adult Taenia ... 900 0.0

gb|EL753239.1| AD0016277 Taenia solium UNAM-cd1_adult Taenia ... 900 0.0

gb|EL752613.1| AD0014154 Taenia solium UNAM-cd1_adult Taenia ... 900 0.0

gb|EL752600.1| AD0014141 Taenia solium UNAM-cd1_adult Taenia ... 900 0.0

gb|EL752136.1| AD0012164 Taenia solium UNAM-cd1_adult Taenia ... 900 0.0

gb|EL751525.1| AD0010018 Taenia solium UNAM-cd1_adult Taenia ... 900 0.0

gb|EL750213.1| AD0004128 Taenia solium UNAM-cd1_adult Taenia ... 900 0.0

gb|EL749625.1| AD0001248 Taenia solium UNAM-cd1_adult Taenia ... 900 0.0

Query= TS.seq.screen.trim.Contig120

Length=360

Score E

Sequences producing significant alignments: (Bits) Value

gb|EL758718.1| AD0110027 Taenia solium UNAM-cd1_adult Taenia ... 307 9e-84

gb|EL742073.1| LV0236002 Taenia solium UNAM-cd2_larva Taenia ... 300 1e-81

gb|EL762780.1| AD0193027 Taenia solium UNAM-cd1_adult Taenia ... 292 2e-79

gb|EL761558.1| AD0165056 Taenia solium UNAM-cd1_adult Taenia ... 292 2e-79

gb|EL756614.1| AD0030146 Taenia solium UNAM-cd1_adult Taenia ... 292 2e-79

gb|EL755574.1| AD0026097 Taenia solium UNAM-cd1_adult Taenia ... 292 2e-79

gb|EL752711.1| AD0014252 Taenia solium UNAM-cd1_adult Taenia ... 292 2e-79

gb|EL752073.1| AD0012101 Taenia solium UNAM-cd1_adult Taenia ... 292 2e-79

gb|EL750293.1| AD0004208 Taenia solium UNAM-cd1_adult Taenia ... 292 2e-79

gb|EL749640.1| AD0002004 Taenia solium UNAM-cd1_adult Taenia ... 292 2e-79

Query= TS.seq.screen.trim.Contig121

Length=459

Score E

Sequences producing significant alignments: (Bits) Value

gb|EL761452.1| AD0164008 Taenia solium UNAM-cd1_adult Taenia ... 508 2e-144

gb|EL757908.1| AD0097051 Taenia solium UNAM-cd1_adult Taenia ... 508 2e-144

gb|EL757690.1| AD0094030 Taenia solium UNAM-cd1_adult Taenia ... 505 3e-143

gb|EL742371.1| LV0240066 Taenia solium UNAM-cd2_larva Taenia ... 505 3e-143

gb|EL761301.1| AD0161018 Taenia solium UNAM-cd1_adult Taenia ... 503 1e-142

gb|EL751816.1| AD0011059 Taenia solium UNAM-cd1_adult Taenia ... 503 1e-142

gb|EL760473.1| AD0143015 Taenia solium UNAM-cd1_adult Taenia ... 499 1e-141

gb|EL759781.1| AD0129021 Taenia solium UNAM-cd1_adult Taenia ... 499 1e-141

gb|EL759317.1| AD0121013 Taenia solium UNAM-cd1_adult Taenia ... 499 1e-141

gb|EL756590.1| AD0030122 Taenia solium UNAM-cd1_adult Taenia ... 499 1e-141

Query= TS.seq.screen.trim.Contig125

Length=465

Score E

Sequences producing significant alignments: (Bits) Value

gb|EL744802.1| LV0280028 Taenia solium UNAM-cd2_larva Taenia ... 835 0.0

gb|EL747064.1| LV0317061 Taenia solium UNAM-cd2_larva Taenia ... 830 0.0

gb|EL759563.1| AD0125021 Taenia solium UNAM-cd1_adult Taenia ... 826 0.0

gb|EL755026.1| AD0024043 Taenia solium UNAM-cd1_adult Taenia ... 826 0.0

gb|EL740944.1| LV0218055 Taenia solium UNAM-cd2_larva Taenia ... 822 0.0

gb|EL748908.1| LV0348019 Taenia solium UNAM-cd2_larva Taenia ... 821 0.0

gb|EL742091.1| LV0236020 Taenia solium UNAM-cd2_larva Taenia ... 821 0.0

gb|EL753060.1| AD0016098 Taenia solium UNAM-cd1_adult Taenia ... 817 0.0

gb|EL751101.1| AD0008112 Taenia solium UNAM-cd1_adult Taenia ... 791 0.0

gb|EL748967.1| LV0349016 Taenia solium UNAM-cd2_larva Taenia ... 739 0.0

Query= TS.seq.screen.trim.Contig128

Length=410

Score E

Sequences producing significant alignments: (Bits) Value

gb|EL742253.1| LV0239005 Taenia solium UNAM-cd2_larva Taenia ... 291 9e-79

Query= TS.seq.screen.trim.Contig129

Length=604

Score E

Sequences producing significant alignments: (Bits) Value

gb|EL740935.1| LV0218046 Taenia solium UNAM-cd2_larva Taenia ... 483 1e-136

gb|EL746404.1| LV0308054 Taenia solium UNAM-cd2_larva Taenia ... 390 8e-109

gb|EL763414.1| AD0203059 Taenia solium UNAM-cd1_adult Taenia ... 363 2e-100

Query= TS.seq.screen.trim.Contig130

Length=587

Score E

Sequences producing significant alignments: (Bits) Value

gb|EL741256.1| LV0223034 Taenia solium UNAM-cd2_larva Taenia ... 385 4e-107

Query= TS.seq.screen.trim.Contig131

Length=367

Score E

Sequences producing significant alignments: (Bits) Value

gb|EL747231.1| LV0320028 Taenia solium UNAM-cd2_larva Taenia ... 614 5e-176

gb|EL744443.1| LV0273001 Taenia solium UNAM-cd2_larva Taenia ... 320 1e-87

gb|EL741854.1| LV0232059 Taenia solium UNAM-cd2_larva Taenia ... 56.5 3e-08

Query= TS.seq.screen.trim.Contig132

Length=340

Score E

Sequences producing significant alignments: (Bits) Value

gb|EL746249.1| LV0305033 Taenia solium UNAM-cd2_larva Taenia ... 595 2e-170

gb|EL746497.1| LV0309067 Taenia solium UNAM-cd2_larva Taenia ... 586 1e-167

gb|EL742725.1| LV0246008 Taenia solium UNAM-cd2_larva Taenia ... 586 1e-167

gb|EL745708.1| LV0295066 Taenia solium UNAM-cd2_larva Taenia ... 582 1e-166

gb|EL740247.1| LV0205027 Taenia solium UNAM-cd2_larva Taenia ... 580 5e-166

gb|EL741635.1| LV0229046 Taenia solium UNAM-cd2_larva Taenia ... 573 8e-164

gb|EL743151.1| LV0252052 Taenia solium UNAM-cd2_larva Taenia ... 560 6e-160

gb|EL745739.1| LV0296029 Taenia solium UNAM-cd2_larva Taenia ... 473 8e-134

gb|EL743462.1| LV0258002 Taenia solium UNAM-cd2_larva Taenia ... 473 8e-134

gb|EL745641.1| LV0294056 Taenia solium UNAM-cd2_larva Taenia ... 472 3e-133

Query= TS.seq.screen.trim.Contig136

Length=487

Score E

Sequences producing significant alignments: (Bits) Value

gb|EL741725.1| LV0231003 Taenia solium UNAM-cd2_larva Taenia ... 252 4e-67

Query= TS.seq.screen.trim.Contig138

Length=412

Score E

Sequences producing significant alignments: (Bits) Value

gb|GT227454.1| tscaa0_001355.z1.scf Taenia solium adult full-... 739 0.0

gb|EL747023.1| LV0317020 Taenia solium UNAM-cd2_larva Taenia ... 645 0.0

gb|EL747349.1| LV0322026 Taenia solium UNAM-cd2_larva Taenia ... 315 5e-86

Query= TS.seq.screen.trim.Contig139

Length=598

Score E

Sequences producing significant alignments: (Bits) Value

gb|EL742172.1| LV0237060 Taenia solium UNAM-cd2_larva Taenia ... 1040 0.0

gb|EL740477.1| LV0210001 Taenia solium UNAM-cd2_larva Taenia ... 915 0.0

gb|GT227616.1| tscaa0_001777.z1.scf Taenia solium adult full-... 702 0.0

gb|EL761495.1| AD0164051 Taenia solium UNAM-cd1_adult Taenia ... 252 4e-67

gb|EL761019.1| AD0155007 Taenia solium UNAM-cd1_adult Taenia ... 196 2e-50

Query= TS.seq.screen.trim.Contig143

Length=599

Score E

Sequences producing significant alignments: (Bits) Value

gb|EL742285.1| LV0239037 Taenia solium UNAM-cd2_larva Taenia ... 942 0.0

gb|EL743349.1| LV0256010 Taenia solium UNAM-cd2_larva Taenia ... 244 7e-65

Query= TS.seq.screen.trim.Contig144

Length=437

Score E

Sequences producing significant alignments: (Bits) Value

gb|EL748438.1| LV0340012 Taenia solium UNAM-cd2_larva Taenia ... 575 2e-164

gb|GT226988.1| tscaa0_001894.z1.scf Taenia solium adult full-... 444 6e-125

gb|EL744701.1| LV0278020 Taenia solium UNAM-cd2_larva Taenia ... 268 4e-72

gb|EL748437.1| LV0340011 Taenia solium UNAM-cd2_larva Taenia ... 148 6e-36

gb|EL744700.1| LV0278019 Taenia solium UNAM-cd2_larva Taenia ... 148 6e-36

Query= TS.seq.screen.trim.Contig145

Length=816

Score E

Sequences producing significant alignments: (Bits) Value

gb|EL747414.1| LV0323023 Taenia solium UNAM-cd2_larva Taenia ... 695 0.0

gb|EL748431.1| LV0340005 Taenia solium UNAM-cd2_larva Taenia ... 601 4e-172

gb|EL743439.1| LV0257056 Taenia solium UNAM-cd2_larva Taenia ... 303 1e-82

gb|EL740995.1| LV0219045 Taenia solium UNAM-cd2_larva Taenia ... 111 7e-25

gb|EL747769.1| LV0329007 Taenia solium UNAM-cd2_larva Taenia ... 110 3e-24

Query= TS.seq.screen.trim.Contig146

Length=799

Score E

Sequences producing significant alignments: (Bits) Value

gb|EL742639.1| LV0244070 Taenia solium UNAM-cd2_larva Taenia ... 957 0.0

gb|EL759207.1| AD0119007 Taenia solium UNAM-cd1_adult Taenia ... 497 5e-141

gb|EL742638.1| LV0244069 Taenia solium UNAM-cd2_larva Taenia ... 191 9e-49

gb|GT227238.1| tscaa0_000551.z1.scf Taenia solium adult full-... 141 9e-34

gb|EL758403.1| AD0105022 Taenia solium UNAM-cd1_adult Taenia ... 137 1e-32

Query= TS.seq.screen.trim.Contig147

Length=333

Score E

Sequences producing significant alignments: (Bits) Value

gb|EL758791.1| AD0111056 Taenia solium UNAM-cd1_adult Taenia ... 431 5e-121

gb|EL763423.1| AD0203068 Taenia solium UNAM-cd1_adult Taenia ... 425 2e-119

gb|EL745760.1| LV0296050 Taenia solium UNAM-cd2_larva Taenia ... 383 1e-106

gb|EL744762.1| LV0279031 Taenia solium UNAM-cd2_larva Taenia ... 363 2e-100

gb|EL741430.1| LV0226049 Taenia solium UNAM-cd2_larva Taenia ... 278 7e-75

gb|EL743882.1| LV0264011 Taenia solium UNAM-cd2_larva Taenia ... 259 2e-69

Query= TS.seq.screen.trim.Contig148

Length=420

Score E

Sequences producing significant alignments: (Bits) Value

gb|EL741232.1| LV0223010 Taenia solium UNAM-cd2_larva Taenia ... 501 4e-142

gb|EL741854.1| LV0232059 Taenia solium UNAM-cd2_larva Taenia ... 56.5 3e-08

Query= TS.seq.screen.trim.Contig149

Length=645

Score E

Sequences producing significant alignments: (Bits) Value

gb|EL751146.1| AD0008157 Taenia solium UNAM-cd1_adult Taenia ... 1022 0.0

gb|EL755576.1| AD0026099 Taenia solium UNAM-cd1_adult Taenia ... 1011 0.0

gb|EL762999.1| AD0197008 Taenia solium UNAM-cd1_adult Taenia ... 898 0.0

gb|EL757432.1| AD0035025 Taenia solium UNAM-cd1_adult Taenia ... 883 0.0

gb|EL747912.1| LV0331005 Taenia solium UNAM-cd2_larva Taenia ... 878 0.0

gb|EL755209.1| AD0024226 Taenia solium UNAM-cd1_adult Taenia ... 804 0.0

gb|EL753742.1| AD0019029 Taenia solium UNAM-cd1_adult Taenia ... 793 0.0

gb|EL751398.1| AD0009136 Taenia solium UNAM-cd1_adult Taenia ... 754 0.0

gb|EL761981.1| AD0175027 Taenia solium UNAM-cd1_adult Taenia ... 612 2e-175

gb|EL757047.1| AD0032059 Taenia solium UNAM-cd1_adult Taenia ... 597 5e-171

Query= TS.seq.screen.trim.Contig151

Length=471

Score E

Sequences producing significant alignments: (Bits) Value

gb|EL746011.1| LV0300042 Taenia solium UNAM-cd2_larva Taenia ... 833 0.0

gb|EL748446.1| LV0340020 Taenia solium UNAM-cd2_larva Taenia ... 632 0.0

gb|EL740876.1| LV0217059 Taenia solium UNAM-cd2_larva Taenia ... 580 5e-166

gb|EL755947.1| AD0028005 Taenia solium UNAM-cd1_adult Taenia ... 571 3e-163

gb|EL755875.1| AD0027171 Taenia solium UNAM-cd1_adult Taenia ... 571 3e-163

gb|EL755189.1| AD0024206 Taenia solium UNAM-cd1_adult Taenia ... 562 2e-160

gb|EL752930.1| AD0015189 Taenia solium UNAM-cd1_adult Taenia ... 558 2e-159

gb|EL740670.1| LV0213052 Taenia solium UNAM-cd2_larva Taenia ... 540 8e-154

gb|EL761217.1| AD0159014 Taenia solium UNAM-cd1_adult Taenia ... 536 1e-152

gb|EL748588.1| LV0342039 Taenia solium UNAM-cd2_larva Taenia ... 527 6e-150

Query= TS.seq.screen.trim.Contig152

Length=571

Score E

Sequences producing significant alignments: (Bits) Value

gb|EL741854.1| LV0232059 Taenia solium UNAM-cd2_larva Taenia ... 58.4 1e-08

Query= TS.seq.screen.trim.Contig155

Length=463

Score E

Sequences producing significant alignments: (Bits) Value

gb|EL744819.1| LV0280045 Taenia solium UNAM-cd2_larva Taenia ... 841 0.0

gb|EL741229.1| LV0223007 Taenia solium UNAM-cd2_larva Taenia ... 798 0.0

gb|EL757593.1| AD0035186 Taenia solium UNAM-cd1_adult Taenia ... 780 0.0

gb|EL759089.1| AD0116052 Taenia solium UNAM-cd1_adult Taenia ... 776 0.0

gb|EL763153.1| AD0199037 Taenia solium UNAM-cd1_adult Taenia ... 771 0.0

gb|EL758372.1| AD0104057 Taenia solium UNAM-cd1_adult Taenia ... 771 0.0

gb|EL762708.1| AD0192006 Taenia solium UNAM-cd1_adult Taenia ... 765 0.0

gb|EL751483.1| AD0009221 Taenia solium UNAM-cd1_adult Taenia ... 765 0.0

gb|EL763227.1| AD0200042 Taenia solium UNAM-cd1_adult Taenia ... 760 0.0

gb|EL762457.1| AD0186021 Taenia solium UNAM-cd1_adult Taenia ... 760 0.0

Query= TS.seq.screen.trim.Contig156

Length=470

Score E

Sequences producing significant alignments: (Bits) Value

gb|EL755968.1| AD0028026 Taenia solium UNAM-cd1_adult Taenia ... 854 0.0

gb|EL750440.1| AD0005103 Taenia solium UNAM-cd1_adult Taenia ... 852 0.0

gb|EL755967.1| AD0028025 Taenia solium UNAM-cd1_adult Taenia ... 848 0.0

gb|EL757486.1| AD0035079 Taenia solium UNAM-cd1_adult Taenia ... 845 0.0

gb|EL755671.1| AD0026194 Taenia solium UNAM-cd1_adult Taenia ... 808 0.0

gb|EL750193.1| AD0004108 Taenia solium UNAM-cd1_adult Taenia ... 800 0.0

gb|EL756861.1| AD0031134 Taenia solium UNAM-cd1_adult Taenia ... 798 0.0

gb|EL756753.1| AD0031026 Taenia solium UNAM-cd1_adult Taenia ... 728 0.0

gb|EL759190.1| AD0118029 Taenia solium UNAM-cd1_adult Taenia ... 669 0.0

Query= TS.seq.screen.trim.Contig158

Length=481

Score E

Sequences producing significant alignments: (Bits) Value

gb|EL758551.1| AD0107044 Taenia solium UNAM-cd1_adult Taenia ... 811 0.0

gb|EL750502.1| AD0005165 Taenia solium UNAM-cd1_adult Taenia ... 736 0.0

gb|EL750270.1| AD0004185 Taenia solium UNAM-cd1_adult Taenia ... 730 0.0

gb|EL746388.1| LV0308038 Taenia solium UNAM-cd2_larva Taenia ... 532 1e-151

gb|EL761088.1| AD0156028 Taenia solium UNAM-cd1_adult Taenia ... 58.4 1e-08

Query= TS.seq.screen.trim.Contig159

Length=463

Score E

Sequences producing significant alignments: (Bits) Value

gb|EL749024.1| LV0351012 Taenia solium UNAM-cd2_larva Taenia ... 115 6e-26

gb|EL741854.1| LV0232059 Taenia solium UNAM-cd2_larva Taenia ... 56.5 3e-08

Query= TS.seq.screen.trim.Contig160

Length=427

Score E

Sequences producing significant alignments: (Bits) Value

gb|EL761902.1| AD0173023 Taenia solium UNAM-cd1_adult Taenia ... 73.1 3e-13

Query= TS.seq.screen.trim.Contig163

Length=510

Score E

Sequences producing significant alignments: (Bits) Value

gb|EL745282.1| LV0288064 Taenia solium UNAM-cd2_larva Taenia ... 880 0.0

gb|EL751330.1| AD0009068 Taenia solium UNAM-cd1_adult Taenia ... 856 0.0

gb|EL753490.1| AD0018028 Taenia solium UNAM-cd1_adult Taenia ... 833 0.0

gb|EL758683.1| AD0109058 Taenia solium UNAM-cd1_adult Taenia ... 784 0.0

gb|EL760453.1| AD0142051 Taenia solium UNAM-cd1_adult Taenia ... 739 0.0

gb|EL752526.1| AD0014067 Taenia solium UNAM-cd1_adult Taenia ... 730 0.0

gb|EL748058.1| LV0333009 Taenia solium UNAM-cd2_larva Taenia ... 688 0.0

gb|EL752793.1| AD0015052 Taenia solium UNAM-cd1_adult Taenia ... 510 6e-145

gb|EL746432.1| LV0309002 Taenia solium UNAM-cd2_larva Taenia ... 490 8e-139

gb|EL751073.1| AD0008084 Taenia solium UNAM-cd1_adult Taenia ... 479 2e-135

Query= TS.seq.screen.trim.Contig167

Length=517

Score E

Sequences producing significant alignments: (Bits) Value

gb|EL741978.1| LV0234039 Taenia solium UNAM-cd2_larva Taenia ... 676 0.0

gb|EL744678.1| LV0277055 Taenia solium UNAM-cd2_larva Taenia ... 196 2e-50

Query= TS.seq.screen.trim.Contig168

Length=507

Score E

Sequences producing significant alignments: (Bits) Value

gb|EL744084.1| LV0267023 Taenia solium UNAM-cd2_larva Taenia ... 667 0.0

gb|EL742771.1| LV0246054 Taenia solium UNAM-cd2_larva Taenia ... 553 1e-157

gb|EL744928.1| LV0282054 Taenia solium UNAM-cd2_larva Taenia ... 416 1e-116

gb|EL749270.1| LV0357014 Taenia solium UNAM-cd2_larva Taenia ... 361 7e-100

gb|EL761029.1| AD0155017 Taenia solium UNAM-cd1_adult Taenia ... 246 2e-65

gb|EL747566.1| LV0325045 Taenia solium UNAM-cd2_larva Taenia ... 237 1e-62

Query= TS.seq.screen.trim.Contig169

Length=502

Score E

Sequences producing significant alignments: (Bits) Value

gb|EL746117.1| LV0302012 Taenia solium UNAM-cd2_larva Taenia ... 848 0.0

Query= TS.seq.screen.trim.Contig171

Length=455

Score E

Sequences producing significant alignments: (Bits) Value

gb|EL757991.1| AD0099003 Taenia solium UNAM-cd1_adult Taenia ... 754 0.0

gb|EL748377.1| LV0339015 Taenia solium UNAM-cd2_larva Taenia ... 739 0.0

gb|EL757923.1| AD0098008 Taenia solium UNAM-cd1_adult Taenia ... 734 0.0

gb|EL753583.1| AD0018121 Taenia solium UNAM-cd1_adult Taenia ... 732 0.0

gb|EL740634.1| LV0213016 Taenia solium UNAM-cd2_larva Taenia ... 730 0.0

gb|EL759489.1| AD0124003 Taenia solium UNAM-cd1_adult Taenia ... 726 0.0

gb|EL758829.1| AD0112028 Taenia solium UNAM-cd1_adult Taenia ... 726 0.0

gb|EL748166.1| LV0334049 Taenia solium UNAM-cd2_larva Taenia ... 726 0.0

gb|EL749345.1| LV0359023 Taenia solium UNAM-cd2_larva Taenia ... 721 0.0

gb|GT227145.1| tscaa0_002549.z1.scf Taenia solium adult full-... 719 0.0

Query= TS.seq.screen.trim.Contig172

Length=642

Score E

Sequences producing significant alignments: (Bits) Value

gb|EL746088.1| LV0301043 Taenia solium UNAM-cd2_larva Taenia ... 1140 0.0

gb|EL740743.1| LV0215033 Taenia solium UNAM-cd2_larva Taenia ... 1094 0.0

gb|EL759007.1| AD0115032 Taenia solium UNAM-cd1_adult Taenia ... 1074 0.0

gb|EL745542.1| LV0293020 Taenia solium UNAM-cd2_larva Taenia ... 1011 0.0

gb|EL749094.1| LV0353006 Taenia solium UNAM-cd2_larva Taenia ... 994 0.0

gb|EL758978.1| AD0115003 Taenia solium UNAM-cd1_adult Taenia ... 845 0.0

gb|EL763176.1| AD0199060 Taenia solium UNAM-cd1_adult Taenia ... 802 0.0

gb|EL762962.1| AD0196015 Taenia solium UNAM-cd1_adult Taenia ... 802 0.0

gb|EL760455.1| AD0142053 Taenia solium UNAM-cd1_adult Taenia ... 802 0.0

gb|EL759972.1| AD0133017 Taenia solium UNAM-cd1_adult Taenia ... 802 0.0

Query= TS.seq.screen.trim.Contig173

Length=443

Score E

Sequences producing significant alignments: (Bits) Value

gb|EL760198.1| AD0137031 Taenia solium UNAM-cd1_adult Taenia ... 285 4e-77

gb|EL740656.1| LV0213038 Taenia solium UNAM-cd2_larva Taenia ... 117 2e-26

Query= TS.seq.screen.trim.Contig174

Length=743

Score E

Sequences producing significant alignments: (Bits) Value

gb|GT227606.1| tscaa0_001742.z1.scf Taenia solium adult full-... 257 9e-69

gb|EL751274.1| AD0009012 Taenia solium UNAM-cd1_adult Taenia ... 246 2e-65

gb|EL751000.1| AD0008011 Taenia solium UNAM-cd1_adult Taenia ... 237 1e-62

Query= TS.seq.screen.trim.Contig175

Length=633

Score E

Sequences producing significant alignments: (Bits) Value

gb|EL753689.1| AD0018227 Taenia solium UNAM-cd1_adult Taenia ... 520 1e-147

gb|EL753688.1| AD0018226 Taenia solium UNAM-cd1_adult Taenia ... 139 3e-33

gb|EL741005.1| LV0219055 Taenia solium UNAM-cd2_larva Taenia ... 139 3e-33

gb|EL741854.1| LV0232059 Taenia solium UNAM-cd2_larva Taenia ... 58.4 1e-08

Query= TS.seq.screen.trim.Contig176

Length=515

Score E

Sequences producing significant alignments: (Bits) Value

gb|EL748649.1| LV0343034 Taenia solium UNAM-cd2_larva Taenia ... 398 5e-111

gb|EX150538.1| TSEDTS1003D10 Cysti Taenia solium cDNA, mRNA s... 340 9e-94

Query= TS.seq.screen.trim.Contig177

Length=290

Score E

Sequences producing significant alignments: (Bits) Value

gb|EL756638.1| AD0030170 Taenia solium UNAM-cd1_adult Taenia ... 521 3e-148

gb|EL757130.1| AD0032142 Taenia solium UNAM-cd1_adult Taenia ... 518 4e-147

gb|EL762100.1| AD0178034 Taenia solium UNAM-cd1_adult Taenia ... 475 2e-134

gb|EL747229.1| LV0320026 Taenia solium UNAM-cd2_larva Taenia ... 446 2e-125

gb|EL761891.1| AD0173012 Taenia solium UNAM-cd1_adult Taenia ... 416 1e-116

gb|EL761751.1| AD0170022 Taenia solium UNAM-cd1_adult Taenia ... 416 1e-116

Query= TS.seq.screen.trim.Contig178

Length=455

Score E

Sequences producing significant alignments: (Bits) Value

gb|EL744675.1| LV0277052 Taenia solium UNAM-cd2_larva Taenia ... 752 0.0

gb|EL761928.1| AD0174007 Taenia solium UNAM-cd1_adult Taenia ... 628 2e-180

gb|EL746431.1| LV0309001 Taenia solium UNAM-cd2_larva Taenia ... 460 6e-130

gb|EL744627.1| LV0277004 Taenia solium UNAM-cd2_larva Taenia ... 418 4e-117

gb|EL741506.1| LV0227062 Taenia solium UNAM-cd2_larva Taenia ... 320 1e-87

gb|EL752268.1| AD0013035 Taenia solium UNAM-cd1_adult Taenia ... 82.4 6e-16

gb|EL751933.1| AD0011176 Taenia solium UNAM-cd1_adult Taenia ... 82.4 6e-16

gb|EL751812.1| AD0011055 Taenia solium UNAM-cd1_adult Taenia ... 82.4 6e-16

gb|EL751378.1| AD0009116 Taenia solium UNAM-cd1_adult Taenia ... 82.4 6e-16

gb|EL749887.1| AD0003029 Taenia solium UNAM-cd1_adult Taenia ... 82.4 6e-16

Query= TS.seq.screen.trim.Contig179

Length=400

Score E

Sequences producing significant alignments: (Bits) Value

gb|EL745832.1| LV0297061 Taenia solium UNAM-cd2_larva Taenia ... 545 2e-155

gb|EL746354.1| LV0308004 Taenia solium UNAM-cd2_larva Taenia ... 488 3e-138

gb|EL747966.1| LV0331059 Taenia solium UNAM-cd2_larva Taenia ... 486 1e-137

gb|EL742159.1| LV0237047 Taenia solium UNAM-cd2_larva Taenia ... 457 8e-129

gb|EL758707.1| AD0110016 Taenia solium UNAM-cd1_adult Taenia ... 361 7e-100

gb|EL758508.1| AD0107001 Taenia solium UNAM-cd1_adult Taenia ... 339 3e-93

gb|EL741108.1| LV0221009 Taenia solium UNAM-cd2_larva Taenia ... 327 7e-90

gb|EL746148.1| LV0302043 Taenia solium UNAM-cd2_larva Taenia ... 302 4e-82

gb|EL748555.1| LV0342006 Taenia solium UNAM-cd2_larva Taenia ... 209 3e-54

Query= TS.seq.screen.trim.Contig180

Length=399

Score E

Sequences producing significant alignments: (Bits) Value

gb|EL762143.1| AD0179036 Taenia solium UNAM-cd1_adult Taenia ... 425 2e-119

Query= TS.seq.screen.trim.Contig182

Length=510

Score E

Sequences producing significant alignments: (Bits) Value

gb|EL744495.1| LV0273053 Taenia solium UNAM-cd2_larva Taenia ... 176 3e-44

gb|EL744753.1| LV0279022 Taenia solium UNAM-cd2_larva Taenia ... 174 9e-44

gb|EL748656.1| LV0343041 Taenia solium UNAM-cd2_larva Taenia ... 172 3e-43

gb|EL742076.1| LV0236005 Taenia solium UNAM-cd2_larva Taenia ... 167 2e-41

gb|EL748504.1| LV0341020 Taenia solium UNAM-cd2_larva Taenia ... 165 6e-41

gb|EL741463.1| LV0227019 Taenia solium UNAM-cd2_larva Taenia ... 163 2e-40

gb|EL748391.1| LV0339029 Taenia solium UNAM-cd2_larva Taenia ... 161 7e-40

gb|EL747944.1| LV0331037 Taenia solium UNAM-cd2_larva Taenia ... 159 3e-39

gb|EL748710.1| LV0344033 Taenia solium UNAM-cd2_larva Taenia ... 158 9e-39

gb|EL763324.1| AD0202024 Taenia solium UNAM-cd1_adult Taenia ... 156 3e-38

Query= TS.seq.screen.trim.Contig183

Length=517

Score E

Sequences producing significant alignments: (Bits) Value

gb|EL757251.1| AD0034067 Taenia solium UNAM-cd1_adult Taenia ... 896 0.0

gb|EL754329.1| AD0021119 Taenia solium UNAM-cd1_adult Taenia ... 896 0.0

gb|EL753524.1| AD0018062 Taenia solium UNAM-cd1_adult Taenia ... 896 0.0

gb|EL752487.1| AD0014028 Taenia solium UNAM-cd1_adult Taenia ... 896 0.0

gb|EL750291.1| AD0004206 Taenia solium UNAM-cd1_adult Taenia ... 896 0.0

gb|EL757361.1| AD0034177 Taenia solium UNAM-cd1_adult Taenia ... 880 0.0

gb|EL756021.1| AD0028079 Taenia solium UNAM-cd1_adult Taenia ... 861 0.0

gb|EL759972.1| AD0133017 Taenia solium UNAM-cd1_adult Taenia ... 835 0.0

gb|EL750521.1| AD0005184 Taenia solium UNAM-cd1_adult Taenia ... 835 0.0

gb|EL744897.1| LV0282023 Taenia solium UNAM-cd2_larva Taenia ... 817 0.0

Query= TS.seq.screen.trim.Contig184

Length=539

Score E

Sequences producing significant alignments: (Bits) Value

gb|EL741854.1| LV0232059 Taenia solium UNAM-cd2_larva Taenia ... 60.2 3e-09

Query= TS.seq.screen.trim.Contig185

Length=527

Score E

Sequences producing significant alignments: (Bits) Value

gb|EL746129.1| LV0302024 Taenia solium UNAM-cd2_larva Taenia ... 809 0.0

gb|GT227229.1| tscaa0_000525.z1.scf Taenia solium adult full-... 481 5e-136

Query= TS.seq.screen.trim.Contig186

Length=507

Score E

Sequences producing significant alignments: (Bits) Value

gb|EL761459.1| AD0164015 Taenia solium UNAM-cd1_adult Taenia ... 865 0.0

gb|EL741065.1| LV0220038 Taenia solium UNAM-cd2_larva Taenia ... 846 0.0

gb|EL749170.1| LV0354029 Taenia solium UNAM-cd2_larva Taenia ... 771 0.0

gb|EL749198.1| LV0355012 Taenia solium UNAM-cd2_larva Taenia ... 756 0.0

gb|EL748480.1| LV0340054 Taenia solium UNAM-cd2_larva Taenia ... 756 0.0

gb|EL748479.1| LV0340053 Taenia solium UNAM-cd2_larva Taenia ... 743 0.0

gb|EL756002.1| AD0028060 Taenia solium UNAM-cd1_adult Taenia ... 569 1e-162

gb|EL758631.1| AD0109006 Taenia solium UNAM-cd1_adult Taenia ... 562 2e-160

gb|EL759085.1| AD0116048 Taenia solium UNAM-cd1_adult Taenia ... 536 1e-152

gb|EL762688.1| AD0191049 Taenia solium UNAM-cd1_adult Taenia ... 472 3e-133

Query= TS.seq.screen.trim.Contig189

Length=433

Score E

Sequences producing significant alignments: (Bits) Value

gb|EL746568.1| LV0310065 Taenia solium UNAM-cd2_larva Taenia ... 704 0.0

gb|EL749076.1| LV0352031 Taenia solium UNAM-cd2_larva Taenia ... 399 1e-111

Query= TS.seq.screen.trim.Contig190

Length=373

Score E

Sequences producing significant alignments: (Bits) Value

gb|EL747850.1| LV0330015 Taenia solium UNAM-cd2_larva Taenia ... 621 3e-178

gb|EL759919.1| AD0132010 Taenia solium UNAM-cd1_adult Taenia ... 604 3e-173

gb|EL759282.1| AD0120035 Taenia solium UNAM-cd1_adult Taenia ... 604 3e-173

gb|EL760754.1| AD0148032 Taenia solium UNAM-cd1_adult Taenia ... 601 4e-172

gb|EL750722.1| AD0006161 Taenia solium UNAM-cd1_adult Taenia ... 595 2e-170

gb|EL749688.1| AD0002052 Taenia solium UNAM-cd1_adult Taenia ... 586 1e-167

gb|EL756978.1| AD0031251 Taenia solium UNAM-cd1_adult Taenia ... 532 1e-151

gb|EL757597.1| AD0035190 Taenia solium UNAM-cd1_adult Taenia ... 446 2e-125

gb|EL741854.1| LV0232059 Taenia solium UNAM-cd2_larva Taenia ... 62.1 7e-10

Query= TS.seq.screen.trim.Contig192

Length=670

Score E

Sequences producing significant alignments: (Bits) Value

gb|EL745514.1| LV0292048 Taenia solium UNAM-cd2_larva Taenia ... 69.4 4e-12

gb|EL742672.1| LV0245027 Taenia solium UNAM-cd2_larva Taenia ... 63.9 2e-10

Query= TS.seq.screen.trim.Contig193

Length=534

Score E

Sequences producing significant alignments: (Bits) Value

gb|EL760380.1| AD0141017 Taenia solium UNAM-cd1_adult Taenia ... 966 0.0

gb|EL755364.1| AD0025134 Taenia solium UNAM-cd1_adult Taenia ... 966 0.0

gb|EL754347.1| AD0021137 Taenia solium UNAM-cd1_adult Taenia ... 966 0.0

gb|EL746631.1| LV0311060 Taenia solium UNAM-cd2_larva Taenia ... 948 0.0

gb|EL755265.1| AD0025035 Taenia solium UNAM-cd1_adult Taenia ... 946 0.0

gb|EL740850.1| LV0217033 Taenia solium UNAM-cd2_larva Taenia ... 902 0.0

gb|GT227207.1| tscaa0_000457.z1.scf Taenia solium adult full-... 667 0.0

gb|EL745035.1| LV0285007 Taenia solium UNAM-cd2_larva Taenia ... 464 5e-131

gb|EL755485.1| AD0026008 Taenia solium UNAM-cd1_adult Taenia ... 388 3e-108

gb|EL757474.1| AD0035067 Taenia solium UNAM-cd1_adult Taenia ... 387 1e-107

Query= TS.seq.screen.trim.Contig197

Length=441

Score E

Sequences producing significant alignments: (Bits) Value

gb|EL758390.1| AD0105009 Taenia solium UNAM-cd1_adult Taenia ... 785 0.0

gb|EL755647.1| AD0026170 Taenia solium UNAM-cd1_adult Taenia ... 771 0.0

gb|EL747723.1| LV0328011 Taenia solium UNAM-cd2_larva Taenia ... 745 0.0

gb|EL755849.1| AD0027145 Taenia solium UNAM-cd1_adult Taenia ... 743 0.0

gb|EL743290.1| LV0255004 Taenia solium UNAM-cd2_larva Taenia ... 736 0.0

gb|EL759636.1| AD0126033 Taenia solium UNAM-cd1_adult Taenia ... 702 0.0

gb|EL762002.1| AD0176008 Taenia solium UNAM-cd1_adult Taenia ... 649 0.0

gb|EL763066.1| AD0198004 Taenia solium UNAM-cd1_adult Taenia ... 638 0.0

gb|EL745517.1| LV0292051 Taenia solium UNAM-cd2_larva Taenia ... 531 5e-151

gb|EL761997.1| AD0176003 Taenia solium UNAM-cd1_adult Taenia ... 479 2e-135

Query= TS.seq.screen.trim.Contig199

Length=693

Score E

Sequences producing significant alignments: (Bits) Value

gb|EL752801.1| AD0015060 Taenia solium UNAM-cd1_adult Taenia ... 246 2e-65

gb|EL752537.1| AD0014078 Taenia solium UNAM-cd1_adult Taenia ... 172 3e-43

gb|EL746585.1| LV0311014 Taenia solium UNAM-cd2_larva Taenia ... 78.7 7e-15

Query= TS.seq.screen.trim.Contig200

Length=519

Score E

Sequences producing significant alignments: (Bits) Value

gb|EL746011.1| LV0300042 Taenia solium UNAM-cd2_larva Taenia ... 518 4e-147

gb|EL758765.1| AD0111030 Taenia solium UNAM-cd1_adult Taenia ... 401 4e-112

gb|EL741960.1| LV0234021 Taenia solium UNAM-cd2_larva Taenia ... 381 5e-106

gb|EL741645.1| LV0229056 Taenia solium UNAM-cd2_larva Taenia ... 381 5e-106

gb|EL740670.1| LV0213052 Taenia solium UNAM-cd2_larva Taenia ... 350 1e-96

gb|EL758023.1| AD0099035 Taenia solium UNAM-cd1_adult Taenia ... 311 7e-85

gb|EL743084.1| LV0251049 Taenia solium UNAM-cd2_larva Taenia ... 265 5e-71

gb|EL748061.1| LV0333012 Taenia solium UNAM-cd2_larva Taenia ... 241 9e-64

gb|EL748391.1| LV0339029 Taenia solium UNAM-cd2_larva Taenia ... 224 9e-59

gb|EL757953.1| AD0098038 Taenia solium UNAM-cd1_adult Taenia ... 211 7e-55

Query= TS.seq.screen.trim.Contig201

Length=683

Score E

Sequences producing significant alignments: (Bits) Value

gb|EL740515.1| LV0210039 Taenia solium UNAM-cd2_larva Taenia ... 451 4e-127

gb|EL743471.1| LV0258011 Taenia solium UNAM-cd2_larva Taenia ... 368 4e-102

Query= TS.seq.screen.trim.Contig203

Length=629

Score E

Sequences producing significant alignments: (Bits) Value

gb|EL741506.1| LV0227062 Taenia solium UNAM-cd2_larva Taenia ... 987 0.0

gb|EL746431.1| LV0309001 Taenia solium UNAM-cd2_larva Taenia ... 902 0.0

gb|EL761928.1| AD0174007 Taenia solium UNAM-cd1_adult Taenia ... 534 4e-152

gb|EL744675.1| LV0277052 Taenia solium UNAM-cd2_larva Taenia ... 514 5e-146

gb|EL744627.1| LV0277004 Taenia solium UNAM-cd2_larva Taenia ... 412 2e-115

gb|EL752268.1| AD0013035 Taenia solium UNAM-cd1_adult Taenia ... 324 9e-89

gb|EL751933.1| AD0011176 Taenia solium UNAM-cd1_adult Taenia ... 324 9e-89

gb|EL751812.1| AD0011055 Taenia solium UNAM-cd1_adult Taenia ... 324 9e-89

gb|EL751378.1| AD0009116 Taenia solium UNAM-cd1_adult Taenia ... 324 9e-89

gb|EL749887.1| AD0003029 Taenia solium UNAM-cd1_adult Taenia ... 324 9e-89

Query= TS.seq.screen.trim.Contig205

Length=473

Score E

Sequences producing significant alignments: (Bits) Value

gb|EL743128.1| LV0252029 Taenia solium UNAM-cd2_larva Taenia ... 874 0.0

gb|EL748035.1| LV0332057 Taenia solium UNAM-cd2_larva Taenia ... 638 0.0

gb|EL747761.1| LV0328049 Taenia solium UNAM-cd2_larva Taenia ... 279 2e-75

Query= TS.seq.screen.trim.Contig206

Length=447

Score E

Sequences producing significant alignments: (Bits) Value

gb|EL748241.1| LV0336017 Taenia solium UNAM-cd2_larva Taenia ... 708 0.0

gb|EL749059.1| LV0352014 Taenia solium UNAM-cd2_larva Taenia ... 704 0.0

gb|EL748805.1| LV0345057 Taenia solium UNAM-cd2_larva Taenia ... 649 0.0

gb|EL746521.1| LV0310018 Taenia solium UNAM-cd2_larva Taenia ... 265 5e-71

Query= TS.seq.screen.trim.Contig207

Length=614

Score E

Sequences producing significant alignments: (Bits) Value

gb|EL759400.1| AD0122044 Taenia solium UNAM-cd1_adult Taenia ... 503 1e-142

gb|EL744607.1| LV0276025 Taenia solium UNAM-cd2_larva Taenia ... 331 5e-91

gb|EL761878.1| AD0172045 Taenia solium UNAM-cd1_adult Taenia ... 198 5e-51

gb|EL758164.1| AD0101044 Taenia solium UNAM-cd1_adult Taenia ... 176 3e-44

gb|EL758275.1| AD0103019 Taenia solium UNAM-cd1_adult Taenia ... 152 4e-37

Query= TS.seq.screen.trim.Contig208

Length=538

Score E

Sequences producing significant alignments: (Bits) Value

gb|EL755855.1| AD0027151 Taenia solium UNAM-cd1_adult Taenia ... 822 0.0

Query= TS.seq.screen.trim.Contig209

Length=671

Score E

Sequences producing significant alignments: (Bits) Value

gb|EL747019.1| LV0317016 Taenia solium UNAM-cd2_larva Taenia ... 784 0.0

gb|EL745858.1| LV0298015 Taenia solium UNAM-cd2_larva Taenia ... 693 0.0

gb|EL747052.1| LV0317049 Taenia solium UNAM-cd2_larva Taenia ... 689 0.0

gb|EL747133.1| LV0318065 Taenia solium UNAM-cd2_larva Taenia ... 617 4e-177

gb|EL759892.1| AD0131031 Taenia solium UNAM-cd1_adult Taenia ... 551 4e-157

gb|EL747409.1| LV0323018 Taenia solium UNAM-cd2_larva Taenia ... 551 4e-157

Query= TS.seq.screen.trim.Contig210

Length=444

Score E

Sequences producing significant alignments: (Bits) Value

gb|EL756723.1| AD0030255 Taenia solium UNAM-cd1_adult Taenia ... 784 0.0

gb|EL756512.1| AD0030044 Taenia solium UNAM-cd1_adult Taenia ... 784 0.0

gb|EL749626.1| AD0001249 Taenia solium UNAM-cd1_adult Taenia ... 784 0.0

gb|EL758239.1| AD0102050 Taenia solium UNAM-cd1_adult Taenia ... 316 1e-86

gb|EL746848.1| LV0314061 Taenia solium UNAM-cd2_larva Taenia ... 145 7e-35

gb|EL757086.1| AD0032098 Taenia solium UNAM-cd1_adult Taenia ... 110 3e-24

Query= TS.seq.screen.trim.Contig211

Length=343

Score E

Sequences producing significant alignments: (Bits) Value

gb|EL762739.1| AD0192037 Taenia solium UNAM-cd1_adult Taenia ... 553 1e-157

gb|EL751030.1| AD0008041 Taenia solium UNAM-cd1_adult Taenia ... 508 2e-144

gb|EL763037.1| AD0197046 Taenia solium UNAM-cd1_adult Taenia ... 503 1e-142

gb|EL761936.1| AD0174015 Taenia solium UNAM-cd1_adult Taenia ... 363 2e-100

gb|EL741854.1| LV0232059 Taenia solium UNAM-cd2_larva Taenia ... 56.5 3e-08

Query= TS.seq.screen.trim.Contig213

Length=644

Score E

Sequences producing significant alignments: (Bits) Value

gb|EL753375.1| AD0017133 Taenia solium UNAM-cd1_adult Taenia ... 1171 0.0

gb|EL753134.1| AD0016172 Taenia solium UNAM-cd1_adult Taenia ... 1022 0.0

gb|EL756568.1| AD0030100 Taenia solium UNAM-cd1_adult Taenia ... 632 0.0

Query= TS.seq.screen.trim.Contig214

Length=402

Score E

Sequences producing significant alignments: (Bits) Value

gb|EL759497.1| AD0124011 Taenia solium UNAM-cd1_adult Taenia ... 702 0.0

gb|EL752201.1| AD0012229 Taenia solium UNAM-cd1_adult Taenia ... 699 0.0

gb|EL743214.1| LV0253061 Taenia solium UNAM-cd2_larva Taenia ... 699 0.0

gb|EL758296.1| AD0103040 Taenia solium UNAM-cd1_adult Taenia ... 693 0.0

gb|EL743353.1| LV0256014 Taenia solium UNAM-cd2_larva Taenia ... 676 0.0

gb|EL762166.1| AD0180008 Taenia solium UNAM-cd1_adult Taenia ... 649 0.0

gb|EL744519.1| LV0274022 Taenia solium UNAM-cd2_larva Taenia ... 623 8e-179

gb|EL760889.1| AD0152014 Taenia solium UNAM-cd1_adult Taenia ... 555 3e-158

gb|EL743545.1| LV0259008 Taenia solium UNAM-cd2_larva Taenia ... 520 1e-147

gb|EL740600.1| LV0212036 Taenia solium UNAM-cd2_larva Taenia ... 344 7e-95

Query= TS.seq.screen.trim.Contig215

Length=568

Score E

Sequences producing significant alignments: (Bits) Value

gb|EL746162.1| LV0303013 Taenia solium UNAM-cd2_larva Taenia ... 191 9e-49

Query= TS.seq.screen.trim.Contig216

Length=426

Score E

Sequences producing significant alignments: (Bits) Value

gb|EL758029.1| AD0099041 Taenia solium UNAM-cd1_adult Taenia ... 246 2e-65

Query= TS.seq.screen.trim.Contig217

Length=282

Score E

Sequences producing significant alignments: (Bits) Value

gb|EL745283.1| LV0288065 Taenia solium UNAM-cd2_larva Taenia ... 466 1e-131

gb|EL742506.1| LV0243004 Taenia solium UNAM-cd2_larva Taenia ... 466 1e-131

gb|EL743770.1| LV0262011 Taenia solium UNAM-cd2_larva Taenia ... 462 2e-130

gb|EL748502.1| LV0341018 Taenia solium UNAM-cd2_larva Taenia ... 163 2e-40

Query= TS.seq.screen.trim.Contig218

Length=887

Score E

Sequences producing significant alignments: (Bits) Value

gb|EL745787.1| LV0297016 Taenia solium UNAM-cd2_larva Taenia ... 939 0.0

Query= TS.seq.screen.trim.Contig219

Length=479

Score E

Sequences producing significant alignments: (Bits) Value

gb|EL742340.1| LV0240035 Taenia solium UNAM-cd2_larva Taenia ... 843 0.0

gb|EL740375.1| LV0207059 Taenia solium UNAM-cd2_larva Taenia ... 835 0.0

gb|EL753806.1| AD0019093 Taenia solium UNAM-cd1_adult Taenia ... 833 0.0

gb|EL740970.1| LV0219020 Taenia solium UNAM-cd2_larva Taenia ... 802 0.0

gb|EL747042.1| LV0317039 Taenia solium UNAM-cd2_larva Taenia ... 758 0.0

gb|EL743400.1| LV0257017 Taenia solium UNAM-cd2_larva Taenia ... 353 1e-97

Query= TS.seq.screen.trim.Contig221

Length=511

Score E

Sequences producing significant alignments: (Bits) Value

gb|EL743754.1| LV0261063 Taenia solium UNAM-cd2_larva Taenia ... 896 0.0

gb|EL762379.1| AD0184020 Taenia solium UNAM-cd1_adult Taenia ... 843 0.0

gb|EL747131.1| LV0318063 Taenia solium UNAM-cd2_larva Taenia ... 531 5e-151

gb|EL760067.1| AD0135013 Taenia solium UNAM-cd1_adult Taenia ... 497 5e-141

Query= TS.seq.screen.trim.Contig222

Length=438

Score E

Sequences producing significant alignments: (Bits) Value

gb|EL753553.1| AD0018091 Taenia solium UNAM-cd1_adult Taenia ... 636 0.0

gb|EL742297.1| LV0239049 Taenia solium UNAM-cd2_larva Taenia ... 455 3e-128

gb|EL741854.1| LV0232059 Taenia solium UNAM-cd2_larva Taenia ... 56.5 3e-08

Query= TS.seq.screen.trim.Contig224

Length=660

Score E

Sequences producing significant alignments: (Bits) Value

gb|EL762981.1| AD0196034 Taenia solium UNAM-cd1_adult Taenia ... 1000 0.0

gb|EL761076.1| AD0156016 Taenia solium UNAM-cd1_adult Taenia ... 538 3e-153

gb|EL761026.1| AD0155014 Taenia solium UNAM-cd1_adult Taenia ... 246 2e-65

gb|EL759753.1| AD0128042 Taenia solium UNAM-cd1_adult Taenia ... 119 4e-27

gb|EL744414.1| LV0272029 Taenia solium UNAM-cd2_larva Taenia ... 119 4e-27

Query= TS.seq.screen.trim.Contig229

Length=375

Score E

Sequences producing significant alignments: (Bits) Value

gb|EL762533.1| AD0188023 Taenia solium UNAM-cd1_adult Taenia ... 191 9e-49

gb|EL757705.1| AD0094045 Taenia solium UNAM-cd1_adult Taenia ... 75.0 1e-13

Query= TS.seq.screen.trim.Contig230

Length=524

Score E

Sequences producing significant alignments: (Bits) Value

gb|EL755947.1| AD0028005 Taenia solium UNAM-cd1_adult Taenia ... 931 0.0

gb|EL755875.1| AD0027171 Taenia solium UNAM-cd1_adult Taenia ... 931 0.0

gb|EL755189.1| AD0024206 Taenia solium UNAM-cd1_adult Taenia ... 931 0.0

gb|EL752700.1| AD0014241 Taenia solium UNAM-cd1_adult Taenia ... 852 0.0

gb|EL748588.1| LV0342039 Taenia solium UNAM-cd2_larva Taenia ... 809 0.0

gb|EL752930.1| AD0015189 Taenia solium UNAM-cd1_adult Taenia ... 795 0.0

gb|EL761217.1| AD0159014 Taenia solium UNAM-cd1_adult Taenia ... 773 0.0

gb|EL748446.1| LV0340020 Taenia solium UNAM-cd2_larva Taenia ... 747 0.0

gb|EL748829.1| LV0346019 Taenia solium UNAM-cd2_larva Taenia ... 743 0.0

gb|EL761850.1| AD0172017 Taenia solium UNAM-cd1_adult Taenia ... 737 0.0

Query= TS.seq.screen.trim.Contig231

Length=680

Score E

Sequences producing significant alignments: (Bits) Value

gb|EL744862.1| LV0281022 Taenia solium UNAM-cd2_larva Taenia ... 1181 0.0

gb|EL741406.1| LV0226025 Taenia solium UNAM-cd2_larva Taenia ... 1175 0.0

gb|EL744539.1| LV0275014 Taenia solium UNAM-cd2_larva Taenia ... 1103 0.0

gb|EL744101.1| LV0267040 Taenia solium UNAM-cd2_larva Taenia ... 994 0.0

gb|EL745059.1| LV0285031 Taenia solium UNAM-cd2_larva Taenia ... 977 0.0

gb|EL747269.1| LV0320066 Taenia solium UNAM-cd2_larva Taenia ... 911 0.0

gb|GT227125.1| tscaa0_001530.z1.scf Taenia solium adult full-... 754 0.0

gb|EL758711.1| AD0110020 Taenia solium UNAM-cd1_adult Taenia ... 318 4e-87

gb|EL758158.1| AD0101038 Taenia solium UNAM-cd1_adult Taenia ... 99.0 6e-21

gb|EL741854.1| LV0232059 Taenia solium UNAM-cd2_larva Taenia ... 58.4 1e-08

Query= TS.seq.screen.trim.Contig233

Length=484

Score E

Sequences producing significant alignments: (Bits) Value

gb|EL763283.1| AD0201046 Taenia solium UNAM-cd1_adult Taenia ... 869 0.0

gb|EL746702.1| LV0312063 Taenia solium UNAM-cd2_larva Taenia ... 865 0.0

gb|EL743689.1| LV0260077 Taenia solium UNAM-cd2_larva Taenia ... 863 0.0

gb|EL761502.1| AD0164058 Taenia solium UNAM-cd1_adult Taenia ... 857 0.0

gb|EL745207.1| LV0287061 Taenia solium UNAM-cd2_larva Taenia ... 817 0.0

gb|EL748530.1| LV0341046 Taenia solium UNAM-cd2_larva Taenia ... 815 0.0

gb|EL752904.1| AD0015163 Taenia solium UNAM-cd1_adult Taenia ... 811 0.0

gb|EL751921.1| AD0011164 Taenia solium UNAM-cd1_adult Taenia ... 811 0.0

gb|EL756035.1| AD0028093 Taenia solium UNAM-cd1_adult Taenia ... 804 0.0

gb|EL755752.1| AD0027048 Taenia solium UNAM-cd1_adult Taenia ... 804 0.0

Query= TS.seq.screen.trim.Contig234

Length=321

Score E

Sequences producing significant alignments: (Bits) Value

gb|EL749796.1| AD0002160 Taenia solium UNAM-cd1_adult Taenia ... 265 5e-71

gb|EL750022.1| AD0003164 Taenia solium UNAM-cd1_adult Taenia ... 259 2e-69

gb|EL762211.1| AD0181008 Taenia solium UNAM-cd1_adult Taenia ... 248 5e-66

gb|EL751324.1| AD0009062 Taenia solium UNAM-cd1_adult Taenia ... 246 2e-65

gb|EL759014.1| AD0115039 Taenia solium UNAM-cd1_adult Taenia ... 243 2e-64

gb|EL762298.1| AD0182054 Taenia solium UNAM-cd1_adult Taenia ... 213 2e-55

gb|EL741854.1| LV0232059 Taenia solium UNAM-cd2_larva Taenia ... 56.5 3e-08

Query= TS.seq.screen.trim.Contig235

Length=466

Score E

Sequences producing significant alignments: (Bits) Value

gb|EL759031.1| AD0115056 Taenia solium UNAM-cd1_adult Taenia ... 364 5e-101

Query= TS.seq.screen.trim.Contig236

Length=470

Score E

Sequences producing significant alignments: (Bits) Value

gb|EL740586.1| LV0212022 Taenia solium UNAM-cd2_larva Taenia ... 817 0.0

gb|EL744111.1| LV0267050 Taenia solium UNAM-cd2_larva Taenia ... 813 0.0

gb|EL745779.1| LV0297008 Taenia solium UNAM-cd2_larva Taenia ... 811 0.0

gb|EL745964.1| LV0299053 Taenia solium UNAM-cd2_larva Taenia ... 414 5e-116

gb|EL742750.1| LV0246033 Taenia solium UNAM-cd2_larva Taenia ... 377 7e-105

Query= TS.seq.screen.trim.Contig237

Length=529

Score E

Sequences producing significant alignments: (Bits) Value

gb|EL757426.1| AD0035019 Taenia solium UNAM-cd1_adult Taenia ... 963 0.0

gb|EL756886.1| AD0031159 Taenia solium UNAM-cd1_adult Taenia ... 963 0.0

gb|EL744822.1| LV0280048 Taenia solium UNAM-cd2_larva Taenia ... 963 0.0

gb|EL758903.1| AD0113044 Taenia solium UNAM-cd1_adult Taenia ... 953 0.0

gb|EL762961.1| AD0196014 Taenia solium UNAM-cd1_adult Taenia ... 935 0.0

gb|EL751099.1| AD0008110 Taenia solium UNAM-cd1_adult Taenia ... 917 0.0

gb|EL748381.1| LV0339019 Taenia solium UNAM-cd2_larva Taenia ... 815 0.0

gb|EL762614.1| AD0190016 Taenia solium UNAM-cd1_adult Taenia ... 693 0.0

gb|EL762750.1| AD0192048 Taenia solium UNAM-cd1_adult Taenia ... 593 6e-170

gb|EL746909.1| LV0315051 Taenia solium UNAM-cd2_larva Taenia ... 536 1e-152

Query= TS.seq.screen.trim.Contig240

Length=571

Score E

Sequences producing significant alignments: (Bits) Value

gb|EL760730.1| AD0148008 Taenia solium UNAM-cd1_adult Taenia ... 1037 0.0

gb|EL743429.1| LV0257046 Taenia solium UNAM-cd2_larva Taenia ... 939 0.0

gb|EL762213.1| AD0181010 Taenia solium UNAM-cd1_adult Taenia ... 737 0.0

Query= TS.seq.screen.trim.Contig241

Length=526

Score E

Sequences producing significant alignments: (Bits) Value

gb|EL761083.1| AD0156023 Taenia solium UNAM-cd1_adult Taenia ... 529 2e-150

Query= TS.seq.screen.trim.Contig243

Length=431

Score E

Sequences producing significant alignments: (Bits) Value

gb|FD661577.1| TSEDTS1024B03 Cysti Taenia solium cDNA, mRNA s... 516 1e-146

gb|EX150475.1| TSEDTS1008F06 Cysti Taenia solium cDNA, mRNA s... 307 9e-84

gb|EX150388.1| TSEDTS1008B08 Cysti Taenia solium cDNA, mRNA s... 243 2e-64

gb|EX150532.1| TSEDTS1003D11 Cysti Taenia solium cDNA, mRNA s... 165 6e-41

gb|FD661335.1| TSEDTS1008H04 Cysti Taenia solium cDNA, mRNA s... 158 9e-39

gb|EL745158.1| LV0287012 Taenia solium UNAM-cd2_larva Taenia ... 139 3e-33

Query= TS.seq.screen.trim.Contig244

Length=490

Score E

Sequences producing significant alignments: (Bits) Value

gb|EL745969.1| LV0299058 Taenia solium UNAM-cd2_larva Taenia ... 566 1e-161

gb|EL758056.1| AD0100001 Taenia solium UNAM-cd1_adult Taenia ... 320 1e-87

gb|EL747074.1| LV0318006 Taenia solium UNAM-cd2_larva Taenia ... 102 4e-22

Query= TS.seq.screen.trim.Contig245

Length=543

Score E

Sequences producing significant alignments: (Bits) Value

gb|EL760920.1| AD0152045 Taenia solium UNAM-cd1_adult Taenia ... 843 0.0

gb|EL742021.1| LV0235010 Taenia solium UNAM-cd2_larva Taenia ... 725 0.0

Query= TS.seq.screen.trim.Contig246

Length=466

Score E

Sequences producing significant alignments: (Bits) Value

gb|EL755801.1| AD0027097 Taenia solium UNAM-cd1_adult Taenia ... 845 0.0

gb|EL748173.1| LV0334056 Taenia solium UNAM-cd2_larva Taenia ... 839 0.0

gb|EL742213.1| LV0238032 Taenia solium UNAM-cd2_larva Taenia ... 837 0.0

gb|EL742598.1| LV0244029 Taenia solium UNAM-cd2_larva Taenia ... 821 0.0

gb|EL749147.1| LV0354006 Taenia solium UNAM-cd2_larva Taenia ... 789 0.0

gb|EL758541.1| AD0107034 Taenia solium UNAM-cd1_adult Taenia ... 734 0.0

gb|EL762247.1| AD0182003 Taenia solium UNAM-cd1_adult Taenia ... 662 0.0

gb|EL758171.1| AD0101051 Taenia solium UNAM-cd1_adult Taenia ... 411 6e-115

Query= TS.seq.screen.trim.Contig247

Length=401

Score E

Sequences producing significant alignments: (Bits) Value

gb|EL758441.1| AD0105060 Taenia solium UNAM-cd1_adult Taenia ... 693 0.0

gb|EL762878.1| AD0194049 Taenia solium UNAM-cd1_adult Taenia ... 684 0.0

gb|EL758420.1| AD0105039 Taenia solium UNAM-cd1_adult Taenia ... 682 0.0

gb|EL755529.1| AD0026052 Taenia solium UNAM-cd1_adult Taenia ... 678 0.0

gb|EL745318.1| LV0289030 Taenia solium UNAM-cd2_larva Taenia ... 678 0.0

gb|EL763453.1| AD0204016 Taenia solium UNAM-cd1_adult Taenia ... 675 0.0

gb|EL749765.1| AD0002129 Taenia solium UNAM-cd1_adult Taenia ... 673 0.0

gb|EL762956.1| AD0196009 Taenia solium UNAM-cd1_adult Taenia ... 656 0.0

gb|EL762833.1| AD0194004 Taenia solium UNAM-cd1_adult Taenia ... 630 0.0

gb|EL762156.1| AD0179049 Taenia solium UNAM-cd1_adult Taenia ... 588 3e-168

Query= TS.seq.screen.trim.Contig248

Length=500

Score E

Sequences producing significant alignments: (Bits) Value

gb|EL740690.1| LV0214015 Taenia solium UNAM-cd2_larva Taenia ... 889 0.0

gb|EL741512.1| LV0227068 Taenia solium UNAM-cd2_larva Taenia ... 874 0.0

gb|EL763047.1| AD0197056 Taenia solium UNAM-cd1_adult Taenia ... 870 0.0

gb|EL745475.1| LV0292009 Taenia solium UNAM-cd2_larva Taenia ... 867 0.0

gb|GT227138.1| tscaa0_002708.z1.scf Taenia solium adult full-... 846 0.0

gb|EL763354.1| AD0202054 Taenia solium UNAM-cd1_adult Taenia ... 839 0.0

gb|EL758667.1| AD0109042 Taenia solium UNAM-cd1_adult Taenia ... 839 0.0

gb|EL758290.1| AD0103034 Taenia solium UNAM-cd1_adult Taenia ... 839 0.0

gb|EL746819.1| LV0314032 Taenia solium UNAM-cd2_larva Taenia ... 839 0.0

gb|EL753486.1| AD0018024 Taenia solium UNAM-cd1_adult Taenia ... 833 0.0

Query= TS.seq.screen.trim.Contig249

Length=451

Score E

Sequences producing significant alignments: (Bits) Value

gb|EL742391.1| LV0241017 Taenia solium UNAM-cd2_larva Taenia ... 656 0.0

gb|EL749126.1| LV0353038 Taenia solium UNAM-cd2_larva Taenia ... 579 2e-165

gb|EL748941.1| LV0348052 Taenia solium UNAM-cd2_larva Taenia ... 562 2e-160

gb|EL746378.1| LV0308028 Taenia solium UNAM-cd2_larva Taenia ... 549 1e-156

gb|EL741960.1| LV0234021 Taenia solium UNAM-cd2_larva Taenia ... 549 1e-156

gb|EL741645.1| LV0229056 Taenia solium UNAM-cd2_larva Taenia ... 549 1e-156

gb|EL755189.1| AD0024206 Taenia solium UNAM-cd1_adult Taenia ... 512 2e-145

gb|EL755947.1| AD0028005 Taenia solium UNAM-cd1_adult Taenia ... 488 3e-138

gb|EL755188.1| AD0024205 Taenia solium UNAM-cd1_adult Taenia ... 486 1e-137

gb|EL758765.1| AD0111030 Taenia solium UNAM-cd1_adult Taenia ... 484 4e-137

Query= TS.seq.screen.trim.Contig251

Length=740

Score E

Sequences producing significant alignments: (Bits) Value

gb|EL748632.1| LV0343017 Taenia solium UNAM-cd2_larva Taenia ... 1013 0.0

gb|EL762738.1| AD0192036 Taenia solium UNAM-cd1_adult Taenia ... 121 1e-27

gb|EL741854.1| LV0232059 Taenia solium UNAM-cd2_larva Taenia ... 56.5 3e-08

Query= TS.seq.screen.trim.Contig252

Length=448

Score E

Sequences producing significant alignments: (Bits) Value

gb|EL742412.1| LV0241038 Taenia solium UNAM-cd2_larva Taenia ... 756 0.0

gb|EL741874.1| LV0233007 Taenia solium UNAM-cd2_larva Taenia ... 750 0.0

gb|EL760941.1| AD0153015 Taenia solium UNAM-cd1_adult Taenia ... 747 0.0

gb|EL748281.1| LV0337002 Taenia solium UNAM-cd2_larva Taenia ... 741 0.0

gb|EL762584.1| AD0189041 Taenia solium UNAM-cd1_adult Taenia ... 688 0.0

gb|EL748439.1| LV0340013 Taenia solium UNAM-cd2_larva Taenia ... 510 6e-145

gb|EL744582.1| LV0275057 Taenia solium UNAM-cd2_larva Taenia ... 215 5e-56

Query= TS.seq.screen.trim.Contig254

Length=274

Score E

Sequences producing significant alignments: (Bits) Value

gb|EL761223.1| AD0159020 Taenia solium UNAM-cd1_adult Taenia ... 473 8e-134

Query= TS.seq.screen.trim.Contig255

Length=534

Score E

Sequences producing significant alignments: (Bits) Value

gb|EL747339.1| LV0322016 Taenia solium UNAM-cd2_larva Taenia ... 289 3e-78

gb|EL743732.1| LV0261041 Taenia solium UNAM-cd2_larva Taenia ... 250 1e-66

Query= TS.seq.screen.trim.Contig256

Length=526

Score E

Sequences producing significant alignments: (Bits) Value

gb|EL763084.1| AD0198022 Taenia solium UNAM-cd1_adult Taenia ... 303 1e-82

gb|EL763085.1| AD0198023 Taenia solium UNAM-cd1_adult Taenia ... 292 2e-79

Query= TS.seq.screen.trim.Contig257

Length=447

Score E

Sequences producing significant alignments: (Bits) Value

gb|EL741854.1| LV0232059 Taenia solium UNAM-cd2_larva Taenia ... 60.2 3e-09

Query= TS.seq.screen.trim.Contig258

Length=437

Score E

Sequences producing significant alignments: (Bits) Value

gb|EL742313.1| LV0240008 Taenia solium UNAM-cd2_larva Taenia ... 492 2e-139

gb|EL741902.1| LV0233035 Taenia solium UNAM-cd2_larva Taenia ... 414 5e-116

Query= TS.seq.screen.trim.Contig259

Length=489

Score E

Sequences producing significant alignments: (Bits) Value

gb|EL762993.1| AD0197002 Taenia solium UNAM-cd1_adult Taenia ... 604 3e-173

gb|EL758384.1| AD0105003 Taenia solium UNAM-cd1_adult Taenia ... 604 3e-173

gb|EL749259.1| LV0357003 Taenia solium UNAM-cd2_larva Taenia ... 604 3e-173

gb|EL749167.1| LV0354026 Taenia solium UNAM-cd2_larva Taenia ... 604 3e-173

gb|EL747871.1| LV0330036 Taenia solium UNAM-cd2_larva Taenia ... 604 3e-173

gb|EL747151.1| LV0319010 Taenia solium UNAM-cd2_larva Taenia ... 604 3e-173

gb|EL746871.1| LV0315013 Taenia solium UNAM-cd2_larva Taenia ... 604 3e-173

gb|EL745102.1| LV0286018 Taenia solium UNAM-cd2_larva Taenia ... 604 3e-173

gb|EL743678.1| LV0260066 Taenia solium UNAM-cd2_larva Taenia ... 604 3e-173

gb|EL743656.1| LV0260044 Taenia solium UNAM-cd2_larva Taenia ... 604 3e-173

Query= TS.seq.screen.trim.Contig260

Length=328

Score E

Sequences producing significant alignments: (Bits) Value

gb|EL741854.1| LV0232059 Taenia solium UNAM-cd2_larva Taenia ... 56.5 3e-08

Query= TS.seq.screen.trim.Contig262

Length=1009

Score E

Sequences producing significant alignments: (Bits) Value

gb|EL754841.1| AD0023097 Taenia solium UNAM-cd1_adult Taenia ... 340 9e-94

Query= TS.seq.screen.trim.Contig263

Length=218

Score E

Sequences producing significant alignments: (Bits) Value

gb|EL748810.1| LV0345062 Taenia solium UNAM-cd2_larva Taenia ... 300 1e-81

gb|EL747211.1| LV0320008 Taenia solium UNAM-cd2_larva Taenia ... 300 1e-81

gb|EL743180.1| LV0253027 Taenia solium UNAM-cd2_larva Taenia ... 300 1e-81

gb|EL742207.1| LV0238026 Taenia solium UNAM-cd2_larva Taenia ... 296 2e-80

gb|EL761762.1| AD0170033 Taenia solium UNAM-cd1_adult Taenia ... 261 7e-70

gb|EL761501.1| AD0164057 Taenia solium UNAM-cd1_adult Taenia ... 257 9e-69

gb|EL749050.1| LV0352005 Taenia solium UNAM-cd2_larva Taenia ... 187 1e-47

gb|EL744363.1| LV0271045 Taenia solium UNAM-cd2_larva Taenia ... 178 7e-45

gb|EL740504.1| LV0210028 Taenia solium UNAM-cd2_larva Taenia ... 104 1e-22

gb|EL741854.1| LV0232059 Taenia solium UNAM-cd2_larva Taenia ... 56.5 3e-08

Query= TS.seq.screen.trim.Contig264

Length=325

Score E

Sequences producing significant alignments: (Bits) Value

gb|EL749298.1| LV0358009 Taenia solium UNAM-cd2_larva Taenia ... 486 1e-137

gb|EL748200.1| LV0335015 Taenia solium UNAM-cd2_larva Taenia ... 486 1e-137

gb|EL748676.1| LV0343061 Taenia solium UNAM-cd2_larva Taenia ... 481 5e-136

gb|EL743371.1| LV0256032 Taenia solium UNAM-cd2_larva Taenia ... 481 5e-136

gb|EL758495.1| AD0106038 Taenia solium UNAM-cd1_adult Taenia ... 425 2e-119

gb|EL747526.1| LV0325005 Taenia solium UNAM-cd2_larva Taenia ... 300 1e-81

gb|EL763240.1| AD0201003 Taenia solium UNAM-cd1_adult Taenia ... 239 3e-63

gb|EL763239.1| AD0201002 Taenia solium UNAM-cd1_adult Taenia ... 169 4e-42

gb|EL741854.1| LV0232059 Taenia solium UNAM-cd2_larva Taenia ... 60.2 3e-09

gb|EL740885.1| LV0217068 Taenia solium UNAM-cd2_larva Taenia ... 60.2 3e-09

Query= TS.seq.screen.trim.Contig265

Length=522

Score E

Sequences producing significant alignments: (Bits) Value

gb|EL754841.1| AD0023097 Taenia solium UNAM-cd1_adult Taenia ... 525 2e-149

Query= TS.seq.screen.trim.Contig266

Length=431

Score E

Sequences producing significant alignments: (Bits) Value

gb|EL747828.1| LV0329066 Taenia solium UNAM-cd2_larva Taenia ... 756 0.0

gb|EL748322.1| LV0338011 Taenia solium UNAM-cd2_larva Taenia ... 532 1e-151

Query= TS.seq.screen.trim.Contig267

Length=295

Score E

Sequences producing significant alignments: (Bits) Value

gb|EL741854.1| LV0232059 Taenia solium UNAM-cd2_larva Taenia ... 56.5 3e-08

Query= TS.seq.screen.trim.Contig269

Length=445

Score E

Sequences producing significant alignments: (Bits) Value

gb|EL759427.1| AD0123006 Taenia solium UNAM-cd1_adult Taenia ... 750 0.0

gb|EL759398.1| AD0122042 Taenia solium UNAM-cd1_adult Taenia ... 750 0.0

gb|EL754396.1| AD0021186 Taenia solium UNAM-cd1_adult Taenia ... 750 0.0

gb|EL753153.1| AD0016191 Taenia solium UNAM-cd1_adult Taenia ... 750 0.0

gb|EL749481.1| AD0001104 Taenia solium UNAM-cd1_adult Taenia ... 750 0.0

gb|EL740383.1| LV0208003 Taenia solium UNAM-cd2_larva Taenia ... 750 0.0

gb|EL754380.1| AD0021170 Taenia solium UNAM-cd1_adult Taenia ... 747 0.0

gb|EL757194.1| AD0034010 Taenia solium UNAM-cd1_adult Taenia ... 725 0.0

gb|EL745824.1| LV0297053 Taenia solium UNAM-cd2_larva Taenia ... 411 6e-115

Query= TS.seq.screen.trim.Contig272

Length=284

Score E

Sequences producing significant alignments: (Bits) Value

gb|EL742968.1| LV0250009 Taenia solium UNAM-cd2_larva Taenia ... 475 2e-134

gb|EL748012.1| LV0332034 Taenia solium UNAM-cd2_larva Taenia ... 392 2e-109

gb|EL746875.1| LV0315017 Taenia solium UNAM-cd2_larva Taenia ... 211 7e-55

Query= TS.seq.screen.trim.Contig273

Length=329

Score E

Sequences producing significant alignments: (Bits) Value

gb|EL760928.1| AD0153002 Taenia solium UNAM-cd1_adult Taenia ... 370 1e-102

Query= TS.seq.screen.trim.Contig276

Length=482

Score E

Sequences producing significant alignments: (Bits) Value

gb|EL740930.1| LV0218041 Taenia solium UNAM-cd2_larva Taenia ... 80.5 2e-15

Query= TS.seq.screen.trim.Contig278

Length=641

Score E

Sequences producing significant alignments: (Bits) Value

gb|EL748467.1| LV0340041 Taenia solium UNAM-cd2_larva Taenia ... 248 5e-66

gb|EL742835.1| LV0247053 Taenia solium UNAM-cd2_larva Taenia ... 248 5e-66

gb|EL743899.1| LV0264028 Taenia solium UNAM-cd2_larva Taenia ... 187 1e-47

gb|EL762043.1| AD0177014 Taenia solium UNAM-cd1_adult Taenia ... 97.1 2e-20

gb|EL758392.1| AD0105011 Taenia solium UNAM-cd1_adult Taenia ... 91.6 1e-18

gb|EL762550.1| AD0189007 Taenia solium UNAM-cd1_adult Taenia ... 89.8 3e-18

gb|EL749510.1| AD0001133 Taenia solium UNAM-cd1_adult Taenia ... 89.8 3e-18

gb|EL748293.1| LV0337014 Taenia solium UNAM-cd2_larva Taenia ... 86.1 4e-17

gb|EL762646.1| AD0191007 Taenia solium UNAM-cd1_adult Taenia ... 82.4 6e-16

gb|EL755085.1| AD0024102 Taenia solium UNAM-cd1_adult Taenia ... 75.0 1e-13

Query= TS.seq.screen.trim.Contig279

Length=499

Score E

Sequences producing significant alignments: (Bits) Value

gb|EL748668.1| LV0343053 Taenia solium UNAM-cd2_larva Taenia ... 259 2e-69

gb|EL742806.1| LV0247024 Taenia solium UNAM-cd2_larva Taenia ... 259 2e-69

gb|EL747541.1| LV0325020 Taenia solium UNAM-cd2_larva Taenia ... 257 9e-69

gb|EL763125.1| AD0199009 Taenia solium UNAM-cd1_adult Taenia ... 248 5e-66

gb|EL763017.1| AD0197026 Taenia solium UNAM-cd1_adult Taenia ... 248 5e-66

gb|EL758641.1| AD0109016 Taenia solium UNAM-cd1_adult Taenia ... 248 5e-66

gb|EL757893.1| AD0097036 Taenia solium UNAM-cd1_adult Taenia ... 248 5e-66

gb|EL755070.1| AD0024087 Taenia solium UNAM-cd1_adult Taenia ... 248 5e-66

gb|EL758425.1| AD0105044 Taenia solium UNAM-cd1_adult Taenia ... 246 2e-65

gb|EL757831.1| AD0096034 Taenia solium UNAM-cd1_adult Taenia ... 235 4e-62

Query= TS.seq.screen.trim.Contig280

Length=497

Score E

Sequences producing significant alignments: (Bits) Value

gb|EL743470.1| LV0258010 Taenia solium UNAM-cd2_larva Taenia ... 440 8e-124

gb|EL741737.1| LV0231015 Taenia solium UNAM-cd2_larva Taenia ... 303 1e-82

gb|EL741733.1| LV0231011 Taenia solium UNAM-cd2_larva Taenia ... 303 1e-82

gb|EL741528.1| LV0228010 Taenia solium UNAM-cd2_larva Taenia ... 270 1e-72

gb|EL741891.1| LV0233024 Taenia solium UNAM-cd2_larva Taenia ... 226 3e-59

gb|EL761964.1| AD0175010 Taenia solium UNAM-cd1_adult Taenia ... 187 1e-47

gb|EL740584.1| LV0212020 Taenia solium UNAM-cd2_larva Taenia ... 132 6e-31

gb|EL741854.1| LV0232059 Taenia solium UNAM-cd2_larva Taenia ... 56.5 3e-08

gb|EL741314.1| LV0224034 Taenia solium UNAM-cd2_larva Taenia ... 52.8 4e-07

Query= TS.seq.screen.trim.Contig285

Length=460

Score E

Sequences producing significant alignments: (Bits) Value

gb|EL747208.1| LV0320005 Taenia solium UNAM-cd2_larva Taenia ... 603 1e-172

gb|EL741067.1| LV0220040 Taenia solium UNAM-cd2_larva Taenia ... 603 1e-172

gb|EL753819.1| AD0019106 Taenia solium UNAM-cd1_adult Taenia ... 599 1e-171

gb|EL752555.1| AD0014096 Taenia solium UNAM-cd1_adult Taenia ... 599 1e-171

gb|EL752502.1| AD0014043 Taenia solium UNAM-cd1_adult Taenia ... 599 1e-171

gb|EL755196.1| AD0024213 Taenia solium UNAM-cd1_adult Taenia ... 593 6e-170

gb|EL761488.1| AD0164044 Taenia solium UNAM-cd1_adult Taenia ... 592 2e-169

gb|EL755194.1| AD0024211 Taenia solium UNAM-cd1_adult Taenia ... 590 8e-169

gb|EL761941.1| AD0174020 Taenia solium UNAM-cd1_adult Taenia ... 588 3e-168

gb|EL752813.1| AD0015072 Taenia solium UNAM-cd1_adult Taenia ... 582 1e-166

Query= TS.seq.screen.trim.Contig289

Length=747

Score E

Sequences producing significant alignments: (Bits) Value

gb|EL741285.1| LV0224005 Taenia solium UNAM-cd2_larva Taenia ... 538 3e-153

Query= TS.seq.screen.trim.Contig290

Length=568

Score E

Sequences producing significant alignments: (Bits) Value

gb|EL747543.1| LV0325022 Taenia solium UNAM-cd2_larva Taenia ... 632 0.0

Query= TS.seq.screen.trim.Contig292

Length=622

Score E

Sequences producing significant alignments: (Bits) Value

gb|EL741115.1| LV0221016 Taenia solium UNAM-cd2_larva Taenia ... 641 0.0

gb|EL740254.1| LV0205034 Taenia solium UNAM-cd2_larva Taenia ... 438 3e-123

gb|EL741854.1| LV0232059 Taenia solium UNAM-cd2_larva Taenia ... 60.2 3e-09

Query= TS.seq.screen.trim.Contig293

Length=743

Score E

Sequences producing significant alignments: (Bits) Value

gb|EL742816.1| LV0247034 Taenia solium UNAM-cd2_larva Taenia ... 686 0.0

gb|EL740643.1| LV0213025 Taenia solium UNAM-cd2_larva Taenia ... 686 0.0

gb|EL744430.1| LV0272045 Taenia solium UNAM-cd2_larva Taenia ... 588 3e-168

Query= TS.seq.screen.trim.Contig294

Length=451

Score E

Sequences producing significant alignments: (Bits) Value

gb|EL740277.1| LV0206009 Taenia solium UNAM-cd2_larva Taenia ... 763 0.0

gb|EL743616.1| LV0260004 Taenia solium UNAM-cd2_larva Taenia ... 754 0.0

gb|EL745390.1| LV0290045 Taenia solium UNAM-cd2_larva Taenia ... 638 0.0

gb|EL745234.1| LV0288016 Taenia solium UNAM-cd2_larva Taenia ... 483 1e-136

gb|EL747141.1| LV0318073 Taenia solium UNAM-cd2_larva Taenia ... 292 2e-79

gb|EL745233.1| LV0288015 Taenia solium UNAM-cd2_larva Taenia ... 263 2e-70

Query= TS.seq.screen.trim.Contig295

Length=537

Score E

Sequences producing significant alignments: (Bits) Value

gb|EL744106.1| LV0267045 Taenia solium UNAM-cd2_larva Taenia ... 769 0.0

gb|EL742967.1| LV0250008 Taenia solium UNAM-cd2_larva Taenia ... 577 6e-165

gb|EL743301.1| LV0255015 Taenia solium UNAM-cd2_larva Taenia ... 556 8e-159

gb|EL745892.1| LV0298049 Taenia solium UNAM-cd2_larva Taenia ... 551 4e-157

gb|EL740459.1| LV0209037 Taenia solium UNAM-cd2_larva Taenia ... 479 2e-135

gb|EL743336.1| LV0255050 Taenia solium UNAM-cd2_larva Taenia ... 383 1e-106

gb|EL746395.1| LV0308045 Taenia solium UNAM-cd2_larva Taenia ... 333 1e-91

Query= TS.seq.screen.trim.Contig297

Length=482

Score E

Sequences producing significant alignments: (Bits) Value

gb|EL754319.1| AD0021109 Taenia solium UNAM-cd1_adult Taenia ... 623 8e-179

gb|EL748900.1| LV0348011 Taenia solium UNAM-cd2_larva Taenia ... 571 3e-163

Query= TS.seq.screen.trim.Contig299

Length=712

Score E

Sequences producing significant alignments: (Bits) Value

gb|EL745321.1| LV0289033 Taenia solium UNAM-cd2_larva Taenia ... 375 2e-104

Query= TS.seq.screen.trim.Contig300

Length=603

Score E

Sequences producing significant alignments: (Bits) Value

gb|EX150879.1| TSEDTS1024G12 Cysti Taenia solium cDNA, mRNA s... 219 4e-57

Query= TS.seq.screen.trim.Contig301

Length=737

Score E

Sequences producing significant alignments: (Bits) Value

gb|EL756348.1| AD0029143 Taenia solium UNAM-cd1_adult Taenia ... 1280 0.0

gb|EL758009.1| AD0099021 Taenia solium UNAM-cd1_adult Taenia ... 1118 0.0

Query= TS.seq.screen.trim.Contig302

Length=662

Score E

Sequences producing significant alignments: (Bits) Value

gb|EL751997.1| AD0012025 Taenia solium UNAM-cd1_adult Taenia ... 1160 0.0

gb|EL749182.1| LV0354041 Taenia solium UNAM-cd2_larva Taenia ... 1011 0.0

gb|EL762955.1| AD0196008 Taenia solium UNAM-cd1_adult Taenia ... 1005 0.0

gb|EL744680.1| LV0277057 Taenia solium UNAM-cd2_larva Taenia ... 994 0.0

gb|EL762142.1| AD0179035 Taenia solium UNAM-cd1_adult Taenia ... 961 0.0

gb|EL763155.1| AD0199039 Taenia solium UNAM-cd1_adult Taenia ... 828 0.0

gb|EL752256.1| AD0013023 Taenia solium UNAM-cd1_adult Taenia ... 734 0.0

gb|EL762400.1| AD0185005 Taenia solium UNAM-cd1_adult Taenia ... 695 0.0

gb|EL743859.1| LV0263028 Taenia solium UNAM-cd2_larva Taenia ... 459 2e-129

Query= TS.seq.screen.trim.Contig303

Length=691

Score E

Sequences producing significant alignments: (Bits) Value

gb|EL758027.1| AD0099039 Taenia solium UNAM-cd1_adult Taenia ... 597 5e-171

Query= TS.seq.screen.trim.Contig304

Length=797

Score E

Sequences producing significant alignments: (Bits) Value

gb|EL754352.1| AD0021142 Taenia solium UNAM-cd1_adult Taenia ... 1367 0.0

gb|EL749157.1| LV0354016 Taenia solium UNAM-cd2_larva Taenia ... 1236 0.0

gb|EL748371.1| LV0339009 Taenia solium UNAM-cd2_larva Taenia ... 1230 0.0

gb|EL749109.1| LV0353021 Taenia solium UNAM-cd2_larva Taenia ... 1177 0.0

gb|EL751822.1| AD0011065 Taenia solium UNAM-cd1_adult Taenia ... 981 0.0

gb|EL741352.1| LV0225003 Taenia solium UNAM-cd2_larva Taenia ... 944 0.0

gb|EL747047.1| LV0317044 Taenia solium UNAM-cd2_larva Taenia ... 798 0.0

gb|EL749315.1| LV0358026 Taenia solium UNAM-cd2_larva Taenia ... 736 0.0

gb|EL760916.1| AD0152041 Taenia solium UNAM-cd1_adult Taenia ... 508 2e-144

gb|EL740826.1| LV0217009 Taenia solium UNAM-cd2_larva Taenia ... 412 2e-115

Query= TS.seq.screen.trim.Contig305

Length=741

Score E

Sequences producing significant alignments: (Bits) Value

gb|EL755971.1| AD0028029 Taenia solium UNAM-cd1_adult Taenia ... 1081 0.0

Query= TS.seq.screen.trim.Contig307

Length=902

Score E

Sequences producing significant alignments: (Bits) Value

gb|EL751593.1| AD0010086 Taenia solium UNAM-cd1_adult Taenia ... 1018 0.0

gb|EL756654.1| AD0030186 Taenia solium UNAM-cd1_adult Taenia ... 1016 0.0

gb|EL752637.1| AD0014178 Taenia solium UNAM-cd1_adult Taenia ... 941 0.0

gb|EL755029.1| AD0024046 Taenia solium UNAM-cd1_adult Taenia ... 852 0.0

gb|EL759060.1| AD0116023 Taenia solium UNAM-cd1_adult Taenia ... 765 0.0

gb|EL757012.1| AD0032024 Taenia solium UNAM-cd1_adult Taenia ... 763 0.0

gb|EL754968.1| AD0023224 Taenia solium UNAM-cd1_adult Taenia ... 763 0.0

gb|EL755561.1| AD0026084 Taenia solium UNAM-cd1_adult Taenia ... 747 0.0

gb|EL751831.1| AD0011074 Taenia solium UNAM-cd1_adult Taenia ... 645 0.0

gb|EL752883.1| AD0015142 Taenia solium UNAM-cd1_adult Taenia ... 628 2e-180

Query= TS.seq.screen.trim.Contig308

Length=471

Score E

Sequences producing significant alignments: (Bits) Value

gb|EL740951.1| LV0219001 Taenia solium UNAM-cd2_larva Taenia ... 278 7e-75

gb|EL740799.1| LV0216033 Taenia solium UNAM-cd2_larva Taenia ... 278 7e-75

gb|EL758712.1| AD0110021 Taenia solium UNAM-cd1_adult Taenia ... 265 5e-71

gb|EL743869.1| LV0263038 Taenia solium UNAM-cd2_larva Taenia ... 215 5e-56

Query= TS.seq.screen.trim.Contig310

Length=508

Score E

Sequences producing significant alignments: (Bits) Value

gb|EL757550.1| AD0035143 Taenia solium UNAM-cd1_adult Taenia ... 880 0.0

gb|EL756621.1| AD0030153 Taenia solium UNAM-cd1_adult Taenia ... 880 0.0

gb|EL755818.1| AD0027114 Taenia solium UNAM-cd1_adult Taenia ... 880 0.0

gb|EL754236.1| AD0021026 Taenia solium UNAM-cd1_adult Taenia ... 880 0.0

gb|GT227139.1| tscaa0_000819.z1.scf Taenia solium adult full-... 826 0.0

gb|EL755514.1| AD0026037 Taenia solium UNAM-cd1_adult Taenia ... 758 0.0

gb|EL752223.1| AD0012251 Taenia solium UNAM-cd1_adult Taenia ... 758 0.0

gb|EL759010.1| AD0115035 Taenia solium UNAM-cd1_adult Taenia ... 747 0.0

gb|EL751391.1| AD0009129 Taenia solium UNAM-cd1_adult Taenia ... 717 0.0

gb|EL744616.1| LV0276034 Taenia solium UNAM-cd2_larva Taenia ... 699 0.0

Query= TS.seq.screen.trim.Contig311

Length=456

Score E

Sequences producing significant alignments: (Bits) Value

gb|EL761811.1| AD0171028 Taenia solium UNAM-cd1_adult Taenia ... 163 2e-40

gb|EL761255.1| AD0160006 Taenia solium UNAM-cd1_adult Taenia ... 163 2e-40

gb|EL761060.1| AD0155048 Taenia solium UNAM-cd1_adult Taenia ... 163 2e-40

gb|EL744814.1| LV0280040 Taenia solium UNAM-cd2_larva Taenia ... 163 2e-40

gb|EL742074.1| LV0236003 Taenia solium UNAM-cd2_larva Taenia ... 163 2e-40

gb|EL745839.1| LV0297068 Taenia solium UNAM-cd2_larva Taenia ... 148 6e-36

gb|EL761574.1| AD0166015 Taenia solium UNAM-cd1_adult Taenia ... 108 9e-24

gb|EL745495.1| LV0292029 Taenia solium UNAM-cd2_larva Taenia ... 71.3 1e-12

gb|EL754425.1| AD0021215 Taenia solium UNAM-cd1_adult Taenia ... 65.8 6e-11

Query= TS.seq.screen.trim.Contig312

Length=615

Score E

Sequences producing significant alignments: (Bits) Value

gb|EL759795.1| AD0129035 Taenia solium UNAM-cd1_adult Taenia ... 134 2e-31

Query= TS.seq.screen.trim.Contig313

Length=445

Score E

Sequences producing significant alignments: (Bits) Value

gb|EL748462.1| LV0340036 Taenia solium UNAM-cd2_larva Taenia ... 710 0.0

gb|EL741164.1| LV0221065 Taenia solium UNAM-cd2_larva Taenia ... 667 0.0

gb|EL744522.1| LV0274025 Taenia solium UNAM-cd2_larva Taenia ... 652 0.0

gb|EL762769.1| AD0193016 Taenia solium UNAM-cd1_adult Taenia ... 588 3e-168

gb|EL758106.1| AD0100051 Taenia solium UNAM-cd1_adult Taenia ... 575 2e-164

gb|EL759683.1| AD0127024 Taenia solium UNAM-cd1_adult Taenia ... 564 5e-161

gb|EL740513.1| LV0210037 Taenia solium UNAM-cd2_larva Taenia ... 497 5e-141

gb|EL760877.1| AD0152002 Taenia solium UNAM-cd1_adult Taenia ... 394 6e-110

gb|EL761116.1| AD0157012 Taenia solium UNAM-cd1_adult Taenia ... 390 8e-109

gb|EL759967.1| AD0133012 Taenia solium UNAM-cd1_adult Taenia ... 387 1e-107

Query= TS.seq.screen.trim.Contig314

Length=578

Score E

Sequences producing significant alignments: (Bits) Value

gb|EL747452.1| LV0323061 Taenia solium UNAM-cd2_larva Taenia ... 566 1e-161

gb|EL741854.1| LV0232059 Taenia solium UNAM-cd2_larva Taenia ... 56.5 3e-08

Query= TS.seq.screen.trim.Contig315

Length=638

Score E

Sequences producing significant alignments: (Bits) Value

gb|EL756711.1| AD0030243 Taenia solium UNAM-cd1_adult Taenia ... 1083 0.0

gb|EL756125.1| AD0028183 Taenia solium UNAM-cd1_adult Taenia ... 929 0.0

gb|EL763296.1| AD0201059 Taenia solium UNAM-cd1_adult Taenia ... 795 0.0

gb|EL740987.1| LV0219037 Taenia solium UNAM-cd2_larva Taenia ... 763 0.0

gb|EL762321.1| AD0183022 Taenia solium UNAM-cd1_adult Taenia ... 654 0.0

gb|EL747552.1| LV0325031 Taenia solium UNAM-cd2_larva Taenia ... 643 0.0

gb|EL741854.1| LV0232059 Taenia solium UNAM-cd2_larva Taenia ... 56.5 3e-08

Query= TS.seq.screen.trim.Contig316

Length=830

Score E

Sequences producing significant alignments: (Bits) Value

gb|EL758189.1| AD0101069 Taenia solium UNAM-cd1_adult Taenia ... 553 1e-157

Query= TS.seq.screen.trim.Contig317

Length=955

Score E

Sequences producing significant alignments: (Bits) Value

gb|EL750944.1| AD0007180 Taenia solium UNAM-cd1_adult Taenia ... 1158 0.0

gb|EL750713.1| AD0006152 Taenia solium UNAM-cd1_adult Taenia ... 950 0.0

gb|EL761147.1| AD0157043 Taenia solium UNAM-cd1_adult Taenia ... 634 0.0

Query= TS.seq.screen.trim.Contig318

Length=395

Score E

Sequences producing significant alignments: (Bits) Value

gb|EL746512.1| LV0310009 Taenia solium UNAM-cd2_larva Taenia ... 446 2e-125

gb|EL746273.1| LV0306024 Taenia solium UNAM-cd2_larva Taenia ... 355 3e-98

Query= TS.seq.screen.trim.Contig320

Length=251

Score E

Sequences producing significant alignments: (Bits) Value

gb|EL745478.1| LV0292012 Taenia solium UNAM-cd2_larva Taenia ... 279 2e-75

gb|EL745744.1| LV0296034 Taenia solium UNAM-cd2_larva Taenia ... 270 1e-72

gb|EL742765.1| LV0246048 Taenia solium UNAM-cd2_larva Taenia ... 270 1e-72

gb|EL742090.1| LV0236019 Taenia solium UNAM-cd2_larva Taenia ... 270 1e-72

gb|EL745757.1| LV0296047 Taenia solium UNAM-cd2_larva Taenia ... 268 4e-72

gb|EL740740.1| LV0215030 Taenia solium UNAM-cd2_larva Taenia ... 265 5e-71

gb|EL761479.1| AD0164035 Taenia solium UNAM-cd1_adult Taenia ... 198 5e-51

gb|EL742283.1| LV0239035 Taenia solium UNAM-cd2_larva Taenia ... 193 3e-49

gb|EL746330.1| LV0307044 Taenia solium UNAM-cd2_larva Taenia ... 161 7e-40

gb|EL741780.1| LV0231058 Taenia solium UNAM-cd2_larva Taenia ... 93.5 3e-19

Query= TS.seq.screen.trim.Contig322

Length=531

Score E

Sequences producing significant alignments: (Bits) Value

gb|EL748055.1| LV0333006 Taenia solium UNAM-cd2_larva Taenia ... 793 0.0

gb|EL741665.1| LV0230012 Taenia solium UNAM-cd2_larva Taenia ... 793 0.0

gb|EL741207.1| LV0222040 Taenia solium UNAM-cd2_larva Taenia ... 780 0.0

gb|EL747296.1| LV0321027 Taenia solium UNAM-cd2_larva Taenia ... 778 0.0

gb|EL743379.1| LV0256040 Taenia solium UNAM-cd2_larva Taenia ... 778 0.0

gb|EL742488.1| LV0242052 Taenia solium UNAM-cd2_larva Taenia ... 778 0.0

gb|EL747162.1| LV0319021 Taenia solium UNAM-cd2_larva Taenia ... 776 0.0

gb|EL745219.1| LV0288001 Taenia solium UNAM-cd2_larva Taenia ... 776 0.0

gb|EL747078.1| LV0318010 Taenia solium UNAM-cd2_larva Taenia ... 771 0.0

gb|EL746196.1| LV0304021 Taenia solium UNAM-cd2_larva Taenia ... 771 0.0

Query= TS.seq.screen.trim.Contig323

Length=418

Score E

Sequences producing significant alignments: (Bits) Value

gb|EL741854.1| LV0232059 Taenia solium UNAM-cd2_larva Taenia ... 56.5 3e-08

Query= TS.seq.screen.trim.Contig324

Length=920

Score E

Sequences producing significant alignments: (Bits) Value

gb|EL745649.1| LV0295007 Taenia solium UNAM-cd2_larva Taenia ... 1024 0.0

gb|EL745524.1| LV0293002 Taenia solium UNAM-cd2_larva Taenia ... 996 0.0

gb|EL745002.1| LV0284033 Taenia solium UNAM-cd2_larva Taenia ... 974 0.0

gb|EL748024.1| LV0332046 Taenia solium UNAM-cd2_larva Taenia ... 948 0.0

gb|EL748061.1| LV0333012 Taenia solium UNAM-cd2_larva Taenia ... 922 0.0

gb|EL743779.1| LV0262020 Taenia solium UNAM-cd2_larva Taenia ... 896 0.0

gb|EL740945.1| LV0218056 Taenia solium UNAM-cd2_larva Taenia ... 891 0.0

gb|EL744879.1| LV0282005 Taenia solium UNAM-cd2_larva Taenia ... 880 0.0

gb|EL742678.1| LV0245033 Taenia solium UNAM-cd2_larva Taenia ... 880 0.0

gb|EL746653.1| LV0312014 Taenia solium UNAM-cd2_larva Taenia ... 865 0.0

Query= TS.seq.screen.trim.Contig326

Length=823

Score E

Sequences producing significant alignments: (Bits) Value

gb|EL741854.1| LV0232059 Taenia solium UNAM-cd2_larva Taenia ... 58.4 1e-08

Query= TS.seq.screen.trim.Contig327

Length=377

Score E

Sequences producing significant alignments: (Bits) Value

gb|EL756658.1| AD0030190 Taenia solium UNAM-cd1_adult Taenia ... 588 3e-168

gb|EL749835.1| AD0002199 Taenia solium UNAM-cd1_adult Taenia ... 588 3e-168

gb|EL755611.1| AD0026134 Taenia solium UNAM-cd1_adult Taenia ... 582 1e-166

gb|EL750058.1| AD0003200 Taenia solium UNAM-cd1_adult Taenia ... 562 2e-160

gb|EL757841.1| AD0096044 Taenia solium UNAM-cd1_adult Taenia ... 497 5e-141

gb|EL755499.1| AD0026022 Taenia solium UNAM-cd1_adult Taenia ... 497 5e-141

Query= TS.seq.screen.trim.Contig328

Length=710

Score E

Sequences producing significant alignments: (Bits) Value

gb|EL748263.1| LV0336039 Taenia solium UNAM-cd2_larva Taenia ... 1046 0.0

gb|EL740626.1| LV0213008 Taenia solium UNAM-cd2_larva Taenia ... 898 0.0

gb|EL744071.1| LV0267010 Taenia solium UNAM-cd2_larva Taenia ... 584 4e-167

gb|EL749164.1| LV0354023 Taenia solium UNAM-cd2_larva Taenia ... 529 2e-150

gb|EL761817.1| AD0171034 Taenia solium UNAM-cd1_adult Taenia ... 518 4e-147

gb|EL740986.1| LV0219036 Taenia solium UNAM-cd2_larva Taenia ... 392 2e-109

gb|EL756139.1| AD0028197 Taenia solium UNAM-cd1_adult Taenia ... 106 3e-23

Query= TS.seq.screen.trim.Contig330

Length=698

Score E

Sequences producing significant alignments: (Bits) Value

gb|EL741577.1| LV0228059 Taenia solium UNAM-cd2_larva Taenia ... 628 2e-180

gb|EL741308.1| LV0224028 Taenia solium UNAM-cd2_larva Taenia ... 412 2e-115

gb|EL745924.1| LV0299013 Taenia solium UNAM-cd2_larva Taenia ... 407 8e-114

gb|EL745854.1| LV0298011 Taenia solium UNAM-cd2_larva Taenia ... 313 2e-85

gb|EL746872.1| LV0315014 Taenia solium UNAM-cd2_larva Taenia ... 292 2e-79

Query= TS.seq.screen.trim.Contig331

Length=359

Score E

Sequences producing significant alignments: (Bits) Value

gb|EL752615.1| AD0014156 Taenia solium UNAM-cd1_adult Taenia ... 608 2e-174

gb|EL755749.1| AD0027045 Taenia solium UNAM-cd1_adult Taenia ... 545 2e-155

gb|EL755646.1| AD0026169 Taenia solium UNAM-cd1_adult Taenia ... 545 2e-155

gb|EL760458.1| AD0142056 Taenia solium UNAM-cd1_adult Taenia ... 508 2e-144

gb|EL744204.1| LV0269015 Taenia solium UNAM-cd2_larva Taenia ... 409 2e-114

gb|EL754025.1| AD0020070 Taenia solium UNAM-cd1_adult Taenia ... 287 1e-77

gb|EL758454.1| AD0105073 Taenia solium UNAM-cd1_adult Taenia ... 248 5e-66

Query= TS.seq.screen.trim.Contig334

Length=767

Score E

Sequences producing significant alignments: (Bits) Value

gb|EL742649.1| LV0245004 Taenia solium UNAM-cd2_larva Taenia ... 688 0.0

gb|EL742803.1| LV0247021 Taenia solium UNAM-cd2_larva Taenia ... 628 2e-180

gb|EL744970.1| LV0284001 Taenia solium UNAM-cd2_larva Taenia ... 586 1e-167

gb|EL743513.1| LV0258053 Taenia solium UNAM-cd2_larva Taenia ... 569 1e-162

gb|EL751005.1| AD0008016 Taenia solium UNAM-cd1_adult Taenia ... 499 1e-141

gb|EL748975.1| LV0349024 Taenia solium UNAM-cd2_larva Taenia ... 464 5e-131

gb|EL744670.1| LV0277047 Taenia solium UNAM-cd2_larva Taenia ... 366 1e-101

gb|EL745180.1| LV0287034 Taenia solium UNAM-cd2_larva Taenia ... 329 2e-90

gb|EL757273.1| AD0034089 Taenia solium UNAM-cd1_adult Taenia ... 315 5e-86

gb|EL746494.1| LV0309064 Taenia solium UNAM-cd2_larva Taenia ... 255 3e-68

Query= TS.seq.screen.trim.Contig335

Length=883

Score E

Sequences producing significant alignments: (Bits) Value

gb|GT227874.1| tscaa0_002633.z1.scf Taenia solium adult full-... 241 9e-64

Query= TS.seq.screen.trim.Contig336

Length=630

Score E

Sequences producing significant alignments: (Bits) Value

gb|EL741287.1| LV0224007 Taenia solium UNAM-cd2_larva Taenia ... 444 6e-125

Query= TS.seq.screen.trim.Contig338

Length=555

Score E

Sequences producing significant alignments: (Bits) Value

gb|EL755854.1| AD0027150 Taenia solium UNAM-cd1_adult Taenia ... 974 0.0

gb|EL752971.1| AD0016009 Taenia solium UNAM-cd1_adult Taenia ... 970 0.0

gb|EL757624.1| AD0035217 Taenia solium UNAM-cd1_adult Taenia ... 959 0.0

gb|EL755103.1| AD0024120 Taenia solium UNAM-cd1_adult Taenia ... 957 0.0

gb|EL754675.1| AD0022200 Taenia solium UNAM-cd1_adult Taenia ... 957 0.0

gb|EL754171.1| AD0020216 Taenia solium UNAM-cd1_adult Taenia ... 957 0.0

gb|EL753139.1| AD0016177 Taenia solium UNAM-cd1_adult Taenia ... 957 0.0

gb|EL757626.1| AD0035219 Taenia solium UNAM-cd1_adult Taenia ... 955 0.0

gb|EL757373.1| AD0034189 Taenia solium UNAM-cd1_adult Taenia ... 953 0.0

gb|EL754457.1| AD0021247 Taenia solium UNAM-cd1_adult Taenia ... 952 0.0

Query= TS.seq.screen.trim.Contig339

Length=660

Score E

Sequences producing significant alignments: (Bits) Value

gb|EL748546.1| LV0341062 Taenia solium UNAM-cd2_larva Taenia ... 780 0.0

gb|EL748760.1| LV0345012 Taenia solium UNAM-cd2_larva Taenia ... 477 6e-135

gb|EL744273.1| LV0270020 Taenia solium UNAM-cd2_larva Taenia ... 292 2e-79

gb|EL746999.1| LV0316076 Taenia solium UNAM-cd2_larva Taenia ... 189 3e-48

gb|EL741854.1| LV0232059 Taenia solium UNAM-cd2_larva Taenia ... 56.5 3e-08

Query= TS.seq.screen.trim.Contig341

Length=910

Score E

Sequences producing significant alignments: (Bits) Value

gb|EL746502.1| LV0309072 Taenia solium UNAM-cd2_larva Taenia ... 584 4e-167

gb|EL741123.1| LV0221024 Taenia solium UNAM-cd2_larva Taenia ... 484 4e-137

gb|EL741615.1| LV0229026 Taenia solium UNAM-cd2_larva Taenia ... 401 4e-112

gb|EL740338.1| LV0207022 Taenia solium UNAM-cd2_larva Taenia ... 246 2e-65

gb|EL758129.1| AD0101009 Taenia solium UNAM-cd1_adult Taenia ... 230 2e-60

gb|EL741959.1| LV0234020 Taenia solium UNAM-cd2_larva Taenia ... 224 9e-59

gb|EL745600.1| LV0294015 Taenia solium UNAM-cd2_larva Taenia ... 217 2e-56

gb|EL745015.1| LV0284046 Taenia solium UNAM-cd2_larva Taenia ... 167 2e-41

Query= TS.seq.screen.trim.Contig342

Length=460

Score E

Sequences producing significant alignments: (Bits) Value

gb|EL742887.1| LV0249002 Taenia solium UNAM-cd2_larva Taenia ... 826 0.0

gb|EL762641.1| AD0191002 Taenia solium UNAM-cd1_adult Taenia ... 813 0.0

gb|EL758561.1| AD0107054 Taenia solium UNAM-cd1_adult Taenia ... 809 0.0

gb|EL752084.1| AD0012112 Taenia solium UNAM-cd1_adult Taenia ... 809 0.0

gb|EL742947.1| LV0249062 Taenia solium UNAM-cd2_larva Taenia ... 809 0.0

gb|EL752229.1| AD0012257 Taenia solium UNAM-cd1_adult Taenia ... 804 0.0

gb|EL752060.1| AD0012088 Taenia solium UNAM-cd1_adult Taenia ... 804 0.0

gb|EL752453.1| AD0013220 Taenia solium UNAM-cd1_adult Taenia ... 782 0.0

gb|EL763323.1| AD0202023 Taenia solium UNAM-cd1_adult Taenia ... 750 0.0

gb|EL752310.1| AD0013077 Taenia solium UNAM-cd1_adult Taenia ... 741 0.0

Query= TS.seq.screen.trim.Contig343

Length=801

Score E

Sequences producing significant alignments: (Bits) Value

gb|EL741854.1| LV0232059 Taenia solium UNAM-cd2_larva Taenia ... 58.4 1e-08

Query= TS.seq.screen.trim.Contig344

Length=590

Score E

Sequences producing significant alignments: (Bits) Value

gb|EL760921.1| AD0152046 Taenia solium UNAM-cd1_adult Taenia ... 861 0.0

gb|EL749269.1| LV0357013 Taenia solium UNAM-cd2_larva Taenia ... 752 0.0

gb|EL744556.1| LV0275031 Taenia solium UNAM-cd2_larva Taenia ... 300 1e-81

gb|EL745123.1| LV0286040 Taenia solium UNAM-cd2_larva Taenia ... 252 4e-67

Query= TS.seq.screen.trim.Contig345

Length=488

Score E

Sequences producing significant alignments: (Bits) Value

gb|EL758189.1| AD0101069 Taenia solium UNAM-cd1_adult Taenia ... 414 5e-116

Query= TS.seq.screen.trim.Contig347

Length=422

Score E

Sequences producing significant alignments: (Bits) Value

gb|EL749550.1| AD0001173 Taenia solium UNAM-cd1_adult Taenia ... 667 0.0

Query= TS.seq.screen.trim.Contig348

Length=858

Score E

Sequences producing significant alignments: (Bits) Value

gb|EL743347.1| LV0256008 Taenia solium UNAM-cd2_larva Taenia ... 250 1e-66

Query= TS.seq.screen.trim.Contig349

Length=446

Score E

Sequences producing significant alignments: (Bits) Value

gb|EL751772.1| AD0011015 Taenia solium UNAM-cd1_adult Taenia ... 726 0.0

gb|EL751527.1| AD0010020 Taenia solium UNAM-cd1_adult Taenia ... 726 0.0

gb|EL746281.1| LV0306032 Taenia solium UNAM-cd2_larva Taenia ... 244 7e-65

Query= TS.seq.screen.trim.Contig353

Length=260

Score E

Sequences producing significant alignments: (Bits) Value

gb|EL749796.1| AD0002160 Taenia solium UNAM-cd1_adult Taenia ... 231 5e-61

gb|EL750022.1| AD0003164 Taenia solium UNAM-cd1_adult Taenia ... 226 3e-59

gb|EL762211.1| AD0181008 Taenia solium UNAM-cd1_adult Taenia ... 215 5e-56

gb|EL751324.1| AD0009062 Taenia solium UNAM-cd1_adult Taenia ... 213 2e-55

gb|EL759014.1| AD0115039 Taenia solium UNAM-cd1_adult Taenia ... 209 3e-54

gb|EL762298.1| AD0182054 Taenia solium UNAM-cd1_adult Taenia ... 180 2e-45

Query= TS.seq.screen.trim.Contig354

Length=280

Score E

Sequences producing significant alignments: (Bits) Value

gb|EL758465.1| AD0106008 Taenia solium UNAM-cd1_adult Taenia ... 255 3e-68

gb|EL744398.1| LV0272013 Taenia solium UNAM-cd2_larva Taenia ... 243 2e-64

gb|EL758117.1| AD0100062 Taenia solium UNAM-cd1_adult Taenia ... 239 3e-63

gb|EL743297.1| LV0255011 Taenia solium UNAM-cd2_larva Taenia ... 182 5e-46

gb|EL758859.1| AD0112058 Taenia solium UNAM-cd1_adult Taenia ... 161 7e-40

gb|EX150988.1| TSEDTS1028B09 Cysti Taenia solium cDNA, mRNA s... 156 3e-38

gb|EL740371.1| LV0207055 Taenia solium UNAM-cd2_larva Taenia ... 93.5 3e-19

gb|EL741854.1| LV0232059 Taenia solium UNAM-cd2_larva Taenia ... 63.9 2e-10

Query= TS.seq.screen.trim.Contig355

Length=499

Score E

Sequences producing significant alignments: (Bits) Value

gb|EL744650.1| LV0277027 Taenia solium UNAM-cd2_larva Taenia ... 876 0.0

Query= TS.seq.screen.trim.Contig356

Length=312

Score E

Sequences producing significant alignments: (Bits) Value

gb|EL763196.1| AD0200011 Taenia solium UNAM-cd1_adult Taenia ... 457 8e-129

gb|EL755977.1| AD0028035 Taenia solium UNAM-cd1_adult Taenia ... 457 8e-129

gb|EL755786.1| AD0027082 Taenia solium UNAM-cd1_adult Taenia ... 457 8e-129

gb|EL762359.1| AD0183060 Taenia solium UNAM-cd1_adult Taenia ... 453 1e-127

gb|EL753849.1| AD0019136 Taenia solium UNAM-cd1_adult Taenia ... 446 2e-125

gb|EL743014.1| LV0250055 Taenia solium UNAM-cd2_larva Taenia ... 433 1e-121

gb|EL751176.1| AD0008187 Taenia solium UNAM-cd1_adult Taenia ... 412 2e-115

gb|EL744959.1| LV0283024 Taenia solium UNAM-cd2_larva Taenia ... 335 4e-92

gb|EL742866.1| LV0248028 Taenia solium UNAM-cd2_larva Taenia ... 279 2e-75

Query= TS.seq.screen.trim.Contig357

Length=566

Score E

Sequences producing significant alignments: (Bits) Value

gb|EL747739.1| LV0328027 Taenia solium UNAM-cd2_larva Taenia ... 909 0.0

gb|EL761079.1| AD0156019 Taenia solium UNAM-cd1_adult Taenia ... 710 0.0

gb|EL741130.1| LV0221031 Taenia solium UNAM-cd2_larva Taenia ... 652 0.0

gb|EL744647.1| LV0277024 Taenia solium UNAM-cd2_larva Taenia ... 636 0.0

gb|EL742613.1| LV0244044 Taenia solium UNAM-cd2_larva Taenia ... 630 0.0

Query= TS.seq.screen.trim.Contig358

Length=580

Score E

Sequences producing significant alignments: (Bits) Value

gb|EL741191.1| LV0222024 Taenia solium UNAM-cd2_larva Taenia ... 752 0.0

gb|EL741854.1| LV0232059 Taenia solium UNAM-cd2_larva Taenia ... 62.1 7e-10

Query= TS.seq.screen.trim.Contig359

Length=594

Score E

Sequences producing significant alignments: (Bits) Value

gb|EL743054.1| LV0251019 Taenia solium UNAM-cd2_larva Taenia ... 1020 0.0

gb|EL763402.1| AD0203047 Taenia solium UNAM-cd1_adult Taenia ... 736 0.0

gb|EL763283.1| AD0201046 Taenia solium UNAM-cd1_adult Taenia ... 736 0.0

gb|EL745207.1| LV0287061 Taenia solium UNAM-cd2_larva Taenia ... 730 0.0

gb|EL743689.1| LV0260077 Taenia solium UNAM-cd2_larva Taenia ... 730 0.0

gb|EL760303.1| AD0139039 Taenia solium UNAM-cd1_adult Taenia ... 725 0.0

gb|EL759495.1| AD0124009 Taenia solium UNAM-cd1_adult Taenia ... 725 0.0

gb|EL757276.1| AD0034092 Taenia solium UNAM-cd1_adult Taenia ... 725 0.0

gb|EL756035.1| AD0028093 Taenia solium UNAM-cd1_adult Taenia ... 725 0.0

gb|EL755752.1| AD0027048 Taenia solium UNAM-cd1_adult Taenia ... 725 0.0

Query= TS.seq.screen.trim.Contig360

Length=547

Score E

Sequences producing significant alignments: (Bits) Value

gb|EL745731.1| LV0296021 Taenia solium UNAM-cd2_larva Taenia ... 913 0.0

gb|EL741854.1| LV0232059 Taenia solium UNAM-cd2_larva Taenia ... 56.5 3e-08

Query= TS.seq.screen.trim.Contig361

Length=398

Score E

Sequences producing significant alignments: (Bits) Value

gb|EL755624.1| AD0026147 Taenia solium UNAM-cd1_adult Taenia ... 560 6e-160

gb|EL763122.1| AD0199006 Taenia solium UNAM-cd1_adult Taenia ... 436 1e-122

gb|EL763027.1| AD0197036 Taenia solium UNAM-cd1_adult Taenia ... 431 5e-121

gb|EL745244.1| LV0288026 Taenia solium UNAM-cd2_larva Taenia ... 375 2e-104

Query= TS.seq.screen.trim.Contig362

Length=700

Score E

Sequences producing significant alignments: (Bits) Value

gb|EL743776.1| LV0262017 Taenia solium UNAM-cd2_larva Taenia ... 1033 0.0

gb|EL741234.1| LV0223012 Taenia solium UNAM-cd2_larva Taenia ... 612 2e-175

gb|EL745696.1| LV0295054 Taenia solium UNAM-cd2_larva Taenia ... 449 1e-126

gb|EL747664.1| LV0327007 Taenia solium UNAM-cd2_larva Taenia ... 387 1e-107

Query= TS.seq.screen.trim.Contig363

Length=517

Score E

Sequences producing significant alignments: (Bits) Value

gb|EL758508.1| AD0107001 Taenia solium UNAM-cd1_adult Taenia ... 893 0.0

gb|EL748555.1| LV0342006 Taenia solium UNAM-cd2_larva Taenia ... 880 0.0

gb|EL741108.1| LV0221009 Taenia solium UNAM-cd2_larva Taenia ... 870 0.0

gb|EL746148.1| LV0302043 Taenia solium UNAM-cd2_larva Taenia ... 833 0.0

gb|EL758842.1| AD0112041 Taenia solium UNAM-cd1_adult Taenia ... 654 0.0

gb|EL746354.1| LV0308004 Taenia solium UNAM-cd2_larva Taenia ... 326 2e-89

gb|EL758707.1| AD0110016 Taenia solium UNAM-cd1_adult Taenia ... 183 2e-46

gb|EL745832.1| LV0297061 Taenia solium UNAM-cd2_larva Taenia ... 97.1 2e-20

Query= TS.seq.screen.trim.Contig364

Length=526

Score E

Sequences producing significant alignments: (Bits) Value

gb|EL742169.1| LV0237057 Taenia solium UNAM-cd2_larva Taenia ... 274 9e-74

gb|EL740611.1| LV0212047 Taenia solium UNAM-cd2_larva Taenia ... 228 7e-60

Query= TS.seq.screen.trim.Contig365

Length=593

Score E

Sequences producing significant alignments: (Bits) Value

gb|EL742890.1| LV0249005 Taenia solium UNAM-cd2_larva Taenia ... 815 0.0

gb|EL741854.1| LV0232059 Taenia solium UNAM-cd2_larva Taenia ... 56.5 3e-08

Query= TS.seq.screen.trim.Contig366

Length=1028

Score E

Sequences producing significant alignments: (Bits) Value

gb|EL741706.1| LV0230053 Taenia solium UNAM-cd2_larva Taenia ... 891 0.0

gb|EL742147.1| LV0237035 Taenia solium UNAM-cd2_larva Taenia ... 881 0.0

gb|EL745616.1| LV0294031 Taenia solium UNAM-cd2_larva Taenia ... 867 0.0

gb|EL748938.1| LV0348049 Taenia solium UNAM-cd2_larva Taenia ... 854 0.0

gb|EL747284.1| LV0321015 Taenia solium UNAM-cd2_larva Taenia ... 839 0.0

gb|EL742678.1| LV0245033 Taenia solium UNAM-cd2_larva Taenia ... 617 4e-177

gb|EL745649.1| LV0295007 Taenia solium UNAM-cd2_larva Taenia ... 592 2e-169

gb|EL763063.1| AD0198001 Taenia solium UNAM-cd1_adult Taenia ... 579 2e-165

gb|EL751655.1| AD0010148 Taenia solium UNAM-cd1_adult Taenia ... 575 2e-164

gb|EL743422.1| LV0257039 Taenia solium UNAM-cd2_larva Taenia ... 575 2e-164

Query= TS.seq.screen.trim.Contig367

Length=390

Score E

Sequences producing significant alignments: (Bits) Value

gb|EL741854.1| LV0232059 Taenia solium UNAM-cd2_larva Taenia ... 56.5 3e-08

Query= TS.seq.screen.trim.Contig369

Length=654

Score E

Sequences producing significant alignments: (Bits) Value

gb|EL743266.1| LV0254047 Taenia solium UNAM-cd2_larva Taenia ... 1155 0.0

gb|EL748703.1| LV0344026 Taenia solium UNAM-cd2_larva Taenia ... 1007 0.0

gb|EL748568.1| LV0342019 Taenia solium UNAM-cd2_larva Taenia ... 972 0.0

gb|EL747892.1| LV0330057 Taenia solium UNAM-cd2_larva Taenia ... 929 0.0

gb|EL745358.1| LV0290013 Taenia solium UNAM-cd2_larva Taenia ... 918 0.0

gb|EL744786.1| LV0280012 Taenia solium UNAM-cd2_larva Taenia ... 904 0.0

gb|EL746245.1| LV0305029 Taenia solium UNAM-cd2_larva Taenia ... 857 0.0

gb|EL743637.1| LV0260025 Taenia solium UNAM-cd2_larva Taenia ... 833 0.0

gb|EL742782.1| LV0246065 Taenia solium UNAM-cd2_larva Taenia ... 833 0.0

gb|EL741588.1| LV0228070 Taenia solium UNAM-cd2_larva Taenia ... 833 0.0

Query= TS.seq.screen.trim.Contig370

Length=532

Score E

Sequences producing significant alignments: (Bits) Value

gb|GT227655.1| tscaa0_001871.z1.scf Taenia solium adult full-... 442 2e-124

Query= TS.seq.screen.trim.Contig371

Length=450

Score E

Sequences producing significant alignments: (Bits) Value

gb|EL759127.1| AD0117030 Taenia solium UNAM-cd1_adult Taenia ... 121 1e-27

gb|EL763110.1| AD0198048 Taenia solium UNAM-cd1_adult Taenia ... 86.1 4e-17

gb|EL741854.1| LV0232059 Taenia solium UNAM-cd2_larva Taenia ... 56.5 3e-08

Query= TS.seq.screen.trim.Contig372

Length=635

Score E

Sequences producing significant alignments: (Bits) Value

gb|EL747429.1| LV0323038 Taenia solium UNAM-cd2_larva Taenia ... 977 0.0

gb|EL748042.1| LV0332064 Taenia solium UNAM-cd2_larva Taenia ... 966 0.0

gb|EL741905.1| LV0233038 Taenia solium UNAM-cd2_larva Taenia ... 957 0.0

gb|EL746269.1| LV0306020 Taenia solium UNAM-cd2_larva Taenia ... 813 0.0

gb|EL748313.1| LV0338002 Taenia solium UNAM-cd2_larva Taenia ... 765 0.0

gb|EL742361.1| LV0240056 Taenia solium UNAM-cd2_larva Taenia ... 628 2e-180

gb|EL747064.1| LV0317061 Taenia solium UNAM-cd2_larva Taenia ... 577 6e-165

gb|EL759563.1| AD0125021 Taenia solium UNAM-cd1_adult Taenia ... 569 1e-162

gb|EL755026.1| AD0024043 Taenia solium UNAM-cd1_adult Taenia ... 569 1e-162

gb|EL744802.1| LV0280028 Taenia solium UNAM-cd2_larva Taenia ... 566 1e-161

Query= TS.seq.screen.trim.Contig373

Length=367

Score E

Sequences producing significant alignments: (Bits) Value

gb|EL740723.1| LV0215013 Taenia solium UNAM-cd2_larva Taenia ... 496 2e-140

gb|EL762052.1| AD0177023 Taenia solium UNAM-cd1_adult Taenia ... 407 8e-114

gb|EX151213.1| TPEG001001H09 Cysti-host Taenia solium cDNA, m... 327 7e-90

gb|EX150563.1| TSEDTS1008G12 Cysti Taenia solium cDNA, mRNA s... 152 4e-37

gb|EX151282.1| TPEG001002G05 Cysti-host Taenia solium cDNA, m... 135 4e-32

Query= TS.seq.screen.trim.Contig374

Length=533

Score E

Sequences producing significant alignments: (Bits) Value

gb|EL744813.1| LV0280039 Taenia solium UNAM-cd2_larva Taenia ... 560 6e-160

gb|EL744031.1| LV0266031 Taenia solium UNAM-cd2_larva Taenia ... 555 3e-158

gb|EL763471.1| AD0204034 Taenia solium UNAM-cd1_adult Taenia ... 496 2e-140

gb|EL761846.1| AD0172013 Taenia solium UNAM-cd1_adult Taenia ... 359 2e-99

gb|EL743302.1| LV0255016 Taenia solium UNAM-cd2_larva Taenia ... 326 2e-89

gb|GT227219.1| tscaa0_000503.z1.scf Taenia solium adult full-... 230 2e-60

gb|EL743347.1| LV0256008 Taenia solium UNAM-cd2_larva Taenia ... 226 3e-59

gb|EL745545.1| LV0293023 Taenia solium UNAM-cd2_larva Taenia ... 163 2e-40

Query= TS.seq.screen.trim.Contig375

Length=925

Score E

Sequences producing significant alignments: (Bits) Value

gb|EL763282.1| AD0201045 Taenia solium UNAM-cd1_adult Taenia ... 1151 0.0

gb|EL748640.1| LV0343025 Taenia solium UNAM-cd2_larva Taenia ... 1107 0.0

gb|EL761670.1| AD0168030 Taenia solium UNAM-cd1_adult Taenia ... 946 0.0

gb|EL744523.1| LV0274026 Taenia solium UNAM-cd2_larva Taenia ... 824 0.0

gb|EL749028.1| LV0351016 Taenia solium UNAM-cd2_larva Taenia ... 821 0.0

gb|EL762164.1| AD0180006 Taenia solium UNAM-cd1_adult Taenia ... 676 0.0

gb|GT227116.1| tscaa0_002476.z1.scf Taenia solium adult full-... 604 3e-173

gb|EL758546.1| AD0107039 Taenia solium UNAM-cd1_adult Taenia ... 584 4e-167

gb|EL748660.1| LV0343045 Taenia solium UNAM-cd2_larva Taenia ... 520 1e-147

gb|EL755730.1| AD0027026 Taenia solium UNAM-cd1_adult Taenia ... 403 1e-112

Query= TS.seq.screen.trim.Contig376

Length=421

Score E

Sequences producing significant alignments: (Bits) Value

gb|EL740436.1| LV0209014 Taenia solium UNAM-cd2_larva Taenia ... 726 0.0

gb|EL742923.1| LV0249038 Taenia solium UNAM-cd2_larva Taenia ... 721 0.0

gb|EL762559.1| AD0189016 Taenia solium UNAM-cd1_adult Taenia ... 712 0.0

gb|EL745681.1| LV0295039 Taenia solium UNAM-cd2_larva Taenia ... 693 0.0

gb|EL758800.1| AD0111065 Taenia solium UNAM-cd1_adult Taenia ... 678 0.0

gb|EL745999.1| LV0300030 Taenia solium UNAM-cd2_larva Taenia ... 673 0.0

gb|EL743999.1| LV0265071 Taenia solium UNAM-cd2_larva Taenia ... 669 0.0

gb|EL745424.1| LV0291016 Taenia solium UNAM-cd2_larva Taenia ... 252 4e-67

gb|EL741538.1| LV0228020 Taenia solium UNAM-cd2_larva Taenia ... 241 9e-64

Query= TS.seq.screen.trim.Contig377

Length=442

Score E

Sequences producing significant alignments: (Bits) Value

gb|EL747142.1| LV0319001 Taenia solium UNAM-cd2_larva Taenia ... 758 0.0

gb|EL743436.1| LV0257053 Taenia solium UNAM-cd2_larva Taenia ... 758 0.0

gb|EL742333.1| LV0240028 Taenia solium UNAM-cd2_larva Taenia ... 758 0.0

gb|EL742250.1| LV0239002 Taenia solium UNAM-cd2_larva Taenia ... 752 0.0

gb|EL740494.1| LV0210018 Taenia solium UNAM-cd2_larva Taenia ... 750 0.0

gb|EL757635.1| AD0035228 Taenia solium UNAM-cd1_adult Taenia ... 743 0.0

gb|EL748692.1| LV0344015 Taenia solium UNAM-cd2_larva Taenia ... 734 0.0

gb|GT226972.1| tscaa0_002712.z1.scf Taenia solium adult full-... 706 0.0

gb|EL757543.1| AD0035136 Taenia solium UNAM-cd1_adult Taenia ... 680 0.0

gb|EL756959.1| AD0031232 Taenia solium UNAM-cd1_adult Taenia ... 680 0.0

Query= TS.seq.screen.trim.Contig378

Length=1010

Score E

Sequences producing significant alignments: (Bits) Value

gb|EL742678.1| LV0245033 Taenia solium UNAM-cd2_larva Taenia ... 1031 0.0

gb|EL758656.1| AD0109031 Taenia solium UNAM-cd1_adult Taenia ... 1009 0.0

gb|EL748024.1| LV0332046 Taenia solium UNAM-cd2_larva Taenia ... 977 0.0

gb|EL743779.1| LV0262020 Taenia solium UNAM-cd2_larva Taenia ... 977 0.0

gb|EL745532.1| LV0293010 Taenia solium UNAM-cd2_larva Taenia ... 968 0.0

gb|EL745649.1| LV0295007 Taenia solium UNAM-cd2_larva Taenia ... 907 0.0

gb|EL744879.1| LV0282005 Taenia solium UNAM-cd2_larva Taenia ... 865 0.0

gb|EL745002.1| LV0284033 Taenia solium UNAM-cd2_larva Taenia ... 857 0.0

gb|EL743422.1| LV0257039 Taenia solium UNAM-cd2_larva Taenia ... 850 0.0

gb|EL748304.1| LV0337025 Taenia solium UNAM-cd2_larva Taenia ... 808 0.0

Query= TS.seq.screen.trim.Contig379

Length=499

Score E

Sequences producing significant alignments: (Bits) Value

gb|EL756435.1| AD0029230 Taenia solium UNAM-cd1_adult Taenia ... 708 0.0

gb|EL749594.1| AD0001217 Taenia solium UNAM-cd1_adult Taenia ... 708 0.0

gb|EL754674.1| AD0022199 Taenia solium UNAM-cd1_adult Taenia ... 680 0.0

gb|EL742733.1| LV0246016 Taenia solium UNAM-cd2_larva Taenia ... 643 0.0

gb|EL760751.1| AD0148029 Taenia solium UNAM-cd1_adult Taenia ... 636 0.0

gb|EL740258.1| LV0205038 Taenia solium UNAM-cd2_larva Taenia ... 394 6e-110

gb|EL741868.1| LV0233001 Taenia solium UNAM-cd2_larva Taenia ... 270 1e-72

Query= TS.seq.screen.trim.Contig380

Length=543

Score E

Sequences producing significant alignments: (Bits) Value

gb|EL741854.1| LV0232059 Taenia solium UNAM-cd2_larva Taenia ... 63.9 2e-10

Query= TS.seq.screen.trim.Contig381

Length=992

Score E

Sequences producing significant alignments: (Bits) Value

gb|EL753051.1| AD0016089 Taenia solium UNAM-cd1_adult Taenia ... 893 0.0

gb|EL762773.1| AD0193020 Taenia solium UNAM-cd1_adult Taenia ... 883 0.0

gb|EL755954.1| AD0028012 Taenia solium UNAM-cd1_adult Taenia ... 883 0.0

gb|EL758188.1| AD0101068 Taenia solium UNAM-cd1_adult Taenia ... 881 0.0

gb|EL753310.1| AD0017068 Taenia solium UNAM-cd1_adult Taenia ... 876 0.0

gb|EL744082.1| LV0267021 Taenia solium UNAM-cd2_larva Taenia ... 850 0.0

gb|EL762502.1| AD0187021 Taenia solium UNAM-cd1_adult Taenia ... 699 0.0

gb|EL741618.1| LV0229029 Taenia solium UNAM-cd2_larva Taenia ... 669 0.0

gb|EL761075.1| AD0156015 Taenia solium UNAM-cd1_adult Taenia ... 630 0.0

gb|EL748149.1| LV0334032 Taenia solium UNAM-cd2_larva Taenia ... 628 2e-180

Query= TS.seq.screen.trim.Contig382

Length=624

Score E

Sequences producing significant alignments: (Bits) Value

gb|EL742954.1| LV0249069 Taenia solium UNAM-cd2_larva Taenia ... 745 0.0

Query= TS.seq.screen.trim.Contig383

Length=899

Score E

Sequences producing significant alignments: (Bits) Value

gb|EL750177.1| AD0004092 Taenia solium UNAM-cd1_adult Taenia ... 1476 0.0

gb|EL750424.1| AD0005087 Taenia solium UNAM-cd1_adult Taenia ... 1365 0.0

gb|EL761396.1| AD0163005 Taenia solium UNAM-cd1_adult Taenia ... 1323 0.0

gb|EL757308.1| AD0034124 Taenia solium UNAM-cd1_adult Taenia ... 1275 0.0

gb|EL755137.1| AD0024154 Taenia solium UNAM-cd1_adult Taenia ... 1264 0.0

gb|EL742812.1| LV0247030 Taenia solium UNAM-cd2_larva Taenia ... 1160 0.0

gb|EL755445.1| AD0025215 Taenia solium UNAM-cd1_adult Taenia ... 1158 0.0

gb|EL759059.1| AD0116022 Taenia solium UNAM-cd1_adult Taenia ... 1040 0.0

gb|EL757839.1| AD0096042 Taenia solium UNAM-cd1_adult Taenia ... 1040 0.0

gb|EL755533.1| AD0026056 Taenia solium UNAM-cd1_adult Taenia ... 1033 0.0

Query= TS.seq.screen.trim.Contig384

Length=960

Score E

Sequences producing significant alignments: (Bits) Value

gb|EL748061.1| LV0333012 Taenia solium UNAM-cd2_larva Taenia ... 1101 0.0

gb|EL747407.1| LV0323016 Taenia solium UNAM-cd2_larva Taenia ... 987 0.0

gb|EL748391.1| LV0339029 Taenia solium UNAM-cd2_larva Taenia ... 977 0.0

gb|EL745649.1| LV0295007 Taenia solium UNAM-cd2_larva Taenia ... 939 0.0

gb|EL761345.1| AD0162006 Taenia solium UNAM-cd1_adult Taenia ... 935 0.0

gb|EL745002.1| LV0284033 Taenia solium UNAM-cd2_larva Taenia ... 917 0.0

gb|EL748024.1| LV0332046 Taenia solium UNAM-cd2_larva Taenia ... 904 0.0

gb|EL751655.1| AD0010148 Taenia solium UNAM-cd1_adult Taenia ... 883 0.0

gb|EL761851.1| AD0172018 Taenia solium UNAM-cd1_adult Taenia ... 881 0.0

gb|EL762205.1| AD0181002 Taenia solium UNAM-cd1_adult Taenia ... 880 0.0

Query= TS.seq.screen.trim.Contig385

Length=589

Score E

Sequences producing significant alignments: (Bits) Value

gb|EL741864.1| LV0232069 Taenia solium UNAM-cd2_larva Taenia ... 887 0.0

gb|EL758104.1| AD0100049 Taenia solium UNAM-cd1_adult Taenia ... 876 0.0

gb|EL741337.1| LV0224057 Taenia solium UNAM-cd2_larva Taenia ... 861 0.0

gb|EL761994.1| AD0175040 Taenia solium UNAM-cd1_adult Taenia ... 313 2e-85

Query= TS.seq.screen.trim.Contig386

Length=773

Score E

Sequences producing significant alignments: (Bits) Value

gb|EL744385.1| LV0271067 Taenia solium UNAM-cd2_larva Taenia ... 961 0.0

gb|EL747504.1| LV0324040 Taenia solium UNAM-cd2_larva Taenia ... 601 4e-172

gb|EL747524.1| LV0325003 Taenia solium UNAM-cd2_larva Taenia ... 525 2e-149

gb|EL748691.1| LV0344014 Taenia solium UNAM-cd2_larva Taenia ... 494 6e-140

gb|EL742491.1| LV0242055 Taenia solium UNAM-cd2_larva Taenia ... 337 1e-92

gb|EL740294.1| LV0206026 Taenia solium UNAM-cd2_larva Taenia ... 318 4e-87

gb|EL744194.1| LV0269005 Taenia solium UNAM-cd2_larva Taenia ... 272 3e-73

Query= TS.seq.screen.trim.Contig387

Length=553

Score E

Sequences producing significant alignments: (Bits) Value

gb|EL762982.1| AD0196035 Taenia solium UNAM-cd1_adult Taenia ... 965 0.0

gb|EL744479.1| LV0273037 Taenia solium UNAM-cd2_larva Taenia ... 965 0.0

gb|EL743236.1| LV0254017 Taenia solium UNAM-cd2_larva Taenia ... 965 0.0

gb|EL743566.1| LV0259029 Taenia solium UNAM-cd2_larva Taenia ... 961 0.0

gb|EL759338.1| AD0121034 Taenia solium UNAM-cd1_adult Taenia ... 952 0.0

gb|EL758831.1| AD0112030 Taenia solium UNAM-cd1_adult Taenia ... 950 0.0

gb|EL756202.1| AD0028260 Taenia solium UNAM-cd1_adult Taenia ... 950 0.0

gb|EL754286.1| AD0021076 Taenia solium UNAM-cd1_adult Taenia ... 948 0.0

gb|EL741763.1| LV0231041 Taenia solium UNAM-cd2_larva Taenia ... 941 0.0

gb|EL754495.1| AD0022020 Taenia solium UNAM-cd1_adult Taenia ... 935 0.0

Query= TS.seq.screen.trim.Contig388

Length=471

Score E

Sequences producing significant alignments: (Bits) Value

gb|EL751030.1| AD0008041 Taenia solium UNAM-cd1_adult Taenia ... 507 8e-144

gb|EL762739.1| AD0192037 Taenia solium UNAM-cd1_adult Taenia ... 407 8e-114

gb|EL763037.1| AD0197046 Taenia solium UNAM-cd1_adult Taenia ... 387 1e-107

gb|EL761936.1| AD0174015 Taenia solium UNAM-cd1_adult Taenia ... 302 4e-82

gb|EL753780.1| AD0019067 Taenia solium UNAM-cd1_adult Taenia ... 300 1e-81

gb|EL753779.1| AD0019066 Taenia solium UNAM-cd1_adult Taenia ... 202 4e-52

Query= TS.seq.screen.trim.Contig391

Length=810

Score E

Sequences producing significant alignments: (Bits) Value

gb|EL740644.1| LV0213026 Taenia solium UNAM-cd2_larva Taenia ... 917 0.0

gb|GT227724.1| tscaa0_002167.z1.scf Taenia solium adult full-... 699 0.0

gb|EL741014.1| LV0219064 Taenia solium UNAM-cd2_larva Taenia ... 562 2e-160

gb|EL747076.1| LV0318008 Taenia solium UNAM-cd2_larva Taenia ... 510 6e-145

gb|EL750163.1| AD0004078 Taenia solium UNAM-cd1_adult Taenia ... 444 6e-125

gb|EL758489.1| AD0106032 Taenia solium UNAM-cd1_adult Taenia ... 115 6e-26

gb|EL746007.1| LV0300038 Taenia solium UNAM-cd2_larva Taenia ... 91.6 1e-18

Query= TS.seq.screen.trim.Contig392

Length=561

Score E

Sequences producing significant alignments: (Bits) Value

gb|EL760100.1| AD0135046 Taenia solium UNAM-cd1_adult Taenia ... 946 0.0

gb|EL762244.1| AD0181041 Taenia solium UNAM-cd1_adult Taenia ... 915 0.0

gb|EL747273.1| LV0321004 Taenia solium UNAM-cd2_larva Taenia ... 881 0.0

gb|EL758304.1| AD0103048 Taenia solium UNAM-cd1_adult Taenia ... 826 0.0

Query= TS.seq.screen.trim.Contig393

Length=546

Score E

Sequences producing significant alignments: (Bits) Value

gb|EL741854.1| LV0232059 Taenia solium UNAM-cd2_larva Taenia ... 58.4 1e-08

Query= TS.seq.screen.trim.Contig394

Length=521

Score E

Sequences producing significant alignments: (Bits) Value

gb|EL741914.1| LV0233047 Taenia solium UNAM-cd2_larva Taenia ... 944 0.0

gb|EL744461.1| LV0273019 Taenia solium UNAM-cd2_larva Taenia ... 939 0.0

gb|EL742432.1| LV0241058 Taenia solium UNAM-cd2_larva Taenia ... 852 0.0

gb|EL743670.1| LV0260058 Taenia solium UNAM-cd2_larva Taenia ... 784 0.0

gb|EL747602.1| LV0326018 Taenia solium UNAM-cd2_larva Taenia ... 675 0.0

gb|EL745580.1| LV0293058 Taenia solium UNAM-cd2_larva Taenia ... 270 1e-72

Query= TS.seq.screen.trim.Contig395

Length=807

Score E

Sequences producing significant alignments: (Bits) Value

gb|EL756118.1| AD0028176 Taenia solium UNAM-cd1_adult Taenia ... 1282 0.0

gb|EL742988.1| LV0250029 Taenia solium UNAM-cd2_larva Taenia ... 675 0.0

gb|EL743326.1| LV0255040 Taenia solium UNAM-cd2_larva Taenia ... 652 0.0

gb|EL742395.1| LV0241021 Taenia solium UNAM-cd2_larva Taenia ... 418 4e-117

gb|EL744287.1| LV0270034 Taenia solium UNAM-cd2_larva Taenia ... 292 2e-79

gb|EL748336.1| LV0338025 Taenia solium UNAM-cd2_larva Taenia ... 119 4e-27

Query= TS.seq.screen.trim.Contig396

Length=1029

Score E

Sequences producing significant alignments: (Bits) Value

gb|EL756844.1| AD0031117 Taenia solium UNAM-cd1_adult Taenia ... 1465 0.0

gb|EL763180.1| AD0199064 Taenia solium UNAM-cd1_adult Taenia ... 1269 0.0

gb|EL754636.1| AD0022161 Taenia solium UNAM-cd1_adult Taenia ... 1266 0.0

gb|EL753661.1| AD0018199 Taenia solium UNAM-cd1_adult Taenia ... 1266 0.0

gb|EL754411.1| AD0021201 Taenia solium UNAM-cd1_adult Taenia ... 1264 0.0

gb|EL755775.1| AD0027071 Taenia solium UNAM-cd1_adult Taenia ... 1258 0.0

gb|EL749251.1| LV0356029 Taenia solium UNAM-cd2_larva Taenia ... 1221 0.0

gb|EL753695.1| AD0018233 Taenia solium UNAM-cd1_adult Taenia ... 1214 0.0

gb|EL748653.1| LV0343038 Taenia solium UNAM-cd2_larva Taenia ... 1164 0.0

gb|EL757585.1| AD0035178 Taenia solium UNAM-cd1_adult Taenia ... 1133 0.0

Query= TS.seq.screen.trim.Contig397

Length=899

Score E

Sequences producing significant alignments: (Bits) Value

gb|EL749524.1| AD0001147 Taenia solium UNAM-cd1_adult Taenia ... 1384 0.0

gb|EL755565.1| AD0026088 Taenia solium UNAM-cd1_adult Taenia ... 990 0.0

gb|EL761840.1| AD0172007 Taenia solium UNAM-cd1_adult Taenia ... 983 0.0

gb|EL762920.1| AD0195040 Taenia solium UNAM-cd1_adult Taenia ... 961 0.0

gb|EL741846.1| LV0232051 Taenia solium UNAM-cd2_larva Taenia ... 876 0.0

gb|EL755564.1| AD0026087 Taenia solium UNAM-cd1_adult Taenia ... 874 0.0

gb|EL743652.1| LV0260040 Taenia solium UNAM-cd2_larva Taenia ... 682 0.0

gb|EL758719.1| AD0110028 Taenia solium UNAM-cd1_adult Taenia ... 665 0.0

gb|EL744890.1| LV0282016 Taenia solium UNAM-cd2_larva Taenia ... 628 2e-180

gb|EL744889.1| LV0282015 Taenia solium UNAM-cd2_larva Taenia ... 610 6e-175

Query= TS.seq.screen.trim.Contig398

Length=953

Score E

Sequences producing significant alignments: (Bits) Value

gb|EL763440.1| AD0204003 Taenia solium UNAM-cd1_adult Taenia ... 422 3e-118

gb|EL741854.1| LV0232059 Taenia solium UNAM-cd2_larva Taenia ... 56.5 3e-08

Query= TS.seq.screen.trim.Contig399

Length=569

Score E

Sequences producing significant alignments: (Bits) Value

gb|EL746823.1| LV0314036 Taenia solium UNAM-cd2_larva Taenia ... 606 8e-174

gb|EL746648.1| LV0312009 Taenia solium UNAM-cd2_larva Taenia ... 446 2e-125

gb|EL745626.1| LV0294041 Taenia solium UNAM-cd2_larva Taenia ... 377 7e-105

gb|EL761178.1| AD0158020 Taenia solium UNAM-cd1_adult Taenia ... 86.1 4e-17

gb|EL742214.1| LV0238033 Taenia solium UNAM-cd2_larva Taenia ... 86.1 4e-17

gb|EL746190.1| LV0304015 Taenia solium UNAM-cd2_larva Taenia ... 84.2 2e-16

gb|EL741854.1| LV0232059 Taenia solium UNAM-cd2_larva Taenia ... 58.4 1e-08

gb|EL745526.1| LV0293004 Taenia solium UNAM-cd2_larva Taenia ... 54.7 1e-07

Query= TS.seq.screen.trim.Contig400

Length=819

Score E

Sequences producing significant alignments: (Bits) Value

gb|EL745768.1| LV0296058 Taenia solium UNAM-cd2_larva Taenia ... 1149 0.0

Query= TS.seq.screen.trim.Contig401

Length=587

Score E

Sequences producing significant alignments: (Bits) Value

gb|EL740335.1| LV0207019 Taenia solium UNAM-cd2_larva Taenia ... 774 0.0

gb|EL747528.1| LV0325007 Taenia solium UNAM-cd2_larva Taenia ... 767 0.0

gb|EL744157.1| LV0268024 Taenia solium UNAM-cd2_larva Taenia ... 767 0.0

gb|EL743103.1| LV0252004 Taenia solium UNAM-cd2_larva Taenia ... 767 0.0

gb|EL747846.1| LV0330011 Taenia solium UNAM-cd2_larva Taenia ... 765 0.0

gb|EL743630.1| LV0260018 Taenia solium UNAM-cd2_larva Taenia ... 765 0.0

gb|EL741002.1| LV0219052 Taenia solium UNAM-cd2_larva Taenia ... 763 0.0

gb|EL747161.1| LV0319020 Taenia solium UNAM-cd2_larva Taenia ... 760 0.0

gb|EL750310.1| AD0004225 Taenia solium UNAM-cd1_adult Taenia ... 752 0.0

gb|EL753000.1| AD0016038 Taenia solium UNAM-cd1_adult Taenia ... 750 0.0

Query= TS.seq.screen.trim.Contig402

Length=727

Score E

Sequences producing significant alignments: (Bits) Value

gb|EL763335.1| AD0202035 Taenia solium UNAM-cd1_adult Taenia ... 265 5e-71

Query= TS.seq.screen.trim.Contig403

Length=546

Score E

Sequences producing significant alignments: (Bits) Value

gb|EL741006.1| LV0219056 Taenia solium UNAM-cd2_larva Taenia ... 939 0.0

gb|EL747707.1| LV0327050 Taenia solium UNAM-cd2_larva Taenia ... 538 3e-153

gb|EL747709.1| LV0327052 Taenia solium UNAM-cd2_larva Taenia ... 438 3e-123

gb|EL758207.1| AD0102018 Taenia solium UNAM-cd1_adult Taenia ... 274 9e-74

gb|EL762744.1| AD0192042 Taenia solium UNAM-cd1_adult Taenia ... 261 7e-70

gb|EL741854.1| LV0232059 Taenia solium UNAM-cd2_larva Taenia ... 56.5 3e-08

Query= TS.seq.screen.trim.Contig405

Length=869

Score E

Sequences producing significant alignments: (Bits) Value

gb|EL752183.1| AD0012211 Taenia solium UNAM-cd1_adult Taenia ... 1282 0.0

gb|EL741250.1| LV0223028 Taenia solium UNAM-cd2_larva Taenia ... 1208 0.0

gb|EL743502.1| LV0258042 Taenia solium UNAM-cd2_larva Taenia ... 1203 0.0

gb|EL760086.1| AD0135032 Taenia solium UNAM-cd1_adult Taenia ... 1188 0.0

gb|EL745948.1| LV0299037 Taenia solium UNAM-cd2_larva Taenia ... 1188 0.0

gb|EL743863.1| LV0263032 Taenia solium UNAM-cd2_larva Taenia ... 1182 0.0

gb|EL740624.1| LV0213006 Taenia solium UNAM-cd2_larva Taenia ... 1179 0.0

gb|EL758138.1| AD0101018 Taenia solium UNAM-cd1_adult Taenia ... 1173 0.0

gb|EL753839.1| AD0019126 Taenia solium UNAM-cd1_adult Taenia ... 1074 0.0

gb|EL748187.1| LV0335002 Taenia solium UNAM-cd2_larva Taenia ... 994 0.0

Query= TS.seq.screen.trim.Contig407

Length=716

Score E

Sequences producing significant alignments: (Bits) Value

gb|GT227395.1| tscaa0_001093.z1.scf Taenia solium adult full-... 468 4e-132

gb|EL740318.1| LV0207002 Taenia solium UNAM-cd2_larva Taenia ... 106 3e-23

Query= TS.seq.screen.trim.Contig408

Length=640

Score E

Sequences producing significant alignments: (Bits) Value

gb|EL757991.1| AD0099003 Taenia solium UNAM-cd1_adult Taenia ... 396 2e-110

gb|EL757923.1| AD0098008 Taenia solium UNAM-cd1_adult Taenia ... 392 2e-109

gb|EL740634.1| LV0213016 Taenia solium UNAM-cd2_larva Taenia ... 388 3e-108

gb|EL760199.1| AD0137032 Taenia solium UNAM-cd1_adult Taenia ... 385 4e-107

gb|EL759489.1| AD0124003 Taenia solium UNAM-cd1_adult Taenia ... 385 4e-107

gb|EL759465.1| AD0123044 Taenia solium UNAM-cd1_adult Taenia ... 385 4e-107

gb|EL758902.1| AD0113043 Taenia solium UNAM-cd1_adult Taenia ... 385 4e-107

gb|EL758829.1| AD0112028 Taenia solium UNAM-cd1_adult Taenia ... 385 4e-107

gb|EL758222.1| AD0102033 Taenia solium UNAM-cd1_adult Taenia ... 385 4e-107

gb|EL756854.1| AD0031127 Taenia solium UNAM-cd1_adult Taenia ... 385 4e-107

Query= TS.seq.screen.trim.Contig409

Length=582

Score E

Sequences producing significant alignments: (Bits) Value

gb|EL741854.1| LV0232059 Taenia solium UNAM-cd2_larva Taenia ... 56.5 3e-08

Query= TS.seq.screen.trim.Contig410

Length=850

Score E

Sequences producing significant alignments: (Bits) Value

gb|EL745649.1| LV0295007 Taenia solium UNAM-cd2_larva Taenia ... 928 0.0

gb|EL742784.1| LV0247002 Taenia solium UNAM-cd2_larva Taenia ... 907 0.0

gb|EL748024.1| LV0332046 Taenia solium UNAM-cd2_larva Taenia ... 896 0.0

gb|EL742678.1| LV0245033 Taenia solium UNAM-cd2_larva Taenia ... 839 0.0

gb|EL745002.1| LV0284033 Taenia solium UNAM-cd2_larva Taenia ... 832 0.0

gb|EL743779.1| LV0262020 Taenia solium UNAM-cd2_larva Taenia ... 828 0.0

gb|EL751655.1| AD0010148 Taenia solium UNAM-cd1_adult Taenia ... 819 0.0

gb|EL744879.1| LV0282005 Taenia solium UNAM-cd2_larva Taenia ... 797 0.0

gb|EL748061.1| LV0333012 Taenia solium UNAM-cd2_larva Taenia ... 795 0.0

gb|EL745524.1| LV0293002 Taenia solium UNAM-cd2_larva Taenia ... 780 0.0

Query= TS.seq.screen.trim.Contig411

Length=485

Score E

Sequences producing significant alignments: (Bits) Value

gb|EL741854.1| LV0232059 Taenia solium UNAM-cd2_larva Taenia ... 58.4 1e-08

Query= TS.seq.screen.trim.Contig413

Length=696

Score E

Sequences producing significant alignments: (Bits) Value

gb|EL758019.1| AD0099031 Taenia solium UNAM-cd1_adult Taenia ... 857 0.0

gb|EL748179.1| LV0334062 Taenia solium UNAM-cd2_larva Taenia ... 673 0.0

gb|EL743577.1| LV0259040 Taenia solium UNAM-cd2_larva Taenia ... 305 3e-83

Query= TS.seq.screen.trim.Contig414

Length=388

Score E

Sequences producing significant alignments: (Bits) Value

gb|EL748227.1| LV0336003 Taenia solium UNAM-cd2_larva Taenia ... 534 4e-152

gb|EL742012.1| LV0235001 Taenia solium UNAM-cd2_larva Taenia ... 534 4e-152

gb|EX151138.1| TPEG001001A07 Cysti-host Taenia solium cDNA, m... 396 2e-110

gb|EX151156.1| TPEG001001C03 Cysti-host Taenia solium cDNA, m... 316 1e-86

gb|EL749249.1| LV0356027 Taenia solium UNAM-cd2_larva Taenia ... 298 5e-81

gb|EL757934.1| AD0098019 Taenia solium UNAM-cd1_adult Taenia ... 292 2e-79

gb|EL748580.1| LV0342031 Taenia solium UNAM-cd2_larva Taenia ... 291 9e-79

gb|EX151449.1| TPEG001004H05 Cysti-host Taenia solium cDNA, m... 246 2e-65

gb|EL749173.1| LV0354032 Taenia solium UNAM-cd2_larva Taenia ... 244 7e-65

gb|EL743493.1| LV0258033 Taenia solium UNAM-cd2_larva Taenia ... 165 6e-41

Query= TS.seq.screen.trim.Contig415

Length=558

Score E

Sequences producing significant alignments: (Bits) Value

gb|EL745590.1| LV0294005 Taenia solium UNAM-cd2_larva Taenia ... 970 0.0

gb|EL743987.1| LV0265059 Taenia solium UNAM-cd2_larva Taenia ... 970 0.0

gb|EL763349.1| AD0202049 Taenia solium UNAM-cd1_adult Taenia ... 961 0.0

gb|EL746994.1| LV0316071 Taenia solium UNAM-cd2_larva Taenia ... 961 0.0

gb|EL743873.1| LV0264002 Taenia solium UNAM-cd2_larva Taenia ... 719 0.0

gb|EL741453.1| LV0227009 Taenia solium UNAM-cd2_larva Taenia ... 671 0.0

gb|EL746280.1| LV0306031 Taenia solium UNAM-cd2_larva Taenia ... 569 1e-162

gb|EL743155.1| LV0253002 Taenia solium UNAM-cd2_larva Taenia ... 490 8e-139

gb|EL740529.1| LV0211006 Taenia solium UNAM-cd2_larva Taenia ... 477 6e-135

gb|EL741650.1| LV0229061 Taenia solium UNAM-cd2_larva Taenia ... 255 3e-68

Query= TS.seq.screen.trim.Contig416

Length=465

Score E

Sequences producing significant alignments: (Bits) Value

gb|EL743589.1| LV0259052 Taenia solium UNAM-cd2_larva Taenia ... 750 0.0

gb|EL740683.1| LV0214008 Taenia solium UNAM-cd2_larva Taenia ... 658 0.0

gb|EL742763.1| LV0246046 Taenia solium UNAM-cd2_larva Taenia ... 451 4e-127

gb|EL740919.1| LV0218030 Taenia solium UNAM-cd2_larva Taenia ... 374 8e-104

gb|EL747054.1| LV0317051 Taenia solium UNAM-cd2_larva Taenia ... 285 4e-77

gb|EL741854.1| LV0232059 Taenia solium UNAM-cd2_larva Taenia ... 58.4 1e-08

Query= TS.seq.screen.trim.Contig417

Length=742

Score E

Sequences producing significant alignments: (Bits) Value

gb|EL760289.1| AD0139025 Taenia solium UNAM-cd1_adult Taenia ... 1321 0.0

gb|EL751993.1| AD0012021 Taenia solium UNAM-cd1_adult Taenia ... 1299 0.0

gb|EL758115.1| AD0100060 Taenia solium UNAM-cd1_adult Taenia ... 1273 0.0

gb|EL757793.1| AD0095068 Taenia solium UNAM-cd1_adult Taenia ... 1223 0.0

gb|EL752252.1| AD0013019 Taenia solium UNAM-cd1_adult Taenia ... 1208 0.0

gb|EL743249.1| LV0254030 Taenia solium UNAM-cd2_larva Taenia ... 1083 0.0

gb|EL746981.1| LV0316058 Taenia solium UNAM-cd2_larva Taenia ... 1027 0.0

gb|EL748577.1| LV0342028 Taenia solium UNAM-cd2_larva Taenia ... 1009 0.0

gb|EL759310.1| AD0121006 Taenia solium UNAM-cd1_adult Taenia ... 998 0.0

gb|EL748973.1| LV0349022 Taenia solium UNAM-cd2_larva Taenia ... 881 0.0

Query= TS.seq.screen.trim.Contig418

Length=1107

Score E

Sequences producing significant alignments: (Bits) Value

gb|EL746964.1| LV0316041 Taenia solium UNAM-cd2_larva Taenia ... 379 2e-105

Query= TS.seq.screen.trim.Contig419

Length=887

Score E

Sequences producing significant alignments: (Bits) Value

gb|EL741164.1| LV0221065 Taenia solium UNAM-cd2_larva Taenia ... 1101 0.0

gb|EL748462.1| LV0340036 Taenia solium UNAM-cd2_larva Taenia ... 1079 0.0

gb|EL744522.1| LV0274025 Taenia solium UNAM-cd2_larva Taenia ... 1062 0.0

gb|EL762769.1| AD0193016 Taenia solium UNAM-cd1_adult Taenia ... 1002 0.0

gb|EL760877.1| AD0152002 Taenia solium UNAM-cd1_adult Taenia ... 819 0.0

gb|EL761116.1| AD0157012 Taenia solium UNAM-cd1_adult Taenia ... 782 0.0

gb|EL758106.1| AD0100051 Taenia solium UNAM-cd1_adult Taenia ... 713 0.0

gb|EL759683.1| AD0127024 Taenia solium UNAM-cd1_adult Taenia ... 582 1e-166

gb|EL740592.1| LV0212028 Taenia solium UNAM-cd2_larva Taenia ... 529 2e-150

gb|EL740513.1| LV0210037 Taenia solium UNAM-cd2_larva Taenia ... 520 1e-147

Query= TS.seq.screen.trim.Contig420

Length=757

Score E

Sequences producing significant alignments: (Bits) Value

gb|EL750390.1| AD0005053 Taenia solium UNAM-cd1_adult Taenia ... 1271 0.0

gb|EL756210.1| AD0029005 Taenia solium UNAM-cd1_adult Taenia ... 1179 0.0

gb|EL753463.1| AD0018001 Taenia solium UNAM-cd1_adult Taenia ... 1158 0.0

gb|EL751540.1| AD0010033 Taenia solium UNAM-cd1_adult Taenia ... 1158 0.0

gb|EL752839.1| AD0015098 Taenia solium UNAM-cd1_adult Taenia ... 1105 0.0

gb|EL754604.1| AD0022129 Taenia solium UNAM-cd1_adult Taenia ... 1096 0.0

gb|EL751783.1| AD0011026 Taenia solium UNAM-cd1_adult Taenia ... 1077 0.0

gb|EL759743.1| AD0128032 Taenia solium UNAM-cd1_adult Taenia ... 1075 0.0

gb|EL754932.1| AD0023188 Taenia solium UNAM-cd1_adult Taenia ... 1059 0.0

gb|EL752580.1| AD0014121 Taenia solium UNAM-cd1_adult Taenia ... 1053 0.0

Query= TS.seq.screen.trim.Contig421

Length=1195

Score E

Sequences producing significant alignments: (Bits) Value

gb|EL748513.1| LV0341029 Taenia solium UNAM-cd2_larva Taenia ... 1122 0.0

gb|GT227066.1| tscaa0_001616.z1.scf Taenia solium adult full-... 854 0.0

gb|EL748188.1| LV0335003 Taenia solium UNAM-cd2_larva Taenia ... 726 0.0

gb|EL740298.1| LV0206030 Taenia solium UNAM-cd2_larva Taenia ... 414 5e-116

gb|EL746343.1| LV0307057 Taenia solium UNAM-cd2_larva Taenia ... 176 3e-44

Query= TS.seq.screen.trim.Contig422

Length=421

Score E

Sequences producing significant alignments: (Bits) Value

gb|EL755894.1| AD0027190 Taenia solium UNAM-cd1_adult Taenia ... 732 0.0

gb|EL749426.1| AD0001049 Taenia solium UNAM-cd1_adult Taenia ... 725 0.0

gb|EL744757.1| LV0279026 Taenia solium UNAM-cd2_larva Taenia ... 725 0.0

gb|EL743531.1| LV0258071 Taenia solium UNAM-cd2_larva Taenia ... 725 0.0

gb|EL740569.1| LV0212005 Taenia solium UNAM-cd2_larva Taenia ... 725 0.0

gb|EL762950.1| AD0196003 Taenia solium UNAM-cd1_adult Taenia ... 723 0.0

gb|EL753619.1| AD0018157 Taenia solium UNAM-cd1_adult Taenia ... 723 0.0

gb|EL757365.1| AD0034181 Taenia solium UNAM-cd1_adult Taenia ... 721 0.0

gb|EL754052.1| AD0020097 Taenia solium UNAM-cd1_adult Taenia ... 719 0.0

gb|EL742030.1| LV0235019 Taenia solium UNAM-cd2_larva Taenia ... 717 0.0

Query= TS.seq.screen.trim.Contig423

Length=526

Score E

Sequences producing significant alignments: (Bits) Value

gb|EL748055.1| LV0333006 Taenia solium UNAM-cd2_larva Taenia ... 913 0.0

gb|EL747296.1| LV0321027 Taenia solium UNAM-cd2_larva Taenia ... 913 0.0

gb|EL747162.1| LV0319021 Taenia solium UNAM-cd2_larva Taenia ... 913 0.0

gb|EL747078.1| LV0318010 Taenia solium UNAM-cd2_larva Taenia ... 913 0.0

gb|EL747071.1| LV0318003 Taenia solium UNAM-cd2_larva Taenia ... 913 0.0

gb|EL746196.1| LV0304021 Taenia solium UNAM-cd2_larva Taenia ... 913 0.0

gb|EL743038.1| LV0251003 Taenia solium UNAM-cd2_larva Taenia ... 913 0.0

gb|EL741380.1| LV0225031 Taenia solium UNAM-cd2_larva Taenia ... 913 0.0

gb|EL741207.1| LV0222040 Taenia solium UNAM-cd2_larva Taenia ... 913 0.0

gb|EL745219.1| LV0288001 Taenia solium UNAM-cd2_larva Taenia ... 909 0.0

Query= TS.seq.screen.trim.Contig425

Length=591

Score E

Sequences producing significant alignments: (Bits) Value

gb|EL752539.1| AD0014080 Taenia solium UNAM-cd1_adult Taenia ... 1037 0.0

gb|EL745726.1| LV0296016 Taenia solium UNAM-cd2_larva Taenia ... 994 0.0

gb|EL752802.1| AD0015061 Taenia solium UNAM-cd1_adult Taenia ... 715 0.0

gb|EL757932.1| AD0098017 Taenia solium UNAM-cd1_adult Taenia ... 662 0.0

Query= TS.seq.screen.trim.Contig426

Length=618

Score E

Sequences producing significant alignments: (Bits) Value

gb|EL763190.1| AD0200005 Taenia solium UNAM-cd1_adult Taenia ... 822 0.0

gb|EL761636.1| AD0167026 Taenia solium UNAM-cd1_adult Taenia ... 743 0.0

gb|EL758544.1| AD0107037 Taenia solium UNAM-cd1_adult Taenia ... 623 8e-179

gb|EL760960.1| AD0153034 Taenia solium UNAM-cd1_adult Taenia ... 265 5e-71

Query= TS.seq.screen.trim.Contig427

Length=437

Score E

Sequences producing significant alignments: (Bits) Value

gb|EL741573.1| LV0228055 Taenia solium UNAM-cd2_larva Taenia ... 702 0.0

gb|EL756703.1| AD0030235 Taenia solium UNAM-cd1_adult Taenia ... 701 0.0

gb|EL747589.1| LV0326005 Taenia solium UNAM-cd2_larva Taenia ... 701 0.0

gb|EL744912.1| LV0282038 Taenia solium UNAM-cd2_larva Taenia ... 701 0.0

gb|EL741602.1| LV0229013 Taenia solium UNAM-cd2_larva Taenia ... 701 0.0

gb|EL741030.1| LV0220003 Taenia solium UNAM-cd2_larva Taenia ... 701 0.0

gb|EL740866.1| LV0217049 Taenia solium UNAM-cd2_larva Taenia ... 699 0.0

gb|EL744752.1| LV0279021 Taenia solium UNAM-cd2_larva Taenia ... 695 0.0

gb|EL746520.1| LV0310017 Taenia solium UNAM-cd2_larva Taenia ... 693 0.0

gb|EL742622.1| LV0244053 Taenia solium UNAM-cd2_larva Taenia ... 691 0.0

Query= TS.seq.screen.trim.Contig428

Length=521

Score E

Sequences producing significant alignments: (Bits) Value

gb|EL742429.1| LV0241055 Taenia solium UNAM-cd2_larva Taenia ... 486 1e-137

gb|EL740821.1| LV0217004 Taenia solium UNAM-cd2_larva Taenia ... 486 1e-137

gb|EL749115.1| LV0353027 Taenia solium UNAM-cd2_larva Taenia ... 479 2e-135

gb|EL757991.1| AD0099003 Taenia solium UNAM-cd1_adult Taenia ... 381 5e-106

gb|EL748377.1| LV0339015 Taenia solium UNAM-cd2_larva Taenia ... 366 1e-101

gb|EL757923.1| AD0098008 Taenia solium UNAM-cd1_adult Taenia ... 361 7e-100

gb|EL753583.1| AD0018121 Taenia solium UNAM-cd1_adult Taenia ... 359 2e-99

gb|EL740634.1| LV0213016 Taenia solium UNAM-cd2_larva Taenia ... 357 8e-99

gb|EL760199.1| AD0137032 Taenia solium UNAM-cd1_adult Taenia ... 353 1e-97

gb|EL759489.1| AD0124003 Taenia solium UNAM-cd1_adult Taenia ... 353 1e-97

Query= TS.seq.screen.trim.Contig429

Length=565

Score E

Sequences producing significant alignments: (Bits) Value

gb|EL749603.1| AD0001226 Taenia solium UNAM-cd1_adult Taenia ... 904 0.0

gb|EL763347.1| AD0202047 Taenia solium UNAM-cd1_adult Taenia ... 889 0.0

gb|EL757298.1| AD0034114 Taenia solium UNAM-cd1_adult Taenia ... 887 0.0

gb|EL755634.1| AD0026157 Taenia solium UNAM-cd1_adult Taenia ... 885 0.0

gb|EL763042.1| AD0197051 Taenia solium UNAM-cd1_adult Taenia ... 883 0.0

gb|EL757969.1| AD0098054 Taenia solium UNAM-cd1_adult Taenia ... 876 0.0

gb|EL748097.1| LV0333048 Taenia solium UNAM-cd2_larva Taenia ... 876 0.0

gb|EL761113.1| AD0157009 Taenia solium UNAM-cd1_adult Taenia ... 846 0.0

gb|EL762764.1| AD0193011 Taenia solium UNAM-cd1_adult Taenia ... 460 6e-130

gb|EL760490.1| AD0143032 Taenia solium UNAM-cd1_adult Taenia ... 459 2e-129

Query= TS.seq.screen.trim.Contig430

Length=440

Score E

Sequences producing significant alignments: (Bits) Value

gb|GT227134.1| tscaa0_000921.z1.scf Taenia solium adult full-... 106 3e-23

gb|EX151156.1| TPEG001001C03 Cysti-host Taenia solium cDNA, m... 106 3e-23

gb|EL748959.1| LV0349008 Taenia solium UNAM-cd2_larva Taenia ... 106 3e-23

gb|EL748580.1| LV0342031 Taenia solium UNAM-cd2_larva Taenia ... 106 3e-23

gb|EL745155.1| LV0287009 Taenia solium UNAM-cd2_larva Taenia ... 106 3e-23

gb|EL743493.1| LV0258033 Taenia solium UNAM-cd2_larva Taenia ... 106 3e-23

gb|EL742012.1| LV0235001 Taenia solium UNAM-cd2_larva Taenia ... 106 3e-23

gb|EL748227.1| LV0336003 Taenia solium UNAM-cd2_larva Taenia ... 100 2e-21

gb|EX151195.1| TPEG001001F09 Cysti-host Taenia solium cDNA, m... 95.3 7e-20

gb|EX151138.1| TPEG001001A07 Cysti-host Taenia solium cDNA, m... 75.0 1e-13

Query= TS.seq.screen.trim.Contig433

Length=874

Score E

Sequences producing significant alignments: (Bits) Value

gb|EL745083.1| LV0285055 Taenia solium UNAM-cd2_larva Taenia ... 1247 0.0

gb|EL762810.1| AD0193057 Taenia solium UNAM-cd1_adult Taenia ... 1197 0.0

gb|EL748432.1| LV0340006 Taenia solium UNAM-cd2_larva Taenia ... 1182 0.0

gb|EL741867.1| LV0232072 Taenia solium UNAM-cd2_larva Taenia ... 1142 0.0

gb|EL760404.1| AD0142002 Taenia solium UNAM-cd1_adult Taenia ... 1118 0.0

gb|EL749347.1| LV0359025 Taenia solium UNAM-cd2_larva Taenia ... 1018 0.0

gb|EL740244.1| LV0205024 Taenia solium UNAM-cd2_larva Taenia ... 1003 0.0

gb|EL761726.1| AD0169043 Taenia solium UNAM-cd1_adult Taenia ... 974 0.0

gb|EL762217.1| AD0181014 Taenia solium UNAM-cd1_adult Taenia ... 887 0.0

gb|EL760415.1| AD0142013 Taenia solium UNAM-cd1_adult Taenia ... 869 0.0

Query= TS.seq.screen.trim.Contig434

Length=546

Score E

Sequences producing significant alignments: (Bits) Value

gb|EL741253.1| LV0223031 Taenia solium UNAM-cd2_larva Taenia ... 944 0.0

gb|GT227596.1| tscaa0_001711.z1.scf Taenia solium adult full-... 854 0.0

gb|EL747889.1| LV0330054 Taenia solium UNAM-cd2_larva Taenia ... 654 0.0

gb|EL748237.1| LV0336013 Taenia solium UNAM-cd2_larva Taenia ... 305 3e-83

gb|EL741854.1| LV0232059 Taenia solium UNAM-cd2_larva Taenia ... 58.4 1e-08

Query= TS.seq.screen.trim.Contig435

Length=542

Score E

Sequences producing significant alignments: (Bits) Value

gb|EL760418.1| AD0142016 Taenia solium UNAM-cd1_adult Taenia ... 950 0.0

gb|EL753859.1| AD0019146 Taenia solium UNAM-cd1_adult Taenia ... 950 0.0

gb|EL750256.1| AD0004171 Taenia solium UNAM-cd1_adult Taenia ... 950 0.0

gb|EL762104.1| AD0178038 Taenia solium UNAM-cd1_adult Taenia ... 944 0.0

gb|EL741114.1| LV0221015 Taenia solium UNAM-cd2_larva Taenia ... 944 0.0

gb|EL761216.1| AD0159013 Taenia solium UNAM-cd1_adult Taenia ... 939 0.0

gb|EL762131.1| AD0179024 Taenia solium UNAM-cd1_adult Taenia ... 935 0.0

gb|EL759527.1| AD0124041 Taenia solium UNAM-cd1_adult Taenia ... 933 0.0

gb|EL750493.1| AD0005156 Taenia solium UNAM-cd1_adult Taenia ... 929 0.0

gb|EL763472.1| AD0204035 Taenia solium UNAM-cd1_adult Taenia ... 928 0.0

Query= TS.seq.screen.trim.Contig436

Length=432

Score E

Sequences producing significant alignments: (Bits) Value

gb|EL750022.1| AD0003164 Taenia solium UNAM-cd1_adult Taenia ... 375 2e-104

gb|EL751324.1| AD0009062 Taenia solium UNAM-cd1_adult Taenia ... 364 5e-101

gb|EL759014.1| AD0115039 Taenia solium UNAM-cd1_adult Taenia ... 353 1e-97

gb|EL762211.1| AD0181008 Taenia solium UNAM-cd1_adult Taenia ... 348 5e-96

gb|EL762298.1| AD0182054 Taenia solium UNAM-cd1_adult Taenia ... 329 2e-90

gb|EL749796.1| AD0002160 Taenia solium UNAM-cd1_adult Taenia ... 298 5e-81

gb|EL741854.1| LV0232059 Taenia solium UNAM-cd2_larva Taenia ... 56.5 3e-08

Query= TS.seq.screen.trim.Contig437

Length=417

Score E

Sequences producing significant alignments: (Bits) Value

gb|EL750022.1| AD0003164 Taenia solium UNAM-cd1_adult Taenia ... 368 4e-102

gb|EL751324.1| AD0009062 Taenia solium UNAM-cd1_adult Taenia ... 357 8e-99

gb|EL759014.1| AD0115039 Taenia solium UNAM-cd1_adult Taenia ... 346 2e-95

gb|EL762211.1| AD0181008 Taenia solium UNAM-cd1_adult Taenia ... 340 9e-94

gb|EL762298.1| AD0182054 Taenia solium UNAM-cd1_adult Taenia ... 322 3e-88

gb|EL749796.1| AD0002160 Taenia solium UNAM-cd1_adult Taenia ... 298 5e-81

gb|EL741854.1| LV0232059 Taenia solium UNAM-cd2_larva Taenia ... 56.5 3e-08

Query= TS.seq.screen.trim.Contig438

Length=1085

Score E

Sequences producing significant alignments: (Bits) Value

gb|EL743130.1| LV0252031 Taenia solium UNAM-cd2_larva Taenia ... 346 2e-95

Query= TS.seq.screen.trim.Contig441

Length=822

Score E

Sequences producing significant alignments: (Bits) Value

gb|EX151204.1| TPEG001001G09 Cysti-host Taenia solium cDNA, m... 1367 0.0

gb|EL741653.1| LV0229064 Taenia solium UNAM-cd2_larva Taenia ... 1149 0.0

gb|EL749812.1| AD0002176 Taenia solium UNAM-cd1_adult Taenia ... 1098 0.0

gb|EL749064.1| LV0352019 Taenia solium UNAM-cd2_larva Taenia ... 1037 0.0

gb|EL749049.1| LV0352004 Taenia solium UNAM-cd2_larva Taenia ... 937 0.0

gb|EL740490.1| LV0210014 Taenia solium UNAM-cd2_larva Taenia ... 904 0.0

gb|EL749183.1| LV0354042 Taenia solium UNAM-cd2_larva Taenia ... 850 0.0

gb|EL756674.1| AD0030206 Taenia solium UNAM-cd1_adult Taenia ... 737 0.0

gb|EL748652.1| LV0343037 Taenia solium UNAM-cd2_larva Taenia ... 675 0.0

gb|EL757789.1| AD0095064 Taenia solium UNAM-cd1_adult Taenia ... 645 0.0

Query= TS.seq.screen.trim.Contig443

Length=1023

Score E

Sequences producing significant alignments: (Bits) Value

gb|EL742994.1| LV0250035 Taenia solium UNAM-cd2_larva Taenia ... 503 1e-142

gb|EL762716.1| AD0192014 Taenia solium UNAM-cd1_adult Taenia ... 233 1e-61

Query= TS.seq.screen.trim.Contig444

Length=688

Score E

Sequences producing significant alignments: (Bits) Value

gb|EL758509.1| AD0107002 Taenia solium UNAM-cd1_adult Taenia ... 1184 0.0

gb|EL761332.1| AD0161049 Taenia solium UNAM-cd1_adult Taenia ... 1158 0.0

gb|EL762826.1| AD0193073 Taenia solium UNAM-cd1_adult Taenia ... 990 0.0

gb|EL757767.1| AD0095042 Taenia solium UNAM-cd1_adult Taenia ... 857 0.0

gb|EL740311.1| LV0206043 Taenia solium UNAM-cd2_larva Taenia ... 750 0.0

gb|EL747416.1| LV0323025 Taenia solium UNAM-cd2_larva Taenia ... 737 0.0

gb|EL756132.1| AD0028190 Taenia solium UNAM-cd1_adult Taenia ... 604 3e-173

gb|EL744592.1| LV0276010 Taenia solium UNAM-cd2_larva Taenia ... 604 3e-173

gb|EL758752.1| AD0111017 Taenia solium UNAM-cd1_adult Taenia ... 588 3e-168

gb|EL744505.1| LV0274008 Taenia solium UNAM-cd2_larva Taenia ... 525 2e-149

Query= TS.seq.screen.trim.Contig445

Length=1163

Score E

Sequences producing significant alignments: (Bits) Value

gb|EL757065.1| AD0032077 Taenia solium UNAM-cd1_adult Taenia ... 313 2e-85

gb|EL743442.1| LV0257059 Taenia solium UNAM-cd2_larva Taenia ... 128 7e-30

Query= TS.seq.screen.trim.Contig446

Length=882

Score E

Sequences producing significant alignments: (Bits) Value

gb|EL753812.1| AD0019099 Taenia solium UNAM-cd1_adult Taenia ... 723 0.0

gb|EL743570.1| LV0259033 Taenia solium UNAM-cd2_larva Taenia ... 580 5e-166

gb|EL753813.1| AD0019100 Taenia solium UNAM-cd1_adult Taenia ... 547 5e-156

gb|EL743569.1| LV0259032 Taenia solium UNAM-cd2_larva Taenia ... 429 2e-120

gb|EL740468.1| LV0209046 Taenia solium UNAM-cd2_larva Taenia ... 344 7e-95

Query= TS.seq.screen.trim.Contig447

Length=637

Score E

Sequences producing significant alignments: (Bits) Value

gb|EL761928.1| AD0174007 Taenia solium UNAM-cd1_adult Taenia ... 989 0.0

gb|EL744675.1| LV0277052 Taenia solium UNAM-cd2_larva Taenia ... 972 0.0

gb|EL741506.1| LV0227062 Taenia solium UNAM-cd2_larva Taenia ... 654 0.0

gb|EL746431.1| LV0309001 Taenia solium UNAM-cd2_larva Taenia ... 451 4e-127

gb|EL744627.1| LV0277004 Taenia solium UNAM-cd2_larva Taenia ... 418 4e-117

gb|EL752268.1| AD0013035 Taenia solium UNAM-cd1_adult Taenia ... 416 1e-116

gb|EL751933.1| AD0011176 Taenia solium UNAM-cd1_adult Taenia ... 416 1e-116

gb|EL749887.1| AD0003029 Taenia solium UNAM-cd1_adult Taenia ... 416 1e-116

gb|EL751812.1| AD0011055 Taenia solium UNAM-cd1_adult Taenia ... 411 6e-115

gb|EL751378.1| AD0009116 Taenia solium UNAM-cd1_adult Taenia ... 394 6e-110

Query= TS.seq.screen.trim.Contig448

Length=998

Score E

Sequences producing significant alignments: (Bits) Value

gb|EL763055.1| AD0197064 Taenia solium UNAM-cd1_adult Taenia ... 130 2e-30

Query= TS.seq.screen.trim.Contig449

Length=607

Score E

Sequences producing significant alignments: (Bits) Value

gb|EL757588.1| AD0035181 Taenia solium UNAM-cd1_adult Taenia ... 797 0.0

gb|EL751845.1| AD0011088 Taenia solium UNAM-cd1_adult Taenia ... 795 0.0

gb|EL751350.1| AD0009088 Taenia solium UNAM-cd1_adult Taenia ... 795 0.0

gb|EL751289.1| AD0009027 Taenia solium UNAM-cd1_adult Taenia ... 795 0.0

gb|EL750887.1| AD0007123 Taenia solium UNAM-cd1_adult Taenia ... 795 0.0

gb|EL750877.1| AD0007113 Taenia solium UNAM-cd1_adult Taenia ... 795 0.0

gb|EL750076.1| AD0003218 Taenia solium UNAM-cd1_adult Taenia ... 795 0.0

gb|EL749971.1| AD0003113 Taenia solium UNAM-cd1_adult Taenia ... 795 0.0

gb|EL749903.1| AD0003045 Taenia solium UNAM-cd1_adult Taenia ... 795 0.0

gb|EL749871.1| AD0003013 Taenia solium UNAM-cd1_adult Taenia ... 795 0.0

Query= TS.seq.screen.trim.Contig450

Length=1627

Score E

Sequences producing significant alignments: (Bits) Value

gb|EL751013.1| AD0008024 Taenia solium UNAM-cd1_adult Taenia ... 1229 0.0

gb|GT227128.1| tscaa0_001691.z1.scf Taenia solium adult full-... 861 0.0

gb|EL754973.1| AD0023229 Taenia solium UNAM-cd1_adult Taenia ... 425 2e-119

gb|EL754974.1| AD0023230 Taenia solium UNAM-cd1_adult Taenia ... 259 2e-69

Query= TS.seq.screen.trim.Contig451

Length=317

Score E

Sequences producing significant alignments: (Bits) Value

gb|EL762298.1| AD0182054 Taenia solium UNAM-cd1_adult Taenia ... 300 1e-81

gb|EL750022.1| AD0003164 Taenia solium UNAM-cd1_adult Taenia ... 300 1e-81

gb|EL751324.1| AD0009062 Taenia solium UNAM-cd1_adult Taenia ... 296 2e-80

gb|EL762211.1| AD0181008 Taenia solium UNAM-cd1_adult Taenia ... 283 1e-76

gb|EL759014.1| AD0115039 Taenia solium UNAM-cd1_adult Taenia ... 272 3e-73

gb|EL749796.1| AD0002160 Taenia solium UNAM-cd1_adult Taenia ... 231 5e-61

Query= TS.seq.screen.trim.Contig453

Length=694

Score E

Sequences producing significant alignments: (Bits) Value

gb|EL740611.1| LV0212047 Taenia solium UNAM-cd2_larva Taenia ... 460 6e-130

gb|EL742169.1| LV0237057 Taenia solium UNAM-cd2_larva Taenia ... 429 2e-120

Query= TS.seq.screen.trim.Contig454

Length=999

Score E

Sequences producing significant alignments: (Bits) Value

gb|EL755390.1| AD0025160 Taenia solium UNAM-cd1_adult Taenia ... 1330 0.0

gb|EL748382.1| LV0339020 Taenia solium UNAM-cd2_larva Taenia ... 1240 0.0

gb|EL748421.1| LV0339059 Taenia solium UNAM-cd2_larva Taenia ... 1205 0.0

gb|EL761411.1| AD0163020 Taenia solium UNAM-cd1_adult Taenia ... 1173 0.0

gb|EL759863.1| AD0131002 Taenia solium UNAM-cd1_adult Taenia ... 1170 0.0

gb|EL750151.1| AD0004066 Taenia solium UNAM-cd1_adult Taenia ... 1133 0.0

gb|EL750396.1| AD0005059 Taenia solium UNAM-cd1_adult Taenia ... 1129 0.0

gb|EL744546.1| LV0275021 Taenia solium UNAM-cd2_larva Taenia ... 1098 0.0

gb|EL741862.1| LV0232067 Taenia solium UNAM-cd2_larva Taenia ... 1096 0.0

gb|EL754546.1| AD0022071 Taenia solium UNAM-cd1_adult Taenia ... 1038 0.0

Query= TS.seq.screen.trim.Contig455

Length=925

Score E

Sequences producing significant alignments: (Bits) Value

gb|EL750222.1| AD0004137 Taenia solium UNAM-cd1_adult Taenia ... 1112 0.0

gb|EL759374.1| AD0122018 Taenia solium UNAM-cd1_adult Taenia ... 1105 0.0

gb|EL750817.1| AD0007053 Taenia solium UNAM-cd1_adult Taenia ... 1101 0.0

gb|EL750466.1| AD0005129 Taenia solium UNAM-cd1_adult Taenia ... 1096 0.0

gb|EL742019.1| LV0235008 Taenia solium UNAM-cd2_larva Taenia ... 1005 0.0

gb|EL761606.1| AD0166047 Taenia solium UNAM-cd1_adult Taenia ... 955 0.0

gb|EL750035.1| AD0003177 Taenia solium UNAM-cd1_adult Taenia ... 900 0.0

gb|EL760632.1| AD0146017 Taenia solium UNAM-cd1_adult Taenia ... 880 0.0

gb|EL751895.1| AD0011138 Taenia solium UNAM-cd1_adult Taenia ... 830 0.0

gb|EL755791.1| AD0027087 Taenia solium UNAM-cd1_adult Taenia ... 821 0.0

Query= TS.seq.screen.trim.Contig456

Length=201

Score E

Sequences producing significant alignments: (Bits) Value

gb|EL740723.1| LV0215013 Taenia solium UNAM-cd2_larva Taenia ... 274 9e-74

gb|EL762052.1| AD0177023 Taenia solium UNAM-cd1_adult Taenia ... 268 4e-72

gb|EX151213.1| TPEG001001H09 Cysti-host Taenia solium cDNA, m... 174 9e-44

gb|EX151282.1| TPEG001002G05 Cysti-host Taenia solium cDNA, m... 135 4e-32

gb|EL741854.1| LV0232059 Taenia solium UNAM-cd2_larva Taenia ... 56.5 3e-08

gb|EL746752.1| LV0313035 Taenia solium UNAM-cd2_larva Taenia ... 52.8 4e-07

Query= TS.seq.screen.trim.Contig457

Length=945

Score E

Sequences producing significant alignments: (Bits) Value

gb|EL746823.1| LV0314036 Taenia solium UNAM-cd2_larva Taenia ... 1103 0.0

gb|EL746648.1| LV0312009 Taenia solium UNAM-cd2_larva Taenia ... 987 0.0

gb|EX151382.1| TPEG001003H07 Cysti-host Taenia solium cDNA, m... 381 5e-106

gb|EL745626.1| LV0294041 Taenia solium UNAM-cd2_larva Taenia ... 372 3e-103

gb|EL756045.1| AD0028103 Taenia solium UNAM-cd1_adult Taenia ... 363 2e-100

gb|EL751722.1| AD0010215 Taenia solium UNAM-cd1_adult Taenia ... 351 4e-97

gb|EL751949.1| AD0011192 Taenia solium UNAM-cd1_adult Taenia ... 243 2e-64

gb|EX151211.1| TPEG001001H07 Cysti-host Taenia solium cDNA, m... 195 7e-50

gb|EX151376.1| TPEG001003G12 Cysti-host Taenia solium cDNA, m... 106 3e-23

gb|EX151282.1| TPEG001002G05 Cysti-host Taenia solium cDNA, m... 84.2 2e-16

Query= TS.seq.screen.trim.Contig458

Length=438

Score E

Sequences producing significant alignments: (Bits) Value

gb|EL754033.1| AD0020078 Taenia solium UNAM-cd1_adult Taenia ... 497 5e-141

gb|EL760116.1| AD0136007 Taenia solium UNAM-cd1_adult Taenia ... 279 2e-75

gb|EL749934.1| AD0003076 Taenia solium UNAM-cd1_adult Taenia ... 279 2e-75

gb|EL749711.1| AD0002075 Taenia solium UNAM-cd1_adult Taenia ... 265 5e-71

gb|EL741854.1| LV0232059 Taenia solium UNAM-cd2_larva Taenia ... 56.5 3e-08

Query= TS.seq.screen.trim.Contig459

Length=1091

Score E

Sequences producing significant alignments: (Bits) Value

gb|EL747590.1| LV0326006 Taenia solium UNAM-cd2_larva Taenia ... 1002 0.0

Query= TS.seq.screen.trim.Contig461

Length=1120

Score E

Sequences producing significant alignments: (Bits) Value

gb|EL749400.1| AD0001023 Taenia solium UNAM-cd1_adult Taenia ... 1277 0.0

gb|EX151135.1| TPEG001001A04 Cysti-host Taenia solium cDNA, m... 1171 0.0

gb|EL749366.1| LV0359044 Taenia solium UNAM-cd2_larva Taenia ... 1162 0.0

gb|EL762340.1| AD0183041 Taenia solium UNAM-cd1_adult Taenia ... 1129 0.0

gb|EL743278.1| LV0254059 Taenia solium UNAM-cd2_larva Taenia ... 1079 0.0

gb|GT227110.1| tscaa0_001068.z1.scf Taenia solium adult full-... 1005 0.0

gb|EL747711.1| LV0327054 Taenia solium UNAM-cd2_larva Taenia ... 931 0.0

gb|EL758859.1| AD0112058 Taenia solium UNAM-cd1_adult Taenia ... 928 0.0

gb|EL740371.1| LV0207055 Taenia solium UNAM-cd2_larva Taenia ... 885 0.0

gb|EX151201.1| TPEG001001G04 Cysti-host Taenia solium cDNA, m... 857 0.0

Query= TS.seq.screen.trim.Contig462

Length=425

Score E

Sequences producing significant alignments: (Bits) Value

gb|EL750022.1| AD0003164 Taenia solium UNAM-cd1_adult Taenia ... 372 3e-103

gb|EL751324.1| AD0009062 Taenia solium UNAM-cd1_adult Taenia ... 355 3e-98

gb|EL759014.1| AD0115039 Taenia solium UNAM-cd1_adult Taenia ... 350 1e-96

gb|EL762211.1| AD0181008 Taenia solium UNAM-cd1_adult Taenia ... 344 7e-95

gb|EL762298.1| AD0182054 Taenia solium UNAM-cd1_adult Taenia ... 326 2e-89

gb|EL749796.1| AD0002160 Taenia solium UNAM-cd1_adult Taenia ... 298 5e-81

gb|EL741854.1| LV0232059 Taenia solium UNAM-cd2_larva Taenia ... 56.5 3e-08

Query= TS.seq.screen.trim.Contig463

Length=728

Score E

Sequences producing significant alignments: (Bits) Value

gb|EL743911.1| LV0264040 Taenia solium UNAM-cd2_larva Taenia ... 1170 0.0

gb|EL740514.1| LV0210038 Taenia solium UNAM-cd2_larva Taenia ... 1061 0.0

gb|EL760824.1| AD0149040 Taenia solium UNAM-cd1_adult Taenia ... 797 0.0

gb|EL748089.1| LV0333040 Taenia solium UNAM-cd2_larva Taenia ... 597 5e-171

gb|GT227629.1| tscaa0_001811.z1.scf Taenia solium adult full-... 586 1e-167

gb|EL742067.1| LV0235056 Taenia solium UNAM-cd2_larva Taenia ... 584 4e-167

gb|EL741735.1| LV0231013 Taenia solium UNAM-cd2_larva Taenia ... 566 1e-161

gb|EL745069.1| LV0285041 Taenia solium UNAM-cd2_larva Taenia ... 558 2e-159

gb|EL746066.1| LV0301021 Taenia solium UNAM-cd2_larva Taenia ... 523 8e-149

gb|EL759027.1| AD0115052 Taenia solium UNAM-cd1_adult Taenia ... 475 2e-134

Query= TS.seq.screen.trim.Contig464

Length=638

Score E

Sequences producing significant alignments: (Bits) Value

gb|EL741854.1| LV0232059 Taenia solium UNAM-cd2_larva Taenia ... 63.9 2e-10

Query= TS.seq.screen.trim.Contig465

Length=458

Score E

Sequences producing significant alignments: (Bits) Value

gb|GT227146.1| tscaa0_001801.z1.scf Taenia solium adult full-... 782 0.0

gb|EL758135.1| AD0101015 Taenia solium UNAM-cd1_adult Taenia ... 780 0.0

gb|EL760258.1| AD0138036 Taenia solium UNAM-cd1_adult Taenia ... 778 0.0

gb|GT227145.1| tscaa0_002549.z1.scf Taenia solium adult full-... 776 0.0

gb|GT227056.1| tscaa0_001529.z1.scf Taenia solium adult full-... 776 0.0

gb|EL759619.1| AD0126016 Taenia solium UNAM-cd1_adult Taenia ... 776 0.0

gb|EL758005.1| AD0099017 Taenia solium UNAM-cd1_adult Taenia ... 776 0.0

gb|EL760635.1| AD0146020 Taenia solium UNAM-cd1_adult Taenia ... 774 0.0

gb|GT227023.1| tscaa0_000994.z1.scf Taenia solium adult full-... 771 0.0

gb|EL760719.1| AD0147051 Taenia solium UNAM-cd1_adult Taenia ... 767 0.0

Query= TS.seq.screen.trim.Contig466

Length=423

Score E

Sequences producing significant alignments: (Bits) Value

gb|EL741854.1| LV0232059 Taenia solium UNAM-cd2_larva Taenia ... 58.4 1e-08

Query= TS.seq.screen.trim.Contig467

Length=1157

Score E

Sequences producing significant alignments: (Bits) Value

gb|EL754279.1| AD0021069 Taenia solium UNAM-cd1_adult Taenia ... 1240 0.0

gb|EL752175.1| AD0012203 Taenia solium UNAM-cd1_adult Taenia ... 1197 0.0

gb|EL762753.1| AD0192051 Taenia solium UNAM-cd1_adult Taenia ... 1158 0.0

gb|EL763065.1| AD0198003 Taenia solium UNAM-cd1_adult Taenia ... 1136 0.0

gb|EL740635.1| LV0213017 Taenia solium UNAM-cd2_larva Taenia ... 1099 0.0

gb|EL757388.1| AD0034204 Taenia solium UNAM-cd1_adult Taenia ... 1070 0.0

gb|EL740959.1| LV0219009 Taenia solium UNAM-cd2_larva Taenia ... 1048 0.0

gb|GT226938.1| tscaa0_001025.z1.scf Taenia solium adult full-... 741 0.0

gb|EL760346.1| AD0140027 Taenia solium UNAM-cd1_adult Taenia ... 678 0.0

gb|EL762975.1| AD0196028 Taenia solium UNAM-cd1_adult Taenia ... 555 3e-158

Query= TS.seq.screen.trim.Contig469

Length=1546

Score E

Sequences producing significant alignments: (Bits) Value

gb|GT227635.1| tscaa0_001825.z1.scf Taenia solium adult full-... 411 6e-115

Query= TS.seq.screen.trim.Contig470

Length=1412

Score E

Sequences producing significant alignments: (Bits) Value

gb|EL746716.1| LV0312077 Taenia solium UNAM-cd2_larva Taenia ... 972 0.0

gb|EL740938.1| LV0218049 Taenia solium UNAM-cd2_larva Taenia ... 632 0.0

gb|EL741854.1| LV0232059 Taenia solium UNAM-cd2_larva Taenia ... 56.5 3e-08

Query= TS.seq.screen.trim.Contig471

Length=621

Score E

Sequences producing significant alignments: (Bits) Value

gb|EL741854.1| LV0232059 Taenia solium UNAM-cd2_larva Taenia ... 58.4 1e-08

Query= TS.seq.screen.trim.Contig472

Length=986

Score E

Sequences producing significant alignments: (Bits) Value

gb|EL747431.1| LV0323040 Taenia solium UNAM-cd2_larva Taenia ... 789 0.0

gb|EL748123.1| LV0334006 Taenia solium UNAM-cd2_larva Taenia ... 531 5e-151

gb|EL741183.1| LV0222016 Taenia solium UNAM-cd2_larva Taenia ... 475 2e-134

gb|EL745797.1| LV0297026 Taenia solium UNAM-cd2_larva Taenia ... 298 5e-81

gb|EL744496.1| LV0273054 Taenia solium UNAM-cd2_larva Taenia ... 296 2e-80

gb|EL741854.1| LV0232059 Taenia solium UNAM-cd2_larva Taenia ... 58.4 1e-08

Query= TS.seq.screen.trim.Contig473

Length=1132

Score E

Sequences producing significant alignments: (Bits) Value

gb|EL744248.1| LV0269059 Taenia solium UNAM-cd2_larva Taenia ... 1282 0.0

gb|EL742595.1| LV0244026 Taenia solium UNAM-cd2_larva Taenia ... 1280 0.0

gb|EL751109.1| AD0008120 Taenia solium UNAM-cd1_adult Taenia ... 1203 0.0

gb|EL749193.1| LV0355007 Taenia solium UNAM-cd2_larva Taenia ... 1171 0.0

gb|EL751990.1| AD0012018 Taenia solium UNAM-cd1_adult Taenia ... 1142 0.0

gb|EL752249.1| AD0013016 Taenia solium UNAM-cd1_adult Taenia ... 1044 0.0

gb|EL761781.1| AD0170052 Taenia solium UNAM-cd1_adult Taenia ... 1002 0.0

gb|EL748612.1| LV0342063 Taenia solium UNAM-cd2_larva Taenia ... 994 0.0

gb|EL746748.1| LV0313031 Taenia solium UNAM-cd2_larva Taenia ... 983 0.0

gb|EL743924.1| LV0264053 Taenia solium UNAM-cd2_larva Taenia ... 965 0.0

Query= TS.seq.screen.trim.Contig474

Length=672

Score E

Sequences producing significant alignments: (Bits) Value

gb|EL763237.1| AD0200052 Taenia solium UNAM-cd1_adult Taenia ... 1146 0.0

gb|EL760891.1| AD0152016 Taenia solium UNAM-cd1_adult Taenia ... 950 0.0

gb|EL746828.1| LV0314041 Taenia solium UNAM-cd2_larva Taenia ... 918 0.0

gb|EL742106.1| LV0236035 Taenia solium UNAM-cd2_larva Taenia ... 911 0.0

gb|EL751865.1| AD0011108 Taenia solium UNAM-cd1_adult Taenia ... 872 0.0

gb|EL759637.1| AD0126034 Taenia solium UNAM-cd1_adult Taenia ... 869 0.0

gb|EL763049.1| AD0197058 Taenia solium UNAM-cd1_adult Taenia ... 863 0.0

gb|EL758674.1| AD0109049 Taenia solium UNAM-cd1_adult Taenia ... 863 0.0

gb|EL751631.1| AD0010124 Taenia solium UNAM-cd1_adult Taenia ... 859 0.0

gb|EL743317.1| LV0255031 Taenia solium UNAM-cd2_larva Taenia ... 859 0.0

Query= TS.seq.screen.trim.Contig475

Length=707

Score E

Sequences producing significant alignments: (Bits) Value

gb|EL747569.1| LV0325048 Taenia solium UNAM-cd2_larva Taenia ... 793 0.0

gb|EL758248.1| AD0102059 Taenia solium UNAM-cd1_adult Taenia ... 791 0.0

gb|EL758206.1| AD0102017 Taenia solium UNAM-cd1_adult Taenia ... 791 0.0

gb|EL749222.1| LV0355036 Taenia solium UNAM-cd2_larva Taenia ... 791 0.0

gb|EL747321.1| LV0321052 Taenia solium UNAM-cd2_larva Taenia ... 791 0.0

gb|EL743976.1| LV0265048 Taenia solium UNAM-cd2_larva Taenia ... 791 0.0

gb|EL742217.1| LV0238036 Taenia solium UNAM-cd2_larva Taenia ... 791 0.0

gb|EL746879.1| LV0315021 Taenia solium UNAM-cd2_larva Taenia ... 789 0.0

gb|EL742596.1| LV0244027 Taenia solium UNAM-cd2_larva Taenia ... 782 0.0

gb|EL753067.1| AD0016105 Taenia solium UNAM-cd1_adult Taenia ... 780 0.0

Query= TS.seq.screen.trim.Contig476

Length=1012

Score E

Sequences producing significant alignments: (Bits) Value

gb|EL759583.1| AD0125041 Taenia solium UNAM-cd1_adult Taenia ... 1242 0.0

gb|EL754884.1| AD0023140 Taenia solium UNAM-cd1_adult Taenia ... 1242 0.0

gb|EL754472.1| AD0021262 Taenia solium UNAM-cd1_adult Taenia ... 1242 0.0

gb|EL754309.1| AD0021099 Taenia solium UNAM-cd1_adult Taenia ... 1242 0.0

gb|EL754016.1| AD0020061 Taenia solium UNAM-cd1_adult Taenia ... 1242 0.0

gb|EL753647.1| AD0018185 Taenia solium UNAM-cd1_adult Taenia ... 1242 0.0

gb|EL753480.1| AD0018018 Taenia solium UNAM-cd1_adult Taenia ... 1242 0.0

gb|EL753055.1| AD0016093 Taenia solium UNAM-cd1_adult Taenia ... 1242 0.0

gb|EL752036.1| AD0012064 Taenia solium UNAM-cd1_adult Taenia ... 1242 0.0

gb|EL751999.1| AD0012027 Taenia solium UNAM-cd1_adult Taenia ... 1242 0.0

Query= TS.seq.screen.trim.Contig477

Length=1120

Score E

Sequences producing significant alignments: (Bits) Value

gb|EL760116.1| AD0136007 Taenia solium UNAM-cd1_adult Taenia ... 575 2e-164

gb|EL749934.1| AD0003076 Taenia solium UNAM-cd1_adult Taenia ... 564 5e-161

gb|EL749711.1| AD0002075 Taenia solium UNAM-cd1_adult Taenia ... 553 1e-157

gb|EL742126.1| LV0237014 Taenia solium UNAM-cd2_larva Taenia ... 553 1e-157

gb|EL740529.1| LV0211006 Taenia solium UNAM-cd2_larva Taenia ... 383 1e-106

gb|EL746994.1| LV0316071 Taenia solium UNAM-cd2_larva Taenia ... 379 2e-105

gb|EL743873.1| LV0264002 Taenia solium UNAM-cd2_larva Taenia ... 379 2e-105

gb|EL743155.1| LV0253002 Taenia solium UNAM-cd2_larva Taenia ... 379 2e-105

gb|EL741453.1| LV0227009 Taenia solium UNAM-cd2_larva Taenia ... 379 2e-105

gb|EL745590.1| LV0294005 Taenia solium UNAM-cd2_larva Taenia ... 377 7e-105

Query= TS.seq.screen.trim.Contig478

Length=1011

Score E

Sequences producing significant alignments: (Bits) Value

gb|EL740723.1| LV0215013 Taenia solium UNAM-cd2_larva Taenia ... 499 1e-141

gb|EL762052.1| AD0177023 Taenia solium UNAM-cd1_adult Taenia ... 438 3e-123

gb|EX151213.1| TPEG001001H09 Cysti-host Taenia solium cDNA, m... 327 7e-90

gb|EX150563.1| TSEDTS1008G12 Cysti Taenia solium cDNA, mRNA s... 239 3e-63

gb|EX151282.1| TPEG001002G05 Cysti-host Taenia solium cDNA, m... 135 4e-32

Query= TS.seq.screen.trim.Contig479

Length=1182

Score E

Sequences producing significant alignments: (Bits) Value

gb|EL763025.1| AD0197034 Taenia solium UNAM-cd1_adult Taenia ... 1164 0.0

gb|EX150392.1| TSEDTS1009G01 Cysti Taenia solium cDNA, mRNA s... 1057 0.0

gb|EL743454.1| LV0257071 Taenia solium UNAM-cd2_larva Taenia ... 1003 0.0

gb|EL743807.1| LV0262048 Taenia solium UNAM-cd2_larva Taenia ... 998 0.0

gb|EL758807.1| AD0112006 Taenia solium UNAM-cd1_adult Taenia ... 953 0.0

gb|EL748320.1| LV0338009 Taenia solium UNAM-cd2_larva Taenia ... 935 0.0

gb|EL758545.1| AD0107038 Taenia solium UNAM-cd1_adult Taenia ... 905 0.0

gb|EL757709.1| AD0094049 Taenia solium UNAM-cd1_adult Taenia ... 900 0.0

gb|EL745521.1| LV0292055 Taenia solium UNAM-cd2_larva Taenia ... 893 0.0

gb|EL742279.1| LV0239031 Taenia solium UNAM-cd2_larva Taenia ... 887 0.0

Query= TS.seq.screen.trim.Contig480

Length=851

Score E

Sequences producing significant alignments: (Bits) Value

gb|EL757991.1| AD0099003 Taenia solium UNAM-cd1_adult Taenia ... 1264 0.0

gb|EL753583.1| AD0018121 Taenia solium UNAM-cd1_adult Taenia ... 1258 0.0

gb|EL750810.1| AD0007046 Taenia solium UNAM-cd1_adult Taenia ... 1249 0.0

gb|EL752089.1| AD0012117 Taenia solium UNAM-cd1_adult Taenia ... 1247 0.0

gb|EL751299.1| AD0009037 Taenia solium UNAM-cd1_adult Taenia ... 1247 0.0

gb|GT227145.1| tscaa0_002549.z1.scf Taenia solium adult full-... 1238 0.0

gb|EL753018.1| AD0016056 Taenia solium UNAM-cd1_adult Taenia ... 1230 0.0

gb|EL750771.1| AD0007007 Taenia solium UNAM-cd1_adult Taenia ... 1225 0.0

gb|EL750052.1| AD0003194 Taenia solium UNAM-cd1_adult Taenia ... 1208 0.0

gb|EL762425.1| AD0185030 Taenia solium UNAM-cd1_adult Taenia ... 1192 0.0

Query= TS.seq.screen.trim.Contig481

Length=931

Score E

Sequences producing significant alignments: (Bits) Value

gb|EL746287.1| LV0307001 Taenia solium UNAM-cd2_larva Taenia ... 680 0.0

gb|EL740984.1| LV0219034 Taenia solium UNAM-cd2_larva Taenia ... 671 0.0

gb|EL748642.1| LV0343027 Taenia solium UNAM-cd2_larva Taenia ... 667 0.0

gb|EL742380.1| LV0241006 Taenia solium UNAM-cd2_larva Taenia ... 667 0.0

gb|EL745611.1| LV0294026 Taenia solium UNAM-cd2_larva Taenia ... 665 0.0

gb|EL761249.1| AD0159046 Taenia solium UNAM-cd1_adult Taenia ... 656 0.0

gb|EL748529.1| LV0341045 Taenia solium UNAM-cd2_larva Taenia ... 654 0.0

gb|EL751385.1| AD0009123 Taenia solium UNAM-cd1_adult Taenia ... 651 0.0

gb|EL763243.1| AD0201006 Taenia solium UNAM-cd1_adult Taenia ... 649 0.0

gb|EL746630.1| LV0311059 Taenia solium UNAM-cd2_larva Taenia ... 649 0.0

Query= TS.seq.screen.trim.Contig482

Length=1167

Score E

Sequences producing significant alignments: (Bits) Value

gb|EL763074.1| AD0198012 Taenia solium UNAM-cd1_adult Taenia ... 1260 0.0

gb|EL748121.1| LV0334004 Taenia solium UNAM-cd2_larva Taenia ... 1247 0.0

gb|EL746614.1| LV0311043 Taenia solium UNAM-cd2_larva Taenia ... 1101 0.0

gb|EL741319.1| LV0224039 Taenia solium UNAM-cd2_larva Taenia ... 981 0.0

gb|EL741221.1| LV0222054 Taenia solium UNAM-cd2_larva Taenia ... 976 0.0

gb|EL743050.1| LV0251015 Taenia solium UNAM-cd2_larva Taenia ... 939 0.0

gb|EL743202.1| LV0253049 Taenia solium UNAM-cd2_larva Taenia ... 911 0.0

gb|EL744777.1| LV0280003 Taenia solium UNAM-cd2_larva Taenia ... 878 0.0

gb|EL743059.1| LV0251024 Taenia solium UNAM-cd2_larva Taenia ... 599 1e-171

Query= TS.seq.screen.trim.Contig483

Length=878

Score E

Sequences producing significant alignments: (Bits) Value

gb|EL741854.1| LV0232059 Taenia solium UNAM-cd2_larva Taenia ... 56.5 3e-08

Query= TS.seq.screen.trim.Contig484

Length=667

Score E

Sequences producing significant alignments: (Bits) Value

gb|EL741854.1| LV0232059 Taenia solium UNAM-cd2_larva Taenia ... 56.5 3e-08

gb|EL746752.1| LV0313035 Taenia solium UNAM-cd2_larva Taenia ... 52.8 4e-07

Query= TS.seq.screen.trim.Contig485

Length=1086

Score E

Sequences producing significant alignments: (Bits) Value

gb|GT227145.1| tscaa0_002549.z1.scf Taenia solium adult full-... 1698 0.0

gb|EL755806.1| AD0027102 Taenia solium UNAM-cd1_adult Taenia ... 1509 0.0

gb|EL751999.1| AD0012027 Taenia solium UNAM-cd1_adult Taenia ... 1487 0.0

gb|EL752036.1| AD0012064 Taenia solium UNAM-cd1_adult Taenia ... 1447 0.0

gb|EL752089.1| AD0012117 Taenia solium UNAM-cd1_adult Taenia ... 1434 0.0

gb|EL753583.1| AD0018121 Taenia solium UNAM-cd1_adult Taenia ... 1430 0.0

gb|EL753055.1| AD0016093 Taenia solium UNAM-cd1_adult Taenia ... 1423 0.0

gb|EL756173.1| AD0028231 Taenia solium UNAM-cd1_adult Taenia ... 1393 0.0

gb|EL753731.1| AD0019018 Taenia solium UNAM-cd1_adult Taenia ... 1373 0.0

gb|EL753647.1| AD0018185 Taenia solium UNAM-cd1_adult Taenia ... 1362 0.0

Query= TS.seq.screen.trim.Contig486

Length=767

Score E

Sequences producing significant alignments: (Bits) Value

gb|EL751337.1| AD0009075 Taenia solium UNAM-cd1_adult Taenia ... 953 0.0

gb|EL751081.1| AD0008092 Taenia solium UNAM-cd1_adult Taenia ... 950 0.0

gb|EL749490.1| AD0001113 Taenia solium UNAM-cd1_adult Taenia ... 658 0.0

gb|EL753346.1| AD0017104 Taenia solium UNAM-cd1_adult Taenia ... 654 0.0

gb|EL757485.1| AD0035078 Taenia solium UNAM-cd1_adult Taenia ... 651 0.0

gb|EL753100.1| AD0016138 Taenia solium UNAM-cd1_adult Taenia ... 651 0.0

gb|EL757404.1| AD0034220 Taenia solium UNAM-cd1_adult Taenia ... 645 0.0

Query= TS.seq.screen.trim.Contig487

Length=442

Score E

Sequences producing significant alignments: (Bits) Value

gb|EL742012.1| LV0235001 Taenia solium UNAM-cd2_larva Taenia ... 719 0.0

gb|EL748227.1| LV0336003 Taenia solium UNAM-cd2_larva Taenia ... 713 0.0

gb|EX151138.1| TPEG001001A07 Cysti-host Taenia solium cDNA, m... 584 4e-167

gb|EX151156.1| TPEG001001C03 Cysti-host Taenia solium cDNA, m... 505 3e-143

gb|EL748580.1| LV0342031 Taenia solium UNAM-cd2_larva Taenia ... 473 8e-134

gb|EL749249.1| LV0356027 Taenia solium UNAM-cd2_larva Taenia ... 449 1e-126

gb|EL743493.1| LV0258033 Taenia solium UNAM-cd2_larva Taenia ... 353 1e-97

gb|EX151195.1| TPEG001001F09 Cysti-host Taenia solium cDNA, m... 311 7e-85

gb|GT227134.1| tscaa0_000921.z1.scf Taenia solium adult full-... 289 3e-78

gb|EL757934.1| AD0098019 Taenia solium UNAM-cd1_adult Taenia ... 289 3e-78

Query= TS.seq.screen.trim.Contig488

Length=616

Score E

Sequences producing significant alignments: (Bits) Value

gb|EL746287.1| LV0307001 Taenia solium UNAM-cd2_larva Taenia ... 1020 0.0

gb|EL745611.1| LV0294026 Taenia solium UNAM-cd2_larva Taenia ... 1011 0.0

gb|EL751385.1| AD0009123 Taenia solium UNAM-cd1_adult Taenia ... 996 0.0

gb|EL761249.1| AD0159046 Taenia solium UNAM-cd1_adult Taenia ... 992 0.0

gb|EL752363.1| AD0013130 Taenia solium UNAM-cd1_adult Taenia ... 985 0.0

gb|EL750856.1| AD0007092 Taenia solium UNAM-cd1_adult Taenia ... 985 0.0

gb|EL752868.1| AD0015127 Taenia solium UNAM-cd1_adult Taenia ... 983 0.0

gb|EL753398.1| AD0017156 Taenia solium UNAM-cd1_adult Taenia ... 981 0.0

gb|EL750072.1| AD0003214 Taenia solium UNAM-cd1_adult Taenia ... 981 0.0

gb|EL752315.1| AD0013082 Taenia solium UNAM-cd1_adult Taenia ... 977 0.0

Query= TS.seq.screen.trim.Contig489

Length=982

Score E

Sequences producing significant alignments: (Bits) Value

gb|EL742187.1| LV0238006 Taenia solium UNAM-cd2_larva Taenia ... 843 0.0

gb|EL763309.1| AD0202009 Taenia solium UNAM-cd1_adult Taenia ... 828 0.0

gb|EL751121.1| AD0008132 Taenia solium UNAM-cd1_adult Taenia ... 828 0.0

gb|EL749562.1| AD0001185 Taenia solium UNAM-cd1_adult Taenia ... 828 0.0

gb|EL751376.1| AD0009114 Taenia solium UNAM-cd1_adult Taenia ... 819 0.0

gb|EL746458.1| LV0309028 Taenia solium UNAM-cd2_larva Taenia ... 809 0.0

gb|EL742444.1| LV0242008 Taenia solium UNAM-cd2_larva Taenia ... 806 0.0

gb|EL761370.1| AD0162031 Taenia solium UNAM-cd1_adult Taenia ... 756 0.0

gb|EL742455.1| LV0242019 Taenia solium UNAM-cd2_larva Taenia ... 627 6e-180

gb|EL748741.1| LV0344064 Taenia solium UNAM-cd2_larva Taenia ... 603 1e-172

Query= TS.seq.screen.trim.Contig490

Length=1002

Score E

Sequences producing significant alignments: (Bits) Value

gb|GT227145.1| tscaa0_002549.z1.scf Taenia solium adult full-... 1696 0.0

gb|EL755806.1| AD0027102 Taenia solium UNAM-cd1_adult Taenia ... 1526 0.0

gb|EL751999.1| AD0012027 Taenia solium UNAM-cd1_adult Taenia ... 1504 0.0

gb|EL752036.1| AD0012064 Taenia solium UNAM-cd1_adult Taenia ... 1463 0.0

gb|EL752089.1| AD0012117 Taenia solium UNAM-cd1_adult Taenia ... 1445 0.0

gb|EL753583.1| AD0018121 Taenia solium UNAM-cd1_adult Taenia ... 1439 0.0

gb|EL753055.1| AD0016093 Taenia solium UNAM-cd1_adult Taenia ... 1432 0.0

gb|EL756173.1| AD0028231 Taenia solium UNAM-cd1_adult Taenia ... 1410 0.0

gb|EL753731.1| AD0019018 Taenia solium UNAM-cd1_adult Taenia ... 1389 0.0

gb|EL755652.1| AD0026175 Taenia solium UNAM-cd1_adult Taenia ... 1376 0.0

Query= TS.seq.screen.trim.Contig491

Length=1063

Score E

Sequences producing significant alignments: (Bits) Value

gb|EL749173.1| LV0354032 Taenia solium UNAM-cd2_larva Taenia ... 1190 0.0

gb|EL749069.1| LV0352024 Taenia solium UNAM-cd2_larva Taenia ... 1114 0.0

gb|EL748268.1| LV0336044 Taenia solium UNAM-cd2_larva Taenia ... 1079 0.0

gb|EL748897.1| LV0348008 Taenia solium UNAM-cd2_larva Taenia ... 891 0.0

gb|EL749051.1| LV0352006 Taenia solium UNAM-cd2_larva Taenia ... 761 0.0

gb|EL757934.1| AD0098019 Taenia solium UNAM-cd1_adult Taenia ... 739 0.0

gb|EL748227.1| LV0336003 Taenia solium UNAM-cd2_larva Taenia ... 725 0.0

gb|EL742012.1| LV0235001 Taenia solium UNAM-cd2_larva Taenia ... 641 0.0

gb|EL748205.1| LV0335020 Taenia solium UNAM-cd2_larva Taenia ... 558 2e-159

gb|EL740723.1| LV0215013 Taenia solium UNAM-cd2_larva Taenia ... 488 3e-138

Query= TS.seq.screen.trim.Contig492

Length=1111

Score E

Sequences producing significant alignments: (Bits) Value

gb|EL758005.1| AD0099017 Taenia solium UNAM-cd1_adult Taenia ... 852 0.0

gb|EL758135.1| AD0101015 Taenia solium UNAM-cd1_adult Taenia ... 850 0.0

gb|EL760258.1| AD0138036 Taenia solium UNAM-cd1_adult Taenia ... 848 0.0

gb|EL759619.1| AD0126016 Taenia solium UNAM-cd1_adult Taenia ... 846 0.0

gb|EL760635.1| AD0146020 Taenia solium UNAM-cd1_adult Taenia ... 845 0.0

gb|EL759570.1| AD0125028 Taenia solium UNAM-cd1_adult Taenia ... 843 0.0

gb|EL754978.1| AD0023234 Taenia solium UNAM-cd1_adult Taenia ... 843 0.0

gb|EL753731.1| AD0019018 Taenia solium UNAM-cd1_adult Taenia ... 843 0.0

gb|EL760719.1| AD0147051 Taenia solium UNAM-cd1_adult Taenia ... 837 0.0

gb|EL754172.1| AD0020217 Taenia solium UNAM-cd1_adult Taenia ... 837 0.0

Query= TS.seq.screen.trim.Contig493

Length=1122

Score E

Sequences producing significant alignments: (Bits) Value

gb|EL757991.1| AD0099003 Taenia solium UNAM-cd1_adult Taenia ... 723 0.0

gb|EL748377.1| LV0339015 Taenia solium UNAM-cd2_larva Taenia ... 708 0.0

gb|EL757923.1| AD0098008 Taenia solium UNAM-cd1_adult Taenia ... 702 0.0

gb|EL753583.1| AD0018121 Taenia solium UNAM-cd1_adult Taenia ... 701 0.0

gb|EL740634.1| LV0213016 Taenia solium UNAM-cd2_larva Taenia ... 699 0.0

gb|EL759489.1| AD0124003 Taenia solium UNAM-cd1_adult Taenia ... 697 0.0

gb|EL758829.1| AD0112028 Taenia solium UNAM-cd1_adult Taenia ... 697 0.0

gb|EL748166.1| LV0334049 Taenia solium UNAM-cd2_larva Taenia ... 697 0.0

gb|GT227145.1| tscaa0_002549.z1.scf Taenia solium adult full-... 691 0.0

gb|EL762425.1| AD0185030 Taenia solium UNAM-cd1_adult Taenia ... 691 0.0

Query= TS.seq.screen.trim.Contig494

Length=965

Score E

Sequences producing significant alignments: (Bits) Value

gb|EL746348.1| LV0307062 Taenia solium UNAM-cd2_larva Taenia ... 785 0.0

gb|EL761287.1| AD0161004 Taenia solium UNAM-cd1_adult Taenia ... 780 0.0

gb|EL750022.1| AD0003164 Taenia solium UNAM-cd1_adult Taenia ... 375 2e-104

gb|EL751324.1| AD0009062 Taenia solium UNAM-cd1_adult Taenia ... 364 5e-101

gb|EL759014.1| AD0115039 Taenia solium UNAM-cd1_adult Taenia ... 353 1e-97

gb|EL762211.1| AD0181008 Taenia solium UNAM-cd1_adult Taenia ... 348 5e-96

gb|EL762298.1| AD0182054 Taenia solium UNAM-cd1_adult Taenia ... 329 2e-90

gb|EL749796.1| AD0002160 Taenia solium UNAM-cd1_adult Taenia ... 298 5e-81

Query= TS.seq.screen.trim.Contig495

Length=817

Score E

Sequences producing significant alignments: (Bits) Value

gb|EL757991.1| AD0099003 Taenia solium UNAM-cd1_adult Taenia ... 1123 0.0

gb|EL762425.1| AD0185030 Taenia solium UNAM-cd1_adult Taenia ... 1118 0.0

gb|EL750810.1| AD0007046 Taenia solium UNAM-cd1_adult Taenia ... 1118 0.0

gb|EL750052.1| AD0003194 Taenia solium UNAM-cd1_adult Taenia ... 1118 0.0

gb|EL749345.1| LV0359023 Taenia solium UNAM-cd2_larva Taenia ... 1118 0.0

gb|EL753018.1| AD0016056 Taenia solium UNAM-cd1_adult Taenia ... 1116 0.0

gb|EL752089.1| AD0012117 Taenia solium UNAM-cd1_adult Taenia ... 1116 0.0

gb|EL751299.1| AD0009037 Taenia solium UNAM-cd1_adult Taenia ... 1116 0.0

gb|GT227145.1| tscaa0_002549.z1.scf Taenia solium adult full-... 1107 0.0

gb|EL753583.1| AD0018121 Taenia solium UNAM-cd1_adult Taenia ... 1105 0.0

Query= TS.seq.screen.trim.Contig496

Length=835

Score E

Sequences producing significant alignments: (Bits) Value

gb|EL748580.1| LV0342031 Taenia solium UNAM-cd2_larva Taenia ... 1116 0.0

gb|EL743493.1| LV0258033 Taenia solium UNAM-cd2_larva Taenia ... 1000 0.0

gb|EX151156.1| TPEG001001C03 Cysti-host Taenia solium cDNA, m... 990 0.0

gb|GT227134.1| tscaa0_000921.z1.scf Taenia solium adult full-... 968 0.0

gb|EL742012.1| LV0235001 Taenia solium UNAM-cd2_larva Taenia ... 917 0.0

gb|EL748227.1| LV0336003 Taenia solium UNAM-cd2_larva Taenia ... 800 0.0

gb|EL748959.1| LV0349008 Taenia solium UNAM-cd2_larva Taenia ... 717 0.0

gb|EL745155.1| LV0287009 Taenia solium UNAM-cd2_larva Taenia ... 713 0.0

gb|EL744320.1| LV0271002 Taenia solium UNAM-cd2_larva Taenia ... 641 0.0

gb|EX151138.1| TPEG001001A07 Cysti-host Taenia solium cDNA, m... 634 0.0

Query= TS.seq.screen.trim.Contig497

Length=1284

Score E

Sequences producing significant alignments: (Bits) Value

gb|EL755733.1| AD0027029 Taenia solium UNAM-cd1_adult Taenia ... 1652 0.0

gb|EL756302.1| AD0029097 Taenia solium UNAM-cd1_adult Taenia ... 1611 0.0

gb|EL752619.1| AD0014160 Taenia solium UNAM-cd1_adult Taenia ... 1531 0.0

gb|EL752122.1| AD0012150 Taenia solium UNAM-cd1_adult Taenia ... 1526 0.0

gb|EL756333.1| AD0029128 Taenia solium UNAM-cd1_adult Taenia ... 1524 0.0

gb|EL756846.1| AD0031119 Taenia solium UNAM-cd1_adult Taenia ... 1498 0.0

gb|EL752066.1| AD0012094 Taenia solium UNAM-cd1_adult Taenia ... 1495 0.0

gb|EL756496.1| AD0030028 Taenia solium UNAM-cd1_adult Taenia ... 1458 0.0

gb|EL755343.1| AD0025113 Taenia solium UNAM-cd1_adult Taenia ... 1448 0.0

gb|EL753164.1| AD0016202 Taenia solium UNAM-cd1_adult Taenia ... 1443 0.0

Query= TS.seq.screen.trim.Contig498

Length=1105

Score E

Sequences producing significant alignments: (Bits) Value

gb|EL750810.1| AD0007046 Taenia solium UNAM-cd1_adult Taenia ... 1352 0.0

gb|EL752089.1| AD0012117 Taenia solium UNAM-cd1_adult Taenia ... 1345 0.0

gb|EL753018.1| AD0016056 Taenia solium UNAM-cd1_adult Taenia ... 1339 0.0

gb|EL753583.1| AD0018121 Taenia solium UNAM-cd1_adult Taenia ... 1338 0.0

gb|GT227145.1| tscaa0_002549.z1.scf Taenia solium adult full-... 1336 0.0

gb|EL751299.1| AD0009037 Taenia solium UNAM-cd1_adult Taenia ... 1293 0.0

gb|EL753647.1| AD0018185 Taenia solium UNAM-cd1_adult Taenia ... 1291 0.0

gb|EL755806.1| AD0027102 Taenia solium UNAM-cd1_adult Taenia ... 1290 0.0

gb|EL754016.1| AD0020061 Taenia solium UNAM-cd1_adult Taenia ... 1280 0.0

gb|EL750771.1| AD0007007 Taenia solium UNAM-cd1_adult Taenia ... 1271 0.0

Query= TS.seq.screen.trim.Contig499

Length=803

Score E

Sequences producing significant alignments: (Bits) Value

gb|EL757991.1| AD0099003 Taenia solium UNAM-cd1_adult Taenia ... 603 1e-172

gb|EL748377.1| LV0339015 Taenia solium UNAM-cd2_larva Taenia ... 592 2e-169

gb|EL757923.1| AD0098008 Taenia solium UNAM-cd1_adult Taenia ... 590 8e-169

gb|EL740634.1| LV0213016 Taenia solium UNAM-cd2_larva Taenia ... 586 1e-167

gb|EL753583.1| AD0018121 Taenia solium UNAM-cd1_adult Taenia ... 584 4e-167

gb|EL760199.1| AD0137032 Taenia solium UNAM-cd1_adult Taenia ... 582 1e-166

gb|EL759489.1| AD0124003 Taenia solium UNAM-cd1_adult Taenia ... 582 1e-166

gb|EL758829.1| AD0112028 Taenia solium UNAM-cd1_adult Taenia ... 582 1e-166

gb|EL758222.1| AD0102033 Taenia solium UNAM-cd1_adult Taenia ... 582 1e-166

gb|EL748166.1| LV0334049 Taenia solium UNAM-cd2_larva Taenia ... 582 1e-166

Query= TS.seq.screen.trim.Contig500

Length=1269

Score E

Sequences producing significant alignments: (Bits) Value

gb|EL755733.1| AD0027029 Taenia solium UNAM-cd1_adult Taenia ... 1663 0.0

gb|EL756302.1| AD0029097 Taenia solium UNAM-cd1_adult Taenia ... 1622 0.0

gb|EL752619.1| AD0014160 Taenia solium UNAM-cd1_adult Taenia ... 1543 0.0

gb|EL752122.1| AD0012150 Taenia solium UNAM-cd1_adult Taenia ... 1537 0.0

gb|EL756333.1| AD0029128 Taenia solium UNAM-cd1_adult Taenia ... 1535 0.0

gb|EL756846.1| AD0031119 Taenia solium UNAM-cd1_adult Taenia ... 1509 0.0

gb|EL752066.1| AD0012094 Taenia solium UNAM-cd1_adult Taenia ... 1506 0.0

gb|EL756496.1| AD0030028 Taenia solium UNAM-cd1_adult Taenia ... 1469 0.0

gb|EL755343.1| AD0025113 Taenia solium UNAM-cd1_adult Taenia ... 1459 0.0

gb|EL753164.1| AD0016202 Taenia solium UNAM-cd1_adult Taenia ... 1454 0.0

Query= TS.seq.screen.trim.Contig501

Length=1886

Score E

Sequences producing significant alignments: (Bits) Value

gb|EL753227.1| AD0016265 Taenia solium UNAM-cd1_adult Taenia ... 1572 0.0

gb|EL753046.1| AD0016084 Taenia solium UNAM-cd1_adult Taenia ... 1507 0.0

gb|EL749526.1| AD0001149 Taenia solium UNAM-cd1_adult Taenia ... 1489 0.0

gb|EL752510.1| AD0014051 Taenia solium UNAM-cd1_adult Taenia ... 1434 0.0

gb|EL757290.1| AD0034106 Taenia solium UNAM-cd1_adult Taenia ... 1424 0.0

gb|EL756469.1| AD0030001 Taenia solium UNAM-cd1_adult Taenia ... 1408 0.0

gb|EL751510.1| AD0010003 Taenia solium UNAM-cd1_adult Taenia ... 1408 0.0

gb|EL753451.1| AD0017209 Taenia solium UNAM-cd1_adult Taenia ... 1406 0.0

gb|EL750993.1| AD0008004 Taenia solium UNAM-cd1_adult Taenia ... 1387 0.0

gb|EL754776.1| AD0023032 Taenia solium UNAM-cd1_adult Taenia ... 1378 0.0

Query= TS.seq.screen.trim.Contig502

Length=1073

Score E

Sequences producing significant alignments: (Bits) Value

gb|GT227145.1| tscaa0_002549.z1.scf Taenia solium adult full-... 1716 0.0

gb|EL755806.1| AD0027102 Taenia solium UNAM-cd1_adult Taenia ... 1535 0.0

gb|EL751999.1| AD0012027 Taenia solium UNAM-cd1_adult Taenia ... 1515 0.0

gb|EL752036.1| AD0012064 Taenia solium UNAM-cd1_adult Taenia ... 1474 0.0

gb|EL752089.1| AD0012117 Taenia solium UNAM-cd1_adult Taenia ... 1454 0.0

gb|EL753583.1| AD0018121 Taenia solium UNAM-cd1_adult Taenia ... 1450 0.0

gb|EL753055.1| AD0016093 Taenia solium UNAM-cd1_adult Taenia ... 1443 0.0

gb|EL756173.1| AD0028231 Taenia solium UNAM-cd1_adult Taenia ... 1421 0.0

gb|EL753731.1| AD0019018 Taenia solium UNAM-cd1_adult Taenia ... 1400 0.0

gb|EL755652.1| AD0026175 Taenia solium UNAM-cd1_adult Taenia ... 1387 0.0

Query= TS.seq.screen.trim.Contig503

Length=1413

Score E

Sequences producing significant alignments: (Bits) Value

gb|EL760918.1| AD0152043 Taenia solium UNAM-cd1_adult Taenia ... 798 0.0

gb|EL747470.1| LV0324006 Taenia solium UNAM-cd2_larva Taenia ... 789 0.0

gb|EL747710.1| LV0327053 Taenia solium UNAM-cd2_larva Taenia ... 150 2e-36

gb|EL741854.1| LV0232059 Taenia solium UNAM-cd2_larva Taenia ... 56.5 3e-08

Query= TSBW.R74.esd 577 0 577 ESD GOOD: 98-287

Length=190

Score E

Sequences producing significant alignments: (Bits) Value

gb|EL747053.1| LV0317050 Taenia solium UNAM-cd2_larva Taenia ... 276 2e-74

gb|EL742728.1| LV0246011 Taenia solium UNAM-cd2_larva Taenia ... 204 1e-52

Query= TSAO.R59.esd 681 0 681 ESD GOOD: 97-677

Length=581

Score E

Sequences producing significant alignments: (Bits) Value

gb|GT227601.1| tscaa0_001733.z1.scf Taenia solium adult full-... 180 2e-45

Query= TSAC.R39.esd 560 0 560 ESD GOOD: 93-501

Length=409

Score E

Sequences producing significant alignments: (Bits) Value

gb|EL761658.1| AD0168018 Taenia solium UNAM-cd1_adult Taenia ... 510 6e-145

gb|EL744115.1| LV0267054 Taenia solium UNAM-cd2_larva Taenia ... 387 1e-107

Query= TSBO.R4.esd 700 0 700 ESD GOOD: 112-350

Length=239

Score E

Sequences producing significant alignments: (Bits) Value

gb|EL748035.1| LV0332057 Taenia solium UNAM-cd2_larva Taenia ... 294 7e-80

gb|EL747761.1| LV0328049 Taenia solium UNAM-cd2_larva Taenia ... 291 9e-79

gb|EL743128.1| LV0252029 Taenia solium UNAM-cd2_larva Taenia ... 171 1e-42

Query= TSCA.R62.esd 700 0 700 ESD GOOD: 134-425

Length=292

Score E

Sequences producing significant alignments: (Bits) Value

gb|EL756015.1| AD0028073 Taenia solium UNAM-cd1_adult Taenia ... 435 4e-122

gb|EL752031.1| AD0012059 Taenia solium UNAM-cd1_adult Taenia ... 435 4e-122

gb|EL752286.1| AD0013053 Taenia solium UNAM-cd1_adult Taenia ... 401 4e-112

gb|EL758838.1| AD0112037 Taenia solium UNAM-cd1_adult Taenia ... 350 1e-96

Query= TSAH.R59.esd 648 0 648 ESD GOOD: 100-621

Length=522

Score E

Sequences producing significant alignments: (Bits) Value

gb|EL745335.1| LV0289047 Taenia solium UNAM-cd2_larva Taenia ... 226 3e-59

gb|EL748502.1| LV0341018 Taenia solium UNAM-cd2_larva Taenia ... 139 3e-33

Query= TSBY.R10.esd 539 0 539 ESD GOOD: 96-539

Length=444

Score E

Sequences producing significant alignments: (Bits) Value

gb|EL759657.1| AD0126054 Taenia solium UNAM-cd1_adult Taenia ... 747 0.0

gb|EL753240.1| AD0016278 Taenia solium UNAM-cd1_adult Taenia ... 747 0.0

gb|EL753180.1| AD0016218 Taenia solium UNAM-cd1_adult Taenia ... 747 0.0

gb|EL746071.1| LV0301026 Taenia solium UNAM-cd2_larva Taenia ... 747 0.0

gb|EL741580.1| LV0228062 Taenia solium UNAM-cd2_larva Taenia ... 741 0.0

gb|EL743631.1| LV0260019 Taenia solium UNAM-cd2_larva Taenia ... 723 0.0

gb|EL742787.1| LV0247005 Taenia solium UNAM-cd2_larva Taenia ... 691 0.0

gb|EL743922.1| LV0264051 Taenia solium UNAM-cd2_larva Taenia ... 656 0.0

gb|EL745614.1| LV0294029 Taenia solium UNAM-cd2_larva Taenia ... 91.6 1e-18

Query= TSAO.R29.esd 678 0 678 ESD GOOD: 114-554

Length=441

Score E

Sequences producing significant alignments: (Bits) Value

gb|EX150561.1| TSEDTS1008G10 Cysti Taenia solium cDNA, mRNA s... 220 1e-57

gb|EL747128.1| LV0318060 Taenia solium UNAM-cd2_larva Taenia ... 206 3e-53

Query= TSAL.R77.esd 562 0 562 ESD GOOD: 105-456

Length=352

Score E

Sequences producing significant alignments: (Bits) Value

gb|EL748444.1| LV0340018 Taenia solium UNAM-cd2_larva Taenia ... 529 2e-150

Query= TSAR.R40.esd 584 0 584 ESD GOOD: 92-461

Length=370

Score E

Sequences producing significant alignments: (Bits) Value

gb|EL741012.1| LV0219062 Taenia solium UNAM-cd2_larva Taenia ... 584 4e-167

gb|EL761100.1| AD0156040 Taenia solium UNAM-cd1_adult Taenia ... 318 4e-87

gb|EL750012.1| AD0003154 Taenia solium UNAM-cd1_adult Taenia ... 191 9e-49

gb|EL749788.1| AD0002152 Taenia solium UNAM-cd1_adult Taenia ... 167 2e-41

gb|EL741336.1| LV0224056 Taenia solium UNAM-cd2_larva Taenia ... 117 2e-26

Query= TSAC.R27.esd 648 0 648 ESD GOOD: 110-436

Length=327

Score E

Sequences producing significant alignments: (Bits) Value

gb|EL742108.1| LV0236037 Taenia solium UNAM-cd2_larva Taenia ... 475 2e-134

Query= TSBH.R45.esd 566 0 566 ESD GOOD: 101-539

Length=439

Score E

Sequences producing significant alignments: (Bits) Value

gb|EL740596.1| LV0212032 Taenia solium UNAM-cd2_larva Taenia ... 363 2e-100

gb|EL743753.1| LV0261062 Taenia solium UNAM-cd2_larva Taenia ... 257 9e-69

Query= TSAZ.R36.esd 745 0 745 ESD GOOD: 113-431

Length=319

Score E

Sequences producing significant alignments: (Bits) Value

gb|EL742133.1| LV0237021 Taenia solium UNAM-cd2_larva Taenia ... 388 3e-108

gb|EL761104.1| AD0156044 Taenia solium UNAM-cd1_adult Taenia ... 309 2e-84

gb|EL742390.1| LV0241016 Taenia solium UNAM-cd2_larva Taenia ... 239 3e-63

gb|EL755908.1| AD0027204 Taenia solium UNAM-cd1_adult Taenia ... 196 2e-50

gb|EL749236.1| LV0356014 Taenia solium UNAM-cd2_larva Taenia ... 187 1e-47

Query= TSAW.R17.esd 733 0 733 ESD GOOD: 96-426

Length=331

Score E

Sequences producing significant alignments: (Bits) Value

gb|EL742847.1| LV0248009 Taenia solium UNAM-cd2_larva Taenia ... 490 8e-139

gb|EL746601.1| LV0311030 Taenia solium UNAM-cd2_larva Taenia ... 326 2e-89

Query= TSAL.R27.esd 655 0 655 ESD GOOD: 104-387

Length=284

Score E

Sequences producing significant alignments: (Bits) Value

gb|GT227036.1| tscaa0_001171.z1.scf Taenia solium adult full-... 481 5e-136

Query= TSBU.R26.esd 842 0 842 ESD GOOD: 103-618

Length=516

Score E

Sequences producing significant alignments: (Bits) Value

gb|EL752957.1| AD0015216 Taenia solium UNAM-cd1_adult Taenia ... 939 0.0

gb|EL752736.1| AD0014277 Taenia solium UNAM-cd1_adult Taenia ... 939 0.0

gb|EL763443.1| AD0204006 Taenia solium UNAM-cd1_adult Taenia ... 935 0.0

Query= TSBA.R2.esd 621 0 621 ESD GOOD: 96-571

Length=476

Score E

Sequences producing significant alignments: (Bits) Value

gb|EL741448.1| LV0227004 Taenia solium UNAM-cd2_larva Taenia ... 387 1e-107

gb|EL742144.1| LV0237032 Taenia solium UNAM-cd2_larva Taenia ... 292 2e-79

Query= TSBJ.R39.esd 211 0 211 ESD GOOD: 91-211

Length=121

Score E

Sequences producing significant alignments: (Bits) Value

gb|EL745115.1| LV0286032 Taenia solium UNAM-cd2_larva Taenia ... 202 4e-52

Query= TSAB.R23.esd 682 0 682 ESD GOOD: 101-590

Length=490

Score E

Sequences producing significant alignments: (Bits) Value

gb|EL746554.1| LV0310051 Taenia solium UNAM-cd2_larva Taenia ... 893 0.0

Query= TSAA.R4.esd 699 0 699 ESD GOOD: 101-557

Length=457

Score E

Sequences producing significant alignments: (Bits) Value

gb|EL744646.1| LV0277023 Taenia solium UNAM-cd2_larva Taenia ... 289 3e-78

gb|EL744645.1| LV0277022 Taenia solium UNAM-cd2_larva Taenia ... 180 2e-45

Query= TSBN.R76.esd 718 0 718 ESD GOOD: 102-201

Length=100

Score E

Sequences producing significant alignments: (Bits) Value

gb|EL743991.1| LV0265063 Taenia solium UNAM-cd2_larva Taenia ... 165 6e-41

gb|EL741958.1| LV0234019 Taenia solium UNAM-cd2_larva Taenia ... 95.3 7e-20

Query= TSCC.R39.esd 422 0 422 ESD GOOD: 99-422

Length=324

Score E

Sequences producing significant alignments: (Bits) Value

gb|EL743539.1| LV0259002 Taenia solium UNAM-cd2_larva Taenia ... 523 8e-149

Query= TSAJ.R73.esd 558 0 558 ESD GOOD: 109-492

Length=384

Score E

Sequences producing significant alignments: (Bits) Value

gb|EL760304.1| AD0139040 Taenia solium UNAM-cd1_adult Taenia ... 544 6e-155

gb|EL756057.1| AD0028115 Taenia solium UNAM-cd1_adult Taenia ... 172 3e-43

Query= TSBC.R73.esd 460 0 460 ESD GOOD: 105-427

Length=323

Score E

Sequences producing significant alignments: (Bits) Value

gb|EL742711.1| LV0245066 Taenia solium UNAM-cd2_larva Taenia ... 440 8e-124

Query= TSAI.R12.esd 637 0 637 ESD GOOD: 98-603

Length=506

Score E

Sequences producing significant alignments: (Bits) Value

gb|EL749302.1| LV0358013 Taenia solium UNAM-cd2_larva Taenia ... 58.4 1e-08

Query= TSBA.R62.esd 652 0 652 ESD GOOD: 101-552

Length=452

Score E

Sequences producing significant alignments: (Bits) Value

gb|EL759161.1| AD0117064 Taenia solium UNAM-cd1_adult Taenia ... 760 0.0

gb|EL761280.1| AD0160031 Taenia solium UNAM-cd1_adult Taenia ... 732 0.0

gb|EL762016.1| AD0176022 Taenia solium UNAM-cd1_adult Taenia ... 643 0.0

gb|EL757881.1| AD0097024 Taenia solium UNAM-cd1_adult Taenia ... 339 3e-93

gb|EL756850.1| AD0031123 Taenia solium UNAM-cd1_adult Taenia ... 172 3e-43

Query= TSAG.R28.esd 596 0 596 ESD GOOD: 98-559

Length=462

Score E

Sequences producing significant alignments: (Bits) Value

gb|EL740510.1| LV0210034 Taenia solium UNAM-cd2_larva Taenia ... 496 2e-140

Query= TSAV.R52.esd 641 0 641 ESD GOOD: 106-279

Length=174

Score E

Sequences producing significant alignments: (Bits) Value

gb|EL748206.1| LV0335021 Taenia solium UNAM-cd2_larva Taenia ... 309 2e-84

gb|EL743402.1| LV0257019 Taenia solium UNAM-cd2_larva Taenia ... 259 2e-69

Query= TSAC.R80.esd 697 0 697 ESD GOOD: 106-567

Length=462

Score E

Sequences producing significant alignments: (Bits) Value

gb|GT227163.1| tscaa0_000148.z1.scf Taenia solium adult full-... 403 1e-112

Query= TSBG.R5.esd 442 0 442 ESD GOOD: 89-442

Length=354

Score E

Sequences producing significant alignments: (Bits) Value

gb|EL753260.1| AD0017018 Taenia solium UNAM-cd1_adult Taenia ... 455 3e-128

gb|EL750554.1| AD0005217 Taenia solium UNAM-cd1_adult Taenia ... 455 3e-128

gb|EL757639.1| AD0035232 Taenia solium UNAM-cd1_adult Taenia ... 451 4e-127

gb|EL756784.1| AD0031057 Taenia solium UNAM-cd1_adult Taenia ... 451 4e-127

gb|EL756561.1| AD0030093 Taenia solium UNAM-cd1_adult Taenia ... 451 4e-127

gb|EL755400.1| AD0025170 Taenia solium UNAM-cd1_adult Taenia ... 451 4e-127

gb|EL750328.1| AD0004243 Taenia solium UNAM-cd1_adult Taenia ... 451 4e-127

gb|EL760566.1| AD0144053 Taenia solium UNAM-cd1_adult Taenia ... 449 1e-126

gb|EL760092.1| AD0135038 Taenia solium UNAM-cd1_adult Taenia ... 449 1e-126

gb|EL756007.1| AD0028065 Taenia solium UNAM-cd1_adult Taenia ... 449 1e-126

Query= TSAE.R60.esd 607 0 607 ESD GOOD: 94-605

Length=512

Score E

Sequences producing significant alignments: (Bits) Value

gb|EL749305.1| LV0358016 Taenia solium UNAM-cd2_larva Taenia ... 303 1e-82

Query= TSAJ.R39.esd 545 0 545 ESD GOOD: 97-540

Length=444

Score E

Sequences producing significant alignments: (Bits) Value

gb|EL745767.1| LV0296057 Taenia solium UNAM-cd2_larva Taenia ... 706 0.0

gb|GT226999.1| tscaa0_002406.z1.scf Taenia solium adult full-... 701 0.0

gb|EL746284.1| LV0306035 Taenia solium UNAM-cd2_larva Taenia ... 484 4e-137

gb|EL758823.1| AD0112022 Taenia solium UNAM-cd1_adult Taenia ... 62.1 7e-10

Query= TSBP.R33.esd 627 0 627 ESD GOOD: 90-557

Length=468

Score E

Sequences producing significant alignments: (Bits) Value

gb|EL742430.1| LV0241056 Taenia solium UNAM-cd2_larva Taenia ... 416 1e-116

Query= TSBQ.R32.esd 548 0 548 ESD GOOD: 89-506

Length=418

Score E

Sequences producing significant alignments: (Bits) Value

gb|EL746121.1| LV0302016 Taenia solium UNAM-cd2_larva Taenia ... 420 1e-117

Query= TSAT.R35.esd 826 0 826 ESD GOOD: 108-456

Length=349

Score E

Sequences producing significant alignments: (Bits) Value

gb|EL748755.1| LV0345007 Taenia solium UNAM-cd2_larva Taenia ... 195 7e-50

Query= TSBH.R67.esd 530 0 530 ESD GOOD: 98-452

Length=355

Score E

Sequences producing significant alignments: (Bits) Value

gb|GT227863.1| tscaa0_002604.z1.scf Taenia solium adult full-... 514 5e-146

gb|EL747297.1| LV0321028 Taenia solium UNAM-cd2_larva Taenia ... 298 5e-81

Query= TSBC.R26.esd 534 0 534 ESD GOOD: 145-530

Length=386

Score E

Sequences producing significant alignments: (Bits) Value

gb|GT227235.1| tscaa0_000538.z1.scf Taenia solium adult full-... 547 5e-156

Query= TSAY.R47.esd 563 0 563 ESD GOOD: 84-521

Length=438

Score E

Sequences producing significant alignments: (Bits) Value

gb|EL743360.1| LV0256021 Taenia solium UNAM-cd2_larva Taenia ... 544 6e-155

Query= TSBH.R84.esd 591 0 591 ESD GOOD: 82-589

Length=508

Score E

Sequences producing significant alignments: (Bits) Value

gb|GT227509.1| tscaa0_001502.z1.scf Taenia solium adult full-... 180 2e-45

Query= TSBO.R74.esd 833 0 833 ESD GOOD: 95-480

Length=386

Score E

Sequences producing significant alignments: (Bits) Value

gb|EL762925.1| AD0195045 Taenia solium UNAM-cd1_adult Taenia ... 394 6e-110

gb|EL762585.1| AD0189042 Taenia solium UNAM-cd1_adult Taenia ... 388 3e-108

Query= TSBE.R66.esd 656 0 656 ESD GOOD: 104-502

Length=399

Score E

Sequences producing significant alignments: (Bits) Value

gb|EL745291.1| LV0289003 Taenia solium UNAM-cd2_larva Taenia ... 604 3e-173

gb|GT227039.1| tscaa0_001952.z1.scf Taenia solium adult full-... 545 2e-155

Query= TSCB.R33.esd 680 0 680 ESD GOOD: 97-589

Length=493

Score E

Sequences producing significant alignments: (Bits) Value

gb|EL742874.1| LV0248036 Taenia solium UNAM-cd2_larva Taenia ... 793 0.0

Query= TSAF.R64.esd 465 0 465 ESD GOOD: 95-371

Length=277

Score E

Sequences producing significant alignments: (Bits) Value

gb|EL751636.1| AD0010129 Taenia solium UNAM-cd1_adult Taenia ... 316 1e-86

gb|EL758237.1| AD0102048 Taenia solium UNAM-cd1_adult Taenia ... 311 7e-85

gb|EL758311.1| AD0103055 Taenia solium UNAM-cd1_adult Taenia ... 163 2e-40

Query= TSBS.R36.esd 732 0 732 ESD GOOD: 92-649

Length=558

Score E

Sequences producing significant alignments: (Bits) Value

gb|EL749274.1| LV0357018 Taenia solium UNAM-cd2_larva Taenia ... 928 0.0

gb|EL758876.1| AD0113017 Taenia solium UNAM-cd1_adult Taenia ... 233 1e-61

gb|EL757360.1| AD0034176 Taenia solium UNAM-cd1_adult Taenia ... 231 5e-61

Query= TSAU.R77.esd 695 0 695 ESD GOOD: 106-244

Length=139

Score E

Sequences producing significant alignments: (Bits) Value

gb|EL761385.1| AD0162046 Taenia solium UNAM-cd1_adult Taenia ... 193 3e-49

Query= TSBK.R32.esd 656 0 656 ESD GOOD: 122-622

Length=501

Score E

Sequences producing significant alignments: (Bits) Value

gb|EL746930.1| LV0316007 Taenia solium UNAM-cd2_larva Taenia ... 62.1 7e-10

Query= TSBV.R95.esd 534 0 534 ESD GOOD: 110-516

Length=407

Score E

Sequences producing significant alignments: (Bits) Value

gb|EL749306.1| LV0358017 Taenia solium UNAM-cd2_larva Taenia ... 329 2e-90

gb|EL757693.1| AD0094033 Taenia solium UNAM-cd1_adult Taenia ... 259 2e-69

gb|EL747451.1| LV0323060 Taenia solium UNAM-cd2_larva Taenia ... 126 3e-29

gb|EL743941.1| LV0265013 Taenia solium UNAM-cd2_larva Taenia ... 78.7 7e-15

Query= TSBN.R10.esd 467 0 467 ESD GOOD: 94-466

Length=373

Score E

Sequences producing significant alignments: (Bits) Value

gb|EL761443.1| AD0163052 Taenia solium UNAM-cd1_adult Taenia ... 604 3e-173

gb|EL761535.1| AD0165033 Taenia solium UNAM-cd1_adult Taenia ... 418 4e-117

gb|EL748284.1| LV0337005 Taenia solium UNAM-cd2_larva Taenia ... 241 9e-64

Query= TSAI.R33.esd 594 0 594 ESD GOOD: 100-471

Length=372

Score E

Sequences producing significant alignments: (Bits) Value

gb|GT227263.1| tscaa0_000615.z1.scf Taenia solium adult full-... 486 1e-137

gb|FD661327.1| TSEDTS1005G04 Cysti Taenia solium cDNA, mRNA s... 438 3e-123

gb|FD661322.1| TSEDTS1003G04 Cysti Taenia solium cDNA, mRNA s... 438 3e-123

gb|EX150361.1| TSEDTS1000E05 Cysti Taenia solium cDNA, mRNA s... 431 5e-121

gb|FD661448.1| TSEDTS1016G07 Cysti Taenia solium cDNA, mRNA s... 335 4e-92

gb|FD661318.1| TSEDTS1000D05 Cysti Taenia solium cDNA, mRNA s... 302 4e-82

gb|EL748526.1| LV0341042 Taenia solium UNAM-cd2_larva Taenia ... 268 4e-72

gb|EX150544.1| TSEDTS1003H09 Cysti Taenia solium cDNA, mRNA s... 248 5e-66

gb|EX150362.1| TSEDTS1000G10 Cysti Taenia solium cDNA, mRNA s... 248 5e-66

gb|EX150535.1| TSEDTS1003G09 Cysti Taenia solium cDNA, mRNA s... 235 4e-62

Query= TSAG.R38.esd 587 0 587 ESD GOOD: 92-583

Length=492

Score E

Sequences producing significant alignments: (Bits) Value

gb|EL748306.1| LV0337027 Taenia solium UNAM-cd2_larva Taenia ... 891 0.0

gb|EL743458.1| LV0257075 Taenia solium UNAM-cd2_larva Taenia ... 278 7e-75

Query= TSBI.R10.esd 718 0 718 ESD GOOD: 146-456

Length=311

Score E

Sequences producing significant alignments: (Bits) Value

gb|GT227208.1| tscaa0_000461.z1.scf Taenia solium adult full-... 287 1e-77

Query= TSAB.R2.esd 597 0 597 ESD GOOD: 97-597

Length=501

Score E

Sequences producing significant alignments: (Bits) Value

gb|EL748510.1| LV0341026 Taenia solium UNAM-cd2_larva Taenia ... 872 0.0

Query= TSBR.R88.esd 747 0 747 ESD GOOD: 96-503

Length=408

Score E

Sequences producing significant alignments: (Bits) Value

gb|EL741669.1| LV0230016 Taenia solium UNAM-cd2_larva Taenia ... 610 6e-175

gb|EL747508.1| LV0324044 Taenia solium UNAM-cd2_larva Taenia ... 303 1e-82

Query= TSAZ.R73.esd 671 0 671 ESD GOOD: 125-618

Length=494

Score E

Sequences producing significant alignments: (Bits) Value

gb|EL742900.1| LV0249015 Taenia solium UNAM-cd2_larva Taenia ... 327 7e-90

Query= TSCG.R69.esd 627 0 627 ESD GOOD: 94-468

Length=375

Score E

Sequences producing significant alignments: (Bits) Value

gb|EL747355.1| LV0322032 Taenia solium UNAM-cd2_larva Taenia ... 481 5e-136

gb|EL746166.1| LV0303017 Taenia solium UNAM-cd2_larva Taenia ... 481 5e-136

Query= TSCC.R93.esd 647 0 647 ESD GOOD: 102-641

Length=540

Score E

Sequences producing significant alignments: (Bits) Value

gb|EL757983.1| AD0098068 Taenia solium UNAM-cd1_adult Taenia ... 979 0.0

gb|EL752576.1| AD0014117 Taenia solium UNAM-cd1_adult Taenia ... 977 0.0

gb|EL762902.1| AD0195022 Taenia solium UNAM-cd1_adult Taenia ... 961 0.0

gb|EL755609.1| AD0026132 Taenia solium UNAM-cd1_adult Taenia ... 915 0.0

gb|EL753774.1| AD0019061 Taenia solium UNAM-cd1_adult Taenia ... 905 0.0

gb|EL753223.1| AD0016261 Taenia solium UNAM-cd1_adult Taenia ... 905 0.0

gb|EL757390.1| AD0034206 Taenia solium UNAM-cd1_adult Taenia ... 686 0.0

gb|GT227681.1| tscaa0_001951.z1.scf Taenia solium adult full-... 592 2e-169

gb|EL758519.1| AD0107012 Taenia solium UNAM-cd1_adult Taenia ... 575 2e-164

gb|EL742470.1| LV0242034 Taenia solium UNAM-cd2_larva Taenia ... 427 6e-120

Query= TSBC.R71.esd 685 0 685 ESD GOOD: 104-653

Length=550

Score E

Sequences producing significant alignments: (Bits) Value

gb|EL761658.1| AD0168018 Taenia solium UNAM-cd1_adult Taenia ... 686 0.0

Query= TSAY.R35.esd 738 0 738 ESD GOOD: 99-573

Length=475

Score E

Sequences producing significant alignments: (Bits) Value

gb|EL763235.1| AD0200050 Taenia solium UNAM-cd1_adult Taenia ... 863 0.0

gb|EL762777.1| AD0193024 Taenia solium UNAM-cd1_adult Taenia ... 863 0.0

gb|EL752192.1| AD0012220 Taenia solium UNAM-cd1_adult Taenia ... 863 0.0

gb|EL746034.1| LV0300065 Taenia solium UNAM-cd2_larva Taenia ... 863 0.0

gb|EL745133.1| LV0286050 Taenia solium UNAM-cd2_larva Taenia ... 863 0.0

gb|EL743540.1| LV0259003 Taenia solium UNAM-cd2_larva Taenia ... 863 0.0

gb|EL741540.1| LV0228022 Taenia solium UNAM-cd2_larva Taenia ... 863 0.0

gb|EL744122.1| LV0267061 Taenia solium UNAM-cd2_larva Taenia ... 857 0.0

gb|EL744027.1| LV0266027 Taenia solium UNAM-cd2_larva Taenia ... 821 0.0

gb|EL756022.1| AD0028080 Taenia solium UNAM-cd1_adult Taenia ... 813 0.0

Query= TSBT.R54.esd 785 0 785 ESD GOOD: 93-346

Length=254

Score E

Sequences producing significant alignments: (Bits) Value

gb|EL741153.1| LV0221054 Taenia solium UNAM-cd2_larva Taenia ... 322 3e-88

gb|EL746419.1| LV0308069 Taenia solium UNAM-cd2_larva Taenia ... 298 5e-81

gb|EL747587.1| LV0326003 Taenia solium UNAM-cd2_larva Taenia ... 267 1e-71

Query= TSBY.R13.esd 730 0 730 ESD GOOD: 97-531

Length=435

Score E

Sequences producing significant alignments: (Bits) Value

gb|EL742355.1| LV0240050 Taenia solium UNAM-cd2_larva Taenia ... 699 0.0

gb|EL743164.1| LV0253011 Taenia solium UNAM-cd2_larva Taenia ... 442 2e-124

Query= TSBM.R20.esd 638 0 638 ESD GOOD: 96-569

Length=474

Score E

Sequences producing significant alignments: (Bits) Value

gb|EL754239.1| AD0021029 Taenia solium UNAM-cd1_adult Taenia ... 852 0.0

gb|EL757117.1| AD0032129 Taenia solium UNAM-cd1_adult Taenia ... 846 0.0

gb|EL757242.1| AD0034058 Taenia solium UNAM-cd1_adult Taenia ... 673 0.0

gb|EL761470.1| AD0164026 Taenia solium UNAM-cd1_adult Taenia ... 592 2e-169

gb|EL746989.1| LV0316066 Taenia solium UNAM-cd2_larva Taenia ... 398 5e-111

Query= TSAR.R49.esd 667 0 667 ESD GOOD: 104-624

Length=521

Score E

Sequences producing significant alignments: (Bits) Value

gb|EL746972.1| LV0316049 Taenia solium UNAM-cd2_larva Taenia ... 619 1e-177

gb|EL740776.1| LV0216010 Taenia solium UNAM-cd2_larva Taenia ... 486 1e-137

gb|EL743010.1| LV0250051 Taenia solium UNAM-cd2_larva Taenia ... 353 1e-97

Query= TSAA.R85.esd 686 0 686 ESD GOOD: 129-594

Length=466

Score E

Sequences producing significant alignments: (Bits) Value

gb|EL744957.1| LV0283022 Taenia solium UNAM-cd2_larva Taenia ... 412 2e-115

gb|EL744353.1| LV0271035 Taenia solium UNAM-cd2_larva Taenia ... 412 2e-115

gb|EX150570.1| TSEDTS1008F09 Cysti Taenia solium cDNA, mRNA s... 324 9e-89

Query= TSAM.R59.esd 706 0 706 ESD GOOD: 103-546

Length=444

Score E

Sequences producing significant alignments: (Bits) Value

gb|EL744165.1| LV0268032 Taenia solium UNAM-cd2_larva Taenia ... 76.8 3e-14

Query= TSCG.R24.esd 664 0 664 ESD GOOD: 106-629

Length=524

Score E

Sequences producing significant alignments: (Bits) Value

gb|EL746030.1| LV0300061 Taenia solium UNAM-cd2_larva Taenia ... 141 9e-34

Query= TSBA.R52.esd 494 0 494 ESD GOOD: 96-336

Length=241

Score E

Sequences producing significant alignments: (Bits) Value

gb|EL759835.1| AD0130025 Taenia solium UNAM-cd1_adult Taenia ... 403 1e-112

Query= TSAI.R42.esd 710 0 710 ESD GOOD: 102-355

Length=254

Score E

Sequences producing significant alignments: (Bits) Value

gb|EL742575.1| LV0244006 Taenia solium UNAM-cd2_larva Taenia ... 294 7e-80

Query= TSBW.R68.esd 331 0 331 ESD GOOD: 95-331

Length=237

Score E

Sequences producing significant alignments: (Bits) Value

gb|EL743149.1| LV0252050 Taenia solium UNAM-cd2_larva Taenia ... 429 2e-120

gb|EL755233.1| AD0025003 Taenia solium UNAM-cd1_adult Taenia ... 422 3e-118

gb|EL747985.1| LV0332007 Taenia solium UNAM-cd2_larva Taenia ... 422 3e-118

gb|EL743510.1| LV0258050 Taenia solium UNAM-cd2_larva Taenia ... 422 3e-118

gb|EL741096.1| LV0220069 Taenia solium UNAM-cd2_larva Taenia ... 422 3e-118

gb|EL743089.1| LV0251054 Taenia solium UNAM-cd2_larva Taenia ... 411 6e-115

gb|EL758212.1| AD0102023 Taenia solium UNAM-cd1_adult Taenia ... 379 2e-105

gb|EL747681.1| LV0327024 Taenia solium UNAM-cd2_larva Taenia ... 355 3e-98

gb|EL743651.1| LV0260039 Taenia solium UNAM-cd2_larva Taenia ... 355 3e-98

gb|EL747783.1| LV0329021 Taenia solium UNAM-cd2_larva Taenia ... 353 1e-97

Query= TSCB.R94.esd 563 0 563 ESD GOOD: 98-563

Length=466

Score E

Sequences producing significant alignments: (Bits) Value

gb|EL750284.1| AD0004199 Taenia solium UNAM-cd1_adult Taenia ... 754 0.0

gb|EL750515.1| AD0005178 Taenia solium UNAM-cd1_adult Taenia ... 750 0.0

Query= TSBW.R6.esd 674 0 674 ESD GOOD: 100-246

Length=147

Score E

Sequences producing significant alignments: (Bits) Value

gb|EL749243.1| LV0356021 Taenia solium UNAM-cd2_larva Taenia ... 231 5e-61

gb|GT227096.1| tscaa0_002809.z1.scf Taenia solium adult full-... 226 3e-59

gb|EL745126.1| LV0286043 Taenia solium UNAM-cd2_larva Taenia ... 226 3e-59

Query= TSBU.R46.esd 860 0 860 ESD GOOD: 98-648

Length=551

Score E

Sequences producing significant alignments: (Bits) Value

gb|EL758662.1| AD0109037 Taenia solium UNAM-cd1_adult Taenia ... 976 0.0

gb|EL754289.1| AD0021079 Taenia solium UNAM-cd1_adult Taenia ... 974 0.0

gb|EL751012.1| AD0008023 Taenia solium UNAM-cd1_adult Taenia ... 963 0.0

gb|EL752461.1| AD0014002 Taenia solium UNAM-cd1_adult Taenia ... 894 0.0

Query= TSAV.R4.esd 653 0 653 ESD GOOD: 102-368

Length=267

Score E

Sequences producing significant alignments: (Bits) Value

gb|EL750061.1| AD0003203 Taenia solium UNAM-cd1_adult Taenia ... 351 4e-97

gb|EL749837.1| AD0002201 Taenia solium UNAM-cd1_adult Taenia ... 307 9e-84

Query= TSBJ.R42.esd 643 0 643 ESD GOOD: 91-538

Length=448

Score E

Sequences producing significant alignments: (Bits) Value

gb|EL761468.1| AD0164024 Taenia solium UNAM-cd1_adult Taenia ... 699 0.0

gb|EL748199.1| LV0335014 Taenia solium UNAM-cd2_larva Taenia ... 699 0.0

gb|EL747077.1| LV0318009 Taenia solium UNAM-cd2_larva Taenia ... 621 3e-178

gb|EL747620.1| LV0326036 Taenia solium UNAM-cd2_larva Taenia ... 586 1e-167

gb|EL746312.1| LV0307026 Taenia solium UNAM-cd2_larva Taenia ... 580 5e-166

gb|EL740933.1| LV0218044 Taenia solium UNAM-cd2_larva Taenia ... 403 1e-112

gb|EL761128.1| AD0157024 Taenia solium UNAM-cd1_adult Taenia ... 239 3e-63

gb|EL740749.1| LV0215039 Taenia solium UNAM-cd2_larva Taenia ... 198 5e-51

gb|EL740993.1| LV0219043 Taenia solium UNAM-cd2_larva Taenia ... 69.4 4e-12

Query= TSAH.R38.esd 661 0 661 ESD GOOD: 127-561

Length=435

Score E

Sequences producing significant alignments: (Bits) Value

gb|EL740445.1| LV0209023 Taenia solium UNAM-cd2_larva Taenia ... 69.4 4e-12

Query= TSBO.R19.esd 792 0 792 ESD GOOD: 99-685

Length=587

Score E

Sequences producing significant alignments: (Bits) Value

gb|EL761420.1| AD0163029 Taenia solium UNAM-cd1_adult Taenia ... 571 3e-163

gb|EL759347.1| AD0121043 Taenia solium UNAM-cd1_adult Taenia ... 455 3e-128

gb|EL748368.1| LV0339006 Taenia solium UNAM-cd2_larva Taenia ... 300 1e-81

gb|EL754175.1| AD0020220 Taenia solium UNAM-cd1_adult Taenia ... 279 2e-75

gb|EL743750.1| LV0261059 Taenia solium UNAM-cd2_larva Taenia ... 211 7e-55

Query= TSAW.R3.esd 724 0 724 ESD GOOD: 101-350

Length=250

Score E

Sequences producing significant alignments: (Bits) Value

gb|EL742546.1| LV0243044 Taenia solium UNAM-cd2_larva Taenia ... 62.1 7e-10

Query= TSAD.R61.esd 660 0 660 ESD GOOD: 97-569

Length=473

Score E

Sequences producing significant alignments: (Bits) Value

gb|EL745547.1| LV0293025 Taenia solium UNAM-cd2_larva Taenia ... 390 8e-109

Query= TSAW.R85.esd 549 0 549 ESD GOOD: 98-544

Length=447

Score E

Sequences producing significant alignments: (Bits) Value

gb|EL749134.1| LV0353046 Taenia solium UNAM-cd2_larva Taenia ... 641 0.0

Query= TSBM.R39.esd 589 0 589 ESD GOOD: 97-213

Length=117

Score E

Sequences producing significant alignments: (Bits) Value

gb|EL747365.1| LV0322042 Taenia solium UNAM-cd2_larva Taenia ... 202 4e-52

Query= TSBP.R44.esd 647 0 647 ESD GOOD: 94-565

Length=472

Score E

Sequences producing significant alignments: (Bits) Value

gb|EL741216.1| LV0222049 Taenia solium UNAM-cd2_larva Taenia ... 761 0.0

Query= TSBQ.R88.esd 865 0 865 ESD GOOD: 91-465

Length=375

Score E

Sequences producing significant alignments: (Bits) Value

gb|EL744375.1| LV0271057 Taenia solium UNAM-cd2_larva Taenia ... 601 4e-172

gb|EL747777.1| LV0329015 Taenia solium UNAM-cd2_larva Taenia ... 538 3e-153

Query= TSAZ.R70.esd 471 0 471 ESD GOOD: 108-345

Length=238

Score E

Sequences producing significant alignments: (Bits) Value

gb|EL746344.1| LV0307058 Taenia solium UNAM-cd2_larva Taenia ... 375 2e-104

gb|EL763465.1| AD0204028 Taenia solium UNAM-cd1_adult Taenia ... 313 2e-85

gb|EL743163.1| LV0253010 Taenia solium UNAM-cd2_larva Taenia ... 257 9e-69

gb|EL740523.1| LV0210047 Taenia solium UNAM-cd2_larva Taenia ... 257 9e-69

Query= TSAJ.R5.esd 666 0 666 ESD GOOD: 109-590

Length=482

Score E

Sequences producing significant alignments: (Bits) Value

gb|EL745369.1| LV0290024 Taenia solium UNAM-cd2_larva Taenia ... 545 2e-155

gb|EL740679.1| LV0214004 Taenia solium UNAM-cd2_larva Taenia ... 545 2e-155

gb|EL759604.1| AD0126001 Taenia solium UNAM-cd1_adult Taenia ... 206 3e-53

Query= TSAK.R53.esd 382 0 382 ESD GOOD: 92-382

Length=291

Score E

Sequences producing significant alignments: (Bits) Value

gb|EL744296.1| LV0270043 Taenia solium UNAM-cd2_larva Taenia ... 69.4 4e-12

Query= TSAQ.R57.esd 715 0 715 ESD GOOD: 83-558

Length=476

Score E

Sequences producing significant alignments: (Bits) Value

gb|EL762467.1| AD0186031 Taenia solium UNAM-cd1_adult Taenia ... 99.0 6e-21

Query= TSAG.R44.esd 701 0 701 ESD GOOD: 99-580

Length=482

Score E

Sequences producing significant alignments: (Bits) Value

gb|EL744501.1| LV0274004 Taenia solium UNAM-cd2_larva Taenia ... 416 1e-116

gb|EL761992.1| AD0175038 Taenia solium UNAM-cd1_adult Taenia ... 108 9e-24

gb|GT227942.1| tscaa0_002803.z1.scf Taenia solium adult full-... 106 3e-23

Query= TSAP.R6.esd 620 0 620 ESD GOOD: 105-287

Length=183

Score E

Sequences producing significant alignments: (Bits) Value

gb|EL740441.1| LV0209019 Taenia solium UNAM-cd2_larva Taenia ... 128 7e-30

Query= TSAP.R92.esd 626 0 626 ESD GOOD: 93-561

Length=469

Score E

Sequences producing significant alignments: (Bits) Value

gb|EL741700.1| LV0230047 Taenia solium UNAM-cd2_larva Taenia ... 846 0.0

gb|EL741594.1| LV0229005 Taenia solium UNAM-cd2_larva Taenia ... 833 0.0

gb|EL745395.1| LV0290050 Taenia solium UNAM-cd2_larva Taenia ... 830 0.0

gb|EL744453.1| LV0273011 Taenia solium UNAM-cd2_larva Taenia ... 457 8e-129

Query= TSAN.R29.esd 649 0 649 ESD GOOD: 99-494

Length=396

Score E

Sequences producing significant alignments: (Bits) Value

gb|EL758549.1| AD0107042 Taenia solium UNAM-cd1_adult Taenia ... 678 0.0

gb|EL741908.1| LV0233041 Taenia solium UNAM-cd2_larva Taenia ... 678 0.0

gb|EL763053.1| AD0197062 Taenia solium UNAM-cd1_adult Taenia ... 654 0.0

gb|EL743775.1| LV0262016 Taenia solium UNAM-cd2_larva Taenia ... 560 6e-160

Query= TSAE.R43.esd 696 0 696 ESD GOOD: 101-625

Length=525

Score E

Sequences producing significant alignments: (Bits) Value

gb|EL747021.1| LV0317018 Taenia solium UNAM-cd2_larva Taenia ... 900 0.0

gb|EL742651.1| LV0245006 Taenia solium UNAM-cd2_larva Taenia ... 124 9e-29

Query= TSBZ.R95.esd 714 0 714 ESD GOOD: 105-522

Length=418

Score E

Sequences producing significant alignments: (Bits) Value

gb|EL744126.1| LV0267065 Taenia solium UNAM-cd2_larva Taenia ... 704 0.0

gb|EL752303.1| AD0013070 Taenia solium UNAM-cd1_adult Taenia ... 640 0.0

gb|EL752053.1| AD0012081 Taenia solium UNAM-cd1_adult Taenia ... 438 3e-123

gb|EL744875.1| LV0282001 Taenia solium UNAM-cd2_larva Taenia ... 283 1e-76

gb|EL746267.1| LV0306018 Taenia solium UNAM-cd2_larva Taenia ... 169 4e-42

Query= TSCE.R57.esd 685 0 685 ESD GOOD: 99-653

Length=555

Score E

Sequences producing significant alignments: (Bits) Value

gb|GT227349.1| tscaa0_000957.z1.scf Taenia solium adult full-... 752 0.0

Query= TSAV.R54.esd 606 0 606 ESD GOOD: 107-474

Length=368

Score E

Sequences producing significant alignments: (Bits) Value

gb|EL745158.1| LV0287012 Taenia solium UNAM-cd2_larva Taenia ... 363 2e-100

Query= TSAQ.R66.esd 575 0 575 ESD GOOD: 91-546

Length=456

Score E

Sequences producing significant alignments: (Bits) Value

gb|EL744267.1| LV0270014 Taenia solium UNAM-cd2_larva Taenia ... 793 0.0

Query= TSBF.R33.esd 548 0 548 ESD GOOD: 95-499

Length=405

Score E

Sequences producing significant alignments: (Bits) Value

gb|EL758097.1| AD0100042 Taenia solium UNAM-cd1_adult Taenia ... 278 7e-75

gb|EL752079.1| AD0012107 Taenia solium UNAM-cd1_adult Taenia ... 278 7e-75

gb|EL761780.1| AD0170051 Taenia solium UNAM-cd1_adult Taenia ... 276 2e-74

gb|EL752325.1| AD0013092 Taenia solium UNAM-cd1_adult Taenia ... 276 2e-74

gb|EL742891.1| LV0249006 Taenia solium UNAM-cd2_larva Taenia ... 272 3e-73

gb|EL761207.1| AD0159004 Taenia solium UNAM-cd1_adult Taenia ... 259 2e-69

gb|EL761779.1| AD0170050 Taenia solium UNAM-cd1_adult Taenia ... 254 1e-67

gb|EL761206.1| AD0159003 Taenia solium UNAM-cd1_adult Taenia ... 246 2e-65

Query= TSBQ.R66.esd 801 0 801 ESD GOOD: 94-446

Length=353

Score E

Sequences producing significant alignments: (Bits) Value

gb|EL741578.1| LV0228060 Taenia solium UNAM-cd2_larva Taenia ... 398 5e-111

Query= TSBP.R80.esd 675 0 675 ESD GOOD: 84-552

Length=469

Score E

Sequences producing significant alignments: (Bits) Value

gb|EL761932.1| AD0174011 Taenia solium UNAM-cd1_adult Taenia ... 508 2e-144

Query= TSAW.R89.esd 576 0 576 ESD GOOD: 105-298

Length=194

Score E

Sequences producing significant alignments: (Bits) Value

gb|EL753383.1| AD0017141 Taenia solium UNAM-cd1_adult Taenia ... 75.0 1e-13

gb|EL752344.1| AD0013111 Taenia solium UNAM-cd1_adult Taenia ... 75.0 1e-13

gb|EL751485.1| AD0009223 Taenia solium UNAM-cd1_adult Taenia ... 75.0 1e-13

gb|EL751480.1| AD0009218 Taenia solium UNAM-cd1_adult Taenia ... 75.0 1e-13

Query= TSCC.R81.esd 691 0 691 ESD GOOD: 101-626

Length=526

Score E

Sequences producing significant alignments: (Bits) Value

gb|EL744331.1| LV0271013 Taenia solium UNAM-cd2_larva Taenia ... 521 3e-148

gb|EL745018.1| LV0284049 Taenia solium UNAM-cd2_larva Taenia ... 508 2e-144

gb|EL744146.1| LV0268013 Taenia solium UNAM-cd2_larva Taenia ... 507 8e-144

gb|EL744908.1| LV0282034 Taenia solium UNAM-cd2_larva Taenia ... 503 1e-142

gb|EL747759.1| LV0328047 Taenia solium UNAM-cd2_larva Taenia ... 501 4e-142

gb|EL746927.1| LV0316004 Taenia solium UNAM-cd2_larva Taenia ... 501 4e-142

gb|EL746391.1| LV0308041 Taenia solium UNAM-cd2_larva Taenia ... 501 4e-142

gb|EL746377.1| LV0308027 Taenia solium UNAM-cd2_larva Taenia ... 501 4e-142

gb|EL745724.1| LV0296014 Taenia solium UNAM-cd2_larva Taenia ... 501 4e-142

gb|EL745370.1| LV0290025 Taenia solium UNAM-cd2_larva Taenia ... 501 4e-142

Query= TSBV.R74.esd 621 0 621 ESD GOOD: 113-557

Length=445

Score E

Sequences producing significant alignments: (Bits) Value

gb|EL740902.1| LV0218013 Taenia solium UNAM-cd2_larva Taenia ... 492 2e-139

gb|EL744620.1| LV0276038 Taenia solium UNAM-cd2_larva Taenia ... 65.8 6e-11

Query= TSBW.R92.esd 637 0 637 ESD GOOD: 92-343

Length=252

Score E

Sequences producing significant alignments: (Bits) Value

gb|GT227116.1| tscaa0_002476.z1.scf Taenia solium adult full-... 241 9e-64

gb|EL762453.1| AD0186017 Taenia solium UNAM-cd1_adult Taenia ... 185 4e-47

gb|EL758546.1| AD0107039 Taenia solium UNAM-cd1_adult Taenia ... 183 2e-46

Query= TSAS.R89.esd 755 0 755 ESD GOOD: 119-308

Length=190

Score E

Sequences producing significant alignments: (Bits) Value

gb|EL742150.1| LV0237038 Taenia solium UNAM-cd2_larva Taenia ... 324 9e-89

gb|EL745464.1| LV0291056 Taenia solium UNAM-cd2_larva Taenia ... 302 4e-82

Query= TSBN.R31.esd 586 0 586 ESD GOOD: 97-430

Length=334

Score E

Sequences producing significant alignments: (Bits) Value

gb|EL740748.1| LV0215038 Taenia solium UNAM-cd2_larva Taenia ... 307 9e-84

Query= TSAJ.R49.esd 721 0 721 ESD GOOD: 103-719

Length=617

Score E

Sequences producing significant alignments: (Bits) Value

gb|EL742696.1| LV0245051 Taenia solium UNAM-cd2_larva Taenia ... 255 3e-68

Query= TSAI.R43.esd 648 0 648 ESD GOOD: 112-602

Length=491

Score E

Sequences producing significant alignments: (Bits) Value

gb|EL745360.1| LV0290015 Taenia solium UNAM-cd2_larva Taenia ... 675 0.0

Query= TSAB.R74.esd 687 0 687 ESD GOOD: 100-437

Length=338

Score E

Sequences producing significant alignments: (Bits) Value

gb|EL753470.1| AD0018008 Taenia solium UNAM-cd1_adult Taenia ... 414 5e-116

Query= TSBH.R82.esd 621 0 621 ESD GOOD: 92-488

Length=397

Score E

Sequences producing significant alignments: (Bits) Value

gb|EL745833.1| LV0297062 Taenia solium UNAM-cd2_larva Taenia ... 669 0.0

gb|EL745695.1| LV0295053 Taenia solium UNAM-cd2_larva Taenia ... 486 1e-137

Query= TSCH.R20.esd 739 0 739 ESD GOOD: 109-403

Length=295

Score E

Sequences producing significant alignments: (Bits) Value

gb|EL759461.1| AD0123040 Taenia solium UNAM-cd1_adult Taenia ... 176 3e-44

gb|EL756918.1| AD0031191 Taenia solium UNAM-cd1_adult Taenia ... 176 3e-44

gb|EL750439.1| AD0005102 Taenia solium UNAM-cd1_adult Taenia ... 176 3e-44

gb|EL750359.1| AD0005022 Taenia solium UNAM-cd1_adult Taenia ... 176 3e-44

gb|EL750192.1| AD0004107 Taenia solium UNAM-cd1_adult Taenia ... 176 3e-44

gb|EL744933.1| LV0282059 Taenia solium UNAM-cd2_larva Taenia ... 176 3e-44

gb|EL743758.1| LV0261067 Taenia solium UNAM-cd2_larva Taenia ... 176 3e-44

gb|EL750107.1| AD0004022 Taenia solium UNAM-cd1_adult Taenia ... 171 1e-42

gb|EL748039.1| LV0332061 Taenia solium UNAM-cd2_larva Taenia ... 171 1e-42

gb|EL747406.1| LV0323015 Taenia solium UNAM-cd2_larva Taenia ... 161 7e-40

Query= TSCG.R92.esd 675 0 675 ESD GOOD: 103-494

Length=392

Score E

Sequences producing significant alignments: (Bits) Value

gb|EL762215.1| AD0181012 Taenia solium UNAM-cd1_adult Taenia ... 673 0.0

Query= TSAD.R95.esd 757 0 757 ESD GOOD: 127-345

Length=219

Score E

Sequences producing significant alignments: (Bits) Value

gb|EL752361.1| AD0013128 Taenia solium UNAM-cd1_adult Taenia ... 274 9e-74

gb|EL761688.1| AD0169005 Taenia solium UNAM-cd1_adult Taenia ... 263 2e-70

Query= TSAE.R73.esd 608 0 608 ESD GOOD: 91-527

Length=437

Score E

Sequences producing significant alignments: (Bits) Value

gb|EL747576.1| LV0325055 Taenia solium UNAM-cd2_larva Taenia ... 69.4 4e-12

Query= TSBR.R47.esd 742 0 742 ESD GOOD: 112-462

Length=351

Score E

Sequences producing significant alignments: (Bits) Value

gb|EL743575.1| LV0259038 Taenia solium UNAM-cd2_larva Taenia ... 276 2e-74

Query= TSAG.R49.esd 703 0 703 ESD GOOD: 89-526

Length=438

Score E

Sequences producing significant alignments: (Bits) Value

gb|EL744695.1| LV0278014 Taenia solium UNAM-cd2_larva Taenia ... 688 0.0

Query= TSBQ.R92.esd 786 0 786 ESD GOOD: 99-566

Length=468

Score E

Sequences producing significant alignments: (Bits) Value

gb|EL747899.1| LV0330064 Taenia solium UNAM-cd2_larva Taenia ... 739 0.0

gb|EL745644.1| LV0295002 Taenia solium UNAM-cd2_larva Taenia ... 625 2e-179

gb|EL745091.1| LV0286007 Taenia solium UNAM-cd2_larva Taenia ... 549 1e-156

gb|EL745239.1| LV0288021 Taenia solium UNAM-cd2_larva Taenia ... 219 4e-57

gb|EL746984.1| LV0316061 Taenia solium UNAM-cd2_larva Taenia ... 171 1e-42

gb|EL740654.1| LV0213036 Taenia solium UNAM-cd2_larva Taenia ... 106 3e-23

Query= TSBG.R81.esd 785 0 785 ESD GOOD: 102-392

Length=291

Score E

Sequences producing significant alignments: (Bits) Value

gb|EL743621.1| LV0260009 Taenia solium UNAM-cd2_larva Taenia ... 379 2e-105

Query= TSAZ.R91.esd 768 0 768 ESD GOOD: 100-599

Length=500

Score E

Sequences producing significant alignments: (Bits) Value

gb|EL745499.1| LV0292033 Taenia solium UNAM-cd2_larva Taenia ... 684 0.0

gb|EL740930.1| LV0218041 Taenia solium UNAM-cd2_larva Taenia ... 438 3e-123

Query= TSBZ.R14.esd 589 0 589 ESD GOOD: 94-536

Length=443

Score E

Sequences producing significant alignments: (Bits) Value

gb|EL758810.1| AD0112009 Taenia solium UNAM-cd1_adult Taenia ... 448 5e-126

gb|EL756280.1| AD0029075 Taenia solium UNAM-cd1_adult Taenia ... 448 5e-126

gb|EL755458.1| AD0025228 Taenia solium UNAM-cd1_adult Taenia ... 448 5e-126

gb|EL755351.1| AD0025121 Taenia solium UNAM-cd1_adult Taenia ... 448 5e-126

gb|EL754316.1| AD0021106 Taenia solium UNAM-cd1_adult Taenia ... 448 5e-126

gb|EL753822.1| AD0019109 Taenia solium UNAM-cd1_adult Taenia ... 448 5e-126

gb|EL753582.1| AD0018120 Taenia solium UNAM-cd1_adult Taenia ... 448 5e-126

gb|EL752558.1| AD0014099 Taenia solium UNAM-cd1_adult Taenia ... 448 5e-126

gb|EL746024.1| LV0300055 Taenia solium UNAM-cd2_larva Taenia ... 448 5e-126

gb|EL746765.1| LV0313048 Taenia solium UNAM-cd2_larva Taenia ... 444 6e-125

Query= TSBV.R11.esd 537 0 537 ESD GOOD: 101-537

Length=437

Score E

Sequences producing significant alignments: (Bits) Value

gb|EL757924.1| AD0098009 Taenia solium UNAM-cd1_adult Taenia ... 623 8e-179

gb|EL748482.1| LV0340056 Taenia solium UNAM-cd2_larva Taenia ... 592 2e-169

gb|EL756606.1| AD0030138 Taenia solium UNAM-cd1_adult Taenia ... 497 5e-141

gb|EL759676.1| AD0127017 Taenia solium UNAM-cd1_adult Taenia ... 453 1e-127

Query= TSAP.R80.esd 694 0 694 ESD GOOD: 90-545

Length=456

Score E

Sequences producing significant alignments: (Bits) Value

gb|EL742820.1| LV0247038 Taenia solium UNAM-cd2_larva Taenia ... 774 0.0

gb|EL746325.1| LV0307039 Taenia solium UNAM-cd2_larva Taenia ... 691 0.0

gb|EL753189.1| AD0016227 Taenia solium UNAM-cd1_adult Taenia ... 619 1e-177

gb|EL760499.1| AD0143041 Taenia solium UNAM-cd1_adult Taenia ... 616 1e-176

gb|EL752037.1| AD0012065 Taenia solium UNAM-cd1_adult Taenia ... 597 5e-171

gb|EL752289.1| AD0013056 Taenia solium UNAM-cd1_adult Taenia ... 590 8e-169

gb|EL756468.1| AD0029263 Taenia solium UNAM-cd1_adult Taenia ... 571 3e-163

gb|EL753418.1| AD0017176 Taenia solium UNAM-cd1_adult Taenia ... 460 6e-130

gb|EL758253.1| AD0102064 Taenia solium UNAM-cd1_adult Taenia ... 368 4e-102

gb|EL760879.1| AD0152004 Taenia solium UNAM-cd1_adult Taenia ... 364 5e-101

Query= TSBK.R2.esd 565 0 565 ESD GOOD: 90-560

Length=471

Score E

Sequences producing significant alignments: (Bits) Value

gb|EL761279.1| AD0160030 Taenia solium UNAM-cd1_adult Taenia ... 645 0.0

gb|EL744073.1| LV0267012 Taenia solium UNAM-cd2_larva Taenia ... 296 2e-80

Query= TSBC.R83.esd 625 0 625 ESD GOOD: 91-400

Length=310

Score E

Sequences producing significant alignments: (Bits) Value

gb|EL758234.1| AD0102045 Taenia solium UNAM-cd1_adult Taenia ... 363 2e-100

gb|EL748476.1| LV0340050 Taenia solium UNAM-cd2_larva Taenia ... 357 8e-99

gb|EL745259.1| LV0288041 Taenia solium UNAM-cd2_larva Taenia ... 351 4e-97

Query= TSAV.R40.esd 731 0 731 ESD GOOD: 102-340

Length=239

Score E

Sequences producing significant alignments: (Bits) Value

gb|GT227533.1| tscaa0_001553.z1.scf Taenia solium adult full-... 422 3e-118

gb|EL758670.1| AD0109045 Taenia solium UNAM-cd1_adult Taenia ... 422 3e-118

gb|EL756696.1| AD0030228 Taenia solium UNAM-cd1_adult Taenia ... 414 5e-116

gb|EL746072.1| LV0301027 Taenia solium UNAM-cd2_larva Taenia ... 104 1e-22

Query= TSBN.R2.esd 467 0 467 ESD GOOD: 87-365

Length=279

Score E

Sequences producing significant alignments: (Bits) Value

gb|EL756928.1| AD0031201 Taenia solium UNAM-cd1_adult Taenia ... 477 6e-135

gb|EL754710.1| AD0022235 Taenia solium UNAM-cd1_adult Taenia ... 477 6e-135

gb|EL742849.1| LV0248011 Taenia solium UNAM-cd2_larva Taenia ... 460 6e-130

Query= TSBO.R7.esd 664 0 664 ESD GOOD: 104-618

Length=515

Score E

Sequences producing significant alignments: (Bits) Value

gb|EL744022.1| LV0266022 Taenia solium UNAM-cd2_larva Taenia ... 911 0.0

gb|EL743320.1| LV0255034 Taenia solium UNAM-cd2_larva Taenia ... 614 5e-176

Query= TSBG.R59.esd 554 0 554 ESD GOOD: 105-333

Length=229

Score E

Sequences producing significant alignments: (Bits) Value

gb|EL756928.1| AD0031201 Taenia solium UNAM-cd1_adult Taenia ... 411 6e-115

gb|EL754710.1| AD0022235 Taenia solium UNAM-cd1_adult Taenia ... 411 6e-115

Query= TSAA.R24.esd 713 0 713 ESD GOOD: 103-379

Length=277

Score E

Sequences producing significant alignments: (Bits) Value

gb|EL749272.1| LV0357016 Taenia solium UNAM-cd2_larva Taenia ... 193 3e-49

Query= TSBJ.R49.esd 595 0 595 ESD GOOD: 96-362

Length=267

Score E

Sequences producing significant alignments: (Bits) Value

gb|EL741178.1| LV0222011 Taenia solium UNAM-cd2_larva Taenia ... 311 7e-85

gb|EL763211.1| AD0200026 Taenia solium UNAM-cd1_adult Taenia ... 287 1e-77

Query= TSBN.R15.esd 469 0 469 ESD GOOD: 77-306

Length=230

Score E

Sequences producing significant alignments: (Bits) Value

gb|EL762747.1| AD0192045 Taenia solium UNAM-cd1_adult Taenia ... 342 2e-94

gb|EL758336.1| AD0104021 Taenia solium UNAM-cd1_adult Taenia ... 342 2e-94

Query= TSBA.R28.esd 619 0 619 ESD GOOD: 105-359

Length=255

Score E

Sequences producing significant alignments: (Bits) Value

gb|EL744893.1| LV0282019 Taenia solium UNAM-cd2_larva Taenia ... 374 8e-104

gb|EL744868.1| LV0281028 Taenia solium UNAM-cd2_larva Taenia ... 374 8e-104

gb|EL744660.1| LV0277037 Taenia solium UNAM-cd2_larva Taenia ... 374 8e-104

gb|EL743996.1| LV0265068 Taenia solium UNAM-cd2_larva Taenia ... 374 8e-104

gb|EL743515.1| LV0258055 Taenia solium UNAM-cd2_larva Taenia ... 374 8e-104

gb|EL743380.1| LV0256041 Taenia solium UNAM-cd2_larva Taenia ... 374 8e-104

gb|EL742482.1| LV0242046 Taenia solium UNAM-cd2_larva Taenia ... 374 8e-104

gb|EL760401.1| AD0141038 Taenia solium UNAM-cd1_adult Taenia ... 370 1e-102

gb|EL758748.1| AD0111013 Taenia solium UNAM-cd1_adult Taenia ... 370 1e-102

gb|EL752191.1| AD0012219 Taenia solium UNAM-cd1_adult Taenia ... 370 1e-102

Query= TSBY.R79.esd 760 0 760 ESD GOOD: 106-641

Length=536

Score E

Sequences producing significant alignments: (Bits) Value

gb|EL745228.1| LV0288010 Taenia solium UNAM-cd2_larva Taenia ... 291 9e-79

gb|EL742656.1| LV0245011 Taenia solium UNAM-cd2_larva Taenia ... 291 9e-79

gb|EL742148.1| LV0237036 Taenia solium UNAM-cd2_larva Taenia ... 291 9e-79

gb|EL746868.1| LV0315010 Taenia solium UNAM-cd2_larva Taenia ... 180 2e-45

gb|EL744177.1| LV0268044 Taenia solium UNAM-cd2_larva Taenia ... 56.5 3e-08

Query= TSAK.R79.esd 713 0 713 ESD GOOD: 93-584

Length=492

Score E

Sequences producing significant alignments: (Bits) Value

gb|EL744408.1| LV0272023 Taenia solium UNAM-cd2_larva Taenia ... 835 0.0

gb|EL751944.1| AD0011187 Taenia solium UNAM-cd1_adult Taenia ... 708 0.0

Query= TSBY.R62.esd 774 0 774 ESD GOOD: 111-574

Length=464

Score E

Sequences producing significant alignments: (Bits) Value

gb|EL746216.1| LV0304041 Taenia solium UNAM-cd2_larva Taenia ... 590 8e-169

Query= TSBG.R15.esd 637 0 637 ESD GOOD: 104-395

Length=292

Score E

Sequences producing significant alignments: (Bits) Value

gb|EL757819.1| AD0096022 Taenia solium UNAM-cd1_adult Taenia ... 512 2e-145

gb|EL743425.1| LV0257042 Taenia solium UNAM-cd2_larva Taenia ... 512 2e-145

Query= TSBT.R26.esd 593 0 593 ESD GOOD: 18-570

Length=553

Score E

Sequences producing significant alignments: (Bits) Value

gb|EL763274.1| AD0201037 Taenia solium UNAM-cd1_adult Taenia ... 553 1e-157

gb|GT227428.1| tscaa0_001185.z1.scf Taenia solium adult full-... 403 1e-112

Query= TSBA.R29.esd 633 0 633 ESD GOOD: 102-375

Length=274

Score E

Sequences producing significant alignments: (Bits) Value

gb|GT227287.1| tscaa0_000676.z1.scf Taenia solium adult full-... 99.0 6e-21

Query= TSBS.R30.esd 554 0 554 ESD GOOD: 92-405

Length=314

Score E

Sequences producing significant alignments: (Bits) Value

gb|EL745209.1| LV0287063 Taenia solium UNAM-cd2_larva Taenia ... 89.8 3e-18

Query= TSAQ.R17.esd 602 0 602 ESD GOOD: 87-408

Length=322

Score E

Sequences producing significant alignments: (Bits) Value

gb|EL741533.1| LV0228015 Taenia solium UNAM-cd2_larva Taenia ... 329 2e-90

Query= TSBN.R28.esd 596 0 596 ESD GOOD: 88-294

Length=207

Score E

Sequences producing significant alignments: (Bits) Value

gb|EL741569.1| LV0228051 Taenia solium UNAM-cd2_larva Taenia ... 183 2e-46

Query= TSBU.R84.esd 753 0 753 ESD GOOD: 105-442

Length=338

Score E

Sequences producing significant alignments: (Bits) Value

gb|EL762895.1| AD0195015 Taenia solium UNAM-cd1_adult Taenia ... 292 2e-79

Query= TSAO.R32.esd 632 0 632 ESD GOOD: 104-632

Length=529

Score E

Sequences producing significant alignments: (Bits) Value

gb|EL749112.1| LV0353024 Taenia solium UNAM-cd2_larva Taenia ... 739 0.0

gb|EL747584.1| LV0325063 Taenia solium UNAM-cd2_larva Taenia ... 527 6e-150

gb|EL759641.1| AD0126038 Taenia solium UNAM-cd1_adult Taenia ... 505 3e-143

gb|EL756328.1| AD0029123 Taenia solium UNAM-cd1_adult Taenia ... 494 6e-140

gb|EL758089.1| AD0100034 Taenia solium UNAM-cd1_adult Taenia ... 486 1e-137

gb|EL749275.1| LV0357019 Taenia solium UNAM-cd2_larva Taenia ... 337 1e-92

gb|EL749226.1| LV0356004 Taenia solium UNAM-cd2_larva Taenia ... 337 1e-92

gb|EL743445.1| LV0257062 Taenia solium UNAM-cd2_larva Taenia ... 182 5e-46

gb|EX150516.1| TSEDTS1000B12 Cysti Taenia solium cDNA, mRNA s... 130 2e-30

Query= TSCG.R72.esd 725 0 725 ESD GOOD: 103-628

Length=526

Score E

Sequences producing significant alignments: (Bits) Value

gb|EL749174.1| LV0354033 Taenia solium UNAM-cd2_larva Taenia ... 953 0.0

gb|EL749030.1| LV0351018 Taenia solium UNAM-cd2_larva Taenia ... 953 0.0

gb|EL746558.1| LV0310055 Taenia solium UNAM-cd2_larva Taenia ... 953 0.0

gb|EL743029.1| LV0250070 Taenia solium UNAM-cd2_larva Taenia ... 953 0.0

gb|EL742929.1| LV0249044 Taenia solium UNAM-cd2_larva Taenia ... 937 0.0

gb|EL743905.1| LV0264034 Taenia solium UNAM-cd2_larva Taenia ... 800 0.0

gb|EL749117.1| LV0353029 Taenia solium UNAM-cd2_larva Taenia ... 782 0.0

gb|EL745668.1| LV0295026 Taenia solium UNAM-cd2_larva Taenia ... 771 0.0

gb|EL761570.1| AD0166011 Taenia solium UNAM-cd1_adult Taenia ... 641 0.0

gb|EL741609.1| LV0229020 Taenia solium UNAM-cd2_larva Taenia ... 580 5e-166

Query= TSBL.R14.esd 576 0 576 ESD GOOD: 86-575

Length=490

Score E

Sequences producing significant alignments: (Bits) Value

gb|EL746694.1| LV0312055 Taenia solium UNAM-cd2_larva Taenia ... 821 0.0

gb|EL748125.1| LV0334008 Taenia solium UNAM-cd2_larva Taenia ... 202 4e-52

Query= TSAU.R47.esd 559 0 559 ESD GOOD: 96-556

Length=461

Score E

Sequences producing significant alignments: (Bits) Value

gb|EL745899.1| LV0298056 Taenia solium UNAM-cd2_larva Taenia ... 761 0.0

Query= TSAK.R32.esd 617 0 617 ESD GOOD: 100-563

Length=464

Score E

Sequences producing significant alignments: (Bits) Value

gb|EL745332.1| LV0289044 Taenia solium UNAM-cd2_larva Taenia ... 385 4e-107

gb|EL740692.1| LV0214017 Taenia solium UNAM-cd2_larva Taenia ... 243 2e-64

gb|EL745331.1| LV0289043 Taenia solium UNAM-cd2_larva Taenia ... 183 2e-46

Query= TSAV.R59.esd 636 0 636 ESD GOOD: 100-599

Length=500

Score E

Sequences producing significant alignments: (Bits) Value

gb|GT227767.1| tscaa0_002361.z1.scf Taenia solium adult full-... 440 8e-124

gb|EL759667.1| AD0127008 Taenia solium UNAM-cd1_adult Taenia ... 128 7e-30

gb|EL759486.1| AD0123065 Taenia solium UNAM-cd1_adult Taenia ... 122 3e-28

gb|EL755621.1| AD0026144 Taenia solium UNAM-cd1_adult Taenia ... 122 3e-28

gb|EL756149.1| AD0028207 Taenia solium UNAM-cd1_adult Taenia ... 119 4e-27

gb|EL754618.1| AD0022143 Taenia solium UNAM-cd1_adult Taenia ... 119 4e-27

gb|EL752712.1| AD0014253 Taenia solium UNAM-cd1_adult Taenia ... 119 4e-27

gb|EL754346.1| AD0021136 Taenia solium UNAM-cd1_adult Taenia ... 117 2e-26

gb|EL756495.1| AD0030027 Taenia solium UNAM-cd1_adult Taenia ... 100 2e-21

gb|EL750329.1| AD0004244 Taenia solium UNAM-cd1_adult Taenia ... 100 2e-21

Query= TSAM.R34.esd 659 0 659 ESD GOOD: 92-512

Length=421

Score E

Sequences producing significant alignments: (Bits) Value

gb|EL742465.1| LV0242029 Taenia solium UNAM-cd2_larva Taenia ... 436 1e-122

gb|EL742129.1| LV0237017 Taenia solium UNAM-cd2_larva Taenia ... 387 1e-107

Query= TSCF.R13.esd 591 0 591 ESD GOOD: 108-458

Length=351

Score E

Sequences producing significant alignments: (Bits) Value

gb|EL742881.1| LV0248043 Taenia solium UNAM-cd2_larva Taenia ... 525 2e-149

gb|EL742752.1| LV0246035 Taenia solium UNAM-cd2_larva Taenia ... 523 8e-149

gb|EL741105.1| LV0221006 Taenia solium UNAM-cd2_larva Taenia ... 521 3e-148

Query= TSAF.R50.esd 478 0 478 ESD GOOD: 92-408

Length=317

Score E

Sequences producing significant alignments: (Bits) Value

gb|EL743140.1| LV0252041 Taenia solium UNAM-cd2_larva Taenia ... 575 2e-164

gb|EL741300.1| LV0224020 Taenia solium UNAM-cd2_larva Taenia ... 560 6e-160

gb|EL763080.1| AD0198018 Taenia solium UNAM-cd1_adult Taenia ... 520 1e-147

gb|EL757564.1| AD0035157 Taenia solium UNAM-cd1_adult Taenia ... 520 1e-147

gb|EL757025.1| AD0032037 Taenia solium UNAM-cd1_adult Taenia ... 520 1e-147

gb|EL753882.1| AD0019169 Taenia solium UNAM-cd1_adult Taenia ... 520 1e-147

gb|EL750248.1| AD0004163 Taenia solium UNAM-cd1_adult Taenia ... 520 1e-147

gb|EL757258.1| AD0034074 Taenia solium UNAM-cd1_adult Taenia ... 514 5e-146

gb|EL753979.1| AD0020024 Taenia solium UNAM-cd1_adult Taenia ... 514 5e-146

gb|EL759153.1| AD0117056 Taenia solium UNAM-cd1_adult Taenia ... 497 5e-141

Query= TSBH.R95.esd 449 0 449 ESD GOOD: 79-443

Length=365

Score E

Sequences producing significant alignments: (Bits) Value

gb|EL763089.1| AD0198027 Taenia solium UNAM-cd1_adult Taenia ... 505 3e-143

Query= TSBT.R91.esd 782 0 782 ESD GOOD: 90-621

Length=532

Score E

Sequences producing significant alignments: (Bits) Value

gb|EL759604.1| AD0126001 Taenia solium UNAM-cd1_adult Taenia ... 473 8e-134

Query= TSCG.R34.esd 664 0 664 ESD GOOD: 95-638

Length=544

Score E

Sequences producing significant alignments: (Bits) Value

gb|EL761209.1| AD0159006 Taenia solium UNAM-cd1_adult Taenia ... 909 0.0

gb|EL740792.1| LV0216026 Taenia solium UNAM-cd2_larva Taenia ... 773 0.0

gb|EL763290.1| AD0201053 Taenia solium UNAM-cd1_adult Taenia ... 763 0.0

gb|EL740480.1| LV0210004 Taenia solium UNAM-cd2_larva Taenia ... 763 0.0

gb|EL753708.1| AD0018246 Taenia solium UNAM-cd1_adult Taenia ... 758 0.0

gb|EL743855.1| LV0263024 Taenia solium UNAM-cd2_larva Taenia ... 676 0.0

gb|EL741281.1| LV0224001 Taenia solium UNAM-cd2_larva Taenia ... 619 1e-177

gb|EL742423.1| LV0241049 Taenia solium UNAM-cd2_larva Taenia ... 503 1e-142

Query= TSAO.R39.esd 534 0 534 ESD GOOD: 105-534

Length=430

Score E

Sequences producing significant alignments: (Bits) Value

gb|EL744554.1| LV0275029 Taenia solium UNAM-cd2_larva Taenia ... 191 9e-49

Query= TSBW.R48.esd 523 0 523 ESD GOOD: 100-523

Length=424

Score E

Sequences producing significant alignments: (Bits) Value

gb|EL762715.1| AD0192013 Taenia solium UNAM-cd1_adult Taenia ... 512 2e-145

gb|EL747303.1| LV0321034 Taenia solium UNAM-cd2_larva Taenia ... 379 2e-105

Query= TSBN.R9.esd 544 0 544 ESD GOOD: 91-544

Length=454

Score E

Sequences producing significant alignments: (Bits) Value

gb|EL740420.1| LV0208040 Taenia solium UNAM-cd2_larva Taenia ... 231 5e-61

Query= TSBB.R50.esd 597 0 597 ESD GOOD: 97-578

Length=482

Score E

Sequences producing significant alignments: (Bits) Value

gb|EL755218.1| AD0024235 Taenia solium UNAM-cd1_adult Taenia ... 749 0.0

Query= TSBU.R78.esd 840 0 840 ESD GOOD: 106-545

Length=440

Score E

Sequences producing significant alignments: (Bits) Value

gb|EL752559.1| AD0014100 Taenia solium UNAM-cd1_adult Taenia ... 542 2e-154

gb|EL758804.1| AD0112003 Taenia solium UNAM-cd1_adult Taenia ... 361 7e-100

gb|EL752817.1| AD0015076 Taenia solium UNAM-cd1_adult Taenia ... 113 2e-25

Query= TSCC.R62.esd 671 0 671 ESD GOOD: 93-598

Length=506

Score E

Sequences producing significant alignments: (Bits) Value

gb|EL745608.1| LV0294023 Taenia solium UNAM-cd2_larva Taenia ... 337 1e-92

Query= TSAW.R94.esd 572 0 572 ESD GOOD: 91-555

Length=465

Score E

Sequences producing significant alignments: (Bits) Value

gb|EL745342.1| LV0289054 Taenia solium UNAM-cd2_larva Taenia ... 846 0.0

Query= TSBA.R41.esd 582 0 582 ESD GOOD: 101-272

Length=172

Score E

Sequences producing significant alignments: (Bits) Value

gb|EL748620.1| LV0343005 Taenia solium UNAM-cd2_larva Taenia ... 124 9e-29

Query= TSAJ.R41.esd 666 0 666 ESD GOOD: 101-466

Length=366

Score E

Sequences producing significant alignments: (Bits) Value

gb|EL763460.1| AD0204023 Taenia solium UNAM-cd1_adult Taenia ... 383 1e-106

Query= TSAZ.R56.esd 667 0 667 ESD GOOD: 101-618

Length=518

Score E

Sequences producing significant alignments: (Bits) Value

gb|EL741048.1| LV0220021 Taenia solium UNAM-cd2_larva Taenia ... 335 4e-92

Query= TSAQ.R20.esd 657 0 657 ESD GOOD: 86-476

Length=391

Score E

Sequences producing significant alignments: (Bits) Value

gb|EL748985.1| LV0349034 Taenia solium UNAM-cd2_larva Taenia ... 590 8e-169

gb|EL749247.1| LV0356025 Taenia solium UNAM-cd2_larva Taenia ... 344 7e-95

gb|EL749093.1| LV0353005 Taenia solium UNAM-cd2_larva Taenia ... 122 3e-28

Query= TSAQ.R62.esd 679 0 679 ESD GOOD: 93-595

Length=503

Score E

Sequences producing significant alignments: (Bits) Value

gb|EL749471.1| AD0001094 Taenia solium UNAM-cd1_adult Taenia ... 776 0.0

gb|EL753717.1| AD0019004 Taenia solium UNAM-cd1_adult Taenia ... 761 0.0

Query= TSCA.R93.esd 754 0 754 ESD GOOD: 99-616

Length=518

Score E

Sequences producing significant alignments: (Bits) Value

gb|GT227197.1| tscaa0_000429.z1.scf Taenia solium adult full-... 309 2e-84

Query= TSAA.R62.esd 594 0 594 ESD GOOD: 99-439

Length=341

Score E

Sequences producing significant alignments: (Bits) Value

gb|EL744563.1| LV0275038 Taenia solium UNAM-cd2_larva Taenia ... 435 4e-122

gb|EL746766.1| LV0313049 Taenia solium UNAM-cd2_larva Taenia ... 427 6e-120

gb|EL761299.1| AD0161016 Taenia solium UNAM-cd1_adult Taenia ... 407 8e-114

Query= TSCB.R28.esd 501 0 501 ESD GOOD: 101-498

Length=398

Score E

Sequences producing significant alignments: (Bits) Value

gb|EL753403.1| AD0017161 Taenia solium UNAM-cd1_adult Taenia ... 603 1e-172

gb|EL753172.1| AD0016210 Taenia solium UNAM-cd1_adult Taenia ... 344 7e-95

gb|EL745026.1| LV0284057 Taenia solium UNAM-cd2_larva Taenia ... 285 4e-77

Query= TSAN.R66.esd 700 0 700 ESD GOOD: 105-553

Length=449

Score E

Sequences producing significant alignments: (Bits) Value

gb|EL763079.1| AD0198017 Taenia solium UNAM-cd1_adult Taenia ... 804 0.0

Query= TSBA.R44.esd 440 0 440 ESD GOOD: 160-440

Length=281

Score E

Sequences producing significant alignments: (Bits) Value

gb|EL758087.1| AD0100032 Taenia solium UNAM-cd1_adult Taenia ... 374 8e-104

Query= TSCG.R30.esd 532 0 532 ESD GOOD: 99-484

Length=386

Score E

Sequences producing significant alignments: (Bits) Value

gb|EL749100.1| LV0353012 Taenia solium UNAM-cd2_larva Taenia ... 699 0.0

Query= TSAR.R33.esd 565 0 565 ESD GOOD: 102-565

Length=464

Score E

Sequences producing significant alignments: (Bits) Value

gb|EL743711.1| LV0261020 Taenia solium UNAM-cd2_larva Taenia ... 270 1e-72

Query= TSCG.R78.esd 463 0 463 ESD GOOD: 99-431

Length=333

Score E

Sequences producing significant alignments: (Bits) Value

gb|EL762592.1| AD0189049 Taenia solium UNAM-cd1_adult Taenia ... 283 1e-76

Query= TSCG.R16.esd 457 0 457 ESD GOOD: 98-457

Length=360

Score E

Sequences producing significant alignments: (Bits) Value

gb|EL742011.1| LV0234072 Taenia solium UNAM-cd2_larva Taenia ... 640 0.0

gb|EL745704.1| LV0295062 Taenia solium UNAM-cd2_larva Taenia ... 505 3e-143

gb|EL740689.1| LV0214014 Taenia solium UNAM-cd2_larva Taenia ... 479 2e-135

gb|EL746993.1| LV0316070 Taenia solium UNAM-cd2_larva Taenia ... 433 1e-121

gb|EL741658.1| LV0230005 Taenia solium UNAM-cd2_larva Taenia ... 350 1e-96

gb|EL744843.1| LV0281003 Taenia solium UNAM-cd2_larva Taenia ... 207 9e-54

Query= TSAJ.R19.esd 678 0 678 ESD GOOD: 100-575

Length=476

Score E

Sequences producing significant alignments: (Bits) Value

gb|EL761350.1| AD0162011 Taenia solium UNAM-cd1_adult Taenia ... 769 0.0

gb|EL740733.1| LV0215023 Taenia solium UNAM-cd2_larva Taenia ... 652 0.0

gb|EL742022.1| LV0235011 Taenia solium UNAM-cd2_larva Taenia ... 481 5e-136

gb|EL744989.1| LV0284020 Taenia solium UNAM-cd2_larva Taenia ... 453 1e-127

Query= TSCE.R62.esd 644 0 644 ESD GOOD: 97-585

Length=489

Score E

Sequences producing significant alignments: (Bits) Value

gb|EL743330.1| LV0255044 Taenia solium UNAM-cd2_larva Taenia ... 702 0.0

Query= TSCG.R50.esd 602 0 602 ESD GOOD: 99-465

Length=367

Score E

Sequences producing significant alignments: (Bits) Value

gb|EL748117.1| LV0333068 Taenia solium UNAM-cd2_larva Taenia ... 612 2e-175

Query= TSAK.R14.esd 717 0 717 ESD GOOD: 88-487

Length=400

Score E

Sequences producing significant alignments: (Bits) Value

gb|EL745597.1| LV0294012 Taenia solium UNAM-cd2_larva Taenia ... 651 0.0

gb|EL757354.1| AD0034170 Taenia solium UNAM-cd1_adult Taenia ... 641 0.0

gb|EL750109.1| AD0004024 Taenia solium UNAM-cd1_adult Taenia ... 632 0.0

gb|EL762159.1| AD0180001 Taenia solium UNAM-cd1_adult Taenia ... 577 6e-165

gb|EL741848.1| LV0232053 Taenia solium UNAM-cd2_larva Taenia ... 573 8e-164

gb|EL750361.1| AD0005024 Taenia solium UNAM-cd1_adult Taenia ... 571 3e-163

gb|EL742181.1| LV0237069 Taenia solium UNAM-cd2_larva Taenia ... 440 8e-124

gb|EL748629.1| LV0343014 Taenia solium UNAM-cd2_larva Taenia ... 420 1e-117

gb|EL758451.1| AD0105070 Taenia solium UNAM-cd1_adult Taenia ... 394 6e-110

gb|EL746753.1| LV0313036 Taenia solium UNAM-cd2_larva Taenia ... 387 1e-107

Query= TSBA.R33.esd 583 0 583 ESD GOOD: 124-535

Length=412

Score E

Sequences producing significant alignments: (Bits) Value

gb|EL751839.1| AD0011082 Taenia solium UNAM-cd1_adult Taenia ... 719 0.0

gb|EL754332.1| AD0021122 Taenia solium UNAM-cd1_adult Taenia ... 444 6e-125

gb|EL751599.1| AD0010092 Taenia solium UNAM-cd1_adult Taenia ... 444 6e-125

gb|EL760568.1| AD0144055 Taenia solium UNAM-cd1_adult Taenia ... 348 5e-96

gb|EL759950.1| AD0132041 Taenia solium UNAM-cd1_adult Taenia ... 237 1e-62

Query= TSAP.R2.esd 806 0 806 ESD GOOD: 90-584

Length=495

Score E

Sequences producing significant alignments: (Bits) Value

gb|EL748215.1| LV0335030 Taenia solium UNAM-cd2_larva Taenia ... 523 8e-149

gb|EL742560.1| LV0243058 Taenia solium UNAM-cd2_larva Taenia ... 196 2e-50

Query= TSAO.R88.esd 655 0 655 ESD GOOD: 116-552

Length=437

Score E

Sequences producing significant alignments: (Bits) Value

gb|EL749205.1| LV0355019 Taenia solium UNAM-cd2_larva Taenia ... 449 1e-126

Query= TSAB.R57.esd 610 0 610 ESD GOOD: 98-440

Length=343

Score E

Sequences producing significant alignments: (Bits) Value

gb|EL747938.1| LV0331031 Taenia solium UNAM-cd2_larva Taenia ... 503 1e-142

Query= TSBN.R63.esd 513 0 513 ESD GOOD: 103-204

Length=102

Score E

Sequences producing significant alignments: (Bits) Value

gb|EL748131.1| LV0334014 Taenia solium UNAM-cd2_larva Taenia ... 176 3e-44

gb|EL744672.1| LV0277049 Taenia solium UNAM-cd2_larva Taenia ... 176 3e-44

gb|EL760440.1| AD0142038 Taenia solium UNAM-cd1_adult Taenia ... 174 9e-44

gb|EL754932.1| AD0023188 Taenia solium UNAM-cd1_adult Taenia ... 174 9e-44

gb|EL754736.1| AD0022261 Taenia solium UNAM-cd1_adult Taenia ... 174 9e-44

gb|EL754192.1| AD0020237 Taenia solium UNAM-cd1_adult Taenia ... 174 9e-44

gb|EL760500.1| AD0143042 Taenia solium UNAM-cd1_adult Taenia ... 171 1e-42

gb|EL758258.1| AD0103002 Taenia solium UNAM-cd1_adult Taenia ... 171 1e-42

gb|EL750145.1| AD0004060 Taenia solium UNAM-cd1_adult Taenia ... 171 1e-42

gb|EL763226.1| AD0200041 Taenia solium UNAM-cd1_adult Taenia ... 167 2e-41

Query= TSAC.R53.esd 477 0 477 ESD GOOD: 109-477

Length=369

Score E

Sequences producing significant alignments: (Bits) Value

gb|EL757686.1| AD0094026 Taenia solium UNAM-cd1_adult Taenia ... 231 5e-61

Query= TSBV.R92.esd 575 0 575 ESD GOOD: 102-513

Length=412

Score E

Sequences producing significant alignments: (Bits) Value

gb|EL743441.1| LV0257058 Taenia solium UNAM-cd2_larva Taenia ... 431 5e-121

gb|EL741749.1| LV0231027 Taenia solium UNAM-cd2_larva Taenia ... 431 5e-121

Query= TSBV.R21.esd 556 0 556 ESD GOOD: 107-440

Length=334

Score E

Sequences producing significant alignments: (Bits) Value

gb|EL748522.1| LV0341038 Taenia solium UNAM-cd2_larva Taenia ... 392 2e-109

Query= TSBR.R43.esd 686 0 686 ESD GOOD: 100-605

Length=506

Score E

Sequences producing significant alignments: (Bits) Value

gb|EL760437.1| AD0142035 Taenia solium UNAM-cd1_adult Taenia ... 261 7e-70

Query= TSAG.R17.esd 622 0 622 ESD GOOD: 91-426

Length=336

Score E

Sequences producing significant alignments: (Bits) Value

gb|GT227714.1| tscaa0_002143.z1.scf Taenia solium adult full-... 496 2e-140

Query= TSBZ.R96.esd 792 0 792 ESD GOOD: 102-632

Length=531

Score E

Sequences producing significant alignments: (Bits) Value

gb|EL751256.1| AD0008267 Taenia solium UNAM-cd1_adult Taenia ... 867 0.0

gb|EL751501.1| AD0009239 Taenia solium UNAM-cd1_adult Taenia ... 837 0.0

gb|EL741323.1| LV0224043 Taenia solium UNAM-cd2_larva Taenia ... 259 2e-69

Query= TSCE.R5.esd 605 0 605 ESD GOOD: 93-561

Length=469

Score E

Sequences producing significant alignments: (Bits) Value

gb|EL761504.1| AD0165002 Taenia solium UNAM-cd1_adult Taenia ... 475 2e-134

Query= TSBF.R88.esd 463 0 463 ESD GOOD: 89-463

Length=375

Score E

Sequences producing significant alignments: (Bits) Value

gb|EL757843.1| AD0096046 Taenia solium UNAM-cd1_adult Taenia ... 379 2e-105

gb|EL746236.1| LV0305020 Taenia solium UNAM-cd2_larva Taenia ... 200 2e-51

gb|EL746309.1| LV0307023 Taenia solium UNAM-cd2_larva Taenia ... 97.1 2e-20

Query= TSAR.R80.esd 686 0 686 ESD GOOD: 99-498

Length=400

Score E

Sequences producing significant alignments: (Bits) Value

gb|EL740795.1| LV0216029 Taenia solium UNAM-cd2_larva Taenia ... 396 2e-110

gb|EL762462.1| AD0186026 Taenia solium UNAM-cd1_adult Taenia ... 387 1e-107

Query= TSBA.R30.esd 372 0 372 ESD GOOD: 98-263

Length=166

Score E

Sequences producing significant alignments: (Bits) Value

gb|EL747914.1| LV0331007 Taenia solium UNAM-cd2_larva Taenia ... 222 3e-58

Query= TSAU.R30.esd 663 0 663 ESD GOOD: 88-439

Length=352

Score E

Sequences producing significant alignments: (Bits) Value

gb|EL755463.1| AD0025233 Taenia solium UNAM-cd1_adult Taenia ... 335 4e-92

gb|EL753550.1| AD0018088 Taenia solium UNAM-cd1_adult Taenia ... 335 4e-92

gb|EL743313.1| LV0255027 Taenia solium UNAM-cd2_larva Taenia ... 327 7e-90

Query= TSCC.R47.esd 710 0 710 ESD GOOD: 111-563

Length=453

Score E

Sequences producing significant alignments: (Bits) Value

gb|EL747020.1| LV0317017 Taenia solium UNAM-cd2_larva Taenia ... 808 0.0

gb|GT227495.1| tscaa0_001467.z1.scf Taenia solium adult full-... 588 3e-168

gb|EL760629.1| AD0146014 Taenia solium UNAM-cd1_adult Taenia ... 156 3e-38

gb|EL758634.1| AD0109009 Taenia solium UNAM-cd1_adult Taenia ... 156 3e-38

Query= TSAW.R72.esd 621 0 621 ESD GOOD: 94-584

Length=491

Score E

Sequences producing significant alignments: (Bits) Value

gb|EL748804.1| LV0345056 Taenia solium UNAM-cd2_larva Taenia ... 457 8e-129

Query= TSCE.R89.esd 543 0 543 ESD GOOD: 103-512

Length=410

Score E

Sequences producing significant alignments: (Bits) Value

gb|EL741724.1| LV0231002 Taenia solium UNAM-cd2_larva Taenia ... 102 4e-22

Query= TSAT.R56.esd 671 0 671 ESD GOOD: 106-645

Length=540

Score E

Sequences producing significant alignments: (Bits) Value

gb|EL744024.1| LV0266024 Taenia solium UNAM-cd2_larva Taenia ... 532 1e-151

gb|EL742677.1| LV0245032 Taenia solium UNAM-cd2_larva Taenia ... 531 5e-151

Query= TSAF.R38.esd 655 0 655 ESD GOOD: 101-616

Length=516

Score E

Sequences producing significant alignments: (Bits) Value

gb|EL745364.1| LV0290019 Taenia solium UNAM-cd2_larva Taenia ... 429 2e-120

gb|EL745319.1| LV0289031 Taenia solium UNAM-cd2_larva Taenia ... 396 2e-110

Query= TSBG.R66.esd 678 0 678 ESD GOOD: 106-415

Length=310

Score E

Sequences producing significant alignments: (Bits) Value

gb|EL757505.1| AD0035098 Taenia solium UNAM-cd1_adult Taenia ... 558 2e-159

gb|EL755907.1| AD0027203 Taenia solium UNAM-cd1_adult Taenia ... 558 2e-159

gb|EL749789.1| AD0002153 Taenia solium UNAM-cd1_adult Taenia ... 558 2e-159

gb|EL747639.1| LV0326055 Taenia solium UNAM-cd2_larva Taenia ... 558 2e-159

gb|EL759839.1| AD0130029 Taenia solium UNAM-cd1_adult Taenia ... 542 2e-154

gb|EL745562.1| LV0293040 Taenia solium UNAM-cd2_larva Taenia ... 361 7e-100

Query= TSAK.R49.esd 710 0 710 ESD GOOD: 95-588

Length=494

Score E

Sequences producing significant alignments: (Bits) Value

gb|EL748623.1| LV0343008 Taenia solium UNAM-cd2_larva Taenia ... 374 8e-104

gb|EL742565.1| LV0243063 Taenia solium UNAM-cd2_larva Taenia ... 366 1e-101

gb|EL741696.1| LV0230043 Taenia solium UNAM-cd2_larva Taenia ... 366 1e-101

gb|EL748163.1| LV0334046 Taenia solium UNAM-cd2_larva Taenia ... 324 9e-89

gb|EL747383.1| LV0322060 Taenia solium UNAM-cd2_larva Taenia ... 311 7e-85

gb|EL745659.1| LV0295017 Taenia solium UNAM-cd2_larva Taenia ... 298 5e-81

gb|EL743536.1| LV0258076 Taenia solium UNAM-cd2_larva Taenia ... 230 2e-60

Query= TSAQ.R70.esd 628 0 628 ESD GOOD: 87-593

Length=507

Score E

Sequences producing significant alignments: (Bits) Value

gb|EL746632.1| LV0311061 Taenia solium UNAM-cd2_larva Taenia ... 549 1e-156

Query= TSCE.R1.esd 628 0 628 ESD GOOD: 98-628

Length=531

Score E

Sequences producing significant alignments: (Bits) Value

gb|EL747727.1| LV0328015 Taenia solium UNAM-cd2_larva Taenia ... 749 0.0

gb|EL762844.1| AD0194015 Taenia solium UNAM-cd1_adult Taenia ... 608 2e-174

gb|EL755545.1| AD0026068 Taenia solium UNAM-cd1_adult Taenia ... 329 2e-90

gb|EL748592.1| LV0342043 Taenia solium UNAM-cd2_larva Taenia ... 320 1e-87

gb|EL748087.1| LV0333038 Taenia solium UNAM-cd2_larva Taenia ... 281 5e-76

gb|GT227350.1| tscaa0_000959.z1.scf Taenia solium adult full-... 257 9e-69

gb|EL744389.1| LV0272004 Taenia solium UNAM-cd2_larva Taenia ... 248 5e-66

gb|EL758627.1| AD0109002 Taenia solium UNAM-cd1_adult Taenia ... 75.0 1e-13

Query= TSCC.R72.esd 746 0 746 ESD GOOD: 117-612

Length=496

Score E

Sequences producing significant alignments: (Bits) Value

gb|EL741467.1| LV0227023 Taenia solium UNAM-cd2_larva Taenia ... 789 0.0

gb|EL760524.1| AD0144011 Taenia solium UNAM-cd1_adult Taenia ... 122 3e-28

gb|EL751218.1| AD0008229 Taenia solium UNAM-cd1_adult Taenia ... 122 3e-28

gb|EL751057.1| AD0008068 Taenia solium UNAM-cd1_adult Taenia ... 122 3e-28

gb|EL762557.1| AD0189014 Taenia solium UNAM-cd1_adult Taenia ... 95.3 7e-20

Query= TSBJ.R74.esd 579 0 579 ESD GOOD: 96-523

Length=428

Score E

Sequences producing significant alignments: (Bits) Value

gb|EL749346.1| LV0359024 Taenia solium UNAM-cd2_larva Taenia ... 739 0.0

gb|EL749221.1| LV0355035 Taenia solium UNAM-cd2_larva Taenia ... 739 0.0

gb|EL749184.1| LV0354043 Taenia solium UNAM-cd2_larva Taenia ... 739 0.0

gb|EL749101.1| LV0353013 Taenia solium UNAM-cd2_larva Taenia ... 739 0.0

gb|EL748974.1| LV0349023 Taenia solium UNAM-cd2_larva Taenia ... 713 0.0

Query= TSBN.R58.esd 621 0 621 ESD GOOD: 88-323

Length=236

Score E

Sequences producing significant alignments: (Bits) Value

gb|EL742092.1| LV0236021 Taenia solium UNAM-cd2_larva Taenia ... 182 5e-46

Query= TSAD.R9.esd 675 0 675 ESD GOOD: 94-623

Length=530

Score E

Sequences producing significant alignments: (Bits) Value

gb|GT227462.1| tscaa0_001379.z1.scf Taenia solium adult full-... 643 0.0

Query= TSAH.R78.esd 588 0 588 ESD GOOD: 105-537

Length=433

Score E

Sequences producing significant alignments: (Bits) Value

gb|GT227121.1| tscaa0_000189.z1.scf Taenia solium adult full-... 627 6e-180

gb|EL749215.1| LV0355029 Taenia solium UNAM-cd2_larva Taenia ... 449 1e-126

gb|EL748995.1| LV0350004 Taenia solium UNAM-cd2_larva Taenia ... 449 1e-126

gb|EL757979.1| AD0098064 Taenia solium UNAM-cd1_adult Taenia ... 436 1e-122

gb|EL755166.1| AD0024183 Taenia solium UNAM-cd1_adult Taenia ... 436 1e-122

gb|EL755072.1| AD0024089 Taenia solium UNAM-cd1_adult Taenia ... 436 1e-122

gb|EL745820.1| LV0297049 Taenia solium UNAM-cd2_larva Taenia ... 436 1e-122

gb|EL743959.1| LV0265031 Taenia solium UNAM-cd2_larva Taenia ... 436 1e-122

gb|EL748715.1| LV0344038 Taenia solium UNAM-cd2_larva Taenia ... 427 6e-120

gb|EL748726.1| LV0344049 Taenia solium UNAM-cd2_larva Taenia ... 399 1e-111

Query= TSBU.R19.esd 673 0 673 ESD GOOD: 101-564

Length=464

Score E

Sequences producing significant alignments: (Bits) Value

gb|EL741294.1| LV0224014 Taenia solium UNAM-cd2_larva Taenia ... 575 2e-164

Query= TSAT.R13.esd 682 0 682 ESD GOOD: 105-476

Length=372

Score E

Sequences producing significant alignments: (Bits) Value

gb|EL761234.1| AD0159031 Taenia solium UNAM-cd1_adult Taenia ... 453 1e-127

Query= TSBJ.R36.esd 513 0 513 ESD GOOD: 120-513

Length=394

Score E

Sequences producing significant alignments: (Bits) Value

gb|EL740765.1| LV0215055 Taenia solium UNAM-cd2_larva Taenia ... 80.5 2e-15

Query= TSAJ.R91.esd 703 0 703 ESD GOOD: 162-657

Length=496

Score E

Sequences producing significant alignments: (Bits) Value

gb|EL742115.1| LV0237003 Taenia solium UNAM-cd2_larva Taenia ... 278 7e-75

Query= TSBJ.R64.esd 754 0 754 ESD GOOD: 99-217

Length=119

Score E

Sequences producing significant alignments: (Bits) Value

gb|GT227762.1| tscaa0_002352.z1.scf Taenia solium adult full-... 159 3e-39

gb|EL759361.1| AD0122005 Taenia solium UNAM-cd1_adult Taenia ... 159 3e-39

Query= TSBH.R55.esd 415 0 415 ESD GOOD: 114-414

Length=301

Score E

Sequences producing significant alignments: (Bits) Value

gb|EL762995.1| AD0197004 Taenia solium UNAM-cd1_adult Taenia ... 538 3e-153

gb|EL762705.1| AD0192003 Taenia solium UNAM-cd1_adult Taenia ... 538 3e-153

gb|EL743867.1| LV0263036 Taenia solium UNAM-cd2_larva Taenia ... 527 6e-150

gb|EL754940.1| AD0023196 Taenia solium UNAM-cd1_adult Taenia ... 501 4e-142

gb|EL758753.1| AD0111018 Taenia solium UNAM-cd1_adult Taenia ... 464 5e-131

gb|EL752871.1| AD0015130 Taenia solium UNAM-cd1_adult Taenia ... 296 2e-80

gb|EL752370.1| AD0013137 Taenia solium UNAM-cd1_adult Taenia ... 255 3e-68

gb|EL761885.1| AD0173006 Taenia solium UNAM-cd1_adult Taenia ... 148 6e-36

gb|EL761808.1| AD0171025 Taenia solium UNAM-cd1_adult Taenia ... 139 3e-33

gb|EL761102.1| AD0156042 Taenia solium UNAM-cd1_adult Taenia ... 137 1e-32

Query= TSCD.R49.esd 571 0 571 ESD GOOD: 96-550

Length=455

Score E

Sequences producing significant alignments: (Bits) Value

gb|EL748633.1| LV0343018 Taenia solium UNAM-cd2_larva Taenia ... 632 0.0

Query= TSBP.R3.esd 424 0 424 ESD GOOD: 90-424

Length=335

Score E

Sequences producing significant alignments: (Bits) Value

gb|EL745210.1| LV0287064 Taenia solium UNAM-cd2_larva Taenia ... 464 5e-131

Query= TSBV.R13.esd 554 0 554 ESD GOOD: 107-402

Length=296

Score E

Sequences producing significant alignments: (Bits) Value

gb|EL741533.1| LV0228015 Taenia solium UNAM-cd2_larva Taenia ... 418 4e-117

Query= TSAB.R53.esd 741 0 741 ESD GOOD: 112-586

Length=475

Score E

Sequences producing significant alignments: (Bits) Value

gb|EL749226.1| LV0356004 Taenia solium UNAM-cd2_larva Taenia ... 856 0.0

gb|EL749275.1| LV0357019 Taenia solium UNAM-cd2_larva Taenia ... 824 0.0

gb|EL743445.1| LV0257062 Taenia solium UNAM-cd2_larva Taenia ... 761 0.0

gb|EL762778.1| AD0193025 Taenia solium UNAM-cd1_adult Taenia ... 725 0.0

gb|EL762277.1| AD0182033 Taenia solium UNAM-cd1_adult Taenia ... 686 0.0

gb|EL759641.1| AD0126038 Taenia solium UNAM-cd1_adult Taenia ... 662 0.0

gb|EL756328.1| AD0029123 Taenia solium UNAM-cd1_adult Taenia ... 647 0.0

gb|EL747584.1| LV0325063 Taenia solium UNAM-cd2_larva Taenia ... 381 5e-106

gb|EL749112.1| LV0353024 Taenia solium UNAM-cd2_larva Taenia ... 333 1e-91

gb|EL758089.1| AD0100034 Taenia solium UNAM-cd1_adult Taenia ... 294 7e-80

Query= TSBR.R2.esd 821 0 821 ESD GOOD: 98-637

Length=540

Score E

Sequences producing significant alignments: (Bits) Value

gb|EL761686.1| AD0169003 Taenia solium UNAM-cd1_adult Taenia ... 894 0.0

gb|EL761038.1| AD0155026 Taenia solium UNAM-cd1_adult Taenia ... 843 0.0

gb|EL762221.1| AD0181018 Taenia solium UNAM-cd1_adult Taenia ... 529 2e-150

gb|EL762220.1| AD0181017 Taenia solium UNAM-cd1_adult Taenia ... 252 4e-67

Query= TSBK.R87.esd 604 0 604 ESD GOOD: 86-551

Length=466

Score E

Sequences producing significant alignments: (Bits) Value

gb|EL763071.1| AD0198009 Taenia solium UNAM-cd1_adult Taenia ... 399 1e-111

gb|EL755711.1| AD0027007 Taenia solium UNAM-cd1_adult Taenia ... 399 1e-111

gb|EL755580.1| AD0026103 Taenia solium UNAM-cd1_adult Taenia ... 399 1e-111

gb|EL751672.1| AD0010165 Taenia solium UNAM-cd1_adult Taenia ... 394 6e-110

gb|EL751898.1| AD0011141 Taenia solium UNAM-cd1_adult Taenia ... 363 2e-100

gb|EL755581.1| AD0026104 Taenia solium UNAM-cd1_adult Taenia ... 359 2e-99

gb|EL751673.1| AD0010166 Taenia solium UNAM-cd1_adult Taenia ... 359 2e-99

gb|EL763072.1| AD0198010 Taenia solium UNAM-cd1_adult Taenia ... 357 8e-99

gb|EL755712.1| AD0027008 Taenia solium UNAM-cd1_adult Taenia ... 340 9e-94

gb|EL751899.1| AD0011142 Taenia solium UNAM-cd1_adult Taenia ... 272 3e-73

Query= TSBH.R75.esd 459 0 459 ESD GOOD: 94-455

Length=362

Score E

Sequences producing significant alignments: (Bits) Value

gb|EL744368.1| LV0271050 Taenia solium UNAM-cd2_larva Taenia ... 198 5e-51

gb|EL741673.1| LV0230020 Taenia solium UNAM-cd2_larva Taenia ... 191 9e-49

Query= TSBU.R43.esd 786 0 786 ESD GOOD: 96-433

Length=338

Score E

Sequences producing significant alignments: (Bits) Value

gb|EL746816.1| LV0314029 Taenia solium UNAM-cd2_larva Taenia ... 603 1e-172

gb|EL745884.1| LV0298041 Taenia solium UNAM-cd2_larva Taenia ... 287 1e-77

Query= TSBW.R80.esd 757 0 757 ESD GOOD: 97-572

Length=476

Score E

Sequences producing significant alignments: (Bits) Value

gb|EL742306.1| LV0240001 Taenia solium UNAM-cd2_larva Taenia ... 819 0.0

gb|EL742049.1| LV0235038 Taenia solium UNAM-cd2_larva Taenia ... 813 0.0

gb|EL752713.1| AD0014254 Taenia solium UNAM-cd1_adult Taenia ... 809 0.0

gb|EL758326.1| AD0104011 Taenia solium UNAM-cd1_adult Taenia ... 787 0.0

gb|EL762470.1| AD0186034 Taenia solium UNAM-cd1_adult Taenia ... 503 1e-142

gb|EL752938.1| AD0015197 Taenia solium UNAM-cd1_adult Taenia ... 167 2e-41

Query= TSBN.R18.esd 622 0 622 ESD GOOD: 98-372

Length=275

Score E

Sequences producing significant alignments: (Bits) Value

gb|EL745477.1| LV0292011 Taenia solium UNAM-cd2_larva Taenia ... 294 7e-80

gb|EL747089.1| LV0318021 Taenia solium UNAM-cd2_larva Taenia ... 244 7e-65

Query= TSAC.R2.esd 575 0 575 ESD GOOD: 99-575

Length=477

Score E

Sequences producing significant alignments: (Bits) Value

gb|EL747225.1| LV0320022 Taenia solium UNAM-cd2_larva Taenia ... 193 3e-49

Query= TSAN.R50.esd 720 0 720 ESD GOOD: 101-402

Length=302

Score E

Sequences producing significant alignments: (Bits) Value

gb|EL746545.1| LV0310042 Taenia solium UNAM-cd2_larva Taenia ... 484 4e-137

gb|EL742605.1| LV0244036 Taenia solium UNAM-cd2_larva Taenia ... 484 4e-137

gb|EL740287.1| LV0206019 Taenia solium UNAM-cd2_larva Taenia ... 484 4e-137

gb|EL751290.1| AD0009028 Taenia solium UNAM-cd1_adult Taenia ... 479 2e-135

gb|EL740878.1| LV0217061 Taenia solium UNAM-cd2_larva Taenia ... 84.2 2e-16

Query= TSBA.R22.esd 652 0 652 ESD GOOD: 102-615

Length=514

Score E

Sequences producing significant alignments: (Bits) Value

gb|EL759159.1| AD0117062 Taenia solium UNAM-cd1_adult Taenia ... 627 6e-180

Query= TSBG.R13.esd 673 0 673 ESD GOOD: 112-267

Length=156

Score E

Sequences producing significant alignments: (Bits) Value

gb|EL745326.1| LV0289038 Taenia solium UNAM-cd2_larva Taenia ... 154 1e-37

gb|EL757965.1| AD0098050 Taenia solium UNAM-cd1_adult Taenia ... 150 2e-36

gb|EL762737.1| AD0192035 Taenia solium UNAM-cd1_adult Taenia ... 141 9e-34

gb|EL761508.1| AD0165006 Taenia solium UNAM-cd1_adult Taenia ... 134 2e-31

Query= TSBP.R95.esd 683 0 683 ESD GOOD: 100-369

Length=270

Score E

Sequences producing significant alignments: (Bits) Value

gb|EL746220.1| LV0305004 Taenia solium UNAM-cd2_larva Taenia ... 388 3e-108

Query= TSBY.R45.esd 749 0 749 ESD GOOD: 103-613

Length=511

Score E

Sequences producing significant alignments: (Bits) Value

gb|EL741699.1| LV0230046 Taenia solium UNAM-cd2_larva Taenia ... 501 4e-142

Query= TSAI.R14.esd 411 0 411 ESD GOOD: 115-411

Length=297

Score E

Sequences producing significant alignments: (Bits) Value

gb|EL745476.1| LV0292010 Taenia solium UNAM-cd2_larva Taenia ... 241 9e-64

gb|EL758822.1| AD0112021 Taenia solium UNAM-cd1_adult Taenia ... 159 3e-39

Query= TSAY.R83.esd 514 0 514 ESD GOOD: 101-452

Length=352

Score E

Sequences producing significant alignments: (Bits) Value

gb|EL755931.1| AD0027227 Taenia solium UNAM-cd1_adult Taenia ... 625 2e-179

gb|EL754235.1| AD0021025 Taenia solium UNAM-cd1_adult Taenia ... 558 2e-159

gb|EL745094.1| LV0286010 Taenia solium UNAM-cd2_larva Taenia ... 472 3e-133

gb|EL747430.1| LV0323039 Taenia solium UNAM-cd2_larva Taenia ... 318 4e-87

gb|EL749307.1| LV0358018 Taenia solium UNAM-cd2_larva Taenia ... 291 9e-79

gb|EL761095.1| AD0156035 Taenia solium UNAM-cd1_adult Taenia ... 228 7e-60

Query= TSBH.R47.esd 563 0 563 ESD GOOD: 99-532

Length=434

Score E

Sequences producing significant alignments: (Bits) Value

gb|EL742247.1| LV0238066 Taenia solium UNAM-cd2_larva Taenia ... 510 6e-145

Query= TSAK.R2.esd 530 0 530 ESD GOOD: 99-432

Length=334

Score E

Sequences producing significant alignments: (Bits) Value

gb|EL746575.1| LV0311004 Taenia solium UNAM-cd2_larva Taenia ... 536 1e-152

Query= TSBL.R46.esd 619 0 619 ESD GOOD: 94-333

Length=240

Score E

Sequences producing significant alignments: (Bits) Value

gb|EL742240.1| LV0238059 Taenia solium UNAM-cd2_larva Taenia ... 387 1e-107

Query= TSAA.R14.esd 646 0 646 ESD GOOD: 100-466

Length=367

Score E

Sequences producing significant alignments: (Bits) Value

gb|EL758476.1| AD0106019 Taenia solium UNAM-cd1_adult Taenia ... 616 1e-176

gb|EL750822.1| AD0007058 Taenia solium UNAM-cd1_adult Taenia ... 616 1e-176

gb|EL743723.1| LV0261032 Taenia solium UNAM-cd2_larva Taenia ... 616 1e-176

gb|EL743639.1| LV0260027 Taenia solium UNAM-cd2_larva Taenia ... 616 1e-176

gb|EL740292.1| LV0206024 Taenia solium UNAM-cd2_larva Taenia ... 616 1e-176

gb|EL742681.1| LV0245036 Taenia solium UNAM-cd2_larva Taenia ... 612 2e-175

gb|EL761577.1| AD0166018 Taenia solium UNAM-cd1_adult Taenia ... 610 6e-175

gb|EL757635.1| AD0035228 Taenia solium UNAM-cd1_adult Taenia ... 610 6e-175

gb|EL740766.1| LV0215056 Taenia solium UNAM-cd2_larva Taenia ... 610 6e-175

gb|EL763446.1| AD0204009 Taenia solium UNAM-cd1_adult Taenia ... 603 1e-172

Query= TSAV.R33.esd 766 0 766 ESD GOOD: 101-636

Length=536

Score E

Sequences producing significant alignments: (Bits) Value

gb|EL753933.1| AD0019220 Taenia solium UNAM-cd1_adult Taenia ... 926 0.0

Query= TSAS.R81.esd 525 0 525 ESD GOOD: 94-456

Length=363

Score E

Sequences producing significant alignments: (Bits) Value

gb|EL742242.1| LV0238061 Taenia solium UNAM-cd2_larva Taenia ... 597 5e-171

gb|EL746807.1| LV0314020 Taenia solium UNAM-cd2_larva Taenia ... 593 6e-170

gb|EL743635.1| LV0260023 Taenia solium UNAM-cd2_larva Taenia ... 431 5e-121

Query= TSAH.R47.esd 623 0 623 ESD GOOD: 97-573

Length=477

Score E

Sequences producing significant alignments: (Bits) Value

gb|EL746394.1| LV0308044 Taenia solium UNAM-cd2_larva Taenia ... 780 0.0

Query= TSCG.R36.esd 694 0 694 ESD GOOD: 106-587

Length=482

Score E

Sequences producing significant alignments: (Bits) Value

gb|GT226930.1| tscaa0_000908.z1.scf Taenia solium adult full-... 691 0.0

Query= TSCF.R96.esd 372 0 372 ESD GOOD: 94-372

Length=279

Score E

Sequences producing significant alignments: (Bits) Value

gb|EL743355.1| LV0256016 Taenia solium UNAM-cd2_larva Taenia ... 449 1e-126

Query= TSBB.R23.esd 518 0 518 ESD GOOD: 103-518

Length=416

Score E

Sequences producing significant alignments: (Bits) Value

gb|EL757724.1| AD0094064 Taenia solium UNAM-cd1_adult Taenia ... 673 0.0

gb|EL748698.1| LV0344021 Taenia solium UNAM-cd2_larva Taenia ... 667 0.0

gb|EL740786.1| LV0216020 Taenia solium UNAM-cd2_larva Taenia ... 510 6e-145

gb|EL757568.1| AD0035161 Taenia solium UNAM-cd1_adult Taenia ... 496 2e-140

gb|EL749585.1| AD0001208 Taenia solium UNAM-cd1_adult Taenia ... 496 2e-140

gb|EL760495.1| AD0143037 Taenia solium UNAM-cd1_adult Taenia ... 494 6e-140

gb|EL760253.1| AD0138031 Taenia solium UNAM-cd1_adult Taenia ... 494 6e-140

gb|EL759913.1| AD0132004 Taenia solium UNAM-cd1_adult Taenia ... 494 6e-140

gb|EL759850.1| AD0130040 Taenia solium UNAM-cd1_adult Taenia ... 494 6e-140

gb|EL759822.1| AD0130012 Taenia solium UNAM-cd1_adult Taenia ... 494 6e-140

Query= TSAM.R37.esd 508 0 508 ESD GOOD: 94-377

Length=284

Score E

Sequences producing significant alignments: (Bits) Value

gb|EL741247.1| LV0223025 Taenia solium UNAM-cd2_larva Taenia ... 477 6e-135

gb|EL740874.1| LV0217057 Taenia solium UNAM-cd2_larva Taenia ... 477 6e-135

gb|EL753404.1| AD0017162 Taenia solium UNAM-cd1_adult Taenia ... 385 4e-107

gb|EL744882.1| LV0282008 Taenia solium UNAM-cd2_larva Taenia ... 167 2e-41

Query= TSAL.R57.esd 647 0 647 ESD GOOD: 135-448

Length=314

Score E

Sequences producing significant alignments: (Bits) Value

gb|EL755725.1| AD0027021 Taenia solium UNAM-cd1_adult Taenia ... 544 6e-155

gb|EL740780.1| LV0216014 Taenia solium UNAM-cd2_larva Taenia ... 544 6e-155

gb|EL750562.1| AD0006001 Taenia solium UNAM-cd1_adult Taenia ... 521 3e-148

gb|EL754400.1| AD0021190 Taenia solium UNAM-cd1_adult Taenia ... 501 4e-142

gb|EL758113.1| AD0100058 Taenia solium UNAM-cd1_adult Taenia ... 368 4e-102

gb|EL757834.1| AD0096037 Taenia solium UNAM-cd1_adult Taenia ... 368 4e-102

gb|EL759020.1| AD0115045 Taenia solium UNAM-cd1_adult Taenia ... 318 4e-87

gb|EL758521.1| AD0107014 Taenia solium UNAM-cd1_adult Taenia ... 318 4e-87

gb|EL761465.1| AD0164021 Taenia solium UNAM-cd1_adult Taenia ... 252 4e-67

gb|EL761398.1| AD0163007 Taenia solium UNAM-cd1_adult Taenia ... 243 2e-64

Query= TSAZ.R63.esd 708 0 708 ESD GOOD: 102-411

Length=310

Score E

Sequences producing significant alignments: (Bits) Value

gb|GT227601.1| tscaa0_001733.z1.scf Taenia solium adult full-... 446 2e-125

Query= TSBO.R35.esd 896 0 896 ESD GOOD: 111-613

Length=503

Score E

Sequences producing significant alignments: (Bits) Value

gb|EL742912.1| LV0249027 Taenia solium UNAM-cd2_larva Taenia ... 782 0.0

gb|GT227081.1| tscaa0_002123.z1.scf Taenia solium adult full-... 652 0.0

gb|EL762149.1| AD0179042 Taenia solium UNAM-cd1_adult Taenia ... 433 1e-121

gb|EL763458.1| AD0204021 Taenia solium UNAM-cd1_adult Taenia ... 161 7e-40

gb|EL762901.1| AD0195021 Taenia solium UNAM-cd1_adult Taenia ... 161 7e-40

gb|EL759092.1| AD0116055 Taenia solium UNAM-cd1_adult Taenia ... 161 7e-40

gb|EL758303.1| AD0103047 Taenia solium UNAM-cd1_adult Taenia ... 161 7e-40

gb|EL758063.1| AD0100008 Taenia solium UNAM-cd1_adult Taenia ... 161 7e-40

gb|EL757343.1| AD0034159 Taenia solium UNAM-cd1_adult Taenia ... 161 7e-40

gb|EL756449.1| AD0029244 Taenia solium UNAM-cd1_adult Taenia ... 161 7e-40

Query= TSAF.R45.esd 781 0 781 ESD GOOD: 141-657

Length=517

Score E

Sequences producing significant alignments: (Bits) Value

gb|EL763087.1| AD0198025 Taenia solium UNAM-cd1_adult Taenia ... 462 2e-130

gb|EL751623.1| AD0010116 Taenia solium UNAM-cd1_adult Taenia ... 462 2e-130

gb|EL751624.1| AD0010117 Taenia solium UNAM-cd1_adult Taenia ... 438 3e-123

gb|EL763088.1| AD0198026 Taenia solium UNAM-cd1_adult Taenia ... 329 2e-90

Query= TSBQ.R4.esd 706 0 706 ESD GOOD: 85-364

Length=280

Score E

Sequences producing significant alignments: (Bits) Value

gb|EL741854.1| LV0232059 Taenia solium UNAM-cd2_larva Taenia ... 54.7 1e-07

gb|EL746752.1| LV0313035 Taenia solium UNAM-cd2_larva Taenia ... 52.8 4e-07

Query= TSAH.R11.esd 692 0 692 ESD GOOD: 103-570

Length=468

Score E

Sequences producing significant alignments: (Bits) Value

gb|EL745523.1| LV0293001 Taenia solium UNAM-cd2_larva Taenia ... 529 2e-150

gb|GT227189.1| tscaa0_000402.z1.scf Taenia solium adult full-... 69.4 4e-12

Query= TSCB.R15.esd 580 0 580 ESD GOOD: 103-324

Length=222

Score E

Sequences producing significant alignments: (Bits) Value

gb|GT227105.1| tscaa0_000807.z1.scf Taenia solium adult full-... 340 9e-94

gb|EL749358.1| LV0359036 Taenia solium UNAM-cd2_larva Taenia ... 298 5e-81

gb|EL741042.1| LV0220015 Taenia solium UNAM-cd2_larva Taenia ... 298 5e-81

Query= TSBU.R21.esd 736 0 736 ESD GOOD: 106-621

Length=516

Score E

Sequences producing significant alignments: (Bits) Value

gb|EL742966.1| LV0250007 Taenia solium UNAM-cd2_larva Taenia ... 885 0.0

gb|EL763255.1| AD0201018 Taenia solium UNAM-cd1_adult Taenia ... 737 0.0

gb|EL748133.1| LV0334016 Taenia solium UNAM-cd2_larva Taenia ... 617 4e-177

gb|EL763306.1| AD0202006 Taenia solium UNAM-cd1_adult Taenia ... 569 1e-162

Query= TSAV.R86.esd 614 0 614 ESD GOOD: 106-613

Length=508

Score E

Sequences producing significant alignments: (Bits) Value

gb|EL743004.1| LV0250045 Taenia solium UNAM-cd2_larva Taenia ... 520 1e-147

gb|EL747960.1| LV0331053 Taenia solium UNAM-cd2_larva Taenia ... 353 1e-97

Query= TSAZ.R75.esd 533 0 533 ESD GOOD: 99-512

Length=414

Score E

Sequences producing significant alignments: (Bits) Value

gb|EL761309.1| AD0161026 Taenia solium UNAM-cd1_adult Taenia ... 385 4e-107

gb|EL745750.1| LV0296040 Taenia solium UNAM-cd2_larva Taenia ... 355 3e-98

Query= TSCA.R55.esd 649 0 649 ESD GOOD: 89-474

Length=386

Score E

Sequences producing significant alignments: (Bits) Value

gb|EL760261.1| AD0138039 Taenia solium UNAM-cd1_adult Taenia ... 669 0.0

gb|EL757598.1| AD0035191 Taenia solium UNAM-cd1_adult Taenia ... 669 0.0

gb|EL744644.1| LV0277021 Taenia solium UNAM-cd2_larva Taenia ... 669 0.0

gb|EL757908.1| AD0097051 Taenia solium UNAM-cd1_adult Taenia ... 660 0.0

gb|EL761301.1| AD0161018 Taenia solium UNAM-cd1_adult Taenia ... 654 0.0

gb|EL743116.1| LV0252017 Taenia solium UNAM-cd2_larva Taenia ... 652 0.0

gb|EL762497.1| AD0187016 Taenia solium UNAM-cd1_adult Taenia ... 651 0.0

gb|EL751896.1| AD0011139 Taenia solium UNAM-cd1_adult Taenia ... 647 0.0

gb|EL761452.1| AD0164008 Taenia solium UNAM-cd1_adult Taenia ... 643 0.0

gb|EL751816.1| AD0011059 Taenia solium UNAM-cd1_adult Taenia ... 638 0.0

Query= TSAF.R26.esd 412 0 412 ESD GOOD: 100-412

Length=313

Score E

Sequences producing significant alignments: (Bits) Value

gb|EL747314.1| LV0321045 Taenia solium UNAM-cd2_larva Taenia ... 556 8e-159

gb|EL758247.1| AD0102058 Taenia solium UNAM-cd1_adult Taenia ... 503 1e-142

gb|EL749154.1| LV0354013 Taenia solium UNAM-cd2_larva Taenia ... 394 6e-110

gb|EL741962.1| LV0234023 Taenia solium UNAM-cd2_larva Taenia ... 302 4e-82

gb|EL757713.1| AD0094053 Taenia solium UNAM-cd1_adult Taenia ... 292 2e-79

gb|EL748830.1| LV0346020 Taenia solium UNAM-cd2_larva Taenia ... 276 2e-74

Query= TSCG.R33.esd 564 0 564 ESD GOOD: 97-564

Length=468

Score E

Sequences producing significant alignments: (Bits) Value

gb|EL742048.1| LV0235037 Taenia solium UNAM-cd2_larva Taenia ... 841 0.0

gb|EL744579.1| LV0275054 Taenia solium UNAM-cd2_larva Taenia ... 320 1e-87

gb|EL747375.1| LV0322052 Taenia solium UNAM-cd2_larva Taenia ... 292 2e-79

Query= TSAJ.R93.esd 685 0 685 ESD GOOD: 95-396

Length=302

Score E

Sequences producing significant alignments: (Bits) Value

gb|EL758639.1| AD0109014 Taenia solium UNAM-cd1_adult Taenia ... 276 2e-74

Query= TSBI.R83.esd 711 0 711 ESD GOOD: 104-461

Length=358

Score E

Sequences producing significant alignments: (Bits) Value

gb|EL757853.1| AD0096056 Taenia solium UNAM-cd1_adult Taenia ... 204 1e-52

Query= TSAL.R85.esd 701 0 701 ESD GOOD: 107-558

Length=452

Score E

Sequences producing significant alignments: (Bits) Value

gb|EL741688.1| LV0230035 Taenia solium UNAM-cd2_larva Taenia ... 289 3e-78

Query= TSBU.R68.esd 768 0 768 ESD GOOD: 111-406

Length=296

Score E

Sequences producing significant alignments: (Bits) Value

gb|EL748991.1| LV0349040 Taenia solium UNAM-cd2_larva Taenia ... 497 5e-141

Query= TSCC.R68.esd 765 0 765 ESD GOOD: 107-409

Length=303

Score E

Sequences producing significant alignments: (Bits) Value

gb|EL743459.1| LV0257076 Taenia solium UNAM-cd2_larva Taenia ... 331 5e-91

gb|EL746151.1| LV0303002 Taenia solium UNAM-cd2_larva Taenia ... 311 7e-85

gb|EL752414.1| AD0013181 Taenia solium UNAM-cd1_adult Taenia ... 303 1e-82

gb|EL743795.1| LV0262036 Taenia solium UNAM-cd2_larva Taenia ... 296 2e-80

gb|EL747087.1| LV0318019 Taenia solium UNAM-cd2_larva Taenia ... 104 1e-22

gb|EL742396.1| LV0241022 Taenia solium UNAM-cd2_larva Taenia ... 87.9 1e-17

Query= TSBB.R18.esd 523 0 523 ESD GOOD: 76-267

Length=192

Score E

Sequences producing significant alignments: (Bits) Value

gb|EL760187.1| AD0137020 Taenia solium UNAM-cd1_adult Taenia ... 248 5e-66

gb|EL754715.1| AD0022240 Taenia solium UNAM-cd1_adult Taenia ... 246 2e-65

gb|EL749726.1| AD0002090 Taenia solium UNAM-cd1_adult Taenia ... 226 3e-59

gb|EL749947.1| AD0003089 Taenia solium UNAM-cd1_adult Taenia ... 185 4e-47

gb|EL760885.1| AD0152010 Taenia solium UNAM-cd1_adult Taenia ... 174 9e-44

Query= TSAV.R61.esd 509 0 509 ESD GOOD: 114-509

Length=396

Score E

Sequences producing significant alignments: (Bits) Value

gb|EL747040.1| LV0317037 Taenia solium UNAM-cd2_larva Taenia ... 544 6e-155

Query= TSBV.R42.esd 583 0 583 ESD GOOD: 102-370

Length=269

Score E

Sequences producing significant alignments: (Bits) Value

gb|EL746552.1| LV0310049 Taenia solium UNAM-cd2_larva Taenia ... 303 1e-82

gb|EL742487.1| LV0242051 Taenia solium UNAM-cd2_larva Taenia ... 303 1e-82

gb|EL743475.1| LV0258015 Taenia solium UNAM-cd2_larva Taenia ... 302 4e-82

Query= TSAT.R84.esd 791 0 791 ESD GOOD: 105-640

Length=536

Score E

Sequences producing significant alignments: (Bits) Value

gb|EL749190.1| LV0355004 Taenia solium UNAM-cd2_larva Taenia ... 562 2e-160

gb|EL748558.1| LV0342009 Taenia solium UNAM-cd2_larva Taenia ... 161 7e-40

gb|GT227370.1| tscaa0_001035.z1.scf Taenia solium adult full-... 137 1e-32

Query= TSBT.R50.esd 553 0 553 ESD GOOD: 97-553

Length=457

Score E

Sequences producing significant alignments: (Bits) Value

gb|EL743110.1| LV0252011 Taenia solium UNAM-cd2_larva Taenia ... 261 7e-70

Query= TSBG.R21.esd 847 0 847 ESD GOOD: 105-633

Length=529

Score E

Sequences producing significant alignments: (Bits) Value

gb|EL744679.1| LV0277056 Taenia solium UNAM-cd2_larva Taenia ... 817 0.0

gb|EL742420.1| LV0241046 Taenia solium UNAM-cd2_larva Taenia ... 287 1e-77

Query= TSBV.R53.esd 456 0 456 ESD GOOD: 108-303

Length=196

Score E

Sequences producing significant alignments: (Bits) Value

gb|EL743861.1| LV0263030 Taenia solium UNAM-cd2_larva Taenia ... 298 5e-81

Query= TSAU.R92.esd 385 0 385 ESD GOOD: 90-246

Length=157

Score E

Sequences producing significant alignments: (Bits) Value

gb|EL745897.1| LV0298054 Taenia solium UNAM-cd2_larva Taenia ... 261 7e-70

Query= TSCD.R46.esd 540 0 540 ESD GOOD: 89-540

Length=452

Score E

Sequences producing significant alignments: (Bits) Value

gb|EL748232.1| LV0336008 Taenia solium UNAM-cd2_larva Taenia ... 459 2e-129

Query= TSAK.R66.esd 559 0 559 ESD GOOD: 102-535

Length=434

Score E

Sequences producing significant alignments: (Bits) Value

gb|GT227303.1| tscaa0_000822.z1.scf Taenia solium adult full-... 778 0.0

gb|EL748318.1| LV0338007 Taenia solium UNAM-cd2_larva Taenia ... 252 4e-67

gb|EL742505.1| LV0243003 Taenia solium UNAM-cd2_larva Taenia ... 209 3e-54

Query= TSBC.R28.esd 637 0 637 ESD GOOD: 99-372

Length=274

Score E

Sequences producing significant alignments: (Bits) Value

gb|GT226955.1| tscaa0_001625.z1.scf Taenia solium adult full-... 394 6e-110

gb|EL759273.1| AD0120026 Taenia solium UNAM-cd1_adult Taenia ... 270 1e-72

gb|EL758190.1| AD0102001 Taenia solium UNAM-cd1_adult Taenia ... 268 4e-72

Query= TSAA.R59.esd 696 0 696 ESD GOOD: 98-479

Length=382

Score E

Sequences producing significant alignments: (Bits) Value

gb|EL746218.1| LV0305002 Taenia solium UNAM-cd2_larva Taenia ... 669 0.0

gb|EL740632.1| LV0213014 Taenia solium UNAM-cd2_larva Taenia ... 654 0.0

Query= TSAU.R46.esd 668 0 668 ESD GOOD: 87-368

Length=282

Score E

Sequences producing significant alignments: (Bits) Value

gb|EL747384.1| LV0322061 Taenia solium UNAM-cd2_larva Taenia ... 254 1e-67

gb|EL755599.1| AD0026122 Taenia solium UNAM-cd1_adult Taenia ... 250 1e-66

gb|EL755598.1| AD0026121 Taenia solium UNAM-cd1_adult Taenia ... 115 6e-26

gb|EL756599.1| AD0030131 Taenia solium UNAM-cd1_adult Taenia ... 113 2e-25

gb|EL758473.1| AD0106016 Taenia solium UNAM-cd1_adult Taenia ... 93.5 3e-19

gb|EL740517.1| LV0210041 Taenia solium UNAM-cd2_larva Taenia ... 93.5 3e-19

Query= TSBR.R3.esd 657 0 657 ESD GOOD: 102-544

Length=443

Score E

Sequences producing significant alignments: (Bits) Value

gb|EL741890.1| LV0233023 Taenia solium UNAM-cd2_larva Taenia ... 529 2e-150

gb|EX150550.1| TSEDTS1008B09 Cysti Taenia solium cDNA, mRNA s... 67.6 2e-11

Query= TSAL.R68.esd 499 0 499 ESD GOOD: 105-499

Length=395

Score E

Sequences producing significant alignments: (Bits) Value

gb|EL741598.1| LV0229009 Taenia solium UNAM-cd2_larva Taenia ... 693 0.0

gb|EL744841.1| LV0281001 Taenia solium UNAM-cd2_larva Taenia ... 688 0.0

gb|EL741740.1| LV0231018 Taenia solium UNAM-cd2_larva Taenia ... 684 0.0

gb|EL746579.1| LV0311008 Taenia solium UNAM-cd2_larva Taenia ... 682 0.0

gb|EL744711.1| LV0278030 Taenia solium UNAM-cd2_larva Taenia ... 654 0.0

gb|EL741779.1| LV0231057 Taenia solium UNAM-cd2_larva Taenia ... 608 2e-174

gb|EL744456.1| LV0273014 Taenia solium UNAM-cd2_larva Taenia ... 604 3e-173

gb|EL741783.1| LV0231061 Taenia solium UNAM-cd2_larva Taenia ... 593 6e-170

gb|EL747754.1| LV0328042 Taenia solium UNAM-cd2_larva Taenia ... 566 1e-161

gb|EL743281.1| LV0254062 Taenia solium UNAM-cd2_larva Taenia ... 350 1e-96

Query= TSBI.R33.esd 594 0 594 ESD GOOD: 91-371

Length=281

Score E

Sequences producing significant alignments: (Bits) Value

gb|EL747824.1| LV0329062 Taenia solium UNAM-cd2_larva Taenia ... 490 8e-139

gb|EL742871.1| LV0248033 Taenia solium UNAM-cd2_larva Taenia ... 490 8e-139

Query= TSCC.R54.esd 694 0 694 ESD GOOD: 102-499

Length=398

Score E

Sequences producing significant alignments: (Bits) Value

gb|EL754337.1| AD0021127 Taenia solium UNAM-cd1_adult Taenia ... 627 6e-180

gb|EL753123.1| AD0016161 Taenia solium UNAM-cd1_adult Taenia ... 627 6e-180

gb|EL741939.1| LV0233072 Taenia solium UNAM-cd2_larva Taenia ... 606 8e-174

gb|EL755674.1| AD0026197 Taenia solium UNAM-cd1_adult Taenia ... 604 3e-173

gb|EL762824.1| AD0193071 Taenia solium UNAM-cd1_adult Taenia ... 597 5e-171

gb|EL753669.1| AD0018207 Taenia solium UNAM-cd1_adult Taenia ... 595 2e-170

gb|EL758342.1| AD0104027 Taenia solium UNAM-cd1_adult Taenia ... 593 6e-170

gb|EL751978.1| AD0012006 Taenia solium UNAM-cd1_adult Taenia ... 457 8e-129

gb|EL756262.1| AD0029057 Taenia solium UNAM-cd1_adult Taenia ... 414 5e-116

gb|EL747627.1| LV0326043 Taenia solium UNAM-cd2_larva Taenia ... 335 4e-92

Query= TSBA.R73.esd 642 0 642 ESD GOOD: 125-560

Length=436

Score E

Sequences producing significant alignments: (Bits) Value

gb|EL753493.1| AD0018031 Taenia solium UNAM-cd1_adult Taenia ... 377 7e-105

gb|EL753492.1| AD0018030 Taenia solium UNAM-cd1_adult Taenia ... 102 4e-22

Query= TSBD.R29.esd 551 0 551 ESD GOOD: 90-245

Length=156

Score E

Sequences producing significant alignments: (Bits) Value

gb|EL743382.1| LV0256043 Taenia solium UNAM-cd2_larva Taenia ... 159 3e-39

gb|EL746498.1| LV0309068 Taenia solium UNAM-cd2_larva Taenia ... 135 4e-32

gb|EL758530.1| AD0107023 Taenia solium UNAM-cd1_adult Taenia ... 115 6e-26

Query= TSBY.R5.esd 503 0 503 ESD GOOD: 97-284

Length=188

Score E

Sequences producing significant alignments: (Bits) Value

gb|EL759015.1| AD0115040 Taenia solium UNAM-cd1_adult Taenia ... 331 5e-91

gb|EL746180.1| LV0304005 Taenia solium UNAM-cd2_larva Taenia ... 331 5e-91

gb|EL741078.1| LV0220051 Taenia solium UNAM-cd2_larva Taenia ... 161 7e-40

Query= TSAY.R75.esd 547 0 547 ESD GOOD: 89-356

Length=268

Score E

Sequences producing significant alignments: (Bits) Value

gb|EL745090.1| LV0286006 Taenia solium UNAM-cd2_larva Taenia ... 451 4e-127

Query= TSAB.R72.esd 625 0 625 ESD GOOD: 94-546

Length=453

Score E

Sequences producing significant alignments: (Bits) Value

gb|EL741135.1| LV0221036 Taenia solium UNAM-cd2_larva Taenia ... 606 8e-174

gb|EL759057.1| AD0116020 Taenia solium UNAM-cd1_adult Taenia ... 429 2e-120

Query= TSBU.R3.esd 677 0 677 ESD GOOD: 92-317

Length=226

Score E

Sequences producing significant alignments: (Bits) Value

gb|EL743736.1| LV0261045 Taenia solium UNAM-cd2_larva Taenia ... 281 5e-76

gb|EL742066.1| LV0235055 Taenia solium UNAM-cd2_larva Taenia ... 279 2e-75

gb|EL744364.1| LV0271046 Taenia solium UNAM-cd2_larva Taenia ... 270 1e-72

Query= TSBC.R7.esd 623 0 623 ESD GOOD: 100-623

Length=524

Score E

Sequences producing significant alignments: (Bits) Value

gb|EL745976.1| LV0300007 Taenia solium UNAM-cd2_larva Taenia ... 126 3e-29

Query= TSBN.R27.esd 452 0 452 ESD GOOD: 78-420

Length=343

Score E

Sequences producing significant alignments: (Bits) Value

gb|EL745867.1| LV0298024 Taenia solium UNAM-cd2_larva Taenia ... 529 2e-150

gb|EL743045.1| LV0251010 Taenia solium UNAM-cd2_larva Taenia ... 507 8e-144

gb|EL748562.1| LV0342013 Taenia solium UNAM-cd2_larva Taenia ... 189 3e-48

Query= TSCA.R83.esd 709 0 709 ESD GOOD: 107-613

Length=507

Score E

Sequences producing significant alignments: (Bits) Value

gb|EL743410.1| LV0257027 Taenia solium UNAM-cd2_larva Taenia ... 732 0.0

Query= TSAA.R28.esd 654 0 654 ESD GOOD: 102-598

Length=497

Score E

Sequences producing significant alignments: (Bits) Value

gb|EL741208.1| LV0222041 Taenia solium UNAM-cd2_larva Taenia ... 532 1e-151

Query= TSBF.R1.esd 564 0 564 ESD GOOD: 94-564

Length=471

Score E

Sequences producing significant alignments: (Bits) Value

gb|EL745671.1| LV0295029 Taenia solium UNAM-cd2_larva Taenia ... 845 0.0

gb|EL740797.1| LV0216031 Taenia solium UNAM-cd2_larva Taenia ... 845 0.0

gb|EL758992.1| AD0115017 Taenia solium UNAM-cd1_adult Taenia ... 839 0.0

gb|EL758107.1| AD0100052 Taenia solium UNAM-cd1_adult Taenia ... 645 0.0

gb|EL761260.1| AD0160011 Taenia solium UNAM-cd1_adult Taenia ... 281 5e-76

Query= TSAR.R13.esd 652 0 652 ESD GOOD: 101-611

Length=511

Score E

Sequences producing significant alignments: (Bits) Value

gb|EL741210.1| LV0222043 Taenia solium UNAM-cd2_larva Taenia ... 392 2e-109

Query= TSBI.R82.esd 770 0 770 ESD GOOD: 104-575

Length=472

Score E

Sequences producing significant alignments: (Bits) Value

gb|EL745811.1| LV0297040 Taenia solium UNAM-cd2_larva Taenia ... 614 5e-176

gb|EL748523.1| LV0341039 Taenia solium UNAM-cd2_larva Taenia ... 490 8e-139

gb|EL742708.1| LV0245063 Taenia solium UNAM-cd2_larva Taenia ... 76.8 3e-14

Query= TSAO.R3.esd 705 0 705 ESD GOOD: 105-591

Length=487

Score E

Sequences producing significant alignments: (Bits) Value

gb|EL743772.1| LV0262013 Taenia solium UNAM-cd2_larva Taenia ... 571 3e-163

Query= TSBW.R79.esd 588 0 588 ESD GOOD: 91-588

Length=498

Score E

Sequences producing significant alignments: (Bits) Value

gb|EL762284.1| AD0182040 Taenia solium UNAM-cd1_adult Taenia ... 689 0.0

gb|EL741961.1| LV0234022 Taenia solium UNAM-cd2_larva Taenia ... 368 4e-102

Query= TSBD.R1.esd 539 0 539 ESD GOOD: 94-539

Length=446

Score E

Sequences producing significant alignments: (Bits) Value

gb|EL762725.1| AD0192023 Taenia solium UNAM-cd1_adult Taenia ... 654 0.0

Query= TSAU.R27.esd 672 0 672 ESD GOOD: 95-314

Length=220

Score E

Sequences producing significant alignments: (Bits) Value

gb|EL740254.1| LV0205034 Taenia solium UNAM-cd2_larva Taenia ... 300 1e-81

Query= TSBT.R36.esd 552 0 552 ESD GOOD: 93-510

Length=418

Score E

Sequences producing significant alignments: (Bits) Value

gb|EL745184.1| LV0287038 Taenia solium UNAM-cd2_larva Taenia ... 418 4e-117

gb|EX150552.1| TSEDTS1008G06 Cysti Taenia solium cDNA, mRNA s... 141 9e-34

Query= TSBI.R42.esd 637 0 637 ESD GOOD: 109-447

Length=339

Score E

Sequences producing significant alignments: (Bits) Value

gb|EL746289.1| LV0307003 Taenia solium UNAM-cd2_larva Taenia ... 592 2e-169

gb|EL753437.1| AD0017195 Taenia solium UNAM-cd1_adult Taenia ... 353 1e-97

gb|EL750989.1| AD0007225 Taenia solium UNAM-cd1_adult Taenia ... 353 1e-97

gb|EL749863.1| AD0003005 Taenia solium UNAM-cd1_adult Taenia ... 353 1e-97

gb|EL762007.1| AD0176013 Taenia solium UNAM-cd1_adult Taenia ... 351 4e-97

gb|EL758270.1| AD0103014 Taenia solium UNAM-cd1_adult Taenia ... 348 5e-96

gb|EL753212.1| AD0016250 Taenia solium UNAM-cd1_adult Taenia ... 340 9e-94

gb|EL749642.1| AD0002006 Taenia solium UNAM-cd1_adult Taenia ... 307 9e-84

Query= TSAA.R85.esd 718 0 718 ESD GOOD: 108-602

Length=495

Score E

Sequences producing significant alignments: (Bits) Value

gb|GT227102.1| tscaa0_000437.z1.scf Taenia solium adult full-... 857 0.0

gb|EL760106.1| AD0135052 Taenia solium UNAM-cd1_adult Taenia ... 451 4e-127

gb|EL759475.1| AD0123054 Taenia solium UNAM-cd1_adult Taenia ... 451 4e-127

gb|EL757947.1| AD0098032 Taenia solium UNAM-cd1_adult Taenia ... 451 4e-127

gb|EL756448.1| AD0029243 Taenia solium UNAM-cd1_adult Taenia ... 451 4e-127

gb|EL752150.1| AD0012178 Taenia solium UNAM-cd1_adult Taenia ... 451 4e-127

gb|EL752093.1| AD0012121 Taenia solium UNAM-cd1_adult Taenia ... 449 1e-126

gb|EL757338.1| AD0034154 Taenia solium UNAM-cd1_adult Taenia ... 448 5e-126

gb|EL754866.1| AD0023122 Taenia solium UNAM-cd1_adult Taenia ... 448 5e-126

gb|EL751026.1| AD0008037 Taenia solium UNAM-cd1_adult Taenia ... 448 5e-126

Query= TSCH.R25.esd 611 0 611 ESD GOOD: 97-480

Length=384

Score E

Sequences producing significant alignments: (Bits) Value

gb|EL744641.1| LV0277018 Taenia solium UNAM-cd2_larva Taenia ... 649 0.0

Query= TSCB.R62.esd 683 0 683 ESD GOOD: 105-641

Length=537

Score E

Sequences producing significant alignments: (Bits) Value

gb|EL757702.1| AD0094042 Taenia solium UNAM-cd1_adult Taenia ... 510 6e-145

Query= TSAV.R51.esd 625 0 625 ESD GOOD: 106-378

Length=273

Score E

Sequences producing significant alignments: (Bits) Value

gb|EL741035.1| LV0220008 Taenia solium UNAM-cd2_larva Taenia ... 357 8e-99

gb|EL740702.1| LV0214027 Taenia solium UNAM-cd2_larva Taenia ... 351 4e-97

gb|EL752882.1| AD0015141 Taenia solium UNAM-cd1_adult Taenia ... 324 9e-89

gb|EL762335.1| AD0183036 Taenia solium UNAM-cd1_adult Taenia ... 294 7e-80

gb|EL751830.1| AD0011073 Taenia solium UNAM-cd1_adult Taenia ... 291 9e-79

gb|EL761352.1| AD0162013 Taenia solium UNAM-cd1_adult Taenia ... 270 1e-72

Query= TSBA.R20.esd 553 0 553 ESD GOOD: 39-552

Length=514

Score E

Sequences producing significant alignments: (Bits) Value

gb|EL744558.1| LV0275033 Taenia solium UNAM-cd2_larva Taenia ... 521 3e-148

gb|EL749088.1| LV0352043 Taenia solium UNAM-cd2_larva Taenia ... 512 2e-145

gb|EL748684.1| LV0344007 Taenia solium UNAM-cd2_larva Taenia ... 473 8e-134

gb|EL752247.1| AD0013014 Taenia solium UNAM-cd1_adult Taenia ... 407 8e-114

gb|EL750476.1| AD0005139 Taenia solium UNAM-cd1_adult Taenia ... 407 8e-114

gb|EL750234.1| AD0004149 Taenia solium UNAM-cd1_adult Taenia ... 407 8e-114

gb|EL751988.1| AD0012016 Taenia solium UNAM-cd1_adult Taenia ... 357 8e-99

gb|EL752720.1| AD0014261 Taenia solium UNAM-cd1_adult Taenia ... 350 1e-96

gb|EL761055.1| AD0155043 Taenia solium UNAM-cd1_adult Taenia ... 267 1e-71

Query= TSBG.R77.esd 699 0 699 ESD GOOD: 139-419

Length=281

Score E

Sequences producing significant alignments: (Bits) Value

gb|EL749344.1| LV0359022 Taenia solium UNAM-cd2_larva Taenia ... 372 3e-103

gb|EL743291.1| LV0255005 Taenia solium UNAM-cd2_larva Taenia ... 372 3e-103

gb|EL744132.1| LV0267071 Taenia solium UNAM-cd2_larva Taenia ... 320 1e-87

Query= TSBM.R85.esd 497 0 497 ESD GOOD: 105-497

Length=393

Score E

Sequences producing significant alignments: (Bits) Value

gb|EL758938.1| AD0114008 Taenia solium UNAM-cd1_adult Taenia ... 165 6e-41

gb|EL753474.1| AD0018012 Taenia solium UNAM-cd1_adult Taenia ... 158 9e-39

Query= TSAH.R46.esd 602 0 602 ESD GOOD: 98-584

Length=487

Score E

Sequences producing significant alignments: (Bits) Value

gb|EL763461.1| AD0204024 Taenia solium UNAM-cd1_adult Taenia ... 542 2e-154

gb|GT227274.1| tscaa0_000641.z1.scf Taenia solium adult full-... 536 1e-152

Query= TSCD.R94.esd 603 0 603 ESD GOOD: 89-532

Length=444

Score E

Sequences producing significant alignments: (Bits) Value

gb|EL744940.1| LV0283004 Taenia solium UNAM-cd2_larva Taenia ... 582 1e-166

gb|EL747812.1| LV0329050 Taenia solium UNAM-cd2_larva Taenia ... 573 8e-164

gb|EL747776.1| LV0329014 Taenia solium UNAM-cd2_larva Taenia ... 499 1e-141

gb|EL744018.1| LV0266018 Taenia solium UNAM-cd2_larva Taenia ... 302 4e-82

gb|EL741546.1| LV0228028 Taenia solium UNAM-cd2_larva Taenia ... 62.1 7e-10

Query= TSAF.R29.esd 659 0 659 ESD GOOD: 98-470

Length=373

Score E

Sequences producing significant alignments: (Bits) Value

gb|EL743990.1| LV0265062 Taenia solium UNAM-cd2_larva Taenia ... 143 3e-34

Query= TSAZ.R60.esd 710 0 710 ESD GOOD: 115-615

Length=501

Score E

Sequences producing significant alignments: (Bits) Value

gb|EL747719.1| LV0328007 Taenia solium UNAM-cd2_larva Taenia ... 422 3e-118

Query= TSCD.R43.esd 654 0 654 ESD GOOD: 100-389

Length=290

Score E

Sequences producing significant alignments: (Bits) Value

gb|EL761059.1| AD0155047 Taenia solium UNAM-cd1_adult Taenia ... 134 2e-31

Query= TSAT.R7.esd 689 0 689 ESD GOOD: 105-592

Length=488

Score E

Sequences producing significant alignments: (Bits) Value

gb|EL752318.1| AD0013085 Taenia solium UNAM-cd1_adult Taenia ... 725 0.0

gb|EL762606.1| AD0190008 Taenia solium UNAM-cd1_adult Taenia ... 311 7e-85

Query= TSBC.R58.esd 587 0 587 ESD GOOD: 70-520

Length=451

Score E

Sequences producing significant alignments: (Bits) Value

gb|EL747036.1| LV0317033 Taenia solium UNAM-cd2_larva Taenia ... 625 2e-179

gb|EL746596.1| LV0311025 Taenia solium UNAM-cd2_larva Taenia ... 374 8e-104

Query= TSBQ.R56.esd 808 0 808 ESD GOOD: 95-620

Length=526

Score E

Sequences producing significant alignments: (Bits) Value

gb|EL742546.1| LV0243044 Taenia solium UNAM-cd2_larva Taenia ... 601 4e-172

gb|EL744913.1| LV0282039 Taenia solium UNAM-cd2_larva Taenia ... 540 8e-154

gb|EL740238.1| LV0205018 Taenia solium UNAM-cd2_larva Taenia ... 405 3e-113

gb|EL740519.1| LV0210043 Taenia solium UNAM-cd2_larva Taenia ... 267 1e-71

Query= TSAT.R9.esd 648 0 648 ESD GOOD: 105-648

Length=544

Score E

Sequences producing significant alignments: (Bits) Value

gb|EL760069.1| AD0135015 Taenia solium UNAM-cd1_adult Taenia ... 902 0.0

Query= TSAO.R47.esd 712 0 712 ESD GOOD: 103-629

Length=527

Score E

Sequences producing significant alignments: (Bits) Value

gb|EL762803.1| AD0193050 Taenia solium UNAM-cd1_adult Taenia ... 305 3e-83

gb|EL761057.1| AD0155045 Taenia solium UNAM-cd1_adult Taenia ... 305 3e-83

gb|EL753291.1| AD0017049 Taenia solium UNAM-cd1_adult Taenia ... 305 3e-83

gb|EL753025.1| AD0016063 Taenia solium UNAM-cd1_adult Taenia ... 305 3e-83

gb|EL762419.1| AD0185024 Taenia solium UNAM-cd1_adult Taenia ... 263 2e-70

Query= TSAJ.R89.esd 676 0 676 ESD GOOD: 96-241

Length=146

Score E

Sequences producing significant alignments: (Bits) Value

gb|GT227715.1| tscaa0_002144.z1.scf Taenia solium adult full-... 161 7e-40

gb|EL747128.1| LV0318060 Taenia solium UNAM-cd2_larva Taenia ... 161 7e-40

Query= TSBQ.R51.esd 798 0 798 ESD GOOD: 99-278

Length=180

Score E

Sequences producing significant alignments: (Bits) Value

gb|EL752334.1| AD0013101 Taenia solium UNAM-cd1_adult Taenia ... 220 1e-57

Query= TSAB.R80.esd 722 0 722 ESD GOOD: 96-583

Length=488

Score E

Sequences producing significant alignments: (Bits) Value

gb|EL741399.1| LV0226018 Taenia solium UNAM-cd2_larva Taenia ... 108 9e-24

Query= TSBF.R44.esd 650 0 650 ESD GOOD: 94-598

Length=505

Score E

Sequences producing significant alignments: (Bits) Value

gb|EL753771.1| AD0019058 Taenia solium UNAM-cd1_adult Taenia ... 689 0.0

gb|EL748536.1| LV0341052 Taenia solium UNAM-cd2_larva Taenia ... 431 5e-121

gb|EL755558.1| AD0026081 Taenia solium UNAM-cd1_adult Taenia ... 411 6e-115

gb|EL757715.1| AD0094055 Taenia solium UNAM-cd1_adult Taenia ... 409 2e-114

gb|EL742337.1| LV0240032 Taenia solium UNAM-cd2_larva Taenia ... 361 7e-100

gb|EL761166.1| AD0158008 Taenia solium UNAM-cd1_adult Taenia ... 252 4e-67

gb|EL745114.1| LV0286031 Taenia solium UNAM-cd2_larva Taenia ... 180 2e-45

gb|EL744755.1| LV0279024 Taenia solium UNAM-cd2_larva Taenia ... 124 9e-29

gb|EL741282.1| LV0224002 Taenia solium UNAM-cd2_larva Taenia ... 106 3e-23

Query= TSAW.R55.esd 503 0 503 ESD GOOD: 70-503

Length=434

Score E

Sequences producing significant alignments: (Bits) Value

gb|EL745742.1| LV0296032 Taenia solium UNAM-cd2_larva Taenia ... 113 2e-25

Query= TSAI.R47.esd 706 0 706 ESD GOOD: 97-613

Length=517

Score E

Sequences producing significant alignments: (Bits) Value

gb|EL759061.1| AD0116024 Taenia solium UNAM-cd1_adult Taenia ... 928 0.0

Query= TSAS.R87.esd 656 0 656 ESD GOOD: 97-255

Length=159

Score E

Sequences producing significant alignments: (Bits) Value

gb|EL752120.1| AD0012148 Taenia solium UNAM-cd1_adult Taenia ... 243 2e-64

Query= TSCE.R49.esd 583 0 583 ESD GOOD: 98-581

Length=484

Score E

Sequences producing significant alignments: (Bits) Value

gb|EL741026.1| LV0219076 Taenia solium UNAM-cd2_larva Taenia ... 712 0.0

gb|EL747039.1| LV0317036 Taenia solium UNAM-cd2_larva Taenia ... 440 8e-124

Query= TSBL.R19.esd 506 0 506 ESD GOOD: 105-502

Length=398

Score E

Sequences producing significant alignments: (Bits) Value

gb|EL761675.1| AD0168035 Taenia solium UNAM-cd1_adult Taenia ... 715 0.0

gb|EL761214.1| AD0159011 Taenia solium UNAM-cd1_adult Taenia ... 649 0.0

gb|EL761778.1| AD0170049 Taenia solium UNAM-cd1_adult Taenia ... 643 0.0

gb|EL752424.1| AD0013191 Taenia solium UNAM-cd1_adult Taenia ... 643 0.0

gb|EL761030.1| AD0155018 Taenia solium UNAM-cd1_adult Taenia ... 638 0.0

gb|EL758913.1| AD0113054 Taenia solium UNAM-cd1_adult Taenia ... 512 2e-145

gb|EL755695.1| AD0026218 Taenia solium UNAM-cd1_adult Taenia ... 438 3e-123

gb|EL746797.1| LV0314010 Taenia solium UNAM-cd2_larva Taenia ... 438 3e-123

gb|EL744863.1| LV0281023 Taenia solium UNAM-cd2_larva Taenia ... 438 3e-123

gb|EL762938.1| AD0195058 Taenia solium UNAM-cd1_adult Taenia ... 436 1e-122

Query= TSAW.R12.esd 580 0 580 ESD GOOD: 70-537

Length=468

Score E

Sequences producing significant alignments: (Bits) Value

gb|EL760764.1| AD0148042 Taenia solium UNAM-cd1_adult Taenia ... 440 8e-124

gb|EL760146.1| AD0136037 Taenia solium UNAM-cd1_adult Taenia ... 440 8e-124

gb|EL758801.1| AD0111066 Taenia solium UNAM-cd1_adult Taenia ... 198 5e-51

Query= TSCB.R30.esd 520 0 520 ESD GOOD: 117-467

Length=351

Score E

Sequences producing significant alignments: (Bits) Value

gb|EL748109.1| LV0333060 Taenia solium UNAM-cd2_larva Taenia ... 638 0.0

Query= TSBS.R88.esd 607 0 607 ESD GOOD: 96-343

Length=248

Score E

Sequences producing significant alignments: (Bits) Value

gb|EL758939.1| AD0114009 Taenia solium UNAM-cd1_adult Taenia ... 444 6e-125

gb|EL762746.1| AD0192044 Taenia solium UNAM-cd1_adult Taenia ... 442 2e-124

gb|EL763270.1| AD0201033 Taenia solium UNAM-cd1_adult Taenia ... 416 1e-116

gb|EL746261.1| LV0306012 Taenia solium UNAM-cd2_larva Taenia ... 226 3e-59

gb|EL746846.1| LV0314059 Taenia solium UNAM-cd2_larva Taenia ... 102 4e-22

Query= TSBA.R10.esd 563 0 563 ESD GOOD: 98-522

Length=425

Score E

Sequences producing significant alignments: (Bits) Value

gb|EL758663.1| AD0109038 Taenia solium UNAM-cd1_adult Taenia ... 115 6e-26

gb|EL752054.1| AD0012082 Taenia solium UNAM-cd1_adult Taenia ... 115 6e-26

Query= TSBJ.R95.esd 424 0 424 ESD GOOD: 92-358

Length=267

Score E

Sequences producing significant alignments: (Bits) Value

gb|EL762135.1| AD0179028 Taenia solium UNAM-cd1_adult Taenia ... 383 1e-106

gb|EL762648.1| AD0191009 Taenia solium UNAM-cd1_adult Taenia ... 377 7e-105

gb|EL762374.1| AD0184015 Taenia solium UNAM-cd1_adult Taenia ... 372 3e-103

Query= TSBW.R86.esd 742 0 742 ESD GOOD: 72-535

Length=464

Score E

Sequences producing significant alignments: (Bits) Value

gb|EL759053.1| AD0116016 Taenia solium UNAM-cd1_adult Taenia ... 508 2e-144

gb|EL756659.1| AD0030191 Taenia solium UNAM-cd1_adult Taenia ... 466 1e-131

gb|GT227953.1| tscaa0_002829.z1.scf Taenia solium adult full-... 52.8 4e-07

Query= TSBF.R58.esd 559 0 559 ESD GOOD: 90-553

Length=464

Score E

Sequences producing significant alignments: (Bits) Value

gb|EL741662.1| LV0230009 Taenia solium UNAM-cd2_larva Taenia ... 795 0.0

gb|EL744920.1| LV0282046 Taenia solium UNAM-cd2_larva Taenia ... 252 4e-67

Query= TSBO.R32.esd 757 0 757 ESD GOOD: 111-600

Length=490

Score E

Sequences producing significant alignments: (Bits) Value

gb|EL760441.1| AD0142039 Taenia solium UNAM-cd1_adult Taenia ... 652 0.0

gb|EL743078.1| LV0251043 Taenia solium UNAM-cd2_larva Taenia ... 569 1e-162

gb|EL743223.1| LV0254004 Taenia solium UNAM-cd2_larva Taenia ... 568 4e-162

Query= TSAK.R19.esd 835 0 835 ESD GOOD: 103-499

Length=397

Score E

Sequences producing significant alignments: (Bits) Value

gb|EL746676.1| LV0312037 Taenia solium UNAM-cd2_larva Taenia ... 645 0.0

Query= TSBD.R19.esd 612 0 612 ESD GOOD: 105-595

Length=491

Score E

Sequences producing significant alignments: (Bits) Value

gb|GT227531.1| tscaa0_001550.z1.scf Taenia solium adult full-... 464 5e-131

Query= TSCG.R80.esd 669 0 669 ESD GOOD: 127-572

Length=446

Score E

Sequences producing significant alignments: (Bits) Value

gb|GT227151.1| tscaa0_000115.z1.scf Taenia solium adult full-... 549 1e-156

Query= TSBG.R38.esd 631 0 631 ESD GOOD: 94-613

Length=520

Score E

Sequences producing significant alignments: (Bits) Value

gb|EL761034.1| AD0155022 Taenia solium UNAM-cd1_adult Taenia ... 837 0.0

gb|EL742828.1| LV0247046 Taenia solium UNAM-cd2_larva Taenia ... 804 0.0

gb|EL740651.1| LV0213033 Taenia solium UNAM-cd2_larva Taenia ... 712 0.0

gb|EL745950.1| LV0299039 Taenia solium UNAM-cd2_larva Taenia ... 222 3e-58

gb|EL756696.1| AD0030228 Taenia solium UNAM-cd1_adult Taenia ... 102 4e-22

gb|EL746072.1| LV0301027 Taenia solium UNAM-cd2_larva Taenia ... 78.7 7e-15

Query= TSAB.R46.esd 594 0 594 ESD GOOD: 99-306

Length=208

Score E

Sequences producing significant alignments: (Bits) Value

gb|EL760754.1| AD0148032 Taenia solium UNAM-cd1_adult Taenia ... 291 9e-79

gb|EL759919.1| AD0132010 Taenia solium UNAM-cd1_adult Taenia ... 291 9e-79

gb|EL757597.1| AD0035190 Taenia solium UNAM-cd1_adult Taenia ... 291 9e-79

gb|EL750722.1| AD0006161 Taenia solium UNAM-cd1_adult Taenia ... 291 9e-79

gb|EL749688.1| AD0002052 Taenia solium UNAM-cd1_adult Taenia ... 291 9e-79

gb|EL747850.1| LV0330015 Taenia solium UNAM-cd2_larva Taenia ... 276 2e-74

gb|EL743352.1| LV0256013 Taenia solium UNAM-cd2_larva Taenia ... 231 5e-61

gb|EL756978.1| AD0031251 Taenia solium UNAM-cd1_adult Taenia ... 185 4e-47

Query= TSCA.R20.esd 652 0 652 ESD GOOD: 107-628

Length=522

Score E

Sequences producing significant alignments: (Bits) Value

gb|EL758645.1| AD0109020 Taenia solium UNAM-cd1_adult Taenia ... 159 3e-39

Query= TSCA.R11.esd 731 0 731 ESD GOOD: 104-586

Length=483

Score E

Sequences producing significant alignments: (Bits) Value

gb|EL748442.1| LV0340016 Taenia solium UNAM-cd2_larva Taenia ... 204 1e-52

Query= TSAA.R48.esd 645 0 645 ESD GOOD: 95-513

Length=419

Score E

Sequences producing significant alignments: (Bits) Value

gb|EL746184.1| LV0304009 Taenia solium UNAM-cd2_larva Taenia ... 614 5e-176

gb|EL740266.1| LV0205046 Taenia solium UNAM-cd2_larva Taenia ... 508 2e-144

Query= TSAM.R62.esd 447 0 447 ESD GOOD: 94-396

Length=303

Score E

Sequences producing significant alignments: (Bits) Value

gb|EL743620.1| LV0260008 Taenia solium UNAM-cd2_larva Taenia ... 532 1e-151

gb|EL742762.1| LV0246045 Taenia solium UNAM-cd2_larva Taenia ... 169 4e-42

Query= TSBT.R73.esd 764 0 764 ESD GOOD: 104-379

Length=276

Score E

Sequences producing significant alignments: (Bits) Value

gb|EL743458.1| LV0257075 Taenia solium UNAM-cd2_larva Taenia ... 241 9e-64

gb|EL748306.1| LV0337027 Taenia solium UNAM-cd2_larva Taenia ... 213 2e-55

Query= TSBM.R76.esd 799 0 799 ESD GOOD: 101-214

Length=114

Score E

Sequences producing significant alignments: (Bits) Value

gb|EL742018.1| LV0235007 Taenia solium UNAM-cd2_larva Taenia ... 196 2e-50

Query= TSAM.R40.esd 676 0 676 ESD GOOD: 110-379

Length=270

Score E

Sequences producing significant alignments: (Bits) Value

gb|EL740378.1| LV0207062 Taenia solium UNAM-cd2_larva Taenia ... 254 1e-67

Query= TSAQ.R58.esd 674 0 674 ESD GOOD: 87-557

Length=471

Score E

Sequences producing significant alignments: (Bits) Value

gb|EL763078.1| AD0198016 Taenia solium UNAM-cd1_adult Taenia ... 848 0.0

Query= TSAM.R32.esd 612 0 612 ESD GOOD: 85-528

Length=444

Score E

Sequences producing significant alignments: (Bits) Value

gb|EL757578.1| AD0035171 Taenia solium UNAM-cd1_adult Taenia ... 758 0.0

gb|EL756845.1| AD0031118 Taenia solium UNAM-cd1_adult Taenia ... 758 0.0

gb|EL756648.1| AD0030180 Taenia solium UNAM-cd1_adult Taenia ... 758 0.0

gb|EL756349.1| AD0029144 Taenia solium UNAM-cd1_adult Taenia ... 758 0.0

gb|EL755295.1| AD0025065 Taenia solium UNAM-cd1_adult Taenia ... 758 0.0

gb|EL752673.1| AD0014214 Taenia solium UNAM-cd1_adult Taenia ... 758 0.0

gb|EL751496.1| AD0009234 Taenia solium UNAM-cd1_adult Taenia ... 758 0.0

gb|EL755709.1| AD0027005 Taenia solium UNAM-cd1_adult Taenia ... 750 0.0

gb|EL753633.1| AD0018171 Taenia solium UNAM-cd1_adult Taenia ... 750 0.0

gb|EL753502.1| AD0018040 Taenia solium UNAM-cd1_adult Taenia ... 750 0.0

Query= TSAP.R91.esd 757 0 757 ESD GOOD: 93-586

Length=494

Score E

Sequences producing significant alignments: (Bits) Value

gb|GT227694.1| tscaa0_001978.z1.scf Taenia solium adult full-... 767 0.0

Query= TSBD.R56.esd 528 0 528 ESD GOOD: 95-456

Length=362

Score E

Sequences producing significant alignments: (Bits) Value

gb|EL747789.1| LV0329027 Taenia solium UNAM-cd2_larva Taenia ... 401 4e-112

gb|EL741151.1| LV0221052 Taenia solium UNAM-cd2_larva Taenia ... 401 4e-112

gb|EL748037.1| LV0332059 Taenia solium UNAM-cd2_larva Taenia ... 396 2e-110

Query= TSBU.R74.esd 494 0 494 ESD GOOD: 107-348

Length=242

Score E

Sequences producing significant alignments: (Bits) Value

gb|EL755370.1| AD0025140 Taenia solium UNAM-cd1_adult Taenia ... 176 3e-44

gb|GT226995.1| tscaa0_000549.z1.scf Taenia solium adult full-... 137 1e-32

Query= TSBU.R59.esd 680 0 680 ESD GOOD: 98-606

Length=509

Score E

Sequences producing significant alignments: (Bits) Value

gb|EL748364.1| LV0339002 Taenia solium UNAM-cd2_larva Taenia ... 507 8e-144

Query= TSCC.R4.esd 557 0 557 ESD GOOD: 97-450

Length=354

Score E

Sequences producing significant alignments: (Bits) Value

gb|EL742033.1| LV0235022 Taenia solium UNAM-cd2_larva Taenia ... 577 6e-165

gb|EL745130.1| LV0286047 Taenia solium UNAM-cd2_larva Taenia ... 566 1e-161

Query= TSBE.R78.esd 603 0 603 ESD GOOD: 96-291

Length=196

Score E

Sequences producing significant alignments: (Bits) Value

gb|EL752021.1| AD0012049 Taenia solium UNAM-cd1_adult Taenia ... 283 1e-76

Query= TSBB.R30.esd 763 0 763 ESD GOOD: 67-267

Length=201

Score E

Sequences producing significant alignments: (Bits) Value

gb|EL743632.1| LV0260020 Taenia solium UNAM-cd2_larva Taenia ... 95.3 7e-20

Query= TSBO.R18.esd 754 0 754 ESD GOOD: 97-314

Length=218

Score E

Sequences producing significant alignments: (Bits) Value

gb|EL743968.1| LV0265040 Taenia solium UNAM-cd2_larva Taenia ... 56.5 3e-08

Query= TSBE.R41.esd 511 0 511 ESD GOOD: 108-436

Length=329

Score E

Sequences producing significant alignments: (Bits) Value

gb|EL759105.1| AD0117008 Taenia solium UNAM-cd1_adult Taenia ... 520 1e-147

Query= TSBB.R85.esd 610 0 610 ESD GOOD: 91-530

Length=440

Score E

Sequences producing significant alignments: (Bits) Value

gb|EL742824.1| LV0247042 Taenia solium UNAM-cd2_larva Taenia ... 701 0.0

gb|EL741172.1| LV0222005 Taenia solium UNAM-cd2_larva Taenia ... 684 0.0

Query= TSAK.R83.esd 754 0 754 ESD GOOD: 100-402

Length=303

Score E

Sequences producing significant alignments: (Bits) Value

gb|EL748635.1| LV0343020 Taenia solium UNAM-cd2_larva Taenia ... 508 2e-144

gb|EL748749.1| LV0345001 Taenia solium UNAM-cd2_larva Taenia ... 507 8e-144

gb|EL748392.1| LV0339030 Taenia solium UNAM-cd2_larva Taenia ... 496 2e-140

gb|EL749354.1| LV0359032 Taenia solium UNAM-cd2_larva Taenia ... 405 3e-113

gb|EL740392.1| LV0208012 Taenia solium UNAM-cd2_larva Taenia ... 385 4e-107

Query= TSAW.R93.esd 617 0 617 ESD GOOD: 85-556

Length=472

Score E

Sequences producing significant alignments: (Bits) Value

gb|EL759950.1| AD0132041 Taenia solium UNAM-cd1_adult Taenia ... 832 0.0

gb|EL754332.1| AD0021122 Taenia solium UNAM-cd1_adult Taenia ... 832 0.0

gb|EL760568.1| AD0144055 Taenia solium UNAM-cd1_adult Taenia ... 821 0.0

gb|EL751599.1| AD0010092 Taenia solium UNAM-cd1_adult Taenia ... 806 0.0

Query= TSCE.R88.esd 613 0 613 ESD GOOD: 98-574

Length=477

Score E

Sequences producing significant alignments: (Bits) Value

gb|EL761863.1| AD0172030 Taenia solium UNAM-cd1_adult Taenia ... 473 8e-134

gb|EL761835.1| AD0172002 Taenia solium UNAM-cd1_adult Taenia ... 473 8e-134

gb|EL761024.1| AD0155012 Taenia solium UNAM-cd1_adult Taenia ... 457 8e-129

Query= TSCD.R72.esd 683 0 683 ESD GOOD: 99-584

Length=486

Score E

Sequences producing significant alignments: (Bits) Value

gb|EL740937.1| LV0218048 Taenia solium UNAM-cd2_larva Taenia ... 712 0.0

gb|EL744578.1| LV0275053 Taenia solium UNAM-cd2_larva Taenia ... 595 2e-170

Query= TSBV.R45.esd 598 0 598 ESD GOOD: 105-597

Length=493

Score E

Sequences producing significant alignments: (Bits) Value

gb|EL750471.1| AD0005134 Taenia solium UNAM-cd1_adult Taenia ... 368 4e-102

gb|EL750228.1| AD0004143 Taenia solium UNAM-cd1_adult Taenia ... 368 4e-102

gb|EL748792.1| LV0345044 Taenia solium UNAM-cd2_larva Taenia ... 233 1e-61

gb|EL745581.1| LV0293059 Taenia solium UNAM-cd2_larva Taenia ... 135 4e-32

gb|EL740645.1| LV0213027 Taenia solium UNAM-cd2_larva Taenia ... 75.0 1e-13

Query= TSAJ.R6.esd 374 0 374 ESD GOOD: 102-374

Length=273

Score E

Sequences producing significant alignments: (Bits) Value

gb|EL746412.1| LV0308062 Taenia solium UNAM-cd2_larva Taenia ... 377 7e-105

gb|EL749116.1| LV0353028 Taenia solium UNAM-cd2_larva Taenia ... 363 2e-100

Query= TSBI.R69.esd 793 0 793 ESD GOOD: 103-618

Length=516

Score E

Sequences producing significant alignments: (Bits) Value

gb|EL740522.1| LV0210046 Taenia solium UNAM-cd2_larva Taenia ... 765 0.0

Query= TSBA.R15.esd 336 0 336 ESD GOOD: 63-280

Length=218

Score E

Sequences producing significant alignments: (Bits) Value

gb|EL741170.1| LV0222003 Taenia solium UNAM-cd2_larva Taenia ... 224 9e-59

gb|EL762505.1| AD0187024 Taenia solium UNAM-cd1_adult Taenia ... 176 3e-44

Query= TSBR.R14.esd 935 0 935 ESD GOOD: 103-611

Length=509

Score E

Sequences producing significant alignments: (Bits) Value

gb|EL748092.1| LV0333043 Taenia solium UNAM-cd2_larva Taenia ... 889 0.0

gb|EL743764.1| LV0262005 Taenia solium UNAM-cd2_larva Taenia ... 885 0.0

gb|EL746681.1| LV0312042 Taenia solium UNAM-cd2_larva Taenia ... 880 0.0

gb|EL743061.1| LV0251026 Taenia solium UNAM-cd2_larva Taenia ... 470 1e-132

gb|EL741648.1| LV0229059 Taenia solium UNAM-cd2_larva Taenia ... 130 2e-30

Query= TSBS.R78.esd 572 0 572 ESD GOOD: 97-411

Length=315

Score E

Sequences producing significant alignments: (Bits) Value

gb|EL744799.1| LV0280025 Taenia solium UNAM-cd2_larva Taenia ... 505 3e-143

Query= TSBZ.R35.esd 561 0 561 ESD GOOD: 107-555

Length=449

Score E

Sequences producing significant alignments: (Bits) Value

gb|EL742776.1| LV0246059 Taenia solium UNAM-cd2_larva Taenia ... 551 4e-157

gb|EL743860.1| LV0263029 Taenia solium UNAM-cd2_larva Taenia ... 135 4e-32

Query= TSAD.R14.esd 562 0 562 ESD GOOD: 114-349

Length=236

Score E

Sequences producing significant alignments: (Bits) Value

gb|EL758380.1| AD0104065 Taenia solium UNAM-cd1_adult Taenia ... 409 2e-114

gb|EL744659.1| LV0277036 Taenia solium UNAM-cd2_larva Taenia ... 409 2e-114

gb|EL741907.1| LV0233040 Taenia solium UNAM-cd2_larva Taenia ... 409 2e-114

gb|EL744804.1| LV0280030 Taenia solium UNAM-cd2_larva Taenia ... 383 1e-106

Query= TSAL.R61.esd 701 0 701 ESD GOOD: 95-506

Length=412

Score E

Sequences producing significant alignments: (Bits) Value

gb|EL746488.1| LV0309058 Taenia solium UNAM-cd2_larva Taenia ... 108 9e-24

Query= TSBG.R20.esd 495 0 495 ESD GOOD: 106-388

Length=283

Score E

Sequences producing significant alignments: (Bits) Value

gb|EL757651.1| AD0035244 Taenia solium UNAM-cd1_adult Taenia ... 501 4e-142

gb|EL760617.1| AD0146002 Taenia solium UNAM-cd1_adult Taenia ... 412 2e-115

Query= TSAA.R1.esd 680 0 680 ESD GOOD: 96-573

Length=478

Score E

Sequences producing significant alignments: (Bits) Value

gb|GT227545.1| tscaa0_001588.z1.scf Taenia solium adult full-... 111 7e-25

Query= TSAO.R53.esd 463 0 463 ESD GOOD: 104-462

Length=359

Score E

Sequences producing significant alignments: (Bits) Value

gb|EL747524.1| LV0325003 Taenia solium UNAM-cd2_larva Taenia ... 508 2e-144

gb|EL744385.1| LV0271067 Taenia solium UNAM-cd2_larva Taenia ... 305 3e-83

Query= TSBN.R66.esd 661 0 661 ESD GOOD: 92-410

Length=319

Score E

Sequences producing significant alignments: (Bits) Value

gb|EL753551.1| AD0018089 Taenia solium UNAM-cd1_adult Taenia ... 532 1e-151

Query= TSBL.R30.esd 687 0 687 ESD GOOD: 95-198

Length=104

Score E

Sequences producing significant alignments: (Bits) Value

gb|EL752301.1| AD0013068 Taenia solium UNAM-cd1_adult Taenia ... 178 7e-45

Query= TSCG.R85.esd 699 0 699 ESD GOOD: 142-516

Length=375

Score E

Sequences producing significant alignments: (Bits) Value

gb|EL745267.1| LV0288049 Taenia solium UNAM-cd2_larva Taenia ... 665 0.0

gb|EL743777.1| LV0262018 Taenia solium UNAM-cd2_larva Taenia ... 665 0.0

gb|EL761406.1| AD0163015 Taenia solium UNAM-cd1_adult Taenia ... 658 0.0

gb|EL749997.1| AD0003139 Taenia solium UNAM-cd1_adult Taenia ... 658 0.0

gb|EL749774.1| AD0002138 Taenia solium UNAM-cd1_adult Taenia ... 647 0.0

gb|EL744393.1| LV0272008 Taenia solium UNAM-cd2_larva Taenia ... 377 7e-105

Query= TSAG.R52.esd 649 0 649 ESD GOOD: 91-243

Length=153

Score E

Sequences producing significant alignments: (Bits) Value

gb|EL741260.1| LV0223038 Taenia solium UNAM-cd2_larva Taenia ... 54.7 1e-07

Query= TSAR.R91.esd 423 0 423 ESD GOOD: 58-307

Length=250

Score E

Sequences producing significant alignments: (Bits) Value

gb|EL750859.1| AD0007095 Taenia solium UNAM-cd1_adult Taenia ... 222 3e-58

gb|EL742709.1| LV0245064 Taenia solium UNAM-cd2_larva Taenia ... 191 9e-49

Query= TSBF.R68.esd 719 0 719 ESD GOOD: 99-437

Length=339

Score E

Sequences producing significant alignments: (Bits) Value

gb|EL742020.1| LV0235009 Taenia solium UNAM-cd2_larva Taenia ... 244 7e-65

Query= TSAA.R75.esd 618 0 618 ESD GOOD: 102-453

Length=352

Score E

Sequences producing significant alignments: (Bits) Value

gb|EL757512.1| AD0035105 Taenia solium UNAM-cd1_adult Taenia ... 614 5e-176

gb|EL752171.1| AD0012199 Taenia solium UNAM-cd1_adult Taenia ... 614 5e-176

gb|EL749511.1| AD0001134 Taenia solium UNAM-cd1_adult Taenia ... 614 5e-176

gb|EL749453.1| AD0001076 Taenia solium UNAM-cd1_adult Taenia ... 614 5e-176

gb|EL756368.1| AD0029163 Taenia solium UNAM-cd1_adult Taenia ... 610 6e-175

gb|EL752407.1| AD0013174 Taenia solium UNAM-cd1_adult Taenia ... 311 7e-85

gb|EL763467.1| AD0204030 Taenia solium UNAM-cd1_adult Taenia ... 289 3e-78

gb|EL760397.1| AD0141034 Taenia solium UNAM-cd1_adult Taenia ... 110 3e-24

Query= TSAO.R61.esd 662 0 662 ESD GOOD: 101-575

Length=475

Score E

Sequences producing significant alignments: (Bits) Value

gb|EL742452.1| LV0242016 Taenia solium UNAM-cd2_larva Taenia ... 246 2e-65

Query= TSAN.R68.esd 671 0 671 ESD GOOD: 101-576

Length=476

Score E

Sequences producing significant alignments: (Bits) Value

gb|EL754842.1| AD0023098 Taenia solium UNAM-cd1_adult Taenia ... 859 0.0

gb|EL741624.1| LV0229035 Taenia solium UNAM-cd2_larva Taenia ... 747 0.0

Query= TSAM.R92.esd 653 0 653 ESD GOOD: 79-380

Length=302

Score E

Sequences producing significant alignments: (Bits) Value

gb|EL746489.1| LV0309059 Taenia solium UNAM-cd2_larva Taenia ... 536 1e-152

Query= TSBP.R43.esd 670 0 670 ESD GOOD: 77-579

Length=503

Score E

Sequences producing significant alignments: (Bits) Value

gb|EL752116.1| AD0012144 Taenia solium UNAM-cd1_adult Taenia ... 863 0.0

gb|EL752358.1| AD0013125 Taenia solium UNAM-cd1_adult Taenia ... 630 0.0

gb|EL753693.1| AD0018231 Taenia solium UNAM-cd1_adult Taenia ... 621 3e-178

gb|EL759004.1| AD0115029 Taenia solium UNAM-cd1_adult Taenia ... 597 5e-171

gb|EL746571.1| LV0310068 Taenia solium UNAM-cd2_larva Taenia ... 451 4e-127

gb|GT227515.1| tscaa0_001517.z1.scf Taenia solium adult full-... 261 7e-70

Query= TSAO.R24.esd 685 0 685 ESD GOOD: 116-558

Length=443

Score E

Sequences producing significant alignments: (Bits) Value

gb|EL749152.1| LV0354011 Taenia solium UNAM-cd2_larva Taenia ... 603 1e-172

Query= TSCG.R88.esd 615 0 615 ESD GOOD: 102-544

Length=443

Score E

Sequences producing significant alignments: (Bits) Value

gb|EL756880.1| AD0031153 Taenia solium UNAM-cd1_adult Taenia ... 795 0.0

gb|EL762056.1| AD0177027 Taenia solium UNAM-cd1_adult Taenia ... 745 0.0

gb|EL758611.1| AD0108042 Taenia solium UNAM-cd1_adult Taenia ... 743 0.0

gb|EL763116.1| AD0198054 Taenia solium UNAM-cd1_adult Taenia ... 379 2e-105

Query= TSCF.R18.esd 600 0 600 ESD GOOD: 99-322

Length=224

Score E

Sequences producing significant alignments: (Bits) Value

gb|EL740870.1| LV0217053 Taenia solium UNAM-cd2_larva Taenia ... 363 2e-100

gb|EL746855.1| LV0314068 Taenia solium UNAM-cd2_larva Taenia ... 361 7e-100

gb|EL746410.1| LV0308060 Taenia solium UNAM-cd2_larva Taenia ... 361 7e-100

gb|EL742017.1| LV0235006 Taenia solium UNAM-cd2_larva Taenia ... 361 7e-100

gb|EL740836.1| LV0217019 Taenia solium UNAM-cd2_larva Taenia ... 326 2e-89

gb|EL740810.1| LV0216044 Taenia solium UNAM-cd2_larva Taenia ... 261 7e-70

gb|EL747865.1| LV0330030 Taenia solium UNAM-cd2_larva Taenia ... 119 4e-27

Query= TSAD.R19.esd 628 0 628 ESD GOOD: 88-581

Length=494

Score E

Sequences producing significant alignments: (Bits) Value

gb|EL763341.1| AD0202041 Taenia solium UNAM-cd1_adult Taenia ... 893 0.0

gb|EL758958.1| AD0114028 Taenia solium UNAM-cd1_adult Taenia ... 893 0.0

gb|EL757663.1| AD0094003 Taenia solium UNAM-cd1_adult Taenia ... 893 0.0

gb|EL758216.1| AD0102027 Taenia solium UNAM-cd1_adult Taenia ... 887 0.0

gb|EL762702.1| AD0191063 Taenia solium UNAM-cd1_adult Taenia ... 881 0.0

gb|EL758502.1| AD0106045 Taenia solium UNAM-cd1_adult Taenia ... 881 0.0

gb|EL750962.1| AD0007198 Taenia solium UNAM-cd1_adult Taenia ... 881 0.0

gb|EL761072.1| AD0156012 Taenia solium UNAM-cd1_adult Taenia ... 856 0.0

gb|EL762046.1| AD0177017 Taenia solium UNAM-cd1_adult Taenia ... 832 0.0

gb|EL750733.1| AD0006172 Taenia solium UNAM-cd1_adult Taenia ... 558 2e-159

Query= TSAN.R80.esd 723 0 723 ESD GOOD: 144-663

Length=520

Score E

Sequences producing significant alignments: (Bits) Value

gb|EL744144.1| LV0268011 Taenia solium UNAM-cd2_larva Taenia ... 686 0.0

Query= TSAA.R60.esd 631 0 631 ESD GOOD: 105-428

Length=324

Score E

Sequences producing significant alignments: (Bits) Value

gb|EL745801.1| LV0297030 Taenia solium UNAM-cd2_larva Taenia ... 568 4e-162

Query= TSCF.R37.esd 558 0 558 ESD GOOD: 103-558

Length=456

Score E

Sequences producing significant alignments: (Bits) Value

gb|EL744055.1| LV0266055 Taenia solium UNAM-cd2_larva Taenia ... 761 0.0

gb|EL746784.1| LV0313067 Taenia solium UNAM-cd2_larva Taenia ... 612 2e-175

gb|EL747190.1| LV0319049 Taenia solium UNAM-cd2_larva Taenia ... 315 5e-86

Query= TSBN.R13.esd 487 0 487 ESD GOOD: 85-487

Length=403

Score E

Sequences producing significant alignments: (Bits) Value

gb|EL760957.1| AD0153031 Taenia solium UNAM-cd1_adult Taenia ... 346 2e-95

Query= TSCG.R58.esd 645 0 645 ESD GOOD: 95-578

Length=484

Score E

Sequences producing significant alignments: (Bits) Value

gb|GT227698.1| tscaa0_001990.z1.scf Taenia solium adult full-... 457 8e-129

Query= TSBP.R51.esd 676 0 676 ESD GOOD: 91-252

Length=162

Score E

Sequences producing significant alignments: (Bits) Value

gb|EL747074.1| LV0318006 Taenia solium UNAM-cd2_larva Taenia ... 279 2e-75

gb|EL743832.1| LV0263001 Taenia solium UNAM-cd2_larva Taenia ... 274 9e-74

gb|EL758056.1| AD0100001 Taenia solium UNAM-cd1_adult Taenia ... 191 9e-49

gb|EL745969.1| LV0299058 Taenia solium UNAM-cd2_larva Taenia ... 102 4e-22

Query= TSBR.R17.esd 746 0 746 ESD GOOD: 104-300

Length=197

Score E

Sequences producing significant alignments: (Bits) Value

gb|EL756696.1| AD0030228 Taenia solium UNAM-cd1_adult Taenia ... 333 1e-91

gb|EL746072.1| LV0301027 Taenia solium UNAM-cd2_larva Taenia ... 324 9e-89

gb|EL758670.1| AD0109045 Taenia solium UNAM-cd1_adult Taenia ... 182 5e-46

Query= TSCF.R14.esd 745 0 745 ESD GOOD: 97-565

Length=469

Score E

Sequences producing significant alignments: (Bits) Value

gb|EL750788.1| AD0007024 Taenia solium UNAM-cd1_adult Taenia ... 457 8e-129

gb|EL763229.1| AD0200044 Taenia solium UNAM-cd1_adult Taenia ... 451 4e-127

gb|EL750579.1| AD0006018 Taenia solium UNAM-cd1_adult Taenia ... 399 1e-111

gb|EL763230.1| AD0200045 Taenia solium UNAM-cd1_adult Taenia ... 311 7e-85

gb|EL750580.1| AD0006019 Taenia solium UNAM-cd1_adult Taenia ... 311 7e-85

gb|EL750787.1| AD0007023 Taenia solium UNAM-cd1_adult Taenia ... 303 1e-82

Query= TSAK.R80.esd 831 0 831 ESD GOOD: 97-491

Length=395

Score E

Sequences producing significant alignments: (Bits) Value

gb|EL748474.1| LV0340048 Taenia solium UNAM-cd2_larva Taenia ... 113 2e-25

Query= TSBP.R25.esd 647 0 647 ESD GOOD: 18-572

Length=555

Score E

Sequences producing significant alignments: (Bits) Value

gb|GT227514.1| tscaa0_001516.z1.scf Taenia solium adult full-... 503 1e-142

Query= TSBC.R21.esd 578 0 578 ESD GOOD: 96-575

Length=480

Score E

Sequences producing significant alignments: (Bits) Value

gb|EL749307.1| LV0358018 Taenia solium UNAM-cd2_larva Taenia ... 231 5e-61

Query= TSBL.R3.esd 578 0 578 ESD GOOD: 109-464

Length=356

Score E

Sequences producing significant alignments: (Bits) Value

gb|EL757058.1| AD0032070 Taenia solium UNAM-cd1_adult Taenia ... 610 6e-175

Query= TSAL.R22.esd 592 0 592 ESD GOOD: 103-364

Length=262

Score E

Sequences producing significant alignments: (Bits) Value

gb|EL743174.1| LV0253021 Taenia solium UNAM-cd2_larva Taenia ... 453 1e-127

gb|EL746428.1| LV0308078 Taenia solium UNAM-cd2_larva Taenia ... 387 1e-107

gb|EL762331.1| AD0183032 Taenia solium UNAM-cd1_adult Taenia ... 257 9e-69

gb|EL746933.1| LV0316010 Taenia solium UNAM-cd2_larva Taenia ... 180 2e-45

Query= TSBL.R60.esd 685 0 685 ESD GOOD: 99-564

Length=466

Score E

Sequences producing significant alignments: (Bits) Value

gb|GT227006.1| tscaa0_000687.z1.scf Taenia solium adult full-... 828 0.0

gb|EL758052.1| AD0099064 Taenia solium UNAM-cd1_adult Taenia ... 817 0.0

gb|EL759024.1| AD0115049 Taenia solium UNAM-cd1_adult Taenia ... 765 0.0

gb|EL742442.1| LV0242006 Taenia solium UNAM-cd2_larva Taenia ... 521 3e-148

gb|EL759052.1| AD0116015 Taenia solium UNAM-cd1_adult Taenia ... 396 2e-110

gb|EL746761.1| LV0313044 Taenia solium UNAM-cd2_larva Taenia ... 141 9e-34

Query= TSCE.R78.esd 733 0 733 ESD GOOD: 104-441

Length=338

Score E

Sequences producing significant alignments: (Bits) Value

gb|EL743953.1| LV0265025 Taenia solium UNAM-cd2_larva Taenia ... 106 3e-23

Query= TSCH.R61.esd 611 0 611 ESD GOOD: 104-479

Length=376

Score E

Sequences producing significant alignments: (Bits) Value

gb|EL758396.1| AD0105015 Taenia solium UNAM-cd1_adult Taenia ... 636 0.0

gb|EL758145.1| AD0101025 Taenia solium UNAM-cd1_adult Taenia ... 636 0.0

gb|EL758832.1| AD0112031 Taenia solium UNAM-cd1_adult Taenia ... 630 0.0

Query= TSBC.R35.esd 682 0 682 ESD GOOD: 86-405

Length=320

Score E

Sequences producing significant alignments: (Bits) Value

gb|EL749105.1| LV0353017 Taenia solium UNAM-cd2_larva Taenia ... 475 2e-134

gb|EL741224.1| LV0223002 Taenia solium UNAM-cd2_larva Taenia ... 399 1e-111

gb|EL758493.1| AD0106036 Taenia solium UNAM-cd1_adult Taenia ... 158 9e-39

Query= TSAP.R46.esd 637 0 637 ESD GOOD: 84-593

Length=510

Score E

Sequences producing significant alignments: (Bits) Value

gb|EL752016.1| AD0012044 Taenia solium UNAM-cd1_adult Taenia ... 904 0.0

gb|EL757636.1| AD0035229 Taenia solium UNAM-cd1_adult Taenia ... 885 0.0

gb|EL748405.1| LV0339043 Taenia solium UNAM-cd2_larva Taenia ... 885 0.0

gb|EL758632.1| AD0109007 Taenia solium UNAM-cd1_adult Taenia ... 846 0.0

gb|EL749216.1| LV0355030 Taenia solium UNAM-cd2_larva Taenia ... 651 0.0

gb|EL752270.1| AD0013037 Taenia solium UNAM-cd1_adult Taenia ... 606 8e-174

Query= TSAF.R33.esd 824 0 824 ESD GOOD: 101-613

Length=513

Score E

Sequences producing significant alignments: (Bits) Value

gb|EL748576.1| LV0342027 Taenia solium UNAM-cd2_larva Taenia ... 158 9e-39

Query= TSAO.R75.esd 567 0 567 ESD GOOD: 97-444

Length=348

Score E

Sequences producing significant alignments: (Bits) Value

gb|EL747120.1| LV0318052 Taenia solium UNAM-cd2_larva Taenia ... 333 1e-91

Query= TSBM.R38.esd 265 0 265 ESD GOOD: 39-202

Length=164

Score E

Sequences producing significant alignments: (Bits) Value

gb|GT227350.1| tscaa0_000959.z1.scf Taenia solium adult full-... 206 3e-53

gb|EL762844.1| AD0194015 Taenia solium UNAM-cd1_adult Taenia ... 200 2e-51

Query= TSAK.R90.esd 728 0 728 ESD GOOD: 91-475

Length=385

Score E

Sequences producing significant alignments: (Bits) Value

gb|EL756294.1| AD0029089 Taenia solium UNAM-cd1_adult Taenia ... 492 2e-139

gb|EL755979.1| AD0028037 Taenia solium UNAM-cd1_adult Taenia ... 492 2e-139

gb|EL751304.1| AD0009042 Taenia solium UNAM-cd1_adult Taenia ... 490 8e-139

gb|EL750070.1| AD0003212 Taenia solium UNAM-cd1_adult Taenia ... 490 8e-139

gb|EL759948.1| AD0132039 Taenia solium UNAM-cd1_adult Taenia ... 486 1e-137

gb|EL759081.1| AD0116044 Taenia solium UNAM-cd1_adult Taenia ... 486 1e-137

gb|EL752133.1| AD0012161 Taenia solium UNAM-cd1_adult Taenia ... 486 1e-137

gb|EL752436.1| AD0013203 Taenia solium UNAM-cd1_adult Taenia ... 484 4e-137

gb|EL752371.1| AD0013138 Taenia solium UNAM-cd1_adult Taenia ... 484 4e-137

gb|EL751876.1| AD0011119 Taenia solium UNAM-cd1_adult Taenia ... 484 4e-137

Query= TSBP.R86.esd 618 0 618 ESD GOOD: 100-594

Length=495

Score E

Sequences producing significant alignments: (Bits) Value

gb|EL745293.1| LV0289005 Taenia solium UNAM-cd2_larva Taenia ... 169 4e-42

Query= TSAQ.R11.esd 866 0 866 ESD GOOD: 91-396

Length=306

Score E

Sequences producing significant alignments: (Bits) Value

gb|EL760210.1| AD0137043 Taenia solium UNAM-cd1_adult Taenia ... 355 3e-98

Query= TSAN.R56.esd 697 0 697 ESD GOOD: 109-447

Length=339

Score E

Sequences producing significant alignments: (Bits) Value

gb|EL758307.1| AD0103051 Taenia solium UNAM-cd1_adult Taenia ... 318 4e-87

gb|EL742734.1| LV0246017 Taenia solium UNAM-cd2_larva Taenia ... 307 9e-84

gb|EL761071.1| AD0156011 Taenia solium UNAM-cd1_adult Taenia ... 305 3e-83

gb|EL743721.1| LV0261030 Taenia solium UNAM-cd2_larva Taenia ... 298 5e-81

gb|EL743601.1| LV0259064 Taenia solium UNAM-cd2_larva Taenia ... 183 2e-46

Query= TSAO.R11.esd 801 0 801 ESD GOOD: 113-643

Length=531

Score E

Sequences producing significant alignments: (Bits) Value

gb|EL747805.1| LV0329043 Taenia solium UNAM-cd2_larva Taenia ... 403 1e-112

Query= TSBK.R42.esd 618 0 618 ESD GOOD: 109-530

Length=422

Score E

Sequences producing significant alignments: (Bits) Value

gb|EL745154.1| LV0287008 Taenia solium UNAM-cd2_larva Taenia ... 104 1e-22

Query= TSCB.R52.esd 472 0 472 ESD GOOD: 102-433

Length=332

Score E

Sequences producing significant alignments: (Bits) Value

gb|EL746206.1| LV0304031 Taenia solium UNAM-cd2_larva Taenia ... 337 1e-92

gb|EL746179.1| LV0304004 Taenia solium UNAM-cd2_larva Taenia ... 276 2e-74

Query= TSBQ.R18.esd 798 0 798 ESD GOOD: 101-203

Length=103

Score E

Sequences producing significant alignments: (Bits) Value

gb|EL757742.1| AD0095017 Taenia solium UNAM-cd1_adult Taenia ... 158 9e-39

gb|EL750645.1| AD0006084 Taenia solium UNAM-cd1_adult Taenia ... 134 2e-31

Query= TSAQ.R31.esd 563 0 563 ESD GOOD: 84-489

Length=406

Score E

Sequences producing significant alignments: (Bits) Value

gb|EL757638.1| AD0035231 Taenia solium UNAM-cd1_adult Taenia ... 725 0.0

gb|EL757155.1| AD0033017 Taenia solium UNAM-cd1_adult Taenia ... 693 0.0

gb|EL755055.1| AD0024072 Taenia solium UNAM-cd1_adult Taenia ... 689 0.0

gb|GT227585.1| tscaa0_001679.z1.scf Taenia solium adult full-... 281 5e-76

gb|EL742417.1| LV0241043 Taenia solium UNAM-cd2_larva Taenia ... 180 2e-45

gb|EL741320.1| LV0224040 Taenia solium UNAM-cd2_larva Taenia ... 108 9e-24

Query= TSAG.R15.esd 504 0 504 ESD GOOD: 92-460

Length=369

Score E

Sequences producing significant alignments: (Bits) Value

gb|EL743560.1| LV0259023 Taenia solium UNAM-cd2_larva Taenia ... 532 1e-151

gb|EL743544.1| LV0259007 Taenia solium UNAM-cd2_larva Taenia ... 276 2e-74

gb|EL749273.1| LV0357017 Taenia solium UNAM-cd2_larva Taenia ... 130 2e-30

Query= TSAG.R24.esd 782 0 782 ESD GOOD: 91-597

Length=507

Score E

Sequences producing significant alignments: (Bits) Value

gb|EL741304.1| LV0224024 Taenia solium UNAM-cd2_larva Taenia ... 285 4e-77

gb|EL761543.1| AD0165041 Taenia solium UNAM-cd1_adult Taenia ... 224 9e-59

Query= TSAT.R78.esd 625 0 625 ESD GOOD: 101-522

Length=422

Score E

Sequences producing significant alignments: (Bits) Value

gb|EL745821.1| LV0297050 Taenia solium UNAM-cd2_larva Taenia ... 542 2e-154

Query= TSBT.R7.esd 701 0 701 ESD GOOD: 91-608

Length=518

Score E

Sequences producing significant alignments: (Bits) Value

gb|EL762472.1| AD0186036 Taenia solium UNAM-cd1_adult Taenia ... 564 5e-161

gb|EL746079.1| LV0301034 Taenia solium UNAM-cd2_larva Taenia ... 333 1e-91

gb|EL744693.1| LV0278012 Taenia solium UNAM-cd2_larva Taenia ... 231 5e-61

Query= TSBG.R16.esd 529 0 529 ESD GOOD: 83-441

Length=359

Score E

Sequences producing significant alignments: (Bits) Value

gb|EL763181.1| AD0199065 Taenia solium UNAM-cd1_adult Taenia ... 472 3e-133

gb|EL746446.1| LV0309016 Taenia solium UNAM-cd2_larva Taenia ... 466 1e-131

gb|GT226948.1| tscaa0_001437.z1.scf Taenia solium adult full-... 457 8e-129

gb|EL757055.1| AD0032067 Taenia solium UNAM-cd1_adult Taenia ... 449 1e-126

gb|EL748283.1| LV0337004 Taenia solium UNAM-cd2_larva Taenia ... 448 5e-126

gb|EL741708.1| LV0230055 Taenia solium UNAM-cd2_larva Taenia ... 442 2e-124

gb|EL763222.1| AD0200037 Taenia solium UNAM-cd1_adult Taenia ... 435 4e-122

gb|EL747367.1| LV0322044 Taenia solium UNAM-cd2_larva Taenia ... 431 5e-121

gb|EL752933.1| AD0015192 Taenia solium UNAM-cd1_adult Taenia ... 388 3e-108

gb|EL748308.1| LV0337029 Taenia solium UNAM-cd2_larva Taenia ... 351 4e-97

Query= TSAY.R44.esd 680 0 680 ESD GOOD: 96-580

Length=485

Score E

Sequences producing significant alignments: (Bits) Value

gb|EL748247.1| LV0336023 Taenia solium UNAM-cd2_larva Taenia ... 431 5e-121

Query= TSAQ.R46.esd 593 0 593 ESD GOOD: 85-563

Length=479

Score E

Sequences producing significant alignments: (Bits) Value

gb|GT227094.1| tscaa0_002503.z1.scf Taenia solium adult full-... 750 0.0

Query= TSCA.R15.esd 599 0 599 ESD GOOD: 105-295

Length=191

Score E

Sequences producing significant alignments: (Bits) Value

gb|EL745793.1| LV0297022 Taenia solium UNAM-cd2_larva Taenia ... 327 7e-90

Query= TSBB.R74.esd 585 0 585 ESD GOOD: 87-343

Length=257

Score E

Sequences producing significant alignments: (Bits) Value

gb|EL750833.1| AD0007069 Taenia solium UNAM-cd1_adult Taenia ... 267 1e-71

gb|EL761182.1| AD0158024 Taenia solium UNAM-cd1_adult Taenia ... 165 6e-41

Query= TSAB.R59.esd 560 0 560 ESD GOOD: 129-255

Length=127

Score E

Sequences producing significant alignments: (Bits) Value

gb|EX151138.1| TPEG001001A07 Cysti-host Taenia solium cDNA, m... 154 1e-37

gb|EL749249.1| LV0356027 Taenia solium UNAM-cd2_larva Taenia ... 154 1e-37

gb|EL748580.1| LV0342031 Taenia solium UNAM-cd2_larva Taenia ... 154 1e-37

gb|EL748227.1| LV0336003 Taenia solium UNAM-cd2_larva Taenia ... 154 1e-37

gb|EL742012.1| LV0235001 Taenia solium UNAM-cd2_larva Taenia ... 154 1e-37

gb|EX151156.1| TPEG001001C03 Cysti-host Taenia solium cDNA, m... 124 9e-29

gb|EL743493.1| LV0258033 Taenia solium UNAM-cd2_larva Taenia ... 124 9e-29

gb|EX151195.1| TPEG001001F09 Cysti-host Taenia solium cDNA, m... 87.9 1e-17

Query= TSBP.R9.esd 478 0 478 ESD GOOD: 91-478

Length=388

Score E

Sequences producing significant alignments: (Bits) Value

gb|EL751486.1| AD0009224 Taenia solium UNAM-cd1_adult Taenia ... 525 2e-149

gb|EL751281.1| AD0009019 Taenia solium UNAM-cd1_adult Taenia ... 525 2e-149

gb|EL755381.1| AD0025151 Taenia solium UNAM-cd1_adult Taenia ... 254 1e-67

gb|EL755676.1| AD0026199 Taenia solium UNAM-cd1_adult Taenia ... 239 3e-63

gb|EL757549.1| AD0035142 Taenia solium UNAM-cd1_adult Taenia ... 193 3e-49

gb|EL755965.1| AD0028023 Taenia solium UNAM-cd1_adult Taenia ... 187 1e-47

gb|EL754093.1| AD0020138 Taenia solium UNAM-cd1_adult Taenia ... 137 1e-32

gb|EL753835.1| AD0019122 Taenia solium UNAM-cd1_adult Taenia ... 97.1 2e-20

gb|EL751011.1| AD0008022 Taenia solium UNAM-cd1_adult Taenia ... 97.1 2e-20

gb|EL751241.1| AD0008252 Taenia solium UNAM-cd1_adult Taenia ... 63.9 2e-10

Query= TSBA.R92.esd 592 0 592 ESD GOOD: 168-555

Length=388

Score E

Sequences producing significant alignments: (Bits) Value

gb|EL762292.1| AD0182048 Taenia solium UNAM-cd1_adult Taenia ... 536 1e-152

gb|EL743522.1| LV0258062 Taenia solium UNAM-cd2_larva Taenia ... 484 4e-137

Query= TSBE.R29.esd 336 0 336 ESD GOOD: 93-259

Length=167

Score E

Sequences producing significant alignments: (Bits) Value

gb|GT227096.1| tscaa0_002809.z1.scf Taenia solium adult full-... 243 2e-64

gb|EL749243.1| LV0356021 Taenia solium UNAM-cd2_larva Taenia ... 243 2e-64

gb|EL748506.1| LV0341022 Taenia solium UNAM-cd2_larva Taenia ... 243 2e-64

gb|EX150885.1| TSEDTS1024F10 Cysti Taenia solium cDNA, mRNA s... 237 1e-62

gb|EL749183.1| LV0354042 Taenia solium UNAM-cd2_larva Taenia ... 167 2e-41

gb|EL745126.1| LV0286043 Taenia solium UNAM-cd2_larva Taenia ... 58.4 1e-08

Query= TSAD.R73.esd 656 0 656 ESD GOOD: 94-545

Length=452

Score E

Sequences producing significant alignments: (Bits) Value

gb|EL740920.1| LV0218031 Taenia solium UNAM-cd2_larva Taenia ... 800 0.0

gb|EL761298.1| AD0161015 Taenia solium UNAM-cd1_adult Taenia ... 756 0.0

gb|EL755232.1| AD0025002 Taenia solium UNAM-cd1_adult Taenia ... 756 0.0

gb|EL762734.1| AD0192032 Taenia solium UNAM-cd1_adult Taenia ... 737 0.0

gb|EL757187.1| AD0034003 Taenia solium UNAM-cd1_adult Taenia ... 448 5e-126

gb|EL755808.1| AD0027104 Taenia solium UNAM-cd1_adult Taenia ... 440 8e-124

gb|EL746393.1| LV0308043 Taenia solium UNAM-cd2_larva Taenia ... 359 2e-99

gb|EL761593.1| AD0166034 Taenia solium UNAM-cd1_adult Taenia ... 291 9e-79

gb|EL744090.1| LV0267029 Taenia solium UNAM-cd2_larva Taenia ... 219 4e-57

gb|EL743964.1| LV0265036 Taenia solium UNAM-cd2_larva Taenia ... 191 9e-49

Query= TSBT.R8.esd 602 0 602 ESD GOOD: 103-578

Length=476

Score E

Sequences producing significant alignments: (Bits) Value

gb|EL740524.1| LV0211001 Taenia solium UNAM-cd2_larva Taenia ... 73.1 3e-13

Query= TSCB.R84.esd 615 0 615 ESD GOOD: 108-519

Length=412

Score E

Sequences producing significant alignments: (Bits) Value

gb|EL742481.1| LV0242045 Taenia solium UNAM-cd2_larva Taenia ... 701 0.0

Query= TSAZ.R27.esd 595 0 595 ESD GOOD: 95-373

Length=279

Score E

Sequences producing significant alignments: (Bits) Value

gb|EL740980.1| LV0219030 Taenia solium UNAM-cd2_larva Taenia ... 193 3e-49

Query= TSAQ.R67.esd 757 0 757 ESD GOOD: 99-597

Length=499

Score E

Sequences producing significant alignments: (Bits) Value

gb|EL742671.1| LV0245026 Taenia solium UNAM-cd2_larva Taenia ... 241 9e-64

Query= TSBR.R51.esd 668 0 668 ESD GOOD: 97-513

Length=417

Score E

Sequences producing significant alignments: (Bits) Value

gb|EL741044.1| LV0220017 Taenia solium UNAM-cd2_larva Taenia ... 697 0.0

gb|EL740458.1| LV0209036 Taenia solium UNAM-cd2_larva Taenia ... 673 0.0

gb|EL762188.1| AD0180030 Taenia solium UNAM-cd1_adult Taenia ... 311 7e-85

gb|EL740859.1| LV0217042 Taenia solium UNAM-cd2_larva Taenia ... 204 1e-52

gb|EL742463.1| LV0242027 Taenia solium UNAM-cd2_larva Taenia ... 110 3e-24

Query= TSAD.R18.esd 490 0 490 ESD GOOD: 96-471

Length=376

Score E

Sequences producing significant alignments: (Bits) Value

gb|EL762972.1| AD0196025 Taenia solium UNAM-cd1_adult Taenia ... 346 2e-95

gb|EL750910.1| AD0007146 Taenia solium UNAM-cd1_adult Taenia ... 346 2e-95

gb|EL746775.1| LV0313058 Taenia solium UNAM-cd2_larva Taenia ... 346 2e-95

gb|EL746434.1| LV0309004 Taenia solium UNAM-cd2_larva Taenia ... 346 2e-95

gb|EL741023.1| LV0219073 Taenia solium UNAM-cd2_larva Taenia ... 346 2e-95

gb|EL747511.1| LV0324047 Taenia solium UNAM-cd2_larva Taenia ... 100 2e-21

gb|EL744547.1| LV0275022 Taenia solium UNAM-cd2_larva Taenia ... 95.3 7e-20

Query= TSBA.R44.esd 598 0 598 ESD GOOD: 103-549

Length=447

Score E

Sequences producing significant alignments: (Bits) Value

gb|EL740755.1| LV0215045 Taenia solium UNAM-cd2_larva Taenia ... 691 0.0

Query= TSBZ.R36.esd 652 0 652 ESD GOOD: 94-597

Length=504

Score E

Sequences producing significant alignments: (Bits) Value

gb|EL742658.1| LV0245013 Taenia solium UNAM-cd2_larva Taenia ... 374 8e-104

Query= TSAY.R52.esd 316 0 316 ESD GOOD: 53-206

Length=154

Score E

Sequences producing significant alignments: (Bits) Value

gb|EL749194.1| LV0355008 Taenia solium UNAM-cd2_larva Taenia ... 178 7e-45

Query= TSAO.R20.esd 770 0 770 ESD GOOD: 104-518

Length=415

Score E

Sequences producing significant alignments: (Bits) Value

gb|EL746126.1| LV0302021 Taenia solium UNAM-cd2_larva Taenia ... 710 0.0

gb|EL740822.1| LV0217005 Taenia solium UNAM-cd2_larva Taenia ... 372 3e-103

Query= TSAH.R28.esd 610 0 610 ESD GOOD: 107-560

Length=454

Score E

Sequences producing significant alignments: (Bits) Value

gb|EL758524.1| AD0107017 Taenia solium UNAM-cd1_adult Taenia ... 765 0.0

gb|EL758708.1| AD0110017 Taenia solium UNAM-cd1_adult Taenia ... 676 0.0

gb|EL745868.1| LV0298025 Taenia solium UNAM-cd2_larva Taenia ... 623 8e-179

gb|EL741507.1| LV0227063 Taenia solium UNAM-cd2_larva Taenia ... 374 8e-104

gb|EL740593.1| LV0212029 Taenia solium UNAM-cd2_larva Taenia ... 374 8e-104

Query= TSAI.R91.esd 801 0 801 ESD GOOD: 198-590

Length=393

Score E

Sequences producing significant alignments: (Bits) Value

gb|EL740350.1| LV0207034 Taenia solium UNAM-cd2_larva Taenia ... 124 9e-29

gb|EL746507.1| LV0310004 Taenia solium UNAM-cd2_larva Taenia ... 122 3e-28

Query= TSAW.R75.esd 547 0 547 ESD GOOD: 101-482

Length=382

Score E

Sequences producing significant alignments: (Bits) Value

gb|EL747518.1| LV0324054 Taenia solium UNAM-cd2_larva Taenia ... 255 3e-68

Database: GenBank non-mouse and non-human EST entries

Posted date: Jul 2, 2010 5:45 PM

Number of letters in database: 11,404,901

Number of sequences in database: 25,860

Lambda K H

1.33 0.621 1.12

Gapped

Lambda K H

1.28 0.460 0.850

Matrix: blastn matrix:1 -2

Gap Penalties: Existence: 0, Extension: 0

Number of Sequences: 25860

Number of Hits to DB: 0

Number of extensions: 0

Number of successful extensions: 0

Number of sequences better than 10: 0

Number of HSP's better than 10 without gapping: 0

Number of HSP's gapped: 0

Number of HSP's successfully gapped: 0

Length of database: 11404901

A: 0

X1: 11 (21.1 bits)

X2: 32 (59.1 bits)

X3: 54 (99.7 bits)

S1: 11 (21.4 bits)
